# IFNγ augments TKI efficacy by alleviating protein unfolding stress to promote GSDME-mediated pyroptosis in hepatocellular carcinoma

**DOI:** 10.1038/s41419-025-07839-y

**Published:** 2025-07-11

**Authors:** Xiaoxiao Li, Fujia Lu, Jie Zhou, Xiong Li, Yan Li, Weijie Ye, Jing Li, Liguo Yang, Shi Tang, Yuhan Zhou, Songlin Yin, Yuan Gao, Haotian Shang, Tengfei Chao, Xiang Cheng, Qian Chu, Weimin Wang

**Affiliations:** 1 Department of Immunology, School of Basic Medicine, Tongji Medical College and State Key Laboratory for Diagnosis and Treatment of Severe Zoonotic Infectious Diseases, Huazhong University of Science and Technology, Wuhan, China; 2 Department of Gynecology & Obstetrics, the Central Hospital of Wuhan, Tongji Medical College, Huazhong University of Science and Technology, Wuhan, China; 3 Department of Oncology, Tongji Hospital, Tongji Medical College, Huazhong University of Science and Technology, Wuhan, China; 4 Department of Cardiology, Union Hospital, Tongji Medical College, Huazhong University of Science and Technology, Wuhan, China; 5 The Key Laboratory for Drug Target Researches and Pharmacodynamic Evaluation of Hubei Province, Wuhan, China; 6 Cell Architecture Research Institute, Huazhong University of Science and Technology, Wuhan, China

**Keywords:** Cancer immunotherapy, Cell death

## Abstract

Tyrosine kinase inhibitors (TKIs) are the standard treatment for advanced hepatocellular carcinoma (HCC). However, their therapeutic efficacy is often limited by drug resistance, primarily driven by tumoral intrinsic mechanisms. In this study, we demonstrate that IFNγ in the tumor microenvironment can potentiate TKI response, and that ablation of IFNγ receptor on HCC cells leads to TKI resistance in vivo. Mechanistically, IFNγ synergizes with TKI to induce GSDME-mediated pyroptosis of HCC cells. The PERK-mediated unfolded protein response (UPR) protects HCC cells from TKI-induced pyroptosis. IFNγ attenuates PERK activation by inducing the expression of PDIA1, which alleviates the stress of protein unfolding. In vivo, PERK inhibition augments TKI therapy, and elevated PERK expression correlates with poor overall survival of patients with HCC. Moreover, IFNγ-producing CD8^+^ T cells can potentiate TKI efficacy. Combining PD-1 blockade to activate T-cell response with TKI therapy synergistically suppresses the growth of GSDME-expressing HCC tumors, which is further enhanced by the PERK inhibitor. Our findings reveal how IFNγ signaling modulates TKI response and demonstrate the potential of a sequential combination of ICB-mediated immunotherapy and TKI therapy for patients with GSDME^+^ HCC.

**Figure:**
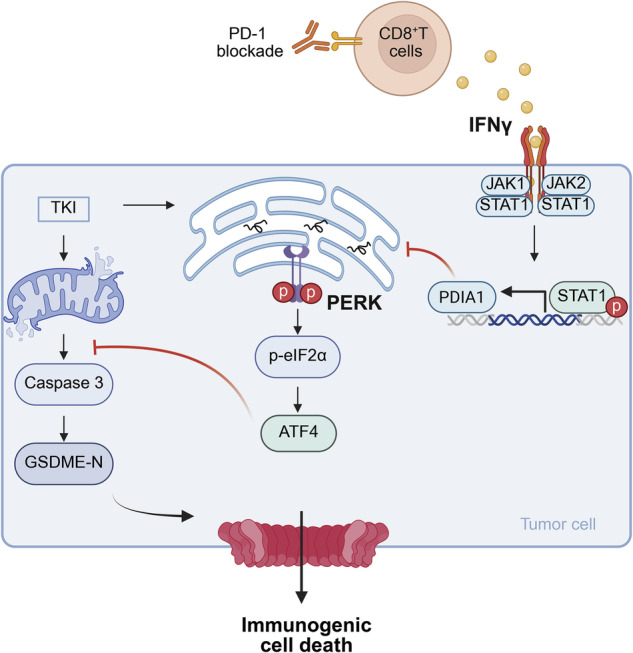
**T cell-derived IFNγ enhances TKI-induced pyroptosis in HCC**. Mechanistic illustration of IFNγ secreted from CD8^+^ T cells enhancing TKI-induced GSDME-mediated pyroptosis in hepatocellular carcinoma via suppression of the PERK pathway. Created with BioRender.com.

**T cell-derived IFNγ enhances TKI-induced pyroptosis in HCC**. Mechanistic illustration of IFNγ secreted from CD8^+^ T cells enhancing TKI-induced GSDME-mediated pyroptosis in hepatocellular carcinoma via suppression of the PERK pathway. Created with BioRender.com.

## Introduction

HCC is the most common type of primary liver cancer and typically develops in a background of chronic viral infections, alcoholic liver disease, or nonalcoholic steatohepatitis [1, 2]. Three main categories of systemic therapies have been applied for the management of advanced HCC, including immune checkpoint blockades (ICB), TKI, and monoclonal antibodies [3–5]. Recently, the combination of atezolizumab (anti-PD-L1 antibody) and bevacizumab (anti-VEGF antibody) has demonstrated improved overall survival compared with TKI alone and has become the standard initial therapy for advanced HCC [6]. In addition, many ongoing trials are exploring new therapeutic approaches for HCC, including the combination of ICB and TKI or multiple ICB together [7–11]. However, the sequence of combined treatments requires further optimization, and proper biomarkers remain to be identified to predict the responsive subgroup of patients.

Sorafenib is the first TKI to receive approval for HCC patients [12], and it took more than a decade before lenvatinib was approved [13]. Later, two other TKIs, regorafenib and cabozantinib, were approved as second-line therapy after sorafenib [14]. These TKIs block receptor tyrosine kinase signaling and inhibit downstream Raf kinase activity in tumor cells and vascular endothelial cells, resulting in the inhibition of cancer proliferation and angiogenesis [4, 15, 16]. More interestingly, among these TKIs, both sorafenib and regorafenib were reported to induce tumoral ferroptosis [17]. However, in many cases, primary resistance or acquired resistance developed during TKI treatment largely limits their therapeutic efficacy. Previous studies have exclusively focused on tumoral intrinsic mechanisms conferring TKIs resistance, such as alternative pathways bypassing target inhibition, or alterations in drug transportation and metabolism [18, 19]. However, the effects of stimuli from tumor microenvironments, particularly immune cells, on TKIs responses are overlooked [20, 21].

IFNγ is the uppermost cytokine implicated in antitumor immunity, and the integrity of IFNγ signaling is required for the clinical efficacy of immunotherapies [22]. Besides its immunomodulatory effects on tumor-infiltrating immune cells like macrophages [23], dendritic cells [24], and T cells [23], IFNγ can exert its antitumor activity by directly inhibiting the proliferation of tumor cells and endothelial cells [25], or by promoting tumoral cell death. A high level of IFNγ was shown to induce apoptosis of non-small cell lung cancer [26]. By inhibiting the cystine uptake, CD8^+^ T cell-derived IFNγ could enhance ferroptosis of human melanoma cells [27]. IFNγ also promoted NK cell-mediated pyroptosis in target tumor cells by upregulating GSDMB expression [28]. In addition, co-treatment of IFNγ and TNFα induced caspase-8/FADD-mediated PANoptosis through nitric oxide production [29]. Here, we found that IFNγ augmented TKIs-induced HCC cell death, and the deficiency of IFNγ signaling impaired the antitumor efficacy of TKI in vivo. IFNγ synergized with TKI to induce GSDME-mediated pyroptosis by inhibition of the PERK branch of UPR. IFNγ-producing tumor-specific CD8^+^ T cells could also sensitize HCC cells to TKI. In GSDME-expressing HCC tumors, PD-1 blockade in combination with TKI exerted synergistic antitumor efficacy, which could be further improved by the PERK inhibitor.

## Results

### IFNγ potentiates TKI therapy for HCC

**Fig. 1:**
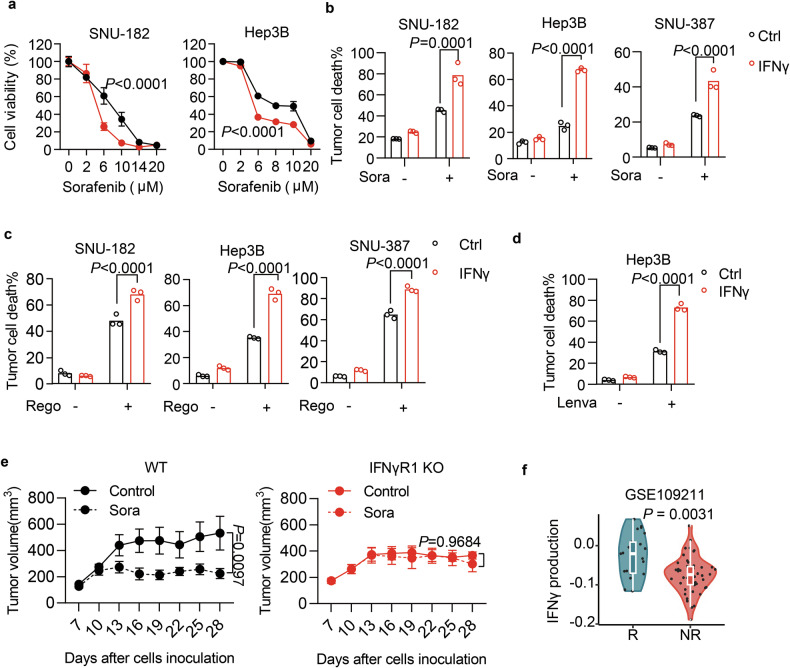
IFNγ sensitizes liver cancer cells to TKI therapy. **a** Cell viability of SNU-182 or Hep3B cells primed with recombinant IFNγ (10 ng/mL) followed by various concentrations of sorafenib for 24 h. **b** Cell death of indicated HCC cells primed with IFNγ (10 ng/mL) for 24 h, followed by sorafenib (8 μM or 15 μM) for 24 h. **c** The death of indicated HCC cells primed with IFNγ (10 ng/mL), followed by regorafenib treatment (15 μM or 20 μM) for 36 h. **d** Cell death of Hep3B cells primed with IFNγ, followed by lenvatinib (10 μM) for 24 h. **e** Tumor growth of wildtype (WT) or IFNγR1 deficient (KO) Hep-55.1C cells inoculated into C57BL/6 mice that were treated with vehicle control or sorafenib (20 mg/kg). *n* = 7–10 mice/group. **f** Violin plot comparing IFNγ production signatures between responders and non-responders of HCC patients who received sorafenib treatment (GSE109211). Data were shown as mean or mean ± SEM. *p*-values were calculated by two-way ANOVA (**a**–**e**), and the Mann–Whitney test (**f**).

Sorafenib was reported as a ferroptosis inducer with relatively weaker potency [30]. We previously identified that IFNγ could sensitize tumor cells to ferroptosis by down-regulating the expression of SLC7A11 [27]. Thus, we hypothesized that IFNγ would enhance the antitumor efficacy of sorafenib by inducing tumoral ferroptosis. Indeed, in several human HCC cell lines, including SNU-182, Hep3B, and SNU-387, we observed that pre-treatment with recombinant IFNγ augmented sorafenib to inhibit the cell viability (Fig. 1a) and to enhance cell death (Fig. 1b). In murine hepatocarcinoma Hepa1-6 and Hep-55.1C cell lines, IFNγ prime also enhanced sorafenib-induced cell death (Fig. S1a, b). Regorafenib was reported to induce ferroptosis as well, and we found that its cytotoxicity on HCC cells was enhanced by IFNγ (Fig. 1c). Interestingly, lenvatinib was able to induce cell death of Hep3B cells (Fig. 1d), which could be further enhanced by IFNγ.

To further investigate whether the TKI sensitivity could be regulated by IFNγ signaling in vivo, we established IFNγ receptor 1 (IFNGR1)-deficient Hep-55.1C cells and confirmed their disability to induce major histocompatibility complex class I expression in response to IFNγ (Fig. S1c). In vitro, IFNGR1 deficiency had no effects on cell proliferation (Fig. S1d) and cellular responses against sorafenib (Fig. S1e), regorafenib (Fig. S1f), or lenvatinib (Fig. S1g). IFNγ priming also failed to enhance sorafenib-induced cell death of IFNGR1-deficient Hep-55.1C cells (Fig. S1h). After their inoculation into immunocompetent C57BL/6 mice, wild-type tumors were responsive to sorafenib treatment, whereas IFNGR1-deficient tumors failed to respond (Fig. 1e), suggesting that the integrity of IFNγ signaling in HCC cells is required for TKI response in vivo. Furthermore, by analyzing gene expression in HCC tumor tissues from patients enrolled in the STORM trial [31], we observed that IFNγ production was associated with a better response to sorafenib (Fig. 1f). These results thus indicate that IFNγ can sensitize HCC cells to TKI-mediated cytotoxicity in vitro and in vivo.

### IFNγ and TKIs synergistically induce GSDME-mediated pyroptosis of HCC

**Fig. 2:**
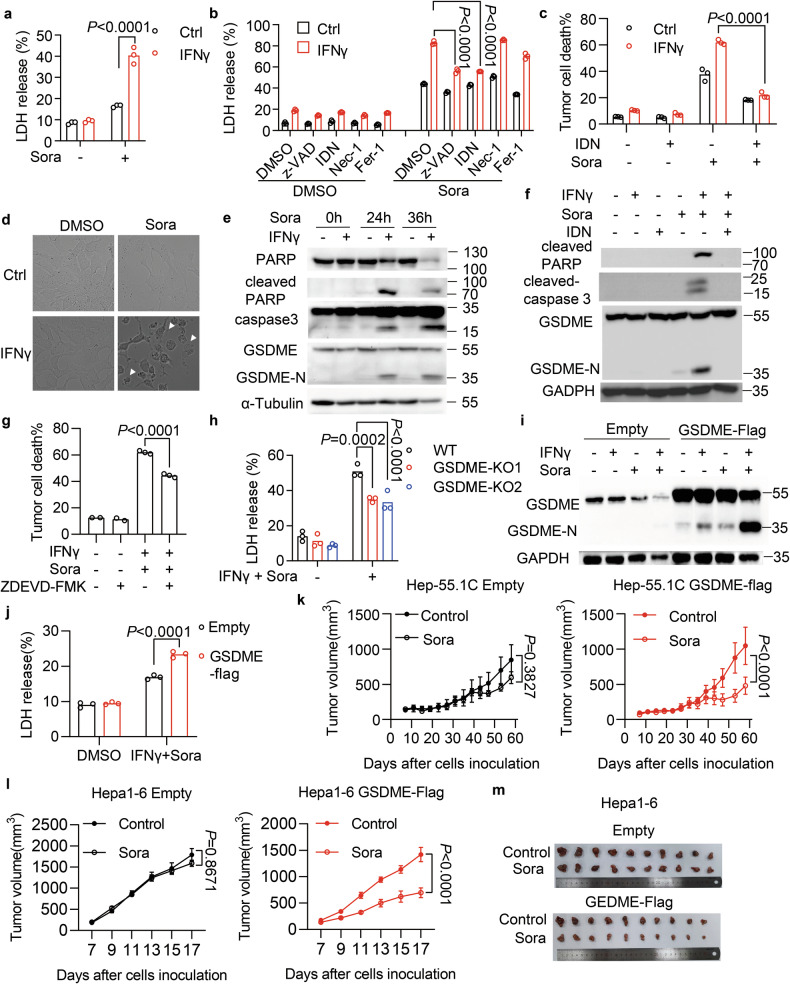
IFNγ synergizes with TKI to induce GSDME-mediated pyroptosis of HCC. **a** The content of LDH released from SNU-387 cells primed with IFNγ (10 ng/mL) for 24 h, followed by sorafenib (15 μM) for 24 h. **b** LDH released from Hep3B cells treated with IFNγ and sorafenib in the presence of cell death inhibitors including z-VAD (100 μM), IDN (20 μM), Nec-1(40 μM), or Fer-1 (10 μM). **c** Cell death of SNU-182 cells primed with IFNγ and then treated with sorafenib (15 μM) plus IDN (40 μM) for 48 h. **d** Representative images showing pyroptosis morphology with large bubbles from the plasma membrane in Hep3B cells treated with IFNγ and sorafenib. **e** Immunoblots of human PARP, caspase 3, and GSDME in IFNγ-primed Hep3B cells treated with sorafenib (8 μM) for 24 or 36 h. α-Tubulin served as a loading control. **f** Immunoblots of human cleaved-PARP, cleaved-caspase3, and GSDME in SNU-387 cells primed with IFNγ followed by sorafenib (15 μM) plus IDN (40 μM) for 24 h. **g** Cell death of SNU-182 cells primed by IFNγ, followed by sorafenib (15 μM) in the presence of caspase 3 inhibitor Z-DEVD-FMK (40 μM) for 48 h. **h** LDH released from WT or GSDME-deficient SNU-182 cells treated with IFNγ and sorafenib (12 μM) for 24 h. **i**, **j** Hep-55.1C cells stably expressing Empty or GSDME-Flag were treated with a combination of mIFNγ (10 ng/mL) and sorafenib (20 μM). GSDME cleavage was determined by immunoblotting (**i**), and LDH content in the culture medium was quantified (**j**). **k** Tumor growth of Empty or GSDME-Flag expressing Hep-55.1C cells inoculated in C57BL/6 mice that were treated with vehicle control or sorafenib (10 mg/kg). *n* = 6–7 mice/group. **l**, **m** Empty or GSDME-Flag expressing Hepa1-6 cells were inoculated in C57BL/6 mice and treated with vehicle control or sorafenib (10 mg/kg). Tumor growth was monitored over time for each group (**l**). Subcutaneous tumors were surgically removed and presented (**m**) at the endpoint. *n* = 10 mice per group. Data were shown as mean or mean ± SEM. *p*-values were calculated by one-way ANOVA (**g**) and two-way ANOVA (**a**–**c**, **h**, **j**–**l**).

Next, we tested whether ferroptosis was involved in the cell death induced by the combination of IFNγ and TKIs. Lipid peroxidation is a hallmark of ferroptosis, and it was found to be induced by sorafenib in Hep3B cells (Fig. S2a). However, a lipid peroxidation scavenger ferrostatin-1 failed to block the cell death induced by IFNγ and sorafenib (Fig. S2b), suggesting that ferroptosis may not be involved in our system. We then tried to pharmacologically define the type of cell death using pathway-specific modulators. Lactate dehydrogenase (LDH) release has been observed in the occurrence of multiple types of cell death that is mediated by the permeabilization of the plasma membrane [32–34]. We detected higher levels of LDH in the culture medium of SNU-387 and Hep3B cells upon IFNγ and sorafenib treatment (Figs. 2a and S2c). Again, ferrostatin-1 failed to block the LDH release (Fig. 2b). The RIP1 kinase inhibitor necrostatin-1 also failed to inhibit LDH release (Fig. 2b). Whereas, the pan-caspase inhibitors zVAD-fmk and IDN-6556 were able to reduce LDH release (Fig. 2b). Furthermore, the inhibition effect of IDN-6556 on cell death induced by IFNγ and sorafenib was confirmed by flow cytometry (Figs. 2c and S2d). These results indicate that IFNγ synergizes with sorafenib to induce caspase-dependent cell death.

We then noticed that the dead cells induced by IFNγ and sorafenib showed pyroptosis morphology, with cellular swelling and large bubbles from the plasma membrane (Fig. 2d). GSDME cleavage by caspase 3 represents the most common pyroptosis pathway in cancer cells [35, 36]. We found that IFNγ synergized with sorafenib to induce the activation of caspase 3 and cleavage of GSDME in SNU-182 (Fig. 2e), SNU-387 (Fig. 2f), and Hep3B (Fig. S2e) cells, which were all inhibited in the presence of IDN-6556 (Figs. 2f and S2f). Interestingly, IFNγ synergized with lenvatinib could also induce caspase 3 and GSDME-mediated pyroptosis in Hep3B cells (Fig. S2g). Furthermore, a caspase 3-specific inhibitor rescued the HCC cell death induced by IFNγ plus sorafenib (Fig. 2g). And GSDME deficiency in HCC cells (Fig. S2h and S2i) abrogated the LDH release induced by IFNγ and sorafenib (Figs. 2h and S2j). These results suggest that IFNγ and TKIs synergistically induce caspase 3 and GSDME-mediated pyroptosis of HCCs.

### GSDME ectopic expression sensitizes HCC to TKI in vivo

Pyroptosis is considered an immunogenic cell death [37], and GSDME overexpression in cancer cells has been shown to facilitate immune cell infiltration and to enhance antitumor immunity [38]. In murine Hep-55.1C and Hepa1-6 cells with low expression of GSDME, we ectopically expressed the Flag-tagged GSDME and found that IFNγ combined with sorafenib caused a much stronger cleavage of GSDME (Figs. 2i and S2k) and more release of LDH (Figs. 2j and S2l), compared to empty plasmid-expressing cells. We then inoculated Hep-55.1C cells expressing the empty vector or GSDME-Flag into immunocompetent C57BL/6 mice. Tumors with GSDME-Flag expression had an increased number of CD8^+^ T-cell infiltration and IFNγ production (Fig. S2m). We thus speculated that these GSDME-expressing tumors might become more sensitive to sorafenib. Indeed, we found that a low dose of sorafenib failed to inhibit the growth of control tumors, but significantly reduced the volume of GSDME-expressing Hep-55.1C tumors (Figs. 2k and S2n). Similarly, after inoculation of Hepa1-6 cells expressing GSDME-Flag into C57BL/6 mice, we observed that GSDME-overexpressing tumors exhibited increased sensitivity to sorafenib compared to control tumors expressing empty plasmid (Fig. 2l, m). These results indicate that GSDME expression in tumor cells enhances IFNγ production and sensitizes HCC tumors to TKI in vivo.

### UPR protects HCC from TKI-induced pyroptosis

**Fig. 3:**
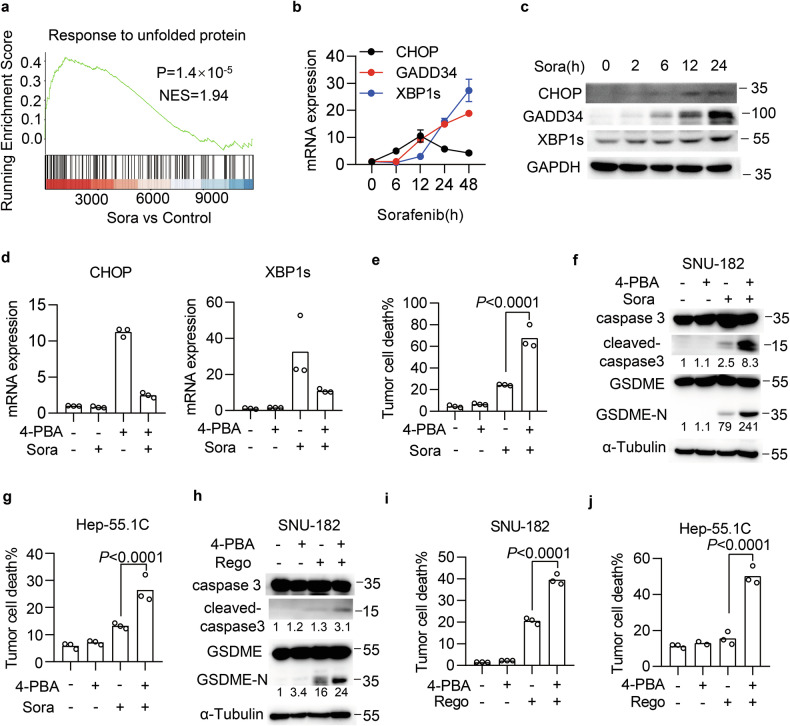
UPR protects HCC cells from TKI-induced pyroptosis. **a** Enrichment in the expression of RNA transcripts containing a response to unfolded protein upon sorafenib treatment in Hep3B cells. *n* = 3 for each condition. **b**, **c** Relative mRNA expressions and protein levels of CHOP, GADD34, and XBP1s in Hep3B cells following sorafenib treatment (8 μM) at indicated time points. **d**–**f** SNU-182 cells were treated with sorafenib in the presence of 4-PBA (1 mM) for 12 h (**d**) or 24 h (**e**, **f**). Relative mRNA expression of CHOP and XBP1s (**d**), percentage of cell death (**e**), and cleavage of caspase 3 and GSDME (**f**) were analyzed. **g** Cell death of Hep-55.1C cells treated with sorafenib (20 μM) in the presence of 4-PBA for 24 h. **h**, **i** SNU-182 cells were treated with regorafenib (15 μM) in the presence of 4-PBA (1 mM) for 36 h (**h**) or 65 h (**i**). The cleavage of caspase 3 and GSDME (**h**) and the percentage of cell death (**i**) were analyzed. **j**. Cell death of Hep-55.1C treated with regorafenib (15 μM) in the presence of 4-PBA for 65 h. Data were shown as mean or mean ± SEM. *p*-values were calculated by one-way ANOVA (**e**, **g**, **i**, **j**).

Meanwhile, we dissected the mechanism by which IFNγ synergizes with TKIs to induce pyroptosis. First, we focused on upstream signals that regulate sorafenib-induced cell death. Induction of endoplasmic reticulum (ER) stress and activation of the UPR have recently been linked to cell death in sorafenib-treated cancer cells [39, 40]. By analyzing the transcriptome of sorafenib-treated Hep3B cells (Supplementary Table 1), we found that sorafenib treatment indeed caused the enrichment of the UPR signature (Fig. 3a) and induced UPR downstream genes (Fig. S3a). We validated this regulation and found that the mRNA expressions and protein levels of CHOP, GADD34, and XBP1s increased in a time-dependent manner upon sorafenib treatment (Fig. 3b, c). We then investigated whether UPR activation contributes to sorafenib-induced pyroptosis. 4-Phenylbutyric acid (4-PBA) suppresses UPR activation by acting as a chemical chaperone to attenuate ER stress [41]. We found that 4-PBA efficiently blocked the induction of CHOP and XBP1s mRNA caused by sorafenib (Fig. 3d). Surprisingly, 4-PBA did not inhibit but rather enhanced the sorafenib-induced death of SNU-182 and Hep3B cells (Figs. 3e and S3b, c) and promoted the cleavage of caspase 3 and GSDME (Fig. 3f). In murine Hep-55.1C and Hepa1-6 cells, 4-PBA treatment also augmented sorafenib-induced cell death (Figs. 3g and S3d). Moreover, 4-PBA abrogated CHOP induction in response to regorafenib (Fig. S3e), and promoted the cleavages of caspase 3 and GSDME (Figs. 3h and S3f) and enhanced regorafenib cytotoxicity in both human (Fig. 3i) and murine HCC cells (Figs. 3j and S3g). These results suggest that activated UPR protects HCC cells from TKI-induced pyroptosis.

### Inhibition of the PERK branch of UPR sensitizes HCC to TKI therapy

**Fig. 4:**
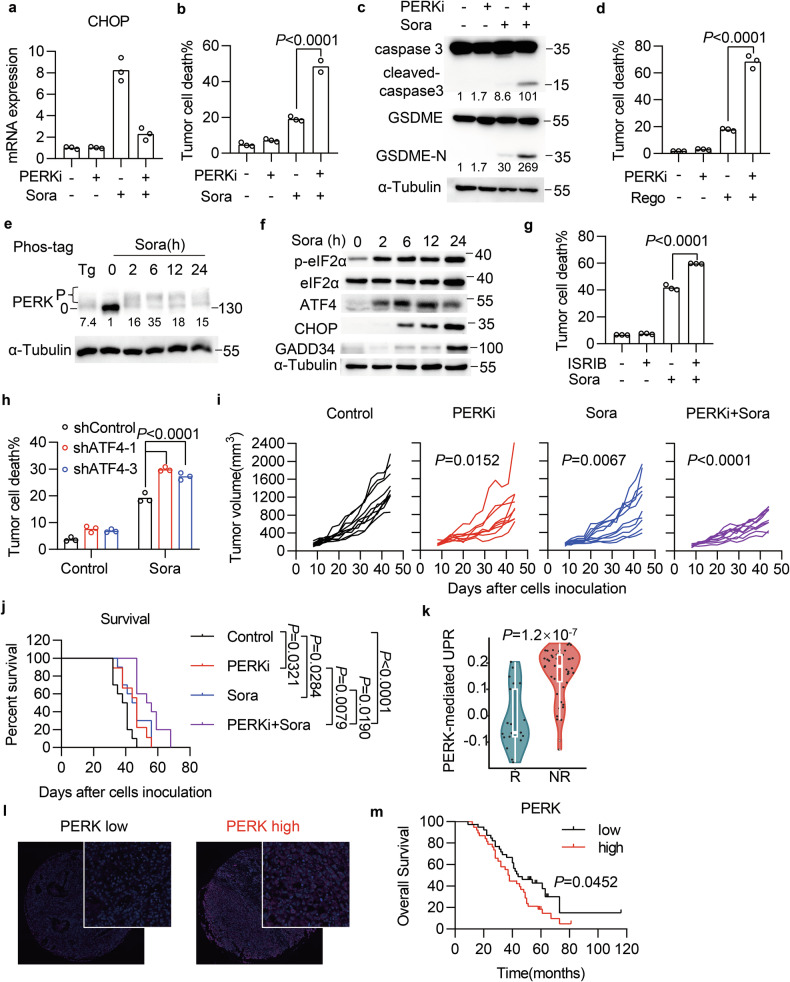
Inhibition of the PERK branch of UPR sensitizes HCC to TKI. **a–c** SNU-182 cells were treated with sorafenib in the presence of the PERK inhibitor GSK2606414 (PERKi, 1 μM) for 12 h (**a**) or 24 h (**b**, **c**). Relative CHOP mRNA levels (**a**), percentage of cell death (**b**), and cleavage of caspase 3 and GSDME (**c**) were analyzed. **d** Cell death of SNU-182 cells treated with regorafenib (20 μM) and PERK inhibitor for 65 h. **e**, **f** Immunoblots of PERK phosphorylation (**e**) and its downstream signals, including p-eIF2α, ATF4, CHOP, and GADD34 (**f**), in tumor cells treated with sorafenib for the indicated time points. **g** Cell death of SNU-182 cells treated with sorafenib in the presence of ISRIB (100 nM) for 65 h. **h** Cell death of SNU-182 cells expressing scramble or ATF4 shRNA following sorafenib treatment for 24 h. **i**, **j** C57BL/6 mice implanted with Hep-55.1C tumors were treated with GSK2606414 (50 mg/kg), sorafenib (20 mg/kg), or their combination. Individual tumor growth in each group was monitored over time (**i**). Kaplan–Meier survival curves of these animals were plotted (**j**). *n* = 10 mice per group. **k** Violin plot comparing PERK-mediated UPR between responders and non-responders of HCC patients who received sorafenib treatment (GSE109211). **l** Representative images of PERK staining in human HCC specimens. **m** Kaplan–Meier survival curves for patients with HCC with high (*n* = 38) or low (*n* = 39) PERK expression. Data were shown as means. *p*-values were calculated by one-way ANOVA (**b**, **d**, **g**), two-way ANOVA (**h**, **i**), log-rank test (**j**, **m**), and Mann–Whitney test (**k**).

The UPR pathway consists of three major signaling cascades initiated by inositol-requiring enzyme type 1α (IRE1α), activated transcription factor 6 (ATF6), and PERK located on the ER membrane [42]. Next, we attempted to elucidate the branches that contribute to sorafenib resistance. The 4μ8C is a selective inhibitor of IRE1α, which splices XBP1 mRNA encoding a transcription factor [43]. We found that 4μ8C efficiently blocked the splicing of XBP1 mRNA in sorafenib-treated SNU-182 cells (Fig. S4a), but failed to promote sorafenib-induced cell death (Fig. S4b). We also used the site 1 protease (S1P) inhibitor PF429242 to block the cleavage of ATF6 and found that it had no effect on sorafenib-induced cell death (Fig. S4c). Whereas the PERK inhibitor GSK2606414 completely blocked the induction of CHOP mRNA (Fig. 4a), ATF4 and GADD34 proteins (Fig. S4d), and enhanced cell death (Fig. 4b) and cleavages of caspase 3 and GSDME (Fig. 4c) in sorafenib-treated cells. The PERK inhibitor also augmented sorafenib-induced death of other human and mouse HCC cells (Fig. S4e). In addition, GSK2606414 synergized with regorafenib (Figs. 4d and S4f) or lenvatinib (Fig. S4g) to induce HCC cell death. These results suggest that the PERK branch of the UPR contributes to TKI resistance. Indeed, we found that sorafenib treatment caused the rapid phosphorylation of PERK (Fig. 4e), which then phosphorylated eukaryotic translation initiation factor 2α (eIF2α), resulting in the increased translation of ATF4 and the induction of downstream genes CHOP and GADD34 (Fig. 4f). To reinforce our findings, we further manipulated the activation of eIF2α and ATF4. The inhibitor ISRIB, which allosterically antagonizes the inhibitory effect of phosphorylated eIF2α [44], and ATF4 knockdown with shRNAs could enhance sorafenib-induced cell death (Fig. 4g, h). Taken together, these results indicate that PERK-mediated UPR activation protects HCC cells from TKI-induced pyroptosis.

We further evaluated the effect of PERK inhibition on TKI therapy in vivo. We inoculated Hep-55.1C cells into C57BL/6 mice and treated them with GSK2606414, sorafenib, or a combination of both. The single treatment caused slight suppression of tumor growth, while the combination therapy showed more potent antitumor activity to reduce tumor volume (Figs. 4i and S4h) and improve survival compared with either single treatment (Fig. 4j). We also explored the relationship between PERK expression and sorafenib response by analyzing the STORM data and found that high expression of the PERK-UPR signature genes was associated with sorafenib resistance (Fig. 4k). Meanwhile, analysis of the Kaplan–Meier plotter database showed that high levels of PERK mRNA were negatively correlated with overall survival in HCC patients (Fig. S4i). In addition, we evaluated PERK expression at the protein level by immunostaining a tissue microarray (TMA) containing 77 HCC specimens. All HCC specimens displayed PERK expression from low to high levels (Fig. 4l), and low levels of PERK protein correlated with improved overall survival (Figs. 4m and S4j). These data suggest that the PERK-mediated UPR pathway confers TKI resistance and correlates with poor outcomes of patients with HCC.

### IFNγ potentiates TKI therapy by attenuating PERK activation

**Fig. 5:**
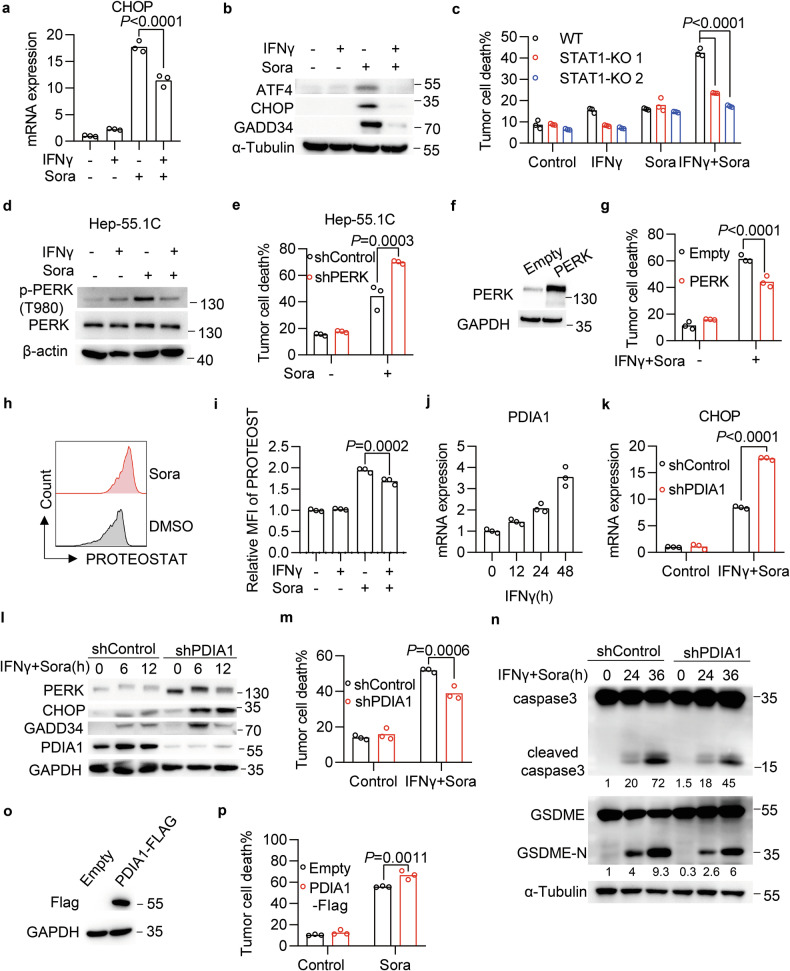
IFNγ attenuates PERK activation by inducing PDIA1 expression. **a**, **b** mRNA expression of CHOP (**a**) and protein expression of ATF4, CHOP, and GADD34 (**b**) in Hep3B cells primed with IFNγ, followed by sorafenib treatment for 12 h. **c** Cell death of Hep3B cells expressing scramble sgRNA (WT) or two independent sgRNAs targeting STAT1 (STAT1-KO1/KO2), treated with IFNγ plus sorafenib for 30 h. **d** Immunoblot of mouse p-PERK (T980) in Hep-55.1C cells primed with IFNγ, followed by sorafenib treatment (20 μM) for 1 h. **e** Cell death of Hep-55.1C cells expressing scramble or PERK shRNA after treatment with sorafenib for 56 h. **f**, **g** Immunoblot of PERK in Hep3B cells following transfection with an Empty or PERK-expressing plasmid (**f**). Cell death of these cells treated with IFNγ and sorafenib for 30 h (**g**). **h**, **i**. Flow cytometry analysis of PROTEOSTAT staining in Hep3B cells treated with sorafenib (**h**). Relative mean fluorescence intensity of PROTEOSTAT after treatment with IFNγ and sorafenib for 12 h (**i**). **j** PDIA1 mRNA level in Hep3B cells treated with IFNγ for different time points. **k**–**n** Hep3B cells expressing scramble or PDIA1 shRNA were treated with a combination of IFNγ and sorafenib. mRNA expression of CHOP (**k**) or protein expression of PERK, CHOP, GADD34, and PDIA1 (**l**) were analyzed by qPCR or immunoblotting. Cell death was quantified using flow cytometry (**m**). The cleavages of caspase3 and GSDME were determined using immunoblotting (**n**). **o**, **p** Immunoblot of Flag in SNU-182 cells expressing Empty or PDIA1-Flag. GAPDH served as a loading control (**o**). The above cells were treated with sorafenib (20 μM) for 48 h. Cell death was quantified using flow cytometry (**p**). Data were shown as means. *p*-values were calculated by one-way ANOVA (**a**, **i**) and two-way ANOVA (**c**, **e**, **g**, **k**, **m**, **p**).

Since IFNγ could augment sorafenib-induced pyroptosis, we hypothesized that IFNγ might interfere with the PERK-mediated UPR pathway to exert the sorafenib sensitization. Expectedly, we found that IFNγ priming attenuated sorafenib-induced expression of CHOP mRNA (Fig. 5a) but not XBP1s mRNA (Fig. S5a). In both human and murine HCC cells, IFNγ attenuated the protein induction of ATF4, CHOP, and GADD34 in response to sorafenib (Figs. 5b and S5b, c). In addition, ATF4 and CHOP induction mediated by regorafenib were reversed by IFNγ treatment (Fig. S5d). Thus, we conclude that IFNγ inhibits activation of the PERK branch of the UPR.

We then dissected the crosstalk between IFNγ signaling and PERK-mediated UPR pathway. Signaling by the IFNγ receptor activates the Janus kinase (JAK) and signal transducer and activator of transcription 1 (STAT1) to regulate the expression of interferon-stimulated genes (ISG) [45]. We found that JAK inhibitor I largely reversed IFNγ-mediated sorafenib sensitization of HCC cells (Fig. S5e). Genetic ablation of STAT1 (Fig. S5f) could also attenuate the cell death induced by IFNγ and sorafenib (Fig. 5c). And in STAT1-deficient cells, IFNγ failed to inhibit the sorafenib-induced expression of CHOP and GADD34 proteins (Fig. S5g). These results suggest that IFNγ-mediated attenuation of UPR requires the transduction of JAK-STAT1 signaling and the expression of certain ISGs. We also attempted to identify which node of the PERK pathway was inhibited by IFNγ. IFNγ treatment reduced sorafenib-induced phosphorylation of PERK but had no effect on the total PERK protein (Fig. 5d). Downregulation of PERK itself could enhance sorafenib-induced cell death (Figs. 5e and S5h), whereas ectopic PERK expression attenuated IFNγ-mediated sorafenib sensitization (Fig. 5f, g). These results suggest that IFNγ-mediated UPR attenuation acts on the node of PERK activation or its upstream, such as protein misfolding.

We then measured the level of misfolded proteins using PROTEOSTAT, a molecular rotor dye that specifically binds to misfolded proteins. Sorafenib significantly induced PROTEOSTAT signaling (Fig. 5h), which was partially reversed by IFNγ treatment (Fig. 5i), suggesting that IFNγ can reduce the accumulation of misfolded proteins. We thus paid attention to IFNγ-responsive genes that are able to regulate protein misfolding. From analysis of the transcriptome of IFNγ-treated Hep3B cells (Supplementary Table 1), we found that multiple family members of protein disulfide isomerases (PDIs) were induced by IFNγ (Fig. S5i). Among them, PDIA1 mRNA has the highest expression (Fig. S5j), and its upregulation induced by IFNγ was confirmed at both the mRNA (Fig. 5j) and protein levels (Fig. S5k). The knockdown of PDIA1 expression (Fig. S5l) elevated the levels of misfolded proteins (Fig. S5m) and augmented the activation of PERK in response to IFNγ plus sorafenib (Fig. 5k, l). In line with this, PDIA1 knockdown inhibited cell death (Fig. 5m) and cleavages of caspase 3 and GSDME induced by IFNγ plus sorafenib (Fig. 5n). In response to the combination of IFNγ and regorafenib, PDIA1 knockdown also reduced cell death (Fig. S5n) and cleavages of caspase 3 and GSDME (Fig. S5o). Additionally, we ectopically expressed PDIA1-Flag in HCC cells (Fig. 5o) and found it could enhance sorafenib-induced cell death (Fig. 5p). Taken together, these data indicate that IFNγ attenuates the PERK branch of the UPR and sensitizes HCC cells to TKI-induced cell death via induction of PDIA1.

### GSDME-expressing tumors respond to the combination of PD-1 blockade and TKI, whose efficacy is further enhanced by PERK inhibitor

**Fig. 6:**
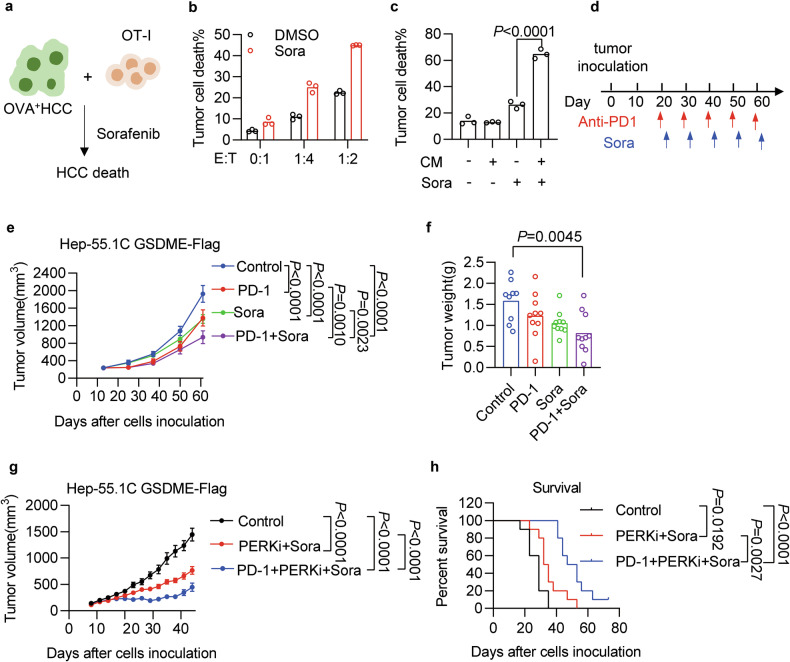
PD-1 blockade to activate T-cell response in combination with TKI exerts synergistic antitumor efficacy in GSDME-expressing tumors. **a** Experimental design for the co-culture of CFSE-labeled ovalbumin-expressing (OVA^+^) HCC and activated OT-I cells, followed by sorafenib treatment. **b** Cell death of Hep-55.1C-OVA cells co-cultured with OT-I cells, followed by sorafenib (20 μM) for 48 h. **c** Cell death of Hep-55.1C-OVA cells primed with conditioned medium (CM) from OT-I cells, followed by sorafenib (20 μM) for 60 h. **d–f** C57BL/6 mice implanted with GSDME-expressing Hep-55.1C tumors were treated with anti-PD-1 (100 μg), sorafenib (20 mg/kg), or their combination (**d**). Tumor growth was monitored over time for each group (**e**). Subcutaneous tumors were surgically removed, and their weights (**f**) were measured at the endpoint. n = 9 or 10 mice per group. **g**, **h** C57BL/6 mice implanted with GSDME flag-expressing Hep-55.1C tumors were treated with vehicle control, GSK2606414(50 mg/kg) and sorafenib (20 mg/kg) combination, anti-PD-1 (100 μg), GSK2606414, and sorafenib combination. Tumor growth was monitored over time for each group (**g**). Kaplan–Meier survival curves of these animals were plotted (**h**). *n* = 10 mice per group. Data were shown as mean or mean ± SEM. *p*-values were calculated by one-way ANOVA (**c**, **f**), two-way ANOVA (**e**, **g**), and log-rank test (**h**).

Since CD8^+^ T cells are a major source of IFNγ in the tumor microenvironment, we then tested whether tumor-specific CD8^+^ T cells could augment sorafenib sensitivity. We established ovalbumin (OVA) expressing Hep-55.1C cells, and co-cultured them with activated OVA-specific CD8^+^ T (OT-I) cells following with sorafenib treatment (Fig. 6a). OT-I and sorafenib synergistically induced tumor cell death (Fig. 6b). Similarly, the conditioned medium from OT-I cells could also enhance sorafenib-induced HCC cell death (Fig. 6c). Since GSDME ectopic expression caused increased tumoral T-cell infiltration (Fig. S2m) and ICB functions by recovering or enhancing the effector activity of CD8^+^ T cells, we speculated that ICB therapy might synergize with TKI therapy, particularly in GSDME-expressing HCC. We thus inoculated GSDME-Flag expressing Hep-55.1C cells into C57BL/6 mice and treated them with anti-PD-1 antibody followed by sorafenib (Fig. 6d). Compared with single treatment, the combination therapy showed more potent antitumor activity to reduce tumor volume (Figs. 6e and S6a) and tumor weight (Figs. 6f and S6b). We also evaluated the synergistic effect between PD-1 blockade and TKI therapy in control Hep-55.1C cells. The results showed that PD-1 blockade or sorafenib monotherapy could inhibit tumor growth, whereas their combination failed to further improve the therapeutic efficacy compared to either treatment alone (Fig. S6c, d).

Considering that PERK inhibitor sensitizes TKI therapy in vivo (Fig. 4i), we further speculated that the triple combination of PD-1 blockade, PERK inhibitor, and sorafenib might generate the most potent antitumor activity. To this end, we performed treatments on GSDME-expressing Hep-55.1C tumors with only three cycles using anti-PD-1 antibody, GSK2606414, and sorafenib. Compared with the control group, a double combination of GSK2606414 and sorafenib significantly inhibited tumor growth. While their triple combination showed more potent antitumor activity to reduce tumor volume (Fig. 6g) and improve animal survival (Fig. 6h) compared to the double combination. These data suggest a potential strategy for the combination therapy for GSDME-positive tumors utilizing checkpoint blockade, TKI, and PERK inhibitor.

## Discussion

The cytotoxicity of sorafenib across a wide range of cancer cell lines has been extensively studied. However, the type of cell death induced by sorafenib is still controversial [30, 46, 47]. We observed that IFNγ synergized with sorafenib to induce HCC pyroptosis. Although sorafenib was reported to induce ferroptosis in certain HCC cell lines [48] and IFNγ is able to promote ferroptosis [27], we eventually found that ferroptosis inhibitors failed to block the cell death induced by IFNγ plus sorafenib. Consistently, a recent study systematically tested the ability of sorafenib to induce ferroptosis and concluded that sorafenib failed to trigger ferroptosis across multiple cancer cell lines, including HCC [49]. We revealed that cell death induced by IFNγ plus sorafenib involved the cleavages of caspase 3 and GSDME. It has been recognized that previously identified apoptotic stimuli were able to induce pyroptosis through caspase 3 activation in GSDME-expressing cells [36]. These stimuli include many chemotherapeutic drugs, molecular targeted therapies, and cytotoxic lymphocytes such as NK and CD8^+^ T cells [35]. In terms of TKIs like sorafenib and regorafenib, GSDME-mediated pyroptosis is a previously unrecognized mechanism of their action.

Furthermore, pyroptosis is considered an immunogenic cell death to potentiate antitumor immunity [38, 50]. Since murine HCC cells barely express GSDME, we ectopically expressed mGSDME and found that it enhanced the infiltration of CD8^+^ T cells and the production of IFNγ in the tumor microenvironment, resulting in increased tumoral sensitivity to sorafenib. Similarly, Zhang et al. observed that ectopic GSDME expression in murine 4T1 and B16 tumors increased NK cell infiltration and enhanced effector functions of NK and CD8^+^ T cells [38]. Mechanistically, enhanced phagocytosis of GSDME-expressing tumor cells by macrophages might contribute to increased lymphocyte infiltration [38]. Meanwhile, the combination of BRAF inhibitor and MEK inhibitor was shown to induce GSDME-dependent pyroptosis in mouse melanoma cells and increase T cell infiltration [51]. More recent studies have revealed the immunostimulatory function of multiple pyroptosis inducers, including a mesothelin-targeting antibody drug conjugate, oncolytic parapoxvirus ovis (ORFV), and its recombinant therapeutic derivatives [52]. Our results suggest that GSDME-expressing tumors have a better response to sorafenib alone and its combination with PD-1 blockade. We thus proposed that TKIs in combination with ICB might be an effective approach to improve their therapeutic efficacy in HCC patients whose tumors express GSDME.

ER stress-activated UPR triggers a set of gene expressions to restore ER homeostasis and promote cell survival. However, if ER stress is not alleviated, cells undergo apoptosis. The UPR is mediated by three branches of ER stress sensors, IRE1α, PERK, and ATF6 [42]. An earlier study showed that sorafenib induced IRE1α- and PERK-mediated UPR in human leukemia cells U937, and knockdown of IRE1α or inhibition of PERK could reduce sorafenib-induced apoptosis [16]. Sorafenib-induced UPR was also observed in HepG2 cells [53], and co-treatment with the ER stress inhibitor 4-PBA could increase sorafenib-induced apoptosis [54]. Consistently, we found that 4-PBA inhibited sorafenib-induced UPR, but enhanced cell death. The PERK/p-eIF2α/ATF4 branch of the UPR functions as an adaptive defense mechanism against the cytotoxicity of TKI, including sorafenib and regorafenib. And clinically, PERK expression is negatively correlated with the overall survival of patients with HCC. In an animal model, we found that the PERK inhibitor could synergize with sorafenib to exert antitumor activity. Moreover, its supplementation further potentiated the combinational therapy of PD-1 blockade and sorafenib. We thus proposed a triple combination strategy for HCC therapy.

In addition, we revealed a crosstalk between IFNγ signaling and PERK-mediated UPR pathway. IFNγ priming attenuated the activation of the PERK branch of UPR in response to TKI stimulation, and inhibition of PERK in turn promoted TKI-induced cell death. In contrast, previous studies reported that IFNγ could induce UPR to facilitate apoptosis in lung cancer cells or in conjunctival goblet cells [55, 56], but the molecular mechanism by which UPR is activated by IFNγ has not been revealed. We identified the negative regulation of IFNγ-STAT1-IRF1 signaling on PERK-mediated UPR in HCC. Mechanistically, the induction of an interferon-stimulated gene (ISG), PDIA1, mediated the attenuation of PERK phosphorylation. PDIA1 can function as a chaperone to support protein folding in the ER. Knockdown of PDIA1 increased TKI-induced cell death in HCC. Notably, the PDIA1 inhibitor has been shown to synergize with regorafenib to induce cell death [57]. Thus, PDIA1 is a potential target to overcome the TKI resistance.

Taken together, our results identify that IFNγ signaling is required for the response to TKI therapy. IFNγ attenuates the activation of PERK-mediated UPR and synergizes with TKI to induce GSDME-mediated pyroptosis. ICB therapy-activated IFNγ-producing CD8^+^ T cells can also sensitize HCC to TKI therapy, and the combination of ICB and TKI represents a promising approach for the treatment of patients with HCC, particularly in the subgroup with GSDME expression.

## Materials and methods

### Reagents

Sorafenib, 4-Phenylbutyric acid (4-PBA), GSK2606414, 4μ8C, PF429242, ISRIB, Ferrostatin-1 (Fer-1), and Baricitinib were purchased from MedChemExpress. Regorafenib and Lenvatinib were purchased from APExBIO. Necrostatin-1 (Nec-1) and Z-VAD-FMK (Z-VAD) were purchased from Cayman Chemical. Emricasan (IDN-6556) was purchased from Selleck Chemical. BODIPY™ 581/591 C11 was purchased from Thermo Fisher Scientific.

### Cell culture

Human HEK 293T/17 (CRL-11268, RRID: CVCL_1926) cells were obtained from the American Type Culture Collection (ATCC). The human hepatocarcinoma cell lines SNU-182 (SCSP-5047, RRID: CVCL_0090), Hep3B (SCSP-5045, CVCL_0326), and SNU-387 (SCSP-5046, RRID: CVCL_0250) and the murine hepatocarcinoma cell line Hepa1-6 (SCSP-512, RRID: CVCL_0327) were obtained from the China Center for Type Culture Collection (CCTCC). The murine Hepatoma cell line Hep-55.1C (CLS-400201, RRID: CVCL_5766) was purchased from the CLS Cell Lines Service. SNU-182, SNU-387, and Hepa1-6 were cultured in RPMI-1640 medium (Procell), Hep3B cells were cultured in MEM medium (Procell), and HEK 293T/17 and Hep-55.1C cells were cultured in DMEM medium (Procell). All culture media were supplemented with 10% FBS (Cegrogen Biotech). Hepa1-6 cell line was authenticated using short tandem repeat (STR) analysis, and other cell lines were not authenticated. All the cell lines were routinely tested for the presence of mycoplasma using a PCR-based method.

### Cell viability assay

The cells were plated in flat-bottom 96-well plates (3 × 10^3^ cells/well). Cells were cultured with or without IFNγ (10 ng/mL, R&D, 285-IF) for 24 h, followed by treatment with different compounds or DMSO. At the experimental endpoints, CCK8 detection solution (Abbkine) was added, and the absorbance was detected using a microplate reader (wavelength 450 nm) after 1–4 h of incubation. The percentage of tumor cell viability was calculated according to the manufacturer’s protocol.

### LDH release assay

The cells were seeded in flat-bottom 96-well plates (3 × 10^3^ cells/well). Cells were pretreated with or without IFNγ (10 ng/mL, R&D) for 24 h, followed by treatment with different compounds as indicated. At the experimental endpoints, LDH release in each well was calculated according to the manufacturer’s instructions (Cytotoxicity LDH Assay Kit; Dojindo). First, a final volume of 10% lysate was added to one well of each drug concentration treatment group, and the other wells were filled to the same volume with the medium. The plate was incubated at 37 °C for 30 min and centrifuged at 250×*g* for 2 min. The 100 μL of each supernatant was transferred to a new 96-well plate containing 100 μL of the working solution. The absorbance of each well was measured at a microplate reader (wavelength of 490 nm). LDH release rate (%) = (OD_Experi__ment_ − OD_Blank_)/(OD_Control_ − OD_Blank_) × 100%. The lysate-treated wells were used as controls for each drug concentration treatment group, and the blank wells were the group with only medium added.

### Cell death, lipid peroxidation, and immune profiling by flow cytometry

For cell death assays, tumor cells (2–5 × 10^4^ cells/well) were seeded into 24-well plates. Cells were treated with IFNγ (10 ng/mL) followed by treatment with different compounds for the indicated times. Both the cells and their supernatants were collected, centrifuged, and resuspended in phosphate-buffered saline (PBS) containing 1 μg/mL propidium iodide (PI) or 7-AAD. The cells were incubated at RT for 10 min and analyzed immediately by flow cytometry (BD FACSVerse, New Jersey, USA). PI or 7-AAD positive population is shown as the percentage of dead cells.

To analyze the lipid peroxidation of tumor cells, cells (2–5 × 10^4^ cells/well) were seeded into 24-well plates. Cells were treated with IFNγ (10 ng/mL) followed by treatment with different compounds for the indicated times. Subsequently, the cells were harvested through trypsinization, centrifugation, and resuspended in 300 μL of PBS containing 5 μM BODIPY 581/591 C11. The cells were incubated for 20 min at 37 °C in a tissue culture incubator, washed, and resuspended in 200 μL of PBS. They were then immediately analyzed on a flow cytometer. The mean fluorescence intensities (MFI) from the PE channel (reduced C11) and the FITC channel (oxidized C11) were monitored, and their ratio was calculated for each sample. The data were normalized to the control sample, as demonstrated by the relative lipid ROS.

To analyze the infiltration of immune cells, fresh tumor tissue from the animal models was harvested and ground into a single-cell suspension through a strainer (70 μm). Tumor-infiltrating lymphocytes (TILs) were enriched by density gradient centrifugation using lymphocyte separation medium (MP Biomedicals) and resuspended in 1 mL culture medium supplemented with PMA (20 ng/mL), Ionomycin (1 μg/mL), Brefeldin A (1: 1000), and Monensin (1:1000) a 37 °C incubator. The cells were then stained with Zombie NIR dye at RT for 15 min. After washing with PBS, the cells were stained with surface markers (anti-CD45 (30-F11, 103133; RRID: AB_10899570), anti-CD3 (145-2C11, 562600; RRID: AB_11153670), anti-CD4 (GK1.5, 100434; RRID: AB_893330), anti-CD8 (53-6.7,100705; RRID: AB_312745)) at RT for 20 min. Then, cells were washed and permeabilized in 200 μL Fixation/perm solution (Biolegend, 420801) at room temperature for 20 min, washed with the Perm/Wash buffer (Biolegend, 421002), and then stained with anti-IFNγ (XMG1.2, 505809; RRID: AB_315403). The cells were then washed twice and suspended in 200 μL PBS for flow cytometry analysis. All antibodies were purchased from BioLegend. Flow cytometry data were collected using a flow cytometer and analyzed using FlowJo X software.

### Immunoblotting

Tumor cells were harvested and lysed in radioimmunoprecipitation assay (RIPA) buffer (Thermo Fisher Scientific) supplemented with 1× protease inhibitor cocktail (MedChemExpress, HY-K0010) and phosphatase inhibitor cocktail (MedChemExpress, HY-K0022, HY-K0023) for 30 min on ice. The supernatants were collected by centrifugation for 15 min (12,500 rpm, 4 °C). The protein concentration was quantified using a BCA Protein Assay Kit (Thermo Fisher Scientific, 23225). Total protein in the loading buffer (Thermo Fisher Scientific) was denatured at 95 °C for 10 min. Proteins were separated via SDS-polyacrylamide gel electrophoresis and transferred to a 0.2 μm PVDF membrane (Millipore). After blocking for 1 h at RT with 5% nonfat dry milk or 5% BSA in TBS containing 0.1% Tween-20, the blots were incubated with specific primary antibodies overnight at 4 °C, then with HRP-conjugated secondary antibodies (Thermo Fisher Scientific) for 1.5 h at RT. The luminescent signal was detected with a chemiluminescent HRP substrate (Millipore) using a ChemiDoc Imaging System (Tanon-5200 Multi, China). The following primary antibodies were used: anti-PARP (CST, 9542, 1:1000, RRID: AB_2160739), anti-cleaved PARP (CST, 5625, 1:1000, RRID:AB_10699459), anti-GSDME (abcam, ab215191, 1:1000, RRID:AB_2737000), anti-caspase 3 (Santa Cruz, sc-56053, 1:1000, RRID:AB_781826),anti-XBP1S(ABclonal, A22546, 1:1000, RRID:AB_3665308), anti-phospho-PERK (Invitrogen, MA5-15033, 1:1000, RRID:AB_10980432), anti-PERK (Proteintech, 20582-1-AP, 1:1000,RRID:AB_10695760), anti-eIF2α (CST, 5324, 1:2000, AB_10692650), anti-EIF2S1 (phospho S51) (Abcam, ab32157, 1:1000, RRID:AB_732117), anti-ATF4 (Santa Cruz, sc-390063, 1:1000,RRID:AB_2810998), anti-CHOP (CST, 5554, 1:1000, RRID:AB_10694399), anti-GADD34 (Proteintech, 10449-1-AP, 1:1000, RRID:AB_2168724), anti-PDIA1 (Proteintech, 11245-1-AP, 1:1000, RRID:AB_2298937), anti-GAPDH (Proteintech, 60004-1-1g, 1:5000, RRID: AB_2107436) and anti-alpha tubulin (Proteintech, 11224-1-AP, 1:5000, RRID:AB_2210206), anti-beta actin (Santa Cruz, sc-47778, 1:1000, RRID:AB_626632).

### Quantitative PCR analysis

Total RNA was extracted from cultured cells or tumor tissue of experimental mice using RNAiso Plus (TaKaRa, 9108), followed by cDNA synthesis using PrimeScript RT Master Mix (TaKaRa, RR036A) with poly-dT and random hexamer primers. Quantitative PCR (qPCR) was conducted using PowerUp SYBR Green Master Mix (Thermo Fisher Scientific, 4309155) on a QuantStudio® 3 Real-Time PCR System (Thermo Fisher Scientific, Waltham, MA, USA). The following primers were used for gene expression quantification: human GAPDH forward: TGGTATCGTGGAAGGACTC, human GAPDH reverse: AGTAGAGGCAGGGATGATG; human CHOP forward: GCACCTCCCAGAGCCCTCACTCTCC; human CHOP reverse: GTCTACTCCAAGCCTTCCCCCTGCG; human GADD34 forward: GTACCTGGAGAGAAGCCACC, human GADD34 reverse: GAGAAGCGCACCTTTCTGG; human XBP1s forward: TGCTGAGTCCGCAGCAGGTG, human XBP1s reverse: GCTGGCAGGCTCTGGGGAAG; human PDIA1 forward: GGCTATCCCACCATCAAGTTC, human PDIA1 reverse: TCACGATGTCATCAGCCTCTC. The qPCR products were confirmed as a single specific band by gel electrophoresis. Endogenous GAPDH expression quantification was used as a normalization control. The 2^−ΔΔCT^ method was used to calculate the relative mRNA change levels.

### Generation of knockout and knockdown cells

Specific gene knockout tumor cell lines were generated using CRISPR-Cas9 technology. Single guide RNA (sgRNA) or scrambled gRNA was synthesized and cloned into the lentiCRISPR v2 vector (Addgene, 52961). The plasmids were validated by Sanger sequencing and purified for virus production in HEK 293T/17 cells. The plasmid was transfected into HEK 293T/17 cells, together with psPAX2 and pMD2.G plasmids, using Lipofectamine 2000 (Thermo Fisher Scientific). The culture medium was changed 12 h after transfection, and the supernatant containing the lentivirus was collected after 3 days. Then, the tumor cell lines were infected with lentiviruses expressing each sgRNA. After 2–3 days of infection, cells were selected with puromycin for 3–4 days, and the efficiency of knockout in tumor cells was identified by immunoblotting. Single-cell clones were screened and expanded. Multiple validated deficient clones were pooled for the experiments. Guide RNA sequences to target human STAT1 were GAGGTCATGAAAACGGATGG or GGTGGCAAATGAAACATCAT; Guide RNA sequences to target human GSDME were: TAAGTTACAGCTTCTAAGTC or CAGTTTTTATCCCTCACCCT; Guide RNA sequences to target mouse IFNγR1 were ATTAGAACATTCGTCGGTAC.

ATF4, PDIA1, and PERK knockdown cells were generated using lentivirus-mediated expression of short hairpin RNAs (shRNAs). The shRNA oligonucleotide was cloned into the pLKO.1 vector (Addgene, 10878). The experimental procedure was similar to that previously described. The lentiviruses were obtained and collected. Tumor cells were infected with lentivirus, and the positive cells were selected with puromycin for 3–5 days. The efficiency of knockdown was validated by qPCR or immunoblotting. The following shRNA-targeting genes were used in this study: Human shATF4-1: GGAGATCCAGTACCTGAAAGA; Human shATF4-3: GCCTAGGTCTCTTAGATGATT; Human shPDIA1: TGCTGTTCTTGCCCAAGAGTG; Mouse shPERK: CCTCTACTGTTCACTCAGAAA.

### Generation of PERK, GSDME, and PDIA1 ectopic expressing cells

A plasmid containing the open reading frame of human PERK (NM_004836.5) was purchased from Sino Biological, and the cDNA of human or mouse GSDME and human PDIA1 was amplified from genomic DNA. These cDNAs were subcloned into the lentivector pLenti-CMV-3xFlag using the ClonExpress II One-Step Cloning Kit (Vazyme, C112). Plasmid DNA was validated by sequencing and was purified for transfection experiments. An empty pLenti-CMV-3xFlag vector was used as a control. Lentiviruses were produced and harvested, as described above. Tumor cells were infected with lentivirus, and positive cells were screened with puromycin for 3–5 days. Ectopic expression of PERK, GSDME, or PDIA1 genes was validated by immunoblotting.

### OT-I cells and tumor cells co-culture

Spleens from OT-I mice, C57BL/6-Tg (TcraTcrb) 1100Mjb/J (RRID: IMSR_JAX:003831), were isolated, and after centrifugation, the cells were resuspended in 5 mL red blood cell lysis buffer (Thermo Fisher Scientific) for 5 min on ice. The lymphocytes were then pelleted and resuspended at 2 × 10^6^ cells/mL in RPMI culture medium containing 2.5 µg/mL OVA_257-264_ peptide (GenScript, RP10611CN), 10 ng/mL mouse recombinant IL-2 (SinoBiological,51061-nmae), and 50 µM beta-mercaptoethanol (Sigma, M3148). The cells were stimulated and expanded for 3 days. CD8^+^ T cells were then isolated by negative selection using the EasySep^TM^ Human CD8^+^ T Cell Enrichment Kit (StemCell Technologies,19053).

Exponentially growing adherent cells were trypsinized and then labeled with CFSE (1 μM) according to the manufacturer’s protocol. Hep-55.1C-OVA^+^ cells were then plated in flat-bottomed 24-well plates (5 × 10^4^/well). After cell attachment, T cells at the indicated E:T ratios or T cell culture medium were added for co-culture for 24 h, then sorafenib or dimethyl sulfoxide (DMSO) was added to the plates. After incubation, the cells were collected by trypsinization and centrifugation and stained with 7-AAD for 10 min. Tumor cell death was analyzed by gating the CFSE^+^ 7-AAD^+^ cell population using a flow cytometer.

### Animal experiments

The 6–8-week-old wild-type C57BL/6(RRID: MGI:2159769) male mice were obtained from the Beijing Vital River Laboratory Animal Technology Co., Ltd. All animals were housed and bred under specific pathogen-free conditions at the animal facility of Huazhong University of Science and Technology. Animal studies were performed in accordance with the Institutional Animal Care and Use Committee of Huazhong University of Science and Technology (IACUC Number:4593).

For the murine Hep-55.1C tumor model, 5 × 10^6^ Hep-55.1C cells were subcutaneously inoculated into the right armpit of the male C57BL/6 mice. Tumor-bearing mice were randomly divided into four groups. To test the effect of PERK inhibition on the sorafenib response, the PERK inhibitor GSK2606414 (50 mg/kg) was administered orally, followed by sorafenib (20 mg/kg) on the indicated days. For combination therapy with PD-1 blockade and sorafenib, tumor-bearing mice were treated with anti-PD-1 antibody (100 μg/mouse, BioXCell), sorafenib (20 mg/kg), or their combination. The anti-PD-1 antibody was administered intraperitoneally. To explore the effect of IFNγ signaling or GSDME expression on sorafenib response, wild-type or IFNγR1-deficient Hep-55.1c cells, or Empty or GSDME-overexpressing Hep-55.1c cells, were subcutaneously injected into C57BL/6 mice. Mice were orally treated with sorafenib (10 mg/kg) or vehicle every two days. For the murine Hepa1-6 tumor model, 5 × 10^6^ Empty or GSDME-overexpressing Hepa1-6 cells were subcutaneously inoculated into the right armpit of the male C57BL/6 mice. Tumor-bearing mice were treated with sorafenib (10 mg/kg) or vehicle every two days. Tumor volume was measured every two or three days when tumors were palpable using calipers and calculated by length × width^2^/2. Tumor tissues were surgically removed, and their weights were measured at the endpoint. Mice were euthanized when their tumor volume reached 2000 mm^3^ or when they reached a humane endpoint, such as rapid weight loss, hunched back posture, or difficulty in feeding. Blinding was not performed during the experiment or when assessing the outcomes.

### RNA sequencing

Total RNA was extracted using RNAiso Plus (Takara), according to the manufacturer’s instructions. RNA concentration and purity were quantified using NanoDrop 2000 (Thermo Fisher Scientific), and RNA integrity was evaluated using the RNA Nano 6000 Assay Kit of the Agilent Bioanalyzer 2100 system (Agilent Technologies, California, USA). Oligo(dT)-bound magnetic beads were used for the mRNA purification. After reverse transcription, incubation with Tailing Mix and RNA Index Adapters, purification with Ampure XP Beads, and formation of single-stranded circular DNA, the final library was amplified to produce DNA nanoballs (DNBs). Sufficient-quality DNBs were then loaded onto patterned nanoarrays using the high-intensity DNA nanochip technique and sequenced by combinatorial probe-anchor synthesis on the Illumina HiSeq 2000 platform (BioMarker, China). Raw fastq format reads were first processed using in-house Perl scripts, and the Q20, Q30, GC content, and sequence duplication levels of the clean data were simultaneously calculated for quality control purposes. Qualified clean reads were mapped to the reference genome sequence using the Hisat2 tool. Gene expression levels were estimated using FPKM, which represents fragments per kilobase of transcript per million fragments mapped. Differential expression analysis of the two groups was performed using DESeq2 software. Based on these differentially expressed genes, Gene Ontology (GO) and Kyoto Encyclopedia of Genes and Genomes (KEGG) enrichment analyses were performed using the cluster Profiler R package. The processed transcriptomic data have been included in Supplementary Table 1.

### Unfolded protein load assay

Tumor cells (2–5 × 10^4^ cells/well) were seeded in 24-well flat-bottomed plates. After allowing attachment overnight, various drug treatments were applied, and the unfolded protein load was detected using PROTEOSTAT® Aggresome Detection Kit (Enzo, ENZ-51035-0025) according to the manufacturer’s protocol. The cells were digested with trypsin, washed twice with PBS, and then fixed with 4% paraformaldehyde for 30 min at RT. The cells were then incubated with the permeabilizing solution for 30 min on ice. Cells were resuspended in assay buffer containing PROTEOSTAT Aggresome dye and incubated for 30 min at RT in the dark. Fluorescence intensity was measured by flow cytometry (BL3 channel) after incubation, reflecting the extent of protein unfolding.

### Multiplexed immunofluorescence assay on TMA

HCC TMAs were purchased from Shanghai OUTDO BIOTECH Co., Ltd. (Shanghai, China). The cohort (HLivH180Su30) included 77 patients with cancerous tissue. Briefly, the TMA slide was deparaffinized and repaired with EDTA (pH 9.0) antigen repair solution at high temperature and pressure. After the repair solution was cooled to room temperature, the TMA slide was blocked with 3% H_2_O_2_ for 30 min at RT. TMA was then placed in TBST, and circles were drawn with an immunohistochemistry pen, followed by incubation with 10% goat serum at 37 °C for 30 min. The primary antibody PERK (Proteintech, 20582-1-AP, 1:150) was incubated at 4 °C overnight, and the secondary antibody goat anti-rabbit IgG H&L (HRP) was incubated at 37 °C for 45 min. Cy5 stock solution was added to 0.003% H_2_O_2_ (1:400) for TSA staining and incubated at room temperature for 10 min. DAPI (1:500) was used to stain the nuclei at RT for 5 min. The blocked slides were then examined under a microscope, and images were captured and analyzed. Images were screened using an automated slide-scanning platform (3DHistech, Pannoramic MID, Hungary). Specific staining of the tumor area at each tissue core in the TMA was selected with the help of experienced pathologists. Three fields from each core were randomly selected. The staining intensity was measured using the average gray value of the tumor areas by ImageJ. Tumor tissues were categorized into low and high PERK expression groups based on the median average gray value of each core.

### Statistical analysis

Statistical analyses were performed using GraphPad Prism 8.0.1 (GraphPad Software, Inc.), and the data were presented as the mean or mean ± SEM of the indicated numbers. No statistical methods were used to predetermine sample size. Data met the assumptions of homogeneity of variance and normality. Unpaired two-tailed Student’s *t*-tests were used for comparisons between two independent groups. ANOVA models were used for comparisons between multiple experimental groups. The log-rank test was used for survival curves. Statistical significance was set at *p* < 0.05.

## Supplementary information

Supplementary figures and figure legends

Supplementary Table 1

Unprocessed original images of Western blots

## Data Availability

The raw RNA-seq data generated in this study have been deposited in the Genome Sequence Archive for Humans (GSA-Human) at the National Genomics Data Center (China) under accession number HRA011334 (https://ngdc.cncb.ac.cn/search/specific?db=hra&q=HRA011334). All other supporting data, materials, and analytical protocols are available from the corresponding authors upon reasonable request.
