# Supplementary material for: IFNγ augments TKI efficacy by alleviating protein unfolding stress to promote GSDME-mediated pyroptosis in hepatocellular carcinoma
Source: Cell Death Dis. 2025 Jul 11;16(1):512. doi: 10.1038/s41419-025-07839-y (PMC12254303; doi:10.1038/s41419-025-07839-y)
Supplement: Supplementary file 1 — Supplementary figures and figure legends [file 41419_2025_7839_MOESM1_ESM.docx]

**SUPPLEMENTARY FIGURES AND FIGURE LEGENDS**


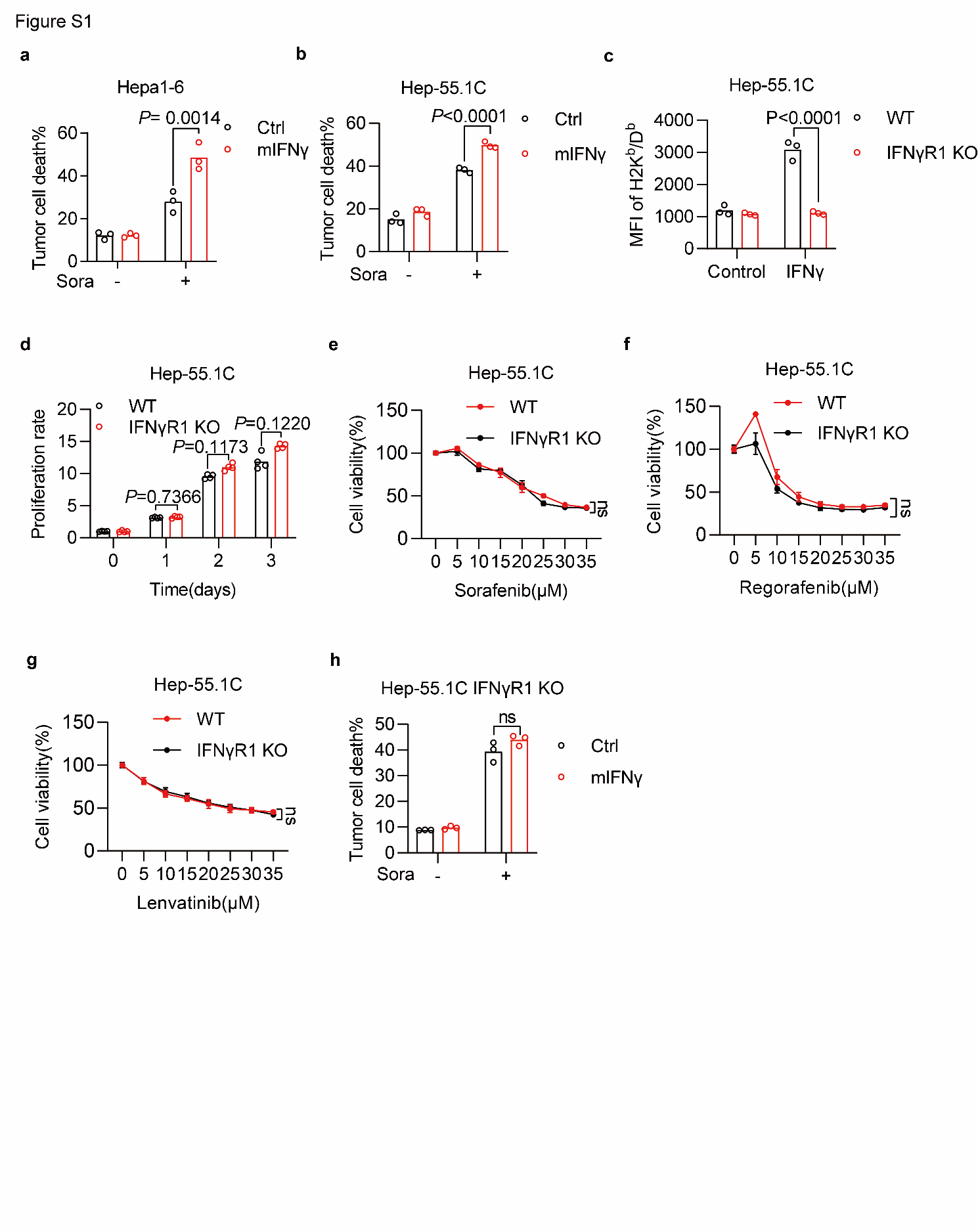


**Figure S1. IFNγ sensitizes liver cancer cells to TKI.**

**a, b**. Cell death of murine Hepa1-6 (**a**) or Hep-55.1C (**b**) cells primed with mIFNγ (10 ng/mL) and followed with sorafenib (**a**, 15 μM or **b**, 20 μM) for 48 h.

**c**. Cell surface H2K^b^/D^b^ expression in IFNγ-treated WT or IFNγR1 KO Hep-55.1C cells.

**d**. The relative proliferation rate of IFNγR1-KO and WT Hep-55.1C cells during a 3-day *in vitro* culture period.

**e-g**. Relative cell viabilities of IFNγR1-KO and WT Hep-55.1C cells treated with sorafenib (**e**), regorafenib (**f**) or lenvatinib (**g**) at different concentrations for 12 h, 24 h or 72 h.

**h**. Cell death of IFNγR1-KO Hep-55.1C cells primed with mIFNγ (10 ng/mL) for 24 h followed by sorafenib (20 μM) for 48 h.

Data were shown as mean or mean ± SEM. p values were calculated by one-way ANOVA (d) and two-way ANOVA (a-c, e-h).


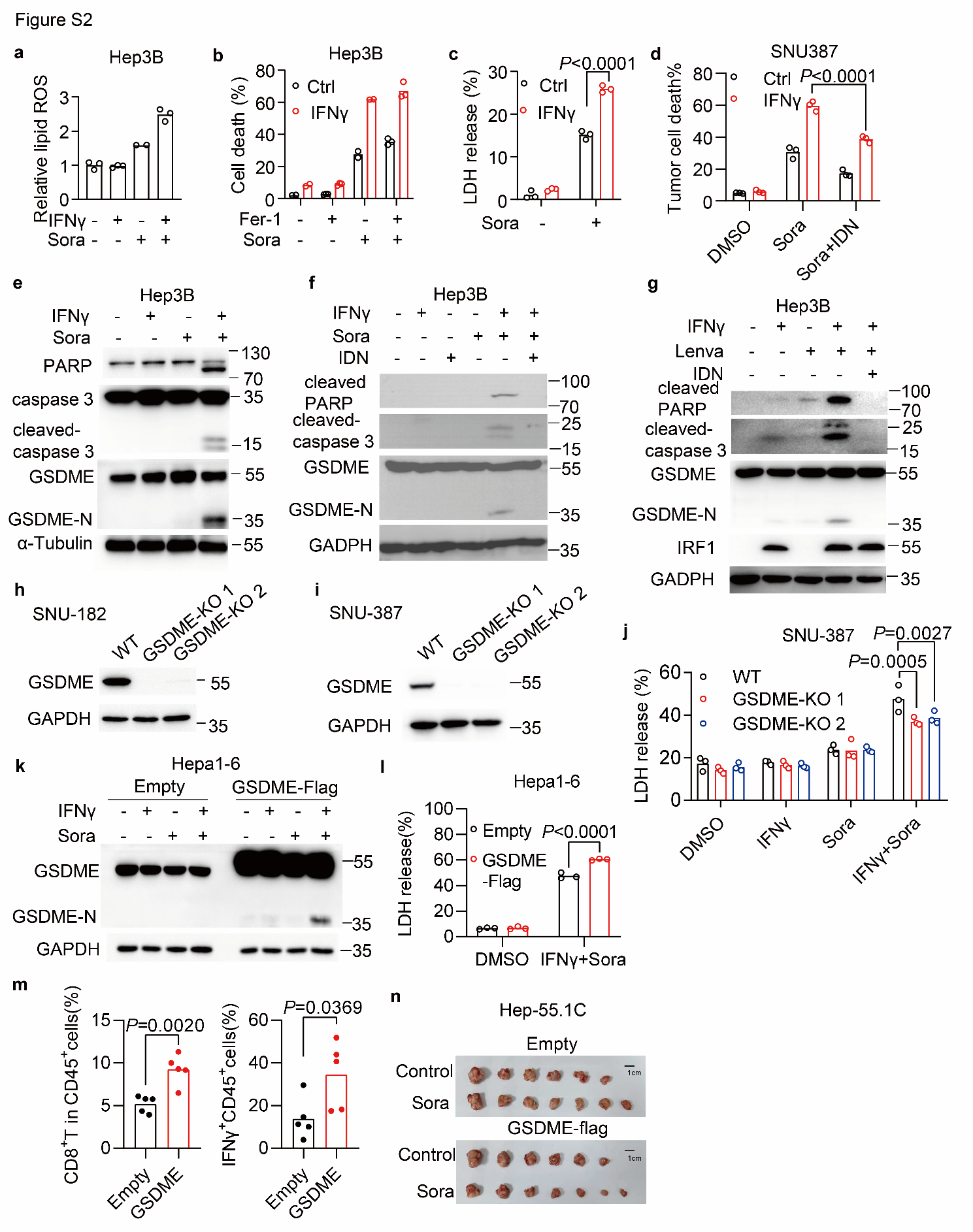


**Figure S2. IFNγ promotes TKI-induced GSDME-mediated pyroptosis**

**a**. Lipid ROS of Hep3B cells primed with IFNγ (10 ng/mL) for 24 h, followed by sorafenib (8 μM).

**b**. Cell death of Hep3B cells treated by the combination of IFNγ and sorafenib in the presence of ferroptosis inhibitor ferrostatin 1 (Fer-1, 10 μM) for 24 h.

**c**. The content of LDH released from Hep3B cells primed with IFNγ (10 ng/mL) for 24 h, followed by sorafenib (8 μM) for 24 h.

**d**. Cell death of SNU-387 cells treated by the combination of IFNγ and sorafenib in the presence of pan-caspase inhibitor IDN-6556 (IDN, 40 μM) for 48 h.

**e- g**. Immunoblots of PARP, caspase 3, GSDME and their cleavages in Hep3B cells treated by IFNγ combined with sorafenib (**e, f**) or lenvatinib (**f**) or in the presence of IDN (**f, g**) for 36 h (**e, f**) or 48 h (**e**). GAPDH served as loading controls.

**h, i**. Immunoblots of GSDME in SNU-182 (**h**) or SNU-387(**i**) cells expressing scramble sgRNA (WT) or two independent sgRNAs targeting GSDME (KO).

**j**. Percentage of LDH release in WT or GSDME deficient (GSDME-KO1/KO2) SNU-387 cells treated with IFNγ and sorafenib (12 μM) for 36 h.

**k**. Immunoblots of GSDME in Empty or GSDME-Flag expressing Hepa1-6 cells primed with mIFNγ (10 ng/ml) followed by sorafenib (15 μM) for 36 h. GAPDH served as a loading control.

**l**. LDH released from Empty or GSDME-Flag expressing Hepa1-6 cells treated by mIFNγ (10 ng/ml) plus sorafenib (15 μM) for 24 h.

**m**. The percentages of CD8^+^ T cells and IFNγ infiltrating in Hep-55.1C tumors expressing Empty or GSDME-Flag.

**n**. Tumor picture of Empty or GSDME-Flag expressing Hep-55.1C cells inoculated in C57BL/6 mice that were treated with vehicle control or sorafenib (10 mg/kg). n = 6-7 mice/group.

Data were shown as mean. p values were calculated by Student’s t-test(m), and two-way ANOVA (c, d, j, l).

**
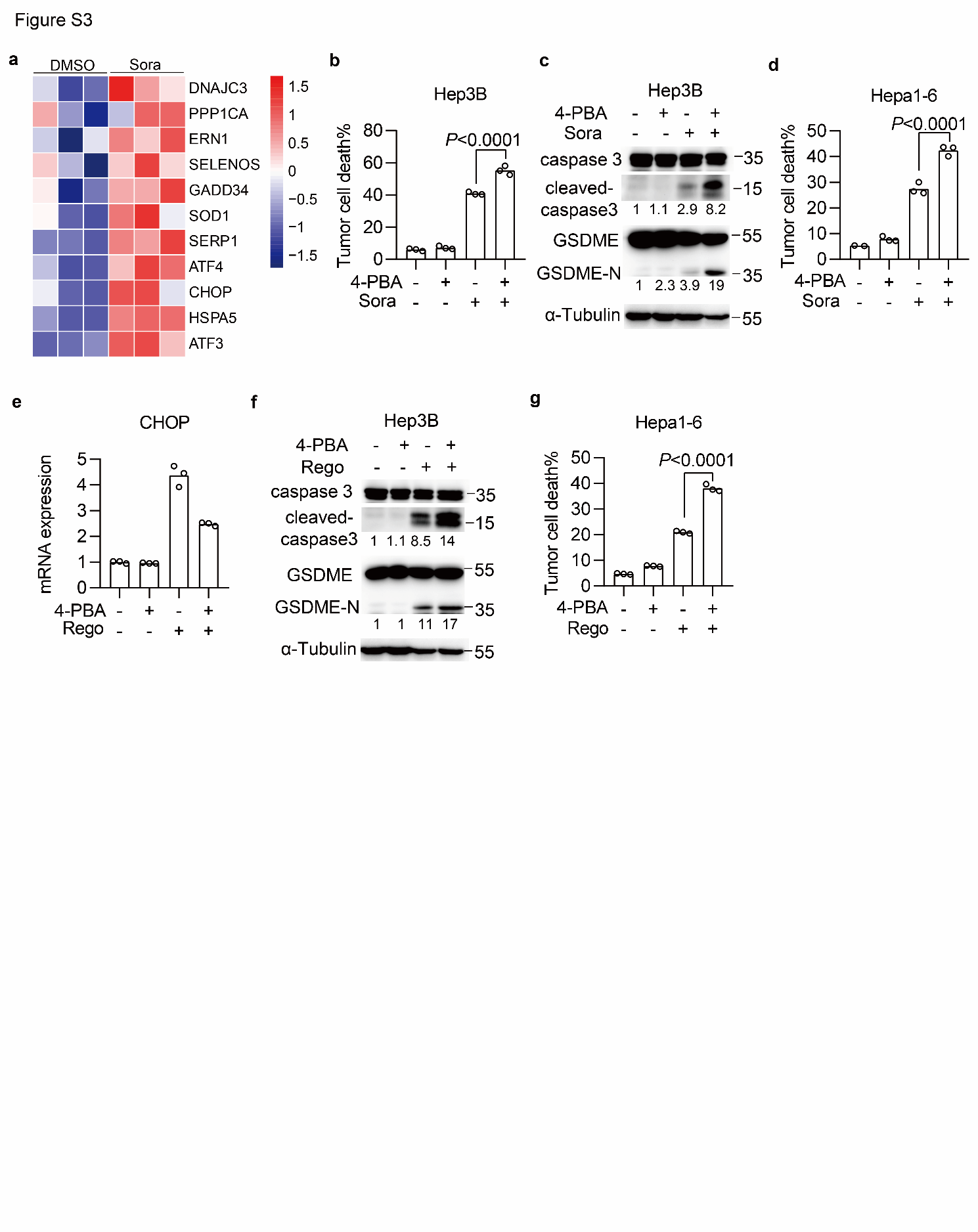
**

**Figure S3. UPR protects HCC from TKI-induced cell death**

**a**. Heatmap of differently expressed UPR-related genes in Hep3B cells upon sorafenib treatment.

**b**. Cell death of Hep3B (**b**) or Hepa1-6 (**c**) cells treated with sorafenib in the presence of 4-PBA (1 mM) for 65 h.

**c**. Immunoblots of caspase 3 and GSDME in Hep3B cells treated with sorafenib (8 μM) in the presence of 4-PBA (1 mM) for 36 h. α-Tubulin served as a loading control.

**d**. Cell death of Hepa1-6 cells treated with sorafenib in the presence of 4-PBA (1 mM) for 65 h.

**e**. Relative CHOP mRNA level in SNU-182 cells treated with sorafenib plus 4-PBA for 12 h.

**f**. Immunoblots of caspase 3 and GSDME in Hep3B cells treated with regorafenib (15 μM) in the presence of 4-PBA (1 mM) for 36 h. α-Tubulin served as a loading control.

**g**. Cell death of Hepa1-6 cells treated with regorafenib (15 μM) in the presence of 4-PBA for 65 h.

Data were shown as mean. p values were calculated by one-way ANOVA (b, d, g).

**
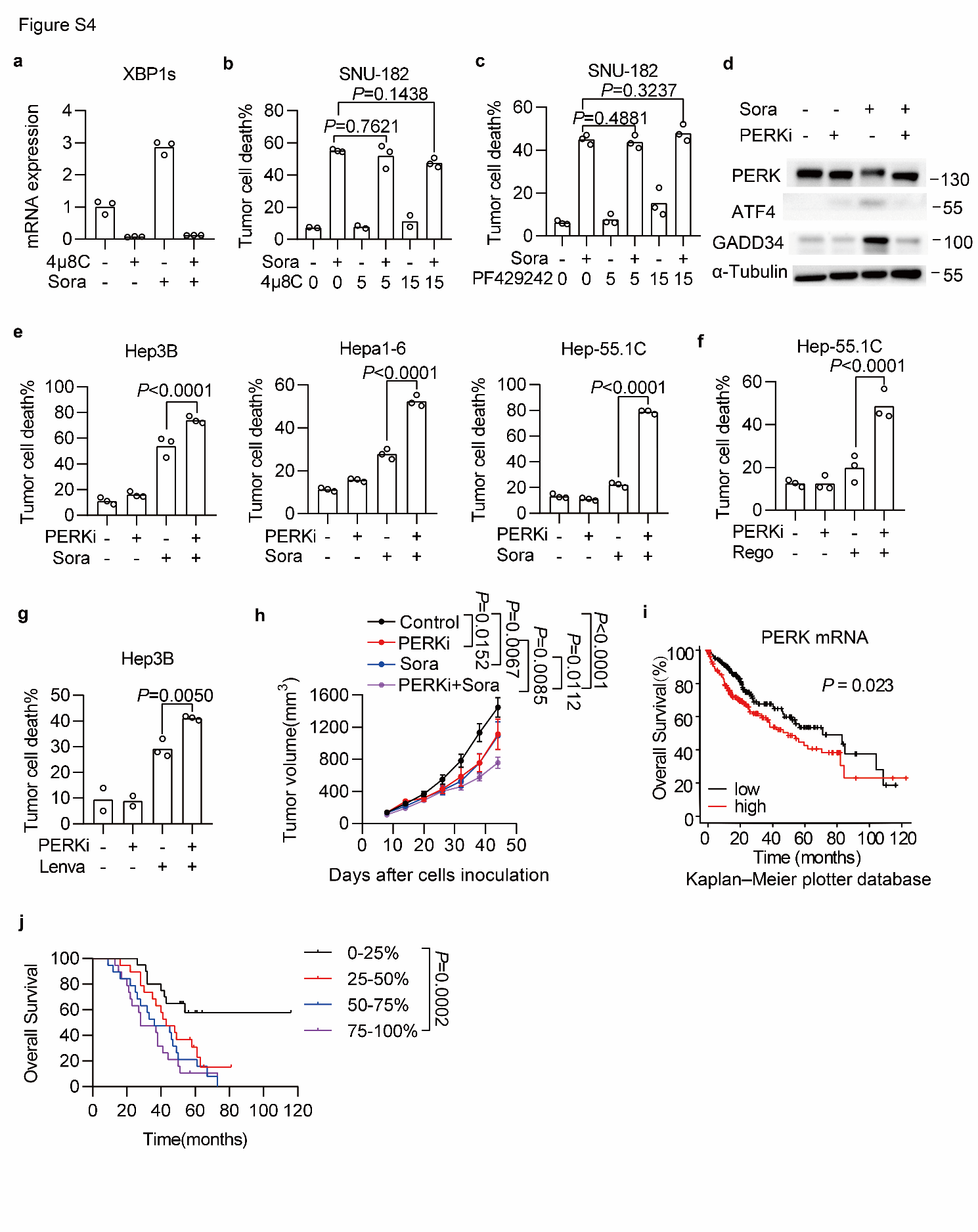
**

**Figure S4. PERK inhibition, but not IRE1α or ATF6 inhibition, promotes TKI-induced cell death.**

**a**. Relative XBP1s mRNA level in SNU-182 cells treated with sorafenib plus IRE1α inhibitor 4μ8C (5 μM) for 12 h.

**b, c**. Cell death of SNU-182 cells treated with sorafenib in the presence of IRE1α inhibitor 4μ8C (**i**, 5 or 15 μM) or ATF6 inhibitor PF429242 (**j**, 5 or 15 μM) for 72 h.

**d**. Immunoblots of PERK, ATF4 and GADD34 in SNU-182 cells treated by sorafenib plus PERK inhibitor.

**e-g**. Cell death of indicated cell lines following sorafenib (**e**), regorafenib (**f**) or lenvatinib (**g**) treatment in the presence of PERK inhibitor for 48 h (**e**) or 65 h (**f**, **g**).

**h**. C57BL/6 mice implanted with Hep-55.1C tumors were treated with GSK2606414 (50 mg/kg), sorafenib (20 mg/kg) or their combination. Tumor growth in four groups was monitored over time.

**i**. Kaplan-Meier survival curves for patients with liver cancer with high (n =197) or low (n = 173) PERK mRNA expression (Kaplan–Meier plotter).

**j**. Kaplan-Meier survival curves for patients with HCC categorized by the proportion of PERK-positive cells (0-25%(n=20); 25-50%(n=19); 50-75% (n=19); 75-100%(n=19)).

Data were shown as mean or mean ± SEM. p values were calculated by one-way ANOVA (e-g), two-way ANOVA (b, c, h), and log-rank test (i, j).

**
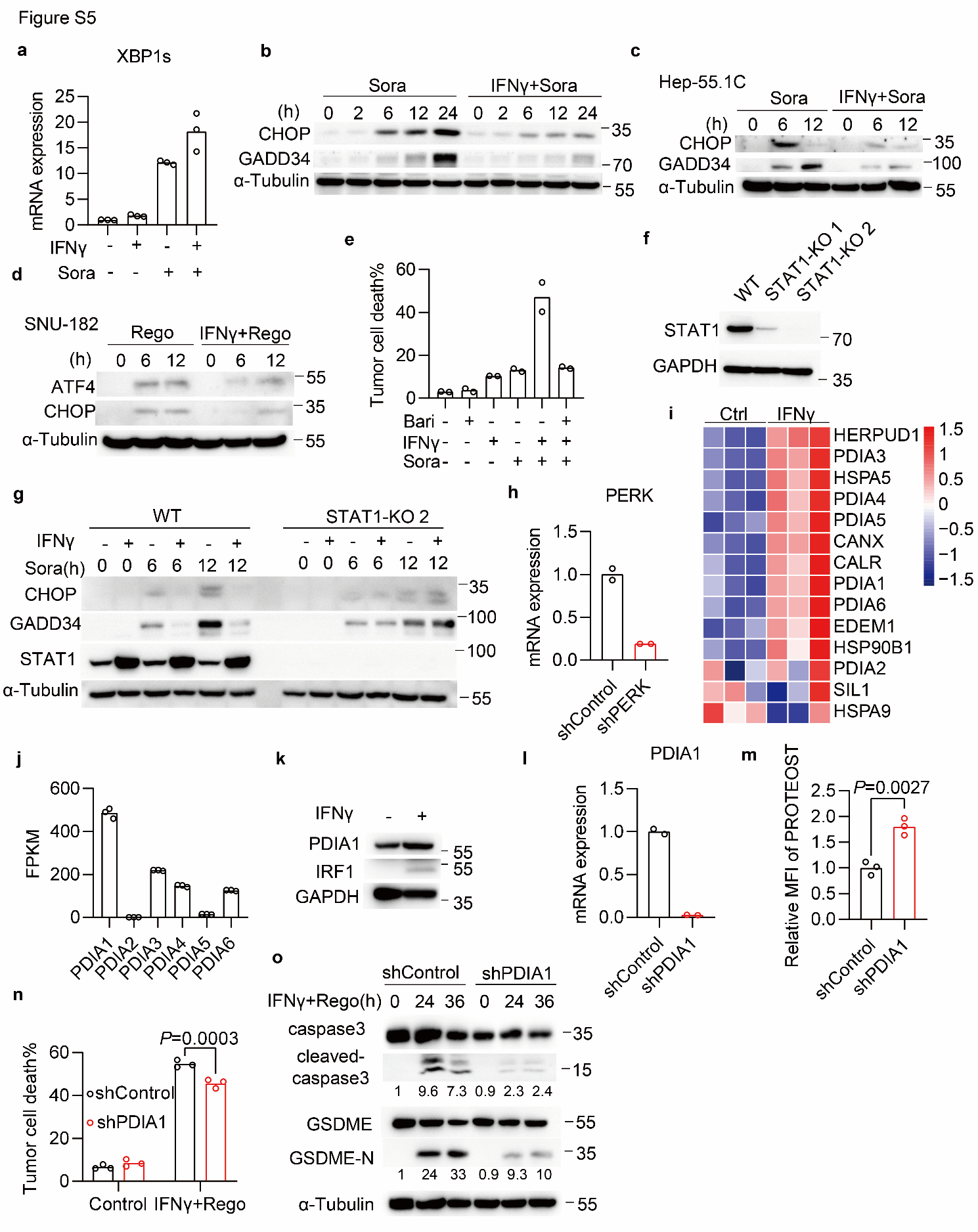
**

**Figure S5. IFNγ prime attenuates TKI-activated UPR by inducing PDIA1 expression**

**a**. Relative XBP1s mRNA expression in Hep3B cells treated by IFNγ and sorafenib.

**b- d**. Immunoblots of CHOP, GADD34 or ATF4 in Hep3B (**b**), Hep-55.1C (**c**) or SNU-182 (**d**) cells treated with IFNγ and followed with sorafenib (**b, c**) or regorafenib (**d**) for indicated time points. α-Tubulin served as loading controls.

**e**. Cell death of Hep3B cells treated with IFNγ and sorafenib in the presence of Jak inhibitor Baricitinib (0.5 μM) for 48 h.

**f, g**. Immunoblots of STAT1 (**f**) or CHOP and GADD34 (**g**) in Hep3B cells expressing scramble (WT) or STAT1-targeting sgRNA (STAT1-KO1/2). These cells were treated with IFNγ and sorafenib for indicated time points (**g**).

**h**. Relative PERK mRNA level in Hep-55.1C cells expressing control or PERK-targeting shRNA.

**i, j**. Heatmap of differently expressed molecular chaperones genes (**i**) or FPKM values of PDI family genes (**j**) in Hep3B cells upon IFNγ treatment.

**k**. Immunoblots of PDIA1 and IRF1 in IFNγ-primed Hep3B cells.

**l, m**. Relative PDIA1 mRNA level (l) or PROTEOST staining intensity in Hep3B cells expressing control or PDIA1-targeting shRNA. p value was calculated by Student's t-test.

**n, o**. Cell death of Hep3B cells expressing scramble or PDIA1 shRNA treated with IFNγ plus regorafenib (20 μM) (**n**). The cleavages of caspase3 and GSDME in these cells were determined by immunoblotting (**o**). α-Tubulin served as a loading control.

Data were shown as mean. p value was calculated by two-way ANOVA(n).

**
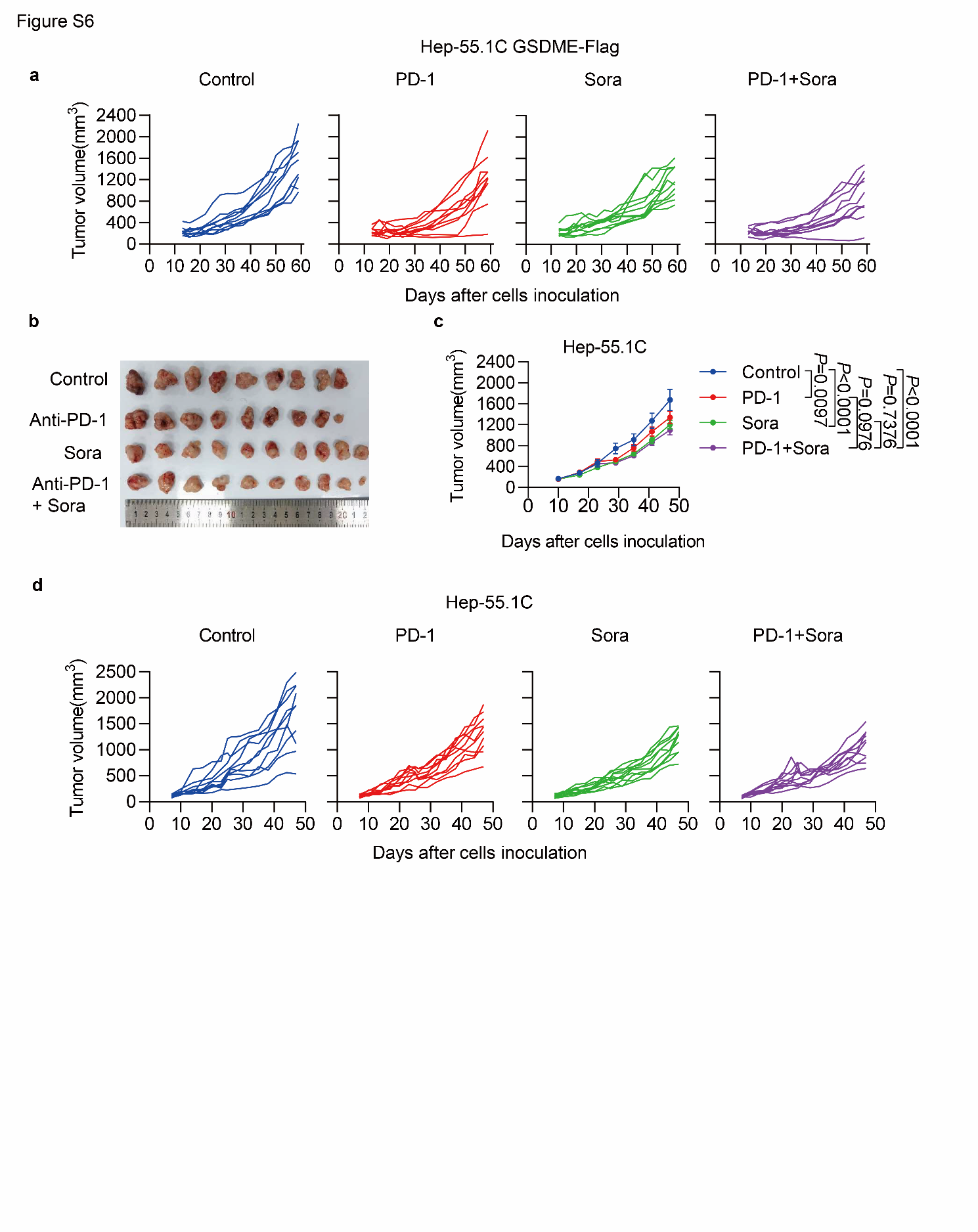
**

**Figure S6. GSDME-expressing tumors respond to the combination of PD-1 blockade and TKI**

**a**. Individual growth of Hep-55.1C tumors in treated with anti-PD-1 (100 μg), sorafenib (20 mg/kg) or their combination.

**b**. C57BL/6 mice implanted with GSDME-Flag expressing Hep-55.1C tumors treated with vehicle control, anti-PD-1 (100 μg), sorafenib (20 mg/kg), or their combination. Subcutaneous tumors were surgically removed and presented at the endpoint.

**c, d**. C57BL/6 mice implanted with Hep-55.1C tumors were treated with anti-PD-1 (100 μg), sorafenib (20 mg/kg), or their combination. Tumor growth was monitored over time (**c**) and individual tumor growth in each group was plotted (**d**). n = 10 mice per group.

Data were shown as mean or mean ± SEM. p values were calculated by two-way ANOVA (c).
