# Supplementary material for: IFNγ augments TKI efficacy by alleviating protein unfolding stress to promote GSDME-mediated pyroptosis in hepatocellular carcinoma
Source: Cell Death Dis. 2025 Jul 11;16(1):512. doi: 10.1038/s41419-025-07839-y (PMC12254303; doi:10.1038/s41419-025-07839-y)
Supplement: Supplementary file 2 — Supplementary Table 1 [file 41419_2025_7839_MOESM2_ESM.pdf]

Gene ID	Gene name	Ctrl-1 FPKM	Ctrl-2 FPKM	Ctrl-3 FPKM	IFNγ-1 FPKM	IFNγ-2 FPKM	IFNγ-3 FPKM	Sora-1 FPKM	Sora-2 FPKM	Sora-3 FPKM	Ctrl vs IFNγ DESeq2 FDR	Ctrl vs IFNγ DESeq2 log2FC	Ctrl vs Sora DESeq2 FDR	Ctrl vs Sora DESeq2 log2FC
ENSG00000130822	PNCK	4.721704	1.939131	0.606431	0.014606	0.058703	0.114421	0	0.153983	0	0.0000167	-4.812154062	0.000485992	-6.30338075
NewGene 2735	--	2.423292	2.349352	2.776307	1.099907	1.392319	0.912754	0.068686	0.102082	0.162251	0.00000023	-1.002391498	1.01E-47	-4.362383388
NewGene 1924	--	0.439456	0.504174	0.583322	0.62921	0.365104	0.286932	0	0.045287	0.035053	0.779557134	-0.112078171	0.000274947	-4.091992753
NewGene 5116	--	0.719652	0.760956	0.481645	1.555413	1.281201	1.099819	0.056618	0	0.046026	0.00000377	1.124369042	2.84E-13	-4.018982779
ENSG00000119737	GPR75	1.51857	1.435551	1.740072	1.532154	0.90961	1.009383	0.081054	0.117409	0.118852	0.328323726	-0.303195943	4.41E-22	-3.730499787
ENSG00000277726	AL109811.3	2.086636	2.7262	3.273176	2.824728	3.410978	3.152469	0.426015	0.097131	0.353722	0.695054822	-0.210516997	3.59E-11	-3.389508316
NewGene 3994	--	0.366441	0.358944	0.578532	0.383056	0.345002	0.25215	0.042442	0	0.075626	0.454906971	-0.259294292	0.016287874	-3.353687572
NewGene 6493	--	9.49145	8.027608	8.389718	7.508474	8.416015	7.992118	0.748805	0.899157	0.851066	0.43355889	0.101292964	1.38E-103	-3.295909009
ENSG00000184619	KRBA2	1.03856	1.072022	0.662257	0.38286	0.664996	0.549017	0.115974	0	0.125963	0.098662255	-0.587520835	0.014996269	-3.199633019
NewGene 3686	--	1.855962	2.087589	1.732471	2.305888	2.357102	2.037346	0.180055	0.213077	0.211544	0.0000408	0.436533847	2.54E-94	-3.097269879
ENSG00000147437	GNRH1	0.779187	0.830563	0.713977	0.757784	0.496809	0.506812	0.074399	0.115014	0.046781	0.584956656	-0.259771648	1.56E-08	-3.019660733
NewGene 5007	--	0.388276	0.619174	0.616961	0.682253	0.532966	0.473945	0.066315	0.048191	0.073365	0.558115667	0.196366125	3.61E-16	-3.010386289
ENSG00000163739	CXCL1	11.265047	11.909865	14.641148	3.375206	4.187348	3.610745	1.529347	1.408058	1.520486	6.51E-26	-1.607190673	1.92E-63	-2.97967528
NewGene 3240	--	0.535681	0.493451	0.834545	0.425166	0.519843	0.411128	0.089401	0.036584	0.094067	0.509413161	-0.2981716	8.78E-09	-2.924156981
NewGene 3231	--	0.493133	0.711381	0.536439	0.725533	0.739466	0.694621	0.071696	0.042799	0.097827	0.027075727	0.459683229	8.32E-21	-2.919796096
ENSG00000265972	TXNIP	6.982853	5.269945	1.662622	6.92829	4.944285	5.534293	0.605767	0.594648	0.582351	0.573827233	0.470703389	0.0000047	-2.858716515
NewGene 2050	--	0.571862	0.433942	0.672035	0.716398	0.642159	0.385957	0.177826	0.146521	0.044555	0.975648072	-0.014620573	7.51E-13	-2.846384341
ENSG00000123999	INHA	1.909742	1.1663	0.743846	0.166923	0.481604	0.357855	0.160856	0.25526	0.066187	0.000203669	-1.75157445	0.000000049	-2.814897083
NewGene 3941	--	0.482915	0.560715	0.444656	0.545129	0.627757	0.464697	0.040853	0.119042	0.038263	0.357171915	0.377313995	0.000000214	-2.804941987
NewGene 3597	--	0.540995	0.654464	0.71815	0.979464	1.083065	0.971673	0.109397	0.09597	0.057874	0.000184453	0.810590739	1.25E-13	-2.708326472
NewGene 3365	--	0.887438	0.965678	1.015838	0.807624	0.868831	0.534803	0.162492	0.181687	0.137454	0.05235985	-0.370216568	1.26E-32	-2.702687731
NewGene 3638	--	1.930501	1.97418	1.936214	2.107529	2.32949	1.833934	0.790858	0.468883	0.483184	0.008097275	0.454683756	2.87E-28	-2.696933682
NewGene 277	--	1.300662	1.560881	1.538721	1.308662	1.081865	1.04951	0.069454	0.306603	0.253514	0.632376587	-0.213636111	0.00000016	-2.61891095
NewGene 4961	--	1.256576	1.211179	1.612335	1.458431	1.361713	1.225964	0.186065	0.255723	0.210593	0.540642539	0.133989066	7.85E-28	-2.528499777
NewGene 3476	--	0.949984	1.122646	0.73945	0.757942	0.949914	0.81241	0.293628	0.235201	0.172591	0.454092152	-0.18584005	9.4E-23	-2.489876547
NewGene 3790	--	1.058019	0.813676	0.683159	1.185044	1.190658	0.929431	0.139936	0.150578	0.102526	0.153992058	0.412601461	1.73E-12	-2.476145691
NewGene 4633	--	0.746041	0.771317	0.965364	0.663388	0.623512	0.60016	0.198779	0.149328	0.058208	0.359909022	-0.231640553	2.17E-14	-2.463007706
ENSG00000107159	CA9	14.116001	7.051108	4.433023	1.676108	1.82964	1.421271	1.585353	1.321533	1.465672	0.0000483	-2.229868222	0.00000344	-2.453237097
NewGene 405	--	0.804023	0.628838	0.794301	0.563758	0.558662	0.824245	0.081839	0.113636	0.185119	0.931035113	-0.046583713	0.000000163	-2.441971189
NewGene 5405	--	0.996718	0.911026	1.15685	1.072105	1.138038	1.004543	0.212659	0.128059	0.165802	0.837221361	0.061880273	8.31E-18	-2.440376616
NewGene 3982	--	1.161117	1.415878	1.145036	1.149332	0.588843	0.309051	0.189432	0.128537	0.312167	0.085341767	-0.713843838	1.36E-10	-2.417111999
ENSG00000137331	IER3	83.920173	78.608367	86.707786	36.470808	37.830706	40.413759	11.987692	13.975308	19.404221	1.08E-51	-0.974119426	3.56E-33	-2.399094667
ENSG00000184602	SNN	8.754383	8.331722	7.227065	5.42943	5.366591	5.141057	1.440223	1.488606	1.448937	0.00000594	-0.462780047	2.4E-84	-2.376897838
NewGene 414	--	1.013529	1.035032	0.764724	1.031333	0.800532	1.016373	0.218433	0.146735	0.141331	0.614269551	0.166585739	1.39E-11	-2.356127565
ENSG00000179431	FJX1	3.038498	2.273234	3.274084	1.446877	1.374451	1.407903	0.36652	0.591151	0.60391	0.00000631	-0.87118322	5.76E-23	-2.352071986
ENSG00000009724	MASP2	2.199939	2.085012	1.617593	1.631697	1.505843	1.31687	0.210029	0.482689	0.418701	0.396599345	-0.22882662	3.66E-16	-2.340744714
ENSG00000105679	GAPDHS	0.758535	0.815491	0.845815	0.834213	0.772339	0.693408	0.132081	0.125829	0.18791	0.893005008	0.072040347	0.00000393	-2.332580194
ENSG00000213625	LEPROT	2.701229	2.282368	2.585231	2.115365	2.663449	1.706684	0.347647	0.425897	0.487105	0.843695418	-0.04543658	1.46E-33	-2.312383385
ENSG00000237440	ZNF737	3.42316	2.944843	2.376384	1.942996	2.182548	1.939344	0.581436	0.442978	0.723403	0.052934853	-0.369655229	1.77E-27	-2.304455858
NewGene 57	--	0.917888	0.845626	1.021998	1.355833	1.300447	1.335027	0.225158	0.162965	0.13275	0.0000354	0.665219574	2.87E-19	-2.296756496
ENSG00000145075	CCDC39	1.083491	1.235382	1.603064	1.711129	1.421898	1.230395	0	0.300337	0.449086	0.175119538	0.301521353	0.215094762	-2.296311872
ENSG00000139679	LPAR6	2.995183	3.238876	3.47057	2.024681	2.162243	1.885458	0.597678	0.585832	0.705242	0.000468629	-0.528929359	4.76E-37	-2.266789209
ENSG00000143971	ETAA1	9.39691	9.61993	10.796084	8.371572	7.698855	7.708956	1.828396	1.642187	2.295388	0.105840037	-0.160330797	5.1E-85	-2.225230836
NewGene 4344	--	0.759752	0.981557	1.073318	0.931448	0.892956	0.777425	0.228279	0.160455	0.167154	0.955615997	0.019727865	1.32E-14	-2.223353657
ENSG00000213213	CCDC183	5.493533	2.658345	1.16867	0.796359	0.554923	0.481471	0.403732	0.654675	0.733331	0.003530168	-2.137922436	0.002019431	-2.219830928
NewGene 5112	--	1.019396	1.187943	1.035925	0.448422	0.78437	0.734795	0.173515	0.189575	0.309389	0.151753816	-0.554250847	0.000000123	-2.206553499
NewGene 1427	--	1.00565	1.497921	1.486562	1.585253	1.193016	1.093057	0.156557	0.246658	0.323536	0.766856509	-0.126121581	2.66E-08	-2.186037778
NewGene 2710	--	0.480961	0.531998	0.620702	0.461122	0.583678	0.561912	0.205178	0.047772	0.072765	0.602728688	0.21439153	0.00000223	-2.185925243
ENSG00000070601	FRMPD1	0.226322	0.248388	0.272107	0.181669	0.157412	0.117773	0.046371	0.087451	0.018577	0.191614218	-0.569798449	0.0000266	-2.176804096
ENSG00000180626	ZNF594	2.417873	2.220432	2.136509	1.918789	1.908608	2.005692	0.664491	0.570554	0.488723	0.669612923	-0.07825332	1.84E-35	-2.16746209
NewGene 4964	--	1.237641	0.953202	0.851591	0.891989	0.85859	0.7531	0.230117	0.300462	0.19393	0.713411608	-0.142305886	2.81E-09	-2.1638011
NewGene 4557	--	0.616061	0.519925	0.741816	0.582218	0.557139	0.420226	0.150802	0.112583	0.108483	0.784678666	-0.126782195	0.000000413	-2.146821933
NewGene 2120	--	2.606431	2.977173	3.463017	2.010391	2.094009	1.898018	0.745689	0.502006	0.707617	0.000916361	-0.442604998	1.04E-39	-2.118855225
ENSG00000168806	LCMT2	3.582343	3.326133	3.634928	2.718308	2.710711	2.960694	0.730785	0.745586	0.77237	0.150191869	-0.15753028	7.57E-76	-2.107878244
NewGene 165	--	0.635724	0.782192	0.638984	0.643982	0.471745	0.504848	0.300664	0.121209	0.134726	0.673236912	-0.19797673	0.00000639	-2.076870193
ENSG00000100307	CBX7	0.288663	0.43535	0.531907	0.52953	0.461744	0.408362	0.111534	0.065866	0.101816	0.497331135	0.273378237	0.00000203	-2.065271494
NewGene 1342	--	3.272517	3.642335	3.662838	2.694037	2.047496	3.230542	1.083344	0.914613	0.623757	0.472349876	-0.340820188	1.1E-17	-2.064605566
NewGene 3759	--	1.908399	2.893909	3.18831	2.967873	2.382724	2.360435	0.584099	0.765346	0.673491	0.705829045	0.094432214	5.12E-24	-2.059266087
ENSG00000274349	ZNF658	0.408203	0.512683	0.325077	0.44416	0.501207	0.435475	0.064032	0.118635	0.084619	0.497879701	0.259931923	0.00000178	-2.051293644
ENSG00000152433	ZNF547	0.885507	1.252843	1.075658	0.845092	0.729277	0.835593	0.252117	0.234366	0.334881	0.372538841	-0.301079688	3.7E-09	-2.029939523
NewGene 1236	--	3.268703	4.706445	4.509696	3.741125	3.452204	3.978978	1.098954	1.235701	1.329933	0.344414454	-0.133963977	3.82E-61	-2.025810944
ENSG00000168970	IMJD7-PLA2G4I	0	3.341052	3.856447	3.564073	0	0	0	1.653353	0	0.891751387	-0.854704982	0.683429723	-1.988941681
ENSG00000132825	PPP1R3D	0.840657	1.106801	1.029334	0.929601	0.981867	0.895609	0.200755	0.320799	0.170978	0.852530742	0.060345072	8.38E-11	-1.988132782
NewGene 4626	--	0.692717	0.641511	0.798372	0.831139	0.371251	0.621532	0.156948	0.093637	0.195752	0.834585282	-0.104932566	0.00000325	-1.980537263
ENSG00000198182	ZNF607	3.341867	3.408285	4.306548	2.920708	2.758433	2.91925	1.258658	1.148253	0.754015	0.17724329	-0.197027458	0.021837735	-1.952992112
NewGene 6578	--	0.615588	0.625255	0.802455	0.531457	0.418036	0.442399	0.116128	0.135623	0.065667	0.984962581	-0.01041385	0.0000149	-1.946224983
NewGene 6546	--	0.621937	0.517469	0.519136	0.680776	0.476744	0.538912	0.086611	0.150985	0.152657	0.676662538	0.193903951	0.0000356	-1.924358227
NewGene 4336	--	1.430836	2.127169	1.941516	1.919755	1.576209	1.831831	0.457583	0.565268	0.371508	0.70994729	0.084647028	1.35E-19	-1.919929627
NewGene 1748	--	1.424481	1.806981	2.046005	1.495142	1.201979	1.664701	0.207428	0.574613	0.318953	0.498631422	-0.26323989	0.000000042	-1.917973907
NewGene 5625	--	0.460329	0.429598	0.445726	0.331635	0.435889	0.22283	0.164821	0.109426	0.044654	0.60897833	-0.27324846	0.000236091	-1.913155331
NewGene 622	--	0.577345	0.585416	0.551572	0.558439	0.573214	0.484565	0.154392	0.075844	0.197641	0.870039748	0.071477	0.0000036	-1.899822492
NewGene 276	--	0.960663	1.327822	0.975831	1.318688	0.877667	0.99008	0.210036	0.306891	0.288702	0.768185222	0.112013452	2.11E-08	-1.893452457
ENSG00000173825	TIGD3	0.888472	0.813501	0.825085	1.209211	1.116651	1.34859	0.123174	0.278948	0.233393	0.017237377	0.679394018	0.00000678	-1.880061924
NewGene 5659	--	0.640167	0.931028	0.668014	0.777597	0.67837	0.553711	0.125498	0.327064	0.220918	0.98538095	-0.008969222	0.00000114	-1.865286021

Gene ID	Gene name	Ctrl-1 FPKM	Ctrl-2 FPKM	Ctrl-3 FPKM	IFNγ-1 FPKM	IFNγ-2 FPKM	IFNγ-3 FPKM	Sora-1 FPKM	Sora-2 FPKM	Sora-3 FPKM	Ctrl vs IFNγ DESeq2 FDR	Ctrl vs IFNγ DESeq2 log2FC	Ctrl vs Sora DESeq2 FDR	Ctrl vs Sora DESeq2 log2FC
ENSG00000174804	FZD4	1.62633	1.760889	1.760125	1.617728	1.649333	1.677243	0.45482	0.427103	0.440836	0.554889302	0.090519503	1.19E-36	-1.860713768
ENSG00000163293	NIPAL1	2.33503	2.587234	2.535843	2.02103	1.90229	1.919798	0.735366	0.555082	0.636401	0.167605361	-0.19228535	2.78E-34	-1.860087108
ENSG00000129595	EPB41L4A	0.536235	0.692847	0.659227	0.534118	0.302389	0.495233	0.174326	0.185766	0.122888	0.286287794	-0.34969868	3.79E-09	-1.836612924
ENSG00000128342	LIF	1.234931	1.193297	1.137401	1.234304	1.110282	1.331119	0.223556	0.288274	0.424733	0.383538359	0.19404009	7.4E-13	-1.831855325
ENSG00000167384	ZNF180	5.382776	4.807689	4.91	4.671707	5.208336	4.345239	1.099268	1.469069	1.25475	0.899440418	0.020461732	1.97E-47	-1.824837915
NewGene_1154	--	0.926525	0.923281	0.766107	0.786928	0.657572	0.612732	0.204587	0.255649	0.186113	0.502316752	-0.133908428	8.2E-22	-1.823334617
NewGene_4577	--	0.444391	0.82103	0.78217	0.728401	0.497216	0.526422	0.174898	0.167387	0.186799	0.909767406	0.049773397	0.000000141	-1.817682858
ENSG00000118515	SGK1	13.776714	14.584685	14.908437	15.415243	11.88204	14.785903	3.768058	3.500441	4.564547	0.441350658	0.095159141	2.92E-61	-1.801217673
NewGene_5308	--	2.261632	2.725403	3.080195	3.050407	2.835522	2.621579	0.778992	0.753488	0.663583	0.329004573	0.146191082	1.88E-33	-1.793959423
ENSG00000261150	EPPK1	1.223502	1.266982	0.85621	1.008952	0.948238	1.182561	0.319392	0.326532	0.266223	0.712328337	0.07374279	1.01E-27	-1.771243367
NewGene_6247	--	0.593008	0.581054	0.330096	0.445209	0.382473	0.242088	0.072046	0.16439	0.094536	0.761768675	-0.159523221	0.000118566	-1.770851708
ENSG00000253276	CCDC71L	1.001319	0.970737	0.753878	1.019703	1.069779	0.953599	0.276429	0.175236	0.294178	0.198458767	0.303710412	2.28E-10	-1.770807313
NewGene_4066	--	1.296364	1.076348	1.457658	1.184692	0.924421	1.016283	0.428419	0.25038	0.361218	0.701254745	-0.157251225	0.00000274	-1.770629644
ENSG00000196110	ZNF699	0.791864	0.716023	0.515202	0.468067	0.692024	0.433209	0.198402	0.198089	0.148984	0.629944774	-0.198800077	0.000000698	-1.767692035
ENSG00000139318	DUSP6	23.90382	21.474236	21.252985	22.737489	22.796636	22.149505	4.266027	5.657029	8.753653	0.001653501	0.215510984	3.6E-12	-1.767443898
ENSG00000168906	MAT2A	138.226517	151.051136	150.30698	130.503077	123.09618	121.744993	43.111907	37.278611	43.541696	0.166251012	-0.084971732	2.68E-172	-1.760659916
NewGene_1305	--	1.179607	0.921951	1.174878	1.353417	1.624365	1.053542	0.339804	0.392765	0.231546	0.659939263	0.099237041	2.04E-16	-1.746620535
NewGene_1125	--	1.219475	1.257269	1.194519	1.137102	0.09792	0.976473	0.145825	0.536981	0.35243	0.620041913	-0.60670766	1.37E-08	-1.740925264
NewGene_4320	--	0.636868	0.560385	0.610933	0.210316	0.270923	0.161074	0.143551	0.135444	0.106641	0.001566923	-1.120753149	0.00000261	-1.736865773
NewGene_2946	--	0.38941	0.34256	0.431607	0.541137	0.577637	0.520394	0.063048	0.063698	0.193337	0.039580946	0.606820143	0.000104072	-1.735658371
NewGene_1235	--	1.495384	1.137248	1.656493	1.687067	1.613228	1.525195	0.357491	0.402124	0.450042	0.019352012	0.315333655	3.6E-31	-1.733438025
ENSG00000164938	TP53INP1	38.92169	38.58726	38.495755	69.174755	68.07334	66.157589	10.76484	9.916574	12.31936	5.85E-104	0.957363073	1.14E-150	-1.723307807
ENSG00000205730	ITPRIPL2	1.197914	1.160662	0.913216	1.273491	1.014552	1.10533	0.313892	0.323687	0.292069	0.305471136	0.195854692	5.94E-19	-1.719422023
NewGene_5947	--	0.563336	0.332153	0.601467	0.681822	0.694296	0.700514	0.123324	0.147094	0.152009	0.09924639	0.583731838	0.000132453	-1.706997318
ENSG00000271741	AC114490.2	1.343241	0	0.872745	0.285204	0.417324	0.438661	0.236072	0.060288	0.348121	0.761503002	-0.808984228	0.451882609	-1.696811694
ENSG00000213588	ZBTB9	7.346526	8.186914	8.384935	5.614621	5.691505	5.478462	3.130262	1.722264	2.079454	0.000549551	-0.363906735	5.89E-25	-1.696106615
NewGene_6067	--	1.640038	2.464049	2.708949	2.241549	2.136788	2.295922	0.671571	0.648523	0.615042	0.70462257	0.116213132	6.26E-10	-1.694182248
NewGene_1822	--	0.572465	0.645107	0.775247	0.733335	0.721917	0.852284	0.14244	0.208351	0.225836	0.400857161	0.215759133	1.76E-09	-1.683102665
ENSG00000179583	CIITA	0.144942	0.192505	0.188032	0.263536	0.26718	0.19781	0.039155	0.045695	0.066098	0.015082564	0.588263979	0.00000012	-1.681064904
NewGene_5972	--	0.866942	1.146121	1.495934	1.366859	0.977885	0.791766	0.384095	0.3636	0.271989	0.999370817	-0.000340605	2.37E-08	-1.678778374
ENSG00000198855	FICD	1.250958	1.707617	1.602677	1.866099	1.678532	1.727515	0.737307	0.697135	0.797525	0.070539775	0.368677396	3.34E-10	-1.678355891
NewGene_2469	--	1.928144	2.478397	2.575071	1.929126	1.866242	2.174333	0.791918	0.684734	0.855656	0.866165712	-0.041764411	1.06E-17	-1.669301196
NewGene_3669	--	1.052038	1.124712	0.96802	0.791905	0.888702	0.942555	0.29238	0.282647	0.350886	0.750772846	-0.10919371	2.77E-08	-1.662432745
ENSG00000115137	DNAJC27	0.286608	0.320823	0.541262	0.303737	0.294928	0.338893	0.161616	0.144282	0.080593	0.742176808	-0.154380049	0.000166677	-1.653323032
NewGene_4484	--	1.571224	1.466359	2.327459	2.518742	2.433853	2.243393	0.641245	0.279621	0.669274	0.041058354	0.573448156	0.0000181	-1.648106994
NewGene_1229	--	4.946588	6.344272	5.534441	5.594993	4.420505	4.051754	3.413534	3.146665	3.265671	0.427440942	-0.099950443	1.73E-68	-1.640019087
ENSG00000254402	LRRC24	0.597502	0.359286	4.796281	0.252749	0.382167	0.417314	0.416979	0.535967	0.761081	--	--	0.201521468	-1.63561663
NewGene_4063	--	0.665882	0.571829	0.620415	0.265488	0.436433	0.411603	0.281837	0.183546	0.216577	0.223742144	-0.566353174	0.000350914	-1.632534429
ENSG00000178935	ZNF552	4.234356	3.874075	3.802665	3.934174	3.683423	3.946942	1.435774	1.135266	0.992443	0.843705594	0.040287869	1.25E-20	-1.63234031
ENSG00000177283	FZD8	2.024083	1.302174	1.406758	1.967871	1.905488	2.020465	0.469768	0.389995	0.589972	0.016797419	0.460427898	4.82E-12	-1.619016297
ENSG00000119227	PIGZ	6.866257	5.003716	4.316693	2.057624	2.018561	2.295829	1.499725	1.546872	2.056473	2.19E-12	-1.207024184	2.1E-19	-1.617868804
NewGene_4409	--	2.088374	2.697132	2.763135	0.917814	0.700117	1.099095	0.829061	0.545635	0.685084	3.15E-10	-1.342695079	2.7E-14	-1.613718911
ENSG00000124217	MOCS3	2.86446	3.151071	2.742062	2.85755	2.440608	2.513591	1.035847	0.708468	0.950183	0.92020154	-0.018739385	3.43E-27	-1.608965459
ENSG00000136158	SPRY2	12.124109	14.724529	15.138439	9.215846	8.091362	8.987061	3.765256	4.565776	4.849608	0.0000139	-0.450282516	3.94E-43	-1.608655559
ENSG00000178074	C2orf69	2.455413	2.582111	2.83491	1.944568	2.206297	1.989193	0.794769	0.726867	0.90031	0.206326306	-0.209868464	1.53E-22	-1.607462061
NewGene_6635	--	1.492512	1.804201	2.058683	1.735475	1.660182	1.967731	0.790355	0.414295	0.782224	0.044004716	0.334601886	1.87E-15	-1.604140991
NewGene_1214	--	5.972671	5.066587	4.923893	4.393766	3.264633	4.487715	1.564397	1.346207	2.047297	0.224001974	-0.246812643	2.86E-16	-1.595073841
ENSG00000274750	HIST1H3E	0.612036	0.680708	0.53971	0.456718	0.495783	0.466686	0.209137	0.205448	0.173082	0.683922094	-0.180862416	0.0000687	-1.593705751
ENSG00000183718	TRIM52	3.47543	3.856338	4.164814	4.07299	3.976036	3.385129	1.069694	1.15699	1.364944	0.368790982	0.138820571	1.37E-26	-1.585273815
ENSG00000164287	CDC20B	0.755284	0.682283	0.831704	0.372012	0.316343	0.29914	0.281583	0.122797	0.27125	0.004235575	-0.987302154	0.0000242	-1.581665562
ENSG00000242689	CNTF	1.811393	1.874083	1.933433	2.040341	1.44968	2.070252	0.516773	0.437188	0.801825	0.654890292	0.134018423	1.74E-08	-1.578195332
ENSG00000128011	LRFN1	0.395139	0.547601	0.443089	0.441515	0.387351	0.387694	0.142891	0.117267	0.168199	0.947435906	-0.03414182	0.000115327	-1.572690954
ENSG00000166343	MSS51	0.498593	0.428477	0.540665	0.370357	0.339287	0.39207	0.138889	0.149029	0.128469	0.788285263	-0.148063587	0.001175746	-1.570096563
ENSG00000137462	TLR2	0.438797	0.320052	0.437808	0.483274	0.700358	0.887338	0.100589	0.169024	0.036632	0.002656234	0.984209081	0.002114933	-1.566851727
ENSG00000186628	FSD2	0.6104	0.592855	0.50046	0.510929	0.557421	0.526169	0.159841	0.156816	0.220534	0.861538631	0.055140048	4.95E-09	-1.56635906
ENSG00000145860	RNF145	16.751781	16.510841	17.685882	10.800144	10.495851	10.579631	5.029504	5.348109	5.898424	1.41E-15	-0.534959411	5.6E-81	-1.554899905
NewGene_391	--	0.871738	1.009699	1.187862	0.450235	0.547638	0.914126	0.202078	0.20564	0.41538	0.290639268	-0.338754018	0.000000372	-1.554084618
ENSG00000145416	1-Mar	0.742006	0.972284	1.116613	1.224498	0.844072	0.957441	0.440801	0.277041	0.179313	0.33146186	0.242338713	0.000000117	-1.554001693
NewGene_5558	--	0.861654	0.78703	0.735259	1.313163	0.802443	0.991522	0.154139	0.203383	0.396331	0.108247328	0.52527198 5	0.000167708	-1.547272903
NewGene_4750	--	1.016212	0.897675	1.069747	0.774134	0.844125	0.875848	0.623398	0.349279	0.236513	0.773430767	-0.080458501	1.48E-10	-1.545520532
NewGene_5291	--	1.398844	1.277601	1.036154	1.187744	1.141418	0.991419	0.448262	0.290829	0.29648	0.52237022	0.245640116	0.0000908	-1.543631418
NewGene_262	--	0.581601	0.596869	0.523556	0.61379	0.748097	0.649032	0	0	0.568459	0.387352483	0.382855174	0.60020672	-1.540796064
ENSG00000163874	ZC3H12A	3.660863	4.132668	3.573107	2.936603	3.007239	3.200353	1.395858	1.029344	1.231178	0.370844714	-0.152537437	5.34E-20	-1.535953311
NewGene_2204	--	1.03969	0.904146	1.071871	1.243975	1.107726	1.127607	0.608106	0.561248	0.577949	0.684131363	0.093265126	5.74E-15	-1.528593839
ENSG00000182141	ZNF708	3.823971	4.176984	3.54241	3.127174	3.062388	2.975441	1.363203	1.216193	1.200544	0.110842441	-0.208202658	1.53E-28	-1.527635918
ENSG00000135441	BLOC1S1	45.107609	42.619625	34.922302	34.263106	37.531143	41.212843	14.198548	16.09849	9.16936	0.973662659	0.006604214	7.73E-21	-1.519622119
NewGene_4543	--	1.787499	2.628481	2.211974	2.058309	1.611143	1.414686	0.874911	0.900974	0.799867	0.176006014	-0.245009026	1.57E-19	-1.51545875
NewGene_310	--	0.70242	0.75159	0.715394	0.952681	1.040391	0.830659	0.233387	0.217763	0.246059	0.192531727	0.516142086	0.001518911	-1.510284361
NewGene_2149	--	2.234078	2.195322	2.028737	2.049615	1.846386	1.807141	0.829857	0.580632	0.717945	0.853917749	-0.034050971	2.12E-22	-1.50667746
NewGene_518	--	2.086972	2.552624	2.506853	3.415076	3.172479	2.996121	0.73655	0.732734	1.016507	0.000000119	0.568340666	1.65E-24	-1.499243192
NewGene_1466	--	0.768609	0.827768	0.880422	0.728287	0.615268	0.902427	0.224406	0.250586	0.335537	0.955679584	0.022646049	0.000000642	-1.496875731
NewGene_4949	--	1.254424	1.001132	0.75633	0.575373	0.77381	0.768043	0.306142	0.403069	0.304987	0.003992708	-0.894446195	0.00000115	-1.493799394
NewGene_4826	--	0.872821	0.632754	0.625912	0.679126	0.407163	0.584368	0.273692	0.182833	0.195453	0.528064488	-0.172124447	1.43E-08	-1.487428743
NewGene_3093	--	0.36177	0.595506	0.477759	0.057227	0.071088	0.07781	0.164953	0.133063	0.174707	1.38E-10	-2.607959143	0.0000277	-1.482653043

Gene ID	Gene name	Ctrl-1 FPKM	Ctrl-2 FPKM	Ctrl-3 FPKM	IFNγ-1 FPKM	IFNγ-2 FPKM	IFNγ-3 FPKM	Sora-1 FPKM	Sora-2 FPKM	Sora-3 FPKM	Ctrl vs IFNγ DESeq2 FDR	Ctrl vs IFNγ DESeq2 log2FC	Ctrl vs Sora DESeq2 FDR	Ctrl vs Sora DESeq2 log2FC
NewGene 3668	--	0.599046	0.787943	0.489773	0.512885	0.394509	0.495398	0.142617	0.291129	0.178964	0.580006492	-0.270082148	0.00138358	-1.478852248
ENSG00000141668	CBLN2	1.187968	0.672176	0.82371	0.249205	0.270884	0.43553	0.187227	0.250621	0.345558	0.002863763	-1.094178305	0.0000527	-1.470965389
ENSG00000197124	ZNF682	3.061537	3.68757	4.965759	4.891401	3.814102	3.533327	1.825684	1.321993	1.320935	0.884704738	0.036693541	4.35E-14	-1.469193886
NewGene 2789	--	1.254288	1.521934	1.276613	1.058008	0.900529	1.095933	0.361355	0.404612	0.606197	0.51253327	-0.245836913	0.000038	-1.464031902
ENSG00000165379	LRFN5	0.514932	0.575816	0.508486	0.112478	0.115201	0.143225	0.136772	0.241783	0.154478	0.000000222	-1.91227974	0.0000698	-1.463457866
NewGene 5072	--	1.362773	1.326695	1.576675	1.299371	1.152656	1.267942	0.416207	0.390359	0.589262	0.907863907	-0.033548787	5.6E-10	-1.459191117
ENSG00000169758	TMEM266	0.601305	0.68293	0.579037	0.661309	0.416945	0.527283	0.242749	0.225198	0.13954	0.935992945	-0.048647463	0.002475481	-1.448102335
ENSG00000134851	TMEM165	42.383499	44.612007	44.15511	37.080903	36.140315	37.05525	16.571201	15.15016	14.884571	0.095260052	-0.114937271	5.02E-75	-1.447060462
NewGene 2002	--	0.999879	0.659908	0.491418	0.175227	0.370245	0.221237	0.210496	0.237357	0.436816	0.0000966	-1.604031121	0.000173895	-1.44467729
NewGene 2009	--	0.455635	0.492553	0.411712	0.712415	0.496011	0.533792	0.159245	0.125419	0.183541	0.197472764	0.49921736	0.001463145	-1.44091385
NewGene 3153	--	1.502505	1.619392	1.542067	1.199523	1.003874	1.0535	0.635598	0.472769	0.722229	0.067148083	-0.32195078	7.28E-15	-1.43633994
ENSG00000135052	GOLM1	3.773344	3.741773	4.347446	3.438981	3.395968	2.902179	1.508877	1.398713	1.191857	0.403541368	-0.138425331	4.2E-20	-1.435112402
ENSG00000212127	TAS2R14	1.361849	1.769703	1.858855	1.77919	1.475298	1.611521	0.467028	0.655331	0.582087	0.727258781	0.113844907	0.000000973	-1.434817819
ENSG00000164379	FOXQ1	5.658913	6.294094	4.854599	4.367383	5.368431	4.783414	1.637833	2.163499	2.015061	0.7573537	-0.06164802	4.79E-19	-1.433623504
ENSG00000179476	C14orf28	1.500337	1.369843	1.399696	1.484385	1.326672	1.453742	0.589775	0.510353	0.384122	0.560474542	0.145878596	1.49E-08	-1.427351373
ENSG00000185112	FAM43A	1.338742	1.419558	1.684985	1.247082	1.252996	0.81316	0.530057	0.467105	0.536322	0.332972217	-0.284696574	2.91E-08	-1.426892079
NewGene 5557	--	0.586898	0.46179	0.47883	0.737179	0.958998	0.661567	0.191206	0.17392	0.168365	0.001302156	0.76454322	0.0000018	-1.422252635
ENSG00000135245	HILPDA	7.544001	8.814736	7.298442	5.044832	4.02663	4.463998	2.41337	2.724771	3.893313	0.001653501	-0.563702999	5.43E-15	-1.412368014
NewGene 3735	--	3.296561	3.145583	3.748194	3.143076	3.02495	3.072217	1.245294	1.067178	1.357137	0.99376425	-0.002070685	2.25E-16	-1.407313163
ENSG00000234444	ZNF736	4.956142	5.42423	5.325161	5.101681	4.062899	4.287057	1.565173	3.356383	2.58849	0.501970262	-0.079457493	0.077363427	-1.403015273
NewGene 1151	--	40.108482	40.429821	42.249928	43.792744	40.76342	43.854538	15.200292	12.931879	15.507134	0.0000832	0.211882646	1.4E-100	-1.401907541
ENSG00000204923	FBXO48	0.318966	0.414941	0.438518	0.264229	0.299668	0.383145	0.180547	0.09042	0.142974	0.719254197	-0.145643114	0.0000756	-1.401848999
ENSG00000112182	BACH2	0.397009	0.445848	0.590267	0.271976	0.266495	0.253539	0.203728	0.233589	0.132881	0.003386351	-0.707442039	0.00000012	-1.398764029
ENSG00000165494	PCF11	7.905513	8.035803	8.411519	7.941293	7.988898	7.765343	2.578103	2.604556	3.608134	0.298846624	0.086628593	2.28E-39	-1.398389398
ENSG00000163734	CXCL3	1.848326	1.970909	1.727077	0.815095	1.029127	1.259258	0.804747	0.381023	0.76428	0.052583834	-0.682604702	0.000142542	-1.394745388
ENSG00000167232	ZNF91	3.727502	4.090232	3.930436	4.079597	3.786472	3.924223	1.826749	1.761252	1.178682	0.617163424	0.071534636	8.28E-24	-1.393186188
ENSG00000120738	EGR1	7.624581	6.935523	8.185744	4.138445	4.041518	4.16724	1.811096	2.354518	4.031744	7.29E-13	-0.732867554	0.000126595	-1.388896682
ENSG00000069020	MAST4	1.788899	1.802062	1.924883	1.807394	1.81662	1.796112	1.011366	0.670201	0.756673	0.493077097	0.087116327	0.000108056	-1.387126044
NewGene 2996	--	0.610456	0.922435	0.723244	0.480501	0.740649	0.586887	0.301049	0.24809	0.243243	0.719189376	-0.161074816	0.000177019	-1.387037737
ENSG00000081041	CXCL2	0.962787	1.754586	1.487695	4.395576	3.598125	3.855699	0.673827	0.460952	0.358471	5.92E-12	1.633487404	0.000949872	-1.378906122
ENSG00000178386	ZNF223	3.794614	3.802026	4.057746	3.446883	3.703215	3.383107	1.786125	1.365964	1.337808	0.799717979	0.052113334	6.15E-16	-1.378124288
ENSG00000170871	KIAA0232	8.27594	7.7442	7.809666	8.362354	8.275247	7.862682	3.013662	2.720733	2.968339	0.013191307	0.177716644	1.53E-69	-1.377411723
ENSG00000196981	WDR5B	2.677325	2.415422	2.493412	2.208529	1.83436	2.030024	0.921965	0.784443	1.03434	0.31174797	-0.173267757	4.58E-17	-1.376584306
ENSG00000114315	HES1	23.242826	23.037912	23.378227	23.356787	21.744427	21.583578	8.218904	7.971761	8.980445	0.375826063	0.083434345	2.67E-54	-1.376203997
NewGene 1291	--	1.052828	1.656865	1.615937	1.293603	1.466145	0.945165	0.355784	0.47616	0.679728	0.930917384	0.025634423	1.38E-11	-1.372480346
NewGene 4970	--	2.239897	2.690923	2.300474	2.313164	2.539437	2.389618	0.966688	0.911814	0.952146	0.270096724	0.143006544	2.41E-25	-1.370051483
ENSG00000069667	RORA	1.828273	1.623786	2.012133	1.495668	1.67915	1.308418	1.206268	1.250806	1.199006	0.520377193	-0.109012643	8.32E-25	-1.368357427
ENSG00000196268	ZNF493	1.670381	1.881461	1.52044	1.667421	1.676748	1.272699	0.870859	0.810427	0.265343	0.736396081	-0.096177428	0.000000298	-1.367806093
ENSG00000138080	EMILIN1	0.975945	0.791317	0.509396	0.6745	0.841064	0.801177	0.3247	0.185443	0.316403	0.80924395	0.093200216	0.0000422	-1.365570496
NewGene 726	--	1.528844	1.89086	1.851901	1.579213	1.349081	1.998447	0.745819	0.650622	0.699584	0.940090745	0.020422313	1.03E-12	-1.363265265
ENSG00000234616	JRK	4.742369	5.05124	6.916685	4.432024	4.10189	4.476265	1.889229	1.560789	1.820122	0.983796386	-0.002586323	9.86E-52	-1.361793712
ENSG00000162783	IER5	2.705194	2.419096	2.688222	2.309702	2.425335	2.492947	0.944336	0.911518	0.989854	0.850029868	0.036602415	9.16E-20	-1.360632664
NewGene 3667	--	1.054408	0.726881	0.975769	0.693387	0.789531	0.645179	0.422809	0.239086	0.358255	0.540077403	-0.222307939	0.0000385	-1.355957428
ENSG00000257594	GALNT4	1.893768	2.317025	2.608695	1.955829	1.866601	1.915835	0.861812	0.641412	0.998751	0.590091577	-0.100216194	5.54E-15	-1.354389205
ENSG00000171016	PYGO1	0.261702	0.273613	0.253787	0.149854	0.217705	0.186477	0.095295	0.122564	0.060091	0.236526384	-0.401806041	0.000139876	-1.354273332
ENSG00000066230	SLC9A3	2.676463	2.089449	2.515101	2.350157	2.207995	2.452527	0.798971	0.9066	1.006653	0.616131418	0.088180114	3.12E-18	-1.350060883
NewGene 58	--	0.764507	0.882656	0.87695	0.943207	1.149454	0.965786	0.175688	0.265234	0.406143	0.066195807	0.498417944	0.000115828	-1.348733386
ENSG00000078401	EDN1	0.950737	1.10028	1.339638	0.271864	0.203757	0.454621	0.283381	0.476694	0.487876	0.00000575	-1.677776505	0.000098	-1.34193242
NewGene 1578	--	7.233522	7.664526	8.497129	9.242625	8.158632	8.093197	2.823707	2.687815	3.140533	0.00509915	0.270730033	1.03E-37	-1.34158801
ENSG00000168040	FADD	47.661625	43.028778	42.186409	39.423283	39.246075	40.259533	15.348469	17.679607	16.095829	0.903733296	-0.012930553	1.31E-53	-1.339912116
NewGene 5132	--	15.759623	16.845067	14.782366	17.01656	16.107346	14.816615	6.100994	6.001444	7.046162	0.018143526	0.196233513	1.34E-59	-1.337316604
NewGene 4538	--	4.79981	5.192709	5.028017	4.058742	3.060754	3.552343	1.78674	1.589425	2.20321	0.028786025	-0.309629569	1.51E-20	-1.329349245
NewGene 2706	--	1.624857	0.796488	0.766852	1.05049	0.805535	0.923754	0.449702	0.307993	0.435704	0.894794052	-0.056325666	0.0000265	-1.326299982
ENSG00000186496	ZNF396	0.431476	0.430136	0.412705	0.42454	0.246829	0.120619	0.155484	0.196506	0.112018	0.125115313	-0.772681386	0.004253607	-1.322751298
ENSG00000215251	FASTKD5	15.616616	15.003398	14.412261	13.262192	13.359742	13.086536	5.318231	5.609199	6.024466	0.74476262	-0.034804098	2.01E-51	-1.317028775
ENSG00000177692	DNAJC28	1.512517	2.00568	1.876088	1.970399	2.116233	1.91561	0.784315	0.840841	0.586877	0.497992814	0.197620218	0.00000249	-1.316882391
ENSG00000121454	LHX4	0.199215	0.32571	0.22932	0.228744	0.242062	0.188715	0.081799	0.106443	0.092971	0.94466665	-0.038768117	0.001806136	-1.316335986
ENSG00000160888	IER2	28.684242	25.730516	24.810223	15.074717	15.523457	15.794822	9.301392	12.37887	11.53139	1.09E-18	-0.622946874	1.79E-49	-1.316056491
NewGene 1370	--	1.263315	0.933927	0.704813	0.674527	0.365318	0.555339	0.403614	0.319676	0.297441	0.00497304	-0.72750595	0.000000175	-1.314553106
ENSG00000171295	ZNF440	2.961425	3.553855	3.839458	3.065195	2.82433	2.733339	1.416057	1.122474	1.313072	0.570473552	-0.094346651	1.32E-19	-1.311484698
NewGene 1198	--	0.576161	0.515324	0.629652	0.806591	1.146617	0.529936	0.599103	0.132194	0.498092	0.123957982	0.431992383	0.000078	-1.310193149
NewGene 1990	--	0.890537	1.708492	1.777178	1.180705	1.430395	1.207607	0.626145	0.440421	0.654982	0.613302027	-0.180656821	0.0000217	-1.308474897
ENSG00000228672	PROB1	0.384436	0.373918	0.311614	0.333664	0.3135	0.323951	0.104442	0.119453	0.176753	0.981452818	0.01138752	0.000400584	-1.306893935
ENSG00000165646	SLC18A2	0.305741	0.267156	0.321859	0.246469	0.325381	0.246369	0.092298	0.08297	0.160044	0.98679231	0.009603512	0.00517625	-1.305284966
ENSG00000170681	CAVIN4	4.942423	5.311867	5.38032	2.856061	2.365997	2.090358	2.225527	1.753331	1.964508	5.04E-12	-0.9524368	1.07E-20	-1.299841526
NewGene 1779	--	3.709799	3.235054	3.510185	3.667986	3.065369	3.343638	2.003599	1.519599	1.734748	0.830388108	0.025397048	6.73E-45	-1.298893572
ENSG00000128645	HOXD1	3.856514	3.584748	5.122641	0.855881	1.418656	0.989354	1.32463	1.306334	2.176452	9.18E-17	-1.792798384	1.73E-09	-1.298769033
ENSG00000256771	ZNF253	4.907847	5.168131	5.288551	4.310846	4.447139	3.590195	1.895822	2.262844	1.769059	0.172874428	-0.182859123	4.44E-22	-1.297090601
ENSG00000166189	HPS6	4.831394	4.823866	3.941061	4.945914	5.053266	4.663219	1.692804	1.493756	2.018116	0.069163643	0.254969461	1.01E-16	-1.295159717
ENSG00000161664	ASB16	1.914416	1.935874	1.743042	1.629759	1.551175	1.434085	0.578776	0.640972	0.924299	0.630414294	-0.132748885	0.000000296	-1.293050672
ENSG00000164651	SP8	6.693716	8.050089	8.385113	2.983487	3.642533	3.781518	2.849029	3.13746	3.006242	1.8E-15	-0.976348592	3.99E-27	-1.292555696
NewGene 1931	--	0.412626	0.694048	0.555331	0.462542	0.54489	0.475795	0.146366	0.175039	0.305427	0.995913804	0.003044132	0.001970259	-1.292142552
ENSG00000269343	ZNF587B	8.397296	8.600735	7.826457	7.494069	7.487332	7.77451	3.311922	2.395618	3.755363	0.926590225	-0.01376584	1.48E-25	-1.290561527
NewGene 156	--	2.937247	3.228586	2.910879	3.40878	2.856634	3.022589	1.197509	1.14264	1.234079	0.417082002	0.152520469	5.28E-13	-1.290378936

Gene ID	Gene name	Ctrl-1 FPKM	Ctrl-2 FPKM	Ctrl-3 FPKM	IFNγ-1 FPKM	IFNγ-2 FPKM	IFNγ-3 FPKM	Sora-1 FPKM	Sora-2 FPKM	Sora-3 FPKM	Ctrl vs IFNγ DESeq2 FDR	Ctrl vs IFNγ DESeq2 log2FC	Ctrl vs Sora DESeq2 FDR	Ctrl vs Sora DESeq2 log2FC
ENSG00000188070	C11orf95	14.254372	13.34767	13.940569	13.310463	12.307918	12.81306	5.474107	4.907668	6.041946	0.856107824	0.017602259	1.49E-64	-1.289367126
ENSG00000164056	SPRY1	14.786436	15.223075	14.477954	11.886325	12.094152	12.039287	4.538084	4.938484	7.981275	0.215140198	-0.120831748	4.51E-20	-1.288962747
NewGene_544	--	1.587894	1.348973	1.457193	0.944519	0.976089	1.408911	0.427905	0.603578	0.581055	0.933658623	0.028515194	0.00000015	-1.286805643
ENSG00000164171	ITGA2	6.885478	7.109623	6.714956	4.105577	3.784114	3.834308	2.339979	2.569526	3.022965	2.01E-21	-0.66674875	6.49E-46	-1.286173693
ENSG00000133858	ZFC3H1	3.714354	4.056276	3.621939	3.672563	3.219019	3.020231	1.423973	1.454405	1.687293	0.70505554	-0.051876506	3.19E-32	-1.286042553
ENSG00000102870	ZNF629	10.637271	10.258601	10.021291	10.804403	10.610985	10.538279	3.86998	3.59812	4.444936	0.003897035	0.194468546	4.02E-57	-1.285405274
ENSG00000179242	CDH4	0.428501	0.446618	0.361814	0.43981	0.31828	0.328766	0.125415	0.171997	0.1735	0.909995367	-0.044696851	0.0000195	-1.285234679
ENSG00000243207	PPAN-P2RY11	5.738863	4.791949	5.803957	4.565349	3.833749	4.502701	3.411411	2.767785	0	0.199830243	-0.192139224	0.583337958	-1.284995564
ENSG00000141026	MED9	10.381647	9.801409	11.132382	7.341744	7.254908	7.592021	3.85782	4.160986	4.189418	0.000719678	-0.345364747	5.62E-31	-1.284853702
ENSG00000183208	GDPGP1	1.04341	0.386554	0.578327	1.109807	0.41804	0.686494	0.275718	0.145181	0.479774	0.188315197	0.411261715	0.000430153	-1.279656807
NewGene_3267	--	0.780429	0.74406	1.097399	1.108686	0.695973	0.94125	0.38228	0.41384	0.242105	0.586714127	0.246453075	0.002972856	-1.278289209
ENSG00000196247	ZNF107	3.015335	4.041946	3.627867	3.642661	3.281107	3.437699	1.284688	1.425446	1.663186	0.555805639	0.089438358	5.95E-21	-1.277802104
ENSG00000074181	NOTCH3	0.875279	0.761492	0.948216	0.500304	0.48085	0.580459	0.302604	0.333727	0.360674	0.001493448	-0.569715818	3.83E-12	-1.276301237
ENSG00000178951	ZBTB7A	7.620735	8.430083	6.733745	6.475128	7.138089	7.205486	2.662034	2.727485	3.100837	0.147765655	0.14996312	4.88E-35	-1.276101827
ENSG00000174151	CYB561D1	1.146744	0.942106	0.948823	1.140943	1.317164	1.494479	0.533461	0.285839	0.490357	0.17780324	0.286867159	0.000000145	-1.273756956
ENSG00000134323	MYCN	1.695333	2.144558	1.638147	2.09605	1.899472	2.576021	0.704599	0.609439	0.809104	0.052675728	0.410412896	3.73E-08	-1.268443076
ENSG00000196109	ZNF676	4.284898	4.166087	4.161802	2.997359	3.208192	2.943492	2.030414	1.711502	1.198692	0.012478878	-0.344076276	6.83E-13	-1.268193805
ENSG00000188060	RAB42	1.196926	1.231441	0.826879	0.385567	0.532039	0.502337	0.31916	0.196267	0.799544	0.020100644	-0.908348938	0.0026293	-1.265950107
ENSG00000178567	EPM2AIP1	3.658528	3.469829	3.840005	4.024869	3.960441	3.734297	1.520349	1.261654	1.498357	0.006547853	0.242751008	4E-35	-1.265718168
ENSG00000198435	NRARP	5.253227	3.556475	4.506629	1.921349	2.275085	2.07076	1.828993	1.622599	1.752261	0.000000971	-0.935998931	8.25E-11	-1.26069353
ENSG00000173218	VANGL1	2.453732	1.970317	2.212886	2.206184	2.087747	2.063591	1.258506	0.766236	1.295316	0.248801154	0.142496062	1.31E-24	-1.26004096
ENSG00000106003	LFNG	4.252442	2.463646	2.380223	0.693965	0.599227	0.524716	0.875506	1.296632	1.311246	5.56E-16	-2.103899369	0.00000113	-1.259657997
ENSG00000117010	ZNF684	1.751169	2.033988	1.678047	0.707067	0.855915	0.722271	0.628475	0.812437	0.732937	0.0000056	-1.11501832	0.000000676	-1.256984517
ENSG00000213859	KCTD11	1.703522	1.19098	1.023193	1.050147	0.896184	1.148904	0.562177	0.514438	0.531914	0.401141043	-0.257556977	0.00000446	-1.256158511
NewGene_2492	--	2.080333	2.509919	2.767016	1.674705	2.078054	1.577129	1.021677	0.706298	1.169985	0.335721396	-0.313179313	0.0000524	-1.254663758
ENSG00000146278	PNRC1	35.76078	35.119267	36.15012	29.929701	32.98312	32.102549	12.938532	14.622127	14.839612	0.837939708	-0.020472272	9.9E-60	-1.254362988
ENSG00000138166	DUSP5	5.272465	4.439967	5.010719	3.246232	3.431173	2.838224	1.714757	1.609761	2.49568	0.000657362	-0.483726415	2.2E-14	-1.252866783
NewGene_3821	--	1.232622	0.964205	0.940295	0.990252	0.931954	0.929795	0.405615	0.391006	0.438893	0.969143463	0.008649314	6.07E-17	-1.251684163
ENSG00000236609	ZNF853	6.230734	5.452128	6.030478	4.006462	4.334533	4.327613	2.268068	2.072541	2.650132	0.001782064	-0.336046905	2.55E-27	-1.251145189
ENSG00000105497	ZNF175	1.767561	1.548694	1.857439	1.361964	1.298204	1.21223	0.673814	0.62004	0.742268	0.096897882	-0.255905667	2.02E-16	-1.249958654
ENSG00000114853	ZBTB47	1.621731	1.594549	1.607161	1.268162	1.141997	1.020887	0.654868	0.453763	0.795881	0.078884995	-0.343138716	4.71E-09	-1.248335832
ENSG00000277150	F8A3	0.689599	1.066717	0.895812	0.322375	0.8992	0.978621	0.283382	0.440529	0.303081	0.859986979	-0.108157202	0.002578747	-1.242860775
NewGene_5156	--	0.503782	0.272332	0.317262	0.734839	0.648742	0.663562	0.154296	0.15385	0.118762	0.000397909	1.039367173	0.003098061	-1.24262519
ENSG00000188211	NCR3LG1	1.258588	1.211579	1.194216	0.974769	0.859986	1.161551	0.493031	0.470208	0.491747	0.498224042	-0.140311629	2.48E-13	-1.239144431
ENSG00000164930	FZD6	3.976484	4.609067	4.201684	3.539995	2.912024	3.242247	1.660276	1.550074	1.874912	0.03899751	-0.277824597	4.63E-22	-1.238808315
ENSG00000189057	FAM111B	14.675689	17.2751	16.864318	14.993657	13.795451	13.330122	6.441654	6.492413	6.497951	0.538713903	-0.065522868	5.51E-46	-1.238794448
ENSG00000163596	ICA1L	0.168306	0.272588	0.224425	0.297378	0.237458	0.569089	0.068124	0.065563	0.110349	0.069130651	0.594399993	0.001729696	-1.237740325
ENSG00000174125	TLR1	0.912536	0.786252	0.685069	0.798332	0.892224	1.120178	0.400592	0.333534	0.196725	0.196280012	0.39220844	0.000425105	-1.235013523
ENSG00000187566	NHLRC1	2.974964	2.97358	2.401464	2.370572	2.242339	2.226465	1.179891	1.070889	1.068198	0.521649402	-0.142842748	5.03E-10	-1.233976662
NewGene_4946	--	0.613432	0.54329	0.406628	0.135161	0.136459	0.196309	0.2444	0.27796	0.076788	0.001522975	-1.510386157	0.016993404	-1.233931595
ENSG00000244242	IFITM10	0.161745	0.65254	0.293709	0.128015	0.779975	1.163656	0.069426	0.288844	0.073441	0.424669724	1.043486547	0.050074783	-1.231167204
ENSG00000196876	SCN8A	0.173572	0.102036	0.173711	0.095581	0.094212	0.083119	0.055439	0.074662	0.062751	0.114529112	-0.633260341	0.001924719	-1.231048539
ENSG00000185220	PGBD2	2.905937	2.673643	2.326328	2.498685	1.9134	2.185459	0.836985	1.252918	1.053698	0.517341183	-0.140875878	1.17E-09	-1.231036707
NewGene_1494	--	0.703317	0.676381	0.859051	0.825906	0.742988	0.694667	0.287238	0.221915	0.384032	0.511437252	0.164378973	0.000000779	-1.228975388
ENSG00000062282	DGAT2	3.096675	3.086094	2.564297	3.425909	3.13115	2.74465	1.393007	0.937921	1.162756	0.23344137	0.223167253	4.56E-10	-1.227623883
ENSG00000182405	PGBD4	0.785642	0.820109	0.851258	0.6611	0.780234	0.686082	0.245203	0.317677	0.423193	0.818894795	-0.059619328	4.53E-08	-1.227277538
ENSG00000196862	RGPD4	0.250613	0.255628	0.299011	0.321196	0.274428	0.258631	0.111998	0.167766	0.031207	0.576972113	0.229612839	0.01437918	-1.226153237
ENSG00000141232	TOB1	20.283188	21.090485	23.136368	22.51059	22.203104	22.872429	7.752565	8.4229	9.77993	0.006820883	0.215539396	9.69E-38	-1.224459199
ENSG00000090905	TNRC6A	9.969125	10.286272	10.693946	14.358665	11.667489	10.615556	5.62301	5.346049	5.413478	0.006740154	0.177570501	3.61E-61	-1.223905338
ENSG00000150991	UBC	282.131491	241.095398	215.229128	258.593058	249.206052	247.313632	85.407848	80.594041	92.851599	0.031344011	0.162935689	1.73E-60	-1.223561456
ENSG00000221944	TIGD1	1.971603	1.814859	1.544123	1.984865	1.836671	1.546931	0.780948	0.604203	0.756986	0.548647326	0.152137972	9.13E-08	-1.222425665
NewGene_6706	--	0.918091	0.905339	0.699765	0.686992	0.483578	0.6492	0.419999	0.515941	0.578169	0.331634583	-0.325113753	0.0000264	-1.221153905
ENSG00000124201	ZNFX1	3.327192	3.409198	3.281922	9.991262	9.501723	9.413152	1.246664	1.172564	1.647041	1.27E-125	1.659277877	1.52E-24	-1.220124171
ENSG00000127329	PTPRB	0.304902	0.158829	0.195668	0.107466	0.050867	0.061852	0.060463	0.101825	0.081185	0.00000103	-1.721218763	0.000526461	-1.219496659
NewGene_4125	--	1.172776	1.581075	0.950154	1.257262	1.080957	0.791049	0.588274	0.439478	0.455986	0.806304488	-0.100141613	0.00012207	-1.218156327
ENSG00000183260	ABHD16B	3.566717	3.761282	3.850066	2.69286	2.272225	2.723647	1.198493	1.482924	1.817381	0.051439011	-0.392283227	9.91E-09	-1.21623027
NewGene_5770	--	1.241122	1.428758	1.271244	1.215868	1.134686	1.061863	0.472514	0.456244	0.654655	0.804566511	-0.063466975	1.37E-08	-1.214625857
NewGene_5939	--	1.179704	0.618164	1.153865	1.105356	0.744355	0.709611	0.247175	0.143658	0.609439	0.950252767	0.032888523	0.008176694	-1.213623146
ENSG00000063978	RNF4	29.401424	30.682968	31.067781	25.639176	26.63807	27.397515	12.677252	12.651044	14.07997	0.87441714	-0.014700992	1.75E-66	-1.212600915
ENSG00000144560	VGLL4	19.923048	19.581344	19.810401	15.754831	14.431378	14.707747	8.046565	7.241976	9.149551	0.0000538	-0.275123207	1.82E-53	-1.208601096
NewGene_1615	--	1.671334	1.668191	1.880455	1.798046	1.875156	1.459261	0.48809	0.570632	1.038459	0.827011529	0.116980611	0.012369982	-1.20471757
ENSG00000221874	NF816-ZNF321	2.167613	1.278755	1.602612	1.223074	1.080534	1.012779	0.783074	0.654111	0.59274	0.217638033	-0.455480649	0.000755694	-1.200483915
ENSG00000172409	CLP1	18.681733	17.400169	17.697746	15.81743	16.493599	16.244854	6.551467	7.39552	6.737719	0.681847625	0.045948828	1.19E-36	-1.199997238
ENSG00000159388	BTG2	4.217567	3.726356	4.402027	2.355348	2.343876	2.54669	1.611569	1.877109	1.480212	0.0000161	-0.607594058	6.84E-14	-1.19910032
NewGene_3057	--	0.382832	0.570754	0.482581	0.182434	0.175154	0.188006	0.269422	0.098135	0.211232	0.002400457	-1.226011434	0.005738876	-1.199061863
NewGene_1628	--	0.469768	0.429762	0.349933	0.497993	0.42893	0.472297	0.080662	0.242605	0.17683	0.475740608	0.306982411	0.013605981	-1.198233828
NewGene_1085	--	1.512225	1.681195	1.687955	1.941287	1.782058	1.363013	0.719013	0.667745	0.59442	0.389151433	0.201776465	0.000000029	-1.195413239
NewGene_5565	--	1.32611	1.934424	1.017116	0.833699	1.532788	1.683382	0.399278	0.44227	0.490725	0.68921751	0.117553125	0.00000477	-1.193699484
ENSG00000165355	FBXO33	4.449874	4.451694	5.111753	4.281672	4.841225	3.923213	1.793315	1.750573	2.220186	0.797504037	0.043781319	9.7E-21	-1.190876744
ENSG00000155903	RASA2	2.59136	2.327443	3.102297	2.342476	2.467141	2.373855	1.01236	1.09559	1.257492	0.95987988	-0.010764964	4.26E-16	-1.18964846
ENSG00000117525	F3	1.593609	1.190012	1.303689	1.193887	0.883725	0.773537	0.734399	0.46216	0.487243	0.197472764	-0.390312218	0.0000602	-1.182282705
ENSG00000132801	ZSWIM3	1.708698	1.849243	1.631841	1.615337	1.909945	1.869143	0.71961	0.671908	0.754743	0.362943797	0.202414291	4.06E-08	-1.181825616
ENSG00000166507	NDST2	1.260099	0.892082	1.397018	2.773667	2.46211	2.95764	0.519836	0.566613	0.341297	1.13E-15	1.36177296	0.0000203	-1.181426765
ENSG00000104419	NDRG1	18.439799	12.484007	6.96934	7.781819	8.179665	7.093424	6.266465	5.141026	4.330149	0.254704319	-0.570494688	0.011208209	-1.181063248

Gene ID	Gene name	Ctrl-1 FPKM	Ctrl-2 FPKM	Ctrl-3 FPKM	IFNγ-1 FPKM	IFNγ-2 FPKM	IFNγ-3 FPKM	Sora-1 FPKM	Sora-2 FPKM	Sora-3 FPKM	Ctrl vs IFNγ DESeq2 FDR	Ctrl vs IFNγ DESeq2 log2FC	Ctrl vs Sora DESeq2 FDR	Ctrl vs Sora DESeq2 log2FC
ENSG00000164509	IL31RA	0.723548	0.557766	0.953519	0.049084	0.179199	0.208117	0.409643	0.280263	0.187059	0.0000126	-1.973115348	0.00225877	-1.180040044
ENSG00000174282	ZBTB4	4.330953	4.95355	4.739057	4.514318	4.057334	4.431267	2.091163	1.699789	2.027894	0.756997048	0.040555463	1.04E-27	-1.177538817
ENSG00000176102	CSTF3	27.639242	28.818927	28.537663	28.792825	28.311883	29.443849	14.573821	12.154918	12.819781	0.014887434	0.160548366	1.03E-58	-1.174730204
ENSG00000258311	AC009779.3	4.527246	4.655577	5.507724	3.158162	3.317582	3.166895	2.094394	1.672648	2.242546	0.000485225	-0.434361589	1.65E-17	-1.173358326
ENSG00000112796	ENPP5	1.409705	1.01678	1.157398	0.940331	0.782677	0.837883	0.335036	0.584333	0.451472	0.164563844	-0.360708904	0.00000603	-1.169958764
ENSG00000186017	ZNF566	3.023709	2.040667	2.615425	1.576399	1.760447	1.646684	1.142537	1.110269	1.137997	0.020509051	-0.442750447	8.48E-09	-1.168624276
ENSG00000182310	SPACA6	0.892373	1.300544	1.530079	1.594544	1.003319	1.263095	0.444083	0.581291	0.55369	0.701877516	0.19038661	0.00751105	-1.167596629
ENSG00000120055	C10orf95	1.026716	0.83915	0.881269	0.92161	0.893658	0.889261	0.492216	0.211572	0.444743	0.783767223	0.130495557	0.008755579	-1.167423191
ENSG00000185716	MOSMO	3.744768	3.97541	4.460702	3.978769	3.286009	3.950507	2.26272	2.924358	1.878031	0.723279636	-0.069178366	3.53E-15	-1.166910761
ENSG00000180938	ZNF572	1.110617	1.109998	1.190157	0.92961	1.074313	0.937007	0.615565	0.526119	0.265944	0.832431415	-0.065752731	0.0000882	-1.165723584
NewGene_4889	--	1.498573	1.191868	1.232139	1.430125	1.410392	1.176964	0.376618	0.623021	0.636311	0.649986257	0.170212355	0.001063005	-1.163728731
ENSG00000237988	OR2I1P	13.293031	11.682279	10.448855	99.858688	119.130882	130.512268	4.360945	5.697586	4.725312	1.04E-105	3.450899889	9.48E-24	-1.163387946
ENSG00000271079	CTAGE15	0.606393	0.332438	1.177749	0.890565	0.93881	0.838723	0.313912	0.400342	0.157547	0.274963419	0.480892294	0.020030513	-1.162479828
ENSG00000143344	RGL1	2.232039	1.833573	2.173379	1.507371	1.909033	1.696709	0.734423	0.778021	0.94511	0.482644471	-0.136317905	1.98E-12	-1.162353884
ENSG00000169429	CXCL8	5.089048	6.042411	7.502482	2.146954	3.157398	2.567631	2.317924	2.231865	3.14982	3.53E-09	-1.09704019	1.38E-09	-1.161983236
ENSG00000181016	LSMEM1	1.318672	1.269271	1.386534	1.277883	1.248095	0.893676	0.707135	0.398636	0.396455	0.680361256	-0.163415398	0.000615691	-1.160853747
ENSG00000120832	MTERF2	8.301723	7.592852	7.492681	7.44499	7.526345	6.08829	3.792405	4.42722	3.82518	0.916383811	0.017481637	8.8E-24	-1.160125423
ENSG00000174963	ZIC4	1.945113	1.242467	0.859817	0.855759	1.043296	0.978062	0.802484	0.394229	0.388287	0.928861081	-0.029937636	0.00000378	-1.159859001
NewGene_5204	--	0.82395	1.055393	1.215666	1.407173	1.31672	1.122575	0.522341	0.330224	0.429187	0.149325809	0.458271429	0.001506102	-1.159250623
NewGene_6645	--	1.087283	0.841792	0.787217	0.776313	0.790202	0.723954	0.419643	0.325955	0.393592	0.77383775	-0.101513187	0.0000598	-1.158380461
ENSG00000182983	ZNF662	2.034133	1.864402	2.00398	2.19284	1.444231	1.542614	1.079888	0.655159	0.882605	0.931035113	-0.023641667	2.94E-09	-1.157841237
ENSG00000155090	KLF10	10.045569	10.685481	10.969554	11.969799	11.039063	11.395967	4.19506	4.036318	5.274641	0.000309372	0.286478948	4.41E-30	-1.157661515
ENSG00000239305	RNF103	26.719498	28.095396	30.46278	20.489569	21.558424	19.87649	11.43032	11.576196	12.893232	0.00000969	-0.313986007	2.38E-54	-1.156050462
ENSG00000187123	LYPD6	3.634918	3.11099	3.213051	2.019923	2.012279	1.994602	1.358923	1.284634	1.524005	0.0000068	-0.568522616	2.32E-17	-1.15590461
ENSG00000139190	VAMP1	1.280398	1.231824	1.252265	2.215179	1.879649	1.832492	0.726099	0.509927	0.51334	0.0000023	0.796066713	0.000000735	-1.154890794
NewGene_31	--	1.332493	1.251227	1.403655	1.0361	0.84829	0.923836	0.59297	0.507917	0.864681	0.164392456	-0.289499639	1.58E-08	-1.15437663
ENSG00000148082	SHC3	0.146665	0.145433	0.159574	0.104881	0.177138	0.131806	0.06165	0.046896	0.067521	0.92561642	-0.051175209	0.005211426	-1.154176209
ENSG00000164749	HNF4G	5.600013	4.383131	5.39121	8.135692	7.739719	7.68839	4.091863	3.810185	4.006081	0.044677956	1.06148016	0.022693885	-1.15395429
ENSG00000131910	NR0B2	46.069088	36.047638	29.112265	34.345818	38.027359	37.799183	14.9367	15.063182	16.90974	0.65734144	0.133854793	3.02E-18	-1.153941174
NewGene_2432	--	0.701956	0.642051	0.549838	0.605071	0.716826	0.592812	0.324022	0.278619	0.184338	0.75405536	0.163505515	0.015421731	-1.150858843
ENSG00000130821	SLC6A8	11.609661	6.737774	6.138772	3.150983	3.42971	3.321076	3.20013	3.185427	4.251804	0.000491081	-1.119756208	0.000681436	-1.149094547
ENSG00000197782	ZNF780A	2.463111	3.031004	3.077958	3.274116	3.109342	2.816278	1.351023	1.256395	1.397848	0.310333492	0.142032136	3.51E-18	-1.147460463
NewGene_2947	--	1.587948	1.549465	1.146684	1.799214	1.51407	2.064255	0.603349	0.54781	0.649516	0.0460549	0.450426789	0.00000326	-1.147254947
NewGene_4208	--	3.671034	3.949098	4.065088	2.433148	2.156905	1.972501	1.661225	1.559937	1.743382	0.000000741	-0.708810037	5.63E-15	-1.146378737
ENSG00000197933	ZNF823	3.749173	3.397204	3.82045	2.640631	1.975304	2.209666	1.968638	1.175445	1.512682	0.00065338	-0.552225528	1.1E-09	-1.144420869
NewGene_5838	--	1.469567	1.273417	1.179683	0.878384	0.628455	1.069068	0.445454	0.580449	0.51058	0.079941909	-0.485332372	0.0000202	-1.142944029
ENSG00000083844	ZNF264	1.570119	2.000975	1.715924	1.768386	1.619808	1.722029	0.734438	0.870075	0.89845	0.827672643	0.034758059	7.26E-21	-1.140123707
ENSG00000129965	INS-IGF2	4.864856	4.479724	3.535133	5.33737	4.855517	5.601151	0	2.933719	2.517127	0.053344809	0.438750912	0.579299476	-1.138626453
NewGene_2115	--	0.901025	1.00269	0.642804	0.589037	0.834676	0.775135	0.468077	0.363903	0.246966	0.857746898	-0.06473389	0.0000981	-1.138325295
ENSG00000130544	ZNF557	3.223035	2.952912	3.016274	2.974646	3.159305	2.831885	1.365359	1.43817	1.35699	0.613075495	0.080486226	5.61E-17	-1.137958622
NewGene_2016	--	2.720723	4.082229	2.582936	5.724096	5.405573	3.681919	1.548882	1.644061	1.10758	0.000000116	0.737576639	3.62E-12	-1.136953227
ENSG00000197363	ZNF517	3.042882	2.074132	2.407945	1.379447	1.825461	1.794883	1.333112	0.913669	0.951454	0.393223997	-0.218746233	0.000000345	-1.135882736
NewGene_3032	--	0.926888	0.939994	0.857637	0.446713	0.659757	0.465552	0.365603	0.452719	0.435952	0.002228639	-0.724880516	0.00000168	-1.135580302
NewGene_4379	--	0.653901	0.623755	0.725309	0.6491	0.717683	0.637233	0.140812	0.26714	0.447197	0.777867674	0.144747137	0.021817993	-1.135188329
NewGene_5297	--	10.232139	9.152493	10.206219	7.097932	6.762413	7.003655	6.505385	4.960951	3.871796	0.058209908	-0.267839451	1.89E-12	-1.134781171
ENSG00000163257	DCAF16	21.194715	23.790051	24.889643	21.29689	17.731102	19.456898	9.950237	9.319118	10.650344	0.232201575	-0.109983021	4.64E-50	-1.132173908
ENSG00000180917	CMTR2	2.89799	2.819094	3.612235	2.683289	2.635025	2.834185	1.52911	1.11503	1.201263	0.929366118	0.017713007	3.19E-11	-1.125213969
NewGene_771	--	1.582834	1.713215	1.849992	1.790956	1.436352	1.532483	0.776649	0.790749	0.838322	0.853136186	0.035724934	7.48E-17	-1.125106467
ENSG00000145826	LECT2	3.138014	1.907305	2.022556	1.744258	1.286664	1.739061	0.626307	1.459454	0.942002	0.097127259	-0.534904613	0.001055471	-1.124063586
NewGene_5267	--	0.805285	0.769403	0.746951	0.871912	0.743427	0.626226	0.376608	0.290836	0.33145	0.719645105	0.092952583	0.000000077	-1.123009318
ENSG00000261611	AC010547.4	0.780898	0.794624	1.96764	0.697575	0.071012	0.06639	0.063715	1.240022	0.177404	0.158689518	-1.947956281	0.448111231	-1.122728087
ENSG00000168612	ZSWIM1	4.262234	4.504042	4.236872	3.696083	3.777901	3.745192	1.759903	2.009505	1.820918	0.700135039	-0.064881944	3.7E-15	-1.12232288
ENSG00000103044	HAS3	1.827055	1.077551	0.840619	0.746574	0.580729	0.79376	0.471297	0.647968	0.557904	0.00030477	-1.015006366	0.0000266	-1.119719322
ENSG00000137185	ZSCAN9	10.979544	10.683603	10.697198	9.076853	8.06615	7.94085	4.989524	4.606887	4.504499	0.095364653	-0.194974633	7.53E-23	-1.119010742
ENSG00000198556	ZNF789	7.596837	8.991574	9.487521	8.795343	6.950703	7.750335	3.820743	3.830464	3.795887	0.911915897	0.021482595	2.6E-18	-1.11827895
ENSG00000188229	TUBB4B	290.577179	279.833008	265.002289	228.334244	221.327515	225.499542	121.238312	118.880608	120.723412	0.003309161	-0.160122362	1.49E-82	-1.117933534
ENSG00000167380	ZNF226	6.832708	5.72974	6.756752	6.625011	5.708781	5.767064	3.461199	4.543852	3.035963	0.983088931	-0.003782888	4.51E-20	-1.117445374
ENSG00000138640	FAM13A	2.938379	2.495063	1.33528	1.160061	1.072398	0.986851	0.627863	0.792627	0.870988	0.000398516	-0.725783382	7.54E-08	-1.116810092
NewGene_6044	--	0.65441	0.472664	0.433478	0.265205	0.337836	0.292481	0.253003	0.304659	0.12031	0.162234389	-0.653772478	0.020274413	-1.115444719
ENSG00000183850	ZNF730	2.745768	2.618246	1.939928	2.011726	1.050551	1.116625	0.800101	0.741607	0.801997	0.184782145	-0.35546726	0.00000822	-1.114954704
ENSG00000213865	C8orf44	1.200217	1.588932	1.950543	1.239822	2.142469	2.340177	0.614046	0.726582	0.520029	0.243519394	0.387124109	0.002332675	-1.114403441
ENSG00000111799	COL12A1	0.15618	0.219606	0.36736	0.091199	0.068562	0.071247	0.115223	0.071367	0.074325	0.000302882	-1.238196269	0.001464849	-1.113591491
NewGene_6160	--	2.366501	2.545558	2.238957	2.638295	2.088224	2.199501	0.942585	0.921363	1.222	0.758556721	0.105373693	0.000134323	-1.112803315
ENSG00000114978	MOB1A	19.497959	20.800106	23.619402	24.773922	24.460752	25.544207	9.602334	8.643454	9.501657	0.000000217	0.374307227	5.87E-43	-1.111463572
ENSG00000165879	FRAT1	3.835566	3.308777	3.02533	3.649209	3.923578	3.754445	1.391486	1.526614	1.484531	0.050353882	0.30234496	4.5E-11	-1.109826179
NewGene_645	--	0.942188	1.047958	0.980134	0.822565	0.68049	0.929161	0.467451	0.392769	0.421169	0.76640527	-0.130506389	0.001930371	-1.108464533
ENSG00000188343	FAM92A	1.212235	1.632217	1.479073	1.360978	1.162981	1.53373	1.183821	0.369545	1.19747	0.33519979	-0.233684987	0.0000559	-1.108203231
NewGene_61	--	1.537878	1.161456	0.96282	0.830149	1.031447	0.706748	0.646738	0.401752	0.51927	0.334363696	-0.362785823	0.001584738	-1.107799804
ENSG00000163820	FYCO1	1.810258	1.911281	2.006152	2.366728	1.983341	2.509976	0.798253	0.94908	0.879604	0.000413947	0.388094247	1.33E-19	-1.107173464
ENSG00000182810	DDX28	6.700988	5.621679	5.605923	5.851819	5.425387	5.992342	2.618811	2.594961	2.594222	0.569901302	0.093582017	6.72E-15	-1.103455089
ENSG00000168386	FILIP1L	4.557069	5.417467	6.314116	5.352102	5.409544	4.849586	2.352003	2.563464	2.277966	0.584285529	0.091644383	1.5E-14	-1.101499158
ENSG00000178201	VN1R1	1.090353	1.016865	1.334417	1.186105	0.65493	1.008279	0.614493	0.436933	0.445546	0.759913294	-0.120681357	0.000255279	-1.101057069
NewGene_2118	--	1.876731	2.169471	1.857401	1.891794	1.798733	1.533105	0.80231	0.910071	0.859	0.920275254	-0.02921833	0.000000129	-1.100815537
NewGene_440	--	0.416729	0.559749	0.761444	0.747156	0.430545	0.57768	0.24293	0.21136	0.301995	0.576226822	0.165511913	0.00000171	-1.097513828

Gene ID	Gene name	Ctrl-1 FPKM	Ctrl-2 FPKM	Ctrl-3 FPKM	IFNγ-1 FPKM	IFNγ-2 FPKM	IFNγ-3 FPKM	Sora-1 FPKM	Sora-2 FPKM	Sora-3 FPKM	Ctrl vs IFNγ DESeq2 FDR	Ctrl vs IFNγ DESeq2 log2FC	Ctrl vs Sora DESeq2 FDR	Ctrl vs Sora DESeq2 log2FC
NewGene 1263	--	1.750138	1.773702	1.789001	1.390009	1.213507	1.183442	0.784777	0.735913	0.636177	0.080209647	-0.259944502	1.8E-11	-1.096464832
ENSG00000166839	ANKDD1A	0.856422	1.237083	1.189966	1.271837	1.017876	0.85565	0.361514	0.485082	0.429935	0.774693151	0.113141382	0.001008969	-1.095884937
ENSG00000155592	ZKSCAN2	1.782277	1.824134	1.506917	1.569837	1.659396	1.49762	0.925374	0.635257	0.722125	0.855402859	0.034889955	2.39E-12	-1.095818332
ENSG00000179886	TIGD5	1.629867	1.607898	1.470953	1.356114	1.682355	1.931395	0.729501	0.717535	0.611082	0.220086729	0.229496042	1.24E-10	-1.095064593
ENSG00000196267	ZNF836	2.194246	2.087666	1.977516	0.894494	1.563796	1.211316	1.239746	0.829265	0.67304	0.00338822	-0.654570745	0.00000224	-1.094397749
ENSG00000120549	KIAA1217	2.454399	2.487768	2.926084	4.533286	3.894589	4.227945	1.266558	1.064252	1.130301	4.62E-18	0.834604021	4.33E-18	-1.093524317
ENSG00000198888	MT-ND1	3163.887695	3497.44165	3591.343262	3584.431885	3291.028076	3627.355469	1383.153198	1554.303711	1571.958496	0.0029849	0.18189257	2.88E-57	-1.091659896
ENSG00000132010	ZNF20	2.229968	2.324332	2.176298	1.594532	2.107694	2.074947	0.776732	1.269315	0.970098	0.676940014	-0.099655828	9.58E-08	-1.089713985
ENSG00000009413	REV3L	2.920445	3.362604	3.168866	3.01742	2.842354	2.848382	1.970018	1.368063	1.617785	0.734758287	0.039366329	2.18E-25	-1.088530306
NewGene 2114	--	0.623469	0.59644	0.670263	0.488626	0.281757	0.429473	0.281445	0.235061	0.318291	0.149428443	-0.495920337	0.000543741	-1.084575788
ENSG00000214562	NUTM2D	0.963941	0.647518	0.668693	0.891239	0.871441	0.571509	0.30111	0.267278	0.427522	0.979566581	-0.012240178	0.000957401	-1.08281013
ENSG00000175787	ZNF169	2.15405	2.091773	2.373093	1.832216	1.410769	1.47	1.064256	0.763214	0.975712	0.105154459	-0.359644489	0.00000037	-1.082312691
ENSG00000183741	CBX6	2.485437	2.406788	2.662161	1.994357	1.797349	1.881288	1.078947	1.059634	1.184763	0.0252232	-0.28084687	7.26E-18	-1.081132111
ENSG00000079308	TNS1	1.487897	1.560786	1.174101	0.676499	0.941016	0.762395	0.58703	0.555602	0.944905	0.00000717	-0.761853607	6.4E-09	-1.080786444
ENSG00000196670	ZFP62	7.039817	7.782911	7.489462	7.535776	6.6477	6.159719	3.355235	3.469687	3.24649	0.97208061	0.005951922	4.24E-25	-1.080470114
ENSG00000151117	TMEM86A	1.092652	0.958724	0.976645	2.482659	2.644556	2.662789	0.361866	0.576698	0.391889	4.76E-23	1.507883164	0.00007	-1.077734775
ENSG00000197013	ZNF429	2.282183	1.555131	2.2422	1.395303	2.12679	2.062752	1.057251	0.791378	0.910577	0.948132983	0.024244155	0.0000107	-1.077455783
NewGene 4334	--	5.334533	5.943464	5.377139	6.22097	5.584083	4.845698	2.19668	1.97226	2.814804	0.279347586	0.100998346	4.1E-28	-1.074928132
ENSG00000168876	ANKRD49	4.729905	5.312963	4.911215	5.129478	4.43022	4.789313	2.314898	2.188862	2.037491	0.421462925	0.117344251	2.96E-14	-1.073074987
ENSG00000163026	WDCP	3.715484	4.012884	4.239634	3.185939	3.084806	2.608969	1.745639	1.643849	1.961278	0.033839895	-0.285738016	5.6E-17	-1.072922711
ENSG00000168795	ZBTB5	11.516528	11.19589	11.542075	11.716604	11.207303	10.916641	5.572424	4.999904	4.682334	0.089541161	0.129305444	3.56E-33	-1.072008599
ENSG00000187189	TSPYL4	2.66307	2.605958	2.334763	2.212232	2.220118	2.271136	1.21207	1.057454	1.118196	0.858960315	-0.034791295	8.16E-13	-1.071785355
NewGene 75	--	0.735104	1.049442	0.792931	0.831675	0.813026	0.77651	0.389073	0.267082	0.500505	0.814838746	0.087230907	0.001083565	-1.069909316
ENSG00000170881	RNF139	13.584178	12.750791	13.119735	12.061794	12.11402	10.762002	5.850537	5.580825	6.241606	0.793920517	-0.032322468	3.52E-33	-1.069483627
ENSG00000184500	PROS1	27.573465	32.307041	30.843759	34.948307	34.580912	36.112673	15.580314	13.268753	11.16472	5.95E-08	0.362211933	2.87E-24	-1.069391309
ENSG00000050130	JKAMP	11.204473	12.121652	13.239234	9.253684	10.645089	10.241952	5.207193	5.969957	5.209343	0.597539251	-0.071434503	1.57E-19	-1.069168519
NewGene 3027	--	0.755444	0.659736	1.185904	1.044832	0.479463	0.522131	0.355983	0.313349	0.373609	0.803385483	-0.124331968	0.00657392	-1.067777155
ENSG00000107968	MAP3K8	5.191067	4.315202	3.752057	5.59191	5.478649	4.656781	2.085879	2.312072	2.154155	0.002140458	0.465425869	3.55E-10	-1.067282563
ENSG00000168389	MFSD2A	0.798486	0.699356	1.085377	0.316958	0.0685	0.210693	0.27596	0.427501	0.449294	0.0000214	-1.926250095	0.004424437	-1.064699856
ENSG00000273604	EPOP	3.225279	2.887229	2.969882	2.00797	1.944523	1.754241	1.245665	1.179716	1.666562	0.000167523	-0.524594982	8.56E-12	-1.062946818
NewGene 5375	--	1.174313	0.830271	1.081809	0.411176	0.499977	0.550153	0.642619	0.461908	0.598051	0.00000459	-0.91389706	0.000000231	-1.062096315
ENSG00000284773	AC114490.3	0.900528	0.952801	0.900359	0.752862	0.600821	0.753545	0.352098	0.366313	0.515389	0.447482041	-0.233327992	0.000166582	-1.06066101
ENSG00000196724	ZNF418	2.132497	2.578485	2.805215	2.443298	1.878136	1.974797	1.615232	1.327804	0.966688	0.720925121	-0.074317932	2.32E-08	-1.059944582
ENSG00000170191	NANP	4.079665	4.583238	4.417317	4.645051	4.782544	4.670805	1.838224	1.651302	2.421445	0.018982528	0.255651639	8.64E-15	-1.059219931
ENSG00000162733	DDR2	0.129996	0.136165	0.207157	0.2532	0.270471	0.182451	0.095135	0.061462	0.076385	0.030745899	0.7124057	0.01236302	-1.05870342
ENSG00000186812	ZNF397	3.086264	3.551766	3.538829	2.747261	3.143353	2.539199	1.805572	1.749994	1.712532	0.21120837	-0.190798668	1.2E-14	-1.057736549
ENSG00000198429	ZNF69	3.287772	4.092179	4.066463	2.237951	2.027096	2.129788	2.12094	1.895912	1.0186	0.000574376	-0.654605723	0.0000409	-1.055106654
NewGene 3497	--	1.468407	2.105581	2.040143	2.156608	1.861107	1.487982	1.017822	0.691556	1.101539	0.802439433	0.063869267	1.35E-09	-1.053574275
ENSG00000100354	TNRC6B	3.458445	3.899369	3.198503	3.325435	3.074912	3.390899	1.840193	1.747035	2.496504	0.00638026	0.193972956	2.65E-36	-1.053079883
ENSG00000188215	DCUN1D3	3.320596	3.782244	4.178652	3.293112	3.626795	3.456644	1.518919	1.711047	1.870538	0.858960315	0.028914577	5.51E-18	-1.052922475
ENSG00000124659	TBCC	13.306166	11.834579	12.258653	14.784774	13.933345	15.191213	5.850017	5.004059	6.073907	0.000097	0.37867312	9.59E-20	-1.052588111
ENSG00000214029	ZNF891	0.684796	0.75757	0.657573	0.834183	0.645702	0.602921	0.359212	0.306831	0.281988	0.481719288	0.130582735	1.87E-11	-1.051279189
ENSG00000204311	PJVK	2.895096	2.794764	2.881385	3.339122	2.05865	2.587643	1.678177	1.502814	0.911649	0.97025095	-0.013443006	0.0000517	-1.050928611
ENSG00000240053	LY6G5B	11.386127	14.229552	14.454245	12.172601	9.15623	10.24534	7.092567	6.222821	6.245156	0.045767583	-0.252967824	2.66E-21	-1.050326833
ENSG00000163877	SNIP1	4.510976	4.583839	4.286951	4.036372	4.076919	4.473901	2.233134	1.957686	2.170964	0.743063667	0.045849319	2.04E-20	-1.049367431
ENSG00000184923	NUTM2A	0.646441	0.556949	0.519556	0.636518	0.506444	0.532273	0.267558	0.195612	0.311209	0.757339068	0.123609446	0.002360934	-1.049154386
NewGene 4978	--	0.821645	0.653185	0.756801	0.778973	0.666065	0.539592	0.535279	0.395061	0.19644	0.993484931	0.005462627	0.013650915	-1.047413788
ENSG00000196227	FAM217B	4.394106	4.948551	4.798037	5.472913	5.028979	5.106052	2.253774	2.079653	2.126643	0.001696192	0.295185257	5.06E-23	-1.046982439
ENSG00000161835	GRASP	0.632603	0.513874	0.756934	0.800295	0.350678	0.64921	0.300978	0.25552	0.250873	0.857097384	0.106463559	0.019915445	-1.046807819
ENSG00000196659	TTC30B	1.073059	1.12022	0.948514	0.837744	0.720332	0.961721	0.597967	0.408884	0.410614	0.539428214	-0.167463421	0.0000192	-1.046511055
ENSG00000251247	ZNF345	0.704547	1.396424	1.283872	0.848685	0.883595	0.662405	0.588359	0.506559	0.604001	0.181274616	-0.423699621	0.000626014	-1.042913952
ENSG00000197557	TTC30A	0.65665	0.721484	0.735927	0.536582	0.540681	0.53038	0.303154	0.275468	0.383978	0.30661191	-0.247894481	0.00000767	-1.041165048
ENSG00000066279	ASPM	23.103416	26.10383	26.344055	30.470408	26.494041	27.211983	11.837502	10.634942	12.037713	0.0000064	0.308226583	3.41E-51	-1.040731228
NewGene 4511	--	0.983042	1.169592	1.373	0.927657	0.802297	0.934929	0.650009	0.619859	0.574718	0.164689679	-0.303185318	0.000000228	-1.040657207
NewGene 6221	--	0.437502	0.701857	0.503716	0.586334	0.734396	0.876411	0.234431	0.127297	0.309142	0.029938484	0.587220058	0.00161073	-1.038699999
NewGene 1851	--	1.07109	0.543875	0.696845	0.644814	0.559655	0.643977	0.326099	0.413727	0.272535	0.784458984	0.10693376	0.001766112	-1.035326518
ENSG00000196757	ZNF700	9.141586	10.412712	12.457517	9.917363	9.630379	9.538543	5.067871	4.756382	4.689672	0.920959485	0.017313697	7.95E-18	-1.035054569
ENSG00000171223	JUNB	23.635216	23.355503	23.933216	12.800803	12.428921	14.495356	10.859745	10.030826	11.612372	8.98E-17	-0.68686238	3.97E-36	-1.035044206
ENSG00000186417	GLDN	0.256337	0.317425	0.446838	0.982132	0.99643	1.133023	0.299988	0.207111	0.138581	0.00000111	1.300663452	0.0139557	-1.033656922
ENSG00000198040	ZNF84	14.235458	12.855479	13.893062	11.257348	10.785099	10.537298	6.780844	7.13272	6.591781	0.008494616	-0.214015243	4.44E-36	-1.030169655
ENSG00000149289	ZC3H12C	0.493695	0.583233	0.681695	0.535489	0.438913	0.511143	0.265277	0.239987	0.303026	0.692289045	-0.103662473	0.00000117	-1.029734297
ENSG00000244694	PTCHD4	0.758324	0.830128	1.106349	0.605255	0.567388	0.339338	0.404201	0.310258	0.499782	0.041487469	-0.663459091	0.00061342	-1.029630098
ENSG00000184378	ACTRT3	5.413213	4.472133	4.438293	3.509913	3.255265	3.812971	2.144438	2.248139	2.192259	0.087394779	-0.287869044	1.96E-10	-1.027719145
ENSG00000173542	MOB1B	6.295814	7.4219	7.87323	7.146871	6.384497	6.558394	3.371635	2.906195	3.688576	0.7573537	0.039321502	6.39E-25	-1.027343185
ENSG00000124120	TTPAL	3.449599	3.451711	3.894304	3.318166	3.200639	3.454504	1.713181	1.554309	1.834967	0.846614545	0.02954812	5.6E-19	-1.025670195
ENSG00000183808	RBM12B	3.673481	3.847914	3.310667	3.655274	3.040475	3.372623	1.717323	1.672036	1.650049	0.734880104	0.049338006	2.3E-21	-1.024587703
ENSG00000170345	FOS	0.995921	1.001777	1.080202	0.656116	0.520712	0.565778	0.446032	0.42543	0.691928	0.008092283	-0.873491314	0.002085507	-1.023962509
ENSG00000116701	NCF2	4.473226	3.423329	3.468027	0.959315	0.84709	0.706367	1.619459	1.767763	2.519941	2.24E-30	-2.402326662	8.53E-08	-1.023902202
NewGene 5626	--	2.9629	3.190871	3.678608	2.714269	2.121696	2.475115	1.9121	1.376154	1.909722	0.002325122	-0.407413591	2.51E-13	-1.022886369
ENSG00000204569	PPP1R10	19.665836	19.532047	19.909836	20.118326	19.498003	19.802744	8.855289	8.4352	10.034521	0.010774449	0.154765962	3.13E-48	-1.022638648
ENSG00000188295	ZNF669	10.854164	10.132615	11.435107	8.800949	7.38853	8.664189	5.363033	4.78736	4.691789	0.05887382	-0.233673224	1.33E-17	-1.022461366
ENSG00000135048	CEMIP2	57.764467	57.05515	65.456936	44.849051	44.862725	43.403299	26.838466	31.08124	32.331511	0.000000854	-0.294644886	1.09E-36	-1.021908819
NewGene 5161	--	0.734976	0.721863	0.774733	0.092492	0.187661	0.133111	0.419284	0.30629	0.32322	1.46E-13	-2.199206999	0.000105103	-1.021669957
ENSG00000175329	ISX	4.515588	3.439879	2.646247	2.204804	2.121876	2.357647	1.376005	1.430094	1.994177	0.007628439	-0.487963354	0.000000154	-1.021345621

Gene ID	Gene name	Ctrl-1 FPKM	Ctrl-2 FPKM	Ctrl-3 FPKM	IFNγ-1 FPKM	IFNγ-2 FPKM	IFNγ-3 FPKM	Sora-1 FPKM	Sora-2 FPKM	Sora-3 FPKM	Ctrl vs IFNγ DESeq2 FDR	Ctrl vs IFNγ DESeq2 log2FC	Ctrl vs Sora DESeq2 FDR	Ctrl vs Sora DESeq2 log2FC
ENSG00000112218	GPR63	0.407719	0.323812	0.458019	0.192986	0.306628	0.328213	0.14953	0.157258	0.240145	0.305083664	-0.376169567	0.001717155	-1.021273299
NewGene_1470	--	1.581431	1.455578	1.324506	1.199005	1.378187	1.185974	0.657967	0.510974	0.86183	0.789813008	-0.09875539	0.001082377	-1.019880676
NewGene_4990	--	0.520418	0.517636	0.365602	0.323843	0.295712	0.406655	0.18293	0.217443	0.216473	0.621352115	-0.220596773	0.006391616	-1.016736221
ENSG00000141034	GID4	1.275395	1.591141	1.952509	1.843248	1.73838	2.001571	0.838573	0.867344	0.680187	0.211994398	0.262242291	0.00000161	-1.016392636
ENSG00000275183	LENG9	1.433953	1.13618	0.718106	1.189215	0.99155	0.885391	0.57657	0.425539	0.521357	0.937811121	0.038634315	0.005330065	-1.014716853
ENSG00000204237	OXLD1	14.50479	13.761451	12.046154	11.959097	11.600401	13.468702	5.635819	6.291534	7.319421	0.810197669	0.04779465	9.54E-11	-1.01414114
ENSG00000170340	B3GNT2	13.999942	13.835721	15.346542	14.815511	14.8749	14.664638	6.179419	6.611132	7.257147	0.025984279	0.18619373	2.75E-28	-1.014025651
ENSG00000160233	LRRC3	0.270591	0.224765	0.24282	0.167633	0.190889	0.212883	0.10939	0.149068	0.079595	0.628409138	-0.222499947	0.015094939	-1.012702967
ENSG00000111859	NEDD9	30.032991	28.387676	31.245598	13.691866	16.673021	14.912193	12.985643	14.418884	15.706357	7.49E-28	-0.835584432	2.47E-42	-1.012567441
ENSG00000212719	C17orf51	1.005583	0.909247	0.991071	0.729386	0.688416	0.683357	0.519481	0.426822	0.403056	0.059557132	-0.321321999	1.49E-08	-1.012111389
ENSG00000164061	BSN	0.143688	0.08454	0.099937	0.090664	0.057149	0.084313	0.053935	0.037937	0.06175	0.415447262	-0.352043879	0.008799606	-1.011320049
ENSG00000165029	ABCA1	9.031091	9.570707	9.339584	11.218774	10.640356	10.481328	4.750667	4.149602	4.631628	1.57E-10	0.360286102	2.85E-47	-1.010867771
ENSG00000173875	ZNF791	4.805452	5.301204	5.373909	4.5469	4.765275	4.614728	2.362543	2.136784	2.655721	0.935248325	-0.012190609	4.71E-22	-1.010247701
ENSG00000244462	RBM12	29.563012	30.223997	27.964497	27.50099	26.626462	24.266083	12.287777	14.498771	13.813369	0.719438662	-0.030667445	2.01E-51	-1.009167963
ENSG00000188549	CCDC9B	4.753304	4.837869	5.230193	2.657196	2.26797	2.673492	2.473288	1.968759	2.502113	1.37E-13	-0.806566818	3.03E-17	-1.00707462
NewGene_3203	--	0.312723	0.467157	0.408383	0.558053	0.392187	0.342104	0.269589	0.161986	0.133354	0.606710225	0.228554534	0.014961068	-1.002271836
ENSG00000181449	SOX2	7.527578	6.7566	7.867921	5.996093	5.472257	6.509571	3.282822	3.621806	3.450576	0.254139521	-0.153235	1.75E-16	-1.001473037
ENSG00000197852	INKA2	0.66771	0.826759	0.578229	0.763443	0.593642	0.581152	0.356127	0.306515	0.305587	0.875055826	0.051821364	0.0000438	-1.000426667
ENSG00000166750	SLFN5	0.506994	0.385768	0.536199	0.931577	1.014539	0.805335	0.214324	0.235014	0.1636	7.05E-15	1.178182016	0.0000171	-0.998573715
ENSG00000114268	PFKFB4	3.194263	2.010603	1.836876	2.045043	1.888867	1.994361	1.158782	0.931623	1.20156	0.720005308	-0.099805116	0.00000558	-0.998248553
ENSG00000198346	ZNF813	1.689912	1.670935	1.629629	1.287399	1.081539	1.128185	0.72342	0.752086	0.853922	0.006699846	-0.390027451	1.43E-11	-0.998058455
ENSG00000118762	PKD2	2.202592	2.605068	2.767768	2.200354	1.96797	1.891434	1.156429	1.200072	1.233834	0.291820222	-0.175873652	1.67E-11	-0.997305843
ENSG00000186765	FSCN2	1.661865	1.333919	0.970594	1.082726	1.367393	1.152171	0.609675	0.512347	0.717682	0.981400982	0.011791729	0.004024997	-0.996384546
ENSG00000108932	SLC16A6	1.169283	1.572787	1.647864	1.759471	1.45337	1.599822	0.630215	0.674478	0.691044	0.116578401	0.379316433	0.0000903	-0.995875894
NewGene_4987	--	0.638763	0.732253	0.408973	0.778133	0.678615	0.599317	0.35101	0.192141	0.470731	0.2204903	0.485447663	0.023586965	-0.995630901
ENSG00000182022	CHST15	0.553418	0.468644	0.463154	0.459902	0.402243	0.366697	0.268649	0.205212	0.335424	0.798186325	-0.100266748	0.001384928	-0.993519708
ENSG00000140297	GCNT3	1.14365	0.904659	0.979936	1.222821	1.179947	1.034942	0.391811	0.528578	0.57884	0.311554078	0.223061601	0.00000765	-0.991421081
ENSG00000125968	ID1	124.992785	129.780141	137.599981	108.18323	104.925781	105.047512	54.428383	63.81437	66.358742	0.009201281	-0.165536802	3.28E-34	-0.988905697
ENSG00000146757	ZNF92	6.31747	6.354617	7.724024	7.782196	7.318588	7.107549	3.337768	3.173822	3.167212	0.01505982	0.272469629	1.45E-16	-0.988262724
ENSG00000188707	ZBED6CL	41.229118	37.069935	39.857826	61.509762	75.00502	67.805145	16.555193	17.458763	22.002638	4.41E-36	0.938459312	4.05E-29	-0.987755064
ENSG00000160229	ZNF66	2.412812	2.620741	2.761738	1.705582	1.561804	2.14946	1.506524	1.106242	1.98742	0.408962264	-0.261402891	0.000819625	-0.984841627
ENSG00000198521	ZNF43	7.524094	8.620286	8.529857	7.303819	6.061675	6.342155	4.595069	3.075374	3.735829	0.221280589	-0.135158143	1.49E-20	-0.984550201
NewGene_6296	--	2.093927	2.415584	2.468096	3.856476	3.477669	3.467697	1.512	1.13938	1.162182	1.83E-14	0.711954456	4.77E-14	-0.984083649
ENSG00000143147	GPR161	2.162334	1.950065	2.159635	0.757101	0.917473	1.268279	0.964251	0.996363	0.950703	1.76E-16	-1.135336592	1.1E-14	-0.983807684
ENSG00000124875	CXCL6	3.144455	2.23847	3.581526	0.776444	1.212633	1.338901	1.388294	1.220862	1.6292	0.00000122	-1.257039896	0.00003	-0.980812692
ENSG00000007944	MYLIP	5.21532	4.895065	4.558695	6.362586	5.966008	6.301235	2.301896	2.01163	2.903379	0.000024	0.466032675	8.99E-12	-0.979507933
NewGene_1716	--	0.501818	0.624216	0.458138	0.611463	0.576634	0.483113	0.278031	0.20349	0.272545	0.45398648	0.221741457	0.000562629	-0.978284654
ENSG00000116539	ASH1L	8.682173	8.816051	8.827849	9.642993	9.69843	9.235488	4.319618	3.882175	4.48097	0.00000889	0.258732656	2.6E-47	-0.977171731
ENSG00000183153	GJD3	3.085188	2.50709	2.271936	2.854778	2.882014	3.493157	1.603802	0.950222	1.194849	0.20840587	0.372633096	0.002825046	-0.976955315
ENSG00000198774	RASSF9	5.508379	6.14649	6.482809	3.263234	3.342127	3.195238	2.605358	2.672523	3.388241	2.26E-16	-0.740551581	1.64E-19	-0.97609457
ENSG00000170100	ZNF778	1.534621	1.475247	1.682922	1.397774	1.192052	1.319308	0.72946	0.867271	0.943569	0.249627511	-0.162465654	8.99E-14	-0.975396578
ENSG00000134294	SLC38A2	94.314947	94.224312	105.017428	83.297339	91.717621	87.360116	44.39859	44.076549	55.144971	0.781743957	-0.027162049	1.02E-34	-0.97355899
ENSG00000128000	ZNF780B	1.258662	0.892611	0.942784	1.279073	1.129213	0.992562	0.348761	0.381766	0.645863	0.614842188	0.119255513	0.000000512	-0.973445682
ENSG00000164631	ZNF12	13.472945	14.145312	15.072231	12.129051	12.734863	11.817468	7.448372	5.94107	7.019478	0.407010393	-0.07316008	1.16E-30	-0.972620477
ENSG00000166454	ATMIN	11.950766	10.590083	11.281311	10.396798	11.001306	10.098841	5.140454	4.96346	6.147184	0.797804134	0.028336055	2.65E-29	-0.972406626
ENSG00000172403	SYNPO2	0.241208	0.305964	0.242013	0.080093	0.126324	0.107281	0.117992	0.112754	0.070064	0.000592348	-1.293131193	0.008978943	-0.972234294
NewGene_5737	--	1.216978	1.17726	1.625314	1.170838	1.098273	0.943084	0.483537	0.681287	0.738187	0.697527231	-0.177989222	0.012673555	-0.97146258
NewGene_6383	--	3.940079	4.05335	3.818495	3.427071	2.952209	2.912872	2.043138	1.828942	2.225228	0.086221313	-0.184610199	1.5E-21	-0.970569945
NewGene_979	--	0.676246	0.969811	0.958215	0.619354	0.717691	0.454845	0.408767	0.315723	0.507921	0.412938387	-0.384134175	0.021258548	-0.969114874
ENSG00000157193	LRP8	1.686296	1.919163	2.257141	2.779493	2.319741	2.421664	1.281779	0.936871	1.188296	0.000037	0.509334076	4.83E-11	-0.968178818
ENSG00000174306	ZHX3	5.28864	5.005359	4.096098	5.102114	6.460317	5.748173	2.518107	2.217166	3.010005	0.003251874	0.258090544	1.19E-26	-0.967487095
ENSG00000197134	ZNF257	0.44278	0.643323	0.601064	0.345929	0.271918	0.494645	0.195782	0.249969	0.372708	0.223594642	-0.482897222	0.010968223	-0.967314546
ENSG00000175344	CHRNA7	0.93983	1.067298	1.46469	0.866873	0.62438	0.933196	0.580651	0.804718	0.548874	0.2310193	-0.386439134	0.001382802	-0.967296118
NewGene_741	--	7.501208	12.532436	11.629426	4.670643	3.40276	3.024799	7.738491	5.09359	5.35181	1.06E-18	-1.281718178	5.91E-10	-0.967245549
ENSG00000234284	ZNF879	0.99967	1.352527	1.292426	1.186582	1.070194	1.270364	0.543495	0.420303	0.641892	0.973662659	0.012584519	0.000155817	-0.966714905
ENSG00000145794	MEGF10	0.373149	0.274084	0.367874	0.057876	0.091844	0.053756	0.134196	0.090492	0.262874	1.25E-09	-2.143117541	0.008756536	-0.965465094
ENSG00000170160	CCDC144A	0.293676	0.302775	0.190839	0.319265	0.215735	0.270191	0.194889	0.143945	0.109559	0.644051845	0.179479551	0.004097135	-0.965123824
ENSG00000175984	DENND2C	0.637049	0.617297	0.716857	0.756467	0.62452	0.604049	0.370578	0.343054	0.226659	0.558208888	0.153331758	0.000166982	-0.964950717
ENSG00000271425	NBPF10	0.912828	1.129398	1.464379	1.087366	0.829113	0.833033	0.636322	0.53768	0.507044	0.303344509	-0.203407196	2.02E-08	-0.964029996
ENSG00000164951	PDP1	1.208112	1.524547	1.543497	1.554488	1.557064	1.34193	0.73386	0.579213	0.662574	0.962563224	-0.014949857	0.00000297	-0.964026233
ENSG00000058673	ZC3H11A	21.431947	18.299907	17.804314	17.183737	32.121227	13.034359	18.780805	6.840197	4.378208	0.853136186	0.146620003	0.277700339	-0.962150772
ENSG00000130772	MED18	8.931895	8.283079	8.466191	5.820498	5.149198	5.724321	4.503352	3.936628	3.919042	0.0000553	-0.473493587	2.41E-14	-0.961384418
ENSG00000162702	ZNF281	25.304042	24.100318	27.106686	21.936456	20.172863	20.34341	12.25821	11.43327	12.308916	0.0252232	-0.147702816	4.1E-44	-0.9611139
ENSG00000182318	ZSCAN22	1.782731	1.825251	1.906404	1.645971	1.699234	1.664581	0.807309	0.72216	1.133977	0.967524609	0.009495492	8.13E-08	-0.959967419
NewGene_3414	--	1.811874	0.750027	2.451212	1.912221	1.093238	2.065886	0.795466	0.779575	0.947782	0.765058229	0.16144298	0.027653029	-0.95570041
ENSG00000162599	NFIA	2.57291	2.880647	3.078989	2.722921	2.824051	2.805047	2.034543	1.794872	2.189465	0.593750741	0.068272644	9.38E-18	-0.954741676
ENSG00000186260	MRTFB	5.296813	5.628831	5.941615	5.053398	4.657147	5.023709	3.0735	2.813349	2.803708	0.771319307	-0.032816834	3.52E-30	-0.954723098
ENSG00000144355	DLX1	0.541316	0.805071	0.805183	0.40267	0.179114	0.243854	0.177737	0.308311	0.437548	0.011573959	-1.088236534	0.024083438	-0.954506055
ENSG00000171448	ZBTB26	4.440351	4.455176	3.883078	3.328831	3.01615	3.012684	2.208319	2.014494	1.898987	0.012077527	-0.291388976	6.16E-15	-0.953944033
ENSG00000136881	BAAT	0.440346	0.536479	0.286817	0.214307	0.190271	0.158567	0.23568	0.140574	0.193395	0.062469102	-0.851389062	0.029581428	-0.95385274
ENSG00000109705	NKX3-2	0.665464	0.584162	0.404889	0.528082	0.523475	0.54877	0.276984	0.239972	0.275949	0.841894674	0.096329873	0.015715733	-0.953016549
ENSG00000061656	SPAG4	2.448938	2.226113	1.618387	1.202268	1.023314	1.363041	0.756309	0.931723	1.377109	0.026031442	-0.680667909	0.003895579	-0.951836007
ENSG00000256229	ZNF486	2.830098	3.051218	3.154635	2.420215	2.278761	2.37952	1.693398	1.831434	1.141649	0.095481733	-0.243623113	1.01E-08	-0.951306871
ENSG00000167378	IRGQ	4.890812	5.082734	5.203317	3.532268	3.640208	3.636184	2.524998	2.105068	2.740067	0.00000375	-0.342794922	2.42E-26	-0.951240178

Gene ID	Gene name	Ctrl-1 FPKM	Ctrl-2 FPKM	Ctrl-3 FPKM	IFNγ-1 FPKM	IFNγ-2 FPKM	IFNγ-3 FPKM	Sora-1 FPKM	Sora-2 FPKM	Sora-3 FPKM	Ctrl vs IFNγ DESeq2 FDR	Ctrl vs IFNγ DESeq2 log2FC	Ctrl vs Sora DESeq2 FDR	Ctrl vs Sora DESeq2 log2FC
NewGene 1238	--	3.880187	4.242288	5.487936	4.817886	3.626183	2.90446	1.699044	3.468259	2.834213	0.902346286	0.021695511	4.85E-17	-0.949458915
ENSG00000189410	SH2D5	3.986783	4.226719	4.680524	2.673139	1.62581	3.272797	1.540366	2.438044	2.659222	0.227378044	-0.845470834	2.27E-10	-0.948200854
NewGene 1926	--	0.810504	1.17602	1.349527	0.912346	0.749106	0.784895	0.551711	0.774341	0.592654	0.899142427	-0.034128971	0.000001	-0.947059843
ENSG00000175376	EIF1AD	12.752709	14.301942	15.005852	13.66858	13.390599	14.167791	8.086778	9.707129	8.102761	0.14049724	0.15068436	1.56E-20	-0.946195142
NewGene 1435	--	0.667229	0.596666	0.698575	0.514928	0.337552	0.537975	0.354319	0.343153	0.249347	0.44500273	-0.340223076	0.017116928	-0.944470923
ENSG00000127080	IPPK	3.682366	3.780431	4.32492	3.225557	2.467801	2.959182	1.746359	1.951733	2.072245	0.014116736	-0.322285056	6.64E-14	-0.943058168
ENSG00000180425	C11orf71	0.831633	1.129774	1.073277	1.099941	1.318239	1.140701	0.520343	0.598401	0.410473	0.224643755	0.3830811	0.006952145	-0.94281646
ENSG00000197961	ZNF121	6.387569	8.432758	8.446257	7.96412	7.461011	6.826367	3.985583	3.643808	4.520675	0.652062089	0.065138318	3.58E-16	-0.942663234
ENSG00000152454	ZNF256	6.923851	5.17584	6.450622	5.171446	5.380396	5.288392	3.034667	3.011225	3.036414	0.638351631	-0.085525916	1.48E-10	-0.941955117
ENSG00000173334	TRIB1	16.035649	16.276286	16.157331	14.188593	13.388603	13.936144	7.378845	7.252636	9.064838	0.342873019	-0.076148179	8.02E-29	-0.94146548
ENSG00000114209	PDCD10	43.146309	37.707376	40.312651	41.042507	37.512376	33.800029	18.78042	20.446642	22.281412	0.800613943	0.028388106	1.8E-30	-0.940283796
ENSG00000117461	PIK3R3	3.897737	3.673588	3.461076	5.395345	4.778184	4.377898	1.805184	1.948397	1.59656	3.95E-08	0.534959037	3.24E-14	-0.939442802
ENSG00000225190	PLEKHM1	3.356307	3.403999	2.392765	3.359347	2.922347	3.000922	1.679541	1.531387	1.751595	0.360775318	0.153743464	7.84E-10	-0.938605334
ENSG00000176749	CDK5R1	2.137367	2.05424	2.005599	1.379348	1.358563	1.286272	1.091181	0.925156	1.046107	0.00128256	-0.486538894	2.57E-09	-0.938574899
NewGene 183	--	0.467205	0.490638	0.593781	0.214566	0.396057	0.187612	0.271147	0.18745	0.286612	0.05783339	-0.936685647	0.043148695	-0.938084244
ENSG00000184635	ZNF93	6.261857	6.411155	7.379818	4.940723	4.502994	4.532689	3.468394	2.972384	3.12141	0.02201938	-0.281252603	6.35E-13	-0.936845327
ENSG00000177463	NR2C2	12.63163	13.213168	14.790043	12.94289	12.345476	11.931327	7.306381	6.662774	9.62751	0.637919123	0.047825099	2.35E-31	-0.935237388
NewGene 409	--	1.068805	1.588626	1.088603	0.949489	1.503035	1.452402	1.170818	0.656137	0.407009	0.889239124	0.039364125	0.0000984	-0.934603521
ENSG00000204178	MACO1	11.865211	11.211538	11.128244	11.293818	11.478825	11.350272	6.002211	5.206761	5.575147	0.076178887	0.143754535	1.67E-27	-0.934529466
ENSG00000276409	CCL14	0.731612	0.857813	0.673013	0.932969	0.786322	0.741358	0.508919	0.481568	0.31376	0.242951335	0.348634916	0.003681969	-0.934194438
ENSG00000135842	FAM129A	0.187354	0.13604	0.17461	0.134927	0.143006	0.240238	0.1563	0.08812	0.061385	0.949758019	0.037829717	0.039270775	-0.932395527
NewGene 2440	--	0.726644	0.737866	0.919067	0.877981	0.684589	0.801366	0.336383	0.395854	0.436482	0.679308924	0.139669857	0.001427202	-0.930367115
ENSG00000162695	SLC30A7	4.312865	4.592802	5.252031	6.43855	6.345037	6.262688	2.60568	2.770934	2.235014	9.7E-12	0.560128346	7.53E-18	-0.930334527
ENSG00000159173	TNNI1	1.144425	1.231155	0.936832	0.654557	0.641261	0.748844	0.573085	0.475858	0.579804	0.004123107	-0.546000145	0.00000101	-0.930320666
ENSG00000158470	B4GALT5	9.66365	9.630581	10.468478	9.171322	8.989336	8.946802	4.804042	4.634714	5.224098	0.912650612	0.012664886	1.54E-29	-0.929877564
ENSG00000089335	ZNF302	13.73712	14.738024	14.164567	14.470401	14.956722	12.997965	6.395878	6.759137	7.829345	0.283147828	0.10356735	3.73E-25	-0.928572912
ENSG00000130283	GDF1	1.573928	2.843557	0.920594	0.343394	0.649498	1.142424	0.952662	0.603033	1.073625	0.154294455	-1.159921876	0.008109404	-0.928238342
ENSG00000197020	ZNF100	1.947141	3.242661	3.241204	2.548278	2.037915	2.290793	1.493522	1.285919	1.40977	0.510124624	-0.141182129	0.000000162	-0.928230731
ENSG00000213654	GPSM3	1.403157	0.962883	0.897025	0.828497	0.642919	0.825895	0.571703	0.658489	0.388821	0.435418409	-0.339254801	0.021489457	-0.928068502
ENSG00000134240	HMGCS2	1.009619	0.799849	0.395255	0.56909	0.375325	0.531858	0.525001	0.271697	0.279689	0.357335903	-0.427345114	0.036878062	-0.927906476
NewGene 500	--	2.621955	2.519966	2.420535	2.364502	2.022707	1.645039	1.632447	1.057695	1.301181	0.349391701	-0.204184419	0.000000159	-0.92783001
ENSG00000120784	ZFP30	1.812941	2.102806	2.046268	1.937404	1.582052	2.086761	1.082212	0.914744	0.76406	0.960644742	0.010819005	2.22E-08	-0.925495929
ENSG00000259330	INAFM2	3.330538	3.029012	2.653632	3.090262	2.988996	3.449661	1.54665	1.367287	1.548106	0.165944892	0.228070255	1.23E-08	-0.924150538
ENSG00000278318	ZNF229	3.662197	4.351897	4.132795	3.523665	3.501367	3.391331	1.84503	2.044589	2.09566	0.572990751	-0.076464256	2.24E-15	-0.924104099
ENSG00000040199	PHLPP2	1.983623	1.962968	2.242291	2.296086	1.621879	1.603036	1.283372	0.929236	1.440927	0.226860712	-0.176338565	0.000000567	-0.92370244
ENSG00000176928	GCNT4	0.753676	0.843044	0.954673	0.598276	0.756328	0.63474	0.330038	0.512909	0.406916	0.412604629	-0.207630262	0.00010931	-0.923480807
NewGene 860	--	2.058017	2.45422	2.326759	2.002738	1.746834	1.38987	1.464637	0.96504	0.970806	0.208417716	-0.265073219	0.00000849	-0.923185415
ENSG00000176597	B3GNT5	8.932186	9.85601	9.266987	9.813042	8.998519	9.501463	4.353588	4.162042	5.424812	0.059514694	0.161840093	4.38E-21	-0.922199456
NewGene 1782	--	1.484148	1.669962	1.145348	1.038237	0.769394	0.755054	0.700525	0.720154	0.715443	0.008562563	-0.656014545	0.000129045	-0.921816506
ENSG00000131016	AKAP12	11.875794	9.392258	8.828015	3.227314	2.826597	2.800066	4.933196	4.530955	5.614239	2.7E-60	-1.615776777	8.16E-19	-0.921050429
ENSG00000139718	SETD1B	4.164097	4.036555	4.158159	5.031652	4.2366	4.400105	2.184488	1.887158	2.256379	0.000590858	0.295723939	1.41E-22	-0.920724101
ENSG00000174516	PELI3	5.115001	4.442398	3.352778	3.87146	3.838026	4.074411	2.23363	1.815102	2.248916	0.857097384	0.044459587	0.000000555	-0.920567339
ENSG00000163513	TGFBR2	23.190681	20.906524	21.185059	21.681253	21.173881	19.913297	10.920219	9.951633	11.540481	0.253199201	0.090404308	2.11E-37	-0.919919978
ENSG00000196172	ZNF681	0.841283	1.063081	1.00765	0.776388	0.706125	0.743517	0.504208	0.406267	0.532925	0.192420067	-0.264074969	0.00000263	-0.919863106
NewGene 4094	--	1.371235	1.610197	1.380803	1.747887	1.385034	1.456071	0.681638	0.639114	0.788942	0.397929519	0.180912838	0.00000225	-0.919597619
ENSG00000130856	ZNF236	2.752372	2.630909	2.412122	2.161833	2.371988	2.293967	1.429841	1.272113	1.388053	0.727823582	-0.047989408	4.63E-16	-0.916215632
ENSG00000112486	CCR6	1.54718	1.478109	1.334101	1.068935	0.975584	0.959984	0.603129	0.759064	0.797559	0.047601148	-0.388637085	0.00000492	-0.915946375
ENSG00000131620	ANO1	1.435971	0.846726	0.704434	0.444042	0.568172	0.365897	0.628234	0.426293	0.312313	0.003058307	-0.923225992	0.005194683	-0.915846431
ENSG00000134853	PDGFRA	4.808338	4.56935	4.911379	1.697943	1.764375	1.668777	2.062878	2.452017	2.924796	1.21E-44	-1.306542873	3.1E-16	-0.91575167
ENSG00000146676	PURB	12.126243	13.494855	12.949424	11.683336	11.6637	11.891696	6.647574	5.781924	6.784853	0.849500343	0.017592621	2.08E-37	-0.914318536
ENSG00000277161	PIGW	13.707712	15.447154	16.154839	13.307961	12.107718	13.850652	6.913085	7.013382	8.618667	0.598604664	-0.064382752	6.1E-18	-0.913462611
ENSG00000162849	KIF26B	0.409954	0.3261	0.335113	0.34777	0.312478	0.319789	0.208462	0.13543	0.253011	0.787305016	0.08382962	0.000701554	-0.912720667
ENSG00000077063	CTTNBP2	0.90307	0.752922	0.744095	0.827475	0.59616	0.68425	0.445713	0.388074	0.341337	0.999202724	-0.000329967	0.0000313	-0.911333644
ENSG00000123505	AMD1	78.287061	71.573021	79.209448	62.026014	66.854429	63.482612	32.093679	40.209621	46.315034	0.557019239	-0.048646766	8.72E-30	-0.910945422
ENSG00000115507	OTX1	4.562862	3.738913	4.417656	4.629312	4.552502	4.919267	1.899231	2.117735	2.162304	0.024363089	0.304908728	6.69E-10	-0.910719996
ENSG00000179941	BBS10	4.460087	5.418011	5.092089	4.034997	4.142946	3.785185	2.408871	2.382186	2.658015	0.179712827	-0.177496016	1.12E-13	-0.909692589
NewGene 1208	--	1.287493	1.037847	1.11809	0.600892	0.662599	0.771089	0.452481	0.534865	0.919758	0.080347182	-0.531657989	0.011972773	-0.909523575
ENSG00000173559	NABP1	6.721378	6.876703	7.007856	5.473797	5.495162	4.595518	4.392463	4.060756	4.273273	0.000863831	-0.296872871	1.26E-27	-0.908545576
ENSG00000243943	ZNF512	0.666928	0.815657	1.054405	0.457239	0.516302	0.399273	0.398976	0.449754	0.410671	0.00928778	-0.753570314	0.001381272	-0.908423224
ENSG00000122257	RBBP6	17.707943	17.585089	17.162869	20.594419	18.814195	19.193083	7.715062	7.661617	9.479536	8.48E-08	0.294828621	2.41E-28	-0.908396953
ENSG00000179409	GEMIN4	17.754126	18.363333	18.385352	15.306702	14.720147	15.868147	8.543862	8.31337	10.32583	0.267734255	-0.086744218	7.72E-32	-0.908131728
ENSG00000144120	TMEM177	12.376814	13.019957	12.413358	10.549825	11.36383	13.313555	6.295825	6.610173	6.32551	0.792303931	0.043665893	6.71E-14	-0.907986284
ENSG00000117036	ETV3	5.575978	6.08817	5.911399	4.99737	5.270232	5.182437	2.915368	3.15791	3.180191	0.822475674	-0.028564049	1.48E-21	-0.907673336
ENSG00000132669	RIN2	0.416635	0.431294	0.621369	0.668592	0.510793	0.335462	0.263884	0.270571	0.28126	0.890289578	-0.062787743	0.00701316	-0.906660445
ENSG00000161298	ZNF382	1.012975	1.308372	1.103787	1.130052	0.89829	0.902769	0.54296	0.497143	0.601173	0.342421836	-0.221394389	0.0000222	-0.906360939
ENSG00000144802	NFKBIZ	11.515204	12.504651	13.064677	11.716937	11.913229	11.363791	5.860391	5.681298	7.459266	0.560618879	0.058231364	6.67E-19	-0.906212493
ENSG00000198331	HYLS1	2.20268	2.443819	2.243737	1.788735	1.89444	1.591226	1.268782	0.977545	0.922632	0.634992113	-0.13097196	0.000145841	-0.903892335
ENSG00000204920	ZNF155	5.372377	5.987196	5.966124	5.171243	4.833418	4.706344	3.039386	2.773302	3.293656	0.51448991	-0.094699602	1.25E-12	-0.903665019
ENSG00000135870	RC3H1	3.217173	3.758502	4.691631	3.579622	3.459406	3.457633	1.860591	1.706293	1.917221	0.344413819	0.103445518	7.61E-20	-0.903370491
ENSG00000138639	ARHGAP24	0.715208	1.562914	0.945712	0.599241	0.776098	1.02895	0.752334	0.405509	0.442584	0.802747044	-0.084968525	0.000715393	-0.902910703
ENSG00000168916	ZNF608	3.614294	3.512452	3.225945	3.756023	3.25582	3.534833	1.514663	1.595262	1.805648	0.244973934	0.146657424	5.47E-15	-0.90168807
ENSG00000188786	MTF1	0.888749	0.996623	0.848274	1.038957	1.053164	0.955693	0.541845	0.400903	0.42837	0.063201822	0.302601566	0.00000115	-0.900424259
ENSG00000177076	ACER2	0.986189	1.070299	1.244808	0.729039	0.712048	0.658994	0.500012	0.541412	0.59697	0.106736293	-0.499944862	0.002617072	-0.900086651
NewGene 1061	--	1.844257	1.799969	2.151182	1.513777	1.570816	1.37883	0.892363	0.926721	1.096697	0.22592017	-0.231476535	0.000000474	-0.899344958

Gene ID	Gene name	Ctrl-1 FPKM	Ctrl-2 FPKM	Ctrl-3 FPKM	IFNγ-1 FPKM	IFNγ-2 FPKM	IFNγ-3 FPKM	Sora-1 FPKM	Sora-2 FPKM	Sora-3 FPKM	Ctrl vs IFNγ DESeq2 FDR	Ctrl vs IFNγ DESeq2 log2FC	Ctrl vs Sora DESeq2 FDR	Ctrl vs Sora DESeq2 log2FC
NewGene 2601	--	0.671241	0.476586	0.525927	0.554133	0.558869	0.657428	0.19581	0.403703	0.382373	0.853962939	0.101193251	0.055711796	-0.898760904
ENSG00000124766	SOX4	60.609951	59.023968	59.790012	43.798004	45.139915	43.271988	28.76016	29.66873	31.865547	2.34E-09	-0.293022622	1.45E-58	-0.898441385
ENSG00000164684	ZNF704	1.468647	1.550967	1.469368	0.956973	0.956902	0.922285	0.956308	0.707853	0.877431	0.0000007	-0.51428613	2.83E-12	-0.896736078
ENSG00000112242	E2F3	31.435524	33.184356	34.388772	29.199016	26.323193	28.226906	15.73023	15.393573	18.782353	0.188118324	-0.09146696	1.61E-38	-0.896028197
ENSG00000167565	SERTAD3	6.031062	6.252898	5.714219	6.135391	5.793844	7.101794	2.926594	3.230891	2.706871	0.164066136	0.216702225	9.28E-09	-0.893971424
NewGene 5096	--	0.87553	1.006256	0.897793	1.11983	0.822187	1.115301	0.542952	0.523599	0.332869	0.38430961	0.281906663	0.007762295	-0.89327095
ENSG00000170468	RIOX1	5.285017	4.162127	5.620299	4.708469	5.068306	5.241839	2.454797	2.506249	2.639518	0.405089374	0.143291742	5.26E-09	-0.893181286
ENSG00000283154	IQCJ-SCHIP1	0.798065	0.562264	0.731038	0.37625	0.400405	0.427706	0.136236	0.172579	0.6848	0.194258371	-0.521514562	0.084339395	-0.89296352
ENSG00000187775	DNAH17	0.213372	0.184032	0.194748	0.168581	0.17624	0.182589	0.123638	0.077879	0.098191	0.964192542	-0.017743577	0.002381399	-0.892036784
ENSG00000167447	SMG8	14.798252	15.060441	14.654982	11.837858	12.431465	12.935569	8.071626	7.724483	6.51934	0.23424226	-0.103043696	5.82E-19	-0.891966816
ENSG00000125848	FLRT3	11.87681	13.268733	13.156474	9.17637	9.722828	10.106681	5.605615	6.283823	7.685104	0.005545155	-0.230196287	1.52E-26	-0.891958348
ENSG00000065308	TRAM2	6.771248	6.17875	6.562132	5.722854	5.676248	5.935089	3.377591	2.924538	3.589609	0.837948229	-0.023107658	5.49E-24	-0.890167943
ENSG00000117020	AKT3	0.66434	0.500829	0.387107	0.29823	0.290757	0.353118	0.239617	0.251301	0.29622	0.010265707	-0.74139963	0.001746631	-0.887688388
ENSG00000166801	FAM111A	7.197106	7.296587	8.584777	12.199574	10.673334	10.771827	3.886394	3.946883	4.196793	1.67E-17	0.738768128	7.53E-18	-0.887656466
ENSG00000130749	ZC3H4	7.734557	7.644614	7.026129	7.517795	7.18004	7.701841	3.957581	3.301904	4.038717	0.099645686	0.1374386	2.93E-23	-0.886238311
ENSG00000174500	GCSAM	0.442055	0.604436	0.639809	0.462893	0.569136	0.394085	0.259628	0.279472	0.33164	0.732306435	-0.148587669	0.011586013	-0.884596444
ENSG00000152192	POU4F1	2.41379	2.246414	2.740551	1.849104	1.525695	1.994672	1.180404	1.189531	1.388212	0.05187456	-0.314574502	2.63E-09	-0.88413284
ENSG00000133640	LRRIQ1	0.340673	0.27414	0.377724	0.132782	0.055256	0.054653	0.206526	0.143499	0.171171	0.00010365	-1.801076274	0.021180193	-0.884125107
ENSG00000121406	ZNF549	2.658721	2.689317	2.802186	2.142626	2.166189	1.838725	1.594426	1.238488	1.288495	0.104719574	-0.246689065	2.89E-09	-0.883800965
ENSG00000203326	ZNF525	1.805708	2.007205	1.744783	1.424676	1.266779	1.722501	1.313328	0.919216	0.852408	0.00561902	-0.492486661	0.0000207	-0.883773934
ENSG00000198315	ZKSCAN8	11.936824	13.135493	14.75715	13.238686	12.782167	12.148863	7.136892	6.505896	6.786491	0.321193258	0.092336271	3.61E-25	-0.883570866
ENSG00000169981	ZNF35	4.993626	4.683741	5.100555	4.85058	4.43797	4.320227	2.522157	2.611506	2.36494	0.606710225	-0.08774333	6.38E-11	-0.883339143
NewGene 3369	--	2.760216	2.540248	2.523755	1.847657	1.992149	1.633329	1.485066	1.105085	1.269282	0.013150953	-0.368754862	0.00000002	-0.880584292
ENSG00000116273	PHF13	11.524044	9.880512	10.307598	8.566285	8.812626	8.459473	5.650844	4.973365	5.544852	0.131961316	-0.148992599	8.77E-21	-0.880495815
ENSG00000223496	EXOSC6	5.223512	5.407946	5.334444	4.482458	3.909888	3.99696	2.612334	2.428379	3.110706	0.025211634	-0.219719331	8.24E-18	-0.880269596
ENSG00000204604	ZNF468	10.142358	6.716777	6.349039	5.093684	5.380774	5.448034	4.949192	4.066625	4.389555	0.246143112	-0.49542962	0.019623602	-0.879985465
NewGene 1562	--	0.679695	0.637089	0.613309	0.99394	0.965479	1.227376	0.33224	0.440837	0.242525	8.18E-08	0.837536175	0.0000476	-0.879886475
ENSG00000178917	ZNF852	2.435675	2.145052	1.546569	2.30551	2.417683	1.837817	1.033636	1.2257	0.987277	0.295612068	0.291079164	0.00353252	-0.879544817
ENSG00000131323	TRAF3	7.290065	7.0847	6.200136	4.125395	5.804018	5.659717	4.171857	3.79834	3.751932	0.021079369	-0.213480703	2.19E-19	-0.879404318
ENSG00000145241	CENPC	4.371718	4.608663	4.701506	4.719845	4.576059	4.324916	2.433891	2.413969	2.952466	0.491504028	0.076161284	2.62E-20	-0.879235409
ENSG00000088179	PTPN4	2.228639	2.558456	2.737522	2.092031	2.147033	2.033689	1.709421	1.531367	1.48736	0.226677788	-0.144723247	2.45E-14	-0.878579453
ENSG00000232040	ZBED9	1.830524	1.673607	1.631331	1.27372	1.653584	1.565418	0.735789	0.851299	0.944013	0.900757507	-0.030536779	0.000000142	-0.878304617
ENSG00000204514	ZNF814	3.23504	4.280602	3.879563	3.507119	3.633809	3.099111	2.38953	1.95901	2.500203	0.856483342	0.041050845	0.0000002	-0.878275313
ENSG00000182372	CLN8	4.982263	7.838052	4.965488	2.490968	2.550092	2.374709	2.829668	2.488661	2.457529	6.43E-32	-0.983886305	7.17E-22	-0.877773592
ENSG00000140104	CLBA1	1.9287	2.316488	1.534972	1.140215	1.026992	1.106237	1.335595	0.871123	0.74426	0.006323594	-0.630458649	0.001266605	-0.877258905
ENSG00000186834	HEXIM1	20.923946	19.998156	22.161507	13.321015	13.647941	12.955722	10.253982	10.88271	11.070589	3.76E-14	-0.512364349	6.25E-31	-0.876414518
ENSG00000187391	MAGI2	0.648703	0.749843	0.732626	0.365421	0.509539	0.509448	0.355806	0.293616	0.413504	0.058014834	-0.442390293	0.0000665	-0.875514028
NewGene 1861	--	1.558169	1.633068	1.740598	0.928941	1.095217	0.809105	1.168727	0.717843	0.985013	0.000573516	-0.790030899	0.000158782	-0.874257734
ENSG00000072422	RHOBTB1	7.793479	7.502122	7.342772	5.360505	5.178349	5.370026	3.86918	3.79146	3.918624	0.0000115	-0.363229072	4.81E-23	-0.874195105
ENSG00000081189	MEF2C	2.584709	2.741538	3.023844	5.642318	4.594344	4.85661	1.903687	1.660894	1.465014	2E-22	0.987309874	6.96E-10	-0.873769461
ENSG00000006432	MAP3K9	2.052635	1.484988	1.603588	1.207831	1.498499	1.155542	0.789542	0.947051	0.871769	0.000139018	-0.476366391	1.44E-10	-0.87333357
ENSG00000119699	TGFB3	0.83432	0.735248	0.902409	0.410918	0.426459	0.614766	0.436855	0.49116	0.399339	0.070539775	-0.64071659	0.009034518	-0.872480339
ENSG00000275111	ZNF2	1.359516	1.751858	1.685627	1.403346	1.556914	1.381418	1.061866	0.697958	0.747769	0.662087393	-0.106689276	0.0000391	-0.87209608
ENSG00000153395	LPCAT1	10.403409	9.720061	9.690153	8.164915	6.961742	7.953166	4.689066	4.925716	5.702541	0.015662572	-0.221234228	2.43E-21	-0.870182268
NewGene 5023	--	1.38585	1.595805	1.866179	1.491398	1.081532	1.139067	0.868708	0.741923	0.963441	0.227317022	-0.228538806	9.11E-08	-0.870024365
ENSG00000135632	SMYD5	19.971423	20.556403	19.768265	15.746863	12.943736	14.190542	9.966029	10.326827	11.547939	0.0000171	-0.352369862	6.71E-27	-0.869894835
ENSG00000177613	CSTF2T	12.01016	12.190774	11.95915	12.621892	11.02605	12.007827	6.437402	5.921442	6.19923	0.126137463	0.126347008	8.96E-29	-0.869871407
ENSG00000177932	ZNF354C	2.439441	2.677363	2.698257	2.48278	2.519455	2.372969	1.256457	1.356204	1.393345	0.684290694	0.06374922	1.57E-11	-0.86909571
NewGene 872	--	0.626261	0.934889	0.756488	0.607907	0.49159	0.537151	0.342377	0.346024	0.502987	0.164472651	-0.351747907	0.000444428	-0.868959931
NewGene 4260	--	2.215412	2.634759	3.0201	1.801269	1.471496	2.000275	1.413062	1.426174	1.53695	0.062469102	-0.351531457	0.00000108	-0.86883504
ENSG00000154734	ADAMTS1	1.531056	1.554532	1.734107	0.99436	0.947165	0.856346	0.806564	0.720462	0.943412	0.000102292	-0.628310648	0.000000286	-0.868135514
ENSG00000224420	ADM5	3.507944	3.47679	3.712252	2.180002	1.386059	2.460972	1.98177	1.776298	1.726608	0.002338672	-0.674908742	0.0000036	-0.867683904
ENSG00000162757	C1orf74	1.477657	1.588651	1.576382	1.143021	1.24343	1.106307	0.86101	0.759556	0.762476	0.141339079	-0.266240508	0.00000051	-0.867383358
ENSG00000112419	PHACTR2	4.890416	4.675684	5.658027	4.365224	4.251865	4.209145	3.043011	2.456119	2.936137	0.14885841	-0.151077453	1.1E-18	-0.866128663
ENSG00000145626	UGT3A1	0.743271	0.419805	0.377729	0.080564	0.080495	0.088252	0.283287	0.206826	0.293257	1.13E-09	-2.28473844	0.011375565	-0.864073281
ENSG00000063127	SLC6A16	3.11693	3.564077	4.472175	2.775413	2.680309	3.466802	1.831095	2.151999	2.717796	0.522516291	-0.142041738	0.0000031	-0.863531057
NewGene 6320	--	2.43755	1.777447	2.183963	1.646885	1.991024	1.399215	1.84981	1.408954	1.519489	0.444541781	-0.131522	2.74E-08	-0.863260714
ENSG00000151093	OXSM	9.185471	9.234411	8.107454	7.399087	6.599134	7.151976	4.640479	4.796076	4.370476	0.350202099	-0.141831785	4.09E-10	-0.863183727
NewGene 4047	--	2.801897	3.117285	3.259055	2.92703	3.045029	2.652392	1.796656	1.238023	1.396614	0.946121012	0.014542757	4.18E-09	-0.862739196
ENSG00000146858	ZC3HAV1L	4.433828	4.180322	3.972717	4.478105	3.981885	4.202252	2.294812	2.177204	1.993047	0.396475132	0.15498039	0.000000738	-0.862540515
ENSG00000198824	CHAMP1	18.697331	18.404288	19.049911	18.635131	19.017851	18.819736	9.394253	8.630438	10.712233	0.036393783	0.142054417	4.52E-29	-0.860231573
ENSG00000128191	DGCR8	13.526332	13.615884	14.093588	13.182339	12.06136	11.77888	6.61652	6.578112	8.068528	0.897008801	-0.013702184	2.44E-25	-0.859663561
NewGene 1155	--	1.227099	0.954129	0.98967	0.993442	0.462784	1.159191	0.498526	0.541281	0.667701	0.686674823	-0.224909902	0.035972895	-0.858766519
ENSG00000186666	BCDIN3D	1.04656	1.341521	1.150567	0.98356	0.952664	1.163551	0.622905	0.699268	0.494275	0.918672101	-0.034964079	0.000731172	-0.858761095
ENSG00000135835	KIAA1614	0.367137	0.327011	0.349464	0.225354	0.225205	0.246986	0.181941	0.155691	0.17872	0.192622922	-0.36493796	0.001123782	-0.858417787
NewGene 1227	--	3.691215	4.423433	5.382784	5.621441	3.178015	6.624613	5.634208	1.038593	4.619622	0.467575269	0.093866411	0.1317182	-0.857767399
ENSG00000183054	RGPD6	2.316921	2.540495	2.648716	1.922714	2.065058	1.98184	1.30902	1.361198	1.227452	0.095032188	-0.20615982	4.5E-13	-0.857551669
ENSG00000137040	RANBP6	10.220058	10.117784	9.420776	9.831872	10.310579	10.160053	5.205906	4.965027	5.113641	0.023863979	0.18202765	2.17E-25	-0.857244998
ENSG00000173041	ZNF680	4.055085	4.82382	5.865996	4.038394	3.239409	4.153036	2.492134	2.603379	2.786736	0.202998735	-0.223240371	1.28E-08	-0.857230593
ENSG00000256294	ZNF225	1.337566	1.507267	1.71277	1.364431	1.588243	1.305838	0.952568	0.70365	0.776058	0.542871668	0.135360512	0.0000244	-0.856996394
ENSG00000169871	TRIM56	4.701094	4.765326	4.77774	9.047637	8.171014	8.438546	2.379458	2.231907	2.804014	1.97E-57	0.929500251	1.6E-23	-0.856340013
ENSG00000167637	ZNF283	1.367906	1.7678	1.804399	1.647532	1.620896	1.69783	1.129978	0.843769	0.683962	0.514261619	0.139031542	0.0000629	-0.856095152
ENSG00000089091	DZANK1	0.651637	0.398967	0.333788	0.456633	0.298633	0.28311	0.152004	0.265482	0.265804	0.638351631	-0.227736542	0.036522863	-0.854303147
ENSG00000180855	ZNF443	5.060364	5.686088	5.003409	3.719403	3.256303	3.937362	2.07748	2.897173	3.168345	0.004646678	-0.377972505	4.84E-08	-0.854096083

Gene ID	Gene name	Ctrl-1 FPKM	Ctrl-2 FPKM	Ctrl-3 FPKM	IFNγ-1 FPKM	IFNγ-2 FPKM	IFNγ-3 FPKM	Sora-1 FPKM	Sora-2 FPKM	Sora-3 FPKM	Ctrl vs IFNγ DESeq2 FDR	Ctrl vs IFNγ DESeq2 log2FC	Ctrl vs Sora DESeq2 FDR	Ctrl vs Sora DESeq2 log2FC
ENSG00000178295	GEN1	4.210167	4.967121	5.358881	5.329534	4.330574	4.552332	2.793024	2.556158	2.790508	0.876616011	0.026364739	7.17E-17	-0.853905914
ENSG00000168772	CXXC4	0.537055	0.640322	0.496395	0.492574	0.479838	0.385012	0.29992	0.302059	0.262769	0.618821056	-0.161985447	0.001573535	-0.852538408
ENSG00000189431	RASSF10	0.963233	0.734918	0.763934	0.372531	0.395647	0.544953	0.398046	0.420806	0.46457	0.031371246	-0.752074204	0.009157498	-0.851403072
ENSG00000159885	ZNF222	2.906253	2.93852	3.497782	2.439742	2.375069	2.555078	1.814485	1.545691	1.353652	0.446866766	-0.18222212	0.000117732	-0.851180067
ENSG00000146966	DENND2A	0.487635	0.293716	0.337702	0.200223	0.132261	0.136063	0.214885	0.159572	0.212204	0.012814303	-1.101055817	0.040083738	-0.850868121
ENSG00000151835	SACS	0.70053	0.772981	0.827859	0.855129	0.720727	0.807003	0.416509	0.383145	0.409449	0.213150257	0.182220127	1.2E-09	-0.85070915
NewGene 487	--	4.04277	6.195567	5.018226	5.280996	5.325803	4.973003	2.367017	1.958502	3.508856	0.425057561	0.332936258	0.122248691	-0.850117552
ENSG00000215271	HOMEZ	4.918216	4.68122	4.658367	4.317148	4.58819	4.315879	2.505282	2.383806	2.525679	0.774468642	0.038361696	3.43E-17	-0.849223814
ENSG00000143384	MCL1	89.20712	90.590305	90.835413	105.618956	105.75083	100.372862	46.001849	43.218726	51.97885	3.74E-14	0.349377356	4.4E-45	-0.848541191
NewGene 731	--	0.913722	1.183101	1.142935	0.960042	0.560967	1.18864	0.540059	0.40701	0.63481	0.894203329	-0.061441111	0.010441087	-0.848185309
ENSG00000106993	CDC37L1	3.658478	3.517441	4.054514	3.053317	3.365598	3.286819	2.330062	1.666699	1.937587	0.807410252	-0.044813577	9.72E-09	-0.847920633
NewGene 3632	--	0.607149	0.576486	0.809652	0.54237	0.699925	0.654511	0.52823	0.340187	0.36809	0.936585886	-0.033844903	0.005663137	-0.847270118
ENSG00000235194	PPP1R3E	1.846664	1.803791	1.953759	1.889044	1.911193	1.743819	0.93693	0.83783	0.702699	0.014260247	0.435265513	0.0000534	-0.845619549
ENSG00000099769	IGFALS	1.82224	1.476594	0.927584	1.206361	1.35091	1.093773	0.755274	0.53857	0.868731	0.946098761	-0.029704441	0.007932325	-0.845188755
NewGene 4779	--	1.625414	1.758757	1.792267	1.391978	1.947003	1.591812	1.327676	1.129305	0.674839	0.900797649	0.042481439	0.004932099	-0.844560173
NewGene 553	--	0.880517	1.93524	1.507734	1.687283	1.859871	1.65949	0.630072	0.583223	1.311254	0.078052823	0.510418273	0.009838883	-0.844109652
ENSG00000182993	C12orf60	0.917967	0.891832	0.889389	0.587023	0.845113	0.542602	0.317566	0.514299	0.545998	0.489774016	-0.30744321	0.031516117	-0.842692398
ENSG00000047457	CP	24.894086	19.777693	15.183693	23.151797	22.611391	22.416512	9.764718	10.549764	10.947075	0.190181597	0.328357263	0.000122565	-0.842055854
NewGene 4058	--	0.659811	0.618107	0.691698	0.691416	0.610049	0.586863	0.503179	0.314318	0.286754	0.819470504	0.090510052	0.012151067	-0.841353056
ENSG00000102780	DGKH	1.76263	2.000886	2.327932	1.357449	1.196555	1.286774	1.398843	1.271385	1.50654	0.00030749	-0.488615835	1.84E-09	-0.84124873
ENSG00000266086	AC015813.2	3.427029	4.482147	3.51808	5.835819	3.686898	4.050154	1.972924	2.333379	1.647865	0.043502427	0.390287511	0.0000116	-0.840757439
ENSG00000113205	PCDHB3	0.306773	0.375016	0.298719	0.161057	0.137019	0.157869	0.205474	0.175927	0.119349	0.047697346	-0.93767933	0.070719156	-0.839740063
ENSG00000151474	FRMD4A	0.781883	0.751763	0.77758	0.429757	0.346299	0.282902	0.427593	0.532048	0.409708	0.004906894	-0.667823842	0.000276563	-0.839450712
ENSG00000146592	CREB5	1.926722	2.260204	2.671569	0.460256	0.438308	0.444503	1.182579	1.422246	1.383693	1.26E-46	-2.088021063	1.15E-09	-0.83883658
ENSG00000141404	GNAL	0.648824	0.446747	0.462157	0.715491	0.606875	0.600531	0.227077	0.302131	0.217137	0.00509915	0.638492303	0.003652803	-0.837825651
ENSG00000157617	C2CD2	1.777593	1.789919	1.724104	2.60247	2.488801	2.480747	0.95011	0.826313	0.98815	1.35E-08	0.620591832	2.32E-09	-0.83782038
ENSG00000256806	C17orf100	2.390811	1.952433	2.344619	2.544812	3.25341	2.587091	0.883732	0.978314	1.664807	0.030487197	0.469545305	0.001958686	-0.836985976
ENSG00000139291	TMEM19	6.146376	6.494758	5.939003	4.268194	4.367301	4.599201	3.222841	3.042562	3.551312	0.0000428	-0.345185976	1.58E-20	-0.836600824
ENSG00000183060	LYSMD4	15.26075	14.042098	13.335925	12.353044	13.082419	11.569442	6.709985	7.192412	8.469412	0.572887633	-0.063935935	4.97E-19	-0.836192728
NewGene 5597	--	0.486695	0.581312	0.705736	0.832219	0.539895	0.529902	0.379684	0.261021	0.464067	0.344410299	0.295651015	0.003923599	-0.835966123
ENSG00000144026	ZNF514	3.586879	4.148752	3.984285	4.028338	3.124015	3.318743	1.914231	1.845433	2.681845	0.964783422	-0.010762357	0.00000045	-0.835960257
ENSG00000030419	IKZF2	0.694482	1.053396	0.851826	0.396382	0.559389	0.625304	0.68894	0.293992	0.444643	0.02463243	-0.442818061	0.0000831	-0.835569809
ENSG00000285641	AL358472.6	1.211713	0.890458	1.174594	0.870939	0.569122	0.775542	0.727242	0.385671	0.61314	0.288015339	-0.411208914	0.022939184	-0.835435499
NewGene 5921	--	1.226469	2.066386	1.16611	1.222345	1.124723	0.95542	0.699047	1.05864	0.486684	0.987497051	0.00561262	0.002715471	-0.835152915
ENSG00000117616	RSRP1	5.718702	6.237791	7.412253	7.285078	7.30487	6.217922	3.236912	3.12327	4.281157	0.704931167	0.06596771	1.36E-08	-0.834637166
ENSG00000137460	FHDC1	5.457647	4.890145	5.523429	3.943395	4.277342	3.750732	2.586679	2.638896	3.117082	0.009279572	-0.25679283	1.28E-17	-0.834100559
ENSG00000163501	IHH	1.944443	1.732348	1.337721	2.287395	2.00819	2.373492	0.699437	0.983885	0.948241	0.013476178	0.555484242	0.002383367	-0.833922141
ENSG00000092200	RPGRIP1	1.085313	1.605058	1.225407	1.73171	1.109631	1.135336	0.408441	0.423917	0.963855	0.472564888	0.195206449	0.001136999	-0.832615448
NewGene 3353	--	1.802236	0.859518	1.42109	0.610649	0.552012	0.4582	0.465476	0.497688	0.705358	--	--	0.073158264	-0.831110341
ENSG00000198535	C2CD4A	0.308189	0.364506	0.286374	0.225794	0.192615	0.256804	0.143286	0.191954	0.16664	0.495889583	-0.346329538	0.065784225	-0.831098196
NewGene 3315	--	1.240581	1.106733	1.239435	1.110348	1.188203	1.04179	0.720256	0.493404	0.743943	0.946098761	0.022056859	0.00039531	-0.830092549
ENSG00000139910	NOVA1	3.37731	4.242161	3.60082	2.975095	3.3868	1.972721	3.134462	2.029236	2.749854	0.010756458	-0.373417846	2.66E-09	-0.829918745
NewGene 5436	--	2.435714	2.377904	2.529346	1.022158	1.551667	1.176855	1.767927	1.759112	1.63028	0.002735382	-0.671493954	0.0000435	-0.82942363
ENSG00000264058	AC073508.2	0.340558	0.293886	0.175696	0.293252	0.333097	0.249163	0.180672	0.090653	0.200294	0.820852658	0.119037341	0.071012783	-0.829185114
ENSG00000185085	INTS5	10.335437	10.209612	9.903202	9.391943	9.121379	9.660455	4.573812	5.377956	6.13856	0.751964832	0.035943851	3.48E-15	-0.828418618
ENSG00000175305	CCNE2	6.643496	7.75578	7.586621	7.717366	9.551813	8.236735	3.62793	3.57366	4.191186	0.400021915	0.137373798	7.49E-14	-0.82803178
ENSG00000063587	ZNF275	2.487501	2.061294	2.15927	2.143363	1.758078	1.807213	1.218127	0.95621	1.364191	0.626581457	-0.083381381	9.12E-09	-0.827981668
ENSG00000133059	DSTYK	1.385519	1.383944	1.181748	1.022953	0.930518	0.869941	0.856702	0.675384	0.539384	0.025422221	-0.345215936	0.00000491	-0.827693916
ENSG00000158806	NPM2	3.343221	3.723226	2.970173	1.375958	1.886717	1.229641	1.774394	2.028617	2.216698	6.65E-08	-1.181863053	0.0000696	-0.825669106
ENSG00000038295	TLL1	0.365854	0.154698	0.337346	0.070044	0.04159	0.085643	0.0899	0.122671	0.219659	0.0000257	-1.882373932	0.059811707	-0.825474934
ENSG00000185947	ZNF267	4.198392	4.585612	4.084301	5.633307	5.927165	5.357773	2.159564	2.177919	2.41134	9.97E-08	0.563837193	1.72E-10	-0.824278509
NewGene 5577	--	4.936282	4.023115	2.968771	1.390073	1.724198	1.299982	1.299261	2.037001	2.726049	2.75E-12	-0.82022101	8.13E-13	-0.823915206
ENSG00000131351	HAUS8	9.48761	8.465767	9.566459	8.072214	6.775878	7.485058	5.026384	4.131261	4.574611	0.571028914	-0.089459629	4.45E-10	-0.823080351
ENSG00000119231	SENP5	6.841671	6.945533	7.482875	7.322715	6.343433	6.44136	3.863381	3.982363	3.948477	0.451983544	0.077322891	2.69E-23	-0.822541189
ENSG00000187758	ADH1A	1.82002	1.861053	0.806072	1.638786	2.028045	1.865726	0.901333	0.776731	0.671917	0.208620939	0.451269716	0.030532872	-0.822493234
ENSG00000196458	ZNF605	5.587006	5.61977	5.431879	5.542705	4.667038	4.471664	2.82889	2.909126	2.82221	0.984779395	0.002336073	3.67E-25	-0.822358384
ENSG00000179981	TSHZ1	1.889054	1.767344	1.950202	1.748251	1.331736	1.51946	0.953299	0.902624	1.106554	0.485255719	-0.131761733	0.000000105	-0.822066303
ENSG00000168936	TMEM129	17.384224	17.217637	16.184122	15.235916	13.819194	17.108123	8.646015	8.275751	8.888718	0.557919542	-0.063247127	1.65E-20	-0.821673532
NewGene 5333	--	1.901028	2.283561	2.304079	2.238257	2.120741	1.932824	1.575088	0.66359	1.510151	0.546672065	0.137383068	0.000722325	-0.821672311
ENSG00000164344	KLKB1	0.773907	0.522936	0.519697	0.74894	1.135277	0.464491	0.187747	0.291121	0.472968	0.204447933	0.541961516	0.06426574	-0.820994456
ENSG00000172461	FUT9	0.341758	0.38436	0.496296	0.425044	0.320835	0.398618	0.19138	0.187574	0.270711	0.85863081	0.053678022	0.000386218	-0.820526642
NewGene 258	--	3.999942	3.996447	5.960076	1.965861	2.670884	1.958901	1.772389	2.57493	3.004602	9.48E-08	-0.92897306	0.0000145	-0.820347242
NewGene 3549	--	2.545663	3.347546	2.855494	2.724432	2.078421	2.575844	2.17462	1.169004	1.711195	0.935554599	0.015667364	0.000000358	-0.819760776
ENSG00000166272	WBP1L	8.888762	8.458836	7.855019	7.501322	7.772916	7.781377	4.373289	4.163223	4.87718	0.839955297	0.025344814	2.32E-18	-0.81918892
NewGene 3709	--	5.243529	5.135686	5.698611	5.153144	5.577939	4.636175	2.943884	2.109028	3.776691	0.565815685	0.09159005	0.00000143	-0.819140263
ENSG00000165300	SLITRK5	0.129592	0.154464	0.180993	0.169293	0.133381	0.151699	0.091637	0.090232	0.062863	0.716690592	0.115789318	0.00367213	-0.818626436
ENSG00000236104	ZBTB22	8.300135	7.114948	6.343338	7.316256	6.924088	7.681676	3.959777	3.817306	3.742604	0.222960632	0.163060609	9.68E-11	-0.818551576
ENSG00000141298	SSH2	2.297846	3.401011	3.037922	2.465043	2.237671	2.479351	2.483779	2.019111	1.958025	0.394899676	-0.115643959	2.02E-10	-0.81808832
ENSG00000164506	STXBP5	2.019453	2.34424	2.213607	2.261832	1.692817	1.605657	1.31174	0.932848	1.239458	0.178255917	-0.488213848	2.48E-10	-0.818032854
ENSG00000180257	ZNF816	2.246931	2.381303	2.196951	1.528491	1.820946	1.986854	1.352659	1.334238	1.190043	0.219770533	-0.26223053	0.0000249	-0.81681755
ENSG00000115526	CHST10	4.098466	3.637455	3.540587	3.43901	2.852419	3.133709	1.942644	2.027445	2.032946	0.582052708	-0.100792715	0.000000034	-0.816739637
ENSG00000105227	PRX	0.373272	0.350775	0.321552	0.394891	0.458343	0.402128	0.175538	0.13092	0.257099	0.262235701	0.360425368	0.022698431	-0.816155474
ENSG00000010319	SEMA3G	0.947179	0.818087	0.542794	0.638091	0.512676	0.326344	0.460684	0.42665	0.31982	0.108317644	-0.538481711	0.003750439	-0.815185187
ENSG00000099875	MKNK2	33.354423	31.543125	30.315547	26.083189	26.050084	27.484559	18.009329	18.435087	20.02467	0.062469102	-0.135027201	3.09E-27	-0.814921628

Gene ID	Gene name	Ctrl-1 FPKM	Ctrl-2 FPKM	Ctrl-3 FPKM	IFNγ-1 FPKM	IFNγ-2 FPKM	IFNγ-3 FPKM	Sora-1 FPKM	Sora-2 FPKM	Sora-3 FPKM	Ctrl vs IFNγ DESeq2 FDR	Ctrl vs IFNγ DESeq2 log2FC	Ctrl vs Sora DESeq2 FDR	Ctrl vs Sora DESeq2 log2FC
ENSG00000168062	BATF2	1.068842	1.340116	1.071295	102.226634	106.268995	105.170996	0.592489	0.662717	0.577224	0	6.632556663	0.00657392	-0.814912617
ENSG00000154080	CHST9	3.640465	3.85206	3.842023	1.025113	1.016919	0.811767	2.17017	2.062893	2.57809	1.67E-66	-2.001153606	4.64E-14	-0.814862359
ENSG00000118276	B4GALT6	2.468093	3.023462	3.326186	3.116546	2.521533	2.750858	1.826941	1.434997	1.549713	0.967820559	0.010230097	0.000000658	-0.814620683
NewGene_386	--	1.031259	1.180051	0.700206	0.843489	0.710138	0.724538	0.321779	0.495111	0.698532	0.550783992	-0.167550704	0.00306144	-0.813909918
ENSG00000106617	PRKAG2	2.285388	2.742978	2.673022	2.20761	2.552682	2.281494	1.351878	1.413788	1.515368	0.944477548	-0.018973566	0.0000141	-0.813692943
ENSG00000166664	CHRFAM7A	0.198279	0.256527	0.258053	0.229829	0.160803	0.15779	0.122335	0.123185	0.131375	0.629307326	-0.228047523	0.036297457	-0.813037572
ENSG00000113971	NPHP3	0.782515	1.124956	1.060833	1.157523	1.233623	1.406831	0.946384	0.472292	0.472768	0.006495769	0.603766835	0.009760678	-0.812747273
ENSG00000175548	ALG10B	2.317183	3.085283	3.12849	2.170197	1.906231	1.843867	1.474946	1.525552	1.522251	0.001556443	-0.403665842	6.55E-11	-0.812034059
ENSG00000205413	SAMD9	0.428266	0.315622	0.375401	1.595587	1.334444	1.66076	0.236073	0.154466	0.206033	1.79E-38	2.172332812	0.00759745	-0.811382398
ENSG00000065802	ASB1	8.88823	9.349284	9.623988	6.305425	6.25204	7.014061	5.329709	6.490947	6.073388	4.85E-08	-0.380063151	1.86E-22	-0.811260288
ENSG00000197016	ZNF470	1.043997	1.127778	1.134513	1.001331	0.993253	0.796107	0.657725	0.549428	0.625673	0.508802707	-0.133709585	0.00000126	-0.811091118
ENSG00000244693	CTAGE8	2.163298	2.468865	2.100724	1.919955	2.000246	1.884199	0.809608	1.325177	1.46468	0.790538783	-0.065809313	0.00039531	-0.810698141
ENSG00000119401	TRIM32	5.026271	5.179351	4.308615	4.872625	3.673322	4.256993	2.502258	2.374218	2.843505	0.889239124	-0.026603559	6.28E-11	-0.810397516
NewGene_3081	--	0.803496	0.83613	0.665596	0.527655	0.457213	0.389058	0.291111	0.354575	0.649728	0.074554951	-0.570782447	0.012946763	-0.810341082
ENSG00000177427	MIEF2	3.415876	3.552908	3.206826	3.10205	4.150845	4.90895	2.154653	1.579419	1.889196	0.258115844	0.178546086	0.000000041	-0.810116365
ENSG00000144891	AGTR1	150.911633	135.174757	139.551027	141.745115	149.842662	144.731105	67.696638	71.259668	86.372403	0.004014712	0.180394425	3.12E-26	-0.810064732
ENSG00000132623	ANKEF1	5.617508	5.915566	6.038858	4.45347	3.898451	4.404177	3.19174	3.201441	3.055461	0.000392356	-0.344892991	6.7E-17	-0.808467312
NewGene_5217	--	0.702966	1.312561	1.189523	1.503246	1.308911	1.298246	0.613748	0.50641	0.387795	0.00000614	0.779502679	0.000121702	-0.807971475
ENSG00000177853	ZNF518A	16.143856	17.223142	18.266537	19.912695	17.471336	17.912329	8.788475	9.071156	9.843945	0.000152042	0.240788152	9.59E-33	-0.807605168
ENSG00000183735	TBK1	12.112162	13.129169	13.308437	14.634656	13.389401	13.393692	6.738894	6.653684	7.365873	0.001393393	0.252650286	1.5E-21	-0.807455937
ENSG00000171522	PTGER4	3.356166	3.420869	3.731356	1.363222	1.475211	1.364856	1.576902	1.792515	2.273311	3.52E-19	-1.171338581	3.79E-08	-0.806656191
ENSG00000134852	CLOCK	3.474543	3.904608	4.080908	4.080902	3.653636	3.623396	1.996095	2.192196	2.313149	0.153605318	0.140511541	1.02E-18	-0.805219487
ENSG00000152439	ZNF773	2.954281	2.968929	3.533129	2.650828	2.546978	2.356061	1.807165	1.221936	1.605806	0.59195448	-0.107077253	0.00000154	-0.804841184
ENSG00000196371	FUT4	1.116757	1.077099	1.028312	0.695769	0.721207	0.768356	0.484758	0.531224	0.713371	0.025003926	-0.409455501	0.000031	-0.80475664
ENSG00000182013	PNMA8A	0.773269	1.33492	1.137008	0.77701	0.699156	0.72196	0.52611	0.651495	0.643515	0.096940554	-0.454781482	0.002461346	-0.803339988
ENSG00000124209	RAB22A	2.676798	2.519635	2.729009	2.522684	2.216488	2.175435	1.354939	1.352141	1.557173	0.708556741	-0.051298735	1.85E-14	-0.803104668
ENSG00000131196	NFATC1	1.715535	2.193256	2.183002	2.17258	2.052093	2.062385	0.956739	0.780895	1.536963	0.212913304	0.211021132	0.0000889	-0.802736424
ENSG00000174405	LIG4	5.965395	5.909001	5.792999	6.442285	7.001406	6.042123	3.24624	3.077712	3.092261	0.00239891	0.294690532	2.86E-15	-0.802683751
ENSG00000132464	ENAM	0.526225	0.373741	0.344493	0.292339	0.351589	0.307389	0.211006	0.14118	0.279846	0.643494914	-0.183808103	0.022522413	-0.800637454
ENSG00000101191	DIDO1	20.537783	20.371391	19.016721	17.298299	18.271979	16.696458	13.399149	12.079136	12.928309	0.106429864	0.10558438	2.07E-32	-0.800356392
ENSG00000129167	TPH1	0.276816	0.477407	0.426381	0.531871	0.355764	0.370441	0.167646	0.273263	0.232354	0.699293518	0.157037369	0.02293484	-0.799970769
ENSG00000148411	NACC2	6.958349	7.326794	6.617909	6.523395	5.892684	6.448789	3.543154	3.417115	4.322132	0.936822988	0.009741313	2.54E-18	-0.799613516
ENSG00000181856	SLC2A4	3.210478	3.287937	3.143038	3.246558	2.94366	2.954721	1.888517	1.662953	1.756588	0.7573537	0.055313049	2.01E-08	-0.798974325
NewGene_4922	--	0.948972	1.086914	1.272038	1.203871	1.228461	1.257127	0.697752	0.504073	0.580278	0.054890956	0.305929902	0.00000948	-0.79857674
ENSG00000123342	MMP19	0.820171	0.915359	1.198541	0.895757	0.845109	0.760688	0.503552	0.631317	0.644472	0.832611806	-0.071516735	0.002030788	-0.798000196
NewGene_1142	--	1.118279	0.834248	0.78421	0.993574	0.555349	0.592329	0.563775	0.343984	0.563006	0.719645105	-0.189641271	0.058570885	-0.797690716
ENSG00000010818	HIVEP2	1.905469	1.889556	2.283583	1.256676	1.188341	1.159184	1.150356	0.999893	1.128541	4.07E-08	-0.620998515	3.04E-11	-0.797668649
ENSG00000170190	SLC16A5	1.371706	1.556442	1.502241	0.827666	0.523549	1.238527	0.726037	0.688517	0.992438	0.037778703	-0.670983322	0.006624301	-0.797601804
ENSG00000125266	EFNB2	5.133963	3.821009	4.561497	2.587921	3.975081	3.121003	2.168003	2.192222	2.890171	0.053289319	-0.315859941	7.48E-09	-0.795174
ENSG00000197857	ZNF44	4.399011	5.338743	5.679978	5.627925	4.904379	4.25144	2.830346	2.702602	3.037375	0.551700773	0.105108255	3.02E-08	-0.79507026
ENSG00000178988	MRFAP1L1	41.946224	39.335423	38.262459	35.89373	36.927933	33.182793	19.78376	21.810366	23.093626	0.80407858	-0.026627046	2.14E-22	-0.793477126
ENSG00000189337	KAZN	0.245092	0.254747	0.197626	0.169843	0.160336	0.240107	0.096846	0.076878	0.197112	0.932222296	-0.046801503	0.071422179	-0.792577826
ENSG00000131845	ZNF304	3.541432	3.351238	3.978517	3.337907	2.975484	3.083445	2.052322	1.61746	2.220882	0.636123855	-0.077945673	8.31E-08	-0.790951631
NewGene_638	--	2.722227	2.888537	3.214401	1.502659	1.360268	1.353068	1.506965	1.846147	1.618746	8.86E-12	-0.835245552	4.31E-09	-0.790767495
ENSG00000131725	WDR44	1.61357	1.50595	1.758156	1.513948	1.288392	1.461324	0.728877	0.744072	1.183151	0.846879112	-0.047036198	0.000142304	-0.790733516
ENSG00000103707	MTFMT	3.587453	3.471274	3.61111	2.651707	3.075436	2.900523	2.160654	1.941806	1.669761	0.367309808	-0.15485382	0.00000136	-0.78991863
NewGene_834	--	1.100975	1.622365	1.18645	1.146404	1.407783	1.21249	0.780724	0.507933	0.833448	0.8066486	0.099887948	0.02143387	-0.789100683
ENSG00000236761	CTAGE9	0.497175	0.714944	0.40514	0.388118	0.508408	0.699229	0.288968	0.232716	0.348601	0.803384882	0.137774309	0.0751994	-0.78878938
ENSG00000196782	MAML3	1.550463	1.669062	1.397407	1.596766	1.530957	1.718033	0.887859	0.807566	0.838033	0.121785572	0.226630183	0.00000011	-0.78841706
ENSG00000275410	HNF1B	10.094805	10.620775	11.075843	6.552721	6.811325	6.643906	5.570964	5.974858	6.005932	4.02E-09	-0.510992154	8.52E-17	-0.788325327
ENSG00000148965	SAA4	5.939256	8.43711	6.546928	1.77575	2.576688	2.374889	3.715867	3.763814	3.818673	6.66E-10	-1.478280625	0.000308851	-0.787958046
ENSG00000104142	VPS18	12.695149	13.297821	11.476717	12.056427	11.994974	12.25652	6.5979	6.019426	7.824518	0.2924085	0.098713686	1.37E-16	-0.78779594
ENSG00000123636	BAZ2B	4.376492	4.188664	4.122341	4.084865	3.759251	3.628956	2.403549	2.155303	2.747602	0.802747044	-0.03128764	8.96E-15	-0.78721444
ENSG00000102524	TNFSF13B	0.407273	0.514518	0.707657	3.038992	3.474712	2.976185	0.294271	0.211617	0.377588	5.81E-39	2.661943033	0.061187904	-0.787139853
ENSG00000198142	SOWAHC	19.433735	20.085155	21.210752	16.500553	15.533103	16.077745	9.696976	10.435387	12.941948	0.004057457	-0.188396045	1.01E-18	-0.786848749
ENSG00000162878	PKDCC	3.979717	4.696813	3.250174	2.631216	2.351753	2.621606	2.134201	2.125563	2.210346	0.003341909	-0.499368971	0.00000289	-0.786116031
ENSG00000132467	UTP3	26.295546	26.677086	29.719791	24.929451	24.208082	24.476456	13.947227	15.740355	15.277557	0.856107824	-0.020219545	4.79E-19	-0.784544088
NewGene_37	--	5.165478	4.664955	4.44925	4.429498	4.375673	4.430859	2.250309	2.522187	3.015976	0.802747044	0.036047519	1.72E-10	-0.784044282
ENSG00000170390	DCLK2	0.231767	0.252123	0.358045	0.246852	0.292363	0.294585	0.120622	0.124448	0.175968	0.824598021	0.118643811	0.082506433	-0.783896701
ENSG00000189079	ARID2	4.18596	4.436272	4.537807	3.829949	3.393377	3.561431	2.577567	2.192191	2.408286	0.150124885	-0.133131977	7.87E-19	-0.783821667
NewGene_2928	--	1.567303	1.36172	1.314218	1.142834	1.136426	0.978016	0.807055	0.672603	0.815723	0.148952883	-0.296981063	0.0000462	-0.783088595
ENSG00000163661	PTX3	1.333891	0.890147	2.431468	0.428829	0.250135	0.179016	0.692448	0.965113	0.867243	2.98E-08	-2.283916165	0.032756218	-0.781102249
NewGene_1942	--	1.227122	1.375256	1.359818	1.071577	1.450926	1.433148	0.7531	0.676772	0.726532	0.555296026	0.147676204	0.000123823	-0.78063932
ENSG00000179627	ZBTB42	2.680021	2.163943	1.740118	2.413345	2.621326	3.653173	1.029078	0.896589	1.460969	0.013490596	0.42542374	0.0000784	-0.780015837
ENSG00000171453	POLR1C	19.793767	19.648404	21.04137	14.986032	14.206573	14.929047	10.161744	10.325888	11.717609	0.002017253	-0.295311428	5.15E-15	-0.779510147
ENSG00000104853	CLPTM1	44.096458	40.072959	40.778925	41.141656	39.328458	38.506266	19.331303	20.859971	27.996747	0.302038479	0.078502419	1.25E-13	-0.778464337
ENSG00000132141	CCT6B	1.333646	1.520393	2.098701	2.047701	1.650237	1.757292	1.136544	0.722648	0.757682	0.35923172	0.283955541	0.012937059	-0.778111694
ENSG00000181274	FRAT2	13.786054	14.47449	12.462064	12.150734	13.401758	14.894461	7.638874	7.361156	7.256622	0.247390022	0.138999649	6.22E-15	-0.777567673
ENSG00000137338	PGBD1	4.508395	4.374217	4.035378	3.653907	3.26571	3.371022	2.509124	2.056567	2.509754	0.194043638	-0.182242867	6.59E-09	-0.777409097
ENSG00000173546	CSPG4	0.696621	0.504901	0.323891	0.464654	0.420471	0.474496	0.352829	0.184203	0.299237	0.969688597	-0.016307897	0.012937748	-0.777352671
NewGene_705	--	1.553115	2.191378	1.929376	1.32788	0.911827	0.737417	1.102192	0.76484	1.398348	0.00000886	-0.84877818	0.00017901	-0.777226798
ENSG00000145675	PIK3R1	12.793324	12.11424	13.564546	16.11946	16.169046	14.384784	9.627259	6.482679	8.668549	2.54E-08	0.333584852	7.03E-31	-0.776433308
ENSG00000106799	TGFBR1	6.742863	7.08894	6.608639	6.029582	5.425569	5.627376	3.857176	3.483025	3.69528	0.219050501	-0.109577758	1.71E-20	-0.776060965
ENSG00000005238	FAM214B	2.078607	1.902979	2.240605	1.739391	1.760527	1.953844	0.650864	1.23303	1.271344	0.736847597	-0.073711869	0.0000107	-0.775609316

Gene ID	Gene name	Ctrl-1 FPKM	Ctrl-2 FPKM	Ctrl-3 FPKM	IFNγ-1 FPKM	IFNγ-2 FPKM	IFNγ-3 FPKM	Sora-1 FPKM	Sora-2 FPKM	Sora-3 FPKM	Ctrl vs IFNγ DESeq2 FDR	Ctrl vs IFNγ DESeq2 log2FC	Ctrl vs Sora DESeq2 FDR	Ctrl vs Sora DESeq2 log2FC
ENSG00000164296	TIGD6	1.830892	1.665356	1.255833	0.972583	1.086072	1.129166	0.877672	0.676167	0.935089	0.142821046	-0.357618423	0.000626537	-0.774688225
ENSG00000118520	ARG1	4.959223	4.042192	2.839697	1.526238	1.568171	1.368508	2.247863	2.411697	1.793384	5.26E-08	-1.259538856	0.001164333	-0.774437044
ENSG00000152127	MGAT5	3.642014	3.566644	3.825198	2.368845	2.3448	2.24392	1.72485	1.917179	2.427533	1.28E-09	-0.525875383	1.33E-11	-0.772708972
ENSG00000148200	NR6A1	3.09397	2.987178	2.970618	2.731644	2.693062	2.533344	1.686741	1.44899	1.7475	0.853962939	0.027374615	2.97E-12	-0.771344243
ENSG00000198954	KIF1BP	6.514201	6.798336	7.34292	5.765368	5.666735	5.851297	3.813686	3.991569	3.493809	0.411741878	-0.108440359	4.84E-10	-0.770169944
NewGene_1127	--	1.804672	2.182691	2.088976	1.849244	2.996725	1.49624	1.786254	0.875846	1.076439	0.568318579	0.183606934	0.001134502	-0.769572552
ENSG00000140299	BNIP2	16.799385	17.991979	22.785715	17.383034	17.858354	16.675029	10.739568	10.856026	15.877226	0.819940554	0.024621155	1.89E-20	-0.768895239
ENSG00000261308	FIGNL2	1.88584	1.433028	1.202204	0.916332	0.996905	0.884823	0.793399	0.920199	0.764742	0.008307894	-0.544365228	0.000222555	-0.768847408
ENSG00000141040	ZNF287	1.306892	1.203321	1.0626	0.86541	0.651452	1.048931	0.844595	0.59233	0.443472	0.520027944	-0.202320264	0.007280493	-0.768829668
ENSG00000075539	FRYL	5.750221	6.181416	6.739053	6.652158	6.101975	5.214215	3.473529	3.172968	3.197485	0.087458223	0.140256449	2.27E-20	-0.768523039
ENSG00000055957	ITIH1	2.479315	1.709244	0.836919	1.056168	1.132533	1.109144	0.822352	0.949539	0.632215	0.244790135	-0.390990403	0.016596019	-0.768222885
ENSG00000186212	SOWAHB	3.429884	2.558853	2.281602	2.800012	2.669418	2.612883	1.413979	1.561689	1.576223	0.615063418	0.111646394	0.0000352	-0.768013599
ENSG00000108839	ALOX12	1.152828	1.072292	1.112971	1.246598	1.038989	1.016503	0.636419	0.617337	0.568291	0.692325672	0.13155786	0.006619385	-0.767280936
ENSG00000205060	SLC35B4	7.330744	7.369822	7.487824	6.168309	5.781875	5.877609	3.842499	4.127424	4.394856	0.027193128	-0.167418392	6.61E-22	-0.767074406
ENSG00000124508	BTN2A2	10.494409	6.944117	6.491561	4.589393	4.800024	4.106977	3.802865	3.194293	4.5911	0.0000405	-0.44886347	1.67E-11	-0.766857956
ENSG00000185818	NAT8L	12.995147	13.287511	11.980125	10.097697	10.003687	11.098955	6.661377	6.85734	7.52463	0.096395321	-0.138856285	1.83E-23	-0.766715688
ENSG00000169860	P2RY1	0.377472	0.416808	0.460637	0.317332	0.255424	0.228417	0.184861	0.210084	0.292539	0.089182879	-0.501115957	0.007754735	-0.766566677
ENSG00000179029	TMEM107	1.145137	1.697735	0.985732	0.854576	0.43652	0.501169	0.747225	0.932626	0.586696	0.03670111	-0.78809091	0.035413723	-0.766386309
ENSG00000185946	RNPC3	3.898652	3.62424	4.162834	4.197466	3.694595	3.339347	2.775154	2.179484	1.882864	0.71725458	0.071831578	0.00000179	-0.765994877
ENSG00000182795	C1orf116	3.263106	2.974255	3.090251	2.374079	2.672458	2.250696	1.48491	1.521762	2.039863	0.149043838	-0.198551878	0.000000011	-0.765391237
ENSG00000124688	MAD2L1BP	28.677818	27.439355	26.154506	24.33412	24.346502	25.115387	13.917706	15.696038	14.945604	0.903642404	-0.014885078	3.02E-16	-0.764881672
NewGene_6229	--	1.023459	0.68381	0.882751	0.6537	0.936545	0.909097	0.615848	0.324419	0.431416	0.853136186	0.079747053	0.031518019	-0.76391218
ENSG00000156509	FBXO43	0.663676	1.079639	1.030417	0.92129	0.440933	0.718704	0.478917	0.619179	0.40199	0.463394609	-0.285867352	0.017531698	-0.762598965
ENSG00000129521	EGLN3	1.525375	1.339522	1.386378	1.99725	1.795607	1.669031	0.850024	0.713999	0.803275	0.016067065	0.486174288	0.001038304	-0.762521292
ENSG00000228570	NUTM2E	0.382623	0.321513	0.292881	0.311666	0.376571	0.232797	0.150969	0.194519	0.204318	0.99155889	-0.006903749	0.075426402	-0.762508992
ENSG00000055732	MCOLN3	7.410648	8.870466	10.245739	5.512956	5.326296	4.936835	4.811128	4.090981	5.860413	0.00000073	-0.600099632	3.85E-08	-0.762326148
NewGene_898	--	0.492214	0.620262	0.603943	0.730852	0.531217	0.559138	0.304535	0.190339	0.456694	0.422098926	0.229556273	0.014925654	-0.761670193
ENSG00000258659	TRIM34	2.439545	2.942825	2.359944	4.528503	4.392229	4.79146	1.526759	1.28231	1.472568	1.11E-11	0.946730587	0.0000612	-0.761566853
ENSG00000227507	LTB	5.868553	3.681737	3.79759	0.098947	0.330889	0.466167	2.80417	2.005846	2.558127	1.58E-21	-3.637549316	0.004529346	-0.761503552
NewGene_5429	--	0.976388	1.135618	0.705285	1.076959	1.22343	0.399044	0.579675	0.648153	0.513898	0.870646545	0.094604496	0.059057022	-0.760276647
NewGene_4503	--	1.535289	2.212142	2.777138	2.420407	2.136625	1.533703	1.310613	1.015152	1.286713	0.887825072	0.045651353	0.000316567	-0.760112672
ENSG00000153234	NR4A2	1.448309	1.582969	1.177803	0.790553	0.762532	0.949828	0.657988	0.558706	0.878962	0.063479849	-0.459836435	0.002408369	-0.760065299
ENSG00000213793	ZNF888	1.714743	1.977601	1.904254	1.873069	1.700945	1.32709	1.082592	1.033039	0.982938	0.85312246	-0.046496348	0.00000624	-0.757706317
NewGene_3037	--	0.731247	0.730397	0.899745	0.488784	0.348094	0.477436	0.33293	0.413997	0.498429	0.073017893	-0.675268573	0.035103258	-0.757552615
ENSG00000106608	URGCP	10.889414	11.744601	10.051509	9.597549	9.468194	10.148688	6.403632	5.072366	7.039695	0.910440372	-0.013459228	1.05E-16	-0.756981367
ENSG00000196418	ZNF124	17.05956	18.554237	21.968673	17.299544	16.385086	15.559389	10.581973	10.812327	10.652489	0.646056258	-0.063486719	3.96E-12	-0.756112681
ENSG00000064763	FAR2	0.629293	0.4587	0.798517	1.084661	0.848799	0.923437	0.379361	0.398265	0.293663	0.007649103	0.766711127	0.036866232	-0.75540313
ENSG00000204611	ZNF616	3.63632	3.263604	3.349169	2.378133	2.345296	2.229374	2.15806	1.816614	1.836969	0.000417815	-0.436323592	1.18E-08	-0.755209728
ENSG00000164252	AGGF1	6.160962	6.682993	6.719391	6.325663	5.651821	6.127065	3.604728	3.40102	3.884887	0.776721304	0.035657094	1.65E-15	-0.754139246
ENSG00000134884	ARGLU1	23.776611	23.733791	25.044926	24.53688	23.015013	22.276651	12.914785	13.240636	14.19655	0.224129304	0.091295262	1.97E-27	-0.753789006
NewGene_1366	--	0.364985	0.556593	0.399743	0.300964	0.406898	0.582061	0.274055	0.145455	0.322273	0.850311031	0.109398884	0.108783697	-0.753432026
ENSG00000109654	TRIM2	3.453124	3.294054	3.361002	3.737759	3.904882	3.285496	1.943262	1.836966	2.139451	0.021085019	0.243066027	1.75E-13	-0.753148455
ENSG00000277462	ZNF670	12.520857	12.941366	16.058647	12.069991	13.714317	12.288877	7.554591	7.737451	7.799002	0.894794052	0.022778028	6.59E-11	-0.752952592
ENSG00000175283	DOLK	12.506952	11.243954	11.613216	11.892977	10.68514	10.771885	6.442784	5.547507	7.758219	0.624324268	0.062095548	1.07E-10	-0.752619749
NewGene_728	--	1.419683	1.859675	1.865169	0.922084	0.816624	0.957379	0.929309	0.862279	1.246164	8.67E-10	-0.896513744	0.000000926	-0.752360931
ENSG00000171790	SLFNL1	0.250068	0.331907	0.426823	0.298032	0.333725	0.286084	0.243646	0.136056	0.162741	0.794959716	0.126193355	0.072501263	-0.752271933
ENSG00000225932	CTAGE4	1.748943	1.580723	2.04197	1.700808	1.990374	1.918946	0.839717	0.732445	1.444554	0.358433692	0.213441913	0.004104985	-0.751751187
NewGene_4652	--	1.552907	1.25056	1.283681	0.778985	1.313485	0.977431	0.932473	0.766079	1.153905	0.018136654	-0.493352949	0.000241383	-0.751422801
NewGene_686	--	0.772604	0.859325	0.788299	0.680371	0.526552	0.495833	0.379215	0.502007	0.53566	0.194043638	-0.319070872	0.001616959	-0.75074327
ENSG00000189114	BLOC1S3	5.140245	4.820975	4.569976	5.051349	4.3752	4.638045	2.439633	2.525269	2.946282	0.480776838	0.108774179	5.25E-08	-0.750069463
NewGene_49	--	0.498612	0.51965	0.420203	0.789419	0.760188	0.715096	0.349114	0.273031	0.204925	0.030761026	0.757436284	0.096530068	-0.749791843
ENSG00000177374	HIC1	0.28139	0.283373	0.318206	0.297686	0.26043	0.401317	0.30147	0.161128	0.115046	0.790138943	0.114164866	0.036889878	-0.74971096
ENSG00000005469	CROT	3.986919	4.706319	4.458567	6.505153	6.0444	5.54545	2.771884	2.597485	2.930497	0.000000124	0.623956726	4.79E-08	-0.749256099
ENSG00000108848	LUC7L3	35.808463	35.170632	39.952498	38.891581	31.763634	33.510653	21.830841	25.364518	28.936789	0.186717451	0.105075542	9.54E-25	-0.748996125
ENSG00000197841	ZNF181	4.332107	2.370865	2.81032	2.42253	3.115499	3.551505	1.536587	1.894294	2.324113	0.592133492	0.097027772	0.0000141	-0.7486839
ENSG00000161013	MGAT4B	82.281772	79.740267	81.543588	55.532899	51.902925	54.029117	42.037925	42.180579	43.980226	6.38E-13	-0.406550252	1.29E-32	-0.748556283
NewGene_6485	--	0.27965	0.568487	0.436891	0.27078	0.227583	0.322521	0.186647	0.224817	0.307196	0.268262912	-0.484499929	0.066630259	-0.747017645
NewGene_3540	--	2.652906	2.80257	3.153386	4.167317	2.847202	2.917615	1.647771	1.703232	1.525375	0.537966923	0.084227934	8.9E-11	-0.746541022
ENSG00000131370	SH3BP5	2.169965	1.892304	1.925339	1.312743	1.130986	1.564729	0.975855	1.089768	1.306534	0.050102404	-0.4192845	0.000177019	-0.746507817
ENSG00000277203	F8A1	2.348492	1.3479	1.420403	1.629373	1.478675	1.592252	1.067052	0.648686	1.174247	0.966532859	0.018703938	0.022844902	-0.746378155
ENSG00000183340	JRKL	1.355262	1.679957	1.491128	1.565154	1.264206	1.153754	0.769311	0.773044	0.993287	0.896584156	-0.041266699	0.001079957	-0.746369938
ENSG00000167074	TEF	4.084285	3.898763	3.737165	3.75994	4.030422	4.003109	2.335466	1.990286	2.244626	0.19780147	0.156285218	2.27E-10	-0.74462415
ENSG00000169851	PCDH7	1.792615	1.911452	2.328407	0.713161	0.643846	0.804833	1.197518	1.210561	1.285152	6.58E-18	-1.317389402	0.000000527	-0.744546373
ENSG00000112149	CD83	7.094031	6.491063	6.719786	2.203008	2.664372	3.000676	4.022133	3.658761	3.676816	5.11E-20	-1.21499386	4.52E-10	-0.744172887
NewGene_234	--	0.49358	0.730333	0.54671	0.518206	0.300422	0.725679	0.415951	0.302441	0.270479	0.958082541	-0.030780969	0.043926484	-0.743532339
ENSG00000172687	ZNF738	4.60281	4.334809	4.293745	3.019471	3.174752	3.028659	2.315594	2.469944	2.61622	0.054847136	-0.307471035	0.00000237	-0.74252729
ENSG00000206557	TRIM71	19.994152	20.508652	21.462669	18.886261	18.55562	18.658989	11.044852	10.985312	12.741652	0.964192542	0.003969975	5.23E-30	-0.742511872
ENSG00000180336	MEIOC	0.65755	0.929766	0.908774	0.652011	0.745262	0.624349	0.472673	0.406709	0.662517	0.602293131	-0.162962561	0.00611302	-0.742454869
NewGene_2426	--	1.123955	1.112423	1.155678	1.112239	0.934653	1.221156	0.710842	0.537821	0.65116	0.786631919	0.091502	0.006515379	-0.741601278
ENSG00000162396	PARS2	2.091558	1.892005	1.907906	1.799222	1.714938	1.806643	1.449425	1.077597	0.765682	0.99503435	-0.002087443	0.003647812	-0.741153926
ENSG00000175311	ANKS4B	3.861584	3.07189	2.678778	3.656042	4.055719	4.398295	1.705801	1.831397	1.847027	0.000820595	0.481064999	0.00000148	-0.740927788
ENSG00000244486	SCARF2	9.971505	10.586396	9.969203	5.401276	4.534807	5.17988	5.655625	5.384427	6.118103	4.54E-22	-0.867330764	1E-16	-0.740517139
ENSG00000197063	MAFG	11.556197	11.277335	11.324306	10.470286	10.164466	10.403748	6.198539	6.062075	6.927116	0.960983569	0.005341557	3.35E-23	-0.740499192
ENSG00000162241	SLC25A45	7.262518	6.458791	5.986114	7.316914	6.560466	6.654013	3.017719	4.233026	3.516926	0.04700802	0.259745839	0.00000212	-0.740444237

Gene ID	Gene name	Ctrl-1 FPKM	Ctrl-2 FPKM	Ctrl-3 FPKM	IFNγ-1 FPKM	IFNγ-2 FPKM	IFNγ-3 FPKM	Sora-1 FPKM	Sora-2 FPKM	Sora-3 FPKM	Ctrl vs IFNγ DESeq2 FDR	Ctrl vs IFNγ DESeq2 log2FC	Ctrl vs Sora DESeq2 FDR	Ctrl vs Sora DESeq2 log2FC
NewGene 6036	--	0.786575	0.537235	0.519955	0.355094	0.546934	0.504566	0.321794	0.476936	0.397285	0.543725349	-0.288649697	0.062446794	-0.740189433
NewGene 664	--	1.459781	2.474957	2.910115	1.020517	1.123341	0.960858	1.437185	0.909765	1.47843	3.95E-09	-1.00452925	0.0000779	-0.740167363
NewGene 3523	--	3.072596	3.698096	3.77744	3.614069	3.667763	2.972668	1.924687	2.382931	2.577972	0.598405799	0.079897812	2.35E-09	-0.740047824
ENSG00000198783	ZNF830	8.082031	8.233394	8.307565	7.345105	7.858842	8.014364	4.490641	4.432982	5.361767	0.710501556	0.052143087	1.86E-11	-0.739401395
ENSG00000116903	EXOC8	5.192045	5.63956	6.719916	5.549875	5.318923	5.321236	3.237062	3.037743	3.597337	0.837295786	0.031187531	6.23E-11	-0.73880272
ENSG00000149308	NPAT	3.286635	3.323108	3.892978	3.570829	3.647761	3.46033	2.036457	1.901077	1.919972	0.121282955	0.182377402	3.07E-10	-0.738711381
ENSG00000166133	RPUSD2	6.410796	6.309272	5.932828	6.655567	7.129286	7.141991	3.263602	3.126536	3.707738	0.004066883	0.325801092	1.42E-08	-0.737523888
ENSG00000217128	FNIP1	9.031431	9.700755	10.685451	9.317006	9.055167	8.215598	5.751591	4.84769	6.113265	0.878215984	-0.020105877	6.14E-15	-0.73693404
ENSG00000160991	ORAI2	2.526091	4.719398	2.684766	4.054039	3.779219	1.73058	2.231012	2.070695	3.521658	0.322256873	-0.135951833	0.000000867	-0.735742982
ENSG00000171451	DSEL	0.916682	0.933984	0.821464	0.558359	0.42119	0.473131	0.462972	0.561251	0.475069	0.00000911	-0.732745426	0.0000124	-0.735735426
ENSG00000143869	GDF7	0.615381	0.533188	0.533028	0.507357	0.453266	0.625171	0.367739	0.311785	0.263526	0.815484005	0.063575314	0.000503258	-0.735530433
ENSG00000176641	RNF152	0.690496	0.999153	0.763703	0.554708	0.574193	0.500969	0.524681	0.427161	0.635647	0.07490832	-0.360429321	0.00046257	-0.735356116
ENSG00000143756	FBXO28	10.942699	11.379603	12.997163	10.168398	10.083231	10.676649	6.705916	5.994276	7.311197	0.604579771	-0.054865143	1.5E-16	-0.735047148
NewGene 4277	--	0.747003	0.893423	0.857653	0.780365	0.681413	0.815616	0.552762	0.537363	0.304709	0.88938211	-0.044644731	0.007633645	-0.734967514
ENSG00000047056	WDR37	2.710911	2.506537	2.909404	2.805963	2.677985	2.861848	1.480893	1.415604	1.553793	0.169132359	0.198211273	0.000000261	-0.733125641
ENSG00000164692	COL1A2	0.231343	0.223011	0.254447	0.240613	0.156118	0.255132	0.129669	0.138651	0.129964	0.976457703	0.017922699	0.073203085	-0.732743733
ENSG00000051341	POLQ	3.831587	4.453767	4.716611	4.413994	3.573316	3.931204	2.51254	2.400328	2.417875	0.889945453	0.021074569	1.51E-13	-0.732663217
ENSG00000124788	ATXN1	0.933139	0.915345	0.918166	1.398003	1.477074	1.286668	0.558777	0.43923	0.550751	1.25E-10	0.751306911	0.0000018	-0.731691813
ENSG00000048052	HDAC9	4.879327	5.569808	5.815323	2.028178	1.565576	1.626969	2.775728	3.114557	3.395572	7.15E-31	-1.473232225	7.7E-10	-0.731285668
ENSG00000072832	CRMP1	1.282732	1.130105	1.20988	0.970721	0.9776	0.732957	0.766049	0.568748	0.710226	0.286119699	-0.284989242	0.00225877	-0.73096762
ENSG00000175505	CLCF1	3.670436	3.582615	3.154092	2.102499	1.526785	1.901451	2.065139	2.018374	1.747785	0.0000938	-0.749136831	0.000104723	-0.730016565
ENSG00000143753	DEGS1	26.64732	27.230913	27.950459	26.993607	26.459509	28.667263	14.81965	14.41245	17.100454	0.042438539	0.152968906	1.34E-20	-0.729897768
ENSG00000085063	CD59	54.175721	39.651255	40.956151	21.414833	24.288145	22.033021	22.067349	26.154182	27.46214	6.5E-50	-0.742592822	6.73E-33	-0.729571473
ENSG00000197296	FITM2	2.071406	2.437399	2.095788	1.875658	1.594794	1.855816	1.202203	1.251599	1.279918	0.344730475	-0.162789777	0.00000114	-0.729239718
ENSG00000235453	SMIM27	2.3556	2.161637	2.383233	1.813384	2.184409	2.009457	1.234567	1.731853	1.114296	0.024680062	-0.710616902	0.035485132	-0.728904708
ENSG00000135679	MDM2	8.79571	10.265568	11.383334	10.090427	9.637363	9.634276	5.704997	4.932089	6.285346	0.335153595	0.093128408	4.29E-15	-0.728597289
ENSG00000138756	BMP2K	4.700076	4.58711	5.677874	5.297171	4.663606	4.0174	3.502313	2.554578	2.909249	0.85863081	0.075410421	0.00000561	-0.727791405
NewGene 1068	--	0.720744	0.69426	0.596009	0.696467	0.629969	0.624227	0.290157	0.340875	0.523846	0.706330466	0.109457038	0.007585181	-0.727589757
ENSG00000101745	ANKRD12	3.362622	4.096352	4.572704	4.656058	4.830996	4.233544	2.185654	2.570056	2.414276	0.001089567	0.33342281	3.2E-12	-0.727219751
NewGene 4488	--	0.98508	0.519036	0.938526	0.595092	0.81966	0.819117	0.283692	0.438054	0.622313	0.913903952	0.05608963	0.068892101	-0.72685361
ENSG00000110723	EXPH5	0.545416	0.571707	0.674541	0.474174	0.469431	0.438978	0.429464	0.311441	0.28098	0.283017423	-0.224802843	0.000610678	-0.726802097
ENSG00000159588	CCDC17	0.380574	0.60081	0.980546	0.517125	0.503418	0.409918	0.230855	0.366473	0.467345	0.703182333	-0.219606758	0.139889068	-0.726733243
ENSG00000173517	PEAK1	3.405248	3.244399	3.802582	2.465422	2.587408	2.187597	1.919814	2.007327	1.625884	0.00016528	-0.366755728	3E-14	-0.726727474
NewGene 3198	--	0.423084	0.384168	0.473473	0.397666	0.288987	0.398661	0.250961	0.305311	0.190254	0.810854547	-0.117974705	0.068708176	-0.726502698
ENSG00000161958	FGF11	1.394058	0.844337	0.746309	0.620712	0.682275	0.656473	0.653393	0.575282	0.460706	0.109733641	-0.539413817	0.022961793	-0.726326917
ENSG00000162139	NEU3	1.868105	1.980829	2.164416	2.291291	1.510269	2.031047	1.223815	0.97431	1.064664	0.447638222	-0.132366494	0.000000331	-0.726297882
ENSG00000185670	ZBTB3	2.025541	1.893968	1.658433	0.589964	1.426124	1.576875	1.088784	0.937455	0.856426	0.645901618	-0.647667213	0.000598491	-0.725995675
ENSG00000110218	PANX1	8.278394	7.65547	7.743663	9.951477	10.231005	9.811934	4.286704	4.491209	4.634851	2.37E-08	0.487959814	6.21E-12	-0.72566015
NewGene 1902	--	0.860743	0.983931	1.158176	0.952132	1.539538	1.264158	0.444257	0.510987	0.73925	0.030951762	0.472699773	0.001519497	-0.725566675
ENSG00000174136	RGMB	3.450499	3.373755	3.482931	3.163587	3.168663	3.356752	2.338849	1.697257	2.071435	0.688028743	0.061029761	0.00000012	-0.723749206
NewGene 535	--	1.571852	1.942765	1.601611	1.450212	1.568736	1.368532	0.888455	0.961127	1.061965	0.795079518	-0.078732597	0.002088473	-0.723493992
ENSG00000254221	PCDHGB1	2.268419	2.001663	2.288923	1.50372	1.601826	1.392248	1.598194	1.386984	1.398059	0.009501201	-0.450684761	0.000251558	-0.723373233
ENSG00000159958	TNFRSF13C	0.372714	0.384303	0.433827	0.436861	0.505406	0.423871	0.18198	0.294767	0.188225	0.343944718	0.341414936	0.064687702	-0.722186696
ENSG00000134982	APC	4.939241	3.837055	4.263571	3.041787	4.170887	3.471485	2.171387	2.264338	2.583662	0.577724418	-0.198787405	3.04E-15	-0.722147056
NewGene 3116	--	4.064383	3.726247	3.380058	2.488465	2.784545	2.709557	2.449569	2.434956	2.196831	0.021545405	-0.334479187	0.000000269	-0.721778972
ENSG00000259316	AC087632.1	1.909924	2.24572	1.651209	1.117637	1.094301	1.084767	1.023182	1.518898	0.725592	0.134531962	-0.622841888	0.09153451	-0.721398927
ENSG00000167525	PROCA1	1.446166	1.158887	1.71568	1.007231	0.850504	1.115892	0.616719	0.89473	0.873369	0.311152598	-0.408050108	0.049995731	-0.721391151
ENSG00000159917	ZNF235	2.338237	2.654028	3.086445	2.119677	2.370876	1.799321	1.804585	2.096167	1.81352	0.298386137	-0.207566026	0.0000349	-0.721373146
ENSG00000138411	HECW2	0.186042	0.153665	0.139703	0.155843	0.100376	0.092584	0.113886	0.093418	0.070906	0.360052709	-0.360281089	0.04087878	-0.721315592
NewGene 5871	--	1.279905	1.185968	1.414252	0.93903	0.974981	0.762692	0.697339	1.052438	1.019726	0.089628487	-0.492116303	0.013317683	-0.721177995
ENSG00000186652	PRG2	1.080994	1.914279	1.340954	1.617439	0.874408	1.282764	1.000172	0.865938	0.598894	0.852661433	-0.090449146	0.049117638	-0.7207632
ENSG00000164086	DUSP7	1.826678	1.759507	1.998484	2.636027	2.87656	2.941625	1.068411	1.058896	1.042162	0.000000209	0.748134644	0.0000909	-0.719573859
ENSG00000160785	SLC25A44	17.797074	17.92885	17.710807	19.019803	18.827266	18.954648	9.46178	9.44216	11.67354	0.000266755	0.231098501	3.16E-18	-0.719439453
ENSG00000163093	BBS5	0.49179	0.715098	0.571304	0.34223	0.512766	0.561413	0.465509	0.250797	0.316699	0.437624309	-0.302227354	0.0374383	-0.718865021
NewGene 815	--	2.451732	1.990126	1.587366	1.184311	1.205629	1.013237	1.536094	1.160525	1.187129	0.002100674	-0.56825184	0.000237803	-0.718274488
ENSG00000170949	ZNF160	4.055351	4.436108	4.867287	3.750933	4.13655	3.973059	2.694771	2.406731	2.473682	0.6672993	-0.062806161	1.25E-09	-0.717873833
ENSG00000236287	ZBED5	17.423239	18.674339	17.160035	17.595141	15.184603	16.139819	9.936526	9.99112	10.083959	0.900713623	-0.014598801	3.32E-18	-0.716380514
ENSG00000084112	SSH1	2.68943	2.597407	3.003708	2.1763	1.947849	2.038277	1.68869	1.518251	1.831157	0.000795252	-0.304407061	9.66E-14	-0.715795387
ENSG00000176945	MUC20	0.88237	0.67387	0.769132	0.691008	0.665437	0.554957	0.426111	0.482096	0.431961	0.598604664	-0.196695704	0.023015541	-0.715308016
ENSG00000213047	DENND1B	2.138176	2.557809	2.628564	2.803585	3.086816	2.546404	1.583001	1.27134	1.554555	0.003697118	0.355811386	4.14E-08	-0.714546071
ENSG00000147852	VLDLR	1.302256	1.631724	1.637874	2.014915	1.585058	1.6023	1.062217	0.860674	0.825046	0.58756523	0.09644395	0.00000145	-0.713911146
ENSG00000187607	ZNF286A	4.181213	3.952709	4.290904	3.141768	2.819936	3.285084	2.425432	2.23654	2.960155	0.000241699	-0.383076186	9.05E-10	-0.713045112
ENSG00000178163	ZNF518B	0.283124	0.336494	0.298634	0.318888	0.348354	0.334457	0.157036	0.186481	0.180004	0.402243707	0.268762329	0.024853299	-0.713008603
ENSG00000166341	DCHS1	1.157284	0.727741	0.715854	0.547259	0.472734	0.516883	0.331372	0.567045	0.588745	0.002483438	-0.613200277	0.002602832	-0.712968335
ENSG00000169239	CA5B	1.057335	0.816399	0.887952	1.022444	0.931568	1.157622	0.765213	0.393754	1.058271	0.409129111	-0.194308475	0.002213607	-0.711332281
ENSG00000182902	SLC25A18	0.720487	1.080119	0.850145	0.793853	0.590462	0.764392	0.546912	0.442495	0.523546	0.730370143	-0.146283047	0.0363244	-0.711026953
ENSG00000143387	CTSK	1.881893	1.768669	1.52842	1.057903	1.173522	1.357397	0.744442	0.93138	1.329953	0.175912352	-0.398486281	0.014430937	-0.71077281
ENSG00000104894	CD37	2.370478	2.353047	1.929846	1.927258	1.780023	2.215895	0.913614	1.442792	1.171292	0.868949537	0.062398747	0.023047471	-0.710148538
ENSG00000268350	FAM156A	4.6384	2.52858	3.005509	2.237029	2.081418	2.272692	1.711318	1.786419	1.98151	0.034178512	-0.449895797	0.000446443	-0.709982154
ENSG00000197980	LEKR1	0.975454	1.050859	1.023326	0.891459	0.763483	0.822854	0.701111	0.636618	0.54621	0.489215459	-0.259118421	0.040189739	-0.709664922
ENSG00000224470	ATXN1L	4.635246	4.21963	4.014461	4.365338	4.309378	4.470047	2.62692	2.433213	2.31204	0.057253	0.177805189	2.2E-12	-0.709319698
ENSG00000119669	IRF2BPL	2.197013	1.678653	1.727591	2.322395	2.735231	2.637552	0.919716	1.027702	1.269027	0.0000842	0.605625932	0.000155786	-0.708540185
ENSG00000188542	DUSP28	1.31646	1.061763	1.027363	1.281809	0.992215	1.339819	0.685255	0.509719	0.774323	0.361702115	0.241155494	0.006624301	-0.708421053
ENSG00000181690	PLAG1	1.032227	1.235395	1.302183	1.352513	1.313876	1.146747	0.702638	0.700943	0.752202	0.314830374	0.177429112	0.0000181	-0.707997422

Gene ID	Gene name	Ctrl-1 FPKM	Ctrl-2 FPKM	Ctrl-3 FPKM	IFNγ-1 FPKM	IFNγ-2 FPKM	IFNγ-3 FPKM	Sora-1 FPKM	Sora-2 FPKM	Sora-3 FPKM	Ctrl vs IFNγ DESeq2 FDR	Ctrl vs IFNγ DESeq2 log2FC	Ctrl vs Sora DESeq2 FDR	Ctrl vs Sora DESeq2 log2FC
ENSG00000175087	PDIK1L	2.040989	2.464318	1.917131	2.299257	2.239079	2.024936	1.540383	1.283034	0.863513	0.630442292	0.093439178	0.000137682	-0.707795809
NewGene_6232	--	0.911902	1.087605	1.151461	0.847601	0.799623	0.859944	0.621434	0.645974	0.55142	0.56969373	-0.184924324	0.010438877	-0.707663799
ENSG00000272886	DCP1A	5.236245	5.989197	6.037716	6.492811	6.447734	6.734792	3.322665	3.328082	3.177827	0.0000272	0.355279963	4.8E-13	-0.707612127
NewGene_4802	--	5.704694	5.530029	4.13176	5.338475	4.030143	5.354694	3.453973	2.022549	3.434396	0.855944088	0.082409456	0.054465865	-0.706330236
ENSG00000108187	PBLD	2.176161	1.733164	2.189555	2.716084	2.128972	2.60538	1.437379	1.451808	0.760459	0.0524613	0.402826254	0.003119976	-0.706206478
NewGene_4842	--	0.464158	0.354939	0.444288	0.327095	0.275059	0.474361	0.206192	0.220949	0.302412	0.870122796	-0.081254153	0.050647276	-0.706066344
ENSG00000164100	NDST3	0.218283	0.177679	0.220295	0.109104	0.129771	0.0979	0.087497	0.123201	0.144355	0.112419251	-0.71370152	0.101540434	-0.706040199
ENSG00000283932	AL121722.1	34.255024	34.454041	31.930021	39.93317	36.494347	39.026649	20.286188	18.796703	18.74235	6.45E-10	0.345042924	1.84E-25	-0.705759248
ENSG00000171988	JMJD1C	7.430614	7.964646	8.084941	6.155998	6.528498	6.01022	4.956113	4.192009	4.798919	0.013740598	-0.17373448	1.76E-20	-0.705689529
ENSG00000145358	DDIT4L	3.840728	2.700961	2.857072	3.471075	3.622083	3.997471	1.933781	1.532251	1.973297	0.023869973	0.395558433	0.000200853	-0.704951779
ENSG00000158023	WDR66	3.614359	4.081242	4.128238	0.349374	0.387736	0.33808	2.162198	2.355405	1.939188	1.54E-74	-3.050381318	0.000000238	-0.703994343
NewGene_800	--	2.39069	2.962452	3.620518	2.418527	1.540598	2.803317	1.932355	1.697099	1.521258	0.347454067	-0.257917033	0.000915689	-0.703579992
ENSG00000188690	UROS	26.140328	26.460324	24.72631	25.964431	27.461107	28.26881	14.790002	15.453592	14.932711	0.000670672	0.268327439	1.61E-15	-0.703408607
ENSG00000145817	YIPF5	10.038151	11.289346	11.281453	11.341165	11.613342	11.559468	6.891085	5.480187	6.462253	0.051922688	0.17731545	1.77E-12	-0.702754921
ENSG00000033100	CHPF2	11.237944	10.472352	10.690272	11.308848	11.295625	11.12963	5.607129	5.248698	6.66289	0.006360813	0.225236623	2.31E-13	-0.701842711
NewGene_5746	--	4.90357	5.859978	5.683166	5.756735	4.976265	6.010749	3.540241	3.618886	3.545619	0.257878549	0.127245902	6.17E-14	-0.701629594
ENSG00000175928	LRRN1	2.944051	2.372876	2.472839	1.126423	0.907801	1.026702	1.387422	1.417065	1.702216	8.17E-14	-1.198192175	0.0000101	-0.700337794
ENSG00000064309	CDON	1.409961	1.115879	1.422734	0.783862	0.845364	0.708183	0.830203	0.744491	0.939286	0.0000953	-0.611168272	0.00000895	-0.69987693
ENSG00000184486	POU3F2	0.475641	0.453392	0.444837	0.414555	0.339355	0.360117	0.234216	0.347055	0.207437	0.674800863	-0.156245001	0.035501946	-0.699668641
ENSG00000182963	GJC1	6.964653	7.8696	7.858864	5.090724	5.137063	5.0803	4.716331	4.298787	4.851941	0.000000341	-0.414633446	5.03E-17	-0.697858663
ENSG00000130164	LDLR	36.545667	31.88944	33.530446	33.07838	32.54627	32.447977	19.835449	18.844088	23.229235	0.168993206	0.099218837	7.88E-18	-0.697165643
ENSG00000149212	SESN3	5.496618	6.605277	6.39048	3.296868	3.55993	3.134196	3.763554	3.183584	3.799989	1.88E-18	-0.747028936	3.25E-14	-0.69667171
NewGene_307	--	0.657023	0.727082	0.673999	0.745173	0.76601	0.745369	0.33311	0.348	0.463216	0.180660556	0.247285024	0.000223633	-0.696502228
ENSG00000214960	ISPD	1.52673	1.561993	1.796226	0.440536	0.398184	0.502805	0.934648	0.900145	0.978354	9.13E-23	-1.746154572	0.0000201	-0.696257328
ENSG00000105708	ZNF14	1.288375	1.722735	1.557224	1.408708	1.382638	1.11137	0.783045	0.885236	0.968368	0.785417546	-0.081189396	0.00218915	-0.695642971
ENSG00000130921	C12orf65	8.169977	8.325335	10.045229	8.058423	7.324085	8.174583	4.643405	4.733394	5.219512	0.99264631	0.001835231	1.92E-09	-0.695190759
ENSG00000267680	ZNF224	4.141731	5.243369	5.294653	6.994171	5.682251	5.437882	2.736236	2.912335	2.943102	0.001393393	0.388340662	3.35E-08	-0.694962612
ENSG00000125375	DMAC2L	2.008055	2.625879	2.013084	1.779839	1.806323	2.21415	1.220149	1.405778	1.424205	0.70263363	-0.066451522	0.00000026	-0.694949744
ENSG00000129354	AP1M2	1.688005	1.763353	1.738471	1.624355	1.308955	1.568287	1.093818	1.1985	0.750338	0.797504037	-0.089097251	0.015423058	-0.694892173
ENSG00000159658	EFCAB14	6.599028	6.79339	6.580918	7.613096	7.098516	6.569484	3.340791	3.676792	4.958161	0.008308208	0.227840013	5.92E-10	-0.694759415
NewGene_1925	--	1.236357	1.172374	1.215279	1.053338	1.179712	1.604079	0.947489	0.758193	1.138804	0.519963889	0.133595474	0.0000939	-0.694489357
ENSG00000101974	ATP11C	3.375257	3.778822	4.26103	3.960028	4.005636	3.219234	2.240132	2.120751	2.366151	0.140684485	0.171076201	9.8E-09	-0.694105575
ENSG00000081665	ZNF506	3.815565	3.848859	4.252371	2.85718	2.421279	2.919355	2.478526	2.773859	2.276173	0.104924213	-0.228328817	0.00000285	-0.69407865
ENSG00000249242	TMEM150C	0.299397	0.453472	0.607721	0.544695	0.573307	0.521676	0.16535	0.298985	0.296163	0.402936183	0.344613313	0.112644921	-0.694025407
ENSG00000107819	SFXN3	2.118911	1.756069	1.679703	0.843386	0.994742	0.981934	1.097305	1.126986	1.120253	0.0000734	-0.795091465	0.000385299	-0.693766745
ENSG00000177352	CCDC71	12.508639	10.172971	9.683039	9.899556	7.731296	9.233054	6.408212	5.893767	6.482114	0.440881865	-0.124306421	4.66E-08	-0.692920979
ENSG00000116141	MARK1	1.890427	1.286513	1.904449	1.199108	1.301941	1.532803	1.181447	0.990066	0.683088	0.236526384	-0.248358991	0.000724871	-0.692917575
ENSG00000112941	TENT4A	7.028855	8.199928	9.825219	8.184541	7.245989	7.545284	5.003133	4.237062	5.200601	0.922606069	0.016654765	3.88E-09	-0.692657747
ENSG00000181007	ZFP82	4.071011	4.715968	4.774008	3.88453	4.362421	3.946338	2.866549	2.892979	2.350029	0.909669446	-0.023857043	0.0000124	-0.69260219
ENSG00000136141	LRCH1	1.427236	1.218513	1.537835	2.148471	2.096851	1.901444	0.713739	0.818963	0.898842	0.00000815	0.660498653	0.000244093	-0.692446693
ENSG00000177946	CENPBD1	3.531071	3.804971	3.782399	3.358329	3.409802	2.963419	1.943399	2.210928	2.286901	0.801881548	-0.04659772	0.00000088	-0.692179697
ENSG00000213886	UBD	117.773682	102.349319	108.314774	1375.170166	1362.369873	1273.466431	64.781311	65.246887	60.324375	0	3.756912648	3.9E-18	-0.692095096
ENSG00000114541	FRMD4B	0.836382	0.970309	0.939941	1.282093	1.30752	1.028958	0.640388	0.48078	0.390347	0.010090312	0.51716458	0.003771123	-0.691767299
ENSG00000189042	ZNF567	4.208827	4.47011	5.074965	3.415308	3.257603	3.526728	2.595439	3.021565	2.289252	0.072733199	-0.255594815	0.00000865	-0.691408272
ENSG00000186889	TMEM17	1.260756	0.914485	1.264562	0.524584	0.697936	1.241233	0.826522	0.561647	0.587477	0.483835373	-0.313950472	0.042956698	-0.691321691
ENSG00000171121	KCNMB3	4.580715	4.734015	5.269496	4.223059	3.594276	4.211419	3.238379	3.074493	2.885963	0.56120453	-0.104876689	0.00000698	-0.69080836
ENSG00000152256	PDK1	2.636502	2.985933	2.228019	1.659979	1.728397	1.53545	1.509409	1.325271	1.450009	0.026883579	-0.28829659	1.16E-08	-0.69061531
ENSG00000261609	GAN	0.599595	0.659123	0.681517	0.598051	0.465799	0.540317	0.397322	0.317135	0.398442	0.314872797	-0.170240327	0.0000077	-0.690362333
ENSG00000166401	SERPINB8	2.930435	1.939318	2.115067	1.400725	1.044297	1.470804	1.356757	1.205128	1.779769	0.000868109	-0.620054973	0.000117732	-0.689660834
NewGene_4709	--	0.686459	0.757962	0.582789	0.750209	0.558519	0.579096	0.382151	0.220313	0.582946	0.93333198	0.043937207	0.108906405	-0.689626876
ENSG00000128016	ZFP36	5.812146	7.117284	5.145503	7.810673	9.0576	8.397832	3.508402	2.989088	3.990425	0.00000678	0.625210941	0.0000312	-0.689583619
ENSG00000187498	COL4A1	0.171857	0.246253	0.226726	0.450032	0.314712	0.395991	0.092174	0.145659	0.134229	0.000786051	0.996404979	0.093878763	-0.689340962
ENSG00000203872	C6orf163	0.822847	1.08708	1.332254	0.967249	0.95136	0.872522	0.803797	0.578468	0.420216	0.91974969	-0.050791573	0.078102232	-0.688697525
ENSG00000174718	KIAA1551	7.044772	7.842097	6.874159	5.831254	5.486422	5.647901	4.032242	4.500011	4.127523	0.013545312	-0.210725869	3.24E-13	-0.687870256
NewGene_167	--	3.379652	2.908102	2.666225	3.418874	2.698146	3.350596	1.926223	2.195898	2.486589	0.485747598	0.121783138	0.0000105	-0.687469722
ENSG00000154721	JAM2	0.532685	0.376639	0.519559	0.05453	0.107808	0.152098	0.186815	0.341065	0.299183	0.000000649	-2.029031871	0.063406024	-0.687234157
ENSG00000100916	BRMS1L	4.470956	4.263096	4.504835	4.046563	3.811784	3.661733	2.429139	2.850761	2.552968	0.640357447	-0.080218762	0.00000131	-0.6870738
ENSG00000139219	COL2A1	10.052119	8.131605	6.86207	6.387802	6.349665	6.393123	4.187482	4.584624	5.866388	0.058679897	-0.242222728	0.000000438	-0.686879808
ENSG00000019485	PRDM11	3.508274	2.848985	2.925774	2.800066	2.434049	2.387282	2.210038	1.92513	2.425625	0.476766194	-0.093830993	8E-10	-0.686866927
ENSG00000116353	MECR	6.601925	8.110372	6.988981	6.291411	4.567147	6.808072	4.341507	4.842493	4.048038	0.219679828	-0.18155609	0.000000552	-0.686679216
ENSG00000186871	ERCC6L	2.001893	1.932578	2.116614	1.650693	1.424363	1.38597	1.082158	1.239877	1.184638	0.072999776	-0.297931855	0.0000159	-0.686538332
ENSG00000165406	8-Mar	3.631227	3.727748	3.464004	3.555891	4.037877	3.869812	2.273894	1.841811	1.91004	0.036000128	0.23626096	1.49E-08	-0.68644548
ENSG00000129514	FOXA1	52.913409	53.321223	53.009597	50.762791	51.037229	50.063696	31.800832	28.220884	32.876257	0.214643132	0.077079195	6.24E-26	-0.686370631
ENSG00000078687	TNRC6C	2.736205	2.744143	2.773643	2.252438	2.030527	2.14704	1.941022	1.707568	1.529711	0.012282189	-0.239678389	0.00000373	-0.685836483
ENSG00000282034	AC106886.5	7.401146	7.873969	8.847736	4.866014	7.114816	10.600023	5.850865	2.873794	5.427451	0.925199945	0.05535509	0.044807637	-0.684825004
ENSG00000112137	PHACTR1	0.563878	0.367632	0.666084	0.279295	0.407171	0.490653	0.278716	0.45016	0.234457	0.387525469	-0.317580706	0.037262198	-0.684733937
ENSG00000175414	ARL10	0.83821	0.877159	1.214824	0.914733	0.671423	0.900217	0.558766	0.36284	0.545173	0.634597865	-0.132512133	0.005119669	-0.68407973
ENSG00000203499	IQANK1	1.725266	2.078189	1.932431	1.639132	1.321032	1.504629	1.323321	0.996185	1.022426	0.363222525	-0.209290856	0.001334692	-0.682897114
ENSG00000125398	SOX9	29.427537	29.02937	32.208192	18.711428	20.374667	18.564973	15.496488	16.364547	21.291605	6.41E-14	-0.506272036	4.64E-13	-0.68253467
ENSG00000183496	MEX3B	1.87994	1.373022	1.425654	0.670782	0.821407	0.743597	0.764868	0.709681	1.131203	0.0000115	-0.937754065	0.001518968	-0.682181321
ENSG00000154359	LONRF1	2.810533	2.624184	2.85984	3.984244	4.670613	4.359414	1.608107	1.786402	1.914585	1.25E-10	0.804221704	0.00000896	-0.682004922
NewGene_3710	--	1.25868	1.342576	1.496329	1.345324	0.963138	1.756281	0.959286	0.568355	1.226906	0.874684317	0.056825675	0.010347654	-0.680442
ENSG00000198934	MAGEE1	0.380428	0.281177	0.265127	0.27127	0.228036	0.338116	0.184189	0.145587	0.205241	0.995250116	0.004034683	0.131183573	-0.680161703
ENSG00000141086	CTRL	0.6285	0.616442	0.793696	1.10482	1.464434	1.307025	0.369588	0.410543	0.477241	0.022611421	0.773043593	0.117385862	-0.679972668

Gene ID	Gene name	Ctrl-1 FPKM	Ctrl-2 FPKM	Ctrl-3 FPKM	IFNγ-1 FPKM	IFNγ-2 FPKM	IFNγ-3 FPKM	Sora-1 FPKM	Sora-2 FPKM	Sora-3 FPKM	Ctrl vs IFNγ DESeq2 FDR	Ctrl vs IFNγ DESeq2 log2FC	Ctrl vs Sora DESeq2 FDR	Ctrl vs Sora DESeq2 log2FC
NewGene 3885	--	1.010483	0.93706	0.984903	0.714745	0.714643	0.597091	0.51279	0.58685	0.616424	0.245162531	-0.36303715	0.018178567	-0.679540862
ENSG00000105429	MEGF8	4.633174	4.329439	3.738208	4.130762	3.992937	3.83899	2.336002	2.638723	2.712789	0.69134097	0.049155507	2.78E-10	-0.679511619
ENSG00000248405	PRR5-ARHGAP8	2.190016	1.241905	1.772547	2.460611	0.815888	0.841108	0.872968	0.698923	1.492814	0.708556741	-0.197841239	0.049129754	-0.678891117
ENSG00000128617	OPN1SW	1.434912	1.633649	1.456605	1.185223	1.294324	1.272105	1.037393	0.741752	0.865026	0.791523326	-0.118048372	0.061255144	-0.678173948
ENSG00000115325	DOK1	1.395208	1.270917	1.473802	1.046635	1.041702	1.134031	0.908345	0.497875	1.041218	0.680172192	-0.13440931	0.015130557	-0.678027605
ENSG00000197937	ZNF347	1.345274	1.91349	2.165733	1.228779	1.010218	1.105185	0.81909	0.740946	1.178024	0.005842198	-0.497660638	0.0000783	-0.677067067
ENSG00000111846	GCNT2	21.886783	23.348419	25.707978	19.433127	20.486939	19.351852	13.184686	13.68766	14.761754	0.173003943	-0.113635014	9.66E-19	-0.676759224
ENSG00000112406	HECA	1.546893	1.666722	1.618229	1.206661	1.184832	1.422044	0.944198	0.874955	1.018009	0.275320213	-0.190907661	0.00000811	-0.676699742
ENSG00000155256	ZFYVE27	7.211936	7.073427	7.01335	6.41763	5.616063	6.455159	3.407896	4.471045	4.42415	0.689578493	-0.054365338	0.000000059	-0.676463305
ENSG00000175073	VCPIP1	1.974259	2.175175	2.422635	2.757535	2.597359	2.619685	1.244449	1.246236	1.365762	0.0000146	0.426594015	2.89E-09	-0.676283505
ENSG00000119523	ALG2	15.608957	14.827088	15.622197	20.556012	19.570368	21.327669	8.732359	8.272325	9.199703	4.31E-15	0.534997786	1.72E-16	-0.675929429
ENSG00000135917	SLC19A3	4.779167	5.577305	6.456841	2.902819	3.828254	3.160195	2.958993	3.15039	3.574886	0.00000418	-0.586264297	0.000000104	-0.675057234
NewGene 2134	--	0.586321	0.742748	0.914939	0.640692	0.722112	0.736105	0.433825	0.390915	0.498737	0.884511742	0.051917136	0.010938767	-0.6749633
ENSG00000143494	VASH2	5.122437	4.998828	5.49461	5.593014	5.701264	8.131911	4.484177	3.121212	3.07572	0.026647948	0.221424453	6.57E-08	-0.674642262
ENSG00000204519	ZNF551	3.41231	3.426278	3.067272	3.055431	2.844109	2.846681	1.925223	1.945802	1.943156	0.877692914	-0.02639142	4.87E-08	-0.674519006
ENSG00000189266	PNRC2	54.635484	57.046031	56.632671	40.657458	39.763801	39.697837	31.015275	33.563274	32.872691	4.38E-09	-0.318086117	2.49E-24	-0.674269551
ENSG00000141965	FEM1A	12.832758	12.44819	12.236259	11.366829	10.473381	11.607265	7.343718	7.064217	7.651699	0.839850264	-0.018296024	5.68E-28	-0.674013382
ENSG00000162614	NEXN	0.542263	0.960576	0.889668	0.462797	0.437022	0.516267	0.502354	0.571584	0.378467	0.043447996	-0.692895832	0.038951106	-0.67398921
ENSG00000171428	NAT1	2.555199	3.313458	2.911967	3.308601	2.588276	2.312729	1.88593	1.565941	1.664879	0.897697024	0.034683913	0.000539319	-0.673757972
ENSG00000132680	KHDC4	14.558214	16.162342	19.102918	15.925726	13.857783	13.527471	9.978582	11.18215	10.605903	0.923985945	-0.015799447	2.49E-10	-0.673499871
ENSG00000166747	AP1G1	14.628241	15.539075	14.929041	15.65136	15.565922	15.117341	9.212355	8.492239	9.412739	0.001263891	0.190782081	2.78E-25	-0.67306168
ENSG00000018869	ZNF582	3.28293	3.49765	2.432256	2.508613	2.338527	2.829536	1.571107	1.754274	1.723716	0.686674823	-0.102143143	0.000724871	-0.673059676
NewGene 4228	--	1.02716	0.779641	0.703305	0.93176	0.689561	0.925028	0.368098	0.456183	0.593558	0.748856144	0.146099988	0.085189006	-0.672698465
ENSG00000189241	TSPYL1	32.564686	32.343788	33.945633	30.322397	29.18651	29.636545	20.406914	18.850124	18.940123	0.984350144	-0.00185722	1.97E-22	-0.670797191
ENSG00000174796	THAP6	2.631134	3.951185	4.250936	4.869712	3.77089	3.689509	1.971727	1.764946	2.119509	0.324686148	0.201577903	0.000312774	-0.670502125
NewGene 4713	--	4.356175	4.165108	5.172119	4.9767	4.285995	4.927803	2.834	2.775251	3.798322	0.425781292	0.104420386	5.03E-09	-0.670368747
ENSG00000278129	ZNF8	5.083447	5.088348	5.254699	4.350718	4.113394	4.405511	3.230292	2.725776	3.147012	0.175330656	-0.113726603	1.05E-15	-0.670128077
ENSG00000109787	KLF3	26.829882	26.745028	28.773713	26.930632	27.462015	25.628413	22.828677	23.090073	23.127318	0.33459168	0.071835814	4.45E-25	-0.669311808
ENSG00000184992	BRI3BP	10.015343	11.917596	11.996839	9.533901	8.530169	9.866121	6.581217	5.924957	7.542855	0.17313768	-0.132829752	3.49E-13	-0.669297849
ENSG00000227051	C14orf132	4.301703	3.382004	3.654069	4.484568	5.218828	4.854152	2.11511	2.080561	2.512128	0.000000727	0.510566658	4.49E-09	-0.668705247
ENSG00000080200	CRYBG3	0.6261	0.771112	0.744469	0.884428	0.717342	0.908773	0.38183	0.404198	0.477285	0.021973661	0.378243567	0.000131964	-0.668387727
ENSG00000135482	ZC3H10	1.777086	1.540047	1.348963	1.419986	1.332988	1.330537	1.008988	0.826574	0.970393	0.984223267	0.004754817	0.000026	-0.668278845
ENSG00000007541	PIGQ	10.178937	9.754924	8.777231	8.353274	7.731176	9.632224	5.915818	5.220852	6.205794	0.982392413	-0.003842628	4.46E-10	-0.668022245
ENSG00000213967	ZNF726	3.012189	3.864043	3.190022	2.826216	1.713122	2.600288	2.165432	2.493386	1.978103	0.175046143	-0.313912376	0.001618098	-0.667920364
ENSG00000197147	LRRC8B	4.550313	5.811923	5.698031	4.699925	3.918528	4.0803	3.056856	2.969791	3.528491	0.071191138	-0.204293559	5.12E-10	-0.667892881
ENSG00000198464	ZNF480	6.548565	7.154695	6.947612	5.489344	5.23629	5.232941	4.175692	3.968786	4.051474	0.011727793	-0.224590673	2.23E-13	-0.667890316
ENSG00000277117	FP565260.3	0.689844	0.581299	0.494155	0.319946	0.353998	0.353822	0.547974	0.317508	0.16213	0.036643919	-0.738277765	0.119233116	-0.667761563
ENSG00000065325	GLP2R	1.361272	1.126449	1.221803	0.450949	0.715336	0.527608	0.661152	0.77021	0.628479	0.0000372	-1.060914587	0.002031006	-0.667567545
NewGene 848	--	1.466041	1.544076	1.529162	1.229343	1.311691	1.060413	1.141046	0.884007	0.911705	0.590143008	-0.143699898	0.002998361	-0.667030741
ENSG00000197808	ZNF461	1.253703	1.242447	1.48625	1.124557	1.219837	1.022367	0.720258	0.838475	0.74191	0.736016601	-0.104244411	0.007711367	-0.666765926
ENSG00000122870	BICC1	10.799759	11.378727	12.608414	5.776144	5.580514	5.616172	7.151034	6.081151	7.601743	4.51E-32	-0.888326288	1.68E-13	-0.666636469
ENSG00000143315	PIGM	3.781456	3.681263	3.675414	3.903659	3.853719	3.813217	2.063454	1.985054	2.547201	0.025745844	0.202081255	2.59E-10	-0.666452261
ENSG00000101115	SALL4	3.060756	2.844062	3.183396	2.711415	2.689788	2.542862	1.863131	1.60574	1.866377	0.839189393	-0.034511464	0.00000012	-0.66632496
ENSG00000107779	BMPR1A	5.664942	5.964373	5.791908	4.85327	4.520235	4.615087	3.137163	3.944058	3.449136	0.106634103	-0.128635565	1.31E-15	-0.666173584
ENSG00000180921	FAM83H	23.804171	23.227282	20.985014	20.631956	19.430891	22.378904	13.238845	11.348052	15.753791	0.819218635	0.024130268	1.88E-13	-0.665816727
ENSG00000255508	AP002990.1	27.174288	21.283646	13.832742	16.28544	13.309921	12.85777	11.341859	10.305039	15.348609	0.27615705	-0.407850137	0.060286604	-0.665291903
ENSG00000140265	ZSCAN29	9.649153	9.140428	8.831318	8.521405	9.378176	8.251721	6.407756	5.427026	6.832591	0.874684317	0.017899857	7.5E-15	-0.664159005
ENSG00000105137	SYDE1	4.36917	3.548393	4.33371	2.532824	1.965419	2.472667	2.300305	1.993313	2.950454	0.00000354	-0.657903689	0.0000176	-0.66402823
ENSG00000138604	GLCE	5.666516	5.543143	5.438462	5.204893	5.60902	5.617602	2.859063	3.352193	3.654589	0.159238251	0.141241881	2.47E-10	-0.6633015
ENSG00000106683	LIMK1	13.742182	13.924499	12.801568	14.301036	12.250173	13.10277	6.881969	7.395362	8.895239	0.20733768	0.108970296	1.39E-12	-0.663091165
ENSG00000179922	ZNF784	5.836481	4.124146	4.347766	3.569372	3.894107	4.503321	2.927957	2.442842	3.118717	0.703543164	-0.088456284	0.000164435	-0.662923703
ENSG00000273136	NBPF26	1.571237	2.341753	1.786244	1.946393	1.907564	1.828884	0.892423	0.890531	1.105198	0.73517571	-0.074828553	0.000150753	-0.662656854
ENSG00000197062	ZSCAN26	3.298807	3.826605	3.589511	2.580513	3.172928	2.641688	2.287864	2.055798	2.017193	0.403818302	-0.152855531	0.0000364	-0.662443378
ENSG00000113361	CDH6	6.866049	6.293804	6.432185	3.146977	2.505124	2.349985	4.800774	4.008956	4.785141	6.98E-46	-1.322023404	6.02E-16	-0.661395953
ENSG00000146834	MEPCE	22.719324	22.118775	22.724215	19.518443	19.25838	21.79413	13.356865	12.605875	14.577223	0.915061926	-0.012202189	2.3E-20	-0.661022452
ENSG00000162512	SDC3	6.300447	5.247063	4.618358	3.161875	2.759652	2.729624	2.849351	2.973114	3.728228	1.66E-11	-0.754915739	5.02E-08	-0.660709096
ENSG00000152661	GJA1	19.251821	21.574726	28.285392	10.489184	10.164991	10.181201	12.725753	13.321616	15.054632	2.33E-21	-1.006637302	0.000000015	-0.660347989
ENSG00000174173	TRMT10C	26.366175	28.711009	30.468788	25.405796	24.310926	26.454772	16.607374	16.655336	17.521108	0.870331558	-0.019467361	2.37E-15	-0.659513648
ENSG00000124762	CDKN1A	1.265664	0.853209	0.981024	0.682273	0.924891	0.942429	0.671891	0.53683	0.633596	0.706381791	-0.152182048	0.038022828	-0.658945517
NewGene 650	--	12.30242	9.871936	9.287077	4.511493	3.763345	4.219651	8.134503	5.23633	8.264598	1.77E-36	-1.12915231	4.43E-13	-0.658793933
NewGene 3708	--	2.118516	1.582312	2.14914	1.395567	1.525435	1.265223	1.299594	1.195015	1.252716	0.517902581	-0.200913643	0.010862021	-0.65773459
ENSG00000049239	H6PD	7.838086	6.884402	5.904639	8.489524	8.301909	8.926742	4.268889	3.428553	4.63213	0.000000423	0.450422768	3.64E-09	-0.657719263
ENSG00000189369	GSPT2	5.799059	4.911501	5.730588	5.378523	5.348959	5.554498	3.656634	3.292506	2.79729	0.333889598	0.13307527	0.00000469	-0.657633665
ENSG00000183837	PNMA3	9.570139	8.408923	7.671624	7.297407	7.134931	7.231435	5.072317	4.63049	5.446746	0.429343308	-0.095241926	3.18E-10	-0.65725499
NewGene 5319	--	0.778633	0.63444	0.74414	0.657375	0.60997	0.580561	0.344242	0.366531	0.562054	0.865635807	-0.07571494	0.066542001	-0.657203754
ENSG00000166436	TRIM66	0.805479	0.870116	0.951412	0.908486	0.721135	0.664869	0.576993	0.386695	0.507033	0.810146693	0.052026727	0.0000915	-0.657199913
NewGene 5987	--	1.33676	1.277994	1.009387	1.498945	1.691767	1.435356	0.685786	0.622011	0.858783	0.110059744	0.477368571	0.049968038	-0.6571591
ENSG00000183647	ZNF530	2.378027	1.961098	3.197337	1.968486	2.214071	2.287023	1.896621	1.60661	1.605409	0.930136318	-0.026271386	0.001967246	-0.657078513
ENSG00000182253	SYNM	4.117468	3.388455	4.305453	3.105137	2.736868	3.141332	2.133275	2.207075	2.749164	0.028594731	-0.24899544	5.1E-09	-0.656993287
NewGene 546	--	0.612393	0.565563	0.768437	0.345027	0.416888	0.269047	0.304393	0.424464	0.435552	0.076166888	-0.75753402	0.103515016	-0.656760783
ENSG00000092964	DPYSL2	4.283377	3.825367	4.096292	1.846424	2.112535	1.937826	2.493314	2.6002	2.165559	3.73E-15	-0.89430522	0.000000131	-0.656245367
NewGene 6225	--	2.3279	2.357233	2.439947	2.633079	2.846799	2.923662	1.274191	1.558298	1.557945	0.002319718	0.396861858	0.0000263	-0.656023468
ENSG00000168575	SLC20A2	45.380823	43.442547	43.427007	36.465297	39.150628	36.354321	25.793194	23.595284	24.848945	0.168169476	-0.09461093	8.95E-25	-0.655732367
ENSG00000082512	TRAF5	1.757627	1.797698	1.987317	3.268267	2.696635	2.783879	1.056121	1.022034	1.214981	2.09E-09	0.80584101	0.000116913	-0.655570099

Gene ID	Gene name	Ctrl-1 FPKM	Ctrl-2 FPKM	Ctrl-3 FPKM	IFNγ-1 FPKM	IFNγ-2 FPKM	IFNγ-3 FPKM	Sora-1 FPKM	Sora-2 FPKM	Sora-3 FPKM	Ctrl vs IFNγ DESeq2 FDR	Ctrl vs IFNγ DESeq2 log2FC	Ctrl vs Sora DESeq2 FDR	Ctrl vs Sora DESeq2 log2FC
ENSG00000171649	ZIK1	5.311228	5.372423	5.062133	3.921877	3.722642	3.533181	3.806772	3.343994	2.944821	0.001107327	-0.351948083	1.36E-08	-0.655496446
ENSG00000167981	ZNF597	1.799882	2.096174	1.980246	1.619828	1.491433	1.674439	1.088268	1.187024	1.215032	0.375659989	-0.145321903	0.00000607	-0.655469169
ENSG00000177000	MTHFR	5.772203	5.254409	4.744635	5.492527	5.164063	6.407151	3.137407	3.11015	3.687389	0.074332097	0.189609661	3.95E-09	-0.654524672
ENSG00000120875	DUSP4	0.616441	0.512652	0.57292	0.176771	0.246537	0.158648	0.256801	0.339332	0.41935	0.00000133	-1.391991326	0.017857203	-0.654477565
ENSG00000112308	C6orf62	42.864613	42.562634	45.627201	47.369267	46.683784	46.645739	25.806267	25.110765	27.194248	0.00000297	0.249189773	9.06E-28	-0.654442389
ENSG00000139926	FRMD6	9.452435	10.605957	11.039657	2.725361	3.925168	2.994652	5.61677	5.614115	6.707939	3.71E-39	-1.578629007	2.47E-11	-0.654145189
ENSG00000174106	LEMD3	14.276635	15.106694	14.709351	12.59841	11.526151	11.832607	8.552768	8.66423	9.059902	0.040239286	-0.147078917	1.58E-20	-0.653821358
ENSG00000180758	GPR157	1.582468	1.50624	1.561514	0.930877	1.219518	1.156621	0.967405	0.840954	0.954714	0.059421757	-0.338090946	0.0000386	-0.65363966
ENSG00000104369	JPH1	0.281565	0.380843	0.361216	0.251089	0.238763	0.271012	0.283809	0.10529	0.184518	0.644051845	-0.222534692	0.150751357	-0.653618512
ENSG00000138231	DBR1	14.518947	15.867797	14.68476	13.001433	11.296434	13.392418	8.102998	8.436038	10.370045	0.289185996	-0.110052367	1.81E-11	-0.653580665
ENSG00000188112	C6orf132	3.488461	4.081537	4.063008	2.775821	2.320326	2.920167	3.088336	2.832735	1.992274	0.008185299	-0.370512189	0.0000597	-0.653462
ENSG00000104643	MTMR9	2.051859	2.438958	2.387904	1.958639	1.809656	1.903118	1.298522	1.285776	1.512566	0.173003943	-0.179657703	0.000000124	-0.653425702
ENSG00000166450	PRTG	3.254308	3.052679	3.323483	2.039014	1.853026	2.057373	2.620924	2.149695	2.530424	8.81E-15	-0.707541189	3.44E-11	-0.653416985
ENSG00000181392	SYNE4	1.584632	1.403424	1.180286	1.012093	0.951285	1.078129	0.766189	0.786286	0.822667	0.476752642	-0.278602429	0.056869765	-0.653168482
ENSG00000186130	ZBTB6	4.943478	5.996395	6.297446	5.265168	5.465991	5.779496	3.537834	3.303848	3.425325	0.536988368	0.08760013	2.25E-08	-0.652932143
ENSG00000133121	STARD13	3.470996	3.811036	4.596208	2.024837	2.347086	2.154379	2.407255	2.107555	2.642063	4.39E-09	-0.714563933	0.000000171	-0.652382734
ENSG00000122641	INHBA	0.713427	0.622842	0.624034	0.669263	0.569796	0.504017	0.422995	0.344248	0.40803	0.966075753	-0.01526499	0.00871685	-0.652216117
ENSG00000184402	SS18L1	5.135012	6.133738	7.197545	5.459377	4.897792	4.952156	3.996889	3.202844	4.066288	0.355258734	-0.137521657	0.00000146	-0.651492045
ENSG00000134532	SOX5	2.32269	2.525166	2.798568	2.026851	2.692295	2.300484	1.911098	2.075912	1.326767	0.617386784	0.093878585	0.00000172	-0.65123916
ENSG00000084652	TXLNA	12.201225	12.21774	11.836008	13.52336	12.510471	13.267654	7.231197	6.46838	7.950651	0.0000226	0.273343683	1.64E-16	-0.650572245
ENSG00000183864	TOB2	8.693447	9.769038	10.788091	9.063062	9.205948	8.957081	5.633495	5.49407	6.332733	0.703178361	0.046114806	9.94E-12	-0.650401993
ENSG00000138185	ENTPD1	0.896633	0.97774	0.859977	0.925602	0.780713	0.613851	0.512656	0.481435	0.621208	0.802300032	-0.075614522	0.002825046	-0.649765749
ENSG00000180616	SSTR2	6.038313	6.61693	6.487398	5.282735	5.279418	4.771427	3.583742	3.738269	4.125906	0.04373781	-0.173212659	5.24E-15	-0.649640899
NewGene 282	--	0.596292	0.623778	0.544182	0.400592	0.275222	0.315858	0.581081	0.326738	0.340782	0.088080853	-0.642499019	0.071254563	-0.649476255
ENSG00000184898	RBM43	1.277241	1.118252	0.926615	1.881658	2.057157	2.25913	0.652973	0.727672	0.552025	2.22E-09	0.992328486	0.005326452	-0.649415209
NewGene 529	--	2.62933	3.27264	2.342777	2.560258	2.085368	2.168054	1.520172	1.422748	1.967231	0.986626376	-0.003822287	0.000068	-0.649389088
ENSG00000182700	IGIP	0.696374	0.365168	0.560248	0.585766	0.298508	0.535072	0.348784	0.290758	0.331421	0.930787487	-0.048981445	0.079968802	-0.649117527
ENSG00000009335	UBE3C	18.444683	18.491654	19.783536	18.222184	17.319416	16.694016	10.925953	10.791518	12.278956	0.754752285	0.027841669	4.5E-21	-0.648729094
ENSG00000144152	FBLN7	1.249684	0.955012	0.636968	0.579965	0.668231	0.58184	0.565322	0.55927	0.543893	0.381354774	-0.338912721	0.07085883	-0.648446921
ENSG00000124613	ZNF391	0.445612	0.760179	0.698322	0.699693	0.720739	0.568995	0.537853	0.438772	0.532849	0.475671909	0.246409743	0.481672997	-0.648323406
ENSG00000169641	LUZP1	3.864026	3.813606	3.884123	3.50519	3.272353	3.383878	2.386796	2.230677	2.331325	0.680498731	-0.045742722	2.33E-13	-0.648069788
ENSG00000197191	CYSRT1	2.922245	2.776802	2.970697	3.718648	2.794657	2.821002	1.762131	1.582482	1.848071	0.31381098	0.250224023	0.005375548	-0.647511246
ENSG00000135953	MFSD9	5.250976	5.149047	5.352606	5.517333	5.892218	6.00681	2.828986	2.978311	4.269647	0.002840943	0.303454981	1.16E-08	-0.647403521
ENSG00000169908	TM4SF1	5.334925	5.15206	4.822132	1.767171	2.63344	2.200329	3.285751	2.908417	2.71156	3.2E-09	-1.024153391	0.000109625	-0.647403145
ENSG00000160298	C21orf58	1.897411	2.186207	2.361091	1.5048	1.468683	1.353715	1.416963	1.52442	1.178731	0.124591635	-0.333373118	0.001766067	-0.646994063
ENSG00000185551	NR2F2	8.683529	8.864305	9.053827	10.725809	8.995893	11.14709	5.094261	4.959778	6.920068	0.010949676	0.206197387	1.86E-13	-0.646323424
ENSG00000119866	BCL11A	1.575567	1.844791	1.498192	1.403611	1.257111	1.019009	0.857498	1.269457	0.957297	0.063451504	-0.330444539	0.000313207	-0.646077755
NewGene 3299	--	7.908231	8.473419	7.415151	6.151755	6.115388	6.426745	5.110791	4.524733	4.932717	0.065577005	-0.172362091	1.02E-12	-0.645974093
ENSG00000125834	STK35	8.079591	7.91733	8.677774	7.464076	7.176826	6.99061	4.991713	4.31617	5.519223	0.664545939	-0.042952739	1.5E-13	-0.64594869
ENSG00000240891	PLCXD2	0.566745	0.606906	0.520161	0.41487	0.480343	0.50373	0.40798	0.368817	0.332736	0.885310407	-0.04631455	0.005368981	-0.645740683
ENSG00000133134	BEX2	13.030589	12.067671	10.577867	8.523335	8.99273	9.029927	6.832067	7.623985	6.784276	0.109493785	-0.258526474	0.0000346	-0.644452338
ENSG00000100075	SLC25A1	97.294941	92.946678	86.021462	106.138607	109.923939	113.434018	57.09339	54.999465	54.360309	5.44E-10	0.395239936	2.22E-19	-0.64436806
ENSG00000196387	ZNF140	10.590937	10.783763	11.620261	8.60297	8.041303	7.649048	5.756976	5.884781	6.923322	0.024752445	-0.218419845	1.11E-10	-0.644222931
ENSG00000196422	PPP1R26	3.948272	3.516056	3.305232	2.987277	3.153495	3.088715	2.067193	2.036187	2.36848	0.628127627	-0.072796602	0.000000076	-0.643992049
ENSG00000101670	LIPG	0.186103	0.218951	0.215266	0.114808	0.101959	0.112301	0.114042	0.152174	0.09906	0.11948575	-0.566694165	0.069746482	-0.64396364
ENSG00000253958	CLDN23	1.752288	1.798137	1.560143	2.558149	2.555694	2.888603	0.931324	1.028013	1.109437	0.0000131	0.796761456	0.00683833	-0.643884818
NewGene 5146	--	0.967426	1.043981	1.212088	1.053904	1.256729	1.450651	0.672372	0.626448	0.705436	0.033570834	0.49497085	0.017255754	-0.643636114
ENSG00000145388	METTL14	6.028782	5.897985	7.284093	4.945624	5.574492	5.360099	4.35781	3.80977	3.972738	0.232742924	-0.118985578	6.39E-12	-0.643431581
NewGene 3799	--	0.639172	0.380781	0.211461	0.376895	0.359493	0.329307	0.253932	0.121732	0.289573	0.864647521	0.096120823	0.184283426	-0.643307329
ENSG00000188811	NHLRC3	6.417974	5.483888	5.20739	5.933294	6.129089	5.70072	3.367475	3.053877	4.001777	0.106049772	0.192505029	0.000000331	-0.642867373
NewGene 540	--	0.740211	0.761471	0.718005	0.42536	0.284055	0.292637	0.323192	0.378337	0.622294	0.005087165	-1.133878372	0.105835154	-0.642391094
ENSG00000253304	TMEM200B	3.356299	3.683962	3.203797	2.232865	1.899846	2.499604	1.812851	2.225467	2.118257	0.004450634	-0.479675264	0.000098	-0.641966824
ENSG00000178826	TMEM139	3.225589	2.442571	2.375751	2.42108	2.2492	2.260458	1.740571	1.644768	1.324936	0.896378002	-0.034752397	0.001837659	-0.641922206
NewGene 3950	--	2.467265	2.178159	2.849474	3.14202	3.095751	2.973901	1.471215	1.550359	1.465648	0.038473807	0.44126493	0.007945846	-0.641665331
ENSG00000134698	AGO4	2.238829	2.161017	2.173338	1.713985	1.83423	1.575007	1.259385	1.134985	1.569036	0.100600211	-0.212827434	0.0000009	-0.641629201
ENSG00000178093	TSSK6	2.666041	2.368822	1.537656	1.950525	1.823986	1.814686	1.257954	1.393627	1.344728	0.771141659	-0.10558128	0.024310307	-0.641414381
NewGene 5441	--	1.132989	0.861606	0.688156	0.39113	0.228525	0.391645	0.709929	0.485689	0.387716	0.000784109	-1.143101632	0.056814382	-0.64131195
ENSG00000159915	ZNF233	1.465656	1.431211	1.425297	1.35135	1.456153	1.084522	0.957353	0.858667	0.733591	0.993484931	0.003186289	0.007318124	-0.641148487
ENSG00000114383	TUSC2	17.664422	16.632566	17.124113	15.090094	14.644815	13.937408	9.596638	11.605595	10.641475	0.446509019	-0.085009023	2.39E-09	-0.640746306
ENSG00000129003	VPS13C	6.460829	6.034317	8.13614	8.463309	8.351575	7.666285	4.124502	4.10796	5.513055	3.37E-17	0.573376651	1.25E-13	-0.640523701
NewGene 471	--	0.696274	0.693328	0.517651	0.355702	0.245965	0.358864	0.395912	0.503957	0.26651	0.007698607	-0.845593484	0.056383453	-0.639982789
ENSG00000224051	CPTP	11.258366	10.991129	10.201597	9.555684	8.983528	10.667321	5.638196	6.133119	7.663726	0.998597326	-0.000453362	8.58E-08	-0.639458311
NewGene 6274	--	0.83178	0.621622	0.580384	0.367608	0.412514	0.492961	0.367816	0.508864	0.338886	0.083056118	-0.519979767	0.032471459	-0.639394696
ENSG00000176422	SPRYD4	1.799513	1.670089	1.622613	1.643128	1.620211	1.573189	1.002703	1.050893	1.009826	0.593874974	0.072279329	2.79E-08	-0.638428622
ENSG00000256683	ZNF350	3.850443	3.873478	3.466066	3.193597	3.241701	3.149663	2.11342	2.020769	2.701626	0.638184924	-0.091624274	0.000260975	-0.638235141
ENSG00000255398	HCAR3	1.687069	1.543465	1.146882	1.109211	1.575845	1.033781	0.829777	1.020879	0.775133	0.817106916	-0.088873034	0.024218616	-0.63802443
ENSG00000152822	GRM1	0.264897	0.271914	0.305087	0.066156	0.049722	0.072314	0.200693	0.130743	0.177602	0.00000022	-1.975047337	0.059520092	-0.637729733
ENSG00000106415	GLCCI1	12.326465	13.705667	15.394814	9.355665	8.723915	9.362728	8.545886	7.150685	9.331847	0.000000131	-0.446091578	1.32E-10	-0.637675896
ENSG00000175189	INHBC	0.670088	0.704464	0.614398	0.81802	0.980099	0.9124	0.265063	0.345133	0.598535	0.083425197	0.589975752	0.146908411	-0.636643645
ENSG00000033011	ALG1	15.66084	14.460416	13.976632	11.74973	11.504353	11.450435	8.960385	8.410838	9.281252	0.042142971	-0.192649762	6.91E-12	-0.636293769
NewGene 2810	--	1.101644	1.364391	0.967025	1.671886	1.617255	1.70882	0.528975	0.600231	0.953706	0.003605953	0.685581241	0.046759693	-0.63627871
ENSG00000197969	VPS13A	5.894482	6.634581	6.862234	5.393813	4.462451	4.543886	4.472967	4.79021	4.568452	0.00000207	-0.321726156	1.13E-17	-0.635797622
ENSG00000248771	SMIM31	1.493192	1.272758	1.101901	0.508258	0.646793	0.731459	0.74099	0.815959	0.807206	0.0000137	-1.068940537	0.007433514	-0.635572863
ENSG00000091986	CCDC80	0.641987	0.671877	0.78802	0.222076	0.214429	0.228408	0.627607	0.457501	0.435915	3.12E-11	-1.173008991	0.000230807	-0.63521326

Gene ID	Gene name	Ctrl-1 FPKM	Ctrl-2 FPKM	Ctrl-3 FPKM	IFNγ-1 FPKM	IFNγ-2 FPKM	IFNγ-3 FPKM	Sora-1 FPKM	Sora-2 FPKM	Sora-3 FPKM	Ctrl vs IFNγ DESeq2 FDR	Ctrl vs IFNγ DESeq2 log2FC	Ctrl vs Sora DESeq2 FDR	Ctrl vs Sora DESeq2 log2FC
ENSG00000197056	ZMYM1	3.506919	4.415997	4.139341	3.639492	3.362993	2.963882	2.744901	1.988973	2.545208	0.416500249	-0.126687715	0.00000697	-0.635000438
ENSG00000138463	DIRC2	3.868489	3.627375	4.257363	3.836037	2.878747	3.493567	2.377873	2.219733	2.503175	0.761503002	-0.057025163	0.000000818	-0.634887267
ENSG00000188613	NANOS1	1.427078	1.451857	1.669264	0.485816	0.607777	0.600028	0.834842	0.763458	1.128967	1.04E-10	-1.337726529	0.003275103	-0.634725903
ENSG00000151233	GXYLT1	6.462273	7.588875	7.466081	5.378416	4.801547	5.151349	4.652949	3.926976	4.576733	0.0000368	-0.343432582	1.89E-12	-0.633283258
ENSG00000185127	C6orf120	17.665352	17.479441	16.313984	16.010376	16.669886	15.813075	10.534301	9.542358	11.080709	0.530956895	0.061511872	1.64E-14	-0.633046698
ENSG00000064042	LIMCH1	0.567158	0.399683	0.507093	0.410742	0.433702	0.459877	0.286404	0.323845	0.262067	0.916020296	-0.040911376	0.025926024	-0.632862971
ENSG00000117707	PROX1	28.979919	28.31971	30.954454	29.062201	26.278491	26.690653	19.616833	15.57949	18.694114	0.398128125	0.072271722	1.24E-16	-0.631781235
NewGene_4461	--	2.292211	2.761272	3.549482	2.413692	2.824341	2.089745	2.056254	1.53636	1.613039	0.768046031	-0.082573219	0.002981057	-0.631572625
ENSG00000085788	DDHD2	8.48452	9.990338	10.415831	9.347399	8.159044	7.843254	6.5256	5.316089	5.780179	0.607793847	-0.064691445	1.25E-10	-0.631267197
ENSG00000065559	MAP2K4	14.372227	15.480602	14.597936	14.864568	15.202085	14.295479	9.118683	8.732453	9.134845	0.04493453	0.151102941	1.02E-16	-0.630877569
ENSG00000101236	RNF24	1.874367	1.849936	2.030949	3.36915	3.346881	3.730914	1.125213	1.148232	1.27636	1.49E-23	0.983059319	0.0000247	-0.630821288
ENSG00000134324	LPIN1	1.298941	0.95416	1.082075	2.122033	2.8513	2.84544	0.679023	0.613372	0.769318	6.02E-21	1.384900374	0.001539241	-0.630740349
ENSG00000169375	SIN3A	14.12977	15.093938	15.594142	15.228769	14.539402	14.3307	9.623637	8.393714	9.652115	0.0108024	0.159812216	1.84E-21	-0.630593839
NewGene_1043	--	1.813559	1.613478	1.411481	1.717669	1.437801	1.540864	0.95105	1.092875	0.877787	0.732050855	0.103015237	0.012521288	-0.630508047
ENSG00000167191	GPRC5B	2.207569	1.912512	2.128441	1.373887	1.5955	1.527002	1.068013	1.469028	1.234643	0.026655316	-0.335752006	0.000115012	-0.630319081
ENSG00000131389	SLC6A6	21.481584	20.231492	22.19336	20.860792	21.683911	20.521585	13.059903	12.896853	15.010051	0.072098255	0.122450097	4.12E-20	-0.630091488
ENSG00000132294	EFR3A	6.501195	6.736172	7.159509	11.326901	10.53688	9.991729	4.141074	3.977344	4.234524	1.62E-27	0.788759076	3.56E-13	-0.630058785
ENSG00000170522	ELOVL6	10.040999	9.250196	8.355048	10.251484	10.689516	11.333838	6.235287	6.15071	6.545617	0.00000345	0.344779657	2.41E-13	-0.629427799
ENSG00000132434	LANCL2	13.353335	11.572962	11.793287	10.149776	10.210489	10.411323	7.538455	7.175196	7.546502	0.24993249	-0.108504374	9.97E-14	-0.629404658
ENSG00000196466	ZNF799	5.295075	4.616131	4.911766	3.236952	3.491783	4.307083	2.959568	2.717917	3.33395	0.073907051	-0.279452418	0.00000322	-0.628770932
ENSG00000158792	SPATA2L	5.368871	5.130667	3.968938	4.350477	4.234243	4.557096	3.254025	2.654727	2.846686	0.92320945	0.021954854	0.0000764	-0.628462525
ENSG00000166971	AKTIP	3.445552	2.928597	3.270132	3.918197	3.007923	3.349346	2.081312	1.790627	1.861941	0.187301258	0.232583768	0.000391781	-0.627953209
ENSG00000116731	PRDM2	3.538596	3.884644	3.66985	3.183398	2.986941	3.068478	2.375352	2.123009	2.323123	0.350687823	-0.105143413	7.7E-09	-0.627579728
NewGene_6041	--	0.655606	0.71044	0.900744	0.450732	0.710894	0.410256	0.519087	0.460707	0.371527	0.441161726	-0.377500404	0.13441229	-0.627550867
ENSG00000111249	CUX2	1.497391	1.248809	1.645615	0.883815	0.655437	0.808156	0.965411	0.69564	1.013008	0.00000584	-0.754229978	0.000308549	-0.62732208
ENSG00000083812	ZNF324	2.667854	2.265865	2.389026	2.129057	2.459282	2.203869	1.747599	1.254981	1.378	0.581657825	0.098477015	0.00027218	-0.627276631
ENSG00000172817	CYP7B1	2.056049	1.912001	2.251952	1.770458	1.539592	1.540859	1.077146	1.304923	1.386589	0.384095841	-0.211425359	0.004286772	-0.627272942
ENSG00000254470	AP5B1	7.951815	7.187302	6.464858	7.228913	6.619493	7.557685	4.143343	3.865312	5.153581	0.233336725	0.133587228	2.19E-08	-0.626976253
ENSG00000117877	CD3EAP	3.706094	5.095448	5.533317	3.434788	2.100518	3.279878	2.715289	3.083061	3.077251	0.006287617	-0.501030692	0.0000336	-0.626761601
ENSG00000165821	SALL2	15.81876	13.609496	13.789844	9.747806	9.928746	10.142219	8.785683	8.550327	9.014535	5.14E-08	-0.432877961	1.13E-14	-0.626741774
ENSG00000100664	EIF5	59.370824	64.572701	61.487953	57.985048	57.931777	54.616706	43.673311	38.216009	40.222378	0.402846385	0.048496559	2.64E-27	-0.626517217
ENSG00000146587	RBAK	6.123453	6.384988	6.750982	6.671812	6.931039	6.144327	4.224126	3.429145	4.112867	0.039840223	0.182263314	2.01E-11	-0.626296402
ENSG00000129317	PUS7L	2.939434	3.472166	3.55752	3.166854	3.022017	3.191549	2.2778	1.885512	2.048846	0.319250041	0.112455313	1.04E-09	-0.626057777
ENSG00000174652	ZNF266	10.816275	10.518398	11.928871	9.789634	8.347564	8.739353	7.080376	6.296513	6.395849	0.090650062	-0.1568948	5.44E-12	-0.625607273
ENSG00000147118	ZNF182	2.316609	2.305055	2.325221	1.875678	1.799177	1.796003	1.496572	1.081738	1.667428	0.272992871	-0.190039956	0.000434948	-0.625518259
ENSG00000198938	MT-CO3	9275.969727	9468.851562	9165.152344	8635.47168	9504.040039	8988.535156	5527.27002	6362.810547	5016.87207	0.076840674	0.107152598	1.11E-12	-0.625505215
NewGene_1885	--	1.197308	1.197387	0.875757	1.055522	1.02187	1.485666	0.612566	0.645644	0.850451	0.72544502	0.13331706	0.051772662	-0.624749909
ENSG00000196812	ZSCAN16	14.010039	12.95691	12.009368	10.676335	10.616657	10.188528	7.503661	7.941445	8.236335	0.21134356	-0.161878621	0.000000174	-0.624694509
ENSG00000215012	RTL10	3.466167	3.413521	2.84745	3.474689	3.141624	3.263714	2.073673	1.839592	2.040028	0.219050452	0.149635656	0.000000069	-0.624693355
NewGene_6606	--	7.31365	9.80782	7.474889	6.577982	6.425914	7.496322	5.428541	4.911885	4.547036	0.647793264	-0.109773774	0.00132849	-0.624503081
ENSG00000186026	ZNF284	1.262383	1.330415	1.508522	1.113396	1.254469	1.014958	0.903772	0.809303	0.777051	0.58480384	-0.129530866	0.001202458	-0.624292933
ENSG00000110367	DDX6	19.303602	23.919474	19.566992	20.116003	19.172139	20.594836	12.296251	12.318676	11.867665	0.09302372	0.112026184	1.36E-18	-0.623244288
ENSG00000275835	TUBGCP5	8.553937	9.744461	10.006043	8.639284	7.401916	8.081219	6.055134	5.221577	5.909906	0.364798207	-0.09966707	7.08E-11	-0.622727112
ENSG00000125046	SSUH2	1.948714	1.026768	0.593298	0.611621	0.405551	0.510835	0.79492	0.795147	0.504204	0.012133444	-1.076747658	0.14586904	-0.622158501
NewGene_3316	--	1.510588	1.941236	1.745078	2.312167	1.822879	1.792433	1.080327	1.266346	1.039493	0.015924639	0.418236207	0.002522361	-0.621929064
ENSG00000115594	IL1R1	7.521718	7.207527	7.220591	6.966977	7.168181	6.462124	4.006864	4.577863	4.887075	0.914056357	0.01475483	4E-11	-0.621271887
ENSG00000204130	RUFY2	1.473714	1.966047	2.081876	1.446942	1.175003	1.560245	1.351291	1.162507	1.081883	0.405880822	-0.482372997	0.001195036	-0.621032012
ENSG00000166704	ZNF606	5.904191	6.715192	5.932795	5.561603	5.075183	5.947218	3.685625	3.134131	3.159666	0.414134795	-0.100728991	0.000000118	-0.620660608
ENSG00000115020	PIKFYVE	5.172255	5.424768	5.708847	7.383762	7.229235	6.644045	3.225925	2.910162	4.126738	1.03E-13	0.510425411	7.15E-10	-0.620513538
ENSG00000167202	TBC1D2B	1.927224	1.735263	1.907053	3.91149	3.897699	3.57453	1.026187	1.036591	1.344035	5.56E-32	1.175368997	0.0000277	-0.620078557
ENSG00000135447	PPP1R1A	1.513881	1.522044	1.008461	1.862585	1.410557	1.657158	0.918236	0.870586	0.627306	0.143275296	0.426163995	0.054593252	-0.619996892
ENSG00000187187	ZNF546	0.319886	0.335316	0.4825	0.433564	0.35719	0.528969	0.298056	0.254539	0.45669	0.572346807	0.179661415	0.025255339	-0.619735248
ENSG00000100890	KIAA0391	16.541362	16.366545	15.650514	8.277448	12.513319	13.497409	3.85718	14.199482	11.865199	0.000098	-0.448570215	0.438752312	-0.619630545
ENSG00000163807	KIAA1143	7.629936	8.648236	8.22131	6.589566	6.354368	6.658162	5.145894	4.591956	5.227	0.04804836	-0.17346053	1.43E-12	-0.619049888
ENSG00000035681	NSMAF	5.444551	5.561837	5.438581	5.012074	5.310451	5.318684	3.55678	3.078423	3.234866	0.496333684	0.086520855	3.41E-08	-0.618443075
ENSG00000106266	SNX8	10.483057	10.1344	9.104518	8.393681	7.824149	8.791311	6.059296	5.488538	6.614915	0.246752874	-0.114273454	3.19E-12	-0.618409547
ENSG00000166068	SPRED1	4.770475	5.116692	5.824825	3.340883	3.035336	3.055904	3.282801	2.895496	3.454873	9.42E-11	-0.588814803	3.01E-10	-0.617620512
NewGene_2937	--	1.917378	1.776925	2.326227	2.360051	1.849172	1.773512	1.278577	1.151362	0.943283	0.552246723	0.188024303	0.031044924	-0.617575251
ENSG00000112337	SLC17A2	2.021281	2.359626	1.450731	1.462611	1.309419	1.144095	1.323983	1.150722	0.94752	0.140499544	-0.380999485	0.012780682	-0.617487874
ENSG00000178718	RPP25	0.645438	0.897965	0.898383	0.539413	0.617576	0.55207	0.5633	0.475557	0.440299	0.281047245	-0.357246732	0.044551642	-0.616938712
ENSG00000163348	PYGO2	29.612067	28.337094	27.122343	25.078069	24.612828	23.735965	16.571095	17.355175	18.291494	0.413123558	-0.06478231	2.12E-18	-0.61693114
ENSG00000151612	ZNF827	0.921158	1.127717	1.142915	1.423974	1.163268	1.188173	0.667724	0.810565	0.616712	0.015359385	0.378233242	0.000432662	-0.616664445
ENSG00000173120	KDM2A	49.537285	46.236342	46.411064	45.155435	43.376037	45.088914	31.28853	29.19336	34.500503	0.424171694	0.052403821	1.17E-20	-0.615987984
ENSG00000183840	GPR39	0.907896	1.164807	0.916627	1.163953	0.903758	0.956426	0.74442	0.476333	0.610508	0.626047589	0.159334769	0.035830899	-0.615926427
ENSG00000145217	SLC26A1	14.855894	14.275029	10.250679	16.703931	17.695026	17.619644	8.39173	7.283712	8.626171	0.000000864	0.547199835	0.000000739	-0.615832983
ENSG00000135503	ACVR1B	8.300233	7.840156	8.079275	9.499663	8.866426	8.589537	5.057483	4.752489	5.088355	0.0000157	0.324363697	5.03E-13	-0.615322879
NewGene_1794	--	0.368261	0.545524	0.76825	0.243418	0.271743	0.217454	0.370662	0.237119	0.411433	0.002924626	-0.898817286	0.042743781	-0.614941382
ENSG00000175691	ZNF77	4.515187	5.165947	5.191213	4.564327	5.043807	4.863273	2.817571	3.015153	3.26983	0.529989048	0.109910413	0.0000435	-0.614740452
ENSG00000166783	MARF1	8.668435	8.734776	8.522469	7.979382	8.597829	8.025604	5.781735	5.184014	5.791808	0.492548336	0.060100508	2.88E-12	-0.614671287
ENSG00000111912	NCOA7	4.126319	5.520254	5.590531	6.913828	7.999288	9.076139	2.517599	2.348588	3.768089	6.58E-13	0.720087728	0.00000168	-0.614563687
ENSG00000196083	IL1RAP	23.810379	24.528344	24.312076	18.189385	16.814386	16.734607	16.105455	14.785549	15.971574	2.67E-09	-0.344727547	5.04E-23	-0.614301925
ENSG00000159164	SV2A	3.16984	3.158302	2.822677	2.801105	2.847896	3.117674	1.86893	1.892872	1.742907	0.521212162	0.099186176	0.00000366	-0.613819801
ENSG00000180354	MTURN	0.374215	0.452518	0.462763	0.374756	0.550551	0.503799	0.294424	0.377058	0.328903	0.956357735	-0.022550318	0.036063421	-0.613375048
ENSG00000157240	FZD1	1.085458	1.106099	0.829788	0.974638	0.830013	0.838743	0.610096	0.68775	0.545384	0.854804261	-0.047302463	0.00181529	-0.613162398

Gene ID	Gene name	Ctrl-1 FPKM	Ctrl-2 FPKM	Ctrl-3 FPKM	IFNγ-1 FPKM	IFNγ-2 FPKM	IFNγ-3 FPKM	Sora-1 FPKM	Sora-2 FPKM	Sora-3 FPKM	Ctrl vs IFNγ DESeq2 FDR	Ctrl vs IFNγ DESeq2 log2FC	Ctrl vs Sora DESeq2 FDR	Ctrl vs Sora DESeq2 log2FC
ENSG00000126003	PLAGL2	19.237427	18.929663	19.937037	20.474602	19.387962	19.828291	11.850471	11.230094	12.592045	0.001407224	0.185952547	7.2E-22	-0.612279017
ENSG00000183826	BTBD9	0.951808	0.934002	0.86152	1.570067	0.874693	0.930156	0.58358	0.47948	0.633505	0.557019239	0.118001734	0.000605183	-0.612275778
ENSG00000139178	C1RL	13.753973	12.961232	12.669929	19.119949	20.10949	20.18939	7.604014	7.373934	8.118348	4.87E-25	0.733364566	4.12E-13	-0.611471931
ENSG00000167395	ZNF646	5.513858	4.959911	4.265509	4.725584	4.708123	5.159361	2.998992	2.781452	3.263485	0.280239793	0.121582457	2.28E-09	-0.611389594
ENSG00000111348	ARHGDIB	1.344607	1.749655	1.728253	0.177324	0.192739	0.207468	0.841448	1.055593	0.7779	5.16E-09	-2.576094082	0.084407511	-0.610959851
ENSG00000116729	WLS	5.241535	4.590114	5.031836	3.608278	3.814799	3.627663	2.844962	3.480091	2.82121	0.021223361	-0.304522321	0.0000408	-0.610938468
ENSG00000136492	BRIP1	6.320507	7.769764	7.643272	7.270364	6.115971	6.725259	5.336106	4.097266	5.305757	0.756023487	0.044821526	0.000000016	-0.610839024
ENSG00000126733	DACH2	0.928444	1.009274	0.856919	0.40828	0.275571	0.382514	0.640189	0.646102	0.565121	6.96E-08	-1.329848762	0.008565373	-0.610824362
ENSG00000198393	ZNF26	3.267999	3.293156	2.7407	2.965222	2.798638	2.645319	2.17551	2.031474	2.587645	0.0537467	-0.201023009	1.13E-09	-0.610426159
ENSG00000151553	FAM160B1	2.940253	3.141984	3.259173	4.637725	4.422177	3.918133	2.231421	1.843547	1.716486	3.01E-10	0.61571879	0.00000405	-0.610249069
ENSG00000168421	RHOH	2.291075	1.824389	1.146529	0.780235	0.571643	0.591536	0.89797	0.818544	1.793627	0.000183703	-1.066224044	0.042634225	-0.610155722
ENSG00000167767	KRT80	0.321217	0.413248	0.58127	0.053749	0.046823	0.028657	0.284623	0.190529	0.336586	8.09E-10	-3.081161659	0.123823531	-0.609780024
NewGene_1488	--	43.825998	43.994288	41.128911	41.128654	37.780426	38.361949	26.851046	25.264701	28.333482	0.91968243	-0.008242447	5.57E-28	-0.60914717
ENSG00000160877	NACC1	31.063559	32.626322	28.398619	27.440565	25.984835	26.952407	18.839071	18.345421	20.968357	0.395407934	-0.06395184	6.11E-20	-0.609048604
ENSG00000169245	CXCL10	1.668032	1.450513	1.314949	298.656738	271.940308	280.567139	1.180945	0.562177	0.989222	0	7.717996126	0.111947483	-0.608953303
NewGene_6001	--	2.428549	3.490744	4.048843	2.794098	2.381324	2.787759	1.998169	1.634721	2.461905	0.623313618	-0.169203041	0.036889878	-0.608347912
ENSG00000148468	FAM171A1	8.424879	7.640485	7.840818	4.49842	4.221291	4.668905	4.805003	4.462169	5.549838	5.73E-15	-0.69408421	5.49E-10	-0.60758951
ENSG00000155287	SLC25A28	6.04183	6.94697	5.535212	11.982941	11.254807	12.152315	3.878457	3.800392	3.775452	4.67E-20	1.072854278	0.00016123	-0.607381605
ENSG00000170954	ZNF415	1.133549	1.158642	0.981478	0.736764	0.840368	0.825259	0.497476	0.938503	0.385329	0.43925095	-0.255383809	0.087697733	-0.607081953
ENSG00000167553	TUBA1C	169.155048	167.710898	159.665549	123.446625	115.221752	120.540457	96.400245	108.124099	101.374291	1.82E-11	-0.32272512	3.98E-19	-0.606530556
NewGene_5975	--	17.531574	18.167992	17.66885	18.781557	16.839266	16.448303	11.327475	10.575387	11.391678	0.031769856	0.134704405	6.8E-24	-0.606388737
ENSG00000129007	CALML4	5.150581	5.12113	5.913191	3.502912	4.390755	4.542789	4.109456	3.021155	2.273481	0.104430458	-0.327048142	0.005466921	-0.60633968
ENSG00000198324	PHETA1	5.921744	4.905704	4.50776	3.830128	4.566572	4.09405	2.977204	2.920511	3.074464	0.356413052	-0.130734659	0.000000672	-0.606210672
ENSG00000181284	TMEM102	3.211699	2.941738	2.350668	2.56527	2.362022	2.572468	1.605372	1.57168	2.083114	0.902295008	-0.034707499	0.004123518	-0.606202581
ENSG00000175066	GK5	1.740301	1.805407	1.803907	1.857953	1.312819	1.612153	1.105612	1.002892	1.249679	0.705790194	-0.064534807	0.00000573	-0.60602844
NewGene_1254	--	12.020528	15.119343	15.730834	12.089651	11.423427	11.580655	8.348057	9.460881	10.604631	0.272147339	-0.108535397	4.53E-10	-0.605962624
ENSG00000186376	ZNF75D	1.29702	1.367544	1.159439	1.495944	1.301113	1.330205	0.788998	0.999487	0.64054	0.139298945	0.253728063	0.002053234	-0.605831909
NewGene_3623	--	2.261032	2.361999	1.761598	1.947385	1.625142	1.480022	1.31717	1.124329	1.497772	0.524750223	-0.191985049	0.017270227	-0.605767401
ENSG00000003137	CYP26B1	11.742648	11.715193	10.638722	12.152103	11.444393	12.229714	6.057082	6.733618	7.626448	0.001119222	0.258252201	1.31E-10	-0.605267393
ENSG00000099260	PALMD	7.369331	6.670166	7.245766	3.909075	3.319752	3.461728	4.645983	4.431711	4.009148	1.32E-13	-0.875723615	0.000000738	-0.605229621
ENSG00000008441	NFIX	4.437175	3.648656	3.164167	5.80557	5.442587	5.12405	2.369775	1.926621	2.509917	7.87E-18	0.814143307	0.00000358	-0.604794332
ENSG00000186222	BLOC1S4	13.967591	11.873324	11.496302	11.988334	12.894341	13.740775	7.246295	7.725713	8.043658	0.118198779	0.196577721	0.000000367	-0.604706411
ENSG00000102317	RBM3	84.574681	98.912895	101.735544	96.961192	83.811444	92.504995	61.599711	56.61926	57.162212	0.314470383	0.089246841	3.08E-16	-0.604270054
ENSG00000100281	HMGXB4	12.777764	12.776786	13.399548	11.530371	10.106635	11.23269	8.168405	7.43793	7.850299	0.333444279	-0.088260879	3.5E-14	-0.60387086
NewGene_3199	--	0.423507	0.602058	0.659858	0.304678	0.407321	0.355197	0.323632	0.440928	0.47001	0.122175522	-0.616068661	0.112895567	-0.603850464
ENSG00000198515	CNGA1	0.751927	0.440897	0.369944	0.313663	0.565944	0.243474	0.462135	0.45621	0.110781	0.674800863	-0.222232416	0.223764004	-0.603309559
NewGene_1453	--	0.878595	0.94644	0.88225	0.789644	0.777324	1.136989	0.594765	0.491447	0.591806	0.742895473	0.147631668	0.102586808	-0.603069496
ENSG00000157657	ZNF618	3.58634	2.625677	2.226754	3.36416	3.050993	3.207457	1.593113	1.154968	1.960703	2.01E-09	0.546245434	0.00000639	-0.603019668
ENSG00000134250	NOTCH2	2.02605	1.81371	2.101737	1.547406	1.190463	1.389678	1.22685	1.150459	1.352073	0.001254049	-0.385531565	0.000000037	-0.602968835
NewGene_2173	--	0.455771	0.52251	0.306066	0.29015	0.600619	0.530826	0.317034	0.253672	0.237872	0.876275612	0.089180228	0.167358637	-0.602680877
NewGene_3764	--	0.957893	0.794218	0.675757	1.039073	0.788601	0.672117	0.635357	0.258631	0.66586	0.635898411	0.168523222	0.073296491	-0.601865468
ENSG00000127863	TNFRSF19	2.069715	1.922792	2.289084	1.37714	1.298988	1.513012	1.202565	1.352126	1.296401	0.006718673	-0.444614792	0.000183838	-0.601657946
ENSG00000183853	KIRREL1	6.768928	6.819324	6.579	2.595848	2.33261	2.56519	3.925681	4.010664	3.94851	7.6E-49	-1.266839345	5.24E-12	-0.601316202
ENSG00000152443	ZNF776	5.764957	6.417574	6.543834	5.692668	5.183031	5.405316	3.906358	3.593826	4.219574	0.618848284	-0.058279727	1.76E-10	-0.601034135
NewGene_4030	--	0.842143	1.110828	0.656478	0.678291	0.763029	0.783309	0.57508	0.564083	0.464587	0.824598021	-0.077730788	0.026833313	-0.601019925
ENSG00000108256	NUFIP2	13.660411	15.318047	16.572575	15.888897	14.753032	14.953392	9.783477	8.872579	9.582304	0.059109657	0.147829444	8.02E-15	-0.599718273
ENSG00000118058	KMT2A	6.294838	6.465776	6.935773	6.645462	6.487534	6.274798	4.503033	3.960716	4.358732	0.008571495	0.164609435	1.05E-17	-0.599687512
ENSG00000132326	PER2	1.131824	1.391065	1.260169	1.535135	1.323557	1.293778	0.90866	0.564536	0.875781	0.087957494	0.28006896	0.001969502	-0.599586016
ENSG00000121104	FAM117A	7.558812	8.412917	7.59769	8.237987	8.693177	8.388671	4.351567	4.627838	5.621488	0.020557522	0.248972494	0.000000821	-0.599267277
NewGene_5193	--	1.339856	2.05869	1.32011	1.570293	1.322451	1.41168	0.855947	0.899675	1.072562	0.695054822	0.12731899	0.030997516	-0.599144666
ENSG00000064703	DDX20	9.294448	8.619473	8.917522	9.301227	9.185859	8.922529	6.077907	4.932526	5.523454	0.03266075	0.177480138	2.83E-10	-0.598915712
ENSG00000187764	SEMA4D	3.612574	3.506159	3.503419	4.747022	5.150278	4.341029	2.0504	1.861895	2.1256	0.000000227	0.542290799	0.0000023	-0.598102419
NewGene_5551	--	0.54426	0.619307	0.561739	0.382967	0.438352	0.571433	0.423754	0.341836	0.297649	0.634870881	-0.194037498	0.072038095	-0.597869244
NewGene_382	--	2.859556	2.980455	2.368122	0.91948	1.256968	0.929377	1.783814	1.758437	1.46081	0.000000696	-1.224416511	0.009075317	-0.597357706
ENSG00000134253	TRIM45	1.170767	1.001847	1.217914	0.885513	0.688455	0.689957	0.676474	0.704523	0.733804	0.079352275	-0.435537149	0.009639105	-0.596975634
ENSG00000243364	EFNA4	15.626186	14.109459	12.604428	11.031774	8.160481	9.579891	7.557163	9.361257	8.919542	0.002592439	-0.41387806	0.0000148	-0.596312972
ENSG00000166619	BLCAP	8.894046	10.330821	10.011851	8.764391	8.847106	8.14123	5.566755	5.862798	8.033962	0.649212013	-0.065681407	0.00000324	-0.595336699
ENSG00000120937	NPPB	3.968226	3.526409	3.824257	0.26312	0.14426	0.235933	1.803306	2.607031	2.595954	2.43E-18	-3.921417532	0.04269085	-0.59533212
ENSG00000184083	FAM120C	0.572799	0.855839	0.763945	0.663887	0.545547	0.686063	0.461195	0.287282	0.289092	0.927110729	-0.034694346	0.02775965	-0.594687534
ENSG00000125741	OPA3	5.716134	5.465794	5.266496	4.839133	5.126229	5.265644	3.426391	3.597662	3.684291	0.907003281	-0.016812592	6.69E-10	-0.594402597
ENSG00000160352	ZNF714	3.419199	4.381302	4.295662	3.784737	3.43275	3.773335	2.737628	2.568905	2.754596	0.964700899	-0.008342956	0.00000184	-0.594140553
ENSG00000187164	SHTN1	7.340985	7.199785	8.196128	7.25065	5.597564	6.127387	4.809212	4.452533	5.149357	0.454644015	-0.088748135	3.41E-11	-0.594101691
ENSG00000083093	PALB2	12.890601	12.924443	12.482992	12.326819	12.016045	11.799943	8.754661	7.276768	7.912816	0.484637755	0.063056796	1.06E-12	-0.594097932
NewGene_5749	--	5.051809	6.016091	5.48818	5.024451	5.505245	5.589095	3.967683	3.607155	3.49416	0.642427547	-0.060350181	3.21E-10	-0.593800242
ENSG00000001631	KRIT1	12.23584	8.797427	10.900632	12.536016	8.744513	11.421824	7.673311	7.527199	8.071697	0.803385483	0.031131292	7.32E-11	-0.592964434
ENSG00000154309	DISP1	0.737098	0.734315	0.796009	0.746894	0.87118	0.742348	0.370771	0.491726	0.547783	0.431311469	0.202446172	0.017876308	-0.592823836
ENSG00000164603	BMT2	1.412481	1.233785	1.267776	1.322848	1.238532	1.350433	1.09105	0.584858	0.758044	0.507547427	0.148421626	0.013686368	-0.59268878
ENSG00000114554	PLXNA1	12.26697	11.868036	11.36323	7.61606	6.952381	7.613459	6.767161	6.686061	8.687981	7.36E-18	-0.531200616	5.56E-12	-0.592310297
NewGene_1682	--	0.96408	0.427553	0.675877	0.937344	0.411128	0.38185	0.67905	0.308989	0.286121	0.405571402	-0.493734587	0.121529847	-0.592065601
ENSG00000132646	PCNA	192.362781	196.560013	200.025876	179.710532	175.395229	180.984969	118.070914	118.629613	129.982753	0.877161766	0.011591189	1.09E-25	-0.591277771
ENSG00000092421	SEMA6A	4.953889	5.337615	5.736841	4.06617	3.545059	3.857059	3.14413	3.277772	3.825188	0.000136256	-0.346278372	1.96E-09	-0.589505299
ENSG00000066629	EML1	0.605592	0.538178	0.537882	0.437232	0.581578	0.507034	0.373171	0.403235	0.262165	0.793623764	-0.09736287	0.047722216	-0.589444226
ENSG00000164327	RICTOR	7.577665	8.39894	8.615425	8.540673	8.32297	7.737866	5.000634	4.789688	5.600396	0.060295729	0.148578228	3.98E-14	-0.589119659
ENSG00000160551	TAOK1	10.35521	10.443434	10.599744	10.376084	9.781282	9.761135	6.695348	6.030811	7.141667	0.231874278	0.077498993	7.71E-20	-0.588982912

Gene ID	Gene name	Ctrl-1 FPKM	Ctrl-2 FPKM	Ctrl-3 FPKM	IFNγ-1 FPKM	IFNγ-2 FPKM	IFNγ-3 FPKM	Sora-1 FPKM	Sora-2 FPKM	Sora-3 FPKM	Ctrl vs IFNγ DESeq2 FDR	Ctrl vs IFNγ DESeq2 log2FC	Ctrl vs Sora DESeq2 FDR	Ctrl vs Sora DESeq2 log2FC
ENSG00000135074	ADAM19	1.280236	1.060383	1.200487	0.805491	0.799497	0.820007	0.746912	0.75956	1.081477	0.020283418	-0.421668936	0.002234774	-0.588737669
ENSG00000100711	ZFYVE21	7.010579	7.386618	7.577992	6.711021	5.81206	5.642917	3.748037	4.338661	6.032452	0.361875201	-0.152168378	0.000625217	-0.588727311
NewGene_3164	--	1.372369	0.946672	1.230913	0.679878	0.75314	0.652758	0.376294	0.739828	1.082273	0.132324241	-0.649593551	0.203886115	-0.588639885
ENSG00000149483	TMEM138	15.160108	14.920191	15.821894	12.255664	11.277778	11.368989	9.489847	9.516949	10.227356	0.030241725	-0.205988135	1.65E-10	-0.588584276
ENSG00000076351	SLC46A1	9.355916	8.883175	8.70213	7.563985	7.207485	7.242802	5.368757	5.272531	6.187914	0.080758447	-0.140470903	2.33E-13	-0.588411541
ENSG00000137275	RIPK1	11.940609	11.052949	12.118974	14.65906	14.45319	13.488426	7.213299	7.098973	7.565797	2.58E-09	0.423101462	2.27E-13	-0.588370203
ENSG00000109107	ALDOC	15.306128	12.375439	10.684135	12.327033	12.85987	13.902087	7.581336	7.472966	8.620629	0.385514145	0.127987311	0.00000561	-0.588219382
ENSG00000119812	FAM98A	9.666231	9.554336	11.263332	9.699993	8.504727	8.344528	6.709854	6.379248	5.876927	0.702227752	-0.053165833	9.74E-08	-0.588066712
NewGene_420	--	0.51319	0.642483	0.55442	0.557834	0.545543	0.463167	0.350533	0.334461	0.307235	0.832849612	0.09802964	0.117539832	-0.587719956
ENSG00000144580	CNOT9	30.390291	31.553129	31.290511	28.553389	28.240118	28.260331	19.010181	18.879642	20.2352	0.703845799	-0.031133038	1.42E-19	-0.587715461
ENSG00000189298	ZKSCAN3	2.372605	2.400642	2.568421	2.576165	2.151837	2.111265	1.404254	1.609464	2.07518	0.922429647	0.019270026	0.0000769	-0.587691786
NewGene_1746	--	1.733808	1.77656	1.621974	1.486563	1.382731	1.17162	1.035612	1.308433	0.838416	0.560798651	-0.23430842	0.108736562	-0.587580356
ENSG00000119684	MLH3	3.97522	3.434579	3.738121	2.562452	2.735456	2.440661	2.274716	2.066992	2.451298	0.001966919	-0.303531318	3.8E-09	-0.587460384
ENSG00000120696	KBTBD7	2.902104	2.929771	2.836059	2.039203	2.328557	2.121763	1.831308	1.745602	1.824337	0.043857121	-0.268294406	0.00000209	-0.587286091
NewGene_2102	--	1.168159	0.53013	0.556146	0.639546	0.691852	0.486919	0.305803	0.31036	0.391778	0.109611999	0.411117103	0.034696164	-0.586835405
ENSG00000150687	PRSS23	7.010336	6.6085	6.358914	8.435614	4.161424	6.389056	3.983175	4.39565	4.663014	0.739807622	0.054058528	0.000000715	-0.586046877
ENSG00000013392	RWDD2A	3.895781	3.407738	3.510485	4.9016	4.528053	4.733623	2.209381	2.356154	2.185922	0.000000638	0.535004173	0.000011	-0.585771293
ENSG00000129654	FOXJ1	15.761222	12.455295	11.066101	6.30934	6.669755	7.225031	8.200066	7.51524	8.878157	1.64E-11	-0.811509397	0.00000199	-0.585726235
ENSG00000169258	GPRIN1	3.314337	2.951477	3.228248	2.462229	2.10202	2.870695	1.957141	1.648616	2.347105	0.206326306	-0.203144655	0.0000404	-0.585685314
ENSG00000109436	TBC1D9	5.910891	5.420579	5.802486	3.458846	2.997997	2.97769	3.700108	3.294092	3.718751	1.27E-14	-0.715262087	4.58E-10	-0.5856636
ENSG00000104998	IL27RA	1.752316	1.67164	1.475289	0.700165	0.704321	0.625481	0.989208	1.006563	1.059535	0.000000186	-1.12245194	0.004757448	-0.585355405
ENSG00000196388	INCA1	1.508398	1.211738	1.019548	1.173136	0.737805	1.235371	0.817755	0.880354	0.638789	0.829027322	-0.106117227	0.122581968	-0.584535232
ENSG00000149970	CNKSR2	0.953589	0.896879	0.943477	1.162043	0.908352	0.912144	0.599162	0.727039	0.436332	0.226935849	0.24842068	0.010415054	-0.584508575
ENSG00000128512	DOCK4	7.749081	7.795754	7.92495	5.831541	5.525199	5.967019	4.514444	4.585691	5.251811	0.000632535	-0.269756273	1.11E-11	-0.58402744
ENSG00000125962	ARMCX5	6.082476	5.73716	4.763778	4.977433	3.780268	3.882961	3.829844	3.267833	4.605725	0.093457408	-0.241511985	0.0000429	-0.583939712
ENSG00000116151	MORN1	1.584931	1.90842	1.588138	1.350085	1.288969	1.277894	0.946516	0.851999	1.246618	0.720422904	0.106114028	0.02447515	-0.583715452
ENSG00000152056	AP1S3	3.633129	4.873589	4.352143	5.053273	4.360121	4.179402	2.788723	2.874296	3.097155	0.24096363	0.199045774	0.000392078	-0.583661184
ENSG00000204439	C6orf47	17.656658	15.096904	15.350008	15.073847	15.946163	16.422447	10.197187	9.993891	9.895278	0.217501262	0.127903782	6.52E-10	-0.583643303
ENSG00000270170	NCBP2-AS2	37.953342	37.143188	36.057503	32.135014	34.402882	33.961082	21.753489	23.560257	24.203512	0.982866264	0.003008501	1.12E-10	-0.583322413
ENSG00000159110	IFNAR2	5.018072	5.175414	7.532225	10.145911	10.398432	10.559222	4.573548	4.775553	4.58266	5.48E-17	0.821176434	0.00000602	-0.583068702
ENSG00000162931	TRIM17	0.60731	0.859992	0.85743	0.870984	0.527192	0.929357	0.447521	0.452142	0.496558	0.832084717	0.101001713	0.107524131	-0.582672916
ENSG00000164743	C8orf48	1.350542	1.219999	0.692377	0.688098	0.890681	0.819175	0.837601	0.493619	0.717548	0.53817511	-0.2912769	0.165351916	-0.582415355
ENSG00000185561	TLCD2	0.707571	0.671984	0.576059	1.25293	1.599764	1.545295	0.421517	0.333907	0.472802	6.81E-15	1.31103404	0.015037052	-0.581936574
ENSG00000176714	CCDC121	1.20724	1.697373	0.951984	0.974233	1.113031	0.876509	0.62322	0.892017	0.77939	0.375250066	-0.303805193	0.066269398	-0.58153965
ENSG00000172888	ZNF621	5.308973	5.291611	5.915868	5.598119	5.406779	4.877362	3.555345	3.324372	3.683493	0.162234389	0.149520849	3.71E-08	-0.580988947
ENSG00000171940	ZNF217	17.014227	15.948345	15.886924	18.073661	17.456794	17.056204	9.905705	10.189267	10.646577	0.0000482	0.249288841	1.36E-17	-0.580722484
ENSG00000010539	ZNF200	4.358028	4.558909	4.602304	3.428806	4.509144	3.660539	2.957989	2.307024	3.203027	0.80753236	-0.045404776	0.0000368	-0.580698469
NewGene_6058	--	2.892602	3.528728	3.813162	2.720722	3.111262	2.808001	2.260093	2.153381	2.421061	0.667830333	-0.097590805	0.00130159	-0.580589395
ENSG00000176014	TUBB6	7.885011	7.989976	9.677836	3.512429	2.891027	3.722422	6.061552	5.352154	5.310975	1.59E-19	-1.203966103	0.00000635	-0.580415025
ENSG00000163701	IL17RE	2.500818	1.943874	1.451039	2.612066	2.970771	3.019651	1.272868	1.224827	1.109307	0.000661405	0.669156605	0.010960216	-0.580337349
ENSG00000183309	ZNF623	6.192229	6.430307	6.311487	6.279099	5.728254	5.538114	4.729592	4.013298	4.386547	0.948988526	-0.008395338	1.83E-10	-0.580276058
ENSG00000196968	FUT11	2.911081	3.411767	3.145048	2.74355	2.804348	2.604446	1.810131	1.837629	2.205112	0.830023999	-0.05197109	0.001629966	-0.580239086
ENSG00000186230	ZNF749	2.581649	2.45585	2.29731	2.205218	2.085716	1.769481	1.337066	1.668955	1.631576	0.49996059	-0.146183886	0.002206657	-0.580202945
ENSG00000174705	SH3PXD2B	1.692964	1.369754	1.410013	1.094251	0.987431	0.854663	1.146519	1.17334	0.994174	0.000754213	-0.522679937	0.000387545	-0.580162948
ENSG00000256087	ZNF432	2.082889	2.31119	2.427593	2.534694	1.911733	1.992141	1.484813	1.40671	1.475087	0.653089167	0.090423601	0.000117737	-0.579806035
ENSG00000134333	LDHA	471.505051	408.527109	371.293249	337.663668	315.839665	318.029758	253.582722	241.847279	255.879192	0.017124092	-0.195801496	6.42E-13	-0.579667082
ENSG00000135521	LTV1	29.415171	29.40304	34.19698	29.177624	23.14732	23.942198	18.920391	20.700388	18.612741	0.200229718	-0.140635602	0.000000001	-0.579659094
ENSG00000280236	OR12D2	0.717264	0.904374	0.898923	0	0.175255	0.055069	0.401198	0.359971	0.848993	0.000000356	-3.375410614	0.236857194	-0.579369717
ENSG00000198131	ZNF544	15.448188	15.562703	16.657206	12.489477	11.0848	12.113468	9.578067	10.096219	11.306747	0.0000648	-0.298940249	9.12E-15	-0.579293186
ENSG00000138764	CCNG2	10.829411	9.767955	9.159845	7.575012	7.968346	8.565022	5.902847	6.363188	6.213997	0.870039748	-0.020158893	1.23E-10	-0.578968691
ENSG00000076258	FMO4	1.902293	2.0971	2.029796	1.310696	1.147718	1.371487	1.233195	1.042025	1.514478	0.022540741	-0.502537085	0.00898658	-0.578967464
ENSG00000154957	ZNF18	2.336475	2.259794	2.321151	2.183799	2.241629	1.871112	1.499909	1.544931	1.16507	0.939869928	0.02160183	0.006332726	-0.578945133
NewGene_1170	--	1.006286	1.210168	0.944397	0.601153	0.6222	0.451696	0.64044	0.593144	0.338117	0.011805492	-0.742820638	0.074042617	-0.578889465
ENSG00000102466	FGF14	0.117378	0.108471	0.337059	0.15364	0.263662	0.141239	0.116149	0.107088	0.0569	0.11826864	0.512942148	0.132925168	-0.578844924
ENSG00000250312	ZNF718	5.794441	6.682772	6.789148	7.001844	6.850937	6.243879	4.121174	3.679009	4.944073	0.072675048	0.21147682	0.0000028	-0.578680382
ENSG00000041353	RAB27B	0.331091	0.25286	0.238198	0.093492	0.16675	0.153463	0.124813	0.08801	0.296096	0.037435531	-0.793574151	0.188899402	-0.578400273
ENSG00000128346	C22orf23	1.847556	2.044583	1.608105	1.493407	1.656518	1.260496	1.338201	1.055445	1.043583	0.590833768	-0.169831425	0.028405653	-0.577728959
ENSG00000112584	FAM120B	7.040765	6.724216	6.63751	6.112542	5.79473	5.941297	4.202154	4.209877	4.417763	0.668451346	-0.046777686	2.5E-11	-0.576530075
ENSG00000151883	PARP8	5.419832	4.824805	5.04156	5.482364	5.917226	5.923831	2.926114	4.307234	4.004951	0.002735102	0.269243312	8.32E-09	-0.576169885
ENSG00000183943	PRKX	4.098146	4.459263	4.666455	3.013467	2.710282	3.090352	2.712814	2.637983	2.972425	0.00000977	-0.436719229	0.000000008	-0.576129021
ENSG00000153201	RANBP2	13.459264	14.995657	15.129426	14.153176	14.053139	14.312236	8.952347	8.630494	9.872276	0.100414903	0.112033467	3.15E-17	-0.575175025
ENSG00000145632	PLK2	7.10566	6.348246	7.371423	2.169527	2.412981	2.387503	4.444258	3.806756	4.90214	1.01E-34	-1.429541697	0.00000111	-0.574616145
ENSG00000184708	EIF4ENIF1	9.527124	9.125065	8.932646	9.180308	7.860157	7.844868	5.566589	5.514559	6.295333	0.951885383	-0.008840892	1.51E-10	-0.574248587
ENSG00000183621	ZNF438	0.776643	0.67223	0.976746	0.654271	0.750633	0.632772	0.397046	0.475003	0.603437	0.461876353	-0.243414635	0.0571355	-0.573684454
ENSG00000168502	MTCL1	5.664086	5.210992	5.727523	3.327394	2.824506	3.238043	3.257158	3.311574	3.610381	6.31E-11	-0.639255749	0.000000013	-0.573362913
ENSG00000132329	RAMP1	1.546878	1.166597	1.381556	1.527145	1.163726	1.045697	0.768795	1.077153	0.887669	0.999202724	-0.000945881	0.180290673	-0.573234994
ENSG00000147162	OGT	16.017552	16.028398	16.00138	15.988062	16.125241	14.940868	10.028075	9.663373	10.253363	0.089990841	0.11618666	8.51E-19	-0.572870522
ENSG00000174695	TMEM167A	29.886072	30.878813	29.964022	23.625757	24.82366	23.957085	18.784975	18.694046	19.742565	0.002970022	-0.177700584	5.26E-22	-0.572425712
ENSG00000197343	ZNF655	15.720947	16.676309	20.018577	22.215065	18.935225	18.92914	10.311308	12.02338	11.315168	0.000000665	0.379846216	3.16E-11	-0.572336196
NewGene_5287	--	1.300226	0.653077	0.749277	0.994164	0.570771	1.079791	0.344906	0.875105	0.398437	0.774766555	0.152040697	0.243744246	-0.571834881
NewGene_148	--	2.33878	2.034874	1.418878	0.927955	1.074074	1.178028	1.193817	1.34238	1.469771	0.038943286	-0.605137967	0.033786459	-0.57176016
ENSG00000260643	AC092718.3	0.938442	0.234925	1.80974	0.497705	0.15954	0.30057	0.177834	0.677655	0.984999	--	--	0.685186131	-0.571581731
ENSG00000173227	SYT12	1.008474	1.237336	1.166812	0.273499	0.376705	0.478108	0.796118	0.757814	0.785358	9.58E-10	-1.425932204	0.005044666	-0.571451703
ENSG00000253719	ATXN7L3B	32.978951	32.567421	31.349628	32.438751	30.199989	31.882896	20.500893	19.640081	21.025679	0.050152378	0.111213894	8.42E-25	-0.57136309

Gene ID	Gene name	Ctrl-1 FPKM	Ctrl-2 FPKM	Ctrl-3 FPKM	IFNγ-1 FPKM	IFNγ-2 FPKM	IFNγ-3 FPKM	Sora-1 FPKM	Sora-2 FPKM	Sora-3 FPKM	Ctrl vs IFNγ DESeq2 FDR	Ctrl vs IFNγ DESeq2 log2FC	Ctrl vs Sora DESeq2 FDR	Ctrl vs Sora DESeq2 log2FC
ENSG00000168675	LDLRAD4	0.305232	0.268938	0.293767	0.255785	0.39304	0.256758	0.190147	0.209209	0.146042	0.975935104	-0.01458022	0.058674441	-0.570949127
NewGene 5105	--	1.45121	1.292832	0.649312	0.784882	0.594309	0.847197	0.773481	0.667383	0.558549	0.389075455	-0.369508626	0.140456298	-0.570487155
ENSG00000111412	C12orf49	41.981628	39.901959	41.286012	23.036356	24.262345	23.607822	26.316835	24.925126	28.062311	2.64E-37	-0.657089589	1.84E-24	-0.570428532
ENSG00000169905	TOR1AIP2	12.062949	14.108363	14.962339	14.64489	13.790942	13.771468	8.764124	8.67264	9.208078	0.053807932	0.162554686	3.21E-12	-0.570170353
NewGene 4925	--	2.42918	2.640337	2.975245	2.510352	1.956014	2.245607	1.874269	1.873023	2.465629	0.221867516	-0.183502651	0.000162652	-0.570124845
ENSG00000180011	ZADH2	2.337046	2.259569	2.740399	2.142287	2.08241	2.15723	1.376906	1.337589	1.831708	0.770507182	-0.046343834	0.0000321	-0.570092961
NewGene 3714	--	1.600969	1.77537	1.708863	1.728649	1.631188	1.392	1.159663	1.057131	1.226288	0.630512427	0.079478144	0.0000124	-0.570066697
NewGene 5170	--	0.81416	0.685971	0.687234	0.438211	0.551463	0.523871	0.490452	0.451992	0.56366	0.126681049	-0.61924831	0.134369807	-0.569996402
ENSG00000185900	POMK	4.017316	3.742964	3.734389	3.359379	2.833105	3.434986	2.520244	1.99488	2.714161	0.676662538	-0.091694452	0.001351105	-0.569949115
NewGene 1560	--	0.818146	0.605715	0.734159	0.523418	0.586443	0.540823	0.366065	0.520414	0.469333	0.534021932	-0.240868944	0.096026985	-0.569841435
ENSG00000087448	KLHL42	3.527134	3.927383	3.951877	3.798273	3.136397	3.385362	2.784176	2.552928	3.241969	0.969567734	-0.006651122	0.000000801	-0.569297287
ENSG00000155657	TTN	0.23068	0.234607	0.238297	0.272243	0.286426	0.225595	0.142008	0.141338	0.153475	0.415640768	0.098841244	5.17E-09	-0.569072851
ENSG00000079691	CARMIL1	7.08651	6.504289	6.352916	6.350849	6.726335	6.047239	4.235619	4.016598	4.35893	0.427112583	0.086696778	2.48E-10	-0.568937744
ENSG00000182903	ZNF721	20.047818	21.968691	22.31529	22.2539	22.777003	17.727809	16.643053	15.555639	16.36299	0.759573467	0.127562394	0.00000417	-0.568642341
NewGene 1237	--	5.795883	5.941485	6.639	5.857416	6.275525	5.636818	4.247161	5.16983	5.189034	0.26239509	0.098049079	3.45E-10	-0.568435596
ENSG00000010244	ZNF207	51.437282	57.405048	40.638487	40.20314	45.451394	38.623494	31.396521	37.481405	32.735442	0.507111924	0.041319042	7.36E-20	-0.568324702
ENSG00000214717	ZBED1	9.825468	8.264198	7.304247	7.919687	7.228751	8.21412	5.440477	4.622994	6.35776	0.933602721	0.013802788	0.00000129	-0.56817152
ENSG00000198440	ZNF583	1.220424	1.124513	1.582598	0.950412	0.950916	0.995332	0.903214	0.655362	0.81105	0.32823744	-0.208568004	0.004261647	-0.567444615
ENSG00000175395	ZNF25	0.161759	0.271513	0.384983	0.213807	0.235827	0.305103	0.129697	0.14013	0.241111	0.964072562	0.030806319	0.263977895	-0.567222334
ENSG00000179862	CITED4	6.397995	5.452999	4.935576	2.85481	3.359027	4.164636	3.305015	3.397933	3.935452	0.007061157	-0.542014937	0.001572913	-0.566747992
NewGene 3624	--	2.254102	2.782847	2.742747	1.751661	1.936513	2.310233	1.831112	1.518881	1.564894	0.334407097	-0.220411491	0.004070605	-0.566727509
ENSG00000273820	USP27X	1.255538	1.061968	0.784039	1.067235	0.943965	0.916007	0.657524	0.555848	0.750322	0.872283178	0.062779635	0.060230968	-0.566654954
ENSG00000267041	ZNF850	1.718542	2.149616	2.134363	1.290157	1.072296	1.192782	1.302072	1.176402	1.540404	0.00000521	-0.603737855	0.0000192	-0.566498068
ENSG00000137843	PAK6	2.261111	4.895046	3.971946	4.568059	3.840425	3.466575	3.705177	2.340877	0.820524	0.690316114	0.099215617	0.634986047	-0.566488957
ENSG00000005882	PDK2	4.355135	3.737545	3.318778	3.611494	3.462248	3.674862	2.379757	2.56884	2.105904	0.589028756	0.09102207	0.000194646	-0.566352626
ENSG00000165424	ZCCHC24	4.772904	4.552699	4.896887	2.86393	2.874047	2.838619	3.226173	2.634664	3.156851	1.12E-09	-0.584704523	0.00000013	-0.566054304
ENSG00000174839	DENND6A	5.340411	6.369329	5.908917	4.99288	4.598207	4.944046	3.6713	3.593329	3.847377	0.298386137	-0.120059365	0.000000028	-0.566009957
ENSG00000138356	AOX1	1.714771	2.030233	2.058096	1.089244	1.038239	1.083185	1.170228	1.164284	1.364484	0.00000258	-0.704587141	0.000239242	-0.565987866
ENSG00000168246	UBTD2	11.638931	11.796929	11.11377	9.621939	8.777623	9.498315	6.894668	7.068015	7.93627	0.067827177	-0.161182544	7.49E-11	-0.565795444
ENSG00000140367	UBE2Q2	11.046731	13.192107	12.317962	11.83257	10.61164	11.352202	7.516049	7.136383	8.588366	0.743494467	0.041247999	1.24E-08	-0.565362057
NewGene 623	--	0.420494	0.813463	0.950902	0.880684	0.546083	0.539754	0.464915	0.559761	0.356178	0.98538095	-0.010974347	0.18130392	-0.565164634
ENSG00000171862	PTEN	5.319589	6.00889	5.495808	6.43845	6.270475	5.89212	4.021268	3.409933	3.600776	0.0000834	0.288761669	1.91E-10	-0.565119704
ENSG00000139292	LGR5	15.767676	15.497854	17.154275	14.111816	11.424754	12.43755	10.381923	9.45159	10.765733	0.02112821	-0.200474148	9.59E-14	-0.565028073
NewGene 3448	--	0.39403	0.378304	0.428836	0.267321	0.076782	0.575557	0.278335	0.314207	0.211517	0.815581929	-0.284892208	0.090046306	-0.564823404
ENSG00000267508	ZNF285	0.417674	0.532019	0.328901	0.647333	0.832358	0.33922	0.315064	0.292377	0.140171	0.977285732	-0.013350262	0.119233116	-0.564692613
ENSG00000184277	TM2D3	19.169073	19.192813	19.564512	16.202863	17.845443	16.866722	10.66046	12.669719	12.393841	0.785417546	-0.034887074	5.81E-09	-0.564527972
ENSG00000105750	ZNF85	4.048178	4.415464	4.383849	3.26176	3.37484	3.114886	2.792529	2.778838	3.580361	0.072836674	-0.291152191	0.000338913	-0.564463277
ENSG00000111834	RSPH4A	1.016	0.831396	0.910226	0.721039	0.459592	0.521204	0.609807	0.664071	0.46102	0.086829482	-0.541555822	0.060437211	-0.564427277
ENSG00000135116	HRK	0.768602	0.995724	0.768639	0.409674	0.396303	0.345871	0.696615	0.544923	0.520466	0.0000079	-0.988845837	0.011470102	-0.564414235
NewGene 5704	--	0.778935	0.859558	0.751086	0.988512	1.08717	1.117801	0.475896	0.610096	0.414675	0.018136654	0.567174371	0.060289726	-0.564279539
ENSG00000132664	POLR3F	7.080688	7.750191	9.038327	7.540582	7.147305	7.078672	5.586138	4.585198	4.955192	0.933602721	0.015157125	0.0000134	-0.564041606
ENSG00000204186	ZDBF2	0.418202	0.512585	0.511135	0.455093	0.483723	0.45427	0.312991	0.273332	0.267958	0.584956656	0.147665657	0.014333938	-0.563674516
ENSG00000213937	CLDN9	0.604291	0.710755	0.971869	0.227752	0.430491	0.320069	0.537108	0.489756	0.409971	0.013606828	-1.060399711	0.15105551	-0.563550956
NewGene 4033	--	1.191067	0.999385	0.670558	0.688117	0.860045	0.970897	0.945933	0.498198	0.423825	0.960732867	-0.032168033	0.263189722	-0.563431161
ENSG00000003987	MTMR7	0.825844	1.070113	0.740641	1.051298	0.777148	1.274639	0.714128	0.554904	0.399516	0.187301258	0.370362577	0.064338066	-0.562995326
ENSG00000080546	SESN1	5.238076	4.334964	4.572792	4.575485	4.750788	5.010164	3.109072	2.573861	3.05139	0.145499929	0.190643337	0.0000089	-0.562975053
ENSG00000138031	ADCY3	0.487103	0.523646	0.403383	0.456646	0.385734	0.452306	0.301764	0.334937	0.276749	0.974602911	0.014734088	0.067064115	-0.562304712
ENSG00000196372	ASB13	7.807988	8.360178	6.900119	7.017332	6.546956	7.400214	5.29212	4.508655	4.857563	0.933337999	0.014471758	0.00000185	-0.561901156
ENSG00000197579	TOPORS	11.009724	10.827144	10.044459	9.454724	9.953956	9.050376	6.427361	6.002939	7.910894	0.875857468	-0.019283209	7.99E-09	-0.561750372
ENSG00000125484	GTF3C4	9.856941	10.23665	10.515761	8.746984	8.234331	8.863444	6.276132	6.209074	6.972045	0.183723597	-0.096020264	1.35E-16	-0.56166274
ENSG00000107560	RAB11FIP2	1.487657	1.784054	1.932045	2.214059	1.941838	1.865538	1.181484	0.889143	1.246851	0.014124855	0.357514184	0.000725147	-0.561532937
ENSG00000165572	KBTBD6	1.990912	2.267116	2.219985	1.979339	1.909419	1.914039	1.46906	1.163955	1.488279	0.958082541	-0.011030286	0.000127539	-0.561205138
ENSG00000110583	NAA40	24.504019	23.687098	25.637354	22.18854	22.195172	20.39027	15.59061	14.085985	17.122304	0.174061598	-0.099461356	3.02E-13	-0.561170316
ENSG00000080007	DDX43	1.953399	2.07829	2.513905	1.444937	1.468834	1.627015	1.560072	1.363985	1.220049	0.076178887	-0.377646904	0.007774558	-0.56070627
ENSG00000011114	BTBD7	4.411919	4.395018	5.086179	4.289549	3.807229	3.888373	3.74556	2.964072	3.770085	0.793856738	-0.033738639	0.000000036	-0.560701479
ENSG00000088992	TESC	98.188179	87.196986	98.399896	32.687202	38.406579	36.708736	53.470002	63.259474	65.233726	1.54E-54	-1.245133728	3.27E-10	-0.560259762
ENSG00000184675	AMER1	1.386723	1.216946	1.114921	1.793834	1.621009	1.786037	0.77388	0.742775	0.848646	0.000000375	0.628263087	0.000191823	-0.560143728
ENSG00000140320	BAHD1	9.301885	9.227339	8.784694	8.524121	8.585063	9.031751	5.927875	5.509364	5.856702	0.345023709	0.087251991	7.39E-12	-0.560109288
ENSG00000100784	RPS6KA5	0.110606	0.111736	0.158602	0.164474	0.283714	0.18196	0.075024	0.064543	0.201242	0.001237391	0.691002849	0.038743647	-0.559937759
ENSG00000167695	FAM57A	20.898912	21.228384	20.093733	16.514494	16.207956	16.944505	12.45055	13.467237	13.856541	0.016163171	-0.189371946	2.39E-11	-0.559474671
ENSG00000180155	LYNX1	1.778502	1.626666	1.337849	1.10025	1.307507	1.058296	0.794549	1.161921	1.022404	0.179712827	-0.273398189	0.005863476	-0.559076575
ENSG00000163577	EIF5A2	1.645015	1.82421	2.289438	1.467041	1.40372	1.511814	1.240543	1.170159	1.330426	0.176926808	-0.237907168	0.000596928	-0.558901793
ENSG00000067715	SYT1	1.334755	0.873371	0.829178	0.724006	0.720354	1.249579	0.820936	1.149524	0.659564	0.517558999	-0.222685809	0.067052324	-0.558542645
ENSG00000128805	ARHGAP22	0.357838	0.703809	0.623662	0.193267	0.393136	0.296759	0.432697	0.299098	0.344135	0.203694494	-0.615015855	0.206607251	-0.558301522
ENSG00000037749	MFAP3	7.075459	6.388541	7.751358	7.597478	5.908925	5.353894	3.195658	3.829272	4.111778	0.63852072	0.06733917	0.0000135	-0.558246705
ENSG00000166510	CCDC68	6.132352	6.823915	8.192368	13.939667	12.919937	13.544991	4.86021	4.026274	4.78792	1.48E-32	1.083451826	0.00000294	-0.558240108
ENSG00000204616	TRIM31	1.841195	1.748925	1.728837	10.025579	9.844838	9.113627	1.098153	1.108818	1.173938	8.92E-98	2.587663862	0.017693657	-0.557856847
NewGene 2428	--	10.598133	10.908448	11.841546	10.625436	9.052859	10.027574	7.04945	7.092146	8.428901	0.820690829	0.022485985	7.13E-15	-0.557717244
ENSG00000125875	TBC1D20	10.238112	9.853058	10.435614	10.829413	10.459824	10.336852	6.495555	5.71472	7.728066	0.012116954	0.202663329	1.21E-09	-0.557708486
ENSG00000270181	BIVM-ERCC5	2.529221	9.851429	2.755493	0	4.071494	1.00E-06	8.074276	1.972758	0	0.630862844	-1.796087935	0.848514695	-0.557373287
ENSG00000177971	IMP3	58.976436	59.514732	53.176979	46.32783	50.495258	37.13949	34.114198	36.744817	36.55016	0.615498785	0.048710462	1.53E-11	-0.557315597
NewGene 2669	--	0.624724	0.791227	0.616595	0.8298	0.417289	0.388321	0.514926	0.304873	0.477221	0.747091931	-0.163005852	0.131562849	-0.557159607
ENSG00000137177	KIF13A	34.013751	35.221863	34.454843	28.133123	28.22471	28.208548	23.935384	23.047859	26.540325	0.010098882	-0.15739544	3.85E-15	-0.557065843
ENSG00000112425	EPM2A	2.482293	2.212513	2.694927	2.344955	2.61295	2.520243	1.641221	1.472104	1.460554	0.294856855	0.182773315	0.000772267	-0.557039443

Gene ID	Gene name	Ctrl-1 FPKM	Ctrl-2 FPKM	Ctrl-3 FPKM	IFNγ-1 FPKM	IFNγ-2 FPKM	IFNγ-3 FPKM	Sora-1 FPKM	Sora-2 FPKM	Sora-3 FPKM	Ctrl vs IFNγ DESeq2 FDR	Ctrl vs IFNγ DESeq2 log2FC	Ctrl vs Sora DESeq2 FDR	Ctrl vs Sora DESeq2 log2FC
NewGene 3737	--	1.045723	1.319411	1.242105	0.750812	0.986816	1.147651	0.939478	0.702899	0.465621	0.703543164	-0.152783612	0.126158269	-0.556399212
ENSG00000165244	ZNF367	7.838474	9.252062	9.736122	8.277799	7.984822	8.843769	6.040303	5.269714	5.80424	0.694696774	0.053184772	0.000000136	-0.556095454
NewGene 3076	--	0.811607	0.694226	0.747014	0.886045	1.261492	1.021672	0.608394	0.364362	0.588204	0.05168254	0.554332637	0.104410537	-0.555316281
ENSG00000120322	PCDHB8	0.51702	0.260051	0.464997	0.339888	0.361277	0.227021	0.360558	0.196394	0.248234	0.603884024	-0.292353299	0.253270165	-0.55526793
ENSG00000158246	TENT5B	4.073226	3.303999	3.704559	4.744481	5.230573	4.886582	1.92065	2.119864	3.054574	0.000027	0.570317389	0.003248496	-0.555146969
ENSG00000119720	NRDE2	2.929032	3.040749	2.974563	2.70043	2.929842	2.960275	2.133609	1.946981	1.97696	0.531620391	0.061876806	6.72E-11	-0.554802238
ENSG00000181873	IBA57	1.600336	1.560197	1.201909	1.388533	1.264782	1.278997	0.928572	0.888894	0.970018	0.986291654	-0.003951325	0.000299556	-0.554735902
ENSG00000164663	USP49	2.837038	2.87196	2.973786	2.70799	2.47101	2.718565	1.964384	1.772638	1.869201	0.818462962	-0.032856508	0.000000706	-0.554632799
ENSG00000176399	DMRTA1	1.211532	1.349218	1.476089	1.185438	1.142946	1.177508	0.806887	0.985915	0.772268	0.812277542	-0.053239323	0.002816401	-0.554591668
ENSG00000108654	DDX5	244.380154	241.807532	261.667417	209.856609	214.605747	214.054668	155.702992	154.094686	162.619357	0.298386137	-0.059418979	1.87E-23	-0.55457619
ENSG00000139697	SBNO1	9.680158	10.591486	10.656345	8.947012	8.843309	8.661187	6.720547	6.031419	7.029134	0.459571717	-0.059068314	1.11E-15	-0.554525281
ENSG00000176396	EID2	6.957881	6.445944	5.916969	7.925568	8.561194	8.025857	4.151594	4.141043	4.027118	0.0000922	0.489469504	0.000151001	-0.554265339
NewGene 4658	--	1.573026	0.888463	1.162617	0.711781	0.631261	0.645693	0.527227	1.209756	0.560862	0.052772628	-0.785632127	0.224454436	-0.554231835
NewGene 4095	--	0.962184	0.947069	1.090808	0.963318	0.605407	0.814482	0.539319	0.546654	0.832753	0.517558999	-0.183559057	0.020610246	-0.554205479
ENSG00000189180	ZNF33A	6.336287	7.149556	7.290412	6.981056	6.379676	5.989927	4.10136	4.931354	4.796356	0.912798218	0.015733393	1.83E-08	-0.554173193
ENSG00000176994	SMCR8	7.91322	7.781788	7.273734	7.974787	7.289787	7.378477	5.043197	4.558636	5.078475	0.104555067	0.125829358	1.91E-13	-0.554152339
ENSG00000177042	TMEM80	15.666012	15.663788	15.442094	13.039414	12.773815	13.312651	8.732345	9.226576	10.72938	0.177820475	-0.158268011	0.00000328	-0.554120464
ENSG00000197077	KIAA1671	6.021835	5.432992	5.963746	6.450914	5.976305	6.300296	3.506131	2.844273	4.015282	0.00000309	0.323424458	1.55E-09	-0.553852516
ENSG00000155324	GRAMD2B	5.52874	4.679458	5.048362	3.727209	4.308928	3.748394	3.227474	3.277217	2.9697	0.160765321	-0.193263936	0.0000226	-0.553734847
ENSG00000173705	SUSD5	0.340373	0.290874	0.221212	0.20314	0.159401	0.134321	0.177987	0.150863	0.209931	0.153992058	-0.620288145	0.170033734	-0.553630994
ENSG00000120458	MSANTD2	4.826565	5.066949	5.039353	3.770589	3.724259	3.163557	3.123629	2.657656	3.386303	0.074899771	-0.24656262	0.0000346	-0.553593931
ENSG00000176907	TCIM	33.142212	32.831417	32.150631	19.955921	21.169497	19.465958	20.033169	21.165649	21.47258	3.27E-15	-0.548404219	1.27E-13	-0.553268674
ENSG00000137310	TCF19	13.255434	14.146559	14.743003	12.287869	11.14896	12.167097	8.990197	8.398633	9.306988	0.288282137	-0.098552374	8.21E-12	-0.553090061
ENSG00000086506	HBQ1	2.095074	2.593828	2.131961	2.086066	2.042437	1.29169	1.734408	1.522089	1.107158	0.70876923	-0.19745417	0.202066019	-0.553084196
ENSG00000108423	TUBD1	9.042242	10.353508	11.637308	9.600033	7.853081	10.0384	6.134529	5.929868	6.824499	0.992844663	0.00187929	0.0000191	-0.553034582
ENSG00000184349	EFNA5	0.40855	0.385673	0.489076	3.068034	2.990599	3.087304	0.199877	0.3532	0.24393	6.27E-93	2.979891733	0.106915204	-0.552358977
ENSG00000029993	HMGB3	14.205215	15.461218	15.585786	15.059642	10.299322	10.597123	9.368713	9.498393	9.000472	0.001389943	-0.273975643	4.62E-11	-0.552169971
ENSG00000172748	ZNF596	1.380714	1.56629	1.012842	1.196843	1.101784	1.49925	0.860593	0.557239	0.972771	0.982392413	0.008841201	0.048740108	-0.551656702
ENSG00000088386	SLC15A1	3.75017	3.718048	3.37768	6.36624	5.104618	5.372647	2.47366	2.134604	2.327698	1.72E-11	0.779723322	0.0000841	-0.551007117
ENSG00000100503	NIN	2.932786	2.886337	3.32986	2.252056	2.158551	1.860609	2.063864	2.092929	2.189798	0.0000045	-0.44603314	9.32E-09	-0.550599936
ENSG00000112297	CRYBG1	14.759694	16.768722	17.161501	16.578924	16.260805	15.303241	10.096847	9.647045	11.415496	0.000145777	0.270775614	1.32E-11	-0.550499177
ENSG00000104081	BMF	59.757521	54.468227	51.079469	57.298078	59.145592	57.691754	34.034249	32.494857	38.077464	0.001548644	0.210528156	1.2E-14	-0.550458952
ENSG00000158270	COLEC12	0.259741	0.202139	0.261237	0.307083	0.454868	0.459184	0.119214	0.134615	0.212169	0.004727405	0.893764886	0.184794659	-0.550358145
ENSG00000197951	ZNF71	3.557511	2.917233	3.396314	2.623468	2.130275	2.562162	2.524786	2.163087	2.462556	0.005944121	-0.358447353	0.0000717	-0.549792446
ENSG00000130684	ZNF337	3.64464	3.851827	3.656525	4.060465	3.488873	3.505715	2.692553	2.304982	2.415565	0.503914926	0.104578679	0.0000423	-0.549671094
ENSG00000196584	XRCC2	5.792599	6.412566	6.148642	5.61129	4.295674	5.148358	4.375923	3.601591	3.778455	0.319134831	-0.139399758	0.00000409	-0.549612477
ENSG00000107562	CXCL12	43.131461	32.356749	28.811154	26.671083	27.655282	25.17881	23.273847	23.919377	24.566593	0.330067615	-0.243085972	0.007857784	-0.549326159
ENSG00000198099	ADH4	4.464114	5.378536	4.864807	3.339407	3.17361	3.609784	2.844926	3.280007	2.660049	0.086303942	-0.28826298	0.000738641	-0.549192971
ENSG00000005812	FBXL3	6.472348	7.193073	7.356672	7.332009	6.873292	6.750303	4.928879	4.24314	4.312131	0.165726484	0.151097717	0.000000588	-0.549069429
ENSG00000171608	PIK3CD	0.211939	0.226754	0.243276	0.103317	0.152266	0.113249	0.155094	0.147533	0.115555	0.13362872	-0.687850754	0.197876442	-0.54903838
ENSG00000147124	ZNF41	2.587662	2.295255	2.557698	2.089922	3.203957	2.12801	1.550657	1.542538	1.662607	0.640603532	-0.085918677	0.000139361	-0.548870368
ENSG00000269743	SLC25A53	0.289436	0.393851	0.334891	0.274374	0.249612	0.337442	0.231739	0.220067	0.194782	0.836164767	-0.088122779	0.090799018	-0.548864812
ENSG00000239521	CASTOR3	2.160995	2.506361	2.088684	2.487564	2.629803	2.493979	1.199627	1.600528	1.50548	0.068842774	0.319654219	0.005816013	-0.548498853
ENSG00000055609	KMT2C	4.938432	5.064618	4.697111	5.387524	4.78694	4.822438	2.795937	2.522475	3.069211	0.429130477	0.076617118	6.54E-11	-0.547674336
ENSG00000136381	IREB2	26.007486	28.679584	29.955273	26.576764	26.383078	25.443261	18.157712	17.25343	19.095225	0.573537829	0.045559585	2.37E-16	-0.547300645
ENSG00000069493	CLEC2D	2.094004	2.742127	3.05044	2.690682	2.780919	2.655924	1.696523	1.256451	1.293747	0.097753787	0.326580889	0.009372435	-0.547027984
ENSG00000164151	ICE1	8.977963	9.298119	9.285173	11.129899	10.982077	10.531064	6.071711	5.446335	6.193314	2.84E-11	0.391011749	9.61E-15	-0.547014056
ENSG00000134285	FKBP11	3.72529	4.211073	3.37519	3.911049	2.94999	1.956804	2.16094	1.86947	3.121257	0.439035071	-0.263652804	0.046203687	-0.546926654
ENSG00000140406	TLNRD1	4.382605	4.60903	4.334865	4.192383	4.149679	4.37219	2.778296	2.611187	3.176241	0.508802707	0.080181315	0.000000304	-0.546920032
ENSG00000259288	BUB1B-PAK6	2.362351	0.916475	1.152334	0.604974	0.377497	0.717464	0.345701	0.037916	2.585135	0.000717615	-1.238172945	0.764476648	-0.546804542
ENSG00000129194	SOX15	1.508377	1.396071	0.934944	1.162301	0.786174	1.150661	0.558048	1.027309	0.934293	0.66557697	-0.202273505	0.173147736	-0.546751439
ENSG00000143970	ASXL2	3.176651	3.391231	3.65434	3.653865	3.285915	3.38425	2.247707	2.009802	2.309409	0.076048026	0.157357169	5.43E-10	-0.546604933
ENSG00000272414	FAM47E-STBD1	9.844252	9.301001	8.926681	7.326699	7.09657	5.130549	0	8.84411	9.184501	0.003403008	-0.377025195	0.841034707	-0.546424258
ENSG00000177732	SOX12	4.878203	4.940526	4.036715	4.904527	4.738194	4.813976	2.920701	2.757252	3.228566	0.069583515	0.208664726	0.00000312	-0.546413878
ENSG00000135643	KCNMB4	0.50954	0.591008	0.549733	0.427217	0.350755	0.453183	0.461808	0.238551	0.427781	0.466254712	-0.244339268	0.07415223	-0.546088154
ENSG00000185344	ATP6V0A2	10.573389	11.071975	12.115833	10.990401	10.186174	10.399669	7.714469	6.794473	7.63864	0.508771073	0.062183987	2.5E-11	-0.545256961
ENSG00000139266	9-Mar	3.920693	3.638292	4.132607	4.004331	3.668781	3.575531	2.607602	2.377984	2.785168	0.732284131	0.0646482	0.000588591	-0.544399449
ENSG00000181467	RAP2B	5.797214	5.558595	6.692317	4.087035	4.633561	4.278557	3.633535	3.545596	4.44978	0.000566667	-0.325389891	4.63E-08	-0.544277212
ENSG00000100479	POLE2	8.825351	8.055931	8.516378	8.340565	7.251312	6.769906	5.587826	5.501062	5.346148	0.788165344	-0.045494354	0.0000084	-0.544069891
ENSG00000085511	MAP3K4	4.726078	4.421921	4.255529	4.294097	4.141949	3.84638	3.08301	2.530182	3.161754	0.936822988	0.012018088	0.000000368	-0.543758654
NewGene 1995	--	0.814935	0.762194	1.055735	1.172685	0.715654	0.722882	0.537352	0.328561	0.792386	0.623900885	0.132512351	0.033231068	-0.543404957
ENSG00000150455	TIRAP	2.708672	3.08588	2.415589	2.451974	2.174678	2.0071	1.570558	1.923592	1.835847	0.774468642	-0.076773118	0.008832127	-0.543159704
ENSG00000176381	PRR18	0.670877	0.799652	0.720095	0.525328	0.680352	0.731315	0.475725	0.373999	0.547804	0.979347475	-0.012439205	0.085173682	-0.542625588
ENSG00000166321	NUDT13	1.531026	2.233692	2.144585	1.473351	1.070443	1.673828	0.834374	1.405476	1.447201	0.211577625	-0.339156657	0.038396407	-0.542410127
ENSG00000225973	PIGBOS1	6.294958	5.901278	5.927614	5.917182	6.13716	6.044103	3.532685	3.602621	4.589771	0.503394249	0.149801292	0.008270673	-0.542134499
ENSG00000128242	GAL3ST1	8.062453	6.470313	6.819053	5.894814	6.411367	6.349102	4.54875	4.493217	4.459702	0.891757531	-0.028332338	0.000174702	-0.542066759
ENSG00000141699	RETREG3	9.019343	9.127674	9.036016	8.038455	7.343422	7.920999	5.627052	5.487791	6.332731	0.406305596	-0.083503933	4.95E-10	-0.541728919
ENSG00000123427	EEF1AKMT3	2.360079	2.243148	2.068007	2.357396	2.979444	2.319239	1.465192	1.629985	1.304214	0.41307238	0.165648299	0.004934494	-0.541726466
NewGene 3459	--	2.703183	2.938873	3.245308	3.139919	2.624906	2.819321	1.875552	1.852356	1.99174	0.533715958	0.097474373	0.0000241	-0.541523326
ENSG00000122912	SLC25A16	5.825217	6.310778	8.145196	5.889465	5.247253	6.023996	4.20543	3.855026	4.324374	0.491676078	0.103057637	0.00000748	-0.541125413
ENSG00000183513	COA5	8.630595	10.173221	10.265937	9.808867	9.94636	9.147136	6.082263	6.692753	5.869958	0.336190984	0.129893885	0.0000324	-0.5410312
ENSG00000158711	ELK4	4.114952	5.24637	5.220283	7.04104	6.433819	6.21899	3.199158	3.257608	3.749425	9.88E-11	0.593185192	0.000000331	-0.540949275
ENSG00000043143	JADE2	5.715396	6.182076	5.203415	8.138717	9.676445	9.985722	4.337668	3.827557	3.869822	2.15E-22	0.834399471	4.87E-08	-0.540928909
ENSG00000162613	FUBP1	26.201889	26.510889	25.034293	23.340914	19.020126	22.418471	16.526581	16.574144	16.835343	0.202944614	-0.12009257	1.47E-13	-0.540438779

Gene ID	Gene name	Ctrl-1 FPKM	Ctrl-2 FPKM	Ctrl-3 FPKM	IFNγ-1 FPKM	IFNγ-2 FPKM	IFNγ-3 FPKM	Sora-1 FPKM	Sora-2 FPKM	Sora-3 FPKM	Ctrl vs IFNγ DESeq2 FDR	Ctrl vs IFNγ DESeq2 log2FC	Ctrl vs Sora DESeq2 FDR	Ctrl vs Sora DESeq2 log2FC
ENSG00000169629	RGPD8	1.1263	1.510742	1.197661	1.477456	0.953932	1.503605	0.773001	0.809044	1.031981	0.541689859	0.149596682	0.003567696	-0.540368097
ENSG00000176261	ZBTB8OS	17.96005	16.911867	17.823414	17.854439	18.136781	17.08383	11.351917	12.708013	12.569534	0.317471473	0.118469495	0.000000611	-0.540002451
ENSG00000049246	PER3	1.052337	1.146519	1.18838	1.282032	1.385735	1.369008	0.939743	0.727948	0.698075	0.02751222	0.350501698	0.003224825	-0.539637628
ENSG00000139044	B4GALNT3	1.063905	0.797567	0.747855	0.597823	0.462056	0.569914	0.350155	0.673635	0.556472	0.091996712	-0.437746224	0.047400676	-0.539158876
ENSG00000106366	SERPINE1	22.637114	14.310076	12.873613	4.422646	4.19735	4.220372	9.29844	10.388865	12.538049	1.14E-10	-1.808851219	0.106263996	-0.539143515
ENSG00000284874	AC000093.1	2.772683	2.198048	2.68086	0.785153	1.755242	0.663164	0.801659	1.462256	2.720055	0.041884303	-1.102069453	0.361887097	-0.539085787
ENSG00000118246	FASTKD2	11.461743	12.347886	12.292877	11.296771	11.674547	10.844389	7.862756	7.300075	7.99289	0.685556873	0.044269031	5.21E-10	-0.53898575
ENSG00000263002	ZNF234	2.608232	2.777118	3.121254	2.449644	2.371296	2.128228	1.938728	1.938375	1.851501	0.188012103	-0.212537125	0.000210182	-0.538363478
ENSG00000170776	AKAP13	6.289879	5.911194	5.892404	4.887136	5.853966	5.181503	4.491992	3.770761	4.515262	0.003526444	-0.203369291	4.64E-13	-0.5380763
ENSG00000083097	DOP1A	4.833009	5.16181	4.912861	4.708629	4.492243	4.641955	3.156065	3.142062	3.333022	0.704723294	0.039334652	5.72E-11	-0.537951434
ENSG00000198466	ZNF587	6.114554	6.112389	6.067631	6.24856	6.403894	5.917185	4.262067	3.674018	3.896172	0.016899954	0.188596026	4.18E-10	-0.536716801
ENSG00000186174	BCL9L	6.957371	6.410938	6.123602	6.84374	6.09382	6.172994	4.275583	3.626078	5.205835	0.189918155	0.105267893	5.62E-08	-0.53641522
ENSG00000055917	PUM2	36.449366	35.703198	37.240149	32.246392	32.895009	34.346402	24.658077	21.96207	25.539791	0.94948337	-0.00546102	2.41E-19	-0.536308433
NewGene_4849	--	3.194894	3.378165	3.295303	2.14085	2.053591	2.058811	2.03614	2.035039	2.417304	0.000109527	-0.529967176	0.00016155	-0.536209859
NewGene_1367	--	2.358325	2.372527	1.915567	1.928651	2.042287	2.048593	1.45156	1.224789	1.728483	0.979402462	0.005190769	0.000142091	-0.536175024
ENSG00000154358	OBSCN	0.763343	0.440998	0.413072	0.264647	0.266827	0.233372	0.336336	0.342057	0.297473	0.000155987	-0.687006546	0.003709961	-0.535969847
ENSG00000135631	RAB11FIP5	2.544121	2.605748	2.604153	2.375959	2.195043	1.924646	1.923413	1.752339	1.688694	0.278835108	-0.155446504	0.0000301	-0.535729309
NewGene_1449	--	1.183297	1.408151	1.624258	1.531092	1.306977	1.299125	0.751663	0.99208	0.93466	0.607793847	0.113728005	0.003183968	-0.535282857
ENSG00000247315	ZCCHC3	22.331415	21.115564	20.03665	19.823837	19.074883	19.29204	14.112456	13.928251	12.9877	0.832218166	0.021290311	7.79E-11	-0.535215209
ENSG00000182324	KCNJ14	0.401287	0.366063	0.530321	0.234147	0.273763	0.366775	0.355564	0.235248	0.167095	0.534132284	-0.274504269	0.186853998	-0.535108706
ENSG00000138685	FGF2	0.378603	0.621926	0.633276	0.627133	0.637965	0.583023	0.518743	0.292187	0.406954	0.456228118	0.217566806	0.073751666	-0.534808493
NewGene_4507	--	2.175091	2.443945	2.577775	2.140859	1.689347	2.279805	1.332677	1.797812	1.510266	0.796477871	-0.086114394	0.041566301	-0.534595151
ENSG00000122482	ZNF644	12.068481	13.834155	13.368513	12.192516	11.175276	10.698675	9.588728	8.381946	8.411014	0.412765328	-0.073523961	3.1E-11	-0.534271682
ENSG00000143614	GATAD2B	5.662451	6.115671	6.796215	6.138905	6.190444	6.352859	4.300048	3.939442	3.770851	0.089541161	0.157524991	6.31E-08	-0.534046228
ENSG00000116062	MSH6	30.671538	29.706511	28.393202	30.681823	29.15358	27.503451	17.402839	17.734913	17.471944	0.000224112	0.218828954	9.32E-17	-0.533872885
ENSG00000183688	RFLNB	2.277879	1.988848	2.487777	1.407934	1.338017	1.139935	1.509309	1.214957	1.656845	0.000136241	-0.65457892	0.002270535	-0.533818098
ENSG00000154274	C4orf19	16.19981	16.246342	15.365496	18.114311	18.538124	18.564018	10.998898	9.9447	10.245966	0.00000354	0.366165979	2.14E-08	-0.533613825
ENSG00000162825	NBPF20	0.771242	0.827568	0.896043	0.5858	0.573463	0.556783	0.523303	0.513243	0.531347	0.00622938	-0.348349936	0.0000264	-0.533375607
ENSG00000189007	ADAT2	2.963682	4.042397	4.98193	3.700121	2.833975	3.078213	2.638816	2.605722	2.558506	0.591425036	-0.186259262	0.00081386	-0.533107069
ENSG00000114805	PLCH1	0.656549	0.706713	0.543832	1.074462	0.752448	0.71893	0.462675	0.396519	0.373002	0.010633587	0.563265586	0.024491857	-0.532990988
ENSG00000169914	OTUD3	1.443952	1.744062	1.948898	1.539057	1.363672	1.493883	1.226575	0.86518	1.250242	0.703839571	-0.076027383	0.002036805	-0.532824224
ENSG00000118454	ANKRD13C	4.241124	4.900798	5.126263	4.893048	4.522424	4.866325	3.229901	3.072996	3.063065	0.24867952	0.145184853	0.0000099	-0.531596818
ENSG00000135956	TMEM127	11.386552	10.024442	7.991472	9.365509	9.519368	10.216214	5.45744	6.337627	6.692321	0.246929701	0.116480444	9.45E-09	-0.531469728
ENSG00000124279	FASTKD3	6.458577	6.52835	8.072887	6.808871	7.051244	7.217638	4.667988	4.543626	4.487424	0.218918764	0.167947815	0.0000471	-0.530491525
ENSG00000198846	TOX	1.078264	0.896066	1.004386	0.486874	0.640122	0.55287	0.610414	0.571503	0.75311	0.004646678	-0.676256179	0.021160809	-0.530440519
NewGene_777	--	0.553	0.809346	0.735424	0.727759	0.544024	0.389507	0.490235	0.674047	0.578261	0.355318101	-0.535101523	0.152579239	-0.53024064
NewGene_1338	--	0.594894	0.429364	0.638028	0.397509	0.361093	0.596568	0.265948	0.362415	0.460471	0.784458984	-0.148073115	0.207516429	-0.529630943
ENSG00000175792	RUVBL1	42.940362	47.648856	44.119026	38.000471	35.21617	37.318079	29.605402	30.147273	28.616743	0.03767197	-0.149306292	4.1E-12	-0.529138068
ENSG00000187772	LIN28B	24.476148	27.182709	30.242247	23.685981	23.147657	22.187548	17.279976	17.361116	18.588846	0.265033076	-0.099284205	3.06E-11	-0.529000599
ENSG00000172318	B3GALT1	3.3897	3.23602	3.357008	2.23598	2.080978	2.009719	2.115948	1.800298	2.593211	0.00000453	-0.510461332	0.0000538	-0.528915176
ENSG00000135974	C2orf49	5.081613	6.094978	6.940093	5.578872	5.686095	5.356744	3.921544	4.044609	4.614278	0.952873394	0.010764481	0.0000109	-0.528805061
ENSG00000185869	ZNF829	1.08123	1.344672	1.095362	0.83323	0.822416	0.998928	0.804756	0.511634	0.911996	0.256477613	-0.25214596	0.020157579	-0.52825356
ENSG00000121380	BCL2L14	1.612601	0.864434	1.031337	2.946126	2.132364	2.460704	0.906142	0.787667	0.759415	0.00000418	1.197386259	0.145165248	-0.527617516
ENSG00000266074	BAHCC1	1.340577	1.535664	1.167852	2.163079	1.649888	1.828974	0.906984	0.769457	0.963036	0.00000113	0.604329356	0.000185374	-0.52742675
ENSG00000106346	USP42	9.462904	9.248329	9.151324	14.261493	12.720791	13.156687	6.229421	5.457119	6.493459	1.08E-25	0.669128745	1.64E-10	-0.527297759
ENSG00000107643	MAPK8	5.830577	5.232102	5.354077	5.204061	4.831683	4.428436	4.413709	4.303598	4.083799	0.208542844	-0.127194272	0.000000017	-0.526439435
ENSG00000164574	GALNT10	4.151325	4.023794	3.798006	3.005606	2.596707	3.075741	2.46687	2.56652	2.739496	0.003868861	-0.306052969	0.000000214	-0.52585176
ENSG00000125657	TNFSF9	1.9464	1.614561	2.117958	1.533327	0.902926	1.300891	0.921718	1.279838	1.503111	0.127219372	-0.45987825	0.061140019	-0.525618141
ENSG00000179387	ELMOD2	14.784982	15.989859	16.118965	12.774251	13.083545	13.03963	10.309782	10.25892	10.847636	0.132529022	-0.118108746	1.38E-12	-0.523604719
ENSG00000135720	DYNC1LI2	19.990521	22.737549	23.963342	21.99527	19.698588	18.878014	14.307083	13.532507	15.911898	0.934598105	0.011061646	6.52E-10	-0.523362595
ENSG00000165322	ARHGAP12	11.029034	12.419733	12.520741	16.184639	12.797905	13.237207	7.730707	7.178047	8.074723	0.000000306	0.388591739	4.84E-11	-0.52316766
ENSG00000059145	UNKL	2.799998	3.033881	3.761409	2.157667	2.287718	2.037435	2.568494	1.870803	1.916897	0.006393687	-0.419653138	0.000924494	-0.522960573
ENSG00000175595	ERCC4	2.442453	2.75344	3.042391	2.567708	2.549293	2.630159	2.256082	1.552091	1.760103	0.606710225	0.075207221	0.000226702	-0.522844074
ENSG00000113300	CNOT6	7.807358	8.294108	8.77597	7.583589	7.353753	8.088584	5.391638	4.891625	5.850262	0.759804519	0.033480005	4.5E-10	-0.522822258
ENSG00000181638	ZFP41	1.916355	2.200417	1.949586	2.130498	2.004184	1.942665	1.449979	1.220197	1.450179	0.721412778	0.068537526	0.000590903	-0.522660976
ENSG00000107984	DKK1	327.440521	334.397675	357.020905	152.82225	139.954193	150.226318	204.194061	218.491837	242.955414	6.71E-97	-1.054481162	4.86E-15	-0.522608395
NewGene_2919	--	0.672393	0.780955	0.920197	0.709359	0.648575	0.530434	0.55195	0.525463	0.469543	0.536923396	-0.183936509	0.035517123	-0.522520466
ENSG00000123444	KBTBD4	21.769436	21.999116	22.829943	18.479499	18.268403	19.315521	14.454512	14.224077	14.196417	0.139891828	-0.1161873	2.83E-12	-0.522455653
ENSG00000188277	C15orf62	1.001663	1.352252	1.346016	1.065509	1.284505	1.107538	0.765782	0.778599	0.866889	0.891466941	0.050844011	0.058105529	-0.522400898
ENSG00000164080	RAD54L2	2.036815	2.098306	2.450592	2.3827	2.139373	2.070709	1.618822	1.31982	1.360243	0.237784249	0.147365292	0.0000259	-0.522358231
ENSG00000082701	GSK3B	15.520287	14.929932	15.697693	14.219994	13.617648	13.789843	10.596901	9.629096	10.89132	0.81163759	-0.020656406	1.84E-13	-0.521426026
ENSG00000077585	GPR137B	1.998275	1.946964	1.956779	1.27301	1.369894	1.483105	1.66571	1.133995	1.144229	0.050074156	-0.464708391	0.030517752	-0.520840037
ENSG00000213190	MLLT11	1.217813	1.197992	1.343983	0.943795	0.718004	0.744088	0.843432	0.769228	0.843898	0.020248243	-0.497249506	0.00916757	-0.520606051
ENSG00000101003	GINS1	11.649794	12.48595	13.004009	9.510701	8.367795	11.397429	9.197932	6.974674	8.123137	0.102679601	-0.194211961	0.000000428	-0.520596687
ENSG00000125817	CENPB	25.014158	23.029936	21.287571	22.569048	21.437458	23.461941	13.499213	15.030479	16.850924	0.235875296	0.107734661	1.73E-08	-0.520545562
ENSG00000131779	PEX11B	4.691201	4.606276	5.008275	4.913841	4.912923	5.232734	3.555555	3.005334	2.774261	0.156699786	0.223359014	0.002094913	-0.520485814
NewGene_1960	--	2.150035	1.748308	1.748041	1.216927	1.846222	1.400233	1.231678	1.359894	1.249319	0.612869226	-0.125757601	0.00057287	-0.520268314
ENSG00000100483	VCPKMT	3.973672	4.972886	5.172217	4.773975	4.984599	3.867767	3.144858	2.922232	2.985895	0.676940014	0.093104812	0.002211486	-0.519387042
ENSG00000154040	CABYR	1.941322	2.473556	2.623821	2.853255	2.840208	2.076385	1.462034	1.559584	1.543365	0.194556902	0.327669888	0.037523685	-0.519380709
ENSG00000107929	LARP4B	10.598369	10.262452	11.383848	10.42406	11.519898	10.13975	7.261526	6.313348	8.872634	0.261162201	0.086935617	1.42E-08	-0.519370838
ENSG00000111331	OAS3	0.795145	0.724675	0.831919	13.0563	11.410675	11.642861	0.602435	0.414822	0.491003	0	4.188260247	0.031303111	-0.518637386
ENSG00000154127	UBASH3B	3.096159	3.299624	4.746604	0.436664	0.442273	0.429978	2.04723	2.343356	2.920254	7.72E-77	-2.937729041	0.001256485	-0.518402606
ENSG00000137561	TTPA	0.618035	0.754095	0.816341	0.524195	0.421265	0.585819	0.557382	0.509564	0.349099	0.372538841	-0.358007567	0.166794483	-0.518402109
ENSG00000115207	GTF3C2	27.815766	26.671739	28.164881	20.905189	20.29294	20.779084	16.906464	17.505033	19.608636	0.0000141	-0.274328684	9.11E-13	-0.51822725

Gene ID	Gene name	Ctrl-1 FPKM	Ctrl-2 FPKM	Ctrl-3 FPKM	IFNγ-1 FPKM	IFNγ-2 FPKM	IFNγ-3 FPKM	Sora-1 FPKM	Sora-2 FPKM	Sora-3 FPKM	Ctrl vs IFNγ DESeq2 FDR	Ctrl vs IFNγ DESeq2 log2FC	Ctrl vs Sora DESeq2 FDR	Ctrl vs Sora DESeq2 log2FC
ENSG00000180901	KCTD2	9.418587	7.74499	8.794477	7.780555	8.314024	7.844647	5.638421	5.228775	6.145431	0.837948229	0.029037297	0.000000475	-0.518068311
ENSG00000137393	RNF144B	2.061818	2.547147	2.861274	1.692377	1.618811	1.696259	1.781714	1.425576	1.681612	0.004824934	-0.427664326	0.000945983	-0.51803646
ENSG00000188315	C3orf62	1.758174	2.101235	1.977876	1.598834	1.574814	1.323691	1.311862	1.017537	1.503718	0.194669345	-0.236697236	0.003479286	-0.517700413
ENSG00000091656	ZFHX4	2.264145	2.113169	2.064502	2.32972	2.263189	2.22402	1.546768	1.284413	1.528232	0.036952249	0.194556028	6.63E-08	-0.517596657
ENSG00000102053	ZC3H12B	0.248713	0.306923	0.297558	0.266179	0.330197	0.247772	0.191084	0.135766	0.232976	0.742322054	0.128725378	0.12264861	-0.517403451
ENSG00000183196	CHST6	0.212271	0.38464	0.277425	0.08305	0.094666	0.092836	0.180785	0.133116	0.187516	0.000382943	-1.304734759	0.139889068	-0.516965333
ENSG00000235098	ANKRD65	1.7971	1.340422	1.446792	1.979196	2.004834	2.931228	1.207537	0.872687	1.043648	0.016435074	0.604681639	0.080752833	-0.516933497
ENSG00000187372	PCDHB13	0.368916	0.290414	0.320942	0.210851	0.223761	0.176252	0.192769	0.170749	0.27654	0.162625882	-0.542192265	0.165763051	-0.51691085
ENSG00000198885	ITPRIPL1	4.012508	3.705286	3.488359	2.90581	3.467992	2.713776	2.49591	2.34761	2.888781	0.152316246	-0.204838452	0.000144596	-0.516695215
ENSG00000155893	PXYLP1	2.644226	2.402851	2.601976	3.105864	2.614415	2.786379	1.827258	1.714734	1.522967	0.030126136	0.326102036	0.001605809	-0.516496106
ENSG00000164073	MFSD8	4.643737	4.937259	3.931069	4.834946	5.135352	5.123927	3.395161	2.880919	2.748295	0.00097653	0.341034064	0.00000663	-0.51647574
ENSG00000166167	BTRC	2.388164	2.438643	2.18572	2.396849	2.380359	2.370971	1.388773	1.650703	1.524993	0.178364828	0.172161874	0.000102412	-0.516427334
ENSG00000048140	TSPAN17	6.867315	7.605138	6.421543	8.869655	7.547007	7.982769	4.787708	4.327792	4.380418	0.000657338	0.375311131	0.0000275	-0.516361303
ENSG00000075407	ZNF37A	1.509281	1.441447	1.942972	2.203681	2.337678	1.760304	0.981293	1.227136	1.247495	0.005309853	0.393249414	0.000880684	-0.516007643
ENSG00000151240	DIP2C	1.555921	1.520374	1.458307	1.324137	1.283502	1.344677	1.241314	0.917698	1.028492	0.750219939	-0.054907367	0.000101254	-0.515844674
ENSG00000110987	BCL7A	12.623655	11.929173	12.172237	8.837644	8.16838	8.314222	8.199366	7.577538	9.371434	8.45E-10	-0.415500568	1.96E-10	-0.515604806
ENSG00000092820	EZR	41.789513	43.926043	42.92218	38.259378	37.852331	38.620733	28.833076	27.972833	27.463535	0.835045222	-0.01693605	5.17E-15	-0.5154605
ENSG00000008083	JARID2	7.440688	7.282702	7.44642	7.554275	7.98753	7.613346	5.116426	4.711742	4.684673	0.008711064	0.204454572	1.47E-09	-0.515362875
ENSG00000101605	MYOM1	0.532711	0.434455	0.579745	0.366555	0.292724	0.433999	0.360401	0.333933	0.315491	0.134439294	-0.46377342	0.057708278	-0.514399956
ENSG00000142686	C1orf216	2.501501	2.835202	2.525016	1.655036	1.54174	1.892996	1.650475	1.582157	1.929929	0.003982575	-0.476586171	0.001371752	-0.514087327
ENSG00000125945	ZNF436	2.451129	2.061796	1.944411	1.934277	2.140069	1.806922	1.291031	1.381087	1.563854	0.987497051	0.00384375	0.002146226	-0.513372202
ENSG00000135373	EHF	0.645439	0.82215	0.881624	0.372609	0.719715	0.376821	0.47961	0.470949	0.66296	0.016525672	-0.795688533	0.09745921	-0.513211871
ENSG00000127824	TUBA4A	3.70866	2.905175	2.823888	2.103063	1.197921	1.491256	1.934485	2.359535	1.927701	0.000124525	-0.843578298	0.010092517	-0.512787238
ENSG00000185278	ZBTB37	1.173693	1.504821	1.346151	1.412807	1.077258	1.934738	0.858546	0.778584	1.086292	0.803529716	-0.35008016	0.0000527	-0.512252295
ENSG00000062370	ZNF112	2.268634	1.869212	2.141793	1.818459	2.146016	1.825365	1.502759	1.392261	1.238059	0.927540381	0.023359432	0.004215923	-0.511790956
ENSG00000221886	ZBED8	0.939738	1.555958	1.346879	0.773086	0.767473	0.771648	0.676394	0.885303	0.960233	0.03553293	-0.578794523	0.065494505	-0.511763977
ENSG00000167552	TUBA1A	19.611917	23.640265	17.341757	17.214329	12.149073	11.970105	17.50522	13.306291	12.432501	2.23E-08	-0.498361533	0.000000409	-0.511749871
ENSG00000173914	RBM4B	31.432112	32.516115	29.604662	28.424638	27.614862	27.883467	19.060432	20.164582	23.141322	0.975527107	0.00383889	6.16E-09	-0.51152955
ENSG00000183530	PRR14L	4.189672	4.543797	4.292902	4.281526	4.05064	4.483881	2.885088	2.772606	3.00075	0.142138153	0.128269769	1.7E-10	-0.51152635
ENSG00000170571	EMB	0.459806	0.697899	0.704693	0.549136	0.401442	0.316003	0.492794	0.32456	0.408692	0.235903016	-0.416939282	0.103702224	-0.511338924
NewGene_2929	--	0.665713	0.937855	0.830036	0.386791	0.553003	0.683806	0.45524	0.611185	0.606685	0.189343735	-0.46065615	0.099138672	-0.511239627
ENSG00000164742	ADCY1	0.345691	0.502628	0.371204	0.486608	0.508975	0.442769	0.353299	0.232579	0.343196	0.025929109	0.442100325	0.030983783	-0.510446031
ENSG00000009780	FAM76A	0.787726	1.070323	1.366675	0.918151	1.051116	0.963906	0.953676	0.841147	0.403451	0.979566581	0.010180672	0.085010155	-0.510204459
ENSG00000183397	C19orf71	2.355023	2.308906	1.92454	2.048392	1.837309	1.957398	1.296649	1.108307	1.95013	0.943080528	-0.037126731	0.197737196	-0.509930129
ENSG00000181418	DDN	1.338639	1.404931	2.052422	1.964733	2.185744	1.857637	0.909648	1.003147	1.244934	0.017969466	0.473288382	0.024220875	-0.509715765
NewGene_3519	--	0.851108	1.172325	0.940277	1.067178	0.753859	0.723272	0.820645	0.723538	0.677053	0.871240463	-0.059453337	0.048222044	-0.509158513
ENSG00000188171	ZNF626	2.847572	2.584814	2.132309	2.126242	2.253023	2.375764	3.949252	2.873488	2.466929	0.409919267	-0.155501993	0.006235389	-0.508894018
ENSG00000178184	PARD6G	3.865984	3.872889	3.467054	3.113122	3.10772	3.229813	2.224884	2.453352	2.912814	0.463025823	-0.11115565	0.000183719	-0.508533475
ENSG00000184371	CSF1	3.523801	2.729279	2.626188	11.850066	11.155258	11.707194	1.805443	1.917302	2.307383	5.95E-95	2.132344429	0.002579816	-0.507992615
ENSG00000136848	DAB2IP	13.267626	11.455729	11.419101	14.9518	15.536625	16.400409	7.908951	7.017797	9.363522	3.46E-14	0.538268643	9.46E-08	-0.507625875
ENSG00000185963	BICD2	8.652086	8.686449	8.752988	6.760966	6.467792	7.015288	5.200272	5.475636	6.52423	0.002201609	-0.230115993	2.23E-08	-0.507408805
ENSG00000197142	ACSL5	0.652336	0.581611	0.337792	5.9844	5.251434	5.51141	0.343054	0.38511	0.298498	1.41E-105	3.540201665	0.190269743	-0.507276971
ENSG00000167523	SPATA33	1.259247	1.738585	1.47484	1.698773	1.007431	1.313894	0.817914	0.939315	1.282999	0.790366517	-0.093296191	0.063230454	-0.507100431
ENSG00000119661	DNAL1	0.668533	0.81122	0.685658	0.523914	0.488542	0.636404	0.52476	0.609801	0.515318	0.282819996	-0.241582407	0.012020207	-0.506587332
ENSG00000149781	FERMT3	0.743747	0.4822	0.373831	0.714314	0.388452	0.34676	0.249749	0.406295	0.454013	0.979402462	-0.017810502	0.282418787	-0.506433541
ENSG00000103064	SLC7A6	9.141918	8.678445	11.949421	6.513942	7.089525	5.515985	6.713155	6.122669	6.676915	0.05374339	-0.453319954	0.000012	-0.506377042
ENSG00000119042	SATB2	0.728315	1.034614	0.850296	0.726913	0.76017	0.721519	0.594376	0.484844	0.63918	0.69745326	-0.107319257	0.024011464	-0.506155835
ENSG00000066739	ATG2B	2.371046	2.239213	2.412514	2.057405	2.169173	2.045399	1.461503	1.473732	1.706668	0.903882386	-0.015700829	6.66E-08	-0.505848468
ENSG00000135211	TMEM60	20.35215	19.189613	20.404617	14.378369	14.737158	13.56327	13.173773	13.26686	13.115224	0.001183952	-0.343136733	0.00000223	-0.505716033
ENSG00000181826	RELL1	8.589972	6.994773	7.223502	5.250982	6.353397	5.379172	5.265889	4.281301	5.529803	0.102165891	-0.277430845	0.00132514	-0.505595154
ENSG00000177125	ZBTB34	4.817548	5.085625	5.252766	4.226054	4.007559	3.823386	3.303746	3.021304	3.707114	0.047255609	-0.1851398	7.75E-08	-0.505367952
ENSG00000167562	ZNF701	2.74691	3.308425	3.248566	2.223813	2.440063	2.480854	2.088043	2.189277	2.232481	0.054056011	-0.249225492	0.0000504	-0.505304396
ENSG00000263715	NC02210-CRHF	1.197872	0.667858	0.986663	0.910288	0.749906	0.715298	0.746315	0.539191	0.601502	0.766058623	-0.120748725	0.120492158	-0.505271553
ENSG00000167548	KMT2D	5.811532	5.95921	5.40467	6.906908	5.532866	5.770638	3.565858	3.370509	4.229815	0.004339633	0.226765435	2.66E-10	-0.505149787
ENSG00000263956	NBPF11	4.85735	5.15496	4.395522	4.916881	3.962658	3.908424	3.638778	2.73466	3.457983	0.381979679	-0.114158333	0.00000443	-0.50502571
ENSG00000120798	NR2C1	13.680548	13.350891	14.875047	12.628232	13.518278	11.932318	9.772791	8.962361	9.131284	0.999202724	0.000134113	7.02E-10	-0.504490186
ENSG00000123416	TUBA1B	903.51436	887.917333	828.861668	647.421071	603.487907	652.045981	553.483839	604.766169	571.259703	0.000000001	-0.313948492	3.06E-14	-0.504088037
ENSG00000006210	CX3CL1	17.857088	14.458936	15.066739	11.339607	11.998218	11.957402	9.652419	10.051565	11.648369	0.00447359	-0.274284901	0.00000051	-0.503364889
ENSG00000189190	ZNF600	2.801879	2.952065	3.225271	2.426043	2.356799	2.266299	1.971522	1.909714	2.185373	0.187822677	-0.204719261	0.000440241	-0.503000171
NewGene_937	--	1.324036	1.856548	1.933251	0.820448	1.293829	1.362627	1.30625	1.404737	1.349217	0.075128765	-0.534437564	0.066007523	-0.502876768
ENSG00000171823	FBXL14	6.061624	5.442263	4.387857	4.055758	3.574349	3.839874	3.262248	3.461491	3.794246	0.048571078	-0.324689641	0.001570721	-0.50225655
ENSG00000101751	POLI	2.299326	2.094686	1.964313	2.10985	2.181811	2.014312	1.232748	1.004623	1.484552	0.490611698	0.117742351	0.001036086	-0.50214534
ENSG00000167895	TMC8	3.896906	2.675547	2.514552	0.831791	0.639282	0.649031	1.670282	2.104522	2.314656	8.04E-29	-2.04865729	0.006326792	-0.502010999
ENSG00000134086	VHL	25.646365	25.242323	22.469067	21.339613	21.419172	21.714616	16.102318	15.004766	17.172792	0.633048625	-0.044081128	9.14E-12	-0.501933823
NewGene_1472	--	1.344864	1.565149	1.52982	1.069477	1.617499	1.432579	0.938402	0.952164	1.13149	0.90346123	-0.046243937	0.061059664	-0.501816952
ENSG00000135837	CEP350	6.009253	5.941153	5.925527	6.207729	5.771597	5.683153	3.871649	3.725342	4.155301	0.017793067	0.160210803	4.58E-12	-0.501583654
ENSG00000119900	OGFRL1	4.726982	5.71963	5.842192	3.878655	3.384175	3.587869	3.650824	3.195015	3.953948	0.00000363	-0.436352318	0.000000759	-0.501581221
ENSG00000135374	ELF5	5.645717	5.595799	8.131179	3.761704	4.331796	3.514645	3.873214	3.820316	5.169112	0.000265876	-0.597395103	0.003241211	-0.501208413
ENSG00000137124	ALDH1B1	43.773192	39.258604	41.074295	21.546211	24.121303	23.931091	26.863837	26.076123	29.955671	1.28E-23	-0.692344077	4.64E-13	-0.50113995
ENSG00000174327	SLC16A13	10.28513	8.982755	8.266981	10.12496	11.716077	11.380512	6.044261	5.827413	6.389728	0.000516222	0.419047609	0.0000829	-0.501085487
ENSG00000101544	ADNP2	10.608727	11.202206	11.443989	10.431344	10.472408	10.706031	8.612049	6.626644	7.181091	0.398669696	0.070629665	0.000000168	-0.501075381
ENSG00000160131	VMA21	10.910536	13.88617	14.280065	12.117867	11.167816	12.668904	8.314414	8.009992	9.718196	0.90346123	0.018101211	0.00000207	-0.500820078
ENSG00000180263	FGD6	2.835354	3.362723	3.586364	2.552351	2.260471	2.537567	2.441708	2.083204	2.044477	0.063276855	-0.213333267	0.00000794	-0.500236081
ENSG00000145016	RUBCN	2.518275	2.073896	2.745389	2.545886	2.396552	2.369033	1.673483	1.570987	1.918287	0.588783885	0.088999957	0.000347013	-0.500105287

Gene ID	Gene name	Ctrl-1 FPKM	Ctrl-2 FPKM	Ctrl-3 FPKM	IFNγ-1 FPKM	IFNγ-2 FPKM	IFNγ-3 FPKM	Sora-1 FPKM	Sora-2 FPKM	Sora-3 FPKM	Ctrl vs IFNγ DESeq2 FDR	Ctrl vs IFNγ DESeq2 log2FC	Ctrl vs Sora DESeq2 FDR	Ctrl vs Sora DESeq2 log2FC
ENSG00000118200	CAMSAP2	8.291137	9.320693	10.236532	8.32679	7.151216	7.869032	5.965956	5.618647	6.925478	0.475704677	-0.079252766	4.58E-08	-0.499478898
ENSG00000173530	TNFRSF10D	14.164738	12.920426	13.249304	3.257388	3.345934	3.389133	8.623588	8.792808	9.345111	2.37E-109	-1.864690831	8.81E-10	-0.499426716
NewGene_3673	--	0.478027	0.568101	0.541492	0.491314	0.466305	0.480204	0.229982	0.310579	0.512702	0.983443072	0.009338283	0.160799448	-0.499354744
ENSG00000117335	CD46	110.452293	115.220163	114.421844	116.871994	115.897602	109.786849	71.069708	71.861657	85.64266	0.004574451	0.146090757	1.89E-14	-0.499111979
ENSG00000196230	TUBB	1612.98569	1543.571685	1488.562237	1153.580223	1102.122772	1163.445342	1021.009078	1013.880801	1045.640068	8.8E-10	-0.294172486	2.79E-20	-0.499088137
ENSG00000160190	SLC37A1	2.743811	2.649873	2.404396	4.516928	3.459305	4.048263	1.658031	1.585008	2.04352	2.97E-08	0.743970559	0.001569328	-0.498906518
ENSG00000267216	AC020915.1	0.570801	0.698262	0.446582	0.460229	0.690792	0.333253	0.460295	0.474396	0.194035	0.894098044	-0.064450723	0.19473582	-0.498741641
ENSG00000183665	TRMT12	4.757795	6.162713	4.883529	5.135443	5.241796	5.8839	3.211838	3.370004	3.923543	0.239044778	0.191443521	0.001243051	-0.4987069
ENSG00000023516	AKAP11	3.359984	3.37496	3.632328	2.959482	2.977009	2.860023	2.330549	2.172887	2.379685	0.378310671	-0.089190391	1.65E-08	-0.498253823
ENSG00000158480	SPATA2	2.299417	2.510073	2.565013	2.571549	2.55845	2.595156	1.575485	1.527845	1.750295	0.140782023	0.213739461	0.000641323	-0.498066894
ENSG00000100665	SERPINA4	2.067805	1.817357	1.151451	1.061616	1.108691	0.867386	1.072508	1.165574	1.056589	0.056582493	-0.575941627	0.082374789	-0.497241663
ENSG00000042429	MED17	6.476645	4.257363	5.416044	5.468225	5.517641	5.66957	3.132106	3.125945	3.786043	0.657926355	-0.060139793	0.00000377	-0.497202043
ENSG00000100523	DDHD1	1.798866	2.174114	1.989971	2.159538	2.011348	2.037805	1.016402	1.437433	1.378302	0.200654605	0.170315888	0.0000796	-0.4970092
ENSG00000166526	ZNF3	37.301077	34.503094	33.774641	29.431668	29.259103	27.759771	23.833926	22.914847	23.274063	0.080190699	-0.124249266	2.06E-12	-0.496977875
NewGene_6725	--	0.643503	0.404695	0.667205	0.572449	0.376226	0.447981	0.289661	0.342632	0.187014	0.899969632	-0.069679585	0.256247748	-0.496774979
NewGene_2501	--	0.806665	0.482401	0.511906	1.218539	1.076919	1.103096	0.362635	0.333939	0.498839	0.000701184	1.047571976	0.255331237	-0.496705215
ENSG00000285043	AC093512.2	33.444589	29.262692	24.76256	24.084955	18.278417	21.954446	17.314021	18.211242	22.725721	0.005701575	-0.307768721	0.00000409	-0.496593427
NewGene_699	--	10.164916	6.390687	9.447203	4.1403	4.695136	5.604065	4.749877	6.711461	7.645514	8.57E-13	-0.843397232	0.000251553	-0.49652267
ENSG00000187959	CPSF4L	3.296832	3.418731	2.814538	1.337925	1.69406	0.968475	2.327943	2.161751	2.161598	0.000343844	-1.173054431	0.100106976	-0.496325563
ENSG00000137204	SLC22A7	0.713199	0.510756	0.484221	0.680093	0.735633	0.45962	0.382852	0.398927	0.357949	0.628580881	0.22944642	0.242087514	-0.496156934
ENSG00000168672	FAM84B	0.639158	0.608783	0.647853	0.497793	0.521515	0.475801	0.487874	0.318786	0.458335	0.497331135	-0.192460923	0.055229523	-0.496028902
ENSG00000100650	SRSF5	96.982035	88.182306	89.615735	91.40128	85.458357	88.604671	58.635287	57.054451	60.296169	0.20057796	0.079988552	1.5E-13	-0.495799806
ENSG00000135378	PRRG4	3.187222	3.199413	3.304251	5.241476	5.576195	5.739586	2.279506	2.149323	2.0025	2.82E-26	0.92030042	0.0000236	-0.495795521
ENSG00000004139	SARM1	1.006539	1.215288	0.788965	0.655456	1.199484	0.821898	0.54606	0.729866	0.760886	0.879330396	-0.088385841	0.011641524	-0.495791956
ENSG00000284946	AC068831.7	10.842607	12.356807	9.378288	10.913908	6.007315	9.610651	10.778183	5.898496	6.391534	0.684290694	-0.169294913	0.186993156	-0.495689181
ENSG00000166938	DIS3L	5.445662	5.698894	5.711035	6.25857	5.31018	5.690123	4.137267	3.552293	3.702375	0.156657351	0.159282656	0.0000188	-0.495538434
NewGene_3110	--	1.064882	1.819213	1.79038	2.134749	1.532006	1.507269	1.189644	0.850927	1.265835	0.92320945	0.038956242	0.1022273	-0.495382725
ENSG00000165434	PGM2L1	0.177767	0.221745	0.22336	0.115112	0.123922	0.07508	0.118885	0.159173	0.133179	0.027341192	-0.840902044	0.165214126	-0.495305357
ENSG00000057019	DCBLD2	10.4232	11.377617	11.852138	9.291099	9.507024	8.981808	7.313673	7.246019	7.907828	0.102965083	-0.131580421	9.26E-11	-0.494709874
ENSG00000089723	OTUB2	0.72061	0.502086	0.535171	0.58943	0.45722	0.381786	0.315265	0.355545	0.581137	0.821895035	-0.094647876	0.123181098	-0.494571893
ENSG00000104490	NCALD	2.947663	3.500815	3.324933	3.432149	2.915845	2.860775	2.600695	2.169782	2.586088	0.979353403	0.006089659	0.001318789	-0.494199972
NewGene_983	--	0.689126	0.628515	0.610932	0.479733	0.394899	0.498641	0.505021	0.39102	0.464805	0.10997774	-0.319640676	0.007073778	-0.494086774
ENSG00000124789	NUP153	52.565739	56.966471	59.053434	48.057108	43.423231	47.360913	38.179773	33.422077	38.501355	0.138566588	-0.09543844	2.59E-15	-0.493941699
ENSG00000198794	SCAMP5	8.964128	10.521117	7.725608	6.157838	7.153367	6.336345	5.146935	5.450009	6.221874	0.045848947	-0.228480818	0.00000639	-0.493912185
ENSG00000128965	CHAC1	7.570971	6.90927	9.074777	10.199195	12.880244	12.095494	4.741825	4.856361	6.396658	5.93E-08	0.699616073	0.001458445	-0.493718608
ENSG00000167470	MIDN	45.833376	41.842752	38.051181	38.021298	36.323675	41.587604	26.694459	25.893924	31.248055	0.778275433	0.029906569	4.54E-10	-0.493711173
ENSG00000015568	RGPD5	1.905531	2.134973	2.485991	1.755327	1.512045	1.733655	1.656206	1.205627	1.30558	0.290161002	-0.186644627	0.005478904	-0.493451815
ENSG00000100413	POLR3H	11.258395	11.815636	12.32385	10.763935	10.334992	10.895745	7.783622	6.952408	8.656736	0.654890292	0.047658374	8.53E-08	-0.493325957
ENSG00000213762	ZNF134	5.258398	5.360662	5.814012	4.377865	4.724517	4.50388	3.502242	3.541023	3.878513	0.256355123	-0.124967964	0.000000555	-0.493218214
ENSG00000130590	SAMD10	2.756514	2.892713	2.63126	2.371248	2.00403	2.292474	1.797113	1.676628	1.82689	0.757381357	-0.077847439	0.011962195	-0.493084862
ENSG00000180773	SLC36A4	4.22101	5.074591	5.846662	4.760899	4.703522	4.735554	3.259167	3.073675	3.866495	0.530057555	0.093510168	0.000222059	-0.49308019
NewGene_1025	--	0.979649	0.908832	0.971244	0.754369	0.760441	0.582117	0.59362	0.780165	0.639929	0.475957534	-0.298039113	0.187838204	-0.493076632
ENSG00000180884	ZNF792	5.193158	4.468678	4.250193	3.901878	3.750753	3.478231	2.393254	3.124425	3.833186	0.185182843	-0.18044758	0.001639581	-0.492657138
ENSG00000114491	UMPS	14.296625	17.842692	13.668737	12.578996	14.063369	14.104307	10.612139	10.631033	9.885139	0.371759649	-0.069819665	1.03E-11	-0.492456436
ENSG00000077092	RARB	2.085809	2.023787	2.352861	1.066747	1.211294	1.269783	1.30255	1.41275	1.442913	0.000291517	-0.679127644	0.00703999	-0.492394967
ENSG00000123836	PFKFB2	9.138699	9.203427	9.108762	10.265216	10.13801	9.636688	6.30789	5.960662	6.448455	0.0000129	0.28756071	2.08E-11	-0.49224671
ENSG00000139436	GIT2	8.403093	7.195189	7.87742	9.277251	8.299048	7.768396	6.094583	5.642996	6.286298	0.00414122	0.264455386	6.43E-08	-0.491697772
NewGene_3396	--	0.562049	0.52716	0.582726	0.913413	0.690904	0.767707	0.425873	0.341067	0.39287	0.057803585	0.611545516	0.193847924	-0.491592496
ENSG00000074621	SLC24A1	5.569731	4.785097	6.327279	6.858423	4.067671	3.498616	4.14777	3.747892	4.220032	0.254482865	-0.148551218	0.000101109	-0.491430044
ENSG00000177426	TGIF1	11.038579	8.911601	10.582481	9.535996	9.08397	9.196922	5.828426	7.114684	7.39335	0.784458984	-0.041215769	0.0000767	-0.491253014
ENSG00000136379	ABHD17C	2.70963	3.075425	2.445167	2.496683	2.424361	2.426578	1.95091	1.544534	2.010232	0.95047057	-0.016645075	0.009838883	-0.491120972
ENSG00000142149	HUNK	1.346647	1.222446	1.170312	0.745811	0.880827	0.787284	0.896661	0.710113	0.902871	0.001131226	-0.506671613	0.002179826	-0.490469816
ENSG00000187792	ZNF70	0.539049	0.540193	0.501482	0.655896	0.547593	0.571447	0.424416	0.270601	0.360296	0.169424095	0.313903218	0.051756798	-0.490260912
ENSG00000158321	AUTS2	2.380216	2.208702	2.597015	1.875327	1.311381	1.679737	1.929752	1.247352	1.415141	0.007583736	-0.414556683	0.002275167	-0.490230305
ENSG00000160685	ZBTB7B	13.820676	13.002576	12.805967	13.378697	15.051523	13.525798	8.804514	8.164078	9.734847	0.005869758	0.218848649	6.9E-09	-0.490063961
ENSG00000166181	API5	41.19497	43.135372	43.000445	41.854824	45.461688	38.001121	27.441104	23.380823	29.919713	0.152118102	0.114055465	3.95E-09	-0.489652041
ENSG00000176108	CHMP6	14.167999	12.863001	11.202781	11.297739	11.250816	11.91503	8.996165	7.926411	8.788463	0.99155889	0.002083274	0.0000445	-0.489503335
ENSG00000136040	PLXNC1	2.456122	2.240448	2.626421	1.799484	1.389125	1.422995	1.741855	1.407607	1.87052	0.00000906	-0.550025717	0.0000826	-0.489456466
ENSG00000180998	GPR137C	2.151689	2.886149	3.479167	1.828158	1.889021	2.092564	1.985827	1.891984	1.920409	0.054791375	-0.364620011	0.007678789	-0.489202546
ENSG00000103034	NDRG4	1.012511	0.871385	0.991011	0.699014	0.736497	0.976551	0.753883	0.54371	0.680031	0.653163359	-0.149457216	0.085173682	-0.488939346
ENSG00000114790	ARHGEF26	8.387848	8.349671	8.314247	7.034827	6.11678	6.267782	6.074382	4.989613	6.283109	0.010098882	-0.218515744	4.09E-08	-0.488866229
ENSG00000124568	SLC17A1	0.959144	1.708203	0.569134	0.687269	0.689442	0.369322	0.731235	0.673858	0.71639	0.141403354	-0.693244055	0.243329613	-0.488674805
ENSG00000178409	BEND3	4.375112	4.898167	4.863812	4.008682	3.547545	3.956642	3.332067	2.8674	3.308499	0.102275762	-0.172463309	0.000000799	-0.48863125
ENSG00000054967	RELT	1.626574	1.459116	1.72277	0.940867	1.141033	0.832311	1.032151	1.086212	1.038583	0.000693318	-0.679268847	0.013055816	-0.488487207
ENSG00000183354	KIAA2026	4.654205	4.911693	4.535342	4.79227	5.480843	5.39612	3.47474	2.826205	4.056189	0.409313246	0.083920554	0.000000174	-0.488241349
ENSG00000132424	PNISR	26.611363	28.989775	30.050756	29.044554	25.811164	26.14473	18.095854	18.596863	19.659353	0.5522711	0.05103517	2.82E-12	-0.487964601
ENSG00000215790	SLC35E2A	4.348379	4.407434	4.605875	3.715838	3.459387	3.315708	2.950937	2.90595	2.905535	0.055534699	-0.196612582	0.000000801	-0.487915617
ENSG00000131069	ACSS2	12.729965	9.61777	9.427181	26.498607	29.354043	27.354643	7.178212	7.790301	6.562478	6.51E-54	1.506090641	0.00020808	-0.487597532
ENSG00000283201	AC092329.3	0.668114	0.771456	0.804454	0.709057	0.573608	0.57552	0.678771	0.346747	0.476385	0.814736115	-0.114413728	0.239464512	-0.487538399
ENSG00000166086	JAM3	0.755846	0.795118	0.794007	0.49487	0.4946	0.470918	0.467396	0.384744	0.732487	0.044466102	-0.535468914	0.094959006	-0.487392086
ENSG00000212747	RTL8B	10.658073	9.968731	9.09535	9.365881	8.850474	10.602614	6.695303	7.16762	5.978665	0.45330249	0.104236259	0.000113655	-0.486569992
ENSG00000164023	SGMS2	0.912934	1.146001	1.247729	1.028467	0.845362	1.077896	0.728256	0.691076	0.833691	0.998214326	0.001018544	0.010673189	-0.486516756
ENSG00000159792	PSKH1	11.20846	10.312263	8.815051	8.606552	8.962914	9.271358	6.66688	6.626872	7.053906	0.818282535	-0.033335357	0.0000029	-0.485909617
ENSG00000181827	RFX7	5.224059	5.772206	5.822061	5.930347	5.496849	5.451156	3.92649	3.403117	3.92297	0.049603028	0.153525447	2.63E-09	-0.485904462

Gene ID	Gene name	Ctrl-1 FPKM	Ctrl-2 FPKM	Ctrl-3 FPKM	IFNγ-1 FPKM	IFNγ-2 FPKM	IFNγ-3 FPKM	Sora-1 FPKM	Sora-2 FPKM	Sora-3 FPKM	Ctrl vs IFNγ DESeq2 FDR	Ctrl vs IFNγ DESeq2 log2FC	Ctrl vs Sora DESeq2 FDR	Ctrl vs Sora DESeq2 log2FC
ENSG00000148677	ANKRD1	36.882568	36.437153	48.143864	5.40397	6.73677	6.770521	29.984209	27.458055	23.70472	9.87E-117	-2.53230432	0.0000443	-0.485896987
ENSG00000106692	FKTN	3.443473	5.475873	3.692844	3.777381	5.825906	4.22625	2.635957	2.733883	3.648213	0.065028043	0.189872114	0.00000506	-0.48562791
ENSG00000143322	ABL2	4.509202	5.032183	4.860089	4.888386	4.655151	4.648543	4.130056	3.328805	3.510528	0.1166117	0.126718744	0.00000208	-0.485507769
ENSG00000104093	DMXL2	2.742968	3.207246	3.266873	2.397363	2.124915	1.960234	2.286742	1.862702	1.924758	0.004222917	-0.337797929	0.0000231	-0.48532952
NewGene_4907	--	4.105238	4.026826	5.069654	4.600933	4.800026	4.267741	3.217411	2.636346	2.979096	0.392153048	0.19809237	0.026352576	-0.485217174
ENSG00000152147	GEMIN6	4.936174	4.74235	2.982906	1.810384	3.300466	2.999223	3.934048	3.056911	3.057151	0.050294072	-0.313870957	0.001837659	-0.485183755
ENSG00000093000	NUP50	32.574246	33.421932	36.375944	33.043653	31.80687	32.594814	22.744186	22.699019	23.931617	0.315145953	0.073631868	5.39E-13	-0.484192491
ENSG00000196935	SRGAP1	0.080164	0.068407	0.06458	0.029707	0.037371	0.051291	0.034132	0.066898	0.070041	0.087150779	-0.685734589	0.223859242	-0.484184727
ENSG00000204618	RNF39	2.742349	2.641861	2.606436	2.309231	2.215429	1.832813	1.537449	1.728623	1.910906	0.56498133	-0.138041308	0.013573176	-0.484065478
ENSG00000173706	HEG1	5.280297	5.564851	5.851942	8.258732	7.490526	7.683009	3.781107	3.383247	3.978585	8.52E-18	0.62359149	2.12E-08	-0.483217846
ENSG00000153898	MCOLN2	0.488314	0.408868	0.527078	0.249615	0.400439	0.283601	0.247279	0.369822	0.308471	0.377796517	-0.410567104	0.248506366	-0.48303969
ENSG00000154114	TBCEL	4.427068	4.733776	5.349034	4.538727	4.676021	4.496476	3.380204	3.315918	3.148243	0.575380682	0.076837193	0.0000311	-0.482974901
ENSG00000221867	MAGEA3	0.811007	0.8368	0.507589	1.01438	0.674487	0.757772	0.436996	0.401862	0.601324	0.507660303	0.304414701	0.272439789	-0.482831829
ENSG00000204634	TBC1D8	22.631691	22.524694	24.74843	25.585656	22.897583	21.364898	16.13145	13.836443	17.262486	0.10565317	0.137756162	1.34E-09	-0.482584528
ENSG00000109189	USP46	4.10775	4.390943	4.963461	4.710354	4.315664	4.542746	2.982707	2.735068	3.884672	0.057531968	0.176597102	0.000000649	-0.482330191
ENSG00000184743	ATL3	5.548573	7.183879	6.641357	6.772489	6.596319	6.672639	4.51095	3.865905	4.401161	0.014156312	0.223786229	0.00000113	-0.482285063
ENSG00000178974	FBXO34	7.381072	7.602524	8.078655	6.712769	6.024995	6.143793	5.412979	4.996888	5.155926	0.178740219	-0.142981191	0.000000789	-0.482231615
ENSG00000140987	ZSCAN32	5.450179	6.300058	6.462549	5.634592	5.889713	5.740728	4.096039	3.771976	3.78631	0.567036565	0.085047568	0.00010141	-0.482172639
ENSG00000180423	HARBI1	2.819744	2.526275	2.192546	2.582309	2.791564	2.854102	1.763998	1.599092	1.445551	0.120809181	0.332311399	0.030701009	-0.48205758
ENSG00000257093	KIAA1147	10.133503	10.196516	10.314412	8.824281	8.263235	8.626798	6.842073	6.2449	7.528625	0.135280474	-0.105841697	4.62E-11	-0.481577907
ENSG00000142611	PRDM16	2.336755	1.876755	1.29778	2.646341	2.070724	2.54445	1.213296	0.93298	1.460591	0.000266116	0.481811177	0.001428879	-0.481440906
ENSG00000083799	CYLD	3.593377	4.733095	4.246924	6.889919	7.814395	8.206245	3.430776	2.975934	3.523272	0.00031134	1.018287984	0.0022043	-0.481350231
ENSG00000197170	PSMD12	20.289511	22.087378	23.358615	21.297738	19.363966	20.012792	15.566134	13.953564	14.677044	0.774878487	0.029171855	6.97E-10	-0.481240914
ENSG00000073910	FRY	3.5965	3.945715	4.230186	2.838877	2.857356	2.579604	2.571219	2.597044	2.804075	0.0000363	-0.356262758	2.92E-08	-0.48119506
ENSG00000186532	SMYD4	10.183176	9.759196	9.824024	8.397599	8.015409	8.59985	7.091603	5.985016	7.404066	0.245126321	-0.102765237	5.68E-08	-0.481101041
ENSG00000152990	ADGRA3	41.369126	39.254815	38.609748	20.776753	19.380495	19.074469	25.824105	26.030679	28.791209	3.06E-56	-0.865637747	4.07E-14	-0.480997494
ENSG00000259956	RBM15B	20.214243	18.513674	17.284626	16.578459	16.1996	16.322283	12.313117	11.297122	14.106755	0.641526564	-0.043069034	3.73E-09	-0.480930861
ENSG00000084093	REST	10.116216	11.93694	12.356605	9.970844	9.606987	9.827501	7.630286	7.362856	8.05328	0.400496476	-0.081170225	8.42E-09	-0.480915789
ENSG00000050327	ARHGEF5	8.998189	8.627435	7.78418	8.197076	8.1472	8.759129	5.264761	5.568131	6.478973	0.133433485	0.136825824	0.000000274	-0.480907704
ENSG00000165105	RASEF	6.427928	6.973203	7.388006	4.752397	5.028881	4.992129	4.863329	4.333605	4.947471	0.0000167	-0.370659123	0.00000024	-0.480843387
ENSG00000196693	ZNF33B	2.886635	3.310196	3.329555	2.807998	3.392174	2.467759	2.371179	2.151881	2.160111	0.66669413	-0.068720732	0.0000775	-0.480819125
ENSG00000107036	RIC1	9.91964	9.590087	9.349997	8.797965	8.451866	8.26989	6.257039	6.415017	7.656064	0.936653548	-0.008262399	2.61E-10	-0.480786546
ENSG00000186104	CYP2R1	9.926899	9.772965	10.660245	10.307198	10.255438	9.487209	6.6208	6.7158	7.149998	0.195161076	0.149972605	0.00000766	-0.48067719
ENSG00000168273	SMIM4	2.1797	2.58275	2.355342	2.142035	2.262484	2.75359	1.820191	2.088612	1.078754	0.662412972	0.124799654	0.066705012	-0.480584651
ENSG00000119729	RHOQ	11.76559	12.3128	14.053215	10.983838	10.979544	10.690968	8.696796	7.437663	9.495598	0.534088813	-0.072190579	0.00000356	-0.480404959
ENSG00000130818	ZNF426	2.805618	2.971185	2.422983	2.807229	2.777717	2.549442	1.935996	1.554648	2.057773	0.596780401	0.076204908	0.000174648	-0.48033317
ENSG00000141098	GFOD2	4.37589	4.760981	4.872264	4.079013	3.910785	4.11983	3.240893	3.177826	4.633946	0.485568092	-0.102421579	0.000803479	-0.480312771
NewGene_5857	--	0.359454	0.471052	0.392668	0.165912	0.275708	0.372445	0.413786	0.210301	0.269321	0.217490258	-0.631401576	0.30567017	-0.47991656
ENSG00000127903	ZNF835	1.461415	1.375442	1.459196	1.267903	1.153976	1.264261	1.059445	0.881901	0.892362	0.820501776	-0.061564525	0.021410666	-0.479588261
ENSG00000121486	TRMT1L	3.532189	3.830329	3.721118	3.82646	3.716341	3.564003	2.963579	2.298609	2.197937	0.230564479	0.147078775	0.000587425	-0.479496307
ENSG00000181218	HIST3H2A	2.387604	1.760872	2.076982	0.873211	0.97736	2.243505	1.373287	1.79093	0.965352	0.347056717	-0.441524312	0.201749854	-0.479389279
ENSG00000087494	PTHLH	11.53139	11.442903	12.924147	12.641607	12.7315	13.196985	7.373494	6.642686	8.449071	0.043858795	0.221622557	0.0000735	-0.47926284
ENSG00000163624	CDS1	1.231254	1.18493	1.04661	1.095007	1.256649	1.159639	0.697227	0.762789	0.871973	0.46664896	0.16389533	0.019223427	-0.47919938
ENSG00000167604	NFKBID	1.483772	1.573696	1.861565	1.64237	1.067313	1.293008	0.951013	1.066734	1.23754	0.741084056	-0.122900139	0.093395023	-0.479098568
ENSG00000168779	SHOX2	0.412982	0.563867	0.705059	0.775927	0.785002	0.42374	0.32987	0.361046	0.256546	0.745022619	0.160965153	0.269282795	-0.478769991
ENSG00000173926	3-Mar	1.190583	1.200498	1.416376	0.732532	0.835251	0.942663	0.859372	0.944811	0.790775	0.024718807	-0.477731776	0.023316006	-0.478228506
ENSG00000188322	SBK1	9.141111	7.912342	7.197414	4.320553	4.046159	3.972408	5.193066	5.580063	5.54217	8.34E-18	-0.82825989	0.00000409	-0.478122873
NewGene_187	--	0.770196	0.92784	1.153026	0.831238	1.031252	0.928566	0.600863	0.660372	0.662177	0.786416615	0.115978532	0.172737912	-0.478072862
ENSG00000151575	TEX9	1.501189	2.153066	1.445608	0.949548	0.970838	1.389736	1.398549	1.275424	0.915342	0.196809392	-0.43279585	0.115947101	-0.478064901
ENSG00000100557	CCDC198	5.395742	3.68949	4.091751	2.285249	1.804474	2.231572	2.788716	2.5797	2.915881	0.000000359	-0.883500324	0.005805388	-0.477987942
ENSG00000136160	EDNRB	0.95585	0.800504	1.04813	0.109927	0.190095	0.100602	0.542778	0.580544	0.612276	7.9E-17	-2.520301502	0.042514538	-0.477948984
ENSG00000091622	PITPNM3	0.230069	0.421236	0.248747	0.177101	0.223518	0.166514	0.16508	0.150342	0.225579	0.14144818	-0.53946013	0.177634763	-0.477947998
ENSG00000131503	ANKHD1	15.359331	12.461434	9.176056	11.571156	12.399712	17.003789	10.918181	8.083882	10.562273	0.710281099	0.212753422	0.328110779	-0.477929842
ENSG00000012983	MAP4K5	8.162314	7.476297	8.360759	6.302075	6.056814	6.499983	5.080929	5.233075	5.967005	0.02985937	-0.201708787	0.000000503	-0.477794974
ENSG00000157833	GAREM2	2.282127	2.290284	2.118307	1.73549	1.550574	1.9995	1.408299	1.385688	1.702979	0.264255745	-0.196989803	0.001760625	-0.477466545
ENSG00000205808	PLPP6	8.912632	8.460384	8.626007	8.348495	8.15128	7.793108	6.53525	5.643478	5.313476	0.692289045	0.048543954	0.0000191	-0.477100749
ENSG00000197622	CDC42SE1	31.084792	30.460962	32.386552	22.450118	22.544452	21.783742	19.355918	20.672162	23.166481	2.88E-08	-0.340643608	3.63E-10	-0.476855177
NewGene_6627	--	1.468867	1.51262	1.563578	0.945336	0.901228	1.070356	1.176028	0.956701	0.848069	0.033932775	-0.449481696	0.02404178	-0.476738678
ENSG00000120519	SLC10A7	2.961162	2.953238	2.604551	3.489958	2.940032	2.970783	1.928268	2.014307	1.655265	0.0115539	0.352746252	0.004277627	-0.476318825
NewGene_2805	--	1.045257	0.833744	0.94115	0.843096	0.907783	0.828866	0.56255	0.592672	0.815116	0.575970302	-0.157271085	0.036343734	-0.4762838
ENSG00000266094	RASSF5	0.383079	0.707581	0.657018	0.317638	0.607863	0.555589	0.481542	0.247345	0.418069	0.931035113	-0.044095618	0.177315145	-0.476197076
ENSG00000151948	GLT1D1	1.636372	1.746868	1.731198	1.921257	1.42377	1.716495	1.491596	0.905287	1.087951	0.621524903	0.125797464	0.03795982	-0.47576363
ENSG00000139173	TMEM117	2.837875	3.055634	3.008502	2.440213	2.387333	2.295939	2.252136	2.029512	1.668102	0.365398048	-0.162557672	0.006607722	-0.475741803
ENSG00000137731	FXYD2	20.060801	12.709427	14.35102	5.445025	6.966692	6.10825	10.960606	11.304431	9.436459	1.71E-09	-1.18743893	0.016769295	-0.475726071
ENSG00000163162	RNF149	14.072234	15.444485	16.595206	17.009999	16.538498	15.838602	9.918824	9.403907	11.661495	0.004423058	0.24946683	0.00000177	-0.475467337
ENSG00000157985	AGAP1	3.774625	3.549165	3.549653	2.844679	2.745253	2.866415	2.597507	2.345953	2.70574	0.143277913	-0.149427802	0.00000192	-0.475086821
ENSG00000079689	SCGN	6.629189	5.889234	5.542395	1.287219	1.602449	1.370388	3.952758	4.012052	3.760657	1.27E-23	-1.88280383	0.004998759	-0.47508682
ENSG00000125633	CCDC93	10.940383	10.644072	10.723692	8.889705	8.653014	8.226332	7.135861	6.713259	8.015821	0.008081017	-0.180426938	9.35E-11	-0.474989191
ENSG00000116128	BCL9	12.689854	12.688039	11.24142	9.466625	9.521947	9.424396	8.223712	7.607021	8.915271	0.003225098	-0.218762211	1.42E-09	-0.474826411
ENSG00000151693	ASAP2	2.089288	2.017965	2.405509	1.661815	1.513084	1.287527	1.397092	1.133106	1.900316	0.006234364	-0.40883391	0.005140689	-0.474722644
ENSG00000138735	PDE5A	2.49829	2.631094	3.606218	1.553666	1.409366	1.3093	1.40148	1.607244	2.069333	1.44E-19	-1.104140482	0.000559268	-0.474655133
ENSG00000155744	FAM126B	1.712254	2.064788	2.02591	1.661805	1.751934	1.748642	1.265324	1.146666	1.369371	0.578666868	0.090195851	0.000750909	-0.473933322
ENSG00000129173	E2F8	4.411233	4.932895	5.925878	4.005551	3.390424	3.545893	4.363882	2.825703	3.144439	0.015299291	-0.338109206	0.004728313	-0.473858107
ENSG00000108510	MED13	12.724945	14.060594	15.156524	15.907318	15.719962	14.859916	9.907988	8.943184	9.634301	0.0000119	0.302201429	3.28E-10	-0.473593848

Gene ID	Gene name	Ctrl-1 FPKM	Ctrl-2 FPKM	Ctrl-3 FPKM	IFNγ-1 FPKM	IFNγ-2 FPKM	IFNγ-3 FPKM	Sora-1 FPKM	Sora-2 FPKM	Sora-3 FPKM	Ctrl vs IFNγ DESeq2 FDR	Ctrl vs IFNγ DESeq2 log2FC	Ctrl vs Sora DESeq2 FDR	Ctrl vs Sora DESeq2 log2FC
ENSG00000149115	TNKS1BP1	13.878819	12.576748	12.148814	18.27595	18.164545	18.668282	8.554944	7.893831	9.285345	1.39E-25	0.652764306	2.07E-09	-0.473547286
ENSG00000188636	RTL6	7.768175	7.19564	6.631218	6.163389	5.744358	6.009261	4.82153	4.480523	5.313554	0.218464918	-0.122928445	0.00000039	-0.473422714
ENSG00000108379	WNT3	1.69304	2.013116	1.962093	1.999536	1.939245	1.585511	1.244189	1.20179	1.376529	0.634369128	0.108947012	0.009781446	-0.473207162
ENSG00000165995	CACNB2	1.465761	1.82577	1.456273	1.373045	1.110067	1.074925	1.250982	0.77778	0.90509	0.693189212	-0.102970985	0.018460051	-0.473166284
ENSG00000172000	ZNF556	0.773812	0.528199	0.669166	0.459041	0.604518	0.532262	0.24405	0.445727	0.486944	0.767070828	-0.096752666	0.083070998	-0.473036508
ENSG00000198863	RUNDC1	4.389362	3.848534	4.066962	4.213114	3.661063	3.718621	3.219028	2.346767	2.742819	0.736016601	0.053859983	0.000610702	-0.472494585
ENSG00000172458	IL17D	3.421277	3.969959	3.970808	2.812683	2.96545	2.890352	2.357961	1.940167	2.95732	0.263986423	-0.23959984	0.023890184	-0.47244667
ENSG00000090470	PDCD7	8.36005	8.06928	8.543179	8.997505	8.449415	8.760852	6.580826	5.026683	5.259011	0.02323095	0.214689905	0.0000839	-0.471949109
ENSG00000239789	MRPS17	18.436625	18.483397	18.976441	15.765599	17.53107	18.02374	13.653005	14.045455	10.531554	0.97025095	0.005420441	0.000132988	-0.47137885
NewGene_2811	--	1.975259	1.569757	1.931263	2.589378	2.186084	2.117593	1.082244	1.297558	1.333412	0.015419955	0.476362705	0.036889878	-0.471008765
ENSG00000196345	ZKSCAN7	2.04406	2.502528	2.270263	1.527033	0.981171	1.351846	1.203599	1.573624	1.223602	0.001968115	-0.610011751	0.016854666	-0.470922327
ENSG00000182568	SATB1	0.954087	0.935166	0.890347	0.528026	0.486684	0.554943	0.803366	0.531412	0.65191	0.000957944	-0.580381508	0.015448552	-0.470769983
ENSG00000134265	NAPG	5.156322	5.281094	6.403894	5.387853	4.754204	4.484982	3.818427	3.65819	4.065649	0.718968101	-0.05938475	0.0000945	-0.470635534
ENSG00000151458	ANKRD50	3.780559	3.951468	3.919258	3.119576	3.090552	3.043794	2.887236	2.458312	2.537419	0.033900075	-0.184847391	0.000000812	-0.47054156
ENSG00000196605	ZNF846	1.370425	0.969761	1.278152	0.673179	0.976886	0.84723	0.918948	0.959899	1.130017	0.283017423	-0.393058988	0.16031922	-0.470293126
ENSG00000133805	AMPD3	1.034788	0.617542	0.945735	0.663619	0.523982	0.475477	0.559228	0.511547	0.673033	0.043305403	-0.616442361	0.107097927	-0.470147017
ENSG00000275004	ZNF280B	0.940629	1.187884	1.119109	1.030338	1.098876	1.038592	0.798178	0.730589	0.685036	0.612441225	0.11361193	0.014627634	-0.470027191
NewGene_6267	--	0.881144	1.461689	1.247716	1.524618	1.388204	0.995065	0.853585	0.706532	0.844736	0.109975752	0.325769735	0.012017129	-0.469603602
ENSG00000137075	RNF38	8.420589	8.630739	8.427995	7.974684	7.212349	7.067585	6.045544	5.540912	5.878292	0.451357381	-0.073170138	4.99E-09	-0.469588937
ENSG00000149231	CCDC82	4.280986	4.411664	4.65905	4.239295	3.765356	3.110925	3.890131	3.428697	3.507895	0.935884345	-0.015421568	0.0000599	-0.469557857
ENSG00000146802	TMEM168	2.526229	3.035705	2.908335	2.263729	2.186083	2.050678	1.851792	1.817245	2.204745	0.177833398	-0.164797641	0.0000568	-0.469442711
ENSG00000038219	BOD1L1	10.669385	11.016462	11.210507	11.201753	10.303322	10.726974	7.244331	6.982132	7.974056	0.017816631	0.147722392	2.46E-12	-0.46932474
ENSG00000166192	SENP8	0.367055	0.681336	0.571701	0.670119	0.442894	0.486498	0.32787	0.405688	0.343741	0.758220277	0.125253081	0.160567405	-0.469275968
ENSG00000151893	CACUL1	3.29153	3.622487	3.90117	3.55285	3.402287	3.388632	2.554606	2.151406	2.641733	0.417218336	0.084339198	0.00000165	-0.469129272
ENSG00000240230	COX19	13.349252	13.03718	12.747537	12.741241	13.848027	13.283718	8.802245	9.120889	8.552155	0.029754036	0.17469236	2.24E-08	-0.469026335
NewGene_511	--	0.468565	0.588558	0.447527	0.612544	0.539408	0.503447	0.280106	0.374164	0.360142	0.412604629	0.278499869	0.167574739	-0.468597519
ENSG00000101126	ADNP	27.550913	25.203433	26.507894	22.88757	23.198665	25.638902	18.79856	16.824718	18.892623	0.64164323	-0.034518738	3.13E-14	-0.46835167
NewGene_4049	--	0.475181	0.311159	0.428336	0.53509	0.434344	0.601913	0.27254	0.343139	0.201234	0.202998735	0.525860085	0.327681238	-0.4682416
ENSG00000172315	TP53RK	3.369048	3.886353	3.58921	2.707185	2.812493	2.867266	2.37768	2.485833	2.666073	0.133136924	-0.214951078	0.000515153	-0.468217405
ENSG00000204385	SLC44A4	0.751716	0.576568	0.908297	0.446885	0.739628	0.865167	0.643546	0.443823	0.420713	0.958082541	0.028745925	0.196091633	-0.467931141
NewGene_5660	--	1.45476	1.766054	1.691251	1.67242	0.834992	1.432465	1.26852	0.916453	0.821982	0.742006832	-0.101871388	0.036201173	-0.467928879
ENSG00000145431	PDGFC	15.351599	16.720098	19.418482	8.891273	9.45005	9.438663	11.810147	9.555971	12.460054	8.22E-17	-0.719686042	0.00000178	-0.46790864
ENSG00000145908	ZNF300	8.935246	8.546953	7.853581	6.247851	6.329287	5.674952	5.869191	5.84231	5.974255	0.003434219	-0.278269241	0.000000558	-0.467764045
NewGene_4164	--	0.881006	0.876902	0.745919	0.613239	0.700154	0.624236	0.762593	0.638794	0.637455	0.519054097	-0.244120301	0.148846283	-0.467569489
ENSG00000138592	USP8	12.107057	10.389342	10.534633	10.70686	12.518084	12.207788	4.700401	9.422398	5.020608	0.018203831	0.162704592	6.51E-11	-0.466871274
ENSG00000076641	PAG1	4.257137	4.03055	4.405669	3.489213	3.504643	3.698747	2.777486	2.765182	3.077359	0.280233493	-0.09956217	1.46E-08	-0.466794955
ENSG00000144935	TRPC1	4.00525	3.484818	4.223984	3.311829	3.130787	2.63476	2.464145	3.171648	2.263273	0.288646702	-0.150303413	0.000241942	-0.465872136
ENSG00000150712	MTMR12	18.984474	18.437782	19.026495	12.918145	13.34402	13.155411	12.11408	11.756495	14.044372	8.81E-10	-0.363487554	6.96E-11	-0.465780566
ENSG00000160271	RALGDS	5.644297	5.664541	5.137954	4.179795	3.583738	4.312524	3.362296	3.517769	3.979603	0.0102597	-0.306693009	0.0000576	-0.465313772
ENSG00000115318	LOXL3	0.81835	1.037274	0.933597	0.533434	0.533712	0.284215	0.614926	0.550745	0.695693	0.068097874	-0.623931177	0.117265847	-0.465168475
ENSG00000164916	FOXK1	5.493854	5.526231	4.772522	6.391674	6.144853	6.756784	5.476099	4.116344	4.268558	8.42E-08	0.420825676	0.0000351	-0.464861006
ENSG00000070778	PTPN21	2.634905	2.430963	3.256052	2.320583	2.157346	2.274535	2.017832	1.526056	2.124818	0.281833888	-0.162790033	0.001956681	-0.464687432
ENSG00000107679	PLEKHA1	4.943241	5.927244	5.815516	4.220955	3.732255	3.346417	5.036007	4.10117	5.481353	2.3E-12	-0.534278895	0.000000647	-0.46436008
ENSG00000170049	KCNAB3	1.160128	0.96051	0.975988	1.259674	0.992075	0.690135	0.737922	0.568124	0.806253	0.973041893	-0.016034857	0.116417023	-0.464248334
ENSG00000196937	FAM3C	15.129756	15.356195	16.358091	12.666855	12.609972	13.713419	9.744152	10.346615	11.798798	0.23371725	-0.114381652	0.000000468	-0.464112302
ENSG00000065413	ANKRD44	0.493186	0.427689	0.543814	0.376364	0.300799	0.354164	0.287256	0.306043	0.362716	0.409036967	-0.250693284	0.082558763	-0.463501618
ENSG00000109771	LRP2BP	0.428676	0.201261	0.477954	0.413409	0.330392	0.130725	0.196144	0.312269	0.291825	0.741638567	-0.189397451	0.28337639	-0.463422926
ENSG00000174206	C12orf66	1.969867	1.993178	2.291071	1.77691	1.803136	1.854386	1.326286	1.387217	1.683818	0.968809578	0.009716592	0.00590123	-0.463279344
ENSG00000165943	MOAP1	6.796219	7.476896	6.612842	5.202258	5.44034	5.226875	4.743536	4.111043	5.383098	0.040179043	-0.246224309	0.000180697	-0.463246578
ENSG00000187605	TET3	10.127714	9.974136	10.933649	8.769615	8.553307	8.370093	7.271677	6.19022	7.690782	0.064577534	-0.125486361	1.72E-09	-0.463142273
ENSG00000101004	NINL	13.178148	12.981142	12.76417	10.947012	10.247894	9.607811	8.426854	8.489144	9.709005	0.011179508	-0.189487487	2.37E-09	-0.463118697
ENSG00000123728	RAP2C	11.814188	13.181621	14.507163	13.271785	12.40903	12.95708	8.908195	8.794948	9.161282	0.243198163	0.115208509	0.000000382	-0.462900041
ENSG00000173894	CBX2	16.641864	15.369792	13.492268	13.119121	12.753758	13.540649	9.820004	9.396464	11.37774	0.634870308	-0.052172824	0.000000406	-0.462659493
ENSG00000132716	DCAF8	32.9311	31.843746	34.111737	33.921234	32.94578	32.87228	22.836064	21.07	24.15631	0.085272983	0.110232335	1.2E-12	-0.462606236
ENSG00000172476	RAB40A	0.694009	0.672314	0.475342	0.269571	0.19904	0.335575	0.298054	0.425653	0.578941	0.003448654	-1.105256862	0.207169435	-0.462499935
ENSG00000184731	FAM110C	3.387314	3.291663	3.821827	2.032413	2.096229	1.722155	2.245662	2.123787	2.79708	0.00000033	-0.697256565	0.001072808	-0.462245511
ENSG00000185658	BRWD1	8.322417	9.345256	8.8338	9.521267	7.784784	9.47165	7.377415	6.546651	7.33157	0.89481531	-0.040388813	1.01E-08	-0.462182876
ENSG00000171109	MFN1	14.364127	17.032131	16.010664	13.251695	13.014106	13.04561	9.903891	9.867431	11.582163	0.288477978	-0.082642544	1.03E-08	-0.461831943
ENSG00000185619	PCGF3	35.457093	34.157043	36.821029	30.759617	28.944807	29.471475	25.756892	24.85436	29.958694	0.06281519	-0.111449249	3.96E-12	-0.461473849
ENSG00000157107	FCHO2	2.246153	2.390692	2.895664	2.554044	2.3317	2.634498	1.789519	1.472218	1.878786	0.383728894	0.140720538	0.002185719	-0.461449934
ENSG00000142961	MOB3C	0.765906	0.715193	0.373037	1.90522	1.903096	1.794157	0.437977	0.44596	0.532518	5.37E-18	1.58904821	0.123429174	-0.461440484
ENSG00000116833	NR5A2	22.289281	22.406338	23.476545	26.162647	29.791765	25.128893	16.528718	15.732468	15.270469	0.000000266	0.348869642	1.06E-09	-0.461381649
ENSG00000163684	RPP14	4.086775	4.624306	4.310747	4.89616	4.15946	5.172464	3.672315	3.029782	3.06156	0.107185567	0.159513567	0.000013	-0.461354421
ENSG00000213015	ZNF580	7.732746	7.217142	5.796202	7.189018	7.694589	7.988333	4.742366	4.201455	5.461134	0.047031046	0.310008907	0.005063799	-0.461316303
ENSG00000268043	NBPF12	7.235846	6.636417	6.874736	6.237513	5.731529	5.826271	4.876333	4.43381	4.702995	0.593938935	-0.057152277	0.000000218	-0.461308938
ENSG00000156140	ADAMTS3	0.85902	0.982398	1.387368	0.462104	0.49908	0.479253	0.713396	0.689817	0.795625	0.00000178	-1.010883028	0.029270744	-0.461221341
NewGene_3993	--	2.453259	2.362707	2.535729	2.811079	2.77561	2.775272	1.842336	2.001401	1.94559	0.00053235	0.369641729	0.000256428	-0.46121629
ENSG00000162836	ACP6	2.272118	1.938272	1.73624	1.673831	2.012334	2.151814	1.327874	1.192641	1.022526	0.889239124	0.030918573	0.004595755	-0.46119219
ENSG00000185219	ZNF445	2.047103	2.261401	2.070733	2.317636	2.044679	2.139199	1.654394	1.544237	1.952845	0.102965083	0.140522568	0.00000142	-0.461181467
ENSG00000213799	ZNF845	2.234222	2.53792	2.265367	2.136005	2.00945	1.736401	1.539376	1.55924	1.713013	0.311893506	-0.146234092	0.000190016	-0.461084772
ENSG00000144815	NXPE3	3.55	3.418578	3.307351	3.175704	3.075761	3.007833	2.553961	2.127653	3.259268	0.953110507	-0.009696274	0.000329792	-0.461001771
ENSG00000130803	ZNF317	15.628972	14.590894	15.784072	12.963437	12.303218	13.023756	9.927281	10.022967	11.286889	0.142417628	-0.11796426	5.1E-09	-0.460887439
ENSG00000124383	MPHOSPH10	74.414667	74.699112	88.89998	64.724477	79.189492	66.979212	51.564545	52.682656	58.458135	0.837909154	-0.027168462	1.03E-08	-0.460707198
ENSG00000100139	MICALL1	15.048544	13.087541	13.252149	12.980296	12.153159	13.021182	8.916207	8.312313	10.852608	0.788556925	0.029587486	0.000000657	-0.460681236

Gene ID	Gene name	Ctrl-1 FPKM	Ctrl-2 FPKM	Ctrl-3 FPKM	IFNγ-1 FPKM	IFNγ-2 FPKM	IFNγ-3 FPKM	Sora-1 FPKM	Sora-2 FPKM	Sora-3 FPKM	Ctrl vs IFNγ DESeq2 FDR	Ctrl vs IFNγ DESeq2 log2FC	Ctrl vs Sora DESeq2 FDR	Ctrl vs Sora DESeq2 log2FC
ENSG00000170961	HAS2	0.531909	0.547569	0.690384	0.269022	0.325752	0.266187	0.407156	0.37052	0.424978	0.004130565	-0.888164906	0.119071901	-0.46065951
ENSG00000158109	TPRG1L	14.758327	16.481098	14.821807	14.530979	14.242793	13.309093	9.177182	10.1653	12.087287	0.896266427	0.016740544	0.0000107	-0.460456263
ENSG00000180694	TMEM64	4.836688	4.586923	4.967157	3.969677	4.060141	3.825279	3.371103	2.72381	3.608769	0.298776216	-0.116594812	0.0000376	-0.460433405
ENSG00000107614	TRDMT1	0.584199	1.031815	0.742902	0.543713	0.771033	0.464328	0.8242	0.566902	0.345335	0.629215098	-0.127886154	0.044265847	-0.460054469
ENSG00000115963	RND3	24.680735	28.002642	28.669377	20.607845	20.865742	20.195382	18.871625	17.948371	18.824367	0.001374389	-0.252127468	0.000000015	-0.459775317
ENSG00000095564	BTAF1	8.889819	9.78054	9.290184	10.060316	9.126811	8.845933	6.467101	5.871631	6.743363	0.054259608	0.150338639	9.55E-10	-0.45960382
ENSG00000266964	FXYD1	3.511689	3.456374	2.234841	1.443176	1.439068	1.928741	1.632659	2.963441	1.433977	0.054114534	-0.728053162	0.256307966	-0.45946252
ENSG00000068745	IP6K2	39.563584	39.44147	40.442089	38.091244	33.261247	34.453102	26.888469	25.005838	31.835146	0.408173048	-0.066549349	3.48E-10	-0.459436113
ENSG00000102781	KATNAL1	1.415952	1.540967	1.619051	1.204215	1.216444	0.940965	0.892742	0.973419	1.308316	0.07027874	-0.281386148	0.002453532	-0.459393153
ENSG00000106459	NRF1	7.366158	7.572192	7.470227	7.671946	6.642192	7.109551	5.032535	5.297005	5.279498	0.890026614	0.020091427	0.00000858	-0.459186703
ENSG00000185829	ARL17A	5.572118	5.412216	5.066237	5.102996	4.952231	4.69863	4.530117	3.822241	3.795278	0.621205276	0.070187137	0.000177019	-0.458726988
ENSG00000196911	KPNA5	3.625719	3.645737	3.610856	4.064027	4.388477	4.409964	2.784891	2.551815	2.288728	0.000128778	0.328420255	0.00000333	-0.458670862
ENSG00000143157	POGK	19.861996	19.211557	19.686757	16.052757	14.946078	15.375545	13.07911	12.677335	14.695857	0.00261597	-0.175146837	3.83E-12	-0.458669211
ENSG00000272916	AC022400.6	1.05649	1.09833	0.565106	1.164947	0.841868	0.734584	0.60629	0.439157	0.824875	0.654622577	0.150643065	0.112417285	-0.458475755
ENSG00000197816	CCDC180	1.114601	1.023721	0.842828	1.152602	1.059409	0.860658	0.626007	0.628371	0.779678	0.441552606	0.183998271	0.030335955	-0.458308247
ENSG00000034677	RNF19A	10.359914	9.445284	10.734392	8.98071	9.593164	9.161485	7.359836	6.750283	6.732793	0.932918593	-0.011418556	0.000000293	-0.458279667
ENSG00000166170	BAG5	7.459494	8.289885	8.341084	7.77587	7.741366	7.634364	5.825715	5.3208	5.467781	0.398370998	0.08274969	0.000000393	-0.458135008
ENSG00000114739	ACVR2B	2.26558	2.60318	2.434454	1.953827	1.944936	2.013735	1.683466	1.579595	1.721224	0.133444493	-0.155701998	0.00000237	-0.457671264
ENSG00000156968	MPV17L	5.637511	6.231138	5.55998	5.252701	4.784012	5.624994	4.270989	3.894833	4.360465	0.840727575	-0.027610887	0.00000217	-0.457653369
ENSG00000204217	BMPR2	3.339518	3.603201	3.68723	2.983688	2.648557	3.01291	2.162152	2.218836	2.927673	0.109823632	-0.150578195	0.000015	-0.457319494
ENSG00000171124	FUT3	0.929782	0.471889	0.599835	0.949528	1.022562	0.822175	0.383666	0.342746	0.575959	0.062924067	0.614620988	0.252965924	-0.457010503
ENSG00000137801	THBS1	42.435463	42.094154	45.105315	61.657712	57.282345	60.761746	28.970499	26.836992	33.188595	7.33E-37	0.618119542	2.31E-11	-0.456722808
ENSG00000275342	PRAG1	5.992683	5.767408	5.706099	3.754094	4.102354	4.225901	3.77794	3.671383	4.614408	0.0000814	-0.376415747	0.0000072	-0.456618682
NewGene_2896	--	0.918355	0.797987	0.671234	0.718867	0.723088	0.743308	0.597086	0.40954	0.618825	0.935884345	0.028417741	0.084144316	-0.456581685
ENSG00000175322	ZNF519	2.69653	3.036781	2.919757	2.260637	1.960719	1.963237	1.885415	1.764371	2.162886	0.057266418	-0.339232916	0.008029307	-0.456399604
ENSG00000133657	ATP13A3	91.497149	98.75885	106.104518	80.039723	80.486506	78.253001	66.937376	65.613662	73.291505	0.009531303	-0.174928713	1.28E-10	-0.456225467
ENSG00000163644	PPM1K	1.7127	1.979391	2.373225	1.438475	1.784625	1.372097	1.591505	1.423073	1.643788	0.108870424	-0.278839782	0.003080994	-0.455964744
NewGene_2774	--	0.980802	0.994773	0.912107	1.201698	0.948981	1.005678	0.525234	0.574445	0.689106	0.02899055	0.397732718	0.016692476	-0.455828554
ENSG00000182704	TSKU	6.046222	6.050832	6.074004	4.878214	4.864441	4.56717	4.209061	4.037331	4.437332	0.145140064	-0.190785137	0.00020739	-0.455791166
ENSG00000156150	ALX3	1.508899	2.013319	1.652934	2.10074	1.544524	1.978907	1.19304	1.39107	0.8665	0.280149449	0.305101241	0.13889107	-0.455303443
ENSG00000108799	EZH1	3.678478	3.51295	3.2958	3.080344	3.95673	2.982133	2.356322	2.127184	2.235647	0.683353196	0.062491529	0.000270873	-0.455134415
ENSG00000047346	FAM214A	11.570057	12.977099	11.421286	14.607148	13.851407	14.320424	8.248675	7.618207	8.017219	5.68E-10	0.447640655	0.000000165	-0.455102691
ENSG00000153767	GTF2E1	7.066964	7.382054	7.286536	6.210043	5.815645	6.07525	5.114067	5.15071	4.5856	0.222211657	-0.136415374	0.0000485	-0.455102157
NewGene_2588	--	1.14499	0.898407	0.971873	0.912871	0.812419	0.974956	0.545736	0.803581	0.704815	0.981400982	-0.009683066	0.118618626	-0.454972385
ENSG00000136286	MYO1G	0.904773	0.697632	0.305509	0.595676	0.555973	0.484176	0.445612	0.400584	0.455716	0.840850496	-0.108652692	0.279073933	-0.454842568
ENSG00000143867	OSR1	1.564316	0.933183	0.705245	0.885735	0.857779	1.144112	0.774705	0.727686	0.683514	0.998321415	-0.001725072	0.233436877	-0.454411031
ENSG00000165323	FAT3	0.313239	0.297761	0.383111	0.213156	0.199568	0.153774	0.250028	0.193951	0.236351	0.000915757	-0.676275549	0.021160809	-0.454384417
ENSG00000006576	PHTF2	11.927719	13.942337	17.453949	8.54148	8.698594	7.260704	9.988715	10.06557	10.697927	0.001225846	-0.688169613	0.000115954	-0.454180388
ENSG00000188321	ZNF559	10.919871	11.846102	9.286166	7.483557	6.688314	7.363841	6.821093	7.382685	8.344337	0.00000204	-0.470525169	0.0000114	-0.453856594
ENSG00000140950	MEAK7	1.375825	1.141217	1.292269	1.081763	0.847635	1.083045	0.693024	0.97863	0.915392	0.27193433	-0.231578284	0.019863765	-0.453830563
ENSG00000253710	ALG11	3.383081	4.188382	3.789609	4.894712	4.926913	4.491464	2.65074	2.868862	2.384983	0.0000758	0.526168653	0.010712981	-0.453658917
ENSG00000121989	ACVR2A	4.915892	5.136676	5.489278	4.742589	4.579744	5.010739	3.460057	3.266277	4.048572	0.806304488	0.031702044	0.00000829	-0.453526511
ENSG00000170946	DNAJC24	8.172708	9.626725	9.749351	7.874737	7.507741	6.587458	6.701974	6.405293	6.259805	0.127115716	-0.215042346	0.000437469	-0.453432173
ENSG00000165490	DDIAS	3.511802	4.301443	4.154201	3.390211	2.513071	2.764987	2.584401	2.914623	2.736257	0.045458719	-0.320343379	0.001325241	-0.45339003
ENSG00000132694	ARHGEF11	10.185848	9.1512	9.636347	10.406142	9.611517	9.582314	6.681657	6.007462	7.183028	0.016295377	0.175608884	1.71E-08	-0.452748059
ENSG00000134508	CABLES1	6.793064	6.197192	6.237449	4.961799	5.458682	5.000991	4.496239	3.969515	4.764108	0.091179945	-0.180678377	0.00000376	-0.452664469
ENSG00000048707	VPS13D	2.129091	2.062519	2.157697	2.056999	1.385114	1.414017	1.446342	1.434514	1.566619	0.0000128	-0.381957073	0.000000123	-0.452639462
ENSG00000016402	IL20RA	1.615038	1.512381	1.500511	1.54184	1.741554	1.342037	1.14232	1.054014	1.037112	0.779288501	0.07199913	0.019624476	-0.45259828
ENSG00000118514	ALDH8A1	0.772971	1.10673	1.047672	0.206658	0.330561	0.286657	0.679384	0.634679	0.684011	0.000000703	-1.671414915	0.133682855	-0.452507222
NewGene_3923	--	4.457469	5.52776	4.9717	6.960855	6.978384	6.607802	3.548834	3.382781	3.60403	7.4E-13	0.715518561	0.0000462	-0.452352648
NewGene_568	--	3.864824	4.102485	3.584591	2.727424	2.741801	2.666774	2.611866	2.32473	2.997691	0.001323127	-0.359316581	0.000171575	-0.452181429
ENSG00000140807	NKD1	1.232067	0.940346	0.96719	0.807973	0.662751	0.775855	0.7126	0.679881	0.763013	0.017801515	-0.336076052	0.000690726	-0.452101278
ENSG00000170915	PAQR8	22.92123	22.322531	29.790081	18.032676	19.959049	19.971377	18.031518	15.629614	20.338117	0.004725506	-0.275483069	8.16E-09	-0.452092555
ENSG00000003056	M6PR	60.617091	64.043015	69.99079	59.213299	56.239782	55.158167	41.114519	42.895196	42.758725	0.447482041	-0.062685346	1.03E-09	-0.451236632
ENSG00000177051	FBXO46	16.760073	15.614368	15.513389	11.670869	12.375588	11.477168	10.084983	8.859296	9.764649	0.004999727	-0.240781997	0.00000123	-0.451085588
ENSG00000170791	CHCHD7	8.415596	8.973558	8.968977	9.235062	8.610985	8.627789	6.120947	6.52035	6.141176	0.171670948	0.176513147	0.000577264	-0.450868909
ENSG00000164849	GPR146	2.505268	1.975936	2.068166	1.693805	1.914162	1.974905	1.380165	1.478724	1.699426	0.598362659	-0.147204349	0.053191475	-0.449933958
ENSG00000259305	ZHX1-C8orf76	1.390442	1.629583	1.742894	1.461572	0.587067	1.199731	1.228162	1.112166	0.973794	0.270067786	-0.453414967	0.184957605	-0.449924793
ENSG00000123124	WWP1	22.355316	21.069221	21.419532	22.715706	22.109866	21.944272	14.48316	15.078783	15.32788	0.00269997	0.18435679	2.41E-11	-0.44975357
ENSG00000114248	LRRC31	4.672239	4.528775	4.211369	2.334791	2.932245	2.640933	3.160355	2.883487	3.122531	0.0000101	-0.636837817	0.001272367	-0.449714192
ENSG00000185774	KCNIP4	3.447255	3.117284	3.712201	2.010323	1.958758	1.849444	2.467405	2.468773	2.172924	0.000111148	-0.674449712	0.012208698	-0.449514059
ENSG00000172006	ZNF554	3.74816	3.987309	3.974806	3.53457	3.249155	3.343565	2.823655	2.564935	2.601706	0.623313618	-0.086472905	0.001574618	-0.449466991
ENSG00000101654	RNMT	9.876592	11.389909	10.563573	10.531998	9.823812	10.517581	9.405748	7.077525	7.986155	0.77178977	-0.029456495	1.86E-08	-0.449301698
ENSG00000171316	CHD7	9.842339	9.895349	10.228316	8.931855	7.851173	8.319113	7.018386	6.54892	7.198419	0.124838828	-0.107954238	6.7E-13	-0.449099743
ENSG00000257529	PL36A-HNRNPH	34.620682	33.534885	16.603523	31.919636	30.839367	32.60434	11.878551	25.102259	21.023266	0.488648433	0.317204448	0.424240388	-0.449071334
NewGene_6721	--	0.662811	1.245436	0.916904	1.166724	1.01177	0.841115	0.629096	0.632631	0.689586	0.592055847	0.236272149	0.260454901	-0.44889617
ENSG00000145864	GABRB2	0.351924	0.386418	0.40254	0.834707	0.863425	1.015312	0.300566	0.261045	0.306874	1.34E-14	1.392765152	0.096756961	-0.448704188
ENSG00000176410	DNAJC30	2.908528	3.307053	2.897105	3.097708	2.790492	2.989021	2.010937	2.208281	2.030172	0.57191187	0.110421951	0.008648885	-0.448567744
ENSG00000226479	TMEM185B	12.516763	12.65379	12.480213	11.091336	9.706985	11.105281	8.059524	8.640525	9.173899	0.426394468	-0.091014534	0.00000373	-0.448124601
ENSG00000109133	TMEM33	33.343102	34.125636	35.003864	30.512697	41.011855	31.210707	25.958117	26.221543	26.361395	0.973662659	0.004168991	1.38E-08	-0.448019697
ENSG00000114948	ADAM23	0.1516	0.189662	0.217896	0.079627	0.093753	0.05262	0.091811	0.147533	0.148383	0.016295391	-1.149951041	0.321291021	-0.447592234
ENSG00000162073	PAQR4	6.034828	5.929774	6.626677	4.521476	4.974113	5.321731	3.969827	4.171846	4.301772	0.255527396	-0.153745764	0.000128458	-0.447313093
ENSG00000169105	CHST14	7.922249	7.440823	7.370711	5.890452	5.611274	6.224092	4.704115	5.178824	5.7664	0.035242008	-0.210926621	0.0000149	-0.447225411
ENSG00000135966	TGFBRAP1	10.243892	9.719741	10.575222	8.336806	8.837891	8.677324	7.154638	6.353261	7.833179	0.281679481	-0.091580162	7.66E-08	-0.447032733

Gene ID	Gene name	Ctrl-1 FPKM	Ctrl-2 FPKM	Ctrl-3 FPKM	IFNγ-1 FPKM	IFNγ-2 FPKM	IFNγ-3 FPKM	Sora-1 FPKM	Sora-2 FPKM	Sora-3 FPKM	Ctrl vs IFNγ DESeq2 FDR	Ctrl vs IFNγ DESeq2 log2FC	Ctrl vs Sora DESeq2 FDR	Ctrl vs Sora DESeq2 log2FC
ENSG00000204872	NAT8B	4.91176	2.833075	2.24929	1.130804	1.539062	0.928593	2.34873	1.880679	2.703214	0.000433141	-1.323345487	0.210776885	-0.446892343
ENSG00000185100	ADSSL1	1.73235	1.653246	1.140552	1.256205	0.920643	1.245552	0.927929	1.342487	1.30825	0.390837423	-0.300765198	0.148544826	-0.446764192
ENSG00000159433	STARD9	0.110658	0.16369	0.133374	0.113516	0.11877	0.104943	0.103011	0.093304	0.082968	0.767070828	-0.121777019	0.178904616	-0.446464736
ENSG00000111816	FRK	6.017563	6.962492	7.005626	6.719861	7.071235	6.948092	4.759605	4.190718	4.813056	0.009131824	0.201862171	3.84E-08	-0.446421167
ENSG00000138772	ANXA3	24.575562	26.532927	29.733412	6.364184	6.552664	5.930555	17.439714	18.215844	18.969017	8.76E-84	-1.918340655	0.00000424	-0.446412714
ENSG00000156011	PSD3	7.216017	6.999995	7.27981	6.649521	6.986098	6.704724	5.265848	4.448184	5.301637	0.188384933	0.094129473	6.01E-10	-0.446321316
ENSG00000119682	AREL1	12.057507	11.262718	11.348655	10.590221	10.638609	10.70874	7.725562	7.718642	8.559708	0.887499446	0.014819522	3.27E-09	-0.446203343
ENSG00000155189	AGPAT5	9.543215	10.550597	11.095697	12.234884	11.189089	12.296804	7.082398	7.097457	7.971828	0.000018	0.350145368	0.000000954	-0.446023585
ENSG00000089280	FUS	161.951839	209.143807	215.834505	213.050143	205.851432	207.124336	134.164039	167.078663	150.994223	0.30072476	0.061788921	1.06E-14	-0.445865484
ENSG00000163492	CCDC141	0.334345	0.233471	0.258162	0.498206	0.641807	0.773136	0.203452	0.193348	0.141376	2.72E-10	1.359050444	0.163143629	-0.445802362
ENSG00000135414	GDF11	3.384896	3.078965	3.201242	3.383178	3.425937	3.217729	2.083146	2.202697	2.349581	0.059417312	0.180608473	0.00000472	-0.445462984
ENSG00000284681	AC007240.1	1.079423	1.127019	1.538048	0.902705	0.761713	0.512667	0.701392	0.894994	1.000974	0.058196399	-0.641518272	0.152806421	-0.445377334
ENSG00000176903	PNMA1	15.305608	14.964863	14.643945	18.966465	18.076063	18.123346	10.777414	9.280678	10.913534	1.37E-10	0.443351345	0.000000325	-0.445308691
ENSG00000108590	MED31	5.421368	5.616093	4.733852	4.937523	5.390793	3.717427	3.7025	3.465168	4.214981	0.747615219	-0.079496044	0.010111591	-0.445274236
ENSG00000100478	AP4S1	1.690667	1.622897	1.458591	1.688254	1.51061	1.924247	0.897366	1.19807	1.30599	0.501453447	0.144993506	0.027402498	-0.445266865
ENSG00000100393	EP300	13.920409	13.756963	13.497932	14.697563	14.031097	14.555536	9.387174	8.502298	10.517394	0.0000487	0.218132062	2.25E-10	-0.445035807
ENSG00000164308	ERAP2	0.16945	0.1801	0.205127	4.723198	3.726518	3.806101	0.166974	0.127816	0.07904	1.03E-110	4.055154815	0.360223677	-0.444836546
ENSG00000110911	SLC11A2	24.316357	25.235295	24.321797	21.034819	20.408959	18.4581	17.004285	16.5839	16.5241	0.004762569	-0.184527029	9.53E-11	-0.444720752
ENSG00000157510	AFAP1L1	0.694902	0.438283	0.498541	0.314035	0.341561	0.267621	0.379183	0.359033	0.384456	0.050102404	-0.670877843	0.170975864	-0.444718435
ENSG00000198146	ZNF770	14.365226	16.983171	17.558762	15.998041	15.704811	16.076884	10.956359	11.268835	11.447766	0.202998735	0.114658254	0.000000192	-0.444593902
ENSG00000186660	ZFP91	38.133675	37.887489	40.572453	37.260887	35.983326	33.932274	27.16556	24.615891	28.675514	0.758720186	0.025302876	2.6E-12	-0.444461432
ENSG00000147408	CSGALNACT1	1.107543	1.259073	1.768574	0.814448	0.795179	0.976746	0.899013	1.073836	0.95986	0.03997825	-0.457336307	0.034011379	-0.443970584
ENSG00000132463	GRSF1	22.826801	36.238998	23.953147	25.557792	24.335521	24.724159	17.051343	15.790327	17.60699	0.986348802	-0.001508304	1.01E-13	-0.443932497
ENSG00000105982	RNF32	2.451513	2.766741	2.703291	2.180816	2.412437	3.046463	1.601207	1.852357	2.222904	0.673370625	0.111714302	0.033179622	-0.443854093
ENSG00000160285	LSS	43.685046	40.982841	38.026625	62.540271	63.721015	62.575245	27.508976	27.090732	30.906085	1.88E-33	0.751605999	6.84E-09	-0.443355691
ENSG00000186575	NF2	4.28533	4.019876	4.198534	4.078783	3.85693	3.897042	3.073655	2.866007	3.796723	0.485914307	0.079755183	0.00000841	-0.443143413
ENSG00000196081	ZNF724	1.909265	2.209907	1.876388	1.424399	1.269018	1.764197	1.31104	1.376465	1.580067	0.149648742	-0.313904966	0.023728869	-0.443032072
ENSG00000177888	ZBTB41	3.696458	4.714777	4.350228	5.555114	5.238927	5.50661	3.076184	2.852521	2.862461	0.000000019	0.501491636	0.000031	-0.442884932
NewGene_2646	--	1.953581	1.871615	1.948744	1.612277	1.636812	1.679292	1.500403	1.372119	1.45843	0.54664615	-0.093903711	0.000462913	-0.44273084
ENSG00000270629	NBPF14	3.375872	3.666304	2.843867	2.309818	2.526808	2.887106	2.551595	3.536144	3.379624	0.002407457	-0.50827134	0.008629302	-0.442656803
ENSG00000166860	ZBTB39	3.610363	3.356075	3.61882	3.519571	3.335923	3.343071	2.319181	2.502729	2.473311	0.428010906	0.093078028	0.0000346	-0.442556403
ENSG00000163735	CXCL5	27.252041	32.974045	38.661137	12.185628	10.117686	10.594563	21.621691	20.306128	26.477961	1.25E-44	-1.440627764	0.000129209	-0.442543028
ENSG00000116406	EDEM3	22.325232	22.370649	22.737539	19.531037	19.340204	19.296497	14.890328	14.981561	16.269723	0.425662762	-0.053042833	2.92E-13	-0.442442179
ENSG00000181896	ZNF101	3.401976	2.945112	3.152178	2.685345	2.887525	3.31315	2.428604	3.321523	2.7264	0.916383811	-0.022124401	0.004927237	-0.442272639
ENSG00000134049	IER3IP1	5.961903	7.081032	6.98017	7.160505	5.78133	6.595927	4.849584	4.28606	4.792508	0.395480851	0.112518541	0.0000573	-0.442218033
NewGene_5037	--	1.415023	0.977938	1.640463	0.898159	1.089255	1.420853	1.094224	0.782014	0.850204	0.835667407	-0.08506487	0.137379953	-0.441994274
ENSG00000138802	SEC24B	8.163011	8.755376	9.795035	9.131403	7.646939	10.143223	5.955312	6.161538	5.865849	0.230928016	0.128741455	0.00000333	-0.44193051
ENSG00000124104	SNX21	2.45464	2.744011	2.522146	2.742695	1.978565	1.939613	2.13342	2.116566	1.378026	0.829798832	-0.066244043	0.122842156	-0.441761354
ENSG00000185730	ZNF696	4.635603	5.172364	4.598871	4.198082	4.000716	4.747935	3.383112	3.282162	4.063843	0.450429056	-0.138476209	0.002842676	-0.441593693
ENSG00000231925	TAPBP	8.362568	8.137392	8.181033	47.652536	45.383683	47.601223	5.812059	5.136216	5.737673	0	2.685204684	0.00000176	-0.441550183
ENSG00000136897	MRPL50	15.876591	15.978302	15.793244	14.740851	13.919458	14.455339	10.574968	11.260215	11.043705	0.977698008	0.003224977	2.01E-08	-0.441165155
ENSG00000139618	BRCA2	3.618819	4.133873	3.8989	3.211141	3.114743	3.065162	2.553183	2.60739	2.815768	0.188384933	-0.124816882	0.00000077	-0.441018031
ENSG00000116132	PRRX1	1.693999	1.958022	2.283723	0.684733	0.212648	0.76569	1.377326	1.302241	1.611131	0.015263459	-1.757691654	0.01645223	-0.440616754
ENSG00000159259	CHAF1B	10.330954	11.003939	11.055371	11.103806	10.07185	10.668035	7.69786	7.045403	7.642	0.13656317	0.122681446	0.000000029	-0.440355696
ENSG00000135437	RDH5	1.311778	1.025188	0.969416	0.542152	0.775451	0.776315	0.82463	0.784466	0.707938	0.276420042	-0.499075392	0.288187125	-0.440252946
ENSG00000116984	MTR	8.036103	8.222565	8.681979	7.827145	7.305514	7.041391	5.677687	5.084263	6.466739	0.834949385	-0.020199724	3.43E-08	-0.440018364
ENSG00000197599	CCDC154	0.920319	0.927014	1.060915	1.07587	1.242713	1.543001	0.614258	0.644926	0.675114	0.032828047	0.631556585	0.217939678	-0.439966747
ENSG00000031691	CENPQ	8.047421	8.136144	9.961763	6.577045	6.049858	6.921824	6.221547	5.949774	5.876073	0.053417653	-0.271329526	0.001079177	-0.439839216
ENSG00000184206	GOLGA6L4	0.600515	0.500678	0.623535	0.443407	0.418121	0.3948	0.395103	0.353093	0.438633	0.389094949	-0.277683373	0.128808039	-0.439831148
NewGene_2838	--	1.851785	2.2876	2.830007	1.258568	0.91741	1.198276	2.090675	1.492206	1.78158	0.00002	-0.949615477	0.044675513	-0.439717719
ENSG00000177842	ZNF620	2.088767	2.173413	2.444337	2.233453	2.307145	2.634464	2.265894	1.833794	2.011394	0.239203308	0.215478185	0.019831355	-0.439545123
ENSG00000198742	SMURF1	10.397046	9.449961	9.639669	9.086681	8.87893	8.271261	7.001805	6.412837	6.975276	0.884679815	-0.016708059	2.71E-08	-0.439449986
ENSG00000107854	TNKS2	12.925988	13.018198	13.642447	12.572468	12.860217	12.246411	9.58962	8.202214	9.617317	0.317390074	0.076128141	3.87E-09	-0.439224296
ENSG00000163072	NOSTRIN	6.908314	7.008344	9.833613	3.88198	4.257115	3.644173	4.900592	5.039972	5.804821	0.000000046	-0.826854845	0.003277496	-0.439197357
NewGene_4007	--	1.772234	1.716313	1.639782	1.520658	1.509387	1.403491	1.527692	1.14805	0.9314	0.784458984	-0.065891745	0.032411391	-0.439001876
ENSG00000168887	C2orf68	17.603393	16.143492	15.236683	13.756792	13.343423	14.437375	10.978776	11.750878	11.923141	0.27557259	-0.104780698	0.00000082	-0.438915214
ENSG00000186951	PPARA	2.677968	2.06808	1.999616	2.534528	2.769849	2.510527	1.27937	1.177375	1.426884	0.451665336	0.825305758	0.670869797	-0.438630452
ENSG00000171574	ZNF584	2.414117	2.27511	2.430584	2.488309	2.706124	2.288666	1.709702	1.555999	1.50282	0.150333332	0.289524027	0.034236946	-0.438501735
ENSG00000048405	ZNF800	4.238257	4.300656	4.908142	4.701802	5.065201	4.759308	3.367634	2.630937	3.428168	0.04467106	0.228336108	0.000604771	-0.438459543
ENSG00000105173	CCNE1	15.195918	16.403326	17.533065	15.439734	15.903886	16.776453	11.270417	11.483455	11.318297	0.174666558	0.134916473	0.00000581	-0.438144008
ENSG00000105136	ZNF419	3.110502	2.57895	3.647342	2.521666	2.199633	2.409875	1.937683	1.871833	1.999182	0.591988928	-0.126310408	0.022706418	-0.437644697
ENSG00000006327	TNFRSF12A	78.559188	72.613245	71.052421	29.558542	30.085843	32.131941	51.445739	48.386006	54.252945	8.53E-56	-1.128630702	3.17E-09	-0.437541429
ENSG00000138119	MYOF	0.706571	0.582337	0.715804	1.11559	1.138733	1.078187	0.532806	0.42717	0.427566	0.00000214	0.805600409	0.055139514	-0.437460788
ENSG00000184985	SORCS2	0.365566	0.250775	0.236149	0.135909	0.157227	0.136177	0.184114	0.228512	0.187963	0.053261911	-0.74306526	0.24006421	-0.437243522
ENSG00000149639	SOGA1	1.413157	1.466183	1.262742	1.01901	1.080017	1.047268	0.991011	0.77348	1.124014	0.026344704	-0.255774301	0.000864113	-0.43721629
ENSG00000198252	STYX	4.27638	5.655417	5.970775	5.996205	6.109838	5.363112	3.634645	3.183067	4.218899	0.026847921	0.283561279	0.001306787	-0.437015386
ENSG00000135040	NAA35	17.326916	17.934786	18.013261	15.736855	16.373424	14.778659	12.457429	11.553629	12.780616	0.699470498	-0.040917823	0.000000024	-0.436767733
ENSG00000074657	ZNF532	16.013239	14.822234	15.478089	13.894799	11.548685	11.95167	9.1738	9.218569	11.379787	0.085728528	-0.139211811	4.5E-09	-0.436505447
NewGene_6434	--	2.770715	2.341161	1.998876	3.080282	2.881804	3.370027	1.881775	1.368061	1.691074	0.001345568	0.538654646	0.032163375	-0.436504675
ENSG00000105732	ZNF574	8.124659	7.261578	6.91276	6.509397	6.21556	6.440695	5.337354	4.986606	5.274974	0.618848284	-0.06871983	0.0000775	-0.436310725
ENSG00000196417	ZNF765	3.383015	3.476846	3.189212	2.510893	2.86659	2.84733	2.029907	2.276716	2.756094	0.376868833	-0.126838196	0.00138161	-0.435943979
ENSG00000183891	TTC32	3.125364	4.631983	4.078109	4.276713	4.142927	5.167897	3.014355	2.455763	2.756208	0.221636618	0.325722171	0.109642267	-0.435910231
ENSG00000113916	BCL6	14.063214	12.355671	13.107987	22.899757	23.470474	20.855086	8.87159	8.787972	9.553014	9.95E-37	0.896364323	0.00000135	-0.435851077
ENSG00000125864	BFSP1	4.661251	3.871774	3.310075	3.440435	2.587416	3.148892	2.753234	2.674436	2.793255	0.272750287	-0.223885815	0.012345734	-0.435823543

Gene ID	Gene name	Ctrl-1 FPKM	Ctrl-2 FPKM	Ctrl-3 FPKM	IFNγ-1 FPKM	IFNγ-2 FPKM	IFNγ-3 FPKM	Sora-1 FPKM	Sora-2 FPKM	Sora-3 FPKM	Ctrl vs IFNγ DESeq2 FDR	Ctrl vs IFNγ DESeq2 log2FC	Ctrl vs Sora DESeq2 FDR	Ctrl vs Sora DESeq2 log2FC
ENSG00000113356	POLR3G	1.492597	1.873016	1.931684	1.719122	1.165893	1.542951	1.417005	1.217643	1.025964	0.947136939	0.022095581	0.055677144	-0.435582403
NewGene_4265	--	1.059971	0.902335	1.001684	0.600224	0.749665	0.830717	0.621881	0.8268	0.531504	0.570948405	-0.269832256	0.301280047	-0.435399486
ENSG00000061676	NCKAP1	9.759525	9.940345	10.113554	10.01868	9.406752	21.396644	6.26489	6.630158	7.889425	0.012078742	0.13590889	5.54E-10	-0.435155927
ENSG00000166266	CUL5	4.615772	5.719715	5.84586	5.587876	5.55537	5.769159	3.779036	3.288326	3.994144	0.040372713	0.215486991	0.0000814	-0.434961451
ENSG00000129315	CCNT1	9.125581	10.104762	10.183257	10.381225	9.593582	9.756187	6.442576	6.307624	7.297466	0.036182556	0.161214626	7.22E-08	-0.434946869
ENSG00000197024	ZNF398	3.079218	3.371587	3.085222	2.997284	2.798508	3.201503	2.277529	1.9291	2.224835	0.252336282	0.144805128	0.000338003	-0.434281098
ENSG00000196653	ZNF502	2.775686	2.950906	3.016089	2.142395	2.218166	2.10578	2.055548	2.017669	1.823054	0.375481153	-0.158164113	0.007502048	-0.434251568
ENSG00000136935	GOLGA1	5.841352	6.034401	6.074673	6.228371	6.052635	5.943601	4.093257	4.0635	4.305193	0.057262546	0.170844418	0.00000196	-0.433918382
ENSG00000137094	DNAJB5	4.938839	4.251508	4.784238	4.48646	4.331878	4.273989	2.860591	3.357624	3.753076	0.783661849	0.049621286	0.004643154	-0.433897543
ENSG00000118322	ATP10B	0.21702	0.207114	0.152681	0.179847	0.123829	0.090515	0.146638	0.113476	0.134367	0.466966435	-0.34823795	0.275352375	-0.433831311
ENSG00000091513	TF	217.284542	288.399335	249.493421	325.384975	347.802503	296.726669	208.080251	191.688945	204.864295	0.241520918	0.329615209	0.097152037	-0.433505926
ENSG00000131943	C19orf12	9.542908	12.154456	9.914222	24.378625	27.503223	24.530265	7.289339	7.287778	7.132478	1.6E-67	1.325182887	0.0000155	-0.433390287
ENSG00000198815	FOXJ3	12.582171	13.788161	14.927595	11.481725	10.022185	10.45709	10.604993	9.019961	9.631205	0.000739852	-0.262017901	0.000000376	-0.433383127
ENSG00000157214	STEAP2	0.411755	0.441198	0.357118	0.354198	0.330674	0.376166	0.390317	0.222216	0.220199	0.962729549	-0.018527635	0.170790535	-0.433320953
ENSG00000163319	MRPS18C	14.501522	14.701413	14.72477	15.201984	13.137828	12.642755	10.322259	11.653626	9.634325	0.976397779	0.004797798	0.000214918	-0.433272656
ENSG00000065526	SPEN	5.261666	5.02628	4.853262	5.204466	4.835054	4.828307	3.345172	3.050652	4.15889	0.170993488	0.110024542	0.00000928	-0.433270743
ENSG00000105856	HBP1	24.341691	22.015739	21.535281	22.062358	23.643303	22.503015	17.036786	15.833178	15.462399	0.086508214	0.147067834	0.000000293	-0.43306035
ENSG00000184208	C22orf46	5.915368	5.758527	5.401319	4.726757	4.511015	4.660034	3.925914	3.951565	3.978116	0.134322428	-0.150162639	0.00000566	-0.432878376
ENSG00000132692	BCAN	17.922259	22.768463	18.124108	13.126551	12.386162	12.88097	13.527055	12.991804	13.671926	0.000216478	-0.511961583	0.001603075	-0.432750203
ENSG00000196312	MFSD14C	4.227273	3.718073	3.951418	4.069748	3.875719	3.277671	3.193477	3.113228	2.775729	0.61378633	0.066123764	0.0000773	-0.432690352
ENSG00000165899	OTOGL	0.36748	0.36167	0.37556	0.171329	0.274099	0.163497	0.28353	0.266487	0.227441	0.007942282	-0.718727538	0.072520836	-0.432627813
NewGene_4703	--	1.156281	0.887091	0.978842	1.08362	1.074456	1.220356	0.644869	0.750579	0.697146	0.188329666	0.309905856	0.076035922	-0.432427878
ENSG00000135164	DMTF1	22.390295	23.158681	24.22509	21.467143	19.769515	18.446302	14.583646	14.748905	17.330089	0.302047108	-0.091336506	7.86E-09	-0.432357209
ENSG00000064651	SLC12A2	8.180722	7.649231	9.445422	4.16124	3.944275	4.266458	5.411439	5.490322	6.821986	5.85E-25	-0.877473488	0.0000125	-0.431961471
ENSG00000066923	STAG3	1.025817	1.010981	1.022252	1.11375	1.020019	0.960855	0.724087	0.723601	0.724017	0.591273441	0.147709293	0.07341184	-0.431783033
ENSG00000254535	PABPC4L	0.586702	1.073664	0.808134	1.018969	1.003039	0.927587	0.656202	0.490971	0.570945	0.106877423	0.403809377	0.114696688	-0.431505054
ENSG00000104524	PYCR3	6.630899	6.593647	5.352029	5.192269	5.326402	5.912676	4.363931	3.646456	4.972511	0.958082541	-0.011188898	0.00432329	-0.431482619
ENSG00000169740	ZNF32	12.01997	11.694382	8.906865	9.522669	8.391166	8.355095	8.213031	7.867381	6.790291	0.310347498	-0.174162667	0.007671776	-0.431337878
ENSG00000100100	PIK3IP1	4.350368	4.128118	3.401811	2.52438	2.290097	2.762367	3.253546	2.475607	2.539239	0.002240859	-0.499184424	0.013621339	-0.431310289
ENSG00000164002	EXO5	6.832239	5.265204	5.689842	4.242089	5.200223	5.411969	3.757401	4.448549	4.288261	0.398128125	-0.141188128	0.002081225	-0.431267764
NewGene_1487	--	0.706587	1.554953	1.121887	1.079472	0.798506	0.978008	0.607095	0.905492	0.830033	0.857026623	-0.106460046	0.354929514	-0.431218246
NewGene_4284	--	1.46504	1.679065	1.353888	0.945938	0.840616	0.972483	0.961339	0.950086	1.051875	0.149664577	-0.388552888	0.088199274	-0.430954679
ENSG00000121879	PIK3CA	5.210924	5.971641	6.003169	5.995642	5.400168	5.002311	3.957864	3.792334	4.239141	0.705904993	0.04249792	0.000000376	-0.430927303
ENSG00000066654	THUMPD1	15.371354	16.007984	15.353613	16.927388	15.470912	16.25954	11.354604	10.300273	10.657271	0.00283212	0.20209309	1.97E-08	-0.430701732
ENSG00000102218	RP2	6.60917	6.841115	7.399131	4.913265	4.748431	4.787097	4.866703	4.183938	5.501827	0.0000535	-0.381584471	0.0000804	-0.430184341
ENSG00000171714	ANO5	1.481949	1.85016	1.86122	1.732965	1.757663	1.97827	1.238006	1.119002	1.252733	0.143386278	0.22471108	0.003687167	-0.4299885
ENSG00000165983	PTER	5.423413	4.68363	6.4535	5.838387	3.885454	4.936452	4.175608	4.289484	3.20177	0.880584609	-0.024885895	0.000158441	-0.429722603
ENSG00000114098	ARMC8	13.519407	12.511664	16.332494	13.616815	12.091405	12.558383	11.154091	10.800779	12.862997	0.930787487	0.011395205	0.000000136	-0.429352393
ENSG00000136451	VEZF1	22.451411	21.787841	21.248358	19.103458	19.278489	19.160213	14.513157	13.918409	15.785196	0.848789089	-0.018662208	1.83E-09	-0.429051404
ENSG00000050344	NFE2L3	20.614626	21.236297	24.14278	17.691131	18.389986	16.258207	15.035657	14.804071	16.137546	0.028321746	-0.1925562	0.000000118	-0.428958632
ENSG00000115183	TANC1	3.758939	3.938506	4.106132	4.755868	4.286314	4.069408	2.798972	2.466677	2.970648	0.000988459	0.297224424	0.00000965	-0.428848737
NewGene_847	--	0.479279	0.424554	0.786959	0.477784	0.558438	0.683498	0.50892	0.379073	0.258022	0.693743249	0.205097124	0.381201772	-0.428369711
ENSG00000128271	ADORA2A	4.023708	0.734212	2.872282	2.311992	3.05741	2.805167	0.561866	0.441252	4.322595	0.889945453	0.235747871	0.822342589	-0.428317629
ENSG00000001629	ANKIB1	8.195414	7.974364	8.046156	8.018591	7.546508	7.433417	5.362483	5.32074	6.451313	0.508650078	0.060120082	0.000000217	-0.428290364
NewGene_4407	--	3.395767	2.45343	2.839502	3.724196	3.607816	3.989169	2.139752	1.548265	2.40851	0.144068746	0.517641049	0.29844769	-0.428176845
ENSG00000197763	TXNRD3	4.441205	5.146292	4.746173	4.267961	4.229366	4.405886	4.156919	3.635274	2.613706	0.855944088	-0.033315845	0.009145819	-0.428073801
ENSG00000131381	RBSN	7.444799	7.832744	7.480188	7.096639	7.306611	7.104544	5.300645	5.027648	5.657421	0.501534029	0.061270872	5.85E-08	-0.427875194
ENSG00000174238	PITPNA	15.501476	11.729472	12.117065	10.622042	13.487253	9.692923	7.644888	11.420223	8.871085	0.257411243	-0.103383655	0.000000915	-0.427399095
ENSG00000114450	GNB4	1.361259	1.593319	1.953252	4.231045	3.854917	4.198806	1.152516	0.97558	1.274772	3.17E-36	1.475435495	0.013344614	-0.427329108
ENSG00000123908	AGO2	6.508488	6.876361	7.703454	6.292586	5.532924	6.032924	4.671758	4.74819	5.349456	0.32134599	-0.08913505	8.24E-08	-0.42731398
ENSG00000115760	BIRC6	7.022559	7.200877	6.902877	6.961617	7.04931	6.926427	4.801723	4.743263	5.201496	0.029333277	0.133292391	1.48E-11	-0.426966319
ENSG00000122194	PLG	0.937175	0.543389	0.643292	0.569526	0.480027	0.483647	0.450245	0.414506	0.614633	0.394899676	-0.338614263	0.242103689	-0.426860062
ENSG00000110693	SOX6	8.498322	11.360019	8.970281	6.223467	6.115673	6.11835	6.065406	5.919017	6.68932	4.37E-08	-0.348016115	1.62E-09	-0.426718847
ENSG00000141378	PTRH2	29.829816	28.694981	27.01694	25.44901	26.012834	27.398929	21.329393	21.85601	20.55372	0.729627134	0.035819357	0.000000617	-0.426679925
ENSG00000146223	RPL7L1	55.538786	55.554651	55.596028	45.63948	43.599227	45.5324	37.028093	38.416697	40.811292	0.00275157	-0.155491219	2.8E-13	-0.426631424
ENSG00000155666	KDM8	4.035956	3.98708	4.019068	3.223398	3.055764	3.069407	2.635554	2.877725	2.89247	0.155778574	-0.217734817	0.003474547	-0.42650856
ENSG00000185621	LMLN	0.333945	0.34066	0.252874	0.290825	0.165263	0.219396	0.221446	0.196068	0.195391	0.317179668	-0.374267785	0.192292905	-0.426387325
ENSG00000117713	ARID1A	14.136181	13.720607	14.360576	15.514103	13.725138	13.975034	9.383973	8.447841	10.402537	0.0172074	0.156529009	3.88E-09	-0.426230878
ENSG00000127995	CASD1	8.289763	9.151714	9.540576	7.851427	7.380617	7.738311	6.287493	5.871571	6.686934	0.427388447	-0.084633287	0.0000041	-0.42613883
ENSG00000089876	DHX32	21.586769	19.951489	21.066113	12.857177	13.10298	12.869991	13.132266	15.680161	15.065069	2.63E-12	-0.525421489	0.00000325	-0.426087617
ENSG00000134278	SPIRE1	1.406493	1.745865	2.289013	0.606981	0.651542	0.610323	0.981132	1.24591	1.721519	1.06E-13	-1.38932563	0.053586974	-0.426015558
ENSG00000198105	ZNF248	1.703421	2.489052	2.315618	2.066968	2.164485	2.062287	1.664389	1.334758	1.397292	0.574667169	0.11316379	0.017102863	-0.425737504
ENSG00000138867	GUCD1	25.730188	25.148181	23.193937	32.571326	33.38428	33.146759	16.581096	16.99943	18.360768	1.24E-22	0.576887406	8.99E-09	-0.425665076
NewGene_675	--	23.995259	21.176997	19.034005	11.008534	11.773464	11.841992	17.874125	19.813948	22.149876	2.96E-31	-0.779460815	4.28E-09	-0.425471803
ENSG00000114698	PLSCR4	3.200785	3.352682	2.386049	3.196556	3.128484	3.060598	2.035521	2.391126	2.062586	0.193886979	0.242008971	0.020108142	-0.425437132
ENSG00000229676	ZNF492	0.870041	1.285759	1.333765	0.99317	0.795718	0.991075	0.735045	0.807764	0.895775	0.527117044	-0.177237422	0.069662903	-0.425339638
ENSG00000177485	ZBTB33	8.653318	9.128929	9.576853	10.10244	9.926659	9.980326	6.315856	6.157461	6.870693	0.0000721	0.28634192	0.000000427	-0.425332986
ENSG00000116095	PLEKHA3	0.866802	1.164074	1.020475	1.164477	0.907984	0.98802	0.697748	0.702783	0.861756	0.197582283	0.194396207	0.003432471	-0.425277309
ENSG00000178623	GPR35	14.783683	15.258653	12.221629	12.272176	12.281657	12.623013	9.794512	9.143449	11.677086	0.769112664	-0.037633271	0.0000391	-0.425151959
ENSG00000101290	CDS2	5.632947	5.297769	5.182851	5.756391	5.954711	5.783597	4.015067	3.41097	3.838986	0.000492259	0.251173115	0.000000546	-0.424980045
ENSG00000099910	KLHL22	9.170922	9.969425	7.729121	9.215737	7.840099	9.109346	5.99985	5.90841	6.260136	0.786075536	0.040933803	0.000166281	-0.424918568
ENSG00000220205	VAMP2	6.059884	6.797391	6.037466	7.125295	5.664524	6.272242	4.258279	4.152161	4.224092	0.319874424	0.150357267	0.001341581	-0.424858842
ENSG00000113441	LNPEP	1.739724	2.05006	2.000338	3.442472	2.983931	3.129168	1.412046	1.113331	1.531364	5.78E-24	0.869417146	0.000485758	-0.424641523
ENSG00000152332	UHMK1	11.608219	13.425615	13.257379	16.619472	15.800263	16.30975	9.24021	8.453785	9.054319	6.36E-15	0.493631406	2.27E-08	-0.424167354

Gene ID	Gene name	Ctrl-1 FPKM	Ctrl-2 FPKM	Ctrl-3 FPKM	IFNγ-1 FPKM	IFNγ-2 FPKM	IFNγ-3 FPKM	Sora-1 FPKM	Sora-2 FPKM	Sora-3 FPKM	Ctrl vs IFNγ DESeq2 FDR	Ctrl vs IFNγ DESeq2 log2FC	Ctrl vs Sora DESeq2 FDR	Ctrl vs Sora DESeq2 log2FC
ENSG00000103150	MLYCD	0.532959	0.556841	0.527599	0.576318	0.561447	0.534544	0.383725	0.375775	0.370289	0.268775847	0.193496914	0.012579651	-0.423960984
ENSG00000070404	FSTL3	8.885307	9.246049	7.524712	4.267347	3.928759	4.138758	6.22834	5.939158	5.929202	4.28E-19	-0.972546981	0.000181647	-0.423709631
ENSG00000164588	HCN1	0.283492	0.152897	0.222527	0.089535	0.028616	0.066671	0.155801	0.135556	0.180786	0.0000734	-1.699097037	0.265275022	-0.423170105
ENSG00000083817	ZNF416	2.57445	2.331792	2.692854	2.24606	2.223663	2.118435	1.711814	2.12542	1.446041	0.802747044	-0.058559439	0.042542196	-0.422900507
ENSG00000071246	VASH1	0.609528	0.664455	0.743068	0.591999	0.553484	0.60505	0.35136	0.469892	0.63833	0.796078229	-0.077536899	0.105825041	-0.422579817
ENSG00000079332	SAR1A	27.611099	29.561883	27.588243	27.915426	29.130477	29.846683	20.083096	20.265626	21.427943	0.000329962	0.213491634	7.14E-11	-0.422520805
ENSG00000133256	PDE6B	1.065202	0.515067	0.755192	0.71372	0.645406	0.478574	0.296547	0.368293	0.681982	0.955226678	-0.027833345	0.280274944	-0.422016449
ENSG00000197323	TRIM33	11.559525	13.215676	14.267977	12.884905	11.934834	11.779167	9.440186	8.412166	9.687863	0.604406065	0.052313695	0.000000204	-0.421889602
ENSG00000104765	BNIP3L	8.279751	7.485128	6.014065	5.511556	5.973783	5.279406	4.915755	4.86131	5.391428	0.089045853	-0.228350508	0.000616399	-0.42117151
ENSG00000187815	ZFP69	1.260959	1.836884	1.393775	0.984912	1.198795	1.087335	1.003452	0.89162	1.076884	0.395103605	-0.227328829	0.070176912	-0.421060607
ENSG00000143126	CELSR2	4.550531	3.765788	3.419831	3.054024	3.128077	2.914071	2.573577	2.421067	3.255124	0.052480202	-0.221084689	0.000543502	-0.420885548
ENSG00000140836	ZFHX3	1.855118	1.837302	1.780489	2.211438	2.261275	2.060453	1.369367	1.175241	1.594554	0.00000484	0.366559131	0.00000768	-0.420802285
ENSG00000174514	MFSD4A	0.316015	0.350939	0.360704	0.512009	0.446067	0.297017	0.290462	0.206711	0.221832	0.270097626	0.427413036	0.289821407	-0.420755395
NewGene_390	--	1.748933	1.321346	1.392086	0.559462	1.468708	0.862387	0.835155	0.694932	1.615881	0.404279871	-0.480436011	0.413123545	-0.42074233
ENSG00000131043	AAR2	17.887895	18.87633	17.341958	17.41267	15.879519	16.893747	12.559003	12.816117	12.340377	0.989770837	-0.001610465	0.000000533	-0.42070437
ENSG00000104331	IMPAD1	15.010954	15.125505	16.331505	12.223045	11.852023	11.887567	11.11993	9.471952	12.855579	0.001098161	-0.207307894	0.00000145	-0.420567233
ENSG00000101152	DNAJC5	23.973244	22.242538	20.329954	22.276246	21.454735	22.858027	15.096206	14.394915	17.243443	0.055534699	0.147894944	0.000000165	-0.420513754
ENSG00000090857	PDPR	7.919811	8.388436	8.662449	8.521571	7.857006	7.972353	5.568447	5.08023	5.889492	0.001968115	0.195419258	4.02E-08	-0.420340353
ENSG00000152229	PSTPIP2	2.282288	2.678135	2.690756	4.329773	3.869433	3.579671	1.811341	1.6632	1.879653	1.52E-08	0.76847925	0.0126747	-0.420208005
ENSG00000161048	NAPEPLD	1.997972	1.963403	2.062469	1.990042	2.0441	1.844695	1.581041	1.43318	1.612393	0.43646571	0.133618115	0.007145634	-0.42012765
ENSG00000171617	ENC1	25.560172	24.612739	26.142728	15.167553	15.823662	15.043125	17.259416	16.472242	19.426789	7.31E-24	-0.575759818	4.58E-10	-0.419585208
NewGene_1260	--	1.934437	1.558643	2.245076	1.920647	1.430565	1.540935	1.769462	1.254471	1.269259	0.904672423	0.047435479	0.171938719	-0.419418302
ENSG00000187866	FAM122A	3.591991	3.698121	3.304743	3.503376	3.642208	3.573751	2.393827	2.164424	2.893225	0.182074012	0.164153198	0.001163496	-0.419308116
ENSG00000131127	ZNF141	5.630201	6.336744	6.048022	5.981749	7.205022	6.419939	4.85012	3.849291	3.478662	0.086015781	0.206700489	0.002311119	-0.419176238
ENSG00000174640	SLCO2A1	0.517485	0.492817	0.537637	0.197944	0.176029	0.188077	0.361013	0.332163	0.394642	3.67E-09	-1.309522488	0.048083111	-0.418577775
ENSG00000158669	GPAT4	23.794811	22.229826	21.704934	18.455858	18.693104	17.945877	15.526414	14.315168	17.738122	0.025228446	-0.146865103	2.65E-08	-0.418462266
ENSG00000144791	LIMD1	2.37144	2.533412	2.234265	2.039838	1.880059	2.130156	1.697	1.487641	1.837856	0.681193672	-0.055353341	0.0000997	-0.418155801
NewGene_4367	--	7.078039	7.350268	7.066514	5.28526	5.072433	5.564766	4.706524	4.717481	5.366786	0.001266793	-0.264410522	0.000000277	-0.417829103
ENSG00000214944	ARHGEF28	5.336192	6.011254	5.362343	6.821779	6.317801	6.633119	4.118478	3.406509	4.551416	0.00000105	0.389847969	0.0000246	-0.417724902
ENSG00000141449	GREB1L	0.305113	0.294238	0.324227	0.187326	0.189284	0.135958	0.26193	0.32902	0.217469	0.089011788	-0.579293266	0.220092764	-0.417712673
ENSG00000145734	BDP1	4.40314	4.853063	4.881355	4.414463	4.686972	4.02041	3.153807	3.222636	3.909807	0.802300032	0.028433632	0.000000274	-0.417698934
ENSG00000109184	DCUN1D4	15.881079	18.707358	20.7991	18.772877	18.00641	16.538629	15.15635	13.080805	14.081044	0.611970775	0.058154826	0.00000622	-0.417313848
ENSG00000151422	FER	1.226063	1.242365	1.077204	1.037945	1.017883	0.967917	0.974465	0.879007	0.756434	0.219402591	-0.172800179	0.00239486	-0.416942234
ENSG00000107282	APBA1	0.845446	0.902384	0.7895	0.443388	0.435936	0.462713	0.714315	0.491566	0.584769	0.0000792	-0.765879353	0.046031295	-0.416411578
ENSG00000134780	DAGLA	15.925232	13.830739	13.677728	14.209041	14.326168	14.0957	9.984553	9.431306	11.173124	0.146492356	0.121274208	0.000000454	-0.416105455
ENSG00000026652	AGPAT4	0.714231	0.581118	0.687037	0.835252	0.983509	0.782804	0.494806	0.57315	0.502271	0.001268133	0.57123624	0.048269994	-0.416035786
ENSG00000116747	TROVE2	8.793249	9.128367	9.290178	11.685059	8.943024	8.136461	10.38475	6.972472	7.679495	0.707731833	-0.041520238	0.00000116	-0.415796243
ENSG00000029153	ARNTL2	3.345884	3.40016	3.609911	3.050558	2.895264	4.620494	2.85726	2.295743	2.937808	0.932918593	-0.013429618	0.00024846	-0.41577988
ENSG00000177054	ZDHHC13	13.503898	12.678811	13.042582	10.873713	11.413042	11.264111	9.455164	8.908076	9.102051	0.531247802	-0.068891253	0.00000592	-0.415539189
ENSG00000228594	FNDC10	0.871157	0.814677	1.145094	0.84151	0.684439	0.880564	0.585532	0.588278	0.809859	0.854804261	-0.082138613	0.228339148	-0.415441886
ENSG00000145390	USP53	3.948874	4.45726	5.319524	3.511256	3.456803	3.184714	3.022381	3.307193	3.228485	0.020698911	-0.281154038	0.000638771	-0.415285337
ENSG00000115762	PLEKHB2	37.566187	38.425672	39.587067	37.07292	37.786804	36.046025	25.650069	28.054393	26.885736	0.103366833	0.102805349	7.32E-11	-0.415181891
ENSG00000168496	FEN1	80.961465	80.526592	76.63621	67.066429	65.631141	69.093165	53.728907	56.649385	56.78735	0.144377121	-0.091862616	5.97E-11	-0.41517552
ENSG00000111875	ASF1A	14.511717	14.70218	15.019048	13.656053	13.369908	14.157931	10.625088	10.158339	10.30866	0.677998356	0.045158886	0.00000172	-0.415067338
ENSG00000213516	RBMXL1	2.206388	2.849709	2.880044	4.154216	3.691595	3.740733	1.751175	1.751137	2.039471	2.77E-09	0.684576128	0.004151927	-0.414961796
ENSG00000177465	ACOT4	1.955636	1.841091	1.729972	2.324829	2.275925	1.919557	1.640957	1.294902	0.935479	0.063201822	0.377899393	0.106338743	-0.414933117
ENSG00000175455	CCDC14	19.829273	20.664411	21.744396	18.156107	17.780128	15.000703	15.116955	13.906432	15.748547	0.139836524	-0.132841709	6.04E-09	-0.414472058
ENSG00000188994	ZNF292	8.225438	8.378071	7.974987	7.700004	8.108959	7.485621	5.96642	5.661177	5.767931	0.426722914	0.068275725	2.5E-09	-0.414398662
ENSG00000132109	TRIM21	4.241387	3.769167	4.862303	27.55912	25.820711	28.461913	3.232289	2.612474	3.002797	3.15E-194	2.844155538	0.018876627	-0.414377711
ENSG00000137145	DENND4C	7.101954	6.578508	7.244632	9.444699	7.83258	8.674005	5.496158	4.699861	5.367591	0.000000253	0.400992431	0.00000383	-0.414041792
ENSG00000100221	JOSD1	17.331659	17.306236	16.92388	14.250305	14.20687	13.928107	12.121358	11.915818	12.374682	0.085543341	-0.125800796	6.77E-09	-0.414020507
ENSG00000184384	MAML2	0.604864	0.444113	0.463857	0.180348	0.20316	0.193059	0.398125	0.269134	0.408855	0.00000525	-1.219068521	0.142615243	-0.413893019
ENSG00000187522	HSPA14	11.525177	11.818883	11.736567	11.624535	12.038564	11.871566	8.245291	8.761808	7.786317	0.119610068	0.160056078	0.000158071	-0.413765635
ENSG00000149658	YTHDF1	28.144298	27.45287	28.495979	27.733766	27.072316	28.539364	19.873712	18.501306	20.951281	0.060117397	0.123965133	4.8E-10	-0.41376479
ENSG00000111674	ENO2	2.072185	1.434768	1.224092	0.469238	0.299489	0.36562	1.058784	1.019868	1.315582	1.66E-10	-1.88961476	0.117309073	-0.413734199
ENSG00000055211	GINM1	5.911764	6.148088	6.745829	6.493917	6.111383	6.389743	4.213763	4.458642	4.322181	0.247783531	0.161868817	0.002556464	-0.413310418
ENSG00000122417	ODF2L	2.861335	3.287073	3.253159	3.124618	2.948353	2.748619	2.912124	2.425093	2.978572	0.230928016	-0.193376044	0.004057175	-0.413302398
ENSG00000101040	ZMYND8	29.328547	27.355358	30.033344	25.34975	25.548545	25.449132	20.70873	20.550623	20.291809	0.887978081	-0.012467891	5.23E-11	-0.413158442
ENSG00000198586	TLK1	15.221742	14.690382	16.501834	14.693994	12.817362	13.755009	11.423289	8.855146	9.956126	0.889239124	-0.014602951	0.000000367	-0.413120303
ENSG00000142556	ZNF614	3.023822	3.573839	2.932967	2.87822	2.890594	2.242194	2.188438	2.483597	2.145752	0.646768043	-0.086507638	0.003019706	-0.413000335
ENSG00000060069	CTDP1	9.11048	8.449614	7.830341	8.965247	7.922777	7.86569	6.002937	5.576914	5.960334	0.258577514	0.11171146	0.00000446	-0.412876947
ENSG00000072121	ZFYVE26	4.695825	4.793508	4.385659	5.233868	4.810291	4.78366	3.1009	3.169878	3.502193	0.00057378	0.256845221	0.00000168	-0.412658098
ENSG00000185129	PURA	1.101364	1.054218	1.066579	0.978974	1.133888	1.157392	0.779187	0.696321	0.794657	0.238725108	0.171772435	0.00161073	-0.412638759
ENSG00000112033	PPARD	5.619772	4.646096	4.831862	4.622866	4.445763	4.64013	3.375462	3.152649	4.068535	0.92133605	0.01734054	0.001214003	-0.412632092
ENSG00000132952	USPL1	7.58652	7.844497	8.338143	7.356844	7.194018	6.947853	5.656687	5.384842	5.756481	0.976457703	-0.004253412	0.00000642	-0.41232644
ENSG00000162408	NOL9	3.427054	3.129915	3.378874	3.081183	2.672148	3.01564	2.531876	2.241058	2.231223	0.822475674	-0.032941744	0.00020097	-0.411562705
NewGene_3699	--	2.508978	2.075717	2.907303	1.380129	1.45204	1.620899	1.445717	1.85364	1.986473	0.028727394	-0.599884351	0.131590139	-0.411389132
ENSG00000184209	SNRNP35	8.857596	8.004124	8.168756	7.966323	8.36353	8.090112	5.613905	6.034115	5.53547	0.361004405	0.120707082	0.000659195	-0.411284823
ENSG00000145147	SLIT2	5.283503	5.191161	5.408079	4.410419	4.57077	4.267042	3.808649	3.517687	4.331345	0.268795685	-0.117271797	0.0000365	-0.410787073
ENSG00000012232	EXTL3	11.174507	10.550672	10.387336	5.432981	5.418219	5.535423	6.828964	7.121462	8.706234	2.71E-35	-0.825822907	0.00000345	-0.410733204
ENSG00000204271	SPIN3	2.31619	2.511343	2.594818	2.319055	1.991632	2.12487	1.506301	2.052739	2.020337	0.813351021	-0.047566258	0.008896443	-0.410708759
ENSG00000155729	KCTD18	3.112207	2.929603	2.51881	2.592457	2.451749	2.827039	1.77873	2.136544	2.245631	0.857026623	0.042567152	0.023163352	-0.410698044
ENSG00000180964	TCEAL8	33.320514	29.69784	27.179256	24.707927	27.783901	25.829909	21.037207	21.276947	20.466591	0.650354808	-0.0612242	0.0000725	-0.410658077
ENSG00000242110	AMACR	9.975264	8.672323	8.01523	8.336892	7.961434	7.625414	6.87629	5.66161	6.681246	0.998966788	-0.000381274	0.000157097	-0.410651001

Gene ID	Gene name	Ctrl-1_FPKM	Ctrl-2_FPKM	Ctrl-3_FPKM	IFNγ-1_FPKM	IFNγ-2_FPKM	IFNγ-3_FPKM	Sora-1_FPKM	Sora-2_FPKM	Sora-3_FPKM	Ctrl_vs_IFNγ_DESeq2_FDR	Ctrl_vs_IFNγ_DESeq2_log2FC	Ctrl_vs_Sora_DESeq2_FDR	Ctrl_vs_Sora_DESeq2_log2FC
ENSG00000064393	HIPK2	3.797645	3.730715	3.857876	4.901021	4.337584	4.852937	2.754272	2.35303	3.050255	1.09E-11	0.454385527	0.00000139	-0.410478364
ENSG00000254726	MEX3A	26.064058	23.484678	23.701473	15.092327	14.953411	15.381544	17.258787	16.456955	17.996572	1.7E-19	-0.542014963	7.32E-10	-0.410453522
ENSG00000170044	ZPLD1	1.756232	1.994947	1.776809	0.960426	1.079826	0.956337	1.360875	1.157402	1.381668	0.0000419	-0.735676639	0.021622088	-0.410422688
ENSG00000112029	FBXO5	18.331032	20.737791	21.753863	18.191317	17.219037	17.056193	15.078751	13.960561	14.028433	0.56548564	-0.06352613	0.0000103	-0.410388558
ENSG00000147799	ARHGAP39	2.015136	1.816298	1.621573	1.474092	1.478323	1.330623	1.235069	1.329193	1.281518	0.258770719	-0.204895832	0.013086758	-0.4102302
ENSG00000110422	HIPK3	11.049137	11.155438	11.244068	12.33608	12.780869	12.564757	7.967284	7.109663	8.585927	4.89E-08	0.31990416	1.86E-08	-0.4101904
ENSG00000119862	LGALSL	7.879119	9.162116	8.481566	8.896972	9.199734	8.926693	5.877725	5.961532	6.054638	0.009429323	0.235833402	0.0000202	-0.410127206
ENSG00000112320	SOBP	10.810868	11.09333	11.187196	7.070652	6.431801	6.619306	7.832015	6.982718	9.022857	4.64E-18	-0.584814607	0.00000122	-0.410115636
ENSG00000172840	PDP2	5.078029	5.570337	5.86311	5.273978	5.442169	5.513884	4.314993	3.620759	3.696252	0.361919656	0.090231285	0.0000789	-0.409903648
ENSG00000139746	RBM26	17.893959	20.594162	23.140342	23.238088	20.146236	19.69475	14.240373	13.969819	15.212889	0.134750556	0.156432969	0.0000118	-0.409578189
ENSG00000095585	BLNK	1.770647	1.126874	1.12433	8.226884	7.303519	6.727576	1.142287	0.722526	0.925282	3.78E-47	2.604338606	0.24043375	-0.409213491
ENSG00000164366	CCDC127	3.212137	3.773479	3.297512	2.770548	2.954852	2.918199	2.439364	2.202374	2.42117	0.162342505	-0.157821389	0.000065	-0.40917004
ENSG00000157350	ST3GAL2	8.419821	7.857572	6.225053	6.451246	6.54383	5.791408	5.175623	4.729379	7.314856	0.243339825	-0.134677722	0.000200123	-0.408802559
ENSG00000166949	SMAD3	9.081806	8.324193	8.027273	8.387335	8.157706	8.156874	6.347076	5.480303	6.246176	0.23044216	0.106829826	0.00000312	-0.408658154
ENSG00000162086	ZNF75A	13.008771	13.991064	14.086717	14.3608	12.610759	13.919444	9.776807	8.26672	11.250323	0.219645216	0.129482837	0.000174139	-0.408386558
ENSG00000007968	E2F2	1.196628	1.368359	1.31836	1.294472	1.02375	1.305386	0.917366	0.847189	0.974512	0.837909154	0.050085052	0.020501683	-0.408085802
ENSG00000137574	TGS1	5.571048	6.141317	6.026704	6.138998	6.056985	5.592187	4.181774	3.520009	4.787364	0.174162401	0.151126898	0.000376459	-0.408081347
ENSG00000177200	CHD9	1.019575	1.396923	1.228415	1.219865	1.230136	1.323153	0.799576	0.725192	0.899426	0.004489215	0.359936	0.003957194	-0.408039841
ENSG00000151332	MBIP	15.238801	15.115478	14.977445	14.071601	12.475504	13.403375	10.386978	10.534535	11.660736	0.764010428	-0.039908078	0.0000406	-0.407976346
ENSG00000163328	GPR155	0.245792	0.195325	0.156138	0.181319	0.22041	0.108795	0.100051	0.131954	0.180466	0.859832006	-0.09745546	0.33028246	-0.407976036
ENSG00000158636	EMSY	3.46905	3.191224	4.334154	3.896543	3.40475	3.282895	2.971579	2.612849	2.82007	0.868547933	0.030751726	0.003119976	-0.407366453
ENSG00000121542	SEC22A	2.77876	4.177757	4.245593	4.002897	3.037785	5.004545	4.750176	2.917156	2.608823	0.973926493	-0.007386565	0.008486811	-0.40717473
ENSG00000111671	SPSB2	7.029254	7.023261	6.224722	5.869434	5.860986	4.93408	4.366588	4.902862	5.442	0.482231511	-0.134903336	0.012963979	-0.407042549
NewGene_6362	--	0.738032	0.540479	0.437339	0.432926	0.396542	0.694222	0.686364	0.314014	0.210875	0.979852235	-0.017653639	0.460369732	-0.407022475
ENSG00000134215	VAV3	6.809528	6.196852	5.996222	5.948974	6.131196	5.229138	4.742856	4.590114	4.422952	0.663582891	-0.060731625	0.000201412	-0.406471931
ENSG00000180113	TDRD6	0.267159	0.221459	0.15482	0.252129	0.133253	0.182102	0.204831	0.133931	0.160433	0.939959435	-0.040412672	0.327080729	-0.405998039
ENSG00000152104	PTPN14	2.125574	2.15006	2.168416	1.410007	1.315458	1.333523	1.613817	1.343535	1.610086	2.78E-09	-0.520119238	0.0000403	-0.405836221
ENSG00000258818	RNASE4	5.881636	4.936622	4.775186	10.310225	12.93912	10.286154	4.082747	3.232424	3.387905	8.08E-25	1.237785108	0.00798841	-0.405671671
ENSG00000116199	FAM20B	20.035206	20.87948	20.516947	17.417892	17.225122	17.370279	14.795985	13.411322	15.322171	0.14324182	-0.092462324	2.23E-10	-0.405663333
ENSG00000146243	IRAK1BP1	0.741521	0.926398	0.588231	0.427515	0.312932	0.50378	0.650619	0.515516	0.728583	0.006161121	-0.774109367	0.114606758	-0.405443515
ENSG00000130479	MAP1S	7.281682	6.122364	5.715979	5.471246	5.563706	5.436713	4.561357	3.871659	5.11493	0.629307326	-0.073394671	0.001830351	-0.405432874
ENSG00000165102	HGSNAT	8.151872	7.960843	6.941849	6.729687	5.925521	6.363379	4.83164	5.215107	6.224721	0.152734409	-0.151193644	0.000162604	-0.405342077
ENSG00000204946	ZNF783	3.361497	3.557612	3.538379	3.730261	2.46663	3.481794	2.498299	2.354789	2.405832	0.851193139	0.041010206	0.002516803	-0.405264264
NewGene_1635	--	0.282586	0.729745	0.972616	0.934456	0.680774	0.698663	0.481079	0.237785	0.703051	0.531247802	0.359969411	0.50539206	-0.40507328
ENSG00000243896	OR2A7	0.783043	1.298892	0.93037	1.243949	1.257684	0.962204	0.544228	0.915107	0.740781	0.078256067	0.546829808	0.255715517	-0.404958046
ENSG00000164169	PRMT9	5.39824	5.461584	4.79921	4.317347	3.945677	4.112681	3.951089	3.691466	3.907641	0.114710002	-0.191805214	0.000477732	-0.404544389
ENSG00000175782	SLC35E3	1.38727	1.458089	1.479707	0.986493	0.948572	1.083027	1.035845	0.94294	1.076491	0.000169	-0.380627599	0.0000398	-0.404016894
ENSG00000180008	SOCS4	4.559093	5.358772	5.697019	4.715873	4.196826	4.961632	3.658536	3.048599	4.146847	0.876744628	-0.023258454	0.000191439	-0.404010625
ENSG00000058668	ATP2B4	0.399189	0.621717	0.337672	0.381917	0.384592	0.288174	0.241797	0.254576	0.375154	0.580006492	-0.171078287	0.137361868	-0.403865969
ENSG00000139163	ETNK1	14.25699	16.825418	18.317672	11.792314	10.829742	10.35716	11.630861	11.081379	13.587426	0.000000285	-0.436872062	0.0000202	-0.403797391
ENSG00000182446	NPLOC4	53.586365	52.382819	50.348037	44.990889	44.658222	47.036924	35.426219	34.658411	39.933261	0.569786779	-0.040804819	7.14E-11	-0.403787483
ENSG00000197019	SERTAD1	1.177451	1.003608	1.164488	0.64211	1.063274	0.905956	0.740805	0.935932	0.692081	0.569786779	-0.215888301	0.195695497	-0.403752851
ENSG00000198740	ZNF652	3.691309	4.414434	4.444134	4.737786	4.609185	4.519174	3.212612	2.741404	2.823499	0.005034776	0.304347447	0.001139993	-0.403672467
ENSG00000139800	ZIC5	2.263283	2.949552	2.995493	2.516044	2.204847	2.304058	1.755247	2.083776	1.964576	0.695336229	-0.075285697	0.009958831	-0.403613602
ENSG00000005483	KMT2E	10.077638	10.844239	10.633392	12.622108	12.06417	11.203847	8.872858	6.931655	8.488098	9.42E-08	0.349202625	0.00000232	-0.403479324
ENSG00000198689	SLC9A6	2.113968	2.359717	1.915106	2.440096	2.238814	1.969639	1.589679	1.327776	1.580808	0.168648294	0.219081908	0.008458722	-0.403237313
ENSG00000168412	CASP8AP2	8.673194	9.537597	9.28191	9.501006	9.309111	8.446546	6.593432	6.141717	6.719631	0.09757559	0.141269817	0.000000296	-0.403062026
ENSG00000165507	DEPP1	4.911896	3.73417	2.731137	4.027433	4.58401	4.30641	2.279808	2.739097	3.125291	0.111521124	0.333946829	0.06677628	-0.402962367
ENSG00000021645	NRXN3	15.79669	19.313985	24.576829	23.123555	17.750622	20.097113	12.188199	12.459928	14.029735	0.0196868	0.241139953	0.0000612	-0.402956745
ENSG00000148158	SNX30	7.979683	7.926071	7.05159	6.767361	6.836547	6.742211	5.612544	5.038803	5.646704	0.78452059	-0.029466793	0.000000902	-0.40291967
ENSG00000166260	COX11	32.718043	39.53334	42.324065	29.211653	27.903865	32.983184	26.773083	28.080634	27.347493	0.096917041	-0.119931105	6.25E-09	-0.402637744
ENSG00000213965	NUDT19	2.838679	3.084466	3.475769	3.653698	3.465977	3.529407	2.939386	1.694155	2.043043	0.022605944	0.327550451	0.047341574	-0.402189012
ENSG00000164465	DCBLD1	7.641051	6.953827	8.061242	6.770036	6.088353	5.995934	5.249777	5.174724	6.062466	0.329599464	-0.113419119	0.000165607	-0.402155595
ENSG00000134909	ARHGAP32	2.446994	1.944459	2.436793	1.697594	1.168036	1.491853	1.471529	1.402268	2.208016	5.97E-10	-0.713363894	0.018807212	-0.402025871
ENSG00000258839	MC1R	0.782196	0.880188	0.798557	0.798068	0.794649	0.566882	0.507128	0.57195	0.682578	0.837140511	-0.077376941	0.152579239	-0.401818304
ENSG00000167842	MIS12	13.175639	14.721559	13.869745	15.721993	16.615389	14.100607	8.950598	9.69539	9.50991	0.0000971	0.336002814	0.0000199	-0.401467747
ENSG00000085552	IGSF9	1.55184	1.55481	1.248143	1.570502	1.262248	1.409188	1.037652	0.93905	1.0526	0.744483248	0.083232068	0.039480752	-0.401403862
ENSG00000148840	PPRC1	13.323073	13.43253	17.584516	12.657118	10.490019	12.357053	11.42577	8.573613	10.537907	0.426973896	-0.074008927	0.000000887	-0.401383897
ENSG00000215301	DDX3X	89.404706	95.11033	77.529545	84.65211	64.022266	85.083264	60.126262	64.13226	69.42946	0.717885906	-0.024108916	3.27E-10	-0.401146946
ENSG00000155545	MIER3	6.011059	6.79414	6.535664	6.50353	6.162084	6.66151	4.9715	4.212924	4.429024	0.103006105	0.155591883	0.0000876	-0.401050682
ENSG00000158411	MITD1	19.037043	17.938339	20.21982	23.931912	24.077527	23.361163	12.838912	14.67807	15.144712	0.00000241	0.444046258	0.000497668	-0.400889494
NewGene_1019	--	3.859181	3.644488	3.222241	3.319684	3.214639	2.77481	2.622588	2.48567	2.511225	0.796506627	-0.059719392	0.019639672	-0.400698783
ENSG00000115464	USP34	19.03806	19.784526	19.700058	15.85052	15.189364	14.13387	13.969377	13.068842	14.936254	0.000243411	-0.212560821	1.71E-12	-0.400662157
ENSG00000104205	SGK3	3.134777	3.720757	3.393447	4.484842	4.168018	4.102872	2.794262	2.060435	2.58846	0.0000565	0.44043333	0.003097109	-0.400521775
NewGene_3707	--	4.443702	3.742998	3.781641	3.945231	4.026104	3.740957	2.582177	2.633257	3.306221	0.430390098	0.114686488	0.003516324	-0.400217322
ENSG00000164125	FAM198B	1.640615	1.434056	1.836733	1.161559	1.004756	1.011628	1.184623	1.141634	1.17299	0.005136687	-0.484751955	0.017884439	-0.400005656
NewGene_6609	--	1.64918	1.46901	1.377598	1.704994	1.027953	0.939938	1.23957	0.642085	1.238407	0.972469547	0.011161763	0.058935011	-0.399894159
ENSG00000177565	TBL1XR1	24.725451	29.303588	30.375604	28.641786	31.539227	27.793512	22.415494	18.763176	21.721921	0.052054962	0.148852985	0.00000064	-0.399805765
NewGene_6509	--	3.065045	3.403873	2.15118	2.333223	2.052755	2.324327	1.466482	2.363851	2.290048	0.628370944	-0.201701009	0.279305275	-0.399554798
ENSG00000280165	PCDH20	1.367558	1.561919	1.416361	0.887518	1.083305	1.120089	1.136843	0.88951	1.066414	0.077519674	-0.338937376	0.026004046	-0.399529182
ENSG00000111860	CEP85L	0.362217	0.511576	0.348374	0.322283	0.265721	0.191958	0.296941	0.268915	0.236911	0.261527328	-0.356251146	0.147014155	-0.398900784
ENSG00000167291	TBC1D16	8.558773	7.447294	6.922821	6.810056	6.19748	6.77201	5.602058	5.009876	6.216676	0.46069016	-0.072583079	0.00000167	-0.39880827
ENSG00000126882	FAM78A	0.300622	0.383526	0.287137	0.176211	0.167685	0.163826	0.157365	0.309432	0.1948	0.100124289	-0.724879373	0.383268832	-0.398539456
ENSG00000241935	HOGA1	1.907792	1.961567	1.841378	1.202342	1.775481	1.688547	1.426333	1.301623	1.131257	0.645687853	-0.143320977	0.095272541	-0.398225495

Gene ID	Gene name	Ctrl-1 FPKM	Ctrl-2 FPKM	Ctrl-3 FPKM	IFNγ-1 FPKM	IFNγ-2 FPKM	IFNγ-3 FPKM	Sora-1 FPKM	Sora-2 FPKM	Sora-3 FPKM	Ctrl vs IFNγ DESeq2 FDR	Ctrl vs IFNγ DESeq2 log2FC	Ctrl vs Sora DESeq2 FDR	Ctrl vs Sora DESeq2 log2FC
ENSG00000136237	RAPGEF5	6.551325	7.043323	7.557441	4.553764	4.323878	4.553014	5.573123	4.703418	5.313768	2.79E-09	-0.494165915	0.0000219	-0.398037448
ENSG00000078114	NEBL	0.929778	0.951862	1.116332	1.82722	1.834113	1.458044	0.592579	0.630219	0.661365	8.25E-17	1.120727271	0.041081535	-0.398028893
ENSG00000188647	PTAR1	6.487132	7.363041	7.969405	6.509131	7.16127	6.632597	5.382648	5.035624	5.480906	0.401525778	0.095221849	0.0000225	-0.39775139
ENSG00000166394	CYB5R2	4.131181	3.31278	3.566795	3.302228	2.742238	3.181849	2.805454	2.51828	2.49457	0.684004718	-0.110787867	0.068531414	-0.397668582
ENSG00000119541	VPS4B	9.487336	10.044074	10.12798	9.435264	9.619906	9.967468	7.004429	6.848488	7.271286	0.205565276	0.12094916	0.00000743	-0.397581783
ENSG00000233198	RNF224	1.848652	2.408155	2.09568	2.736018	1.83574	2.291163	1.64308	1.384916	1.489414	0.322812122	0.258560991	0.100288848	-0.39689507
ENSG00000101639	CEP192	6.3124	6.429277	6.136428	4.973008	4.789692	4.713141	4.379318	4.04529	5.193895	0.00348094	-0.226584301	0.00000405	-0.396877892
ENSG00000173281	PPP1R3B	9.012741	13.53688	9.564709	10.101025	10.697689	8.348686	9.231443	7.086613	7.609637	0.983796386	0.002430762	0.473390948	-0.396698073
ENSG00000072042	RDH11	57.24095	54.35611	49.222323	54.004414	58.04852	53.828984	37.924181	39.624186	35.318002	0.004235575	0.192516116	0.000000511	-0.396629297
ENSG00000114378	HYAL1	6.663017	6.535669	5.670603	6.032932	5.123492	5.508054	4.650069	4.280719	4.843009	0.973662659	0.006855018	0.002485409	-0.396609917
ENSG00000071539	TRIP13	16.757431	17.929167	19.8981	12.021734	10.140132	10.865815	12.952556	12.566255	13.383828	3.41E-11	-0.577660075	0.00000701	-0.395581829
ENSG00000109670	FBXW7	4.161934	4.492627	5.193227	6.779842	6.02423	5.913367	3.514494	2.950494	3.031793	5.46E-09	0.573303139	0.000846499	-0.395143897
ENSG00000131446	MGAT1	26.935564	32.747916	25.051933	33.694513	33.526191	27.699445	19.585747	23.227578	19.007316	0.00006	0.286559291	0.000000311	-0.394957354
ENSG00000131873	CHSY1	4.390184	4.174844	5.20261	6.253583	5.805352	5.338628	3.133883	3.203883	3.460787	0.00000962	0.459955101	0.000791842	-0.394912541
NewGene 173	--	1.535124	1.500507	0.932462	0.745596	0.716995	0.920726	0.837768	0.774097	0.653132	0.007830237	-0.727235896	0.149206811	-0.394792065
ENSG00000176024	ZNF613	1.42878	1.362773	1.864279	1.378883	1.272741	1.634397	1.414914	1.023926	0.851345	0.586946295	-0.136797464	0.098655451	-0.394588975
ENSG00000186272	ZNF17	2.146369	1.751255	1.957347	1.662214	1.746309	1.527502	1.526675	1.455197	1.314834	0.727472409	-0.090738698	0.054905521	-0.394559661
ENSG00000092148	HECTD1	36.967421	37.316944	35.394449	43.534662	38.957326	39.673275	26.063111	26.070591	26.507404	0.000110598	0.214440203	1.55E-11	-0.39455223
ENSG00000172765	TMCC1	4.123315	3.767272	3.774483	3.221427	2.767177	3.098249	2.623249	2.704104	3.247692	0.035789881	-0.224007535	0.000107001	-0.394498754
NewGene 233	--	0.813557	0.70506	0.623504	0.791007	1.044994	0.687242	0.764967	0.622491	0.45478	0.367127877	-0.245571659	0.108271865	-0.394467956
ENSG00000205423	CNEP1R1	2.846434	3.128947	3.259914	3.239299	3.048706	2.762093	3.461725	2.211208	1.92475	0.255642296	0.214862088	0.065399873	-0.394210073
ENSG00000121769	FABP3	2.008743	2.050596	1.460646	1.681714	1.503761	1.133164	0.98977	1.768976	1.124897	0.686674823	-0.201980512	0.369204762	-0.394088527
ENSG00000152977	ZIC1	12.599502	13.633717	12.063464	10.562841	11.885147	11.212601	9.202711	9.20443	10.674424	0.891402334	0.016060724	0.0000044	-0.393974455
ENSG00000085274	MYNN	14.509205	13.632994	13.495984	12.719534	12.207418	12.033996	10.548305	10.170945	10.777131	0.735321503	0.031965097	9.26E-08	-0.393604581
ENSG00000213064	SFT2D2	13.099793	13.895168	13.607768	13.108211	12.220075	12.43518	9.929561	8.989038	10.040255	0.529910005	0.044949988	3.34E-10	-0.393379829
ENSG00000132466	ANKRD17	17.048855	16.770968	17.414316	18.019453	17.747588	17.012223	12.244654	11.193312	13.121143	0.000483671	0.189799537	2.98E-10	-0.393230394
ENSG00000171466	ZNF562	5.181603	5.311056	5.194217	4.411461	3.857496	3.976254	4.107943	4.509594	4.448704	0.003082214	-0.239198114	0.00000164	-0.39257477
ENSG00000116793	PHTF1	3.215789	3.79241	3.859399	3.190357	3.098963	3.259082	2.831452	2.471827	2.469353	0.546116178	-0.108425407	0.012455395	-0.392510682
ENSG00000198492	YTHDF2	50.086806	48.414484	48.291113	44.005494	43.992838	44.506886	35.234831	32.097716	37.887731	0.963346202	0.004330114	1.1E-09	-0.392439649
ENSG00000126775	ATG14	6.458422	6.446423	6.175566	6.499468	6.058761	5.988508	4.598185	4.453918	4.573434	0.285630401	0.105383458	0.0000155	-0.392385914
ENSG00000151131	C12orf45	0.45791	0.598163	0.555176	0.505616	0.534419	0.401947	0.373111	0.411666	0.297683	0.441853303	-0.146767555	0.031479914	-0.392097805
NewGene 1455	--	0.890754	0.69865	0.770775	0.683622	0.538924	0.519281	0.506363	0.511008	0.657318	0.570948405	-0.292928435	0.377807859	-0.392091967
ENSG00000260342	AC138811.2	5.368135	1.779003	0.621807	9.016745	1.31936	2.620081	1.917119	0.688869	3.043941	0.576972113	0.869742295	0.760696683	-0.391986773
ENSG00000092439	TRPM7	12.214983	13.171733	12.373589	10.391675	11.184445	9.913929	8.178342	8.29745	9.85283	0.304999853	-0.073044528	3.62E-08	-0.391974064
ENSG00000162105	SHANK2	4.549988	4.770159	5.409659	4.509097	4.046022	3.550928	4.158287	3.282974	3.770065	0.202128133	-0.146006715	0.000148014	-0.391914295
ENSG00000158987	RAPGEF6	4.516117	5.697456	5.260691	3.430087	3.327964	3.527695	3.945717	3.274352	3.74398	0.00000664	-0.401057794	0.0000702	-0.391853752
ENSG00000159445	THEM4	9.147964	9.024987	9.304503	10.249575	7.635592	8.993731	6.34997	6.429444	6.795797	0.31078515	0.130151651	0.000227266	-0.391503179
ENSG00000101966	XIAP	3.981965	4.491641	4.109385	5.326121	4.758915	5.379601	2.966319	2.78785	3.376947	0.000000104	0.430547263	0.0000448	-0.391256885
ENSG00000076321	KLHL20	4.936028	4.893403	5.267725	5.872936	5.627516	5.879396	3.474703	3.509687	3.810961	0.000473598	0.350590519	0.000616399	-0.390963383
ENSG00000140006	WDR89	4.154847	5.412695	5.123207	4.338831	4.182138	5.438697	3.765663	3.299452	3.555274	0.718439848	0.071117276	0.010777408	-0.39088308
ENSG00000173681	BCLAF3	0.723929	1.029323	1.262665	1.193257	0.81866	0.980009	0.829578	0.82714	0.701991	0.552909521	0.154995625	0.084015258	-0.39003669
ENSG00000159176	CSRP1	18.628109	20.713534	22.160591	13.682474	13.503752	13.396986	13.745589	13.844727	16.482706	0.000000178	-0.452040507	0.0000778	-0.389915238
ENSG00000186001	LRCH3	10.176722	8.888283	7.918855	9.348768	8.636254	8.178452	6.580754	6.324106	6.602465	0.245281347	0.111832668	0.00000603	-0.389830478
ENSG00000198732	SMOC1	23.069694	17.517329	17.796262	12.599313	15.681171	13.483217	12.364365	14.689287	14.976489	0.135280474	-0.344580003	0.000979577	-0.389646275
ENSG00000100330	MTMR3	5.042844	5.122856	5.068771	5.106227	4.755782	4.350427	3.873718	3.584693	3.848253	0.288015339	0.0999099	0.0000113	-0.389323571
ENSG00000168792	ABHD15	2.735404	2.55252	2.14427	2.386218	2.465863	2.416446	1.710368	1.699688	1.915999	0.546624049	0.114350783	0.017449808	-0.388883496
ENSG00000142166	IFNAR1	12.686554	12.981905	14.03636	13.450084	12.406257	12.02838	9.268397	8.662458	10.557513	0.345349362	0.079182484	0.000000863	-0.38871342
ENSG00000110400	NECTIN1	1.683449	1.464955	1.412604	1.936791	1.663836	2.009178	1.295458	1.036066	0.989512	0.003492508	0.419915614	0.02563818	-0.388533978
ENSG00000156381	ANKRD9	3.808092	3.305748	3.503315	3.023698	2.764719	3.280324	2.755204	2.542253	2.88586	0.070850145	-0.233577694	0.002637411	-0.388408289
ENSG00000272325	NUDT3	18.163181	19.236095	18.817444	17.488678	15.462649	16.6525	13.467126	12.654531	14.184308	0.673618271	-0.033661734	6.08E-11	-0.388393721
ENSG00000136052	SLC41A2	1.148985	0.734589	0.75007	0.648369	0.534029	0.739675	0.590443	0.425695	0.756598	0.499360213	-0.218042158	0.197525779	-0.388184802
ENSG00000147679	UTP23	3.809163	5.383172	5.202458	5.167532	4.467419	4.629224	3.348003	3.447965	3.589053	0.451357381	0.123166319	0.006412754	-0.387679151
ENSG00000243477	NAA80	1.744186	2.165604	2.398219	2.260662	2.479954	2.06014	1.435347	1.4921	1.429083	0.219482151	0.319080732	0.134033487	-0.387548305
ENSG00000110756	HPS5	8.614657	8.918541	7.925721	7.400063	7.154974	6.94191	6.429763	5.59217	6.482396	0.252712114	-0.113630114	0.0000195	-0.387365133
NewGene 4840	--	2.315205	1.854786	1.939538	2.071565	1.436365	1.74459	1.505317	1.771398	1.416787	0.996043158	-0.003010421	0.330704691	-0.387246607
NewGene 1821	--	1.145508	1.442814	1.872843	1.298779	1.265034	1.379821	0.994945	1.026939	1.013417	0.947982103	-0.030894731	0.257582015	-0.387192497
ENSG00000108175	ZMIZ1	10.890785	10.078001	11.060512	9.170378	8.47287	8.456216	7.58753	6.973729	8.453468	0.044819252	-0.148932143	0.000000647	-0.386989331
ENSG00000176595	KBTBD11	0.709351	0.768912	0.776725	0.507552	0.503428	0.706215	0.567563	0.492948	0.55911	0.336879028	-0.23761958	0.054949031	-0.386966263
ENSG00000165055	METTL2B	9.187531	9.806406	11.158997	8.569822	7.969484	9.162597	7.445887	6.92618	8.842321	0.307670948	-0.102142148	0.0000289	-0.386929005
ENSG00000198039	ZNF273	1.597721	1.993179	2.581894	1.530956	1.738325	1.422524	1.606344	1.519961	1.684039	0.173259818	-0.304387453	0.055022847	-0.386629351
ENSG00000141642	ELAC1	1.685994	1.42179	1.548846	1.400537	1.048309	1.350548	0.915676	1.098372	1.289469	0.483463255	-0.208010231	0.148509961	-0.386617522
ENSG00000187676	B3GLCT	4.234294	4.417354	4.324748	2.953625	3.380188	3.011835	3.18865	2.963046	3.156342	0.004689947	-0.325941325	0.000406493	-0.386420424
ENSG00000169951	ZNF764	9.264635	10.435117	11.244199	10.052864	10.613414	11.357308	8.326794	6.33617	8.016736	0.063852885	0.199502377	0.00079904	-0.385618843
ENSG00000011007	ELOA	20.572683	15.650812	20.72196	24.183715	22.184948	19.812445	12.190786	14.562641	11.17531	0.000125435	0.239563127	3.48E-08	-0.38546914
ENSG00000204120	GIGYF2	18.605138	16.580645	16.035751	16.946173	16.408173	17.000499	11.826395	11.153095	11.575721	0.000358548	0.211426167	5.17E-09	-0.385336076
ENSG00000118292	C1orf54	1.393624	1.361881	0.703862	0.899033	0.836263	0.887034	0.566338	0.757044	1.156418	0.617386784	-0.24941151	0.399702416	-0.385213263
NewGene 220	--	0.621567	0.881627	0.962168	0.617726	0.6793	0.64252	0.819063	0.53619	0.434142	0.722422701	-0.179605534	0.372777188	-0.385116121
ENSG00000068796	KIF2A	16.318555	16.443816	16.676672	25.506733	24.350119	23.092628	12.178547	12.045639	15.841893	3.44E-17	0.5613046	0.001328101	-0.385002292
ENSG00000179933	C14orf119	17.358811	16.206462	14.952823	12.539035	13.924273	12.755189	10.582892	12.057575	11.899494	0.082511713	-0.159616276	0.000018	-0.384921402
ENSG00000100241	SBF1	12.607924	12.304291	10.308417	12.502715	11.758328	13.286139	7.921536	7.598079	10.021341	0.006296251	0.221958292	0.0000326	-0.38457763
NewGene 3089	--	0.424654	0.421405	0.403345	0.44191	0.376242	0.412603	0.269135	0.204865	0.424068	0.733872352	0.147510412	0.343143227	-0.384345347
ENSG00000033800	PIAS1	10.78547	11.09006	11.626786	10.695618	10.625733	10.599865	8.346127	7.966151	8.547696	0.344972502	0.070045251	2.36E-08	-0.3842743
ENSG00000187713	TMEM203	29.430731	26.325638	24.431208	25.748802	25.682661	23.754307	18.163509	17.650967	21.936348	0.661802607	0.053011359	0.000163653	-0.384204446
NewGene 3318	--	0.867919	1.156337	0.899968	1.176573	1.246221	1.021751	0.63507	0.747974	0.710773	0.078515438	0.383042768	0.101934514	-0.383946772

Gene ID	Gene name	Ctrl-1 FPKM	Ctrl-2 FPKM	Ctrl-3 FPKM	IFNγ-1 FPKM	IFNγ-2 FPKM	IFNγ-3 FPKM	Sora-1 FPKM	Sora-2 FPKM	Sora-3 FPKM	Ctrl vs IFNγ DESeq2 FDR	Ctrl vs IFNγ DESeq2 log2FC	Ctrl vs Sora DESeq2 FDR	Ctrl vs Sora DESeq2 log2FC
ENSG00000101216	GMEB2	12.68427	12.424688	11.928002	10.520343	10.685042	10.82999	8.835189	7.666465	10.028501	0.509363993	-0.060665615	0.0000131	-0.383669583
ENSG00000153922	CHD1	10.174853	10.728415	11.619186	12.804334	11.786905	11.077239	8.278014	8.650975	9.188494	0.201736146	0.10351184	0.00000351	-0.383661154
ENSG00000172348	RCAN2	1.079586	0.975743	1.13786	1.858829	2.070101	2.090216	0.815235	0.761623	0.73031	1.03E-09	1.057600642	0.115271807	-0.383635283
ENSG00000039537	C6	1.420476	0.882135	0.9539	0.379345	0.458431	0.322225	0.750655	0.878243	0.683234	0.00000469	-1.281307441	0.160402202	-0.383554266
ENSG00000133065	SLC41A1	0.909962	0.917345	0.908455	0.694108	0.643885	0.666434	0.574528	0.644611	0.743696	0.187301258	-0.296919618	0.075537641	-0.383509492
ENSG00000082196	C1QTNF3	1.584529	1.713489	1.098641	1.134431	1.679955	1.2033	1.258954	0.777268	1.143839	0.973662659	0.01357417	0.158438422	-0.383419691
ENSG00000131848	ZSCAN5A	9.523313	8.270841	7.75214	5.839015	5.903533	5.95669	4.483479	5.056697	5.879307	0.029635235	-0.273598109	0.00286025	-0.383418248
ENSG00000125482	TTF1	7.089503	7.061701	7.447858	6.119177	6.12801	5.672574	5.343234	4.951225	5.22358	0.37978131	-0.106002139	0.000219227	-0.383158043
ENSG00000136866	ZFP37	0.783301	1.84893	1.204197	1.047941	0.951134	0.971283	0.948198	0.724616	1.058667	0.814355441	-0.06614297	0.074438393	-0.383143162
ENSG00000042062	RIPOR3	0.469505	0.349183	0.335215	0.076087	0.098351	0.159063	0.323485	0.255505	0.255778	0.000145216	-1.601950334	0.30218389	-0.383065882
ENSG00000125149	C16orf70	7.503574	8.03931	6.732818	8.101842	8.042642	7.469318	5.410632	5.343617	4.93364	0.02622649	0.232481014	0.000690757	-0.383016458
ENSG00000007168	PAFAH1B1	24.203379	26.608284	27.160328	23.61125	22.853085	23.188334	18.852356	18.120155	18.784078	0.927169578	-0.008949103	1.79E-08	-0.38293709
ENSG00000206538	VGLL3	0.556983	0.787041	0.937475	0.098189	0.109443	0.165691	0.613313	0.419649	0.623078	1.53E-23	-2.426774073	0.091269433	-0.38284382
ENSG00000158483	FAM86C1	4.559963	4.229097	4.52598	4.167207	3.183346	3.803682	3.092201	3.418325	3.046841	0.488433567	-0.141289005	0.026110714	-0.382153879
ENSG00000062598	ELMO2	6.212507	6.048516	6.149292	6.154374	5.215732	5.837348	4.441431	4.017615	4.590161	0.760876593	0.041233027	0.000187037	-0.382139359
ENSG00000163521	GLB1L	1.264553	1.27796	1.612631	1.152724	0.710975	0.858982	1.147727	0.890744	0.934604	0.100812281	-0.45049635	0.135135462	-0.382088992
ENSG00000140526	ABHD2	15.579015	15.359561	15.075643	15.973203	17.280794	17.025308	10.750317	10.61523	11.678249	0.00000321	0.277743859	3.52E-10	-0.381932339
NewGene_6521	--	5.387556	4.459245	5.345649	4.326596	5.213302	5.534268	3.303071	3.307277	3.283697	0.609875374	0.102741894	0.011327081	-0.381860074
ENSG00000240038	AMY2B	1.680682	1.630924	2.032673	2.374734	2.514071	1.947878	1.301644	1.114109	1.43745	0.023788524	0.54455871	0.166025465	-0.381768354
ENSG00000185115	NSMCE3	2.00291	2.428657	2.198404	2.329638	1.847363	2.200387	1.681981	1.441422	1.647206	0.622107915	0.09208373	0.010221786	-0.381755304
ENSG00000161547	SRSF2	173.290141	174.062472	185.483582	160.41702	159.08555	174.015316	125.130823	118.738543	132.081296	0.198032882	0.081838059	1.32E-09	-0.381605334
ENSG00000156853	ZNF689	8.623484	8.928073	8.411958	7.531075	8.295997	8.088116	6.141794	5.818956	6.751135	0.813360511	0.02996844	0.0000321	-0.381462771
ENSG00000148180	GSN	8.538579	6.828825	5.655247	3.889547	4.795184	4.027166	4.597113	5.131521	5.280567	0.000124567	-0.590325203	0.013296929	-0.3813173
ENSG00000130517	PGPEP1	21.408307	21.111601	18.658118	18.299563	18.896406	18.651661	13.896366	14.362114	15.897808	0.893408733	0.013454182	8.77E-08	-0.381207867
ENSG00000117000	RLF	4.826305	5.21861	5.115997	4.538544	4.723739	4.758232	3.905996	3.530861	3.466746	0.76698146	0.035592054	0.0000962	-0.38092346
ENSG00000118620	ZNF430	1.919763	2.409839	2.637511	2.773697	2.239247	2.208972	1.704945	1.620137	1.883423	0.300381626	0.194396074	0.026164296	-0.380896674
NewGene_5247	--	1.916828	1.834114	1.520174	1.550427	2.081699	1.550475	1.210806	1.536845	1.42711	0.784554098	0.092918861	0.161517731	-0.380861038
ENSG00000104177	MYEF2	13.563373	14.579779	15.357527	7.953934	10.834894	10.329202	13.466106	10.163223	11.625699	0.0000331	-0.347216615	0.0000363	-0.380504089
ENSG00000198231	DDX42	52.808815	54.960737	52.61084	50.674333	48.072516	49.007389	39.807632	38.796826	41.496046	0.688630564	0.03005239	7.26E-11	-0.380404606
ENSG00000224916	APOC4-APOC2	1.225565	3.16382	0.682056	0.361537	0.045493	1.023859	1.051678	1.061518	1.549451	0.254543175	-1.655427414	0.646306073	-0.38037138
ENSG00000198162	MAN1A2	6.010167	6.758468	6.651018	6.278893	6.045298	5.969399	4.63476	4.095528	5.304556	0.576226822	0.053503814	0.000033	-0.380259243
ENSG00000245680	ZNF585B	1.679925	1.832243	1.690456	1.824635	1.504402	1.436059	1.252882	1.122177	1.44575	0.405106736	-0.15074409	0.014114222	-0.380238668
ENSG00000131089	ARHGEF9	1.690034	1.48114	1.606506	2.846251	1.710112	1.473997	1.02499	1.09573	1.87159	0.015027787	0.392737467	0.026397794	-0.380216907
ENSG00000198887	SMC5	8.14747	9.009819	10.428449	7.935272	7.668506	7.495177	6.467289	6.380173	7.03433	0.318350235	-0.108595699	0.0000908	-0.38018154
ENSG00000204315	FKBPL	10.638758	9.408854	9.138824	7.946539	8.533308	8.117346	6.920628	7.661441	6.398296	0.536993218	-0.100039755	0.009075317	-0.379885955
ENSG00000070614	NDST1	27.91852	24.57713	24.865733	24.430449	23.550984	25.538778	18.262542	16.394768	21.536034	0.458810874	0.063676127	0.0000206	-0.379806893
ENSG00000004897	CDC27	20.667029	22.675194	22.180418	21.926272	20.333456	21.021149	17.328898	14.661545	17.047133	0.282391897	0.075374507	0.000000112	-0.379631841
ENSG00000141569	TRIM65	6.065637	6.821593	7.055706	6.117502	6.180156	6.598038	4.760056	4.214422	5.491633	0.634668067	0.064261669	0.001077308	-0.37957271
ENSG00000115170	ACVR1	25.359845	23.109749	24.903821	21.755189	20.153264	19.06331	16.965835	16.62714	18.505905	0.371430736	-0.076744877	0.000000265	-0.378987108
ENSG00000100852	ARHGAP5	8.233378	8.292443	8.943369	11.053064	10.127089	12.043705	6.36565	6.203695	6.445586	1.67E-11	0.421903211	0.000000672	-0.378803552
ENSG00000172731	LRRC20	4.745924	5.158308	3.610164	3.327274	3.337065	3.3256	3.254923	3.177103	3.144049	0.067368849	-0.300428583	0.016596841	-0.378541357
ENSG00000221843	C2orf16	1.728172	1.075316	1.615737	1.021188	0.873218	0.720886	0.76143	0.802382	0.850414	0.00000932	-0.546207641	0.002179826	-0.378318765
ENSG00000178229	ZNF543	1.769868	1.694526	1.744854	1.790652	1.622252	1.667897	1.455765	1.048898	1.258893	0.590091577	0.112000578	0.047643217	-0.378158199
ENSG00000174607	UGT8	2.415541	2.332153	3.024339	2.420618	2.385243	2.366926	1.640836	1.956904	2.199716	0.906689795	0.025685134	0.024379704	-0.378071845
ENSG00000174292	TNK1	2.322521	2.768043	2.686629	2.444201	2.352154	2.501699	1.834921	1.830104	1.900012	0.676948623	0.085326719	0.023362014	-0.378027326
ENSG00000181904	C5orf24	8.382698	10.487913	11.216528	7.868732	9.653868	7.635826	7.135084	6.64707	6.602536	0.373148853	-0.095317492	0.000046	-0.37799938
ENSG00000181619	GPR135	0.199878	0.28253	0.270499	0.348064	0.25753	0.199854	0.10296	0.151165	0.28817	0.829085714	0.126133958	0.464082275	-0.377856736
ENSG00000077514	POLD3	4.738497	5.452572	4.951372	4.940027	4.337811	4.703909	3.838229	3.524127	3.373509	0.876919427	0.024896678	0.001879036	-0.377627758
ENSG00000012048	BRCA1	7.662526	7.519261	7.054747	6.788289	5.593283	6.319154	5.401357	5.267728	5.189523	0.244736231	-0.110387415	0.00000771	-0.377512779
ENSG00000115556	PLCD4	1.170283	1.217495	1.624678	1.263111	1.028619	1.124599	0.924626	0.88297	1.099734	0.783767223	-0.084000643	0.112006331	-0.377127871
ENSG00000197044	ZNF441	1.183461	0.810964	0.657638	0.898965	0.9697	0.938585	0.691465	0.562989	0.486111	0.135667833	0.360517093	0.167662389	-0.376922075
NewGene_6433	--	0.854468	1.534385	1.527209	1.000493	0.82007	0.815013	0.908887	0.884482	0.965811	0.227262571	-0.45065218	0.277290665	-0.376593323
ENSG00000148225	WDR31	0.295794	0.342382	0.314012	0.18297	0.164591	0.20033	0.231616	0.198212	0.247925	0.128305128	-0.614106611	0.323574249	-0.376435986
NewGene_3532	--	2.054979	2.110161	2.246161	2.103088	2.291324	1.561834	1.810668	1.393591	2.062254	0.989026049	-0.003477517	0.041000863	-0.375920029
ENSG00000085276	MECOM	5.929812	6.176231	7.37162	6.044589	6.117346	5.046054	4.682254	4.658481	4.703206	0.596524408	-0.07472776	0.000261051	-0.37584449
ENSG00000082258	CCNT2	17.213881	18.18799	17.440118	17.047788	17.600765	15.464428	14.139147	13.775172	13.896708	0.734426486	0.03293529	0.000000117	-0.375638112
ENSG00000214309	MBLAC1	1.214102	1.176457	0.962432	0.793082	0.893675	0.911468	0.818688	0.80072	0.840313	0.686766581	-0.178536077	0.305208943	-0.374790281
ENSG00000145414	NAF1	5.641128	5.699217	6.065677	5.219794	5.637068	5.881954	4.242787	3.986453	4.498974	0.598604664	0.08959034	0.00780199	-0.374685128
ENSG00000130119	GNL3L	4.242782	4.243185	4.382019	3.739869	3.948658	3.856597	3.122951	3.125917	3.03484	0.965591219	-0.009447641	0.010935001	-0.374684108
ENSG00000125775	SDCBP2	2.302079	1.385395	1.332847	1.216908	1.075853	1.270847	1.057097	0.987485	1.612606	0.338720204	-0.344665691	0.277333508	-0.37454026
ENSG00000102804	TSC22D1	9.924641	8.980385	8.188976	7.115647	8.055064	8.313825	6.698307	5.975829	6.91959	0.784458984	-0.042250493	0.000779595	-0.374416998
ENSG00000196867	ZFP28	2.948253	2.837373	3.233646	3.074751	3.480223	3.331236	2.364734	1.825825	2.408546	0.044945447	0.267753489	0.01076428	-0.374305056
ENSG00000213523	SRA1	7.728473	6.853068	7.939255	7.303983	7.416466	6.801037	6.018172	5.052804	5.729199	0.602260956	0.078370714	0.002829846	-0.374009003
ENSG00000178685	PARP10	0.956817	0.790218	0.629284	10.205297	8.609459	10.066892	0.858532	0.692816	0.712641	1.22E-184	3.917941427	0.238536787	-0.373801887
ENSG00000197386	HTT	14.49511	14.098061	12.60612	15.609453	14.296592	14.271057	9.984603	9.16012	11.153748	0.000356624	0.230868565	0.00000121	-0.373732934
ENSG00000134769	DTNA	13.075441	11.95384	11.935687	7.680605	5.805406	6.208297	8.934122	10.375648	8.960527	2.41E-21	-0.7376263	0.00000724	-0.373688988
ENSG00000043355	ZIC2	2.512956	2.285836	2.46648	1.869379	1.922048	1.766126	1.904067	1.566798	1.798117	0.192033701	-0.239356332	0.030490579	-0.373544647
NewGene_6071	--	4.192548	4.001102	3.617232	3.418833	2.594938	2.960288	2.921478	3.33411	2.950083	0.184793087	-0.184435463	0.003822243	-0.373536834
NewGene_4562	--	1.935421	1.795536	2.235592	3.820369	3.751236	3.823902	1.513076	1.3255	1.7102	6.98E-19	1.046077768	0.021817993	-0.373255352
ENSG00000198585	NUDT16	9.561623	8.916462	8.691825	11.573397	11.576145	11.721848	6.928681	6.416918	5.924689	8.54E-15	0.495380315	0.0000681	-0.373081277
ENSG00000224531	SMIM13	9.428288	11.057068	12.242915	9.406478	9.010708	9.284081	7.947209	7.18302	8.59433	0.422372218	-0.092701905	0.000279416	-0.373080293
ENSG00000119906	SLF2	5.876854	6.524385	6.874121	6.275797	5.759297	5.648841	4.359185	4.423737	4.861763	0.86382996	0.022061872	0.000045	-0.372870296
NewGene_2903	--	1.064166	0.824501	1.011812	0.861908	1.127488	1.118736	0.781185	0.657851	0.809137	0.52820967	0.173076343	0.118016321	-0.372715667
NewGene_1871	--	1.388928	1.052797	1.099214	1.365976	1.567159	1.272454	0.983716	0.809886	1.397874	0.051620153	0.298518551	0.021181242	-0.372549735

Gene ID	Gene name	Ctrl-1 FPKM	Ctrl-2 FPKM	Ctrl-3 FPKM	IFNγ-1 FPKM	IFNγ-2 FPKM	IFNγ-3 FPKM	Sora-1 FPKM	Sora-2 FPKM	Sora-3 FPKM	Ctrl vs IFNγ DESeq2 FDR	Ctrl vs IFNγ DESeq2 log2FC	Ctrl vs Sora DESeq2 FDR	Ctrl vs Sora DESeq2 log2FC
ENSG00000165699	TSC1	7.773292	10.074804	9.504811	7.991143	7.621213	9.57823	7.014537	6.811523	6.6296	0.771605796	0.031380828	0.0000146	-0.37226396
ENSG00000157184	CPT2	9.809657	8.774827	8.339582	8.730368	7.816505	8.00133	6.638222	6.409245	6.41298	0.962563224	0.007674082	0.000709066	-0.372158581
ENSG00000205208	C4orf46	7.574267	7.901282	7.972711	7.362844	6.671172	6.959304	5.754349	5.261777	5.987111	0.928251957	-0.012573845	0.0000824	-0.372154972
ENSG00000116815	CD58	5.565016	5.139796	6.450695	3.055757	2.069442	3.140762	3.233326	4.576157	4.727217	0.0000745	-0.867987106	0.076762463	-0.372126459
ENSG00000136205	TNS3	46.31546	46.243606	44.978658	45.276053	41.424932	42.166673	33.332745	30.313802	36.369158	0.510495438	0.04324791	1.94E-09	-0.37205454
ENSG00000124177	CHD6	6.97618	7.069609	7.021154	6.79808	6.607239	6.564421	5.608017	5.420679	5.707769	0.402164587	0.062949333	3.78E-08	-0.371952575
ENSG00000140948	ZCCHC14	5.91021	6.008451	5.914488	6.457562	5.792585	6.504482	4.815828	3.748516	4.461199	0.007430256	0.214617493	0.0000856	-0.371582749
ENSG00000049192	ADAMTS6	0.573499	0.895818	0.748898	0.962994	0.881942	0.764388	0.484376	0.532504	0.593692	0.076253101	0.378377451	0.100087381	-0.371274998
ENSG00000169118	CSNK1G1	2.91137	2.80868	2.998691	3.058239	2.877444	3.123273	2.611283	2.511372	2.169393	0.317692029	0.109290177	0.00148645	-0.370693769
ENSG00000166821	PEX11A	1.379887	1.726615	1.634256	1.996178	2.290965	2.256176	1.202288	1.268869	1.253565	0.020511971	0.460728916	0.097584065	-0.370498083
ENSG00000095951	HIVEP1	2.48237	2.408317	2.35099	3.663948	2.975886	2.978019	1.793473	1.545746	1.702683	1.89E-09	0.533437779	0.001070282	-0.370131507
ENSG00000074319	TSG101	60.789399	59.336346	58.353913	48.634623	51.307031	49.807745	42.554355	42.614876	44.356178	0.129056753	-0.105424981	5.85E-09	-0.370109896
ENSG00000186106	ANKRD46	8.890505	8.79441	9.22152	7.624284	7.459948	7.325158	7.303458	6.29395	6.684247	0.111798009	-0.165233308	0.000238008	-0.369967562
ENSG00000100373	UPK3A	2.502058	2.468044	2.351496	1.485375	1.50182	2.591607	1.787679	1.720485	1.774086	0.502167751	-0.249129463	0.19132532	-0.369731706
ENSG00000081692	JMJD4	18.113785	18.956853	16.611831	17.965942	15.650195	18.282212	12.48891	12.326099	13.890761	0.349199984	0.097777749	0.0000245	-0.369650903
ENSG00000114126	TFDP2	5.456779	5.706223	4.859602	4.356257	4.231571	4.399074	4.566594	3.701523	4.073496	0.090168488	-0.156804081	0.000193583	-0.369461974
ENSG00000120539	MASTL	5.651659	6.448728	6.674125	7.054898	6.615624	6.593617	4.631065	4.005196	4.801209	0.020303146	0.250154708	0.001453352	-0.369432811
NewGene_4412	--	5.170859	4.534467	5.275956	4.290116	4.236034	4.855048	3.312146	3.3131	4.319467	0.945252109	-0.016862087	0.034086788	-0.369271072
ENSG00000130338	TULP4	3.069737	3.125591	3.24987	2.812878	2.883869	2.775009	2.543468	2.025182	2.437021	0.939869928	-0.009620563	0.000104129	-0.369184828
ENSG00000127603	MACF1	12.765078	12.005037	12.642628	14.61431	11.372326	9.244804	8.088949	8.134774	9.036481	0.010285116	-0.142262601	1.91E-08	-0.369106472
ENSG00000170242	USP47	16.001474	15.854792	17.025134	18.583884	18.398074	15.83568	12.478459	11.635541	9.940756	0.003214483	0.190909922	2.92E-08	-0.36901889
ENSG00000177614	PGBD5	0.101961	0.122833	0.106345	0.048165	0.042641	0.05876	0.096623	0.064219	0.080272	0.028971549	-0.983592478	0.392056136	-0.368979549
ENSG00000185920	PTCH1	1.457962	1.058912	1.110657	0.833796	0.64577	0.664271	0.816123	0.931796	1.002619	0.000777568	-0.54628422	0.018206228	-0.368781504
ENSG00000169016	E2F6	11.559596	11.120117	12.363157	8.940998	10.436786	8.522198	8.409366	8.193389	8.587531	0.178718774	-0.127192087	0.0000307	-0.368622298
ENSG00000053747	LAMA3	0.727429	0.630284	0.60336	0.578275	0.553532	0.525586	0.475952	0.388804	0.503616	0.993484931	-0.002391118	0.041156363	-0.368542528
ENSG00000112964	GHR	6.009685	6.92227	7.553234	3.915086	3.681356	3.524435	4.954861	4.330197	5.483529	3.18E-13	-0.742986192	0.001131342	-0.368395216
ENSG00000164331	ANKRA2	3.526746	3.115314	3.245324	3.437145	3.321852	3.28264	2.274071	2.416576	2.490662	0.361542021	0.169636875	0.03639345	-0.368287363
ENSG00000213096	ZNF254	8.120807	9.383693	9.79736	8.475995	8.723075	7.75383	6.672381	6.678243	6.963488	0.890452401	0.019639165	0.000143736	-0.367924776
ENSG00000121741	ZMYM2	20.162363	18.032446	22.261225	12.804539	11.722762	11.72785	9.729743	13.627425	10.589023	0.861311725	0.017445576	0.00000266	-0.367748723
ENSG00000205213	LGR4	45.333071	46.826721	44.220622	44.219003	43.711862	43.124527	33.451535	30.9597	34.719779	0.114917181	0.090061684	2.78E-10	-0.367747336
ENSG00000136709	WDR33	18.366616	18.598696	18.664349	17.407251	16.407616	16.902874	15.749227	15.49992	16.009523	0.902378968	0.010382656	2.15E-09	-0.367711307
ENSG00000005448	WDR54	4.002083	4.385326	3.259215	2.689392	3.001378	3.080063	2.698071	2.934199	2.711491	0.173851905	-0.330171281	0.106375871	-0.367624014
NewGene_5143	--	3.056609	2.862897	3.093311	2.208986	2.434108	2.15678	2.219521	2.448175	2.308389	0.073189737	-0.302374597	0.023891575	-0.367382176
ENSG00000160209	PDXK	13.74005	14.799226	13.786216	15.689705	14.357753	15.17333	9.993263	9.521046	11.156469	0.0000207	0.258926231	0.000000153	-0.367274283
ENSG00000109743	BST1	0.50965	0.671756	0.773297	0.540377	0.523962	0.547755	0.687236	0.344172	0.385471	0.937334942	-0.045429379	0.443870768	-0.366697041
ENSG00000109118	PHF12	11.199048	11.138408	11.147357	11.738769	11.724609	10.923575	7.407604	6.850681	8.933521	0.015488037	0.182754498	0.00000955	-0.366649401
ENSG00000165997	ARL5B	20.576412	22.846998	23.825331	22.36536	21.833633	21.806293	16.04286	15.693428	17.191805	0.089468051	0.120644801	0.000000213	-0.366649271
ENSG00000221909	FAM200A	3.416989	4.510773	4.735502	4.005386	3.499217	3.642292	2.940936	2.936843	3.289362	0.847922461	-0.043298482	0.025902349	-0.366337567
ENSG00000108669	CYTH1	10.130464	10.078306	10.362388	13.45007	13.736297	13.516759	7.378157	6.682562	8.109009	1.96E-15	0.563470212	0.0000594	-0.366318348
ENSG00000198028	ZNF560	1.597936	2.054884	2.072352	1.436568	1.629446	1.272093	1.444202	1.159763	1.570432	0.297082759	-0.252208064	0.09453836	-0.365800038
ENSG00000144749	LRIG1	12.235958	11.982723	11.822103	10.355956	9.585038	9.88352	8.891315	8.122327	9.252917	0.086508214	-0.126498309	0.000000509	-0.365586239
ENSG00000285253	AC090517.4	0.526136	0.261728	0.307796	0.264829	0.303923	0.289246	0.196652	0.288345	0.312875	0.653113211	-0.209283538	0.365832637	-0.365535877
ENSG00000159363	ATP13A2	6.091024	5.78609	5.841883	4.834082	4.549268	5.067025	4.012646	4.097189	4.811599	0.163708642	-0.156050205	0.000821951	-0.365478712
ENSG00000159882	ZNF230	1.207155	1.690418	1.400269	1.285957	1.127452	1.30268	0.880109	1.153733	1.227913	0.930787487	-0.025869938	0.099165384	-0.365339434
ENSG00000213699	SLC35F6	7.587553	7.657843	8.116789	9.152615	9.624559	9.851371	5.991545	5.556231	5.399065	3.38E-08	0.44067318	0.000155014	-0.365289685
ENSG00000080493	SLC4A4	5.054323	5.013308	5.110483	3.533426	3.058286	3.205809	3.637675	3.820719	4.311936	2.46E-09	-0.496426148	0.0000604	-0.365144976
NewGene_232	--	0.717583	0.822765	0.659171	0.689487	0.477771	0.425497	0.731299	0.361975	0.477075	0.483517508	-0.35629793	0.432909125	-0.364895761
ENSG00000138757	G3BP2	68.091424	70.741015	65.944071	64.587924	62.670533	61.306035	47.200036	48.498609	51.938418	0.536805737	0.041514221	3.62E-10	-0.364520796
ENSG00000112294	ALDH5A1	4.416996	4.683855	4.423265	3.646157	3.425894	3.411813	3.491594	3.152212	3.387707	0.03319117	-0.216818001	0.000285065	-0.364431594
ENSG00000123552	USP45	2.178124	2.393829	2.34978	2.438126	1.900084	2.05002	1.698942	1.633558	1.655949	0.560993643	-0.0961928	0.009026516	-0.364364624
ENSG00000020256	ZFP64	8.48952	8.394671	7.652105	8.06781	7.214266	7.645796	5.418353	5.844849	6.482304	0.548902727	0.071441328	0.000451734	-0.364352877
ENSG00000271383	NBPF19	1.490404	1.344787	1.682956	1.233335	0.860646	1.078054	1.419161	0.999491	1.127177	0.012193892	-0.348681429	0.005221659	-0.364169377
ENSG00000173011	TADA2B	7.911602	8.118632	7.743486	9.432997	9.238616	8.689311	5.98473	5.236968	6.26941	0.00000432	0.35080269	0.0000527	-0.364150427
ENSG00000123992	DNPEP	10.347378	11.648507	11.438596	45.0352	35.637993	40.190027	7.76812	7.818598	7.958893	2.11E-167	1.95968241	0.000349329	-0.364058883
NewGene_3844	--	0.881115	0.920274	1.009172	0.913129	0.987537	1.060383	0.730918	0.67262	0.563276	0.38260201	0.198148824	0.114308073	-0.363879639
ENSG00000132205	EMILIN2	1.584077	1.557889	1.127117	1.438529	1.267121	1.210279	0.957443	1.017326	1.14084	0.940788786	0.019718063	0.046906234	-0.363598263
ENSG00000198836	OPA1	35.851584	41.049096	38.612994	31.671308	28.112338	30.065551	28.353632	25.879001	29.458273	0.006393687	-0.170478487	3.94E-08	-0.363492445
ENSG00000197566	ZNF624	1.768996	1.933999	1.887163	1.602564	1.378837	1.403149	1.557039	1.282635	1.288852	0.245464404	-0.210475641	0.038274935	-0.363306223
ENSG00000146267	FAXC	0.50224	0.621589	0.526538	0.29744	0.251869	0.29615	0.524772	0.387723	0.395168	0.0000153	-0.821336989	0.051885268	-0.36305074
NewGene_644	--	7.128533	7.782494	8.439161	5.198469	4.623375	4.457627	5.353842	5.231005	6.478439	5.83E-10	-0.563128313	0.000316627	-0.363042951
ENSG00000034533	ASTE1	3.481529	3.869086	3.302497	3.58066	4.039609	3.347782	2.79701	2.581581	2.539068	0.30444746	0.175483171	0.019623602	-0.362667829
ENSG00000141905	NFIC	12.064346	10.047514	11.994997	16.509253	9.980354	12.095305	10.952641	7.407145	10.040913	0.203586904	0.109269556	0.0000595	-0.362575116
ENSG00000171017	LRRC8E	2.223142	2.711731	2.687198	2.837934	2.313777	2.860437	1.706699	1.842242	1.807017	0.146139439	0.239006794	0.023040349	-0.362429743
ENSG00000157978	LDLRAP1	1.880784	2.143354	1.735798	2.14279	1.966976	2.224097	1.332866	1.281959	1.587264	0.1367021	0.283597155	0.068254047	-0.362346448
ENSG00000150764	DIXDC1	2.337422	2.52609	2.428076	1.937036	1.790481	1.769922	1.783214	1.660507	1.949581	0.040643797	-0.264734279	0.003800146	-0.362173473
ENSG00000108506	INTS2	4.457383	4.684478	5.062988	4.596052	3.95016	4.070878	3.112922	3.420618	3.477178	0.901396783	-0.018342477	0.000276563	-0.36198751
ENSG00000196357	ZNF565	1.99173	2.45226	2.168138	1.781118	1.562935	2.141132	1.514833	1.649011	1.701737	0.650110032	-0.132692616	0.110906351	-0.361688162
ENSG00000162576	MXRA8	0.736924	0.601706	0.401149	0.446657	0.388459	0.488903	0.485927	0.45694	0.354438	0.703543164	-0.194771038	0.402468661	-0.361614369
ENSG00000072041	SLC6A15	3.844192	3.859107	4.680672	3.353286	3.280384	3.235951	2.568933	3.029529	3.285521	0.22587377	-0.162316755	0.0081862	-0.361594249
NewGene_4353	--	0.79866	1.082944	0.774725	0.805737	0.783787	0.852169	0.685702	0.524549	0.727302	0.930719255	0.03070122	0.162600532	-0.361375282
ENSG00000152926	ZNF117	2.577833	2.75994	2.656053	2.680872	2.445725	2.206476	1.947876	1.968386	1.920235	0.885932195	0.023945513	0.001220964	-0.361215242
NewGene_4271	--	2.920833	3.034684	2.961994	2.829473	3.191881	2.590403	1.971833	2.189915	2.093255	0.200387902	0.246747296	0.070839926	-0.361014301
ENSG00000088854	C20orf194	1.450148	1.189022	1.381498	0.97104	0.845728	0.84644	1.08445	0.758767	1.097213	0.005362338	-0.446902067	0.042632098	-0.360729243
ENSG00000112293	GPLD1	0.556778	0.381315	0.500455	0.260038	0.263264	0.29268	0.39227	0.295787	0.366075	0.081271631	-0.671336569	0.330704691	-0.360719235

Gene ID	Gene name	Ctrl-1 FPKM	Ctrl-2 FPKM	Ctrl-3 FPKM	IFNγ-1 FPKM	IFNγ-2 FPKM	IFNγ-3 FPKM	Sora-1 FPKM	Sora-2 FPKM	Sora-3 FPKM	Ctrl vs IFNγ DESeq2 FDR	Ctrl vs IFNγ DESeq2 log2FC	Ctrl vs Sora DESeq2 FDR	Ctrl vs Sora DESeq2 log2FC
ENSG00000139793	MBNL2	10.904888	12.168725	14.910315	8.785246	10.382904	8.426891	9.009443	9.221276	10.763647	0.001671985	-0.323705098	0.000704554	-0.360675265
ENSG00000178252	WDR6	51.150376	48.077443	46.824426	43.19617	45.046277	43.427962	31.015984	35.736747	38.013323	0.812846092	0.020449707	0.00000188	-0.360669564
ENSG00000020922	MRE11	6.2168	6.995688	6.695831	6.092178	5.613437	5.417733	4.915262	5.466967	5.023662	0.321397965	-0.093200876	0.0000163	-0.360637498
ENSG00000121671	CRY2	8.875341	7.908978	6.779969	6.957574	6.731738	7.596433	6.491589	5.670678	5.07047	0.950252767	-0.010545972	0.005012919	-0.360627021
NewGene 5681	--	0.902202	0.920563	0.998296	0.558786	0.50959	0.452712	0.54486	0.667601	0.704773	0.107807922	-0.590235394	0.312284544	-0.360474682
ENSG00000165476	REEP3	5.432357	6.649383	7.025916	6.102822	5.717767	6.108136	4.603522	4.635591	4.739533	0.713411608	0.051759301	0.001110212	-0.360275593
ENSG00000148943	LIN7C	9.206324	12.682173	13.373096	11.593093	11.025763	10.784939	8.68679	7.780187	9.328099	0.645467018	0.069532285	0.003328784	-0.360243345
ENSG00000136014	USP44	2.800607	3.913951	3.276394	2.774272	3.179653	2.598194	2.45737	2.412976	2.260326	0.238231145	-0.197259186	0.021777775	-0.360200431
ENSG00000081051	AFP	213.459549	187.335861	161.818756	132.476181	130.159714	125.108635	137.530853	134.522934	138.977966	0.00000128	-0.390075433	0.0000358	-0.360061986
ENSG00000146112	PPP1R18	11.072019	8.228019	9.051071	7.665282	8.768578	8.551724	5.715933	5.661135	5.287172	0.233105131	-0.148909419	0.000918085	-0.360048112
ENSG00000167264	DUS2	3.174002	3.010367	2.339092	2.505622	2.84134	3.043467	2.104402	2.112175	2.144805	0.885275856	0.040751097	0.067409352	-0.359883346
ENSG00000124357	NAGK	11.67544	12.857997	10.49907	10.066458	10.256585	11.639687	8.663015	8.480547	8.203034	0.893880662	-0.022098909	0.004796689	-0.359398852
NewGene 4570	--	1.785747	2.343579	2.327773	2.506497	2.222027	1.615672	2.404884	1.364359	2.191362	0.999202724	-0.000391632	0.021145982	-0.359360281
ENSG00000118965	WDR35	3.016241	3.540333	3.695441	2.029059	1.880546	1.789915	2.38694	2.395343	2.842237	5.23E-12	-0.786600994	0.002206886	-0.359301207
ENSG00000150938	CRIM1	5.249	5.238736	5.869381	5.49923	4.785234	5.141111	3.801342	3.969944	4.43943	0.516582609	0.075903114	0.000180122	-0.359262949
ENSG00000181135	ZNF707	4.943958	4.991105	3.451781	3.839581	2.715923	3.487829	2.839679	2.898144	3.597236	0.659905817	-0.09505334	0.042195928	-0.359211291
ENSG00000138449	SLC40A1	32.962433	34.326912	33.513622	26.700575	29.057459	26.885146	26.026428	23.300397	24.413916	0.043773754	-0.140170204	0.000000131	-0.359123103
NewGene 3034	--	0.753338	0.800468	0.668156	0.842215	0.665583	0.630342	0.466079	0.629465	0.610294	0.893775315	0.042789584	0.149206811	-0.359055095
ENSG00000146232	NFKBIE	11.641975	9.898035	9.605405	4.132213	3.694226	3.826115	7.025621	7.569147	8.289811	4.84E-32	-1.268891301	0.001466915	-0.358905136
ENSG00000047365	ARAP2	2.301	2.055659	2.76041	5.532626	5.049472	5.261204	1.748177	1.87264	1.708834	6.16E-38	1.250232201	0.007293842	-0.358850892
ENSG00000170852	KBTBD2	31.550587	33.771225	36.46188	29.616937	28.172541	27.90565	23.940959	22.520604	27.3613	0.23224086	-0.091342045	0.00000439	-0.358807878
ENSG00000175348	TMEM9B	20.7216	20.472738	19.595469	16.333007	18.624725	18.285887	14.363686	15.040153	15.655712	0.398562843	-0.086716418	0.0000222	-0.358663085
ENSG00000153207	AHCTF1	19.818616	21.522955	23.323236	20.589004	19.485873	19.316414	15.998647	15.121696	16.298598	0.818894795	0.022005367	0.000000483	-0.358489282
ENSG00000127526	SLC35E1	14.563397	14.519594	14.057675	11.74411	12.057924	12.277532	10.565375	9.638393	11.731787	0.088760391	-0.1226165	0.00000167	-0.358360272
ENSG00000253293	HOXA10	4.482878	4.403613	4.486537	2.674992	3.396742	3.491665	3.1782	3.158431	3.226222	0.060920532	-0.298535974	0.017567189	-0.358290383
ENSG00000159322	ADPGK	27.691077	25.695505	25.914533	22.214232	22.412611	22.795148	19.49404	19.778677	17.972141	0.144639048	-0.108295673	0.0000117	-0.358166749
ENSG00000056586	RC3H2	7.278505	7.548014	7.354903	7.057866	6.877123	6.566103	6.308183	5.678743	6.179376	0.931690576	0.009496635	0.0000036	-0.357992089
ENSG00000163788	SNRK	2.157829	2.413609	2.685579	3.275995	3.423703	3.246858	2.078666	1.511779	1.680027	0.000000161	0.603149145	0.023227305	-0.357981228
NewGene 3035	--	3.427382	4.028241	4.881874	2.167283	2.213751	2.55877	2.584004	2.979494	2.706866	0.009660381	-0.378684515	0.01802805	-0.357937202
ENSG00000198408	OGA	9.005691	9.242665	9.567234	10.350099	8.418695	9.252667	7.763114	6.218931	6.603826	0.085778836	0.159359945	0.000145401	-0.357865318
ENSG00000151914	DST	14.19619	14.67352	15.654371	9.605661	10.035016	10.188232	11.652601	11.274685	12.707027	1.7E-12	-0.459413439	0.00000144	-0.357749089
ENSG00000121864	ZNF639	28.631001	27.399817	31.83584	29.818339	27.457221	27.804623	24.964766	19.867138	25.212908	0.168169476	0.123867155	0.000093	-0.357407127
ENSG00000113448	PDE4D	3.65032	3.601182	3.777751	6.581214	6.068589	5.904901	3.210353	2.902004	3.474892	3.2E-34	0.934810113	0.000437304	-0.357324367
ENSG00000101945	SUV39H1	6.060135	6.104445	5.63805	4.863446	4.85041	5.451258	4.376317	3.684736	5.020445	0.568731076	-0.082886014	0.005103284	-0.357152527
ENSG00000079432	CIC	14.132545	13.018885	12.040173	14.076832	14.134555	14.085639	9.115246	8.837072	10.987219	0.010108783	0.198346637	0.00010141	-0.3570601
ENSG00000157625	TAB3	19.325515	17.312595	17.745651	17.53777	17.328426	16.267309	12.446931	12.143046	13.832879	0.431311469	0.058350269	2.94E-08	-0.357039316
ENSG00000170846	AC093323.1	5.156744	5.672406	5.33641	5.433458	5.207817	4.81312	4.92464	3.758634	3.280825	0.889945453	0.026752161	0.035859189	-0.356812025
ENSG00000124198	ARFGEF2	12.276056	12.320309	13.193183	13.983706	14.033913	13.133681	9.057916	8.611948	10.0386	0.00000947	0.270085543	0.000000271	-0.356784575
ENSG00000259207	ITGB3	0.381051	0.315028	0.242757	0.065818	0.130515	0.138434	0.276981	0.197767	0.22762	0.006595477	-1.303165508	0.412599976	-0.3565809
ENSG00000136383	ALPK3	6.842282	6.575933	6.859364	4.226475	3.886577	4.203216	4.967362	4.235875	5.69682	6.32E-19	-0.572111186	0.0000606	-0.356482762
ENSG00000105793	GTPBP10	3.436118	4.186573	4.448497	4.0561	3.73422	3.69251	3.298868	3.111321	2.880272	0.697572728	0.057931994	0.00288243	-0.356324195
ENSG00000136715	SAP130	13.137979	12.492008	12.196184	12.156856	11.942057	11.843029	9.31824	8.876342	9.563621	0.399718075	0.072968947	0.0000374	-0.356307139
ENSG00000250644	AC068580.4	6.208023	0.138563	0.869514	5.342091	0.997479	0.801792	2.513685	0.656254	2.280746	0.99264631	-0.027498178	0.840241952	-0.355782878
ENSG00000103404	USP31	4.050347	4.31825	4.779982	4.062257	3.797999	3.809309	3.644415	3.18923	3.860056	0.500495257	-0.072832022	0.000110543	-0.3557266
ENSG00000025708	TYMP	1.393923	1.160388	0.932987	10.235056	8.677851	10.058911	0.642135	0.776278	1.118927	6.33E-89	3.190064929	0.338470469	-0.355585481
ENSG00000006194	ZNF263	14.523217	12.72716	12.893397	11.957078	12.33052	11.326626	9.611204	9.109128	9.107983	0.578152313	-0.059220106	0.0000532	-0.355427574
NewGene 1233	--	6.222549	5.70803	5.397786	4.354613	3.185763	4.491167	4.577008	3.532723	5.184644	0.000000173	-0.523597089	0.000914081	-0.355413849
ENSG00000157765	SLC34A2	6.231686	5.930501	7.027303	0.837823	1.174218	1.05612	4.57471	4.359096	4.970739	1.66E-85	-2.552423402	0.001079177	-0.355149569
ENSG00000115548	KDM3A	20.444143	19.469871	17.894218	16.395185	16.179174	16.279059	13.795066	13.621402	13.894152	0.425781292	-0.065598424	0.000000888	-0.355030278
ENSG00000110906	KCTD10	16.115807	15.406955	15.895178	13.370719	13.104625	13.128371	11.763412	10.947528	12.163607	0.096897882	-0.12304931	0.00000973	-0.35488567
ENSG00000166135	HIF1AN	5.201254	5.457169	5.342396	5.146509	4.389614	4.332997	3.962931	3.586719	4.353093	0.39594485	-0.077600843	0.00000301	-0.354414176
ENSG00000173456	RNF26	25.5865	24.277622	24.112804	22.231146	21.183317	20.880623	18.110796	17.415083	18.780434	0.514033421	-0.055742002	0.000000657	-0.353812542
ENSG00000176208	ATAD5	4.18634	4.795001	4.417979	5.010305	4.100638	4.686439	3.627791	3.084789	3.116796	0.070081588	0.190554091	0.000904773	-0.353706409
ENSG00000134987	WDR36	8.22837	9.97146	9.396612	8.798433	7.802564	8.246995	6.732448	6.136026	7.423952	0.973926493	0.004829304	0.000285424	-0.353620534
NewGene 1157	--	7.348515	6.881552	7.170529	6.456908	5.897408	6.339068	5.455172	5.939551	6.310912	0.912430732	0.020863116	0.010326938	-0.353478953
ENSG00000130675	MNX1	2.959923	3.445671	2.995116	3.613334	2.97252	2.697096	2.244556	1.809566	2.146338	0.610804218	0.120694184	0.071662606	-0.353363354
ENSG00000105088	OLFM2	33.462454	27.024211	26.327564	14.035798	15.049649	14.528113	19.843316	21.790871	21.444631	2.87E-19	-0.845577881	0.000790314	-0.353318647
ENSG00000125430	HS3ST3B1	11.577426	11.978287	14.242172	15.907365	18.769603	15.22558	8.179347	8.512478	10.700955	8.23E-17	0.52873237	0.0000907	-0.353250809
ENSG00000138081	FBXO11	14.493061	15.468806	14.250676	17.219038	16.935855	15.120505	11.049654	9.796218	10.909038	0.001431876	0.23029586	0.000122841	-0.353247771
ENSG00000196263	ZNF471	1.136002	1.035866	0.80164	1.183109	0.771419	0.807394	0.754542	0.617675	0.909239	0.896584156	0.03581354	0.076819666	-0.35313285
ENSG00000089916	GPATCH2L	4.796982	4.559439	4.325534	2.836573	3.575602	3.286561	3.67628	3.50279	3.661794	0.00000262	-0.415819102	0.0000304	-0.353104545
ENSG00000211452	DIO1	0.963588	0.940815	0.510967	0.668259	0.875687	0.588114	0.682497	0.57355	0.363494	0.906752382	0.066782259	0.442374073	-0.353097203
ENSG00000100106	TRIOBP	9.692951	13.586252	7.7334	6.977026	8.953934	8.421971	8.40106	8.837729	9.83078	0.427765571	-0.091478018	0.00013882	-0.352901026
ENSG00000197381	ADARB1	3.443159	4.398637	3.552257	2.544358	3.696603	3.3403	3.082104	3.044433	3.639516	0.394899676	-0.118856194	0.003274015	-0.35255376
ENSG00000173898	SPTBN2	19.396487	18.341502	15.817713	17.809241	15.468066	17.658645	12.667802	11.927285	14.743991	0.487926125	0.07122914	0.0000555	-0.352533386
ENSG00000185305	ARL15	2.897311	3.198641	3.444738	2.495938	2.554756	2.544324	2.390769	2.034498	2.472365	0.41140592	-0.142312088	0.022583076	-0.352438627
NewGene 630	--	4.112532	4.034345	3.597983	3.386248	3.202168	2.948798	2.676463	3.055364	2.884166	0.30947561	-0.154670769	0.011727542	-0.352094739
NewGene 2608	--	1.684162	1.737896	1.950715	1.299394	1.600226	1.529651	1.737644	1.733672	1.673479	0.40710097	-0.182184606	0.064249744	-0.351990424
NewGene 4040	--	3.938707	4.310235	4.523523	4.13379	3.796356	3.874624	2.885862	3.178695	3.268921	0.864880029	0.023891521	0.000752014	-0.35191441
ENSG00000101400	SNTA1	2.322595	2.611346	1.793817	2.063261	1.731567	1.987224	1.841326	1.471563	1.633323	0.804040855	-0.070624604	0.114303831	-0.351878417
ENSG00000227124	ZNF717	2.621299	2.980998	2.025095	2.679608	2.134381	2.020049	1.891613	1.785214	1.52494	0.622917635	-0.219933062	0.271055854	-0.351844556
ENSG00000008853	RHOBTB2	3.112665	2.824299	2.628962	2.122256	2.358906	2.336775	1.932462	2.035039	2.31756	0.207527602	-0.177426948	0.006588051	-0.351814706
ENSG00000147324	MFHAS1	8.550172	8.192263	9.279732	6.54797	6.527975	6.524402	6.131529	6.14514	6.861904	0.00070535	-0.261620301	0.0000213	-0.351723324
ENSG00000108587	GOSR1	12.257803	13.551818	13.159094	13.042021	12.555818	12.180388	10.193002	9.127955	10.093429	0.266241124	0.089107048	0.00000576	-0.351455621

Gene ID	Gene name	Ctrl-1 FPKM	Ctrl-2 FPKM	Ctrl-3 FPKM	IFNγ-1 FPKM	IFNγ-2 FPKM	IFNγ-3 FPKM	Sora-1 FPKM	Sora-2 FPKM	Sora-3 FPKM	Ctrl vs IFNγ DESeq2 FDR	Ctrl vs IFNγ DESeq2 log2FC	Ctrl vs Sora DESeq2 FDR	Ctrl vs Sora DESeq2 log2FC
ENSG00000115514	TXNDC9	34.753088	35.54872	39.988402	34.811566	35.66942	35.235017	26.434789	28.050118	28.210133	0.320079766	0.090248053	0.0000513	-0.351107057
ENSG00000150347	ARID5B	3.344954	3.298193	3.575576	3.26773	3.220757	3.677953	2.460119	2.570342	2.768626	0.154884321	0.149875912	0.000221786	-0.351106049
ENSG00000176731	C8orf59	17.597786	21.089629	21.733697	22.699276	21.302493	22.123413	15.305068	15.469881	12.154485	0.058042119	0.241643549	0.032440989	-0.351042565
ENSG00000132570	PCBD2	2.706617	4.021986	4.160558	2.461295	1.973687	2.811703	2.823046	3.445792	3.116918	0.09835278	-0.340655556	0.086981432	-0.350551727
ENSG00000100902	PSMA6	202.494884	213.280122	204.013161	256.764176	246.509889	270.757815	109.89469	173.843921	160.843879	1.91E-28	0.530520167	0.078706095	-0.350545574
ENSG00000164164	OTUD4	6.638099	6.827934	7.710154	11.011634	10.531497	10.113974	5.516475	4.73686	5.71324	5.18E-26	0.737558123	0.000117123	-0.350462545
ENSG00000166224	SGPL1	7.315155	6.541867	6.624607	6.193047	6.282783	5.700497	5.008458	4.694731	5.439992	0.812230324	-0.029107308	0.0000837	-0.350411163
NewGene_2934	--	0.962508	1.239174	0.713812	1.04905	1.27799	0.975707	0.81066	0.838915	0.599061	0.650110032	0.232586866	0.462615622	-0.3502935
ENSG00000126821	SGPP1	4.001467	4.503881	4.51705	3.487718	3.205112	3.019902	3.187469	2.517504	3.915973	0.036056919	-0.276565668	0.022279605	-0.350054078
ENSG00000172831	CES2	6.31556	5.136958	4.824281	6.877331	7.1931	6.278001	4.333694	3.900995	3.903547	0.000103797	0.43025488	0.006452185	-0.349929221
ENSG00000115446	UNC50	25.075912	20.224618	22.369641	23.490367	22.185343	22.841466	16.742291	16.320979	17.187231	0.019167286	0.221324794	0.000536785	-0.349919102
ENSG00000182149	IST1	46.101904	37.705466	39.549227	40.955209	34.45355	46.807492	27.373228	30.994259	27.523321	0.072182174	0.111253053	3.44E-08	-0.349918747
ENSG00000106261	ZKSCAN1	16.756419	16.75707	16.909273	16.269717	16.894592	16.15802	12.378727	10.468739	13.965248	0.111225899	0.101980395	0.0000124	-0.34991406
ENSG00000181104	F2R	3.992028	4.368334	4.539686	2.424554	2.325612	2.220819	2.98763	2.782142	3.746598	1.45E-10	-0.747627238	0.008364269	-0.349876397
ENSG00000117834	SLC5A9	2.375955	2.760733	2.684672	0.803053	1.030988	1.001007	2.152451	1.768152	2.015155	1.72E-13	-1.277233271	0.031479914	-0.34958004
ENSG00000185361	TNFAIP8L1	59.537617	55.821457	53.123047	49.03919	51.441614	54.288042	41.805067	39.825729	42.401219	0.787608361	0.025538534	5.44E-08	-0.349553067
ENSG00000001167	NFYA	81.119413	79.826835	78.711497	71.875157	70.343621	71.514907	57.376264	57.342344	61.564128	0.79046308	-0.018145322	1.61E-10	-0.34927088
ENSG00000103550	KNOP1	9.224918	9.788765	9.441219	7.80503	7.329142	8.481521	7.497273	6.822567	7.112563	0.128602213	-0.129309937	0.00000335	-0.349234997
ENSG00000147133	TAF1	3.484959	3.632978	4.118256	4.341981	4.291008	3.398539	2.655034	2.74671	2.62992	0.19984644	0.136767431	0.000724755	-0.349210572
ENSG00000070159	PTPN3	12.717001	12.199416	13.111391	13.54318	13.392906	11.427382	9.01403	8.372473	10.395126	0.004528711	0.195202121	0.0000131	-0.348996334
ENSG00000165757	JCAD	0.643092	0.666446	0.806086	0.292287	0.362044	0.393816	0.570533	0.451298	0.535268	0.0000139	-0.858732368	0.068892101	-0.348809778
ENSG00000068305	MEF2A	12.463897	13.260581	14.023171	12.885477	13.153507	12.880921	9.928175	9.148282	10.722161	0.298815542	0.088519315	0.0000152	-0.348613237
ENSG00000134717	BTF3L4	13.806968	14.455323	14.742592	10.578529	10.453975	12.72588	10.906202	11.782729	11.799269	0.020184534	-0.185845976	0.0000075	-0.34853791
ENSG00000139160	ETFBKMT	0.193978	0.253367	0.16696	0.167733	0.272433	0.187906	0.233382	0.102927	0.102113	0.783883804	0.14478359	0.48953244	-0.348235991
ENSG00000143674	MAP3K21	4.676376	5.216459	5.879481	4.473454	4.455772	4.502231	4.24602	3.778809	4.631354	0.13261482	-0.171411882	0.002184107	-0.34811357
ENSG00000126804	ZBTB1	10.832383	10.518161	11.587211	9.573265	9.697445	8.688901	8.414359	7.856709	8.679631	0.305187573	-0.106077772	0.000121274	-0.348083783
ENSG00000163281	GNPDA2	5.424469	6.293563	6.898417	5.740993	5.091173	5.213337	4.790952	4.821528	4.32633	0.947982103	-0.012090051	0.005648108	-0.348054244
NewGene_661	--	1.800738	1.048889	0.901538	1.256538	1.440174	1.472781	0.530188	1.306734	1.09367	0.464621682	0.336279599	0.493480799	-0.348015499
ENSG00000183283	DAZAP2	56.852258	51.07607	47.818802	56.324808	57.398178	55.282177	36.482003	41.589596	36.943372	0.00000321	0.279865391	0.0000148	-0.347880889
ENSG00000168282	MGAT2	17.337225	16.353462	15.492496	20.400902	20.584145	20.025509	11.436563	12.01298	12.805555	1.37E-10	0.457684127	0.0000626	-0.347691838
ENSG00000181722	ZBTB20	0.72811	0.771137	0.882022	0.71218	0.744988	0.69145	0.846806	0.622964	0.873827	0.933602721	0.014033461	0.003233508	-0.347581944
ENSG00000137343	ATAT1	9.404082	10.760933	10.244427	8.916499	7.094098	6.322054	8.849306	8.574843	8.294955	0.000774156	-0.387226695	0.00079522	-0.347483349
NewGene_2539	--	0.77878	1.307535	0.772963	1.076569	1.028094	0.904034	0.870705	0.565804	0.763445	0.412938387	0.228772859	0.187487868	-0.347469643
NewGene_2941	--	9.684035	9.529105	10.029679	9.155503	8.3424	8.186013	6.704422	6.87242	8.006212	0.621690622	-0.040806162	0.00000162	-0.347427144
ENSG00000013503	POLR3B	8.27916	8.773174	8.868195	8.460866	7.846007	7.845703	6.475842	6.04454	6.590543	0.681397579	0.044597848	0.00005	-0.347173495
ENSG00000105248	YJU2	31.26409	29.249997	30.933519	29.369434	29.652961	28.97808	21.477322	23.317177	22.529588	0.325738665	0.087499564	0.0000708	-0.346906118
ENSG00000196177	ACADSB	12.752886	13.153453	11.087142	15.726262	14.979721	14.56616	9.512015	8.740647	9.117882	1.75E-09	0.43599678	0.0000408	-0.346792986
ENSG00000168300	PCMTD1	5.014615	5.68326	6.123065	5.484091	5.425372	4.939394	4.300242	3.975463	3.776723	0.743114477	0.053512389	0.008155047	-0.346540555
ENSG00000035141	FAM136A	58.063712	57.915323	59.820791	50.610188	45.555819	47.449314	43.819197	42.982584	43.426948	0.020639478	-0.151129129	0.000000111	-0.346405059
ENSG00000105808	RASA4	2.830477	2.20758	1.262863	1.367156	2.928342	3.079995	1.884083	1.831687	1.279572	0.747687405	0.201093756	0.128623115	-0.346364051
ENSG00000149925	ALDOA	602.501986	513.606277	498.945588	413.937767	438.150925	431.297873	385.123548	393.366425	420.873434	0.01678134	-0.185929041	0.00000662	-0.346196005
ENSG00000140691	ARMC5	4.124788	3.856549	3.620768	3.992579	3.645248	3.748719	2.73381	2.461879	3.362168	0.334697238	0.130437748	0.006899658	-0.346110363
ENSG00000177432	NAP1L5	1.932446	2.525224	2.049285	2.587982	2.055427	2.53318	1.607444	1.734349	1.430935	0.213158778	0.291351682	0.151154863	-0.345815772
ENSG00000164715	LMTK2	10.810896	10.259862	10.091767	14.142457	12.323639	13.47109	7.81522	6.902713	8.321287	3.91E-16	0.503633076	0.00000301	-0.345629855
ENSG00000170185	USP38	4.876077	4.361245	4.573532	4.728965	4.798984	4.520588	3.125285	2.968678	3.941911	0.040372713	0.184389062	0.001319651	-0.345622223
ENSG00000187624	C17orf97	2.67595	2.378729	2.336408	2.08019	2.086987	1.615208	1.771086	1.903169	1.773688	0.373698839	-0.222798919	0.110656938	-0.34550509
ENSG00000206530	CFAP44	2.321134	2.383744	3.51643	2.633338	2.153421	1.434813	1.51021	2.163895	2.092731	0.185858622	-0.259573921	0.034995358	-0.345403341
ENSG00000186767	SPIN4	1.5522	2.293077	2.377255	2.194232	2.458198	2.04023	1.534599	1.443575	1.619173	0.208322652	0.2529597	0.072329496	-0.345108188
ENSG00000241839	PLEKHO2	2.181452	1.889214	1.51735	1.66363	1.41457	1.424795	1.158174	1.393205	1.578614	0.468155425	-0.165425586	0.091624802	-0.345054541
ENSG00000249471	ZNF324B	1.112143	1.319118	1.214013	1.320541	1.340806	1.34515	1.016181	0.811194	0.956831	0.450792538	0.207480721	0.204096231	-0.344815165
ENSG00000152193	RNF219	7.099258	7.889095	7.495791	6.849072	6.101866	6.596811	5.54738	5.390399	5.658671	0.665682534	-0.054457669	0.00040763	-0.34477392
ENSG00000180834	MAP6D1	0.634411	1.211	0.360231	0.423975	0.217799	0.419647	0.088137	0.637726	0.853866	--	--	0.814652612	-0.344632354
ENSG00000157106	SMG1	8.599609	9.232482	9.740105	13.064012	11.701714	11.547295	7.513024	6.152459	6.984055	2.08E-18	0.54460275	0.000014	-0.344415552
ENSG00000173230	GOLGB1	9.097499	9.687187	8.480688	13.617416	13.797079	12.906496	7.12026	6.714864	7.132893	2.39E-32	0.678812968	0.000000926	-0.344355357
ENSG00000126814	TRMT5	3.16795	3.486877	3.583417	2.930066	3.014193	3.028729	2.661514	2.687933	2.407037	0.810146693	-0.03754618	0.005257426	-0.344246717
ENSG00000198894	CIPC	3.829548	3.872806	3.970441	3.156466	3.181939	2.895489	2.859257	2.890565	2.831644	0.129428181	-0.185559617	0.002837618	-0.344113747
ENSG00000169193	CCDC126	3.80654	4.061781	3.640278	3.538152	4.199768	3.675765	2.535027	3.017555	2.881069	0.537220785	0.116018743	0.031044924	-0.343921927
ENSG00000047315	POLR2B	91.642938	87.230908	91.759148	91.914391	82.972972	87.27789	67.32232	65.907706	71.798507	0.031559467	0.10638107	1.7E-10	-0.343422278
NewGene_2849	--	4.575486	4.015529	5.612852	1.392756	1.71222	1.851141	3.293629	3.46505	4.083583	9.34E-10	-1.38886028	0.109328274	-0.343317079
ENSG00000198160	MIER1	6.775713	7.343318	7.757352	9.766055	8.218328	7.839261	6.198145	5.143776	6.073907	0.000827133	0.301239704	0.001776479	-0.343095557
ENSG00000196159	FAT4	0.372905	0.355534	0.35791	0.59979	0.569777	0.504819	0.303384	0.19771	0.305241	0.000000163	0.767591451	0.105812721	-0.342970793
ENSG00000089195	TRMT6	14.867639	15.024261	17.294076	13.129352	12.333547	12.268399	12.018282	10.83973	11.994292	0.076992166	-0.174006018	0.000358529	-0.342765015
ENSG00000174599	TRAM1L1	1.61349	1.719843	1.71857	1.446304	1.62441	1.722233	1.437057	1.00176	1.29496	0.815012144	0.073196925	0.18161183	-0.342746675
ENSG00000167258	CDK12	26.09855	25.652749	25.312499	33.375595	31.637548	31.220174	19.205605	18.286416	20.324221	4.73E-23	0.467915368	1.04E-08	-0.342696554
ENSG00000111605	CPSF6	36.181995	35.953435	50.345083	36.256055	30.608345	31.983567	24.090447	26.629544	31.879215	0.99539337	0.00056761	1.97E-08	-0.342453523
ENSG00000108861	DUSP3	57.845017	54.995767	57.78701	32.08078	34.460001	35.088471	41.571953	40.323266	44.64662	9.33E-31	-0.62937004	5.35E-09	-0.342450503
ENSG00000175745	NR2F1	7.387789	6.307785	6.124084	5.985336	5.66086	5.781367	4.259086	4.429739	5.994572	0.797269671	-0.037467785	0.010271492	-0.342427565
ENSG00000100558	PLEK2	2.464087	2.015839	2.710869	1.640159	1.481084	1.441757	1.798287	1.82507	1.644827	0.052743632	-0.492919302	0.170294616	-0.342220366
ENSG00000242852	ZNF709	0.247817	0.35042	0.329376	0.170998	0.267483	0.282874	0.247973	0.271417	0.165058	0.683125228	-0.199302231	0.405877657	-0.341903197
ENSG00000121236	TRIM6	3.057694	3.539894	3.761183	2.74791	2.623509	2.48416	2.653967	2.370296	2.396512	0.13005881	-0.232840402	0.021345737	-0.341887693
ENSG00000132561	MATN2	1.405579	1.138278	1.217282	0.719272	0.664242	0.593762	1.187011	0.804567	0.857759	0.000435687	-0.778765306	0.140962509	-0.341805517
ENSG00000196793	ZNF239	3.310903	3.517007	3.441731	2.678207	2.031855	2.958397	2.064913	3.074697	2.376871	0.457408931	-0.157577593	0.079720643	-0.341649658
ENSG00000186416	NKRF	4.672079	5.451981	6.060657	4.851253	4.057776	4.782199	4.172354	3.931163	3.822971	0.591485549	-0.086488016	0.00826619	-0.341533451
ENSG00000149054	ZNF215	1.505071	1.745314	2.131929	1.437996	1.672898	1.6082	1.563044	1.122069	1.27578	0.786785017	-0.072609486	0.113802516	-0.341407163

Gene ID	Gene name	Ctrl-1 FPKM	Ctrl-2 FPKM	Ctrl-3 FPKM	IFNγ-1 FPKM	IFNγ-2 FPKM	IFNγ-3 FPKM	Sora-1 FPKM	Sora-2 FPKM	Sora-3 FPKM	Ctrl vs IFNγ DESeq2 FDR	Ctrl vs IFNγ DESeq2 log2FC	Ctrl vs Sora DESeq2 FDR	Ctrl vs Sora DESeq2 log2FC
ENSG00000160862	AZGP1	25.90723	18.537769	13.416647	7.489529	8.320964	6.821734	14.711028	15.083211	14.010096	0.000130322	-1.232704668	0.341592333	-0.341339724
ENSG00000138182	KIF20B	6.113807	7.089278	7.175748	5.74023	4.549599	5.632829	4.821727	4.879523	5.368711	0.061037342	-0.205883899	0.000242553	-0.341259629
ENSG00000198841	KTI12	7.729199	6.724618	7.821245	7.208639	6.348426	7.224043	5.2818	6.047457	5.120115	0.794221078	0.046935547	0.020383181	-0.341231191
ENSG00000154945	ANKRD40	12.568305	12.554547	14.115926	13.423696	11.827701	11.920285	9.964026	10.158144	10.416918	0.59195448	0.055847921	0.0000366	-0.340886857
ENSG00000135763	URB2	9.252519	9.256731	9.539652	8.540668	7.469312	7.842905	6.978205	6.694673	7.716723	0.106395122	-0.126917351	0.0000195	-0.340792774
ENSG00000110066	KMT5B	58.420015	60.704448	57.861407	51.708544	48.363944	45.90161	48.446981	42.633201	40.958455	0.095481733	-0.095076709	1.49E-08	-0.340537788
ENSG00000110786	HSD17B1	1.912912	2.157334	1.787555	1.77354	1.981544	2.644292	1.834027	1.617551	1.67428	0.149140757	0.225578562	0.027155769	-0.340503704
ENSG00000158716	DUSP23	10.874507	9.612747	9.877326	6.901039	5.920784	6.957833	8.193921	7.636263	6.989568	0.005579666	-0.474223583	0.049952887	-0.339693816
ENSG00000197136	PCNX3	21.234598	19.941975	18.262827	20.446774	19.794144	20.713072	13.494018	13.821239	16.817669	0.008255087	0.183507134	0.000069	-0.339649239
ENSG00000175104	TRAF6	3.669302	3.046749	3.575906	3.956528	3.571032	3.330625	2.523379	2.270845	2.837223	0.087353292	0.22469866	0.010441087	-0.339620416
NewGene 6469	--	8.146633	9.339442	9.468806	8.890838	7.244478	7.995895	6.856794	6.38331	6.744866	0.79129051	-0.039033347	0.001098881	-0.339614325
ENSG00000125686	MED1	19.677802	20.698009	21.021334	20.024279	20.073547	19.621714	15.798637	15.421628	15.425668	0.237667165	0.081231647	0.00000036	-0.33931813
NewGene 3382	--	2.458986	2.319589	2.034579	1.434404	1.994067	1.456184	1.594931	1.507858	1.981265	0.206326306	-0.400573866	0.232105764	-0.339123424
ENSG00000186280	KDM4D	0.464575	0.501753	0.495423	0.392562	0.420675	0.407156	0.371079	0.231684	0.50438	0.860442578	-0.088112256	0.416638449	-0.339057813
ENSG00000174276	ZNHIT2	9.627373	9.037714	7.936954	8.964916	9.381976	10.085646	5.759578	7.1064	6.829922	0.0868698	0.244155673	0.027062399	-0.339040608
ENSG00000163866	SMIM12	11.59379	12.478227	8.198527	7.9491	9.75206	9.064494	5.964521	7.217871	6.213713	0.505202513	-0.084246296	0.002206391	-0.339014024
NewGene 1185	--	1.09566	1.149414	1.262259	2.609834	2.26434	2.180173	0.911122	0.943628	0.905462	8.14E-17	1.224067637	0.123447636	-0.33890275
ENSG00000177383	MAGEF1	27.091978	26.61175	26.208347	28.284317	28.531595	28.436705	18.996634	19.363323	20.915886	0.000890778	0.240564596	0.0000273	-0.338879292
ENSG00000128699	ORMDL1	31.112025	32.794052	36.900055	32.578625	32.241884	31.15789	25.212072	23.649495	23.651289	0.990822061	-0.001563636	0.000159953	-0.338848483
ENSG00000147862	NFIB	0.930786	1.055861	1.175404	1.074086	1.118805	1.109581	1.254229	1.008978	0.901351	0.429130477	0.148449697	0.049632514	-0.338767575
ENSG00000177189	RPS6KA3	14.119567	13.649676	15.418829	16.126865	15.470862	15.767715	12.463065	12.794683	12.716783	0.0000267	0.286366239	0.0000118	-0.338711151
ENSG00000171105	INSR	7.013514	6.873565	7.340158	5.373732	4.993585	5.481589	5.620962	4.955859	5.730667	0.0000799	-0.276925055	0.0000109	-0.33865313
ENSG00000160094	ZNF362	6.035455	6.198391	5.510127	5.646423	5.392425	6.177447	4.492213	4.380744	4.36113	0.430906937	0.105006833	0.003423849	-0.338522742
NewGene 6437	--	4.252423	3.931383	3.912737	5.576364	4.052622	4.122822	3.190874	2.300614	3.10794	0.002308636	0.377287175	0.003212508	-0.338489095
ENSG00000164406	LEAP2	0.633961	0.447585	0.545889	0.476758	0.564838	0.71294	0.448319	0.300643	0.463718	0.572931214	0.264909024	0.446071141	-0.338353418
ENSG00000172954	LCLAT1	3.38187	3.644667	3.733249	2.264333	2.541125	2.469924	2.676432	2.607914	2.898903	0.000356624	-0.414274977	0.004428225	-0.338303864
ENSG00000131941	RHPN2	17.451626	18.574719	18.428426	20.293521	20.368713	20.34742	13.628116	12.056693	14.790147	0.000000676	0.313687488	0.0000272	-0.338129373
ENSG00000198298	ZNF485	1.594437	1.070728	1.24596	1.31978	1.208067	1.052126	1.052825	0.873379	0.978455	0.976455272	0.013059107	0.259464768	-0.338010182
ENSG00000198707	CEP290	2.758131	2.68589	2.752885	2.185411	2.091006	2.260177	2.323427	1.724566	2.003154	0.074943799	-0.217293463	0.004618899	-0.337972691
ENSG00000196150	ZNF250	2.103887	1.548793	1.798325	1.771602	1.81751	1.496896	1.32448	1.350283	1.520389	0.865171003	0.040441883	0.054465865	-0.337883173
ENSG00000163481	RNF25	12.797243	12.262444	11.411326	9.731799	10.421038	11.435094	7.79719	9.664131	9.703667	0.619124037	-0.069186469	0.00471033	-0.337684545
ENSG00000153827	TRIP12	35.327666	34.491494	36.138192	32.5693	33.09059	31.106716	27.304203	25.762489	27.739938	0.934457216	0.006743503	1.12E-08	-0.337547023
ENSG00000136603	SKIL	26.018263	28.1275	37.715105	32.6443	33.211113	32.859497	24.918804	29.254832	33.548865	0.82852494	0.143540423	0.588969956	-0.337441018
ENSG00000102178	UBL4A	17.828294	16.377362	15.5624	14.849751	14.03681	14.204268	11.361443	11.733581	13.967315	0.553364918	-0.064369152	0.000649904	-0.337415488
ENSG00000111237	VPS29	28.173921	28.272022	28.196152	31.905387	32.966594	30.541822	22.896322	21.352414	19.589064	0.000759497	0.243993664	0.000239008	-0.337387207
ENSG00000106829	TLE4	5.118054	4.573002	4.78383	5.205518	4.833045	7.205927	3.031733	3.658341	3.827094	0.112596934	0.185829535	0.005569223	-0.337259433
ENSG00000131781	FMO5	7.277116	6.478333	6.07343	5.446139	6.453678	5.331192	4.841892	4.314677	5.377771	0.709693702	-0.064907986	0.011146192	-0.33722874
ENSG00000177873	ZNF619	1.087289	0.887551	0.979244	1.371115	1.460362	1.61429	0.834963	0.766068	0.460855	0.00437946	0.595718056	0.239100299	-0.337038543
ENSG00000160345	C9orf116	3.207127	2.779569	2.788143	2.072156	2.861591	2.558089	2.728097	1.698939	1.991354	0.95047057	-0.027661619	0.29834133	-0.33674975
ENSG00000147394	ZNF185	0.507201	0.360813	0.260312	0.327508	0.318325	0.20102	0.265262	0.255218	0.202846	0.543428443	-0.279607854	0.403085634	-0.336515943
ENSG00000044524	EPHA3	0.833269	0.954905	1.138717	0.216442	0.218215	0.255852	0.618197	0.694171	0.879906	9.75E-17	-1.918249883	0.132037118	-0.336439631
ENSG00000147439	BIN3	5.164331	4.032029	3.318282	3.093368	3.565162	3.796369	2.724373	2.822226	3.550558	0.755712762	-0.08186844	0.091892316	-0.336278505
ENSG00000124813	RUNX2	0.745032	0.9368	0.788226	1.484944	1.246519	1.111619	0.565868	0.537589	0.592572	0.01798043	0.475560796	0.131774018	-0.336209191
ENSG00000168214	RBPJ	18.54479	20.285238	20.31126	18.382016	17.630944	17.224417	14.949088	15.047882	18.827608	0.785291323	-0.026732256	0.00000549	-0.336042762
NewGene 3406	--	1.86239	1.688542	2.516655	2.023344	1.998573	2.000099	1.617628	1.405055	1.56929	0.004424055	0.341196547	0.014963336	-0.336010673
ENSG00000101346	POFUT1	33.837231	33.015164	32.055288	30.555594	30.088455	29.919632	24.544066	23.011616	26.868733	0.77527327	0.021971369	0.000000139	-0.335947715
ENSG00000164946	FREM1	2.470826	2.122816	3.049955	0.522387	0.661799	0.554224	1.661219	1.585596	1.915952	1.52E-37	-1.790959611	0.007836436	-0.335851281
ENSG00000198890	PRMT6	16.489849	16.189495	16.8067	14.0114	13.780192	15.463064	12.238503	11.858469	12.676404	0.681906578	-0.045050015	0.0000247	-0.335788379
ENSG00000070831	CDC42	60.643388	83.395529	76.07412	76.298251	65.340336	60.431762	51.416459	50.132445	66.987293	0.921032889	0.029872614	0.123724565	-0.335482286
ENSG00000143179	UCK2	31.639931	31.663655	33.088909	28.453693	26.523437	25.064181	37.111087	34.255174	30.349656	0.493077097	-0.062159721	0.000002	-0.335425457
ENSG00000059378	PARP12	9.441539	8.206127	8.490412	32.823085	27.964069	30.980517	7.299555	5.986654	6.672273	7.7E-185	1.92589587	0.000539313	-0.335347585
ENSG00000115486	GGCX	19.416718	18.880461	17.581936	20.795919	20.981019	21.469725	13.943468	13.395999	13.22842	4.27E-09	0.333294966	0.000000772	-0.335282497
ENSG00000130751	NPAS1	2.893018	3.438592	3.419825	1.249204	0.912689	1.067258	2.230962	2.116715	3.174216	3.78E-16	-1.652841279	0.088234204	-0.335280901
ENSG00000129422	MTUS1	12.796955	15.074924	13.013731	19.674474	19.187377	18.413096	11.070658	10.037585	10.390209	1.12E-20	0.567913046	0.000029	-0.335263229
ENSG00000185033	SEMA4B	5.643229	3.942881	3.400483	3.222231	3.286639	3.594274	3.210058	2.930804	3.426225	0.286287794	-0.196944015	0.042634225	-0.335259642
ENSG00000197045	GMFB	24.698368	26.887904	27.242655	23.420586	24.026859	23.766535	18.604139	16.569527	19.758889	0.889254673	0.01361533	0.00000372	-0.335225023
ENSG00000126773	PCNX4	16.917832	16.088368	21.100952	8.336718	8.147614	6.718589	15.097813	13.867992	13.47864	3.68E-57	-1.011689793	0.0000275	-0.334765256
ENSG00000104517	UBR5	14.557357	15.253705	14.974453	17.966209	16.803568	16.566985	11.172551	10.434582	11.536179	1.3E-10	0.354304288	0.000000173	-0.334640334
ENSG00000166348	USP54	2.985891	2.755143	2.318648	1.891827	1.932716	1.786825	2.303106	1.701249	1.925999	0.013848209	-0.321375953	0.016529199	-0.334549082
NewGene 6288	--	12.165156	11.146226	9.882561	11.010077	11.759749	10.791809	7.674942	8.813301	8.590797	0.320849649	0.129560735	0.006902948	-0.334418112
ENSG00000169136	ATF5	7.966729	6.81445	6.737035	7.425479	7.127056	7.447486	4.958243	5.569355	5.596142	0.136397733	0.193598717	0.013393162	-0.334329953
ENSG00000101096	NFATC2	4.580651	3.96966	4.445013	5.485511	5.109863	4.869557	3.548406	2.761715	3.427132	0.00000562	0.394307409	0.002659972	-0.334295279
ENSG00000136147	PHF11	2.348358	2.826078	2.396883	4.926302	5.362043	5.054473	1.814169	1.899299	1.855006	2.49E-11	1.128314706	0.175722227	-0.334294323
ENSG00000204977	TRIM13	4.231572	3.29547	3.962211	3.027966	2.382717	2.992263	3.447791	3.000669	2.817202	0.558734397	-0.102542524	0.016964427	-0.334001651
ENSG00000099889	ARVCF	6.790863	6.145048	5.917268	4.39971	4.643736	4.537331	4.795014	4.122004	5.373904	0.001143688	-0.343643165	0.003977942	-0.333773256
ENSG00000153944	MSI2	16.674195	17.301569	17.5806	15.588436	15.726777	16.392576	13.794186	12.596466	13.64455	0.676662538	0.035527028	0.00000141	-0.333573641
ENSG00000165474	GJB2	1.469236	1.654352	1.759238	1.189573	1.521844	1.328272	1.327604	0.964091	1.329245	0.689714147	-0.120055724	0.175290299	-0.333567784
ENSG00000172534	HCFC1	20.046064	19.698679	18.223567	20.155945	18.486173	19.86263	14.148582	12.576391	16.30129	0.008952412	0.176001424	0.0000437	-0.333524656
ENSG00000156313	RPGR	2.821759	2.586861	2.897343	2.141944	1.62385	1.941448	1.577971	1.59913	2.107473	0.088610504	-0.298905549	0.064058349	-0.333395417
ENSG00000198626	RYR2	0.232412	0.315037	0.35035	0.244342	0.230465	0.275549	0.244622	0.178254	0.246237	0.714379517	-0.105856217	0.164981195	-0.333114759
ENSG00000171813	PWWP2B	4.192114	4.162833	3.437945	3.426174	3.54554	3.622167	2.500588	2.764088	3.668866	0.91583762	-0.022929798	0.048165648	-0.333007907
ENSG00000186020	ZNF529	2.78167	3.136646	4.430103	3.072646	2.828518	2.622424	2.621687	2.457576	2.457611	0.528659741	-0.118544233	0.028838076	-0.332892539
ENSG00000145819	ARHGAP26	2.890386	2.574696	3.513832	1.808912	1.739871	1.718248	3.46215	2.651589	2.093247	0.002884249	-0.338253405	0.011620236	-0.332882708
ENSG00000112379	ARFGEF3	6.856576	6.077879	7.11787	4.60951	4.289399	4.556162	4.550252	4.46833	5.959742	1.85E-09	-0.428449177	0.000841113	-0.332832051

Gene ID	Gene name	Ctrl-1 FPKM	Ctrl-2 FPKM	Ctrl-3 FPKM	IFNγ-1 FPKM	IFNγ-2 FPKM	IFNγ-3 FPKM	Sora-1 FPKM	Sora-2 FPKM	Sora-3 FPKM	Ctrl vs IFNγ DESeq2 FDR	Ctrl vs IFNγ DESeq2 log2FC	Ctrl vs Sora DESeq2 FDR	Ctrl vs Sora DESeq2 log2FC
ENSG00000077238	IL4R	27.144621	27.055654	25.814677	25.377135	24.497245	24.954543	22.450703	18.969334	22.748844	0.670377136	-0.034998442	0.00000285	-0.332716034
NewGene_4954	--	0.410278	0.841566	0.78469	0.494586	0.362675	0.477475	0.653522	0.508799	0.244067	--	--	0.533934388	-0.332609997
ENSG00000196275	GTF2IRD2	1.758997	1.684012	0.886525	0.832055	0.620516	1.155381	0.669877	1.239782	1.003283	0.612402245	-0.159278025	0.199239473	-0.332112057
ENSG00000197479	PCDHB11	0.948439	1.000902	0.77928	0.764357	0.718097	0.554924	0.772649	0.690106	0.621479	0.242400632	-0.31314575	0.180011984	-0.331897823
ENSG00000116985	BMP8B	0.223883	0.292171	0.285344	0.162454	0.182467	0.245265	0.149291	0.206599	0.243842	0.546672065	-0.294638986	0.437999377	-0.331667675
ENSG00000125384	PTGER2	2.475021	2.671747	2.830756	0.394916	0.30055	0.327423	1.549534	2.532442	2.210771	9.47E-30	-2.767250349	0.119945749	-0.331649932
ENSG00000069943	PIGB	5.753485	5.400342	6.26846	4.337171	4.825359	3.817806	3.939206	4.038682	3.803347	0.45064429	-0.134978931	0.021349424	-0.331601618
NewGene_4213	--	1.929747	0.915324	1.796124	1.035976	1.202522	1.332797	0.956399	1.313966	1.160768	0.59536914	-0.228792984	0.374927947	-0.331384334
NewGene_3011	--	3.622689	4.426451	3.872662	2.414287	2.482691	2.185364	3.515943	2.933778	3.083989	6.66E-10	-0.628486406	0.003179351	-0.331234907
ENSG00000168461	RAB31	2.410731	2.704333	2.926034	2.577334	2.31551	2.348925	2.276487	1.838323	1.888917	0.984350144	-0.004338748	0.034314695	-0.331230805
NewGene_1008	--	3.268186	3.347546	3.839685	4.001943	3.596932	4.317793	3.08736	2.572393	2.749878	0.004969751	0.344129005	0.01503244	-0.331219971
ENSG00000119402	FBXW2	17.399126	18.254756	18.599956	19.321333	19.113849	18.108835	14.087066	12.888833	14.399234	0.000199736	0.212343258	0.000000139	-0.331033518
ENSG00000154642	C21orf91	3.517134	3.86786	4.161878	3.134558	3.208163	3.410293	2.637132	2.79767	3.099827	0.601176579	-0.075197918	0.005208836	-0.330993129
ENSG00000158122	PRXL2C	2.440562	2.476843	2.854528	2.14681	2.17937	2.610395	1.685392	2.170305	1.986752	0.881170508	-0.037691028	0.076475166	-0.330891555
ENSG00000127914	AKAP9	6.785385	8.240486	7.971459	6.263867	7.053971	6.785215	5.891723	5.36905	6.012529	0.686469238	0.049789255	0.000164441	-0.330870907
ENSG00000158615	PPP1R15B	21.722363	22.997961	24.860624	25.960527	25.035406	24.197973	17.63825	16.15308	18.119173	0.0000694	0.259093738	0.00000496	-0.330857577
ENSG00000109511	ANXA10	1.570192	1.515739	1.241884	2.728622	2.579359	3.123221	0.972739	1.029725	1.225188	0.00000151	1.103528411	0.314218102	-0.330649121
ENSG00000162976	PQLC3	1.402206	1.624986	1.236273	1.463257	1.364421	1.238914	1.193858	0.862155	1.03427	0.763724685	0.107509813	0.280762709	-0.330488047
ENSG00000153558	FBXL2	0.418571	0.565978	0.309049	0.387028	0.27365	0.216646	0.33516	0.345144	0.450426	0.23825195	-0.506379171	0.416212287	-0.33047793
ENSG00000160117	ANKLE1	0.314722	0.448987	0.386097	0.285779	0.380903	0.421109	0.217785	0.294724	0.364345	0.920936815	0.057833923	0.453030167	-0.330449274
ENSG00000137492	THAP12	13.398722	13.876579	14.552667	12.695149	13.100523	13.160974	10.557317	9.768305	10.55266	0.655825683	0.046734775	0.0000986	-0.330359841
ENSG00000172936	MYD88	9.722388	8.816768	8.742199	16.369918	15.362816	16.71432	8.099343	6.9199	6.429902	3.5E-36	0.951177882	0.001800786	-0.330214873
ENSG00000100592	DAAM1	7.448995	7.877566	8.587625	5.578453	6.227894	5.51631	5.795558	5.488938	6.632679	0.000168847	-0.361025968	0.000380009	-0.329968349
ENSG00000068024	HDAC4	1.592434	1.664	1.555871	2.017577	2.04337	2.112115	1.198962	1.068379	1.351825	0.00000102	0.505467262	0.013055719	-0.329831011
NewGene_1771	--	0.909866	0.930539	0.745383	0.712848	0.713992	0.68859	0.645559	0.495059	0.571591	0.716356324	0.122308406	0.250443853	-0.329735526
ENSG00000154743	TSEN2	15.133171	15.208471	16.733571	11.901113	10.749632	10.762539	10.203659	11.502221	11.813562	0.001486365	-0.296651838	0.001266605	-0.329614718
ENSG00000138246	DNAJC13	10.954097	10.917366	10.11329	10.277863	11.013066	10.296824	7.721385	7.55709	8.570544	0.097152722	0.128012879	0.00000635	-0.329605735
ENSG00000122863	CHST3	3.814276	4.190894	4.256781	1.551758	1.890359	1.610114	3.055706	2.749727	3.357086	3.85E-27	-1.130095446	0.001140752	-0.329496658
ENSG00000107938	EDRF1	6.002108	6.084417	6.478843	5.63445	5.015029	5.006602	4.64768	4.277457	5.32807	0.242374434	-0.129208211	0.001843935	-0.329459624
ENSG00000117308	GALE	32.714346	29.078363	24.947245	20.767455	20.442887	21.938615	21.594365	20.053898	20.488853	0.011373585	-0.239534198	0.000512728	-0.329214028
ENSG00000003402	CFLAR	13.200896	11.762642	9.898428	11.427467	11.913215	11.820843	11.219941	11.52948	10.570568	0.000372423	0.235965901	0.0000141	-0.329107197
ENSG00000129625	REEP5	14.055252	12.455146	13.586378	12.857755	12.38588	11.932851	9.045342	9.662711	10.377446	0.295443448	0.097493693	0.000231963	-0.329000397
ENSG00000082805	ERC1	4.969876	4.834718	4.361751	4.421336	4.615064	4.417558	3.586474	3.361958	3.977958	0.47200878	0.072376764	0.000212655	-0.328713495
ENSG00000156875	MFSD14A	20.95153	20.233164	21.5755	24.923635	22.065081	22.035227	14.833024	16.138956	15.854173	0.0000831	0.283297458	0.0000492	-0.328658389
ENSG00000196700	ZNF512B	10.404392	9.692901	9.173589	9.964588	9.534987	9.799988	6.844833	6.727771	8.337259	0.063201822	0.147970932	0.000308851	-0.328607152
ENSG00000068878	PSME4	25.369995	26.588495	27.509756	21.556574	21.621075	20.312391	19.567776	18.588343	21.250412	0.003359082	-0.176472219	0.000000207	-0.328498456
ENSG00000272674	PCDHB16	3.14028	3.433945	3.478965	2.739455	2.699205	2.460852	2.488622	2.388129	2.747761	0.111238615	-0.199410049	0.005161703	-0.328348534
ENSG00000172794	RAB37	3.088749	2.630669	1.911069	2.661181	2.89634	2.471371	2.06176	1.651413	2.070814	0.270902774	0.241445436	0.124247538	-0.328324677
ENSG00000235106	BRD3OS	0.53424	0.85638	0.625833	0.538007	0.341248	0.472014	0.514026	0.564995	0.484771	0.144639048	-0.436855334	0.211821027	-0.328113711
ENSG00000206560	ANKRD28	16.699737	17.669667	18.148073	16.19399	16.239125	15.341772	14.514778	11.981101	14.07541	0.871669445	-0.014955313	0.00000518	-0.328024315
NewGene_1996	--	3.573561	3.784405	4.534574	3.739332	3.371963	3.199322	3.360717	3.425447	4.520296	0.576668469	0.066149358	0.001079177	-0.328019104
ENSG00000156876	SASS6	5.372596	6.691112	6.681223	5.836206	5.098949	5.404451	4.176815	4.490302	5.357394	0.741254352	-0.050808813	0.009470017	-0.327931537
ENSG00000277443	MARCKS	66.593956	74.119751	70.97982	59.170597	56.139278	62.329521	53.113167	50.92754	54.130066	0.122789326	-0.105333956	8.24E-08	-0.327873574
ENSG00000170482	SLC23A1	0.637271	0.572594	0.571422	0.944806	0.798127	0.534824	0.612223	0.435525	0.275972	0.22159189	0.468929653	0.457461487	-0.327635257
ENSG00000257591	ZNF625	2.653933	1.954378	1.530928	2.174001	2.361911	1.310971	0.791611	1.964802	1.406204	0.690404347	0.145616213	0.335018308	-0.327617721
ENSG00000114023	FAM162A	14.578107	12.20923	11.660126	13.135486	13.162523	11.80239	9.660864	10.210569	8.56573	0.576319153	0.078653628	0.010149322	-0.32715755
ENSG00000163104	SMARCAD1	14.176805	14.618646	15.861807	14.20759	12.893515	13.098372	11.150948	9.939985	12.554265	0.945558401	-0.007780004	0.000124253	-0.327109033
ENSG00000205236	AC105052.1	1.668369	1.988137	2.581136	1.834946	0	0	1.74482	1.588906	1.310562	0.702609262	-1.640550664	0.129145217	-0.326979093
ENSG00000139438	FAM222A	1.242509	1.272817	1.075548	1.019023	1.02475	1.249932	0.917996	0.83702	0.926839	0.930787487	0.02909199	0.159368001	-0.326875439
ENSG00000033122	LRRC7	1.144755	1.257848	1.052016	1.09744	0.992336	0.948024	1.268352	0.719687	1.221155	0.067511757	-0.509564655	0.243710671	-0.326583602
ENSG00000128694	OSGEPL1	5.166569	3.805897	4.775101	2.748023	3.041446	3.408481	3.779218	3.215801	3.15583	0.023216481	-0.414716486	0.073440471	-0.326499638
ENSG00000167985	SDHAF2	32.735276	33.892714	28.019129	33.898353	25.464259	27.079463	21.503714	25.499407	25.47847	0.821895035	-0.025472656	0.00036635	-0.326477229
ENSG00000164823	OSGIN2	8.173594	8.412834	9.636398	9.793026	9.581946	8.88287	6.623262	6.013842	7.040031	0.010093743	0.24184866	0.000798511	-0.326325698
ENSG00000114331	ACAP2	5.890546	6.7191	6.800081	7.154001	6.177334	6.437008	4.863958	4.471795	5.200472	0.081066338	0.160653536	0.000279206	-0.326285073
NewGene_4621	--	1.823469	1.507857	1.762139	1.179017	0.976364	1.077912	0.955104	1.56841	1.615134	0.032688652	-0.432215656	0.106338743	-0.326203299
NewGene_434	--	0.470529	0.339936	0.422047	0.264035	0.436797	0.212645	0.283315	0.122826	0.43997	0.659374201	-0.247467884	0.536474128	-0.326145546
ENSG00000164040	PGRMC2	25.942437	30.202062	29.035813	27.741026	26.385497	32.357238	19.314949	22.537722	22.500032	0.001427832	0.227384322	0.0000147	-0.325878989
ENSG00000138434	ITPRID2	34.432415	34.367158	34.816832	30.714563	29.794462	29.602908	26.571796	24.469982	27.329705	0.331457387	-0.059577962	0.000000013	-0.325599724
ENSG00000105711	SCN1B	1.773669	2.253029	1.448564	1.994552	1.762959	1.73394	1.115137	1.440688	1.466393	0.270464158	0.279702468	0.214346166	-0.325392215
ENSG00000203668	CHML	9.09514	10.054235	10.203517	12.115941	10.877334	11.617443	7.559789	6.724033	7.688946	9.29E-09	0.386406625	0.0000349	-0.325319418
ENSG00000164045	CDC25A	8.089024	8.300692	9.386704	7.734511	6.730198	7.025062	6.064471	6.460403	6.764734	0.262802336	-0.124145674	0.001341744	-0.325144932
ENSG00000160226	CFAP410	15.473472	12.157684	10.449978	10.916742	9.867733	11.636021	9.640943	9.110815	10.295555	0.487961229	-0.11188168	0.013253399	-0.325070777
NewGene_3330	--	1.973055	1.740764	1.866301	1.335847	1.727168	1.291235	1.464602	1.43551	1.353584	0.857676155	-0.045078594	0.075863693	-0.324828817
ENSG00000274180	NATD1	3.1088	2.764877	1.980711	2.120417	1.957654	1.939197	2.044667	1.887552	1.952917	0.19363109	-0.239863555	0.058604618	-0.324799787
ENSG00000268104	SLC6A14	16.290989	16.701752	23.408236	6.998943	8.904743	7.718541	11.972123	13.997556	16.328234	0.000001	-1.105917435	0.194810797	-0.324519793
ENSG00000113645	WWC1	9.581526	9.343515	8.96889	6.3732	5.807125	5.623083	7.230577	6.127283	7.676853	4.46E-12	-0.49388829	0.000298887	-0.32438242
ENSG00000117114	ADGRL2	35.654546	37.646716	34.44679	27.830159	26.340673	26.205121	24.476989	23.533564	28.171916	0.000680549	-0.182920666	0.000000112	-0.324044711
ENSG00000031003	FAM13B	5.62268	5.535684	5.507911	4.313522	4.294946	4.225248	4.704844	3.672892	4.222134	0.016840051	-0.217063336	0.002449877	-0.324042567
ENSG00000144485	HES6	13.182777	10.857952	10.097285	13.19382	13.181274	14.028028	8.476641	9.116095	8.518189	0.002828841	0.361919482	0.019216973	-0.323698571
ENSG00000010404	IDS	1.400468	1.473588	1.426463	1.243032	1.067971	1.182274	1.158943	1.118175	1.10911	0.136565284	-0.238130486	0.033260407	-0.323687276
ENSG00000138380	CARF	0.707967	0.815823	0.691243	0.747148	0.761888	0.654739	0.524666	0.468189	0.599799	0.621524903	0.148011856	0.189069275	-0.323580435
ENSG00000111961	SASH1	3.572037	3.233402	3.480773	3.494987	3.32139	3.353289	2.896362	2.240327	3.229049	0.196888317	0.131668691	0.008109404	-0.323437718
ENSG00000189376	C8orf76	14.524055	12.39301	12.78896	12.750484	13.740641	13.479243	10.594347	10.244521	8.84175	0.220677075	0.156237415	0.015244179	-0.32338388
ENSG00000183762	KREMEN1	1.681712	1.51903	1.476268	1.281949	1.424624	1.394446	1.298895	1.006454	1.291592	0.842906744	-0.041020211	0.045404563	-0.323274507

Gene ID	Gene name	Ctrl-1 FPKM	Ctrl-2 FPKM	Ctrl-3 FPKM	IFNγ-1 FPKM	IFNγ-2 FPKM	IFNγ-3 FPKM	Sora-1 FPKM	Sora-2 FPKM	Sora-3 FPKM	Ctrl vs IFNγ DESeq2 FDR	Ctrl vs IFNγ DESeq2 log2FC	Ctrl vs Sora DESeq2 FDR	Ctrl vs Sora DESeq2 log2FC
ENSG00000129473	BCL2L2	10.621885	11.451053	10.034678	10.889408	10.543905	9.690379	8.506297	7.708738	7.981682	0.209284979	0.121108898	0.000536399	-0.323109285
ENSG00000171867	PRNP	5.519628	5.811186	6.119165	5.240979	3.6123	4.643237	3.659887	4.553451	4.615803	0.25492522	-0.184793273	0.016013796	-0.32298139
ENSG00000204104	TRAF3IP1	3.020029	3.139956	3.062475	3.235919	3.369228	3.102766	2.452597	1.885594	2.569638	0.073240154	0.226842718	0.025902349	-0.322929171
ENSG00000120942	UBIAD1	12.014325	14.047968	13.903202	11.381802	10.318771	10.80657	9.57057	9.109913	11.117848	0.149648742	-0.140754277	0.001003706	-0.322795585
ENSG00000145545	SRD5A1	2.780468	2.995725	2.957667	2.308718	2.129984	2.062706	2.083119	2.190679	2.189227	0.012085883	-0.27200915	0.002581825	-0.322786325
ENSG00000119844	AFTPH	15.749725	18.323675	18.884456	20.096969	16.99006	18.268408	12.600382	14.767073	12.663802	0.005913908	0.191306475	0.0000106	-0.322749424
ENSG00000152582	SPEF2	0.61077	0.594106	0.547506	0.410402	0.313797	0.295936	0.485518	0.455893	0.425293	0.070547782	-0.536944338	0.249569995	-0.322719212
ENSG00000093183	SEC22C	7.905929	7.028263	7.726005	7.181422	7.120625	6.99495	6.254085	5.839907	5.513763	0.615564374	0.057982888	0.000648635	-0.322607675
ENSG00000105058	FAM32A	75.471652	66.886461	65.281065	63.479151	65.050438	63.985033	50.867509	50.85431	54.784404	0.804860179	0.025549326	0.0000332	-0.322560193
ENSG00000174197	MGA	5.837374	6.449421	6.145465	6.841445	5.57978	6.264997	4.962922	4.479834	5.28788	0.050081675	0.148254388	0.0000197	-0.322449392
ENSG00000160404	TOR2A	6.30604	4.154224	4.974072	3.869292	4.002444	3.929927	3.285971	4.161817	3.33703	0.502653138	-0.141329564	0.085371142	-0.32240463
ENSG00000180535	BHLHA15	8.618859	8.054576	6.764373	8.72531	8.175547	8.719186	6.261762	5.229743	6.171771	0.135280474	0.275823555	0.096736512	-0.322378924
ENSG00000280433	FP565260.6	4.684442	5.313056	6.655707	5.971421	5.254523	5.740454	4.655254	3.925701	3.889014	0.23583964	0.174815053	0.028777084	-0.322360602
ENSG00000187824	TMEM220	3.935056	3.520901	3.557486	4.139698	3.922577	3.720298	2.822754	2.198274	2.783926	0.089110664	0.241175089	0.031553661	-0.322334355
ENSG00000157933	SKI	18.834652	18.553864	18.793427	14.47878	13.990964	15.019502	14.219417	12.124182	15.914663	0.000247163	-0.221691658	0.0000766	-0.322188355
ENSG00000071655	MBD3	76.227513	86.179433	77.555772	73.918883	76.529373	75.64861	48.440224	47.226266	61.937423	0.517163014	-0.056238713	0.000158261	-0.321953134
ENSG00000153786	ZDHHC7	17.532732	15.937583	17.850058	11.411517	11.493982	10.779282	13.05334	11.930008	14.067442	1.28E-09	-0.454721512	0.000156074	-0.321847412
ENSG00000082516	GEMIN5	7.566721	7.428167	7.182626	5.862019	5.420158	5.598135	5.529094	5.450627	5.659689	0.002156929	-0.247005917	0.0000998	-0.321723093
ENSG00000104343	UBE2W	3.267352	2.96927	3.174164	2.890495	2.705642	2.733717	2.321977	2.78759	2.890864	0.329708831	0.115821215	0.003450838	-0.321664589
ENSG00000178821	TMEM52	2.147436	1.568588	1.289691	1.568574	1.35201	2.089789	1.706879	1.049051	0.867749	0.658759692	0.192865343	0.443463844	-0.321647413
ENSG00000078804	TP53INP2	7.097184	6.233396	6.21484	6.127619	6.256588	5.92127	4.792149	4.506464	5.356099	0.73812765	0.044276222	0.002032	-0.321619168
ENSG00000107263	RAPGEF1	9.39478	7.772282	7.193984	8.476586	8.231935	8.236478	6.076158	5.456576	6.924613	0.113196024	0.170162999	0.003297284	-0.321134724
ENSG00000010932	FMO1	1.023514	0.7022	0.466563	0.219131	0.213896	0.146657	0.66582	0.577607	0.356888	0.0000124	-1.808745777	0.416255813	-0.321125415
ENSG00000119772	DNMT3A	17.447346	13.578514	14.669446	12.790068	15.951036	10.461272	10.280175	12.235673	11.513576	0.004253393	0.186597143	0.00000333	-0.321081914
ENSG00000147869	CER1	3.11492	3.327703	3.632526	0.219625	0.095625	0.09209	2.304058	2.64902	2.578806	6.44E-28	-4.337880635	0.163463994	-0.321037573
ENSG00000112159	MDN1	5.926435	6.089532	6.309886	5.585371	4.838687	4.981807	4.300053	4.387639	5.088994	0.169103788	-0.104000925	0.0000108	-0.320731947
ENSG00000173275	ZNF449	1.046908	1.245837	1.057341	1.314075	1.300087	1.115856	0.936816	0.828033	0.57965	0.189892489	0.300799904	0.227883466	-0.320684208
ENSG00000204524	ZNF805	1.365782	1.297547	0.643482	0.912727	1.197248	1.094918	1.111272	0.577834	0.876172	0.853376962	0.084751827	0.381838444	-0.320679393
ENSG00000165671	NSD1	3.310862	3.39415	3.420899	3.76207	3.486957	3.342932	3.097106	2.486307	2.821787	0.422593592	0.084993362	0.000893128	-0.320645472
NewGene_566	--	4.021309	3.92216	4.188295	3.738898	3.737737	3.60029	3.06225	2.746256	3.32813	0.939538182	0.014908203	0.021086547	-0.320212341
ENSG00000069702	TGFBR3	0.83713	0.739683	0.615649	0.49386	0.641422	0.427029	0.374688	0.562512	0.719741	0.186246237	-0.343327488	0.22849193	-0.320114442
ENSG00000100767	PAPLN	1.093873	1.189364	1.405478	1.006722	0.749522	0.685848	1.131695	0.766172	0.774584	0.035792404	-0.405229173	0.107668886	-0.319814676
NewGene_4527	--	1.650558	2.328358	2.303313	2.114935	1.437526	1.459107	1.656597	1.316745	1.278301	0.722415077	-0.081361723	0.052722442	-0.319697228
ENSG00000165895	ARHGAP42	1.507874	2.208582	1.612861	1.996523	3.971698	2.053747	1.201691	0.962954	1.129584	0.000034	0.648452571	0.087731677	-0.319576069
ENSG00000188938	FAM120AOS	14.858708	13.966063	16.372602	13.493709	13.913806	14.075461	12.420496	12.388718	12.874622	0.452056817	0.076554769	0.000199571	-0.319535909
ENSG00000169507	SLC38A11	2.734504	2.758682	2.645094	2.240197	2.348456	2.289949	2.0935	2.078405	2.310765	0.559575166	-0.104830842	0.031277371	-0.319506723
ENSG00000108389	MTMR4	17.596253	17.22488	17.161733	20.876758	20.229263	18.415013	13.976378	11.848035	13.979407	1.23E-08	0.34110352	0.000015	-0.319490248
ENSG00000129204	USP6	0.138715	0.227708	0.147111	0.203972	0.230124	0.207237	0.126716	0.093971	0.165716	0.187387159	0.499287806	0.465084879	-0.319296676
ENSG00000142867	BCL10	3.442597	3.639842	3.787406	3.324681	2.964591	3.204676	3.328234	2.813065	2.33688	0.730017908	-0.064692877	0.051803784	-0.319259428
ENSG00000156398	SFXN2	2.138738	2.706388	2.385545	2.265473	1.736851	2.02618	2.042738	1.83004	1.783348	0.637564013	-0.07876599	0.012796097	-0.319191389
ENSG00000164944	VIRMA	15.305201	15.325195	15.303356	15.059219	14.733595	14.668555	12.097419	10.327324	12.137861	0.217139445	0.082885844	0.00000821	-0.319039506
ENSG00000278053	DDX52	12.304847	11.584099	12.460585	10.416778	10.338677	9.272617	9.243133	8.959589	9.721043	0.01722787	-0.177982158	0.0000071	-0.31890482
NewGene_316	--	0.962135	0.941459	1.163735	1.008651	0.694218	1.220762	0.841985	0.819338	0.720795	0.997888811	-0.001753118	0.311911787	-0.318685522
ENSG00000149177	PTPRJ	3.094742	2.980526	2.860737	2.685495	2.571574	2.985253	2.606352	2.286089	1.966571	0.980052589	-0.00424149	0.004277627	-0.318674296
ENSG00000099622	CIRBP	102.801301	98.582771	100.703318	100.634368	92.376994	99.898345	82.020635	76.468703	75.648251	0.179408808	0.090343194	0.00000964	-0.3186665
ENSG00000138942	RNF185	23.379463	21.796645	22.086299	19.135927	18.640007	18.873096	16.376874	16.789221	17.645408	0.194043638	-0.09905611	0.0000179	-0.318596775
NewGene_3132	--	3.368382	3.453856	2.925012	2.938551	2.565753	2.595108	2.244023	2.605874	2.098209	0.026375746	-0.265895776	0.009392642	-0.31850531
ENSG00000176155	CCDC57	5.394952	5.535524	5.206241	3.725103	3.486922	4.42305	4.122048	3.260471	3.564865	0.155445191	-0.213483063	0.015019392	-0.318483747
ENSG00000118707	TGIF2	43.645893	40.221368	43.431131	35.341386	35.310809	34.317473	32.044765	28.790665	35.568877	0.030762938	-0.139531528	0.0000127	-0.318396666
NewGene_5575	--	2.726286	2.992935	3.00649	3.154963	2.420539	2.791333	2.25064	2.122926	3.064459	0.263585217	0.134810253	0.006625313	-0.318301264
ENSG00000117586	TNFSF4	8.37051	8.659992	7.846349	4.496797	4.695467	4.488393	5.24529	5.791943	7.736654	4.05E-15	-0.714603225	0.012963368	-0.31800393
ENSG00000237172	B3GNT9	2.586522	2.671665	2.745118	2.881944	2.696977	2.478637	1.991689	1.906683	2.125178	0.373498811	0.157560619	0.049938891	-0.31795279
ENSG00000171806	METTL18	8.379276	7.968123	8.000045	6.965154	8.289269	7.861651	6.909806	5.552274	6.440654	0.661342354	0.077902103	0.032762668	-0.317724233
ENSG00000136802	LRRC8A	19.868752	17.761455	17.702652	12.038179	12.016844	12.885586	12.947653	12.138961	16.743264	2.51E-09	-0.433179702	0.001663997	-0.317524639
ENSG00000081721	DUSP12	26.859798	28.493164	30.966373	25.676442	23.20905	25.420204	22.12884	21.01896	22.509016	0.546434713	-0.069983364	0.000678989	-0.317492314
ENSG00000166813	KIF7	2.758833	2.663403	2.773075	2.365062	2.130902	2.515088	2.10445	1.861395	2.224342	0.643246801	-0.077622715	0.018587836	-0.317336972
ENSG00000123473	STIL	8.40097	8.452977	8.61056	9.146486	7.437391	8.242663	7.231199	6.163019	6.513129	0.562509369	0.063838154	0.000292234	-0.317079993
ENSG00000101138	CSTF1	13.003554	14.939617	13.853337	12.196289	13.587638	9.937425	10.607189	13.783146	12.356143	0.255379513	-0.09959005	0.0000661	-0.31707216
ENSG00000150401	DCUN1D2	14.128535	16.100555	14.435695	12.765488	12.860657	12.044449	10.631428	10.053685	11.328138	0.21659565	-0.113652529	0.000308851	-0.316870955
ENSG00000111540	RAB5B	16.921311	16.290176	16.522816	15.857716	17.203826	16.646788	12.186515	12.171621	12.749756	0.032050059	0.150579588	0.00000356	-0.316752215
ENSG00000185070	FLRT2	1.209043	1.384264	1.636273	0.545539	0.443512	0.556802	1.035596	0.918101	1.176655	5.07E-33	-1.258833517	0.004625494	-0.31668395
ENSG00000108021	FAM208B	11.003012	11.987182	12.640166	13.979606	12.84848	12.668434	8.338494	8.877568	9.784921	0.0000144	0.293460813	0.00008	-0.316497508
ENSG00000074603	DPP8	10.681896	9.694844	11.863489	11.686125	11.708038	9.26094	9.642868	7.638804	8.83277	0.069663919	0.147133939	0.000142304	-0.316343038
ENSG00000146205	ANO7	0.891717	0.816327	1.083573	0.743939	0.944447	0.916887	0.79556	0.86227	1.161692	0.871930279	-0.052086202	0.771598205	-0.315996275
ENSG00000162688	AGL	5.432352	5.707706	5.6542	5.53241	5.265431	5.482001	4.079076	3.898686	4.683745	0.22912593	0.103294307	0.000284485	-0.315764599
ENSG00000120800	UTP20	10.738134	11.073659	10.935628	8.405122	6.858037	7.023569	7.785635	7.73634	9.188806	2.92E-08	-0.409355319	0.0000161	-0.315539114
ENSG00000177706	FAM20C	18.943014	18.140594	15.407911	18.492891	18.902758	20.05179	13.501242	11.778392	14.360354	0.002279096	0.277720896	0.001588406	-0.315341198
ENSG00000128849	CGNL1	23.93272	23.437994	21.407242	16.923492	17.530331	17.899466	17.285297	16.04318	18.571249	0.000138781	-0.245878307	0.00000487	-0.315313671
ENSG00000136280	CCM2	22.610491	22.984932	20.54868	21.274726	20.248351	20.476164	14.754245	16.46128	18.602302	0.531135935	0.06619851	0.001458399	-0.315275854
ENSG00000158887	MPZ	2.080329	2.007741	2.334676	0.424577	0.326771	0.312266	1.522314	1.45059	1.860661	4.65E-19	-2.425211663	0.169237205	-0.315013254
ENSG00000184588	PDE4B	0.61017	0.762534	0.864113	0.456005	0.497172	0.506036	0.44252	0.512915	0.718029	0.114377824	-0.435718011	0.259232936	-0.314865623
ENSG00000094880	CDC23	15.311118	15.387191	15.514826	12.929763	12.695838	12.318357	11.719656	11.302188	11.878214	0.11382336	-0.127281229	0.0000476	-0.314785617
ENSG00000109534	GAR1	12.409651	12.769852	14.194306	12.068412	12.581687	13.459491	10.427042	10.221306	8.979948	0.513464468	0.094810306	0.017902548	-0.314698313
ENSG00000165626	BEND7	3.680793	2.95312	3.615962	3.113781	2.592196	2.870285	3.090566	2.665571	2.840157	0.503215896	-0.106907339	0.019367728	-0.314490939

Gene ID	Gene name	Ctrl-1 FPKM	Ctrl-2 FPKM	Ctrl-3 FPKM	IFNγ-1 FPKM	IFNγ-2 FPKM	IFNγ-3 FPKM	Sora-1 FPKM	Sora-2 FPKM	Sora-3 FPKM	Ctrl vs IFNγ DESeq2 FDR	Ctrl vs IFNγ DESeq2 log2FC	Ctrl vs Sora DESeq2 FDR	Ctrl vs Sora DESeq2 log2FC
ENSG00000134247	PTGFRN	7.153652	6.169467	6.072496	5.873704	5.260033	5.588924	4.684453	4.571705	5.40305	0.566031606	-0.067481439	0.001478619	-0.314142073
NewGene_780	--	1.291619	1.016415	0.556181	0.395307	0.698058	0.5203	0.503616	0.396533	1.280826	--	--	0.579774844	-0.314116062
ENSG00000269556	TMEM185A	1.326948	1.283824	1.223123	1.208311	0.826251	0.940899	0.927696	0.822433	1.111602	0.420503266	-0.230788238	0.210335451	-0.314092847
ENSG00000063322	MED29	11.17148	10.564333	9.754992	9.515078	9.069773	9.654326	8.068384	8.13563	7.637855	0.999202724	0.000179901	0.001607639	-0.314049478
ENSG00000187650	VMAC	3.042961	2.572503	2.399898	2.255763	2.430864	2.642015	2.141574	1.725677	2.196304	0.950533099	0.017623498	0.115408041	-0.313891677
ENSG00000040731	CDH10	3.244154	3.413887	3.016668	2.072101	2.049658	1.853896	2.594319	2.215753	2.462676	0.0000809	-0.546950668	0.028201741	-0.313436951
ENSG00000152223	EPG5	5.188676	3.116321	4.818694	3.127456	2.979057	2.871184	2.474851	2.324316	2.529118	0.649152618	0.050561273	0.00041254	-0.313432398
ENSG00000226887	ERVMER34-1	2.291535	2.947477	2.835816	1.573349	1.316131	1.589892	1.830792	1.979578	2.056698	0.0000827	-0.650549555	0.060529708	-0.313257382
ENSG00000178761	FAM219B	5.370177	5.291653	6.014572	3.899264	3.468915	4.83317	5.326325	4.324816	4.920512	0.019290989	-0.342467062	0.027114559	-0.313178814
NewGene_5528	--	0.706007	0.644142	0.339187	0.415122	0.646325	0.558217	0.535681	0.273595	0.477661	0.894203329	0.085333573	0.55093407	-0.313031833
ENSG00000065989	PDE4A	1.966861	2.724189	3.131863	2.79936	2.380362	2.549025	1.913273	2.220402	1.891067	0.897697024	-0.028844232	0.059587414	-0.312944779
NewGene_2825	--	2.273016	2.044455	2.004425	1.1854	1.422829	1.175991	1.586452	1.401972	1.370936	0.005669968	-0.504618283	0.086873589	-0.312846845
NewGene_4823	--	1.995743	2.379335	1.821825	2.422845	2.196619	2.419569	1.44292	1.827114	1.733545	0.000511345	0.45876698	0.037145414	-0.31271321
ENSG00000129951	PLPPR3	2.538089	2.224233	1.892127	1.4272	1.186752	1.555108	1.78406	1.565745	1.735759	0.016575529	-0.539514489	0.145553003	-0.312653382
ENSG00000105778	AVL9	21.850544	22.213275	23.068637	21.672231	20.792239	21.179702	17.027043	16.141582	17.925784	0.341718229	0.059801169	0.00000012	-0.312581381
ENSG00000112237	CCNC	46.154303	54.415244	59.707581	50.150651	54.481101	54.304929	39.78509	40.17962	40.853464	0.019766918	0.169106193	0.0000376	-0.312571856
ENSG00000142188	TMEM50B	4.278571	4.754982	5.364636	10.332586	8.900565	8.722007	4.513405	3.534328	3.2991	3.9E-28	1.106069345	0.042111391	-0.312496465
ENSG00000120697	ALG5	37.075817	35.424423	36.138557	36.85537	36.524765	36.134773	28.655733	27.184288	26.10924	0.0447053	0.158655739	0.00041254	-0.31246379
ENSG00000018610	CXorf56	9.943557	9.512827	9.978999	8.283063	9.26119	7.710557	8.31249	7.018224	7.336628	0.005491191	-0.488941958	0.008269366	-0.311994769
ENSG00000182378	PLCXD1	13.81564	13.078243	13.123677	14.932119	16.710041	16.846235	9.01834	10.411577	10.892042	5.92E-10	0.430598675	0.00011337	-0.311826973
ENSG00000123146	ADGRE5	3.501481	3.503644	4.344632	5.426789	4.951527	5.699068	2.306054	2.903568	3.613626	1.76E-08	0.678708106	0.069502899	-0.311701652
ENSG00000123989	CHPF	14.919581	14.685965	14.309009	17.224385	17.920275	18.260329	11.733538	10.725909	11.402416	7.63E-09	0.4106479	0.000104072	-0.311666812
ENSG00000027075	PRKCH	1.452428	1.599093	1.812934	1.066703	0.733625	0.86473	1.296115	1.158926	1.265092	0.0000628	-0.794411662	0.112912469	-0.311642764
ENSG00000011347	SYT7	4.212507	3.490201	3.49215	1.723923	1.603657	1.61242	2.35381	3.336158	2.874072	1.04E-14	-0.992165419	0.041080146	-0.311044883
ENSG00000120137	PANK3	15.75953	17.880568	18.236687	18.611181	18.540703	18.318252	13.308783	12.324627	13.991969	0.00019077	0.240391517	0.0000161	-0.311013754
ENSG00000050405	LIMA1	26.014598	27.473288	32.0959	26.892937	30.017441	26.289528	20.559192	20.944155	21.982242	0.238411614	0.118665844	0.000268721	-0.310968541
ENSG00000198356	ASNA1	67.457522	61.783995	59.325714	49.707239	50.740409	49.829345	42.782685	47.643482	51.437308	0.020903522	-0.174622692	0.000330763	-0.310443949
ENSG00000139083	ETV6	1.580055	1.625969	2.223112	3.530101	3.264729	3.438011	1.401025	1.111614	1.617415	1.14E-19	1.085545028	0.08114118	-0.310342828
ENSG00000044446	PHKA2	8.630166	7.143294	6.432175	7.575634	7.318587	6.981965	5.724451	4.984251	6.119768	0.311195941	0.124478609	0.00733485	-0.310292925
ENSG00000135540	NHSL1	5.955312	5.796584	6.104959	6.74988	6.697931	6.555277	4.776822	3.986454	5.83144	0.00000216	0.332669293	0.001571979	-0.31021251
ENSG00000159592	GPBP1L1	28.960253	28.322939	28.947455	30.018988	29.724047	29.626965	21.756455	21.016992	22.246996	0.000383639	0.201182678	0.000000854	-0.310205258
ENSG00000197008	ZNF138	2.739055	4.802896	3.999916	3.274115	3.310734	2.782813	2.512541	2.612036	2.745367	0.841889215	-0.05142081	0.107108683	-0.310098276
ENSG00000170364	SETMAR	19.133487	20.636443	19.109645	13.966539	17.359253	14.793975	15.897296	15.754853	16.399013	0.050780385	-0.179248669	0.000541853	-0.309848476
ENSG00000183161	FANCF	10.691282	11.252243	10.536419	12.173133	12.96554	12.513655	8.566353	7.840703	8.162761	0.0000068	0.360733646	0.000758305	-0.309808603
ENSG00000162302	RPS6KA4	7.280355	7.842897	7.236486	7.329309	7.242095	7.516849	6.077289	5.087	5.989868	0.228292056	0.129956617	0.00353453	-0.309531891
ENSG00000164690	SHH	1.6173	1.449927	1.301756	1.601731	1.18242	1.464118	0.744666	0.983387	1.504732	0.878842428	-0.046177476	0.239124774	-0.309326195
ENSG00000183579	ZNRF3	6.024204	6.003754	6.133502	4.99491	5.079814	4.948776	4.858427	4.075372	4.913167	0.126664374	-0.130865873	0.000966266	-0.309262325
ENSG00000067208	EVI5	1.608212	1.661583	1.883784	1.85052	1.702248	1.910736	1.458828	1.072152	1.449234	0.100803465	0.215509584	0.034436346	-0.308996146
ENSG00000131944	FAAP24	3.106989	3.422999	3.644153	3.389211	2.374982	2.673425	2.97236	2.682554	2.021239	0.753713402	-0.079934326	0.12555371	-0.308981233
ENSG00000165138	ANKS6	2.801589	3.247765	2.919934	3.032157	2.357169	2.749388	2.355201	1.776915	2.874447	0.97025095	0.007124988	0.028526527	-0.308943207
ENSG00000188266	HYKK	2.055648	1.938771	2.59438	1.130813	1.014132	1.204528	1.705363	1.768229	2.28951	0.045937369	-0.496052843	0.181559547	-0.308654801
ENSG00000164266	SPINK1	2.806817	2.603675	2.546905	1.650573	2.363369	2.69158	1.526815	2.42936	2.030719	0.87441714	-0.080512856	0.441552269	-0.308602622
ENSG00000126391	FRMD8	15.391586	15.388239	13.426348	13.649947	13.255751	14.825347	10.973684	9.840985	13.385203	0.570948405	0.061154605	0.002504639	-0.308530572
ENSG00000226124	FTCDNL1	1.139009	0.600882	0.93362	0.843702	0.884759	0.700133	0.656227	1.079092	0.747207	0.994064494	-0.004737347	0.517473256	-0.308524161
NewGene_2890	--	2.416709	1.943677	1.934533	1.491388	1.583969	1.878257	2.021488	1.446141	1.826509	0.620357377	-0.182807792	0.319494346	-0.308374753
ENSG00000135108	FBXO21	15.143215	14.438702	14.513482	12.712488	12.270934	12.198951	11.578364	10.801003	11.025254	0.089541161	-0.116700285	0.00000539	-0.30818569
ENSG00000126246	IGFLR1	2.438431	1.98828	2.349558	8.769361	14.589278	14.006288	2.059838	1.158803	1.892863	2.57E-43	2.41286243	0.362315019	-0.308136881
ENSG00000127311	HELB	0.468297	0.555603	0.534894	1.134574	0.819724	0.71993	0.357527	0.443225	0.427738	0.708556741	0.353836749	0.095897792	-0.308065974
NewGene_5282	--	1.085817	0.92053	0.888058	2.106737	1.604083	1.386277	0.899783	0.505389	0.764831	0.002557631	0.951803722	0.476753643	-0.307909022
ENSG00000168395	ING5	10.405082	11.296639	10.797178	9.365067	8.743824	9.292552	8.32682	8.30659	8.807937	0.213209997	-0.105255247	0.0000926	-0.307908421
ENSG00000152952	PLOD2	73.327074	57.932101	55.873974	55.771706	59.639441	54.43915	44.420183	45.636534	52.047708	0.969567734	0.005811133	0.001830351	-0.307820788
ENSG00000168137	SETD5	38.896954	37.806627	35.91851	37.312473	36.088551	34.585206	30.026888	26.465173	28.307088	0.015834826	0.125161887	8.19E-08	-0.307811068
ENSG00000185565	LSAMP	1.799813	2.116248	2.038025	1.312519	1.268366	1.325382	1.573192	1.468285	1.580954	0.0000451	-0.455042127	0.010058866	-0.307684096
ENSG00000120328	PCDHB12	0.486872	0.354222	0.362092	0.255281	0.312763	0.221857	0.267394	0.210662	0.297862	0.693088516	-0.20296573	0.472107337	-0.307660355
ENSG00000100105	PATZ1	30.92602	30.5787	30.08449	27.368745	27.714955	29.096226	23.178482	22.001434	23.454688	0.657399754	0.037335227	0.00000159	-0.307537609
ENSG00000136936	XPA	4.81281	5.168378	4.911266	5.00981	5.378691	5.395683	3.374263	3.70697	3.638089	0.169877223	0.232299366	0.073837298	-0.307375147
ENSG00000125827	TMX4	9.863713	9.868516	9.319954	6.696046	6.139634	5.996736	7.359397	6.823484	7.828643	1.28E-11	-0.476721816	0.0000485	-0.307180122
NewGene_1220	--	5.218097	8.788885	8.295179	7.312578	6.920668	7.225533	7.495169	9.786291	11.664515	0.353088745	0.097412583	0.002075069	-0.307154044
ENSG00000115844	DLX2	0.526094	0.540447	0.984539	0.27173	0.237813	0.16929	0.550174	0.455702	0.510717	0.000793246	-1.468920989	0.455925202	-0.307134139
ENSG00000133985	TTC9	0.255142	0.168267	0.28016	0.170764	0.171461	0.115946	0.161863	0.174353	0.197977	--	--	0.487625021	-0.307084123
ENSG00000143341	HMCN1	2.359126	2.090773	2.055535	1.553618	1.617554	1.494923	1.52206	1.617692	1.768474	0.0000732	-0.373874925	0.00189156	-0.307035028
ENSG00000138658	ZGRF1	1.929412	2.40298	2.329679	2.769557	2.325596	2.710595	1.672998	1.337705	3.377564	0.024175969	0.305837035	0.044858694	-0.306968901
NewGene_2459	--	4.188968	5.795943	5.050015	4.258339	4.659847	3.360546	3.031036	3.343936	4.276015	0.681415691	-0.073182962	0.020868942	-0.306693945
ENSG00000120162	MOB3B	2.100746	2.255303	1.850163	3.263143	3.037864	3.112836	1.760543	1.443148	1.498045	1.21E-12	0.747444552	0.034181183	-0.306626349
ENSG00000124151	NCOA3	9.277284	13.318896	12.373948	13.043144	12.872736	12.491207	8.729364	7.573719	7.527342	0.000000636	0.362650967	0.000262171	-0.306618514
ENSG00000146842	TMEM209	11.188171	11.520443	12.641246	11.01126	9.497484	10.112383	9.354607	7.834449	9.755428	0.664576919	-0.049364509	0.001230062	-0.306580035
ENSG00000101911	PRPS2	10.183749	9.98601	10.774438	12.606894	10.477023	11.452762	7.345838	6.516406	9.057915	0.002550094	0.289602428	0.007418457	-0.306484427
ENSG00000196236	XPNPEP3	2.543567	2.813297	2.575683	2.511518	1.798732	2.151518	2.728561	2.108745	2.200543	0.631091423	-0.087704589	0.014255315	-0.306331806
ENSG00000100815	TRIP11	3.365567	3.402998	3.761696	3.642505	3.192407	3.514089	2.60873	2.422285	3.264592	0.331918824	0.098523184	0.001962953	-0.306231496
ENSG00000124422	USP22	78.888434	75.905922	74.938583	62.605872	60.016724	61.626534	58.722016	52.819714	62.97954	0.00049287	-0.172994571	0.000000808	-0.306181846
ENSG00000169926	KLF13	15.515611	13.867486	13.742275	15.423137	15.588838	15.456013	10.761543	9.989588	12.089068	0.000191173	0.252831778	0.000136659	-0.306131948
ENSG00000253598	SLC10A5	0.339861	0.431868	0.47972	0.449672	0.569596	0.634126	0.255083	0.382336	0.310769	0.174666558	0.55354639	0.517806453	-0.305964253
ENSG00000198046	ZNF667	3.49247	3.362923	4.075463	3.114018	3.0791	2.551383	3.391488	2.607975	2.571123	0.191957444	-0.183916486	0.027400473	-0.305890163
ENSG00000170832	USP32	12.276877	12.677574	12.858659	12.927875	13.651213	12.909567	9.494036	8.611294	10.515959	0.002625886	0.210604712	0.0000997	-0.305511231

Gene ID	Gene name	Ctrl-1 FPKM	Ctrl-2 FPKM	Ctrl-3 FPKM	IFNγ-1 FPKM	IFNγ-2 FPKM	IFNγ-3 FPKM	Sora-1 FPKM	Sora-2 FPKM	Sora-3 FPKM	Ctrl vs IFNγ DESeq2 FDR	Ctrl vs IFNγ DESeq2 log2FC	Ctrl vs Sora DESeq2 FDR	Ctrl vs Sora DESeq2 log2FC
ENSG00000052795	FNIP2	14.32134	14.66672	15.650217	10.156176	11.088446	9.231911	10.696333	10.182806	12.673192	0.000000325	-0.400114978	0.00031081	-0.305479176
ENSG00000257315	ZBED6	23.951131	30.460909	32.050883	26.593038	13.312765	32.51122	12.493175	23.202835	30.774053	0.877878947	0.083929873	0.510640588	-0.305282371
ENSG00000111110	PPM1H	14.611759	13.630033	14.704096	12.277447	13.200112	11.455031	10.706049	10.194364	11.741414	0.443757008	-0.070619103	0.0000201	-0.305166411
ENSG00000185615	PDIA2	0.761711	0.353729	0.632709	0.846159	0.568297	0.82224	0.324725	0.605606	0.395618	0.339432048	0.46292546	0.573987032	-0.304946292
ENSG00000176953	NFATC2IP	8.40667	9.133003	10.31887	8.510508	7.619462	7.587793	7.031037	6.685054	7.283828	0.962563224	0.008049319	0.005723636	-0.304829509
ENSG00000119946	CNNM1	1.366328	1.34467	2.075257	2.071534	2.701027	2.225177	0.893683	1.158234	1.595582	0.0000588	0.695652776	0.188406633	-0.304649425
ENSG00000241852	C8orf58	0.629878	0.536265	0.717736	0.220714	0.461505	0.258179	0.455477	0.465776	0.464245	0.062999925	-0.866819189	0.472617251	-0.304537688
ENSG00000163898	LIPH	0.311129	0.449573	0.442508	1.035257	0.999785	0.865875	0.33513	0.262096	0.317983	7.85E-09	1.402862449	0.425862147	-0.304153847
ENSG00000066322	ELOVL1	31.60814	31.693174	30.827498	25.199547	25.648653	25.290842	23.599552	23.513112	24.277697	0.0608846	-0.148496099	0.0000971	-0.304034093
ENSG00000174842	GLMN	4.625966	4.335629	5.028906	3.654133	3.210763	3.283851	3.780271	2.919179	3.899653	0.123013409	-0.261199499	0.066967862	-0.30400305
ENSG00000165219	GAPVD1	8.818259	12.634081	12.668245	11.371214	13.004449	10.462183	8.336132	7.498943	8.221828	0.026756241	0.180646802	0.000511751	-0.303847018
NewGene_50	--	1.809257	3.392284	3.412986	2.857747	2.234457	2.066689	2.018459	2.122848	2.402975	0.740379082	-0.119732243	0.283185945	-0.303759521
ENSG00000197410	DCHS2	0.804395	0.751268	0.718474	0.408799	0.369484	0.339234	0.609824	0.534458	0.635117	7.81E-13	-0.958867064	0.024975143	-0.303670871
ENSG00000167720	SRR	3.091575	2.645496	2.227769	2.860839	2.470464	2.213419	1.627474	1.361897	1.884216	0.983561517	0.007025495	0.190018718	-0.30366988
ENSG00000139826	ABHD13	8.84273	9.916914	9.452905	11.608541	11.930684	11.746053	7.423669	6.664128	7.352786	6.94E-12	0.470856039	0.000270791	-0.303340514
NewGene_5189	--	0.686773	0.723259	0.535302	0.901462	0.746871	0.826488	0.554174	0.476217	0.487819	0.198985092	0.487512544	0.479471579	-0.303058163
NewGene_1485	--	0.497418	0.509518	0.39253	0.332489	0.3827	0.440986	0.47194	0.297291	0.28792	0.828632516	-0.123466141	0.525868938	-0.30275772
NewGene_4440	--	9.189109	9.339681	10.373408	7.257206	6.528365	7.525284	6.696101	6.620329	8.441638	0.006728729	-0.250776573	0.002581152	-0.302529536
ENSG00000131242	RAB11FIP4	7.671601	7.690174	8.442362	8.985141	7.893212	8.722076	6.267657	5.992055	6.31442	0.001064042	0.226003186	0.000102412	-0.302250994
ENSG00000232653	GOLGA8N	0.314718	0.367397	0.227795	0.28869	0.24091	0.174201	0.227134	0.215092	0.199006	0.741254352	-0.162953024	0.439315966	-0.302074191
ENSG00000155506	LARP1	45.569749	44.135029	45.531162	52.36875	47.611164	50.045935	35.306759	32.34994	38.928414	8.96E-09	0.287116911	0.00000422	-0.301895362
ENSG00000125744	RTN2	4.007476	2.967991	3.429339	1.967977	2.068268	2.791514	2.538379	2.323603	2.95528	0.015190575	-0.4874339	0.110675684	-0.301814244
ENSG00000185798	WDR53	5.838007	4.621534	4.787245	4.279021	4.60875	4.707185	3.714206	4.130184	3.851495	0.856217913	-0.044259162	0.093018357	-0.301709847
ENSG00000114529	C3orf52	5.144234	4.400857	5.120166	3.297404	3.252309	3.602325	3.80307	3.282233	3.921277	0.005948737	-0.403453926	0.042907232	-0.30160583
ENSG00000121210	TMEM131L	4.233277	4.553228	4.667655	5.918781	5.961636	5.761745	3.722815	3.110215	3.359304	1.25E-10	0.543286688	0.006642924	-0.301594333
ENSG00000174953	DHX36	13.849834	14.581831	15.877062	20.216974	14.042807	14.988389	11.001453	13.676457	12.173005	0.214532997	0.097846813	0.0000788	-0.30106481
ENSG00000160007	ARHGAP35	23.530542	22.29497	20.72415	22.537256	22.594156	22.838114	16.941793	15.569156	18.216625	0.005061255	0.177727003	0.0000182	-0.300990827
ENSG00000155868	MED7	3.126968	3.759726	2.829989	2.627741	2.518528	2.774467	2.217108	2.41371	2.082255	0.833415781	-0.051794166	0.133325831	-0.30082856
ENSG00000152601	MBNL1	19.543	22.522016	25.145925	22.939867	23.675753	22.953736	16.528946	15.351035	16.619803	0.021368829	0.198409779	0.000648956	-0.300718939
ENSG00000104361	NIPAL2	2.915294	3.566635	4.198813	2.508821	2.404259	2.143906	2.672361	2.60003	2.864521	0.002465704	-0.448246208	0.041689375	-0.300714189
ENSG00000029363	BCLAF1	52.829013	68.463145	64.002688	68.077515	65.745611	63.701909	40.699289	53.485895	49.897778	0.00496204	0.150469783	0.000000633	-0.300645662
NewGene_4385	--	0.600699	0.360812	0.581049	0.813791	0.704381	0.395238	0.435951	0.239096	0.403604	0.55548636	0.302602819	0.541290216	-0.300642824
ENSG00000107290	SETX	11.006835	11.395402	11.618746	12.225263	12.331748	11.995221	9.102601	7.774177	8.684654	1.97E-10	0.345518327	0.00000541	-0.30060741
ENSG00000067064	IDI1	74.757329	77.235415	77.548048	102.012048	110.005283	104.454017	59.659013	54.890619	57.146812	2.13E-34	0.629029688	0.00000268	-0.300552257
ENSG00000127334	DYRK2	2.712601	2.571967	2.560327	2.369534	2.414724	2.645766	2.202515	2.008819	2.371997	0.762790016	0.040192459	0.003709008	-0.300546524
ENSG00000165891	E2F7	4.747587	5.278149	5.734171	4.248769	3.931459	4.39187	4.220439	3.530102	4.213711	0.092686201	-0.182165785	0.005991692	-0.300436309
ENSG00000144579	CTDSP1	44.20629	43.837358	40.630876	44.596336	43.881712	45.197617	30.240129	32.13157	33.59934	0.001427726	0.233631281	0.000233779	-0.300290325
ENSG00000172671	ZFAND4	0.530073	0.425923	0.621963	0.904266	0.875828	1.055045	0.564302	0.473387	0.305587	0.000205717	0.938668689	0.415395886	-0.30028849
ENSG00000127125	PPCS	15.410965	16.296522	14.948427	13.732986	15.680822	16.27778	12.255257	12.901305	10.321072	0.342256721	0.112862973	0.022129096	-0.300119475
ENSG00000035664	DAPK2	1.017333	0.626703	0.890342	1.461787	1.290484	1.817488	0.627757	0.708425	0.621179	0.0000011	0.998683899	0.292684099	-0.300086104
ENSG00000010379	SLC6A13	0.531058	0.500893	0.334984	0.347266	0.29399	0.28727	0.309951	0.181895	0.556371	--	--	0.589100129	-0.300063805
ENSG00000168000	BSCL2	6.730734	6.021794	7.242073	4.402077	6.725383	6.649829	5.2245	5.780209	4.571049	0.922241421	0.030094351	0.071213057	-0.299610846
ENSG00000078043	PIAS2	1.985983	2.024821	2.353306	2.020147	2.12669	1.905455	1.942284	2.208109	2.036509	0.607767106	-0.080584224	0.018681869	-0.299562701
ENSG00000164933	SLC25A32	10.947924	12.441057	13.687445	11.776197	10.034503	12.261551	9.120495	9.071749	9.870823	0.818154459	0.034946302	0.003977942	-0.29952661
ENSG00000135976	ANKRD36	0.499285	0.616036	0.471114	0.676511	0.492155	0.481211	0.497311	0.360944	0.340174	0.498487741	0.204374541	0.301327914	-0.299484793
ENSG00000141252	VPS53	2.896875	2.695662	2.610781	2.641407	2.734069	2.538205	2.291607	1.866505	2.221708	0.384095841	0.0891609	0.00180508	-0.299430471
ENSG00000120705	ETF1	59.792625	59.487091	64.205922	46.61064	50.866837	48.92825	44.673851	45.579549	47.773053	0.006624515	-0.176849161	0.00000334	-0.299049213
ENSG00000153250	RBMS1	11.579893	11.071057	11.011661	9.802664	10.816431	10.440741	8.696067	8.606488	8.906321	0.717593289	0.038220591	0.000198054	-0.299031114
ENSG00000174606	ANGEL2	5.966772	6.271044	6.564818	6.567124	6.719997	6.197222	4.696786	4.503557	5.165432	0.037308038	0.194861481	0.001853857	-0.299020598
ENSG00000121058	COIL	25.285511	26.765938	26.394758	25.119877	23.025656	22.518423	19.126514	20.504143	20.158741	0.972369966	-0.004061363	0.0000906	-0.299001787
ENSG00000172869	DMXL1	2.322474	2.499653	2.512938	2.279946	2.147536	1.895069	1.949919	1.639589	2.085817	0.556208561	-0.075595291	0.00353453	-0.298952007
ENSG00000177576	C18orf32	13.886747	14.475613	14.109044	10.37443	10.030968	12.186625	9.475776	13.974516	11.885724	0.323676058	-0.127626035	0.007933951	-0.298948081
ENSG00000156675	RAB11FIP1	5.733399	5.586586	5.444229	4.310586	4.07804	4.054226	4.295645	3.855825	4.825936	0.001089905	-0.264326521	0.000845878	-0.298774759
NewGene_4934	--	0.668641	1.041514	0.79088	0.783078	0.666753	0.678034	0.662566	0.817574	0.698253	0.919517538	-0.046532924	0.385363503	-0.298639183
ENSG00000131398	KCNC3	0.882748	0.988294	1.064559	1.003155	1.167922	1.110808	0.96946	0.77409	0.633425	0.328897552	0.211899789	0.1885225	-0.298585073
ENSG00000163376	KBTBD8	0.491701	0.266908	0.551769	0.460008	0.38847	0.26579	0.21375	0.131077	0.248909	0.688963051	0.233960133	0.586266433	-0.298551238
ENSG00000261221	ZNF865	4.598558	4.104609	3.715549	3.785292	3.322904	4.153799	2.896304	2.709333	3.898235	0.972606812	0.007402414	0.045286605	-0.29851541
ENSG00000128602	SMO	20.706245	19.70901	17.005859	13.773523	13.351226	13.521778	14.17641	13.86654	16.324898	0.0000111	-0.352276339	0.001001115	-0.298492919
ENSG00000162769	FLVCR1	10.485999	11.448867	11.03445	13.255558	12.734838	12.016262	8.297158	7.785768	9.121858	0.000000116	0.352091439	0.000104129	-0.298347537
ENSG00000129055	ANAPC13	33.39423	31.656969	32.515721	27.36954	28.167918	29.364814	25.059355	26.413967	26.296747	0.458955387	-0.06864267	0.000134074	-0.298331933
ENSG00000183508	TENT5C	0.375046	0.238724	0.367274	0.342903	0.353236	0.289416	0.361354	0.187432	0.201645	0.720333307	0.149121451	0.460520283	-0.29766358
ENSG00000143373	ZNF687	11.365959	11.790793	10.304472	11.64001	11.088417	11.633222	8.587114	7.627801	9.45226	0.05363237	0.157288619	0.001303979	-0.297553613
ENSG00000145833	DDX46	30.263611	31.006241	33.001884	28.135489	28.606898	26.801289	25.270154	23.759506	24.759127	0.724027352	-0.037633605	0.000490054	-0.297525601
ENSG00000180318	ALX1	2.865179	2.917866	3.244144	3.100002	3.07443	3.347841	2.135322	2.647141	2.081301	0.313666348	0.225086632	0.204454285	-0.297397177
NewGene_1312	--	4.664073	5.0404	5.194137	4.229844	4.297556	5.166356	3.990899	2.968325	4.440191	0.921032889	0.032494445	0.224937538	-0.297127353
ENSG00000172738	TMEM217	1.373178	0.87133	1.641595	1.092816	1.112482	1.30263	0.892997	0.905815	1.205494	0.993484931	0.004750894	0.419218134	-0.297056124
ENSG00000123095	BHLHE41	2.032516	2.019094	2.212763	0.403271	0.580474	0.522292	1.545729	1.489226	1.749218	1.38E-23	-1.896233135	0.068816626	-0.29700791
ENSG00000141542	RAB40B	1.34449	1.351829	1.01294	0.992607	1.086788	1.133065	0.974981	0.899944	0.830757	0.880120789	0.046690206	0.202354208	-0.296780405
ENSG00000060749	QSER1	10.939446	13.306329	13.558504	12.410662	10.280321	10.987784	9.929385	9.090018	10.859893	0.902234421	-0.016350762	0.000996514	-0.296699389
ENSG00000187720	THSD4	0.400512	0.351464	0.317125	0.282617	0.166033	0.265311	0.239216	0.284224	0.295786	0.126913612	-0.485914613	0.25995041	-0.296681801
NewGene_2006	--	7.856523	8.193341	8.545144	8.555654	6.795444	6.749278	6.358868	5.975924	6.713884	0.977661662	-0.00430326	0.000638113	-0.296559936
ENSG00000107104	KANK1	26.977879	26.944828	25.345182	28.883421	29.427149	27.852147	19.007117	18.781238	22.283538	0.00000684	0.246946961	0.0000182	-0.296189506
ENSG00000113721	PDGFRB	4.737922	4.341784	4.281982	2.188867	2.392504	2.176891	3.154489	3.214477	3.882298	1.87E-17	-0.84654455	0.010660525	-0.296091979
ENSG00000136870	ZNF189	8.275364	8.343512	8.852058	10.508361	9.844354	9.193345	7.315338	6.200815	6.268965	0.0000318	0.365036935	0.006455582	-0.296083706

Gene ID	Gene name	Ctrl-1 FPKM	Ctrl-2 FPKM	Ctrl-3 FPKM	IFNγ-1 FPKM	IFNγ-2 FPKM	IFNγ-3 FPKM	Sora-1 FPKM	Sora-2 FPKM	Sora-3 FPKM	Ctrl vs IFNγ DESeq2 FDR	Ctrl vs IFNγ DESeq2 log2FC	Ctrl vs Sora DESeq2 FDR	Ctrl vs Sora DESeq2 log2FC
ENSG00000137135	ARHGEF39	10.992106	11.544878	11.338688	8.380879	7.354573	7.686218	8.538587	8.076159	8.83123	0.000000211	-0.400194592	0.000283876	-0.295975632
ENSG00000144711	IQSEC1	16.10345	16.679291	14.447238	15.204642	13.998366	15.307792	11.559755	11.777722	12.664793	0.116222379	0.120371594	0.000155942	-0.295940911
ENSG00000161714	PLCD3	1.743876	1.813383	1.866719	0.691035	0.763474	0.836937	1.321096	1.299509	1.533067	1.21E-14	-1.117103646	0.035965405	-0.295555267
ENSG00000171208	NETO2	1.684893	1.535557	2.013994	1.387262	1.344834	1.493733	1.252232	1.397756	1.469335	0.074551435	-0.29634411	0.073154625	-0.295144179
ENSG00000173193	PARP14	1.461584	1.415066	1.254527	79.336193	70.234207	69.416642	1.039594	1.053676	1.062131	0	5.891683337	0.041388221	-0.295101905
NewGene_6054	--	1.063843	0.60165	0.798291	0.671633	0.424992	0.406955	0.577113	0.48693	0.822972	0.129444379	-0.557849662	0.401293866	-0.295000724
ENSG00000159140	SON	65.894664	68.052088	69.391397	69.366988	68.502494	74.029871	56.910944	48.400001	59.025579	0.000139763	0.172202418	0.000000031	-0.294893958
ENSG00000183479	TREX2	1.174558	1.790239	0.456115	0.545719	0.238053	0.344161	2.060082	0.222389	0.339347	0.00075614	-1.511790916	0.835426294	-0.294839057
ENSG00000007062	PROM1	63.975731	64.979073	71.970712	43.927951	42.327203	43.215111	47.837991	49.756542	56.519856	4.86E-20	-0.455392388	0.00000251	-0.294375108
ENSG00000181938	GINS3	6.812973	7.28868	6.949041	7.186242	5.928134	6.299162	5.377445	5.325139	5.153449	0.930526588	0.016301127	0.018223399	-0.294223704
ENSG00000162413	KLHL21	6.721902	7.008228	7.064077	5.9672	6.084039	5.809274	5.178696	5.336807	6.056216	0.85398473	-0.021990665	0.00169985	-0.294219765
NewGene_6269	--	1.7217	1.230969	1.469636	1.87667	1.654879	1.431173	0.955708	1.509737	1.773592	0.010318515	0.464898692	0.166880175	-0.294193204
ENSG00000133131	MORC4	2.641305	3.145712	3.182728	3.038781	3.195146	2.789285	2.046429	2.50255	2.54117	0.394899676	0.137305867	0.057725692	-0.2940524
ENSG00000165502	RPL36AL	59.586853	57.082943	63.239014	66.801773	75.429642	68.458946	42.352661	54.839027	39.818554	0.00000363	0.37528518	0.023433478	-0.293849559
ENSG00000236699	ARHGEF38	1.205862	1.097418	1.240402	0.565388	0.437619	0.465765	0.872138	0.8842	1.081363	4.39E-09	-1.139426771	0.140602547	-0.293755875
ENSG00000198682	PAPSS2	1.499379	1.437296	1.694891	1.665447	1.726954	1.45645	1.135109	1.185515	1.21332	0.281830379	0.212191187	0.119914159	-0.293701837
ENSG00000165506	DNAAF2	5.324748	5.047659	5.195964	3.469442	3.285518	4.191433	3.826134	3.734081	4.277255	0.007678057	-0.359575186	0.015595524	-0.293648223
ENSG00000143164	DCAF6	25.149095	24.562918	26.247141	24.911981	23.938264	23.356864	19.779503	18.329524	20.085972	0.325738665	0.075711588	0.0000301	-0.293641882
ENSG00000159202	UBE2Z	30.415275	29.877234	29.740982	27.913175	29.455913	28.032108	21.423015	22.034967	25.416134	0.487847821	0.055251731	0.0000891	-0.293625433
ENSG00000177683	THAP5	6.962242	8.291316	8.464787	8.537849	7.827985	8.156773	6.575579	5.514199	5.842495	0.159952429	0.167616044	0.018432918	-0.293612118
ENSG00000171962	DRC3	0.868778	0.62393	0.590457	0.621723	0.55087	0.678885	0.53253	0.444827	0.513844	0.755881993	0.155614125	0.497856593	-0.293593915
ENSG00000198944	SOWAHA	2.460253	2.261234	2.011384	2.520474	2.616466	2.362784	1.661775	1.641657	1.863674	0.072190999	0.300556067	0.096729086	-0.293491398
ENSG00000167491	GATAD2A	26.378711	24.179465	25.408716	22.013187	20.294153	21.80727	20.139999	17.295835	22.382069	0.133209106	-0.104587998	0.000149366	-0.29346498
ENSG00000128908	INO80	11.51849	10.642606	10.861799	12.272635	12.61567	11.93824	8.726593	7.913938	8.764072	0.00000345	0.303906503	0.0000831	-0.29325945
ENSG00000084090	STARD7	54.664502	59.726166	57.428985	54.957962	56.344511	56.94158	44.511434	41.09331	45.858131	0.053715309	0.119278529	0.00000283	-0.293211402
ENSG00000163586	FABP1	106.498424	86.905599	71.354504	94.245706	100.579025	85.656833	81.147982	65.122608	64.524337	0.223344049	0.298903602	0.253518479	-0.293145762
ENSG00000125637	PSD4	2.257119	3.236638	2.817437	2.240515	2.944867	2.551346	2.190874	1.934062	2.384393	0.670534926	-0.071471362	0.018281405	-0.293030121
ENSG00000213066	FGFR1OP	7.598255	9.697709	10.64435	11.420047	10.775438	10.597024	12.507445	12.635257	11.681249	0.927164202	0.013249003	0.002768365	-0.292958917
ENSG00000160602	NEK8	4.740218	3.857133	3.958434	5.223877	5.17861	4.601482	3.13422	2.908451	3.971414	0.005162739	0.337890722	0.035965358	-0.292925995
ENSG00000145725	PPIP5K2	7.048717	8.662659	8.768131	6.755056	7.252543	7.331138	6.177942	6.544277	5.945312	0.793733784	-0.027647696	0.000135293	-0.292810635
ENSG00000259120	SMIM6	6.471873	6.479305	4.979594	2.813342	2.664651	3.423395	4.778286	4.746053	4.271243	0.0000468	-0.859875339	0.162605169	-0.292717267
ENSG00000141696	P3H4	27.432596	27.200551	26.052506	25.235696	25.522924	25.472404	19.180863	20.603301	22.508179	0.417549693	0.063748317	0.000096	-0.292598404
ENSG00000182307	C8orf33	30.129584	31.696313	31.288758	25.118175	24.761506	25.715887	24.735611	23.233199	23.218687	0.020884291	-0.153571375	0.000059	-0.292545174
ENSG00000214654	B3GNT10	1.984946	1.913378	2.393015	2.038846	1.846507	2.15866	1.547419	1.644839	1.862332	0.71273024	0.116130277	0.27834068	-0.292450116
ENSG00000162873	KLHDC8A	2.736183	2.215034	2.448471	0.94228	1.011199	0.819557	1.869163	2.019351	1.797811	5.54E-14	-1.359775398	0.09620187	-0.292397343
ENSG00000139651	ZNF740	7.384688	7.470984	7.134206	6.582369	6.655785	6.442502	5.406666	5.830375	5.582675	0.899856533	-0.012812551	0.00024227	-0.292148701
ENSG00000198840	MT-ND3	749.946777	943.459656	925.79303	981.43689	1235.191406	1107.223389	729.368225	872.424438	385.269409	0.003125519	0.493163391	0.544573148	-0.292038788
ENSG00000170456	DENND5B	5.901753	6.171744	5.90204	5.850088	5.853427	6.043437	6.68485	5.309068	5.413258	0.075066896	0.145070442	0.001519914	-0.292009745
ENSG00000186638	KIF24	2.665042	3.045718	2.824864	2.476259	2.264342	2.251976	2.213411	2.082126	2.269234	0.282183387	-0.139855731	0.012600097	-0.292001266
ENSG00000144228	SPOPL	4.832552	6.474754	6.423654	6.214297	6.6732	6.445598	4.162115	4.273936	5.068604	0.012367412	0.281409709	0.017822715	-0.291989386
ENSG00000274211	SOCS7	5.078185	4.390338	4.658833	4.588037	4.255433	4.408485	3.580431	3.399673	3.94589	0.668274919	0.050072791	0.002598424	-0.291910356
ENSG00000170577	SIX2	2.318206	1.90215	1.582315	2.959009	2.54624	2.76705	0.996245	1.627936	1.827363	0.000775683	0.65582703	0.301636553	-0.291860626
ENSG00000080345	RIF1	11.562577	12.815434	13.878403	13.185019	12.691683	11.595817	9.494736	8.656351	10.529868	0.27641958	0.085489401	0.0000921	-0.291816557
ENSG00000115825	PRKD3	14.253475	14.566959	14.711605	13.288919	13.635341	13.124132	11.24142	11.034259	11.62833	0.852852608	0.017671386	0.0000195	-0.291675232
ENSG00000015479	MATR3	72.599632	88.096811	90.214948	83.820738	74.149278	52.592154	68.309872	58.435471	67.397897	0.619959212	-0.13680205	0.000975502	-0.291626844
ENSG00000163491	NEK10	3.647811	3.680829	4.005393	1.822655	1.498849	1.75924	2.579101	2.598269	3.24862	3.74E-14	-0.990103041	0.046394403	-0.291624824
ENSG00000264522	OTUD7B	8.3571	8.111159	8.704603	7.826104	7.536661	7.885869	6.998808	6.145731	6.130094	0.712832013	0.032923612	0.000472764	-0.291623179
ENSG00000110200	ANAPC15	14.305027	14.411171	12.989003	8.967969	9.695785	9.262239	10.497235	11.231137	10.145911	0.001870499	-0.418665465	0.040282971	-0.29159144
ENSG00000282246	AL157392.5	1.132764	0.994245	1.025592	0.8304	1.427104	0.622314	0.751419	0.694639	0.894674	0.92020154	-0.067403815	0.524417343	-0.291558014
ENSG00000171135	JAGN1	48.536644	47.942448	46.80217	41.327496	45.591317	43.38569	34.598025	37.735131	37.192633	0.88698525	0.015016532	0.0000994	-0.291492033
ENSG00000167487	KLHL26	1.633281	1.424729	1.260556	1.601015	1.495743	1.725215	1.235967	1.072687	0.909197	0.073393145	0.318293607	0.155687477	-0.291384083
ENSG00000139946	PELI2	1.750852	1.787134	1.798633	1.546549	1.578181	1.498323	1.398389	1.224831	1.502396	0.750162397	-0.057714035	0.043079247	-0.291364141
ENSG00000111300	NAA25	12.893276	14.642759	15.085838	15.767267	14.641296	14.797644	11.132376	10.461632	11.113111	0.00378424	0.219667455	0.000354078	-0.29134414
ENSG00000003436	TFPI	37.667022	42.95128	43.848749	32.611121	30.038113	31.264855	30.678872	33.872635	31.512095	0.0000197	-0.318786646	0.000405754	-0.291258327
NewGene_4186	--	1.424952	1.298669	1.127968	0.983717	0.919203	1.002595	0.91361	0.894098	0.612391	0.272458442	-0.316152996	0.314013967	-0.291129049
ENSG00000115282	TTC31	7.649311	7.296682	7.66477	7.513472	7.089503	7.226893	5.524816	5.371378	6.501585	0.400254987	0.093568701	0.005084974	-0.290947426
ENSG00000213853	EMP2	0.34744	0.361897	0.323702	0.26814	0.292654	0.252938	0.270038	0.269971	0.28245	0.359750922	-0.347826487	0.402277298	-0.290907953
ENSG00000020577	SAMD4A	2.189386	2.907244	2.769982	2.490512	2.401007	2.129636	2.246873	1.742707	2.333251	0.735657454	0.061264765	0.046602957	-0.290853361
ENSG00000116260	QSOX1	6.312512	4.317822	4.372613	4.806363	4.97276	5.242899	4.222781	3.493425	4.38164	0.425111717	0.11419353	0.021756963	-0.290740458
ENSG00000178127	NDUFV2	82.063105	80.620547	82.169983	81.778507	84.850902	80.09901	62.884271	63.893902	60.721385	0.019518426	0.157829465	0.000113229	-0.290680111
ENSG00000136828	RALGPS1	2.527552	2.634715	2.62046	3.797411	3.566819	3.088557	2.021531	1.966207	2.167739	0.0000127	0.491580511	0.034908032	-0.290669138
ENSG00000081913	PHLPP1	1.954605	2.23745	1.845163	2.225384	2.000154	1.911962	1.555258	1.358385	1.723671	0.246187839	0.170020675	0.042574703	-0.290398518
ENSG00000184517	ZFP1	2.955468	2.969652	4.181624	2.417505	2.487236	2.342301	3.009415	2.436848	2.58923	0.043279877	-0.316612908	0.061718197	-0.290325632
ENSG00000136161	RCBTB2	0.412246	0.426631	0.466843	0.181424	0.233171	0.151615	0.346653	0.309307	0.342129	0.011887448	-1.055725795	0.45926387	-0.290142614
ENSG00000115977	AAK1	2.073998	2.391876	2.812097	2.41066	2.152714	2.316438	1.897463	1.823669	1.861664	0.839564945	0.035487898	0.027176054	-0.290014673
NewGene_2788	--	0.908952	0.747512	0.898116	0.808028	0.571808	0.522332	0.597187	0.832767	0.741721	0.441449329	-0.268181897	0.350956712	-0.28999647
ENSG00000075420	FNDC3B	49.2162	51.589458	57.595216	50.859847	47.345548	49.465131	44.784533	40.183824	45.167408	0.960202344	-0.004951224	0.0000707	-0.289992017
ENSG00000125107	CNOT1	34.175548	34.276727	35.030157	39.11286	34.657226	35.369658	25.752787	26.196465	27.840691	0.002886313	0.166763942	0.000000139	-0.289867537
ENSG00000132405	TBC1D14	32.591165	31.539185	30.658918	30.048189	29.910164	29.940054	24.600783	22.093879	25.975405	0.193993351	0.081503085	0.000014	-0.289748414
ENSG00000006634	DBF4	18.329793	20.0395	19.624656	22.331221	20.182013	20.369909	13.866294	13.320851	17.230202	0.008837727	0.218141801	0.001118287	-0.289430625
ENSG00000181982	CCDC149	3.101516	2.82713	2.939853	3.285109	3.21216	3.360975	2.382858	2.07271	2.560833	0.018230291	0.301273917	0.050994204	-0.289312361
ENSG00000144674	GOLGA4	9.471561	10.860117	11.715612	11.75215	11.685203	10.589953	8.18726	7.977279	8.540043	0.019286186	0.204017689	0.000987985	-0.289210975
ENSG00000160408	ST6GALNAC6	9.595608	8.117088	6.998714	10.930318	9.400527	7.787781	6.500917	5.84769	6.696212	0.048663624	0.301877341	0.031667898	-0.2891872
ENSG00000118939	UCHL3	15.837133	19.579753	23.5877	15.998135	13.400609	14.949179	14.455242	15.279732	15.810128	0.085995643	-0.272649644	0.056250969	-0.288995683

Gene ID	Gene name	Ctrl-1 FPKM	Ctrl-2 FPKM	Ctrl-3 FPKM	IFNγ-1 FPKM	IFNγ-2 FPKM	IFNγ-3 FPKM	Sora-1 FPKM	Sora-2 FPKM	Sora-3 FPKM	Ctrl vs IFNγ DESeq2 FDR	Ctrl vs IFNγ DESeq2 log2FC	Ctrl vs Sora DESeq2 FDR	Ctrl vs Sora DESeq2 log2FC
NewGene 2790	--	3.441051	3.14957	2.66162	2.580403	2.825109	2.705864	1.7846	2.725082	2.865386	0.898265309	-0.043188192	0.298067146	-0.288948665
ENSG00000187231	SESTD1	9.102531	9.823719	9.490055	6.035283	5.697578	5.56854	7.6712	6.896173	7.582543	9.12E-22	-0.564963695	0.0000187	-0.28888394
ENSG00000278535	DHRS11	3.760544	4.896939	5.522099	3.316647	3.132199	4.231091	3.313176	3.535517	4.017227	0.174236154	-0.29575482	0.138569192	-0.288883058
ENSG00000184949	FAM227A	0.217853	0.180793	0.163253	0.09985	0.085395	0.093483	0.141618	0.154589	0.160115	0.010532317	-0.894554092	0.396212992	-0.288562615
ENSG00000103528	SYT17	0.520317	0.563048	0.483242	0.133581	0.096019	0.481695	0.597763	0.491014	0.177932	0.062708251	-0.827903479	0.463728853	-0.288560432
ENSG00000141956	PRDM15	3.093375	3.292291	3.621187	2.657477	2.709471	2.693965	2.323352	2.178428	2.544375	0.658895243	-0.060720184	0.010064788	-0.288513436
ENSG00000162946	DISC1	1.439769	1.017099	1.343319	1.016285	0.955868	0.926064	1.194405	1.153667	1.230175	0.162234389	-0.266735355	0.137265311	-0.288448068
ENSG00000134262	AP4B1	2.525675	2.793874	2.203527	2.226921	2.078011	2.052457	1.824918	1.829325	2.168244	0.678229972	-0.093668694	0.119371153	-0.288203011
NewGene 3087	--	0.84212	0.748035	0.631349	1.00081	0.847285	0.894402	0.494997	0.736446	0.54884	0.158614444	0.483475321	0.465084879	-0.288116136
ENSG00000072849	DERL2	16.580545	16.517945	18.51844	19.017923	17.524561	18.523963	15.528558	13.65442	15.112188	0.000359016	0.267835056	0.000630879	-0.287814179
ENSG00000124486	USP9X	16.190939	14.904361	15.125936	15.547349	14.999678	14.295791	11.863582	11.097508	11.902805	0.037053518	0.117879805	0.00000219	-0.287744761
NewGene 1434	--	4.794837	5.524505	5.964904	6.05559	4.242335	5.735431	4.494055	3.899437	5.014184	0.834621204	0.03470934	0.012472612	-0.287412335
NewGene 2556	--	3.219708	3.586993	3.274742	3.259081	2.519619	3.921412	2.885226	2.088059	2.845117	0.78013289	0.051710625	0.031766738	-0.287343606
ENSG00000140931	CMTM3	39.054437	32.265339	33.067043	10.619361	10.686038	10.113974	23.344499	25.542427	30.338727	1.99E-74	-1.580057753	0.006161647	-0.287197325
ENSG00000156599	ZDHHC5	33.753427	32.787333	30.790727	33.618231	32.977834	32.264537	24.126833	24.196315	26.553339	0.006214158	0.168520589	0.0000166	-0.287103656
ENSG00000213638	ADAT3	3.92617	3.298486	3.445198	3.333881	3.522096	3.304014	2.184884	2.482387	3.227241	0.669524958	0.10167538	0.180695947	-0.287027329
ENSG00000090989	EXOC1	11.908748	12.352504	12.110553	11.227399	10.486926	10.544392	9.631756	9.112305	9.354616	0.876744628	-0.017416855	0.000496456	-0.286982911
ENSG00000140396	NCOA2	13.384753	13.493863	13.726794	16.175287	14.866256	14.257449	10.826797	9.3687	11.196292	0.000000161	0.306328276	0.0000354	-0.286955526
ENSG00000204116	CHIC1	0.590509	1.035817	1.200796	0.876327	1.228362	1.044312	0.652062	0.895165	0.787433	0.658692466	0.314909278	0.638867718	-0.286871295
ENSG00000103479	RBL2	5.178708	5.197903	5.643074	5.516267	5.353069	5.35785	3.783262	3.891184	4.660745	0.088545856	0.165495922	0.007590284	-0.286717725
ENSG00000161654	LSM12	20.814697	25.156541	22.976685	24.862894	21.969956	20.320206	17.570589	18.585047	16.696077	0.343832347	0.10497271	0.003181709	-0.286700337
ENSG00000066933	MYO9A	0.930295	1.223392	1.027295	0.851082	0.965554	0.935219	0.96433	0.806264	1.152422	0.638129876	-0.090262275	0.063841869	-0.286255721
ENSG00000159086	PAXBP1	24.151429	25.193083	26.191104	23.336099	21.033687	21.865393	20.191636	18.504519	20.836388	0.613210235	-0.043638512	0.0000794	-0.286149417
ENSG00000113194	FAF2	20.407073	20.460249	20.686054	19.587031	19.099111	19.122982	15.62353	14.732401	17.121626	0.396514989	0.060436785	0.0000366	-0.286083732
ENSG00000066468	FGFR2	47.357161	45.10042	53.696229	20.169925	22.579077	19.701033	35.350603	34.339828	40.002613	6.17E-49	-1.071190236	0.000117491	-0.286028467
NewGene 400	--	0.916461	0.711857	0.793631	0.582187	0.668867	0.791757	0.370435	0.749744	0.745751	0.833575834	-0.086302333	0.426959873	-0.285858008
ENSG00000176542	USF3	2.898702	2.978069	2.818304	3.098734	2.924526	2.787717	2.370557	2.13101	2.209032	0.051153945	0.16491062	0.000949166	-0.285373233
ENSG00000188659	SAXO2	1.352912	1.346747	1.126062	1.036651	1.267595	0.942012	0.842512	0.674308	1.15003	0.923649342	0.039451422	0.395752549	-0.285231234
ENSG00000080839	RBL1	3.625932	3.955386	3.961752	3.716299	3.657804	3.563282	2.955566	2.87825	3.111457	0.601655939	0.066277432	0.006980445	-0.285132861
ENSG00000186111	PIP5K1C	7.789556	6.805945	6.29359	6.155455	5.884327	5.956954	4.982	4.896724	6.190757	0.539116263	-0.075839773	0.01198202	-0.285125032
ENSG00000095002	MSH2	30.171462	29.894175	32.097157	25.973459	23.772026	25.139395	24.299242	22.525705	24.144392	0.024184581	-0.151742699	0.0000301	-0.284803241
NewGene 6398	--	9.744209	8.8758	8.17343	11.112994	12.586429	11.571306	6.135839	6.345302	8.264519	2.18E-09	0.53992765	0.017877613	-0.284798177
ENSG00000253953	PCDHGB4	0.599086	0.891497	1.406477	0.437056	0.629816	0.636883	0.575591	0.995742	0.638315	0.059439265	-0.601191159	0.397767262	-0.284667989
ENSG00000139531	SUOX	15.001409	13.870156	12.127977	13.735614	13.348684	12.328724	10.331491	9.815645	11.985437	0.556247988	0.072417565	0.007536061	-0.284577495
NewGene 1595	--	56.825127	56.985861	58.566581	49.60804	48.477439	51.028703	44.547649	41.507752	46.564803	0.33749329	-0.060122567	0.000000914	-0.284510796
ENSG00000105339	DENND3	3.679932	3.923999	3.383103	2.201565	1.742117	1.648536	2.53453	2.337958	3.009566	2.59E-11	-0.878968178	0.023740193	-0.284421713
ENSG00000272602	ZNF595	6.804839	7.745676	7.9948	7.570227	7.041034	7.003792	6.172241	6.00848	5.017803	0.409417381	0.107958115	0.034030156	-0.284298407
ENSG00000196455	PIK3R4	12.93089	13.341874	12.426914	13.036716	12.218395	11.711606	9.859084	9.479882	10.558699	0.341601708	0.079732741	0.00010931	-0.283747756
ENSG00000164687	FABP5	33.22737	38.026962	36.41091	32.154168	33.599753	31.721894	28.56881	29.527322	24.60659	0.973949356	0.004968437	0.014254508	-0.283746532
ENSG00000110851	PRDM4	8.765476	8.344077	8.841503	7.473531	7.634213	7.78201	6.675243	5.993202	7.563649	0.738990656	-0.037313286	0.002856678	-0.283551044
ENSG00000116668	SWT1	2.579818	2.897123	2.903937	2.606751	2.248418	2.856356	2.025944	2.367085	2.149136	0.981452818	0.0054397	0.064760632	-0.283384034
ENSG00000125630	POLR1B	14.733127	15.737886	15.528454	13.390511	13.062383	12.472456	11.882521	10.946416	12.271088	0.080580336	-0.128557416	0.0000901	-0.283332904
ENSG00000134109	EDEM1	6.912104	7.628726	8.370952	9.842766	9.425237	9.804379	6.182355	5.287953	6.512495	3.73E-10	0.488247033	0.004984799	-0.283193508
ENSG00000143801	PSEN2	6.422263	5.943332	6.877083	4.600131	3.574122	4.185364	4.464907	4.958671	5.436335	0.0000541	-0.505087327	0.039445593	-0.283146267
ENSG00000198482	ZNF808	1.447934	1.230137	1.155978	1.232504	1.280208	0.88181	1.090719	1.051074	1.064091	0.754114313	-0.090981314	0.184087145	-0.28312343
ENSG00000163617	CCDC191	0.800273	0.941408	0.860102	0.81201	0.893237	0.703557	0.804522	0.626084	0.717839	0.975983171	0.011234377	0.262319256	-0.283118656
NewGene 4356	--	2.450682	2.294103	2.263649	1.847566	2.152844	1.786772	1.892236	1.605451	1.815616	0.538129919	-0.109443466	0.047623845	-0.282715348
NewGene 762	--	2.523262	2.762273	3.056617	2.238396	1.694962	1.810054	1.823715	2.453952	2.108716	0.000896045	-0.367106951	0.014602432	-0.282403054
ENSG00000132749	TESMIN	6.887575	6.955474	6.861421	4.872014	4.731048	4.447529	5.161043	5.315464	6.219864	0.0000536	-0.396623485	0.009803013	-0.282293189
ENSG00000083168	KAT6A	10.367121	9.63499	9.489313	9.614186	9.697609	10.418343	7.681554	7.298153	7.336026	0.002109651	0.19416248	0.0000685	-0.281253878
ENSG00000138821	SLC39A8	5.027258	5.111395	5.91356	4.578511	4.200095	4.241274	4.183553	4.111466	4.096718	0.277607408	-0.147154202	0.023668956	-0.281133488
ENSG00000168228	ZCCHC4	9.345808	10.421265	11.701575	9.090332	9.062442	8.340856	8.218811	7.501696	8.674672	0.397929519	-0.107157215	0.011229107	-0.281116163
ENSG00000083520	DIS3	3.353146	3.869961	3.738956	3.564343	3.907345	4.110861	2.439183	2.656246	2.498249	0.197424603	0.125836678	0.002861323	-0.281064701
ENSG00000147180	ZNF711	6.724635	6.87977	6.702151	5.86339	5.634601	5.442135	5.503534	4.806909	5.49546	0.21432107	-0.122918451	0.002647934	-0.280990114
NewGene 2012	--	1.915349	1.680581	1.932224	0.94738	0.806981	0.904221	1.241169	1.620782	1.207966	0.000130305	-0.844807402	0.219571204	-0.280966154
ENSG00000151692	RNF144A	1.423827	1.66269	1.442261	1.211353	1.173775	1.001618	1.298621	1.188623	1.044864	0.130246611	-0.26268591	0.099488249	-0.280656879
ENSG00000123643	SLC36A1	3.324683	3.527537	3.697966	2.7832	2.783793	2.78757	2.627485	2.822352	2.752509	0.055577921	-0.210403711	0.011906514	-0.280547423
ENSG00000112078	KCTD20	36.20021	36.285329	36.506257	34.336102	33.679163	33.283065	29.458001	25.981237	29.379487	0.853136186	0.013910907	0.00000477	-0.280421537
NewGene 6358	--	1.34997	1.455589	0.96148	2.230167	1.756472	0.526256	1.340192	1.024891	0.737315	0.795074748	0.297231786	0.385295913	-0.280342672
ENSG00000138061	CYP1B1	0.474615	0.440325	0.530275	0.803142	0.80157	1.043794	0.406775	0.36063	0.344788	0.00000289	1.020588867	0.342902688	-0.280338925
ENSG00000088986	DYNLL1	180.779468	190.911578	168.166197	175.912846	162.695055	179.696915	148.150947	148.44589	123.200022	0.238497065	0.091088592	0.004154003	-0.280326748
ENSG00000099937	SERPIND1	60.619031	49.235643	42.670585	83.500335	82.754702	78.460884	38.50601	40.212205	38.625438	0.000000179	0.829706442	0.010181542	-0.280300494
ENSG00000142065	ZFP14	0.922361	1.153688	1.019266	0.90167	1.184355	1.100636	0.885734	0.774189	0.855086	0.379366132	0.17542061	0.118364775	-0.2801362
ENSG00000099381	SETD1A	11.984338	10.904076	11.066341	10.942447	10.478567	11.230556	7.920062	7.695116	10.711895	0.267266507	0.09012836	0.008219832	-0.279993498
ENSG00000186908	ZDHHC17	9.582789	10.589227	10.758971	10.526058	9.844367	9.447964	8.0051	8.061743	8.206568	0.387773968	0.082104807	0.00078052	-0.279951955
ENSG00000165392	WRN	11.409827	11.8613	12.77212	7.701055	6.649247	7.069284	9.220838	8.647745	9.999861	6.89E-16	-0.603693917	0.000470006	-0.27972707
ENSG00000100207	TCF20	12.933334	12.050307	12.193831	11.74193	11.928332	12.131419	9.129492	8.480332	9.891318	0.112322075	0.120759339	0.000626007	-0.279529128
ENSG00000158234	FAIM	10.207211	10.957353	12.886574	10.357055	9.707082	9.620089	6.806112	8.99972	8.645782	0.962852033	-0.010296219	0.073816318	-0.27941967
ENSG00000159267	HLCS	6.982055	7.529074	6.671916	5.57134	5.186267	5.975444	5.158021	5.545851	6.213432	0.023161351	-0.204894587	0.002190824	-0.279337896
ENSG00000183527	PSMG1	22.045098	21.898486	29.522134	29.206862	22.68254	23.412801	19.102392	16.314035	17.064905	0.703545537	0.048874566	0.007332856	-0.279213403
ENSG00000181444	ZNF467	2.331596	1.492809	1.332257	2.993586	2.865997	3.634533	1.345177	1.306742	1.398566	0.00000577	0.984042302	0.322209271	-0.279184272
ENSG00000187678	SPRY4	3.325712	3.433757	4.125425	2.295085	2.73186	2.621002	2.493254	2.714608	3.30236	0.001578811	-0.393205705	0.042111391	-0.279158656
ENSG00000101846	STS	0.613686	0.462989	0.646218	0.756928	0.652079	0.846061	0.359663	0.556295	0.40802	0.01587151	0.532692795	0.323183116	-0.279071541
ENSG00000116396	KCNC4	0.08536	0.379297	0.147727	0.117525	0.103867	0.144968	0.100508	0.169521	0.175321	--	--	0.600219369	-0.279066462

Gene ID	Gene name	Ctrl-1 FPKM	Ctrl-2 FPKM	Ctrl-3 FPKM	IFNγ-1 FPKM	IFNγ-2 FPKM	IFNγ-3 FPKM	Sora-1 FPKM	Sora-2 FPKM	Sora-3 FPKM	Ctrl vs IFNγ DESeq2 FDR	Ctrl vs IFNγ DESeq2 log2FC	Ctrl vs Sora DESeq2 FDR	Ctrl vs Sora DESeq2 log2FC
ENSG00000167555	ZNF528	4.507121	4.75346	4.94048	3.049708	3.037025	2.639592	4.85686	3.860333	3.378964	0.000000217	-0.64856573	0.028423124	-0.278896128
ENSG00000174137	FAM53A	2.657188	2.591293	2.495984	1.679175	1.876519	1.954333	1.981542	1.717139	2.014743	0.071450549	-0.380032973	0.170033734	-0.27858519
ENSG00000047617	ANO2	9.15402	8.305758	9.932943	8.405357	7.309292	7.310122	7.757947	6.294205	7.181364	0.423642727	-0.096633341	0.011232672	-0.278536134
ENSG00000214367	HAUS3	7.164893	7.992448	11.196903	8.496784	6.786594	6.213311	7.493626	7.08602	6.041055	0.913492494	-0.018412247	0.004682262	-0.278519474
ENSG00000113812	ACTR8	10.341694	10.21035	9.702306	8.596021	8.055026	9.243366	8.02939	7.364703	8.029457	0.307885718	-0.101070615	0.001351271	-0.278363139
NewGene_1687	--	1.222415	1.235512	1.371792	1.214942	0.870437	1.007149	0.91082	1.036103	1.03716	0.738990656	-0.108885668	0.295734806	-0.278325304
ENSG00000182224	CYB5D1	9.388205	9.393269	9.541239	7.697115	7.792061	7.168412	7.831385	7.276953	6.914734	0.063138187	-0.171935396	0.003863499	-0.278301248
ENSG00000121577	POPDC2	0.454128	0.544752	0.610913	0.701608	0.404407	0.484329	0.395952	0.544086	0.305297	0.832431415	0.12131326	0.569002808	-0.278181472
ENSG00000135083	CCNJL	5.700762	4.72353	5.776925	2.29552	2.330749	2.123693	4.081183	3.98409	4.627668	9.19E-19	-1.098341069	0.03362042	-0.278165935
ENSG00000144824	PHLDB2	10.124587	9.226606	8.755781	11.222571	10.468048	10.563351	7.58158	6.957704	7.905124	0.0000006	0.343697335	0.000459736	-0.27769821
ENSG00000040633	PHF23	43.037128	39.709883	38.628707	41.990775	41.947262	51.95451	30.282509	33.519304	29.053147	0.237614106	0.095922611	0.000712808	-0.277646871
ENSG00000075415	SLC25A3	230.174279	247.587963	253.199366	206.992402	220.745866	181.741092	176.651957	182.73767	174.812876	0.096686289	-0.09511562	0.000000707	-0.277643186
ENSG00000198604	BAZ1A	22.824488	22.179673	24.013643	24.311361	22.738416	21.631462	17.129626	16.612035	20.136928	0.04263659	0.139977996	0.000193826	-0.27756983
ENSG00000163781	TOPBP1	28.11982	29.724359	28.436711	26.994941	27.082097	26.762395	22.152799	20.742368	23.191527	0.433714717	0.050724924	0.00000634	-0.277407671
ENSG00000182010	RTKN2	2.815994	3.469342	3.516677	2.851822	2.7375	2.674779	2.912589	2.29784	2.670167	0.280414949	-0.155904232	0.040379517	-0.277375289
ENSG00000143878	RHOB	61.936321	55.111801	52.156319	34.456299	34.343552	35.567482	40.140385	43.758041	47.093903	2.2E-15	-0.549701547	0.001037489	-0.277286323
ENSG00000171827	ZNF570	1.186572	1.125333	1.267209	1.086101	0.935711	0.695505	0.950818	0.906612	0.66361	0.415599268	-0.189738459	0.224634517	-0.277166611
ENSG00000101557	USP14	19.176415	20.376865	22.239256	19.271067	17.733387	17.650478	15.977709	15.019072	17.255485	0.867698761	0.018528389	0.000798007	-0.277100827
ENSG00000183520	UTP11	15.1592	15.970949	18.096287	14.839952	15.683904	15.397053	12.982081	12.745636	12.303745	0.703018754	0.048668647	0.007578005	-0.27708363
ENSG00000168758	SEMA4C	21.329845	19.622263	18.778418	17.731436	16.663516	17.877739	14.807277	14.427412	17.37123	0.372538841	-0.078444692	0.001050987	-0.277036965
ENSG00000188868	ZNF563	1.310404	1.249188	1.316288	1.743344	1.193534	1.562368	1.055345	0.950475	0.790323	0.210298734	0.346292016	0.329480569	-0.276904707
ENSG00000163412	EIF4E3	0.111467	0.114295	0.100774	0.156573	0.093168	0.110559	0.104061	0.082728	0.075112	0.564459985	0.28094353	0.54237339	-0.276833789
ENSG00000139218	SCAF11	15.639979	16.836843	17.800657	20.163157	18.802804	18.804783	14.394816	13.328499	15.458762	0.000000047	0.345692687	0.000129524	-0.276712285
ENSG00000181830	SLC35C1	17.626694	15.350608	15.222352	17.230014	18.540249	18.756054	10.939059	12.600105	13.547263	0.0000468	0.310805106	0.003886037	-0.276676992
ENSG00000065243	PKN2	8.781041	8.531306	8.494651	9.475586	9.224714	9.612359	6.473255	6.312466	6.792489	0.00000566	0.308948995	0.000475023	-0.276592668
ENSG00000126945	HNRNPH2	34.328991	33.24757	37.905628	34.306221	32.429634	33.722404	29.207207	26.747744	25.615602	0.368106683	0.076851577	0.001802277	-0.276547347
ENSG00000126790	L3HYPDH	4.643397	6.152724	4.596389	7.009311	5.293505	5.519944	3.307585	3.64959	3.920614	0.011568741	0.435985906	0.15001165	-0.276435375
ENSG00000163946	FAM208A	11.143705	12.007848	11.815555	11.685164	11.307164	11.193105	8.943772	8.601103	10.009118	0.104280493	0.115019697	0.000226806	-0.276345165
ENSG00000137831	UACA	6.926789	7.597128	7.890471	5.4474	5.282691	5.193	6.051776	5.632635	5.779716	0.00000752	-0.34180154	0.001023972	-0.276249923
NewGene_1392	--	4.031872	5.088303	5.145508	5.084263	4.231423	4.810915	3.580198	3.479426	4.445648	0.556959739	0.094904202	0.074363216	-0.276241522
ENSG00000172493	AFF1	4.173111	4.374888	4.21379	6.432629	5.891294	5.830319	3.410093	3.083438	3.403074	4.22E-21	0.633048986	0.001358174	-0.276077698
ENSG00000129691	ASH2L	21.026684	20.627521	20.111328	19.598257	19.528359	18.194508	15.494452	14.862337	17.137965	0.937715405	0.008412046	0.00030442	-0.276055357
ENSG00000159256	MORC3	7.589494	7.723414	8.646702	8.344566	8.031794	8.161855	5.61847	5.943706	6.951732	0.047601148	0.183053681	0.009071772	-0.27594947
ENSG00000122884	P4HA1	11.519199	7.839372	5.723128	4.294774	4.508181	3.852464	6.662087	6.827031	5.901235	0.017913498	-0.8365442	0.462997614	-0.275860993
ENSG00000136883	KIF12	8.175212	7.562183	6.744027	7.006129	6.896255	6.956808	5.812015	5.835488	5.605094	0.679761472	0.072145488	0.051938059	-0.275787838
ENSG00000156304	SCAF4	18.553044	18.525308	19.292828	21.030167	19.015728	18.538895	13.99724	13.095087	16.714958	0.007268599	0.191939659	0.000838528	-0.27577261
ENSG00000162601	MYSM1	7.86878	9.439842	10.392829	9.154479	8.42723	7.371226	7.256458	7.044827	7.609227	0.837313341	-0.029525198	0.005597655	-0.275623873
ENSG00000165782	PIP4P1	25.301126	24.378196	23.639535	26.807027	27.739725	29.32143	17.610051	19.419396	19.491142	0.00000109	0.371794852	0.002577264	-0.27553901
ENSG00000181026	AEN	8.998233	10.650183	10.583922	8.551648	8.721647	8.356333	7.59518	7.729313	7.959993	0.393465517	-0.100939614	0.008403195	-0.275522895
NewGene_1537	--	1.351125	1.619458	1.215321	0.737395	0.983222	0.695746	1.340047	1.201573	0.748088	0.040132031	-0.815693495	0.533310224	-0.275512491
ENSG00000167799	NUDT8	5.447296	4.500121	3.559239	6.063947	5.776081	7.392946	3.195073	3.917886	3.296881	0.00378436	0.65615195	0.321947789	-0.275392387
ENSG00000205269	TMEM170B	5.329626	5.56582	5.406713	6.131405	6.256524	5.932603	4.344139	3.752346	4.55503	0.0000067	0.315667926	0.001403468	-0.275392365
ENSG00000137807	KIF23	16.740611	17.699213	18.402526	12.627468	10.70845	11.410574	13.285097	12.869013	14.638719	6.14E-10	-0.458803271	0.000289874	-0.275330773
ENSG00000056998	GYG2	9.836782	7.840125	7.620155	6.764293	5.180162	5.308904	6.666521	5.824605	6.599442	0.000544735	-0.382528735	0.014196587	-0.27530076
ENSG00000163904	SENP2	12.395316	13.043357	12.043711	12.452411	11.845726	11.834028	10.761643	9.227356	9.663464	0.264590013	0.085274788	0.000391865	-0.275278632
ENSG00000266338	NBPF15	8.080108	8.229368	8.5027	6.802625	5.866437	6.150558	6.541233	5.933328	6.792808	0.00391066	-0.248253046	0.001246351	-0.275172101
ENSG00000187045	TMPRSS6	14.228948	14.267406	11.642507	10.469755	11.019632	10.708841	9.818449	10.245835	10.9117	0.091996712	-0.176700558	0.006680406	-0.275105874
NewGene_1123	--	0.283129	0.514988	0.392936	0.452099	0.314759	0.437984	0.302642	0.171712	0.459903	0.745077666	0.158637938	0.558412453	-0.274977487
ENSG00000109066	TMEM104	11.650986	11.596931	11.794351	9.249713	9.415072	10.089858	8.383003	8.236522	9.717648	0.094583954	-0.133595315	0.001119209	-0.274546057
ENSG00000267952	AC008878.1	11.842075	14.127746	14.85846	2.045705	0.436955	0.636555	13.794115	16.832027	0.385915	9.29E-26	-3.555146705	0.864445154	-0.274446142
ENSG00000078699	CBFA2T2	3.485608	3.411618	4.160454	4.709847	4.075732	3.04351	3.188735	2.860238	3.61795	0.752785917	-0.048328228	0.015111281	-0.274231829
ENSG00000102241	HTATSF1	15.60991	18.178366	17.451937	15.796483	14.045291	14.763261	13.735442	12.437169	13.070773	0.773400414	-0.032498116	0.002487211	-0.274098579
ENSG00000196074	SYCP2	2.523878	2.820455	3.316808	5.000613	4.203787	4.220526	2.443475	1.909313	2.294648	1.46E-10	0.765762963	0.067580169	-0.274034321
ENSG00000120324	PCDHB10	0.427533	0.445733	0.561984	0.292413	0.368598	0.268614	0.277836	0.350221	0.484035	0.245838001	-0.475867827	0.493480799	-0.273877644
ENSG00000106080	FKBP14	6.440122	7.022857	8.99873	6.687224	6.455921	6.482181	5.86308	5.484823	5.927076	0.847337056	-0.032397992	0.027158794	-0.273785844
ENSG00000165410	CFL2	13.999071	14.31005	14.572442	11.576939	11.815817	12.840939	12.672911	12.377282	11.987582	0.402373996	-0.067303969	0.000244204	-0.273747854
ENSG00000099814	CEP170B	6.839898	6.90713	6.477223	6.247507	6.163134	6.60726	5.196647	4.589243	5.952112	0.555986511	0.057608225	0.00350719	-0.273538569
ENSG00000174945	AMZ1	2.522614	2.909281	2.468288	0.773282	0.648548	0.878374	1.980874	1.818352	2.445247	2.44E-37	-1.820524864	0.046865724	-0.272879439
ENSG00000136546	SCN7A	0.801497	0.998882	1.054323	1.166397	1.484969	1.275911	0.743347	0.6737	0.854492	0.000352578	0.591191927	0.151886009	-0.272800452
ENSG00000152102	FAM168B	60.523188	59.459534	58.900344	51.056757	47.909158	51.094559	46.412458	43.393828	49.980323	0.035251641	-0.110404437	0.00000364	-0.272714636
ENSG00000132199	ENOSF1	10.628934	11.539602	12.077148	9.802446	8.59724	8.640378	10.77328	9.443151	10.426984	0.032552828	-0.200393313	0.002565161	-0.272687621
ENSG00000175054	ATR	5.182025	4.916567	5.385054	5.616273	5.46543	5.00786	4.108723	3.872182	4.246677	0.042587072	0.178186752	0.001358174	-0.272669337
ENSG00000143776	CDC42BPA	6.03738	6.25715	6.565822	5.907611	5.493961	5.685781	4.985355	4.44786	5.446487	0.449210732	-0.061788661	0.000565307	-0.272014895
ENSG00000136560	TANK	33.555041	33.708112	37.279195	41.861535	34.725115	32.000056	27.061817	25.043906	30.252034	0.13362872	0.120328389	0.002625799	-0.271895704
ENSG00000215421	ZNF407	2.520923	2.321665	2.506923	2.187788	2.149298	1.944818	1.971977	1.62592	2.158616	0.694876649	-0.058121917	0.023447286	-0.271866625
ENSG00000145495	6-Mar	28.101411	27.898604	31.116582	27.564998	26.496315	24.532315	26.912746	24.87966	28.509832	0.814234356	-0.0197322	0.0000108	-0.271811032
ENSG00000134644	PUM1	26.543811	27.153638	26.539789	26.86137	25.175977	25.391069	20.978132	19.484249	21.536635	0.350687823	0.060547544	0.00000696	-0.271624342
ENSG00000115904	SOS1	4.2353	4.460539	4.772123	8.104323	7.378412	7.177573	3.209701	3.368366	4.118821	8.83E-32	0.906278634	0.010704159	-0.271557574
ENSG00000118263	KLF7	1.846876	1.756262	1.624514	1.233495	1.211499	1.154527	1.429281	1.677025	1.596728	0.000151681	-0.473465471	0.034560767	-0.271520876
ENSG00000144115	THNSL2	2.675205	2.40326	2.411712	2.629611	2.445193	2.133087	1.717952	2.527639	2.499535	0.671836282	0.092410835	0.138846017	-0.271344868
ENSG00000122687	MRM2	38.430763	37.510491	34.343093	38.933188	37.232113	32.194571	30.828038	29.743987	29.634828	0.071414644	0.122329837	0.0000836	-0.271038565
ENSG00000170537	TMC7	8.008384	7.526382	9.061974	6.041475	5.47211	5.318035	6.269274	6.36382	6.585937	0.00000407	-0.424395056	0.005595606	-0.270932285
ENSG00000125434	SLC25A35	3.495312	3.515003	3.209019	2.717759	3.417904	2.567704	2.218122	2.491895	2.494761	0.920275254	-0.029974701	0.125941836	-0.270540725
ENSG00000163961	RNF168	4.369002	4.232767	4.694901	4.426667	4.427464	4.036711	3.617785	3.05042	3.717167	0.417377321	0.09589844	0.013469351	-0.270275068

Gene ID	Gene name	Ctrl-1 FPKM	Ctrl-2 FPKM	Ctrl-3 FPKM	IFNγ-1 FPKM	IFNγ-2 FPKM	IFNγ-3 FPKM	Sora-1 FPKM	Sora-2 FPKM	Sora-3 FPKM	Ctrl vs IFNγ DESeq2 FDR	Ctrl vs IFNγ DESeq2 log2FC	Ctrl vs Sora DESeq2 FDR	Ctrl vs Sora DESeq2 log2FC
ENSG00000133424	LARGE1	8.960921	8.294761	7.77839	7.089441	7.222437	6.854518	6.921071	6.222134	6.544195	0.342738219	-0.103225197	0.006085631	-0.270256509
ENSG00000256060	TRAPPC2B	8.811806	8.74576	8.219075	8.264508	8.815354	7.398912	6.533853	7.489764	5.706213	0.764941712	0.06749782	0.167760137	-0.270135656
ENSG00000169760	NLGN1	0.32523	0.192919	0.170363	0.164879	0.106989	0.14633	0.176508	0.121757	0.197964	0.66862188	-0.208188002	0.520667268	-0.270082631
ENSG00000148297	MED22	19.545022	17.981893	17.941897	14.900185	14.323629	14.247411	13.795391	13.804137	15.383992	0.004963927	-0.199820107	0.0003833	-0.270067189
ENSG00000145248	SLC10A4	1.293369	1.352697	1.747198	0.531312	0.748705	0.759754	1.17394	0.854883	1.405636	0.002260255	-0.950672483	0.39065115	-0.269905777
ENSG00000143379	SETDB1	19.997679	19.615284	20.102503	21.400316	18.700633	19.321716	15.501188	15.02275	15.071577	0.032956955	0.151971321	0.00010601	-0.269875509
ENSG00000136643	RPS6KC1	9.51047	9.789369	9.870707	10.337395	10.68426	9.987214	7.400495	7.816619	8.313685	0.007444225	0.203963799	0.00071192	-0.269550501
ENSG00000043093	DCUN1D1	11.330535	13.13911	13.855104	11.570465	11.262237	11.610704	10.355242	9.873223	9.725057	0.970155638	0.004924824	0.003083868	-0.269522196
ENSG00000114857	NKTR	13.089479	15.211011	16.495442	16.016639	14.388391	12.799435	11.942206	11.119851	11.877195	0.37464085	0.098010436	0.002988225	-0.269251576
ENSG00000158555	GDPD5	0.949339	1.199884	1.218173	0.533634	0.673154	0.739478	0.987186	0.752297	0.88026	0.002977981	-0.723624684	0.259232095	-0.269242164
ENSG00000116954	RRAGC	16.525438	16.122389	16.217388	16.876045	17.310184	17.232565	12.583943	11.5012	14.015724	0.002639718	0.221057201	0.001714643	-0.26860686
ENSG00000198053	SIRPA	1.975261	1.947116	1.555741	0.860573	0.847179	0.959994	1.419708	1.145309	1.721295	0.000000273	-0.894827251	0.168222931	-0.268477029
ENSG00000185722	ANKFY1	12.943248	12.988218	12.885546	19.066659	16.806653	16.81356	10.139336	9.626194	10.951493	3.81E-24	0.581135104	0.000151612	-0.26843328
ENSG00000153208	MERTK	1.682984	1.085959	1.214196	0.804345	0.738623	0.716361	0.889154	0.819164	1.269517	0.003988345	-0.661444986	0.280559396	-0.26840183
ENSG00000014123	UFL1	11.933441	12.533934	12.839611	14.609855	13.35589	13.56146	9.948439	8.9614	10.164462	0.0000131	0.301454316	0.000768577	-0.26839885
ENSG00000091129	NRCAM	3.50909	3.57826	3.385752	3.912623	3.800838	4.119761	2.718467	2.691977	2.818911	0.00278534	0.296809792	0.009981947	-0.26805605
ENSG00000103274	NUBP1	16.68918	16.089772	16.741792	15.686561	15.218408	14.221092	11.283502	13.432836	13.763432	0.995202877	-0.001055238	0.024571815	-0.267409648
ENSG00000155097	ATP6V1C1	24.427282	25.413241	26.468139	23.389855	22.769248	22.599927	19.884943	19.772191	22.015799	0.984331945	0.001992597	0.000120519	-0.267337882
ENSG00000256223	ZNF10	5.411167	6.513177	6.743485	5.734881	4.711036	4.931653	4.189598	4.183225	3.614713	0.984928337	0.015014086	0.66790766	-0.267171587
NewGene_4139	--	3.012052	3.555347	3.179518	3.40284	3.227448	3.078609	2.865677	2.209517	2.398783	0.154053349	0.188983403	0.068308757	-0.267070541
NewGene_2499	--	0.374686	0.704172	0.455843	0.314776	0.452043	0.304105	0.376741	0.336966	0.48722	0.467370774	-0.373455667	0.558431134	-0.267009687
ENSG00000198746	GPATCH3	11.611341	10.828191	10.901568	10.728571	11.324607	11.125043	8.785463	8.182786	8.855094	0.185525263	0.139322269	0.008082064	-0.266946471
ENSG00000163625	WDFY3	1.665864	1.64065	1.742847	1.471777	1.606026	1.567347	1.35337	1.215423	1.346517	0.845473982	0.027254768	0.011126888	-0.266867241
ENSG00000034693	PEX3	9.386619	10.215036	10.323432	8.046802	8.579698	8.067259	7.056071	8.674173	7.638934	0.458609021	-0.10315565	0.041537872	-0.266822934
ENSG00000187079	TEAD1	11.617913	11.397782	11.422106	8.430129	8.06084	7.955609	8.459389	8.10508	9.875844	0.000222686	-0.264711617	0.000620567	-0.266769301
ENSG00000115271	GCA	10.986712	10.816129	11.870861	12.342929	10.14265	11.057807	9.018916	7.748603	9.264544	0.391718309	0.107712007	0.01528844	-0.266678524
ENSG00000078081	LAMP3	0.479212	0.616242	0.803981	4.033069	4.046193	4.543286	0.529919	0.471419	0.467286	9.93E-68	2.881059291	0.440236835	-0.266648825
ENSG00000157741	UBN2	1.983675	2.21155	2.436433	2.363227	2.090308	2.288613	1.690657	1.759201	1.931347	0.106634103	0.169578289	0.01236302	-0.266463541
ENSG00000109089	CDR2L	2.394387	1.91588	1.713208	1.03984	0.800135	1.381212	1.677428	1.454796	1.573935	0.000703452	-0.753296693	0.170801085	-0.266323386
ENSG00000116679	IVNS1ABP	41.055029	40.440712	41.286893	34.119211	32.867569	32.215024	31.734343	31.611621	34.455317	0.000910217	-0.179591778	0.00000865	-0.266299824
ENSG00000213214	ARHGEF35	6.29737	5.658771	5.531853	7.166724	6.816573	6.604109	4.467869	4.400541	4.782777	0.000732913	0.379851527	0.038143559	-0.26626917
ENSG00000147912	FBXO10	4.299946	3.944889	3.76197	3.659161	3.387765	3.724056	3.077343	2.857437	3.701416	0.831879085	-0.033884767	0.033186067	-0.266256319
ENSG00000145321	GC	506.637238	423.888794	410.488708	480.884644	532.031067	482.329041	339.835266	359.440613	345.445435	0.000113956	0.304825613	0.001649324	-0.266184664
ENSG00000155008	APOOL	5.212342	5.348281	4.983824	4.684112	2.939893	4.42825	4.009646	4.20514	3.946861	0.628370944	0.063339519	0.017122413	-0.266102065
ENSG00000011523	CEP68	2.58024	2.544783	2.121393	2.104353	2.019358	2.206971	1.688707	1.840921	2.034552	0.713663211	-0.063900395	0.06195269	-0.266048518
ENSG00000154914	USP43	0.918298	0.763459	0.771298	0.67993	0.615736	0.426486	0.762824	0.516701	0.527016	0.423851275	-0.251149489	0.351089451	-0.265975237
ENSG00000138193	PLCE1	0.438118	0.395278	0.530061	0.313626	0.248979	0.251776	0.348554	0.349505	0.37474	0.005215768	-0.594285158	0.200565828	-0.265971451
ENSG00000122783	CYREN	38.714971	34.929974	33.072774	27.62666	26.550662	27.213935	26.819375	26.679213	31.522929	0.001688576	-0.256191252	0.003406165	-0.265961304
ENSG00000169855	ROBO1	45.213673	49.714927	50.383947	46.35466	41.312076	44.900698	35.670117	32.407994	40.695683	0.078286774	-0.094083412	0.0000149	-0.265892056
ENSG00000135945	REV1	10.67474	10.328374	11.208995	11.090161	10.073169	9.459406	8.531494	7.87078	8.396528	0.52240999	0.06625814	0.000925855	-0.265876339
ENSG00000153157	SYCP2L	3.437931	3.558026	4.507057	2.374432	2.360151	2.146875	3.303099	2.948294	2.700589	0.0000507	-0.605544619	0.105954716	-0.26555403
ENSG00000105426	PTPRS	5.75985	5.313866	3.93137	5.108802	4.736757	5.102972	4.302138	3.508939	4.199033	0.431331381	0.107892836	0.030539035	-0.26542181
ENSG00000197885	NKIRAS1	2.218544	2.803603	2.906191	3.115334	2.670342	1.984914	2.080287	2.192621	1.854113	0.748994571	0.097255687	0.252952713	-0.265411064
ENSG00000136932	TRMO	9.087691	8.257308	6.534856	7.615686	8.328714	6.310561	7.449382	6.473348	6.822615	0.774032002	0.055521799	0.07020796	-0.265348717
ENSG00000178662	CSRNP3	0.304257	0.313199	0.280533	0.248504	0.26875	0.290394	0.335611	0.251987	0.492716	0.398174776	-0.228968533	0.29167376	-0.26530807
ENSG00000114670	NEK11	0.642033	0.765507	0.448901	0.447902	0.299304	0.372835	0.372084	0.489262	0.501194	0.251717869	-0.474411502	0.512383829	-0.265111311
ENSG00000176853	FAM91A1	15.75036	17.993416	17.683671	17.650139	16.843217	16.963172	13.567649	12.775964	14.170178	0.076822329	0.129792387	0.000528494	-0.26501636
ENSG00000100441	KHNYN	15.199859	12.683942	12.121359	12.719187	12.67201	12.373079	10.026839	9.894597	10.760663	0.311195941	0.099895469	0.003273565	-0.265007764
ENSG00000126458	RRAS	7.26896	7.263591	6.597016	3.805807	4.782497	3.987281	5.893225	5.437939	5.132542	0.000706332	-0.600416046	0.131032961	-0.264794915
ENSG00000122966	CIT	4.456526	4.636256	4.567508	3.352902	2.57712	2.853386	3.607758	3.229213	3.604025	0.000000832	-0.466456985	0.002507026	-0.26437359
NewGene_315	--	2.379638	2.620657	2.407238	1.557067	1.438889	1.351698	1.497324	1.771608	2.492406	0.160977962	-0.615563154	0.541080343	-0.264239522
ENSG00000247626	MARS2	12.73353	13.31196	13.098492	11.444432	11.183459	12.112073	9.774161	10.081111	10.704371	0.834756165	-0.02398807	0.001830351	-0.264099669
NewGene_6359	--	0.318184	0.404511	0.266206	0.66223	0.485889	0.360312	0.297242	0.299858	0.165755	0.042219385	0.750749403	0.568761302	-0.264076833
ENSG00000112739	PRPF4B	30.891759	32.498115	32.719658	31.788721	29.363124	28.200593	26.523079	27.204095	28.457749	0.216059167	0.092296039	0.0000935	-0.264017141
ENSG00000186088	GSAP	7.445548	7.221216	7.799028	5.752267	5.83978	5.781944	5.903936	5.515973	6.375731	0.025725055	-0.22597936	0.01006814	-0.263880567
NewGene_4416	--	0.647222	1.160098	1.419129	0.952962	0.812054	0.743237	1.085802	1.455364	0.940041	0.237136737	-0.467335934	0.512198389	-0.263725073
ENSG00000163626	COX18	2.518573	3.334161	2.642353	2.551592	3.210388	2.417128	2.455587	2.215088	2.33789	0.435296025	0.108303648	0.03460399	-0.263591627
ENSG00000244754	N4BP2L2	10.164591	10.861865	10.649111	10.526689	9.894819	9.876027	8.535964	8.066884	9.640238	0.701726103	-0.036269596	0.000196081	-0.263564936
ENSG00000143036	SLC44A3	9.4901	8.583253	9.194774	5.620138	5.886218	5.51514	6.85203	6.841075	7.107587	0.0000044	-0.50644498	0.017561286	-0.263513962
ENSG00000196912	ANKRD36B	0.309694	0.303593	0.447322	0.542422	0.523438	0.33574	0.221708	0.259305	0.392057	0.151946432	0.437737894	0.43531583	-0.263338273
ENSG00000100422	CERK	15.626547	13.952335	14.281312	9.263295	9.064672	9.178178	11.678693	10.679836	11.944727	9.33E-14	-0.526401098	0.000947486	-0.263253377
ENSG00000103657	HERC1	3.110992	2.873848	2.849757	2.973806	3.101431	2.958836	2.305732	1.971137	2.44481	0.023593733	0.187596593	0.005448759	-0.263246484
ENSG00000131899	LLGL1	10.322361	10.095105	8.539516	9.169009	8.655747	9.263174	7.08256	6.87744	8.74793	0.676662538	0.051156933	0.015657925	-0.263129883
ENSG00000198203	SULT1C2	30.278132	30.23651	24.368589	25.575169	25.496098	25.039834	26.487727	26.546206	26.486678	0.039014774	0.161652173	0.001336005	-0.262861027
NewGene_5968	--	1.697962	1.807011	1.93967	1.757193	1.465357	1.835078	1.664177	1.642929	1.67057	0.227262571	0.203003296	0.080935632	-0.262657216
ENSG00000016082	ISL1	1.443027	1.778111	1.957633	0.822393	0.94964	0.925796	1.577535	1.296917	1.165164	0.000899971	-0.785390053	0.293482293	-0.262558663
ENSG00000100426	ZBED4	10.112207	9.763532	9.887991	9.169782	8.273011	9.316691	7.724048	6.950494	8.643281	0.959048469	-0.005905454	0.001089127	-0.26243225
ENSG00000180667	YOD1	11.472737	12.36702	13.049501	12.629187	12.331622	12.424479	9.349342	9.316338	10.188989	0.020698911	0.167156982	0.000694662	-0.262120301
ENSG00000115392	FANCL	29.042444	30.923445	31.33611	24.154676	23.292584	21.466764	22.456599	23.324611	26.743054	0.0000738	-0.301594057	0.001078981	-0.261883812
ENSG00000105866	SP4	5.911396	5.895164	6.060265	4.200743	3.781973	4.163393	4.580841	4.203058	5.259577	0.000000412	-0.411037327	0.006733625	-0.261845965
ENSG00000162889	MAPKAPK2	21.610057	20.658634	21.96715	18.783689	19.08493	18.571722	16.076416	15.511441	18.8136	0.714712773	-0.034409694	0.00138243	-0.261785693
ENSG00000155016	CYP2U1	1.029994	0.848992	1.059653	0.936986	0.98211	1.033963	0.707596	0.778997	0.81129	0.507512357	0.157862447	0.229699496	-0.261766996
ENSG00000157379	DHRS1	3.699681	3.232164	2.839706	3.12646	2.847089	3.014569	2.555869	2.252673	2.896965	0.868340752	0.046862283	0.235917639	-0.261611612
ENSG00000152137	HSPB8	2.285158	2.547777	2.686333	1.465208	1.332331	1.182624	1.883974	2.161896	1.821058	0.000139783	-0.770513856	0.211837567	-0.261519504

Gene ID	Gene name	Ctrl-1 FPKM	Ctrl-2 FPKM	Ctrl-3 FPKM	IFNγ-1 FPKM	IFNγ-2 FPKM	IFNγ-3 FPKM	Sora-1 FPKM	Sora-2 FPKM	Sora-3 FPKM	Ctrl vs IFNγ DESeq2 FDR	Ctrl vs IFNγ DESeq2 log2FC	Ctrl vs Sora DESeq2 FDR	Ctrl vs Sora DESeq2 log2FC
ENSG00000198663	C6orf89	20.944038	19.336628	24.496056	20.052081	20.170177	19.565923	16.214038	17.471404	15.790946	0.05187456	0.134022783	0.000161999	-0.261475707
ENSG00000010318	PHF7	0.951745	0.713793	1.035996	0.979711	1.079597	0.579577	0.696138	0.814897	0.759565	0.969917136	0.020417434	0.459468556	-0.261429158
ENSG00000176058	TPRN	25.915406	23.780843	21.142055	26.524546	27.654242	26.229646	18.488642	19.483365	16.478428	0.000017	0.3550925	0.018737731	-0.261420649
ENSG00000156103	MMP16	1.347275	1.414322	1.600682	0.812723	0.99041	0.829358	0.98539	1.176632	1.249528	0.00000742	-0.579356545	0.054860825	-0.26135
ENSG00000012963	UBR7	9.465271	8.790388	8.494691	9.08246	8.934222	9.226677	7.586259	6.382316	7.005355	0.057672246	0.170933835	0.008907651	-0.261247194
ENSG00000092201	SUPT16H	78.841249	82.81895	84.582603	77.065012	72.944971	73.906038	64.909773	62.192856	70.096634	0.841894674	0.014841668	0.00000748	-0.261099921
ENSG00000107821	KAZALD1	2.32234	1.687049	0.991898	1.672606	1.497401	1.769316	1.449293	1.330768	1.114559	0.746141725	0.129376792	0.449471758	-0.26106424
ENSG00000126464	PRR12	5.742664	5.793066	5.348053	5.593794	5.072479	5.059615	4.426418	3.856117	4.961544	0.686936471	0.044413339	0.007163333	-0.260972277
ENSG00000100084	HIRA	25.781216	23.062405	24.19466	25.367655	23.075109	24.333277	17.874498	17.319553	21.991859	0.044239773	0.144885722	0.00289441	-0.260923899
ENSG00000139737	SLAIN1	10.466416	8.564008	10.824567	6.885196	7.082788	7.098847	7.454029	7.213238	8.497221	0.000632535	-0.364468532	0.023327344	-0.260903336
NewGene_2277	--	3.246243	2.994739	3.025124	2.581853	2.594285	2.59655	1.694216	2.62012	3.121589	0.529882474	-0.108152827	0.163219855	-0.260771663
ENSG00000132485	ZRANB2	25.586724	28.616817	29.905969	27.242439	24.886333	25.220065	21.399036	21.425366	23.237622	0.790538783	0.028033791	0.001140752	-0.260738011
ENSG00000188157	AGRN	28.322572	25.239165	23.987634	24.046102	20.769071	24.842333	18.160193	18.809856	24.071624	0.94471444	-0.008750259	0.007535803	-0.260674863
ENSG00000262246	CORO7	4.264696	3.906849	3.847909	3.109584	3.853119	3.172595	3.028307	2.925661	3.537301	0.555063429	-0.101118241	0.046668803	-0.260555876
ENSG00000177663	IL17RA	1.824989	1.597454	1.387946	1.437557	1.453349	1.487886	1.24868	1.190584	1.329532	0.95551367	0.011906968	0.069989024	-0.260503375
ENSG00000135709	KIAA0513	0.948217	1.286201	0.865185	0.86591	0.701528	1.18395	0.655873	0.622902	0.701162	0.883333394	0.04080222	0.263397682	-0.26048686
ENSG00000126062	TMEM115	15.108826	13.24335	13.731682	13.821918	12.430162	13.641257	10.667215	10.134218	12.230963	0.556509409	0.069283374	0.01076428	-0.260375316
ENSG00000132436	FIGNL1	11.439676	11.636343	12.105038	10.206082	8.968521	9.581908	9.847437	8.474776	9.30975	0.072675048	-0.157166181	0.003269167	-0.260359939
ENSG00000110344	UBE4A	13.269594	13.846177	14.230104	13.674192	12.726347	12.583271	10.509013	9.993033	11.941192	0.440424068	0.061187018	0.000586935	-0.260265297
ENSG00000160870	CYP3A7	3.018867	3.307012	3.174417	1.759156	2.013387	1.56144	2.291571	2.709548	2.388984	0.000361664	-0.683386635	0.176708755	-0.259739546
ENSG00000138439	FAM117B	4.782675	4.620669	5.199978	7.407526	7.731482	7.869551	3.851242	3.433878	4.176818	9.54E-25	0.803543833	0.011960331	-0.259718878
ENSG00000107130	NCS1	7.402364	6.644639	6.888227	4.044116	3.718448	4.178171	5.274461	4.977647	6.181667	4.16E-14	-0.662262186	0.008957021	-0.259628956
ENSG00000100697	DICER1	9.474883	10.668355	10.120585	10.388606	9.939789	9.629101	8.045989	7.521064	8.521808	0.075562191	0.127706396	0.000437367	-0.259599297
ENSG00000160606	TLCD1	22.21563	21.87247	20.72258	18.116733	19.8314	19.774289	15.440552	16.60424	18.743917	0.80407858	-0.035603568	0.019965782	-0.259547175
ENSG00000164466	SFXN1	12.94898	14.244497	14.073836	13.747287	14.097085	13.821596	10.801965	10.410385	11.238727	0.040662741	0.159721479	0.001092311	-0.259238078
ENSG00000165275	TRMT10B	3.162339	3.468098	3.613426	3.823659	3.439965	3.710449	3.047559	3.16996	2.74804	0.261893748	0.199876313	0.141934195	-0.259103199
ENSG00000196586	MYO6	12.61084	11.851707	11.631212	11.150645	10.05784	10.141957	9.657217	9.345723	9.92998	0.338497714	-0.075403608	0.000338913	-0.259052091
NewGene_3019	--	3.743114	4.275728	3.701051	4.293888	4.332305	4.089022	3.226798	3.051983	3.038793	0.03241085	0.257622231	0.04464485	-0.258975773
ENSG00000074755	ZZEF1	6.572996	6.519274	6.403447	7.021495	7.08994	6.447721	5.516179	4.587618	5.559104	0.001560552	0.218926947	0.001659812	-0.258959533
ENSG00000141295	SCRN2	6.105815	6.452043	6.013162	8.427312	7.715008	7.911137	4.641939	4.303601	5.430565	0.00000571	0.527329424	0.080925359	-0.258929146
ENSG00000164048	ZNF589	1.648083	1.743559	1.400627	1.229096	1.046854	1.3205	1.359122	1.199216	1.241174	0.397661008	-0.197026049	0.198573264	-0.258753235
ENSG00000070718	AP3M2	5.217995	6.866026	6.626827	4.340176	4.175573	4.765921	4.708816	4.928009	4.942058	0.007707761	-0.33522306	0.044000829	-0.258725977
ENSG00000240857	RDH14	7.703228	8.720541	9.702283	9.182866	9.505827	9.167296	7.504425	6.219835	6.756025	0.0706048	0.240508839	0.073016426	-0.258709183
ENSG00000163848	ZNF148	6.362073	6.165723	6.567144	6.770603	6.055545	7.229546	5.107925	5.208315	5.209134	0.406031335	0.073838011	0.000691358	-0.258654571
ENSG00000275832	ARHGAP23	1.43206	1.326734	1.737833	0.908962	0.585097	1.027702	1.334147	0.9635	1.431099	0.00000781	-0.836579979	0.147014155	-0.258572303
ENSG00000144035	NAT8	25.706051	22.541121	20.450926	20.968225	21.533579	24.649048	15.374388	18.208403	20.35984	0.418813299	0.115183337	0.055023486	-0.258411022
ENSG00000165060	FXN	4.44437	4.877629	4.711224	2.679006	3.977721	2.260598	4.627696	3.822292	3.882063	0.085796738	-0.256646783	0.06338583	-0.258405141
ENSG00000169217	CD2BP2	23.642113	21.788575	22.149336	16.140374	18.810899	18.01453	17.436944	17.06812	18.169189	0.012386976	-0.200365026	0.000339269	-0.258229407
ENSG00000260230	FRRS1L	1.283647	1.90262	1.672008	3.01711	2.696356	2.880471	1.529153	1.01373	1.216916	5.87E-16	0.993805112	0.160496225	-0.258200926
ENSG00000162636	FAM102B	0.630046	0.639865	0.602861	0.623603	0.35677	0.395349	0.553107	0.473543	0.437496	0.332631414	-0.295765119	0.308064136	-0.258178236
ENSG00000049130	KITLG	2.140509	2.21415	2.636922	1.625809	1.700516	1.744418	1.71445	1.890578	2.019536	0.04767688	-0.289095825	0.077861507	-0.257952552
ENSG00000175137	SH3BP5L	17.945808	16.147991	15.446424	13.354363	13.579876	14.448951	12.85809	12.266418	13.771811	0.227262571	-0.112127975	0.002024642	-0.257917123
ENSG00000076067	RBMS2	3.39061	3.803554	3.766953	2.82835	2.789225	2.53216	3.08168	2.741478	3.029804	0.002091341	-0.292617492	0.008733602	-0.257857317
ENSG00000143093	STRIP1	7.229704	6.791496	6.918284	6.207079	6.457992	6.142863	5.066249	5.552714	5.815415	0.960142463	-0.007788154	0.016410812	-0.257815662
ENSG00000122644	ARL4A	40.801042	36.935781	37.747804	31.041266	18.620293	30.365053	34.993831	31.497853	28.766243	0.000521836	-0.25823609	0.004174307	-0.25775386
ENSG00000122970	IFT81	3.646632	3.775968	3.901175	2.323745	1.95941	2.099369	3.044995	2.604092	3.352404	0.00000122	-0.676922557	0.073751666	-0.257726906
ENSG00000183723	CMTM4	10.16677	10.405949	10.885967	7.318427	7.025565	7.350111	8.456583	8.001131	7.928423	0.0000289	-0.372438313	0.007268244	-0.257641447
ENSG00000156052	GNAQ	10.288047	10.418904	11.378107	9.134232	9.052913	8.803307	8.463339	8.455776	8.227995	0.206127414	-0.10188932	0.001321474	-0.257598138
ENSG00000181472	ZBTB2	17.683897	17.754257	18.188833	15.387177	15.587193	15.322371	14.5866	13.306458	14.177153	0.452438498	-0.064461141	0.000799765	-0.257511305
ENSG00000155850	SLC26A2	1.00453	0.81204	0.845661	0.713369	0.828643	0.688736	0.661868	0.600812	0.874693	0.632376587	-0.108765201	0.201168754	-0.257093811
ENSG00000137413	TAF8	5.261186	5.270591	5.169113	5.535854	5.812213	5.771359	4.593035	4.137695	4.43806	0.015206113	0.215415998	0.006065068	-0.257046278
ENSG00000188368	PRR19	0.889759	1.053247	1.16656	0.270318	0.800807	1.213511	0.838838	0.804449	0.797091	0.613229658	-0.288905967	0.482880142	-0.257015732
ENSG00000182463	TSHZ2	0.354786	0.314865	0.301584	0.510803	0.550097	0.434757	0.236949	0.290487	0.247292	0.0000739	0.745376423	0.290772263	-0.256957221
ENSG00000205268	PDE7A	7.696058	8.096329	9.555036	6.31401	6.191974	5.874245	6.966075	6.286528	6.572457	0.005053863	-0.30454995	0.023111947	-0.256876372
ENSG00000114423	CBLB	6.536597	5.500571	6.082214	5.958864	5.636571	5.601488	5.501173	4.486348	5.03225	0.354397892	0.091805363	0.005056681	-0.256826223
ENSG00000144792	ZNF660	1.196015	0.791209	1.016331	1.22211	1.136333	1.104048	0.594439	0.531444	0.588847	0.526388098	0.83375445	0.838590824	-0.256795207
ENSG00000176293	ZNF135	9.319147	10.401077	9.173707	6.833091	6.294878	6.111401	7.718026	6.659969	7.474576	0.0000064	-0.406228385	0.007143502	-0.256733286
ENSG00000165475	CRYL1	7.122765	5.564614	4.41212	5.097248	4.98295	5.53542	4.475774	4.529243	4.497588	0.960908763	-0.014313681	0.199297874	-0.256327501
ENSG00000083642	PDS5B	11.752168	12.98943	13.273728	12.618239	12.294959	11.865754	9.415338	10.250459	10.37704	0.196138414	0.099869518	0.000856977	-0.256220957
ENSG00000185909	KLHDC8B	6.641919	4.234717	4.559246	4.066945	3.327197	4.541348	4.188021	4.529902	3.357149	0.321077266	-0.221698437	0.227797217	-0.256187815
ENSG00000187257	RSBN1L	6.132478	6.593406	7.292559	7.577868	6.482071	7.269307	5.348355	5.239527	5.421421	0.010458248	0.235195307	0.007149369	-0.256075628
ENSG00000142731	PLK4	8.172357	8.891386	9.036039	7.314697	6.360031	6.422052	6.522812	7.027259	6.579814	0.032792159	-0.221448789	0.015050179	-0.255785295
ENSG00000099364	FBXL19	14.547569	12.639703	12.234558	11.402125	10.03289	11.736254	8.421199	9.252105	12.11963	0.358778331	-0.098412441	0.032954651	-0.255725052
ENSG00000132256	TRIM5	16.900685	17.034113	18.536654	18.553727	16.859028	17.914244	13.267324	14.121832	14.3308	0.047463176	0.160581411	0.002828187	-0.25569742
ENSG00000139372	TDG	35.686771	34.471845	35.515805	30.771655	30.904977	30.877013	26.299221	27.003081	29.041571	0.5141941	-0.048347125	0.000107125	-0.255516574
ENSG00000114648	KLHL18	4.060298	3.446728	3.915339	3.492951	3.715576	3.064406	2.64393	2.911199	3.639825	0.973662659	-0.006693763	0.069499899	-0.255353166
ENSG00000145014	TMEM44	15.811564	14.587342	14.600023	9.393122	9.16745	10.096618	11.985683	10.612346	12.451646	0.000000011	-0.519043921	0.009078682	-0.255308203
ENSG00000148773	MKI67	14.670067	16.110201	14.610607	16.8432	13.929373	15.008398	11.70136	12.056439	12.109925	0.014498784	0.172828593	0.000028	-0.255087557
NewGene_4077	--	2.041386	1.71157	1.952433	1.930746	1.894911	2.08479	1.26735	1.666228	1.437187	0.66539522	0.137569485	0.372981731	-0.2547316
ENSG00000176055	MBLAC2	0.869743	1.046986	0.975585	1.324394	0.807098	0.867014	0.879032	0.622623	0.734354	0.785417546	0.088337508	0.308593273	-0.254705139
ENSG00000196290	NIF3L1	23.313074	22.806484	24.094601	21.858825	21.908145	19.166129	17.27045	20.242835	17.785588	0.561531714	-0.061741807	0.009890766	-0.254512324
ENSG00000117906	RCN2	39.803507	41.290562	46.470759	29.610213	29.855086	30.262023	30.095656	28.623847	33.121091	6.83E-11	-0.361775677	0.000088	-0.254455764
ENSG00000163947	ARHGEF3	2.565567	2.719121	2.430209	2.787296	2.709932	2.587826	2.145525	1.723833	1.96557	0.084357901	0.259029277	0.123438246	-0.25444915
ENSG00000122779	TRIM24	38.483715	34.377639	37.036461	41.399692	42.597191	40.105631	31.936669	28.585558	30.089873	0.000000195	0.268201917	0.000152238	-0.254446716

Gene ID	Gene name	Ctrl-1 FPKM	Ctrl-2 FPKM	Ctrl-3 FPKM	IFNγ-1 FPKM	IFNγ-2 FPKM	IFNγ-3 FPKM	Sora-1 FPKM	Sora-2 FPKM	Sora-3 FPKM	Ctrl vs IFNγ DESeq2 FDR	Ctrl vs IFNγ DESeq2 log2FC	Ctrl vs Sora DESeq2 FDR	Ctrl vs Sora DESeq2 log2FC
ENSG00000055332	EIF2AK2	2.765811	3.224899	3.194118	7.76151	6.6754	7.26465	2.279773	2.175667	2.309954	6.86E-82	1.480800425	0.013566509	-0.254310669
ENSG00000112144	ICK	3.044326	3.30156	3.920127	5.713777	5.174019	5.403521	2.947603	2.251096	2.900506	5.51E-17	0.812961879	0.06372022	-0.254108667
ENSG00000132170	PPARG	38.645706	38.878153	35.771335	39.576492	36.979468	38.430418	29.361334	30.248435	32.023362	0.051892082	0.139199634	0.000660733	-0.25381662
ENSG00000104356	POP1	5.406835	5.372767	5.513682	4.036254	4.243334	4.411028	4.074183	4.034909	4.836759	0.026785525	-0.221177993	0.012076794	-0.253691335
NewGene_5629	--	6.208466	7.020246	8.189694	5.965026	5.090834	4.939214	5.490622	5.747414	5.134407	0.043583243	-0.237397666	0.026234855	-0.253655059
ENSG00000164190	NIPBL	11.524709	11.492091	11.077608	11.383499	11.249254	10.206335	8.714765	8.306262	9.694376	0.270538263	0.079588223	0.000163356	-0.253402861
ENSG00000137947	GTF2B	10.784698	10.294749	11.239521	12.307434	12.202828	11.452569	7.511626	8.927594	9.00917	0.00778376	0.300942173	0.058767891	-0.253373367
ENSG00000243646	IL10RB	7.332767	8.080315	7.731634	9.413348	9.436726	9.131853	5.597085	6.199994	6.334149	0.000117246	0.405458499	0.047912766	-0.252732687
ENSG00000163697	APBB2	3.783047	3.923603	3.564281	4.472664	2.703085	2.401444	5.441072	3.146995	3.46048	0.0000905	-0.38716002	0.037656443	-0.252596211
ENSG00000107021	TBC1D13	15.265838	14.976133	13.005108	12.193891	12.441315	12.511986	10.865839	10.494946	12.722249	0.492927119	-0.070853185	0.007360554	-0.252335555
ENSG00000124831	LRRFIP1	20.724919	19.553183	22.426549	16.841254	16.892471	16.270868	15.20898	15.11932	18.233674	0.090199786	-0.137230332	0.006437622	-0.251874178
ENSG00000119979	FAM45A	11.483621	10.982181	9.379861	13.138955	12.73417	12.019224	9.436651	8.716561	8.442973	0.009589147	0.253876868	0.017253228	-0.251702265
ENSG00000167676	PLIN4	9.082645	8.601408	8.479231	7.858997	7.650113	8.225134	6.776175	6.314212	7.525133	0.945771509	0.007783929	0.002037839	-0.251369323
ENSG00000146859	TMEM140	18.661951	14.467421	15.178309	23.841354	25.17108	24.228407	12.41293	11.942734	13.777206	1.55E-15	0.746508413	0.029850141	-0.251276773
ENSG00000160588	MPZL3	7.992541	8.991907	10.224569	5.336902	6.091649	5.847295	6.49089	6.685363	8.042248	0.00000131	-0.508366826	0.029204981	-0.251190141
ENSG00000182107	TMEM30B	4.854844	5.539203	5.420018	3.535913	3.628274	3.584902	4.355074	3.792726	4.317556	0.0000545	-0.407723999	0.021223345	-0.251096821
ENSG00000182749	PAQR7	1.209546	0.993147	1.125439	1.164626	0.940968	0.84101	0.907245	0.775833	0.946577	0.928337486	-0.03195156	0.308593273	-0.250910058
ENSG00000143368	SF3B4	47.181059	47.465347	51.298412	46.341835	45.121425	46.01302	36.176153	40.406146	38.475763	0.417328491	0.060990792	0.001783057	-0.25068492
ENSG00000125877	ITPA	43.467708	44.213256	45.94968	30.833171	29.508836	32.162048	33.438029	34.768774	37.559132	0.000000117	-0.391955767	0.002217762	-0.2506548
ENSG00000111877	MCM9	4.053464	3.540877	3.851935	3.681674	4.678027	3.542995	3.146628	2.693652	3.100701	0.131727419	0.198943781	0.046595404	-0.250611029
ENSG00000155629	PIK3AP1	10.982096	9.880437	10.903354	26.870396	26.581356	25.596624	8.324275	7.569561	8.996178	1.39E-133	1.472104346	0.003615686	-0.250609813
NewGene_1530	--	2.410137	1.741641	1.804617	1.786169	1.369968	1.391393	1.557887	1.134958	1.991807	0.564345572	-0.239111482	0.50743062	-0.250562038
ENSG00000204713	TRIM27	43.338759	43.340278	42.389229	44.107079	43.942373	42.814778	33.424574	32.78535	35.540479	0.003224722	0.165844719	0.0000462	-0.250514452
ENSG00000116830	TTF2	2.62715	2.451328	3.023328	2.228959	2.106421	2.078279	2.030458	2.031632	2.349556	0.091473828	-0.196481489	0.031488597	-0.25050383
ENSG00000164647	STEAP1	6.267103	6.69436	5.663341	4.384846	4.475125	4.209736	4.691727	4.748088	5.142887	0.032872302	-0.358237146	0.1317182	-0.250387594
ENSG00000212123	PRR22	4.123126	3.856263	3.972691	3.596125	3.26644	3.394065	2.921865	2.862426	3.572897	0.802586886	-0.060078163	0.204499312	-0.250260225
ENSG00000131018	SYNE1	0.47194	0.6904	0.70628	1.695659	1.715741	1.613363	0.201373	0.264791	0.260693	8.89E-30	1.343042737	0.165454452	-0.250243588
ENSG00000153214	TMEM87B	2.408722	3.313641	3.263851	2.868635	2.669776	2.844504	2.418331	2.050944	2.638486	0.803266367	0.047538362	0.106123786	-0.250071998
ENSG00000188483	IER5L	11.564514	12.365029	11.591539	5.818842	6.020773	6.089685	7.849181	10.137575	9.993908	2.83E-22	-0.838075889	0.032532837	-0.249991082
ENSG00000271092	TMEM56-RWDD	1.361196	2.89216	0.718932	0.937356	1.296542	1.120205	1.298671	0.967933	1.093468	0.64925479	-0.228490655	0.561184797	-0.249678417
NewGene_4738	--	1.369093	1.42077	1.673928	2.641163	2.373441	2.671038	1.376352	1.073765	1.071991	0.0000194	0.927106636	0.421141076	-0.249501319
ENSG00000103043	VAC14	16.394528	15.857359	14.900701	13.610321	13.153049	14.270687	12.304593	11.315424	13.450397	0.61198155	-0.051771675	0.003965952	-0.249402755
ENSG00000143376	SNX27	7.439439	7.533692	8.303463	7.244481	6.494902	7.025743	6.077388	5.64899	6.682198	0.857705619	-0.020754122	0.00403084	-0.249151748
ENSG00000101849	TBL1X	6.277107	5.994333	5.972581	6.781976	6.844032	6.820597	4.649883	4.594454	5.259662	0.000119128	0.298300032	0.005761926	-0.249094019
ENSG00000168175	MAPK1IP1L	14.144156	14.23077	14.628428	12.791754	11.871523	12.800699	11.76635	11.237207	10.894534	0.513792637	-0.051495262	0.000760696	-0.249000358
ENSG00000275074	NUDT18	2.15549	2.65528	1.895738	1.780815	2.375467	1.946832	1.779886	1.860973	1.660306	0.983897243	0.007571873	0.3269169	-0.248240904
ENSG00000111057	KRT18	418.601513	439.065613	443.836991	276.711317	252.432766	277.03846	336.995513	335.240048	356.07655	7.37E-27	-0.541309165	0.0000079	-0.24770993
ENSG00000181915	ADO	6.479215	6.56168	6.736979	5.700175	6.441543	5.583445	5.472592	5.10328	5.03176	0.946348339	-0.010702145	0.017585492	-0.247671642
ENSG00000132953	XPO4	6.328014	6.782627	6.895092	6.457862	5.866373	5.754345	5.449581	4.95754	5.400971	0.998321415	0.000373396	0.001208759	-0.247625783
ENSG00000167615	LENG8	15.320754	15.549606	13.944253	14.925278	12.406716	12.866022	11.758269	9.407981	13.35438	0.673863954	-0.046027494	0.003043581	-0.247570663
ENSG00000182944	EWSR1	155.320046	156.808935	150.851374	148.60952	134.518448	133.02837	126.668428	118.865631	123.926738	0.889945453	-0.010986013	0.0000266	-0.247481221
ENSG00000114999	TTL	3.249144	3.462412	3.739484	2.311555	2.039111	2.192487	2.630197	2.669862	2.95817	4.86E-11	-0.528359385	0.004927237	-0.247391475
ENSG00000130935	NOL11	39.090225	38.018198	43.844601	32.548679	30.375092	30.50792	30.897206	30.198611	30.907726	0.004642359	-0.171244612	0.00013018	-0.247383064
ENSG00000186448	ZNF197	4.46289	4.681748	4.569322	5.023758	5.202292	4.832236	3.733222	3.659554	3.619672	0.008363684	0.283751573	0.042012692	-0.247098975
NewGene_801	--	3.793235	3.615128	3.667674	3.180853	2.994125	3.454317	2.587349	3.492496	2.687709	0.96250165	-0.021999298	0.475252828	-0.247094569
ENSG00000263465	SRSF8	3.018147	2.868908	3.063897	2.093899	2.079354	2.58606	2.403599	2.252832	2.749888	0.116133834	-0.245589276	0.071559552	-0.246986923
ENSG00000149548	CCDC15	0.76683	0.848467	0.898251	0.572443	0.42319	0.61876	0.526466	0.700617	0.759796	0.072539037	-0.49360123	0.361761214	-0.246853936
ENSG00000254004	ZNF260	4.270469	4.353835	4.812454	3.44964	3.863627	3.56585	3.587406	3.514889	3.60876	0.173003943	-0.156465686	0.016939832	-0.24667579
ENSG00000153006	SREK1IP1	2.169594	2.603955	2.879046	2.146825	2.333133	2.023024	2.056446	1.918441	2.073769	0.607793847	-0.08674647	0.06664207	-0.246616917
ENSG00000283526	PRRT1B	4.750968	4.150187	3.629235	3.855128	4.637307	4.562975	3.349505	3.34906	3.210278	0.351443797	0.205856407	0.238222818	-0.246230035
ENSG00000148660	CAMK2G	4.160549	4.611859	4.538157	4.485475	3.799769	4.276925	3.835322	3.371104	3.61376	0.78303259	0.045371827	0.044265847	-0.246199719
ENSG00000157315	TMED6	8.911168	11.059751	10.095069	10.873871	12.194386	13.035975	7.117502	8.758219	7.822358	0.004726291	0.414093037	0.152876091	-0.245731585
ENSG00000173548	SNX33	6.367859	5.99118	5.431304	6.69811	6.730352	6.655747	4.743526	4.101697	5.240752	0.0000358	0.322531727	0.012723319	-0.24573118
ENSG00000103540	CCP110	1.32646	1.705282	1.631996	1.290531	1.449034	1.266655	1.109632	1.06752	1.513245	0.763498046	-0.068439685	0.215015293	-0.245670901
NewGene_4619	--	1.84662	1.984156	3.530836	2.866433	2.379959	2.401859	2.234304	1.61886	1.977618	0.500222086	0.199249131	0.37654534	-0.245634472
ENSG00000151364	KCTD14	1.057994	0.917852	0.871426	1.782141	1.587582	1.471903	0.78537	0.552762	0.924179	0.000799986	0.91124116	0.527790005	-0.245577384
ENSG00000163378	EOGT	2.423519	2.959717	3.008392	2.796947	2.738342	2.557422	2.68229	1.995962	2.330843	0.656037012	0.084843669	0.122859063	-0.245543684
ENSG00000074410	CA12	3.569448	3.369138	3.32511	1.845796	1.441527	1.641044	2.749567	2.735917	2.627344	1.32E-16	-0.917433853	0.029309984	-0.245526642
ENSG00000196214	ZNF766	2.633509	2.915449	2.925003	2.48225	2.395889	1.962159	2.332095	2.129899	2.095092	0.088760391	-0.218346487	0.046838583	-0.24546043
ENSG00000123737	EXOSC9	24.937076	21.337408	30.450094	24.764285	25.758412	22.634374	20.185039	19.608591	18.693936	0.091724874	0.155471457	0.011381911	-0.245360529
ENSG00000152689	RASGRP3	0.73196	0.900516	0.655818	3.17192	2.480048	2.920052	0.498773	0.727619	0.46782	2.05E-50	2.35912679	0.413583287	-0.245347205
ENSG00000147874	HAUS6	17.838893	18.76722	19.925758	17.89611	16.217502	16.420861	14.986897	14.241527	15.932707	0.852285064	-0.018783941	0.000503079	-0.245295272
ENSG00000155366	RHOC	31.803978	30.531635	35.339941	24.959615	26.039377	27.581302	28.006601	27.672977	22.946742	0.245838001	-0.107910693	0.006756628	-0.245145653
ENSG00000122707	RECK	2.051872	1.57527	1.630711	1.376284	1.364893	1.220309	1.31329	1.316612	1.54139	0.152285308	-0.264753524	0.165619465	-0.245040881
ENSG00000103490	PYCARD	2.153166	1.155535	1.430102	2.595556	2.447704	2.666	1.060211	1.645776	0.946016	0.002898418	0.899092748	0.583337958	-0.24495381
ENSG00000058804	NDC1	17.1565	18.662094	19.14954	14.296122	12.017821	12.977556	14.380946	14.064603	15.057724	0.00000432	-0.337614279	0.000677871	-0.244893383
ENSG00000072415	MPP5	5.429902	6.025165	6.279127	5.322533	4.876197	5.074728	4.766052	4.336155	5.031154	0.630414294	-0.059790333	0.01982245	-0.244893104
NewGene_4565	--	0.447149	0.510296	0.560311	0.491121	0.60583	0.469278	0.631042	0.340067	0.410282	0.634559449	0.193978027	0.503360098	-0.244852685
ENSG00000183155	RABIF	8.251163	9.071046	8.42652	7.766552	7.790458	8.107812	6.792621	6.896196	6.656915	0.856107824	0.026643118	0.027400473	-0.24462362
ENSG00000182544	MFSD5	18.037357	16.184566	16.342077	16.158973	16.422279	16.813136	12.47271	11.083071	15.422076	0.273023275	0.114255834	0.025096893	-0.24448506
ENSG00000120437	ACAT2	79.83429	79.293732	79.730904	102.597908	109.562569	100.949944	60.581944	65.385178	62.946571	5.27E-25	0.538230266	0.000218437	-0.244228809
ENSG00000157077	ZFYVE9	11.480713	11.298308	11.297695	6.817831	7.0177	6.754865	8.244788	8.767129	9.796803	1.07E-17	-0.577391674	0.003037263	-0.244222645
ENSG00000073417	PDE8A	28.174656	27.218655	28.951372	23.008672	22.269932	19.147525	21.967406	20.303461	23.140356	0.000669637	-0.24037285	0.000360397	-0.244100846
ENSG00000111801	BTN3A3	1.952366	1.797809	1.980709	12.369393	11.205455	11.5715	1.591935	1.393843	1.345464	2.82E-170	2.794862507	0.234528371	-0.244077908

Gene ID	Gene name	Ctrl-1 FPKM	Ctrl-2 FPKM	Ctrl-3 FPKM	IFNγ-1 FPKM	IFNγ-2 FPKM	IFNγ-3 FPKM	Sora-1 FPKM	Sora-2 FPKM	Sora-3 FPKM	Ctrl vs IFNγ DESeq2 FDR	Ctrl vs IFNγ DESeq2 log2FC	Ctrl vs Sora DESeq2 FDR	Ctrl vs Sora DESeq2 log2FC
NewGene 3796	--	1.313022	0.913955	1.317041	0.61795	0.609205	0.537003	0.591208	1.06641	1.222095	0.001086451	-0.893716006	0.458938006	-0.243396452
ENSG00000133119	RFC3	25.897266	28.991855	28.244441	22.974665	21.735907	22.519352	22.188594	20.754234	23.018457	0.039954145	-0.156662538	0.001676154	-0.243281471
ENSG00000164031	DNAJB14	4.288991	6.420738	5.257524	5.557188	5.632194	5.401309	3.959051	3.834895	4.595914	0.004159695	0.293117326	0.038263192	-0.242953016
ENSG00000149489	ROM1	0.550382	0.623576	0.576201	0.590039	0.664086	0.548738	0.359218	0.557796	0.599295	0.815713715	0.124023679	0.616987414	-0.242890725
NewGene 1776	--	0.865019	0.665631	0.927523	0.596529	0.64387	0.596745	0.621296	0.873949	0.461761	0.344677863	-0.305625982	0.475182827	-0.242754899
ENSG00000144357	UBR3	6.66073	7.091875	6.995492	7.460606	7.007625	7.364114	5.549835	5.198922	5.960696	0.016283824	0.178958781	0.003491188	-0.242723047
ENSG00000126001	CEP250	2.600146	2.300133	2.159939	2.107531	1.929425	1.939984	2.041268	1.68444	1.907286	0.392278627	-0.095694972	0.025238096	-0.24267183
ENSG00000129071	MBD4	13.456679	14.206025	14.374324	11.956384	12.808256	11.809046	10.536243	10.541912	11.729141	0.648040027	-0.052105833	0.007257299	-0.242652319
ENSG00000011566	MAP4K3	18.266569	17.515891	19.313298	16.492234	17.055247	15.34256	14.350061	13.446846	16.06561	0.80407858	-0.025689943	0.002009203	-0.242628303
ENSG00000157014	TATDN2	15.5561	14.936363	15.670419	15.226368	13.974684	15.356928	12.756494	11.366831	12.961568	0.118226622	0.110643431	0.00070433	-0.242499627
ENSG00000134318	ROCK2	9.124299	9.960537	10.728001	11.853899	10.775193	11.719101	7.950215	7.014296	8.729687	0.017112731	0.23003395	0.005827562	-0.242460107
ENSG00000077097	TOP2B	56.696913	61.587213	61.862816	50.861667	49.616267	47.700649	50.223783	44.353403	51.041047	0.013841811	-0.140322705	0.000117675	-0.242282664
ENSG00000141759	TXNL4A	26.487906	28.156936	30.478022	24.560692	26.58683	24.259861	22.790459	24.229224	21.685185	0.818462962	0.023303327	0.002365257	-0.242085893
ENSG00000114982	KANSL3	18.319333	17.260284	17.369461	15.238952	14.195307	14.503862	14.277045	12.870597	15.003989	0.085728528	-0.120326594	0.000828134	-0.24205823
ENSG00000100345	MYH9	13.379754	12.069107	11.961952	10.660312	9.672426	9.781761	10.108421	8.763061	10.84783	0.027561095	-0.165755969	0.002541864	-0.242042544
ENSG00000100865	CINP	12.336963	8.165895	10.096255	6.729133	8.629782	9.191544	11.669321	9.448922	10.866175	0.785781651	-0.041033631	0.035501946	-0.241898154
NewGene 4666	--	1.670912	2.280761	1.360542	1.272911	1.494653	1.025792	0.970755	1.861543	1.332069	--	--	0.637560455	-0.241591334
ENSG00000113593	PPWD1	13.390662	15.128951	13.767671	12.235202	11.6341	12.342011	10.812812	11.101628	11.376372	0.570997808	-0.064124189	0.012682428	-0.241402684
ENSG00000090372	STRN4	38.000342	35.950887	35.950194	31.602185	31.44587	32.514307	27.314219	28.507384	32.58954	0.406987821	-0.060976787	0.001063005	-0.241105294
ENSG00000107672	NSMCE4A	21.018599	19.393386	21.5125	23.748682	21.656714	22.525856	18.030465	16.006	15.087888	0.001645146	0.277589317	0.031596378	-0.240724242
ENSG00000144635	DYNC1LI1	24.596067	22.236884	25.499338	20.416051	20.59925	20.501249	17.598664	18.058569	21.868811	0.348803655	-0.086332996	0.011326433	-0.240441497
ENSG00000137845	ADAM10	17.130456	19.378633	19.318771	19.506397	18.115342	19.130633	14.848193	14.401936	15.219296	0.008043983	0.170257309	0.000471913	-0.240220136
ENSG00000054690	PLEKHH1	2.523077	2.406394	2.642038	2.419302	1.825914	1.966506	2.184284	1.640825	2.282465	0.347771717	-0.141334402	0.086773787	-0.240048291
ENSG00000197930	ERO1A	32.197318	32.454149	32.642535	19.396207	19.273565	19.99958	25.897833	23.748556	28.875087	4.13E-29	-0.571023378	0.00051609	-0.239834716
ENSG00000160993	ALKBH4	4.738298	5.256398	5.372782	4.859886	4.29489	5.003988	3.494257	3.770657	4.821713	0.879493369	0.031440078	0.149672221	-0.239755143
ENSG00000132359	RAP1GAP2	2.435339	2.301186	2.973218	2.347105	2.085083	2.183598	1.812069	1.745699	2.356116	0.947063222	0.012377395	0.077575931	-0.239734231
ENSG00000115009	CCL20	107.304503	107.702324	140.386027	37.812136	47.195142	40.516561	85.371605	92.957612	103.52644	1.47E-14	-1.374690179	0.030136104	-0.23969421
ENSG00000101407	TTI1	17.205087	15.335673	15.012004	13.869315	12.68443	13.555226	12.530987	12.85742	13.582912	0.301081186	-0.100037645	0.006286817	-0.239687949
ENSG00000136490	LIMD2	34.225686	21.230259	19.838996	15.820799	19.492462	15.304867	15.078996	16.271847	17.968094	5.76E-08	-0.438059312	0.011504706	-0.239310088
ENSG00000175213	ZNF408	19.516855	16.797173	16.202847	18.271236	18.006319	18.493042	13.302718	12.811596	15.707712	0.025003926	0.207426224	0.021180193	-0.239216662
ENSG00000255408	PCDHA3	0.453227	0.427218	0.363083	0.156831	0.144686	0.179786	0.383297	0.349395	0.254746	0.000892539	-1.179091947	0.50714825	-0.239187448
ENSG00000169684	CHRNA5	1.669731	1.890846	1.694318	1.087543	0.943018	1.325816	1.572516	1.347877	1.321735	0.004731287	-0.584514172	0.239333404	-0.239161617
ENSG00000183844	FAM3B	3.396415	2.412126	2.617681	2.703356	2.541999	2.332579	2.445153	1.909438	2.146761	0.993875325	0.003494738	0.405401386	-0.239101907
ENSG00000005700	IBTK	20.442548	21.447577	22.84794	24.606312	24.734106	25.589977	17.69331	16.218291	17.922614	0.000000634	0.327976974	0.001480518	-0.239053596
ENSG00000066117	SMARCD1	47.678506	42.384666	45.128461	37.455114	36.458233	36.029453	34.966989	33.598953	38.739778	0.014329718	-0.150974455	0.000469314	-0.238945927
ENSG00000166225	FRS2	11.375688	12.590369	10.470586	14.964873	14.660826	15.223463	10.992594	10.653034	10.822617	1.45E-08	0.380035116	0.004918683	-0.238828307
ENSG00000153179	RASSF3	20.591005	20.99354	23.317305	16.228089	16.529696	16.179155	15.97616	16.916195	18.558023	0.000408461	-0.25795947	0.00445598	-0.238709152
ENSG00000171703	TCEA2	5.186318	10.008677	4.105303	6.219743	7.81063	9.742687	4.67058	4.813543	4.057566	0.000281592	0.729650328	0.315904668	-0.238647589
ENSG00000134046	MBD2	5.413429	5.521502	6.212874	5.566598	5.189726	5.139441	4.767608	4.750063	5.292997	0.728238034	0.04499209	0.024271524	-0.238601661
ENSG00000138593	SECISBP2L	4.672461	5.031479	4.48769	5.497969	4.738212	5.012171	3.963445	3.544839	3.701343	0.002949148	0.26159128	0.012915006	-0.238565531
ENSG00000161955	TNFSF13	5.389029	5.152788	4.713458	4.855709	4.615858	4.557655	4.383117	4.375541	3.389658	0.928866182	-0.020018104	0.138895657	-0.238508242
ENSG00000178904	DPY19L3	10.048087	11.303934	12.518547	10.80216	9.254762	9.488274	8.811733	8.323081	9.835432	0.558064971	-0.064305338	0.011107088	-0.238463382
ENSG00000124370	MCEE	8.958612	9.65919	12.015053	11.199179	10.509191	12.134316	9.156983	7.528259	8.487771	0.297111367	0.187750724	0.174824131	-0.238350014
ENSG00000135316	SYNCRIP	68.114173	73.115508	75.875492	73.16448	68.543602	67.985775	59.821816	56.383336	63.348591	0.1463568	0.09231468	0.000173547	-0.238301637
ENSG00000249459	ZNF286B	0.655156	0.994106	1.047888	0.815746	1.269574	1.07912	1.04496	0.657461	1.087905	0.961621721	-0.018976151	0.402615716	-0.238221444
ENSG00000163249	CCNYL1	9.117918	9.297002	10.525237	11.138134	10.304325	10.415326	7.518867	6.761502	7.719612	0.000880671	0.294559871	0.016860486	-0.238203713
ENSG00000102144	PGK1	63.674995	57.084515	49.486324	41.984276	44.009983	44.189522	43.224648	44.561607	47.6171	0.004328412	-0.23947617	0.006884313	-0.23817485
ENSG00000117523	PRRC2C	53.358823	49.322042	52.619612	62.435691	49.828252	51.474585	37.884504	38.65098	39.695772	0.00000927	0.256092675	0.0000232	-0.237997546
ENSG00000075240	GRAMD4	6.006162	6.610284	5.273468	4.677916	4.735372	4.568455	4.531042	4.722295	4.921773	0.319879873	-0.125366666	0.045857791	-0.237848314
ENSG00000164850	GPER1	18.036008	16.424622	16.899081	19.057233	21.308479	20.835332	13.381662	12.915638	14.947279	0.0000472	0.344665584	0.004859125	-0.237756434
ENSG00000137267	TUBB2A	22.585518	18.099363	16.846247	16.330702	16.475451	17.15682	14.315294	15.487352	15.971937	0.712865669	-0.056755834	0.055771993	-0.237686956
ENSG00000147050	KDM6A	10.3256	9.869693	11.28536	10.27603	11.405261	9.967186	9.053157	8.135331	8.821911	0.10671578	0.146091334	0.004732864	-0.23753961
ENSG00000167528	ZNF641	4.18286	3.517606	2.853518	2.503677	4.120356	2.469889	3.603693	2.798433	2.24364	0.290485578	0.706687971	0.790342625	-0.23750171
ENSG00000178177	LCORL	5.259344	5.204612	6.333869	4.716076	5.182777	4.844826	4.909173	4.318181	5.194377	0.964192542	-0.006997653	0.024684313	-0.237312945
ENSG00000242498	ARPIN	4.952915	4.709	4.40329	4.517241	4.15005	4.165634	3.952604	3.580396	3.954735	0.999202724	0.000211484	0.012110786	-0.237192188
ENSG00000172081	MOB3A	7.822939	6.95577	6.701098	6.26644	6.366401	6.611077	5.658037	5.844694	5.706579	0.832151977	-0.03129934	0.03202171	-0.237068545
ENSG00000053770	AP5M1	2.064256	2.230147	2.253756	1.902488	2.206718	1.93381	1.827018	1.587032	1.923428	0.857876612	0.027101825	0.029537982	-0.236569472
ENSG00000196646	ZNF136	2.186699	2.851012	2.556906	2.367103	2.595336	2.368955	2.140128	2.088734	1.762964	0.628127627	0.096128762	0.187902693	-0.236426191
ENSG00000183421	RIPK4	10.258078	10.041482	10.217296	7.513976	8.70835	8.016197	7.882315	7.142307	9.32785	0.051217372	-0.184389214	0.015422724	-0.23638905
ENSG00000153885	KCTD15	13.462306	14.549671	14.422724	13.706734	13.020497	12.716185	11.355616	11.374537	12.356652	0.922953052	0.010666715	0.004764571	-0.236226983
ENSG00000167461	RAB8A	31.869629	29.946792	29.412071	30.413573	29.640844	28.66737	23.941195	23.822556	24.50908	0.087463603	0.119934972	0.000853002	-0.236061471
ENSG00000183091	NEB	0.617263	0.300028	0.304966	0.172644	0.188132	0.133864	0.209749	0.235045	0.501289	0.00109749	-0.623390418	0.19832922	-0.23593729
ENSG00000128567	PODXL	0.564831	0.542531	0.540438	0.833472	0.64273	0.68885	0.525556	0.433999	0.395656	0.014938121	0.545201395	0.382206845	-0.235864195
ENSG00000112874	NUDT12	4.967056	5.546964	4.771321	4.003909	4.018744	4.054614	3.841943	4.213791	4.22255	0.110347479	-0.197143308	0.060007453	-0.235742571
ENSG00000164241	C5orf63	1.840795	2.198245	2.045167	1.751283	1.221282	1.82382	1.835077	2.083515	2.201543	0.529699782	-0.155204966	0.234477272	-0.235629339
ENSG00000156504	FAM122B	5.035651	5.599161	6.914191	4.144919	3.740129	3.942919	4.524385	4.413183	5.114749	0.001085249	-0.42615553	0.085879547	-0.235360904
ENSG00000134824	FADS2	44.305404	34.693831	33.911364	44.056082	47.316349	47.355616	28.070133	30.123594	33.21847	0.000000025	0.473877282	0.01942015	-0.235359436
ENSG00000142751	GPN2	5.059206	4.955263	5.375141	4.516934	4.55923	4.369829	3.890238	4.214454	4.174425	0.708764618	-0.047676111	0.026475538	-0.234939254
ENSG00000047644	WWC3	2.59315	2.790026	3.04563	2.42373	2.354408	2.42193	2.333616	1.96139	2.431144	0.571625226	-0.079271892	0.058944165	-0.234768997
ENSG00000171444	MCC	4.313129	5.22012	5.940256	3.650957	3.231621	3.518454	4.399776	3.989296	4.187527	0.000106008	-0.424911029	0.04885977	-0.234724367
ENSG00000185630	PBX1	13.759935	14.081306	13.580321	8.556787	8.881998	8.034693	11.955295	12.327406	10.728682	5.46E-20	-0.568550628	0.00265979	-0.23471021
ENSG00000151881	TMEM267	3.872869	4.113702	4.329989	3.726094	4.042224	3.217009	3.535445	2.962221	5.376085	0.531064951	-0.108043295	0.131235868	-0.234690637
ENSG00000196705	ZNF431	1.719122	1.733766	1.86481	1.685689	1.807606	1.460685	1.411636	1.654474	1.584134	0.933602721	-0.012547881	0.024589785	-0.234540769
ENSG00000183495	EP400	5.993699	6.125591	8.52839	6.770687	5.285722	5.397655	4.68082	5.097063	6.989216	0.32511078	0.081993436	0.012237497	-0.234246043

Gene ID	Gene name	Ctrl-1 FPKM	Ctrl-2 FPKM	Ctrl-3 FPKM	IFNγ-1 FPKM	IFNγ-2 FPKM	IFNγ-3 FPKM	Sora-1 FPKM	Sora-2 FPKM	Sora-3 FPKM	Ctrl vs IFNγ DESeq2 FDR	Ctrl vs IFNγ DESeq2 log2FC	Ctrl vs Sora DESeq2 FDR	Ctrl vs Sora DESeq2 log2FC
ENSG00000147231	RADX	2.817454	2.610279	2.886412	2.960247	3.25459	3.135995	2.468412	1.750956	2.382567	0.02463243	0.308907282	0.157955226	-0.234133079
ENSG00000185298	CCDC137	19.860189	21.1384	19.158632	18.910681	20.2036	17.62962	15.737278	15.443504	17.447085	0.522360857	-0.061257321	0.00521886	-0.233988316
ENSG00000125257	ABCC4	5.315863	4.948671	5.819062	6.274971	5.573109	5.866608	4.384482	4.322682	4.01947	0.001510865	0.29311472	0.030576664	-0.233986915
ENSG00000130150	MOSPD2	2.920965	3.24693	3.239637	3.221238	2.917305	3.342914	2.094767	2.989244	2.476461	0.299447269	0.151928768	0.148104127	-0.233760055
ENSG00000116688	MFN2	24.088701	24.338706	23.502206	25.910242	24.178791	24.856121	17.930216	17.843599	21.708706	0.000291354	0.206069308	0.002599712	-0.233708028
ENSG00000144182	LIPT1	2.581766	3.149644	3.189742	4.535081	3.982986	3.685586	2.965261	1.973923	2.225649	0.021712748	0.479770535	0.367769107	-0.233688517
ENSG00000117395	EBNA1BP2	39.34971	39.73266	42.466641	32.552741	27.583297	31.650355	31.271236	31.275358	33.952859	0.002424135	-0.254166776	0.003132983	-0.233461063
ENSG00000176095	IP6K1	8.329378	9.683532	9.762054	8.941071	8.263671	8.637528	7.112622	6.883242	8.325776	0.781443097	0.034396921	0.020234201	-0.23336314
ENSG00000118777	ABCG2	1.509696	1.685855	1.932595	0.993862	0.918956	1.102396	1.154094	1.422972	1.942692	0.00127319	-0.571393474	0.224634517	-0.233319507
NewGene_1514	--	0.688326	0.743518	0.601434	0.67618	0.565216	1.094583	0.661793	0.406537	0.576895	0.593446226	0.253136891	0.579049425	-0.233244246
ENSG00000188227	ZNF793	2.267222	3.15017	2.694442	2.268896	1.551333	2.318506	2.050727	1.483274	1.71558	0.383558961	-0.151393701	0.138779042	-0.233205172
ENSG00000143643	TTC13	12.665822	15.928022	15.697877	16.847	15.839819	15.363955	12.109646	10.808499	12.590626	0.010764958	0.249903364	0.028150688	-0.233154327
ENSG00000146909	NOM1	7.95025	8.410442	8.898682	7.722347	7.04533	7.523706	6.475679	6.375422	7.321373	0.758694529	-0.032978205	0.006356174	-0.233062655
ENSG00000081386	ZNF510	2.684431	2.546328	2.771552	2.544906	2.621476	2.299648	2.006706	2.246158	2.510799	0.980706027	0.004931446	0.066164681	-0.23299517
ENSG00000006607	FARP2	5.680296	6.144946	5.897005	7.137558	6.230936	6.648529	4.781599	4.460523	5.021164	0.000655372	0.289776234	0.009643573	-0.232922643
ENSG00000116017	ARID3A	45.908596	47.513395	46.127385	51.698841	47.878051	52.842068	37.614667	34.048815	40.250831	0.000000228	0.271990551	0.000158733	-0.232491918
ENSG00000120594	PLXDC2	1.319783	1.578243	1.631102	0.479931	0.432423	0.448068	1.160539	1.194571	1.258139	1.28E-36	-1.587140568	0.063217494	-0.23249066
ENSG00000136231	IGF2BP3	28.459858	29.465237	30.174431	31.795903	31.624342	30.922149	24.324105	21.981346	24.103998	0.00000869	0.245049423	0.000440913	-0.23239292
ENSG00000070010	UFD1	44.634548	42.674744	42.633945	44.254704	42.501718	43.54739	36.10616	35.95625	36.918856	0.234487127	0.092808483	0.001256132	-0.232299352
ENSG00000114779	ABHD14B	10.411131	10.326949	8.613756	8.047142	7.84455	9.581614	8.070975	7.526477	7.122715	0.691267941	-0.067054838	0.081680383	-0.23227798
ENSG00000163138	PACRGL	13.612262	14.756594	16.743869	9.546904	9.442864	10.7328	13.13455	11.013082	12.090105	0.000100218	-0.40642031	0.034436346	-0.232268842
ENSG00000121940	CLCC1	7.318674	7.812138	7.33364	6.197157	5.685999	6.365557	6.452903	5.231935	6.153245	0.529699782	-0.073206294	0.019831355	-0.232258537
ENSG00000038382	TRIO	7.837283	8.168481	8.631691	8.911718	7.924733	7.972765	6.793694	5.918864	7.296538	0.042864902	0.151258968	0.004044724	-0.232046286
ENSG00000171067	C11orf24	58.932126	54.891745	54.314148	50.007128	44.546863	48.896355	40.102567	41.692258	51.772725	0.274330832	-0.08494287	0.008555898	-0.232045147
ENSG00000204613	TRIM10	2.665695	2.314664	2.482535	2.079855	2.393446	2.040241	1.979017	1.70893	2.486959	0.613960433	-0.098166294	0.172328311	-0.231920851
ENSG00000064225	ST3GAL6	16.591632	19.482725	21.03538	12.813623	13.733616	13.165772	13.311863	13.937367	17.31643	7.82E-08	-0.525315925	0.027581682	-0.231900594
ENSG00000153790	C7orf31	4.843031	4.499345	5.09356	7.367339	7.466366	8.176812	4.270643	3.176526	3.867177	1.75E-16	0.843356228	0.116087895	-0.231699992
ENSG00000164761	TNFRSF11B	1.634707	1.508552	1.799597	1.356557	1.712595	1.256732	1.295571	1.058584	1.543723	0.897008801	-0.043043054	0.351859247	-0.231635535
ENSG00000139668	WDFY2	2.79846	2.719099	3.271167	2.396986	2.68776	2.408146	2.320998	2.144218	2.630607	0.536451168	-0.084255741	0.042783424	-0.231454735
ENSG00000241360	PDXP	17.517302	18.406267	16.53455	17.209492	16.068082	18.48489	15.003888	12.92946	13.953844	0.203041323	0.127753115	0.016572414	-0.231255099
ENSG00000071243	ING3	13.015384	13.922457	12.45614	9.890414	10.551964	8.945935	10.96683	10.482545	10.362173	0.037388704	-0.203831036	0.017585971	-0.230993848
ENSG00000174233	ADCY6	12.301645	11.299899	11.223064	8.420336	8.391179	8.110178	8.867558	8.671721	10.047255	0.00000457	-0.32510506	0.004675694	-0.230904622
ENSG00000146535	GNA12	26.51568	24.483195	23.9895	22.136806	22.153023	22.472761	18.679283	17.503523	23.946989	0.819682308	-0.02111731	0.017656894	-0.230796385
ENSG00000162062	TEDC2	6.30711	5.77092	5.130847	4.703038	4.457647	4.532668	4.921709	4.165702	5.001466	0.353331675	-0.158088511	0.139914283	-0.230726731
ENSG00000006377	DLX6	4.08466	3.610035	4.149727	3.495695	2.537597	3.162986	3.053772	3.148466	3.296101	0.266170009	-0.208004892	0.150187786	-0.230580071
NewGene_4415	--	0.459654	0.722196	0.789045	0.606769	0.622519	0.76225	0.685722	0.357996	0.473027	0.792965344	0.156328973	0.666277741	-0.230527036
NewGene_6487	--	0.876408	0.825356	0.790496	0.622496	0.990762	0.629541	0.725066	0.484468	0.867294	0.592626156	-0.166884862	0.338945633	-0.230138031
ENSG00000166025	AMOTL1	4.115393	4.067651	4.058479	3.294967	2.995686	3.387158	3.487009	2.891431	3.405882	0.036312773	-0.18760169	0.014510422	-0.230022263
ENSG00000271303	SRXN1	10.511827	10.889198	11.086386	10.324675	9.847455	10.251649	8.321603	8.085988	9.609653	0.636109696	0.053221357	0.018952207	-0.229983838
ENSG00000088888	MAVS	8.217009	8.252511	6.944895	7.65015	7.07494	7.402123	6.234639	5.74074	6.814979	0.508141255	0.06504439	0.008606475	-0.229967393
ENSG00000198932	GPRASP1	0.306664	0.346973	0.330985	0.22011	0.220922	0.238784	0.293779	0.249151	0.235223	0.288077673	-0.377646537	0.500125932	-0.229884267
ENSG00000108599	AKAP10	6.887405	7.243441	7.441883	7.328332	6.589716	6.636333	6.203136	5.188738	5.994079	0.630877533	0.056760489	0.021794996	-0.229875982
NewGene_1525	--	3.627568	4.622284	4.459024	6.633173	5.97213	6.681561	3.286779	3.161391	3.724134	8.87E-13	0.749626665	0.08642673	-0.229859628
ENSG00000132793	LPIN3	2.105294	1.887463	1.764323	2.243755	1.754111	1.739973	1.49247	1.470598	1.662841	0.464210524	0.14129361	0.166826283	-0.229766562
ENSG00000136732	GYPC	5.798093	4.821234	4.825311	7.82572	5.782412	7.722761	4.460881	3.725401	4.120086	0.000309372	0.604271493	0.232655814	-0.229593469
ENSG00000147894	C9orf72	1.061624	1.01569	0.854951	1.262956	1.354158	1.313346	0.954316	0.790073	0.761005	0.043270597	0.468217684	0.41636168	-0.229592807
ENSG00000196155	PLEKHG4	0.285412	0.155046	0.211519	0.308361	0.254596	0.217816	0.186232	0.13459	0.182191	0.165783663	0.527923675	0.594203992	-0.229565861
ENSG00000158292	GPR153	1.192739	1.107759	1.080351	0.956127	0.724559	1.02992	0.875695	0.715649	1.126409	0.534021932	-0.165694138	0.329694475	-0.229529626
ENSG00000162433	AK4	5.292645	5.004021	4.210405	4.870119	5.349887	4.667932	3.745012	3.602053	4.258689	0.089984882	0.190566514	0.036522933	-0.229514103
ENSG00000157637	SLC38A10	25.127309	23.361007	20.64977	23.52348	23.016658	24.779781	18.74308	16.964648	18.812355	0.006849749	0.206624273	0.005028166	-0.229414447
ENSG00000156172	C8orf37	1.042161	1.154033	0.92373	1.340496	1.401772	1.155213	0.880105	0.785315	0.831169	0.037177489	0.463296918	0.366163855	-0.229360908
ENSG00000090612	ZNF268	5.947033	6.524147	4.747697	3.848271	4.751078	4.184684	4.846561	4.058235	4.544381	0.86382996	-0.021023047	0.009438239	-0.229290653
ENSG00000145439	CBR4	5.67718	7.625572	8.071012	6.252945	6.184354	6.518263	5.511714	5.341287	6.326786	0.985560853	0.003657708	0.098646595	-0.229157336
ENSG00000162063	CCNF	8.873642	8.747562	8.401977	7.004723	6.281385	7.110202	7.121849	6.210791	7.557996	0.036858826	-0.190265684	0.013377481	-0.228884603
ENSG00000147789	ZNF7	12.365313	13.48783	11.737933	11.923443	12.690513	11.047602	12.430749	11.381013	12.301064	0.400254987	0.092658755	0.021391074	-0.228867305
ENSG00000143952	VPS54	45.410553	49.188823	50.306801	46.424895	43.595657	44.557245	38.409354	36.809252	41.059002	0.714867652	0.030062514	0.001098169	-0.228834284
NewGene_2760	--	0.478536	0.730849	0.506509	0.403277	0.483272	0.371175	0.341407	0.589189	0.434158	0.557019239	-0.301838625	0.630050229	-0.228827935
ENSG00000008256	CYTH3	4.53675	3.955033	4.159925	3.311261	3.325831	2.638394	3.523032	3.134381	3.47379	0.019785267	-0.303969824	0.050833382	-0.228732454
ENSG00000086102	NFX1	24.932813	25.636036	27.40832	30.928489	28.495054	28.090017	21.570592	20.222305	20.706703	0.000000807	0.314170586	0.0016667	-0.228731418
ENSG00000162521	RBBP4	41.989018	36.323962	38.593006	31.465203	35.847032	31.425704	26.884716	26.510543	29.322846	0.354476218	0.055476364	0.000186944	-0.228621531
NewGene_4181	--	2.101095	1.945017	3.126329	2.839583	2.46814	2.997573	1.898445	1.598194	2.12815	0.008745027	0.390544478	0.200629766	-0.22842113
ENSG00000096696	DSP	80.652184	74.443027	75.322487	53.652334	52.963013	52.916586	60.503656	58.693866	67.075019	1.17E-14	-0.373064607	0.000227437	-0.22840338
ENSG00000072364	AFF4	9.634142	10.227131	10.944709	10.624705	10.633641	10.546413	8.496025	7.288032	8.828969	0.00223172	0.206806967	0.005048698	-0.22837558
ENSG00000163728	TTC14	9.415295	13.150193	10.483592	10.96239	10.930941	10.734595	7.521209	7.790824	8.365413	0.196436902	0.129524368	0.021712206	-0.228344131
ENSG00000054118	THRAP3	51.147624	50.497087	48.87053	54.521517	51.854124	52.285968	40.886054	38.766561	40.521801	0.00000525	0.230088552	0.000124224	-0.22831498
ENSG00000184857	TMEM186	5.1337	6.28584	6.331326	4.885781	5.028156	5.211277	4.08269	5.370607	4.709455	0.69309949	-0.082232955	0.220655782	-0.228180391
ENSG00000090339	ICAM1	39.993778	38.82341	40.264103	179.395386	172.784439	181.671402	28.798491	30.150936	36.580414	0	2.311565585	0.005877907	-0.228014725
ENSG00000101413	RPRD1B	18.383827	19.369543	20.990464	21.89911	20.502717	21.543867	16.762153	14.442199	16.509671	0.0000761	0.266531863	0.006707135	-0.228000256
ENSG00000163131	CTSS	0.781093	0.822052	0.852917	11.904335	11.472317	11.377828	0.631181	0.537186	0.803255	0	3.952428662	0.39065115	-0.227736696
ENSG00000100888	CHD8	14.075862	13.923854	13.937977	14.075394	13.792926	12.928887	11.518697	10.460699	12.033812	0.683922094	0.033525075	0.000723695	-0.227401762
ENSG00000162402	USP24	10.92056	10.275398	10.248879	9.103187	8.853886	8.397077	8.274756	8.163292	9.370674	0.071488172	-0.126619259	0.001403247	-0.226843179
ENSG00000228049	POLR2J2	1.265674	1.290678	2.74045	1.038431	0.814985	1.742099	1.095584	1.365832	1.207185	0.86382996	-0.078228517	0.440218119	-0.226756544
ENSG00000106012	IQCE	3.911325	3.484193	3.408467	4.095505	3.84674	4.154471	3.443603	2.49101	3.581505	0.940949726	0.011739102	0.060826226	-0.226742753
ENSG00000140993	TIGD7	1.590892	1.54607	2.009427	2.272018	1.930536	1.851586	1.455025	1.5163	1.131106	0.046927691	0.372765942	0.305868311	-0.226699684

Gene ID	Gene name	Ctrl-1 FPKM	Ctrl-2 FPKM	Ctrl-3 FPKM	IFNγ-1 FPKM	IFNγ-2 FPKM	IFNγ-3 FPKM	Sora-1 FPKM	Sora-2 FPKM	Sora-3 FPKM	Ctrl vs IFNγ DESeq2 FDR	Ctrl vs IFNγ DESeq2 log2FC	Ctrl vs Sora DESeq2 FDR	Ctrl vs Sora DESeq2 log2FC
ENSG00000065320	NTN1	3.561466	2.892175	2.967802	1.57773	1.562428	1.585828	2.202682	2.736849	2.608655	1.12E-12	-0.847380985	0.10703996	-0.226424704
ENSG00000129646	QRICH2	1.099552	0.966158	1.101079	1.377048	1.297176	1.392646	0.779751	0.74764	1.009292	0.001377122	0.508959196	0.266288911	-0.226410353
ENSG00000103067	ESRP2	13.917919	14.603769	13.551404	15.665902	14.292431	15.085487	10.864057	10.442763	12.739198	0.00031823	0.252220584	0.007784516	-0.226333647
ENSG00000100731	PCNX1	3.621258	3.815041	4.541146	3.082591	3.342364	3.41751	3.541696	2.570281	3.052499	0.067788278	-0.1538986	0.009667208	-0.226300048
ENSG00000119280	C1orf198	24.576729	22.759141	24.762904	20.339129	20.000077	20.825327	19.29908	17.807222	20.771604	0.226677788	-0.092430109	0.002503221	-0.225915255
ENSG00000186193	SAPCD2	22.519987	23.641123	19.990959	16.796556	16.24357	18.689987	17.196562	16.891613	19.001329	0.017361028	-0.20673534	0.006406378	-0.225760482
ENSG00000008294	SPAG9	11.944508	14.058106	14.555609	14.21612	14.535865	13.709544	11.168187	11.229214	11.536351	0.025499731	0.15953943	0.00376329	-0.225504075
ENSG00000100099	HPS4	9.587635	9.14265	10.185459	10.686934	9.669664	9.050169	7.179214	7.018503	7.922077	0.010098882	0.215173232	0.010663474	-0.225279474
ENSG00000096088	PGC	3.181813	2.809512	3.342422	0.958909	1.609419	1.444045	2.48395	2.414858	2.335048	0.00000753	-1.161238305	0.341012744	-0.225239238
NewGene_6707	--	0.788578	0.528324	0.642838	0.532444	0.630582	0.599121	0.516571	0.651751	0.472011	0.953110507	-0.03233372	0.597559905	-0.225094833
ENSG00000142528	ZNF473	6.887136	6.19075	5.98419	4.5413	4.780321	4.757293	5.084826	4.873115	5.020581	0.001581453	-0.299419796	0.023711016	-0.224942286
ENSG00000128923	MINDY2	1.937745	2.104411	2.31401	2.519758	2.462115	2.45302	1.95572	1.615628	2.535339	0.004437037	0.318791633	0.081030987	-0.224825135
ENSG00000167721	TSR1	28.255274	28.363365	31.395104	26.062078	24.111041	25.364015	24.220096	22.365094	24.318946	0.332800591	-0.072717797	0.001200412	-0.224744437
ENSG00000198113	TOR4A	19.571777	18.357019	16.876982	14.531642	15.149817	16.310541	13.373794	14.187154	16.480114	0.262711771	-0.104816478	0.013672821	-0.224706542
ENSG00000129292	PHF20L1	10.021421	10.531132	10.263861	7.617472	9.060527	9.012589	9.021895	6.971108	8.399856	0.951256829	0.007749094	0.010935001	-0.224689087
ENSG00000042088	TDP1	5.692531	5.779887	5.940137	5.059341	4.132671	4.33995	5.734709	5.112063	4.790324	0.211192208	-0.168097617	0.075648723	-0.22451137
ENSG00000278311	GGNBP2	37.347099	38.048093	39.065468	39.410443	37.750399	38.113036	32.615522	29.266532	30.41234	0.020912981	0.138384404	0.001349488	-0.224419208
ENSG00000116514	RNF19B	2.098142	1.543085	1.536986	5.339727	4.37199	4.949699	1.584521	1.113464	1.443795	2.8E-28	1.668659533	0.373681972	-0.224373686
ENSG00000102409	BEX4	1.809954	1.728507	2.016571	0.930353	1.179363	0.847087	1.475323	1.408916	1.568231	0.015994993	-0.749293784	0.451882609	-0.224325786
ENSG00000276293	PIP4K2B	17.834779	16.805166	15.957889	16.700448	15.625085	16.111319	13.010325	12.475975	15.315793	0.260593374	0.085684918	0.005721671	-0.224161593
ENSG00000170927	PKHD1	0.705417	0.551591	0.668591	0.223251	0.191722	0.152036	0.449481	0.517641	0.570742	6.92E-21	-1.634609975	0.181349659	-0.224074411
ENSG00000196655	TRAPPC4	19.909763	18.014534	17.271263	24.638738	27.902159	23.62596	13.525921	16.210459	16.081706	9.47E-16	0.670414693	0.039380389	-0.224045429
ENSG00000146094	DOK3	1.681204	0.80527	1.065545	1.152812	0.802349	0.738233	1.157333	0.841317	1.113711	0.477571011	-0.298799839	0.539221979	-0.223976599
ENSG00000112096	SOD2	26.429908	30.544866	30.460287	66.346691	62.833493	69.579077	26.059966	23.789461	25.533812	5.07E-68	1.095515085	0.00530611	-0.22389838
ENSG00000141380	SS18	15.713222	16.924407	17.645889	13.641966	12.728417	12.725707	13.062738	12.699592	14.407853	0.004147544	-0.232041572	0.008521297	-0.223862966
ENSG00000163013	FBXO41	0.861159	0.947363	0.993946	1.013491	0.879925	0.947453	0.745039	0.736277	0.795361	0.852272749	0.043869938	0.21451921	-0.223812171
NewGene_1936	--	0.586352	0.473485	0.710065	0.798981	0.982925	0.813961	0.422999	0.528513	0.439689	0.007436787	0.666565584	0.481760815	-0.223713526
NewGene_4124	--	1.568073	1.44085	1.735312	1.196401	0.778429	0.750682	1.197755	1.347027	1.162954	0.007094154	-0.584761019	0.263397682	-0.223643999
ENSG00000143390	RFX5	29.62531	26.094993	26.77307	27.883181	27.989979	26.091463	21.759123	21.822695	24.78847	0.632138393	0.043836471	0.002731306	-0.223573065
ENSG00000092931	MFSD11	22.782405	21.573581	19.18965	20.242665	20.550082	20.669174	16.072259	16.80849	17.297188	0.827329095	0.024819234	0.014640559	-0.223542681
ENSG00000162377	COA7	8.896463	9.744185	9.655074	9.236716	9.364855	8.842086	7.479596	7.239269	8.018863	0.272147339	0.103654405	0.011655064	-0.223235041
ENSG00000139437	TCHP	2.256287	2.580537	2.228008	2.478968	1.679124	2.481594	2.065448	1.454017	2.167788	0.785567011	0.06859185	0.251600757	-0.223196977
NewGene_3800	--	0.43082	0.414696	0.346346	0.400137	0.603532	0.375313	0.27588	0.29359	0.375246	0.553222742	0.291820766	0.638178553	-0.223091746
ENSG00000100578	KIAA0586	4.470366	5.463606	4.084588	5.279477	3.287855	4.350448	4.832922	4.410125	4.598098	0.026327997	-0.199767478	0.013650915	-0.223048686
ENSG00000154553	PDLIM3	1.355844	0.882395	0.805671	0.762451	0.704893	0.602582	0.81127	0.754193	0.853155	0.306595688	-0.418081377	0.559409155	-0.222762404
NewGene_268	--	0.661722	0.468938	0.485204	0.539558	0.592682	0.384046	0.260618	0.469081	0.603268	0.930787487	0.044998709	0.603319236	-0.222596086
ENSG00000115590	IL1R2	1.517304	1.343539	0.90784	0.370148	0.55316	0.375385	1.009262	1.022554	0.974749	0.001756892	-1.254639549	0.557841398	-0.222565931
ENSG00000083750	RRAGB	5.937405	5.401124	5.620435	5.060192	4.038845	4.132995	4.560592	4.322066	4.858612	0.204985949	-0.200187664	0.112342628	-0.222342875
ENSG00000176387	HSD11B2	15.813624	14.402665	12.907305	8.404261	9.594371	8.314671	11.019554	11.511036	11.721957	0.000000386	-0.561076039	0.046813664	-0.222332266
ENSG00000187790	FANCM	2.097276	2.44749	2.557385	2.259963	1.973316	2.053822	1.918906	1.794137	2.273382	0.857298009	-0.031496624	0.096488178	-0.222172649
ENSG00000115421	PAPOLG	5.441524	4.989098	5.477183	5.224482	4.605464	5.020087	5.027226	4.042795	4.394204	0.825791017	0.03010231	0.037103106	-0.222050972
ENSG00000140750	ARHGAP17	15.961457	15.59395	16.769913	14.457915	13.95775	13.44354	12.334717	11.607643	14.309551	0.471224299	-0.065052045	0.011385759	-0.222028967
ENSG00000095261	PSMD5	20.618762	21.030715	23.010558	21.285459	20.634481	20.600579	18.553915	15.850917	17.566524	0.215462563	0.097803181	0.008416423	-0.22188934
ENSG00000174871	CNIH2	4.973052	5.216876	4.699914	3.343051	3.507644	3.738929	3.775609	3.64611	4.621581	0.104555067	-0.30482903	0.225819687	-0.221779772
ENSG00000176022	B3GALT6	19.773413	18.230606	18.74445	14.970745	16.254352	16.431817	14.685311	14.394549	16.549707	0.275620507	-0.10113188	0.007064569	-0.22177629
ENSG00000138600	SPPL2A	10.669369	11.766627	12.400833	8.866459	8.723766	8.331073	9.52529	8.630474	10.38836	0.00140058	-0.232493404	0.0053636	-0.221749048
ENSG00000173575	CHD2	16.295239	16.599927	17.006723	15.739829	15.068994	15.071522	15.297086	12.829251	14.792648	0.324212016	0.063478293	0.002202525	-0.221628378
ENSG00000149187	CELF1	25.966885	25.884728	27.855695	24.701098	24.618434	24.198108	21.420746	21.028376	22.814495	0.401039741	0.054542965	0.00024227	-0.221500836
ENSG00000164091	WDR82	30.912969	29.454804	28.990398	33.376816	33.923274	32.145376	24.029301	22.400424	25.208055	0.000000117	0.305160015	0.000810508	-0.221441275
ENSG00000144730	IL17RD	0.437147	0.573784	0.354432	0.307815	0.21869	0.240003	0.343628	0.368	0.386404	0.011099447	-0.686294594	0.402868335	-0.221305986
ENSG00000176170	SPHK1	39.625063	36.594617	37.768158	26.015277	31.780588	30.019661	28.356947	29.375166	34.498118	0.011440986	-0.226402523	0.00935999	-0.221157303
ENSG00000171346	KRT15	0.544501	0.509497	0.789382	0.058858	0.102695	0.060682	0.45221	0.411749	0.642353	1.82E-12	-2.595760322	0.500852053	-0.220882045
ENSG00000206418	RAB12	11.718766	12.376594	14.796791	12.135581	12.594221	11.369369	10.236644	9.551863	11.56052	0.793623764	0.039615093	0.06224486	-0.220779861
ENSG00000138092	CENPO	12.616226	12.393636	11.962308	11.031049	10.001072	10.86555	9.948072	9.71276	10.508602	0.385830044	-0.076946719	0.004300968	-0.220779453
NewGene_4274	--	1.180232	1.12031	1.370978	1.345914	1.412127	1.153058	1.095162	0.891657	0.969229	0.197472764	0.229777582	0.210661532	-0.220729381
ENSG00000255302	EID1	42.322624	46.315887	45.454407	45.792957	44.25864	45.794521	36.024044	34.101025	37.837486	0.00896987	0.166463917	0.00151212	-0.220704785
ENSG00000116544	DLGAP3	0.720906	0.719015	0.537888	0.527115	0.312344	0.459443	0.524397	0.375975	0.708012	0.172334954	-0.458385529	0.505179805	-0.220538201
ENSG00000160201	U2AF1	73.88353	65.655049	65.68894	61.871911	63.02435	56.941955	55.542593	57.073363	54.468999	0.974018322	0.004172736	0.011600991	-0.220489377
ENSG00000151116	UEVLD	4.410716	5.112947	4.85222	5.284467	5.503285	4.812605	3.528879	3.697471	4.167814	0.032284629	0.253341174	0.082257354	-0.220460342
ENSG00000005801	ZNF195	35.044718	40.578088	44.858727	32.621458	32.129344	29.057451	33.905972	29.742472	33.803972	0.05182847	-0.197430429	0.021897655	-0.220391075
ENSG00000109452	INPP4B	0.143483	0.173559	0.203222	0.10094	0.114542	0.106442	0.072354	0.17622	0.133352	0.318933312	-0.455248554	0.631516932	-0.220364191
ENSG00000167766	ZNF83	5.299901	5.708603	5.66958	4.331059	3.797134	4.430197	4.449607	3.952159	4.831246	0.030799433	-0.271490418	0.088598295	-0.220285289
ENSG00000141013	GAS8	5.489431	4.027866	5.569366	3.957326	5.638218	6.032411	3.346803	4.184276	3.493118	0.433392926	0.122415702	0.105812721	-0.220258809
ENSG00000117528	ABCD3	46.210053	51.630443	53.521145	47.565721	48.482873	47.294877	41.193962	38.079018	42.805291	0.443091122	0.061977382	0.002503138	-0.22005519
ENSG00000185753	CXorf38	2.79877	2.685514	3.20271	4.445071	3.838845	3.993825	2.348503	2.008454	2.548442	6.97E-08	0.624062027	0.1395679	-0.219965832
ENSG00000100320	RBFOX2	13.299827	12.65407	14.814679	13.20689	12.056394	18.725754	12.943074	11.546668	13.385639	0.012938556	0.168491085	0.003282727	-0.219875855
ENSG00000101311	FERMT1	0.588231	0.656692	0.697843	0.41496	0.50099	0.470514	0.453563	0.48733	0.626127	0.22159189	-0.334978188	0.409059123	-0.219866428
ENSG00000054793	ATP9A	19.364613	18.358191	17.461145	15.040696	14.124278	13.923123	14.898783	13.755171	15.839729	0.000861065	-0.210421736	0.00155754	-0.219844725
ENSG00000132437	DDC	6.677816	8.138977	5.902897	8.018424	7.692475	7.427735	6.11922	5.481383	5.043239	0.026883579	0.307093472	0.172700802	-0.219829487
ENSG00000107331	ABCA2	3.52463	3.047804	2.635899	2.745879	2.775748	2.864885	2.365016	2.841975	2.660698	0.89670426	0.022524394	0.085262113	-0.219728061
ENSG00000186300	ZNF555	1.933354	1.504791	2.112259	1.675961	2.019996	1.800487	1.612217	1.884685	2.040977	0.930677713	0.016676334	0.140962509	-0.219721122
ENSG00000189319	FAM53B	3.941455	4.073348	3.103074	3.109413	3.003642	3.35198	3.126711	2.475291	3.178703	0.664685895	-0.071010981	0.107046423	-0.21969799
ENSG00000122376	SHLD2	14.564812	16.040353	15.377185	16.064261	16.037941	15.737023	12.004503	12.107879	12.786465	0.003223825	0.213325227	0.006101569	-0.219691474
ENSG00000137142	IGFBPL1	1.647989	1.153379	1.003893	0.579712	0.22536	0.434286	0.816911	1.041683	1.18356	0.002557631	-1.422951733	0.609600119	-0.219686269

Gene ID	Gene name	Ctrl-1 FPKM	Ctrl-2 FPKM	Ctrl-3 FPKM	IFNγ-1 FPKM	IFNγ-2 FPKM	IFNγ-3 FPKM	Sora-1 FPKM	Sora-2 FPKM	Sora-3 FPKM	Ctrl vs IFNγ DESeq2 FDR	Ctrl vs IFNγ DESeq2 log2FC	Ctrl vs Sora DESeq2 FDR	Ctrl vs Sora DESeq2 log2FC
ENSG00000165115	KIF27	1.091523	1.022451	1.064425	0.988862	0.888478	0.694848	0.968325	0.959813	0.863597	0.304378663	-0.252568715	0.305274089	-0.219661379
ENSG00000117174	ZNHIT6	5.383623	4.867398	5.941487	3.183965	3.004128	3.105892	4.799899	3.343449	4.70005	0.001320497	-0.375701113	0.077576177	-0.219652771
ENSG00000107951	MTPAP	4.265783	4.149288	5.236886	4.379909	4.205731	4.105742	4.137939	3.513548	3.676524	0.842748086	0.031166204	0.08152392	-0.219489082
ENSG00000182108	DEXI	9.203183	8.955377	9.469388	8.380874	6.959532	10.230625	9.549065	7.832963	8.79057	0.664614014	0.073296755	0.066806251	-0.219487208
NewGene_2391	--	0.757317	0.769105	0.702831	2.443095	2.750636	3.107442	0.508134	0.987756	0.823353	3.12E-26	2.086264886	0.579299476	-0.219456571
ENSG00000175893	ZDHHC21	2.170481	2.813315	2.748082	2.617648	2.170669	2.064943	2.003213	1.887738	2.343825	0.882375319	-0.027655684	0.091425336	-0.219403184
ENSG00000137266	SLC22A23	10.936636	10.144022	9.627669	11.949569	12.489957	12.13795	7.991172	7.385451	9.812927	1.38E-08	0.396218008	0.035205821	-0.219326172
ENSG00000120948	TARDBP	47.205582	51.282202	47.829505	49.415293	45.741135	44.491069	46.146878	37.674655	38.826303	0.206381209	0.071612164	0.00011664	-0.219269813
ENSG00000169213	RAB3B	4.04704	4.284574	4.193682	3.300803	3.531708	3.534688	3.448849	3.146739	3.498505	0.138481808	-0.124436195	0.005092267	-0.219197387
ENSG00000159593	NAE1	40.739082	41.304278	43.4266	39.620905	33.594997	37.213473	33.240811	33.100527	34.708984	0.42833164	-0.07039889	0.002343067	-0.219159412
ENSG00000157693	TMEM268	3.154546	3.549626	4.159743	3.087824	2.68596	2.386839	3.861051	2.640023	3.450014	0.070205643	-0.273268265	0.125006057	-0.21893205
ENSG00000116191	RALGPS2	13.040435	13.943905	13.721287	12.554428	13.143614	11.787319	13.079929	10.447954	12.488245	0.797691889	-0.023197481	0.0016497	-0.218931583
ENSG00000168143	FAM83B	2.631096	2.852513	1.983783	2.391382	2.262458	2.208377	2.277185	1.738326	2.004298	0.92519573	0.024959506	0.273561447	-0.218852257
ENSG00000241258	CRCP	20.026723	19.449772	20.520497	16.44799	15.932962	17.118766	16.301443	16.483336	16.193882	0.05079995	-0.146009318	0.004309733	-0.218299807
ENSG00000088256	GNA11	23.532766	23.042522	23.391209	23.469946	24.611392	24.893126	18.678939	17.765083	19.840861	0.000705853	0.216445265	0.001148764	-0.218297271
ENSG00000169131	ZNF354A	2.521183	2.856601	3.464096	2.932456	2.704268	2.660064	2.493436	2.30093	2.396226	0.856847974	0.046103905	0.267079604	-0.218053738
ENSG00000124767	GLO1	102.718246	106.993126	107.225639	103.824852	97.562103	99.140617	85.991577	83.460983	86.014412	0.241991981	0.07038804	0.000259911	-0.217949617
ENSG00000169093	ASMTL	0.993637	1.074257	1.151159	0.984572	0.880955	1.404174	0.669517	0.797258	1.102275	0.624372019	0.183695137	0.518012703	-0.217792985
ENSG00000134775	FHOD3	1.388583	1.378404	1.516633	1.517588	1.212962	1.217772	1.203394	1.016041	1.16457	0.994344679	0.002093064	0.236263133	-0.21777463
ENSG00000147536	GINS4	8.729598	8.481182	8.307217	7.497628	5.138707	5.928417	7.077603	7.434563	7.417201	0.005719954	-0.313514461	0.021505473	-0.217766768
ENSG00000037474	NSUN2	44.410786	44.92585	50.242275	41.746245	38.864075	39.402043	36.589546	34.635585	41.309413	0.393189262	-0.068867534	0.005627781	-0.217764217
ENSG00000181610	MRPS23	10.683982	10.682386	10.224356	9.358934	8.573184	10.36432	8.166273	9.062537	8.173648	0.711065074	-0.041204024	0.013266073	-0.21773883
ENSG00000089057	SLC23A2	15.44302	16.694714	18.440598	12.737143	11.14909	11.293374	12.936899	12.694505	15.208872	0.000000632	-0.376794335	0.012133461	-0.217724596
ENSG00000147650	LRP12	3.736876	3.385326	3.396515	2.934	2.687334	3.037552	2.727839	2.854254	2.889433	0.372032775	-0.129694366	0.092924204	-0.217715916
ENSG00000180071	ANKRD18A	0.54887	0.826136	0.647196	0.640877	0.355391	0.589094	0.638541	0.395485	0.581006	0.627838194	-0.188806904	0.501480204	-0.217431056
ENSG00000133997	MED6	16.044367	15.943947	14.598075	14.982077	12.062066	13.168992	12.768029	10.748872	12.625219	0.67599865	-0.051394344	0.024341101	-0.217338571
ENSG00000144161	ZC3H8	2.321951	3.466507	1.840641	1.96762	2.268694	1.397479	2.839941	2.925398	2.389977	0.111024961	-0.273633567	0.204227163	-0.217248893
NewGene_1766	--	0.889286	0.822062	0.643811	0.600328	0.550992	0.612548	0.617005	0.530956	0.755927	0.371430736	-0.271090919	0.451882609	-0.217235396
ENSG00000150457	LATS2	3.972749	3.955157	4.280354	2.43238	2.330584	2.413176	3.127485	2.946282	3.798791	2.96E-10	-0.618772222	0.062861534	-0.217225134
ENSG00000119283	TRIM67	0.555955	0.510434	0.633525	0.136655	0.144516	0.230819	0.460821	0.419359	0.451423	4.47E-10	-1.519653415	0.299802477	-0.217060947
ENSG00000103326	CAPN15	9.653347	9.567223	8.213653	9.896592	9.941968	11.253752	7.053923	6.835719	8.235446	0.000504719	0.315018355	0.029156944	-0.216827593
ENSG00000163125	RPRD2	10.940368	10.800089	9.543151	11.228058	10.219013	10.887069	8.803753	7.849901	9.339442	0.008380267	0.185312915	0.005406533	-0.216668796
ENSG00000150593	PDCD4	20.555168	22.272871	22.447142	21.381382	19.514002	18.918204	18.004184	16.884419	18.503419	0.986348802	0.001864534	0.003651293	-0.21653257
NewGene_5647	--	1.363723	1.409235	1.281953	1.009703	1.005485	1.036309	1.027843	1.177486	1.055878	0.278242765	-0.261480838	0.352089485	-0.216494347
ENSG00000147459	DOCK5	6.081238	7.009741	7.992772	6.533401	5.453013	4.749047	6.041486	5.724323	6.489171	0.098227571	-0.198236717	0.032494616	-0.215747369
ENSG00000160703	NLRX1	5.584003	5.02098	4.313692	4.476531	4.291419	4.392312	3.666418	3.533662	5.021395	0.673863954	-0.066011981	0.149206811	-0.21569548
ENSG00000013563	DNASE1L1	4.070422	4.243147	4.00248	4.357291	4.033981	3.980716	3.353158	3.062016	3.645063	0.122339044	-0.244856471	0.163143629	-0.215665296
ENSG00000087502	ERGIC2	8.725066	8.879289	10.265651	7.208853	7.98186	6.82591	6.597764	6.903946	7.528952	0.16898942	-0.124295101	0.012060712	-0.215625799
ENSG00000143190	POU2F1	3.042789	3.338295	3.235946	3.161554	3.095756	3.086282	2.794471	2.42409	2.495773	0.155459375	0.12148538	0.020809863	-0.215589722
ENSG00000023572	GLRX2	6.609221	6.475974	6.90932	5.728446	5.089175	4.340842	4.667386	8.305029	4.221318	0.282648666	-0.153889415	0.10097178	-0.215567418
ENSG00000170903	MSANTD4	7.921082	7.728322	7.845715	7.527162	6.376311	6.841645	6.804998	5.97453	6.102103	0.983561517	0.002984821	0.035016568	-0.215375945
ENSG00000138382	METTL5	34.219036	35.272792	33.45902	31.036753	27.328393	30.885146	28.391573	28.885587	23.133249	0.440457886	-0.081968098	0.059451602	-0.215281088
ENSG00000182985	CADM1	0.539173	0.676602	0.982981	0.379051	0.259708	0.442643	0.380045	0.410626	0.518507	0.0000646	-0.942144388	0.361755727	-0.215159834
ENSG00000141580	WDR45B	32.32034	29.541642	30.473411	30.820489	32.619424	30.898299	25.349486	23.438517	26.109543	0.083148776	0.132537268	0.004258604	-0.214939357
ENSG00000120278	PLEKHG1	1.507814	1.354229	0.984377	0.961627	0.980948	1.193444	1.154609	0.966671	1.093337	0.166168589	-0.274288383	0.277316314	-0.214932664
ENSG00000175707	KDF1	5.798279	6.519226	6.307018	4.418164	4.683935	4.389196	4.90444	4.274623	5.898629	0.027552699	-0.315712653	0.168906167	-0.214578035
ENSG00000074527	NTN4	1.079098	0.938561	0.758894	1.627488	1.60512	1.660717	0.560656	0.814849	0.880528	0.000000673	0.951433158	0.469828344	-0.214348608
ENSG00000197555	SIPA1L1	7.209105	7.39478	7.657112	7.451129	6.687044	6.584397	5.966004	6.129316	6.695934	0.741254352	0.03448106	0.013714404	-0.214270573
ENSG00000118564	FBXL5	19.886172	18.995827	20.726158	22.559758	20.640701	20.337405	17.983314	18.460885	18.848675	2.99E-14	0.541956144	0.015458536	-0.214239854
ENSG00000133606	MKRN1	14.437906	14.439801	14.616239	16.389963	16.682994	15.545692	12.806362	13.183331	12.442913	0.000183677	0.275469383	0.011600513	-0.213963044
ENSG00000169967	MAP3K2	4.885398	5.116524	4.877722	5.180455	5.542096	5.229308	4.382541	4.028994	3.794219	0.000892828	0.266661239	0.046023687	-0.21385148
ENSG00000165259	HDX	0.386151	0.361692	0.328744	0.877315	0.500449	0.40345	0.27765	0.243432	0.473723	0.022864233	0.670347175	0.557992469	-0.213783364
ENSG00000135093	USP30	5.753742	5.751817	5.340338	8.253349	7.301388	7.023414	4.63482	4.212606	4.739542	0.000396231	0.342238797	0.050048975	-0.213760735
ENSG00000164096	C4orf3	19.60571	19.359791	18.469725	16.101024	13.523903	16.489269	14.494706	14.926715	13.500557	0.074888178	-0.145308716	0.016831123	-0.213541475
ENSG00000036257	CUL3	18.278748	18.855204	20.105974	16.04262	13.21574	15.088697	16.108689	14.707016	16.555307	0.005566326	-0.184683161	0.000999761	-0.213541199
ENSG00000154710	RABGEF1	11.697435	12.560575	13.887548	12.319315	13.485226	11.96025	9.708299	10.035759	11.725898	0.080703786	0.15815731	0.014201542	-0.21341703
ENSG00000158882	TOMM40L	40.484464	37.691567	36.493938	37.263931	39.663179	38.179757	29.742823	29.359148	33.974786	0.034974765	0.152091956	0.004935658	-0.2133737
ENSG00000119185	ITGB1BP1	8.123597	7.244145	7.941807	6.791284	6.506443	6.904364	6.624124	6.048079	7.732777	0.076118738	-0.184141359	0.060843791	-0.213251447
ENSG00000119965	C10orf88	3.404079	3.751248	3.938852	3.329252	3.370376	3.022253	3.491445	2.770282	2.698653	0.83183933	-0.044353586	0.224096765	-0.213049455
ENSG00000158042	MRPL17	20.835746	21.152011	18.056616	18.410182	19.013756	18.817396	15.725229	16.862602	15.78874	0.357683222	0.094699669	0.045286605	-0.213020901
ENSG00000130304	SLC27A1	0.672876	0.830279	0.694681	0.290195	0.250843	0.150099	0.657088	0.544039	0.513992	0.000234886	-1.375665461	0.548278218	-0.212628739
ENSG00000255112	CHMP1B	17.465282	17.519869	19.045074	15.241023	16.223059	16.314054	14.097491	13.62022	16.05689	0.790291183	-0.028917857	0.012151067	-0.212600429
ENSG00000123933	MXD4	47.806987	46.183335	39.350177	39.108701	40.199985	40.277778	33.700437	33.595025	38.432044	0.892345151	-0.015551099	0.011963603	-0.212502897
ENSG00000196363	WDR5	22.477983	22.335758	23.006707	20.804183	20.85037	21.785343	17.509204	16.620623	20.742068	0.555818764	0.049587534	0.011339704	-0.212246739
ENSG00000146373	RNF217	0.781234	1.074	1.101102	0.91017	0.814597	0.892892	0.807496	0.996273	0.864434	0.686674823	-0.084456242	0.220204658	-0.212052454
ENSG00000123684	LPGAT1	21.699208	24.331049	25.227306	19.091058	18.739106	18.486883	19.736773	17.820949	20.280455	0.005725626	-0.19006955	0.005460453	-0.212029998
ENSG00000180182	MED14	6.845853	7.373648	7.637584	6.371465	5.886916	6.104137	5.688396	5.817226	6.489093	0.237804235	-0.102136693	0.010179616	-0.212001487
ENSG00000021488	SLC7A9	1.335155	1.440258	1.031243	0.714192	0.812888	0.926922	1.111524	0.880729	0.933984	0.277625907	-0.389228276	0.523864279	-0.211976096
ENSG00000205476	CCDC85C	3.469634	3.49242	3.282739	3.419913	3.635239	3.742819	2.805749	2.505377	2.957955	0.151889455	0.125120476	0.010916581	-0.211933902
ENSG00000056277	ZNF280C	1.300213	1.435671	1.492953	1.550574	1.30017	1.331514	1.155885	0.999119	1.278168	0.529243372	0.13073983	0.261955776	-0.211886483
ENSG00000164659	KIAA1324L	0.466481	0.337727	0.302521	0.27628	0.337477	0.283574	0.26822	0.286742	0.291025	0.859161971	-0.075234283	0.517971074	-0.211884117
ENSG00000197702	PARVA	3.535217	3.673938	3.514047	3.244907	2.526155	2.54055	3.169148	3.62923	3.128668	0.0000721	-0.346049292	0.028311353	-0.211716087
ENSG00000101193	GID8	19.926994	19.186789	19.370729	20.232414	19.582077	19.871113	15.685918	15.318025	16.371887	0.003997564	0.176286915	0.001783057	-0.211592277
ENSG00000128791	TWSG1	2.065111	2.615116	2.63969	2.311735	2.274183	2.312563	1.94186	1.905437	2.084819	0.772162563	0.060688618	0.209523065	-0.21142168

Gene ID	Gene name	Ctrl-1 FPKM	Ctrl-2 FPKM	Ctrl-3 FPKM	IFNγ-1 FPKM	IFNγ-2 FPKM	IFNγ-3 FPKM	Sora-1 FPKM	Sora-2 FPKM	Sora-3 FPKM	Ctrl vs IFNγ DESeq2 FDR	Ctrl vs IFNγ DESeq2 log2FC	Ctrl vs Sora DESeq2 FDR	Ctrl vs Sora DESeq2 log2FC
ENSG00000143353	LYPLAL1	8.636202	8.950246	9.125152	8.462994	7.797769	8.273501	7.331187	7.395206	6.855137	0.882442267	0.023740851	0.086133549	-0.211399648
ENSG00000144366	GULP1	2.690537	2.94701	3.108807	1.723036	1.922457	2.022333	2.993526	2.200447	2.832527	0.0000263	-0.71852824	0.258979202	-0.211334421
ENSG00000066777	ARFGEF1	6.123602	6.168245	6.464147	5.243663	5.346078	5.384866	4.996339	4.734493	5.580508	0.299670857	-0.092292229	0.014627634	-0.211091637
ENSG00000110031	LPXN	2.105424	1.833067	2.148431	1.808827	1.744607	1.40638	1.848723	1.442079	1.634098	0.680381011	-0.124249677	0.395417112	-0.210965818
ENSG00000169018	FEM1B	7.984596	8.846355	8.34031	9.723691	9.485605	9.185347	7.238079	6.00677	7.421382	0.00000241	0.318344903	0.016552343	-0.2108717
ENSG00000138835	RGS3	11.057345	10.723596	9.338246	5.137571	5.780714	4.744281	7.712287	6.70151	8.118044	4.61E-12	-0.748593701	0.068080266	-0.210759139
ENSG00000115267	IFIH1	3.793144	2.93826	3.309706	13.038597	12.446273	13.327478	3.124901	2.373865	3.256022	4.24E-124	2.093651117	0.190269743	-0.210311155
ENSG00000188763	FZD9	3.895588	3.361151	3.63599	1.591467	2.631142	1.762506	2.544786	3.040668	3.238824	0.000365946	-0.713523866	0.225330331	-0.210310568
ENSG00000152782	PANK1	4.841965	4.820592	4.313299	5.320528	5.292243	5.043297	3.070025	2.87554	3.493969	0.000124525	0.352549014	0.063406024	-0.210205142
NewGene 6614	--	3.275139	3.368358	3.164001	4.405495	3.413588	1.670543	3.149486	1.200379	2.21686	0.630990042	0.149295115	0.365369692	-0.2101294
ENSG00000124602	UNC5CL	4.006511	3.589178	3.410873	4.223378	4.153505	4.159022	3.061112	2.815447	2.996499	0.009843144	0.33273743	0.151207938	-0.210088006
ENSG00000145569	OTULINL	1.539174	1.476852	1.450309	1.159182	1.025315	1.24583	1.253819	1.154251	1.215341	0.289784289	-0.230917808	0.293587024	-0.209915107
ENSG00000166845	C18orf54	0.833086	1.80565	1.396315	1.008729	0.616669	0.85449	0.871253	0.9018	1.166525	0.000536796	-0.823768661	0.405509451	-0.209874454
ENSG00000117682	DHDDS	7.707253	8.372851	8.221527	8.987879	7.678043	7.222115	6.755208	6.992878	6.891758	0.98475795	-0.003124175	0.059956228	-0.20984985
NewGene 5775	--	1.61663	1.61601	1.718622	1.19047	1.26477	1.586453	1.546107	1.541947	1.19662	0.680172192	-0.114729373	0.365367656	-0.209789608
ENSG00000134755	DSC2	3.966757	3.36824	3.602205	3.385163	2.336177	2.571903	2.620322	3.210877	2.194116	0.000521433	-0.297121672	0.021238713	-0.209752304
ENSG00000151353	TMEM18	6.371168	6.365578	6.813554	5.05562	5.265969	5.395868	5.738796	5.063312	4.760066	0.341718229	-0.127277803	0.110889229	-0.209514686
ENSG00000165449	SLC16A9	0.630263	0.605191	0.592039	0.409262	0.396928	0.428456	0.597628	0.435002	0.448494	0.173992594	-0.411419251	0.493030398	-0.209499168
ENSG00000104218	CSPP1	8.166173	9.215922	9.090959	7.03626	6.682907	6.653221	7.008104	7.307941	7.253002	0.004476389	-0.248949946	0.025341097	-0.209469449
ENSG00000151611	MMAA	1.821021	1.312649	1.756584	3.721259	3.258923	1.679673	1.3809	1.341696	1.718011	3.17E-13	1.123696663	0.360262674	-0.209358078
ENSG00000141526	SLC16A3	24.147898	21.672338	17.935998	13.968306	15.795718	14.659208	17.864746	17.75494	21.000281	0.002276988	-0.338195509	0.072386184	-0.209263541
ENSG00000076770	MBNL3	57.472008	67.663905	68.048027	72.425026	66.974386	72.596313	50.602818	48.608708	61.464946	0.000140233	0.270137402	0.00860349	-0.2092489
ENSG00000221995	TIAF1	0.674164	0.732074	0.706639	1.157519	0.686655	0.878563	0.702256	0.447862	0.567661	0.028861664	0.510117758	0.424082188	-0.209223191
ENSG00000136193	SCRN1	45.345166	43.136842	45.48617	34.137746	33.055238	33.567746	36.898753	34.432764	37.414889	0.000000195	-0.264701713	0.000486302	-0.209221547
ENSG00000065883	CDK13	15.20669	13.728182	15.762935	14.551385	14.584573	14.244898	13.086418	11.565964	12.201136	0.004527295	0.172524762	0.004556393	-0.209212167
ENSG00000114127	XRN1	3.037304	3.087436	3.128624	7.683287	7.510708	7.359789	2.562611	2.411586	3.130859	6.54E-119	1.497042823	0.028905926	-0.20919947
ENSG00000137038	DMAC1	5.848867	5.882401	5.980624	4.094829	4.220872	4.793997	5.04479	5.154903	4.129234	0.022272283	-0.28380758	0.138225854	-0.209150822
ENSG00000122490	PQLC1	11.480285	10.737842	9.323255	10.633829	11.602597	12.949625	8.492675	8.116118	9.595915	0.034526676	0.242427692	0.073791755	-0.209049951
ENSG00000124787	RPP40	6.08285	6.905982	6.398665	4.011805	5.681924	5.593251	6.262555	4.810329	4.069787	0.241268123	-0.216230922	0.281161564	-0.208969647
ENSG00000123066	MED13L	8.825667	7.220485	6.581499	7.532193	6.504937	6.394714	7.434632	4.722764	6.511091	0.096854448	0.11290407	0.010259108	-0.208787982
ENSG00000108557	RAI1	12.164487	11.160445	10.520922	10.274721	10.114972	11.016555	10.758786	8.811156	11.031525	0.978907608	-0.00346738	0.042707412	-0.208751224
ENSG00000076706	MCAM	5.52936	4.787262	5.265149	2.456983	1.994869	1.838062	4.11036	3.95243	4.596877	3.43E-19	-1.163007988	0.09030077	-0.208650037
ENSG00000266412	NCOA4	87.671922	82.956859	76.579818	98.098097	101.48055	98.891567	67.928927	66.417432	66.658974	5.97E-12	0.404875949	0.002958574	-0.208622706
ENSG00000123200	ZC3H13	4.709508	4.956171	4.801124	4.392624	4.064682	4.092047	3.714511	3.855955	4.275358	0.697116087	-0.043094329	0.020699505	-0.208554645
ENSG00000155034	FBXL18	3.665156	3.690409	3.565144	3.220267	3.04547	3.203869	2.862863	2.671066	3.619271	0.823670436	-0.0436746	0.168609647	-0.208486421
ENSG00000163349	HIPK1	4.867181	4.914736	5.24361	4.952639	4.53531	4.525545	4.385414	3.691295	4.496975	0.761768675	0.033454544	0.023128729	-0.208211905
ENSG00000128881	TTBK2	1.455285	1.438231	1.724363	1.852389	1.468632	1.626607	1.470931	1.374964	1.391693	0.110260935	0.205475516	0.100363913	-0.208141483
ENSG00000134317	GRHL1	2.675301	2.885734	3.728728	3.113523	2.66772	2.312545	2.627861	2.370476	2.704165	0.737342156	-0.076065553	0.221213491	-0.207971727
ENSG00000128039	SRD5A3	5.087373	5.560603	5.136087	4.49245	3.969447	4.605708	4.463374	3.953578	4.569705	0.21396677	-0.148177294	0.05360855	-0.207963371
ENSG00000182957	SPATA13	6.494976	7.002808	6.722047	7.553894	6.688081	6.788921	5.793685	4.602212	6.039725	0.008772136	0.186869285	0.032690661	-0.207893994
ENSG00000168807	SNTB2	1.500625	1.736973	1.803388	2.111685	1.853784	1.802275	1.635794	1.108422	1.348819	0.003526444	0.341546482	0.184280756	-0.207888181
ENSG00000171634	BPTF	13.162781	13.958632	14.541842	13.785809	13.154033	12.800273	12.611633	10.955864	12.235891	0.92213898	-0.010353755	0.002243285	-0.207874108
ENSG00000187555	USP7	33.790958	34.515071	34.734091	33.045536	30.745171	31.485749	27.819647	25.713256	30.223731	0.681193672	0.028912325	0.001079952	-0.207606464
ENSG00000214194	SMIM30	23.196455	24.594605	29.803487	19.38447	21.190585	21.87364	19.668165	20.615457	21.682181	0.298973828	-0.140057705	0.08148618	-0.207486846
ENSG00000010017	RANBP9	23.596586	23.230988	25.641237	23.756752	24.473309	24.339027	20.271824	18.200504	20.441946	0.041726843	0.149761111	0.007872316	-0.207140653
ENSG00000094975	SUCO	14.885295	15.630202	16.062499	14.121578	13.84078	13.613021	12.807652	12.076561	13.008229	0.872788638	-0.014958988	0.003300538	-0.206921401
ENSG00000162924	REL	2.146952	2.303067	2.53941	2.425669	2.214693	1.978191	2.513882	1.573796	2.100212	0.69592426	0.055263883	0.146146401	-0.206809687
NewGene 900	--	1.30554	1.144801	1.148857	0.815946	1.076952	1.07199	0.949746	0.757551	0.99003	0.471721164	-0.263087679	0.493480799	-0.206697236
ENSG00000029364	SLC39A9	20.426359	19.502114	20.700439	19.453586	18.601517	19.035407	17.058824	16.022438	17.667364	0.593139678	0.042100415	0.002724311	-0.206687149
ENSG00000107651	SEC23IP	9.453774	9.554717	9.895752	9.267557	8.485637	8.505719	9.069532	7.806095	8.098838	0.807909569	-0.023548266	0.008895394	-0.206563086
ENSG00000078668	VDAC3	60.943976	62.04443	61.122698	56.305252	52.521793	53.19703	48.664489	48.124425	52.686183	0.661244638	-0.036562901	0.002619955	-0.206441532
ENSG00000158528	PPP1R9A	5.122072	5.202368	4.586608	4.992314	4.428401	3.255599	4.846635	5.083124	4.305434	0.005262795	-0.244560927	0.013827197	-0.206413054
ENSG00000142279	WTIP	0.544342	0.62855	0.687955	0.419323	0.333712	0.405853	0.543661	0.393198	0.563157	0.000792457	-0.634599519	0.306003018	-0.206411645
ENSG00000101452	DHX35	10.238802	10.589483	10.404061	8.869168	9.071929	8.749156	8.705648	7.427582	9.473654	0.469981828	-0.073213498	0.041478942	-0.206366111
ENSG00000033627	ATP6V0A1	16.115754	15.849024	16.061005	13.695671	12.830448	12.053276	13.123699	11.94034	14.220056	0.028544014	-0.165713032	0.014239475	-0.206214046
ENSG00000167595	PROSER3	5.307726	6.688238	4.526498	4.392191	3.724649	4.044464	4.060085	4.460727	5.613283	0.248145671	-0.197214727	0.198076205	-0.206157609
ENSG00000083857	FAT1	14.292509	12.935965	13.627969	8.672941	8.124715	8.011815	10.594246	10.421179	12.56051	1.38E-22	-0.562244584	0.006220764	-0.206131926
NewGene 2910	--	0.478125	0.528868	0.585284	0.428202	0.377599	0.262846	0.472536	0.458645	0.464335	0.47775706	-0.337222993	0.638550337	-0.206002203
ENSG00000108219	TSPAN14	3.955717	4.290848	5.249374	4.851072	4.032475	2.547982	3.975055	3.884026	3.585099	0.000376941	-0.27153133	0.034306948	-0.205616717
ENSG00000011009	LYPLA2	73.236982	72.937771	69.753527	47.943215	48.519503	53.40917	54.748722	57.929239	60.773147	4.62E-08	-0.360686646	0.004070605	-0.205508725
ENSG00000177034	MTX3	4.575222	5.214393	5.787222	4.998039	4.636693	4.672274	4.147443	4.056041	4.420124	0.89481531	0.0189542	0.049441177	-0.205472882
ENSG00000176171	BNIP3	13.329643	12.42094	9.34098	9.147352	9.697625	10.253336	10.165923	9.08176	9.168123	0.416500249	-0.120238904	0.122581968	-0.205389344
ENSG00000152455	SUV39H2	8.669463	9.598614	10.002024	8.088573	8.255286	10.049369	8.764557	9.77033	7.929685	0.347771717	-0.124778252	0.086226113	-0.205275465
ENSG00000019995	ZRANB1	7.533923	7.996271	8.124178	7.786906	7.678499	7.540545	6.912315	5.913083	6.416465	0.203586904	0.107641701	0.02281116	-0.205238401
NewGene 6592	--	2.87756	2.385517	2.808774	2.352658	2.440546	2.248666	2.135592	1.992674	2.14134	0.950429406	-0.0115776	0.107827701	-0.204980416
ENSG00000139233	LLPH	4.686517	5.33975	5.690836	4.829305	4.242157	4.084309	5.083329	4.619905	4.652602	0.120936842	-0.162811619	0.045289804	-0.204871778
ENSG00000091039	OSBPL8	11.789604	13.983414	14.602074	14.229527	11.775401	11.883923	10.414025	10.329061	12.153326	0.871600571	-0.019550003	0.03019912	-0.204770992
ENSG00000176623	RMDN1	18.618375	21.147692	19.488446	18.981273	18.689317	18.239492	15.938735	16.121747	15.974885	0.815222874	0.025886344	0.014340279	-0.204746394
ENSG00000198642	KLHL9	10.488256	10.438631	9.97456	10.397086	9.829556	10.501963	8.675662	7.754338	8.739311	0.058835491	0.13944405	0.008744629	-0.204656931
ENSG00000106609	TMEM248	26.575912	27.862904	29.102972	29.009226	27.975552	27.633112	22.131007	21.691805	24.670429	0.006349772	0.168920849	0.003677387	-0.204188292
NewGene 2039	--	1.12445	1.339069	0.729788	0.739187	0.605559	0.785424	0.735237	0.642551	1.228865	0.366265179	-0.329406003	0.576492	-0.20413631
ENSG00000128573	FOXP2	2.037559	2.596163	2.297923	2.406915	2.354079	2.155103	1.939792	2.309888	2.728559	0.059544173	0.237137096	0.130153794	-0.203974164
ENSG00000112200	ZNF451	7.626485	8.679989	8.332705	8.830284	8.235411	8.088134	7.733249	6.583642	6.589998	0.054791375	0.164638128	0.037602635	-0.203881978
ENSG00000174109	C16orf91	11.363265	10.250042	9.190514	9.469408	8.731467	10.611336	8.61475	7.858813	8.630562	0.80002832	0.052473092	0.195197495	-0.203847352

Gene ID	Gene name	Ctrl-1 FPKM	Ctrl-2 FPKM	Ctrl-3 FPKM	IFNγ-1 FPKM	IFNγ-2 FPKM	IFNγ-3 FPKM	Sora-1 FPKM	Sora-2 FPKM	Sora-3 FPKM	Ctrl vs IFNγ DESeq2 FDR	Ctrl vs IFNγ DESeq2 log2FC	Ctrl vs Sora DESeq2 FDR	Ctrl vs Sora DESeq2 log2FC
ENSG00000028116	VRK2	19.312178	19.500431	21.998426	18.99679	16.888526	16.030198	17.177201	15.945375	17.261658	0.36936926	-0.095456385	0.026828919	-0.2037986
ENSG00000063438	AHRR	0.932239	0.635509	0.703006	0.63049	1.288569	1.226797	0.779273	0.600767	0.964113	0.354103873	0.2338011	0.453115531	-0.203323906
ENSG00000166147	FBN1	2.256202	2.356814	2.600927	1.841279	2.025459	1.961853	1.842909	1.627692	2.115453	0.253442003	-0.12667408	0.067254955	-0.203246859
ENSG00000087903	RFX2	2.14987	1.674763	1.601446	1.482463	1.498566	1.694175	1.444014	1.432717	1.878005	0.492913437	-0.15586528	0.303104117	-0.203177554
ENSG00000118096	IFT46	2.852203	2.56639	3.124333	2.229964	2.333097	1.986043	2.761008	2.204448	2.157966	0.229031691	-0.275845934	0.366531816	-0.203167885
ENSG00000168994	PXDC1	5.393997	4.759609	5.257254	8.700533	7.890231	8.298977	4.39798	4.136404	3.95191	7.13E-14	0.814655469	0.170801085	-0.203161919
ENSG00000137285	TUBB2B	14.702057	12.497075	10.871199	9.467487	9.909822	9.22891	9.89321	11.690613	9.326834	0.035830593	-0.265257667	0.167030855	-0.20310448
ENSG00000051128	HOMER3	31.940488	26.605507	25.283528	17.641093	16.483735	17.416478	21.5057	23.117623	24.264024	4.73E-10	-0.575734006	0.0553163	-0.202993812
ENSG00000198455	ZXDB	1.405156	1.331929	1.570751	1.444935	1.113057	1.25775	1.294464	1.006827	1.210409	0.902995909	-0.028732681	0.244093236	-0.20288289
ENSG00000180035	ZNF48	9.800959	9.535495	9.180028	7.104595	7.364409	7.804825	6.399518	6.603029	9.261635	0.128716566	-0.151589048	0.115452015	-0.202863701
ENSG00000108666	C17orf75	9.716291	10.346496	9.743306	8.179057	7.312899	7.901041	9.792969	8.766686	8.281526	0.081394205	-0.197327293	0.060843791	-0.202814882
ENSG00000170734	POLH	1.875176	2.29543	1.554371	1.861889	1.489732	1.523599	1.40986	1.724453	1.536727	0.738990656	-0.086856346	0.343893077	-0.202794826
ENSG00000173786	CNP	20.569974	20.360798	19.021258	18.444717	20.359624	21.134444	15.591748	16.596708	17.44071	0.143536745	0.121061385	0.011142423	-0.202758773
ENSG00000124541	RRP36	57.550058	59.009056	57.908038	60.226223	55.302959	57.621897	48.043451	48.348037	46.355342	0.047540263	0.137415348	0.009975624	-0.202672749
ENSG00000141385	AFG3L2	23.636171	22.477148	22.812189	18.637217	18.382191	19.018908	18.059685	18.543568	19.576472	0.029300749	-0.151080591	0.005478904	-0.202649828
ENSG00000160058	BSDC1	9.465871	8.897569	8.707298	8.92022	7.910157	7.871395	8.380394	9.081665	9.725825	0.583011254	-0.058577759	0.025341097	-0.202603189
NewGene 1541	--	5.214726	5.262938	7.026777	5.13801	5.771124	5.218142	4.353857	4.683864	4.92985	0.732306435	0.079380704	0.284668659	-0.202566274
ENSG00000143153	ATP1B1	137.743773	128.815952	146.902671	140.033156	154.595769	141.804375	109.4694	113.801659	117.808822	0.001784921	0.204759533	0.003274863	-0.202394465
ENSG00000144218	AFF3	1.945676	1.502761	1.758274	1.071534	0.673064	1.09843	1.373483	1.102411	1.577718	0.0000241	-0.637811803	0.116369345	-0.202308863
ENSG00000107816	LZTS2	15.344615	12.176597	12.760624	12.193219	12.045697	13.729978	8.870924	9.1544	11.006435	0.629307326	0.051180434	0.031266247	-0.202255108
ENSG00000181894	ZNF329	6.450898	6.5693	5.576994	4.471634	4.058232	3.960368	4.923442	5.186914	5.18384	0.0000028	-0.478389271	0.065729317	-0.202146981
ENSG00000089022	MAPKAPK5	12.015968	12.94926	14.044253	13.186489	10.707647	10.542731	13.719583	10.851956	14.119798	0.526145682	0.05784458	0.004796703	-0.201953023
NewGene 879	--	0.707695	0.547341	0.585784	0.36247	0.574373	0.364537	0.53853	0.45238	0.443979	0.34475111	-0.355661462	0.55493181	-0.201837957
ENSG00000249709	ZNF564	3.259658	3.746813	4.32039	3.734619	3.230264	3.701116	3.797029	3.55163	2.041422	0.802417185	0.050869534	0.63390004	-0.201771057
ENSG00000160326	SLC2A6	10.109316	10.361804	9.665257	8.301502	9.028622	9.187969	7.214218	7.99535	9.589133	0.865635807	-0.024347232	0.088657435	-0.2015865
ENSG00000108443	RPS6KB1	11.002943	12.143555	14.926542	13.079371	13.196463	11.912353	10.110917	11.027176	11.040798	0.136565284	0.152614733	0.044289932	-0.201363884
ENSG00000146197	SCUBE3	1.078839	0.854486	0.805129	0.487495	0.373573	0.332858	0.683346	0.733397	0.821691	0.000000304	-1.053566097	0.314386154	-0.201233512
ENSG00000170619	COMMD5	20.47456	18.435306	19.198619	14.906594	18.587223	18.351087	14.862182	16.503603	15.755977	0.96555674	0.007815444	0.058391909	-0.201053783
ENSG00000110318	CEP126	0.193417	0.12853	0.135899	0.089769	0.076885	0.125793	0.103914	0.109584	0.177824	--	--	0.678505739	-0.201041099
ENSG00000132510	KDM6B	7.584605	6.665185	6.862991	7.027162	6.817833	6.648186	5.346896	5.080188	7.182896	0.43252675	0.077798661	0.049238699	-0.200972097
ENSG00000145907	G3BP1	24.090324	27.975251	25.903398	30.027049	24.461982	24.517157	26.643643	22.427484	47.214848	0.088760391	0.103713855	0.00162439	-0.200945705
ENSG00000163938	GNL3	72.836695	71.889994	73.810912	69.258883	65.094444	65.083529	60.422036	57.309804	60.18973	0.8366477	0.016605125	0.001329415	-0.20084831
ENSG00000123130	ACOT9	6.436231	6.402541	8.949463	5.099958	4.936995	5.459142	5.864535	5.744737	6.666193	0.052072945	-0.244879551	0.111790273	-0.20080569
NewGene 6121	--	1.690862	1.748374	1.545712	1.265031	1.035848	1.242012	1.310132	1.350644	1.491088	0.351381932	-0.293375322	0.492228407	-0.20079417
ENSG00000166415	WDR72	6.248204	6.593695	6.451818	3.813817	4.697009	4.795439	5.049749	4.058809	5.379669	0.0000595	-0.329517989	0.021235782	-0.200780018
ENSG00000141576	RNF157	22.18986	21.264761	19.413739	17.619975	16.618609	16.576044	17.685116	16.269797	17.781382	0.030047639	-0.156307863	0.00685163	-0.200769301
ENSG00000005810	MYCBP2	5.118475	5.700004	5.921736	6.829663	6.034483	5.865697	4.381298	4.315926	6.004985	0.000745584	0.253252896	0.073322976	-0.200728301
ENSG00000148942	SLC5A12	0.928098	0.907072	0.786142	0.285693	0.267431	0.184411	1.175041	0.634405	0.883863	2.56E-13	-1.668646902	0.33652925	-0.200723511
ENSG00000205707	ETFRF1	8.877902	8.903116	10.889481	10.54826	9.70864	9.565621	7.821271	7.766774	7.982893	0.211829999	0.194164888	0.193736171	-0.200713155
ENSG00000174485	DENND4A	8.966484	9.266716	11.189755	13.565653	13.372017	12.521433	7.390197	6.635951	7.639113	8.08E-17	0.65471266	0.038348517	-0.200459642
ENSG00000120158	RCL1	16.309664	16.535828	18.130445	14.76753	13.780324	14.817971	14.687327	13.011264	14.152875	0.339421609	-0.097226046	0.029630277	-0.200239772
ENSG00000166130	IKBIP	6.765215	8.344837	8.378232	7.525991	7.721288	8.238878	6.603734	5.717061	6.78429	0.378310671	0.124755194	0.133440521	-0.200197018
ENSG00000111845	PAK1IP1	49.317127	52.148571	54.205242	41.868942	39.018879	39.931133	42.771862	41.884033	42.352474	0.000863831	-0.218476663	0.006194583	-0.200142295
ENSG00000085719	CPNE3	9.396807	10.447062	9.508169	9.289111	9.53612	9.418601	7.706843	7.594689	8.365034	0.26296514	0.1013999	0.020996608	-0.200141924
ENSG00000256043	CTSO	4.707065	5.33189	5.016564	15.323763	14.493308	13.946816	3.912785	4.216195	4.147825	4.77E-93	1.686322492	0.123603127	-0.200037497
ENSG00000137656	BUD13	10.655198	9.09634	8.626348	8.968963	8.303346	9.088386	8.540778	7.550527	7.984415	0.800631789	0.039777644	0.100412667	-0.199954849
ENSG00000175970	UNC119B	17.623829	17.964481	16.438887	15.40385	15.115029	16.728672	14.406563	13.033442	15.092066	0.93662433	0.009045355	0.009123883	-0.199841976
ENSG00000148396	SEC16A	16.554251	15.188218	13.896262	19.413767	16.769309	18.87773	14.262375	10.565122	13.68487	1.54E-09	0.399729474	0.021560292	-0.199035969
ENSG00000132688	NES	15.170804	13.385941	13.025401	5.333182	5.139173	5.475761	10.233648	11.955982	11.766547	2.7E-60	-1.234844105	0.038479896	-0.198794362
ENSG00000090530	P3H2	2.162515	2.791224	2.87159	0.90241	0.781717	0.729888	2.166757	2.189866	2.01943	3.32E-18	-1.546072783	0.264000735	-0.198788793
ENSG00000151287	TEX30	14.99257	17.161576	17.538513	18.093958	14.763913	16.029039	13.386958	13.738656	14.159979	0.166571419	0.165030632	0.089983616	-0.198556743
NewGene 1136	--	1.257774	1.193064	0.803896	1.01925	0.688676	1.187763	0.974178	0.796692	0.892185	0.960459054	-0.022491294	0.506665153	-0.198248074
ENSG00000129480	DTD2	10.370167	12.204443	12.570987	7.183875	6.741693	7.1019	9.742475	8.780875	10.074407	0.000000797	-0.545005916	0.106650841	-0.198229758
ENSG00000198796	ALPK2	1.374225	1.331334	1.31783	1.792873	1.613737	1.463437	1.182429	1.013479	1.091579	0.001513066	0.421343139	0.193992406	-0.198154826
ENSG00000129474	AJUBA	20.402058	18.281962	19.742066	11.004958	11.959066	12.103238	18.0892	14.456702	17.106405	1.33E-17	-0.589268736	0.015149821	-0.198149905
ENSG00000087077	TRIP6	50.579274	51.170279	49.112051	41.502486	43.716716	45.605742	39.952787	41.895531	43.296601	0.491504028	-0.059657028	0.005945884	-0.198137473
ENSG00000105926	MPP6	34.720861	29.608654	21.999234	23.314122	17.126797	23.277769	16.246113	28.10903	18.785743	0.648680892	-0.047857475	0.034432969	-0.198073434
ENSG00000123595	RAB9A	5.79618	6.1832	6.64997	5.128088	5.108657	5.042977	4.909179	5.286861	5.98999	0.394899676	-0.133237698	0.202449602	-0.198041956
ENSG00000109079	TNFAIP1	17.332201	16.854596	16.818674	13.356362	13.606226	13.253876	13.381243	13.453725	15.266558	0.005525706	-0.194995009	0.012555411	-0.197973225
ENSG00000198728	LDB1	25.740723	24.078247	22.937025	19.759225	19.253113	17.979213	18.647743	19.798581	20.430892	0.018066268	-0.191665012	0.018889771	-0.197885682
ENSG00000163002	NUP35	15.151626	15.005838	16.77795	11.766117	10.448621	11.149774	12.787794	14.130268	14.111907	0.00092183	-0.332435216	0.066630259	-0.197837542
ENSG00000070413	DGCR2	24.4625	24.595207	22.049897	20.479693	19.216138	19.928539	18.26802	18.267117	21.007171	0.203096893	-0.095845797	0.010263731	-0.197693448
ENSG00000072163	LIMS2	0.854115	0.867077	0.693732	0.249011	0.500672	0.274881	0.788248	0.437346	0.930159	0.012749051	-0.964821218	0.611006415	-0.19762709
ENSG00000164338	UTP15	5.51597	5.474294	5.415216	4.832421	4.639906	4.947527	4.578143	4.492421	4.648674	0.495013085	-0.082551228	0.05321397	-0.197541835
ENSG00000111615	KRR1	8.201617	8.971925	9.902684	7.901429	7.610489	7.246939	7.280624	7.092897	7.774361	0.253709184	-0.103299171	0.019190026	-0.197511799
ENSG00000125944	HNRNPR	87.38475	92.731706	90.055359	95.374189	84.980938	85.090157	70.840954	68.492381	77.248606	0.050102404	0.111239984	0.001155524	-0.197503378
ENSG00000169609	C15orf40	2.061168	1.949257	1.914287	1.940704	2.020884	2.085623	2.223588	2.267996	1.935257	0.115904899	0.169092438	0.076475166	-0.197258751
NewGene 5126	--	12.591814	13.177108	14.798872	16.353288	14.936824	13.642656	10.810865	10.634509	11.310468	0.000197365	0.30299809	0.0161255	-0.197188722
ENSG00000182831	C16orf72	14.249536	14.559255	15.310772	16.029712	15.363541	15.342328	13.111122	11.111964	13.746818	0.0000428	0.232819142	0.01045673	-0.196983213
ENSG00000198830	HMGN2	108.041228	124.42832	118.392189	108.433766	94.10856	103.277751	98.609023	93.209276	94.934337	0.591416144	-0.0499309	0.009546329	-0.196976301
ENSG00000221968	FADS3	6.246326	6.22275	5.604241	4.997251	4.673342	4.218474	5.173347	4.834009	4.961568	0.130343251	-0.234359136	0.178060674	-0.196846709
ENSG00000241878	PISD	27.177765	28.277964	27.00361	23.398947	22.451045	21.601073	20.896018	22.293501	24.953551	0.013645503	-0.172819804	0.030480492	-0.196834322
NewGene 181	--	0.448822	0.439657	0.823094	0.765643	0.55888	0.565873	0.502395	0.365897	0.540074	0.595858049	0.280689846	0.702657486	-0.196800293
ENSG00000198912	C1orf174	13.092301	13.025994	13.876064	14.732477	13.592122	13.589179	10.533535	10.29378	11.943641	0.030761026	0.21407253	0.068001259	-0.19670113

Gene ID	Gene name	Ctrl-1 FPKM	Ctrl-2 FPKM	Ctrl-3 FPKM	IFNγ-1 FPKM	IFNγ-2 FPKM	IFNγ-3 FPKM	Sora-1 FPKM	Sora-2 FPKM	Sora-3 FPKM	Ctrl vs IFNγ DESeq2 FDR	Ctrl vs IFNγ DESeq2 log2FC	Ctrl vs Sora DESeq2 FDR	Ctrl vs Sora DESeq2 log2FC
NewGene 6042	--	2.355187	2.429095	2.005852	1.868524	1.874832	1.716074	1.483838	2.042186	1.998708	0.55091875	-0.144944509	0.37867939	-0.196681874
ENSG00000103544	VPS35L	0.616834	0.689572	0.329536	0.556475	0.299684	0.519496	0.450692	0.440966	0.443262	0.79046308	-0.131493368	0.601881172	-0.196614543
ENSG00000115266	APC2	0.323215	0.28868	0.238158	0.21591	0.219553	0.229102	0.230358	0.23272	0.230434	0.500269606	-0.203166939	0.464314726	-0.196512045
ENSG00000144369	FAM171B	3.28094	2.901039	3.090821	2.153868	2.257079	2.330815	2.199567	2.663411	2.715295	0.007643253	-0.309839389	0.135731348	-0.196436172
ENSG00000065613	SLK	5.76044	5.657059	5.908692	5.171208	4.817354	5.042115	4.714086	4.420122	5.086248	0.573838454	-0.054332618	0.018091495	-0.19637168
ENSG00000160325	CACFD1	28.22459	26.23399	22.361305	19.110801	19.972235	21.862889	19.84027	20.072151	22.805123	0.050294072	-0.197097119	0.046397409	-0.196129948
ENSG00000004961	HCCS	10.078462	11.383803	12.877498	9.198415	10.277069	8.860735	11.085338	10.322925	10.185052	0.742269075	-0.047741255	0.095191	-0.195876038
ENSG00000146830	GIGYF1	11.223804	11.864196	10.877385	12.433311	10.838302	11.091729	9.121589	9.253874	9.543082	0.023335543	0.171467653	0.007650903	-0.195811428
ENSG00000088387	DOCK9	1.805038	1.992502	1.989902	4.224876	4.2115	3.963583	1.652104	2.015666	1.732429	3.72E-38	1.119527346	0.115163837	-0.195794966
ENSG00000070444	MNT	5.05058	5.157372	5.39338	5.075621	5.308624	4.773263	4.501628	3.757406	4.482675	0.330009399	0.107781676	0.065779773	-0.19574596
ENSG00000095637	SORBS1	6.076693	5.027014	5.260215	6.142585	6.165628	5.429462	4.774788	4.281547	4.449273	0.036756608	0.222703529	0.070985021	-0.19559368
ENSG00000181450	ZNF678	3.683451	3.324417	3.060278	2.674469	2.911368	2.889275	3.157614	2.702368	2.404782	0.951878966	0.009401931	0.067929309	-0.19558482
ENSG00000146063	TRIM41	18.942795	19.207666	20.124779	19.202296	19.496149	18.741086	16.08103	15.385673	16.191243	0.11730936	0.116395783	0.00829204	-0.195286394
ENSG00000214300	SPDYE3	0.557024	0.637798	0.682898	0.670913	0.46779	0.497987	0.451753	0.475722	0.608862	0.899142427	-0.056805189	0.568761302	-0.195239363
ENSG00000170260	ZNF212	6.194437	7.332076	7.27749	5.925185	5.91946	6.521602	6.365299	5.702742	5.231311	0.800616507	-0.040367375	0.153817725	-0.195070698
ENSG00000205758	CRYZL1	7.836541	7.263205	7.048351	7.218155	6.705638	7.024475	6.73481	5.937473	5.628798	0.924875099	0.018718327	0.174573826	-0.195001392
ENSG00000141179	PCTP	7.283421	8.339482	6.464356	6.687757	6.694932	7.232068	5.604646	5.853873	6.85408	0.944675771	-0.013383887	0.171253656	-0.194929793
ENSG00000123411	IKZF4	2.086346	2.25622	2.565749	2.575117	2.149553	2.671075	1.684645	1.917372	2.156465	0.347056717	0.147388552	0.210600881	-0.194876614
ENSG00000198646	NCOA6	10.904577	10.194556	10.463119	10.699722	9.726148	9.910982	8.2105	7.939956	9.36583	0.30072476	0.080168925	0.014470133	-0.19486732
ENSG00000088448	ANKRD10	40.553368	42.46924	43.288415	37.000619	34.222485	34.180808	34.680737	31.723452	34.430234	0.222543024	-0.089352589	0.005707677	-0.194855191
ENSG00000185728	YTHDF3	17.189728	18.783063	19.245425	17.381614	18.085223	20.701453	18.298149	14.194424	15.487318	0.458022227	0.063013855	0.010185155	-0.194792825
ENSG00000106392	C1GALT1	19.768309	20.149394	22.676522	14.934862	15.757788	15.439797	15.879026	16.778396	18.853053	0.0000205	-0.300897367	0.019325398	-0.19476964
ENSG00000197217	ENTPD4	5.892386	5.598394	6.358932	6.473084	5.597889	5.542643	5.086713	5.065276	5.319004	0.478157154	0.088671141	0.057246756	-0.194723544
NewGene 6051	--	3.265469	3.435073	4.633048	3.53466	4.214981	3.822371	3.495516	3.790407	1.99803	0.689221571	0.177912412	0.660344862	-0.194628198
ENSG00000170088	TMEM192	3.734595	3.681712	4.499079	3.14549	2.937652	3.177246	3.312782	3.355366	3.073817	0.013029838	-0.211388901	0.038022828	-0.194574287
ENSG00000124942	AHNAK	7.20464	6.625131	5.419287	10.856111	10.981206	10.497205	5.614396	5.225411	5.121496	6.44E-29	0.884957367	0.05720514	-0.194520749
ENSG00000255837	TAS2R20	0.8875	1.097937	1.528062	1.480008	1.108785	1.393645	0.776938	1.108762	0.967555	0.394899676	0.332742132	0.6350096	-0.194403648
ENSG00000110048	OSBP	30.839392	32.085083	32.500369	34.517841	33.71014	33.749332	26.43115	24.847969	26.844885	0.00000239	0.242536141	0.001586872	-0.194288608
ENSG00000122694	GLIPR2	4.717911	4.0191	3.966428	2.964679	2.762841	3.50065	3.151969	3.307419	3.923316	0.097753787	-0.306327818	0.278872005	-0.194193954
ENSG00000204856	FAM216A	6.082946	5.983499	5.094424	3.718956	3.593069	3.631894	4.271086	4.34004	5.143963	0.00233668	-0.491682334	0.259777392	-0.194177136
ENSG00000090975	PITPNM2	0.568183	0.658305	0.773376	1.471143	1.357863	1.286439	0.491694	0.481621	0.703003	3.33E-13	1.14456309	0.428536132	-0.194162538
NewGene 6445	--	0.820181	1.047458	1.136987	1.13143	0.768425	0.89321	0.929174	0.786623	0.84312	0.774878487	-0.143295797	0.639011775	-0.194038562
ENSG00000102384	CENPI	6.11077	6.628337	5.777075	6.544818	6.095736	5.673461	5.054768	5.678033	4.100159	0.64859238	0.073854027	0.182673522	-0.19380954
ENSG00000105879	CBLL1	10.549095	10.019333	11.075094	10.732957	10.256501	11.257649	8.628364	8.144686	8.722775	0.021147156	0.193018732	0.028967156	-0.193746047
ENSG00000163191	S100A11	37.176647	34.07769	34.438301	24.844088	22.889856	20.157986	28.531841	32.183449	25.702242	0.00000925	-0.493737189	0.146808932	-0.193739801
ENSG00000141664	ZCCHC2	5.36513	5.425866	5.503325	10.726873	9.690526	8.742035	4.676334	4.442493	4.413844	6.67E-35	1.039487576	0.062224475	-0.193736119
ENSG00000159873	CCDC117	20.103027	20.362113	22.529438	18.590339	17.10433	17.800306	17.357684	15.956871	18.480951	0.313412239	-0.082716714	0.013573176	-0.193666023
ENSG00000186918	ZNF395	6.460121	6.075844	4.847236	3.065615	4.509314	5.551245	5.120367	4.408921	5.238343	0.580700805	-0.48542172	0.10977374	-0.193560951
ENSG00000100580	TMED8	3.263185	3.510565	3.547553	3.29422	2.860879	3.018226	2.828278	2.670503	2.966696	0.869528384	-0.022834833	0.051318388	-0.19348893
ENSG00000196104	SPOCK3	0.687099	0.464377	0.469061	0.110138	0.083123	0.137966	0.290424	0.445089	0.429511	0.000176929	-1.765296843	0.657077145	-0.193446348
ENSG00000185008	ROBO2	1.462242	1.500188	1.821852	0.631128	0.862305	0.655653	1.216513	0.949523	1.459749	0.000102127	-0.669626371	0.256998452	-0.19332607
ENSG00000215845	TSTD1	5.64287	5.02422	4.390191	4.095344	3.047505	4.790585	4.140444	4.411545	3.430732	0.613067596	-0.18245591	0.520667268	-0.193315652
ENSG00000144231	POLR2D	14.993662	17.578227	17.779881	16.539339	14.762325	16.395627	14.285824	14.177952	14.43197	0.709252294	0.037185393	0.013430065	-0.193314067
ENSG00000109046	WSB1	45.287697	54.302429	58.414571	49.040381	43.456444	40.232975	44.888538	37.578054	45.914748	0.106965435	-0.154941316	0.035881619	-0.193298783
ENSG00000173852	DPY19L1	18.376153	19.17203	19.210529	14.279333	14.844517	14.521496	15.488911	14.329955	16.648936	0.000291888	-0.228486077	0.006391603	-0.19328443
NewGene 102	--	1.042945	1.411316	1.120003	1.159199	0.86263	1.114205	1.204952	0.694586	1.024471	0.915061926	0.041859828	0.528953132	-0.192889768
NewGene 4324	--	3.954603	4.230212	3.684077	4.792289	5.32107	4.003921	3.320765	2.997952	3.315223	0.05168264	0.374009737	0.34237466	-0.192846683
ENSG00000181588	MEX3D	30.593519	26.398232	25.264532	20.005788	21.434067	23.647726	21.916968	21.776706	23.951655	0.035317853	-0.208209471	0.034086788	-0.192839934
ENSG00000126878	AIF1L	12.311383	11.805158	12.156636	4.317798	4.842174	5.228127	9.792685	10.173702	10.489818	6.96E-42	-1.18002959	0.0280367	-0.19282218
ENSG00000133641	C12orf29	5.579639	6.491196	7.204299	5.440442	5.335793	5.402788	5.476738	6.729201	5.075321	0.791200698	-0.048195735	0.189379725	-0.192761173
ENSG00000166548	TK2	4.203184	4.102111	3.546717	2.76935	2.429592	2.888785	3.448489	3.372507	3.607223	0.000603989	-0.445976087	0.160496225	-0.192740279
ENSG00000143061	IGSF3	1.350966	1.142964	1.282804	2.397292	1.876561	2.222114	0.926906	1.0324	1.079107	2.19E-13	0.934559261	0.253011972	-0.192686838
ENSG00000177030	DEAF1	35.104076	31.840335	32.489635	28.036667	27.668296	30.184562	25.299563	26.163493	29.734816	0.559750802	-0.056330079	0.025549671	-0.1925752
ENSG00000106635	BCL7B	19.977176	19.705852	19.658612	15.332639	16.465131	16.703352	14.976546	15.215128	18.498384	0.575837233	-0.05989132	0.049837705	-0.192511045
ENSG00000055070	SZRD1	37.997628	36.221268	35.524697	35.072611	34.812042	34.224559	28.57542	29.227378	31.358479	0.157605694	0.092473107	0.003447645	-0.192425562
ENSG00000054654	SYNE2	3.150156	2.132155	3.344135	3.015607	1.969928	2.624569	1.910003	1.727	1.933507	0.032199202	-0.172364865	0.020794126	-0.192344812
NewGene 2802	--	0.67011	0.85839	1.098646	0.271277	0.312595	0.314118	0.614411	0.868211	0.641873	0.000100443	-1.232999735	0.538192918	-0.192269793
ENSG00000148110	MFSD14B	25.227112	26.251689	25.856248	26.558322	26.945722	27.950625	20.687175	20.479631	22.454202	0.00038476	0.220057497	0.005028166	-0.192249746
ENSG00000100154	TTC28	4.573356	4.043077	3.654422	4.344028	3.790382	4.038125	3.768992	2.895354	3.513771	0.14813075	0.145400089	0.080178063	-0.192201856
ENSG00000179454	KLHL28	1.641134	1.630457	1.677822	1.692501	1.583896	1.648467	1.212073	1.14967	1.556945	0.457838513	0.119792128	0.242962916	-0.192174285
ENSG00000066135	KDM4A	19.258701	16.69354	15.593499	15.607631	13.999601	14.284394	13.776844	13.30039	15.393562	0.421613268	-0.084187423	0.040867287	-0.191955641
ENSG00000070961	ATP2B1	34.167233	35.163723	37.053804	36.829098	39.174194	35.927108	27.640734	27.676655	32.142687	0.000510948	0.21577446	0.006294678	-0.191928027
ENSG00000092847	AGO1	3.315389	3.341066	3.55347	2.877266	2.790927	2.843126	2.822809	2.521297	2.990995	0.221591528	-0.10722745	0.029576282	-0.191797293
ENSG00000136925	TSTD2	8.53342	9.229072	9.460433	8.657093	8.687796	8.353441	7.398311	7.031999	7.672632	0.54339025	0.064838399	0.036866232	-0.191784332
ENSG00000177570	SAMD12	0.585434	0.464216	0.40582	0.354473	0.379395	0.403562	0.619363	0.730735	0.54217	0.514598446	-0.201452015	0.493931873	-0.191784018
ENSG00000115363	EVA1A	27.966812	26.613599	24.703555	28.625564	27.486397	26.461101	20.835021	21.884445	22.843659	0.00623855	0.215561083	0.032064775	-0.191740389
ENSG00000156384	SFR1	2.280999	2.739497	2.854207	2.305601	2.151446	2.338293	2.065367	2.000053	2.291482	0.886141116	-0.045700096	0.427648918	-0.191730982
ENSG00000048342	CC2D2A	3.130783	2.761267	3.5759	2.376205	2.353242	2.450186	2.879057	2.529116	3.114993	0.01868032	-0.332066972	0.186246229	-0.191701097
ENSG00000100104	SRRD	7.825287	8.61655	8.861805	7.769816	7.452047	8.157278	7.04684	6.797491	6.921488	0.780303356	0.034591109	0.046690386	-0.191455748
ENSG00000022567	SLC45A4	5.233169	4.518782	4.22843	5.923687	5.843826	6.125981	3.89291	3.553361	4.506673	0.0000111	0.396550325	0.085853949	-0.191280618
ENSG00000118518	RNF146	13.687236	15.668169	14.829171	14.028876	14.649822	14.379154	12.699517	10.918824	11.564538	0.166281516	0.132752729	0.062861534	-0.19116536
ENSG00000162729	IGSF8	8.231883	6.992883	7.774969	6.903965	7.219841	8.311065	6.71363	6.185821	5.934003	0.430390098	0.114818277	0.138846017	-0.191150118
ENSG00000090615	GOLGA3	7.648874	7.316806	7.686136	9.092681	7.907842	8.065658	6.4548	5.52752	6.75843	0.0000211	0.291161472	0.023369665	-0.191042245
ENSG00000180398	MCFD2	49.55838	47.074037	47.649112	48.838269	48.762628	47.43377	38.29002	37.1862	42.017325	0.00363634	0.160730023	0.001961357	-0.191024193

Gene ID	Gene name	Ctrl-1 FPKM	Ctrl-2 FPKM	Ctrl-3 FPKM	IFNγ-1 FPKM	IFNγ-2 FPKM	IFNγ-3 FPKM	Sora-1 FPKM	Sora-2 FPKM	Sora-3 FPKM	Ctrl vs IFNγ DESeq2 FDR	Ctrl vs IFNγ DESeq2 log2FC	Ctrl vs Sora DESeq2 FDR	Ctrl vs Sora DESeq2 log2FC
ENSG00000130844	ZNF331	5.010392	6.5456	5.395231	5.27188	3.837142	4.033764	4.77411	4.377221	4.274684	0.070744342	-0.225950738	0.113802516	-0.190898464
ENSG00000166900	STX3	65.185609	63.242637	69.939249	39.919508	37.662858	36.731765	51.649245	51.770258	57.888908	4.04E-32	-0.653918898	0.00428866	-0.190750202
ENSG00000064012	CASP8	15.532621	14.536739	14.217828	29.12857	30.06918	35.15452	15.17525	15.126042	11.168983	4.75E-52	1.068198496	0.051352097	-0.190702741
ENSG00000269713	NBPF9	6.581412	6.035602	6.135005	5.830268	5.271282	5.370441	5.337062	4.472743	5.377607	0.532573651	-0.068651082	0.071935551	-0.190604203
ENSG00000189339	SLC35E2B	25.739256	23.781687	23.649686	19.02075	18.703259	19.047569	18.504393	18.11558	22.834275	0.000334425	-0.218841978	0.023658296	-0.190488892
ENSG00000062650	WAPL	15.346883	15.900818	16.455181	18.055578	17.60977	17.171324	13.054476	12.781644	13.313397	0.000000229	0.296366557	0.0051118	-0.1904693
ENSG00000111450	STX2	5.32609	6.551515	6.556301	4.591911	3.900157	4.209582	5.315894	4.370731	5.451397	0.002215449	-0.382246778	0.162168098	-0.190460801
ENSG00000140443	IGF1R	3.514304	3.371827	3.682608	2.746129	2.412899	2.470537	2.700101	2.775176	3.288297	0.0000488	-0.336763614	0.047163279	-0.190163774
ENSG00000101752	MIB1	9.953177	11.73888	11.44949	9.373653	8.607843	8.438093	9.025867	8.830742	9.38168	0.024291688	-0.179839142	0.01809152	-0.190130193
ENSG00000177839	PCDHB9	0.661118	0.481723	0.548691	0.358431	0.403325	0.398224	0.391841	0.474478	0.520834	0.226455771	-0.390521396	0.545995308	-0.190122387
NewGene_5015	--	0.430008	0.594111	0.449136	0.419064	0.257868	0.244897	0.339914	0.476402	0.288268	--	--	0.709496735	-0.190085681
ENSG00000168036	CTNNB1	89.200584	106.303573	83.316318	83.623032	92.742255	91.310732	76.389243	75.401174	74.577804	0.853917749	0.014915969	0.00263646	-0.189821809
ENSG00000116120	FARSB	20.814747	22.065536	24.705647	19.815781	18.519646	19.003338	19.070347	18.197849	18.270674	0.322832967	-0.090109436	0.030257351	-0.189670478
ENSG00000213380	COG8	15.210424	14.161295	15.348393	13.180746	14.826958	13.150085	11.970498	11.72942	12.207348	0.705902056	0.038707042	0.023169927	-0.18965966
ENSG00000133398	MED10	26.453217	25.999126	30.348768	25.977207	26.935818	27.221201	22.53636	22.535597	22.980213	0.364181341	0.100886396	0.065552215	-0.189339348
ENSG00000033170	FUT8	0.415378	0.472253	0.48484	0.51684	0.638522	0.722662	0.367737	0.413507	0.357216	0.297082759	0.307660526	0.541940183	-0.189331083
ENSG00000119969	HELLS	14.960083	18.007409	21.230307	15.245073	13.414969	13.651395	15.617068	14.335946	16.881023	0.367834695	-0.202741588	0.13167177	-0.189302626
ENSG00000128591	FLNC	1.171649	1.162575	1.188845	0.710854	0.681737	0.783196	0.946978	0.930501	1.020198	0.0000924	-0.544771592	0.183278183	-0.189230594
ENSG00000141562	NARF	33.128822	25.922764	30.836744	27.856551	25.408401	21.704301	30.94495	21.271806	28.698261	0.02498628	0.204678534	0.040346365	-0.189143573
ENSG00000082146	STRADB	19.268127	17.91891	17.142482	15.901913	15.25061	14.794802	15.656785	14.299489	15.068551	0.329708831	-0.095649278	0.038845822	-0.189027033
ENSG00000255561	FDXACB1	1.43059	1.30665	0.993938	1.145355	1.236508	1.035854	1.073317	1.041313	0.876163	0.94898571	0.024576932	0.507291269	-0.189025171
ENSG00000204390	HSPA1L	0.813152	0.939894	0.896711	0.971438	0.925475	0.549789	0.779025	0.686706	0.719362	0.956046964	0.026350372	0.548847442	-0.189023928
ENSG00000133874	RNF122	5.554216	5.271895	4.38046	3.475982	2.973036	3.462209	3.844272	4.401841	4.254439	0.003974723	-0.468577207	0.267959145	-0.188897205
ENSG00000160783	PMF1	93.886638	83.351428	95.373053	68.016594	72.607975	76.432022	76.013334	72.496444	82.58393	0.001662144	-0.207640896	0.007515203	-0.188895477
ENSG00000136045	PWP1	32.303593	29.590554	33.166544	25.312185	25.544007	24.444202	24.879231	26.468665	27.030339	0.009916495	-0.187684432	0.018412527	-0.188856408
ENSG00000132003	ZSWIM4	4.784267	3.617906	4.134463	2.302599	2.561603	2.399935	3.508561	3.26789	3.564187	0.00000148	-0.647716426	0.172957853	-0.188758527
ENSG00000011021	CLCN6	6.89711	7.842024	7.797287	9.389813	9.117539	8.486465	6.319716	5.608533	6.675815	0.000000297	0.407242041	0.048222044	-0.188697799
ENSG00000107317	PTGDS	15.755505	11.643757	6.844244	9.188632	8.745329	9.593943	10.313789	9.137079	8.824161	0.74672924	-0.171234771	0.6769154	-0.188590816
ENSG00000165240	ATP7A	1.967051	1.624704	1.775166	1.429741	1.436749	1.237997	1.45108	1.475933	1.47319	0.011064128	-0.321294005	0.148544826	-0.188544704
ENSG00000048392	RRM2B	4.765304	4.886447	4.975699	4.585644	4.493634	4.618186	4.057574	3.599166	4.483614	0.711483799	0.046262072	0.085296715	-0.18845173
ENSG00000148153	INIP	7.102695	7.513134	7.544502	6.573251	6.249147	5.942782	5.884977	6.286396	6.268911	0.331634583	-0.100024029	0.047722216	-0.188297152
ENSG00000149792	MRPL49	37.048653	34.885833	33.610103	33.686452	33.61789	34.644803	27.102499	29.008451	31.080498	0.219763617	0.096096945	0.022779888	-0.188182285
ENSG00000179562	GCC1	6.477034	6.997105	6.540655	6.973095	6.720545	7.197912	5.865315	4.801658	5.828968	0.02174997	0.209860062	0.078413802	-0.188152332
ENSG00000237765	FAM200B	6.94211	7.767876	8.238907	6.236094	6.524509	6.265814	6.230701	5.657365	7.058288	0.100953863	-0.190355119	0.112417285	-0.188123948
ENSG00000106344	RBM28	4.229531	4.336189	4.678071	3.784568	3.526667	3.710123	3.604814	3.600425	3.952663	0.130419488	-0.121368323	0.018697731	-0.188118
ENSG00000125629	INSIG2	9.893835	10.052342	9.863828	6.628047	7.465131	6.823021	8.440043	7.442991	9.618099	0.000253026	-0.360048689	0.079986559	-0.187625004
ENSG00000154265	ABCA5	6.979424	8.335745	7.644261	5.739229	5.287639	5.198848	6.879056	5.860463	7.011051	0.091340168	-0.199112651	0.11109921	-0.187608086
NewGene_6375	--	1.100494	0.875908	0.771377	0.780241	0.652311	0.696777	0.909712	0.620749	0.867561	0.712730242	-0.18750637	0.669720903	-0.18758746
ENSG00000135317	SNX14	37.602968	39.216007	36.739129	33.199941	34.49388	33.122696	31.790206	30.143464	31.719098	0.959520262	0.00523964	0.003993833	-0.187490517
ENSG00000151208	DLG5	20.918037	18.390141	19.003936	12.962136	13.220267	12.764608	15.265426	14.358494	18.554775	1.85E-11	-0.433419107	0.038513508	-0.187474373
ENSG00000067167	TRAM1	82.712724	89.021722	91.703124	106.003956	104.010099	101.901621	72.8879	70.787659	76.361598	6.65E-14	0.370433932	0.00189332	-0.18746538
ENSG00000198920	KIAA0753	6.67633	6.00304	5.909738	4.08526	4.123104	4.188005	5.027761	4.969166	5.545071	0.0000949	-0.410817628	0.09675591	-0.187315872
ENSG00000130177	CDC16	41.898376	38.651802	40.428984	38.725842	36.842982	34.557857	32.783715	31.621497	35.14643	0.97480927	0.003650965	0.009127244	-0.187277965
ENSG00000111261	MANSC1	9.357623	7.461688	6.949222	7.957824	9.113927	7.39904	6.544541	7.325147	7.470243	0.426049807	0.0906592	0.05212318	-0.187224484
ENSG00000166046	TCP11L2	1.154063	1.308679	1.011892	1.103736	1.135905	0.947354	1.085973	1.014225	1.009078	0.879121793	-0.066246421	0.55035255	-0.187193252
ENSG00000104067	TJP1	14.229505	12.440314	13.027687	13.260443	11.384459	11.181852	10.827716	10.052759	10.847542	0.426394468	0.068615695	0.007564562	-0.186989616
ENSG00000163430	FSTL1	0.4082	0.475215	0.454056	0.143418	0.209848	0.157737	0.378981	0.268147	0.452957	0.0000657	-1.220357319	0.548139423	-0.186988941
NewGene_3141	--	0.833305	0.908604	0.877169	0.719491	0.623904	0.670637	0.781974	0.542372	0.840127	0.268262912	-0.229748851	0.392561923	-0.186982623
ENSG00000120688	WBP4	5.953794	5.900729	5.940135	5.852676	5.973845	5.913178	4.901952	4.938984	4.802886	0.245522456	0.142942582	0.129145217	-0.186748807
ENSG00000173692	PSMD1	64.520944	70.988089	63.726869	55.648099	55.812717	55.790887	51.423909	52.395686	54.047672	0.563478673	-0.041066457	0.004258604	-0.186557317
ENSG00000180448	ARHGAP45	14.53545	14.306612	12.675509	10.499609	10.315625	10.467497	11.679773	10.237892	13.030941	0.000491978	-0.26898068	0.047473014	-0.186414096
ENSG00000021776	AQR	8.655196	9.368708	8.780392	8.062368	8.013702	7.721996	7.423667	7.406981	8.130313	0.366090395	-0.069557048	0.008350877	-0.18640689
ENSG00000149591	TAGLN	2.008447	2.820198	3.155759	0.761619	0.489868	0.631724	2.291358	2.206296	2.046943	0.000000778	-1.504127458	0.530066127	-0.186331127
ENSG00000166716	ZNF592	8.341391	7.397929	7.387116	7.898465	8.547986	7.641733	6.633851	5.721547	6.73903	0.06648885	0.147740423	0.036370922	-0.186319275
ENSG00000119707	RBM25	24.903654	27.051202	28.894612	31.601438	27.117014	27.859166	23.307876	21.285437	23.903197	0.003653536	0.203343867	0.013930803	-0.186163367
ENSG00000175130	MARCKSL1	164.166428	157.663254	151.498627	121.409218	123.973595	129.701538	123.719208	131.376389	134.990311	0.001565846	-0.1879635	0.005213616	-0.186113059
ENSG00000119912	IDE	12.045239	12.713168	12.657225	12.795514	12.136678	12.402042	10.747702	9.963376	10.291694	0.03189279	0.144320818	0.012499807	-0.185897631
NewGene_6728	--	1.647256	1.762843	2.050698	1.735374	1.789911	1.805536	1.312643	1.485439	1.522808	0.528668375	0.165825901	0.45723117	-0.185868916
ENSG00000159921	GNE	8.497769	8.272453	7.887949	7.504644	6.962811	7.964787	6.626331	6.065365	6.719384	0.555818764	0.062394058	0.031516117	-0.185755807
ENSG00000118898	PPL	5.524704	4.487851	4.525295	2.533911	2.151076	2.149312	4.171943	3.668432	4.16184	3.41E-18	-0.943868034	0.107003218	-0.185728195
ENSG00000154978	VOPP1	34.793978	34.066719	36.426856	27.633709	28.976049	29.741554	27.472174	27.432488	32.121434	0.049942983	-0.138559899	0.014677862	-0.185506776
ENSG00000173744	AGFG1	17.501404	17.939454	19.960249	16.280585	16.321457	15.747598	16.23638	14.821997	16.342361	0.229263206	-0.088431949	0.009844846	-0.185425307
ENSG00000244187	TMEM141	71.230148	64.935257	50.117092	45.014614	47.346836	48.638515	51.504555	54.203018	47.620201	0.036034401	-0.254170374	0.159764875	-0.185343602
ENSG00000111452	ADGRD1	1.390939	1.255731	1.271594	0.902886	0.757225	0.666064	1.335387	0.890986	0.874106	0.031042545	-0.471872249	0.43618677	-0.185305063
ENSG00000100376	FAM118A	8.620671	7.953621	8.321318	9.864398	8.772031	8.787297	6.808064	6.41253	7.570094	0.008196053	0.243438574	0.06591421	-0.185231845
ENSG00000164983	TMEM65	0.866712	0.998306	1.089123	0.95572	0.887495	0.98752	0.740461	0.770982	0.924641	0.664685895	0.086005598	0.287970706	-0.185043195
ENSG00000197037	ZSCAN25	8.630323	9.42882	8.336528	8.368912	8.26984	8.841801	7.457594	6.492817	7.083157	0.045297568	0.168984163	0.046244211	-0.184928212
ENSG00000101811	CSTF2	9.024859	10.41108	10.118976	7.785969	8.242853	7.702099	7.556191	8.193698	8.588592	0.159652864	-0.172826325	0.124785296	-0.184759938
ENSG00000105767	CADM4	9.789949	9.266976	8.360486	6.763953	7.563046	7.207308	7.223537	7.123011	8.289994	0.105814996	-0.20009167	0.123125288	-0.18465867
ENSG00000213463	SYNJ2BP	4.266562	4.948164	4.913039	5.79267	5.829189	5.605477	3.833287	3.620373	4.211466	0.000000159	0.433733468	0.069524976	-0.184624839
ENSG00000127081	ZNF484	1.608399	1.888967	1.63369	1.939876	1.774691	1.59552	1.195238	1.251002	1.696669	0.474699941	0.155709725	0.373824361	-0.184578948
ENSG00000251322	SHANK3	3.628799	3.429942	3.024396	6.517741	5.556776	6.700144	3.116426	2.359148	2.85741	6.18E-30	1.043104563	0.151886009	-0.184574606
ENSG00000162496	DHRS3	21.710649	19.24502	20.104517	21.363335	21.144941	20.696958	16.32044	16.842086	17.765041	0.027545212	0.194330859	0.051892532	-0.184492203
ENSG00000133056	PIK3C2B	14.26614	11.938287	10.447818	13.932154	12.732852	12.906201	9.2784	8.804938	11.141958	0.000140665	0.310072173	0.077667083	-0.18446066

Gene ID	Gene name	Ctrl-1 FPKM	Ctrl-2 FPKM	Ctrl-3 FPKM	IFNγ-1 FPKM	IFNγ-2 FPKM	IFNγ-3 FPKM	Sora-1 FPKM	Sora-2 FPKM	Sora-3 FPKM	Ctrl vs IFNγ DESeq2 FDR	Ctrl vs IFNγ DESeq2 log2FC	Ctrl vs Sora DESeq2 FDR	Ctrl vs Sora DESeq2 log2FC
ENSG00000172057	ORMDL3	68.179868	60.673985	52.384768	88.418582	86.089843	89.016227	49.127613	52.110231	48.11798	7.76E-19	0.685390906	0.068824916	-0.184410448
ENSG00000163719	MTMR14	30.466217	28.384122	26.997772	24.792075	23.590168	25.654677	21.927757	23.600788	25.429376	0.54037332	-0.061916552	0.041978018	-0.184384625
ENSG00000130054	FAM155B	1.528339	1.446637	1.012705	0.98191	0.849948	0.752512	1.058036	0.980573	1.26414	0.041270746	-0.480888761	0.43618677	-0.184138891
ENSG00000243716	NPIPB5	36.123457	46.789068	51.73773	45.685484	38.999033	39.654226	37.603522	36.585073	35.967394	0.742322054	-0.044998361	0.08812846	-0.184061159
ENSG00000185591	SP1	11.923495	12.385629	11.812532	12.644647	12.545123	12.588355	10.959651	10.07349	10.37814	0.000148841	0.223389107	0.009712061	-0.184033205
ENSG00000099250	NRP1	23.937479	23.669187	24.994735	28.301667	26.977758	26.761897	20.216182	19.555458	21.572232	5.8E-09	0.318340249	0.006843471	-0.183945176
ENSG00000082153	BZW1	71.904169	79.692874	78.164357	57.576811	57.61894	55.89227	62.853945	58.725174	62.094093	0.00000159	-0.25179615	0.004326239	-0.183840507
ENSG00000151694	ADAM17	10.255054	10.818903	10.719658	9.948991	8.44564	8.510277	8.52672	8.175234	9.703507	0.075318754	-0.146830391	0.029576282	-0.183761661
ENSG00000188033	ZNF490	0.47	0.565017	0.445115	0.586531	0.582896	0.501163	0.758488	0.545058	0.933643	0.024877595	0.790480255	0.710407765	-0.183749496
ENSG00000008324	SS18L2	12.600271	14.298184	13.651649	13.043548	13.816891	10.352339	10.215357	10.696683	10.530429	0.683353196	0.074698188	0.269807092	-0.1837248
ENSG00000116580	GON4L	15.375634	14.236572	14.071771	12.935383	12.145288	11.752078	12.59483	12.576831	13.814417	0.023474435	-0.155562239	0.009310021	-0.183619838
ENSG00000160214	RRP1	20.571966	20.366628	21.05632	17.200151	16.854538	18.555379	16.322621	16.450613	18.462135	0.264885946	-0.092097852	0.01743312	-0.183508177
ENSG00000104497	SNX16	4.137864	4.811269	4.783458	4.552233	4.071137	3.690395	3.714412	3.770534	5.385209	0.788116342	-0.049057915	0.238649429	-0.183440117
ENSG00000152270	PDE3B	15.788132	15.487179	14.798625	12.107794	11.924804	11.779888	11.97216	12.040008	14.090508	0.000445057	-0.219713285	0.017735936	-0.183426567
ENSG00000283765	AC131160.1	0.895261	0	3.163204	1.809181	3.348046	3.446595	3.351026	0	0	0.657588095	1.2344479	0.972805713	-0.183392878
ENSG00000128585	MKLN1	6.080815	7.290679	6.744898	6.400133	6.191278	5.936018	5.813443	5.30109	5.147639	0.440467452	0.072876381	0.028556199	-0.183376372
ENSG00000183628	DGCR6	4.018605	3.52084	3.606478	2.492118	2.947327	3.140641	3.319917	3.258192	2.849336	0.216125989	-0.25515402	0.355425464	-0.183365754
ENSG00000118655	DCLRE1B	2.830759	2.764556	3.347005	2.477131	2.571839	2.518359	2.940643	2.199502	2.257521	0.403541368	-0.138809826	0.292097309	-0.18335359
ENSG00000185721	DRG1	43.119314	41.901936	38.65207	39.050144	34.714842	36.767626	36.721004	33.614478	32.145933	0.885757996	-0.015893006	0.04251741	-0.18332584
ENSG00000075223	SEMA3C	3.931297	4.365864	5.396963	1.75307	1.834251	1.50973	3.647937	3.391973	4.625908	6.1E-24	-1.304347136	0.222112912	-0.183193821
ENSG00000064607	SUGP2	20.368166	20.723865	20.893861	20.300282	18.31441	17.428942	16.171054	16.051861	18.473162	0.835667407	0.019444486	0.011393403	-0.183186881
ENSG00000156521	TYSND1	3.400333	3.038703	2.419026	2.423021	2.833624	2.716806	2.874666	2.364704	2.079922	0.987266248	-0.00390225	0.342364834	-0.183182417
ENSG00000146282	RARS2	35.401128	33.386627	33.8769	27.15605	25.714783	24.793705	27.787838	27.565847	29.505271	0.00030653	-0.257102179	0.015471292	-0.183126396
ENSG00000135070	ISCA1	14.895787	15.291371	16.114128	12.504808	12.350408	12.725241	12.495109	11.766177	14.133931	0.107188863	-0.156690727	0.073275212	-0.182906495
ENSG00000114738	MAPKAPK3	14.258746	14.033042	12.175176	12.585912	11.627513	11.351128	11.094191	10.66243	11.931973	0.736301226	-0.043015972	0.067184242	-0.182851724
ENSG00000137269	LRRC1	15.521937	14.421973	15.177444	9.890918	9.970491	9.334786	12.741269	12.03352	13.439636	4.94E-10	-0.474514649	0.028879623	-0.182814178
ENSG00000002919	SNX11	7.727995	7.705398	7.437915	6.374609	6.622935	6.156629	6.837112	6.035617	6.593164	0.232726019	-0.144991983	0.114263456	-0.182571313
ENSG00000005100	DHX33	12.342856	13.327783	13.900367	14.065377	12.70574	13.289757	10.84174	10.153138	12.093811	0.036766341	0.161143023	0.02499948	-0.18238879
NewGene_570	--	9.196789	11.732235	12.114324	10.423702	8.652057	9.246459	9.389158	7.537538	10.308829	0.653812172	-0.065774868	0.147182545	-0.182358688
ENSG00000152520	PAN3	8.382887	9.490483	10.182879	8.602071	7.620705	8.51347	7.514065	6.939361	8.510844	0.797804134	-0.033326759	0.080443206	-0.182324885
ENSG00000158156	XKR8	5.439267	4.944023	4.955412	3.800939	4.08936	4.496695	4.498924	3.97682	4.199075	0.284751433	-0.159110542	0.173503099	-0.182264405
ENSG00000119574	ZBTB45	6.001317	5.044459	4.900312	5.235474	4.478441	5.373636	4.060202	4.192649	4.239797	0.586145378	0.095137804	0.222794906	-0.182136351
ENSG00000134308	YWHAQ	160.439664	166.069047	167.452444	145.386961	138.44029	141.175499	136.942422	133.551488	137.906329	0.208620939	-0.069696699	0.001405015	-0.182122683
ENSG00000137274	BPHL	13.440428	11.715884	11.437644	9.299656	8.890046	10.001765	10.330734	10.109768	9.681006	0.060142503	-0.225206444	0.137006677	-0.182072124
ENSG00000149084	HSD17B12	85.00142	91.573611	96.089253	109.110312	107.032713	108.76165	72.938117	72.961964	80.623118	2.57E-13	0.39947572	0.008294389	-0.181970146
ENSG00000179119	SPTY2D1	9.292079	9.571118	9.823244	11.004016	10.965946	10.57927	7.908509	7.434629	8.389892	0.00000042	0.329789245	0.020103349	-0.181856721
ENSG00000126870	WDR60	1.359941	1.336952	1.216089	0.885785	0.968605	1.223154	1.051755	0.939124	1.289237	0.401615438	-0.207761755	0.413123545	-0.181832114
ENSG00000148834	GSTO1	118.202303	134.463349	136.597638	115.06096	114.693082	112.583098	104.719143	104.830508	111.149217	0.742611891	-0.03189839	0.020673527	-0.181826983
ENSG00000149532	CPSF7	58.188095	56.714037	62.701249	57.869524	61.616704	57.953669	48.814912	47.303209	52.972031	0.344410299	0.065792724	0.008598059	-0.181811079
ENSG00000064995	TAF11	44.184104	46.920256	49.829494	42.561659	38.856991	41.042072	37.807349	38.567302	42.179375	0.576972113	-0.050871789	0.022915921	-0.181522874
ENSG00000074590	NUAK1	6.456592	5.590021	5.343079	3.67413	3.417433	3.613338	5.639919	4.261648	4.450287	1.08E-09	-0.549847946	0.148378478	-0.181500293
ENSG00000213024	NUP62	37.437804	33.773919	34.305206	38.052579	34.950944	36.142467	29.05169	26.955784	31.101165	0.003706179	0.180242916	0.011151777	-0.181451343
ENSG00000167196	FBXO22	8.312756	9.641151	10.153333	8.129876	8.745995	7.589668	7.904585	7.579784	6.89895	0.165498173	0.115142985	0.023740193	-0.181275517
NewGene_5104	--	83.943418	86.02321	95.11101	81.470671	80.25766	77.229416	71.580873	71.764391	67.596682	0.946993724	0.00616114	0.013243432	-0.18121238
NewGene_3712	--	1.306485	1.400052	1.16427	1.19127	1.06615	1.175029	1.116919	0.965586	1.115808	0.943839078	-0.026881011	0.517971074	-0.18112807
ENSG00000198551	ZNF627	9.419289	9.337579	8.986312	6.237443	6.842652	6.029898	8.241845	7.326886	7.556141	0.0000861	-0.390390913	0.100466592	-0.18110566
ENSG00000119537	KDSR	6.024427	5.959411	5.117084	8.018743	7.731887	7.738235	4.309408	4.107208	3.833839	7.97E-25	0.760038154	0.090712954	-0.181058142
ENSG00000145555	MYO10	8.761184	5.741286	8.303452	1.893754	1.680853	1.895241	7.402992	4.291746	5.449782	2.68E-91	-1.556572429	0.058510073	-0.181029615
ENSG00000166035	LIPC	19.652264	18.455403	14.608914	14.023581	15.481572	14.781192	12.129414	13.881932	13.48722	0.333976493	-0.122348924	0.105718021	-0.180962056
ENSG00000229809	ZNF688	1.838381	2.153098	1.861878	1.078738	1.155064	1.917634	1.87444	1.49446	1.19706	0.589788211	-0.179294513	0.481800972	-0.180860486
ENSG00000205744	DENND1C	13.262676	11.646825	10.601509	8.628714	9.268129	9.099188	9.758562	9.516659	9.850796	0.058700218	-0.216437062	0.104082345	-0.180737025
ENSG00000100077	GRK3	0.596337	0.607394	0.691499	0.775172	0.767612	0.684081	0.538633	0.509697	0.608623	0.044540541	0.361118284	0.366694192	-0.18058506
ENSG00000150776	NKAPD1	8.359891	9.153126	8.251007	7.034472	7.175916	7.575679	7.163728	6.796917	7.170266	0.611489962	-0.060333811	0.061235844	-0.180402779
NewGene_3438	--	0.693816	0.694894	0.479526	0.588207	0.787541	0.461971	0.424617	0.541119	0.576398	0.209579323	0.373236249	0.559815707	-0.180371069
ENSG00000120963	ZNF706	13.392427	14.615623	13.880792	12.475444	10.188714	11.816925	13.339914	12.879362	11.642273	0.151149766	-0.138102927	0.081642469	-0.180327235
ENSG00000174358	SLC6A19	4.944577	4.734836	4.237468	4.014007	4.069786	3.904866	3.547271	3.806702	4.195668	0.567099278	-0.073557084	0.114256689	-0.179995578
ENSG00000010072	SPRTN	7.91012	9.092067	9.25407	8.551896	8.697439	8.265887	7.140354	6.663462	8.133486	0.321397965	0.108854916	0.094886616	-0.179987963
ENSG00000162998	FRZB	0.568115	0.495801	0.456775	0.077552	0.127208	0.145265	0.45132	0.260898	0.549572	0.00000728	-1.911318697	0.66546209	-0.17979248
ENSG00000242485	MRPL20	101.254798	98.537859	101.023064	93.08319	97.186394	99.787998	83.044091	90.973403	78.132559	0.20373516	0.097431828	0.055461252	-0.179780758
ENSG00000077616	NAALAD2	4.464783	5.726698	6.145617	3.887909	3.876884	3.656421	4.781027	3.458856	5.094584	0.005103831	-0.366535827	0.26795633	-0.179706074
ENSG00000157538	VPS26C	27.036666	25.300554	27.090238	26.614482	27.509173	27.066437	20.986862	21.712394	23.967694	0.007761222	0.178754361	0.017380266	-0.179700652
ENSG00000144278	GALNT13	4.536232	3.984006	4.463074	4.277928	3.44338	3.765028	3.759974	3.492429	3.733508	0.741151585	-0.050081858	0.123823531	-0.179593357
ENSG00000136536	7-Mar	20.195364	21.744475	22.449792	19.433782	18.936759	19.087738	21.662489	19.012229	18.162788	0.287722565	0.069355951	0.004759656	-0.179547771
ENSG00000150471	ADGRL3	0.78206	0.817225	0.613666	0.168404	0.223013	0.161639	0.634219	0.555866	0.546143	1.79E-22	-1.744380369	0.304254512	-0.179403731
ENSG00000049618	ARID1B	8.124922	7.148739	8.120369	7.61333	7.045124	7.630773	6.678293	6.212998	8.085169	0.730197173	0.033747552	0.038028454	-0.179239874
ENSG00000073150	PANX2	0.645281	0.755558	0.739785	0.91447	0.902932	0.860571	0.482254	0.601455	0.786031	0.193548162	0.377900299	0.594203992	-0.179226845
ENSG00000101350	KIF3B	11.999551	11.056024	11.689934	9.89585	9.513	9.72088	9.718122	9.151014	9.925168	0.159598321	-0.107353305	0.017255754	-0.179175903
ENSG00000196136	SERPINA3	7.733855	10.465662	8.329039	2.789491	2.889122	4.51143	6.875308	7.516086	7.453889	9.63E-12	-1.194864965	0.241333079	-0.179138963
ENSG00000166532	RIMKLB	2.365184	3.148482	2.950097	4.402008	3.210039	3.60976	2.926834	2.198233	2.705176	0.000425701	0.531575324	0.340556411	-0.178865206
ENSG00000108854	SMURF2	9.157628	7.524999	8.393768	7.341183	5.194445	6.247003	7.918726	9.719987	7.450205	0.260428601	-0.117348423	0.066093786	-0.178664488
ENSG00000115355	CCDC88A	7.23622	7.741279	7.766753	7.543813	5.983005	6.548436	6.272423	6.90925	6.395734	0.137018332	-0.143927585	0.059687508	-0.178540031
ENSG00000157800	SLC37A3	14.572695	14.463901	15.265175	12.513288	12.550783	12.626391	11.362758	11.725048	13.605785	0.382582091	-0.078122398	0.045157784	-0.178533604
ENSG00000110080	ST3GAL4	3.505799	3.67934	3.646922	2.188749	2.336077	2.439631	2.993636	2.863771	3.18936	0.005302052	-0.49675284	0.325325025	-0.178525964
ENSG00000145723	GIN1	1.529968	1.388471	1.296754	1.095747	1.396252	1.180475	1.143013	1.131718	0.989605	0.828573567	0.064110468	0.446224648	-0.17825573

Gene ID	Gene name	Ctrl-1 FPKM	Ctrl-2 FPKM	Ctrl-3 FPKM	IFNγ-1 FPKM	IFNγ-2 FPKM	IFNγ-3 FPKM	Sora-1 FPKM	Sora-2 FPKM	Sora-3 FPKM	Ctrl vs IFNγ DESeq2 FDR	Ctrl vs IFNγ DESeq2 log2FC	Ctrl vs Sora DESeq2 FDR	Ctrl vs Sora DESeq2 log2FC
ENSG00000160714	UBE2Q1	41.644447	39.130993	41.944168	35.215355	35.823372	37.279514	31.819197	33.533222	36.425697	0.702227752	-0.032434807	0.014237121	-0.17802007
ENSG00000176209	SMIM19	17.119526	19.893864	19.751276	23.405117	20.404699	19.152701	15.655326	14.164078	15.0012	0.004511149	0.296152272	0.144870201	-0.177720259
ENSG00000197818	SLC9A8	3.210348	3.256198	3.39779	3.651613	3.361523	3.53153	2.872961	2.390899	2.919467	0.014542268	0.242662576	0.132012551	-0.177703723
ENSG00000186310	NAP1L3	2.144	1.746498	1.424888	1.61521	1.867756	1.798466	1.452668	1.410292	1.547134	0.618426172	0.136874862	0.461023442	-0.177466193
ENSG00000155792	DEPTOR	1.872354	0.858065	0.593808	0.492267	0.931866	0.661827	1.070694	0.950867	0.776951	0.257878549	-0.516985981	0.681483929	-0.177407655
ENSG00000100628	ASB2	0.443835	0.415327	0.441018	0.138876	0.060949	0.096721	0.286756	0.39544	0.392761	0.000122158	-1.891849329	0.687041256	-0.177293812
ENSG00000275023	MLLT6	15.427669	14.373392	14.815555	14.780877	12.96425	13.994199	11.960884	11.588114	14.056675	0.633448054	0.0418844	0.024307559	-0.177268809
ENSG00000177082	WDR73	6.080362	6.183507	6.18684	5.912416	4.929649	5.095757	5.507183	4.933596	6.52655	0.761578197	-0.039280274	0.077999562	-0.177265511
ENSG00000130695	CEP85	5.444039	5.376527	5.238802	4.950214	3.714654	3.72382	4.525465	4.246087	4.585444	0.003932303	-0.308959104	0.098915864	-0.177241998
ENSG00000130340	SNX9	9.100957	8.928749	8.950885	8.259363	7.602038	8.056993	7.630768	6.875959	7.903049	0.810237562	-0.026788475	0.044265847	-0.177164985
ENSG00000100034	PPM1F	7.540161	7.317203	6.804959	5.655736	5.567129	5.541278	5.806419	5.431135	6.92018	0.009975478	-0.221637516	0.084359543	-0.177135956
ENSG00000175193	PARL	36.193345	41.120433	32.930415	33.887909	30.555227	30.513108	28.739115	30.782233	30.364539	0.758200883	-0.041534634	0.094922836	-0.177012215
ENSG00000181264	TMEM136	0.86849	0.760561	0.68475	0.362578	0.517059	0.621162	0.837829	0.576346	0.464707	0.126748223	-0.471403881	0.573987032	-0.176857556
ENSG00000105738	SIPA1L3	6.843754	6.236231	5.901891	4.93305	4.969205	4.84356	4.857643	4.913728	6.103236	0.010686128	-0.220786491	0.086495329	-0.17677285
NewGene_5532	--	8.016852	8.081028	7.62095	6.757208	6.331031	6.825634	7.006672	6.707261	5.796785	0.361642654	-0.148290427	0.273561447	-0.176740159
ENSG00000219607	PPP1R3G	1.378422	0.721782	0.625605	0.532715	0.621258	0.536016	0.871936	0.672998	0.724448	0.084891795	-0.545210568	0.585476605	-0.1767145
ENSG00000139133	ALG10	2.879344	2.799354	3.875783	3.020303	2.384061	3.059011	2.48752	2.340562	2.817461	0.852810998	0.04161648	0.284428447	-0.176663732
ENSG00000163811	WDR43	24.000343	26.409257	29.192166	26.073638	25.146422	25.731099	22.168925	21.163412	22.843677	0.249147546	0.100764711	0.038022828	-0.176659293
ENSG00000164484	TMEM200A	0.235186	0.406473	0.47489	0.832141	0.793375	0.884551	0.309562	0.263736	0.234171	0.00000168	1.385154291	0.702277669	-0.17662879
ENSG00000108352	RAPGEFL1	3.718064	3.820478	3.869216	4.01199	4.476212	3.411031	2.857856	3.37081	3.126122	0.091611009	0.228886657	0.183045451	-0.176598558
ENSG00000174132	FAM174A	3.125299	2.792532	2.693123	3.168546	3.145482	2.947287	2.406516	2.021946	2.738517	0.277192654	0.251784765	0.473070015	-0.176508336
NewGene_3447	--	1.585724	1.161656	1.663743	1.391347	1.295745	1.133534	1.494969	0.902051	1.447797	0.856107824	-0.053590606	0.460584256	-0.176475361
ENSG00000125388	GRK4	1.488662	1.468568	1.398239	1.925718	1.412565	1.428794	1.056889	1.322191	1.231597	0.312904173	0.272238444	0.517563156	-0.176414046
ENSG00000197128	ZNF772	2.533671	2.939008	2.191991	2.321132	2.553827	2.4324	1.931316	1.755234	1.574778	0.526414878	0.113319815	0.290502508	-0.176400562
ENSG00000010165	EEF1AKNMT	27.37232	26.0583	24.697802	22.647474	20.235253	22.019159	20.701068	20.312808	23.375746	0.174481336	-0.111304554	0.026690158	-0.17637647
ENSG00000068001	HYAL2	23.967212	24.240654	20.333954	11.599427	11.997604	14.613803	22.558345	23.118798	20.751044	2.37E-18	-0.592596093	0.056913017	-0.176258086
ENSG00000177889	UBE2N	30.692725	32.113037	34.175266	25.985553	30.270743	26.912875	27.758908	30.214667	30.159935	0.0756163	-0.122586805	0.017826954	-0.176205977
ENSG00000078967	UBE2D4	5.499215	4.912419	5.170532	4.548195	4.017827	4.559856	5.14486	4.035876	5.257328	0.598362659	-0.082983195	0.156852144	-0.176138403
NewGene_5999	--	3.364159	3.595737	3.591603	3.323498	2.577271	2.522948	2.993697	2.836194	3.044468	0.172169992	-0.184048101	0.14147254	-0.175918197
ENSG00000156256	USP16	21.173356	22.477294	22.932993	21.961217	21.043996	20.842103	18.954295	17.151112	19.178594	0.270717585	0.087210362	0.024472949	-0.17591
ENSG00000135747	ZNF670-ZNF69	0.474344	0.33767	0.601943	0.358604	0.205613	0.260577	0.523676	0.300581	0.360827	--	--	0.711960608	-0.175908675
ENSG00000110321	EIF4G2	385.798151	410.768824	404.414626	353.47921	295.655401	316.391949	306.260092	274.042866	321.383196	0.024950572	-0.108831689	0.000963576	-0.175888183
ENSG00000121903	ZSCAN20	0.909971	0.83812	0.872249	0.619623	0.739004	0.749986	0.785972	0.550667	0.909119	0.501534029	-0.190197797	0.518454934	-0.175859736
ENSG00000258947	TUBB3	5.58454	5.596599	5.42129	4.049468	3.784505	4.02723	4.651569	4.372701	4.730348	0.020633205	-0.341117799	0.241333079	-0.175803795
ENSG00000120868	APAF1	4.60362	4.435112	4.311714	3.599704	4.118069	3.562499	3.77187	3.214036	3.925692	0.057171382	-0.190498359	0.08110611	-0.175748334
ENSG00000174498	IGDCC3	20.012518	18.352753	17.354189	10.444326	10.401803	10.558301	14.208416	15.043894	17.071245	2.74E-22	-0.681116886	0.048873372	-0.175611211
ENSG00000134986	NREP	77.707242	73.465083	71.849495	53.672617	51.824954	54.606012	64.589718	61.481723	62.453946	1.97E-10	-0.351897064	0.010122896	-0.17560582
ENSG00000164209	SLC25A46	10.718219	12.189725	10.810507	12.842442	10.829582	11.315735	10.133218	8.480708	9.166518	0.051467166	0.205161722	0.119762566	-0.175555924
ENSG00000166444	ST5	40.097578	39.183174	38.417961	24.225056	23.409535	19.198694	31.036144	31.248481	33.988895	2.16E-23	-0.613933551	0.018168592	-0.175537897
ENSG00000159842	ABR	13.176431	11.209172	11.422184	8.654101	7.845877	8.930685	10.86223	10.09291	11.777201	0.00000131	-0.404445654	0.063516703	-0.175529099
ENSG00000078747	ITCH	13.286451	11.881674	12.797075	11.763076	11.68072	11.306032	10.52117	9.465848	9.548025	0.076048026	0.124122026	0.032494616	-0.175499185
NewGene_5374	--	1.567353	1.935749	1.720276	0.628812	1.016512	0.676444	1.451688	1.363628	2.264007	0.0000755	-1.079311922	0.509252428	-0.175418576
ENSG00000133114	GPALPP1	2.682356	3.334344	2.695576	2.69017	2.64527	2.837742	2.485969	2.224072	2.524422	0.949163936	-0.014346408	0.270244854	-0.175374868
ENSG00000160710	ADAR	56.206575	55.367802	51.849782	128.33643	119.352805	123.786248	45.605839	41.428495	48.318661	1.79E-177	1.340557476	0.006916327	-0.17528261
ENSG00000162714	ZNF496	19.870285	18.373392	18.1344	16.75419	16.51845	16.690793	15.245258	15.01629	16.925705	0.683958884	-0.035471153	0.021710894	-0.175220346
ENSG00000112851	ERBIN	17.933417	20.720871	20.544651	21.845058	20.948554	22.278313	17.30091	16.439105	16.246131	0.001037742	0.192513605	0.010312731	-0.175194189
ENSG00000133433	GSTT2B	5.41409	5.791372	5.797049	4.615539	5.54065	6.318094	4.1803	5.006953	4.680556	0.730508261	0.085372342	0.375795001	-0.175135766
ENSG00000115053	NCL	230.566267	234.253708	245.582923	232.410037	213.981644	223.252588	195.234227	185.790418	210.780134	0.341718229	0.056620556	0.002360934	-0.175038558
ENSG00000099246	RAB18	6.261066	7.133948	7.418197	7.921351	7.638396	7.600958	6.243177	5.250304	5.747532	0.000559983	0.306400129	0.110668938	-0.175031465
ENSG00000004487	KDM1A	46.086157	46.197958	46.363227	40.741199	39.28069	40.022085	38.153742	37.711309	39.66588	0.322916321	-0.062878233	0.004423362	-0.174958445
ENSG00000152527	PLEKHH2	19.295375	21.078669	24.212712	15.931041	13.56416	13.470201	17.02438	16.927576	19.850526	0.00000016	-0.440556798	0.060619344	-0.174941289
ENSG00000101577	LPIN2	25.15293	23.551486	23.901687	29.729007	32.467307	29.903855	18.674711	18.861876	22.808486	3.44E-13	0.460339691	0.031131481	-0.174889672
ENSG00000130024	PHF10	18.91121	20.734855	22.358059	17.999483	16.510893	15.846804	17.52314	17.067057	17.455972	0.093438478	-0.167543255	0.066471953	-0.17484211
ENSG00000034053	APBA2	9.990048	9.621791	8.874874	8.861653	8.84226	9.206682	7.520422	7.979169	7.942129	0.621524903	0.055480553	0.072913586	-0.174833472
ENSG00000185024	BRF1	4.697109	4.571338	4.022696	4.160702	5.101426	4.705852	3.678453	3.306668	4.30697	0.43096701	0.128171295	0.221878745	-0.174758661
ENSG00000160145	KALRN	1.295838	0.658143	0.480073	0.945917	1.080041	0.819062	0.616626	0.580709	0.647377	0.091853129	0.431185726	0.524055105	-0.174668617
ENSG00000196967	ZNF585A	2.939866	2.293928	2.243414	1.178907	2.085344	1.923503	2.976115	3.211571	2.328788	0.430175095	-0.219883558	0.457225898	-0.174278771
ENSG00000082269	FAM135A	5.066452	4.99346	4.949847	3.874858	4.033737	4.167559	3.564671	3.88361	4.51372	0.071950261	-0.175781186	0.121301607	-0.174152458
ENSG00000125731	SH2D3A	3.119149	3.074459	3.372406	2.171688	1.410948	1.379018	2.672397	2.660825	2.556067	6.45E-08	-0.95243704	0.333229497	-0.17402722
ENSG00000035862	TIMP2	26.506681	23.368997	24.488127	20.834854	21.435566	21.488024	22.07446	18.570626	24.119277	0.254127884	-0.095760698	0.050668298	-0.173952975
ENSG00000278540	ACACA	19.926626	20.06784	19.528549	19.215168	19.070804	19.508355	17.543316	17.073278	17.656034	0.043502427	0.110922824	0.003356324	-0.173917595
ENSG00000240344	PPIL3	17.985919	16.686436	17.756687	17.280678	14.807403	15.0092	14.793644	15.526088	14.794372	0.853962939	-0.029354386	0.137701363	-0.173833957
ENSG00000221923	ZNF880	6.605382	9.159469	8.46895	5.045196	5.218428	4.981084	7.613705	7.705586	7.979562	0.0000758	-0.595624768	0.283167677	-0.1735439
ENSG00000196663	TECPR2	1.318895	1.063162	1.14661	1.000098	0.879969	1.080431	0.954472	1.02342	1.26444	0.277396637	-0.190032055	0.360309992	-0.173500661
ENSG00000066084	DIP2B	16.472149	16.549519	18.040226	15.243486	15.106758	14.732504	14.859131	12.923309	14.718477	0.696135462	-0.032396895	0.019281319	-0.173401893
ENSG00000198908	BHLHB9	0.311956	0.183688	0.208976	0.139581	0.281901	0.197343	0.175603	0.247134	0.195785	0.871685422	-0.099686136	0.712793853	-0.17334619
ENSG00000168778	TCTN2	3.444406	3.740195	3.324823	1.880919	1.70128	1.977386	2.670166	2.663347	3.307121	0.000000545	-0.74574116	0.273332101	-0.173105602
ENSG00000080031	PTPRH	2.024823	1.571336	1.073521	1.101917	1.207175	1.308686	1.207379	1.284044	1.354063	0.472037666	-0.204110977	0.496692248	-0.17306891
ENSG00000198924	DCLRE1A	7.61465	7.679377	7.7504	7.557235	6.332806	6.940341	6.407458	6.532899	6.121575	0.999202724	0.000264218	0.078471005	-0.172917516
ENSG00000008405	CRY1	9.864693	9.065711	11.313164	8.171414	8.277415	8.571733	9.313646	7.738992	9.538683	0.240213547	-0.136458614	0.132422541	-0.172652648
ENSG00000177150	FAM210A	5.639837	5.411704	6.143806	4.900841	5.065061	4.48747	4.673281	4.616191	4.086782	0.970170308	-0.006711673	0.160416543	-0.172645995
ENSG00000164181	ELOVL7	4.090863	4.743009	4.603169	6.772145	7.080535	6.849042	3.60964	3.686042	4.015577	2.64E-16	0.766292886	0.167564792	-0.172596884
ENSG00000111642	CHD4	65.699821	63.711247	61.87081	59.839787	53.817529	57.668599	52.404488	51.947221	57.16487	0.688861443	-0.029289424	0.006595093	-0.172501939
ENSG00000028203	VEZT	32.855655	30.697321	25.292634	26.086912	26.55028	24.599978	24.575115	21.361141	23.544895	0.899142427	0.011831124	0.013417598	-0.172435075

Gene ID	Gene name	Ctrl-1 FPKM	Ctrl-2 FPKM	Ctrl-3 FPKM	IFNγ-1 FPKM	IFNγ-2 FPKM	IFNγ-3 FPKM	Sora-1 FPKM	Sora-2 FPKM	Sora-3 FPKM	Ctrl vs IFNγ DESeq2 FDR	Ctrl vs IFNγ DESeq2 log2FC	Ctrl vs Sora DESeq2 FDR	Ctrl vs Sora DESeq2 log2FC
ENSG00000063660	GPC1	4.496542	4.090353	4.094965	3.150432	3.295091	3.650444	3.419458	3.300378	3.823765	0.133172956	-0.198639958	0.174671264	-0.172430458
ENSG00000164099	PRSS12	2.640204	2.273363	2.766079	1.54563	1.305693	1.291227	2.047981	2.131453	2.213606	0.000000149	-0.744040006	0.240976839	-0.17229408
ENSG00000113163	COL4A3BP	4.394084	3.796686	3.623685	3.504307	3.589575	4.030691	4.905196	3.835168	4.242831	0.466138433	0.085581522	0.103711599	-0.172265785
ENSG00000179715	PCED1B	2.166186	1.627427	2.553083	1.382133	1.827937	1.818005	1.643326	1.647234	1.721606	0.614269551	-0.14540539	0.492994364	-0.172222268
ENSG00000197081	IGF2R	54.388273	57.153724	50.127241	39.837952	41.116356	47.254737	42.799016	47.249713	55.766811	0.0000766	-0.219068529	0.009727278	-0.172218362
ENSG00000134077	THUMPD3	23.846635	23.84526	25.251076	21.233811	21.163872	22.627442	20.034866	20.478364	23.057529	0.724056896	-0.029544849	0.014996269	-0.171937333
ENSG00000183044	ABAT	19.709322	18.871735	18.87695	16.41328	16.689324	16.366781	16.478398	14.980863	16.42083	0.363307202	-0.068052082	0.016151672	-0.171637819
ENSG00000146414	SHPRH	4.185806	4.99139	5.381164	4.70822	4.191417	4.353698	3.846261	3.692172	4.124375	0.70994729	0.050325474	0.125984091	-0.171584904
ENSG00000141367	CLTC	77.844453	82.950399	83.278736	83.276844	82.054588	81.311346	66.948292	68.560316	74.82851	0.002598422	0.146754016	0.002449877	-0.171569521
ENSG00000285053	TBCE	2.706807	4.535854	2.795635	4.169399	3.35851	2.837895	3.410187	2.280382	2.299272	0.477389278	0.187401706	0.530812403	-0.17149502
ENSG00000145362	ANK2	6.799926	7.290927	7.27347	5.422921	5.814196	4.846906	5.344412	5.78674	6.018606	0.15475159	-0.145966672	0.097735181	-0.171343034
ENSG00000054965	FAM168A	6.357507	5.665541	5.481809	5.214715	5.331051	5.602761	4.856111	4.55859	5.277501	0.835045222	0.026479345	0.078757242	-0.171227979
ENSG00000182606	TRAK1	6.333348	7.102146	7.353444	5.097305	6.231942	5.821207	5.451018	6.414636	5.88415	0.3079344	-0.122716399	0.123374761	-0.171166781
ENSG00000091127	PUS7	15.172668	16.945395	17.610396	12.914578	11.628666	12.075644	13.642022	13.205763	14.645405	0.00038959	-0.292135569	0.051675494	-0.17113438
ENSG00000197279	ZNF165	4.265427	4.334011	4.958239	4.14677	4.638304	3.631354	4.022882	3.698225	3.556641	0.938973794	0.018141053	0.296857242	-0.171067668
NewGene 4768	--	3.085862	4.889822	3.506938	4.479141	4.254477	2.796747	4.183391	3.972698	3.681018	0.38965618	0.108525427	0.157062411	-0.17080943
ENSG00000011405	PIK3C2A	18.741571	21.255954	23.31623	27.032804	25.399629	25.164122	16.68478	16.96461	19.45762	1.56E-09	0.439296459	0.065023508	-0.170770416
ENSG00000175105	ZNF654	1.233025	1.383294	1.560812	1.504384	1.668765	1.717073	1.076185	1.212579	1.252475	0.010882307	0.37745851	0.310029972	-0.170713122
ENSG00000134146	DPH6	5.0956	5.696789	4.950376	4.644604	4.695889	4.565069	4.584223	3.981509	4.444864	0.928330535	-0.018168612	0.250377672	-0.170691184
ENSG00000167566	NCKAP5L	8.137798	7.690936	7.945371	7.850969	7.657495	8.248859	6.68816	5.811193	7.550554	0.07448582	0.154551659	0.083732787	-0.170658568
ENSG00000055044	NOP58	64.148896	64.449578	67.667149	63.711662	57.827664	58.407674	54.254378	54.188898	55.288756	0.798970066	0.021697132	0.010221786	-0.170541059
ENSG00000162645	GBP2	1.436563	0.95612	1.206847	33.70241	31.405249	28.698172	0.69534	0.578552	0.795859	0	5.061723813	0.570798784	-0.170299604
NewGene 557	--	5.131234	3.820725	3.717923	4.857426	3.520168	4.496038	3.17662	3.47862	4.378602	0.318855449	0.157684236	0.321377252	-0.17022571
ENSG00000165819	METTL3	17.613222	18.702505	20.06502	13.564816	10.594081	11.265526	14.337687	15.595466	16.942164	6.14E-08	-0.538070838	0.085162685	-0.170070076
ENSG00000110315	RNF141	5.695997	7.298059	7.186939	6.06679	6.072229	5.78205	5.501214	5.231254	6.064225	0.896445957	-0.021161561	0.16347675	-0.169916152
ENSG00000161791	FMNL3	2.726538	2.602066	2.185853	1.044546	1.114778	1.166698	2.007823	2.436945	2.357365	6.44E-16	-0.970879256	0.22536272	-0.169624546
ENSG00000113387	SUB1	70.816257	71.013222	83.590891	57.852827	53.865555	62.884549	59.440539	57.738648	65.616141	0.000811032	-0.186451557	0.007810978	-0.169486448
ENSG00000132640	BTBD3	26.070775	25.462347	26.388611	15.44265	15.215562	14.947765	21.044108	19.066551	25.034177	2.35E-30	-0.627530678	0.047163279	-0.169449706
ENSG00000072609	CHFR	22.674999	17.724823	19.492709	14.157093	19.875232	18.514654	17.693208	18.128741	19.128552	0.935459392	0.008368381	0.024885747	-0.169383533
ENSG00000120733	KDM3B	15.081523	13.852395	13.733503	14.194808	13.442442	13.269898	11.881034	11.052717	13.233012	0.261934068	0.08566267	0.026262542	-0.169373577
ENSG00000139636	LMBR1L	4.605747	4.297991	5.183802	3.193106	3.089296	3.495892	3.097721	3.934063	4.745582	0.05887382	-0.274825552	0.296064941	-0.169098396
ENSG00000163743	RCHY1	4.186318	4.828863	4.615021	5.336785	4.124086	4.445331	3.512757	4.013532	4.284508	0.287842499	0.144593662	0.179917075	-0.169047395
ENSG00000123472	ATPAF1	11.215736	10.618709	10.632559	11.913803	13.755236	11.242062	9.348732	9.72079	9.88423	0.001527605	0.254005116	0.068400165	-0.169009442
ENSG00000018408	WWTR1	6.349706	5.652026	6.078933	3.80487	3.951341	4.145235	5.208471	4.749749	5.802712	0.00000114	-0.465503102	0.11618905	-0.168939135
ENSG00000103415	HMOX2	11.371821	9.003752	7.44978	8.526571	7.989316	9.142162	7.383101	9.040492	6.07866	0.955711474	-0.010849303	0.2641632	-0.168736772
ENSG00000182180	MRPS16	36.091172	37.225895	36.780285	29.652372	30.795061	32.392179	30.847576	30.873974	30.048464	0.205837711	-0.094070346	0.019093574	-0.168648086
NewGene 1715	--	0.510652	0.397383	0.835427	1.852878	0.870668	0.490964	0.656966	0.533587	0.350987	0.029914478	0.883662221	0.71474475	-0.168618738
NewGene 772	--	3.641363	3.291505	3.745753	3.502917	3.302136	3.221857	2.853254	2.85112	3.341419	0.059125448	0.216245056	0.255386618	-0.168508595
ENSG00000163738	MTHFD2L	1.991489	3.171779	2.145605	2.23039	2.47349	1.832812	1.755674	1.785018	2.476591	0.488248295	-0.175820989	0.453857272	-0.16849083
NewGene 1725	--	0.555673	0.646089	0.740871	0.567099	0.291577	0.464309	0.496615	0.670983	0.43555	0.278874814	-0.406073825	0.635179546	-0.168424847
ENSG00000214940	NPIPA8	7.206718	4.999744	4.926053	3.305215	5.32093	3.297153	4.901932	4.269368	3.92684	0.1784381	-0.353075104	0.512198389	-0.168405702
NewGene 1249	--	1.211555	1.279153	1.222271	3.467498	4.352335	3.407802	1.055692	1.099974	0.93784	6.05E-26	1.737230559	0.528675596	-0.168400345
ENSG00000161217	PCYT1A	9.969804	9.46443	9.442327	8.688484	8.956399	9.360674	7.98945	8.148426	8.830276	0.518085063	0.061393301	0.041081535	-0.168399662
ENSG00000105877	DNAH11	0.070828	0.12588	0.088071	0.086114	0.071737	0.046576	0.082833	0.052758	0.101711	0.509573907	-0.335734934	0.717556871	-0.16824089
ENSG00000088899	LZTS3	7.574355	7.539271	8.223198	5.95416	5.768738	6.274332	6.32953	6.10364	6.754889	0.013011403	-0.224967942	0.091088941	-0.168037241
ENSG00000180822	PSMG4	6.324745	7.014281	6.265871	8.030968	8.717154	9.372771	7.39337	6.802777	6.903156	0.0000374	0.496719868	0.284008877	-0.167958857
ENSG00000097046	CDC7	8.249221	9.48664	9.817348	8.428341	8.01825	7.914237	7.489398	7.265345	8.223503	0.851477351	-0.026450626	0.117265847	-0.167917338
ENSG00000186141	POLR3C	25.708273	23.517206	23.397284	20.248701	22.609914	21.454238	20.517579	19.549642	20.745601	0.827011529	-0.026857533	0.055042483	-0.167812794
ENSG00000196730	DAPK1	19.020223	18.109387	17.846483	19.260133	18.243333	18.395992	15.401818	13.666574	16.761432	0.003980348	0.173693483	0.028503342	-0.16762865
ENSG00000137962	ARHGAP29	11.086471	11.240163	12.720384	7.749613	8.391487	7.647653	9.891843	8.536776	10.950536	9.52E-08	-0.415339857	0.077902252	-0.167570243
ENSG00000116685	KIAA2013	23.781131	20.681388	20.024813	19.52086	20.758198	22.541651	16.724708	16.903925	20.344441	0.288176171	0.107035061	0.09213629	-0.167472544
ENSG00000074266	EED	9.284574	10.621632	10.570238	9.496172	8.20568	9.670545	7.883533	7.748418	8.437886	0.904037509	0.019850344	0.160186773	-0.167407485
NewGene 3390	--	7.144204	7.716917	7.457479	9.581066	8.385469	8.714795	6.57722	5.328472	6.327745	0.00000249	0.430847816	0.155687477	-0.167396816
ENSG00000122483	CCDC18	2.056803	2.760667	2.952922	2.960599	1.863839	2.091628	2.184467	2.005717	2.191636	0.949528958	-0.016248896	0.297107435	-0.167391992
ENSG00000143278	F13B	0.460894	0.594243	0.511422	0.242457	0.287995	0.301069	0.450673	0.411003	0.436434	0.154195372	-0.680531819	0.717321391	-0.167272308
ENSG00000103126	AXIN1	8.81665	7.927006	8.142743	7.348737	7.036734	7.039579	7.131791	5.912549	7.684143	0.510124624	-0.078201353	0.160301205	-0.167168217
ENSG00000109929	SC5D	43.423565	44.298849	43.579868	49.144936	50.845152	48.74994	34.255211	36.924618	38.61659	7.81E-10	0.339100943	0.0153449	-0.167154593
ENSG00000169116	PARM1	0.59027	0.582802	0.546433	0.784629	0.701557	0.850969	0.483838	0.443319	0.570445	0.005628742	0.615091206	0.550554416	-0.167117552
ENSG00000146729	NIPSNAP2	58.908586	55.293367	51.63543	46.625299	41.507757	41.938312	47.042304	45.703403	44.833149	0.00061978	-0.217008157	0.023699395	-0.166765662
ENSG00000181220	ZNF746	4.520649	5.113931	4.779805	4.818809	5.25332	4.833694	4.213109	3.367056	4.450948	0.07730396	0.201478948	0.208853242	-0.166724384
NewGene 2507	--	2.340942	2.698305	2.722479	2.316651	2.190301	2.165364	2.335724	1.895682	2.267733	0.640562796	-0.073963686	0.233413494	-0.166681067
ENSG00000152133	GPATCH11	2.001226	1.937745	2.364036	2.002658	2.00893	1.972094	1.679881	1.873325	1.695379	0.70994729	0.07614347	0.353637528	-0.166675759
ENSG00000152700	SAR1B	4.452988	5.384344	5.264379	6.964042	6.729892	6.557236	4.885791	4.162161	4.203472	1.23E-10	0.554555198	0.129240958	-0.166672986
ENSG00000115107	STEAP3	8.566764	9.310256	10.201735	6.881272	7.486012	8.19929	6.464335	7.497769	8.081962	0.508141255	-0.079960185	0.124739601	-0.166590588
ENSG00000118007	STAG1	11.287878	10.761301	11.180558	9.886992	9.701783	9.551611	9.423815	8.634807	9.84105	0.705790194	-0.034145175	0.029404475	-0.166589009
ENSG00000150867	PIP4K2A	12.810675	12.542117	12.232101	17.46295	16.687528	16.359524	10.375281	10.013227	11.416625	3.68E-19	0.569895766	0.0431222	-0.166546469
ENSG00000130311	DDA1	13.934567	13.696359	13.726033	10.630888	10.185905	10.452524	10.758195	11.538452	12.158424	0.000670672	-0.237520227	0.038060322	-0.166501534
ENSG00000180801	ARSJ	1.236649	0.95998	1.131418	2.280007	2.030432	2.284321	0.827857	0.754374	0.903315	3.41E-14	1.131792524	0.455410244	-0.16633261
ENSG00000095319	NUP188	18.739496	19.047714	17.956181	15.469017	13.803522	14.913215	15.629421	14.218695	16.805363	0.004056923	-0.188361562	0.02135921	-0.166245764
ENSG00000111785	RIC8B	5.803974	4.812671	6.387561	5.918776	4.032736	4.112691	4.122262	4.29199	4.540779	0.558815456	-0.084456141	0.197876442	-0.166088803
ENSG00000138448	ITGAV	29.208527	33.440982	38.555976	16.736925	17.116344	16.731339	27.184074	26.28401	31.643583	1.92E-27	-0.851835588	0.092576036	-0.165813394
ENSG00000118513	MYB	2.741683	3.423404	3.276009	3.691302	3.195349	3.824909	3.046804	2.413946	2.653937	0.048692637	0.289776838	0.31402032	-0.16570361
ENSG00000025800	KPNA6	8.11702	7.879825	8.607807	8.532048	7.905139	8.204436	6.812897	6.868446	6.88888	0.045937369	0.149365039	0.040973458	-0.165698968
ENSG00000136895	GARNL3	1.770329	0.998062	1.809422	1.211818	1.066285	0.850833	1.113823	1.264052	1.358522	0.356841899	-0.277745116	0.512198389	-0.1655825

Gene ID	Gene name	Ctrl-1 FPKM	Ctrl-2 FPKM	Ctrl-3 FPKM	IFNγ-1 FPKM	IFNγ-2 FPKM	IFNγ-3 FPKM	Sora-1 FPKM	Sora-2 FPKM	Sora-3 FPKM	Ctrl vs IFNγ DESeq2 FDR	Ctrl vs IFNγ DESeq2 log2FC	Ctrl vs Sora DESeq2 FDR	Ctrl vs Sora DESeq2 log2FC
NewGene 4118	--	7.434877	8.991859	9.749184	8.910107	8.44196	7.826175	7.29576	7.105237	7.829704	0.626581457	0.06598916	0.13450679	-0.165384799
ENSG00000131791	PRKAB2	14.275076	14.739208	15.558357	13.575237	13.757313	12.8072	12.791907	11.910455	12.571425	0.972936091	-0.003935176	0.027533274	-0.165137656
ENSG00000073584	SMARCE1	45.661629	51.047083	50.270801	38.959467	43.798205	43.62522	44.368185	41.992075	40.154316	0.530057555	-0.045626017	0.012203394	-0.164967843
ENSG00000120685	PROSER1	12.075055	11.061117	12.819707	11.770709	10.820016	10.831882	9.205307	8.743064	12.107862	0.630990042	0.049406117	0.144547773	-0.164957008
ENSG00000198835	GJC2	2.0436	1.984117	1.982303	0.700626	0.796936	0.773204	1.38772	1.959805	1.661241	1.91E-08	-1.247941822	0.496334261	-0.164905112
ENSG00000004455	AK2	43.952851	40.439669	38.124951	48.481554	44.048128	42.858922	31.823778	36.618147	36.323476	3.38E-10	0.351798409	0.043948214	-0.164688307
ENSG00000101986	ABCD1	5.202766	4.219127	3.555333	4.166066	4.542479	4.68316	3.601519	3.091291	3.81912	0.189691994	0.210034365	0.310122835	-0.164359476
ENSG00000145685	LHFPL2	4.789262	5.964514	6.146898	3.636498	3.540123	4.037883	4.646906	4.697299	4.623757	0.000153688	-0.426348839	0.171411767	-0.164269258
ENSG00000177602	HASPIN	6.855255	6.137193	6.868568	5.037451	5.292867	4.887783	5.904678	5.368494	5.34358	0.045208228	-0.237791128	0.183928205	-0.163985435
ENSG00000016391	CHDH	10.746894	10.244721	10.050389	8.556485	8.492617	8.179951	8.480677	7.906652	9.703634	0.030761026	-0.150916628	0.042665144	-0.16391667
ENSG00000160360	GPSM1	8.711022	7.348578	7.601639	6.899349	5.48858	6.688187	6.843929	5.929672	7.831288	0.383830573	-0.125415744	0.198040865	-0.163828209
ENSG00000152217	SETBP1	1.870729	1.786177	1.749329	2.005814	2.069018	2.041773	1.634682	1.658927	1.655404	0.007450749	0.281502791	0.18645688	-0.163820939
ENSG00000133703	KRAS	21.69508	24.46447	26.778437	21.330503	20.312955	20.560655	22.018433	19.243833	20.812266	0.272277241	-0.09930756	0.073322976	-0.163722512
ENSG00000121417	ZNF211	3.052947	2.936027	2.80166	2.548367	2.550441	1.998202	2.912279	2.089273	2.322372	0.431210674	-0.162941291	0.394604097	-0.163514806
ENSG00000186854	TRABD2A	8.201825	7.903498	8.81842	4.274383	4.359522	4.344086	6.408295	6.579959	7.739653	5.85E-11	-0.784544709	0.210013198	-0.163513986
ENSG00000111269	CREBL2	3.801791	4.249565	3.875875	2.821869	3.382736	2.716664	3.375705	3.082941	3.539612	0.053964057	-0.271869945	0.20498633	-0.163264973
ENSG00000163581	SLC2A2	1.4052	1.726677	1.650858	1.213523	1.021669	1.190857	1.519665	1.298293	1.163679	0.087820528	-0.377518859	0.476816585	-0.163260835
ENSG00000170365	SMAD1	4.606718	4.912497	5.332674	5.294137	4.097808	4.161548	4.395524	3.804543	4.19498	0.865635807	0.037479427	0.30773301	-0.163213253
ENSG00000081019	RSBN1	2.132342	2.148497	2.296649	1.923628	2.012243	1.992645	1.918967	1.911122	1.964903	0.999571371	0.0000961	0.222517932	-0.163104393
ENSG00000126249	PDCD2L	16.386243	14.158715	17.586882	11.889252	12.584948	13.190257	13.7927	14.467565	12.135792	0.130528775	-0.203158561	0.251929501	-0.163057809
ENSG00000141219	C17orf80	12.045351	12.142983	12.792912	12.46567	11.843116	11.195206	10.533933	9.867902	10.20191	0.436038441	0.076358077	0.064636403	-0.162977402
ENSG00000181625	SLX1B	28.097797	26.005168	26.300024	17.645216	19.191275	20.011287	21.576498	22.611826	24.341071	0.000492259	-0.333158289	0.101640413	-0.162935935
ENSG00000149346	SLX4IP	1.508606	1.753952	1.689267	1.645625	1.501241	1.342905	1.42969	1.054517	1.537768	0.946510268	-0.014746997	0.354560354	-0.162837156
ENSG00000157837	SPPL3	18.974796	18.590781	18.634157	19.627058	19.113435	19.277283	16.206684	14.822755	16.380795	0.001819133	0.191033932	0.022605645	-0.162769475
ENSG00000135776	ABCB10	11.516331	13.211514	12.38074	12.74917	12.693567	12.785258	10.528315	9.953586	10.59654	0.017975051	0.19083141	0.065172818	-0.162702161
ENSG00000132313	MRPL35	6.160704	4.54344	4.33368	4.61743	4.568142	4.326851	4.041318	4.058502	4.557447	0.859832006	0.027889662	0.176539262	-0.162611325
ENSG00000171302	CANT1	24.218474	22.201082	21.626026	19.91967	18.754428	21.322636	18.282959	18.346338	19.999864	0.896009013	0.013378639	0.038924805	-0.162532608
ENSG00000064652	SNX24	3.85885	3.773779	3.625494	2.359544	2.442093	2.481768	2.483444	3.29233	3.966769	0.00278562	-0.500882467	0.399702416	-0.162520935
ENSG00000115657	ABCB6	4.955399	6.438294	6.11864	6.641882	6.895133	5.346295	3.747902	5.617522	5.127922	0.064577534	0.268044456	0.368970486	-0.162452524
ENSG00000128829	EIF2AK4	6.183204	6.868112	6.08468	6.018459	5.160521	5.68146	5.928801	4.954389	5.304784	0.807088209	-0.032511065	0.130664023	-0.162243605
ENSG00000167114	SLC27A4	8.104889	7.772117	7.513787	6.538277	6.361705	5.649847	6.138319	5.915843	7.736983	0.085643864	-0.19301902	0.176158918	-0.162196787
ENSG00000067141	NEO1	1.93205	1.664907	1.443653	1.87801	1.993737	1.835743	1.397064	1.348592	1.493417	0.134341008	0.22983171	0.31270772	-0.162068877
ENSG00000113552	GNPDA1	35.363833	38.403049	36.964304	33.455099	34.884591	32.470644	30.871349	31.884321	30.495456	0.682799116	0.035378199	0.031551116	-0.162058575
ENSG00000127989	MTERF1	6.874923	8.048372	8.533216	8.767225	6.937918	8.363483	5.918588	5.257033	7.9516	0.238030719	0.164168365	0.28763128	-0.16205206
ENSG00000112769	LAMA4	0.553305	0.365936	0.425154	0.213147	0.126219	0.186673	0.332701	0.441273	0.424133	0.000260601	-1.156266365	0.606943314	-0.162002487
ENSG00000196313	POM121	22.782561	24.004872	23.632678	24.390413	22.495072	23.278349	18.489922	19.139248	23.029445	0.021153496	0.133607023	0.037325399	-0.161998477
ENSG00000136044	APPL2	8.262081	8.354376	8.193746	6.752139	6.623603	6.596326	6.393829	5.696393	7.800053	0.118283374	-0.161170345	0.168873513	-0.161956167
ENSG00000163389	POGLUT1	10.871627	12.158848	11.712447	9.746668	9.428303	9.627823	9.389336	10.554893	10.022243	0.180426286	-0.119660317	0.105188139	-0.161915527
ENSG00000162065	TBC1D24	2.773191	2.692891	2.553625	3.126475	2.891118	3.130401	2.345158	2.064089	2.344483	0.00142158	0.322872448	0.174374849	-0.161814573
ENSG00000086848	ALG9	4.368226	4.066486	4.685023	3.786613	3.417431	3.742909	3.960446	3.607528	4.606105	0.669741566	-0.072010727	0.257542171	-0.161810185
ENSG00000096401	CDC5L	17.25783	17.352907	16.520283	15.209094	14.327031	14.391531	14.659813	13.642783	14.571608	0.302641162	-0.072107838	0.016520911	-0.161783826
ENSG00000171456	ASXL1	36.080983	38.481869	39.515769	37.601332	37.231049	33.949085	30.687303	30.69643	35.733649	0.600169385	0.036892591	0.019149662	-0.161738854
ENSG00000110046	ATG2A	12.712406	12.176815	10.07225	15.620673	15.211884	16.349566	8.116359	8.899458	11.645509	1E-13	0.587908402	0.194602011	-0.161728275
ENSG00000148730	EIF4EBP2	33.342251	31.50145	31.315512	31.070107	31.374268	31.08511	27.512033	25.329361	27.833492	0.06874055	0.10731905	0.010191671	-0.161584101
ENSG00000167972	ABCA3	1.071512	0.965056	0.731854	0.900173	0.753295	1.012606	0.700599	0.687915	0.883933	0.628562029	0.12274853	0.46356195	-0.161521957
ENSG00000143751	SDE2	13.151797	13.704283	14.00208	15.44124	13.994354	15.147588	12.279856	10.394558	11.606373	0.0000902	0.273208756	0.063871041	-0.161457956
ENSG00000169627	BOLA2B	37.126319	32.638428	29.527674	26.6188	28.020589	29.168191	27.505743	32.535478	22.610294	0.400254987	-0.107223082	0.298875341	-0.161225028
ENSG00000198369	SPRED2	22.592775	21.891115	23.716622	20.867073	21.454129	19.768041	18.356583	18.350085	22.191196	0.946098761	-0.007209217	0.05870315	-0.161211299
ENSG00000151876	FBXO4	7.04071	9.105182	8.586265	6.871201	7.292233	7.888485	6.853556	6.561111	7.432266	0.842479322	-0.039606007	0.279820985	-0.161163181
ENSG00000185155	MIXL1	0.830984	0.774819	1.051319	0.438065	0.411443	0.685602	0.852064	1.051861	0.491297	0.254139521	-0.479726733	0.696966746	-0.160981425
ENSG00000109686	SH3D19	16.242081	16.381931	17.760009	17.820036	16.734662	15.813801	14.957462	14.157804	15.091133	0.084218833	0.128850378	0.026547843	-0.160970838
ENSG00000196233	LCOR	6.104018	7.085912	7.259807	8.292343	7.962795	8.207552	6.068248	5.437184	5.627874	7.27E-09	0.404327551	0.066035564	-0.160967237
ENSG00000075292	ZNF638	17.678434	17.755881	17.473704	18.112295	16.52142	14.920279	14.319945	15.108664	16.397401	0.878471082	0.015717902	0.018619316	-0.160925451
ENSG00000157911	PEX10	15.874219	15.933532	14.887025	14.999367	14.348492	15.019104	14.585175	13.576693	12.908882	0.367197194	-0.112157686	0.177958503	-0.160911576
NewGene 1128	--	3.754285	4.792996	5.119808	4.34969	4.411047	4.234383	3.559188	3.826379	3.656041	0.879330396	0.031248171	0.304932572	-0.160815514
ENSG00000135678	CPM	1.63636	1.53921	1.467253	1.219507	1.067183	1.2062	1.950591	1.269023	1.175332	0.144754335	-0.228010115	0.306933973	-0.160733801
ENSG00000095574	IKZF5	4.609483	5.7201	5.826717	5.802532	5.501517	5.800446	4.476085	4.096386	4.990001	0.040746088	0.231442849	0.203956196	-0.160624097
ENSG00000203880	PCMTD2	13.701771	13.144722	13.56072	14.308515	12.896461	13.120488	11.413043	10.498192	12.600268	0.193548162	0.112883239	0.058133443	-0.160560247
ENSG00000103111	MON1B	6.992993	6.884634	6.565331	7.661164	6.834276	7.403203	5.88278	5.458922	6.351746	0.007479728	0.2068096	0.087684606	-0.160459775
ENSG00000131467	PSME3	90.798016	92.143238	99.351567	90.045172	81.534473	129.051277	82.60899	80.105919	80.177117	0.949006613	0.00602043	0.015438295	-0.160295289
ENSG00000142459	EVI5L	4.766215	4.553229	3.356828	4.8003	4.487728	4.827291	3.190307	3.606983	3.836929	0.033275723	0.299733064	0.317411349	-0.160247788
ENSG00000170962	PDGFD	3.256181	3.775336	4.076496	2.730451	2.314976	2.749999	2.953937	3.076772	3.269629	0.009365997	-0.359041012	0.252039552	-0.160237358
ENSG00000163945	UVSSA	6.200197	3.162856	6.627545	8.365879	6.277234	7.417107	2.837411	2.439768	6.719026	0.411797364	0.094936608	0.196817799	-0.160142972
ENSG00000104164	BLOC1S6	16.127093	20.428845	20.73458	21.096037	21.254348	20.464171	16.115425	14.974933	16.059261	0.003446235	0.262754158	0.108925734	-0.160122534
ENSG00000135469	COQ10A	4.735527	4.707061	4.689859	5.645642	5.261605	4.264762	3.996134	3.871684	4.164803	0.224613148	0.221130437	0.351859247	-0.160076954
ENSG00000285447	ZNF883	2.784643	3.106988	3.514831	2.388399	2.058515	1.934879	2.568766	2.596587	2.720921	0.036079816	-0.411244098	0.413123545	-0.159472066
ENSG00000088727	KIF9	1.71316	1.083889	1.52584	0.792024	1.063912	1.096587	1.188563	0.975925	0.932391	0.695091178	-0.166690872	0.657607038	-0.159447092
ENSG00000167995	BEST1	24.367064	0.816436	0.782274	16.238967	2.083356	5.557608	0.783192	19.128247	1.213574	0.995606059	0.016905628	0.950597841	-0.159224866
ENSG00000253251	SHLD3	3.236205	3.561804	3.661498	2.896682	2.405372	2.806661	2.464257	2.683274	3.172507	0.550696099	-0.135855425	0.430559456	-0.159208417
NewGene 287	--	1.37739	0.902612	1.311473	0.921092	1.171907	0.927762	0.848208	1.112625	0.949695	0.912634026	-0.046784447	0.623974038	-0.159148085
ENSG00000102393	GLA	15.862755	15.824023	16.925398	13.872877	13.794099	12.766775	12.191859	14.325663	14.318816	0.282417763	-0.126581194	0.186993156	-0.159010486
ENSG00000243667	WDR92	4.838311	5.330941	6.313636	4.155212	4.405478	4.474346	4.420524	3.934124	4.814578	0.353088745	-0.150539573	0.288637948	-0.158977333
ENSG00000171033	PKIA	4.899462	4.723215	4.234449	3.703203	3.281129	3.145612	3.808571	4.008566	4.288217	0.003235	-0.337305904	0.181832195	-0.158846381
ENSG00000139722	VPS37B	10.085294	10.491006	9.309924	6.49645	7.146974	7.473716	8.80877	7.922396	8.380784	0.000394032	-0.351418113	0.117716228	-0.158749537

Gene ID	Gene name	Ctrl-1 FPKM	Ctrl-2 FPKM	Ctrl-3 FPKM	IFNγ-1 FPKM	IFNγ-2 FPKM	IFNγ-3 FPKM	Sora-1 FPKM	Sora-2 FPKM	Sora-3 FPKM	Ctrl vs IFNγ DESeq2 FDR	Ctrl vs IFNγ DESeq2 log2FC	Ctrl vs Sora DESeq2 FDR	Ctrl vs Sora DESeq2 log2FC
ENSG00000103942	HOMER2	5.435292	6.010799	7.574504	3.560588	3.354137	3.438549	4.744118	5.198975	6.307298	0.000000251	-0.732463806	0.325282493	-0.158734728
ENSG00000125457	MIF4GD	7.071503	7.388772	6.356095	6.541518	5.332451	5.600344	5.682637	7.026112	5.196922	0.592379848	-0.100778419	0.358107804	-0.158710857
ENSG00000167106	FAM102A	3.628754	3.630201	3.304172	3.145436	2.749226	3.076525	3.276011	3.072188	2.516368	0.561842644	-0.08901236	0.309613559	-0.157997221
ENSG00000132846	ZBED3	8.163365	8.946161	9.062923	8.499691	7.840717	8.209316	7.495766	6.844705	7.682368	0.56597332	0.055199188	0.062254428	-0.157836767
ENSG00000151413	NUBPL	3.575892	4.370988	4.510445	2.924324	2.841338	3.37191	3.607392	3.401162	3.476214	0.051112108	-0.299995689	0.298449936	-0.157756782
ENSG00000123600	METTL8	3.220482	3.825797	4.154006	2.847579	3.106893	2.884492	3.550058	3.148266	3.425989	0.154477627	-0.180231763	0.205677307	-0.157677323
ENSG00000006118	TMEM132A	26.752224	23.728614	23.593213	22.857292	23.614786	26.196646	18.885696	20.147304	23.520246	0.160967465	0.128810233	0.102676724	-0.157588352
ENSG00000197362	ZNF786	1.648314	1.347911	1.520158	1.202913	1.25	1.188408	1.122088	1.197986	1.469846	0.515917769	-0.163750184	0.497631989	-0.157581989
ENSG00000074800	ENO1	764.613358	705.485592	647.982805	568.081965	555.081467	575.611801	566.514534	601.31602	611.15803	0.010438547	-0.169415638	0.038300294	-0.157552802
ENSG00000132603	NIP7	14.676926	17.427468	18.735732	17.710101	14.329413	15.955831	12.944725	13.766075	15.274667	0.604133002	0.070867751	0.170420492	-0.1574627
ENSG00000013364	MVP	4.991082	3.650602	3.886378	7.893545	7.051102	8.898031	3.332835	3.25837	3.993284	8.93E-20	1.14519503	0.370922181	-0.157373328
ENSG00000183785	TUBA8	1.340832	1.520697	1.760281	1.676906	1.249613	1.313264	0.555942	1.339	1.839322	0.855944088	0.069840878	0.718034249	-0.157356499
ENSG00000167315	ACAA2	25.524032	32.232003	31.927856	24.561608	23.086878	23.32307	23.332505	27.094742	27.191744	6.41E-08	-0.344605769	0.038951106	-0.157297744
ENSG00000164609	SLU7	17.479574	17.017165	18.627239	16.182238	17.408312	15.963057	15.539291	14.808194	14.985191	0.649986257	0.044645763	0.049000056	-0.157269771
ENSG00000110237	ARHGEF17	2.877858	2.792472	2.440553	2.104357	1.72669	1.914639	2.260137	2.369951	2.81424	0.002730268	-0.343499	0.201101313	-0.15720803
ENSG00000186654	PRR5	4.403895	4.358599	3.288887	5.654023	5.279422	5.496962	3.424526	3.764383	2.954869	0.000255998	0.556534448	0.448658399	-0.15719175
ENSG00000148296	SURF6	10.826555	10.907321	10.811035	9.560218	9.70874	9.828918	9.711389	8.123768	9.571909	0.900797649	-0.013541215	0.076475166	-0.157183603
ENSG00000128989	ARPP19	45.966553	52.459961	54.398636	48.066866	46.882648	45.638492	44.331646	40.587846	43.972152	0.60515536	0.049236035	0.055685341	-0.157181644
ENSG00000151366	NDUFC2	27.433038	27.150329	27.18398	31.357195	32.985368	34.166874	23.655685	23.594418	21.768333	3.73E-12	0.442661368	0.080488987	-0.15692703
ENSG00000082898	XPO1	87.569056	92.462282	98.105823	84.327686	83.509407	84.498349	72.401123	68.838957	66.926375	0.975648072	-0.002990608	0.01859499	-0.156820571
ENSG00000198205	ZXDA	0.407328	0.38821	0.206222	0.320194	0.379074	0.285478	0.341427	0.193221	0.307864	0.828374963	0.115430634	0.739403341	-0.156690302
ENSG00000185345	PRKN	0.188118	0.261873	0.247278	0.266384	0.234021	0.20288	0.236919	0.155751	0.241792	0.826252675	0.126341799	0.755344774	-0.156686658
ENSG00000100796	PPP4R3A	21.540895	20.563112	23.222981	22.246693	22.512672	20.872652	18.66023	16.484647	19.524955	0.062754908	0.14195792	0.05921252	-0.156573737
ENSG00000078124	ACER3	1.459892	1.755104	1.418622	1.615881	1.242837	1.452025	1.328302	1.214281	1.337073	0.962004888	-0.010702582	0.298081608	-0.156545496
ENSG00000115539	PDCL3	13.827247	12.576065	13.078808	11.487587	11.606744	12.839995	10.734342	11.206456	11.42872	0.974892029	0.005828347	0.193325557	-0.156409924
ENSG00000117262	GPR89A	22.389234	22.523087	24.605175	22.391552	21.91338	18.834601	18.485207	20.690078	20.033075	0.962563224	-0.006677199	0.094429686	-0.156270139
NewGene_5306	--	1.040579	1.079105	0.914511	1.230546	0.912394	1.088301	0.961463	0.741936	0.782932	0.244226719	0.222280331	0.440119534	-0.156241742
ENSG00000068650	ATP11A	26.390446	26.647521	28.23893	21.256766	23.871106	22.133555	20.88939	20.797157	27.488889	0.068241215	-0.123028113	0.087631517	-0.156216706
ENSG00000145779	TNFAIP8	2.645165	2.62808	3.079784	1.446538	1.064955	1.391953	2.11895	2.080948	2.655971	1.37E-10	-1.011535658	0.413123545	-0.156133887
ENSG00000204305	AGER	3.460464	4.448894	4.018566	3.266518	3.875285	3.172463	2.98156	2.6905	4.39666	0.818462962	-0.06123239	0.508124123	-0.155836378
ENSG00000051382	PIK3CB	7.741242	7.81251	7.423636	6.816685	5.749683	6.074964	6.227848	6.060009	6.807488	0.049891157	-0.182204625	0.089923715	-0.155803502
ENSG00000141564	RPTOR	10.496145	9.478175	9.19597	9.948648	8.502177	10.089039	8.453574	7.958285	9.807369	0.164563844	0.130113136	0.088189379	-0.155708118
ENSG00000162971	TYW5	2.363028	2.857195	3.308895	2.726651	3.180367	2.821635	2.528179	2.24178	2.749247	0.277253133	0.193838063	0.385262058	-0.155655811
ENSG00000276043	UHRF1	31.626817	29.100061	29.594134	23.867609	21.894178	24.150228	26.689277	22.044341	27.581225	0.001942868	-0.206181365	0.044948417	-0.155514747
ENSG00000120253	NUP43	12.976337	14.906689	14.687553	12.597598	12.271431	12.17283	12.025034	11.393867	12.365454	0.558312342	-0.058380342	0.073751666	-0.155451611
ENSG00000271601	LIX1L	4.584521	4.103556	4.109097	3.471154	3.478311	3.589454	3.687455	3.415788	3.686098	0.316670603	-0.132177737	0.207440086	-0.15542514
ENSG00000204334	ERICH2	3.071432	1.920148	2.273565	1.910656	1.567701	1.075389	1.914367	1.962862	2.192516	0.23687522	-0.535472167	0.710496614	-0.155417515
ENSG00000185864	NPIPB4	7.459432	9.241016	11.9374	10.080328	6.406907	8.66858	8.011616	6.701874	6.608544	0.790156759	-0.058558621	0.321738564	-0.155139371
ENSG00000131504	DIAPH1	21.728707	21.816647	21.757912	20.361658	19.748237	19.429825	18.031293	17.331531	19.831478	0.828882123	0.017242658	0.019962503	-0.154736726
ENSG00000129128	SPCS3	20.654413	25.589319	26.754284	25.789057	23.299816	25.283155	18.406265	18.88719	24.356646	0.066950414	0.174379867	0.18407109	-0.154596241
ENSG00000144554	FANCD2	16.13217	15.935322	15.937212	11.989621	10.029333	11.119193	13.21184	12.898788	13.523315	5.73E-08	-0.375790127	0.036483016	-0.154552119
ENSG00000091138	SLC26A3	0.24071	0.468426	0.345298	0.361527	0.331672	0.365574	0.333821	0.298428	0.246571	0.777736871	0.161463365	0.76687663	-0.154541395
ENSG00000122547	EEPD1	9.578478	8.815104	9.075932	5.092091	4.826344	5.041412	7.18557	7.471853	8.515469	2.14E-21	-0.729412263	0.096506968	-0.154536881
ENSG00000096968	JAK2	0.80375	0.828333	0.927748	5.938128	5.33802	5.077736	0.72658	0.657286	0.671181	2.99E-150	2.924949503	0.504681468	-0.15452616
ENSG00000166004	CEP295	3.69901	4.295372	3.787594	4.373208	3.804573	3.799636	3.668102	3.242262	2.921799	0.044588196	0.197046889	0.225634082	-0.154340626
ENSG00000113643	RARS	39.373852	40.311035	38.037106	54.061765	51.178745	52.427738	34.015026	31.918401	33.857629	2.75E-25	0.56759684	0.032272251	-0.154269396
ENSG00000120899	PTK2B	1.364754	1.112737	1.009488	1.207278	1.162592	1.066531	0.935551	0.813489	1.09749	0.550262317	0.142777484	0.501480204	-0.154199965
ENSG00000104728	ARHGEF10	2.904957	2.720924	2.902822	1.979416	1.598503	1.063629	2.446881	2.223727	2.479233	0.0000724	-0.737586359	0.22083366	-0.154128099
ENSG00000076650	GPATCH1	4.458443	3.993373	4.261287	5.03107	4.387804	4.666451	3.871866	3.222841	3.619556	0.011621718	0.293112982	0.254397563	-0.154096461
ENSG00000111224	PARP11	1.869495	2.028869	2.215475	3.519276	3.529924	3.587911	1.856761	1.582896	1.75096	2.2E-15	0.925971569	0.356671467	-0.153739189
ENSG00000162437	RAVER2	2.117388	2.153082	2.051356	1.958843	1.864165	1.884941	1.684271	1.730146	1.897062	0.99775749	-0.000914542	0.329294852	-0.15363835
ENSG00000102100	SLC35A2	13.819907	10.98092	11.738292	10.355954	10.895788	9.751424	10.46776	10.901571	11.308013	0.518978633	-0.084577691	0.183129794	-0.153619065
ENSG00000169087	HSPBAP1	5.280704	5.298883	5.799623	4.394917	4.031908	3.200794	5.083948	4.146072	4.591426	0.031753208	-0.349434292	0.322558022	-0.153474248
NewGene_4680	--	10.892333	4.751153	4.247533	4.911475	3.776312	4.395767	4.409182	4.85574	7.601632	0.464210524	-0.457934799	0.814770309	-0.153416864
ENSG00000111670	GNPTAB	7.080402	7.753505	7.048289	5.317375	5.924225	5.603546	5.17431	6.168905	6.980349	0.007953819	-0.236886304	0.183947183	-0.153390318
ENSG00000115808	STRN	2.018364	1.897077	2.0603	2.038301	2.091712	2.298985	1.819799	1.544397	1.681483	0.028171234	0.254214823	0.230280424	-0.153353221
ENSG00000115687	PASK	4.781657	4.871837	4.753969	4.452745	3.935119	3.675178	4.119013	3.886894	3.986942	0.456155506	-0.095887133	0.154924885	-0.153286127
ENSG00000131408	NR1H2	16.139607	15.199796	14.438945	13.092183	13.81305	15.145843	12.137585	11.714684	15.091043	0.988692795	0.002197605	0.171738974	-0.153252867
ENSG00000145780	FEM1C	4.679005	4.400362	5.250272	5.191646	4.667348	5.047573	4.211326	3.778569	4.101445	0.047485091	0.204152776	0.166739367	-0.1531203
ENSG00000070018	LRP6	10.159082	11.255454	10.780898	9.525026	9.536625	8.736926	9.592574	8.886874	10.388572	0.306000773	-0.082057764	0.039870685	-0.15302681
ENSG00000151276	MAGI1	6.101653	6.238278	6.17431	5.225845	5.635761	5.10694	5.163882	4.65768	5.593952	0.844727866	-0.023839282	0.110683425	-0.15289002
ENSG00000169246	NPIPB3	12.201929	14.568526	16.876973	14.452814	10.695403	12.296685	13.081168	12.112784	12.594204	0.620817749	-0.091654498	0.300003011	-0.152820615
ENSG00000106785	TRIM14	2.169947	2.142932	2.505526	10.326917	9.20611	9.931149	2.841724	2.280382	1.533241	2.73E-139	2.251975247	0.854855373	-0.152796927
ENSG00000203883	SOX18	3.62627	2.768012	2.806919	11.314084	11.50361	13.57962	2.70942	2.450987	2.610201	3.07E-61	2.128963977	0.476686075	-0.152784959
ENSG00000183032	SLC25A21	0.7009	0.616484	0.774851	0.499105	0.481982	0.605479	0.577624	0.50747	0.689513	0.52240999	-0.247740896	0.662661962	-0.15277017
ENSG00000112655	PTK7	22.831463	22.17822	20.542197	16.3842	16.45409	17.290899	17.954884	17.411955	21.160246	0.001161707	-0.229239498	0.061255144	-0.152747568
ENSG00000112167	SAYSD1	10.392198	9.579039	8.596876	11.727218	11.837998	12.208979	7.72985	7.573721	8.708392	0.00000249	0.474654275	0.224154276	-0.152746591
ENSG00000138670	RASGEF1B	6.10253	6.773264	6.533972	4.001501	4.798189	4.042662	5.24086	4.566502	5.937569	0.0000328	-0.493001357	0.220349856	-0.152663145
ENSG00000100722	ZC3H14	16.516647	17.828069	16.612845	14.912614	15.671545	17.045089	13.623001	8.530332	6.713031	0.02728085	0.152063959	0.055677144	-0.15265138
ENSG00000155380	SLC16A1	20.655073	20.862818	21.558942	17.890292	16.274482	16.892981	16.955103	17.118539	19.2714	0.012636758	-0.171139885	0.044349226	-0.152634063
ENSG00000107863	ARHGAP21	10.703681	10.761912	10.962727	13.584547	13.016355	16.257948	9.060636	8.185831	10.215582	1E-12	0.42445408	0.051332125	-0.152478089
ENSG00000114786	ABHD14A-ACY1	6.271386	5.070873	3.680467	0.633889	2.962551	1.242591	1.118202	1.037186	10.214649	0.112293678	-1.186233997	0.905630163	-0.152435646
NewGene_5008	--	1.429268	1.242744	1.454913	0.943044	1.112178	0.911147	1.101233	1.03205	1.115705	0.327390185	-0.282735147	0.565523076	-0.15229494
ENSG00000178694	NSUN3	2.664153	3.256296	3.177822	3.07573	3.044829	3.158037	2.559107	2.168396	2.891937	0.283017423	0.173803287	0.374754603	-0.152177701

Gene ID	Gene name	Ctrl-1 FPKM	Ctrl-2 FPKM	Ctrl-3 FPKM	IFNγ-1 FPKM	IFNγ-2 FPKM	IFNγ-3 FPKM	Sora-1 FPKM	Sora-2 FPKM	Sora-3 FPKM	Ctrl vs IFNγ DESeq2 FDR	Ctrl vs IFNγ DESeq2 log2FC	Ctrl vs Sora DESeq2 FDR	Ctrl vs Sora DESeq2 log2FC
ENSG00000228486	C2orf92	1.462943	1.519226	1.321085	1.146196	1.651082	1.121867	1.144467	1.003442	1.174236	0.965839989	0.025299104	0.720169021	-0.152146657
ENSG00000205572	SERF1B	2.737277	3.136314	3.023094	1.88899	1.937884	2.318525	3.261233	3.322764	2.561274	0.095363276	-0.469489598	0.588434512	-0.152110956
ENSG00000197724	PHF2	10.948172	10.321953	10.723158	9.660369	9.100132	9.263147	9.054235	8.0028	10.005968	0.629387241	-0.044164796	0.082953306	-0.152053313
NewGene_3477	--	17.741267	38.47724	34.331011	25.184059	25.101014	26.844413	34.811738	12.225597	32.120702	0.0000073	-0.368876131	0.1031957	-0.152048493
ENSG00000162881	OXER1	2.156382	2.993182	2.680804	2.669005	2.283241	2.794377	2.532943	1.900505	2.164065	0.615427513	0.133496431	0.535275203	-0.151929204
ENSG00000174749	FAM241A	0.29673	0.392461	0.420517	0.987334	0.93531	0.922113	0.295495	0.294888	0.34474	4.49E-19	1.50436219	0.585091593	-0.151868796
ENSG00000168411	RFWD3	14.366261	15.950075	16.615843	14.563366	13.839444	14.019541	13.642761	12.60611	13.952596	0.8834915	-0.015677852	0.057680612	-0.151861594
ENSG00000086504	MRPL28	55.693671	51.446561	49.297434	48.602985	51.254707	53.838232	41.138925	43.103225	48.750336	0.321194042	0.09367943	0.097584065	-0.151792743
ENSG00000203705	TATDN3	6.37427	8.46463	8.339994	6.294163	6.160799	5.76015	6.761894	6.418503	6.084906	0.197472764	-0.184485619	0.287080028	-0.151790054
ENSG00000198081	ZBTB14	3.641792	3.854374	4.751348	4.11915	4.104477	4.063833	4.124731	3.273659	3.279321	0.529354555	0.101525138	0.303069092	-0.151711142
ENSG00000153107	ANAPC1	15.487337	16.294907	15.827487	14.473329	12.763775	12.94159	13.760211	13.034769	13.370649	0.162234389	-0.099063737	0.023725886	-0.151582905
ENSG00000073331	ALPK1	1.531514	1.764421	1.741886	5.296127	5.171811	5.295399	1.237158	1.143626	1.809594	8.05E-71	1.864247221	0.438232018	-0.151248008
ENSG00000214756	CSKMT	0.987705	0.635025	0.494284	0.610823	0.73392	0.648865	0.608283	0.59424	0.596616	0.925199945	0.05354537	0.732579591	-0.151153895
ENSG00000250571	GLI4	5.077007	6.426762	4.823184	5.056233	5.177836	5.514303	3.960314	4.743963	4.88662	0.633451195	0.112079639	0.47848857	-0.151081611
ENSG00000204138	PHACTR4	16.861046	14.08667	11.775696	12.384444	12.118661	11.479648	13.697453	10.555838	11.680145	0.78487707	0.026003386	0.065928811	-0.151073344
ENSG00000136816	TOR1B	14.166019	13.071258	12.207504	10.386144	11.067219	10.960582	10.268546	10.255593	12.907791	0.18929469	-0.135229833	0.176079565	-0.151029921
ENSG00000197608	ZNF841	3.053512	2.865137	3.192828	3.57444	3.613604	2.583565	2.535069	2.635058	2.542995	0.257622492	0.176936614	0.323042271	-0.150942454
ENSG00000171729	TMEM51	7.421987	6.278849	7.253908	6.973216	7.379784	6.580587	6.143571	5.622983	5.948915	0.315679732	0.148500832	0.295523667	-0.150848759
ENSG00000205765	C5orf51	12.303814	13.39623	13.147939	13.081276	13.31936	13.762418	10.785915	10.928081	11.08626	0.005284624	0.196061746	0.054442775	-0.150743756
ENSG00000140259	MFAP1	24.195072	23.140429	24.164118	22.618208	22.47827	21.479288	19.21472	21.291914	19.813972	0.646768043	0.04387116	0.100106976	-0.15072673
ENSG00000213551	DNAJC9	19.297447	18.472204	17.963921	16.356083	14.820085	15.348547	15.720132	14.928127	16.476215	0.165495205	-0.114059902	0.057331553	-0.15063906
ENSG00000138738	PRDM5	3.147431	3.499331	3.87068	4.219493	3.245919	3.485941	3.27499	3.283219	4.031906	0.446766024	0.120122281	0.274652718	-0.150295846
ENSG00000166484	MAPK7	5.250282	4.205412	4.914477	3.949042	4.853561	4.411758	4.047348	3.677418	4.56988	0.887836101	0.027485389	0.28856139	-0.150206879
ENSG00000178234	GALNT11	10.788626	10.026695	10.473105	8.409603	7.726311	7.764091	9.212091	7.914928	9.267218	0.004304518	-0.276615384	0.16651444	-0.150125857
ENSG00000145391	SETD7	6.384751	7.563472	7.407806	6.432996	6.120604	6.124879	7.036265	5.878399	6.682285	0.646768043	-0.051870744	0.123823531	-0.150025809
ENSG00000137814	HAUS2	13.462237	15.773525	14.099506	11.499883	11.291033	12.227981	13.768598	11.816038	13.41106	0.016697264	-0.210377089	0.119458364	-0.149776004
ENSG00000106028	SSBP1	107.949643	122.649173	107.134136	111.172841	94.086573	100.455944	101.586798	94.806161	88.775716	0.977775655	0.003641432	0.10548289	-0.149679324
ENSG00000137815	RTF1	17.517976	17.996733	17.784526	16.79896	15.831325	16.416452	15.081581	14.103643	15.924558	0.727258781	0.028782239	0.031191003	-0.149591845
ENSG00000133135	RNF128	7.340071	7.985234	6.49659	8.615485	8.750963	7.679079	6.209439	5.854511	6.217778	0.001665728	0.348489376	0.223059292	-0.149559087
ENSG00000172785	CBWD1	37.909537	36.269722	40.117537	27.399978	31.743281	26.452256	30.924127	30.259725	34.262844	0.010798193	-0.252912374	0.064039659	-0.149520898
ENSG00000137496	IL18BP	2.772682	2.833841	2.800626	25.865458	24.76981	28.320802	1.990351	2.210334	2.731705	1.23E-276	2.904247964	0.416212287	-0.149516494
ENSG00000130826	DKC1	50.833101	52.733284	55.39725	44.352655	41.348382	44.396947	42.37477	42.954663	47.517616	0.014069187	-0.152002752	0.03477003	-0.149462157
ENSG00000100749	VRK1	12.478388	12.863735	14.425439	12.953941	10.1701	10.913539	11.447483	11.4692	10.492748	0.604229725	-0.076821743	0.216779194	-0.14918459
ENSG00000134057	CCNB1	67.490662	70.799713	71.338783	50.221584	44.63343	50.834225	59.606974	60.572237	62.873414	3.01E-10	-0.384273011	0.025238096	-0.149177929
ENSG00000132612	VPS4A	15.572886	14.976775	13.026016	14.367716	13.11084	14.353993	12.277078	11.969729	12.610226	0.379869368	0.088060159	0.103025048	-0.14912973
ENSG00000162441	LZIC	11.328477	11.859355	11.449381	8.12106	8.400545	9.460506	10.783264	9.86444	10.123323	0.003343064	-0.290637119	0.181877971	-0.148981154
ENSG00000111145	ELK3	5.797701	5.222201	4.939812	4.229609	3.5734	4.172114	4.12762	4.342117	4.961364	0.016823269	-0.283168591	0.223288596	-0.148934698
ENSG00000152291	TGOLN2	63.2903	63.207743	67.802745	48.404435	45.927363	46.977048	52.872171	52.051504	58.420617	1.35E-10	-0.318117287	0.01851971	-0.148897039
ENSG00000108784	NAGLU	40.226109	39.375802	37.12774	34.104626	34.794007	35.712512	32.1476	31.44602	36.395194	0.655828717	-0.040223881	0.068290902	-0.148711579
ENSG00000106144	CASP2	11.055232	11.03444	12.07479	12.002072	11.405254	11.207322	10.141724	8.883616	10.090388	0.042120609	0.163207547	0.098685871	-0.148586697
ENSG00000153993	SEMA3D	2.295163	2.893056	2.913306	1.241338	1.240697	1.342696	2.289239	2.112971	2.516217	2.52E-11	-0.883551874	0.309510735	-0.148576617
ENSG00000132286	TIMM10B	17.170083	17.301996	16.339897	15.786458	15.273485	15.376947	14.356961	14.117671	14.927931	0.903524855	0.013170378	0.068178482	-0.148568175
ENSG00000133193	FAM104A	15.98626	15.537435	17.134533	14.289937	14.545325	15.38496	13.220134	13.573294	14.610089	0.98194363	0.002959533	0.094201969	-0.148508482
ENSG00000131023	LATS1	5.141589	5.455261	5.884894	6.168945	5.894952	6.154874	4.83282	4.548429	4.881236	0.000275401	0.29163131	0.116626979	-0.148459508
NewGene_3531	--	6.933475	6.237313	6.005639	6.91766	6.360234	6.00914	5.131466	4.50375	6.660265	0.179060508	0.154576309	0.295551281	-0.148452507
NewGene_2412	--	2.106202	1.596902	2.853121	2.157533	2.56453	2.693356	1.817132	2.068056	1.774782	0.099752163	0.372722158	0.543802249	-0.148405463
ENSG00000126214	KLC1	19.271808	16.698489	16.131407	14.951296	13.160225	12.183197	17.196699	16.360952	17.985604	0.360943321	-0.078532033	0.094561782	-0.148404436
ENSG00000163935	SFMBT1	4.368069	4.330768	4.659293	4.470853	4.191976	4.486673	3.798814	3.315416	4.290625	0.340265695	0.114044861	0.229566739	-0.148201842
ENSG00000070950	RAD18	7.287381	8.715489	8.301316	6.974393	6.463239	6.958986	6.905449	6.365245	7.17496	0.237505529	-0.114784542	0.111954732	-0.148089745
ENSG00000112282	MED23	8.737671	9.667654	9.541619	9.269085	9.065412	8.20217	7.258421	7.017031	9.020863	0.392153048	0.080784227	0.148509961	-0.147989824
ENSG00000161813	LARP4	18.725462	20.862948	22.750869	19.993506	18.345828	19.053438	17.713027	16.873305	18.472714	0.74013353	0.038386054	0.116325674	-0.147964692
ENSG00000167394	ZNF668	5.516523	5.203026	4.878044	5.404186	5.430735	5.513498	4.137788	3.526132	5.581919	0.074959035	0.232151446	0.376312113	-0.14790875
ENSG00000136720	HS6ST1	14.026429	13.517076	12.422883	13.352505	13.862138	14.077442	11.84304	9.997272	12.054699	0.01567491	0.194401523	0.116233374	-0.147760603
ENSG00000161904	LEMD2	27.450098	26.759037	26.032042	26.757143	25.342305	28.616756	22.380823	20.284975	23.742757	0.102400477	0.129370986	0.051918767	-0.147715736
ENSG00000129472	RAB2B	8.48634	8.77996	9.31559	10.428328	9.688273	9.213493	7.535813	7.711325	7.265334	0.001691375	0.289000345	0.174190548	-0.147698891
ENSG00000114279	FGF12	1.042722	1.249905	1.486171	0.558619	0.456639	0.612977	1.022499	1.132385	1.185371	0.0000327	-0.969662972	0.541312604	-0.147608198
ENSG00000138614	INTS14	24.593308	25.347936	26.633001	22.285172	19.589839	20.228287	21.010964	21.618418	21.694192	0.040662741	-0.159548204	0.060422172	-0.147602483
ENSG00000100109	TFIP11	15.364575	12.924069	13.185046	14.020125	13.126555	13.599992	11.92217	11.054324	12.512736	0.212292961	0.119412928	0.132422541	-0.147550993
ENSG00000135766	EGLN1	17.449781	18.795139	20.197269	14.77594	15.409296	14.630506	16.116627	14.696146	17.024179	0.010101198	-0.184869185	0.060581703	-0.147496247
ENSG00000078237	TIGAR	4.969956	5.81003	6.120991	5.100973	5.057639	4.48567	4.618373	4.319884	5.407191	0.725704869	-0.059833061	0.299520938	-0.147332988
ENSG00000198075	SULT1C4	14.884515	14.168137	16.318576	6.309764	6.941152	5.348473	13.524506	13.392598	12.923427	3.14E-27	-1.16926767	0.183045451	-0.147241276
ENSG00000072840	EVC	2.106124	2.135895	1.679335	2.036409	1.753378	2.178054	1.4928	1.570592	1.949284	0.318935774	0.162430782	0.368778833	-0.147162649
ENSG00000157212	PAXIP1	12.415035	11.77663	12.618928	12.302121	11.30987	10.996914	9.955753	9.554732	11.687174	0.558724427	0.057264992	0.109642267	-0.147162385
ENSG00000196865	NHLRC2	3.444135	4.183714	4.227914	3.778715	3.616163	3.68573	3.536367	2.902731	3.617802	0.682473236	0.050438984	0.195007143	-0.147107375
ENSG00000160963	COL26A1	3.332088	3.119752	2.515891	1.979737	2.079153	2.47415	2.557264	2.512626	2.548209	0.095193414	-0.310667641	0.405702636	-0.147087341
ENSG00000032742	IFT88	1.997803	2.680714	2.630883	2.159594	1.668503	1.541964	2.084541	1.806427	2.445019	0.230928018	-0.267598296	0.465084879	-0.147076537
ENSG00000023330	ALAS1	29.930215	25.376969	28.689352	29.626973	30.440936	28.450308	23.865025	24.298909	22.876137	0.012226729	0.210559652	0.120488874	-0.146987627
ENSG00000182986	ZNF320	1.458878	4.576465	4.767809	6.184944	3.486919	3.419166	3.077729	2.764466	3.463042	0.781743957	0.421441571	0.911553655	-0.146489792
ENSG00000172175	MALT1	6.298005	5.979272	6.168504	7.540306	6.326266	6.47327	4.523607	4.524765	5.644274	0.000169	0.28907654	0.097735181	-0.146461231
ENSG00000008300	CELSR3	0.834054	0.818903	0.765804	0.825522	0.642665	0.700744	0.656401	0.627447	0.772191	0.959416255	-0.011974494	0.357163962	-0.146386825
ENSG00000115361	ACADL	0.174093	0.546057	0.492407	0.453407	0.216983	0.412191	0.322871	0.367471	0.33838	0.982925209	-0.017216193	0.795870045	-0.146312357
ENSG00000133740	E2F5	17.956532	17.104853	19.810732	16.571023	14.939766	16.451481	13.631903	14.262204	16.101069	0.752665378	-0.035061053	0.141157054	-0.146297812
ENSG00000186501	TMEM222	21.451718	19.139184	20.916811	18.521546	16.862232	17.288758	15.579511	17.629405	17.457016	0.317692029	-0.107521106	0.182526039	-0.146266426
ENSG00000213281	NRAS	26.277431	30.106064	29.287817	25.178028	24.402653	23.56539	24.234812	23.343868	25.02063	0.310333492	-0.080612907	0.051710132	-0.146198901

Gene ID	Gene name	Ctrl-1 FPKM	Ctrl-2 FPKM	Ctrl-3 FPKM	IFNγ-1 FPKM	IFNγ-2 FPKM	IFNγ-3 FPKM	Sora-1 FPKM	Sora-2 FPKM	Sora-3 FPKM	Ctrl vs IFNγ DESeq2 FDR	Ctrl vs IFNγ DESeq2 log2FC	Ctrl vs Sora DESeq2 FDR	Ctrl vs Sora DESeq2 log2FC
ENSG00000093009	CDC45	18.618135	19.316383	19.900886	16.997237	14.528434	15.784238	16.109716	16.546955	17.10166	0.116520751	-0.147517955	0.106216853	-0.14618206
ENSG00000157429	ZNF19	1.179346	1.10508	0.876836	0.747051	1.158057	1.176265	0.862017	1.058258	1.01549	0.938840046	-0.032008487	0.632439683	-0.146111399
ENSG00000157502	MUM1L1	3.622117	3.781188	3.574608	3.315537	2.67646	2.752544	3.386652	2.81943	3.026947	0.188342566	-0.182684667	0.265375119	-0.145955565
ENSG00000169992	NLGN2	4.329825	3.681015	3.754073	4.245075	3.34414	3.68828	3.134997	3.311451	3.58941	0.598604664	0.08235841	0.259427416	-0.145933007
ENSG00000110931	CAMKK2	12.120645	12.530829	12.231126	11.009034	12.770986	11.117141	12.186109	10.676615	11.231303	0.975527107	0.003569935	0.060425445	-0.145800657
ENSG00000116991	SIPA1L2	3.642379	2.697175	4.04476	0.497389	0.639354	0.565452	2.054286	2.627339	3.254996	1.62E-58	-2.129898449	0.29187541	-0.145715175
ENSG00000186446	ZNF501	0.763423	0.733224	0.853338	0.774884	0.738987	0.744204	0.565068	0.774658	0.645617	0.780131293	0.09633813	0.639033375	-0.145708634
ENSG00000113851	CRBN	24.106601	24.267284	22.95993	25.102117	25.557935	24.837873	20.69191	19.732387	19.936178	0.001162497	0.227316703	0.073296491	-0.145638782
ENSG00000109576	AADAT	4.678539	4.208563	5.245384	4.185026	2.988337	3.270836	4.396243	3.643694	4.04339	0.130413823	-0.274207556	0.383088342	-0.145619833
ENSG00000152382	TADA1	16.619654	18.258276	16.261642	16.860453	16.56992	15.507796	15.08699	14.532911	13.664957	0.417328491	0.083664001	0.155977186	-0.145596067
ENSG00000047410	TPR	33.490275	34.634945	35.623258	34.092168	30.895444	29.616421	28.160295	28.598537	30.823464	0.833013023	0.018287517	0.021692647	-0.145549494
ENSG00000135392	DNAJC14	19.776561	19.924396	19.174049	18.626785	16.667999	17.694623	15.539807	16.151202	17.332316	0.907862454	-0.013436658	0.067943169	-0.14553114
ENSG00000156970	BUB1B	55.812791	57.743818	62.641969	38.990263	35.693348	35.633923	50.299403	48.782199	50.361569	3.01E-19	-0.528564826	0.035912856	-0.145517041
ENSG00000026297	RNASET2	6.619601	5.595703	4.5531	5.268541	5.110666	5.344646	4.963025	3.997807	6.08816	0.937751074	-0.020454276	0.499911982	-0.145504441
ENSG00000144647	POMGNT2	5.183908	5.185035	4.899413	3.713606	3.978492	3.849402	4.040503	4.259244	4.657568	0.057121367	-0.254816333	0.289727257	-0.145477447
ENSG00000123485	HJURP	23.036306	24.783234	23.410144	19.693754	16.679289	17.558872	21.956847	20.325681	18.166487	0.000434976	-0.263355454	0.14571483	-0.145427171
ENSG00000188611	ASAH2	0.721692	0.866491	0.907855	0.75024	0.715334	0.760001	0.59683	0.731139	0.693859	0.998487969	-0.000849674	0.499025816	-0.145383314
ENSG00000113719	ERGIC1	47.906433	43.421693	42.439707	40.308069	39.164559	40.701196	37.721162	36.57026	39.188135	0.936052205	-0.008376202	0.050372125	-0.145325473
ENSG00000172071	EIF2AK3	6.329643	5.768302	6.348832	7.082035	6.602542	6.855122	5.268955	4.802045	5.585776	0.000453196	0.302725209	0.159618594	-0.145294698
ENSG00000107815	TWNK	12.337096	11.696151	12.914417	11.361275	10.698004	10.148229	10.264462	9.745432	11.331052	0.033174775	-0.186411836	0.119725527	-0.145244481
ENSG00000007376	RPUSD1	10.008477	8.619429	9.011527	11.460295	8.981068	9.433805	7.595328	7.844148	8.78705	0.044968858	0.230751791	0.254337172	-0.145238903
ENSG00000197140	ADAM32	0.513983	0.466797	0.58156	0.829561	0.674386	0.636388	0.441102	0.454355	0.265935	0.081991954	0.634668989	0.761739684	-0.145108275
ENSG00000086991	NOX4	0.375933	0.764648	0.47603	0.448646	0.377116	0.40194	0.721314	0.285666	0.380948	0.937715405	0.047905559	0.780148905	-0.145107375
ENSG00000131475	VPS25	101.837338	99.602998	93.38059	83.648356	82.680431	87.537933	84.638449	85.582871	80.250657	0.355858472	-0.070963967	0.070610051	-0.145074124
NewGene_607	--	5.382391	6.426713	6.474922	6.576337	6.139367	5.968429	6.222765	5.047826	5.853306	0.256125631	0.12997618	0.204039839	-0.14501302
ENSG00000089094	KDM2B	15.67256	12.983909	13.919986	12.356379	11.44358	11.726931	11.367697	10.103711	12.492874	0.949299904	-0.007850142	0.099843113	-0.144861636
ENSG00000001084	GCLC	11.988631	13.250125	13.12918	10.665741	9.60504	9.841416	10.899136	10.769576	11.038917	0.011739053	-0.206822397	0.095272541	-0.14472392
ENSG00000181061	HIGD1A	72.199895	73.222644	75.851396	67.278877	63.879113	67.732602	61.086808	62.913444	64.543038	0.838533256	-0.01753995	0.036516343	-0.14463012
ENSG00000116863	ADPRHL2	10.925691	10.252867	8.836591	10.762237	10.96059	11.271883	7.466859	8.604844	9.394223	0.016626522	0.284577581	0.31423644	-0.14459479
ENSG00000153975	ZUP1	9.434606	10.353632	11.193012	9.546371	9.91675	10.155022	9.247144	8.803425	8.186253	0.520733747	0.083469929	0.243710671	-0.144581372
ENSG00000068654	POLR1A	8.561069	8.727535	8.289629	8.574815	7.214684	8.308918	6.98098	6.889032	7.873276	0.5813441	0.051283836	0.04464485	-0.144550134
ENSG00000198624	CCDC69	3.577743	3.477815	3.296687	9.90124	9.714047	9.386359	2.863231	3.004268	2.911304	4.9E-78	1.628086396	0.3124865	-0.144434615
ENSG00000104228	TRIM35	4.852426	4.890693	4.660205	4.756802	4.795631	4.677501	4.213715	3.729817	4.298844	0.229198915	0.133581266	0.203049069	-0.144418527
ENSG00000067533	RRP15	6.431594	8.194951	8.646688	6.391623	5.853971	6.52275	6.467548	6.476798	6.791857	0.157145156	-0.162101663	0.200306673	-0.144399882
ENSG00000006831	ADIPOR2	48.928089	46.746391	49.520752	42.923233	43.945366	42.462471	41.229786	39.530048	42.483398	0.805624388	-0.019530729	0.020344809	-0.144387282
ENSG00000196396	PTPN1	13.318038	13.421888	12.751123	12.274965	12.06985	12.941984	11.529737	10.360757	11.663699	0.475914982	0.064300068	0.077093348	-0.144372647
ENSG00000065457	ADAT1	11.531302	10.778726	14.211919	10.779829	8.772787	8.966719	11.462973	10.449857	8.782939	0.249348713	-0.166871705	0.274767301	-0.144238914
ENSG00000165806	CASP7	4.724433	5.605798	4.977788	25.086369	23.580735	23.901067	4.000611	4.013606	4.823298	2.04E-194	2.390025195	0.329976849	-0.144182494
ENSG00000087995	METTL2A	8.822144	8.491339	8.990452	7.533792	7.346141	8.048909	7.883354	7.218785	7.886541	0.68504643	-0.05283649	0.165972804	-0.144084497
ENSG00000155876	RRAGA	55.976585	59.580235	53.387791	51.372189	54.661312	50.826744	46.378666	46.842735	50.170753	0.667574731	0.040651429	0.057589161	-0.144077294
ENSG00000163840	DTX3L	4.334787	4.727015	4.843335	50.954761	46.894429	49.458187	3.879364	3.761438	4.162484	0	3.551271183	0.162600532	-0.144065454
ENSG00000114771	AADAC	1.268825	1.402087	1.128853	0.794518	0.92652	1.001101	1.038307	1.023815	1.130764	0.430110911	-0.290252461	0.669956057	-0.143949042
ENSG00000165688	PMPCA	47.228164	41.79333	43.223948	39.302648	37.011704	40.367862	34.705144	37.489922	40.390314	0.759734822	-0.030431213	0.09061201	-0.143750753
ENSG00000184489	PTP4A3	24.718203	21.694721	22.749042	17.298195	17.745167	17.599118	18.487292	20.516579	21.517656	0.029320488	-0.21799041	0.183757012	-0.143719451
ENSG00000258890	CEP95	17.674904	17.949093	17.439197	17.019822	16.130849	14.67761	14.811392	14.174823	15.443969	0.976845996	-0.003652236	0.067755072	-0.143703115
ENSG00000095485	CWF19L1	14.477905	13.900254	13.331562	12.858262	12.172179	12.61192	12.07961	11.470165	11.573788	0.937715405	-0.010595338	0.156261257	-0.143574172
ENSG00000136108	CKAP2	13.024597	14.828039	14.71611	11.977702	11.347473	11.456077	12.66782	11.711671	12.173944	0.06866153	-0.159031938	0.113841193	-0.143446465
ENSG00000107164	FUBP3	40.228184	40.966518	41.645672	39.241077	38.56414	39.01334	34.047256	32.543869	37.822952	0.23735835	0.074957793	0.036522933	-0.143429222
ENSG00000157450	RNF111	6.123017	6.441077	6.557891	6.616829	5.935585	6.262003	5.913563	4.76531	5.167578	0.485505864	0.076204123	0.186521103	-0.143384266
ENSG00000008869	HEATR5B	4.229925	4.40388	4.172634	3.872226	3.875827	3.837715	3.838218	3.335093	4.077655	0.95551367	-0.007774722	0.169605333	-0.143365273
ENSG00000128731	HERC2	6.386644	5.28074	5.494251	5.768386	4.868701	5.27518	4.883536	4.449543	5.5514	0.761503002	0.036428689	0.171253656	-0.143327021
ENSG00000172466	ZNF24	18.140314	18.83772	18.320951	15.21755	16.980173	16.56879	15.885093	14.494304	16.428146	0.97651393	-0.002986672	0.030518517	-0.143289965
ENSG00000158941	CCAR2	31.439858	28.942823	27.715688	25.32851	21.637179	24.125418	22.798724	22.841849	28.87235	0.005749087	-0.187848353	0.081587744	-0.143118156
ENSG00000114115	RBP1	42.197629	34.099366	30.273416	23.630991	20.883552	23.68018	29.043132	31.664659	28.779994	0.0000221	-0.541011363	0.336525718	-0.142748381
NewGene_2872	--	1.115478	0.512789	0.843871	1.006415	0.79413	1.208396	0.59738	0.431095	0.807243	0.208322652	0.503705572	0.752768288	-0.142739205
ENSG00000155858	LSM11	1.362176	1.56776	1.57322	1.351664	1.282011	1.45883	1.214739	1.187023	1.428036	0.959159752	0.011646569	0.375167251	-0.142692881
NewGene_4414	--	1.08603	1.538548	1.018757	0.958667	1.060479	0.604221	1.283939	1.000958	0.925775	0.373971231	-0.326037542	0.665116608	-0.142670173
ENSG00000184203	PPP1R2	13.631712	15.891604	15.981694	12.961451	13.466648	13.172585	12.709912	11.897901	14.006141	0.685663623	-0.045811771	0.138444389	-0.14264669
ENSG00000007202	KIAA0100	42.471765	38.531848	38.704134	36.04265	34.21709	34.593922	33.064776	32.521005	36.589856	0.441994879	-0.055212684	0.039674371	-0.142583911
ENSG00000120693	SMAD9	2.142596	2.746097	3.153657	1.256217	1.45144	1.333326	2.120769	2.344224	2.370118	2.46E-08	-0.846416349	0.391985928	-0.142299448
ENSG00000105229	PIAS4	19.598238	17.59815	17.595556	16.50424	16.420897	17.041994	15.391961	15.898561	15.226304	0.902295008	0.014022073	0.114635258	-0.1422604
ENSG00000197372	ZNF675	6.038663	6.478186	6.65972	6.41775	5.681433	5.335886	6.170199	5.80446	5.629833	0.920138162	-0.018920435	0.293984625	-0.14220804
ENSG00000188906	LRRK2	0.11313	0.175234	0.171204	0.240721	0.263627	0.198314	0.135953	0.112363	0.122705	0.014891019	0.794551628	0.74570649	-0.142113773
ENSG00000099953	MMP11	8.205278	6.28808	4.951608	3.972972	4.61264	4.394468	6.466343	5.279329	4.975973	0.0115539	-0.449351653	0.477451577	-0.142044915
ENSG00000117411	B4GALT2	18.851142	19.226041	18.015859	15.276706	15.238541	16.110292	14.061709	15.611329	18.508059	0.145825967	-0.135734098	0.155371059	-0.142006418
ENSG00000120008	WDR11	13.706321	13.495165	12.837562	13.293953	13.359911	12.782921	10.350439	11.20382	12.560616	0.096897882	0.125679764	0.116321753	-0.141970481
ENSG00000182534	MXRA7	8.388677	8.380722	10.389178	7.176922	7.581935	6.775859	7.66212	6.84372	9.724058	0.145396041	-0.146066361	0.233543834	-0.141944994
ENSG00000122203	KIAA1191	21.92747	23.184779	22.936749	19.964913	17.333342	18.868801	18.40887	18.838777	20.502756	0.087463603	-0.127339735	0.065441942	-0.141877733
NewGene_3342	--	5.087404	6.046515	4.841257	5.435995	4.672608	5.260909	4.725076	4.344834	4.61946	0.64164323	0.089812216	0.394391375	-0.141746461
ENSG00000184160	ADRA2C	9.095993	7.538594	7.341248	2.783579	3.94201	3.325948	6.177413	6.846782	8.628902	6.57E-19	-1.192379604	0.315904668	-0.141743235
ENSG00000170606	HSPA4	55.293259	55.944225	55.958098	49.486457	50.036907	47.123338	47.280907	46.279022	48.533319	0.530290817	-0.041989012	0.013517503	-0.141602962
ENSG00000172171	TEFM	5.509359	5.953618	5.962386	5.186947	5.079348	5.096883	4.866957	4.632507	5.663349	0.863061552	0.03265196	0.347268665	-0.141528919
ENSG00000101935	AMMECR1	2.674767	2.483882	2.714682	2.537686	1.912553	2.386943	2.155854	2.171091	2.500033	0.741458623	-0.059198149	0.283185945	-0.141252918
ENSG00000105854	PON2	141.44182	156.121601	165.754171	114.140657	105.769473	106.743586	128.600147	121.763618	134.115782	0.000000101	-0.352919994	0.064410212	-0.141250665

Gene ID	Gene name	Ctrl-1 FPKM	Ctrl-2 FPKM	Ctrl-3 FPKM	IFNγ-1 FPKM	IFNγ-2 FPKM	IFNγ-3 FPKM	Sora-1 FPKM	Sora-2 FPKM	Sora-3 FPKM	Ctrl vs IFNγ DESeq2 FDR	Ctrl vs IFNγ DESeq2 log2FC	Ctrl vs Sora DESeq2 FDR	Ctrl vs Sora DESeq2 log2FC
ENSG00000128581	IFT22	7.691753	7.2166	6.250079	5.489884	4.038003	4.488171	6.47958	6.220528	5.966488	0.004746442	-0.479362602	0.405509451	-0.141217009
ENSG00000107771	CCSER2	3.107598	3.052744	2.981412	3.19049	3.213161	3.040257	2.703615	2.625538	2.736117	0.03307227	0.208538328	0.180976699	-0.141163561
NewGene_2110	--	1.58844	1.61417	2.049983	2.111483	1.704922	1.402535	1.415556	1.453481	1.463859	0.772622247	0.0720016	0.473833514	-0.141140122
ENSG00000156983	BRPF1	10.393857	11.616792	11.238848	9.412713	9.022632	9.023588	9.807989	8.705341	9.538613	0.144677005	-0.120331895	0.095122292	-0.14102337
ENSG00000144909	OSBPL11	11.336147	10.715984	11.304584	12.141144	13.00214	12.053926	9.281732	8.995446	10.122795	0.0000453	0.304403165	0.09551131	-0.140919494
ENSG00000154328	NEIL2	1.050879	1.618312	1.42769	1.239149	1.496224	1.147219	1.317572	1.037015	1.329385	0.903733296	0.043647222	0.621529898	-0.14088208
ENSG00000125247	TMTC4	13.516978	12.225103	12.269342	10.580335	10.466216	10.905803	10.80979	10.306815	11.238152	0.262802336	-0.103795033	0.106338743	-0.1408776
ENSG00000161057	PSMC2	57.252263	56.938134	55.096004	52.80528	49.606859	49.824223	47.66399	49.737429	45.089007	0.967524609	-0.003730273	0.068417608	-0.14087531
ENSG00000149311	ATM	3.557377	3.619856	3.639279	3.7381	3.771435	3.559859	3.303267	2.958185	3.303465	0.049515168	0.152763227	0.098212219	-0.140826041
ENSG00000155561	NUP205	36.009501	37.825026	38.840046	33.938216	32.589531	32.375594	30.845688	29.755552	33.815875	0.630990042	-0.033879577	0.027849796	-0.140729526
ENSG00000143499	SMYD2	59.280855	58.936664	59.41413	44.807329	42.508016	44.454309	49.528367	49.992758	51.262959	0.000000948	-0.282709466	0.038965826	-0.140703961
ENSG00000139998	RAB15	6.418851	5.565444	4.801	4.903704	5.199573	4.841692	4.46177	4.588874	5.192293	0.890046226	-0.025050056	0.30878033	-0.140683935
ENSG00000084676	NCOA1	2.496114	1.934568	2.097799	2.888223	2.46245	2.652043	2.117064	1.672189	1.771176	0.000394405	0.431019305	0.365832637	-0.140661195
ENSG00000114573	ATP6V1A	23.395833	25.647849	26.062203	30.784017	31.545094	31.920779	21.892943	19.735544	22.454594	1.35E-15	0.477755564	0.063715723	-0.140600248
ENSG00000105171	POP4	10.210406	8.791143	8.806903	8.412142	9.035519	8.34156	7.782553	8.14847	8.198229	0.889442145	0.021394677	0.214117797	-0.140541778
ENSG00000170445	HARS	25.776023	25.365252	25.067953	22.459506	22.375802	23.667101	21.480964	21.980823	21.303551	0.927540381	0.010351155	0.09098643	-0.140521307
ENSG00000020129	NCDN	10.012326	9.781125	9.552444	7.808811	7.262939	7.22223	7.401643	7.751276	9.450179	0.001349202	-0.276427968	0.195695497	-0.140433655
ENSG00000143727	ACP1	81.438345	85.712128	82.368198	69.665112	71.648697	70.66276	73.384132	72.932336	68.881235	0.093911683	-0.104902364	0.057908644	-0.140430634
ENSG00000124588	NQO2	6.068467	6.866344	5.172894	6.198495	7.788256	6.495029	6.921373	5.017888	4.389111	0.001006335	0.442017825	0.414735956	-0.140294821
ENSG00000259494	MRPL46	26.380627	26.716314	25.498055	25.392933	21.136347	25.932669	23.534317	23.849133	19.254338	0.836110546	0.029953291	0.272872916	-0.140285105
ENSG00000172780	RAB43	15.200952	15.051542	12.812589	18.26771	20.490529	20.234433	11.904662	12.131691	13.398102	4.16E-14	0.599993464	0.130665546	-0.140214151
ENSG00000136146	MED4	13.127157	12.704116	11.150582	10.866842	12.509544	11.646706	11.065472	9.722266	11.154042	0.928866182	0.015019167	0.236661684	-0.140158038
ENSG00000136100	VPS36	3.984967	3.803069	4.121148	3.992891	3.428032	3.331109	3.436684	3.595048	3.024995	0.999202724	-0.00020257	0.309193483	-0.140060624
ENSG00000100994	PYGB	14.228448	13.50475	13.084091	10.568268	10.633833	11.410592	10.672233	11.791557	12.251812	0.032079828	-0.175375589	0.119762566	-0.139843091
ENSG00000173480	ZNF417	4.184537	1.48853	3.360501	3.259424	2.854681	2.724131	2.137539	2.18639	2.201396	0.801752733	0.381996728	0.915088838	-0.139838337
ENSG00000106462	EZH2	33.002362	34.807933	35.120275	31.184023	28.90728	29.357583	30.01126	26.957683	31.070538	0.444235615	-0.062075078	0.072879457	-0.139811643
ENSG00000137944	KYAT3	20.488004	20.308466	21.610669	25.177148	26.249196	24.694881	18.497637	16.932404	18.118851	1.25E-09	0.437464049	0.108151995	-0.139595416
ENSG00000062194	GPBP1	27.475503	28.153783	28.267914	26.477235	25.150239	26.405427	24.974447	23.619111	25.607243	0.704563505	0.033422033	0.049052609	-0.139594581
NewGene_3776	--	1.641892	1.586151	1.921846	0.933875	0.986252	1.023772	1.763803	1.333651	1.428185	0.220347666	-0.39614449	0.667163955	-0.139453776
ENSG00000273841	TAF9	42.187659	41.625642	46.174087	38.685481	35.597844	36.981261	36.872901	37.903182	35.72502	0.596780401	-0.053074484	0.137610197	-0.139418706
ENSG00000088356	PDRG1	13.714091	13.840339	13.206569	9.583568	9.694271	10.61868	11.126327	11.681413	11.882757	0.002005392	-0.298202659	0.169454145	-0.139334548
ENSG00000100567	PSMA3	76.994793	77.761105	82.570209	112.651889	111.451826	111.435325	67.545612	70.938818	63.510994	2.24E-31	0.653160773	0.105326047	-0.139302621
ENSG00000139645	ANKRD52	7.864636	7.690697	6.981255	7.869428	7.239012	7.653291	6.261115	6.142235	6.805757	0.037160814	0.161149367	0.092924204	-0.139080704
ENSG00000170264	FAM161A	2.454245	2.268398	2.363808	2.206199	2.133004	2.251108	2.128385	1.774868	2.163129	0.876472148	0.032680043	0.403958054	-0.139044459
ENSG00000114626	ABTB1	0.438591	0.967737	0.688615	0.471424	0.477374	0.713337	0.576894	0.561483	0.596896	0.86382996	-0.101361045	0.769517184	-0.13889568
ENSG00000130770	ATP5IF1	25.584102	23.230369	23.829434	21.041461	21.750473	21.31082	19.557038	19.51552	17.785864	0.63769093	-0.054577868	0.158199167	-0.138815609
ENSG00000162804	SNED1	0.237861	0.230201	0.253341	0.385013	0.329635	0.245582	0.159873	0.204819	0.249846	0.190663999	0.426000864	0.698185267	-0.138804553
ENSG00000013619	MAMLD1	5.393555	5.710016	6.414465	3.982537	3.550859	3.760843	5.05832	4.420911	5.711199	0.000000871	-0.481717215	0.24006421	-0.138799328
ENSG00000160336	ZNF761	4.761504	5.398804	5.529919	4.992118	3.997052	4.193343	4.708298	4.173342	4.598417	0.519475561	-0.092847281	0.249729634	-0.138616109
ENSG00000171262	FAM98B	16.04867	17.716372	17.472412	14.624195	15.099876	14.453747	14.286088	14.033645	15.813259	0.440881865	-0.066298748	0.078567807	-0.138448343
ENSG00000178927	CYBC1	25.290171	20.514483	20.840789	20.661604	20.638438	20.263434	18.699426	18.640156	21.639414	0.962563224	0.007055157	0.190515379	-0.138386227
ENSG00000111530	CAND1	20.688042	22.597118	21.864608	23.617522	22.942953	22.149981	21.487238	20.061613	21.979675	0.0000809	0.213049862	0.031473821	-0.138375584
ENSG00000153896	ZNF599	0.644328	1.537651	0.836345	1.093281	0.745831	0.966846	1.032478	0.977042	1.004909	0.72293957	-0.151528107	0.699598927	-0.138300361
ENSG00000108179	PPIF	49.449381	50.888712	50.840209	39.105183	37.491022	40.625367	41.161977	40.553181	47.174828	0.000298694	-0.220476469	0.05807368	-0.138257677
ENSG00000096063	SRPK1	41.978797	43.807591	45.95135	39.76375	35.833119	36.274541	38.291511	36.685354	37.371071	0.21071086	-0.087236499	0.048340534	-0.138125124
ENSG00000086827	ZW10	8.199799	7.546526	7.715691	6.0316	6.330767	6.184666	7.127581	6.173488	6.610892	0.093011858	-0.183390836	0.225966777	-0.138099042
ENSG00000173674	EIF1AX	22.168085	25.451517	26.898926	24.45636	21.543364	23.838308	20.969868	20.399303	22.054292	0.479190302	0.064095015	0.083153819	-0.138098421
ENSG00000109762	SNX25	5.387322	4.163455	4.738898	3.8471	4.300441	3.71056	4.001924	3.6867	4.332382	0.503710866	-0.112039193	0.342615023	-0.138040419
NewGene_2302	--	2.061543	1.616421	1.750376	0.998024	1.0526	0.895738	1.917909	1.247817	1.668576	0.002061671	-0.837632434	0.627990852	-0.138013815
ENSG00000058729	RIOK2	5.362734	4.833805	5.523623	5.396668	4.782113	5.173275	5.915322	5.179729	4.926002	0.658303529	0.061581311	0.293905289	-0.137926554
ENSG00000197863	ZNF790	1.124221	0.940675	1.396096	0.918573	0.959942	1.040556	0.83599	0.902367	0.940922	0.827672643	-0.080129211	0.646140517	-0.137877936
ENSG00000180891	CUEDC1	6.653316	5.48933	5.409396	4.95432	4.651806	4.737686	4.438615	4.657313	5.583884	0.30832416	-0.142307244	0.328367891	-0.137853504
ENSG00000075391	RASAL2	4.054704	3.908577	4.025204	3.130588	2.887741	3.147835	5.416087	4.066894	4.29138	0.00000134	-0.369919312	0.25053266	-0.137571752
ENSG00000259417	CTXND1	0.717653	0.724602	0.834819	0.338941	0.523565	0.405089	0.637655	0.600806	0.703118	0.002212306	-0.692089581	0.518012703	-0.137439807
ENSG00000175115	PACS1	27.066302	27.234049	26.98351	20.379996	19.804449	20.171124	22.328113	22.084455	25.863135	0.000000203	-0.283726539	0.058577003	-0.137393644
ENSG00000147526	TACC1	34.242389	31.407265	34.966002	30.397568	30.795304	27.596117	27.746935	28.58449	29.938643	0.25743502	-0.079321429	0.048795998	-0.137378788
ENSG00000107362	ABHD17B	12.912407	12.243651	12.744733	13.058996	14.581795	12.571341	11.620515	10.026576	11.338117	0.018120597	0.220706377	0.19575864	-0.137308421
ENSG00000079785	DDX1	36.932249	36.786129	37.678349	35.22477	33.204342	33.613825	31.918833	32.617823	30.308313	0.788556925	0.022111099	0.081918611	-0.137208203
ENSG00000140650	PMM2	20.082391	19.840024	22.658083	21.292504	18.447678	20.966998	17.592635	16.911589	19.359965	0.297756887	0.102554487	0.145241197	-0.137028764
ENSG00000008517	IL32	8.393483	7.082414	5.565239	36.156832	31.167238	34.0298	6.187322	5.918447	6.0816	6.09E-81	2.327763685	0.518849551	-0.136991909
ENSG00000168924	LETM1	18.044788	18.689716	18.516197	17.814171	16.541729	17.96767	15.321442	14.549884	17.318909	0.342377976	0.069038021	0.065069739	-0.136937901
ENSG00000170473	PYM1	47.176858	41.924878	42.005157	45.067182	47.839803	47.87599	35.389209	37.110635	38.647321	0.001259077	0.256518432	0.126015221	-0.136871076
ENSG00000079756	RAPGEF2	3.752569	3.491533	3.756765	2.992364	2.813251	2.492133	3.099639	2.905969	3.189516	0.022497236	-0.235712294	0.177097986	-0.13672405
ENSG00000153147	SMARCA5	24.544279	24.768045	27.540331	18.569279	17.348783	17.811541	20.953554	21.051083	23.603571	5.6E-10	-0.369243362	0.065922314	-0.136657065
ENSG00000112972	HMGCS1	157.059883	152.389869	142.630525	237.164769	242.56221	235.63527	136.491595	124.02242	125.240203	1.25E-59	0.811892347	0.0562849	-0.13656632
ENSG00000101596	SMCHD1	9.613841	11.212148	10.933058	16.204104	14.433885	14.279971	8.759847	8.593799	9.007237	3.48E-23	0.627958366	0.074363216	-0.136553697
ENSG00000106636	YKT6	78.0532	73.404371	71.41614	70.331091	69.018834	73.0898	63.010878	63.405498	65.14585	0.29069717	0.072507923	0.040853485	-0.136425091
ENSG00000003096	KLHL13	0.848121	1.328079	1.113804	0.873482	0.695827	0.861393	1.125973	0.986642	1.001686	0.149881833	-0.346763433	0.574827118	-0.136389358
ENSG00000078177	N4BP2	11.62595	12.651156	11.814256	14.130102	12.463587	13.204163	10.360801	9.273495	11.28396	0.00000864	0.282924406	0.081177672	-0.136239125
ENSG00000135502	SLC26A10	0.818096	0.841559	0.773883	0.439706	0.391403	0.516083	0.62339	0.499162	0.883396	0.101028338	-0.599990575	0.721230822	-0.136116652
ENSG00000184216	IRAK1	27.504947	26.952375	24.078179	22.70992	21.423186	23.39699	21.631105	22.144481	24.515748	0.422604836	-0.06663876	0.08014625	-0.136089775
ENSG00000241685	ARPC1A	80.731651	80.924095	77.052414	66.653336	68.683838	68.072288	67.804757	68.089073	67.695014	0.22306422	-0.083163102	0.050605539	-0.13594705
ENSG00000183323	CCDC125	8.469775	8.1192	8.46523	11.257605	10.595156	10.870269	7.408696	7.334069	8.140849	1.53E-11	0.505874633	0.155977186	-0.13588001
ENSG00000121774	KHDRBS1	100.065167	105.265242	106.925125	92.64201	86.002095	92.168062	91.497413	83.892813	90.874074	0.388844822	-0.056848704	0.031018531	-0.135767723

Gene ID	Gene name	Ctrl-1 FPKM	Ctrl-2 FPKM	Ctrl-3 FPKM	IFNγ-1 FPKM	IFNγ-2 FPKM	IFNγ-3 FPKM	Sora-1 FPKM	Sora-2 FPKM	Sora-3 FPKM	Ctrl vs IFNγ DESeq2 FDR	Ctrl vs IFNγ DESeq2 log2FC	Ctrl vs Sora DESeq2 FDR	Ctrl vs Sora DESeq2 log2FC
ENSG00000118894	EEF2KMT	7.481396	8.527763	7.573311	5.75272	5.566473	5.674791	6.792049	6.451175	7.364625	0.005320696	-0.318799975	0.275349293	-0.135538475
ENSG00000198743	SLC5A3	3.31965	3.379297	3.628774	2.141368	2.606327	2.421887	2.645571	2.983664	3.185612	0.0000884	-0.377158995	0.18166806	-0.135468902
ENSG00000172167	MTBP	5.703364	5.819758	6.3881	5.853916	4.859211	5.018873	5.473311	4.762347	5.202946	0.784678666	-0.043756578	0.271302638	-0.135427198
ENSG00000258289	CHURC1	7.564429	8.445781	8.29039	8.07452	8.193842	7.861729	7.514249	7.928793	6.722809	0.403507831	0.095064013	0.281766618	-0.135334329
ENSG00000254206	NPIPB11	1.289082	1.353758	1.571539	1.089432	1.021738	0.971047	1.19461	0.958775	1.487594	0.17780324	-0.299407248	0.569846645	-0.135161064
ENSG00000100764	PSMC1	21.628279	21.134363	21.274891	20.676701	19.071339	19.685904	18.2164	18.610876	17.796644	0.623313618	0.039139824	0.074077733	-0.135154547
ENSG00000184939	ZFP90	1.345524	1.589201	1.372873	1.232986	1.376551	1.322581	1.41134	1.030503	1.349249	0.813753427	0.056827923	0.506098656	-0.135060465
ENSG00000165660	ABRAXAS2	7.915585	7.891546	9.1556	7.473433	7.878602	7.701025	7.504386	6.509224	7.321835	0.815254044	0.033393771	0.245036132	-0.134981418
ENSG00000085415	SEH1L	12.867196	15.014014	15.19515	12.880617	12.457714	11.772405	12.329811	11.317181	12.877176	0.540077403	-0.069247226	0.172546262	-0.134924326
ENSG00000197619	ZNF615	1.542871	2.680481	2.482225	1.522324	1.361662	1.514843	1.511472	1.198621	1.823937	0.22906746	-0.239044055	0.500447466	-0.134924317
ENSG00000067334	DNTTIP2	12.703312	12.643353	12.981556	12.064238	11.16743	10.819218	11.71132	11.90865	11.948733	0.871685422	-0.016149163	0.069649715	-0.134855147
ENSG00000116157	GPX7	22.090776	20.517776	19.229837	16.762938	17.889704	17.530561	17.975378	16.620455	18.292801	0.417377321	-0.098096629	0.21520357	-0.134752251
ENSG00000170325	PRDM10	3.999569	4.468348	4.840002	4.69267	4.555453	4.192394	3.628105	3.616567	4.567515	0.181806704	0.155132065	0.232173519	-0.134716974
ENSG00000275066	SYNRG	8.704465	9.078832	9.157954	7.934074	7.301623	7.526437	8.215116	7.587713	8.621167	0.226455771	-0.094178856	0.093586608	-0.134491249
ENSG00000154122	ANKH	8.679793	8.32431	8.825153	6.424276	7.090752	6.509845	7.631541	7.482046	9.252472	0.001713674	-0.236793505	0.144657107	-0.134486473
ENSG00000171467	ZNF318	5.74205	5.48747	4.915663	5.507506	5.601819	5.183185	4.860646	4.106386	4.853919	0.077751024	0.161523745	0.185019113	-0.13415806
ENSG00000154803	FLCN	17.254119	16.880715	17.360835	14.778367	14.90578	16.049975	15.304175	14.864884	16.384027	0.837939708	-0.021260974	0.080511783	-0.13412956
ENSG00000118495	PLAGL1	7.287871	7.203277	11.11445	8.221114	6.519667	7.882141	8.112226	7.552759	8.875987	0.886499985	-0.022680848	0.228143356	-0.134114813
ENSG00000108578	BLMH	23.576968	24.099339	24.410071	20.966543	18.779261	19.689342	20.401248	20.778588	20.401607	0.103357678	-0.128456606	0.102228963	-0.13386903
ENSG00000169955	ZNF747	9.972874	10.590719	10.065643	11.804084	11.311455	11.723743	9.771553	7.725291	9.319403	0.00011348	0.297731961	0.170686404	-0.133855223
ENSG00000188827	SLX4	2.090148	2.028809	1.977737	2.224213	2.004093	2.059138	1.70397	1.510858	2.009531	0.114890107	0.191003669	0.336781599	-0.133847964
ENSG00000106868	SUSD1	9.160353	9.23447	9.687397	6.93965	7.00442	6.10459	7.95918	7.947236	7.993961	0.000267532	-0.348918261	0.180343176	-0.133784838
ENSG00000162104	ADCY9	5.288979	5.21528	5.33118	4.907999	4.906752	4.908227	4.64663	4.109427	4.911228	0.855468392	0.020419934	0.149672221	-0.133771057
ENSG00000105991	HOXA1	4.382473	4.238041	4.480592	3.625417	3.290372	3.911448	3.531505	3.730186	3.619455	0.424073063	-0.134248118	0.386790427	-0.13372851
ENSG00000049883	PTCD2	0.942891	1.065847	0.844647	0.854685	0.945503	1.04972	1.141014	1.07159	0.970378	0.720925121	0.068953332	0.40595692	-0.133672911
ENSG00000204219	TCEA3	20.341359	18.482316	15.700096	33.005545	33.111662	34.403558	16.978176	14.700581	14.965574	2.65E-32	1.024439753	0.28982838	-0.133655452
ENSG00000168646	AXIN2	1.565432	1.294392	1.163628	0.95427	1.100667	1.081037	1.020185	1.346281	1.08605	0.573827233	-0.143425588	0.562576559	-0.133380954
ENSG00000160208	RRP1B	26.072786	26.504761	27.632502	24.061783	23.086586	24.255547	22.800756	20.623919	25.283951	0.803385483	-0.020226165	0.077999562	-0.133308761
ENSG00000266472	MRPS21	41.519456	40.777542	38.717256	32.218617	31.567498	31.177501	34.742023	37.311313	32.563199	0.004019819	-0.208527835	0.167744519	-0.133259656
ENSG00000280789	PAGR1	29.410791	29.316874	26.589569	28.962366	26.966454	30.230888	22.587391	25.063448	25.280418	0.059134934	0.16162547	0.160624166	-0.133120502
ENSG00000184154	LRTOMT	1.612485	1.834689	2.150891	1.392433	1.417947	1.866088	1.588187	2.030185	1.542366	0.702727615	-0.113366616	0.59590656	-0.133070748
ENSG00000121281	ADCY7	0.507858	0.629245	0.435163	0.369462	0.292762	0.379891	0.418872	0.435507	0.425975	0.178470515	-0.387960168	0.635183161	-0.132804579
ENSG00000165312	OTUD1	0.671076	0.544035	0.849838	0.486476	0.556626	0.506853	0.592278	0.58463	0.58356	0.499360213	-0.26471535	0.711215443	-0.132793333
ENSG00000156239	N6AMT1	1.921822	1.979997	2.176883	1.93751	2.24913	1.532004	2.035079	1.90909	1.795266	0.204559868	-0.221298043	0.48021095	-0.132761079
ENSG00000049323	LTBP1	5.31133	4.165274	4.567147	2.15401	2.184484	2.117783	3.627388	4.067239	4.445215	3.13E-23	-1.066697872	0.319286369	-0.132757629
ENSG00000182541	LIMK2	11.633816	12.267192	12.962745	9.77952	9.975908	9.813395	10.128405	10.204341	11.297214	0.052068337	-0.169121213	0.149941762	-0.132327856
ENSG00000143319	ISG20L2	22.657081	22.177769	23.386378	20.562078	20.213318	20.093295	19.206949	19.070608	22.009921	0.837812615	-0.01919166	0.086644294	-0.13221283
ENSG00000158869	FCER1G	2.742527	3.011353	3.945387	2.505621	3.417507	2.400146	3.725961	2.475468	2.106492	0.87827505	-0.072513014	0.740253299	-0.132190379
ENSG00000185339	TCN2	4.70058	2.726842	2.916836	3.021932	3.026037	3.020985	3.067399	2.401381	3.490608	0.765620258	-0.074712319	0.530088591	-0.132111676
ENSG00000163006	CCDC138	6.793924	6.571755	7.3195	5.279985	4.722944	5.297412	6.039709	5.452065	6.015642	0.045315493	-0.259351194	0.319176004	-0.132099488
ENSG00000173905	GOLIM4	32.314198	32.892347	33.696248	27.257435	27.799115	25.816949	26.121831	26.848207	30.946314	0.031861703	-0.134001968	0.072332389	-0.132067703
ENSG00000166582	CENPV	80.54449	79.216368	75.0111	78.301131	78.674098	85.582129	65.92502	65.643413	69.140877	0.032357666	0.151731619	0.062127321	-0.131928893
ENSG00000119801	YPEL5	12.960236	12.027314	11.876313	11.781035	12.868571	12.144389	10.566152	10.549534	10.631418	0.236321572	0.122105316	0.19213021	-0.131922504
ENSG00000136197	C7orf25	11.911112	11.827286	11.504183	10.535416	9.885299	11.234296	10.955541	10.462857	9.372934	0.953310197	0.009385346	0.295523667	-0.131897154
ENSG00000124164	VAPB	10.52535	10.922983	11.454154	10.832075	10.509126	10.62951	9.740019	8.896385	9.417358	0.091665938	0.118245619	0.087450566	-0.131865696
ENSG00000113269	RNF130	9.211921	8.848094	8.893912	5.631733	6.860782	6.478048	8.455574	8.646002	7.636369	0.00037777	-0.383570321	0.238265634	-0.131850436
ENSG00000100335	MIEF1	20.356185	19.860921	18.66919	21.086667	20.25747	20.780535	16.608114	14.865245	18.373974	0.0000369	0.23833179	0.096912507	-0.131676431
ENSG00000172058	SERF1A	2.218928	2.526596	2.43641	1.591731	1.553878	1.795824	2.635257	2.448813	2.044448	0.117989651	-0.447801967	0.650785731	-0.131497157
ENSG00000108588	CCDC47	39.049969	42.20936	44.120301	43.094628	40.972964	40.883903	34.121919	34.762709	38.235088	0.029887292	0.14542674	0.086929667	-0.131492336
ENSG00000172432	GTPBP2	38.412545	35.531321	34.93432	29.960824	28.265361	27.889105	29.30538	30.653313	35.140225	0.00702255	-0.187839296	0.117716228	-0.131418834
ENSG00000151090	THRB	5.454036	5.094146	5.650562	7.148674	6.85623	7.513101	4.931503	4.60077	5.345227	5.15E-15	0.573421782	0.195276269	-0.131337151
ENSG00000121749	TBC1D15	16.248911	17.349775	18.539395	14.389539	15.311035	14.433452	15.789804	13.990945	15.231631	0.718163378	-0.036137346	0.133440521	-0.131216542
ENSG00000175899	A2M	246.693644	200.751126	202.504553	727.041706	757.382666	713.626225	168.884502	189.126342	198.740287	3E-169	1.905054612	0.166452927	-0.131145462
ENSG00000165118	C9orf64	14.517522	14.247709	15.026581	17.404707	15.842115	15.624436	13.004072	12.183506	12.218419	0.0000989	0.307536995	0.167347268	-0.131040459
ENSG00000134744	TUT4	13.090734	12.720754	14.093562	13.953888	12.69889	13.922142	11.64309	10.547129	12.852606	0.148792894	0.11655182	0.12779135	-0.130941323
ENSG00000011275	RNF216	18.292374	18.261705	17.214858	20.316061	17.813044	19.012055	15.985494	14.07227	16.838349	0.000444256	0.238090708	0.118459944	-0.130678183
ENSG00000106278	PTPRZ1	0.507508	0.490568	0.317817	0.256611	0.275755	0.433231	0.305145	0.365861	0.362126	0.150081465	-0.431018772	0.66619098	-0.130638192
ENSG00000117153	KLHL12	15.93712	16.221683	16.314263	13.776408	13.545187	12.891883	14.129984	13.079519	14.069498	0.111024961	-0.126462979	0.102442939	-0.130564267
ENSG00000087586	AURKA	35.234915	36.937954	38.737076	31.226683	27.335336	30.368915	35.307452	33.491956	32.392185	0.006214158	-0.195501474	0.078641615	-0.130520958
ENSG00000100350	FOXRED2	13.297666	12.87684	11.024456	11.301551	9.80883	11.649313	10.640133	9.930651	11.589764	0.983443072	-0.002898744	0.140602547	-0.130470389
ENSG00000108094	CUL2	10.44871	10.976623	10.876684	14.408209	13.967781	13.746765	9.57814	8.963329	9.121437	1.32E-16	0.534578903	0.13587356	-0.130415089
ENSG00000110075	PPP6R3	110.827281	120.371921	119.461272	112.247117	111.58777	109.750476	104.860489	97.246637	108.256418	0.739961102	0.023371838	0.029601608	-0.130371798
ENSG00000184787	UBE2G2	26.639764	27.975303	30.816939	29.252029	28.128305	26.770395	24.199729	23.766322	25.920894	0.215575155	0.101897115	0.105581832	-0.130142227
ENSG00000175938	ORAI3	13.276191	10.489581	8.746031	9.73595	10.547139	10.091503	9.333112	8.967529	9.922189	0.78013289	0.05362338	0.402277298	-0.130132295
NewGene_1987	--	7.894214	9.201794	10.00903	9.877147	8.561426	8.37839	9.347006	8.057015	9.490938	0.30832416	0.105035494	0.172249147	-0.130055156
NewGene_6554	--	7.204755	7.770669	7.709939	5.91272	6.812532	6.080366	6.546076	5.949092	6.322697	0.302908543	-0.140609532	0.301280047	-0.130043728
ENSG00000160299	PCNT	10.342344	9.827621	8.786006	9.701725	9.205162	9.326618	8.334826	7.734275	8.727474	0.197338853	0.10353627	0.112914888	-0.129899741
ENSG00000099849	RASSF7	30.44334	30.251829	25.055677	22.236063	22.730594	23.766464	22.109817	25.166123	25.244415	0.075014823	-0.181116295	0.232036397	-0.129899268
ENSG00000198431	TXNRD1	51.273304	53.687805	52.979827	46.496447	46.730477	45.799337	45.784777	43.358798	46.528477	0.442723709	-0.052092053	0.034688522	-0.129897744
ENSG00000106524	ANKMY2	16.096623	14.938455	14.810987	12.634752	11.509191	12.579907	13.768204	12.990232	12.673738	0.059826162	-0.169105456	0.176775574	-0.129791038
ENSG00000128335	APOL2	2.54953	2.002245	2.337884	32.033493	34.898652	30.063271	1.679666	1.675858	2.056666	4.67E-305	3.647234915	0.538487376	-0.129766863
ENSG00000128578	STRIP2	0.662909	0.826788	0.64679	0.643406	0.708616	0.418786	0.628499	0.562937	0.682836	0.74694155	-0.114598692	0.633724868	-0.129670211
ENSG00000180346	TIGD2	3.634669	4.119748	3.399723	2.518727	2.540444	2.846729	3.415432	3.015621	3.444073	0.003810331	-0.405694307	0.39228887	-0.129659903
ENSG00000141401	IMPA2	10.284902	10.056226	9.545042	7.622972	8.167385	7.616431	8.635345	8.242109	8.829868	0.106694759	-0.209558371	0.311271182	-0.129604294

Gene ID	Gene name	Ctrl-1 FPKM	Ctrl-2 FPKM	Ctrl-3 FPKM	IFNγ-1 FPKM	IFNγ-2 FPKM	IFNγ-3 FPKM	Sora-1 FPKM	Sora-2 FPKM	Sora-3 FPKM	Ctrl vs IFNγ DESeq2 FDR	Ctrl vs IFNγ DESeq2 log2FC	Ctrl vs Sora DESeq2 FDR	Ctrl vs Sora DESeq2 log2FC
ENSG00000048162	NOP16	26.734373	23.819692	27.320041	22.469138	21.45576	21.948455	20.823365	21.670884	23.338149	0.394437281	-0.093337817	0.218687084	-0.129552047
ENSG00000114742	WDR48	12.922809	13.111569	12.804357	12.814891	13.219728	11.784009	10.682628	11.217644	11.382653	0.219880543	0.108010819	0.124324358	-0.129520984
ENSG00000049245	VAMP3	35.807846	40.435806	38.897405	41.289601	39.737347	40.775301	32.574533	31.46615	35.424058	0.000926337	0.223887502	0.105459904	-0.12948074
ENSG00000175895	PLEKHF2	10.290148	10.638766	11.821249	20.851025	18.407021	19.789507	10.321468	8.80783	8.944448	4.24E-39	0.999329933	0.256614517	-0.12942426
ENSG00000136051	WASHC4	10.555301	11.416427	11.835995	13.362073	12.119127	12.401944	10.479819	8.895945	11.048485	0.0000656	0.28166628	0.135731348	-0.129413578
ENSG00000154727	GABPA	14.314087	15.3557	16.077832	13.642819	13.700457	14.283389	12.337126	12.757402	14.242479	0.999202724	0.000119961	0.118398683	-0.129385796
NewGene_6738	--	4.746221	4.487579	4.716386	4.554447	4.802161	4.904751	4.066367	3.609061	4.536825	0.034119405	0.164572637	0.159294989	-0.129313445
ENSG00000025156	HSF2	12.684254	14.911906	13.480432	11.386294	11.431708	12.560943	11.705332	11.649602	11.439092	0.447638222	-0.081838758	0.195159763	-0.129231367
ENSG00000165280	VCP	126.196789	122.485249	117.598453	119.633361	114.920254	115.310162	99.721916	103.316245	110.963945	0.160183771	0.079454112	0.043405434	-0.12919236
ENSG00000121690	DEPDC7	4.654746	3.958192	4.759628	4.0418	5.088241	3.857762	3.679551	4.093355	3.658072	0.638351631	0.105052389	0.487315681	-0.129152777
ENSG00000169057	MECP2	3.015688	2.781002	2.666224	2.570761	2.561514	2.696483	2.744799	2.931471	3.575701	0.534389892	0.073449691	0.232872189	-0.129141606
ENSG00000173402	DAG1	27.020877	26.535143	25.819086	25.736134	24.520579	25.632079	25.116939	21.610169	25.046393	0.121785572	0.097186347	0.060037635	-0.129104667
ENSG00000147224	PRPS1	22.384922	22.768705	22.412723	20.12329	20.069843	19.611506	19.421259	19.268445	19.206614	0.780131293	-0.02815008	0.122313066	-0.129075462
ENSG00000179299	NSUN7	4.069586	3.588262	3.592628	3.540927	3.95758	3.4954	3.460103	3.095703	3.060792	0.427697911	0.12022419	0.364923675	-0.129007481
ENSG00000258461	AC012651.1	0.851385	0.800849	0.44514	0.41381	0.569213	0.516874	0.872757	0.75703	0.158396	0.400817775	-0.315463524	0.900023685	-0.128920237
ENSG00000163428	LRRC58	7.559866	8.740768	9.570765	8.177726	7.790884	7.998478	7.692533	6.606624	7.917224	0.754114313	0.037521393	0.21823771	-0.128862866
ENSG00000100344	PNPLA3	13.776361	13.557837	12.791008	18.888049	19.024099	17.356416	11.586802	11.41154	12.669919	1.34E-15	0.598426141	0.176898707	-0.128839743
ENSG00000111817	DSE	7.07839	6.627838	7.150914	7.171146	8.762736	6.700746	7.100012	6.304664	6.102895	0.009366697	0.238723931	0.214518317	-0.128684973
ENSG00000198042	MAK16	12.707845	13.531177	15.111302	12.360493	11.794791	11.820117	11.763032	11.236005	12.646633	0.575706339	-0.060945101	0.178716765	-0.12863819
ENSG00000154305	MIA3	11.90829	11.661185	10.221984	14.233905	12.199147	11.631875	9.707541	9.953616	11.605267	0.00000515	0.290225679	0.088610216	-0.128634805
ENSG00000122592	HOXA7	5.943748	6.990707	6.218757	4.962475	4.809003	4.551286	5.588569	5.123192	5.876205	0.049542239	-0.272851616	0.369204762	-0.128625823
ENSG00000188895	MSL1	28.124554	28.979168	30.405092	29.303077	27.904498	28.112627	26.130751	22.711531	25.163791	0.11161461	0.107439781	0.079672924	-0.12857002
ENSG00000116138	DNAJC16	6.814993	6.71505	7.661223	7.508997	7.592805	6.783622	6.082115	5.780494	6.504539	0.214998997	0.116149111	0.16385451	-0.128557427
ENSG00000072071	ADGRL1	4.296045	4.186744	4.420874	4.483949	3.886921	4.446318	3.289645	3.44926	4.247968	0.471618837	0.08077392	0.272396721	-0.128460371
ENSG00000105948	TTC26	2.008832	1.931895	1.967151	1.272983	0.9141	1.090228	1.602296	1.669783	1.603258	0.0000481	-0.680145369	0.45723117	-0.128414035
ENSG00000185418	TARSL2	4.906262	4.467647	4.35405	3.89222	3.939034	3.818815	3.991124	3.609748	4.202083	0.533660232	-0.089755816	0.319347038	-0.12840523
ENSG00000165272	AQP3	10.7391	8.912536	7.210741	11.174781	10.139393	10.456804	7.536819	6.974864	8.732394	0.005463768	0.386091979	0.435445838	-0.128383775
ENSG00000117640	MTFR1L	13.020842	12.418759	12.057006	12.034527	13.184102	11.74401	10.19672	10.241388	12.686287	0.189346344	0.140726251	0.288984766	-0.128367981
ENSG00000129518	EAPP	29.35086	28.638819	26.475935	28.950177	27.055778	27.676664	26.661046	23.891987	22.408439	0.145654522	0.13208187	0.243506446	-0.128202871
ENSG00000189060	H1F0	94.092056	75.238503	69.509628	98.896049	102.511337	97.165718	69.971413	66.755829	68.203934	0.003232101	0.469233076	0.22997668	-0.128176562
ENSG00000137563	GGH	48.372349	48.338268	49.310658	38.63784	39.024769	38.626137	41.923153	43.051731	40.160843	0.005250763	-0.180954659	0.118364775	-0.127889721
ENSG00000069956	MAPK6	66.375748	68.422363	64.937637	57.138195	58.384876	55.648479	57.765465	55.261356	58.410583	0.208624896	-0.075173035	0.028695557	-0.127821386
ENSG00000108924	HLF	4.048339	4.94991	5.171338	3.618844	3.282787	3.483639	4.368698	3.489546	4.491109	0.006871837	-0.310538598	0.336862045	-0.127771124
ENSG00000124181	PLCG1	26.027331	24.889167	23.335695	20.43917	19.19879	19.192077	21.353998	19.743054	22.778762	0.002204874	-0.194530651	0.07791416	-0.127720148
ENSG00000166471	TMEM41B	32.418725	39.462407	43.088782	36.543213	34.199058	43.261374	33.948739	25.289866	33.407782	0.011075309	0.178870571	0.116155106	-0.127701293
ENSG00000166326	TRIM44	14.397969	14.496043	14.880305	17.708981	16.258348	17.656042	12.745489	11.867292	12.981	6.69E-14	0.385029364	0.038761964	-0.127663578
ENSG00000170633	RNF34	20.650586	19.957848	20.895722	20.646716	18.732235	23.11544	17.429081	17.632704	20.092224	0.265907504	0.097979263	0.149204902	-0.12736652
ENSG00000140284	SLC27A2	8.806204	8.593529	8.320338	11.425732	10.246832	9.985895	7.586907	6.776737	7.772968	0.00000111	0.448099782	0.259213666	-0.127349568
ENSG00000196814	MVB12B	9.448989	9.211704	8.61687	6.673657	6.801265	6.9957	8.186532	7.54326	9.168577	0.000453244	-0.28506655	0.198040865	-0.127263872
ENSG00000144677	CTDSPL	6.919953	6.681952	7.899006	3.445953	3.372811	3.587989	6.218293	5.548178	6.311334	7.3E-20	-0.856890734	0.241327323	-0.127193952
ENSG00000158301	GPRASP2	0.502157	0.555501	0.342586	0.418274	0.332212	0.430968	0.406174	0.377247	0.401001	0.860472621	-0.084039361	0.739614412	-0.127123678
ENSG00000138771	SHROOM3	1.096169	1.209001	1.044687	0.730948	0.663795	0.780536	0.913429	0.907368	1.056899	0.000545967	-0.473575793	0.384403644	-0.127097627
ENSG00000065609	SNAP91	1.458834	1.668747	1.523637	1.499634	0.811041	1.006928	1.205645	1.5965	2.184363	0.01665657	-0.482446565	0.559475358	-0.127094439
ENSG00000125845	BMP2	2.539439	2.630148	3.237054	2.256362	2.421797	2.727414	2.467596	2.132519	2.637395	0.889757182	-0.032021139	0.460072188	-0.126949437
ENSG00000142632	ARHGEF19	2.260211	2.10539	2.057855	2.050194	2.211252	1.893831	2.139863	1.528534	2.255414	0.78779918	0.066968446	0.550283378	-0.12686696
NewGene_999	--	1.199407	1.082416	1.648163	1.122938	1.156754	1.09581	1.068125	1.181349	1.312609	0.730344062	-0.124721886	0.685670634	-0.126851388
ENSG00000011295	TTC19	12.235921	15.124766	14.389842	13.246371	11.556408	11.727816	12.679374	11.343139	12.989233	0.910266138	-0.014031828	0.181462327	-0.126834172
ENSG00000198909	MAP3K3	4.382389	4.159611	4.198486	4.930642	4.888615	5.383916	3.621094	3.659651	3.898385	0.0000775	0.393592238	0.28286231	-0.12682898
ENSG00000274810	NPHP3-ACAD1	1.793446	1.369427	1.900522	1.627446	0.823684	0.895394	1.476657	1.117198	1.776615	0.300411339	-0.454567984	0.511039796	-0.126619108
ENSG00000101546	RBFA	11.368616	10.059431	10.742376	9.5948	8.812149	10.139465	9.48868	8.60284	9.463657	0.926257614	-0.017268888	0.337836876	-0.126543862
NewGene_2199	--	1.649299	1.08079	1.290508	0.823792	1.305216	0.756253	0.749314	0.899554	1.667721	0.321272889	-0.404266872	0.776546151	-0.126491257
ENSG00000130038	CRACR2A	2.366043	2.127624	2.22732	1.470994	1.549714	1.352207	2.010227	2.109616	1.929992	0.005929014	-0.539061017	0.541940183	-0.126482444
ENSG00000198625	MDM4	8.449234	8.947651	10.386017	9.903876	8.678243	8.964459	9.616789	8.985823	10.339796	0.229198915	0.10900591	0.173250142	-0.126328095
ENSG00000115159	GPD2	7.647302	8.039517	9.264523	7.204491	6.061413	6.845097	7.672477	7.103467	7.972235	0.071169672	-0.178139502	0.20027107	-0.126324804
ENSG00000183597	TANGO2	7.877878	7.274257	6.813193	6.563449	5.762932	5.686652	6.608994	5.734627	7.398654	0.280839317	-0.145515773	0.358325319	-0.126314967
ENSG00000148541	FAM13C	0.931867	1.040022	0.807122	0.684305	0.88021	0.745913	0.725656	0.725608	0.752773	0.754291713	-0.130721497	0.717447314	-0.126295043
ENSG00000104833	TUBB4A	12.361229	11.118396	10.297189	5.345436	5.363316	7.237246	8.995561	10.567805	9.693706	3.56E-10	-0.757127063	0.296141108	-0.126185563
ENSG00000235109	ZSCAN31	4.77382	4.333499	3.538362	3.135548	3.38362	3.537507	3.535726	3.990273	3.723963	0.279432843	-0.182238447	0.442654142	-0.126096689
ENSG00000211460	TSN	66.658712	69.559814	69.133547	61.378895	64.055258	64.670736	55.309459	51.343633	59.745371	0.646566852	0.036458881	0.062640681	-0.126010713
ENSG00000010270	STARD3NL	27.059421	26.613643	27.65777	24.441131	24.206853	25.529268	22.853834	23.414869	24.597723	0.932599785	-0.010137927	0.144008071	-0.125976621
ENSG00000166814	ZSCAN30	3.658392	3.717788	3.870564	3.100207	2.671624	2.938206	3.376122	3.368955	3.675335	0.155245859	-0.186173673	0.346759517	-0.125769798
ENSG00000030582	GRN	110.54968	103.826323	96.126548	93.85617	96.140928	96.431468	86.575488	84.434086	94.749716	0.598604664	0.043099369	0.074696613	-0.125755285
ENSG00000099999	RNF215	1.580091	1.499061	1.488743	1.462304	1.785479	1.402199	1.432627	1.047343	1.193999	0.689749755	0.124146227	0.649273731	-0.12573074
ENSG00000116127	ALMS1	1.882449	1.962538	2.198315	1.919676	1.76074	1.770038	2.09419	1.367099	2.082494	0.556247988	0.078384031	0.299267242	-0.125643585
ENSG00000240849	TMEM189	4.924166	5.195753	5.28992	3.790213	5.187226	4.884735	5.646398	4.732526	6.004374	0.0000493	-0.385236929	0.22512817	-0.125553706
ENSG00000171160	MORN4	0.85388	0.986335	0.980875	0.372128	0.72523	0.838201	0.925914	0.81167	0.721317	0.416502121	-0.375265854	0.75343586	-0.125548174
ENSG00000075213	SEMA3A	4.332358	4.727654	4.727784	4.51093	4.271262	4.725266	3.627264	3.963357	4.253345	0.203104513	0.120377722	0.211646326	-0.125325833
ENSG00000133030	MPRIP	18.825435	15.135816	13.84926	14.168886	12.744108	14.991408	17.888424	13.637033	21.184651	0.54845223	-0.049785868	0.140456298	-0.125172017
ENSG00000161544	CYGB	1.632859	1.327033	1.088976	1.06449	1.090116	1.11398	1.33779	1.213681	0.856749	0.626581457	-0.175504594	0.714357619	-0.12513475
ENSG00000134444	RELCH	3.839176	4.579061	4.126757	4.748285	4.788119	4.443614	4.073812	3.005127	3.577566	0.001937675	0.289346647	0.311771747	-0.124942366
ENSG00000103319	EEF2K	3.714704	4.166302	4.122408	5.28023	4.608465	5.067513	3.54317	3.278306	3.572211	5.52E-08	0.456220416	0.222498101	-0.124702598
ENSG00000154639	CXADR	30.780705	31.294693	30.656714	28.161303	27.977117	27.275124	25.359192	24.192408	25.902158	0.737831657	-0.02574052	0.051544572	-0.12449507
ENSG00000267534	S1PR2	2.791037	2.893177	2.652099	2.677192	2.196108	2.728807	2.525724	2.336011	2.262641	0.940586125	0.016592039	0.430559456	-0.124470494
ENSG00000172339	ALG14	1.580199	1.720803	1.539987	1.622926	1.471239	1.654886	1.28979	1.428716	1.442374	0.37634884	0.120969466	0.354560354	-0.124383678

Gene ID	Gene name	Ctrl-1 FPKM	Ctrl-2 FPKM	Ctrl-3 FPKM	IFNγ-1 FPKM	IFNγ-2 FPKM	IFNγ-3 FPKM	Sora-1 FPKM	Sora-2 FPKM	Sora-3 FPKM	Ctrl vs IFNγ DESeq2 FDR	Ctrl vs IFNγ DESeq2 log2FC	Ctrl vs Sora DESeq2 FDR	Ctrl vs Sora DESeq2 log2FC
ENSG00000140564	FURIN	105.336569	97.422492	95.370249	104.013167	99.137948	105.437352	80.012115	80.114518	97.662668	0.000220825	0.205809658	0.108206569	-0.12424647
ENSG00000110171	TRIM3	1.987843	2.248497	2.139853	2.223953	2.388072	1.996851	1.916083	1.5312	2.01312	0.26125426	0.216853826	0.541290216	-0.124160109
ENSG00000161920	MED11	30.426499	29.714045	29.505784	24.342222	26.773004	26.83733	25.696598	25.681518	25.668532	0.703205084	-0.050422306	0.24006421	-0.124077033
ENSG00000197779	ZNF81	0.462772	0.529057	0.724126	0.584043	0.487693	0.41896	0.416791	0.391303	0.510939	0.669237544	0.12394491	0.616185074	-0.124061687
ENSG00000147251	DOCK11	0.678126	0.810505	0.66548	0.384183	0.320468	0.313672	0.653227	0.488706	0.690088	0.0000239	-0.911204756	0.602842589	-0.124008814
ENSG00000149357	LAMTOR1	42.73582	43.165128	38.380065	36.640656	36.012254	38.620934	33.938989	36.331702	37.314076	0.828494394	-0.024504414	0.170191448	-0.123890054
ENSG00000179041	RRS1	15.113268	16.738447	18.114048	15.775977	13.725317	14.919117	14.681609	13.969744	14.310126	0.881579329	-0.022212374	0.265257875	-0.123814251
ENSG00000116489	CAPZA1	47.925945	50.543461	51.254623	47.941025	48.33997	46.936226	43.057335	41.89579	43.906727	0.199454452	0.083396507	0.060551349	-0.123751102
ENSG00000158079	PTPDC1	1.943649	1.868603	2.091141	1.702404	1.154305	1.282789	1.859799	1.497525	1.740437	0.04198821	-0.369148288	0.467576261	-0.123721512
ENSG00000198780	FAM169A	5.288097	6.014853	5.567797	4.084938	4.152762	3.947899	5.129263	4.739568	5.596186	0.000178426	-0.358701804	0.275506948	-0.123538151
ENSG00000105849	TWISTNB	13.345974	17.112598	15.227317	14.01934	12.591505	14.072345	13.87013	12.267558	13.193497	0.890549819	-0.019109705	0.244994936	-0.123314129
ENSG00000162039	MEIOB	36.507197	37.736392	39.581698	32.151207	26.073142	30.460123	32.659318	30.39923	34.609153	0.034013604	-0.191162794	0.122919419	-0.123310848
ENSG00000091409	ITGA6	18.97648	19.958224	20.518318	21.546624	21.868573	21.769266	16.754649	17.029504	17.383948	0.00000102	0.28106613	0.083007488	-0.123222085
ENSG00000159459	UBR1	3.881756	4.108474	3.848357	5.000033	4.403701	3.990327	3.467157	3.229409	3.55878	0.000561779	0.329152413	0.211136753	-0.123162402
ENSG00000160752	FDPS	315.534413	277.599173	262.961985	297.361067	321.209274	316.169202	237.67912	249.128062	248.95742	0.000175237	0.268837752	0.136563484	-0.123046433
ENSG00000130347	RTN4IP1	6.201836	8.26863	7.578434	7.366331	6.588277	7.53411	6.645619	6.099399	6.222976	0.522819939	0.111934969	0.444792522	-0.123013269
ENSG00000159131	GART	72.208367	70.915675	73.004312	65.655892	65.837952	64.763808	57.497125	58.286588	63.46088	0.563478673	0.039641439	0.05065029	-0.123001205
ENSG00000164751	PEX2	12.064093	12.397785	12.420117	10.115508	10.317651	10.895003	8.83693	10.449244	9.186904	0.933020221	0.010972257	0.249699159	-0.12284994
ENSG00000184226	PCDH9	0.460402	0.603721	0.697753	0.171839	0.221582	0.378074	0.507785	0.458999	0.539812	6.66E-20	-1.463486755	0.464039026	-0.122697002
ENSG00000183386	FHL3	9.549643	9.89753	10.884921	5.942166	5.929689	6.008311	8.091285	8.930467	9.0912	8.26E-08	-0.614131088	0.350697867	-0.122635489
ENSG00000109927	TECTA	0.181272	0.184913	0.204343	0.305334	0.300549	0.220129	0.14226	0.135243	0.22372	0.063761026	0.616731821	0.777022818	-0.12233848
ENSG00000100304	TTLL12	27.758472	28.618615	28.081619	24.357607	21.953059	25.071929	24.038142	22.48333	26.559067	0.214544923	-0.095864934	0.094883641	-0.122192787
NewGene_1138	--	2.853007	2.903524	2.736776	2.115655	2.419482	2.466963	2.756344	2.93008	2.12624	0.133589562	-0.26393713	0.511215955	-0.122135873
ENSG00000108055	SMC3	36.79221	39.200768	42.533161	36.075623	32.4104	33.240768	33.674435	32.35434	36.179256	0.367831121	-0.073628997	0.109344046	-0.122035075
ENSG00000183475	ASB7	4.391906	4.204849	4.727457	5.082542	5.039261	5.343375	4.092921	3.528584	4.073302	0.000242843	0.349072048	0.299720628	-0.121903221
ENSG00000144354	CDCA7	35.858829	39.802459	40.72477	33.659889	31.47046	31.991682	31.716062	31.631305	33.493831	0.114021974	-0.122378393	0.134169421	-0.121852307
ENSG00000104760	FGL1	103.08728	92.603401	84.98121	108.751785	109.226782	113.417429	80.428593	78.413952	83.634923	2.19E-09	0.423472344	0.160410421	-0.121751382
ENSG00000170085	SIMC1	1.87735	1.838252	2.075895	2.741627	2.302845	1.979775	1.656543	1.465121	1.550296	0.036748614	0.394494911	0.542448118	-0.121422824
ENSG00000144040	SFXN5	5.61569	6.708378	6.183683	5.704478	5.917046	5.622661	4.690481	5.305468	5.600598	0.697572728	0.051078266	0.308961875	-0.121302047
ENSG00000263528	IKBKE	5.179385	4.879863	4.627161	2.879772	2.90415	3.360139	4.470647	4.009313	4.249379	0.0000169	-0.533704824	0.36276397	-0.121275202
ENSG00000133111	RFXAP	3.840816	4.165266	5.150878	4.452029	4.13695	4.125194	3.028313	3.884062	4.449977	0.611948042	0.098198427	0.539704187	-0.121136314
ENSG00000283486	FAM95C	1.032484	0.866391	1.586962	0.778323	0.773031	0.447697	0.586968	0.766943	1.268452	0.266561448	-0.478402438	0.752470807	-0.121127032
ENSG00000243156	MICAL3	6.145053	5.783009	5.594342	4.417406	3.599697	4.054977	5.090536	4.582966	5.639671	0.000000643	-0.390919739	0.230815138	-0.120963236
ENSG00000196792	STRN3	14.480078	15.765932	17.129017	16.624391	16.598918	16.167377	14.300472	12.352824	12.991518	0.007988512	0.218375528	0.237635996	-0.120912101
NewGene_521	--	2.057168	1.884294	2.034427	1.394643	1.629856	1.655466	2.596566	2.426402	2.576882	0.142821046	-0.275297398	0.513275087	-0.120760624
ENSG00000213918	DNASE1	3.78335	4.123174	4.821623	3.76989	3.649341	3.542607	3.735484	3.038792	3.988301	0.679330944	-0.057144252	0.311753938	-0.120676325
ENSG00000243649	CFB	38.528272	29.88263	25.072659	163.797403	162.777998	151.694715	26.567871	27.18213	26.895095	2.18E-39	2.49246629	0.603489384	-0.120425517
ENSG00000168092	PAFAH1B2	20.141996	21.84406	22.769616	19.577346	20.103408	19.685336	20.023774	16.534863	19.316674	0.821681942	0.022056519	0.177097986	-0.120364021
ENSG00000174586	ZNF497	0.706185	0.804434	0.976764	0.719415	0.947876	0.871918	0.778511	0.686006	0.674049	0.440703195	0.254910521	0.710028419	-0.120306393
ENSG00000132254	ARFIP2	49.764499	44.884181	48.120732	50.500522	50.441919	46.076165	37.447701	42.969855	47.025426	0.00659306	0.163287	0.192026242	-0.120288333
NewGene_91	--	0.812678	0.882361	0.936726	0.608944	0.347725	0.499564	0.754976	0.906696	0.703978	0.161652131	-0.589457742	0.774060045	-0.120266
ENSG00000121964	GTDC1	2.705984	4.82601	4.429867	3.211111	3.764088	3.964098	3.963854	4.502668	3.433848	0.051083245	-0.276897776	0.401276834	-0.120226042
ENSG00000155096	AZIN1	53.979719	59.71293	59.498605	50.370204	45.964225	45.927908	50.061892	46.606867	51.818304	0.107228837	-0.109676859	0.095272541	-0.120213377
ENSG00000159884	CCDC107	6.394118	5.029259	4.388584	3.91379	3.488739	2.683809	3.93355	4.835834	5.231635	0.01677477	-0.534024769	0.600440512	-0.120119298
ENSG00000071054	MAP4K4	182.810061	188.269077	182.637541	157.659679	134.134996	146.931043	154.929285	147.279057	163.320126	0.000178737	-0.193493869	0.029239095	-0.120094963
ENSG00000164542	KIAA0895	4.691618	4.366406	4.042804	2.99272	3.3282	3.023792	3.507113	3.059539	4.223832	0.007920376	-0.3248708	0.380166766	-0.120090427
ENSG00000154920	EME1	5.870689	7.36847	6.489218	5.616164	4.263294	5.239355	5.483141	5.509958	5.521033	0.129380555	-0.230989615	0.387902403	-0.12007786
ENSG00000100564	PIGH	10.284026	8.7657	7.969766	8.504679	9.134141	9.858417	7.738096	7.405475	8.139855	0.21174321	0.190075808	0.432110481	-0.120076308
ENSG00000144741	SLC25A26	7.81409	7.331372	8.098613	5.68866	7.545474	6.088772	7.338587	7.197022	7.940394	0.040372713	-0.265036429	0.3780526	-0.119983018
ENSG00000135333	EPHA7	0.791733	0.772272	0.891632	0.475622	0.326529	0.463416	0.634574	0.635036	0.860032	0.005564569	-0.622579397	0.600219369	-0.119931142
ENSG00000077147	TM9SF3	40.864125	44.355972	45.958725	46.123059	44.620262	45.442617	37.377032	35.728933	40.230988	0.000582902	0.198995587	0.085655068	-0.119930805
NewGene_6667	--	6.276745	5.266737	4.708857	4.72153	4.325746	5.009389	5.105431	4.658026	4.456656	0.611230036	-0.113109351	0.565735363	-0.11990038
ENSG00000105810	CDK6	11.097266	12.098101	14.531335	9.935132	9.449135	9.705852	10.717177	9.94582	12.196356	0.009405093	-0.232235734	0.256229809	-0.119883431
ENSG00000187626	ZKSCAN4	4.597288	4.526608	4.486391	3.986387	4.379878	4.192399	4.208605	3.768454	3.766679	0.869676208	0.031563264	0.432594052	-0.119523419
ENSG00000127774	EMC6	66.248282	53.689033	51.386609	46.172666	46.808957	49.681599	49.730524	47.620602	49.886548	0.336994055	-0.120507397	0.308108406	-0.119513243
ENSG00000130584	ZBTB46	7.411016	6.584791	6.658187	4.690122	4.985793	5.265593	7.253873	5.168656	6.470794	0.002188638	-0.270409631	0.265506134	-0.11950976
ENSG00000173933	RBM4	144.939469	144.917416	144.367433	137.123322	131.562625	138.190511	135.110577	145.825102	129.187206	0.129144743	0.08589522	0.10238261	-0.119505997
ENSG00000119559	C19orf25	23.164935	24.331574	22.809549	18.15403	18.035214	19.002307	16.460077	18.911499	19.714937	0.002725692	-0.251632196	0.243051537	-0.11946357
ENSG00000088812	ATRN	21.159084	18.996878	17.770562	19.051298	18.568609	17.779246	15.93449	15.04139	16.817899	0.040012656	0.141595298	0.110129966	-0.119457983
ENSG00000015676	NUDCD3	18.494173	18.637514	17.531937	15.455517	14.396882	14.935916	16.237278	14.855546	16.120293	0.019785267	-0.140520944	0.076995084	-0.119340826
ENSG00000161249	DMKN	56.044146	57.006694	55.18584	44.845008	43.123953	43.228059	50.249435	52.880289	46.066788	0.011705299	-0.198550652	0.224792957	-0.119332333
ENSG00000148123	PLPPR1	2.287114	2.81568	1.785488	1.525735	1.125524	1.282422	2.141168	2.123519	1.658354	0.005267546	-0.642942911	0.638190461	-0.119327119
ENSG00000136213	CHST12	6.116109	2.245009	5.151379	3.308905	3.410236	1.966286	1.753073	4.206866	4.535753	0.761768675	-0.039640759	0.243607595	-0.11926248
ENSG00000125445	MRPS7	60.740775	57.938841	56.229826	47.712258	45.937758	49.649993	48.980066	49.698249	52.49997	0.058364617	-0.139721264	0.109331032	-0.119020761
ENSG00000072756	TRNT1	11.707808	14.197778	13.878916	13.704032	12.274384	11.833611	10.94184	10.677935	11.615029	0.734376812	0.047319266	0.294725359	-0.119013282
ENSG00000152942	RAD17	6.450095	7.173764	6.76079	6.573422	5.898651	7.661427	5.700704	5.499191	6.038468	0.42490786	0.110217343	0.304792562	-0.11895833
ENSG00000122512	PMS2	8.584304	7.883934	7.67656	8.17671	7.610046	7.956876	7.505527	6.551181	6.987835	0.247390022	0.126987334	0.301636553	-0.118618824
ENSG00000149636	DSN1	13.979731	13.969287	13.867002	12.683776	12.218043	11.454306	12.406099	11.393529	11.83548	0.709252294	-0.042851921	0.213727017	-0.118327822
ENSG00000130021	PUDP	7.225417	6.770037	6.863357	7.045829	7.396631	7.595852	6.508918	5.765828	6.092328	0.086068961	0.207203794	0.374296603	-0.118266409
ENSG00000101189	MRGBP	13.395755	13.969632	15.700047	13.970883	13.533364	13.746476	11.25334	12.162527	13.821911	0.408098372	0.085487853	0.279688745	-0.11814846
ENSG00000038210	PI4K2B	23.782963	26.925192	26.997955	31.779951	33.161831	33.584148	22.854481	20.568977	23.779665	8.31E-14	0.490802298	0.158713508	-0.118122228
ENSG00000035687	ADSS	39.158108	41.766659	45.733173	33.788433	35.586887	34.071602	37.282036	33.933308	38.311123	0.064858815	-0.143973987	0.149958932	-0.118094577
ENSG00000161921	CXCL16	39.547309	38.412731	40.190961	57.389899	59.25341	58.734044	32.167532	31.784321	39.256354	2.18E-37	0.715058367	0.171844703	-0.117892048
ENSG00000115993	TRAK2	4.567386	4.510285	4.831087	3.745375	3.686663	3.685322	3.993229	3.943745	4.140839	0.053479195	-0.180396853	0.229566739	-0.117832343

Gene ID	Gene name	Ctrl-1 FPKM	Ctrl-2 FPKM	Ctrl-3 FPKM	IFNγ-1 FPKM	IFNγ-2 FPKM	IFNγ-3 FPKM	Sora-1 FPKM	Sora-2 FPKM	Sora-3 FPKM	Ctrl vs IFNγ DESeq2 FDR	Ctrl vs IFNγ DESeq2 log2FC	Ctrl vs Sora DESeq2 FDR	Ctrl vs Sora DESeq2 log2FC
NewGene 685	--	0.830414	0.946328	1.008461	0.630979	0.68904	0.889005	0.741356	0.825119	0.941411	0.608801037	-0.25310753	0.788659251	-0.117675138
ENSG00000205710	C17orf107	0.66016	0.524424	0.556711	0.351842	0.462903	0.500345	0.612791	0.486941	0.527366	0.411741878	-0.326832318	0.746994746	-0.117550388
ENSG00000107140	TESK1	5.548794	6.495011	5.910436	5.353017	4.73932	4.932686	4.982839	4.665087	5.894214	0.478763256	-0.107061753	0.412949893	-0.117500114
ENSG00000136827	TOR1A	23.907869	24.524557	22.706234	19.548349	21.303165	21.838493	20.388254	20.736065	20.323038	0.765119825	-0.033904682	0.180827526	-0.117441105
ENSG00000124143	ARHGAP40	1.590477	1.578906	1.916418	0.406523	0.23487	0.207309	1.587875	1.448571	1.494227	9.74E-18	-2.364723005	0.605583024	-0.117434456
ENSG00000159217	IGF2BP1	34.96991	36.411118	36.623032	36.761177	34.254674	35.451996	31.397095	30.08182	31.889172	0.017075747	0.126325054	0.048283874	-0.117400463
ENSG00000180185	FAHD1	10.716783	10.462828	10.358976	11.635628	10.700913	11.52405	9.760238	8.251897	9.283422	0.010601655	0.250022308	0.316424562	-0.11704088
ENSG00000065833	ME1	8.056681	8.887064	9.731927	6.352632	5.726385	6.233197	7.956992	7.229387	7.881821	0.0000716	-0.394316653	0.292940718	-0.116754815
ENSG00000151715	TMEM45B	9.392598	9.132936	8.769541	6.215228	7.159477	6.191859	6.991774	7.930932	8.967454	0.001162957	-0.354095726	0.357037309	-0.116748921
ENSG00000126883	NUP214	10.02255	10.03105	10.266972	9.802645	8.571665	9.144936	9.648324	7.944724	9.072055	0.927540381	0.009377155	0.128198976	-0.116723811
ENSG00000100219	XBP1	128.809443	135.432431	121.878692	171.762204	189.267984	176.639773	142.833132	131.857366	142.401802	2.09E-23	0.527469235	0.089294337	-0.116512604
ENSG00000146678	IGFBP1	0.960737	0.822898	0.558228	0.704808	0.727652	0.798747	0.862759	0.349012	0.816364	0.8787686	0.083338472	0.819036416	-0.116445298
ENSG00000126218	F10	50.819324	41.328658	34.308281	36.932268	36.777595	36.646719	36.303274	36.439754	36.743528	0.882714182	-0.042276261	0.381141333	-0.116407424
ENSG00000196323	ZBTB44	3.555207	3.850184	4.010829	3.970308	4.003231	3.7432	3.221397	2.928311	3.686567	0.044346705	0.182659834	0.271752337	-0.116324926
ENSG00000138794	CASP6	15.97368	16.707639	15.244865	13.450322	13.326311	13.504089	13.614418	12.804193	14.907142	0.39727772	-0.095540295	0.276378201	-0.116276257
ENSG00000183337	BCOR	6.011991	5.292156	5.997798	6.847101	6.087972	6.7328	5.626734	4.817634	5.341687	0.000475628	0.292079886	0.27111238	-0.116236997
ENSG00000101844	ATG4A	6.241249	5.830582	5.203224	6.026194	6.203168	6.154722	5.187078	5.442931	4.407529	0.062462429	0.233550631	0.458502718	-0.116225298
ENSG00000196873	CBWD3	19.020251	15.604586	18.401509	16.651152	14.747273	15.753315	14.737382	13.956143	17.278248	0.973041893	0.00544912	0.287198456	-0.116214838
ENSG00000150768	DLAT	17.887707	19.523544	20.468243	21.581106	21.156446	21.000012	16.210825	15.864657	18.213789	0.0000177	0.284695954	0.170033734	-0.116044467
ENSG00000046651	OFD1	6.220828	6.650207	6.587588	6.999388	5.633836	5.595027	5.184649	5.42786	6.1109	0.757338312	0.045739155	0.298573671	-0.116029058
ENSG00000083312	TNPO1	16.928911	18.9033	19.084085	14.928256	15.242807	15.132217	15.929429	15.676485	16.942236	0.050861023	-0.138186788	0.127303693	-0.115949411
ENSG00000115840	SLC25A12	0.657496	0.566162	0.384128	0.622753	0.401095	0.429409	0.292106	0.427007	0.677607	0.98710473	-0.00877082	0.800942955	-0.11592548
ENSG00000154781	CCDC174	7.789205	7.963911	8.714785	7.539047	7.682564	7.89742	7.770345	6.192603	7.252087	0.59686794	0.066204173	0.341475802	-0.115896935
ENSG00000197620	CXorf40A	3.601438	2.466265	2.40931	2.747702	2.648418	3.2244	2.045701	2.026806	3.165861	0.321555243	0.271400416	0.700668816	-0.115841931
ENSG00000053372	MRTO4	25.016869	26.259306	25.693501	19.646896	19.019384	19.451056	21.172081	21.769888	23.69632	0.000155702	-0.257645366	0.153406101	-0.115827966
ENSG00000176476	SGF29	11.656559	13.430989	11.375828	12.074992	10.252368	11.070659	11.704047	10.801717	9.332401	0.903882386	0.022225807	0.465661649	-0.115758994
ENSG00000003400	CASP10	3.498988	3.553019	3.179803	5.855167	5.45296	5.583093	2.793455	3.001302	3.752734	6.62E-18	0.747305203	0.329694475	-0.115703245
ENSG00000108244	KRT23	140.734213	146.712123	170.286289	42.335811	38.942854	40.983511	124.808849	131.114955	139.273308	1.44E-153	-1.757096861	0.173147736	-0.115690068
ENSG00000149292	TTC12	1.980008	1.411497	1.817201	1.386638	1.266018	1.031059	1.668523	1.526464	1.384831	0.155646669	-0.334299447	0.627902918	-0.115622777
NewGene 5222	--	0.73556	0.665235	0.669791	0.147093	0.317085	0.169784	0.827881	0.476925	0.473956	0.000651928	-1.485096651	0.788414179	-0.115499451
ENSG00000136813	ECPAS	16.193819	16.382147	15.940816	18.038276	16.138892	16.750127	14.400628	13.578324	13.926907	0.000500668	0.205404618	0.08956948	-0.115421598
ENSG00000160294	MCM3AP	28.423724	27.876003	27.290361	27.086556	24.786402	26.669422	24.250736	23.471112	27.648293	0.854624311	0.015724964	0.10262574	-0.115386761
ENSG00000189164	ZNF527	1.717318	1.695707	1.675721	1.690465	1.553713	1.727605	1.397197	1.209812	1.65101	0.61198155	0.103857081	0.544166718	-0.115185001
ENSG00000185813	PCYT2	46.734781	39.08915	50.331859	48.864775	56.052923	55.247387	38.027841	35.415387	35.23377	6.05E-11	0.510047074	0.201734922	-0.115078423
ENSG00000089050	RBBP9	8.949138	9.93171	9.286414	8.874203	8.076671	9.509049	8.355904	7.476156	8.580675	0.624003207	0.057991076	0.235435419	-0.115068912
NewGene 4540	--	6.003767	6.854301	6.811871	6.601572	5.422788	5.900563	5.510079	5.431	6.025497	0.890046226	-0.022853521	0.353746121	-0.114901314
ENSG00000198538	ZNF28	4.793483	4.578481	5.553997	3.720761	4.571255	3.699046	4.076288	4.475936	4.038614	0.286501381	-0.13319503	0.32845527	-0.114825993
ENSG00000167785	ZNF558	3.947622	3.926744	4.577958	3.434407	4.061874	3.673515	3.412717	4.654659	4.146784	0.584861822	0.067893988	0.292715364	-0.114778528
ENSG00000124160	NCOA5	18.391462	17.0887	17.309997	16.93913	16.78005	16.352246	15.458079	14.219536	16.330837	0.381978121	0.073154011	0.168118601	-0.1145464
ENSG00000092445	TYRO3	10.697632	9.601536	9.1722	5.187053	5.440999	5.240952	7.697872	8.188541	9.844595	4.36E-23	-0.750500991	0.268418629	-0.114461208
ENSG00000272391	POM121C	20.854713	20.340447	20.482017	20.436773	18.569696	19.48727	16.332264	16.412993	21.18856	0.319338523	0.068407353	0.2431097	-0.114384741
ENSG00000058056	USP13	6.103417	6.605288	6.470586	5.476825	4.970482	4.916881	5.783607	5.029297	5.826502	0.031562896	-0.173190379	0.192915467	-0.114315463
ENSG00000140153	WDR20	5.414881	4.41217	4.360077	5.18772	4.809453	5.261965	4.209819	3.580581	4.506777	0.030788555	0.29208165	0.477999819	-0.114261756
ENSG00000119596	YLPM1	11.610015	10.770489	12.018109	9.985162	9.275801	9.883576	9.26802	9.306441	11.053284	0.277621832	-0.090306412	0.197193492	-0.114249462
ENSG00000134283	PPHLN1	36.048836	38.894164	40.11431	35.210222	30.006101	33.938422	30.450189	31.544768	36.461056	0.155449986	-0.109329959	0.152876091	-0.114239429
NewGene 4059	--	1.456126	1.875785	1.286899	1.212282	1.008012	1.229229	1.443207	1.106221	1.186738	0.46628808	-0.284099524	0.760696683	-0.114105161
ENSG00000111490	TBC1D30	4.007891	4.384766	4.037931	4.412568	3.960182	4.58391	3.997729	4.217337	4.344142	0.420484028	0.115074222	0.381258248	-0.114008372
ENSG00000178700	DHFR2	1.383752	1.430745	0.86315	1.046242	1.099265	0.892663	0.919997	0.816072	1.51851	0.699470498	-0.120841081	0.707308955	-0.113961128
ENSG00000157227	MMP14	26.734766	23.5864	22.621087	12.400281	12.099559	13.607862	19.94948	21.914621	21.196585	6.12E-24	-0.786201352	0.229699496	-0.113918821
ENSG00000122692	SMU1	11.444486	12.021551	11.226134	11.210593	10.579469	10.978006	10.229975	10.016503	9.78852	0.398109981	0.064878459	0.15103603	-0.113843757
ENSG00000149480	MTA2	58.021683	58.114902	57.664547	45.80747	45.606437	45.309983	49.183298	49.331156	51.581211	0.000114454	-0.200145844	0.067119073	-0.113768929
ENSG00000163930	BAP1	20.703029	20.566021	18.679198	17.361933	18.067678	17.881367	16.649365	16.357082	19.432537	0.969143463	-0.004302168	0.163480787	-0.113604167
ENSG00000171132	PRKCE	1.254658	0.875333	1.281518	1.117764	1.244582	1.109583	1.146226	1.126614	1.479449	0.621703208	0.126236197	0.621328805	-0.113569181
ENSG00000198208	RPS6KL1	1.978077	1.841964	2.366555	1.079097	1.28465	1.41151	2.112824	1.687753	1.582168	0.1881211	-0.257003894	0.574216766	-0.113507507
ENSG00000167186	COQ7	10.708319	10.875194	10.057035	9.653243	10.040339	9.814212	9.147968	8.609775	9.802352	0.757863016	0.038441372	0.271961287	-0.113455352
ENSG00000170113	NIPA1	8.149288	9.1312	10.166266	7.599167	7.011705	7.773832	7.898274	7.460142	7.784656	0.028810457	-0.191837312	0.24512462	-0.113416453
ENSG00000198919	DZIP3	9.735236	10.153949	9.555027	8.59065	8.164936	8.117917	8.626027	7.641214	9.193147	0.299603606	-0.08946844	0.207090962	-0.113395478
ENSG00000015171	ZMYND11	12.911621	13.551042	14.000189	16.400243	11.67106	12.210242	11.895905	10.990076	12.439006	0.656944194	0.051617176	0.208323769	-0.113393643
ENSG00000215193	PEX26	5.612867	6.194604	5.249758	5.131269	6.088521	6.060804	7.092385	4.827867	6.197033	0.521649402	0.05490049	0.166630448	-0.113246144
ENSG00000118946	PCDH17	0.56032	0.385089	0.355922	0.095827	0.199072	0.133671	0.267963	0.308629	0.436493	0.000152042	-1.414475351	0.773294594	-0.113242497
ENSG00000079246	XRCC5	200.773158	187.911217	192.413645	155.250932	163.342029	160.12974	163.591172	169.733571	163.224006	0.001623874	-0.165149808	0.083265866	-0.113231341
ENSG00000111247	RAD51AP1	32.801783	36.879276	38.152733	33.238219	29.102182	31.838426	31.723534	28.646352	31.621828	0.730017908	-0.03640653	0.176388068	-0.113231035
ENSG00000077458	FAM76B	4.720496	5.282681	5.726263	5.60857	4.137166	5.006817	4.645705	4.355163	4.996409	0.739412421	0.058904677	0.413165245	-0.113219845
ENSG00000175110	MRPS22	39.916389	37.289991	42.39588	36.504766	35.148462	34.644087	35.639184	34.249064	36.000156	0.495471318	-0.064098867	0.18166806	-0.113118721
ENSG00000155760	FZD7	1.051204	0.880424	0.890053	0.557709	0.668082	0.476653	0.8238	0.835532	0.775138	0.027071562	-0.576917196	0.664149573	-0.113059342
ENSG00000146409	SLC18B1	39.826965	39.428539	42.675176	34.615875	35.260425	34.224117	34.065666	34.170616	37.313275	0.295281191	-0.079135431	0.130085878	-0.112954174
ENSG00000116237	ICMT	22.606898	23.005992	22.701932	21.265781	20.004083	19.813481	19.323261	18.504465	19.620788	0.92561642	0.008940758	0.111927688	-0.112891443
ENSG00000166685	COG1	12.834112	12.34679	13.831884	12.696755	12.210412	11.575821	10.51147	11.226655	12.826387	0.709252294	0.041090228	0.266288911	-0.112700514
ENSG00000119139	TJP2	71.124944	71.993074	72.631858	64.686056	67.15113	63.818688	62.927371	59.389351	70.427194	0.78452059	-0.020050929	0.107003218	-0.112545826
ENSG00000108039	XPNPEP1	16.169799	16.89386	15.063812	15.931972	15.025294	14.951764	13.73882	13.883183	14.008771	0.381354774	0.084631303	0.230616349	-0.112502638
ENSG00000183779	ZNF703	19.1187	20.364656	19.676243	16.049625	14.509168	16.816345	15.789748	16.208202	19.405916	0.030617875	-0.172537352	0.216637253	-0.112468467
NewGene 6447	--	2.129834	1.623903	2.603185	2.019583	2.296214	2.44976	1.845558	2.320985	1.512096	0.054504689	0.336203108	0.563934175	-0.112423098
ENSG00000153989	NUS1	22.026985	24.443615	25.791294	25.647861	24.72131	24.121901	20.517038	19.81389	22.409885	0.007580468	0.191252758	0.174573826	-0.112310314
ENSG00000112658	SRF	10.880532	10.783692	10.375499	8.303279	8.595698	8.994095	9.205309	8.630118	10.003411	0.06127745	-0.159023644	0.200486693	-0.111981784

Gene ID	Gene name	Ctrl-1 FPKM	Ctrl-2 FPKM	Ctrl-3 FPKM	IFNγ-1 FPKM	IFNγ-2 FPKM	IFNγ-3 FPKM	Sora-1 FPKM	Sora-2 FPKM	Sora-3 FPKM	Ctrl vs IFNγ DESeq2 FDR	Ctrl vs IFNγ DESeq2 log2FC	Ctrl vs Sora DESeq2 FDR	Ctrl vs Sora DESeq2 log2FC
ENSG00000100027	YPEL1	0.40296	0.361903	0.180161	0.28156	0.444328	0.178543	0.328819	0.327799	0.134917	0.93666192	0.053374966	0.83592916	-0.111947053
ENSG00000256162	SMLR1	6.122198	6.552845	5.699378	6.904749	8.737432	7.973866	5.429895	5.76414	4.703428	0.0000206	0.51018454	0.459390989	-0.111928405
ENSG00000110880	CORO1C	70.236992	70.865322	72.082786	55.7126	54.394211	57.639564	60.665693	60.105757	66.046688	0.00000133	-0.229895348	0.055974709	-0.111913436
ENSG00000069345	DNAJA2	18.6609	24.125683	20.222928	18.289401	20.457729	22.836159	17.786643	16.277757	17.966726	0.613759962	0.047696256	0.167347268	-0.111887397
ENSG00000062725	APPBP2	3.368289	3.605769	3.676952	4.093439	3.911412	3.560054	3.17332	2.932201	3.075494	0.010299381	0.256952023	0.317232261	-0.111823384
ENSG00000124098	FAM210B	19.673168	19.481348	19.802846	13.311933	13.060792	12.320025	16.614121	17.208594	17.400584	3.03E-12	-0.47204745	0.169408824	-0.111803504
ENSG00000113805	CNTN3	1.458149	1.303432	1.267133	0.781066	0.630492	0.694112	1.224078	1.0108	1.27361	0.0000133	-0.791547665	0.568426656	-0.111799581
ENSG00000260097	SPDYE6	0.79024	1.083362	0.828931	1.025688	0.686714	0.877151	0.663931	0.900519	0.76387	0.86382996	0.084655823	0.787232629	-0.111688603
ENSG00000184677	ZBTB40	4.993437	4.464116	4.659621	4.396196	4.265308	4.167366	4.117661	3.686863	4.315142	0.92283814	0.012395751	0.252965924	-0.111624774
ENSG00000151239	TWF1	29.140767	29.101272	30.082546	31.709448	31.414354	31.240212	25.551059	24.035759	26.470297	0.000922733	0.204113224	0.110779754	-0.111591385
NewGene_1242	--	1.527686	1.207885	1.154174	3.667943	4.781201	4.956282	1.125371	1.442846	0.832689	9.7E-26	1.792191838	0.712003105	-0.111444034
ENSG00000180979	LRRC57	4.060793	4.135258	4.274806	2.767288	3.486349	2.171265	3.274961	2.504741	2.204322	0.094002833	-0.20892207	0.427904776	-0.11137251
NewGene_1282	--	1.814241	1.582789	1.408154	1.261937	1.348982	1.608882	1.592022	1.320651	1.338432	0.983695305	0.008332634	0.710028419	-0.111341388
ENSG00000011260	UTP18	39.505162	44.990581	44.880424	34.442598	35.330644	34.149306	38.062708	37.862834	36.220764	0.034220282	-0.164563039	0.216547523	-0.111145898
ENSG00000186472	PCLO	0.197155	0.097334	0.143521	0.106239	0.095598	0.149557	0.105306	0.105019	0.152286	0.866461654	0.070447604	0.75150476	-0.111082276
ENSG00000213977	TAX1BP3	84.7072	73.643375	80.881274	46.770819	46.74088	50.120143	66.101253	69.59098	72.185303	8.65E-18	-0.586112161	0.177943529	-0.111081226
ENSG00000146872	TLK2	21.775834	21.391585	22.130075	20.782161	20.182507	20.022327	19.400273	16.996024	18.647472	0.496759967	0.054739379	0.167081661	-0.111029691
ENSG00000117139	KDM5B	17.2452	17.629957	16.353906	12.973565	12.127409	14.872681	14.225863	12.994834	14.23968	0.005160065	-0.174708343	0.111978239	-0.111015345
ENSG00000099783	HNRNPM	146.925609	147.85249	135.918195	140.994297	131.007152	131.327584	125.180243	124.345812	128.501089	0.649659821	0.031460852	0.06677628	-0.110983179
ENSG00000082458	DLG3	4.702207	4.605879	4.720283	5.766644	5.773663	5.139123	4.117005	4.062506	3.991116	0.0000211	0.397647814	0.326534448	-0.110887857
ENSG00000138376	BARD1	3.978383	5.11663	5.092984	5.18392	4.206881	4.691746	4.420603	3.791906	4.25558	0.51773497	0.09464009	0.417881344	-0.110834006
ENSG00000176087	SLC35A4	20.400221	17.977259	17.710885	14.268801	16.184765	15.878408	15.431167	15.368026	16.700873	0.513051638	-0.067878428	0.176375337	-0.110770188
ENSG00000072135	PTPN18	5.33375	5.814337	4.499642	5.497086	4.364633	4.800426	4.598574	4.419031	4.642403	0.824203497	0.038564085	0.408280915	-0.110701534
ENSG00000133816	MICAL2	0.535075	0.472858	0.644203	0.274758	0.301831	0.316933	0.471718	0.419987	0.637539	0.030799433	-0.715085238	0.748113421	-0.11060188
ENSG00000158773	USF1	45.571188	42.43935	42.887504	46.315366	49.893508	49.326687	36.453792	36.388598	39.50332	0.0000135	0.295089539	0.165739809	-0.110408416
ENSG00000204147	ASAH2B	1.733285	2.324574	2.066098	2.036197	1.803236	2.21874	1.704118	2.04885	2.073407	0.254139521	0.190867618	0.526129564	-0.11006262
ENSG00000186806	VSIG10L	1.094789	0.869511	1.23033	1.027425	0.761074	0.471713	1.060768	0.811493	0.736428	0.417377321	-0.28691712	0.737244881	-0.110007922
ENSG00000139977	NAA30	2.955195	3.191392	3.211412	3.30607	3.219147	3.385427	2.608284	2.888254	2.65932	0.008431192	0.246516395	0.341685358	-0.109841539
ENSG00000177963	RIC8A	31.261731	28.424807	27.366817	30.13255	28.658584	28.753398	24.469021	25.654887	26.642329	0.071792797	0.132404971	0.19832922	-0.109758218
ENSG00000198420	TCAF1	33.182103	32.802714	33.84358	68.895883	64.096879	65.717215	29.350034	26.756362	30.866281	5.08E-133	1.147316786	0.098657751	-0.109642921
ENSG00000163884	KLF15	7.495135	7.873705	7.596001	8.265253	7.688393	8.310203	6.561016	6.450349	6.95372	0.026040459	0.227541234	0.336582927	-0.109607138
ENSG00000168769	TET2	2.931872	2.973428	3.018791	2.929371	3.018694	2.790354	2.788658	2.509513	2.50112	0.196554529	0.127224164	0.287997496	-0.109587137
ENSG00000069509	FUNDC1	11.344562	12.482764	13.221924	7.702621	8.348268	9.196938	10.381094	12.400935	9.295807	0.002354197	-0.402294598	0.512198389	-0.109553634
NewGene_2957	--	7.709512	7.434092	7.609586	6.6077	6.871388	7.151987	6.278318	5.916667	7.590384	0.984775507	0.002975214	0.357112609	-0.109514212
ENSG00000116990	MYCL	1.899537	1.676886	1.320532	0.834431	0.943359	1.072071	1.498478	1.513673	1.162178	0.018666499	-0.577333211	0.679803373	-0.10943826
ENSG00000090432	MUL1	11.205682	10.082756	9.22854	8.788519	9.552312	9.621805	9.08764	8.753862	8.67874	0.890026614	0.021999634	0.361961188	-0.10941358
ENSG00000144426	NBEAL1	1.160651	1.296999	1.121176	1.182173	1.305858	1.185972	1.004239	0.950272	1.158254	0.245947373	0.162376601	0.455433073	-0.109292402
ENSG00000160216	AGPAT3	22.511547	23.729054	21.503601	29.398976	30.028727	30.364305	19.376159	18.512227	20.31193	1.08E-24	0.549513368	0.129236473	-0.109284008
ENSG00000143443	C1orf56	1.266395	1.320088	1.470736	0.89426	1.030422	0.92775	1.107962	1.268492	1.14453	0.225434177	-0.356343125	0.709844881	-0.109270893
ENSG00000037637	FBXO42	3.197	3.096643	3.397609	3.35571	3.191085	2.858938	2.785813	2.770257	3.137735	0.519902479	0.085837059	0.36287839	-0.109216114
ENSG00000099326	MZF1	3.460942	3.211564	3.031405	2.435453	2.325147	2.775193	2.343097	2.59911	3.516584	0.127669416	-0.261947873	0.551328258	-0.109070688
ENSG00000149474	KAT14	8.162668	8.10005	8.847346	7.515941	7.280324	7.386162	7.967092	6.9412	7.633144	0.978644657	-0.003975276	0.293661526	-0.1089031
ENSG00000181396	OGFOD3	15.678891	16.577492	14.12373	13.638647	13.365214	13.720127	13.266005	12.16583	14.684053	0.871600571	-0.019032158	0.260194252	-0.108882578
ENSG00000146477	SLC22A3	6.98853	6.284682	6.297817	5.231858	5.539217	4.906262	5.7851	5.621767	5.607457	0.142634875	-0.173668154	0.350698426	-0.108787906
ENSG00000105379	ETFB	100.331459	104.158782	88.174602	82.59698	86.120602	86.45881	88.006852	88.580769	88.496441	0.446935558	-0.070493969	0.204039839	-0.108708676
ENSG00000054523	KIF1B	5.944519	7.059282	6.937418	4.770893	5.940429	5.924098	6.230932	5.364944	6.688032	0.538258498	-0.211164341	0.258905927	-0.108578496
ENSG00000142875	PRKACB	3.284985	4.457092	3.822696	4.311882	3.840861	3.664611	3.775024	3.063491	3.369228	0.273428987	0.157964815	0.47415744	-0.108566028
ENSG00000039560	RAI14	30.685011	31.009632	33.65457	18.934818	16.813591	19.678175	26.224239	25.294277	29.020638	3.42E-33	-0.668482365	0.11572235	-0.108547037
ENSG00000145332	KLHL8	2.919184	3.095007	3.495923	2.55371	2.957649	2.904625	2.616493	2.175257	2.775284	0.764722504	-0.054077109	0.462916604	-0.108385616
ENSG00000152413	HOMER1	3.147864	3.520684	3.549728	3.135064	2.917191	2.96221	2.890036	3.039796	2.94622	0.82281537	-0.033274775	0.375167251	-0.108374426
ENSG00000129116	PALLD	9.279208	9.299593	9.723564	7.477439	7.286038	7.288906	8.439752	7.627307	8.587937	0.035131271	-0.177682639	0.232873656	-0.10836804
ENSG00000196678	ERI2	8.39232	9.133298	9.406504	8.78422	9.467929	8.36315	8.303523	7.123381	7.936277	0.278795361	0.112988843	0.303157341	-0.108361654
ENSG00000196712	NF1	10.159552	11.054916	11.88267	10.277813	9.766498	9.588968	9.506902	8.748833	10.218466	0.979402462	0.002930106	0.182179724	-0.108322248
ENSG00000166855	CLPX	15.599832	16.843024	17.060924	15.818233	15.717359	15.426623	14.911316	13.746475	14.312142	0.319021432	0.077679667	0.176915361	-0.108240704
ENSG00000117597	UTP25	5.985565	5.912769	6.290881	4.830216	5.150393	4.477626	5.306201	4.916354	5.443859	0.024948407	-0.189089717	0.195683411	-0.108149164
ENSG00000196743	GM2A	8.513338	8.947959	7.39698	20.454924	19.757591	20.434008	7.589584	6.564744	7.479896	3.63E-81	1.420332668	0.342715056	-0.10814096
ENSG00000108641	B9D1	3.657907	4.830595	4.290824	4.44578	3.799633	5.35708	4.151407	3.772119	3.550348	0.514033421	0.169318925	0.649306124	-0.108056426
ENSG00000147454	SLC25A37	1.79016	2.6647	2.032874	2.624556	2.672261	2.514862	1.949111	1.461053	1.834019	0.012873452	0.446629227	0.644669054	-0.107870975
ENSG00000005108	THSD7A	0.374936	0.487288	0.481751	1.193249	1.118061	1.089377	0.425048	0.394636	0.347698	2.5E-25	1.47574051	0.656751861	-0.107863836
ENSG00000170775	GPR37	21.451221	21.651577	22.533607	15.584633	14.368494	14.983545	19.284904	17.286009	20.639828	1.17E-09	-0.39953951	0.192091436	-0.107849948
ENSG00000023228	NDUFS1	36.328422	22.586755	31.883604	24.889802	24.834846	26.510036	32.366169	22.761659	21.090949	0.504043384	-0.045474804	0.095747761	-0.107750428
ENSG00000174446	SNAPC5	23.597017	21.68786	22.8957	22.06481	21.735376	23.122015	18.336633	22.656253	17.163216	0.36644444	0.099386923	0.409807804	-0.107736401
ENSG00000089048	ESF1	14.554095	15.970465	16.817481	12.041641	10.813363	11.278179	13.435454	13.513062	14.23332	0.0000358	-0.342601188	0.241786101	-0.107701651
ENSG00000096093	EFHC1	3.275068	4.26277	3.189945	2.523624	2.679641	2.392605	3.976913	3.42247	3.477447	0.002621142	-0.389060206	0.438192394	-0.1076612
ENSG00000185651	UBE2L3	49.232709	46.490108	46.405341	49.811476	48.906129	47.891617	41.131201	40.872163	41.684037	0.000786051	0.191750871	0.118398683	-0.107412952
ENSG00000182150	ERCC6L2	9.780146	10.794042	11.651037	9.457495	9.944248	8.845605	9.903763	8.38053	9.315163	0.689579289	-0.05066222	0.322388321	-0.107289813
ENSG00000136026	CKAP4	76.43071	73.204971	70.965859	59.528225	59.678082	61.195839	64.50322	59.544353	68.155388	0.015066332	-0.142254406	0.10950974	-0.10727284
ENSG00000101972	STAG2	24.236049	25.682405	24.982347	22.974228	23.706496	21.863061	21.197206	20.610606	23.013451	0.681847625	0.034688911	0.150474826	-0.107266288
ENSG00000164970	FAM219A	2.434346	2.627406	3.032433	2.843822	2.536306	3.16692	2.438349	2.26098	2.268663	0.152316246	0.23217886	0.523864279	-0.107074459
ENSG00000165716	DIPK1B	21.848896	19.941414	18.101517	18.598964	16.317745	17.472075	16.518081	16.642901	18.415777	0.93402599	-0.011953124	0.292606103	-0.107021182
ENSG00000219438	FAM19A5	0.400497	0.294878	0.414472	0.048924	0.028045	0.054501	0.544976	0.384716	0.274881	--	--	0.835348111	-0.106948075
ENSG00000107175	CREB3	20.779318	19.7759	19.155165	16.78031	17.498232	17.405581	17.398674	17.070595	17.516739	0.571625226	-0.060735513	0.255818801	-0.106915545
ENSG00000162378	ZYG11B	3.254434	3.618212	3.029776	3.840878	4.02467	4.129634	3.083872	2.363188	3.265164	0.00000484	0.421908518	0.421199952	-0.106863398
ENSG00000172728	FUT10	3.735342	4.305566	3.762631	3.286126	3.213426	3.449586	3.753849	3.00547	3.489118	0.519292731	-0.09725531	0.460770053	-0.106840769

Gene ID	Gene name	Ctrl-1 FPKM	Ctrl-2 FPKM	Ctrl-3 FPKM	IFNγ-1 FPKM	IFNγ-2 FPKM	IFNγ-3 FPKM	Sora-1 FPKM	Sora-2 FPKM	Sora-3 FPKM	Ctrl vs IFNγ DESeq2 FDR	Ctrl vs IFNγ DESeq2 log2FC	Ctrl vs Sora DESeq2 FDR	Ctrl vs Sora DESeq2 log2FC
ENSG00000102606	ARHGEF7	10.508856	11.01999	12.0867	10.939532	10.319236	10.272043	11.678362	9.30947	10.392544	0.67896931	0.041659712	0.261328306	-0.106776269
ENSG00000197860	SGTB	1.646449	1.541696	1.63256	1.839908	1.559934	1.586476	1.486865	1.552881	1.210744	0.271853665	0.177361548	0.569716084	-0.106701561
ENSG00000130723	PRRC2B	25.101845	20.962867	23.466422	21.776157	19.314574	21.56325	20.974631	17.377052	22.569095	0.827672643	-0.019339221	0.160410421	-0.106682411
ENSG00000198218	QRICH1	29.522525	27.830302	27.395008	24.618378	24.209406	24.941559	24.224478	23.077837	25.418526	0.514311884	-0.053554363	0.156318915	-0.106631119
ENSG00000148606	POLR3A	8.621347	8.448714	8.174567	8.303988	7.532152	6.944805	7.151944	6.87389	7.980187	0.987640184	-0.0019711	0.20553812	-0.106560983
ENSG00000162522	KIAA1522	18.115551	16.735817	16.538414	14.544657	15.19604	15.186462	14.397121	13.664925	16.913069	0.61845816	-0.044863151	0.23012149	-0.106501038
ENSG00000143502	SUSD4	3.553266	3.333134	2.65895	2.112875	2.427228	2.226561	2.436283	3.050226	3.229888	0.167842446	-0.261960064	0.574547576	-0.106427896
ENSG00000179820	MYADM	31.151652	29.338891	28.33583	17.990433	19.492126	17.024001	23.811585	25.579406	29.513944	4.28E-16	-0.548362458	0.212045312	-0.106360116
ENSG00000145868	FBXO38	11.376286	11.822407	11.101625	10.403418	10.189478	9.085137	9.42684	9.542901	10.184577	0.507315693	-0.069580827	0.234201717	-0.106348042
ENSG00000109618	SEPSECS	5.810696	7.497528	7.676782	7.272451	7.209693	7.053721	6.491615	6.257502	6.226174	0.121315713	0.180393485	0.402277298	-0.106310771
ENSG00000204231	RXRB	25.141702	24.352119	23.245531	21.253909	22.382057	23.106808	20.387778	20.186967	22.77561	0.861306934	0.018787019	0.179911617	-0.106112656
ENSG00000164932	CTHRC1	4.1354	4.118763	4.573383	9.986317	8.25341	8.42463	3.834179	3.485047	3.623238	5.89E-18	1.217645249	0.615532248	-0.106022514
ENSG00000162735	PEX19	29.065533	28.598513	27.235391	23.949331	24.778711	24.796685	24.11726	24.472527	25.13131	0.501970262	-0.054230541	0.149283259	-0.105894889
ENSG00000061938	TNK2	4.159804	4.417193	4.056126	5.615261	5.065682	6.416656	3.859855	3.430609	3.397896	1.39E-08	0.56372581	0.396740104	-0.105863411
ENSG00000188001	TPRG1	0.595761	0.653613	0.619129	0.380552	0.32626	0.312148	0.521856	0.549565	0.589833	0.311042176	-0.4930345	0.821342163	-0.105738596
NewGene 3281	--	0.709119	0.769192	0.749136	0.572472	0.579161	0.558201	0.701391	0.746039	0.424109	0.94471444	0.034863061	0.795593736	-0.105564141
ENSG00000187097	ENTPD5	12.240877	10.48833	10.613903	9.580595	8.195092	8.526746	10.5203	9.134963	11.2751	0.060476821	-0.17161605	0.296141108	-0.105534673
ENSG00000178802	MPI	11.051729	10.983555	10.945028	9.842701	10.520107	9.857377	10.395783	9.92138	10.245647	0.449384714	-0.066962272	0.214024197	-0.105532399
ENSG00000068097	HEATR6	9.324993	8.414929	8.452276	8.513065	8.022759	7.496635	7.758864	7.573997	7.650924	0.891751387	0.018553964	0.305923949	-0.105446366
ENSG00000109606	DHX15	124.326164	128.927017	130.605072	114.414169	112.612534	113.798172	110.524078	108.952026	115.170143	0.717885906	-0.024080608	0.073594472	-0.105346365
ENSG00000129250	KIF1C	39.60019	38.72069	35.169814	37.299385	35.548403	37.532836	32.440067	31.139688	35.297172	0.135280474	0.098616298	0.136563484	-0.105333172
ENSG00000041357	PSMA4	94.130635	96.71571	74.199866	113.454713	93.175598	84.399711	85.169161	97.397654	82.329221	3.9E-12	0.339019964	0.189031602	-0.105290016
ENSG00000258429	PDF	8.886435	9.098211	9.459341	8.432743	7.87225	9.586932	7.445503	8.115631	8.351236	0.722654818	0.06596645	0.493480799	-0.105288426
ENSG00000151151	IPMK	1.765978	1.921654	1.911591	1.784066	1.833781	1.879825	1.7026	1.478689	1.709805	0.423642727	0.12159391	0.480176038	-0.105231849
ENSG00000142677	IL22RA1	1.566355	1.14848	1.506008	4.529099	4.31598	4.698544	1.430841	1.239571	0.992823	8.35E-36	1.826537092	0.709844881	-0.104989105
ENSG00000119285	HEATR1	17.11909	18.707128	18.464653	16.256576	14.099102	15.436834	15.11345	15.009374	17.182305	0.205132094	-0.095446909	0.160186773	-0.104600491
ENSG00000186567	CEACAM19	1.500376	1.49032	1.238811	1.466415	1.255783	1.642598	0.992474	0.946058	1.370477	0.520389631	0.212097846	0.752751354	-0.104541698
ENSG00000189306	RRP7A	31.862242	31.889328	29.467762	26.827652	25.135602	27.470514	27.361798	26.189439	28.600498	0.175966099	-0.099702738	0.136407394	-0.104518827
ENSG00000065060	UHRF1BP1	11.125173	11.792426	11.838233	13.499212	13.363142	12.703676	10.397385	9.075023	10.840067	3.59E-09	0.335514815	0.180011984	-0.104460608
ENSG00000114737	CISH	1.500172	1.180109	1.136035	3.183149	3.121892	3.641445	1.272249	1.068389	0.999324	3.97E-16	1.520749145	0.743674908	-0.104396255
ENSG00000008086	CDKL5	0.739164	0.955	0.934823	0.835116	0.670591	0.653162	0.89816	0.69896	0.733977	0.342776859	-0.172804012	0.572140493	-0.104316718
ENSG00000196597	ZNF782	1.539271	1.909927	1.886552	2.05805	1.96005	1.833866	1.514915	1.722882	1.644157	0.120809181	0.275650778	0.597911862	-0.104293136
ENSG00000112742	TTK	24.560597	24.504975	23.616232	18.274123	17.700114	16.417639	20.240828	21.838782	21.614632	0.00000204	-0.321325435	0.169820785	-0.104281313
ENSG00000173065	FAM222B	11.55504	11.043872	12.317051	8.778238	8.650104	8.833636	9.758949	9.359089	11.902928	0.003483487	-0.25790021	0.355561058	-0.104134261
ENSG00000163635	ATXN7	1.040739	0.953377	0.16304	2.048007	1.735419	1.539704	0.14531	0.370517	1.637173	0.09894166	1.445661691	0.944041721	-0.104118608
ENSG00000114520	SNX4	30.187939	32.389824	32.484882	31.231301	29.916479	29.584387	27.61989	26.387291	28.957476	0.338848526	0.073411268	0.171764658	-0.104066338
ENSG00000105176	URI1	22.494741	25.376637	23.814877	20.301968	18.324441	19.769721	21.218011	20.038612	21.155662	0.106608803	-0.122763251	0.194672582	-0.103922806
ENSG00000113319	RASGRF2	2.252384	2.749641	3.244494	1.059233	1.343672	1.079332	2.705703	2.712113	2.969594	5.78E-13	-1.056071871	0.507491852	-0.103884225
ENSG00000184792	OSBP2	2.225552	2.885095	2.082249	2.531578	2.942612	2.485702	2.306612	1.591511	2.223669	0.33459168	0.169906023	0.582359336	-0.103848454
ENSG00000165533	TTC8	2.893833	1.976395	1.644095	1.187363	1.666007	0.746211	0.95794	0.81813	2.095314	0.406323805	-0.19150814	0.628989328	-0.103820395
ENSG00000115368	WDR75	19.588438	21.325139	27.848019	19.988956	21.071083	18.340162	18.143197	18.665306	18.602075	0.439654844	-0.069449515	0.221512525	-0.103818763
ENSG00000102359	SRPX2	0.501106	0.504362	0.747939	0.452188	0.503383	0.372003	0.447066	0.495376	0.805171	0.344912491	-0.276201955	0.717924881	-0.10374344
ENSG00000273045	C2orf15	3.311802	3.730071	3.557533	3.08247	3.186539	3.400444	3.19164	2.715209	3.030007	0.907434405	-0.032950653	0.636695873	-0.10372587
ENSG00000214290	COLCA2	9.395695	8.503851	7.688201	6.431417	5.856497	4.801917	6.592764	7.005216	8.56299	0.003207176	-0.456091014	0.545995308	-0.10369752
ENSG00000174840	PDE12	4.023584	4.5612	4.503124	4.88254	4.868007	4.82676	3.991457	3.513852	4.024373	0.0000973	0.308550873	0.308262976	-0.103695331
ENSG00000138035	PNPT1	16.40792	17.126472	18.070714	17.172494	15.591914	16.30182	14.948684	14.391568	15.724942	0.377441966	0.072376989	0.184087145	-0.103549752
ENSG00000077684	JADE1	11.388349	11.731445	12.76473	8.614135	8.313912	8.897841	11.034481	10.583864	11.874263	0.00000273	-0.366608534	0.248013562	-0.103470274
ENSG00000067365	METTL22	8.496592	7.159242	8.21387	8.111398	6.871801	6.346253	8.047304	6.92676	7.655446	0.675401691	0.059486703	0.395844631	-0.10333277
ENSG00000148331	ASB6	9.386151	9.551545	9.358448	7.899668	7.922383	8.527918	7.890886	7.919505	9.2421	0.44371264	-0.07891056	0.321829162	-0.103243252
ENSG00000063176	SPHK2	56.29137	59.185373	50.938493	49.23078	57.582122	56.049689	46.835452	46.929334	47.566325	0.548529858	0.058857391	0.18166806	-0.103205163
ENSG00000086758	HUWE1	15.491376	14.813958	17.753556	18.625819	15.235825	15.324275	15.371743	14.149002	18.59727	0.120809181	0.10337797	0.160410421	-0.103181379
ENSG00000112079	STK38	10.223272	10.378668	11.176538	9.062355	10.001747	9.09398	9.102689	8.977734	9.667753	0.832151977	-0.026747196	0.2709489	-0.103111902
ENSG00000169230	PRELID1	127.083129	125.363668	118.188268	111.935432	98.993263	107.238484	95.811207	105.602614	117.430063	0.398332231	-0.059645828	0.187277574	-0.103018468
ENSG00000106348	IMPDH1	9.91165	10.980308	11.510892	9.051776	8.395047	9.593204	8.701044	8.569846	11.223597	0.373503341	-0.102572819	0.385262058	-0.103017336
ENSG00000232434	AJM1	0.83788	0.796076	0.555853	0.949208	0.880121	0.833084	0.571785	0.73008	0.607609	0.088395786	0.425382798	0.737244881	-0.103005381
ENSG00000151150	ANK3	2.732788	3.068959	3.715219	2.429226	2.665088	2.508101	3.734742	3.180952	3.846226	0.0000873	-0.398482663	0.353461651	-0.102908511
ENSG00000139324	TMTC3	3.743198	4.523071	4.264365	3.157663	3.263554	3.118985	3.692296	3.478006	3.716627	0.021282101	-0.238918618	0.354560354	-0.102822263
ENSG00000085231	AK6	14.11978	14.592962	14.720214	14.613564	15.264644	14.529615	13.401525	14.026937	12.071174	0.180023997	0.14356619	0.393285547	-0.102796554
ENSG00000135775	COG2	30.964906	29.122655	27.456442	28.097708	27.981122	29.030476	25.87022	24.749541	25.780731	0.150274104	0.111892012	0.204575784	-0.102770266
ENSG00000104731	KLHDC4	8.342455	8.709165	9.917468	8.821722	6.667707	8.072513	8.844514	8.854654	9.238652	0.515917769	-0.095203783	0.392090146	-0.102747436
ENSG00000113790	EHHADH	5.111268	5.753606	4.727351	7.354205	6.739913	6.959129	5.215753	4.264884	4.192345	2.08E-09	0.570308431	0.477833789	-0.102590686
ENSG00000214753	HNRNPUL2	52.101185	51.825901	52.601891	50.70657	53.336605	51.075661	46.90007	42.956928	46.939777	0.015834204	0.134627415	0.09301856	-0.102474141
ENSG00000181666	HKR1	14.021465	13.763538	13.152043	10.735665	9.516253	9.84981	12.23466	12.400003	11.313061	0.002362258	-0.273201162	0.307724885	-0.102326381
ENSG00000105996	HOXA2	1.206488	1.402688	1.007358	0.886752	0.868259	0.835941	0.767322	0.869416	1.544552	0.337730891	-0.33386971	0.793434567	-0.102214075
ENSG00000184007	PTP4A2	28.168879	29.636123	29.520457	35.763998	34.576866	33.414738	25.856814	25.579053	26.917982	2.61E-11	0.358017177	0.140962509	-0.102149843
ENSG00000064199	SPA17	2.244791	1.154273	2.121194	1.403278	0.951955	1.797739	1.905642	1.682413	1.464036	0.129452101	-0.424248431	0.723434152	-0.102126987
ENSG00000187860	CCDC157	0.629155	0.62826	0.708036	0.480364	0.339134	0.442977	0.562345	0.425089	0.671096	0.500269606	-0.323275051	0.81774506	-0.102114561
ENSG00000101361	NOP56	138.720355	135.735926	144.968856	135.47084	116.823838	127.194157	119.007158	117.179026	122.494932	0.915061926	-0.01008449	0.105790058	-0.10202792
ENSG00000130449	ZSWIM6	1.679024	1.855589	1.594435	1.460737	1.699614	1.503405	1.428053	1.522079	1.529635	0.95987988	0.011925585	0.538545276	-0.1019452
ENSG00000180530	NRIP1	16.81423	17.188906	17.432846	14.119768	14.652546	14.515977	14.832565	14.702034	15.501421	0.12170583	-0.100848418	0.122228347	-0.101879923
ENSG00000141429	GALNT1	70.86253	74.108996	72.498522	75.737211	77.297251	77.320862	68.256956	59.707186	61.872229	0.000907328	0.187018366	0.128246521	-0.101858414
ENSG00000011258	MBTD1	15.269581	15.910572	17.049291	13.169693	13.885962	12.677258	14.359002	13.164509	15.081979	0.089376043	-0.127598043	0.182645583	-0.101849325
ENSG00000147044	CASK	5.237436	4.29185	4.761371	3.49613	3.486538	4.030487	4.144008	4.271199	4.064007	0.450922593	-0.084040962	0.321973535	-0.101741474
ENSG00000139644	TMBIM6	311.898148	305.99884	303.386654	299.371092	303.879867	299.207595	262.767839	269.93436	281.027259	0.03364617	0.104166756	0.078069555	-0.101729988

Gene ID	Gene name	Ctrl-1 FPKM	Ctrl-2 FPKM	Ctrl-3 FPKM	IFNγ-1 FPKM	IFNγ-2 FPKM	IFNγ-3 FPKM	Sora-1 FPKM	Sora-2 FPKM	Sora-3 FPKM	Ctrl vs IFNγ DESeq2 FDR	Ctrl vs IFNγ DESeq2 log2FC	Ctrl vs Sora DESeq2 FDR	Ctrl vs Sora DESeq2 log2FC
ENSG00000277972	CISD3	13.304705	13.930049	11.545518	11.926472	11.876358	12.797332	12.423944	12.146961	9.195508	0.629632657	0.064153601	0.510654077	-0.101687883
ENSG00000148719	DNAJB12	8.404874	8.150172	9.137857	6.841085	6.559776	7.358369	6.980501	7.121311	8.542751	0.146186746	-0.142713911	0.343662927	-0.101618516
ENSG00000164124	TMEM144	7.407309	8.107061	7.826689	6.872544	6.198211	7.062185	6.422134	6.300423	7.537659	0.67896931	-0.052559837	0.359311157	-0.101613007
ENSG00000156170	NDUFAF6	15.896186	16.389345	17.792831	32.280118	29.279305	28.998678	14.98108	14.73782	13.665048	1.07E-27	0.866198139	0.418765609	-0.101494329
ENSG00000196411	EPHB4	41.567135	36.425015	34.269605	39.494252	42.047627	42.377348	30.69198	31.397652	36.996919	0.000123201	0.290669307	0.277301941	-0.101361162
ENSG00000130699	TAF4	7.703379	7.36058	8.387129	8.187923	7.390112	6.914101	6.509115	6.032548	8.330787	0.582645755	0.064605614	0.386070372	-0.101345829
ENSG00000168884	TNIP2	17.704121	17.586012	15.905869	17.317069	14.875212	17.336353	14.138002	15.150095	14.905022	0.441345897	0.09075308	0.337797826	-0.101285846
ENSG00000157778	PSMG3	41.454846	39.756286	33.361441	38.011169	38.215075	41.071812	34.699693	36.161882	37.279656	0.072704831	0.151191885	0.260304756	-0.10121447
ENSG00000272968	RBAK-RBAKDN	6.479108	5.4565	5.925908	5.851483	5.005387	5.024158	5.404198	4.784429	5.515278	0.920138162	-0.027446416	0.619241979	-0.101174875
NewGene 4528	--	7.468883	7.645343	9.922941	8.483022	8.676516	8.106341	7.969165	6.181496	8.031123	0.064755341	0.162493517	0.27148907	-0.101063457
ENSG00000136478	TEX2	13.233851	12.569965	12.056793	13.664437	12.982871	13.483959	11.786645	10.44983	11.488933	0.000685516	0.23324854	0.230142168	-0.101046952
NewGene 2287	--	2.182397	1.329594	1.534131	1.355325	0.715501	0.770045	0.869057	0.828311	1.086135	0.239642488	-0.290775093	0.661959846	-0.100996206
ENSG00000112902	SEMA5A	0.10102	0.151165	0.163363	0.017319	0.017189	0.029818	0.13064	0.123182	0.129634	0.000000304	-2.456331752	0.814652612	-0.100988237
ENSG00000153561	RMND5A	18.648087	19.182482	19.649242	15.720994	16.943285	16.41539	16.958647	16.245432	17.054762	0.281946517	-0.079797558	0.139923349	-0.100844976
ENSG00000198947	DMD	3.886876	3.831543	4.720569	3.642431	3.744592	3.454422	3.773545	3.794292	3.96279	0.654890292	-0.069064515	0.456278159	-0.10083008
ENSG00000269858	EGLN2	33.924317	33.70078	31.247129	31.296364	25.688536	32.124444	26.179448	27.592497	32.1595	0.95852397	0.007204386	0.288641203	-0.100536518
ENSG00000184678	HIST2H2BE	1.291651	0.956386	0.856263	0.926097	0.851714	0.887815	0.761841	0.992343	0.970788	0.855435908	-0.076705293	0.77529396	-0.100497056
ENSG00000178202	KDELC2	11.043383	11.811619	13.127492	12.27135	11.465629	12.005216	10.164783	10.000339	11.389436	0.134358578	0.136010363	0.306933973	-0.100425821
ENSG00000113569	NUP155	16.486099	17.922712	17.363308	14.75565	14.346393	14.483414	15.211367	14.682024	16.023881	0.096254084	-0.105459005	0.135142154	-0.100392656
ENSG00000175029	CTBP2	2.729097	2.862511	3.129778	1.53139	1.479419	1.207297	2.694798	2.400611	2.576044	3.95E-08	-0.733753643	0.469035484	-0.100332012
ENSG00000178297	TMPRSS9	0.360852	0.540725	0.438858	0.331971	0.320736	0.308572	0.38547	0.340346	0.446863	0.467011414	-0.313232456	0.806768105	-0.099859571
ENSG00000075975	MKRN2	13.275311	13.149709	12.389635	13.741429	12.960872	12.164019	11.772832	10.952402	11.739036	0.177542313	0.127211795	0.283389346	-0.099814395
ENSG00000143653	SCCPDH	32.655716	31.529516	31.513958	32.447445	31.795158	31.169842	28.077253	27.720232	27.931211	0.029299227	0.142553157	0.186777256	-0.099746052
ENSG00000164402	8-Sep	6.997739	8.173611	7.325127	9.436146	8.939362	8.934019	6.631869	5.982628	6.721489	1.27E-09	0.461407841	0.318726099	-0.099655892
ENSG00000198093	ZNF649	4.985649	5.1055	5.65066	3.877914	3.992351	4.126341	4.131636	5.264327	4.597855	0.064971953	-0.241941734	0.520620144	-0.099183285
ENSG00000081307	UBA5	17.61378	20.881292	19.268733	19.898885	17.72279	18.6371	17.460412	15.712428	17.201985	0.040090816	0.161397205	0.236866369	-0.099141911
ENSG00000009844	VTA1	16.538384	16.65524	15.753355	14.71944	14.161053	16.47351	13.701783	13.064905	14.405819	0.777864361	-0.02441537	0.163843958	-0.099131474
ENSG00000116750	UCHL5	24.388161	21.740718	23.662727	29.796966	23.211187	23.28096	22.087093	23.009874	21.336587	0.184810289	0.116732272	0.239355301	-0.099099801
ENSG00000213462	ERV3-1	3.288089	3.17388	2.662134	3.121128	2.639717	3.496249	2.688115	2.669173	2.623631	0.38260201	0.169533824	0.583562194	-0.099088359
ENSG00000133313	CNDP2	23.598117	25.211825	23.12453	26.543428	26.594137	24.8188	21.12664	21.807475	21.905186	0.002125624	0.186060459	0.167564792	-0.099078622
ENSG00000138660	AP1AR	9.127225	10.691298	10.918492	10.229027	11.339994	10.407971	8.424926	8.463496	9.961583	0.070287972	0.194524634	0.405047597	-0.098914373
ENSG00000157045	NTAN1	23.017108	21.665167	24.703202	15.900676	18.134279	15.057546	22.822444	19.892411	18.962208	0.001920897	-0.346958215	0.421141076	-0.098648623
ENSG00000171984	SHLD1	1.837674	1.698216	1.398221	0.996533	0.904459	1.723064	1.960589	1.014504	1.198515	0.589170519	-0.243970449	0.810788314	-0.098548809
ENSG00000162852	CNST	8.752028	8.778325	9.734327	6.600915	6.809275	6.731273	9.166378	7.273348	8.429464	0.000290833	-0.291975682	0.339292514	-0.098462028
ENSG00000165480	SKA3	7.0935	7.716124	7.881173	5.705976	5.529417	4.667789	6.955458	6.737225	6.347348	0.001088612	-0.365429139	0.416212287	-0.09839312
ENSG00000100201	DDX17	133.973997	134.307539	132.642243	134.705978	129.35146	124.154107	110.812179	105.514307	110.053587	0.381062999	0.051777934	0.089466734	-0.098319853
ENSG00000164032	H2AFZ	259.217865	271.654388	256.166595	201.267105	213.101303	213.790619	221.662338	221.103333	247.213928	0.002550094	-0.17730607	0.134931739	-0.098293941
ENSG00000053900	ANAPC4	20.40649	21.144096	22.88715	21.431306	20.938689	19.839675	18.969534	17.663255	20.127335	0.2851926	0.091880226	0.251866455	-0.098238448
ENSG00000133028	SCO1	7.773562	8.199734	7.837299	8.844603	8.911758	8.90801	7.090951	6.967187	8.23107	0.0000383	0.252748934	0.189397295	-0.098110101
ENSG00000204599	TRIM39	9.185283	14.307998	14.078772	14.49695	9.07152	13.378519	12.371136	11.657526	12.307761	0.368838768	0.103895027	0.361901547	-0.098104721
ENSG00000065361	ERBB3	56.154449	57.25515	60.928451	71.702476	69.562703	66.476587	51.418667	49.401573	53.18817	1.3E-13	0.345062228	0.090929302	-0.098046964
ENSG00000129680	MAP7D3	1.134756	1.178886	1.285948	0.545833	0.855525	0.804314	0.994818	1.105396	1.047866	0.015316256	-0.550599543	0.644669054	-0.097957601
ENSG00000183888	SRARP	3.911569	3.990232	4.420971	1.613635	1.861007	2.380273	3.1504	3.412227	4.25613	4.08E-09	-0.919833463	0.543802249	-0.097953374
ENSG00000182095	TNRC18	19.588935	19.952541	19.001715	19.339566	18.022715	20.271204	17.964086	15.946183	19.969455	0.082399032	0.133488459	0.298696605	-0.097846651
ENSG00000166261	ZNF202	4.099269	4.199945	4.233257	3.734057	3.242463	3.929931	3.591989	3.557764	3.896289	0.626808832	-0.077093622	0.451713207	-0.0978282
ENSG00000176894	PXMP2	16.383191	15.217109	14.245828	15.782268	14.657692	14.729716	13.404686	13.147947	13.149772	0.352138739	0.130315802	0.486253027	-0.097819974
ENSG00000148737	TCF7L2	7.735409	8.627137	8.252031	9.688311	8.270229	9.505554	7.193576	7.262008	8.131757	0.000100351	0.316392409	0.326220339	-0.097818722
ENSG00000132763	MMACHC	2.867564	2.621864	3.228773	2.37163	2.301041	2.460865	2.605248	2.302014	2.74562	0.338147486	-0.139820131	0.498161157	-0.097415619
ENSG00000148291	SURF2	22.016357	19.369938	22.046806	14.634141	12.741482	16.419258	19.131836	17.535769	18.979107	0.00280429	-0.384351437	0.44597892	-0.097343374
ENSG00000138617	PARP16	4.818866	3.389323	4.008704	3.425887	3.284296	3.016247	3.677843	3.632157	3.625717	0.303226824	-0.192753979	0.594132081	-0.097302021
ENSG00000113161	HMGCR	55.544965	53.220464	48.465959	69.059004	70.373543	68.668172	47.714891	44.007872	44.722105	5.12E-21	0.56382171	0.223223065	-0.097298664
ENSG00000163900	TMEM41A	9.090599	9.362263	10.070563	9.656657	9.309673	9.757218	8.624075	8.135806	8.092453	0.093823585	0.163132562	0.368229562	-0.097231817
NewGene 3404	--	1.249235	1.627503	1.230197	1.604618	1.145052	1.349957	1.046393	1.529897	1.130789	0.714259576	0.141070035	0.789955927	-0.097097438
ENSG00000188290	HES4	8.119252	5.72228	5.735692	5.643377	5.013352	6.58915	5.430236	5.93849	5.944196	0.897008801	-0.038692925	0.672794514	-0.097029905
ENSG00000160051	IQCC	1.513998	1.662537	1.720605	1.608695	1.263335	1.519153	1.521744	1.357259	1.430505	0.999202724	0.000561093	0.71749733	-0.096917656
ENSG00000130766	SESN2	8.446852	8.079497	7.752113	7.81555	8.628045	8.040545	7.04105	6.676489	7.599935	0.116449268	0.159547308	0.351089451	-0.096913862
ENSG00000198862	LTN1	11.802776	12.600688	13.247674	12.109481	11.777423	11.489028	11.03735	10.374622	12.02787	0.507860742	0.054222172	0.221777591	-0.096656717
ENSG00000102908	NFAT5	6.543829	7.2308	8.239895	7.380293	7.322568	6.968566	6.483988	5.95126	7.184838	0.083126074	0.163351607	0.368124973	-0.096567736
ENSG00000073464	CLCN4	2.100132	2.145482	2.13929	1.595358	1.612788	1.595063	1.746319	1.827998	2.023047	0.024319897	-0.276935932	0.473090791	-0.096482114
ENSG00000185222	TCEAL9	35.175733	37.973297	42.096272	35.658842	34.104439	35.137633	34.464817	34.309571	34.132502	0.973440947	0.004806656	0.339590275	-0.096378822
ENSG00000144848	ATG3	23.80775	25.023069	25.888887	25.065251	26.039152	26.756947	21.302946	22.940259	22.27027	0.062630419	0.160437412	0.327681238	-0.096308454
ENSG00000110536	PTPMT1	60.232272	54.627784	53.260415	45.521011	45.177118	51.443727	48.825856	50.756236	50.482934	0.520917582	-0.064819738	0.272465042	-0.096265073
ENSG00000204954	C12orf73	9.591724	12.133962	11.658944	10.165439	9.358351	9.952898	10.063018	9.72184	9.381	0.835667047	-0.039403048	0.528959042	-0.096148891
ENSG00000160953	MUM1	18.466836	17.769379	18.037832	15.883713	13.826265	14.440671	15.509654	14.686502	18.224447	0.050815409	-0.147001436	0.295573243	-0.096089841
ENSG00000157500	APPL1	5.701752	5.925788	6.373729	5.681844	5.035659	5.482109	5.159669	4.804752	5.846165	0.964192542	-0.006225996	0.352957841	-0.096053504
ENSG00000143702	CEP170	16.869319	18.586649	19.773489	16.682435	15.817622	15.927083	16.978707	15.430257	18.170424	0.696094327	-0.039410153	0.279289157	-0.095918132
ENSG00000184445	KNTC1	20.744418	21.389477	22.308203	22.798537	18.956722	19.909909	20.117645	17.538421	20.996143	0.784458984	0.025801113	0.181559547	-0.095829203
ENSG00000106780	MEGF9	8.62268	8.530487	8.912534	8.571636	8.316084	9.002212	7.927673	6.653946	8.339452	0.07267193	0.138235421	0.311453078	-0.095673975
ENSG00000135801	TAF5L	13.26784	15.289126	15.683691	15.181127	14.64192	14.96734	12.668311	11.262706	14.370144	0.071778403	0.160978899	0.380526165	-0.095473594
ENSG00000185513	L3MBTL1	0.997735	0.819804	0.82665	0.903412	0.977987	0.683564	1.016383	0.718549	0.856092	0.765488451	0.090487435	0.720002466	-0.095451581
NewGene 5166	--	1.257437	1.038291	1.356117	0.561638	0.985569	0.791877	1.250784	1.129665	0.876938	0.041270746	-0.524047877	0.719882292	-0.095435955
ENSG00000100612	DHRS7	38.404937	34.812947	38.209322	29.479789	25.286955	26.853279	32.564184	31.778822	32.904244	0.002824225	-0.237940691	0.298936485	-0.09542734
ENSG00000173928	SWSAP1	1.712833	1.779975	1.597487	1.616902	1.873089	1.621889	1.300364	1.496685	1.649286	0.605412914	0.15899158	0.745131482	-0.095377657
ENSG00000095015	MAP3K1	3.574154	3.830884	3.852932	3.493937	3.194641	3.0791	3.538557	3.085049	3.255859	0.637975895	-0.058721781	0.389916539	-0.09537422

Gene ID	Gene name	Ctrl-1 FPKM	Ctrl-2 FPKM	Ctrl-3 FPKM	IFNγ-1 FPKM	IFNγ-2 FPKM	IFNγ-3 FPKM	Sora-1 FPKM	Sora-2 FPKM	Sora-3 FPKM	Ctrl vs IFNγ DESeq2 FDR	Ctrl vs IFNγ DESeq2 log2FC	Ctrl vs Sora DESeq2 FDR	Ctrl vs Sora DESeq2 log2FC
ENSG00000135049	AGTPBP1	3.997366	4.095805	4.266665	2.746588	2.933516	2.876863	3.651033	3.325323	3.781542	0.000771931	-0.371704649	0.435278166	-0.095154455
ENSG00000172667	ZMAT3	3.188884	3.065357	3.450779	2.447715	2.169803	2.477827	2.850103	2.680345	3.038304	0.001897793	-0.305167841	0.364527914	-0.095125504
ENSG00000137055	PLAA	13.95541	13.881389	14.853126	12.068511	11.043482	11.942901	12.846731	12.199605	12.582523	0.064577534	-0.143037755	0.254965719	-0.095003339
ENSG00000163510	CWC22	18.68966	18.659695	20.689625	19.235746	16.26407	17.134523	17.171633	17.667719	16.494577	0.814736115	0.025387313	0.289821407	-0.094933861
ENSG00000102931	ARL2BP	5.561809	6.484683	5.626543	6.049683	4.527976	6.070654	5.344662	4.432073	5.6617	0.700683853	0.075435647	0.550134847	-0.094886968
ENSG00000041515	MYO16	0.2546	0.167194	0.257892	0.231215	0.183434	0.220015	0.16042	0.200516	0.238714	0.92635579	0.046905422	0.81637566	-0.094817038
ENSG00000139842	CUL4A	59.24936	75.061553	77.254188	67.197991	62.412042	48.421721	62.238303	49.511632	55.573012	0.180936847	-0.088430806	0.144450576	-0.094796606
ENSG00000140832	MARVELD3	8.624966	8.8066	9.578056	7.373663	7.896325	8.28097	7.428934	7.413679	8.516607	0.707365486	-0.05183987	0.412949939	-0.09479129
ENSG00000197535	MYO5A	1.025921	0.919634	1.384006	1.006783	0.912521	0.822193	0.773772	0.685611	0.946166	0.984779395	0.004293906	0.568411382	-0.094776857
ENSG00000127084	FGD3	0.706401	0.837697	0.606447	0.239873	0.246534	0.203772	0.796509	0.560742	0.485248	0.00000156	-1.484494182	0.77529396	-0.094712062
ENSG00000196449	YRDC	11.883622	11.938457	13.866438	9.339978	9.223597	9.66851	10.776703	9.933862	12.461491	0.014498784	-0.269240665	0.456718026	-0.094673882
ENSG00000196437	ZNF569	1.710772	1.640665	1.987291	1.92373	1.707225	1.763114	1.687631	1.60299	1.876406	0.44912868	0.151293157	0.631870833	-0.094589949
ENSG00000160218	TRAPPC10	10.013264	10.528985	9.346061	9.715411	9.959478	9.256857	9.779742	8.808246	9.232963	0.193993351	0.107809537	0.278008993	-0.094435424
ENSG00000126231	PROZ	5.867301	5.181422	4.6045	5.048492	3.970006	4.801695	4.648957	4.814071	4.266878	0.927169578	-0.023940989	0.626194943	-0.094426816
ENSG00000198225	FKBP1C	1.56724	1.393718	1.143551	1.206941	1.346263	1.361176	1.011975	1.333502	1.266689	0.845194269	0.079192953	0.788393729	-0.094394839
ENSG00000243317	STMP1	19.507248	20.635231	20.984664	16.455239	16.7906	16.631051	18.769511	17.956735	17.358372	0.063939016	-0.14977691	0.302504679	-0.09426911
NewGene_2869	--	1.098216	1.055355	1.657825	0.967424	0.832237	0.893706	1.153528	1.076381	1.10635	0.532678788	-0.320041557	0.841878841	-0.094201227
NewGene_6218	--	3.937534	4.065958	4.552502	3.891985	3.631466	3.034882	3.692344	3.526959	4.29518	0.497325949	-0.103879649	0.468727043	-0.094146241
ENSG00000121289	CEP89	3.558714	3.415539	2.868152	2.596274	2.363733	2.355265	2.869909	2.659393	2.957878	0.026785525	-0.323460348	0.545995308	-0.094129363
ENSG00000104549	SQLE	87.113422	81.768728	82.997208	115.461329	120.103887	112.122706	75.15793	70.301521	77.795896	1.04E-31	0.61069546	0.152664685	-0.094069549
ENSG00000119703	ZC2HC1C	1.418295	0.596946	0.570038	0.540242	0.486665	0.485155	0.809693	0.587193	1.058992	0.201815148	-0.524705158	0.819453958	-0.093878652
ENSG00000182263	FIGN	6.470636	6.682853	7.995014	5.647565	5.123874	4.731034	5.9299	5.831822	6.815429	0.001062357	-0.306200668	0.364543438	-0.093869067
ENSG00000104886	PLEKHJ1	50.87153	46.202388	45.30722	36.701009	36.500507	38.994157	41.55612	41.966859	43.761643	0.00659306	-0.213044179	0.274767301	-0.093736133
ENSG00000168237	GLYCTK	30.041668	30.120463	26.299711	46.898085	51.641737	49.617963	24.84129	23.428563	28.762034	1.25E-39	0.93506013	0.343030234	-0.093664321
ENSG00000147421	HMBOX1	7.075322	6.467084	7.804639	6.044427	4.865961	5.572589	5.190241	5.433234	5.000464	0.021001363	-0.350171385	0.595036698	-0.093582306
ENSG00000179051	RCC2	51.415961	50.483351	52.118484	42.12144	40.35261	42.625848	42.860816	45.155275	47.1593	0.004768702	-0.152239866	0.168118601	-0.093518771
ENSG00000023697	DERA	36.54273	38.335586	35.277978	30.650712	30.520837	29.297924	32.43166	29.259638	33.384737	0.111521124	-0.128285121	0.281248399	-0.093301518
ENSG00000131437	KIF3A	0.73153	0.771414	1.00073	0.772001	0.889071	0.737283	0.688923	0.689404	0.836294	0.762716645	0.082811684	0.69298955	-0.093299735
ENSG00000197417	SHPK	12.277689	11.680434	10.299068	12.820046	12.375222	11.938434	9.419621	10.102684	10.601854	0.002227451	0.262460742	0.37286875	-0.093279924
ENSG00000178896	EXOSC4	24.847718	23.670694	22.0278	22.581133	23.625357	27.872934	19.408006	20.723499	22.103359	0.066645907	0.221370985	0.439583202	-0.093193426
ENSG00000164134	NAA15	14.622273	16.154067	17.866084	14.94679	13.827056	13.849208	14.219333	13.269443	15.143766	0.647036519	-0.047401364	0.308933347	-0.093162729
ENSG00000019144	PHLDB1	9.11639	9.306064	6.897344	4.32972	3.986619	3.315876	6.365534	8.021577	7.353573	2.38E-20	-0.888496857	0.369655328	-0.092878166
ENSG00000115841	RMDN2	1.881219	2.661472	2.512464	2.15989	1.927049	1.421013	1.667545	2.661775	1.595127	0.376868833	-0.247750896	0.747797971	-0.092863763
NewGene_4391	--	1.085598	1.076584	0.76912	1.699682	1.66745	1.801829	0.958191	0.787215	0.873582	0.0000254	0.96706461	0.787989652	-0.092782086
NewGene_4745	--	0.998247	0.829112	0.981362	0.550173	0.765683	0.639904	0.964195	0.712347	0.852544	0.196773525	-0.422809353	0.782533623	-0.092672521
ENSG00000214022	REPIN1	101.021162	105.899241	94.146976	104.235353	86.554128	105.180236	72.978577	79.665366	106.594517	0.015316256	0.152383692	0.312224042	-0.092291865
ENSG00000175387	SMAD2	5.176547	4.713588	5.277415	4.903063	3.555969	5.033283	4.190982	4.739318	4.198281	0.135280474	0.131353009	0.309484392	-0.091983408
ENSG00000153936	HS2ST1	21.214793	23.367223	24.430784	20.994742	19.20815	19.637428	20.562926	18.987615	21.929545	0.455476387	-0.060840529	0.253270165	-0.091858096
ENSG00000276234	TADA2A	5.731927	5.93784	5.03662	5.087687	4.751898	4.625712	6.117482	4.952353	4.65796	0.352928432	-0.126138724	0.535304549	-0.091848709
ENSG00000154222	CC2D1B	8.756583	8.024677	8.511331	8.078143	7.719184	7.194598	7.741327	7.12964	8.191077	0.614909916	0.057478722	0.362865216	-0.091768837
ENSG00000070087	PFN2	25.303539	26.089513	26.681427	21.245184	18.928271	19.115973	23.448822	21.374604	20.971752	0.001460378	-0.254252017	0.365504859	-0.091392063
ENSG00000107882	SUFU	1.507583	1.772481	1.435057	1.466395	1.346687	1.582633	1.26136	1.540775	1.546777	0.750404978	0.07043682	0.637239516	-0.091355625
ENSG00000155636	RBM45	5.397908	4.831705	5.0293	5.147121	3.997166	4.890423	4.471362	4.779164	4.358632	0.89481531	0.029415301	0.588879308	-0.091340937
ENSG00000114030	KPNA1	15.842746	16.672999	18.004398	17.580666	16.323101	15.85333	14.462766	14.362368	15.892525	0.046228348	0.144775518	0.247271863	-0.091330691
NewGene_1130	--	5.088811	4.253804	4.010752	5.045325	4.074138	4.530769	4.079519	3.563066	3.700918	0.236975835	0.181240092	0.538498362	-0.091262079
ENSG00000091317	CMTM6	28.234638	27.965088	28.1926	28.583702	27.434231	28.829273	24.857735	24.192905	25.240149	0.008276043	0.155143779	0.18208497	-0.091079253
ENSG00000179761	PIPOX	0.639429	0.530736	0.411622	0.679138	0.576309	0.693589	0.576622	0.516918	0.297719	0.262959581	0.439675203	0.851780133	-0.091078601
ENSG00000101084	RAB5IF	109.633664	108.344989	106.967529	86.691326	95.540582	91.336435	94.784596	98.717292	94.875412	0.19984644	-0.094610842	0.218089405	-0.091062853
ENSG00000187210	GCNT1	0.7907	0.773647	0.565189	1.110161	1.128144	1.143378	0.608497	0.617529	0.663836	0.0000168	0.805002562	0.731363458	-0.091026188
ENSG00000108671	PSMD11	57.717999	62.388896	57.909616	52.541481	48.230846	47.593572	47.065429	53.679556	50.289334	0.580536314	-0.03798897	0.197193492	-0.090871593
ENSG00000160818	GPATCH4	32.31734	30.003546	31.837838	29.64148	27.031231	27.344346	25.332183	28.956368	28.965682	0.87827505	-0.016577842	0.353303618	-0.090833357
ENSG00000136448	NMT1	38.955347	37.686167	37.148487	37.376665	36.864483	35.835729	33.159856	31.794783	34.701975	0.073736127	0.105165459	0.156669838	-0.09059841
ENSG00000173327	MAP3K11	50.307553	48.348198	43.191614	46.692743	49.404212	50.522663	39.631776	39.651892	45.659258	0.008483107	0.193270221	0.286212595	-0.090578448
ENSG00000119547	ONECUT2	7.215539	7.712339	7.85949	6.837863	6.419921	6.397428	6.493268	6.434741	7.15558	0.367823449	-0.066361528	0.220857915	-0.090517047
ENSG00000163608	NEPRO	13.710153	16.45799	17.462817	14.07265	13.837436	13.019622	15.583099	13.485606	15.160739	0.286287794	-0.098411513	0.325063235	-0.090286494
ENSG00000120314	WDR55	9.750516	10.134636	10.933751	9.755243	9.410445	9.569382	8.61622	8.523829	10.488247	0.587355193	0.060821782	0.430306864	-0.090261466
ENSG00000170779	CDCA4	14.766172	16.404182	15.536992	13.689906	11.84564	13.419919	14.551873	13.001072	13.685061	0.257989858	-0.113848142	0.358572972	-0.090206722
ENSG00000142794	NBPF3	2.644054	2.369424	2.565795	1.401702	1.511478	1.456585	2.366321	2.221617	1.567143	0.002752631	-0.49012968	0.616428366	-0.090157662
ENSG00000149716	LTO1	3.993262	4.318323	4.070741	3.371354	3.538755	3.216785	3.162832	3.696266	3.723912	0.441853303	-0.147539868	0.621779299	-0.090099053
ENSG00000173930	SLCO4C1	2.876692	3.168539	2.632669	5.007421	5.943879	4.940434	2.73348	2.454068	2.455045	1.58E-21	1.019927859	0.529197204	-0.090061206
ENSG00000105792	CFAP69	0.386216	0.686753	0.653683	0.434121	0.254279	0.4178	0.537236	0.425671	0.590035	0.210298734	-0.485036285	0.806315257	-0.090041722
ENSG00000100997	ABHD12	40.07068	37.333621	39.541249	34.419445	33.1223	33.672052	34.472847	29.494332	34.346284	0.791226476	-0.024649971	0.261679447	-0.089964156
ENSG00000124782	RREB1	9.607794	9.590663	9.185197	11.57327	10.896138	11.438055	9.517491	7.264748	8.694838	1.07E-12	0.405711039	0.367882498	-0.089955472
ENSG00000131844	MCCC2	26.857589	26.837944	28.349949	25.850141	23.946305	23.755226	24.7896	21.754426	25.720377	0.890046226	-0.01284302	0.254175427	-0.08994393
ENSG00000090565	RAB11FIP3	4.35366	3.425538	3.339948	2.955689	3.041482	3.322138	3.401912	2.729055	3.401021	0.520250114	-0.108841253	0.562904582	-0.089940143
ENSG00000205356	TECPR1	6.831591	6.392382	5.634974	7.21411	6.782578	7.086394	5.307476	4.752888	6.732628	0.000201741	0.311019318	0.479248872	-0.089928753
ENSG00000083099	LYRM2	10.162466	13.907687	12.007114	10.220852	9.130253	9.90208	10.242903	9.454147	10.701169	0.104015953	-0.150341587	0.364877552	-0.089548027
ENSG00000136247	ZDHHC4	60.541329	62.301045	58.360844	50.484629	50.859276	48.652182	49.864017	52.669493	54.691215	0.100179794	-0.117384931	0.267047058	-0.089528857
ENSG00000164284	GRPEL2	4.132091	4.68082	5.547722	4.863908	4.414924	4.433334	4.501685	3.863194	4.229439	0.576094161	0.087066685	0.53629619	-0.089508141
ENSG00000133316	WDR74	43.973767	40.468836	41.813392	40.314402	35.12026	36.95319	37.692038	34.681833	37.163016	0.838884388	0.021254601	0.307345926	-0.089507316
ENSG00000140391	TSPAN3	40.562294	42.008577	40.047631	36.43047	33.452486	30.069098	30.459357	34.490522	33.619485	0.332972217	-0.061256441	0.168222931	-0.089500527
ENSG00000173482	PTPRM	8.052378	6.976386	7.181736	5.906979	5.913582	5.643534	6.341588	6.221447	6.924064	0.023149594	-0.205628978	0.365832637	-0.089376032
ENSG00000171204	TMEM126B	35.407844	35.782313	35.218514	36.561094	37.038972	40.191634	29.481154	31.147422	32.783743	0.00731691	0.216037249	0.342914161	-0.089371719
ENSG00000184307	ZDHHC23	5.64479	6.047104	5.688713	3.768704	4.15362	3.960288	4.967524	4.628972	5.82988	0.0000117	-0.409943845	0.440696619	-0.089147329

Gene ID	Gene name	Ctrl-1 FPKM	Ctrl-2 FPKM	Ctrl-3 FPKM	IFNγ-1 FPKM	IFNγ-2 FPKM	IFNγ-3 FPKM	Sora-1 FPKM	Sora-2 FPKM	Sora-3 FPKM	Ctrl vs IFNγ DESeq2 FDR	Ctrl vs IFNγ DESeq2 log2FC	Ctrl vs Sora DESeq2 FDR	Ctrl vs Sora DESeq2 log2FC
ENSG00000182782	HCAR2	0.91253	0.755976	0.557588	0.818639	0.815107	0.6391	0.683364	0.506929	0.766726	0.693861754	0.17850904	0.83516328	-0.089063291
ENSG00000040487	PQLC2	10.733005	11.414188	11.011216	11.805173	10.530181	10.688756	9.025717	7.892682	10.344764	0.253641645	0.137327363	0.497171418	-0.08904908
ENSG00000126603	GLIS2	2.538943	2.47889	2.274291	3.033133	3.127016	3.60681	2.008423	2.143309	2.34448	0.0000254	0.564881823	0.598931509	-0.089000752
ENSG00000238227	TMEM250	18.243687	18.339507	16.709413	15.517025	15.871475	15.871448	14.52552	13.876232	17.93851	0.74134123	-0.034587342	0.394580285	-0.0889875
ENSG00000102699	PARP4	12.297744	11.333062	11.217834	14.946576	14.676061	14.98805	9.952792	9.780171	11.031664	1.4E-15	0.503214158	0.298601708	-0.088882137
ENSG00000130779	CLIP1	8.927987	9.845841	9.542719	11.00253	11.154055	9.714007	8.085647	7.535794	9.46118	0.00000122	0.3651602	0.368873039	-0.088826033
ENSG00000178814	OPLAH	3.541227	3.362869	3.240956	3.622596	2.953496	3.280852	2.801279	2.657107	3.51567	0.495904131	0.104268215	0.557783941	-0.08876021
ENSG00000138688	KIAA1109	3.1893	2.601371	2.367328	2.484867	3.328682	2.359622	2.310801	2.426776	2.454808	0.857080917	0.02542848	0.403966472	-0.088715545
ENSG00000163867	ZMYM6	2.421386	3.108746	2.702263	2.776677	2.754404	2.619878	2.90254	2.253886	2.179995	0.406323805	0.141347066	0.635395636	-0.088512
ENSG00000117479	SLC19A2	24.595614	27.977222	30.228719	34.067227	35.5928	33.169983	23.902875	23.557672	26.346445	1.07E-13	0.505554027	0.317614225	-0.088450927
ENSG00000205903	ZNF316	17.538343	16.287337	15.940928	12.578712	11.937539	12.976811	13.857461	13.56919	16.803565	0.000168846	-0.261821304	0.344603777	-0.088424396
ENSG00000168569	TMEM223	18.322368	17.284102	17.170833	15.99923	14.448748	17.260713	16.0764	14.766988	15.108175	0.975935104	0.005395516	0.457594785	-0.088287451
ENSG00000132356	PRKAA1	27.918388	31.713346	33.72845	31.576746	29.165278	29.652772	28.39409	25.34551	28.847089	0.221879434	0.102608266	0.325460647	-0.088266132
ENSG00000172262	ZNF131	12.230889	13.876715	14.377431	13.296659	13.258145	12.71604	13.376277	11.119408	14.314628	0.740236587	0.039585449	0.400665008	-0.088174903
ENSG00000101230	ISM1	1.226791	1.288261	1.477492	0.671704	0.80884	0.815726	1.343079	0.982676	1.181036	0.013011403	-0.640526628	0.751143048	-0.088165421
ENSG00000173611	SCAI	1.400471	1.61618	1.976796	1.503695	1.366889	1.314118	1.264582	1.691039	2.337879	0.684290694	-0.068051943	0.568137629	-0.088018181
ENSG00000176635	HORMAD2	0.617845	0.57354	0.788595	0.671017	0.609404	0.624496	0.706554	0.369753	0.690175	0.838350172	0.097991254	0.844737587	-0.087971255
ENSG00000108010	GLRX3	35.56811	41.216633	37.998048	30.721902	30.765711	32.200583	40.330236	35.242375	32.780646	0.00989875	-0.203008361	0.331960037	-0.087938303
ENSG00000096872	IFT74	3.501974	3.4225	4.252184	3.349566	3.166191	3.188004	3.585248	2.884234	3.334045	0.823527431	-0.049876934	0.643427701	-0.087720989
ENSG00000100647	SUSD6	5.139276	5.235753	4.814268	5.505822	5.086768	5.509227	4.751784	4.144958	4.51712	0.011252265	0.230981088	0.421756367	-0.087547322
ENSG00000139289	PHLDA1	16.577892	16.023853	17.416173	8.009071	7.116343	8.040824	13.963384	12.735003	18.021014	4.54E-58	-0.957630591	0.387449608	-0.087543642
ENSG00000163939	PBRM1	12.961396	13.900855	13.228523	12.421511	12.229873	12.233852	12.413404	12.126443	13.136449	0.911536142	0.011370753	0.239606621	-0.087359141
ENSG00000167840	ZNF232	6.599253	7.331277	5.877241	5.7047	5.891383	6.366311	6.104178	5.892221	6.316696	0.975331761	0.006318405	0.536789531	-0.08735261
ENSG00000085978	ATG16L1	7.164387	6.821951	7.686076	7.366674	6.729259	6.577826	6.273763	5.882127	6.869784	0.542895141	0.075964124	0.450860706	-0.087315313
ENSG00000165813	CCDC186	1.776371	2.022766	2.351695	2.788605	2.290559	2.327823	2.129466	1.675747	2.088748	0.001534128	0.403169617	0.592902862	-0.087271747
ENSG00000167699	GLOD4	44.184533	43.657874	43.576271	41.407152	38.852258	46.447643	40.289722	38.620325	37.305779	0.789312531	0.023774778	0.300996346	-0.087252119
ENSG00000168135	KCNJ4	1.426547	1.133599	1.15405	1.029646	1.004622	0.950423	1.129376	0.79537	1.369906	0.631330105	-0.167223814	0.796211572	-0.087152091
ENSG00000168291	PDHB	42.581339	37.567863	37.854592	42.753276	44.541045	43.439491	34.279394	34.975268	33.415551	0.0000663	0.29848802	0.355561058	-0.086875106
ENSG00000167740	CYB5D2	7.745765	5.674783	5.523442	5.504213	6.027651	7.147901	4.768629	4.528463	5.414168	0.220905088	0.197524294	0.594203992	-0.086855326
ENSG00000120533	ENY2	29.145799	33.18169	31.791921	28.57062	24.123058	30.114147	29.817021	33.430936	25.128747	0.366494619	-0.071301016	0.472988161	-0.086817884
ENSG00000008282	SYPL1	31.428061	32.65954	33.389022	32.555676	30.10246	31.624538	28.37116	26.336043	30.049109	0.066355278	0.128700286	0.266288911	-0.08646328
ENSG00000131263	RLIM	5.952483	7.220767	6.856521	7.130421	7.127536	6.685522	5.98733	5.814081	5.779803	0.010645333	0.219700567	0.388734272	-0.086444428
NewGene_4828	--	0.599797	0.579976	0.443835	0.532531	0.460226	0.352104	0.405717	0.440891	0.636676	--	--	0.869545989	-0.086439301
ENSG00000176974	SHMT1	31.649174	29.994417	31.581749	26.86846	27.859775	26.757857	28.22649	26.233008	29.175284	0.586859461	-0.04790862	0.266957777	-0.08637582
ENSG00000196557	CACNA1H	8.070749	7.447613	7.249102	7.234172	6.538757	7.785265	6.377001	6.010746	7.987552	0.939724429	-0.009446079	0.432208097	-0.086345908
ENSG00000088926	F11	1.488648	1.71935	1.749993	1.362069	1.431409	1.488977	1.727042	1.400027	1.533091	0.734758287	-0.106743859	0.759082337	-0.086273652
ENSG00000119125	GDA	1.242154	1.726664	1.426212	2.143563	1.951663	1.541471	0.920912	1.063112	1.406696	0.0000308	0.676716198	0.705413279	-0.086252514
ENSG00000109586	GALNT7	7.140178	7.099125	7.351157	5.815795	5.049561	5.230681	6.276616	6.269501	6.46642	0.001877311	-0.286542359	0.383265648	-0.08607985
ENSG00000158417	EIF5B	27.917648	30.25228	33.815697	32.775429	30.933071	32.114929	26.6346	26.333467	28.318432	0.004595922	0.20632608	0.315745352	-0.086074343
ENSG00000078142	PIK3C3	4.290583	4.384424	4.240004	4.005119	4.213774	3.892974	3.97738	4.048135	4.108118	0.657747431	0.047154186	0.354945859	-0.086059818
ENSG00000154845	PPP4R1	26.248305	25.486374	22.353981	22.213846	20.22904	20.938913	22.095357	21.04555	22.556403	0.332972217	-0.083863072	0.309484392	-0.086029841
ENSG00000187955	COL14A1	5.177233	4.055576	5.033703	1.348006	1.722665	1.609881	3.43816	4.355023	4.780188	4.74E-34	-1.435659564	0.583281604	-0.085999439
ENSG00000179314	WSCD1	4.126094	4.203872	3.379159	2.918369	2.930577	2.823316	3.484185	3.419205	3.34933	0.193874128	-0.180827632	0.538946058	-0.085926588
ENSG00000105127	AKAP8	15.029629	16.511247	17.628933	16.838705	15.613868	15.208812	15.353568	12.879813	15.313741	0.227511018	0.108434777	0.379726592	-0.08591118
ENSG00000197302	ZNF720	3.358373	3.392487	3.765282	3.679062	3.461489	3.420048	3.606592	3.070257	3.361917	0.70263363	0.064146075	0.559489578	-0.085783241
ENSG00000102753	KPNA3	7.883009	8.611125	9.359657	9.851516	9.463809	9.966657	7.474814	6.976038	8.405077	0.0000847	0.328252343	0.428615721	-0.085764859
NewGene_1313	--	0.598467	0.58433	0.720892	0.635703	0.848593	0.711215	0.569466	0.553861	0.561945	0.110623604	0.3515246	0.720268719	-0.085730213
ENSG00000167112	TRUB2	9.798145	9.738735	9.493277	8.133339	7.897966	8.054217	8.11532	8.792587	8.727077	0.108928672	-0.122112838	0.329772852	-0.085728915
ENSG00000105705	SUGP1	12.191519	10.734486	12.925682	12.226099	11.002565	11.740458	10.453092	10.724593	11.728336	0.252161379	0.124989156	0.455092392	-0.0857166
ENSG00000079739	PGM1	12.141518	12.610819	10.089953	7.52483	8.477921	7.665785	10.003683	10.427798	10.339567	0.000203801	-0.410636508	0.485209937	-0.085656964
ENSG00000109738	GLRB	3.167381	2.785529	3.557378	1.215809	1.863404	1.564433	2.277091	2.787813	3.227292	6.47E-08	-0.933999675	0.664363126	-0.085524711
ENSG00000136682	CBWD2	18.528308	21.90745	24.250507	20.205231	14.249404	18.473405	18.996047	20.154398	17.047805	0.315777101	-0.145621939	0.503505752	-0.085409262
ENSG00000160593	JAML	8.127805	7.457349	6.816866	5.537199	4.943252	5.086186	6.693937	7.117569	5.767091	0.002879182	-0.378360218	0.591738285	-0.085361281
ENSG00000166451	CENPN	23.24636	23.0431	21.445165	18.495751	18.484623	20.865376	21.677956	21.704553	21.681418	0.256125702	-0.12416507	0.399353905	-0.08535601
ENSG00000123091	RNF11	12.392901	14.967346	14.294847	13.38974	12.40029	12.028194	12.947471	10.856237	13.05822	0.958758356	0.007563651	0.444528795	-0.085185321
ENSG00000064999	ANKS1A	14.845907	12.706998	13.758689	10.973733	11.175911	11.591639	12.580435	10.923419	13.107507	0.082737885	-0.142933058	0.357112609	-0.085167417
ENSG00000112852	PCDHB2	5.767354	5.462122	5.029114	3.663657	3.445316	3.820478	4.168727	4.782821	5.154664	0.0000984	-0.412749384	0.51001417	-0.085138353
ENSG00000187486	KCNJ11	0.486334	0.544022	0.604313	0.468877	0.559971	0.37686	0.340121	0.54472	0.558001	0.777573947	-0.13714132	0.84104676	-0.0851237
ENSG00000105968	H2AFV	125.813097	130.681232	124.862574	107.779076	102.679173	107.899461	137.226689	127.715736	141.653611	0.036141387	-0.117322788	0.191034501	-0.085011938
ENSG00000086015	MAST2	8.748841	7.909547	8.856672	7.026285	6.477491	6.293329	7.277562	6.941593	8.423296	0.009266537	-0.216825082	0.383265648	-0.084997061
ENSG00000092098	RNF31	9.723967	10.674252	11.615366	14.233972	14.954188	15.577876	8.860033	9.312232	10.730079	1.29E-13	0.591075004	0.459468556	-0.084888424
ENSG00000184227	ACOT1	8.486196	8.144246	6.141561	8.792362	8.620176	8.839305	7.069286	6.858942	6.131356	0.010711073	0.361031916	0.63555484	-0.084851865
ENSG00000136527	TRA2B	119.617584	119.454547	125.792506	117.438806	105.017361	110.199688	112.613471	107.108683	109.914833	0.450355155	0.046518451	0.163843958	-0.084767437
ENSG00000205531	NAP1L4	84.127326	80.54627	77.394733	74.487006	70.808083	73.188529	69.336853	72.46521	70.764946	0.966950786	0.003552643	0.240469762	-0.084704567
ENSG00000136295	TTYH3	106.810885	102.655656	94.890774	68.524582	69.474082	74.647675	84.831188	83.272287	100.079589	3.26E-09	-0.356180321	0.289821407	-0.084595349
ENSG00000124532	MRS2	25.226474	28.077	28.777569	23.08975	22.307118	23.336736	26.736859	24.226183	25.812217	0.142417628	-0.115150484	0.308806478	-0.084519905
ENSG00000163605	PPP4R2	25.180252	27.998274	27.143813	25.847192	25.646145	24.726508	25.043896	22.403516	25.196596	0.224457375	0.088072045	0.280277303	-0.084430983
ENSG00000259529	AL136295.5	2.816765	2.45215	1.241916	15.42524	12.858901	11.924006	1.888155	1.827899	2.047605	8.27E-14	2.770435459	0.856582279	-0.084390408
ENSG00000116604	MEF2D	9.33178	7.855539	9.273007	9.087948	7.645296	8.029598	7.45503	6.93653	10.40164	0.89481531	0.014731818	0.331210591	-0.08434748
ENSG00000107249	GLIS3	3.527521	3.352757	3.80689	1.304996	1.288536	1.481421	3.451489	3.016349	3.813176	3.17E-27	-1.245361711	0.508180667	-0.084190511
ENSG00000153560	UBP1	18.743577	18.482448	19.273437	18.781178	17.145801	16.749449	17.201731	16.018164	17.135023	0.480925855	0.059398143	0.302709023	-0.084177357
ENSG00000078269	SYNJ2	29.753288	28.181399	33.564389	19.317834	20.468289	19.053596	28.639611	28.428258	33.727686	3.28E-11	-0.458687039	0.344976757	-0.084086477
ENSG00000173638	SLC19A1	16.18325	16.431891	14.504574	13.134489	12.885835	14.062652	13.381589	13.441206	14.854161	0.286006037	-0.097641601	0.354560354	-0.084057522
ENSG00000083896	YTHDC1	27.324613	26.60587	26.533117	30.18184	26.333709	25.762326	27.045445	23.402224	27.593694	0.009191684	0.176665748	0.255533409	-0.084051939

Gene ID	Gene name	Ctrl-1 FPKM	Ctrl-2 FPKM	Ctrl-3 FPKM	IFNγ-1 FPKM	IFNγ-2 FPKM	IFNγ-3 FPKM	Sora-1 FPKM	Sora-2 FPKM	Sora-3 FPKM	Ctrl vs IFNγ DESeq2 FDR	Ctrl vs IFNγ DESeq2 log2FC	Ctrl vs Sora DESeq2 FDR	Ctrl vs Sora DESeq2 log2FC
ENSG00000132970	WASF3	6.134923	5.586229	6.390013	4.904029	4.250253	4.278261	5.758142	4.889282	5.358938	0.005305769	-0.28324402	0.462916604	-0.08404698
ENSG00000143106	PSMA5	22.279813	24.330466	21.719115	33.535885	31.792585	32.585142	19.604411	21.12729	19.606215	5.41E-35	0.693444401	0.345954447	-0.083861105
ENSG00000115233	PSMD14	62.750085	63.957329	67.230767	56.178707	56.46706	59.077366	56.187302	59.883846	55.205494	0.752665378	-0.02676469	0.321166021	-0.08363562
ENSG00000144566	RAB5A	41.197053	40.259262	46.411834	47.703839	42.368484	43.645886	37.866022	35.840029	41.139593	0.004453789	0.210851999	0.324396647	-0.083554415
ENSG00000174483	BBS1	7.426026	7.390632	7.052326	5.060803	3.935907	4.828612	6.678535	5.964963	6.347619	0.00000434	-0.506836489	0.460240707	-0.083455002
ENSG00000139835	GRTP1	26.059054	24.048737	27.015695	24.9448	22.835654	23.838428	22.57244	21.18986	23.95588	0.678686214	0.046017726	0.406498884	-0.083311551
ENSG00000196459	TRAPPC2	1.324759	1.593788	2.539075	1.429047	1.524815	1.630735	1.348229	1.818441	1.594765	0.756088206	-0.105170607	0.779701388	-0.083304774
ENSG00000166197	NOLC1	45.262985	48.077609	51.208963	49.384903	45.300445	46.668262	42.133051	41.827145	45.69682	0.109554337	0.109904758	0.288335354	-0.083267324
ENSG00000115947	ORC4	8.953701	9.707174	9.273768	9.266905	8.489259	8.789823	8.264094	8.753199	8.167641	0.401525778	0.07195203	0.379064307	-0.083079063
NewGene_1241	--	3.390775	4.499757	4.387374	3.953556	2.90888	2.980527	3.743918	4.395464	4.897824	0.106736293	-0.235107734	0.81637566	-0.082897007
ENSG00000123810	B9D2	3.510416	3.190801	2.542792	1.350063	1.805191	2.385753	2.757832	2.901274	2.499189	0.059597433	-0.584140629	0.778439193	-0.082890784
ENSG00000132478	UNK	21.916001	21.651352	21.569413	16.332383	17.334677	17.800543	21.238074	20.335237	21.745756	0.091340168	-0.133825078	0.335725063	-0.082871565
ENSG00000136830	FAM129B	12.886775	12.698861	11.629644	8.122746	9.253632	9.673764	10.267416	10.637856	12.058444	0.000786051	-0.310013054	0.392376163	-0.082765741
ENSG00000106771	TMEM245	12.055991	13.14051	12.976829	15.306628	13.306456	14.666551	11.431825	10.307476	12.238966	0.000000845	0.326630561	0.302709023	-0.08274657
ENSG00000224877	NDUFAF8	20.6784	22.385611	20.663735	16.640724	18.122308	20.444389	19.816822	18.38418	18.215489	0.745548776	-0.058572876	0.566050773	-0.082726098
ENSG00000126005	MMP24OS	8.432058	10.67261	6.66622	8.708136	9.011595	9.168501	7.930415	5.962576	9.299862	0.573827233	0.315403753	0.906506417	-0.082713428
ENSG00000161642	ZNF385A	2.981591	2.702878	2.450399	2.152612	1.887725	2.083989	2.108926	2.428919	2.613269	0.276807638	-0.220389641	0.692302712	-0.082560523
ENSG00000173273	TNKS	5.6166	5.950124	6.286296	5.982546	5.649878	5.548146	5.649334	5.290125	5.782334	0.412269454	0.071150297	0.336171948	-0.082513736
ENSG00000184012	TMPRSS2	3.875841	3.466287	3.981239	5.124322	5.870444	4.813172	3.772035	2.721518	3.40078	0.000000949	0.61104348	0.625711634	-0.082496251
ENSG00000146700	SSC4D	0.73813	0.351926	0.443954	0.361089	0.491443	0.486538	0.504456	0.347052	0.517917	0.918609741	-0.061149861	0.859687282	-0.082493814
ENSG00000186566	GPATCH8	11.640093	10.974388	10.219506	10.78275	10.612504	10.783928	9.666672	9.193103	10.32158	0.157928236	0.122866248	0.377430909	-0.082375503
ENSG00000141655	TNFRSF11A	0.480137	0.379015	0.46984	1.318533	0.882524	0.743092	0.692548	0.285494	0.689104	0.000117242	0.780973459	0.781071175	-0.082339158
ENSG00000197226	TBC1D9B	11.652264	9.892153	9.643522	10.325641	9.94737	10.250147	8.99817	8.857189	9.929643	0.248624914	0.114715457	0.42826018	-0.082280004
ENSG00000118271	TTR	109.041386	100.261496	96.869944	84.736494	88.433579	87.893843	94.02219	92.47599	85.185005	0.16324538	-0.107629241	0.40374266	-0.08223724
ENSG00000112893	MAN2A1	6.613557	6.240959	5.763649	9.350289	9.515119	8.968387	5.714259	5.1531	5.638033	3.9E-20	0.726280386	0.45257038	-0.082223634
ENSG00000221963	APOL6	0.212565	0.20093	0.176788	47.435131	44.33482	46.874233	0.198568	0.181026	0.139691	0	8.008397265	0.821956525	-0.082158249
ENSG00000134138	MEIS2	33.238352	33.719333	36.004163	30.79715	25.327727	29.19073	32.187243	28.338589	32.442007	0.075803658	-0.129583941	0.288833793	-0.081965057
ENSG00000148175	STOM	25.578361	30.038389	32.722371	26.016171	25.018372	24.896881	26.843917	24.91876	26.242842	0.571028914	-0.065346545	0.447225517	-0.08187256
ENSG00000123104	ITPR2	7.798261	8.425304	8.332614	6.42625	5.910445	5.928972	7.328939	6.643234	7.84976	0.0000115	-0.280458331	0.306146725	-0.081848431
ENSG00000131876	SNRPA1	60.460779	59.908819	61.332942	54.967707	53.268213	52.655271	55.869887	54.266564	53.520604	0.596928799	-0.046442488	0.316892275	-0.081834022
ENSG00000082781	ITGB5	42.430546	40.277555	36.2371	25.029417	25.736982	25.976677	32.99678	33.782888	38.012245	1.1E-13	-0.496683464	0.344160193	-0.081803334
ENSG00000134352	IL6ST	10.873692	12.430981	12.963917	14.761969	14.621043	14.194809	10.900458	9.471096	11.84817	2.62E-09	0.40763824	0.396752574	-0.081778716
ENSG00000165996	HACD1	5.064976	6.658767	6.95828	2.304374	2.46066	2.869277	5.755675	4.459672	5.24715	9.94E-14	-1.234969512	0.653264498	-0.081776832
ENSG00000117868	ESYT2	8.469634	8.200936	7.897375	5.985884	6.567662	6.312741	7.632884	6.973491	7.604352	0.006991927	-0.227282964	0.354560354	-0.081681
ENSG00000163132	MSX1	1.27777	1.344154	1.414399	1.155017	0.793163	0.829997	1.165151	1.13345	1.271353	0.225830284	-0.383056502	0.788374141	-0.081632283
ENSG00000169490	TM2D2	15.735623	14.015801	18.925557	14.529076	13.450171	13.647569	16.781395	15.438738	16.76838	0.078515438	-0.162025878	0.410581421	-0.081526656
ENSG00000122591	FAM126A	8.82735	8.069487	8.058408	9.713648	9.44016	8.450975	8.161712	8.262327	8.639978	2.36E-08	0.349032952	0.283185945	-0.081468125
ENSG00000143740	SNAP47	21.788975	20.168201	19.569013	18.602366	17.701544	19.859029	18.317614	17.742938	18.15032	0.840727575	-0.022967792	0.405877657	-0.081429305
ENSG00000149547	EI24	40.063959	40.533167	35.279952	36.231336	36.15121	36.463623	31.251233	39.204048	34.300424	0.058927258	0.128431924	0.299962323	-0.081255923
ENSG00000143149	ALDH9A1	15.518807	15.061196	16.282717	14.207301	13.364687	13.337576	13.928207	14.120957	13.439172	0.635283357	-0.048999822	0.40574091	-0.081243123
ENSG00000100906	NFKBIA	34.928238	33.698589	34.987031	19.969932	21.413305	21.804235	30.095455	29.430594	32.379135	2.24E-14	-0.562359332	0.328912703	-0.081100777
ENSG00000140479	PCSK6	27.202595	25.65477	25.281111	29.660383	28.630715	29.299282	23.424873	22.055886	25.075706	4.36E-08	0.318153863	0.279058496	-0.081015423
ENSG00000135698	MPHOSPH6	20.419802	22.610934	24.619318	22.946855	19.434773	21.232489	20.552845	18.569419	21.490243	0.883130863	0.023555587	0.508109373	-0.080973075
ENSG00000086189	DIMT1	9.842357	11.729738	12.770599	12.237699	11.599663	12.369751	10.315143	8.686996	11.469912	0.036377668	0.227415862	0.550660611	-0.080897573
ENSG00000143862	ARL8A	10.545943	11.032539	12.43639	10.307111	9.456156	9.959039	10.475924	9.896698	9.78604	0.745650814	-0.047842326	0.529136735	-0.080801563
ENSG00000159082	SYNJ1	1.715232	1.660322	1.847178	1.925881	2.0201	1.747399	1.457255	1.342661	1.897303	0.041420194	0.26961676	0.628011711	-0.080468729
ENSG00000041802	LSG1	19.769011	20.730549	21.177086	17.351683	16.011848	16.741941	17.649197	16.869579	20.345213	0.032490381	-0.151622184	0.352848374	-0.080456644
ENSG00000104626	ERI1	3.846013	4.872896	3.844952	3.974317	4.804168	4.224153	4.125634	3.951225	4.126062	0.0795702	0.193688895	0.572291854	-0.080340796
ENSG00000144567	RETREG2	20.988801	19.1819	18.096271	16.588966	17.070041	16.291731	18.039627	16.76145	20.252003	0.54593327	-0.060096453	0.396740104	-0.080321055
ENSG00000196652	ZKSCAN5	6.895745	6.128557	6.29374	6.18994	5.979634	6.076779	5.771317	5.825778	6.081665	0.936604985	0.011318799	0.453115531	-0.080245282
ENSG00000134287	ARF3	39.360457	37.359642	38.568115	35.475388	36.283597	36.829661	33.282922	33.63434	35.280546	0.369038136	0.06019152	0.233046497	-0.08021819
ENSG00000185475	TMEM179B	24.918156	23.17159	23.404347	28.09768	31.112879	29.010908	20.379559	20.33634	23.559876	0.000000464	0.461024127	0.484178257	-0.080098454
ENSG00000108262	GIT1	8.696579	7.319037	7.819853	6.487188	5.867717	6.358386	6.963122	6.728683	7.826463	0.045815162	-0.207369067	0.47778429	-0.080097145
ENSG00000138018	SELENOI	8.909379	9.729856	10.62535	12.201678	12.619643	11.745059	8.776952	8.429706	8.736563	6.99E-11	0.468339134	0.38168624	-0.080046622
ENSG00000173545	ZNF622	23.648306	21.217224	21.384443	22.566114	23.604527	24.167049	19.972271	19.524565	19.243584	0.006856668	0.234117262	0.433315363	-0.07998427
ENSG00000099810	MTAP	12.7165	14.463238	15.191453	12.605249	11.907136	12.801015	13.23902	11.790979	13.810866	0.134029105	-0.136769423	0.416212287	-0.079630423
ENSG00000215126	CBWD6	6.960411	7.441128	7.939955	6.38271	6.101003	6.370954	8.009638	5.757793	6.488599	0.548176869	-0.090206465	0.623292701	-0.079540191
ENSG00000142546	NOSIP	48.947032	48.740997	43.788059	39.083622	41.228959	43.113622	38.981242	42.472959	44.119702	0.677506369	-0.046864491	0.425771129	-0.079452411
ENSG00000078304	PPP2R5C	18.763515	19.346313	20.311067	17.89166	19.830126	18.009342	15.883828	15.133082	18.917693	0.811557721	-0.024643706	0.353746121	-0.079347812
ENSG00000104812	GYS1	10.333899	9.887506	9.185837	10.196593	9.046217	10.083377	8.403635	8.487008	9.270667	0.145311543	0.141499396	0.433089587	-0.079346501
ENSG00000117155	SSX2IP	2.707512	3.123893	3.096483	2.516345	2.432388	2.308609	2.723471	2.350128	3.298595	0.241139783	-0.153728333	0.597057364	-0.079340833
ENSG00000159023	EPB41	17.55287	16.73971	16.433992	22.279495	22.032132	20.961184	15.094589	16.469183	15.309887	6.68E-19	0.51236202	0.301493232	-0.079324714
ENSG00000004866	ST7	16.74723	15.248052	15.011528	16.536664	15.56541	15.489816	14.525703	13.907448	14.044846	0.075066896	0.164138636	0.440236835	-0.079306261
ENSG00000127993	RBM48	5.890422	6.569872	6.695855	5.393933	5.236839	4.67767	5.986546	4.975105	5.686233	0.748225534	-0.05328795	0.581262421	-0.079273506
ENSG00000105185	PDCD5	39.086552	39.947766	40.662341	40.735223	35.993241	39.900849	44.212459	42.515577	34.354978	0.385406739	0.088770625	0.538002262	-0.079245781
ENSG00000188486	H2AFX	32.527805	34.769255	30.202641	29.395766	27.743518	30.118885	27.159846	27.4609	31.930644	0.886101671	-0.017178579	0.426024841	-0.079134359
ENSG00000124593	AL365205.1	25.5078	12.100848	9.392341	19.176384	8.104266	9.460509	21.140449	11.59205	8.45805	0.770729815	-0.231920544	0.912028457	-0.079113679
ENSG00000180370	PAK2	24.329785	25.901195	27.425432	24.483582	24.358607	23.092854	23.059906	21.724329	24.209506	0.666903797	0.036706984	0.291877041	-0.079102261
ENSG00000081138	CDH7	0.416883	0.285664	0.409539	0.046415	0.028757	0.033425	0.25516	0.300836	0.354601	4.22E-14	-2.532018203	0.793595091	-0.078944049
ENSG00000102098	SCML2	1.894848	2.494705	2.299035	2.448321	2.23903	2.372362	1.612047	1.868598	2.249852	0.105528791	0.249024529	0.681838279	-0.078732622
ENSG00000040275	SPDL1	10.72875	11.599094	11.293686	9.569824	8.708532	8.27836	10.73764	9.396372	9.101628	0.051473296	-0.196639533	0.506934229	-0.078697102
ENSG00000107537	PHYH	19.693521	15.440677	12.850849	13.01057	11.662835	12.910807	15.362878	13.037722	14.660088	0.175819914	-0.206504407	0.625294463	-0.078658795
ENSG00000067798	NAV3	0.531316	0.559129	0.725824	0.445236	0.309806	0.321165	0.589718	0.700998	0.568804	0.00276716	-0.731517017	0.75038549	-0.078517734
ENSG00000132300	PTCD3	16.117907	17.193835	17.608831	15.781401	15.029637	14.397254	14.647273	15.073417	15.668831	0.78291294	-0.024998373	0.304200575	-0.078436994

Gene ID	Gene name	Ctrl-1_FPKM	Ctrl-2_FPKM	Ctrl-3_FPKM	IFNγ-1_FPKM	IFNγ-2_FPKM	IFNγ-3_FPKM	Sora-1_FPKM	Sora-2_FPKM	Sora-3_FPKM	Ctrl_vs_IFNγ_DESeq2_FDR	Ctrl_vs_IFNγ_DESeq2_log2FC	Ctrl_vs_Sora_DESeq2_FDR	Ctrl_vs_Sora_DESeq2_log2FC
ENSG00000134030	CTIF	2.913025	2.684136	2.991385	2.625326	2.396478	2.767972	2.407648	2.186437	3.101984	0.962852033	-0.008496966	0.60796692	-0.078427794
ENSG00000165152	TMEM246	5.330269	5.388807	5.153569	4.194347	3.83811	3.901203	4.562899	4.12377	5.461164	0.008713443	-0.265691468	0.535427712	-0.078414
ENSG00000163214	DHX57	4.951317	4.669885	4.832091	4.084767	3.846737	4.104812	4.48282	4.084298	4.494859	0.389722051	-0.098491391	0.483767505	-0.078342555
ENSG00000102081	FMR1	10.107811	12.467	13.309971	14.759016	13.134383	13.690633	12.324538	10.609952	11.682204	0.000217762	0.354950011	0.50646827	-0.078203125
ENSG00000068354	TBC1D25	3.32725	3.37655	3.370592	2.808232	3.599182	3.224666	3.011146	2.710779	3.251246	0.790331636	0.048297122	0.588213791	-0.078180502
ENSG00000198018	ENTPD7	6.773789	5.814194	6.244533	6.644641	5.723057	6.696606	5.47504	5.118966	6.185328	0.169424095	0.16339609	0.531072275	-0.078165206
ENSG00000142599	RERE	15.485006	14.664966	16.245622	15.392715	13.725129	15.240531	13.268731	12.2038	15.584975	0.164600206	0.106470756	0.412823153	-0.078102139
ENSG00000134574	DDB2	7.646265	8.003644	7.71394	7.271885	6.301071	6.481195	6.703229	6.517005	7.603063	0.708556741	-0.060061938	0.571362018	-0.078056997
ENSG00000111653	ING4	14.685273	14.691233	11.378019	9.522597	11.791265	10.431127	10.299172	13.715224	12.823577	0.219050452	-0.176214533	0.639994651	-0.077782581
ENSG00000162419	GMEB1	3.378578	3.34522	3.600499	3.072306	2.813506	2.659133	3.2988	3.548226	4.71305	0.710513917	-0.053446309	0.518849551	-0.077759226
ENSG00000140382	HMG20A	18.369092	18.974835	18.59116	14.916124	15.079585	14.566392	16.323649	16.969411	16.72411	0.007366697	-0.181093207	0.341999206	-0.077735723
ENSG00000185917	SETD4	3.22345	4.198344	4.271392	3.309437	2.913426	3.051437	3.58551	3.861753	2.937432	0.310173129	-0.177327522	0.692441327	-0.077726875
ENSG00000146410	MTFR2	7.77168	7.52485	7.78427	6.654521	6.365906	6.641678	7.186509	6.713875	6.558301	0.385830044	-0.126127435	0.586601581	-0.077671825
ENSG00000110060	PUS3	4.529429	5.417651	7.60155	6.450821	7.274895	7.094798	5.076667	4.092946	4.900592	0.000376941	0.525781071	0.691567945	-0.077509447
ENSG00000172890	NADSYN1	8.355463	7.560559	6.493981	7.462817	6.78895	7.384245	6.131843	5.770227	7.196881	0.514033421	0.095180264	0.575699414	-0.077494122
ENSG00000135930	EIF4E2	39.462078	37.187939	38.400113	34.057537	34.752661	37.040457	39.522783	39.684831	40.529897	0.797950476	0.023080321	0.323103948	-0.077413056
ENSG00000168172	HOOK3	2.978019	3.480739	3.214308	2.992074	2.819408	2.685281	2.960076	2.465732	3.204222	0.706004316	-0.043201995	0.488754168	-0.077381403
ENSG00000137414	FAM8A1	28.876802	31.615503	33.110588	22.755194	23.823875	23.127018	28.177732	25.89682	29.176386	0.0000295	-0.277214502	0.331765345	-0.077368354
ENSG00000159200	RCAN1	11.779858	11.338369	11.130603	13.18542	13.883669	13.198493	9.934322	9.539397	10.876116	0.00000639	0.371891266	0.459468556	-0.07733923
ENSG00000086289	EPDR1	2.345526	2.031922	2.533719	1.497263	1.508369	1.354361	2.307702	1.781239	2.072848	0.008304956	-0.515237229	0.725187026	-0.077315364
ENSG00000122085	MTERF4	17.308067	10.707197	9.123432	18.019284	16.506627	8.177835	10.280777	11.292771	10.303069	0.517763567	0.072222499	0.46599517	-0.077309999
ENSG00000175768	TOMM5	102.168712	113.480078	108.989382	100.1293	99.502198	106.063061	107.266165	102.688308	99.312527	0.631091423	0.041699016	0.405877657	-0.077132543
ENSG00000170604	IRF2BP1	17.957293	16.258978	13.9879	13.435652	14.31273	15.887994	13.914474	13.024525	16.014046	0.981439244	0.004120441	0.525766734	-0.07694951
ENSG00000112146	FBXO9	10.415483	11.038223	11.56714	10.33304	10.612184	10.32499	10.347717	9.482551	11.111091	0.664820565	0.046598982	0.431847005	-0.076922774
ENSG00000179943	FIZ1	11.743369	12.306352	10.573339	10.225004	10.462404	10.143811	10.157928	8.988322	11.562166	0.94442952	0.010213216	0.50307083	-0.076842817
ENSG00000105556	MIER2	3.183847	3.145913	3.019586	2.853416	2.890035	3.156046	2.679806	2.621658	3.105859	0.608165088	0.06830755	0.527953258	-0.076815604
ENSG00000137210	TMEM14B	100.467175	104.18558	97.811675	86.134729	83.136574	92.802222	93.268306	93.36528	81.794829	0.546389819	-0.051159073	0.425207089	-0.076750385
ENSG00000159579	RSPRY1	7.826727	8.400794	8.131491	10.059157	9.939799	9.278871	7.690567	6.638714	7.16326	0.0000008	0.412204402	0.481511412	-0.076712157
NewGene_6715	--	1.706357	1.75457	3.164546	1.720135	1.711393	3.054804	0.756395	2.44804	2.694555	--	--	0.905754924	-0.076666752
ENSG00000197102	DYNC1H1	13.280086	13.346662	12.037796	20.394667	18.134149	19.276544	11.504186	10.416743	12.84072	1.56E-40	0.752341297	0.345696937	-0.076504926
ENSG00000130559	CAMSAP1	6.44226	6.42279	6.175008	5.189846	4.9161	5.196083	5.750885	4.943904	6.093803	0.04172238	-0.162742277	0.428964422	-0.076465862
ENSG00000107099	DOCK8	5.159457	4.719971	4.76746	3.517632	3.333477	3.254208	4.353492	4.321829	4.391261	0.00000391	-0.38985506	0.439575808	-0.076445249
ENSG00000100065	CARD10	16.124604	14.058922	12.634776	9.446552	10.270236	10.632731	12.242101	11.673388	14.524375	0.000299927	-0.346760322	0.502329316	-0.076324139
ENSG00000198952	SMG5	48.539379	46.879227	43.848343	40.14587	38.9412	40.277241	39.337414	38.678688	46.061024	0.269106881	-0.075262732	0.342902688	-0.076251212
ENSG00000175215	CTDSP2	37.965079	36.74577	35.009964	42.658618	41.268345	42.8635	32.2874	31.547655	33.902695	1.34E-11	0.35568349	0.26663503	-0.076174629
ENSG00000128683	GAD1	1.608897	2.010667	1.696534	2.472608	2.162997	1.915265	1.046025	1.764639	2.89816	0.04045692	0.414812819	0.74064672	-0.076132306
ENSG00000067704	IARS2	80.87394	83.068863	82.587669	71.27137	68.055481	69.436699	73.675339	68.646843	77.198425	0.079546131	-0.092680901	0.221536895	-0.075930786
ENSG00000100294	MCAT	7.438577	8.183329	7.435062	5.235186	6.184618	6.223213	6.526798	6.751308	7.604985	0.050527674	-0.26915332	0.599816615	-0.075869832
ENSG00000116661	FBXO2	1.760249	1.625279	2.007453	2.133595	3.104498	3.139772	1.491657	1.765416	1.537679	0.00093164	0.784227471	0.803588908	-0.075800908
ENSG00000178665	ZNF713	1.908667	1.870664	1.640399	1.453534	1.396813	1.31828	1.770628	1.815846	1.384565	0.303098776	-0.197906547	0.717924881	-0.075629698
ENSG00000081014	AP4E1	3.798203	4.182937	4.525073	4.053933	4.073807	3.777024	3.501193	3.22234	4.395195	0.491504028	0.084298804	0.574827118	-0.075383935
ENSG00000110092	CCND1	18.326504	21.354554	22.286519	24.685516	22.253526	23.587649	17.971202	17.167472	20.08551	0.0000125	0.335829506	0.439315966	-0.075361688
NewGene_6499	--	0.854792	0.784935	0.666749	0.947737	1.054682	0.639932	0.539003	0.68802	0.734599	0.312283163	0.364844127	0.843926214	-0.075341473
ENSG00000100292	HMOX1	15.072532	15.646643	14.090137	9.389188	9.72556	9.868189	12.231763	13.842501	13.771193	0.000000274	-0.479681015	0.517398376	-0.075274983
ENSG00000197329	PELI1	37.203739	39.143154	42.387447	36.325668	33.679794	34.431419	35.961369	33.035137	36.755234	0.662495039	-0.037970085	0.339104594	-0.075235095
ENSG00000071073	MGAT4A	16.434049	17.185125	16.814142	14.467682	14.569176	14.351095	15.476931	14.326939	15.168805	0.353142967	-0.064096184	0.281703454	-0.075234117
ENSG00000147416	ATP6V1B2	21.949322	20.790228	21.003587	20.667789	18.964708	19.353569	18.789521	17.957429	19.971363	0.784276129	0.027013177	0.361755727	-0.075202256
ENSG00000099194	SCD	394.745361	342.513977	326.983368	467.439331	478.050079	458.921906	319.835754	302.792542	325.066956	1.83E-17	0.547472769	0.361472901	-0.075175483
ENSG00000153485	TMEM251	9.922779	9.361822	7.561886	8.506342	8.89991	6.991237	8.78855	9.256146	6.011305	0.76370621	-0.063405959	0.720972465	-0.075169662
ENSG00000172159	FRMD3	0.17731	0.228374	0.176366	0.267753	0.363978	0.426824	0.1874	0.154956	0.171375	0.004133909	1.013453107	0.877927524	-0.075036073
ENSG00000105568	PPP2R1A	52.334532	47.10418	43.929308	36.886917	41.319039	41.307598	44.208278	46.127696	46.139909	0.001957818	-0.177798624	0.298496942	-0.075026122
ENSG00000114120	SLC25A36	12.450292	13.092819	13.75198	16.664813	15.672472	15.306791	11.487856	11.93918	12.186423	1.02E-14	0.528926328	0.424153466	-0.074910933
ENSG00000189403	HMGB1	187.624913	239.806934	251.25979	115.14754	162.798159	224.494058	198.551598	117.008296	112.448797	0.90618999	-0.008681822	0.269414641	-0.074767066
ENSG00000117724	CENPF	33.860743	36.454085	34.237901	25.314749	22.712027	23.590759	30.397157	31.143905	32.016767	4.29E-14	-0.400140423	0.261328306	-0.074613764
ENSG00000115806	GORASP2	50.320911	49.334751	51.679767	52.50837	51.416002	51.707591	44.2016	43.38938	47.453531	0.001223003	0.174105603	0.263885368	-0.074612833
ENSG00000073614	KDM5A	8.869487	9.104081	8.686361	9.156657	8.828346	8.876904	8.073492	8.429198	8.206513	0.005948737	0.164655828	0.306326533	-0.074609108
ENSG00000197894	ADH5	98.268628	97.489505	83.700118	96.20122	94.165095	99.942206	90.479709	85.792444	77.255859	0.008202547	0.184541654	0.438521751	-0.07459566
ENSG00000023902	PLEKHO1	22.471641	20.829238	20.776821	16.796778	15.938358	16.542493	17.710542	18.587577	20.770851	0.004122561	-0.231687319	0.451882609	-0.074585717
ENSG00000117602	RCAN3	1.177443	1.196098	1.429943	1.540417	1.66602	1.749458	1.674804	0.945538	1.22364	0.000204116	0.527265218	0.723690268	-0.074302198
ENSG00000169991	IFFO2	1.304864	1.430617	1.418185	1.577498	1.341906	1.524698	1.568221	1.116027	1.02695	0.127603176	0.239399691	0.729339559	-0.074279041
ENSG00000256537	SMIM10L1	7.177825	6.905859	6.136506	5.639335	5.793096	5.962397	6.152738	5.896341	5.951522	0.562481362	-0.069486653	0.500194206	-0.074278567
ENSG00000113597	TRAPPC13	6.064529	6.587792	6.405779	5.826701	6.132839	6.082116	5.796315	5.406898	5.662603	0.538311546	0.084562852	0.568412537	-0.074273609
ENSG00000143669	LYST	0.602528	0.489054	0.437395	0.306644	0.389191	0.303369	0.366475	0.35657	0.511292	0.10040026	-0.333592166	0.735574969	-0.07423384
ENSG00000154277	UCHL1	22.192196	19.844243	20.897839	13.059632	12.235998	14.993127	17.115562	18.600292	19.297429	2.58E-08	-0.554093884	0.51720704	-0.073807498
ENSG00000145246	ATP10D	10.470439	12.256677	13.782238	10.450188	10.417686	9.607349	10.680871	10.163965	11.930519	0.282020692	-0.115647748	0.505981139	-0.073801808
ENSG00000130810	PPAN	19.135263	19.004931	18.985842	17.515638	18.749084	18.65268	14.993526	15.313506	20.935793	0.430906937	0.082199504	0.577594835	-0.073790357
ENSG00000276644	DACH1	0.435828	0.451084	0.659995	0.492996	0.521664	0.469968	0.43471	0.432583	0.476012	0.722659526	0.131378854	0.827497229	-0.073787552
ENSG00000165801	ARHGEF40	7.772217	6.791696	5.366039	6.048212	6.12088	6.32865	6.710974	5.238363	6.286745	0.810146693	0.03961133	0.608856215	-0.07375654
ENSG00000109794	FAM149A	1.291291	0.841009	1.301146	1.354437	1.246568	1.557201	0.801275	1.226816	1.071709	0.248624914	0.328005571	0.82599171	-0.073656308
ENSG00000104312	RIPK2	10.323235	10.791323	11.707459	24.974817	22.911982	24.15068	10.683782	8.10561	10.120391	4.54E-70	1.311325633	0.56505107	-0.073533839
ENSG00000143549	TPM3	223.569259	227.558746	232.051371	226.371112	227.134849	233.40047	194.77112	187.78825	189.049305	0.006742771	0.126432716	0.201826492	-0.073507586
ENSG00000171462	DLK2	0.873497	0.622626	0.531137	0.534615	0.740796	0.508596	0.509031	0.629844	0.639781	0.855402589	-0.101394861	0.871566441	-0.073411746
ENSG00000135185	TMEM243	3.046645	2.90093	3.274849	2.929726	2.327411	2.104884	3.163232	2.342091	3.01375	0.563687604	-0.16898542	0.778688612	-0.072936433
ENSG00000188107	EYS	0.623049	0.750911	0.553282	0.401915	0.541955	0.438142	0.506692	0.614	0.486498	0.093011858	-0.403697304	0.770287521	-0.072813972

Gene ID	Gene name	Ctrl-1 FPKM	Ctrl-2 FPKM	Ctrl-3 FPKM	IFNγ-1 FPKM	IFNγ-2 FPKM	IFNγ-3 FPKM	Sora-1 FPKM	Sora-2 FPKM	Sora-3 FPKM	Ctrl vs IFNγ DESeq2 FDR	Ctrl vs IFNγ DESeq2 log2FC	Ctrl vs Sora DESeq2 FDR	Ctrl vs Sora DESeq2 log2FC
ENSG00000153291	SLC25A27	1.849783	2.335958	2.286672	2.110292	1.929421	1.5927	2.121666	1.965636	2.28074	0.495471318	-0.188337692	0.772285106	-0.072812215
ENSG00000162738	VANGL2	5.426583	5.167574	4.56063	3.854554	3.659653	3.593627	4.550998	3.970966	5.029537	0.003223444	-0.302894029	0.559421822	-0.072800719
ENSG00000141447	OSBPL1A	12.357728	13.329034	14.557907	12.823694	13.462423	12.66962	12.436707	11.898183	11.8473	0.308903325	0.101372416	0.475467516	-0.072688002
ENSG00000138785	INTS12	11.973429	11.62505	11.846363	14.307518	12.705745	13.057292	11.89923	10.173549	10.400193	0.001689032	0.31269486	0.561340359	-0.072642563
ENSG00000167193	CRK	31.844278	30.167473	32.530876	31.570022	31.181881	32.130279	27.847853	26.792587	30.391912	0.021662205	0.142122134	0.32408302	-0.072495875
ENSG00000169499	PLEKHA2	2.352431	3.209488	2.534292	2.771195	4.387024	3.693873	3.473638	2.253269	2.509945	0.02774793	0.301276455	0.642014943	-0.072434548
ENSG00000101850	GPR143	14.33461	16.06035	17.37288	14.573578	12.8153	15.280624	13.55693	14.938869	14.05431	0.881705247	-0.023348378	0.554315078	-0.072377572
ENSG00000180957	PITPNB	34.482867	34.826139	39.518329	40.916407	39.0067	41.644393	31.953089	30.295229	33.483887	0.0000172	0.305979226	0.411128621	-0.072271216
ENSG00000126581	BECN1	27.609274	29.656576	30.001831	28.43192	26.680306	26.657315	26.728524	23.923088	27.442746	0.461920769	0.06792822	0.429963559	-0.072186402
ENSG00000140525	FANCI	25.724051	29.590425	28.966399	21.307364	19.06271	19.647357	23.64719	23.674435	25.389434	0.00000193	-0.31965214	0.374577935	-0.072106959
ENSG00000152795	HNRNPDL	110.358331	109.321221	122.140301	105.061465	109.691699	120.230932	112.830094	101.738661	103.845221	0.33932131	0.064152852	0.320343026	-0.072066749
ENSG00000078900	TP73	0.485784	0.362558	0.289608	0.235962	0.295285	0.304896	0.344538	0.307105	0.240406	0.863464312	-0.080993168	0.855312131	-0.072038459
ENSG00000184110	EIF3C	77.209021	80.857475	79.139669	70.399669	75.121714	68.514086	63.131101	72.535093	75.381506	0.923896921	-0.008748124	0.37797019	-0.071985853
ENSG00000112118	MCM3	102.898952	101.806525	106.014434	88.653531	88.813917	87.43304	90.887398	90.833749	98.932575	0.150502155	-0.080051647	0.250817686	-0.071890179
NewGene_52	--	1.473144	0.738805	0.870397	0.922509	1.109861	1.398489	0.98625	0.826901	0.926241	0.53968218	0.305274555	0.883759646	-0.071855749
ENSG00000048649	RSF1	4.121563	4.20292	4.345906	4.478195	4.301413	4.335413	3.834462	3.512123	4.228801	0.002407457	0.217350986	0.43933896	-0.07181485
ENSG00000069275	NUCKS1	143.041885	152.885849	153.475891	137.153763	134.984039	138.034927	135.537933	127.602592	137.997726	0.825896028	0.01572823	0.248122557	-0.071714392
ENSG00000147100	SLC16A2	0.571713	0.657694	0.554892	0.494052	0.408649	0.574646	0.567135	0.455083	0.573326	0.749459171	-0.119756366	0.822096284	-0.071713656
ENSG00000163932	PRKCD	6.046723	6.39462	5.127168	6.603927	5.348633	5.849876	5.525543	5.125508	5.024275	0.303344509	0.150259515	0.626594138	-0.071674435
ENSG00000150627	WDR17	0.355525	0.367051	0.426392	0.28655	0.267225	0.233571	0.32929	0.346017	0.331657	0.311909577	-0.37651692	0.844335953	-0.071648434
ENSG00000147003	CLTRN	1.010452	1.476851	1.292805	0.448228	0.816504	0.406265	0.988786	1.093323	1.307894	0.010327047	-0.996560059	0.845756645	-0.071388588
ENSG00000103342	GSPT1	53.493892	52.728881	58.156787	51.265039	57.26628	50.634813	49.333895	49.270894	53.933106	0.487717917	0.054997215	0.348210361	-0.071259405
ENSG00000086544	ITPKC	4.770957	4.910578	4.52596	6.556174	6.852703	6.495678	4.178961	3.959571	4.546706	2.52E-11	0.63297439	0.586393963	-0.071123503
ENSG00000067955	CBFB	16.043995	19.059575	20.088981	15.101748	13.079488	13.594047	16.992202	15.615127	16.872921	0.005851421	-0.264149829	0.511710318	-0.071056273
ENSG00000125841	NRSN2	15.26019	12.862084	13.184987	16.514138	16.841619	16.941247	11.654359	11.997261	13.32653	0.00000191	0.431349968	0.550283378	-0.070991138
ENSG00000120071	KANSL1	15.323917	15.461998	15.853368	14.967662	13.740482	14.405823	13.55682	12.987827	14.091471	0.415386536	0.061827087	0.360141921	-0.070970145
ENSG00000100372	SLC25A17	13.843259	12.537495	12.731509	8.932006	8.914147	8.362803	12.043174	11.454885	10.978275	0.0000189	-0.406958691	0.533908751	-0.07075614
ENSG00000169398	PTK2	39.477247	38.128903	39.065289	35.067049	34.815111	33.591316	34.247222	35.134705	36.522942	0.85863081	-0.014298217	0.280855584	-0.070706202
ENSG00000166128	RAB8B	15.334194	16.535313	16.958783	11.659178	12.152837	12.723192	14.356579	13.36326	15.137405	0.004269137	-0.236155133	0.454007051	-0.070687219
ENSG00000070366	SMG6	4.024513	3.913371	3.20097	3.545892	3.045194	3.510915	3.432064	3.099651	3.572451	0.973662659	-0.006418407	0.598931509	-0.070680783
ENSG00000143819	EPHX1	18.008061	17.727206	15.473796	17.731483	17.444332	16.761134	15.459387	15.464635	15.39753	0.023159913	0.214341367	0.534221682	-0.070574549
ENSG00000099942	CRKL	37.769135	38.06301	36.910583	39.234085	37.869712	38.513107	33.352203	29.823492	37.741013	0.000249752	0.183784387	0.386790427	-0.070260509
ENSG00000215252	GOLGA8B	9.488959	11.064477	12.538119	14.043386	10.953577	10.787735	10.389471	8.337184	10.870027	0.032086646	0.259113509	0.596932869	-0.070199827
ENSG00000107372	ZFAND5	70.782486	70.772	72.912595	57.612052	56.449886	59.885095	64.3373	60.745692	65.201374	0.016745468	-0.137833561	0.300915782	-0.070110866
ENSG00000101412	E2F1	11.151428	10.738351	10.848269	9.814178	8.335003	8.642418	9.328902	9.746191	10.16108	0.181800989	-0.143418334	0.51001417	-0.070098296
ENSG00000159596	TMEM69	11.844579	12.427329	12.716569	10.557742	10.095256	9.643604	10.820464	11.081649	11.120707	0.153636745	-0.141371312	0.501374845	-0.070067349
ENSG00000138346	DNA2	7.775967	8.482394	8.5972	6.732583	6.377864	6.046918	7.565733	7.240998	7.374094	0.019739286	-0.212584311	0.497396957	-0.070025789
ENSG00000184900	SUMO3	85.562445	85.988977	88.29826	79.937161	79.341469	85.019275	79.11882	75.812331	76.777478	0.38922693	0.060234232	0.318726099	-0.069986022
ENSG00000170035	UBE2E3	112.982336	118.942471	118.865473	71.973534	80.569373	78.59387	106.079481	104.37337	107.939653	1.32E-16	-0.440662112	0.293482293	-0.069936598
ENSG00000086475	SEPHS1	37.482184	38.278029	38.748877	29.446246	28.837848	31.008434	33.521771	32.396195	35.54239	0.000100456	-0.225373394	0.309613559	-0.069883033
ENSG00000198814	GK	20.787339	24.849142	24.741845	28.0261	28.528359	26.187203	19.622153	20.552778	20.576937	1.35E-08	0.417932827	0.447591738	-0.069802975
ENSG00000145022	TCTA	6.154931	6.654406	7.016957	6.249441	6.071782	6.330843	5.793272	5.827063	6.073658	0.706330466	0.059413776	0.619011244	-0.069652759
ENSG00000115306	SPTBN1	132.73012	130.331426	130.09326	158.898725	153.806733	154.746314	115.280745	108.95547	125.107793	6.27E-22	0.398540871	0.267620598	-0.069634939
ENSG00000198648	STK39	8.379784	7.690264	9.758393	5.583439	4.977819	5.735427	7.200419	6.845807	9.096581	0.000000877	-0.516109669	0.610989471	-0.069611613
ENSG00000164163	ABCE1	36.867441	41.788237	44.748005	39.192618	36.981156	37.462236	36.161971	34.801118	39.363416	0.912733276	0.012313056	0.441900971	-0.069565006
ENSG00000083838	ZNF446	2.840008	2.457916	2.655101	1.849868	2.655261	2.967501	2.614139	2.090656	2.562489	0.528445103	0.136139549	0.716794757	-0.069562746
ENSG00000153012	LGI2	0.378159	0.495076	0.42901	0.217576	0.237114	0.298161	0.333204	0.371425	0.459296	0.03359689	-0.632635412	0.823305481	-0.069542676
ENSG00000179151	EDC3	21.813249	19.485789	19.489869	19.093045	16.662215	17.815016	17.731864	17.846154	19.618838	0.747615219	-0.034193322	0.443514404	-0.069524295
ENSG00000088682	COQ9	18.211277	17.025897	15.111359	12.215895	11.653668	11.100166	14.874719	14.531685	15.118658	0.00028575	-0.364381255	0.545565966	-0.06947386
ENSG00000100918	REC8	1.039468	0.711062	0.756901	8.016547	6.003793	7.059056	0.659767	0.7341	0.528215	3.54E-89	3.428719646	0.868107493	-0.069395104
NewGene_3701	--	16.365987	15.406695	14.483232	32.760681	27.661582	28.107555	11.858579	11.673632	12.85667	3.08E-102	1.198570865	0.39771694	-0.069342145
NewGene_1483	--	1.269071	1.329237	1.147221	0.937484	0.918786	1.366633	1.261208	1.035789	1.367474	0.660555047	-0.185131584	0.855312131	-0.069183505
ENSG00000159461	AMFR	9.995187	10.169685	10.316909	10.789147	10.807926	10.109687	8.3138	9.576255	9.401195	0.017205133	0.192849107	0.513514066	-0.069169864
ENSG00000213020	ZNF611	1.361543	1.375719	1.312804	1.311219	1.018439	0.978072	2.300073	1.136623	1.186389	0.436498572	-0.163941235	0.735211888	-0.069154244
ENSG00000119487	MAPKAP1	39.263586	35.908241	34.858651	28.013388	28.418053	26.270817	33.642531	32.041521	33.347014	0.00000946	-0.271490694	0.350956712	-0.069092051
ENSG00000176273	SLC35G1	5.310396	5.042972	5.350389	3.434564	3.345867	3.571912	4.818388	4.54548	4.863086	0.0000287	-0.468489301	0.591091531	-0.068950781
ENSG00000203724	C1orf53	6.021133	6.193738	5.347029	6.399124	7.291197	6.923621	5.070055	4.685986	6.033229	0.099645686	0.376182144	0.800339102	-0.068915233
ENSG00000198718	TOGARAM1	3.134857	2.983703	3.041789	3.130331	3.002535	2.602514	2.93607	2.202957	3.093109	0.610822294	0.072667612	0.635010775	-0.068721969
NewGene_2749	--	0.722817	0.734391	0.703989	0.46187	0.436542	0.589451	0.671082	0.73968	0.448397	0.345113826	-0.445310249	0.884988884	-0.068645107
ENSG00000141084	RANBP10	5.403567	5.236822	5.813397	4.603085	4.56938	4.396203	5.095011	4.875035	5.463963	0.211701054	-0.134651493	0.544605239	-0.068588539
ENSG00000006530	AGK	10.549762	12.479867	11.887034	9.551517	12.229979	8.629635	11.354724	10.520983	11.330231	0.06866153	-0.15710129	0.46599517	-0.068478315
ENSG00000118420	UBE3D	3.804433	3.671243	3.711542	3.054108	2.423908	2.774425	3.410982	2.896056	3.515888	0.096897882	-0.308183278	0.717972084	-0.068427127
ENSG00000113649	TCERG1	25.851412	27.805882	29.755393	28.399045	25.355608	26.028056	25.138254	22.975727	25.527963	0.287427728	0.090421836	0.419645088	-0.06840133
ENSG00000155755	TMEM237	8.354577	8.432427	17.419216	5.345028	5.393695	10.603964	14.351007	7.279254	8.711443	0.000473347	-0.305690974	0.509520882	-0.068223152
ENSG00000163686	ABHD6	2.876407	2.690359	2.40598	2.255129	2.973003	2.441222	2.6257	2.069041	2.515241	0.703083616	0.092110635	0.743243948	-0.067985929
ENSG00000128641	MYO1B	28.127428	28.911336	30.535044	24.091861	21.76143	23.417405	26.375985	25.455741	26.443945	0.002115278	-0.18970438	0.348059959	-0.067983169
ENSG00000111052	LIN7A	14.339703	16.284054	16.887104	16.244056	15.614298	15.355351	15.002935	15.277789	15.463022	0.135155306	0.117863468	0.437154116	-0.067966072
ENSG00000070367	EXOC5	6.478686	6.443634	6.856488	7.142497	6.401377	6.238111	6.038887	5.723456	6.183999	0.215528456	0.105261959	0.458938006	-0.067916332
ENSG00000106603	COA1	28.384316	28.871469	30.311007	23.56683	25.280142	23.662471	25.104456	26.604105	25.106337	0.093613573	-0.136136578	0.456718026	-0.067844188
ENSG00000168234	TTC39C	10.799031	11.577725	12.644933	12.956851	13.226642	13.278521	12.333517	10.963478	11.792509	0.000164414	0.320569244	0.542422249	-0.067804857
ENSG00000158161	EYA3	4.243831	4.299387	5.01137	4.08439	3.76248	3.654007	4.10506	3.968297	4.002809	0.461031705	-0.093402403	0.573467	-0.067674895
ENSG00000014138	POLA2	14.709835	15.408353	14.586635	14.012537	12.667869	13.250366	13.139534	11.683087	13.990916	0.936907762	-0.009070729	0.460370052	-0.067568074
ENSG00000126903	SLC10A3	4.221871	4.792989	4.506188	4.980894	5.392955	5.31899	3.609032	4.24771	4.557021	0.021317506	0.322184385	0.696401556	-0.067552076
ENSG00000171681	ATF7IP	8.817979	10.007271	9.43633	8.825582	7.996767	7.647531	9.485023	7.914175	8.844535	0.04895811	-0.150288889	0.451882609	-0.067547282

Gene ID	Gene name	Ctrl-1 FPKM	Ctrl-2 FPKM	Ctrl-3 FPKM	IFNγ-1 FPKM	IFNγ-2 FPKM	IFNγ-3 FPKM	Sora-1 FPKM	Sora-2 FPKM	Sora-3 FPKM	Ctrl vs IFNγ DESeq2 FDR	Ctrl vs IFNγ DESeq2 log2FC	Ctrl vs Sora DESeq2 FDR	Ctrl vs Sora DESeq2 log2FC
ENSG00000180044	C3orf80	0.423737	0.423029	0.57458	0.32193	0.447868	0.286903	0.54932	0.395526	0.330583	0.556001666	-0.292961986	0.884487544	-0.067389513
ENSG00000145730	PAM	18.691378	18.135347	18.559678	11.863661	9.082175	10.75503	15.801686	13.649005	16.713632	2.12E-17	-0.575498771	0.410867421	-0.067344025
ENSG00000127663	KDM4B	9.937251	8.669724	7.722731	7.018486	6.944449	7.564157	7.967509	6.571189	8.4925	0.306854027	-0.119662743	0.600924891	-0.067136554
ENSG00000178460	MCMDC2	0.254872	0.302812	0.390734	0.42579	0.193417	0.195553	0.340472	0.211553	0.184091	0.96728994	-0.025102258	0.884487544	-0.067126961
ENSG00000055130	CUL1	31.204428	30.008706	32.063823	29.504291	28.099474	29.257712	28.115731	26.527422	28.876096	0.579457681	0.044364585	0.373734638	-0.067023162
ENSG00000197603	CPLANE1	1.213344	1.268856	1.047551	1.199978	0.777323	0.836152	1.032524	0.899624	0.993922	0.470503013	-0.159009164	0.696038488	-0.066878145
ENSG00000171150	SOCS5	7.4194	7.159266	8.01856	6.019386	6.56002	6.463269	6.793735	6.312429	7.102947	0.307641642	-0.112439266	0.535491651	-0.066843145
ENSG00000133026	MYH10	47.460888	48.183657	45.818863	38.203613	36.946949	37.783437	41.590177	40.497258	45.354629	0.000222385	-0.180608734	0.293587024	-0.06683104
ENSG00000157954	WIPI2	58.407354	55.005815	58.212206	51.977524	51.387794	49.795857	49.825245	49.552617	55.423676	0.303344509	-0.069347183	0.388396494	-0.066810492
ENSG00000166171	DPCD	6.050222	4.440488	3.748931	3.527786	2.755763	2.968108	3.881927	3.770187	4.704376	0.105154459	-0.438014862	0.813763326	-0.066787498
ENSG00000214435	AS3MT	12.970729	14.399319	12.000005	9.077042	9.292432	8.800748	12.966369	11.14001	11.131683	0.0000562	-0.38755293	0.591091531	-0.066729226
ENSG00000155252	PI4K2A	8.824396	7.945109	7.887841	9.579488	9.372632	9.322952	7.167903	7.041107	7.900622	0.0000194	0.343866856	0.522495695	-0.066719651
ENSG00000172239	PAIP1	62.428772	60.164956	63.088366	52.970842	51.561916	49.502241	56.10309	54.503247	59.224516	0.037075245	-0.124667119	0.321802152	-0.066696264
ENSG00000093144	ECHDC1	52.068285	53.919563	56.513994	51.62719	52.19127	55.2109	52.14006	43.355896	48.547991	0.052480202	0.139697405	0.446093919	-0.066655428
ENSG00000165288	BRWD3	1.432417	1.600092	1.61973	1.606262	1.717749	1.417	1.433953	1.171967	1.571326	0.183569108	0.173835867	0.644085667	-0.066606212
ENSG00000163975	MELTF	4.883213	4.465761	4.082682	2.968902	2.589224	3.048497	3.754545	3.874838	4.305069	0.000442224	-0.450193374	0.639451608	-0.06656114
ENSG00000157191	NECAP2	16.963244	16.476895	15.760075	10.921586	12.861969	12.791508	13.908815	14.527289	15.39453	0.006349772	-0.270231716	0.513091833	-0.066508245
ENSG00000121390	PSPC1	54.502644	53.823265	53.881804	47.046028	49.078197	45.512128	46.978502	47.239719	50.344021	0.557019239	-0.044389718	0.342691259	-0.066474543
ENSG00000111669	TPI1	364.712848	384.151832	361.160194	316.927432	330.953857	328.930067	282.925034	356.798302	310.368133	0.102028931	-0.125097655	0.447166424	-0.066432628
ENSG00000151465	CDC123	50.939907	47.819829	53.609656	46.131651	45.491241	45.493496	44.773496	44.473207	47.27996	0.999202724	0.000154961	0.426180834	-0.06641893
ENSG00000164961	WASHC5	21.834444	21.539747	20.852383	20.364393	18.890435	19.050548	18.831861	18.841283	19.659273	0.99264631	0.001017862	0.373195459	-0.066255527
ENSG00000060339	CCAR1	29.352964	32.309848	32.144695	33.860372	30.955308	30.936316	29.251155	27.982053	28.877529	0.074817112	0.137552344	0.426180834	-0.066197613
ENSG00000100321	SYNGR1	1.949941	1.538823	2.095275	2.076682	1.480112	1.686021	1.242055	1.764756	1.761277	0.936822988	-0.031376215	0.835744198	-0.06618216
ENSG00000157870	PRXL2B	21.313393	19.896275	20.535628	20.689191	18.30463	20.601611	18.212955	17.612806	20.008844	0.244782027	0.099771706	0.450562859	-0.066080025
ENSG00000088766	CRLS1	16.853662	18.427138	17.540556	18.628671	18.445836	18.941934	15.543518	15.590248	16.254185	0.001077177	0.215861349	0.422135137	-0.065948773
ENSG00000166575	TMEM135	12.672641	13.377657	12.747874	13.443505	14.228953	12.865313	12.102857	10.635214	11.73255	0.002042366	0.231566547	0.489197376	-0.065896485
ENSG00000124193	SRSF6	90.662243	82.93514	82.208839	73.608822	67.694313	73.415949	86.164612	77.879978	95.004822	0.324557524	-0.05811102	0.305606347	-0.065863553
ENSG00000154864	PIEZO2	5.795161	5.555659	5.420947	4.263797	4.347145	4.074162	5.058987	4.779081	5.165182	0.003147232	-0.244216286	0.476686075	-0.065803134
ENSG00000164251	F2RL1	3.21423	3.792022	2.82491	2.176152	2.281622	1.678842	3.344343	2.360744	2.378036	0.004306565	-0.52588647	0.73948794	-0.065769392
ENSG00000204516	MICB	3.861599	3.301757	4.194058	4.008654	3.535276	3.998231	3.819865	2.871678	3.504442	0.334659868	0.167119497	0.732579591	-0.06573062
ENSG00000008283	CYB561	2.502055	1.908782	1.876326	3.183724	3.084246	3.120917	1.934598	1.728145	1.955213	0.00000474	0.717171513	0.765240502	-0.065724331
ENSG00000265354	TIMM23	46.898594	46.880459	49.042953	44.030121	41.994534	44.271927	41.730347	42.741169	43.460564	0.884679815	0.015048053	0.439900556	-0.065639296
ENSG00000239857	GET4	98.585929	94.6816	91.346159	86.058296	91.9862	91.402684	79.846136	82.331878	94.988333	0.374836942	0.063911242	0.39064188	-0.065537881
ENSG00000180340	FZD2	5.149887	5.115868	4.63908	3.311836	3.373704	3.396384	4.862199	3.942045	4.581301	0.003524364	-0.416190413	0.693110164	-0.065526205
ENSG00000177191	B3GNT8	1.855086	1.271347	1.115065	0.704801	0.836032	0.923345	1.47385	1.07825	1.268908	0.054504689	-0.629099131	0.849692347	-0.065448433
ENSG00000189308	LIN54	5.397906	5.987538	5.900326	7.259761	7.628458	6.888298	5.133023	4.782017	5.344231	0.000000907	0.411154742	0.548899512	-0.065363131
ENSG00000186787	SPIN2B	1.63286	2.314258	1.810638	1.936694	1.85377	1.626947	1.813278	1.661095	1.570125	0.858381851	0.076518681	0.858817044	-0.065344934
ENSG00000101843	PSMD10	17.561739	18.034791	17.615919	16.625047	18.068993	17.835352	16.096804	15.523675	16.31268	0.207893925	0.130659782	0.535427712	-0.065337477
ENSG00000112210	RAB23	1.145587	1.600609	1.729242	1.003876	1.204563	1.088839	1.569272	1.252168	1.427919	0.181553792	-0.2915885	0.768798651	-0.065168772
ENSG00000144747	TMF1	2.894634	2.905576	3.247421	3.451808	3.866454	3.409402	2.805616	2.714544	2.827246	0.001288259	0.324938852	0.597525945	-0.065139852
ENSG00000140092	FBLN5	2.78387	3.385117	3.176125	1.663419	1.770596	1.690319	2.73105	2.774987	2.928526	0.0000721	-0.690533754	0.738554112	-0.065012579
ENSG00000105447	GRWD1	11.7666	11.177909	11.06109	8.818371	8.82525	8.654186	9.364694	9.459796	11.616889	0.000843267	-0.233461425	0.507967528	-0.064917022
NewGene_1938	--	0.403598	0.61449	0.538325	0.508098	0.279538	0.420847	0.511942	0.426216	0.450839	0.706330466	-0.152005119	0.843693632	-0.064604781
ENSG00000116560	SFPQ	89.023917	92.223332	93.742015	86.572241	79.666881	83.203547	83.694649	78.482482	84.434847	0.983734825	0.00171802	0.292684099	-0.06450983
ENSG00000161939	NASEK-C17orf4	3.394807	1.61737	2.983636	2.408464	2.205099	4.358137	1.798412	3.358838	1.986014	0.461031705	0.310295993	0.880432744	-0.064346259
ENSG00000118579	MED28	20.323807	20.284487	20.270958	17.782911	17.721256	17.304416	18.11162	18.195954	18.566763	0.350096931	-0.056767874	0.320061797	-0.064314637
ENSG00000136750	NUP54	22.860755	24.703214	24.662259	22.756872	24.830437	24.291516	19.95368	20.639356	22.708451	0.202791608	0.104135647	0.469870207	-0.064247791
ENSG00000184047	DIABLO	26.164042	26.442286	24.037179	23.641546	21.481408	22.344056	22.833229	24.772417	21.271694	0.761768675	-0.035668393	0.591738285	-0.064106587
ENSG00000079134	THOC1	18.00044	17.801843	19.022384	15.06532	12.918886	15.428214	17.658069	16.872366	16.785485	0.369240621	-0.100494712	0.543574183	-0.064085422
ENSG00000104472	CHRAC1	14.883248	17.912834	18.131169	17.974785	16.634062	18.031104	15.094238	13.979206	15.964154	0.026040941	0.213773288	0.571362018	-0.063975129
ENSG00000131373	HACL1	21.797476	23.820137	20.565564	21.261149	23.217264	24.052275	21.194856	21.153969	18.983323	0.009878499	0.205013758	0.513251865	-0.063962248
ENSG00000065809	FAM107B	39.921722	38.58639	40.196837	36.671042	37.28988	38.098739	34.659281	31.589602	36.36317	0.470154299	0.054645242	0.386945793	-0.063929237
ENSG00000126561	STAT5A	4.103361	3.245217	3.824139	6.261166	6.389193	7.172911	3.801493	3.176852	3.173127	4.98E-19	0.944944536	0.688121407	-0.063872313
ENSG00000102038	SMARCA1	97.120048	93.425482	89.794411	94.718042	95.621587	91.008631	84.740134	77.823491	88.100124	0.008074791	0.148962418	0.349477259	-0.06380679
ENSG00000117533	VAMP4	1.762637	2.260296	2.20562	2.026686	1.942861	2.096405	1.995221	1.947881	1.801154	0.718996037	0.069264395	0.716794757	-0.063790448
ENSG00000111850	SMIM8	2.054322	2.677789	2.426006	2.762708	2.436757	2.292313	1.959678	1.954086	2.336522	0.320118866	0.211139387	0.778726785	-0.063772273
ENSG00000198712	MT-CO2	13807.40625	14109.54395	15311.83203	14154.20215	14240.37695	14260.55078	12478.83398	13581.26563	12672.91504	0.022716029	0.128276209	0.430557798	-0.063751952
ENSG00000138696	BMPR1B	1.599611	1.477275	1.60322	1.111088	0.902643	1.082775	1.50489	1.120492	1.165692	0.197976163	-0.268275351	0.763410877	-0.063706652
ENSG00000142396	ERVK3-1	38.172084	38.192336	35.790166	40.396336	40.916238	44.206882	29.417914	32.428715	30.747599	5.73E-10	0.425440905	0.527177566	-0.063667828
ENSG00000012822	CALCOCO1	3.956717	3.230375	3.800227	3.663175	3.402281	3.624492	3.20313	3.338254	3.312211	0.355424407	0.136222221	0.67926421	-0.063554811
ENSG00000061987	MON2	4.296243	4.867555	5.053686	5.723422	7.157611	6.494155	4.820572	3.985298	6.154324	0.003140466	0.233419798	0.524279595	-0.06348281
NewGene_3670	--	5.745195	6.473464	5.670168	8.564401	6.871705	6.61264	5.893148	4.852076	5.925568	0.00000825	0.448789722	0.539806673	-0.063442686
ENSG00000136630	HLX	0.432172	0.629504	0.494472	0.926565	0.742687	0.630723	0.333665	0.454738	0.566157	0.049507211	0.707066076	0.89540209	-0.063439461
ENSG00000253729	PRKDC	48.98604	49.965233	48.667882	45.295586	43.218058	44.941478	43.812056	40.914163	48.494022	0.992625228	0.000778757	0.34874035	-0.063426617
NewGene_5708	--	0.330009	0.464738	0.37081	0.179063	0.212923	0.368749	0.461301	0.238323	0.339631	0.431105402	-0.44271952	0.903094919	-0.063245262
ENSG00000102531	FNDC3A	7.992295	8.981571	9.846019	9.614353	9.34974	9.876573	7.884433	7.596496	8.381923	0.005033593	0.239505192	0.540152312	-0.063162403
ENSG00000131626	PPFIA1	46.034511	44.24022	47.999932	45.761606	40.263554	41.286756	40.679574	38.764165	46.359424	0.717885906	0.028984188	0.431207696	-0.062989672
ENSG00000164307	ERAP1	5.37409	5.218439	5.085734	25.395842	23.982539	24.698257	4.457007	4.667561	4.943722	0	2.370142679	0.573969532	-0.062988416
ENSG00000060688	SNRNP40	37.916205	36.866392	33.821554	31.209615	33.123531	33.124973	33.213689	31.364699	32.638719	0.931027733	-0.010634015	0.489126102	-0.062963297
ENSG00000147419	CCDC25	12.811048	12.308883	11.960223	13.359572	12.389872	12.147695	11.385985	11.21849	11.324819	0.067352084	0.163464805	0.485209937	-0.062910134
ENSG00000166411	IDH3A	5.993265	6.60932	6.452073	7.213427	6.513468	6.6481	5.651516	5.291503	5.464559	0.045645678	0.202649687	0.583365254	-0.062818682
ENSG00000153046	CDYL	13.887539	12.304007	13.54864	12.12494	11.684864	11.37507	12.03294	11.945534	12.186997	0.760372776	-0.03657404	0.541095175	-0.062783166
NewGene_6394	--	1.471819	1.407379	0.930382	1.658281	0.392238	1.998045	1.801698	0.42565	1.218778	0.856829401	0.192061469	0.89684612	-0.062594177
ENSG00000188760	TMEM198	1.989094	1.655312	1.139687	1.872417	1.845386	1.729359	1.500249	1.327465	1.526978	0.219770533	0.336073856	0.840241952	-0.062576702

Gene ID	Gene name	Ctrl-1 FPKM	Ctrl-2 FPKM	Ctrl-3 FPKM	IFNγ-1 FPKM	IFNγ-2 FPKM	IFNγ-3 FPKM	Sora-1 FPKM	Sora-2 FPKM	Sora-3 FPKM	Ctrl vs IFNγ DESeq2 FDR	Ctrl vs IFNγ DESeq2 log2FC	Ctrl vs Sora DESeq2 FDR	Ctrl vs Sora DESeq2 log2FC
ENSG00000198804	MT-CO1	10049.0293	9761.991211	9625.883789	10104.42285	10240.77441	10027.24121	8781.088867	8885.847656	8752.288086	0.0000209	0.192626094	0.331046526	-0.062303553
ENSG00000163960	UBXN7	4.997024	5.40823	5.374394	5.728223	4.909199	5.231126	5.130445	4.608058	4.522817	0.077479631	0.145965523	0.531305956	-0.062303029
ENSG00000164924	YWHAZ	234.523854	233.959339	228.847461	195.162726	204.865971	204.70576	217.818786	189.844288	207.634612	0.043270597	-0.101718191	0.282486223	-0.062236739
ENSG00000021355	SERPINB1	39.385883	36.346527	38.082031	32.939209	33.372131	32.538898	34.553768	32.993938	34.70575	0.478418479	-0.056290017	0.419218134	-0.06216936
ENSG00000138279	ANXA7	33.619613	34.787005	32.357243	39.692817	36.740183	37.245601	30.145105	31.201872	28.695484	9.61E-08	0.310433838	0.45671642	-0.062148571
ENSG00000170667	RASA4B	3.405051	2.38285	2.25404	2.59238	1.987094	2.365565	1.924173	2.454886	2.822343	0.853443749	-0.052281218	0.799955481	-0.062115991
ENSG00000129493	HEATR5A	8.475247	8.011723	7.978149	6.761816	6.015241	6.156107	7.163156	7.112875	8.013357	0.000378944	-0.252382951	0.463292845	-0.062049651
ENSG00000157087	ATP2B2	12.148979	8.98628	8.18596	10.910929	10.739749	10.843073	8.227078	7.753196	8.945391	0.001888414	0.261916623	0.552514991	-0.06201157
ENSG00000187147	RNF220	30.955043	28.052003	27.296992	24.994301	26.068422	26.938056	24.697882	24.83374	28.091158	0.946098761	0.007909306	0.489014652	-0.06189476
ENSG00000135124	P2RX4	5.88886	4.617037	4.077526	4.687888	3.703962	4.19104	4.867697	4.135576	4.250446	0.690538192	-0.090282008	0.760135361	-0.061560239
ENSG00000122550	KLHL7	13.081122	11.718449	11.492457	7.772045	8.750299	7.899763	10.738119	11.850492	10.349468	0.000011	-0.421622476	0.617338574	-0.061326097
ENSG00000164329	TENT2	12.261081	13.365039	13.740471	13.302453	12.539809	12.623016	12.093409	10.343195	11.989579	0.20696939	0.111530868	0.5445691	-0.061284468
ENSG00000184304	PRKD1	6.733117	5.8113	5.942779	4.446582	4.533361	4.08877	5.440901	5.390419	5.995215	0.000211977	-0.396070521	0.623225192	-0.061221225
ENSG00000167244	IGF2	677.076031	592.858629	547.572399	890.324307	800.463056	885.501301	537.165096	563.593404	541.065932	1.11E-19	0.62430023	0.50539206	-0.061155844
ENSG00000163535	SGO2	7.005988	8.140653	8.472221	6.297928	5.877223	5.701562	6.271843	6.970935	8.0521	0.011150002	-0.259743589	0.635379687	-0.061112514
ENSG00000115525	ST3GAL5	2.337025	3.269189	2.919935	6.748866	7.927741	9.441827	2.709672	2.319436	3.065646	5.37E-45	1.494983386	0.747133298	-0.061057547
ENSG00000127990	SGCE	52.217049	46.615683	45.768341	42.037657	44.865438	39.448726	44.04786	39.697941	42.225204	0.592214826	-0.047768102	0.460770053	-0.061000782
ENSG00000139921	TMX1	13.510217	15.675537	16.389571	14.806004	14.976398	15.72321	12.374188	12.957812	15.790871	0.114529112	0.145575503	0.597673368	-0.06098637
ENSG00000153317	ASAP1	13.427763	13.401436	14.194945	14.719248	13.313544	13.150355	12.63502	11.276606	12.798345	0.042548581	0.157335793	0.504393217	-0.06095516
ENSG00000175727	MLXIP	4.537518	4.303104	4.176333	3.529188	3.361203	3.812209	4.245007	3.71441	4.831758	0.135770595	-0.144313268	0.605703241	-0.060904067
ENSG00000109205	ODAM	1.66801	1.295187	1.905515	0.563554	0.576577	0.414517	1.116905	1.667011	1.737764	0.0000354	-1.595073122	0.883201191	-0.060878726
ENSG00000085733	CTTN	142.600663	136.36114	138.371273	134.373557	124.917071	130.983428	122.882941	122.671863	132.750689	0.416519809	0.04975479	0.332318972	-0.060756801
ENSG00000120709	FAM53C	12.070931	11.467819	12.079897	9.588254	9.729505	9.428029	10.337681	10.860392	11.154801	0.041229543	-0.158681966	0.501106714	-0.060491215
ENSG00000163083	INHBB	4.889069	5.128075	6.038121	2.05632	2.953943	2.598589	4.316572	4.520848	5.619571	1.87E-10	-0.926109768	0.698893635	-0.060415759
ENSG00000101109	STK4	10.366076	10.995321	11.194423	9.973886	9.293242	9.964493	9.316675	9.250892	10.551595	0.994074892	-0.000910275	0.487970604	-0.06031444
ENSG00000233822	HIST1H2BN	1.125701	1.269487	1.217729	1.061363	0.889017	0.616891	0.791877	0.852615	0.875775	0.874000849	-0.062170475	0.836100126	-0.060264406
ENSG00000119638	NEK9	9.635538	9.707237	8.863813	8.371632	7.887043	7.736115	8.654616	7.81713	8.844027	0.363129889	-0.075860868	0.475955121	-0.060183497
ENSG00000185436	IFNLR1	1.916311	1.75281	1.919897	2.430541	2.21611	2.10603	1.81131	1.608313	1.728749	0.003125519	0.414430572	0.747602207	-0.060152199
NewGene_38	--	0.892656	0.825981	0.689899	0.955444	1.714355	2.045531	0.716921	0.893584	0.893269	0.002769781	1.101583775	0.904175935	-0.060028779
ENSG00000174720	LARP7	18.898879	21.885134	22.692814	22.304307	21.811865	20.359368	18.225653	17.705203	17.956004	0.024385025	0.186779455	0.538946058	-0.059911923
ENSG00000156017	CARNMT1	5.650174	6.231145	7.372281	7.827271	6.959851	6.758876	5.534066	5.272089	6.675234	0.006450793	0.304605081	0.675431542	-0.059870538
ENSG00000114021	NIT2	8.070747	8.787991	8.124213	7.909496	9.610492	7.22063	9.621664	10.703971	6.884353	0.454505058	0.065479751	0.541226061	-0.059746951
ENSG00000197586	ENTPD6	18.606792	17.724924	17.980813	15.679109	13.740641	16.342145	15.215548	15.351316	18.185503	0.194754315	-0.120116177	0.547706315	-0.059686503
ENSG00000108064	TFAM	18.256994	19.236642	19.769583	17.994905	16.628232	18.033514	16.238603	16.058466	18.037718	0.724809791	0.033432692	0.484178257	-0.059664278
ENSG00000155957	TMBIM4	39.439314	39.791399	37.279469	24.224265	32.095685	29.637022	33.995754	23.635435	25.62932	0.959391316	-0.005636454	0.538946058	-0.059635231
ENSG00000205643	CDPF1	3.031428	4.162264	3.678262	3.607711	3.883067	4.085879	3.332054	2.762214	3.431817	0.282982395	0.217124374	0.784516217	-0.059440218
ENSG00000165591	FAAH2	1.10342	0.711382	0.410931	0.701987	0.577488	0.694449	0.762622	0.770711	0.458921	0.976457703	-0.019285653	0.907613509	-0.059420451
ENSG00000118046	STK11	41.244416	38.645407	34.111339	39.339361	39.337978	40.822137	32.792984	31.938209	39.104293	0.004901252	0.219253921	0.561232192	-0.059253819
ENSG00000050820	BCAR1	29.237983	24.108495	25.506331	14.873178	15.80256	16.507067	22.476421	22.03604	27.959506	1.65E-13	-0.601665438	0.593644812	-0.059235882
ENSG00000132122	SPATA6	0.917564	0.987541	1.228168	0.421949	0.21744	0.262713	1.158568	0.614532	1.091584	0.000000249	-1.319111333	0.828187992	-0.059225204
ENSG00000189195	BTBD8	0.395285	0.393866	0.32154	0.421513	0.417068	0.374627	0.375917	0.282364	0.343087	0.375494903	0.276405974	0.861660824	-0.059132751
ENSG00000132639	SNAP25	4.152698	4.833473	4.959494	3.723178	3.919214	3.780522	4.828076	3.938242	3.797606	0.419794734	-0.142886485	0.755336029	-0.059029542
ENSG00000180776	ZDHHC20	20.641978	21.086359	20.201464	17.763173	18.520505	17.880485	19.114948	18.192069	18.808534	0.595858049	-0.04147634	0.416359112	-0.059011223
ENSG00000130147	SH3BP4	3.532566	3.096499	3.099719	3.297582	3.00237	2.661513	3.436049	2.600354	2.721516	0.91875658	0.019262332	0.713194756	-0.058916745
ENSG00000167625	ZNF526	2.808594	2.423511	2.541563	3.138046	2.621305	2.707895	2.36782	2.118339	2.439573	0.078086373	0.261538857	0.728112428	-0.058896104
ENSG00000168538	TRAPPC11	5.192218	5.15535	5.074014	5.935691	5.302232	5.662528	4.89103	4.514573	4.439423	0.002387224	0.280174821	0.616746528	-0.058890198
ENSG00000174428	GTF2IRD2B	8.427832	5.645986	7.020806	6.546918	9.649085	8.457961	6.131265	6.404109	6.330807	0.638334092	0.100033512	0.7304585	-0.058854265
ENSG00000198727	MT-CYB	2721.144287	2657.253662	2359.29834	2611.324951	2701.289795	2747.360107	2193.06543	2300.869873	2474.725342	0.001061987	0.206572575	0.459951218	-0.058825018
ENSG00000085840	ORC1	7.240108	7.862347	7.608539	6.90915	6.237364	7.678853	6.870288	6.784893	6.715233	0.850130166	0.029089649	0.609315699	-0.058793532
ENSG00000205464	ATP6AP1L	2.910229	2.060125	3.219758	1.973943	2.756744	2.582722	1.908717	2.362005	3.142494	0.973662659	-0.017097068	0.878442552	-0.058755258
ENSG00000079482	OPHN1	0.223727	0.215764	0.283761	0.234419	0.233114	0.228767	0.220307	0.166701	0.266959	0.826252675	0.09205201	0.877861219	-0.058739107
ENSG00000084073	ZMPSTE24	23.218601	25.393312	27.448359	24.604393	23.565857	23.814421	23.410238	22.289753	22.748987	0.458810874	0.06804028	0.531393136	-0.0587145
ENSG00000196482	ESRRG	1.89174	2.274188	2.455548	1.444351	1.65452	1.231749	1.787653	1.907959	2.378011	0.001228135	-0.526743267	0.74111276	-0.058645527
ENSG00000239887	C1orf226	6.552679	6.629997	7.552615	7.925163	7.130223	7.126401	6.15604	5.66336	6.599748	0.003437341	0.271407873	0.618101636	-0.058598308
ENSG00000171865	RNASEH1	9.752459	9.726373	11.219049	10.103654	9.006201	9.788173	8.217195	8.893186	10.615713	0.722641789	0.050951804	0.668396025	-0.058504645
ENSG00000176438	SYNE3	0.876625	1.072009	0.948799	1.211921	1.319458	1.27949	0.974974	0.848048	0.946188	0.00000344	0.545211597	0.709011946	-0.058434024
ENSG00000103642	LACTB	13.552542	13.751042	14.778026	13.477855	13.878198	13.14527	12.344139	12.234473	13.053908	0.292446409	0.112966806	0.596302299	-0.058298731
ENSG00000158186	MRAS	6.221842	5.773864	7.224594	8.03556	8.686931	8.062467	6.092172	7.313322	7.23463	2.51E-09	0.500481378	0.611978573	-0.058182381
ENSG00000115919	KYNU	0.458385	0.711896	0.973734	0.269351	0.77216	0.969589	1.261317	0.483339	0.500817	0.029870858	-0.443986005	0.798089512	-0.057914912
ENSG00000105011	ASF1B	19.432055	22.740412	20.016255	18.538599	16.454864	17.241796	18.981428	16.859073	20.352984	0.312474694	-0.099596205	0.58259406	-0.057871585
ENSG00000083223	TUT7	4.860113	5.010392	5.3499	5.155681	4.861544	4.597648	4.711022	4.4776	4.634271	0.549650771	0.068247621	0.584950988	-0.05786654
ENSG00000181555	SETD2	11.275553	12.001492	10.79679	11.36417	11.681026	10.699339	11.103105	9.299575	10.461246	0.010330431	0.166995051	0.50656182	-0.057859184
ENSG00000140497	SCAMP2	31.046141	30.138046	29.449923	27.381925	26.784455	29.089709	26.551627	27.157527	27.585762	0.747009163	0.030884046	0.483872939	-0.05782446
ENSG00000145687	SSBP2	2.651357	2.453232	4.677147	1.319837	1.486481	2.037613	3.248723	3.581093	4.47618	5.93E-08	-0.621236095	0.66546209	-0.057809527
NewGene_5127	--	1.576303	1.080796	1.602815	1.480002	2.133722	1.464154	1.389163	2.009188	1.163871	0.960393193	-0.022660346	0.865997538	-0.057489827
ENSG00000106565	TMEM176B	1.171466	1.264087	0.990947	1.558593	1.765848	1.724053	1.037134	0.993403	0.853708	0.036029176	0.676706242	0.894291486	-0.057475186
ENSG00000145743	FBXL17	1.562655	1.47721	1.690506	1.675211	1.610569	1.614115	1.586313	1.31686	1.359345	0.162234389	0.225691353	0.762728769	-0.057461892
ENSG00000113522	RAD50	5.513914	6.024916	6.428424	6.762379	6.038437	5.874909	4.937001	5.079666	5.809993	0.031174704	0.184548839	0.583594003	-0.057428947
ENSG00000072803	FBXW11	23.99954	25.273833	23.571179	17.82362	18.2976	17.116578	19.598438	21.041684	20.403703	0.00214178	-0.206354663	0.474622273	-0.057391808
ENSG00000164182	NDUFAF2	10.690542	9.975439	9.634902	9.99397	11.800474	10.835951	8.952003	10.92258	7.05903	0.135110769	0.257696619	0.807712958	-0.05738473
ENSG00000105327	BBC3	7.637427	7.172861	5.944907	5.63106	5.971262	6.274164	6.371936	5.528462	6.873299	0.717885906	-0.067802746	0.732647672	-0.057232444
ENSG00000198752	CDC42BPB	7.879066	7.986752	6.8915	7.949315	7.049134	7.518227	6.769756	6.103441	7.921012	0.150333332	0.135486189	0.609848518	-0.057096535
NewGene_5994	--	1.450676	1.72219	1.641615	2.491832	2.139325	2.164153	1.999517	1.675588	1.663273	0.021857046	0.460775001	0.821158253	-0.056972775
ENSG00000198453	ZNF568	1.694246	1.854155	1.67612	1.190881	1.031715	1.228739	1.897446	1.31835	1.737468	0.16842201	-0.306768301	0.812053278	-0.056664589

Gene ID	Gene name	Ctrl-1 FPKM	Ctrl-2 FPKM	Ctrl-3 FPKM	IFNγ-1 FPKM	IFNγ-2 FPKM	IFNγ-3 FPKM	Sora-1 FPKM	Sora-2 FPKM	Sora-3 FPKM	Ctrl vs IFNγ DESeq2 FDR	Ctrl vs IFNγ DESeq2 log2FC	Ctrl vs Sora DESeq2 FDR	Ctrl vs Sora DESeq2 log2FC
ENSG00000139719	VPS33A	12.406027	12.441433	12.458155	11.788506	11.548786	11.333567	11.201627	10.9135	11.279768	0.57153149	0.0495453	0.496971442	-0.056605334
ENSG00000125871	MGME1	20.880876	20.837334	20.679747	17.476736	16.577415	17.317101	19.859276	18.040213	18.396569	0.104555067	-0.131261476	0.540235776	-0.056583243
ENSG00000043514	TRIT1	9.614323	9.603627	3.305963	8.910685	6.372507	7.742943	7.814768	4.551175	8.122937	0.093700468	-0.209088647	0.663136458	-0.056582744
ENSG00000088930	XRN2	68.964142	68.783211	69.10762	57.187256	56.424187	56.46677	62.429745	62.047249	61.942802	0.008200477	-0.13502039	0.401293866	-0.056558868
ENSG00000141568	FOXK2	19.991928	18.495866	19.207343	18.9887	18.329752	18.063624	17.467626	15.44915	19.51159	0.201965222	0.091818214	0.55720405	-0.056471759
ENSG00000136243	NUPL2	32.677336	34.797475	37.606328	30.54885	29.075013	30.497294	27.63893	29.95551	30.958777	0.709252294	-0.03775693	0.573026151	-0.056400203
NewGene_1219	--	2.764066	1.404647	2.689597	2.880464	2.025771	2.4432	1.828152	2.474481	2.12544	0.632848055	0.186797725	0.880432744	-0.056321212
ENSG00000158691	ZSCAN12	4.484366	4.667215	5.07352	3.830332	3.878593	3.830572	3.964447	3.893723	4.373782	0.411138843	-0.103953181	0.66619098	-0.056249983
ENSG00000076662	ICAM3	5.26755	4.127506	4.407562	3.683952	4.147433	3.746802	3.750042	3.974307	3.956055	0.96371881	-0.01266154	0.787794914	-0.056225031
ENSG00000143515	ATP8B2	7.619634	7.405511	7.006687	7.632306	7.586347	7.358196	6.081954	6.161538	7.55342	0.010633587	0.196913545	0.58259406	-0.056134616
ENSG00000119929	CUTC	11.959231	12.926531	13.280351	10.671447	12.116162	9.893593	12.468119	11.924021	10.264575	0.564559128	-0.087780184	0.710028419	-0.056130461
NewGene_4772	--	2.654732	2.768876	3.959508	2.698376	2.954532	2.960598	3.508693	2.691319	2.223659	0.927641369	0.028900758	0.842545788	-0.056021237
ENSG00000139874	SSTR1	1.089785	0.954167	0.960741	1.586202	1.568305	1.710392	0.812113	0.905499	0.996508	0.000000418	0.838400655	0.821174576	-0.055947469
ENSG00000005059	MCUB	1.282604	1.422136	1.382271	2.343338	1.837944	2.560031	0.89968	1.468623	1.300851	0.0000204	0.871917758	0.859336002	-0.055712167
ENSG00000182327	GLTPD2	43.876326	38.556305	30.409037	42.902481	47.978046	50.038929	30.590631	33.308933	38.061256	0.000056	0.468615138	0.711199798	-0.05542111
ENSG00000071994	PDCD2	24.905692	23.3199	25.304263	21.299537	21.704408	23.126344	21.732516	20.856728	24.29347	0.613960433	0.047101069	0.51937464	-0.055152118
ENSG00000104756	KCTD9	9.042196	9.680547	10.851513	8.556694	8.429117	7.645811	8.901647	8.189277	9.543277	0.405880822	-0.100897906	0.642196561	-0.055035773
ENSG00000169410	PTPN9	4.646918	4.744387	4.666726	4.063324	4.240406	3.962821	4.49164	3.953907	4.246893	0.699256474	-0.043943968	0.591799415	-0.054955792
ENSG00000124459	ZNF45	4.433329	4.800934	4.969066	4.345267	4.669372	4.396621	4.7332	4.105842	4.073388	0.886046811	0.02308185	0.685800616	-0.054955286
ENSG00000085224	ATRX	9.403282	10.218421	11.211188	9.911782	9.745209	8.747122	10.056725	10.45225	10.897353	0.946618744	-0.008258031	0.56679058	-0.054857016
ENSG00000171564	FGB	17.643826	19.87161	18.024272	22.446734	18.045206	19.341038	19.707777	16.141567	14.071157	0.008042225	0.256059681	0.709844881	-0.054766809
ENSG00000152620	NADK2	85.538691	103.546451	99.53378	91.477357	86.137651	84.861661	85.042512	62.686063	88.155476	0.144739725	0.137156266	0.613972161	-0.054685447
ENSG00000140575	IQGAP1	13.874644	14.390176	14.90584	13.715602	13.49807	13.499241	13.065319	12.586565	13.380766	0.386094882	0.062591608	0.467793711	-0.054641955
ENSG00000169756	LIMS1	16.908208	18.157633	19.189603	17.451458	16.869757	16.465328	16.995334	14.908349	16.936349	0.322906186	0.086783054	0.583337958	-0.054613847
ENSG00000184661	CDCA2	7.736771	8.029906	7.942713	6.666711	5.562	6.158399	7.254153	6.219225	7.882446	0.022460986	-0.223027386	0.635442885	-0.054599283
ENSG00000112031	MTRF1L	7.313536	7.624092	7.219277	6.498964	6.93313	6.490884	6.17017	6.716784	7.055256	0.978324991	-0.00409422	0.62796455	-0.054586774
ENSG00000160050	CCDC28B	2.723059	3.116734	2.287273	1.990427	1.81359	1.561901	3.105672	2.177545	2.040651	0.058981693	-0.51539021	0.856552173	-0.054571213
ENSG00000107959	PITRM1	8.195232	7.945101	8.518856	9.277888	7.8148	7.772581	7.048491	7.489367	8.516132	0.10058735	0.17009153	0.63517794	-0.054563331
ENSG00000162434	JAK1	19.253071	19.405113	19.637835	15.803372	16.56921	15.922045	17.017635	16.999832	18.657686	0.064803207	-0.123927273	0.465084879	-0.05442629
ENSG00000050748	MAPK9	12.133522	13.860345	13.515064	12.056429	12.857984	11.687394	11.6278	11.089593	12.604479	0.975935104	0.003897206	0.569002808	-0.054343271
ENSG00000082213	C5orf22	16.912796	17.658792	17.272401	17.797282	18.004508	16.824911	16.76251	15.130148	15.242408	0.016627791	0.172246952	0.559553801	-0.054221455
ENSG00000138442	WDR12	8.094357	8.860866	9.434363	7.766595	7.386166	7.242236	7.951916	7.931796	7.939014	0.312904173	-0.089705303	0.565164363	-0.054018279
ENSG00000024048	UBR2	5.939484	5.723168	5.665329	8.339689	7.757437	7.59	4.958809	5.104167	5.616393	1.68E-19	0.596961934	0.566212907	-0.05399784
ENSG00000204209	DAXX	43.559222	42.575579	44.210725	40.485558	38.994712	39.763578	37.807402	37.510048	41.978557	0.80407858	0.020305335	0.4757104	-0.053948209
ENSG00000075618	FSCN1	27.782197	23.950972	21.332892	10.886275	10.12062	12.114866	19.853735	22.051486	24.143407	6.72E-25	-0.993064472	0.655903407	-0.053943292
ENSG00000146674	IGFBP3	93.305298	84.988708	75.553824	66.484987	74.423021	70.225189	74.195029	72.598896	82.595747	0.202146621	-0.118214849	0.566425803	-0.053921314
ENSG00000015133	CCDC88C	9.50091	8.547796	7.754253	6.808144	6.130429	6.6802	7.528971	7.158688	8.506274	0.012904847	-0.223002578	0.616185074	-0.053828653
ENSG00000119673	ACOT2	15.807556	12.993236	10.369765	14.776321	13.630562	13.676511	12.092623	12.343965	11.153641	0.064955138	0.256507259	0.74672037	-0.053666313
ENSG00000102034	ELF4	4.449543	4.862682	4.90218	3.568289	3.694483	3.934403	4.155485	4.552171	4.334721	0.073313059	-0.212806078	0.674910129	-0.053637165
ENSG00000117758	STX12	4.071498	4.841617	4.797756	4.399274	4.381085	3.788976	4.237577	3.897473	4.242251	0.91968243	0.020000512	0.720002466	-0.053587138
ENSG00000136636	KCTD3	36.297541	39.602249	40.618128	31.796752	34.764706	31.025066	34.744741	33.778377	37.828964	0.146180589	-0.115136499	0.498161157	-0.053378257
ENSG00000163964	PIGX	6.896809	7.257272	6.937764	6.363552	5.867052	7.116141	7.285136	5.602815	6.419289	0.64164323	0.066662482	0.690658456	-0.053330915
ENSG00000130489	SCO2	20.003944	16.028552	15.767436	25.747857	25.72167	27.821915	13.998697	15.371848	17.551005	1.36E-12	0.759914253	0.738673283	-0.053264353
ENSG00000241127	YAE1	21.249623	20.724814	20.863443	18.654476	19.481803	19.304111	18.204919	19.313213	19.263269	0.90346123	0.018518571	0.671158524	-0.053235831
ENSG00000028528	SNX1	17.789523	24.251283	22.636676	27.740795	32.628231	26.373709	23.041051	28.677571	30.276961	2.18E-16	0.489337143	0.489312977	-0.05322938
ENSG00000137812	KNL1	5.588951	6.559777	5.870846	8.652244	7.65359	8.100674	5.352665	5.130159	5.679013	4.58E-16	0.587034657	0.588636303	-0.052969037
ENSG00000140199	SLC12A6	3.324135	3.536157	3.351782	3.136709	3.191729	2.724696	3.263006	2.889306	3.058712	0.845630596	-0.028340174	0.656640175	-0.052740859
ENSG00000167723	TRPV3	0.334889	0.249797	0.244643	0.131186	0.113533	0.150929	0.234829	0.220634	0.237911	0.052600997	-0.79858069	0.899352327	-0.052665394
ENSG00000196914	ARHGEF12	12.112439	11.622864	11.491741	13.228862	11.23141	10.848284	11.210474	9.972334	11.429799	0.300594483	0.070191021	0.481118646	-0.052572825
ENSG00000119630	PGF	5.491819	4.289752	3.738699	2.889571	4.338128	4.433723	3.287726	4.172497	4.799844	0.866827514	-0.047972757	0.821424349	-0.052567908
ENSG00000087152	ATXN7L3	44.939761	42.87633	38.231796	39.620989	36.907077	39.186615	37.05984	35.667578	41.641864	0.977661662	0.003165974	0.556191063	-0.052539982
ENSG00000152464	RPP38	10.123129	8.195172	9.615336	8.393437	8.678907	8.878818	9.326052	7.917695	7.950691	0.686674823	0.071968609	0.759145136	-0.052500027
ENSG00000144736	SHQ1	4.450386	5.21164	5.233178	4.527185	3.869784	4.329105	4.866104	4.251944	4.350093	0.623313618	-0.081123198	0.735027601	-0.052350857
ENSG00000204444	APOM	31.531335	33.024372	28.501001	29.281258	25.665415	28.025443	30.992824	26.363752	24.117876	0.70994729	-0.047257175	0.6958622	-0.052349798
ENSG00000111737	RAB35	24.351794	22.599039	24.68201	20.695217	21.339281	22.815572	20.948947	21.145678	22.10672	0.974268263	0.003977303	0.535557912	-0.052191313
ENSG00000137770	CTDSPL2	11.418237	12.596106	12.260203	12.356827	12.561261	11.779379	12.413398	10.825875	11.33345	0.043770075	0.149046875	0.552514991	-0.05218716
ENSG00000178209	PLEC	8.093346	7.297084	6.202547	7.461726	6.502131	7.260506	7.139152	5.627586	7.361589	0.315668059	0.104890916	0.657077145	-0.052103123
ENSG00000164530	PI16	1.330859	1.704747	1.885921	1.56068	1.067226	1.521477	1.657865	1.409667	1.390164	0.815995431	-0.079957968	0.852465224	-0.052074704
ENSG00000169964	TMEM42	1.212314	1.137264	1.056908	0.850113	0.773234	0.938942	1.291269	0.950955	0.858927	0.590208565	-0.268809235	0.911430071	-0.052060569
ENSG00000084463	WBP11	28.121657	29.030174	28.264481	25.27338	24.906219	25.204599	25.855529	23.461601	28.281177	0.685663623	-0.030100921	0.506944044	-0.051971731
ENSG00000100030	MAPK1	23.43202	22.791361	23.339758	21.950617	22.670146	21.609288	21.277508	20.192885	22.638015	0.202127313	0.077773084	0.424973579	-0.051882977
ENSG00000196526	AFAP1	6.221881	5.616666	5.671257	3.446773	3.093092	3.749442	5.056384	4.911986	6.473217	2.1E-12	-0.618932578	0.665349731	-0.051745353
ENSG00000187546	AGMO	24.75057	21.531812	20.58053	12.699952	13.305988	13.552657	19.754374	19.582974	21.639542	5.95E-13	-0.620382722	0.628113173	-0.051638141
ENSG00000127337	YEATS4	26.59921	27.914171	29.035349	26.029192	24.956181	25.035871	26.037055	24.87836	24.174152	0.950119683	0.007999346	0.621328805	-0.051620231
ENSG00000196954	CASP4	32.785853	35.77993	38.946785	71.71372	73.469763	55.404983	27.2234	31.898217	33.537192	3.2E-50	0.959108819	0.611641259	-0.051589787
ENSG00000188559	RALGAPA2	11.612883	11.513377	11.848056	9.893635	9.356278	9.050627	10.152053	9.549337	12.295916	0.010535902	-0.165334844	0.583843474	-0.051534857
ENSG00000115816	CEBPZ	16.88036	17.845579	17.655787	15.724289	16.447584	15.610019	15.405719	16.108117	15.891156	0.887849044	0.014989942	0.559815707	-0.051483051
ENSG00000166889	PATL1	39.280876	39.171021	42.329586	43.584274	40.990028	40.868572	36.489761	34.564106	38.324776	0.000536896	0.201461592	0.483767505	-0.051396789
ENSG00000153029	MR1	1.168248	1.442723	1.230814	1.397926	1.647836	1.475475	0.941118	1.13192	1.27819	0.00129889	0.546207709	0.806625361	-0.051369724
ENSG00000078403	MLLT10	11.097463	11.523893	11.354145	10.025656	9.392893	10.258124	10.757126	9.898221	10.18442	0.785828692	-0.028124323	0.571362018	-0.051264379
ENSG00000164081	TEX264	9.192564	8.674673	6.631095	7.278535	8.818373	8.368553	7.226662	8.321587	8.068644	0.361542021	0.133163216	0.75130107	-0.051237238
ENSG00000143442	POGZ	23.132896	23.244338	23.700674	22.893108	20.328507	21.082211	22.061418	19.207204	21.91612	0.98194363	-0.002313557	0.512938045	-0.050965859
ENSG00000188501	LCTL	0.331195	0.364426	0.381136	0.395451	0.431504	0.610088	0.284647	0.353412	0.574564	0.388591778	0.364208968	0.913203239	-0.050949371
ENSG00000144395	CCDC150	10.699896	10.164817	11.128541	7.859019	7.13649	7.402295	10.285474	9.103564	9.413863	0.00000254	-0.405467502	0.629505231	-0.050918571

Gene ID	Gene name	Ctrl-1 FPKM	Ctrl-2 FPKM	Ctrl-3 FPKM	IFNγ-1 FPKM	IFNγ-2 FPKM	IFNγ-3 FPKM	Sora-1 FPKM	Sora-2 FPKM	Sora-3 FPKM	Ctrl vs IFNγ DESeq2 FDR	Ctrl vs IFNγ DESeq2 log2FC	Ctrl vs Sora DESeq2 FDR	Ctrl vs Sora DESeq2 log2FC
ENSG00000140905	GCSH	35.039566	36.702854	37.143175	34.544897	35.913426	34.151094	32.156062	33.630776	38.1826	0.262085581	0.096610793	0.586393963	-0.050876669
ENSG00000088205	DDX18	42.132413	45.759686	45.631001	39.324078	39.532654	40.036485	38.76109	39.161995	42.967865	0.815995431	-0.01942441	0.502998776	-0.050825809
ENSG00000065534	MYLK	4.167388	4.190879	4.591223	3.352904	4.576205	4.655584	4.17289	3.715929	3.993607	0.001718078	0.291070662	0.657607038	-0.050812415
ENSG00000167588	GPD1	1.018715	0.917153	0.976787	0.892527	1.022292	0.793618	1.003677	0.975496	0.635266	0.961904948	0.018271223	0.876109665	-0.050801868
ENSG00000105520	PLPPR2	2.755997	2.839502	2.819413	2.16365	2.52668	2.617527	2.327036	2.344053	3.023498	0.768185222	-0.065289546	0.798657309	-0.050736964
ENSG00000084764	MAPRE3	0.791869	1.021562	0.712848	0.734749	0.829743	0.577066	0.648663	0.765074	0.8526	0.858771125	-0.088641752	0.901344687	-0.050640054
ENSG00000022976	ZNF839	2.599273	2.742587	2.28855	2.817953	2.067831	2.446194	2.103373	2.204864	2.573151	0.943080528	0.01857588	0.806625361	-0.050576445
ENSG00000103769	RAB11A	30.498758	31.679804	33.399444	28.483196	27.165914	26.638487	27.771257	30.066779	28.243439	0.130486751	-0.101229791	0.524785336	-0.05038968
ENSG00000070423	RNF126	56.099116	53.997136	57.119981	61.82292	60.866361	61.935275	53.629366	49.500571	50.870224	0.00000133	0.278583421	0.519456147	-0.050338412
ENSG00000109445	ZNF330	11.712942	12.728013	13.634125	12.94591	10.851011	11.874095	12.210444	11.340731	11.690276	0.6672993	0.058577779	0.686447375	-0.050304672
ENSG00000164825	DEFB1	4.205403	4.896112	4.836616	1.501079	1.781235	2.032529	3.435398	5.17389	3.903681	0.000212388	-1.216779819	0.888357371	-0.050217022
ENSG00000141076	UTP4	32.095011	35.328277	35.540873	30.85939	28.638424	29.246954	30.201529	29.89675	31.55159	0.467011414	-0.067197562	0.577564013	-0.050197561
ENSG00000109084	TMEM97	75.083695	75.852326	74.977252	98.783515	104.008002	103.537502	72.498992	67.626513	65.084358	4.69E-31	0.583848209	0.536627415	-0.050057635
ENSG00000160194	NDUFV3	13.305658	14.125009	14.498098	14.985949	14.249542	13.888104	13.225306	14.185034	13.173904	0.035107725	0.197096714	0.692264639	-0.049841
ENSG00000160781	PAQR6	4.478066	3.493735	3.719112	3.405873	3.20678	3.257135	3.57824	3.241731	3.914752	0.485857676	-0.140132375	0.799235141	-0.049703102
ENSG00000204160	ZDHHC18	3.485072	3.09639	3.461797	3.387167	3.176274	2.969559	2.854011	3.055877	3.15263	0.563401807	0.083181939	0.718174794	-0.049595151
ENSG00000106086	PLEKHA8	8.943567	8.869507	8.490433	7.333304	6.65196	7.292404	7.951514	7.268975	8.397006	0.028732288	-0.155667255	0.55252454	-0.049457857
ENSG00000196405	EVL	10.246197	8.718531	8.754756	9.155653	8.461865	9.857486	9.335911	8.490206	8.702434	0.219840372	0.143773345	0.695620699	-0.04942149
ENSG00000146426	TIAM2	2.075188	2.20728	2.381485	1.36034	1.189487	1.064557	2.406713	1.744927	2.137493	7.48E-08	-0.673433905	0.763410877	-0.049276756
ENSG00000047579	DTNBP1	16.440807	14.196288	15.935445	19.820654	18.710893	22.465114	12.613613	14.649846	14.693782	5.55E-08	0.541600701	0.719855996	-0.049274655
ENSG00000178913	TAF7	60.67514	55.70229	54.474133	50.975858	51.838791	51.068245	52.434231	50.515572	51.876595	0.970689495	-0.003908151	0.540919903	-0.049252972
ENSG00000155368	DBI	301.924125	261.649457	286.119016	312.680526	335.204925	315.478005	267.350032	280.181708	219.857643	0.000000854	0.326900594	0.686624475	-0.04914782
NewGene_1709	--	8.651518	9.160344	8.438894	8.208678	7.688812	8.726167	8.105047	7.88366	7.815971	0.768089365	0.055053037	0.763805651	-0.049118275
ENSG00000188582	PAQR9	23.15905	24.023082	20.712683	37.388935	35.963239	36.958497	21.032624	20.447633	19.99922	1.31E-37	0.8476973	0.626769268	-0.049005727
ENSG00000111725	PRKAB1	16.409241	15.217193	14.97053	11.446391	10.986202	11.708777	13.290885	15.194709	14.358024	0.000258428	-0.304276117	0.660536953	-0.048949731
ENSG00000171606	ZNF274	22.469691	21.928057	22.792221	18.53269	18.19984	17.961659	20.600961	18.705244	21.934511	0.029734241	-0.159150106	0.580533154	-0.04878205
ENSG00000179295	PTPN11	49.490764	51.182393	55.135777	36.754107	35.03056	35.645256	48.283776	43.624364	49.376623	3.35E-13	-0.390635885	0.518451594	-0.048781275
ENSG00000197183	NOL4L	7.870373	7.327144	7.774959	7.516239	6.20769	6.942806	6.880237	7.434229	8.312396	0.638129876	-0.050780284	0.623974038	-0.048764143
ENSG00000114019	AMOTL2	21.605487	19.525658	20.562695	7.798087	8.739987	8.14506	18.96503	17.208332	20.178189	8.28E-63	-1.169625306	0.57581045	-0.048743375
ENSG00000182272	B4GALNT4	15.04365	14.686262	14.99149	12.235196	11.732578	12.807606	13.136468	12.398395	15.363135	0.09361374	-0.136620073	0.621529898	-0.048181958
ENSG00000099139	PCSK5	0.156967	0.181679	0.225388	0.09248	0.122238	0.141197	0.124759	0.167333	0.176196	0.30661191	-0.471741866	0.912847752	-0.048089887
ENSG00000182287	AP1S2	2.846811	2.605987	3.299862	2.133273	2.086104	2.039407	2.257346	2.468425	3.399661	0.076255195	-0.313025473	0.832687557	-0.04801578
ENSG00000215883	CYB5RL	1.174079	1.321477	1.067962	1.218753	0.79342	0.925307	1.593703	1.027759	1.8244	0.310187883	-0.325799512	0.877927524	-0.048005184
ENSG00000086589	RBM22	15.076922	13.632094	14.064209	13.196338	11.811332	13.120646	13.343509	11.819519	13.736963	0.8787686	-0.020242799	0.658526607	-0.047918338
ENSG00000134313	KIDINS220	8.785229	8.71817	7.884726	6.856055	6.994304	7.292758	7.398748	7.403996	7.828405	0.139891828	-0.118780662	0.581262421	-0.047892992
ENSG00000100425	BRD1	11.901996	11.044517	12.837892	12.04447	10.307853	11.321432	13.278331	9.70975	11.705069	0.377441966	0.08170523	0.644085667	-0.047763712
ENSG00000084628	NKAIN1	1.145171	1.251913	1.261694	0.650612	0.783342	0.663798	1.082849	1.046396	1.187851	0.007559461	-0.700308082	0.864813037	-0.047759892
ENSG00000166503	HDGFL3	6.386093	6.673331	6.763488	5.380726	4.606579	4.924491	5.393962	5.428674	5.955525	0.0000069	-0.337157604	0.610372761	-0.047717536
ENSG00000186687	LYRM7	1.788013	2.181429	2.114315	1.706235	1.521172	1.9774	1.968863	1.909619	1.674469	0.700824443	-0.074010692	0.780010678	-0.047675044
ENSG00000135622	SEMA4F	2.282885	2.690846	1.987618	1.827214	1.572618	1.411188	2.486235	1.891808	3.636058	0.000139645	-0.577442789	0.767978063	-0.04767067
ENSG00000075303	SLC25A40	8.61993	9.456273	9.166343	9.664576	9.804941	10.058299	8.533606	7.768124	9.439696	0.008892823	0.222397451	0.653733091	-0.047648074
ENSG00000127324	TSPAN8	17.640662	15.618145	15.145624	11.462536	13.595698	12.836899	14.600874	14.87355	14.474445	0.11844692	-0.204016911	0.721516584	-0.047646837
ENSG00000126351	THRA	4.70377	4.095915	3.229799	4.750149	4.629968	4.350486	4.277783	4.106627	3.523888	0.0000935	0.442640825	0.767715478	-0.047568526
ENSG00000102181	CD99L2	0.570713	0.963647	0.70049	0.864917	0.866997	0.893415	0.897494	0.877424	0.770253	0.557104553	0.169922724	0.86910175	-0.047505012
ENSG00000115524	SF3B1	99.543344	101.79057	95.64124	92.792577	91.578945	88.901754	91.035618	88.578419	92.207113	0.761829818	0.02030646	0.441900971	-0.047500466
ENSG00000163636	PSMD6	49.374194	48.0061	49.873638	45.588654	41.325378	44.21051	44.261028	42.011146	46.574656	0.833823244	-0.020265103	0.559232619	-0.047460569
ENSG00000204580	DDR1	12.691113	12.953982	12.738066	9.382196	8.500059	9.05786	11.657961	10.961915	12.392072	1.18E-08	-0.404704917	0.595160608	-0.047432193
ENSG00000165704	HPRT1	16.100401	19.294155	20.079704	17.036047	16.157845	17.270342	17.827465	14.464248	18.143377	0.940414306	0.012256896	0.735767366	-0.047276512
ENSG00000167670	CHAF1A	33.082685	32.293014	30.961842	29.176309	26.306277	27.310099	28.732376	25.000353	32.858561	0.244160172	-0.083711436	0.616987414	-0.047068854
ENSG00000198561	CTNND1	80.850263	78.535986	81.941187	68.487348	69.57144	63.525249	73.539263	72.230736	70.447936	0.001180446	-0.152229619	0.466998848	-0.047000966
ENSG00000166913	YWHAB	84.395774	90.246652	93.793438	80.327279	74.580497	80.352487	80.840144	78.508087	87.396147	0.605232758	-0.038374073	0.501853004	-0.046957812
ENSG00000130589	HELZ2	2.199443	1.908746	1.651131	7.659761	7.039546	7.865638	1.669013	1.525925	2.048842	4.27E-114	2.11931224	0.786354517	-0.046954155
ENSG00000172572	PDE3A	3.392063	3.506833	3.769833	1.675296	1.631485	1.544843	3.254939	3.27831	3.139924	3.11E-25	-0.989798685	0.688202819	-0.04694594
ENSG00000136111	TBC1D4	5.715021	5.422469	6.07802	4.906512	4.812184	4.911707	4.963922	4.774063	6.051995	0.293512623	-0.102907683	0.685649951	-0.046798274
ENSG00000176871	WSB2	32.009014	28.641566	31.784616	19.705455	20.603347	18.490271	26.172461	25.659122	31.650232	7.82E-14	-0.519740018	0.620430159	-0.046759663
ENSG00000074696	HACD3	90.416583	96.859041	93.097315	78.698389	81.088986	82.248912	83.579409	82.836828	88.665633	0.184793087	-0.079452843	0.474406436	-0.046664319
ENSG00000167700	MFSD3	15.960008	16.215206	11.320181	12.63726	13.543175	14.928922	11.981305	13.972178	13.497847	0.717885906	0.067335351	0.784570597	-0.046562799
ENSG00000124275	MTRR	12.344387	15.659839	15.689317	14.929596	16.693694	14.721168	13.179198	12.862337	15.484087	0.009910749	0.261446947	0.688017548	-0.046460344
ENSG00000168591	TMUB2	16.60425	15.297357	14.547804	14.903864	16.111638	15.231381	11.774126	14.515723	15.277359	0.292147458	0.112983665	0.735418779	-0.046424524
ENSG00000184916	JAG2	2.799534	2.851609	2.849706	2.645118	2.521216	2.718784	2.38569	2.220737	3.167236	0.80753236	0.036700591	0.767927269	-0.046377572
ENSG00000140563	MCTP2	0.724948	0.802068	0.819769	1.094347	0.678324	0.457575	0.817445	0.691071	0.774823	0.665375603	0.128082685	0.862002485	-0.046336626
ENSG00000084731	KIF3C	0.404349	0.347362	0.469195	0.348185	0.411098	0.33319	0.354267	0.361333	0.384964	0.860877732	-0.073369293	0.891797248	-0.046167132
ENSG00000106682	EIF4H	180.482857	170.510422	169.070381	170.433663	166.806416	176.932347	160.134499	145.183489	170.986711	0.01508729	0.135097675	0.523267464	-0.046162326
ENSG00000162631	NTNG1	0.442884	0.687402	0.500499	0.216729	0.31143	0.352861	0.656511	0.492563	0.552134	0.016352417	-0.880241374	0.904794479	-0.046113912
ENSG00000262664	OVCA2	11.271754	10.488793	11.854918	11.295388	10.015542	11.178928	11.676328	10.411654	8.361385	0.527592567	0.098933131	0.805144076	-0.045990644
ENSG00000197747	S100A10	8.717302	7.908004	5.274629	3.549716	3.410253	4.048945	6.835683	6.594131	6.4272	0.0000228	-0.841164147	0.840396715	-0.045893696
ENSG00000102221	JADE3	4.520958	5.184156	5.409358	5.318394	5.108938	5.14645	5.08857	4.261673	4.385624	0.061036791	0.194697125	0.736612264	-0.045859799
ENSG00000177370	TIMM22	8.223216	9.065669	8.354291	7.06926	7.805352	7.106188	7.925943	7.782327	7.557603	0.547964042	-0.074043272	0.691257715	-0.04583462
ENSG00000168288	MMADHC	84.662643	84.907702	89.263717	76.283164	70.030019	75.757795	75.501558	79.108778	76.64446	0.53348322	-0.045577553	0.553761221	-0.045741026
ENSG00000136810	TXN	83.410631	86.8331	93.882423	83.340004	78.929207	84.543936	82.83552	86.14109	70.176025	0.582645755	0.050144364	0.704302517	-0.045690557
ENSG00000104427	ZC2HC1A	2.194179	2.740728	2.219694	2.034407	1.720949	2.079829	2.286	2.051568	2.384169	0.279550364	-0.21209491	0.820090221	-0.045669983
ENSG00000166847	DCTN5	15.3315	16.314973	16.215007	15.349726	14.675384	14.964525	13.801287	13.118887	15.983017	0.310333492	0.064557355	0.521284331	-0.045579599
ENSG00000153406	NMRAL1	19.689896	19.978303	18.349429	19.618066	17.992298	19.873894	15.942422	16.934966	18.184956	0.291460472	0.121221434	0.715712396	-0.045577571
ENSG00000110777	POU2AF1	0.639802	0.537315	0.585555	0.237502	0.523071	0.334601	0.525974	0.523462	0.554124	0.192255225	-0.530381504	0.898487896	-0.045566313

Gene ID	Gene name	Ctrl-1 FPKM	Ctrl-2 FPKM	Ctrl-3 FPKM	IFNγ-1 FPKM	IFNγ-2 FPKM	IFNγ-3 FPKM	Sora-1 FPKM	Sora-2 FPKM	Sora-3 FPKM	Ctrl vs IFNγ DESeq2 FDR	Ctrl vs IFNγ DESeq2 log2FC	Ctrl vs Sora DESeq2 FDR	Ctrl vs Sora DESeq2 log2FC
ENSG00000131981	LGALS3	8.182838	6.787199	7.802518	5.019653	4.357018	4.294512	5.129525	7.33449	8.205534	0.000263273	-0.590536002	0.838561274	-0.045555214
ENSG00000157654	PALM2-AKAP2	6.227864	6.484263	6.672054	4.047921	4.137735	4.704431	6.898941	5.532264	6.674005	1.13E-08	-0.443896845	0.629857405	-0.045535211
ENSG00000177058	SLC38A9	11.317676	10.577977	11.616653	9.58011	10.307812	10.229063	10.525929	10.966929	10.621359	0.231874278	-0.129174218	0.673401191	-0.04552768
ENSG00000107960	STN1	4.648714	4.391344	3.883089	3.677686	2.979307	2.73577	3.916291	4.099189	3.70034	0.144224409	-0.308947298	0.83452905	-0.045460297
ENSG00000163638	ADAMTS9	3.743467	3.490898	2.76946	2.387268	2.174956	2.201783	2.975209	2.965492	3.201165	0.000274689	-0.426979243	0.743889313	-0.045308626
ENSG00000173890	GPR160	20.234814	20.594534	25.288427	13.994173	13.119572	14.788404	20.156784	19.257977	20.926168	0.000000148	-0.516099591	0.705700819	-0.045303062
ENSG00000154059	IMPACT	8.78786	9.846116	11.027467	9.933682	9.714664	9.676795	9.223621	8.026308	9.891301	0.235606365	0.128055244	0.716753793	-0.045243609
ENSG00000213420	GPC2	2.758417	1.949996	1.893932	1.389333	1.149205	1.291066	2.069009	2.593112	1.591167	0.003067585	-0.684198964	0.872100292	-0.045185158
ENSG00000118242	MREG	8.293089	10.382492	9.426342	7.208585	5.863188	6.44758	7.791978	8.250355	9.192853	0.000116338	-0.432234582	0.734223274	-0.045143558
ENSG00000178401	DNAJC22	24.863183	26.044526	25.920234	19.487677	18.181417	18.796412	22.850661	21.995929	24.71438	0.000859013	-0.261512588	0.587093336	-0.045133759
ENSG00000047932	GOPC	12.06139	12.512192	12.823358	13.029881	11.895681	12.612205	11.74936	10.576608	11.83029	0.051083245	0.143641513	0.608937167	-0.045065968
ENSG00000003393	ALS2	14.644777	13.827023	13.287992	10.788091	10.511083	10.520321	14.118039	12.561638	13.194964	0.00024742	-0.264143673	0.621092499	-0.045010159
ENSG00000123575	FAM199X	7.214703	8.028183	7.754442	7.886941	7.335422	7.15027	6.781829	6.567348	7.574482	0.204590981	0.107289059	0.630898035	-0.044995497
ENSG00000134202	GSTM3	10.708802	10.116805	10.686412	7.573687	8.550576	6.266475	8.846482	9.412508	10.516836	7E-12	-0.591312248	0.684998582	-0.044942684
ENSG00000032219	ARID4A	4.954302	5.589645	5.465333	6.057898	6.053671	5.898078	5.123366	4.71105	4.969313	0.000359248	0.308706454	0.693182126	-0.044784126
ENSG00000136807	CDK9	19.113262	17.232182	17.478761	18.876474	17.838229	17.0878	14.64034	16.124505	18.453493	0.109823632	0.14130368	0.696028893	-0.044644671
ENSG00000179241	LDLRAD3	1.402497	1.549432	1.44872	0.788025	0.961617	0.771719	1.291973	1.330748	1.328598	0.000118711	-0.744779185	0.837641334	-0.044638699
ENSG00000105135	ILVBL	23.969127	24.596431	20.291075	20.888719	21.18075	22.435751	20.101897	21.309554	22.587438	0.87244079	0.021257381	0.685847365	-0.044586038
ENSG00000127418	FGFRL1	24.669158	22.64331	22.894453	18.651844	20.118154	20.17207	20.803393	21.053364	22.554208	0.181367264	-0.108979357	0.59877202	-0.044552012
ENSG00000139209	SLC38A4	2.660268	2.001725	1.670988	2.230717	2.440685	2.420911	2.138375	1.610838	2.229782	0.182309036	0.26810176	0.852830205	-0.044519109
ENSG00000123607	TTC21B	9.831713	10.569178	10.563549	8.797001	8.186793	7.67053	9.021354	8.679384	9.56883	0.021445027	-0.181850652	0.614243075	-0.044515425
ENSG00000163362	INAVA	12.546537	11.392634	10.391624	9.032161	8.724006	9.126927	10.469097	9.462904	11.863635	0.031172666	-0.191141364	0.68801931	-0.044510357
ENSG00000188283	ZNF383	0.912841	1.127564	1.337607	0.833711	0.8511	1.1103	0.951021	0.88406	1.297265	0.428704664	-0.17856747	0.839845341	-0.044395516
ENSG00000148444	COMMD3	22.057638	19.757397	20.338806	16.219955	17.477965	15.530824	18.944103	17.628864	18.801338	0.037982926	-0.226991631	0.710932447	-0.044280141
ENSG00000136122	BORA	2.992373	3.649178	3.125644	2.49716	2.351305	2.60008	3.159445	2.959372	2.769036	0.148095937	-0.250322326	0.81397511	-0.044276745
ENSG00000143845	ETNK2	6.908546	5.304525	5.256546	4.568643	4.414143	4.710124	5.204124	5.249833	5.360784	0.227348016	-0.197288813	0.796487825	-0.044227394
ENSG00000008710	PKD1	1.797473	1.629569	1.459066	1.580923	1.497778	1.560435	1.39062	1.303943	1.684923	0.636830398	0.065634536	0.755336029	-0.044128467
ENSG00000162704	ARPC5	21.144395	20.685179	27.250694	16.631763	17.609165	19.354408	18.854985	32.860542	19.156788	0.714293878	-0.032894257	0.571558279	-0.043972216
ENSG00000171169	NAIF1	2.580134	2.329102	2.707409	2.523866	2.217773	2.422494	2.360075	2.191279	2.379311	0.778557715	0.057670858	0.806962785	-0.043890169
ENSG00000198853	RUSC2	5.162719	4.725449	5.075686	2.662117	2.415192	2.808973	4.539032	3.854846	4.912572	3.03E-15	-0.745897499	0.711204872	-0.043804684
ENSG00000089060	SLC8B1	3.407789	1.679104	1.29609	1.128782	0.936983	1.572917	1.922473	1.845018	1.463782	0.042887893	-0.526476195	0.883530071	-0.043747893
ENSG00000105819	PMPCB	38.851288	42.39743	44.102297	42.711404	37.224077	39.736367	38.009321	36.207642	39.187304	0.734404675	0.030707652	0.586266433	-0.043705499
ENSG00000162819	BROX	9.884208	13.047533	13.68085	12.449908	12.408276	11.784773	11.081915	10.228359	12.048621	0.237804235	0.144434239	0.748551461	-0.043609877
ENSG00000129351	ILF3	133.665597	136.746773	138.938084	133.553513	112.021378	116.212269	125.016177	122.745524	131.654248	0.777176651	-0.02136158	0.506605423	-0.04345176
ENSG00000161395	PGAP3	10.782503	11.427399	10.307848	8.48319	7.544452	8.197551	9.610749	9.163578	10.293693	0.016541418	-0.239017713	0.703415152	-0.043421684
ENSG00000109111	SUPT6H	37.056043	36.614296	35.415016	38.248845	37.434657	37.508774	31.911121	29.520009	35.996415	0.000114504	0.19416219	0.586079576	-0.043367568
NewGene 5656	--	0.909579	1.140654	1.156492	1.088722	0.952827	0.996799	0.871596	0.746684	1.303861	0.682799116	0.131635602	0.890499304	-0.04330255
NewGene 2678	--	2.433045	2.402744	2.799006	2.083991	1.805716	1.665066	2.031253	2.10521	3.043264	0.331728434	-0.187863137	0.831392004	-0.043247606
NewGene 779	--	1.17855	1.75684	1.322369	1.067378	1.070794	1.506659	1.223173	1.204871	1.47185	0.488853834	-0.200464475	0.873988967	-0.043183903
ENSG00000171840	NINJ2	2.94142	3.104898	2.779285	3.611568	3.250149	2.707649	3.257738	2.615091	2.119996	0.684290694	0.141231535	0.898386694	-0.043133662
ENSG00000198265	HELZ	6.175676	6.474496	6.601273	7.097661	6.375524	6.495982	6.014529	5.782081	5.971562	0.003186488	0.206056995	0.600775835	-0.043058346
ENSG00000135823	STX6	10.368367	10.818932	10.317017	9.03863	9.110933	9.289537	9.234704	9.448846	9.988619	0.584610775	-0.051096987	0.632145356	-0.043058013
ENSG00000151498	ACAD8	6.369671	5.515153	5.698411	4.834501	5.058842	4.633601	4.93346	5.086356	5.945811	0.458925386	-0.119461583	0.793688284	-0.043024399
ENSG00000168286	THAP11	12.851663	12.215476	11.772537	10.849038	10.727542	12.258289	10.549079	11.320264	11.647469	0.857026623	0.026010072	0.713240802	-0.042984665
ENSG00000254469	AP002495.1	1.020124	1.159745	0.815861	0.962886	1.007189	1.020157	0.678647	0.955893	0.862114	0.649986685	0.220693328	0.927848034	-0.042935857
ENSG00000148814	LRRC27	0.258647	0.662372	0.272855	0.40567	0.271094	0.137319	0.214955	0.209627	0.226921	0.538190721	-0.302194401	0.919767549	-0.04286995
ENSG00000168556	ING2	7.652021	7.053197	7.314019	7.351777	6.895644	7.978658	7.396451	6.299718	5.760861	0.406648642	0.149743468	0.829494762	-0.042858448
ENSG00000134001	EIF2S1	16.840494	17.448762	18.986887	17.896661	16.631273	17.729844	15.518284	16.229999	16.557866	0.134868015	0.115189052	0.633644834	-0.042834177
ENSG00000007520	TSR3	23.348348	22.618025	21.20945	17.685425	18.078829	20.680413	19.454561	19.884812	20.34735	0.448234943	-0.094167987	0.696506836	-0.042812967
ENSG00000120913	PDLIM2	3.237311	3.531752	2.55076	2.534768	3.130175	3.430483	3.09477	3.627417	3.492697	0.973662659	-0.011459407	0.852465224	-0.042803834
ENSG00000145808	ADAMTS19	1.205724	1.602838	1.311739	0.663215	0.694762	1.025276	1.038927	1.206747	1.592831	0.002016077	-0.655384362	0.853932034	-0.042727689
ENSG00000118849	RARRES1	2.207686	1.887819	2.088015	4.759951	5.286234	5.24319	2.102356	1.773115	1.549828	2.86E-23	1.479630211	0.876349296	-0.042660602
ENSG00000110245	APOC3	28.973758	22.585976	17.822499	24.112799	25.194646	25.590502	24.046263	23.177968	15.702588	0.151340708	0.256283968	0.860274519	-0.042650772
ENSG00000162851	TFB2M	24.686045	26.354382	28.170713	20.286289	21.036911	21.269686	23.668396	23.787964	24.626074	0.025260915	-0.19145037	0.665116608	-0.042567507
ENSG00000164305	CASP3	11.785351	12.807593	12.232532	20.747234	18.385414	19.094273	11.269205	10.715091	11.579088	1.85E-30	0.829742272	0.688295156	-0.042437111
ENSG00000103152	MPG	11.342104	10.589752	8.600628	8.959665	9.633781	10.644636	9.824467	8.680176	9.285385	0.650216845	0.085919747	0.80577645	-0.042425257
ENSG00000078140	UBE2K	50.27019	57.565681	58.7041	61.613525	62.711853	62.680606	51.953029	49.194232	51.24865	0.000000516	0.309559701	0.611041823	-0.042418782
ENSG00000112365	ZBTB24	4.75125	5.054477	5.408621	4.165115	3.984979	4.152803	4.926848	4.107487	4.834582	0.124838828	-0.158298913	0.728112428	-0.042397977
ENSG00000169021	UQCRFS1	32.798134	32.214996	31.61904	29.195147	29.875803	30.562349	29.016943	28.418665	30.59511	0.620722991	0.039225812	0.562098656	-0.042320228
ENSG00000144959	NCEH1	2.52783	3.115196	3.558144	2.108213	2.483734	2.017993	2.720124	2.607279	3.038493	0.023492858	-0.347896226	0.803716729	-0.042286997
ENSG00000109861	CTSC	145.804038	142.470744	149.50738	101.629284	106.99859	104.288644	127.412456	136.141722	137.268688	5.3E-11	-0.349200104	0.568411382	-0.04221589
ENSG00000178585	CTNNBIP1	38.704432	38.845289	38.854879	24.287655	29.626106	27.807672	36.71889	39.196638	38.157938	0.000000211	-0.393993173	0.597257253	-0.042088491
ENSG00000096746	HNRNPH3	52.582383	59.114316	61.479937	58.249202	55.202331	54.672492	51.484199	51.888732	55.756028	0.206145591	0.101975639	0.641212786	-0.041964296
ENSG00000091732	ZC3HC1	15.132995	16.453437	17.508447	14.958945	12.072324	12.705783	15.140002	13.46229	14.98778	0.310915464	-0.104882078	0.699398414	-0.041946236
ENSG00000143207	COP1	28.674475	26.062102	25.36768	24.961644	25.194914	25.172952	24.511547	23.137753	23.663265	0.37601374	0.070112962	0.624142459	-0.041815714
ENSG00000111358	GTF2H3	15.576735	18.260779	20.870229	16.614073	15.47277	16.949772	15.76734	14.662972	15.185176	0.133084524	0.128765833	0.660834181	-0.041790649
ENSG00000164880	INTS1	31.607593	29.992411	26.44147	27.302336	25.761535	27.589666	24.063807	24.352499	32.129933	0.85863081	0.018703469	0.722184083	-0.041789652
ENSG00000151687	ANKAR	0.449315	0.532437	0.420883	0.331974	0.288608	0.379398	0.388409	0.509367	0.368721	0.355464357	-0.350519078	0.912847752	-0.04176603
ENSG00000121481	RNF2	9.945754	9.71714	10.444954	8.597543	8.824446	8.002537	9.209393	8.943026	9.288507	0.363224226	-0.094622924	0.690107271	-0.041662561
ENSG00000159348	CYB5R1	25.810625	24.890538	24.749589	19.813338	20.02014	20.309493	23.07903	20.544767	25.081701	0.026570334	-0.183777041	0.683842523	-0.041615428
NewGene 2311	--	0.866276	0.616611	0.427794	0.335652	0.479765	0.35576	0.306495	0.311182	0.535687	0.681847625	-0.236032985	0.937259399	-0.041607342
ENSG00000166188	ZNF319	1.280873	1.409039	1.577834	1.406849	1.381607	1.596625	1.383081	1.150682	1.267759	0.337162301	0.20064842	0.852140447	-0.041455133
ENSG00000157796	WDR19	8.312596	8.114247	7.402352	6.110833	6.688313	6.83022	6.537638	7.148614	8.167996	0.237556219	-0.125837848	0.725286869	-0.041432324
ENSG00000072310	SREBF1	55.660366	53.651636	46.969588	52.813431	51.719712	56.417519	47.45219	45.551756	51.432845	0.007159677	0.202007902	0.653264498	-0.041370819

Gene ID	Gene name	Ctrl-1_FPKM	Ctrl-2_FPKM	Ctrl-3_FPKM	IFNγ-1_FPKM	IFNγ-2_FPKM	IFNγ-3_FPKM	Sora-1_FPKM	Sora-2_FPKM	Sora-3_FPKM	Ctrl_vs_IFNγ_DESeq2_FDR	Ctrl_vs_IFNγ_DESeq2_log2FC	Ctrl_vs_Sora_DESeq2_FDR	Ctrl_vs_Sora_DESeq2_log2FC
ENSG00000184436	THAP7	17.409524	17.823028	16.020306	16.445995	16.451919	16.465061	14.93153	15.215038	16.618909	0.415352174	0.099961834	0.748966844	-0.041340238
ENSG00000078674	PCM1	25.977837	27.150881	27.400242	36.258728	32.108037	31.440055	26.026449	22.941097	26.671475	2.06E-17	0.441370417	0.565735363	-0.041312992
ENSG00000198734	F5	41.931576	37.397823	39.207218	35.473167	33.997379	34.00124	34.871034	35.397411	37.788673	0.511129166	-0.049197341	0.591224545	-0.041310101
ENSG00000139116	KIF21A	21.998985	23.773483	25.230459	20.442307	20.006437	18.418973	23.51966	20.383062	22.635643	0.213012103	-0.094111653	0.604837658	-0.041086655
ENSG00000160124	CCDC58	26.262356	23.499901	24.913345	23.400653	20.941262	22.108602	21.777924	26.26589	20.423205	0.809998033	-0.037477854	0.793547431	-0.041020706
ENSG00000162607	USP1	28.293831	31.997974	32.390316	34.723409	32.565215	33.514515	28.872907	25.60415	29.671333	0.0000167	0.274490321	0.642292743	-0.041010483
ENSG00000189159	JPT1	35.814302	35.660664	35.145243	30.20353	26.946567	28.637444	33.596651	32.917767	30.479343	0.02004497	-0.173242599	0.679660215	-0.040894793
ENSG00000075131	TIPIN	7.920729	8.421614	7.289283	6.89335	6.245172	6.428431	7.326222	6.867424	7.800016	0.571028914	-0.090797342	0.78872154	-0.040765446
NewGene_4147	--	1.423767	1.25133	1.551308	1.359104	0.995619	0.982606	1.737643	1.416591	1.435919	0.152932635	-0.359022024	0.874214	-0.040703726
ENSG00000164038	SLC9B2	8.320111	8.169399	8.798431	7.347772	6.875816	6.903593	6.662062	7.149181	7.44615	0.850678092	-0.028269763	0.748352491	-0.040607422
ENSG00000113758	DBN1	90.663888	81.336596	78.000541	65.444249	61.632989	66.668548	77.318071	71.201918	86.084517	0.001274977	-0.20742294	0.61157586	-0.040549357
ENSG00000142082	SIRT3	8.012985	7.492966	8.649395	9.551076	8.53347	9.586735	8.075067	7.606812	7.905567	0.051112108	0.218522926	0.769672615	-0.04053489
ENSG00000101220	C20orf27	19.793647	21.729959	23.649149	17.325047	15.277188	18.666512	17.551587	18.104062	19.921793	0.003960094	-0.253730939	0.699705814	-0.040533209
ENSG00000126746	ZNF384	30.66973	29.64474	31.777464	29.478143	29.224206	28.141379	27.321532	26.248282	29.497711	0.452665793	0.061763318	0.637352764	-0.040514959
ENSG00000171227	TMEM37	29.195034	25.058831	24.226146	17.019585	21.497346	19.825301	24.259187	21.773551	24.956864	0.027093829	-0.229426259	0.727225191	-0.04051236
NewGene_6392	--	2.190715	2.554338	2.145659	2.691591	2.773037	2.700862	2.099772	1.947964	2.043524	0.001327434	0.376587108	0.794381155	-0.040490195
ENSG00000138069	RAB1A	130.93046	109.200906	134.798367	146.287467	148.980907	165.335111	110.104464	122.020992	123.634199	2.13E-14	0.396292249	0.581916447	-0.040465689
ENSG00000138767	CNOT6L	2.291455	2.963898	2.249364	2.579116	2.37604	2.416924	2.276795	2.060958	1.698726	0.064723235	0.205870188	0.771177779	-0.040444443
ENSG00000161243	FBXO27	1.236565	1.27884	1.120444	1.68676	0.929935	1.018168	1.074732	1.121012	0.891149	0.689808568	0.151102891	0.90840876	-0.040423362
NewGene_3402	--	4.637673	4.723322	4.451035	4.517972	4.120902	4.10402	4.179105	4.154335	4.039937	0.762509547	0.055462587	0.810676022	-0.040378292
ENSG00000175224	ATG13	46.518294	46.369968	47.674027	54.092598	52.488118	56.495664	46.775848	41.139732	48.13291	2.19E-09	0.294017027	0.558782034	-0.040374833
ENSG00000246705	H2AFJ	28.660878	25.659716	22.961847	18.980443	23.060277	21.921897	26.017725	23.005967	26.602562	0.288015339	-0.154371709	0.777343598	-0.040363961
ENSG00000172775	FAM192A	24.717154	25.033169	23.909483	26.699199	36.618549	22.606717	24.885664	19.564989	20.841473	0.444130177	0.066397953	0.662661962	-0.04036129
ENSG00000109805	NCAPG	25.584323	27.61603	27.118064	20.952812	18.695849	19.5768	25.636258	23.141142	25.704163	0.00000484	-0.283938336	0.603756314	-0.040351207
ENSG00000240682	ISY1	11.142641	14.970013	11.125068	13.227465	14.569423	14.831511	10.808493	9.358213	13.292123	0.00000989	0.341839286	0.690061263	-0.040304704
ENSG00000198000	NOL8	14.414823	13.613522	14.409485	13.274447	11.3679	12.3685	12.293243	13.46724	13.748272	0.500269606	-0.062554478	0.670028981	-0.039951112
ENSG00000124006	OBSL1	31.597816	26.564941	21.570965	20.411391	22.58035	23.390546	24.423811	26.317565	30.259071	0.24263992	-0.116238362	0.742076131	-0.039851938
ENSG00000152240	HAUS1	21.991915	21.354731	20.20821	17.215054	19.220676	20.463932	19.580669	19.609261	19.080149	0.926677678	-0.015251468	0.74494178	-0.03963606
ENSG00000126698	DNAJC8	102.972453	99.337542	102.188008	87.515049	85.211787	85.145348	95.461151	91.175693	90.928976	0.11670051	-0.091881482	0.598034083	-0.039615374
ENSG00000161682	FAM171A2	7.500186	6.103611	5.065638	3.862369	3.671491	4.016901	5.565869	5.427339	6.359174	0.000128475	-0.544157738	0.816365969	-0.039600525
ENSG00000170471	RALGAPB	9.586479	9.214813	9.788239	8.993183	8.555851	8.947739	8.781472	7.814183	9.808617	0.797804134	0.022846009	0.661431498	-0.039578652
ENSG00000151718	WWC2	6.735838	6.987499	7.649323	6.056124	5.380419	5.796348	7.267161	5.895952	7.308898	0.016024681	-0.185658466	0.67926421	-0.039572649
ENSG00000102547	CAB39L	1.562869	1.608628	1.36335	1.279778	1.113482	1.025713	1.28811	1.454264	1.399719	0.235079956	-0.291371537	0.878867248	-0.039563235
ENSG00000136463	TACO1	20.042339	19.399406	18.646317	20.181736	21.280872	21.645658	19.427671	17.926422	15.521746	0.002557115	0.267475959	0.771177779	-0.039518567
ENSG00000076003	MCM6	47.362057	49.939514	47.98365	45.170582	42.928944	43.985283	42.698292	44.617161	45.185055	0.902378968	0.009853246	0.5949541	-0.039489494
ENSG00000148429	USP6NL	9.018497	9.078543	9.205609	7.804673	8.064909	7.795783	8.969068	7.84383	9.261475	0.356668486	-0.073241777	0.633724868	-0.039450569
ENSG00000170439	METTL7B	18.598665	19.250902	21.827815	10.512244	9.921905	12.528647	17.205641	17.668852	19.864718	1.91E-10	-0.677961952	0.748536357	-0.039436927
ENSG00000138496	PARP9	3.911933	3.547937	2.897073	50.812723	50.138753	48.57249	3.197548	3.580032	3.18626	0	3.956012266	0.81284516	-0.039063221
ENSG00000102575	ACP5	8.653838	7.398221	6.613765	12.545413	14.311807	15.514163	6.536695	7.455908	6.835532	4.18E-15	0.988931681	0.831722684	-0.038962311
ENSG00000170464	DNAJC18	0.67957	0.567338	0.538647	0.347279	0.477122	0.405475	0.691337	0.52471	0.486807	0.24089193	-0.353910467	0.902212016	-0.03893843
ENSG00000144134	RABL2A	2.353093	3.259673	2.882644	2.422966	2.392322	2.153882	2.300585	1.832752	2.986403	0.755318484	-0.085767373	0.887143556	-0.03885904
ENSG00000116918	TSNAX	22.298326	26.268805	26.403915	24.496698	21.3895	24.191639	22.240369	22.291912	23.964556	0.657702383	0.050199651	0.703357495	-0.038809743
ENSG00000027697	IFNGR1	29.155101	32.477069	37.035657	28.68335	27.691589	27.280474	31.685243	27.55394	32.09395	0.349916431	-0.087945337	0.709496735	-0.038793658
ENSG00000128928	IVD	18.072024	19.653024	17.03485	17.669084	16.43261	17.615756	17.816367	17.016328	17.873575	0.732050855	0.03504395	0.668457283	-0.03873165
ENSG00000158006	PAFAH2	2.917404	3.044436	3.054495	2.428699	2.292467	2.066669	2.702235	2.674497	2.848044	0.065268976	-0.272719506	0.808059881	-0.038716714
ENSG00000187742	SECISBP2	18.09474	17.495122	16.517782	16.060064	16.312131	15.494004	16.124418	15.48144	16.048063	0.948978759	0.007468517	0.668489211	-0.038703493
ENSG00000127838	PNKD	33.737207	31.740771	30.327906	26.077972	27.106504	24.916378	32.9684	30.346518	31.650286	0.002075677	-0.237973569	0.692234799	-0.038689784
ENSG00000057608	GDI2	98.263518	100.753793	98.066986	104.149482	103.872995	106.592603	91.751093	91.418902	88.493329	0.00000663	0.218521334	0.595027153	-0.038689775
ENSG00000165733	BMS1	8.548558	8.723033	8.691348	8.292245	7.428778	7.727913	7.452249	7.830114	8.428571	0.999202724	-0.000186038	0.657919369	-0.038631933
NewGene_2563	--	1.566101	1.968369	1.728695	1.61858	1.479293	1.351799	1.656343	1.535728	1.838923	0.393465517	-0.179351505	0.852465224	-0.038442306
ENSG00000047597	XK	0.560753	0.642736	0.616744	0.743905	0.615307	0.722229	0.61973	0.485682	0.555629	0.159027	0.344143466	0.89403938	-0.038345167
ENSG00000111266	DUSP16	18.66801	17.107802	18.212477	14.520223	15.097858	14.912935	17.863218	15.916234	15.604798	0.081041357	-0.124542499	0.681156965	-0.0383173
ENSG00000120805	ARL1	21.420748	22.068507	23.126538	20.991159	20.122812	20.598641	20.743856	20.338679	20.355719	0.783086851	0.025486863	0.654887623	-0.038246805
ENSG00000118600	RXYLT1	8.563322	11.155256	10.066185	6.748542	6.60965	6.501743	9.056366	8.407413	9.887889	0.001968115	-0.395178967	0.802085198	-0.03822104
ENSG00000149016	TUT1	4.665425	4.048557	5.33402	4.289571	4.44934	4.225735	4.072391	4.413878	4.404747	0.956183417	0.011308402	0.81397511	-0.03809231
ENSG00000154162	CDH12	6.535164	7.130124	7.838298	4.158679	4.020642	4.201865	6.590769	6.053576	7.007656	1.28E-11	-0.646669314	0.753316498	-0.037991768
ENSG00000170421	KRT8	735.855566	746.817013	754.697935	493.558782	480.198748	501.091408	669.034641	672.620659	695.521692	7.68E-26	-0.434771885	0.535913374	-0.037803376
ENSG00000136450	SRSF1	115.917541	122.511015	125.764977	118.226846	110.726228	114.958569	110.261648	105.514192	112.079335	0.25966645	0.071547183	0.594981342	-0.037754053
ENSG00000126705	AHDC1	2.554358	2.274078	2.126041	1.562665	1.814998	1.695302	2.429328	1.767422	2.032815	0.052480202	-0.270803905	0.831421908	-0.037726122
ENSG00000120837	NFYB	16.275208	16.568221	16.490504	15.124417	15.117578	14.142373	13.878341	14.181461	15.250007	0.797804134	-0.028099244	0.697049785	-0.037662923
ENSG00000219481	NBPF1	2.015049	1.75999	1.95806	1.745287	2.028462	1.423825	1.647773	1.68344	1.899289	0.986348802	0.004071987	0.818325223	-0.03765994
ENSG00000007545	CRAMP1	4.711268	4.626593	5.173219	4.591336	4.441481	4.353839	4.501268	3.901165	4.869015	0.86810552	0.020329864	0.738673283	-0.037648661
ENSG00000164219	PGGT1B	2.382496	2.807088	2.98675	2.733898	2.71981	2.87572	2.620049	2.355268	2.440263	0.1311569	0.179220082	0.785764397	-0.037636707
ENSG00000001036	FUCA2	104.341103	105.085155	109.407535	85.060245	80.472177	80.647129	98.056049	95.682012	97.999054	0.00000869	-0.226002315	0.588346602	-0.037607533
ENSG00000107829	FBXW4	9.585414	9.704878	8.376897	8.537683	8.329248	8.932012	8.134192	8.220409	8.944667	0.737599529	0.046942893	0.767715478	-0.037453993
ENSG00000067248	DHX29	5.793806	6.164777	5.619389	6.772521	6.611389	6.66639	5.597637	5.079656	5.391837	0.0000715	0.337211952	0.747057125	-0.037334303
ENSG00000156650	KAT6B	2.235311	2.965722	2.623713	2.273358	2.225728	2.309485	2.316258	1.958997	2.508184	0.724056896	0.050813668	0.785334253	-0.03727306
ENSG00000148154	UGCG	4.04809	4.31149	4.773341	4.80341	4.905062	5.18105	4.654509	3.79943	3.520032	0.002546175	0.329894233	0.821901445	-0.037245182
ENSG00000110917	MLEC	31.918396	31.172649	30.096836	43.911775	42.79021	42.579231	28.334972	27.545794	29.506563	3.52E-38	0.617499997	0.593143935	-0.037170489
NewGene_4364	--	0.609792	1.086132	0.96537	0.718544	0.562378	0.640115	0.787205	0.81839	0.728689	0.591273441	-0.29162208	0.940860894	-0.0371468
ENSG00000115504	EHBP1	21.278172	23.373315	21.500345	23.274935	21.921123	22.15691	20.780494	18.893476	22.932257	0.003143373	0.173441305	0.645905445	-0.036972454
ENSG00000149582	TMEM25	1.471572	1.113011	1.108491	0.890604	1.002233	0.767097	1.062468	0.991869	1.25521	0.499991339	-0.226120397	0.906310374	-0.03686506
ENSG00000005486	RHBDD2	43.384354	35.697525	37.985774	28.709123	29.462778	31.436088	33.670454	35.117942	39.47349	0.000756714	-0.275269536	0.74111276	-0.036593902
ENSG00000117632	STMN1	190.865981	191.946201	191.370899	141.336901	133.847899	143.797106	180.007539	177.028332	164.244329	1.43E-09	-0.29410124	0.660985868	-0.036537304

Gene ID	Gene name	Ctrl-1 FPKM	Ctrl-2 FPKM	Ctrl-3 FPKM	IFNγ-1 FPKM	IFNγ-2 FPKM	IFNγ-3 FPKM	Sora-1 FPKM	Sora-2 FPKM	Sora-3 FPKM	Ctrl vs IFNγ DESeq2 FDR	Ctrl vs IFNγ DESeq2 log2FC	Ctrl vs Sora DESeq2 FDR	Ctrl vs Sora DESeq2 log2FC
NewGene 788	--	1.402042	1.129027	1.224516	1.126975	1.184017	1.179981	1.159834	1.005743	1.331055	0.902161713	0.047550188	0.910318225	-0.036507488
ENSG00000135655	USP15	9.296996	9.599699	9.185434	14.584098	12.32578	13.013223	9.799855	10.814769	11.131555	2.75E-16	0.518179575	0.69378395	-0.036414737
ENSG00000163251	FZD5	24.366861	24.991283	26.305883	25.684381	25.155399	26.581623	23.277803	21.900949	24.049568	0.001933662	0.180786111	0.621529898	-0.036301409
ENSG00000113460	BRIX1	37.693954	39.113537	44.911991	37.759586	36.118458	35.907898	38.064602	37.767502	35.333622	0.989948563	-0.001618842	0.738673283	-0.036098954
ENSG00000260007	AC107871.1	1.368634	1.284436	1.325516	1.821138	1.384732	1.287137	1.343389	1.206248	1.187462	0.168765047	0.317688824	0.884988884	-0.036084983
ENSG00000089234	BRAP	8.815525	9.199537	9.761468	8.738648	8.615331	8.524527	8.780109	8.059888	8.612326	0.705902056	0.04179332	0.731504451	-0.03598924
ENSG00000197312	DDI2	6.047039	6.715236	6.658666	9.455986	9.165002	8.915742	6.303785	5.18618	6.295563	4.49E-26	0.650902292	0.728112428	-0.035930519
ENSG00000171161	ZNF672	24.428197	25.132403	22.103153	17.981692	19.194589	20.709156	20.240173	20.850179	24.252219	0.083083838	-0.153222446	0.723360024	-0.035544157
ENSG00000151834	GABRA2	17.109875	19.378802	20.94922	21.729978	18.81354	20.145902	17.210268	17.28573	19.490722	0.008705599	0.239025024	0.752470807	-0.035518275
ENSG00000115084	SLC35F5	11.11056	11.581991	12.167328	10.998745	10.639683	10.426421	11.101713	9.878377	11.470565	0.691041153	0.038986022	0.707601438	-0.035476887
ENSG00000033867	SLC4A7	10.934913	12.25016	13.462048	9.42012	9.398208	8.818685	10.756295	10.3948	12.310508	0.009269875	-0.25134833	0.769672615	-0.03547541
ENSG00000284820	AC068946.2	4.46567	3.489859	2.688056	5.346521	3.380177	4.934633	3.933208	2.432415	3.409293	0.1443168	0.503736226	0.92640073	-0.035410632
ENSG00000158715	SLC45A3	2.157029	1.795975	1.84322	0.809503	0.615492	0.807994	1.749068	1.690368	1.868796	4.5E-10	-1.223533583	0.864540586	-0.035386215
ENSG00000103978	TMEM87A	27.993465	29.121728	27.897709	24.327081	24.251151	27.270125	25.806703	24.097842	26.870985	0.184059247	-0.09988918	0.66790766	-0.03537001
ENSG00000157895	C12orf43	8.986043	8.203523	9.748295	10.099313	8.729483	10.155694	8.807412	7.709324	9.312955	0.007583736	0.252570816	0.792598574	-0.035338881
ENSG00000118496	FBXO30	2.905746	3.377975	3.375366	3.550852	3.255773	3.198911	3.111846	2.660859	3.073271	0.049507211	0.197744809	0.775997101	-0.0352554
ENSG00000148019	CEP78	7.54657	5.766145	6.046581	6.741826	6.081104	7.912631	7.416922	5.941229	6.553507	0.150081465	0.124972092	0.722184083	-0.035175024
ENSG00000166508	MCM7	158.463798	153.810439	151.990988	131.617156	123.135122	131.27251	136.219141	143.956083	145.316106	0.029870858	-0.116526117	0.621046934	-0.035105727
ENSG00000182670	TTC3	26.058148	23.980513	25.578345	23.889278	20.662514	19.962289	21.424313	20.041601	26.953774	0.007315115	-0.159001975	0.623203992	-0.03498515
ENSG00000167680	SEMA6B	15.05114	15.031319	14.138624	6.422194	7.860378	7.456898	12.024662	13.052081	15.460911	9.81E-25	-0.875190816	0.750514622	-0.034975207
ENSG00000156671	SAMD8	1.395294	1.321962	1.676131	1.437418	1.780237	1.319561	1.249686	1.240689	1.602422	0.46908358	0.143418801	0.869068091	-0.034964819
NewGene 4024	--	1.541419	1.865946	2.087443	2.097976	2.133414	2.0925	1.575083	1.74137	1.699537	0.109733641	0.349800891	0.894873362	-0.034889474
ENSG00000180287	PLD5	7.677643	7.398249	6.292347	3.901935	4.314058	2.982439	7.275373	6.468414	6.968967	5.99E-24	-0.828707962	0.729823374	-0.034869686
ENSG00000109466	KLHL2	4.577362	5.212342	5.561865	4.20724	4.182095	4.288526	4.611294	4.516102	4.782846	0.489818012	-0.103440401	0.812705602	-0.034756065
ENSG00000103995	CEP152	3.285751	3.731968	3.796515	3.096409	2.773942	2.860254	3.432435	3.244004	3.777692	0.158208239	-0.169162512	0.789449911	-0.034688312
ENSG00000167780	SOAT2	2.560999	1.867147	1.352157	1.730382	1.751855	1.529572	2.033727	1.941025	1.261505	0.891516785	-0.051899641	0.918515802	-0.034685501
ENSG00000198826	ARHGAP11A	13.249031	15.780567	15.20706	13.216484	10.715174	11.296288	13.785599	12.386298	15.559161	0.022728151	-0.208060266	0.751143048	-0.034571588
ENSG00000182473	EXOC7	32.770468	30.748587	27.645309	29.447039	27.155481	27.046708	26.346453	27.588277	29.727992	0.994104573	0.000952157	0.722184083	-0.034554134
ENSG00000146826	C7orf43	6.925279	6.61365	6.31915	6.016627	5.536037	5.213781	5.587036	6.271106	6.481587	0.520389631	-0.098006161	0.811107842	-0.034522577
ENSG00000082068	WDR70	10.218209	11.508243	11.135111	9.480364	8.853638	8.940575	9.953614	10.403508	9.667071	0.228292056	-0.121246674	0.768536497	-0.034501703
ENSG00000103855	CD276	27.462128	25.92587	23.867493	25.553389	23.962822	25.489028	24.549376	25.025286	26.711272	0.889945453	0.01559153	0.707601438	-0.034417407
ENSG00000157600	TMEM164	6.805851	6.618363	7.357445	6.426182	6.956789	6.855302	6.592661	6.158288	6.490403	0.311893506	0.100683709	0.733704469	-0.034238117
ENSG00000156642	NPTN	35.937045	39.279603	38.558647	39.881493	38.285426	36.919056	34.784602	34.032804	34.891602	0.013251476	0.159981609	0.675431542	-0.034161493
ENSG00000184162	NR2C2AP	8.817067	9.173362	8.713499	6.817316	5.861574	6.880382	7.548301	8.517288	7.938481	0.026446569	-0.323369083	0.840241952	-0.034130608
ENSG00000142544	CTU1	2.880013	2.527002	2.40776	1.762116	2.369095	2.158588	2.251866	2.433273	2.484016	0.501970262	-0.166695716	0.879355906	-0.033990761
ENSG00000168734	PKIG	6.395195	5.880932	4.354565	5.467346	5.109405	5.324273	5.595528	4.801082	4.739266	0.746869932	0.07965126	0.882838812	-0.033866462
ENSG00000086696	HSD17B2	7.111548	7.991383	6.63235	4.385207	6.125109	5.92902	6.285542	6.2948	7.691746	0.171227886	-0.23068196	0.846376928	-0.033799339
ENSG00000100941	PNN	78.719202	88.10903	92.318211	86.582146	81.616482	79.529327	79.106278	77.139399	82.382259	0.2947605	0.081011972	0.68651323	-0.033749739
ENSG00000121152	NCAPH	25.481267	24.596267	22.818977	20.651215	16.666314	17.325932	22.190789	19.15	23.684034	0.0000507	-0.310855079	0.721007612	-0.03369737
ENSG00000104763	ASAH1	82.76195	88.093227	89.085343	102.491791	100.534115	100.150008	77.695228	81.724281	75.900268	8.35E-13	0.347211809	0.660591165	-0.033651502
ENSG00000171314	PGAM1	123.491539	123.609062	114.857483	102.090858	101.099251	113.254745	97.614738	112.350967	121.816162	0.586946295	-0.046074252	0.728850741	-0.033626285
ENSG00000173156	RHOD	34.96091	32.656529	30.205684	20.834219	19.654074	19.525167	29.477568	27.760863	32.662065	8.59E-11	-0.559566205	0.765340916	-0.033553063
ENSG00000131115	ZNF227	6.953184	7.327512	7.793252	6.398227	7.317251	7.246204	7.278316	6.825839	6.901863	0.520250114	0.086035578	0.786234446	-0.03352284
ENSG00000147041	SYTL5	0.581015	0.428696	0.507408	0.17378	0.171487	0.207201	0.394288	0.456281	0.539752	0.0000584	-1.294662962	0.920950996	-0.033505553
ENSG00000165501	LRR1	8.348404	8.846787	7.916766	7.841412	9.21382	8.60996	8.215403	8.05802	7.949188	0.323337668	0.143720938	0.828094926	-0.033437939
ENSG00000116663	FBXO6	1.657466	2.046844	2.047118	14.591587	14.931027	14.654459	1.557056	2.133526	1.53706	4.37E-130	3.078024066	0.916826733	-0.033423027
ENSG00000135341	MAP3K7	25.709711	26.661552	28.036318	23.286278	22.991434	23.036003	23.397897	24.08983	26.769338	0.349173674	-0.066042946	0.685847365	-0.033422814
ENSG00000198756	COLGALT2	0.936335	0.937994	1.254309	0.937122	0.854429	0.596596	0.816947	0.844229	0.956498	0.336879028	-0.243029299	0.890499304	-0.03336124
ENSG00000146109	ABT1	33.065838	29.754478	28.502041	27.865469	29.212814	29.855131	26.920277	27.222569	29.605721	0.404054563	0.076490835	0.722952357	-0.03332804
ENSG00000165959	CLMN	1.927684	1.833277	2.14357	1.166245	1.446858	1.434029	1.556852	1.965267	2.051245	0.003510303	-0.353273226	0.816335807	-0.033167866
ENSG00000165526	RPUSD4	14.31642	14.993288	15.284866	11.531745	11.994656	11.427295	13.50572	13.369264	13.984628	0.018035915	-0.203785489	0.741167578	-0.033149056
ENSG00000112701	SENP6	15.777413	17.355215	17.840002	17.257552	16.56358	17.166756	15.983364	14.941948	16.862561	0.048663624	0.143278896	0.71749733	-0.033144533
ENSG00000156030	ELMSAN1	7.181757	6.186289	6.805658	6.487823	5.995598	6.442455	6.26245	5.154338	6.523628	0.341824435	0.08484555	0.771667593	-0.032868586
ENSG00000256525	POLG2	4.800333	5.990751	5.219309	5.781853	5.056262	5.718206	4.476202	4.837124	5.606136	0.294380059	0.184009034	0.867823719	-0.032828045
ENSG00000057294	PKP2	13.06856	13.585282	13.068015	11.713215	11.646477	11.397047	12.071719	11.385407	13.029716	0.655422297	-0.041740159	0.717435848	-0.032711831
ENSG00000126970	ZC4H2	4.68552	3.916849	3.640457	4.321899	4.379009	4.793122	3.482566	3.81641	4.056555	0.08608591	0.266580081	0.858135275	-0.032675966
ENSG00000099284	H2AFY2	39.003056	35.777882	32.8333	30.730692	27.456266	29.961557	33.34423	31.957443	33.260223	0.1146425	-0.140687385	0.740617361	-0.032655431
ENSG00000164543	STK17A	41.9295	43.061813	49.926791	23.635345	26.714237	24.281412	42.441068	37.686908	43.77355	7.55E-21	-0.711032264	0.739316607	-0.032557026
ENSG00000183726	TMEM50A	12.782093	13.011807	14.120946	17.770714	16.982157	18.637636	11.845123	11.962186	12.787023	4.16E-14	0.567874199	0.760447391	-0.032412693
ENSG00000111319	SCNN1A	6.532909	6.681349	6.309801	7.82617	7.758707	8.553019	6.016987	5.410475	6.309019	8.42E-08	0.46074898	0.791809524	-0.032322079
ENSG00000168661	ZNF30	1.011007	1.297772	1.034835	0.965695	0.988658	0.896578	0.834362	1.098976	1.130535	0.784554081	-0.095404596	0.918533019	-0.032221022
ENSG00000080986	NDC80	15.057863	16.542851	16.312832	12.706783	10.513966	11.987431	14.770278	14.156605	13.84334	0.001299972	-0.315481662	0.777100147	-0.03218483
ENSG00000197261	C6orf141	1.324885	1.448288	1.898523	1.458174	1.230421	1.261076	1.347686	1.327223	1.559789	0.802747044	-0.095818972	0.922417813	-0.032098815
ENSG00000186522	10-Sep	61.306952	59.171559	63.452167	42.100367	41.745663	40.478574	50.895242	51.382509	60.12723	1.6E-10	-0.368121536	0.706803284	-0.032093902
ENSG00000101019	UQCC1	3.553162	4.115136	3.964734	3.668926	4.090144	3.598858	3.877456	3.882255	3.705135	0.638749338	0.084998429	0.853159272	-0.032081701
ENSG00000074054	CLASP1	11.885173	11.432856	11.49518	10.161672	10.475248	10.538317	10.678124	10.002922	11.290807	0.65686734	-0.036563122	0.686363843	-0.031791618
ENSG00000102921	N4BP1	4.596869	4.507363	4.868183	5.096154	4.906296	4.977376	4.25524	3.754224	4.858467	0.00234125	0.247684538	0.790593362	-0.031734341
ENSG00000166226	CCT2	170.054382	158.463352	161.767411	125.974877	130.966029	132.021253	154.342401	152.161418	151.244608	0.000308392	-0.195041395	0.660560204	-0.031662183
ENSG00000169255	B3GALNT1	6.199068	6.40941	6.445203	7.160418	6.372143	7.164197	6.371398	5.169516	5.880642	0.013792767	0.255149652	0.817806294	-0.031655126
ENSG00000127947	PTPN12	56.061484	57.222277	63.816501	40.253884	41.965106	39.778601	51.258551	49.79232	60.879529	7.16E-11	-0.403875427	0.743243948	-0.031308147
NewGene 3335	--	5.84645	5.581012	5.587665	6.125606	5.37364	5.776584	5.445156	4.736908	5.504049	0.367309808	0.095463069	0.790456343	-0.031263919
ENSG00000173889	PHC3	3.681299	4.048468	4.573643	6.238933	5.935611	5.436255	3.671549	3.418961	3.865458	1.97E-17	0.763848567	0.805865213	-0.031232268
ENSG00000166822	TMEM170A	4.589673	6.001958	5.627744	5.110616	4.7426	5.029191	5.720818	4.623737	5.45239	0.86899113	0.02671491	0.835719911	-0.031102684
ENSG00000132388	UBE2G1	48.494029	46.040576	46.981136	45.274164	43.908464	44.254779	45.533046	43.680835	54.669614	0.073587782	0.108478291	0.682558864	-0.030994295

Gene ID	Gene name	Ctrl-1 FPKM	Ctrl-2 FPKM	Ctrl-3 FPKM	IFNγ-1 FPKM	IFNγ-2 FPKM	IFNγ-3 FPKM	Sora-1 FPKM	Sora-2 FPKM	Sora-3 FPKM	Ctrl vs IFNγ DESeq2 FDR	Ctrl vs IFNγ DESeq2 log2FC	Ctrl vs Sora DESeq2 FDR	Ctrl vs Sora DESeq2 log2FC
ENSG00000203772	SPRN	3.042784	3.256495	3.253244	2.914625	2.629491	2.805708	2.947018	2.795435	3.024765	0.810146693	-0.04594452	0.852830205	-0.030777363
ENSG00000135446	CDK4	199.727321	199.844672	188.809428	175.184051	172.137013	160.763865	154.046092	169.097078	167.445263	0.007304094	-0.139984191	0.709167224	-0.030620412
ENSG00000094804	CDC6	27.635751	26.524727	28.918429	24.083437	25.787394	29.826778	18.633386	27.029016	29.783019	0.256274973	0.08259147	0.710932447	-0.030577897
ENSG00000064726	BTBD1	29.132068	31.45258	33.633547	32.998377	33.233747	31.576646	30.479345	27.178545	29.668923	0.002854419	0.205381168	0.738233923	-0.030417271
ENSG00000167657	DAPK3	31.263999	26.734705	27.828932	23.539845	24.315741	25.448722	24.048679	31.785346	33.374781	0.586859461	-0.05767361	0.793296796	-0.030039204
ENSG00000076513	ANKRD13A	14.998722	15.71781	15.043792	16.827434	19.257847	17.9	13.323955	14.266241	13.801768	6.2E-09	0.412384733	0.749187294	-0.030033049
ENSG00000080189	SLC35C2	10.737358	8.69587	10.419348	8.500543	8.934738	7.953991	9.74304	11.347265	10.019228	0.013458864	-0.211259516	0.768864178	-0.030031791
ENSG00000188735	TMEM120B	4.469218	3.895115	4.913504	4.263986	3.645641	3.103525	4.037072	3.389033	4.591322	0.999571371	0.0000884	0.831722684	-0.029999098
ENSG00000163029	SMC6	9.956728	10.760785	11.824555	11.066216	9.959295	9.645543	10.249466	9.141766	10.529886	0.607261373	0.055092311	0.770475007	-0.029987075
ENSG00000049769	PPP1R3F	0.904539	1.126173	1.31882	0.633117	1.055315	1.096102	1.006661	1.07641	0.8266	0.800575578	-0.094503194	0.921437314	-0.029973759
NewGene_6060	--	11.633557	12.938193	12.998832	14.055949	13.417955	12.488604	12.22784	11.833935	9.559215	0.006803997	0.259154879	0.841097048	-0.029971007
ENSG00000135597	REPS1	13.461452	13.591206	14.581199	13.958672	11.454359	12.164218	13.154769	11.776599	11.677172	0.774197861	0.029706925	0.751995229	-0.029926945
ENSG00000175764	TTLL11	0.815124	0.667758	0.982469	1.290383	1.116321	0.717221	0.763034	0.682254	1.100618	0.076178887	0.558415881	0.937921593	-0.029875225
ENSG00000102189	EEA1	4.644132	5.891975	6.288548	5.685949	5.1304	5.365076	5.182282	4.819564	5.539875	0.475430489	0.0907887	0.820224678	-0.02981025
ENSG00000135723	FHOD1	1.076865	1.108108	1.154211	0.65846	0.656235	0.684404	0.901524	1.042192	1.138421	0.005397611	-0.603353328	0.904850698	-0.029671121
ENSG00000113658	SMAD5	17.900266	18.737834	19.234259	16.743381	15.239592	15.425366	18.691021	15.843916	18.039621	0.104175289	-0.1075759	0.743214906	-0.029661421
ENSG00000188687	SLC4A5	0.14484	0.180106	0.224058	0.173862	0.130488	0.161438	0.149173	0.121223	0.238698	0.699346345	-0.206482312	0.954097361	-0.029604784
ENSG00000055483	USP36	9.948532	10.358631	11.083243	10.402037	8.66995	9.773808	8.884153	7.932645	9.32961	0.76640527	0.033342332	0.769672615	-0.029484948
ENSG00000197321	SVIL	3.664692	3.888684	3.612377	4.15215	4.765309	4.17986	3.297187	3.27911	3.335713	0.00000985	0.377731072	0.79984277	-0.029453446
ENSG00000173457	PPP1R14B	139.08879	131.086224	124.684719	107.507803	107.849282	116.470975	116.468302	124.267962	121.79181	0.176564114	-0.104486274	0.743689892	-0.029277371
ENSG00000228716	DHFR	17.617072	19.91829	19.770001	17.615336	17.503219	17.595608	17.639676	18.454697	16.468042	0.848649189	0.01982572	0.784516217	-0.029206881
ENSG00000168765	GSTM4	6.463139	5.608691	6.491297	4.359259	5.850394	5.462088	6.412559	4.976646	5.226195	0.78319883	-0.059687514	0.876039541	-0.029091162
ENSG00000171492	LRRC8D	25.51543	26.873458	27.029932	26.983624	23.106759	23.294441	23.955626	22.890398	26.268478	0.520358841	-0.050039541	0.719864317	-0.029067657
ENSG00000168301	KCTD6	7.138274	6.91452	6.790075	11.960869	10.888899	10.837097	7.140084	6.19396	6.737269	4.85E-16	0.801928914	0.855805297	-0.029062047
ENSG00000125249	RAP2A	13.674173	13.230436	13.327971	13.720712	13.357815	12.333571	12.331174	11.431802	13.326516	0.121785572	0.116418332	0.738699798	-0.029048595
ENSG00000138443	ABI2	5.640054	5.631625	6.920999	5.317802	5.028453	5.739401	8.086545	5.275741	8.49945	0.58756523	0.052292693	0.781700151	-0.028972404
ENSG00000080815	PSEN1	9.526142	9.168946	10.207914	8.352165	8.67808	7.868999	8.725655	9.066521	10.111635	0.576287792	-0.057380946	0.7755936	-0.028971638
ENSG00000006459	KDM7A	2.752466	2.514859	2.582355	1.874712	2.064007	1.920519	2.540367	2.1218	2.60813	0.008372304	-0.275377927	0.820211267	-0.028909451
ENSG00000090971	NAT14	12.670834	13.352491	12.429249	9.524618	11.078519	10.621124	11.454813	11.869635	12.603874	0.2953168	-0.139303758	0.825284258	-0.028866927
ENSG00000162194	LBHD1	6.430203	5.600919	7.562446	9.351052	9.668547	9.189641	5.100487	6.677244	6.046968	4.69E-10	0.686176408	0.875402461	-0.028817315
ENSG00000198799	LRIG2	1.082268	1.12543	1.456622	1.155517	1.166445	0.99268	1.323293	1.022435	1.019468	0.994074892	0.001887567	0.880844733	-0.028788171
ENSG00000158796	DEDD	18.300969	16.975325	18.274597	18.024462	17.223257	16.446388	16.481034	17.004182	16.032759	0.332972217	0.091911051	0.790393539	-0.028723357
ENSG00000132549	VPS13B	3.729957	3.512808	3.4396	3.641172	3.302608	3.452701	3.232752	3.209251	3.490899	0.208620939	0.108042128	0.767978063	-0.02866852
NewGene_1604	--	63.545597	58.79071	50.944561	56.434689	47.997688	57.152153	46.816238	52.12965	60.601303	0.721281392	0.045945437	0.821213897	-0.028649431
ENSG00000103264	FBXO31	4.570494	4.78047	4.836369	6.980716	5.610276	6.114154	4.592139	4.134748	4.639896	5.73E-10	0.536109993	0.800790965	-0.028616966
ENSG00000104047	DTWD1	2.980466	2.878958	2.896144	3.901922	3.510105	3.460771	3.582454	2.89592	5.837047	0.854624311	0.032143422	0.854855373	-0.028376653
ENSG00000284526	AC015802.6	7.609624	9.124366	10.175768	9.067445	7.816122	8.152061	8.094712	8.124306	8.517017	0.763805865	0.042831497	0.822688248	-0.028355277
ENSG00000239306	RBM14	28.319895	30.181941	28.601655	26.762233	27.115354	27.83554	26.471668	25.94973	28.142171	0.491926916	0.056605418	0.736442176	-0.02829364
ENSG00000213246	SUPT4H1	55.239198	55.923686	52.269514	48.160719	51.599163	52.122301	48.980799	51.307814	49.209366	0.699470498	0.037614822	0.757373564	-0.028052735
ENSG00000179134	SAMD4B	17.260725	16.408794	16.443828	14.346641	13.273332	14.132512	15.239724	14.948609	17.024559	0.192874442	-0.106036603	0.766119487	-0.02795793
ENSG00000132031	MATN3	2.319185	3.094496	3.146317	0.897954	0.829483	0.616434	2.718986	2.088621	3.095301	2.09E-16	-1.719697756	0.905203955	-0.027645369
ENSG00000125037	EMC3	28.893601	27.876846	26.82363	24.387256	25.160939	24.916807	26.057389	25.966464	24.663952	0.960339827	-0.005288248	0.766052664	-0.027604417
ENSG00000177225	GATD1	9.776772	10.607285	9.662433	8.470061	7.621594	7.58693	8.93517	9.683759	9.538532	0.012444454	-0.213112038	0.788414179	-0.027352526
ENSG00000037897	METTL1	9.067334	10.008185	10.228593	8.579265	6.196438	6.702846	9.712711	8.956515	8.26285	0.027071562	-0.288989506	0.848403877	-0.027249416
ENSG00000166016	ABTB2	2.74436	2.402575	2.520723	6.2898	5.131773	5.884877	2.523607	1.946079	2.610444	5.2E-39	1.318838034	0.874214	-0.027205368
ENSG00000149043	SYT8	1.234111	1.346868	0.651154	0.483322	0.444013	0.451559	1.062486	0.904084	0.936773	0.022974267	-0.982715929	0.952603021	-0.027114905
ENSG00000106355	LSM5	38.748699	45.176719	44.294902	41.809929	39.034435	44.012839	40.025026	38.555501	35.574978	0.240425452	0.101083066	0.799415769	-0.027053014
ENSG00000146918	NCAPG2	26.568157	24.574951	25.750527	21.41294	20.75681	19.743952	25.233463	22.374792	25.606396	0.009894372	-0.17551185	0.737020796	-0.026924947
ENSG00000040933	INPP4A	2.053042	1.910381	1.56768	2.258958	1.578552	1.625495	1.82626	1.384095	2.634041	0.341788236	0.13318451	0.853932034	-0.026766109
ENSG00000197299	BLM	8.290987	8.672977	8.650396	7.053194	6.125562	6.304513	7.626378	7.747504	7.422319	0.049591232	-0.179605818	0.797555837	-0.02670155
ENSG00000212864	RNF208	7.915242	8.144633	7.983841	9.323822	10.937748	9.281407	6.55281	7.13922	8.436823	0.001527605	0.445982714	0.885612975	-0.02667123
ENSG00000185379	RAD51D	3.645583	3.790999	3.994804	3.540049	3.33239	3.292005	3.352365	4.000189	3.60988	0.29137301	-0.113553214	0.820224678	-0.026653893
ENSG00000177951	BET1L	20.721698	21.045794	19.899008	26.040275	24.985022	26.023092	18.110425	17.869536	20.748724	1.47E-14	0.470934486	0.7832038	-0.026533115
ENSG00000096654	ZNF184	6.485891	6.319185	5.891526	4.584284	4.661557	4.574001	6.236685	5.426115	5.577641	0.00778376	-0.291406407	0.846065263	-0.026506123
ENSG00000052723	SIKE1	6.037749	6.485726	6.887446	6.873904	6.620813	6.585215	5.911498	5.826156	6.150573	0.025014591	0.19996048	0.812277353	-0.026473428
ENSG00000099904	ZDHHC8	1.006293	1.244505	0.755012	0.989832	1.03207	1.111633	0.917002	0.808511	1.008541	0.251667505	0.277961949	0.922931606	-0.026459083
ENSG00000116133	DHCR24	238.882844	222.825722	218.682037	223.971101	226.61932	235.364437	208.547069	202.511891	216.659141	0.025117567	0.140175076	0.732579591	-0.026395919
NewGene_239	--	0.563742	0.326844	0.539107	0.429787	0.580517	0.620955	0.499178	0.347899	0.358034	0.3296778	0.451567189	0.96128358	-0.026341156
ENSG00000142208	AKT1	33.985284	30.888224	29.866449	27.938892	28.489064	29.909434	26.685279	27.575971	34.682884	0.826685215	0.021291667	0.788414179	-0.026321493
NewGene_2479	--	1.542153	1.798604	1.726043	1.422375	1.474318	1.228813	1.534137	1.52205	1.612704	0.342625501	-0.17188759	0.884465057	-0.026157052
ENSG00000161179	YDJC	19.547293	20.723985	19.737147	18.718392	20.021954	23.320118	17.262178	17.844372	19.622533	0.08282348	0.194518392	0.818288741	-0.02606331
ENSG00000135929	CYP27A1	19.291771	17.92632	17.586214	17.133857	16.140771	17.781656	17.746986	16.449705	16.319113	0.643246801	0.050324022	0.810818487	-0.025897969
ENSG00000129084	PSMA1	106.342213	113.282361	111.037413	132.307612	126.687885	124.112328	100.215073	103.552723	100.947261	6.03E-13	0.371563583	0.742756999	-0.025836541
ENSG00000007866	TEAD3	13.6266	14.246566	13.829237	13.641683	12.976113	11.926292	12.240396	12.505127	14.007682	0.770507182	0.033081881	0.795202808	-0.025798572
ENSG00000149182	ARFGAP2	57.570619	55.60444	53.345975	54.640428	58.713493	57.769461	51.81831	49.477629	53.930304	0.013121573	0.179134699	0.768536497	-0.025693253
ENSG00000063601	MTMR1	9.267462	9.825623	10.271366	11.536466	9.923895	9.335011	10.762948	8.348507	10.401977	0.187788761	0.119843535	0.808233973	-0.02566249
ENSG00000139433	GLTP	5.428036	4.988889	5.195891	4.696866	4.16617	4.908111	4.382515	4.812871	5.276473	0.833264092	-0.038967773	0.873783187	-0.025560953
ENSG00000066427	ATXN3	1.41408	1.85487	1.441353	1.531528	1.204168	1.113296	2.033684	2.152709	1.585193	0.94404299	-0.018246291	0.90201556	-0.025540384
ENSG00000081026	MAGI3	2.727714	2.488005	2.599675	2.621675	2.594439	2.601546	2.551475	2.201141	2.439342	0.172957356	0.164072225	0.857654023	-0.025421234
ENSG00000167711	SERPINF2	90.322071	68.173868	50.900101	125.813915	121.822971	122.581903	70.410605	64.01638	59.997067	0.000369166	0.94871049	0.943620534	-0.025407826
ENSG00000149600	COMMD7	25.656331	25.382325	25.733308	24.510123	22.197371	22.120202	25.02731	21.299746	20.841646	0.735321503	0.035885888	0.822913844	-0.025297527
ENSG00000171720	HDAC3	23.966514	22.914482	23.444155	21.222074	19.649888	19.292177	20.312157	21.90581	22.410177	0.426255558	-0.074648466	0.802271485	-0.025180558
ENSG00000156928	MALSU1	15.743365	15.884686	17.63933	16.823816	17.877495	16.939947	16.793449	14.51372	14.061616	0.007696563	0.215867805	0.835348111	-0.024879244
ENSG00000149313	AASDHPPT	15.167649	16.296736	17.70578	13.97152	13.47093	13.460483	15.06444	13.865597	16.621463	0.200515024	-0.117701302	0.817781205	-0.024869129

Gene ID	Gene name	Ctrl-1 FPKM	Ctrl-2 FPKM	Ctrl-3 FPKM	IFNγ-1 FPKM	IFNγ-2 FPKM	IFNγ-3 FPKM	Sora-1 FPKM	Sora-2 FPKM	Sora-3 FPKM	Ctrl vs IFNγ DESeq2 FDR	Ctrl vs IFNγ DESeq2 log2FC	Ctrl vs Sora DESeq2 FDR	Ctrl vs Sora DESeq2 log2FC
ENSG00000163517	HDAC11	8.494688	6.961046	5.141991	5.711869	5.22416	5.653051	5.718829	5.48434	7.327061	0.459730414	-0.135061798	0.894847831	-0.024866346
ENSG00000114062	UBE3A	17.944205	15.000855	21.627721	20.661938	15.636971	16.614771	14.533075	14.008596	14.199375	0.002487198	0.193824921	0.769072389	-0.024854379
ENSG00000204574	ABCF1	109.36378	112.367719	112.353885	104.854183	101.289416	104.722573	99.931874	96.852294	111.289457	0.444001858	0.045422078	0.734620559	-0.024780338
ENSG00000125898	FAM110A	3.811855	3.36817	2.929357	2.731681	2.959833	2.771434	3.18986	3.060264	2.992585	0.755669091	-0.078970888	0.911553655	-0.024726325
ENSG00000180992	MRPL14	46.394859	44.595535	42.08942	46.205154	49.670773	53.457951	40.162109	42.657642	39.692783	0.000213608	0.314645552	0.822688248	-0.024698302
ENSG00000146904	EPHA1	7.695046	7.108411	6.469107	7.28938	7.017595	7.011116	7.095396	6.217827	6.292283	0.178439505	0.150238865	0.856598945	-0.024517798
ENSG00000065357	DGKA	1.768769	1.443438	1.690734	1.310466	1.278968	0.598124	1.321603	1.630604	1.189382	0.052054962	-0.546397469	0.927639534	-0.024484434
NewGene_2396	--	0.604168	0.523516	0.339426	0.134577	0.539805	0.561073	0.468955	0.425847	0.480848	0.856006078	-0.130066751	0.962592841	-0.024465519
ENSG00000103035	PSMD7	70.345819	75.703001	74.038617	71.224262	64.973463	66.173398	64.476481	69.612608	67.733462	0.728814574	0.028412639	0.777509228	-0.024347177
ENSG00000107185	RGP1	9.149227	8.203867	7.059642	7.170673	7.241771	7.375691	7.367343	6.784575	8.414811	0.904584554	-0.016828335	0.848278115	-0.024167622
ENSG00000198169	ZNF251	5.999483	6.219028	6.373094	6.006129	6.085601	6.291496	6.039999	5.319625	5.726489	0.316892182	0.122404737	0.858399626	-0.024048375
ENSG00000108312	UBTF	46.462982	29.887755	36.05195	32.323088	28.76879	27.31703	29.299961	27.479499	36.420937	0.395452818	-0.080870365	0.822096284	-0.024047302
ENSG00000251192	ZNF674	0.821361	0.796945	0.986145	0.793487	0.676505	0.752069	0.985932	0.747086	0.931452	0.729963478	-0.107827983	0.932058538	-0.02402371
ENSG00000069998	HDHD5	11.112203	10.22501	9.832181	7.887534	6.752382	7.611589	8.733004	9.212361	10.825704	0.003658021	-0.337370728	0.866812643	-0.023916653
ENSG00000113732	ATP6V0E1	44.811746	41.892767	40.742663	37.579422	41.584531	37.430859	37.693381	40.423107	37.222488	0.569707425	0.058225617	0.823919234	-0.023890019
ENSG00000151065	DCP1B	6.714559	5.729826	5.596776	5.825169	5.165616	5.954479	6.188847	5.29966	5.199799	0.742322054	0.059298123	0.890293528	-0.023847044
ENSG00000230124	ACBD6	17.186332	22.52618	15.60784	17.071861	16.88015	18.005767	23.272359	20.693827	18.847228	0.953310197	-0.007036079	0.808848362	-0.0238063
ENSG00000253731	PCDHGA6	0.160711	0.329956	0.245119	0.182249	0.171759	0.172747	0.098434	0.243132	0.314821	0.566623015	-0.284053901	0.966259003	-0.023729045
ENSG00000132305	IMMT	59.5657	57.254313	57.397408	54.614894	54.257933	51.674302	52.113669	48.020889	51.503253	0.753708896	0.0245319	0.752751354	-0.023705453
ENSG00000106546	AHR	40.443931	44.639467	46.423063	42.738978	43.491529	41.191773	40.908063	37.982247	43.922806	0.343671765	0.068439979	0.782205158	-0.023579604
ENSG00000086200	IPO11	9.468356	10.102888	9.824227	8.912021	7.215483	8.103845	9.037106	8.690607	10.963303	0.127387671	-0.142309472	0.811839495	-0.023567165
ENSG00000171469	ZNF561	12.512248	11.035294	11.85292	9.764121	8.84376	10.025302	10.218772	11.300857	10.499958	0.404122671	-0.079020804	0.818108952	-0.023550294
ENSG00000144283	PKP4	26.893624	25.498482	28.115874	19.540379	18.20494	18.398813	23.646993	23.366599	26.601055	3.17E-08	-0.319964698	0.774060045	-0.023510153
ENSG00000198546	ZNF511	11.258223	10.431074	9.911457	8.836357	10.080665	9.822196	9.539613	9.583608	9.909152	0.90692973	-0.020436002	0.866165515	-0.023491937
ENSG00000000457	SCYL3	5.749515	5.247445	5.371374	5.775288	5.14694	5.518111	5.757985	4.81882	5.400503	0.669524958	0.047853382	0.828187992	-0.023485623
ENSG00000197635	DPP4	25.335173	23.563152	26.0753	16.241943	17.563631	16.002619	23.733955	22.152445	23.551211	2.04E-10	-0.437425827	0.785705451	-0.023419495
ENSG00000197785	ATAD3A	27.339092	26.362767	24.721921	23.397335	21.721373	22.73131	23.022932	23.14482	25.918882	0.555063429	-0.053206146	0.803588908	-0.023408659
ENSG00000128891	CCDC32	7.121333	5.925586	7.960222	8.72421	7.261244	7.640765	7.517089	7.423102	8.190217	0.001621747	0.345211726	0.869158434	-0.023348262
ENSG00000131778	CHD1L	36.863865	36.823195	35.74452	29.635224	29.495666	27.320934	31.519642	31.722082	35.709803	0.006586509	-0.177433107	0.782597067	-0.023320917
ENSG00000015475	BID	23.58863	24.624167	24.954654	20.578611	23.01595	20.942542	23.314321	23.164951	21.444523	0.806850794	-0.026892407	0.824545883	-0.023294932
ENSG00000131165	CHMP1A	24.585614	24.613041	22.789806	21.22789	20.798163	23.151939	21.381393	19.915566	22.686752	0.809433032	0.025453482	0.812277353	-0.02328057
ENSG00000164114	MAP9	0.900161	1.212492	1.130503	0.893122	0.78698	1.101007	0.960229	0.882479	1.080471	0.972745303	0.010376203	0.911430071	-0.023269978
ENSG00000125676	THOC2	32.849541	36.193385	34.910287	30.387953	29.761024	29.477376	30.317689	31.70358	32.88279	0.401901776	-0.059320652	0.77834954	-0.023176811
ENSG00000115750	TAF1B	8.950327	9.47597	9.330329	7.328168	8.023453	7.81648	9.086523	8.174834	8.529469	0.292837021	-0.125418825	0.856552173	-0.023051332
ENSG00000144233	AMMECR1L	9.402554	10.496382	10.809475	8.199088	7.680143	7.596989	9.525429	8.640355	10.217848	0.004305125	-0.240114039	0.831722684	-0.022895629
ENSG00000179598	PLD6	3.668743	3.36449	4.161606	4.048637	3.025772	3.840786	3.623324	2.996062	3.725848	0.576972113	0.111414146	0.903273224	-0.022687643
ENSG00000047188	YTHDC2	4.978231	6.358175	6.087956	6.954597	5.762526	6.096598	5.646197	4.957867	5.478441	0.01862741	0.256192951	0.861643701	-0.022627083
ENSG00000162066	AMDHD2	9.453257	10.805557	9.750401	9.177881	8.19629	9.438916	9.980576	9.00975	9.953827	0.870707191	-0.023564634	0.853901454	-0.022614333
ENSG00000275395	FCGBP	0.474316	0.332284	0.320842	0.50264	0.664693	0.516095	0.299247	0.37295	0.363569	0.001352961	0.729853361	0.941592133	-0.022603516
ENSG00000143398	PIP5K1A	54.976495	58.894687	52.594676	47.442558	48.741483	47.648059	45.431456	44.84382	52.353813	0.264219557	0.077191215	0.777054786	-0.022401447
ENSG00000213988	ZNF90	1.576269	2.114322	2.18097	2.063003	1.401787	1.612251	2.275895	1.890531	1.643152	0.91886451	-0.036381916	0.941366673	-0.022248859
ENSG00000175931	UBE2O	6.625087	6.881566	6.677524	6.742817	6.134031	6.293306	6.619443	4.994528	6.94074	0.328897552	0.093312686	0.865486116	-0.02211058
ENSG00000206052	DOK6	1.073118	1.058402	0.937857	0.817536	0.784452	0.733008	0.927157	0.952056	0.97518	0.115603995	-0.257915609	0.90201556	-0.022077516
ENSG00000211456	SACM1L	6.91815	8.049494	8.179018	7.162607	7.248356	6.801301	7.054237	6.97082	7.088754	0.983561517	-0.003592586	0.857750574	-0.021918042
ENSG00000275464	FP565260.1	10.015771	10.724656	11.46437	9.542932	10.735553	8.972856	9.89593	8.902059	11.368333	0.939060584	0.011762164	0.854955032	-0.021911969
ENSG00000118922	KLF12	0.999168	0.915436	0.833621	0.778566	0.884503	0.814047	0.841213	0.838281	0.858675	0.989491432	-0.003098664	0.90100403	-0.021901116
NewGene_6530	--	0.515877	0.840551	0.922704	0.486176	0.915763	0.615001	0.614979	0.752162	0.716115	0.944002353	0.042121491	0.962000402	-0.021822645
ENSG00000156802	ATAD2	59.136651	61.655834	63.227321	49.122215	49.128119	47.683209	56.373449	53.650874	60.115388	0.000196151	-0.192533712	0.760244719	-0.021807342
ENSG00000167515	TRAPPC2L	19.71218	15.952115	14.710148	14.025875	14.603734	15.65337	14.412859	15.962003	15.052547	0.976455272	-0.00475458	0.856659204	-0.021778083
ENSG00000204335	SP5	1.628808	2.155107	1.641275	2.247062	2.634075	2.917975	1.625381	1.671539	1.708683	0.001374389	0.673469238	0.938112505	-0.021754009
ENSG00000141279	NPEPPS	25.935436	27.026188	27.882716	28.393595	28.46417	26.319646	25.425322	23.942521	25.824009	0.00741356	0.17550851	0.788414179	-0.021686708
ENSG00000052126	PLEKHA5	22.622092	23.400902	24.763883	24.096031	23.302911	22.33264	22.068974	20.461163	22.268788	0.172171878	0.097799215	0.793947646	-0.021660925
ENSG00000185697	MYBL1	0.656338	0.59788	0.747907	0.62447	0.567669	0.678768	0.60793	0.754703	0.772202	0.973662659	-0.01248534	0.942949022	-0.021613576
ENSG00000008952	SEC62	35.354974	38.484384	42.92557	33.388474	31.938034	32.313819	35.472774	35.055947	37.453061	0.133064784	-0.123651492	0.827299943	-0.021403384
ENSG00000185347	TEDC1	9.80129	8.870134	8.292163	4.890099	5.552551	5.398386	7.102206	8.010452	9.219575	0.00000759	-0.563086576	0.89684612	-0.021401651
ENSG00000141068	KSR1	1.297399	1.40131	1.640951	1.328899	1.304183	1.23811	1.344945	1.401766	1.241867	0.594618052	-0.104457468	0.912527958	-0.021399645
ENSG00000150556	LYPD6B	7.540969	6.926191	8.424358	4.048642	4.031252	4.258856	7.364599	7.461834	7.138358	0.00000405	-0.674007753	0.902381086	-0.021374284
ENSG00000092020	PPP2R3C	20.590529	19.435927	20.137532	17.051668	15.125047	16.15196	18.880342	18.639903	18.433135	0.039194536	-0.185033378	0.84504681	-0.021303863
ENSG00000114405	C3orf14	3.811932	4.281118	4.190033	5.115484	4.64985	5.947868	4.235259	4.214231	5.09983	0.013011403	0.455408707	0.935827788	-0.021249386
ENSG00000169174	PCSK9	25.974216	21.340185	18.854406	34.207893	35.71138	34.995892	20.323574	19.216618	21.67557	1.34E-19	0.811907398	0.868573578	-0.021240856
NewGene_3521	--	8.430886	9.113691	8.491366	7.591088	6.902733	9.039857	9.275177	6.521116	8.999622	0.939060584	0.012014821	0.868949791	-0.021055234
ENSG00000172939	OXSR1	8.467293	8.358377	8.805763	8.268355	8.662133	8.138208	8.388895	7.526244	7.977017	0.219482151	0.111494428	0.838767836	-0.021036671
ENSG00000198876	DCAF12	27.041955	28.219149	27.58858	23.714417	22.858046	24.184261	24.212826	24.79055	27.565637	0.251698371	-0.07973077	0.803588908	-0.020971893
NewGene_3435	--	7.751013	8.057838	7.336483	6.931798	8.09811	5.927142	8.349739	9.689236	8.378056	0.882292018	0.026643236	0.885985683	-0.020845714
ENSG00000114631	PODXL2	12.474597	13.162524	12.276154	13.379455	11.378231	13.106055	11.28588	11.510008	12.238805	0.164816086	0.145870692	0.854855373	-0.020833223
ENSG00000106351	AGFG2	18.787875	18.819698	18.825251	17.253596	21.360921	18.478186	15.249689	16.547547	20.216072	0.070850145	0.168478776	0.852465224	-0.020764567
ENSG00000182952	HMGN4	39.035561	37.331646	40.75198	38.546158	38.971531	38.115612	36.520851	35.311928	36.435402	0.116836396	0.128616691	0.833495305	-0.020740926
ENSG00000140455	USP3	21.123755	21.785022	23.849938	19.61132	19.189655	20.815476	22.052172	19.591487	21.912908	0.744787437	-0.031415382	0.821702965	-0.020696764
ENSG00000138778	CENPE	5.51161	6.307233	6.559101	4.724055	3.968327	4.081786	5.686993	5.487431	5.67799	0.0000293	-0.379493115	0.852465224	-0.020611647
ENSG00000152253	SPC25	15.756826	15.527374	17.419911	11.602029	10.931749	9.814623	14.147423	15.258405	15.460342	0.0000761	-0.431678063	0.875797399	-0.020512703
ENSG00000111897	SERINC1	57.020626	60.642872	61.211281	54.891964	55.700912	53.469219	54.916389	52.573574	57.983337	0.77447683	0.022927254	0.786066291	-0.02047378
ENSG00000091651	ORC6	14.198426	15.333937	17.623362	12.551588	11.040252	12.215519	14.935177	14.415516	14.314283	0.029887292	-0.25009125	0.879387622	-0.020420765
ENSG00000147364	FBXO25	8.026491	6.480301	5.681099	6.340701	6.088311	6.720855	7.087314	6.190575	8.313034	0.426379119	0.076394448	0.842882672	-0.020353465
ENSG00000145041	DCAF1	7.441452	8.170178	8.714887	7.685565	7.029761	7.401183	7.8506	7.120338	8.595001	0.864192776	-0.020042988	0.849018257	-0.020344212

Gene ID	Gene name	Ctrl-1 FPKM	Ctrl-2 FPKM	Ctrl-3 FPKM	IFNγ-1 FPKM	IFNγ-2 FPKM	IFNγ-3 FPKM	Sora-1 FPKM	Sora-2 FPKM	Sora-3 FPKM	Ctrl vs IFNγ DESeq2 FDR	Ctrl vs IFNγ DESeq2 log2FC	Ctrl vs Sora DESeq2 FDR	Ctrl vs Sora DESeq2 log2FC
ENSG00000083937	CHMP2B	18.445826	20.292919	21.054601	18.802432	18.873835	20.124998	18.85829	18.256496	19.780975	0.39794821	0.080887645	0.841440089	-0.02022393
ENSG00000100442	FKBP3	62.274757	63.671444	63.108354	55.524523	53.854372	53.541961	61.138881	59.184724	53.774677	0.301441498	-0.073896966	0.842545788	-0.020188638
ENSG00000176720	BOK	7.594251	8.189591	6.90264	5.514757	5.634209	6.089598	7.157761	6.372553	7.472156	0.038662008	-0.246491102	0.883496834	-0.020086823
ENSG00000164654	MIOS	14.163595	14.326387	15.088564	11.954892	11.716129	11.538646	13.169734	13.591839	13.230745	0.074899771	-0.15279446	0.853155801	-0.019822005
ENSG00000174013	FBXO45	4.661702	5.375386	6.316053	5.032372	5.045431	5.00946	4.780376	5.1031	5.779995	0.986054396	-0.002973222	0.885612975	-0.019743535
ENSG00000122299	ZC3H7A	31.770631	31.888923	35.685624	32.697522	33.450188	31.800438	33.380935	28.430586	33.256007	0.133136924	0.110138322	0.826342708	-0.019503237
ENSG00000182768	NGRN	23.111563	21.989471	22.103684	17.944736	17.591987	17.844146	19.832097	21.133569	21.130334	0.000941689	-0.184295116	0.813450975	-0.019434027
ENSG00000070759	TESK2	0.769622	0.452927	0.471492	1.031334	1.291856	1.307504	0.567962	0.525036	0.476398	0.00000441	1.239336437	0.964073816	-0.019379336
ENSG00000143799	PARP1	118.587641	114.008695	114.935755	126.011973	117.302601	121.178855	105.159772	103.077615	114.404789	0.00000672	0.214795392	0.777908308	-0.019364218
ENSG00000171055	FEZ2	14.445809	13.277065	14.063765	11.141662	13.464283	12.593694	13.316528	11.743616	13.599587	0.918672101	-0.016632076	0.869545989	-0.019268688
ENSG00000108474	PIGL	7.223588	9.431659	7.926133	8.965572	8.765553	8.815835	8.415272	7.499457	7.896574	0.123929691	0.240602376	0.917995936	-0.019263901
ENSG00000135862	LAMC1	64.89856	62.120133	65.018829	39.774212	39.186359	38.400608	57.308167	56.300232	64.308891	3.43E-35	-0.563229312	0.793536946	-0.019223182
ENSG00000130764	LRRC47	12.847224	13.240878	12.846125	13.37629	13.125337	13.345517	11.955047	11.332829	12.721312	0.005307049	0.191251675	0.834198339	-0.019192833
ENSG00000197892	KIF13B	7.236387	6.352876	6.350252	5.807036	5.96783	5.714177	6.384289	5.459171	6.69938	0.707366062	-0.040332415	0.862461619	-0.019020167
ENSG00000041988	THAP3	7.323867	5.967726	5.431263	5.25737	4.482949	5.152822	5.794208	5.916756	5.934816	0.311195941	-0.215751831	0.929282933	-0.019016966
ENSG00000143569	UBAP2L	79.238288	69.532475	73.057396	69.057647	64.269544	67.812538	66.1481	62.85847	74.726421	0.219402591	0.081397737	0.82080509	-0.018953383
ENSG00000136522	MRPL47	64.772306	64.721345	63.494512	55.011948	52.776814	53.984231	58.522981	60.803302	60.402077	0.150328547	-0.097436621	0.824341092	-0.018780103
ENSG00000101049	SGK2	27.061032	24.177671	20.494868	31.991977	32.421979	30.130443	23.604303	21.572928	21.534916	4.81E-09	0.535198618	0.885898512	-0.018527243
ENSG00000078018	MAP2	1.884408	1.42129	1.767534	1.219264	1.004953	0.884182	1.454361	1.681594	1.799262	0.000581269	-0.603695931	0.928309494	-0.018524505
ENSG00000027001	MIPEP	4.216244	4.267255	4.619523	3.43112	3.034574	3.229805	4.28366	4.0866	3.747188	0.050724639	-0.288755878	0.914949978	-0.01838678
ENSG00000168077	SCARA3	28.630554	25.593385	21.210567	21.943373	24.05868	23.700656	23.063887	21.849033	25.468793	0.802746973	0.034623862	0.882419864	-0.018361538
ENSG00000102984	ZNF821	4.300252	4.24605	3.753382	3.990088	3.033877	4.044543	3.776422	3.932454	4.052718	0.962990989	0.012362992	0.924295683	-0.018303197
ENSG00000179542	SLITRK4	0.249126	0.227833	0.21187	0.30431	0.38723	0.308386	0.181587	0.271928	0.341687	0.975869953	0.014558162	0.961586983	-0.018215992
ENSG00000146950	SHROOM2	2.112161	2.27047	2.303956	1.736105	1.945031	1.784433	2.288769	1.86749	2.051909	0.245838001	-0.155901055	0.901457625	-0.018194769
ENSG00000090621	PABPC4	53.263749	53.802022	54.695562	48.48931	44.01886	45.267026	49.230935	49.015362	50.987196	0.091996712	-0.108348867	0.810939169	-0.018129243
ENSG00000106733	NMRK1	3.104548	3.154294	3.330151	2.818448	3.251652	2.832499	3.66394	2.857516	2.899438	0.998196506	-0.001073165	0.939768355	-0.017837079
ENSG00000075651	PLD1	17.269437	18.144664	19.062662	11.953388	11.693545	11.916877	16.715346	15.572576	17.676992	1.52E-15	-0.491772032	0.837991965	-0.017812842
ENSG00000106305	AIMP2	70.778961	65.718039	71.375591	61.0944	57.501672	61.670128	64.003054	62.992536	66.206867	0.412635041	-0.068017227	0.840241952	-0.017702459
NewGene_4731	--	0.919269	0.852586	0.732916	0.694209	0.838036	0.766228	0.748461	0.766568	0.853268	0.953310197	-0.02879402	0.963407501	-0.017638337
ENSG00000173960	UBXN2A	2.821916	3.055979	3.613087	2.829154	2.471259	3.020792	3.033928	2.692534	3.013089	0.856107824	-0.033640507	0.908042167	-0.017536499
ENSG00000125814	NAPB	3.454911	3.812919	3.825658	3.608419	3.193186	3.180818	3.618716	3.12297	3.43338	0.944487846	0.013239481	0.90840876	-0.017511321
ENSG00000122557	HERPUD2	10.235513	10.737866	9.108743	9.083656	9.0131	8.427013	9.364362	8.222509	9.390112	0.960983569	-0.007268783	0.885049652	-0.017376199
ENSG00000087470	DNM1L	18.190671	20.884149	21.190453	20.797886	18.460626	17.783999	19.078841	18.547671	20.429176	0.986348802	-0.002294266	0.871692859	-0.017360626
ENSG00000258539	AC068896.1	0.393693	0.621691	0.750388	0.56005	0.412651	0.62697	0.437574	0.640188	0.550946	0.979153748	0.01436774	0.967702568	-0.017259658
ENSG00000067066	SP100	9.339672	10.949003	10.213347	29.392053	28.817838	28.453166	8.491615	7.933421	7.940293	5.19E-154	1.799592525	0.898487896	-0.017157341
ENSG00000170248	PDCD6IP	18.311955	18.882551	17.171396	16.013831	15.880943	15.905156	16.4327	16.305309	17.77905	0.735034477	-0.028903541	0.840185326	-0.01700622
ENSG00000169635	HIC2	6.661289	6.608036	5.990404	8.316864	8.132329	8.706763	6.043779	5.356118	6.522094	1.12E-13	0.531276337	0.878048861	-0.016800433
ENSG00000270647	TAF15	80.957143	76.18208	76.671123	75.946821	74.107046	72.061345	74.202043	70.799734	70.574228	0.26447679	0.068989558	0.83551186	-0.016687685
ENSG00000178385	PLEKHM3	1.2347	1.330307	1.499046	1.136497	0.971697	1.060154	1.345509	1.337178	1.232225	0.115971049	-0.225643679	0.916345377	-0.016630995
ENSG00000198792	TMEM184B	19.303408	19.305715	17.818989	13.750691	13.58021	13.75966	16.254218	16.325371	19.645758	0.0000171	-0.30472631	0.875982184	-0.016574497
ENSG00000101856	PGRMC1	39.882903	37.596191	37.284356	39.323478	45.62959	41.469566	34.989883	34.705525	36.674904	0.000203382	0.288049115	0.850934519	-0.016521263
ENSG00000115866	DARS	48.881438	52.168932	50.367753	44.75542	42.791363	45.022613	48.606335	43.898626	47.938468	0.557010488	-0.044603789	0.841732594	-0.016405917
ENSG00000108406	DHX40	12.940518	13.28654	12.678612	10.954005	10.090174	9.428173	11.925565	11.474447	12.744972	0.013640882	-0.205654363	0.862972902	-0.016381188
ENSG00000171045	TSNARE1	2.972882	3.181658	2.914525	3.021733	2.624202	2.446524	2.657408	2.527367	3.626632	0.891241543	-0.035427607	0.941592133	-0.016359194
ENSG00000165684	SNAPC4	8.120449	7.178254	7.308839	7.038038	5.631111	6.357549	6.89441	6.306887	7.902035	0.290291926	-0.123142919	0.890486596	-0.016335002
ENSG00000069329	VPS35	16.936172	17.146076	16.15146	15.172464	15.338057	15.094402	15.432977	15.874634	15.71102	0.955714683	-0.005215019	0.84230252	-0.016098397
ENSG00000072501	SMC1A	18.602677	17.76453	17.880346	16.556489	14.841723	15.385772	16.151176	15.813147	18.330321	0.236377727	-0.078586561	0.846237079	-0.015951608
NewGene_5186	--	1.938023	1.78993	1.822401	0.933637	1.019686	0.811626	1.411922	2.179974	1.548501	0.035603529	-0.851083703	0.972385951	-0.015873164
ENSG00000171241	SHCBP1	4.983473	6.171406	6.357404	6.040948	5.534039	5.394267	6.067999	4.819816	5.364183	0.480467125	0.10177661	0.921081605	-0.015640043
ENSG00000146530	VWDE	3.923177	4.738222	4.975806	3.46007	3.926775	4.050738	4.189194	4.130655	4.319809	0.268049032	-0.136934093	0.906897563	-0.01555421
ENSG00000047621	C12orf4	7.898996	8.635395	9.088974	9.607944	9.527028	9.338187	8.310101	7.262796	8.687284	0.000731547	0.293462963	0.89684612	-0.015519079
ENSG00000213079	SCAF8	16.886139	17.427127	17.836986	16.836822	16.531224	16.882388	17.014526	14.60556	16.744755	0.16035839	0.095684694	0.864909004	-0.015439765
ENSG00000101190	TCFL5	8.002648	8.03314	6.831813	6.880909	6.689466	6.10019	6.951985	6.706388	7.590004	0.633100416	-0.071934274	0.912028457	-0.015421101
ENSG00000150316	CWC15	19.399776	17.506077	17.605999	15.922835	14.974504	14.34257	16.037222	17.20944	17.312275	0.258305478	-0.122553214	0.898660383	-0.015391476
ENSG00000188092	GPR89B	17.022423	19.130273	18.629119	21.445076	20.120006	21.860687	19.935752	14.323308	17.889143	0.0000185	0.346123106	0.909421507	-0.015387966
ENSG00000260238	PMF1-BGLAP	21.932598	22.36623	9.633928	14.106486	14.623637	18.522988	16.539596	21.634508	11.473072	0.95580749	-0.03937528	0.982816335	-0.015331167
ENSG00000273611	ZNHIT3	27.300257	25.420588	25.115388	21.701909	23.592299	24.847322	24.994008	23.93405	22.937098	0.998642855	-0.000433471	0.903308718	-0.015296649
ENSG00000140416	TPM1	37.066911	36.511066	42.91779	26.419393	25.44785	25.419578	38.22304	34.371367	38.167565	4.6E-10	-0.48637402	0.885612975	-0.015226004
ENSG00000146247	PHIP	10.782311	12.881959	13.137078	12.906823	11.21364	11.255028	11.237026	11.035566	11.880732	0.363224226	0.089676179	0.881282409	-0.015221092
ENSG00000169570	DTWD2	1.314012	1.431354	1.422846	1.099383	1.173886	1.180131	1.358101	1.165123	1.374114	0.568445676	-0.135887994	0.947316463	-0.015176108
ENSG00000177854	TMEM187	0.567332	0.67177	0.64487	0.408011	0.505015	0.518128	0.48503	0.66	0.597635	0.649106635	-0.233288141	0.975113162	-0.015079074
ENSG00000197771	MCMBP	33.63989	35.628566	36.528229	31.337242	30.748954	31.477879	32.010519	31.255108	35.99788	0.896584156	-0.012064659	0.861108363	-0.014894135
ENSG00000101367	MAPRE1	95.424675	100.975266	107.039597	89.765724	89.635353	91.482033	96.292473	91.355995	93.908577	0.853822106	-0.015997083	0.856253111	-0.014849042
ENSG00000107554	DNMBP	1.561689	1.311489	1.246069	1.517105	1.342667	1.153002	1.377452	0.940965	1.247002	0.681847625	0.090693092	0.945566624	-0.014686053
ENSG00000106803	SEC61B	156.344016	158.436737	148.926345	178.393036	183.286048	197.895503	147.30527	141.399678	141.232348	8.41E-11	0.419699346	0.869068091	-0.014638534
ENSG00000106327	TFR2	0.798757	0.558723	0.55841	0.846333	0.556866	0.714788	0.743325	0.351245	0.687611	0.472135653	0.266627724	0.973768784	-0.014549859
ENSG00000147274	RBMX	109.73986	89.177546	88.144625	81.040189	90.976533	77.761021	96.767731	111.927417	110.697175	0.757338312	-0.022499954	0.835719911	-0.014505281
ENSG00000149679	CABLES2	5.7389	5.739025	5.42886	7.125927	6.397774	6.683534	5.399986	4.587671	5.746448	0.00000816	0.40298391	0.912847752	-0.014438729
ENSG00000134014	ELP3	10.518882	10.648284	10.425336	10.396779	9.333496	9.784844	9.905534	9.759225	9.353729	0.889945453	0.016785956	0.895677056	-0.013999159
ENSG00000174574	AKIRIN1	67.497831	69.661343	71.235357	48.336905	47.780572	45.621601	64.903565	59.174129	68.733195	1.53E-16	-0.416682183	0.860381035	-0.013923731
ENSG00000103037	SETD6	6.708193	6.798033	6.219895	5.714016	5.525982	5.463806	6.633892	6.73771	6.569262	0.780530674	-0.040395028	0.915392351	-0.013915175
ENSG00000142039	CCDC97	12.860785	11.846443	11.717989	11.41888	11.127092	11.335057	11.648487	11.680954	12.471393	0.942855622	0.009273844	0.89974114	-0.013894798
ENSG00000037241	RPL26L1	28.809806	30.363772	27.105382	24.761218	23.535127	25.947752	26.133729	26.948042	26.622867	0.52240999	-0.085402175	0.917773371	-0.013793302
ENSG00000071794	HLTF	13.446245	14.933919	13.196447	14.52884	14.318561	14.438773	12.903415	11.608332	13.702249	0.010686128	0.188646427	0.890058192	-0.013729445

Gene ID	Gene name	Ctrl-1 FPKM	Ctrl-2 FPKM	Ctrl-3 FPKM	IFNγ-1 FPKM	IFNγ-2 FPKM	IFNγ-3 FPKM	Sora-1 FPKM	Sora-2 FPKM	Sora-3 FPKM	Ctrl vs IFNγ DESeq2 FDR	Ctrl vs IFNγ DESeq2 log2FC	Ctrl vs Sora DESeq2 FDR	Ctrl vs Sora DESeq2 log2FC
ENSG00000104064	GABPB1	13.454806	12.719597	13.657452	12.248739	11.654987	10.877316	15.513369	13.695496	12.178989	0.489188502	-0.068019892	0.903159777	-0.013636063
ENSG00000103005	USB1	19.732777	16.341154	17.099565	14.011825	13.760951	14.440209	14.73202	16.061666	17.891289	0.072708173	-0.183873791	0.914505594	-0.013629704
ENSG00000254087	LYN	4.374876	4.66806	4.735762	4.360019	3.518382	4.061402	4.35468	3.809109	3.943723	0.826676227	0.033959013	0.918942045	-0.013250498
ENSG00000140943	MBTPS1	19.514687	18.431757	18.198497	17.546092	16.209053	16.697651	17.150001	16.155649	18.894614	0.901719606	-0.01237781	0.884622383	-0.013174271
ENSG00000071189	SNX13	11.645682	12.172484	11.780202	13.057239	12.238234	11.557782	10.544471	10.506175	12.090263	0.003685246	0.198312898	0.885985683	-0.013173237
ENSG00000106443	PHF14	21.434594	22.660676	22.063648	25.904083	25.780813	22.543847	26.571836	23.025409	23.604021	0.000181806	0.213615458	0.869143393	-0.013014377
ENSG00000171992	SYNPO	0.44152	0.496327	0.519942	3.235918	3.403481	3.603801	0.355083	0.428403	0.570996	9.64E-110	2.931590591	0.96850965	-0.012977476
ENSG00000115216	NRBP1	32.858141	29.691729	27.072999	30.759278	29.074957	32.687944	26.423048	26.626201	32.970032	0.026655316	0.174581084	0.904307282	-0.012880794
NewGene 3516	--	1.492104	2.239355	1.5306	1.551684	1.535228	1.763851	1.52181	1.820177	1.987046	0.453615273	-0.14714965	0.947317656	-0.012865554
ENSG00000135968	GCC2	8.557314	9.00221	9.990994	8.741254	7.91234	7.524849	8.615057	8.163105	9.833593	0.841889215	-0.022667805	0.908265371	-0.012632891
ENSG00000198554	WDHD1	5.714438	6.163602	6.070784	4.745952	4.62016	4.840357	5.581742	5.577799	5.21788	0.065912719	-0.16695418	0.910569134	-0.012591978
ENSG00000188312	CENPP	2.24032	2.159395	2.634489	2.186151	1.873736	1.79612	2.015883	2.142927	2.46452	0.450744149	-0.108308374	0.929945499	-0.012585015
ENSG00000130204	TOMM40	68.827513	67.719028	65.399968	56.240015	58.827896	59.513486	63.518971	64.121258	65.937863	0.240172266	-0.077242478	0.873630739	-0.012512706
ENSG00000116001	TIA1	34.51179	37.735591	35.280139	35.223911	33.509469	33.088606	36.213361	34.389406	38.249112	0.586859461	0.046319629	0.885688258	-0.012469452
ENSG00000204438	GPANK1	32.514168	28.333047	26.941905	28.759674	31.331553	28.492888	24.823636	27.878091	27.958984	0.133297554	0.143678042	0.911682134	-0.012426833
ENSG00000176619	LMNB2	109.211166	110.096848	105.967506	98.09108	93.686455	99.06562	101.222519	92.169281	109.482063	0.842325548	-0.01414666	0.869679217	-0.012415499
ENSG00000126243	LRFN3	3.765479	2.80442	3.346527	3.114177	3.191043	3.000956	2.8092	2.842009	3.324636	0.906528123	0.025519739	0.95066916	-0.012393497
ENSG00000116350	SRSF4	36.598999	34.020304	37.876391	39.08956	37.712885	33.603991	33.572874	30.235005	36.188608	0.039099581	0.166970148	0.900204778	-0.01229553
ENSG00000177119	ANO6	11.574178	12.014272	11.564535	13.218591	13.698304	13.61336	11.098429	10.093343	11.451055	3.45E-08	0.332511762	0.889608968	-0.012229971
ENSG00000132855	ANGPTL3	27.17927	28.673538	29.83744	22.340721	22.294855	20.597563	26.297356	25.502953	27.914225	0.00046405	-0.246362262	0.890490634	-0.012169058
ENSG00000133104	SPART	19.972403	20.196362	21.313692	15.505638	15.331089	15.493035	19.87933	22.442886	18.492375	0.000545967	-0.219343033	0.885985683	-0.012052134
ENSG00000083544	TDRD3	1.179566	1.156473	0.870338	1.088354	0.904675	0.969187	1.099822	0.790978	0.879902	0.999777513	0.0000823	0.96988122	-0.012026362
ENSG00000086712	TXLNG	7.460347	8.989995	9.327981	7.402438	7.075361	6.925268	7.646276	8.213102	8.079789	0.280233493	-0.122446445	0.925881309	-0.01194771
ENSG00000134330	IAH1	11.140795	10.859532	9.686359	8.369188	7.820865	9.156743	9.719565	10.543419	9.741386	0.828573567	-0.036804254	0.940759682	-0.011940532
ENSG00000132963	POMP	54.563386	57.610828	63.517623	67.095556	67.036244	67.702022	54.749779	60.170326	48.031109	0.000000451	0.348404889	0.927915382	-0.011814017
ENSG00000115129	TP53I3	8.270514	7.996692	7.165346	4.912308	5.384896	5.423803	6.866426	7.409207	7.757765	0.00048727	-0.459351184	0.944041721	-0.011637021
ENSG00000185924	RTN4RL1	2.443702	2.163164	2.032503	2.893157	2.020471	2.874108	2.111206	1.967978	2.094792	0.033992189	0.37626636	0.952790163	-0.011589405
ENSG00000170921	TANC2	0.158913	0.372656	0.29282	0.196354	0.104819	0.098283	0.255266	0.180692	0.229521	0.024387624	-0.777986378	0.975941621	-0.011538701
ENSG00000180304	OAZ2	43.88619	40.194217	40.496694	27.176344	28.022297	28.560592	37.926832	38.483564	40.788929	7.75E-10	-0.427849551	0.902212016	-0.011506669
ENSG00000110442	COMMD9	31.302699	30.10922	24.043769	22.684817	21.360818	22.681881	25.912306	23.030538	32.804382	0.902061001	-0.011887258	0.896001443	-0.011423329
ENSG00000148572	NRBF2	12.862638	13.016177	13.866855	13.217381	14.364484	13.742909	11.921757	12.311218	12.691587	0.038996935	0.205624759	0.924828481	-0.011374748
ENSG00000154655	L3MBTL4	1.507332	1.207623	1.463184	1.85544	1.902613	1.695685	1.441991	1.219146	1.165795	0.003919897	0.512642131	0.965353182	-0.01095963
ENSG00000196950	SLC39A10	16.757653	17.466106	16.462145	12.093194	12.528855	11.790665	14.69623	15.269097	17.433335	0.000000039	-0.349251037	0.908115545	-0.01084353
ENSG00000138095	LRPPRC	67.130689	71.610797	71.576175	63.015244	57.051625	58.94395	66.075146	62.27412	70.123639	0.157588082	-0.08561137	0.88213858	-0.0108014
ENSG00000114861	FOXP1	6.905613	7.652835	6.732838	9.98385	8.506312	8.016643	9.023857	8.723498	8.323317	4.97E-10	0.453117683	0.918942045	-0.010785253
ENSG00000176915	ANKLE2	22.410864	23.272036	25.222488	21.605018	19.763685	20.699801	21.650618	19.106633	23.454624	0.590519698	-0.046851542	0.907613509	-0.01072035
ENSG00000101335	MYL9	6.011816	5.677572	5.762925	2.678877	3.010866	2.913327	5.33666	5.041219	5.516388	1.81E-09	-0.734496293	0.943937035	-0.010713612
ENSG00000142949	PTPRF	50.112586	49.304073	44.524549	38.642729	35.90909	42.257636	40.659391	42.67015	50.058389	0.000103601	-0.218039014	0.910826189	-0.010560832
ENSG00000184207	PGP	14.031544	14.257062	12.29588	13.170453	13.990176	14.761145	12.758469	12.819321	12.193024	0.037880126	0.200529754	0.928903519	-0.010489806
ENSG00000176533	GNG7	1.312859	1.343085	1.69903	1.360161	1.647412	1.432661	1.365674	1.304087	1.360565	0.407010393	0.18291061	0.964229184	-0.010405972
ENSG00000172889	EGFL7	17.271502	14.619596	12.888095	14.846064	15.859712	16.825382	14.043378	14.294352	13.324073	0.082249529	0.233618573	0.949730731	-0.010329383
ENSG00000092295	TGM1	0.677133	0.497214	1.247276	0.596553	0.773165	0.624878	0.742379	0.803901	1.202879	0.68791148	-0.197939467	0.983230348	-0.010260076
ENSG00000143420	ENSA	107.042916	116.369545	110.769785	122.873988	120.457337	120.699646	103.5442	108.502509	97.588763	0.000000153	0.261383654	0.912478833	-0.010256484
ENSG00000167118	URM1	22.557542	23.154671	22.281786	19.209368	18.090031	19.56604	21.192959	21.880745	22.448812	0.068605509	-0.141806026	0.911553655	-0.010200888
ENSG00000120686	UFM1	21.929727	22.293752	24.09511	23.800051	22.635625	22.047004	21.105803	21.70311	21.938394	0.082511713	0.131588888	0.914976479	-0.010194065
ENSG00000124562	SNRPC	193.315537	189.547819	181.453718	165.781241	164.00144	170.560805	174.081462	180.852917	171.960058	0.735321503	-0.026425295	0.906560173	-0.010142921
ENSG00000136874	STX17	7.030255	7.564142	7.330291	11.329303	10.919052	11.142085	7.032452	6.932983	6.757107	5.4E-20	0.753308713	0.939681928	-0.010092304
ENSG00000155508	CNOT8	14.130278	14.954608	18.668903	13.009113	14.302722	11.298325	14.266321	14.094728	17.404462	0.048720859	-0.212031835	0.936838732	-0.010070568
ENSG00000118557	PMFBP1	1.786594	1.68732	1.800742	1.264237	0.987755	1.099122	1.459709	1.64156	1.911518	0.060102802	-0.435154565	0.969970419	-0.009992136
ENSG00000143570	SLC39A1	87.100793	81.665975	83.381916	67.762973	68.117898	69.352334	74.735165	80.987705	81.447165	0.007833511	-0.156343284	0.905636281	-0.009986842
ENSG00000164953	TMEM67	0.588522	0.725507	0.713152	0.445283	0.316961	0.293667	0.719932	0.620486	0.561832	0.010021422	-0.737610368	0.974534926	-0.009966928
ENSG00000115042	FAHD2A	5.744282	5.263386	4.914502	4.537766	4.225771	5.094276	5.701829	5.603419	5.70354	0.931574885	-0.015440242	0.950124417	-0.009759195
ENSG00000137478	FCHSD2	1.825524	2.006618	1.97161	1.880883	1.535302	1.719636	1.926336	1.903941	1.685145	0.769686351	-0.06455874	0.96202619	-0.009715969
ENSG00000168438	CDC40	7.856259	9.952427	10.178446	10.173429	9.643027	10.035297	8.679006	8.424812	8.839477	0.024083364	0.240427276	0.943158719	-0.009688914
ENSG00000110768	GTF2H1	36.490449	37.443176	39.068486	32.95413	32.630538	32.217755	32.818347	34.315677	36.331695	0.393871439	-0.060801365	0.910800899	-0.009601637
ENSG00000075275	CELSR1	9.038419	8.920877	8.918994	6.243219	5.256259	6.088841	7.816602	7.961236	9.387395	6.72E-12	-0.46515384	0.918980072	-0.009366085
ENSG00000161267	BDH1	2.256854	2.357797	1.352548	1.573156	1.721103	1.644655	1.550243	1.861902	1.445391	0.783661849	0.083010356	0.973768784	-0.009274366
ENSG00000177733	HNRNPA0	22.797623	22.82023	21.404377	19.594711	18.912205	19.928343	21.195621	19.472618	21.836893	0.471190672	-0.050483355	0.905630163	-0.009261208
ENSG00000176890	TYMS	69.877531	73.140146	70.373665	59.728441	56.316104	56.682911	65.039173	64.581941	69.243313	0.009366697	-0.155853537	0.90692555	-0.009203982
ENSG00000108424	KPNB1	123.075538	103.752333	115.429684	91.110859	88.363428	93.173805	88.665101	96.26469	98.667641	0.986087904	0.001369511	0.899327971	-0.009028914
ENSG00000127580	WDR24	6.021554	6.476695	6.001477	6.317385	5.924704	6.21603	5.387101	5.811723	6.092704	0.222536624	0.137660388	0.946733803	-0.008985253
NewGene 6419	--	1.849076	2.165366	2.637707	2.336568	2.644148	2.396501	2.692391	2.580111	1.951592	0.467348823	0.15387601	0.969933279	-0.008978934
ENSG00000131652	THOC6	33.160653	31.043564	28.75594	21.803078	21.919163	21.334559	27.924106	28.413029	29.534548	0.0000243	-0.354093476	0.936114134	-0.008950178
ENSG00000152422	XRCC4	6.134913	6.789546	6.991697	4.961961	5.254091	5.507458	6.438861	6.306485	5.584099	0.368790982	-0.152237764	0.962960978	-0.00874186
ENSG00000162458	FBLIM1	33.215965	35.250654	33.108114	27.170845	30.079553	27.330513	29.987302	30.415086	34.058094	0.11555196	-0.117612376	0.91945203	-0.00873049
ENSG00000117748	RPA2	20.731425	18.080098	17.945093	16.23565	17.470163	16.636521	17.870551	17.135871	17.360959	0.871017858	-0.021830473	0.942818963	-0.008536412
ENSG00000130717	UCK1	23.674551	21.495282	20.614597	19.922031	20.054167	20.127443	20.043032	19.800089	21.38103	0.883958856	0.017696124	0.937259399	-0.008429251
ENSG00000113621	TXNDC15	4.580762	4.671125	4.718889	4.284625	4.354071	4.029517	4.210811	4.172586	4.824304	0.961910939	0.008376273	0.954166755	-0.008376502
ENSG00000151849	CENPJ	4.588885	4.403867	4.463859	4.659079	3.627791	3.879638	4.431218	4.112353	4.106701	0.94471444	-0.0125912	0.951677266	-0.008288285
ENSG00000132406	TMEM128	20.351337	23.879734	23.059901	22.531433	23.325174	21.119553	23.058778	18.830234	19.677259	0.205413928	0.139828713	0.954749805	-0.007986514
ENSG00000188677	PARVB	4.02601	4.470984	4.366008	3.490506	3.468808	3.857831	3.997278	3.813978	4.502359	0.322256873	-0.121264924	0.95066916	-0.007955587
ENSG00000174099	MSRB3	1.511651	1.762198	2.061704	1.136136	0.744285	0.864884	1.71083	1.702311	1.70123	0.0000216	-0.824026309	0.97089042	-0.007955186
ENSG00000124356	STAMBP	9.532076	8.741395	9.432609	7.683247	8.525287	8.196494	8.486872	8.327097	8.865515	0.856107824	-0.021192426	0.934167153	-0.00795366
ENSG00000070770	CSNK2A2	18.291163	17.500049	19.293183	17.73245	16.66516	17.71431	19.228337	17.830323	19.326369	0.401909837	0.072255749	0.935366587	-0.007873927

Gene ID	Gene name	Ctrl-1 FPKM	Ctrl-2 FPKM	Ctrl-3 FPKM	IFNγ-1 FPKM	IFNγ-2 FPKM	IFNγ-3 FPKM	Sora-1 FPKM	Sora-2 FPKM	Sora-3 FPKM	Ctrl vs IFNγ DESeq2 FDR	Ctrl vs IFNγ DESeq2 log2FC	Ctrl vs Sora DESeq2 FDR	Ctrl vs Sora DESeq2 log2FC
ENSG00000051180	RAD51	6.607978	6.556523	7.406192	8.779775	6.016737	7.330048	6.299727	5.790834	6.967231	0.737882967	0.056525486	0.962000402	-0.007763532
ENSG00000197798	FAM118B	10.77356	8.382946	9.946952	7.454015	7.584016	7.367271	8.31147	9.981441	8.748803	0.059140708	-0.233531035	0.960770425	-0.007763025
ENSG00000278615	C11orf98	84.55666	86.515804	89.014782	73.762159	67.836033	72.766243	79.649074	84.185543	77.908466	0.088165609	-0.132667498	0.941366673	-0.007735521
NewGene 1041	--	9.937356	10.289943	10.176575	7.937892	6.788898	8.487674	9.69734	8.89619	10.301469	0.083630617	-0.223141209	0.956346132	-0.007393178
ENSG00000080802	CNOT4	4.710405	5.110493	5.635011	6.572383	5.659005	5.927554	4.698105	4.310955	4.440581	0.000748061	0.370418759	0.95962379	-0.007326658
ENSG00000124226	RNF114	22.424127	21.760638	22.563833	28.867609	27.408523	28.376086	21.347364	19.757447	21.057266	9.49E-15	0.471134262	0.938453074	-0.007283752
ENSG00000144744	UBA3	20.85901	20.179483	20.745276	18.400737	18.252419	17.241311	19.413839	19.245599	19.022787	0.63852072	-0.047133557	0.943620534	-0.007202133
ENSG00000152492	CCDC50	50.163943	50.775752	54.872716	36.531035	35.083043	34.134356	51.357191	53.461174	57.682415	4.21E-08	-0.332193206	0.937152538	-0.00709599
ENSG00000187889	FYB2	1.445189	1.799674	1.460223	2.025862	1.697231	1.827178	1.661508	1.384242	1.351767	0.04985399	0.383019612	0.978645714	-0.007034914
ENSG00000147475	ERLIN2	15.55481	15.496039	14.412348	12.024459	11.568158	12.662571	14.359146	13.115212	14.863518	0.017803171	-0.163707832	0.937977487	-0.007019233
ENSG00000204128	C2orf72	35.043346	32.668858	27.411524	33.154064	35.773392	34.662918	29.903801	27.729073	31.224319	0.001899123	0.270721472	0.951123414	-0.00697381
ENSG00000188419	CHM	5.671691	6.223146	6.381947	5.521421	5.028736	5.401312	5.780356	5.530014	5.911051	0.714712773	-0.043983425	0.952877902	-0.006837516
ENSG00000112339	HBS1L	16.256317	17.645459	16.369982	16.880434	14.353815	15.630036	16.390499	14.972769	16.480604	0.531002535	-0.046617119	0.934813803	-0.006823202
NewGene 3018	--	1.365507	1.38439	1.153361	1.079971	0.768837	1.144368	1.323076	1.048247	1.329437	0.511860514	-0.200491868	0.982862391	-0.006716504
ENSG00000085185	BCORL1	2.430823	2.09894	2.114761	2.577672	2.317524	2.470201	1.908781	2.123415	2.205283	0.001348507	0.393920679	0.96850965	-0.006689656
ENSG00000131470	PSMC3IP	6.176183	4.989703	6.048277	4.816933	4.143295	4.11577	4.722385	5.511395	5.453278	0.10058735	-0.320044578	0.976257956	-0.00666421
ENSG00000162989	KCNJ3	0.408024	0.510623	0.496886	0.818775	0.810947	0.890187	0.575493	0.298841	0.462411	0.0000169	0.944920647	0.986971729	-0.006399571
ENSG00000168813	ZNF507	7.920184	9.03419	8.515166	8.791306	7.691914	8.861379	8.990396	7.630258	8.202289	0.159440152	0.124064371	0.95634566	-0.006379617
ENSG00000134697	GNL2	28.174322	27.107859	29.619809	26.102528	24.204856	24.506051	24.97635	26.016168	28.624775	0.653578575	-0.042943248	0.950035312	-0.006372384
ENSG00000278662	GOLGA6L10	0.726045	0.606834	0.805182	0.613982	0.531916	0.577684	0.882464	0.747882	0.64019	0.581774858	-0.17923706	0.986088347	-0.006362232
ENSG00000139197	PEX5	18.22308	19.564141	17.084454	16.708494	17.392381	18.641711	17.015644	16.908708	17.221522	0.313045101	0.087664835	0.947389707	-0.006285082
ENSG00000134686	PHC2	58.070225	53.006303	52.152055	51.255149	50.84236	51.849655	48.939563	48.518212	54.746307	0.415714593	0.062753745	0.944730411	-0.00622995
ENSG00000128915	ICE2	6.811727	7.442105	7.056987	6.808785	6.458854	6.682581	6.760046	5.687735	7.750823	0.889534724	0.015729919	0.962000402	-0.006179424
ENSG00000069849	ATP1B3	57.350882	56.684851	65.247003	42.939499	47.949756	45.705105	50.007448	57.747611	60.076161	0.002174337	-0.230561992	0.953342569	-0.006158829
ENSG00000007923	DNAJC11	23.784185	20.977528	22.001402	20.614574	18.210175	20.490809	19.280546	19.392332	22.023424	0.781743957	-0.026841859	0.947389707	-0.006148663
ENSG00000170275	CRTAP	4.088011	4.086656	3.90224	4.041511	3.792383	3.725591	3.619593	3.587547	4.081381	0.444541781	0.083244811	0.959889611	-0.006140222
ENSG00000137504	CREBZF	11.734862	13.29945	13.072287	13.111063	12.55467	12.194945	12.558516	10.72339	12.963833	0.104544	0.140527688	0.958190472	-0.006113585
ENSG00000087157	PGS1	8.909622	9.460974	8.812255	7.624027	6.983871	8.018966	6.812249	8.386998	8.334548	0.221538451	-0.162114207	0.970131496	-0.005924923
ENSG00000134779	TPGS2	13.245729	14.874983	14.725017	11.413605	10.604902	10.432946	13.499486	12.410084	14.086779	0.014848544	-0.211982023	0.956673647	-0.005919802
ENSG00000091527	CDV3	101.093151	107.694919	110.216887	116.001712	105.042858	112.790941	98.74519	98.053034	95.75695	0.001043625	0.17488639	0.938112505	-0.005866073
ENSG00000182923	CEP63	6.524397	5.445505	5.584034	6.185263	4.750888	7.243253	5.422548	7.793759	7.263117	0.638351631	-0.072207984	0.96965676	-0.005798589
ENSG00000124802	EEF1E1	66.553882	71.528071	72.704462	54.61878	55.825951	51.966003	65.054872	66.251161	67.395745	0.000970466	-0.239716663	0.95279846	-0.005666226
ENSG00000119242	CCDC92	16.15012	15.917683	16.274077	12.489938	12.852482	13.042613	16.553573	15.552222	16.881808	0.00198678	-0.236475153	0.958241991	-0.005251117
ENSG00000172465	TCEAL1	6.492579	5.191903	5.674956	6.971865	6.360607	7.049818	5.955576	4.71834	5.425132	0.015359385	0.394017809	0.982419088	-0.005243313
ENSG00000102805	CLN5	4.387027	4.025582	4.791189	3.994548	4.03035	4.287082	4.004462	4.042309	4.757321	0.069055472	-0.217423033	0.96988122	-0.005127895
ENSG00000179021	C3orf38	7.336211	8.73811	8.780116	8.326944	9.599649	9.109021	8.192634	7.318391	7.493062	0.017534116	0.269292025	0.972789285	-0.005104757
ENSG00000129667	RHBDF2	9.534242	9.644063	9.331096	8.837461	7.884933	9.016977	8.364426	7.975208	10.676874	0.630990042	-0.065238477	0.970991299	-0.004936011
ENSG00000165156	ZHX1	18.552728	20.337151	19.858252	24.032364	23.603213	23.404199	18.869171	19.42918	18.575517	1.78E-12	0.418803846	0.959583891	-0.004933929
ENSG00000138032	PPM1B	18.692416	19.033695	20.418848	18.314109	18.598796	18.435884	19.712076	17.238542	20.412112	0.39727772	0.073385655	0.963407501	-0.004920966
ENSG00000168827	GFM1	30.053282	33.396999	33.368902	31.054499	30.1071	29.286596	28.926097	29.43445	30.511026	0.319879873	0.076534599	0.959889611	-0.004685175
ENSG00000000419	DPM1	45.577718	47.085195	53.226592	49.931626	45.564142	49.020376	44.170667	44.760255	46.942933	0.130649937	0.134032033	0.966259003	-0.00464846
ENSG00000181523	SGSH	10.004914	11.799667	10.134308	10.799823	9.835315	11.22401	9.635655	8.065703	11.593408	0.245838001	0.128935139	0.977616642	-0.004441727
ENSG00000135452	TSPAN31	9.653336	11.039477	8.709057	7.356569	8.085053	8.492104	10.12853	9.344318	9.947511	0.217010806	-0.170560823	0.977793508	-0.00435663
ENSG00000167460	TPM4	126.177846	134.920256	143.100136	75.232492	73.871496	73.129951	123.479868	117.197446	133.257755	5.37E-37	-0.709170993	0.96128358	-0.004345559
ENSG00000183474	GTF2H2C	10.159459	10.132158	11.097604	9.360507	9.433156	10.296842	10.797884	9.506233	11.234534	0.667335158	-0.057831823	0.974751225	-0.004314561
ENSG00000104973	MED25	11.730696	12.655325	11.522653	9.73813	8.985684	10.456071	9.792235	11.349309	12.599269	0.165783663	-0.150166529	0.976333789	-0.004169593
ENSG00000132563	REEP2	0.599217	0.698107	0.511486	0.256158	0.273449	0.2655	0.542523	0.342452	0.821319	0.042642708	-0.906735002	0.993912047	-0.004157909
ENSG00000135220	UGT2A3	4.952423	4.202878	3.072108	3.208642	3.214027	3.0601	3.773344	4.009102	3.643718	0.252161379	-0.221455663	0.985556615	-0.004156576
ENSG00000087008	ACOX3	9.246119	8.854462	9.303568	8.838304	7.638172	9.485308	8.468044	8.235436	9.74417	0.863986127	0.025662626	0.974534926	-0.004102092
ENSG00000181038	METTL23	30.082095	29.868257	31.401377	30.708388	30.185649	31.206287	27.872926	28.550217	26.019632	0.116998218	0.150227238	0.974534926	-0.003984641
ENSG00000142046	TMEM91	0.787275	1.405958	1.346799	1.101751	0.845724	0.907698	1.285196	1.207767	1.181289	0.501970262	-0.293032566	0.993316365	-0.003969779
ENSG00000196510	ANAPC7	35.028305	34.864415	34.805862	28.74017	27.95182	26.851529	31.269156	31.832565	33.182656	0.006042991	-0.167465024	0.962348493	-0.003919928
ENSG00000143079	CTTNBP2NL	1.643607	1.993108	1.749356	1.628293	1.449585	1.620841	1.326496	1.659718	2.063701	0.727758409	-0.066378985	0.986052647	-0.003900213
ENSG00000092853	CLSPN	7.629491	8.22099	8.057304	7.439907	7.159078	7.286698	7.513234	7.073301	8.191484	0.855776162	0.021915021	0.972805713	-0.003864249
ENSG00000171848	RRM2	23.858846	25.662469	26.999145	20.700262	18.21503	20.243339	24.797958	22.545618	24.316517	0.015931392	-0.199340332	0.972199172	-0.003812159
ENSG00000140995	DEF8	11.037438	9.858547	9.791762	10.049419	9.181621	9.897779	9.245697	9.842216	10.984065	0.120695971	0.158657575	0.977325455	-0.003714308
ENSG00000113456	RAD1	11.773254	11.469627	15.652732	14.786099	12.752026	14.987112	11.635266	12.16021	12.904639	0.000110295	0.345113283	0.977749087	-0.003693067
ENSG00000188917	TRMT2B	3.32167	3.122206	2.481623	2.9526	2.1323	2.741273	2.569293	3.091672	2.645048	0.832431415	-0.051761297	0.986971729	-0.003582175
ENSG00000263001	GTF2I	77.631233	78.097784	82.102864	68.597921	65.304732	65.780145	71.570722	70.543694	80.851558	0.054890956	-0.104321797	0.965589123	-0.003501957
ENSG00000154065	ANKRD29	0.123485	0.870687	0.623546	0.997345	1.051462	0.855877	0.851166	0.766898	0.56566	0.355858472	0.514882554	0.996067567	-0.003357083
ENSG00000106069	CHN2	24.45184	23.505122	20.702284	23.189691	23.608633	15.600032	22.427903	20.260738	21.853782	0.984331945	0.007306311	0.976257956	-0.003213553
ENSG00000170860	LSM3	19.658813	20.692495	21.182526	18.697096	18.173462	17.903795	19.066416	20.273989	18.121035	0.826252675	-0.020534029	0.977832997	-0.00309813
ENSG00000125971	DYNLRB1	87.049573	87.631862	93.543994	74.797802	82.820993	80.042514	83.864502	86.885976	80.108382	0.797950476	-0.027701033	0.979867257	-0.002821264
ENSG00000123240	OPTN	7.234102	6.983549	6.958058	20.516639	21.776177	22.556581	7.2485	6.520611	6.664368	5.71E-133	1.76951453	0.984072268	-0.002686724
ENSG00000170322	NFRKB	8.649494	8.150236	8.240004	7.980888	7.533698	7.358649	8.201468	7.273874	8.757046	0.965839989	0.005438768	0.982862391	-0.002632316
ENSG00000166908	PIP4K2C	24.121175	22.556422	23.411533	22.589215	24.425905	23.124045	21.883697	21.250058	22.50584	0.071764662	0.140143965	0.978645714	-0.002565028
ENSG00000137073	UBAP2	26.501065	25.427031	25.357807	21.933615	20.687891	22.634032	23.858331	22.459519	23.621509	0.555819624	-0.046356262	0.976421974	-0.002517954
ENSG00000115073	ACTR1B	31.974583	31.055178	27.8218	23.146502	23.873589	24.8475	27.266453	27.389671	30.492661	0.020521058	-0.190465825	0.983110138	-0.002333354
ENSG00000153404	PLEKHG4B	0.723921	0.981202	0.801362	1.103918	0.853262	0.89998	0.673839	0.694533	0.798366	0.001782064	0.479631738	0.991357307	-0.00228246
ENSG00000105186	ANKRD27	8.071185	8.225548	8.433533	8.629577	7.882282	7.530599	7.378802	7.302538	8.563111	0.30404041	0.100170687	0.985491423	-0.00221275
ENSG00000102796	DHRS12	1.644628	1.130407	1.206648	1.071222	0.967398	0.738234	1.282126	1.187423	1.416601	0.342867814	-0.374424791	0.995895962	-0.002198369
ENSG00000130382	MLLT1	25.744101	27.979845	27.625416	27.794117	26.679981	28.846722	25.107725	22.910696	28.302959	0.003987001	0.182366524	0.983606271	-0.002112075
ENSG00000126653	NSRP1	10.564431	11.214437	14.154017	13.864082	12.822345	12.620116	10.849155	9.992807	11.185547	0.002499071	0.32311758	0.988507632	-0.002091014
ENSG00000119787	ATL2	26.126368	28.037974	30.402738	25.3447	25.410713	23.997701	27.444283	24.229209	26.622943	0.687623939	-0.042188171	0.986052647	-0.002007122

Gene ID	Gene name	Ctrl-1 FPKM	Ctrl-2 FPKM	Ctrl-3 FPKM	IFNγ-1 FPKM	IFNγ-2 FPKM	IFNγ-3 FPKM	Sora-1 FPKM	Sora-2 FPKM	Sora-3 FPKM	Ctrl vs IFNγ DESeq2 FDR	Ctrl vs IFNγ DESeq2 log2FC	Ctrl vs Sora DESeq2 FDR	Ctrl vs Sora DESeq2 log2FC
ENSG00000136068	FLNB	22.053709	22.06692	19.810166	16.432202	16.032371	15.947827	20.267222	18.342335	21.238152	0.0000523	-0.244911011	0.983606271	-0.00190607
ENSG00000163812	ZDHHC3	17.590802	18.45217	18.453553	18.738666	17.484711	18.1295	16.940181	16.523624	17.955805	0.290906482	0.089048196	0.985956969	-0.001838234
ENSG00000144451	SPAG16	6.011047	8.530171	7.331069	7.01864	5.64688	5.52858	7.134889	6.952853	6.813294	0.491504028	-0.146340664	0.993677883	-0.00180333
ENSG00000069424	KCNAB2	6.117758	7.470023	6.693437	4.669335	4.138809	4.61584	7.118253	5.378495	7.397442	0.00000628	-0.423932709	0.990110176	-0.001782425
ENSG00000105939	ZC3HAV1	4.677188	5.014498	5.013659	15.645189	13.114951	13.48063	5.029046	4.240361	5.087566	1.27E-118	1.621127542	0.988218114	-0.001771549
ENSG00000152409	JMY	1.381458	1.416902	1.538398	1.837267	1.803624	1.619438	1.453707	1.247087	1.363457	0.00023391	0.424443364	0.99188037	-0.001755377
ENSG00000140939	NOL3	3.680139	2.935412	2.581766	2.109001	2.182535	2.232557	2.739159	2.760951	2.862388	0.174296573	-0.330531924	0.995462045	-0.00168747
ENSG00000240771	ARHGEF25	4.486541	4.763201	4.312194	3.688873	3.863027	3.675836	4.525191	4.214856	4.061246	0.305745977	-0.159403606	0.99274421	-0.001664854
ENSG00000029725	RABEP1	21.632601	21.784405	21.779693	20.630263	18.308444	18.473129	20.107604	19.818185	21.026399	0.706849807	-0.03111032	0.985012444	-0.001636005
ENSG00000169045	HNRNPH1	200.439504	182.450721	194.046607	217.041345	184.739293	183.163318	205.925564	195.58819	177.829891	0.131774576	0.095972651	0.985491423	-0.001576763
ENSG00000103222	ABCC1	8.101016	7.83802	7.998009	5.179638	4.75958	5.697842	7.346511	7.175046	7.87474	6.26E-09	-0.463440879	0.98736304	-0.001574036
ENSG00000129911	KLF16	11.649723	13.171506	13.073919	11.078744	12.24954	13.122374	12.045459	11.998432	11.412449	0.412936565	0.094826543	0.990303523	-0.001527657
ENSG00000006712	PAF1	27.129483	26.013175	27.59425	27.613093	25.724419	26.630724	25.496959	23.807175	26.557645	0.082945845	0.131485435	0.988507632	-0.001441431
ENSG00000104738	MCM4	78.808951	77.97387	75.028774	71.369647	68.260397	72.276696	72.212376	69.694773	76.940302	0.817538949	0.017218545	0.987093558	-0.001233951
ENSG00000119782	FKBP1B	2.028608	2.82808	2.638108	1.842644	2.483563	1.786972	2.262363	2.312675	2.346637	0.685443728	-0.153943077	0.997268893	-0.001228454
NewGene_1292	--	0.990952	0.646013	0.818627	1.097451	0.946249	0.649301	0.85418	0.641993	1.039151	0.79330056	-0.134901513	0.998207316	-0.001222712
ENSG00000165185	KIAA1958	3.67662	3.414972	3.476209	3.626877	3.380065	3.382983	3.352472	3.333112	3.570512	0.657635938	0.047067115	0.991528208	-0.00120793
ENSG00000152642	GPD1L	8.882089	9.135683	10.627671	7.747509	7.007988	7.098899	8.791824	8.834659	9.174541	0.012205046	-0.243764556	0.992860636	-0.001186045
ENSG00000153832	FBXO36	1.728896	1.866943	1.917694	1.674334	1.400938	1.820261	1.74963	1.7248	1.610791	0.994863922	-0.002148057	0.99652183	-0.001027486
ENSG00000204267	TAP2	2.396719	2.458566	2.698593	97.930323	91.317261	95.079298	2.420964	2.119663	2.48479	0	5.388549805	0.995537159	-0.000955531
ENSG00000085872	CHERP	27.579784	25.101919	25.569702	24.87527	22.703899	25.099776	23.687514	21.797377	28.125968	0.649106635	0.0408181	0.993677883	-0.000941143
ENSG00000111907	TPD52L1	23.67515	18.875583	27.252374	12.738277	14.685118	11.185768	15.995252	19.045695	21.626818	4.17E-14	-0.701874703	0.996113907	-0.000639606
NewGene_1581	--	5.195014	5.255493	4.701218	5.286943	4.682839	3.639701	5.000877	4.925532	6.448552	0.261848449	-0.235844975	0.998299159	-0.00056308
ENSG00000119138	KLF9	1.785956	1.666824	1.578183	1.728479	1.715188	1.710206	1.627375	1.45246	1.638871	0.259530776	0.181812208	0.998030053	-0.000549035
ENSG00000170515	PA2G4	98.572521	97.930838	102.157038	93.681714	89.438905	92.178992	91.913063	94.107281	96.951746	0.676213607	0.028643088	0.99488621	-0.000546785
ENSG00000131724	IL13RA1	17.742177	18.447616	19.545481	12.372253	11.672901	12.390483	17.157487	16.436159	18.79888	1.38E-12	-0.467386674	0.99618878	-0.000475286
ENSG00000203896	LIME1	1.329964	3.53889	0.373284	0.436318	1.318713	0.961064	1.051096	3.614934	0.702715	0.440699196	-1.010298075	0.999844393	-0.000327051
ENSG00000176105	YES1	14.984973	18.488319	19.845154	21.393164	20.919188	21.425278	17.551318	14.84341	17.695363	0.00000293	0.408896121	0.998372516	-0.000307463
ENSG00000197324	LRP10	10.523148	9.121998	8.905327	8.656981	9.745808	9.541224	8.213145	8.336589	10.312918	0.233412422	0.118545176	0.999009787	-0.00018874
ENSG00000047578	KIAA0556	3.906363	3.527645	3.389142	3.039108	2.74769	2.595523	3.522304	2.961112	3.78964	0.072704831	-0.194550523	0.999213296	-0.000178216
ENSG00000214706	IFRD2	26.49891	29.103604	28.308825	20.740406	21.595126	23.069768	25.333374	23.706892	28.839081	0.016073965	-0.197359504	0.999009787	-0.000169076
ENSG00000117266	CDK18	0.814147	0.770022	0.562469	0.54417	0.371787	0.566187	0.588129	0.61066	0.594649	0.303344509	-0.349878862	0.999690265	-0.000159452
ENSG00000025772	TOMM34	20.723026	23.212765	22.314779	16.857059	14.236567	15.831726	20.881668	19.851023	21.404814	0.0000366	-0.350392053	0.999587188	-0.0000807
ENSG00000196943	NOP9	8.490894	8.972988	8.343624	8.666052	8.800676	8.339007	8.116748	7.739266	8.371437	0.060701504	0.146234635	0.999690265	-0.0000519
ENSG00000151657	KIN	2.496702	3.047291	3.125064	2.900927	2.781193	2.78752	2.769613	2.391079	2.950238	0.421553341	0.111553121	0.999923559	-0.0000106
ENSG00000140350	ANP32A	87.602019	87.407287	90.60325	104.548318	98.056609	99.946304	80.954808	84.910223	83.553488	3.81E-12	0.334873155	0.999844393	0.00002
ENSG00000150760	DOCK1	9.177324	8.561583	8.838411	7.42101	6.850166	7.030903	8.072297	7.720742	9.521086	0.02739501	-0.170004263	0.999534418	0.0000903
ENSG00000106484	MEST	51.360802	54.951798	58.139878	43.450389	40.803409	42.482213	50.910599	49.773355	53.225277	0.000418005	-0.225775737	0.999025993	0.00012983
ENSG00000100632	ERH	82.660099	84.742507	91.042791	99.237099	104.834845	105.294621	85.096493	92.202844	64.161594	1.25E-10	0.405664482	0.999599007	0.000185575
ENSG00000143924	EML4	111.391049	110.855903	110.801023	97.577103	94.38589	94.349465	103.043579	98.128673	111.97236	0.158039748	-0.07143078	0.998207316	0.000190319
ENSG00000171552	BCL2L1	116.064842	113.935952	116.666205	83.808206	88.108894	86.463774	106.717304	106.812045	112.746739	0.00000016	-0.282447276	0.996455164	0.000334046
ENSG00000160679	CHTOP	66.403652	65.106026	66.081754	59.099354	58.819176	57.636181	63.059859	60.061054	64.686315	0.898702459	-0.010645269	0.995743621	0.000456896
ENSG00000151725	CENPU	34.210576	37.069922	37.533757	34.015738	29.384057	33.079567	34.659873	32.987759	34.729765	0.867338515	-0.018241417	0.996059533	0.000495865
ENSG00000198873	GRK5	2.590139	2.038598	2.048641	0.921394	1.407254	0.880846	1.807854	2.004943	2.48141	0.0000788	-0.908536683	0.998600672	0.000497788
ENSG00000079999	KEAP1	32.014998	31.316065	29.848436	26.862864	28.009375	27.176126	27.688838	28.523412	30.025373	0.986348802	-0.001749019	0.995776787	0.000527662
ENSG00000137720	C11orf1	3.009977	2.753496	2.550875	2.656391	1.254858	2.814028	2.776712	3.322483	1.823248	0.824036023	-0.073650734	0.998372516	0.000712104
ENSG00000119414	PPP6C	20.645713	22.168413	22.990852	20.881861	21.264652	21.469525	20.356621	20.240716	21.033212	0.163649783	0.105083658	0.994481835	0.000718074
ENSG00000174373	RALGAPA1	2.956749	3.085238	2.854759	2.719021	2.263459	2.345775	2.871858	2.68249	2.882831	0.236313502	-0.139254669	0.995558131	0.000763534
ENSG00000103852	TTC23	5.07948	4.612499	4.909604	5.247576	3.841684	4.248218	4.324551	4.449994	4.829502	0.706096496	-0.061590403	0.996067567	0.000785823
ENSG00000135336	ORC3	14.881201	15.13203	15.071148	13.072483	12.077503	12.475008	14.753013	13.928584	13.947095	0.289618152	-0.0993638	0.994494634	0.00084559
ENSG00000130299	GTPBP3	10.015199	10.574666	12.519536	9.871385	8.809536	9.333939	9.801321	9.472564	11.831832	0.310173129	-0.124523399	0.995462045	0.000913238
ENSG00000054267	ARID4B	10.451616	11.568827	12.132327	12.606187	11.162116	11.475353	11.475206	10.078557	10.527089	0.040671672	0.17833234	0.994022505	0.000921856
ENSG00000125447	GGA3	15.685776	13.842055	13.828993	14.075544	12.457433	12.948441	13.063931	12.579922	14.771533	0.960281562	-0.006422282	0.993466369	0.001026818
ENSG00000148057	IDNK	7.053326	8.001347	7.015192	10.14989	10.396369	10.643422	8.15124	6.404389	7.673801	0.000094	0.560758023	0.996212992	0.001049435
ENSG00000079150	FKBP7	4.627895	4.161326	4.194386	3.469293	3.580024	3.256445	4.476526	5.060019	4.37122	0.184810289	-0.213219694	0.995537159	0.001106564
ENSG00000108797	CNTNAP1	0.983913	0.792588	0.959757	0.814718	0.856555	0.863675	0.950167	0.805495	0.812976	0.902061001	0.035639518	0.996212992	0.001215861
ENSG00000173821	RNF213	4.251131	3.62711	3.358242	16.158181	14.929964	16.290767	3.914206	3.277338	4.43323	1.72E-243	2.193548941	0.992299713	0.001230267
ENSG00000132670	PTPRA	19.531028	18.567727	16.28716	15.792999	14.274205	13.773113	16.868162	16.812618	17.312453	0.0728741	-0.144890286	0.990165548	0.001309865
ENSG00000198793	MTOR	16.429525	16.086697	16.430852	14.358962	13.79175	13.707789	15.37386	14.1262	16.924526	0.123845979	-0.093485662	0.987266185	0.001410878
ENSG00000175832	ETV4	31.984757	30.147925	33.579664	23.462693	25.137532	26.383799	25.909821	29.999847	31.769068	0.001043774	-0.241343891	0.989971927	0.00143322
ENSG00000142634	EFHD2	8.428717	8.285192	7.926896	7.555434	6.996346	7.832092	7.322593	7.034608	8.818152	0.963951837	0.007450579	0.992144292	0.001499697
ENSG00000169710	FASN	121.64767	113.883829	100.666324	162.544742	152.317387	166.786413	99.907953	97.899452	121.619478	4.07E-25	0.66195759	0.988986888	0.001559811
ENSG00000152484	USP12	10.010888	10.286521	10.45326	14.030035	12.670736	11.663566	11.138436	9.397707	9.680008	8.51E-10	0.464845381	0.989571299	0.001612645
ENSG00000166164	BRD7	17.085979	17.066198	16.71569	16.326582	13.775818	14.92072	15.427549	15.043183	17.488202	0.868340752	0.015652443	0.985896153	0.001662148
ENSG00000163512	AZI2	10.596243	10.569903	9.913673	9.315994	9.012048	9.776633	10.666075	10.496599	11.877038	0.8438603	0.025173758	0.986971729	0.001901333
ENSG00000117625	RCOR3	13.951709	14.166244	15.141403	13.852981	13.483203	12.617281	13.942821	13.602916	14.878901	0.406305596	0.087019242	0.986971729	0.001902654
ENSG00000039123	MTREX	18.323526	17.818784	18.780678	18.371772	16.937721	16.433182	18.030515	16.606664	17.2429	0.558546497	0.050349678	0.983975935	0.00195456
ENSG00000180953	ST20	7.643193	8.60143	9.44614	9.13548	8.511946	5.791181	9.337848	8.324076	9.889155	0.86014393	-0.051458102	0.993466369	0.002016731
ENSG00000184785	SMIM10	2.23157	2.177066	2.286865	1.922011	1.900793	2.55025	2.792269	2.154062	1.304762	0.793478382	0.084328725	0.995558131	0.002149804
ENSG00000139496	NUP58	20.897413	24.683929	25.521188	20.751176	19.287982	19.657462	21.59028	20.052877	24.162771	0.250653398	-0.103980481	0.985491423	0.002169395
ENSG00000134463	ECHDC3	17.720322	17.705432	16.265846	16.410458	17.983853	18.002071	15.24434	16.405708	17.220484	0.098934023	0.167475963	0.986052647	0.002272399
ENSG00000115255	REEP6	10.072786	10.264474	6.84599	7.102392	6.535706	7.379687	7.291701	7.990053	7.64244	0.754728526	-0.065421227	0.991193478	0.002303088
ENSG00000054116	TRAPPC3	30.56294	29.864881	26.340862	26.456885	25.214644	25.93482	26.445824	27.4604	28.848047	0.872045495	-0.021027372	0.985491423	0.002306056
ENSG00000104221	BRF2	7.129829	6.720611	6.96506	5.432479	4.757535	5.715915	6.243229	6.852812	6.399505	0.048386646	-0.241955449	0.987093558	0.002433666

Gene ID	Gene name	Ctrl-1 FPKM	Ctrl-2 FPKM	Ctrl-3 FPKM	IFNγ-1 FPKM	IFNγ-2 FPKM	IFNγ-3 FPKM	Sora-1 FPKM	Sora-2 FPKM	Sora-3 FPKM	Ctrl vs IFNγ DESeq2 FDR	Ctrl vs IFNγ DESeq2 log2FC	Ctrl vs Sora DESeq2 FDR	Ctrl vs Sora DESeq2 log2FC
ENSG00000119686	FLVCR2	2.071499	2.559343	2.032337	3.321153	3.452537	3.261813	2.097902	2.130948	2.016569	5.24E-08	0.736342828	0.991102551	0.002455401
ENSG00000156510	HKDC1	3.57351	3.201865	2.868703	1.366805	1.214609	1.193739	3.017022	3.045204	2.991991	1.61E-16	-1.204866294	0.988986888	0.00248709
ENSG00000146670	CDCA5	36.218525	36.696089	36.398232	30.659779	27.106998	29.266099	35.855272	32.020294	34.844774	0.005139234	-0.186086845	0.977996715	0.002496879
ENSG00000103460	TOX3	18.354051	16.620518	18.823414	18.946915	18.843856	18.125816	16.521257	15.512554	18.840172	0.011100835	0.19770827	0.982454875	0.002576367
ENSG00000167930	FAM234A	28.17786	22.679633	17.079885	17.71191	14.024392	20.631894	18.462536	23.068093	18.750354	0.454727039	-0.257172097	0.993912047	0.002679673
ENSG00000164300	SERINC5	7.577591	7.141054	7.082572	8.54676	9.059065	7.566719	6.89996	6.335504	7.298536	0.000024	0.353137137	0.979968848	0.002716887
ENSG00000165914	TTC7B	0.997144	1.001635	0.860508	0.57387	0.755996	0.996684	0.804439	0.795975	0.985481	0.04934451	-0.26707032	0.985902221	0.002752293
ENSG00000139505	MTMR6	4.210948	4.788811	4.929244	4.862475	4.61847	4.341034	4.547832	3.928524	4.626898	0.22557956	0.135608621	0.984072268	0.00279765
ENSG00000109016	DHRS7B	11.218556	10.149325	8.628109	6.607606	6.64338	6.640498	7.811016	7.940415	8.26277	0.050734903	-0.22968485	0.983978371	0.002958321
ENSG00000154144	TBRG1	4.494562	5.001445	5.176771	5.339578	4.662981	4.745236	4.674162	4.594539	4.393139	0.269251634	0.131029186	0.983670489	0.002997154
ENSG00000183048	SLC25A10	33.57507	30.809527	28.101797	30.976087	26.561969	32.667718	29.073255	27.561415	29.414971	0.366604236	0.096548976	0.978754802	0.003046147
ENSG00000196504	PRPF40A	33.240428	30.794793	34.482796	33.692556	32.091575	31.789363	30.218725	33.092758	28.387159	0.04200487	0.134264077	0.973100485	0.003077463
ENSG00000213337	ANKRD39	5.632953	6.142252	5.067096	6.086133	6.508491	6.061143	5.09437	4.775035	5.128309	0.017975051	0.342121631	0.98658697	0.003229325
ENSG00000148702	HABP2	10.860014	9.721882	10.763701	52.581705	53.897272	51.872572	9.033801	9.4313	10.972781	0	2.480374733	0.980486154	0.003259297
ENSG00000147144	CCDC120	2.442511	2.382403	2.617214	2.243338	1.795015	2.340491	2.569097	2.140203	2.30233	0.725192875	-0.072975232	0.986088347	0.003379527
ENSG00000140263	SORD	22.142232	21.209806	18.890517	21.121924	22.225745	21.386496	19.209941	19.477394	28.494323	0.036756608	0.161404063	0.974301982	0.003407464
ENSG00000237649	KIFC1	46.756965	48.346111	47.772901	33.783284	30.907143	34.936594	44.681334	45.635387	45.452456	1.93E-10	-0.386911905	0.968040231	0.003555567
ENSG00000111684	LPCAT3	31.408612	29.660178	29.959249	65.472674	62.132538	64.237578	28.225364	29.291458	29.011865	1.79E-114	1.219655313	0.970866974	0.003570463
ENSG00000081923	ATP8B1	3.25427	2.981556	2.781807	4.694456	4.511685	4.511188	3.03472	2.676344	2.854197	8.48E-16	0.757970539	0.982405494	0.003619645
NewGene_2279	--	2.183594	2.03358	2.454776	2.532328	1.695203	2.240964	1.955859	1.872898	2.208269	0.122467669	0.195136843	0.982419088	0.003634555
ENSG00000213085	CFAP45	0.75457	0.341955	0.442641	0.564798	0.518698	0.567242	0.541508	0.532422	0.388865	0.678068282	0.236117174	0.995389926	0.003719
ENSG00000104856	RELB	8.688083	8.395624	8.300192	2.906561	3.014321	3.168782	7.870082	7.621851	8.505684	4.12E-31	-1.319712619	0.978230505	0.00373651
ENSG00000106263	EIF3B	237.572022	233.837585	231.332827	224.523706	218.797794	221.530895	212.546743	210.976566	238.294265	0.220713173	0.063379919	0.960206127	0.003743513
ENSG00000161021	MAML1	9.634973	9.450222	9.465085	9.691791	8.99553	9.423829	8.787729	8.35759	9.734541	0.096353186	0.124801551	0.969546493	0.003844269
ENSG00000158955	WNT9B	0.878649	0.567876	0.82285	0.083914	0.140138	0.125034	0.496363	0.775529	0.937641	--	--	0.994494634	0.003846631
ENSG00000138107	ACTR1A	33.049545	31.342824	30.308737	25.47172	25.793274	25.723095	28.997788	30.583271	29.365492	0.030745899	-0.15047371	0.968787285	0.00389499
ENSG00000127511	SIN3B	10.684532	11.085009	10.603092	9.948285	8.371112	8.803224	9.679235	9.142633	11.027241	0.281679481	-0.100283228	0.972805713	0.003913789
ENSG00000198108	CHSY3	1.023025	1.07034	1.184323	0.625016	0.810213	0.654385	0.744416	1.033351	1.314988	0.034526676	-0.504223866	0.989971927	0.003926946
ENSG00000204257	HLA-DMA	1.782173	1.379432	1.143489	0.950373	1.148668	1.121182	0.896814	1.633541	1.369878	0.645824668	-0.213375147	0.993752965	0.003926971
ENSG00000004766	VPS50	7.815955	8.968241	9.069754	7.726908	8.61519	7.88747	8.341719	8.519502	8.689233	0.852852608	0.024991654	0.972805713	0.003963462
ENSG00000132164	SLC6A11	8.179878	8.48304	7.838901	7.949992	7.276563	6.905038	7.528112	7.168438	7.719184	0.995031881	-0.000977609	0.970183927	0.004252607
ENSG00000018699	TTC27	10.229173	10.492039	11.084689	8.549569	7.469529	8.177005	9.211159	10.418997	10.29582	0.008494616	-0.249042503	0.972030203	0.004486174
ENSG00000169714	CNBP	300.958605	313.352621	322.415636	325.709282	320.669392	321.586567	295.660641	286.212455	302.775218	0.0000416	0.196384217	0.950124417	0.004503715
ENSG00000104381	GDAP1	2.300179	2.326519	2.175147	2.78568	2.570035	2.224249	2.181616	1.963055	2.124181	0.035136556	0.327467479	0.982069069	0.004537548
ENSG00000110651	CD81	76.42076	72.179644	72.018994	63.445582	61.366815	68.621154	66.386642	63.764324	76.662704	0.599815009	-0.04630275	0.961674004	0.004656011
ENSG00000168944	CEP120	1.982291	2.356345	2.428688	1.778437	1.641875	1.397421	2.146762	1.963364	2.034591	0.056152589	-0.302499593	0.979511226	0.004696568
ENSG00000174851	YIF1A	113.162525	110.394857	105.963928	117.417539	118.595763	122.525569	103.116902	102.131344	102.748899	0.00000541	0.266453137	0.955495263	0.004803299
ENSG00000014164	ZC3H3	7.063065	7.059857	6.162988	9.084742	7.523691	8.913871	6.850826	6.085028	6.232828	0.00000272	0.471526115	0.972648865	0.004877581
ENSG00000225830	ERCC6	1.894475	2.142729	1.875981	1.563282	1.426739	1.788125	1.425719	1.603214	2.118284	0.618563614	-0.085743347	0.980128878	0.004935581
ENSG00000167971	CASKIN1	0.489751	0.603368	0.469334	0.450021	0.378177	0.409799	0.580852	0.407091	0.483384	0.566632786	-0.18261704	0.987562807	0.005052463
ENSG00000269335	IKBKG	5.891048	4.942846	4.491223	6.588499	5.005528	5.601598	4.710836	4.442721	5.146639	0.050891948	0.326726197	0.980463559	0.005087406
ENSG00000147592	LACTB2	11.698628	11.761227	10.136724	8.845681	10.020663	9.657453	9.598115	9.825253	11.168618	0.272089038	-0.150401938	0.97400055	0.005137956
NewGene_2950	--	3.004848	3.235876	2.760753	2.926766	3.417378	2.9701	3.024729	2.755011	2.797326	0.202998735	0.179454259	0.97377102	0.005244231
ENSG00000178764	ZHX2	11.708044	9.185236	8.310477	5.348577	5.883787	5.567101	8.96989	8.781392	9.698149	1.18E-08	-0.648347299	0.972027544	0.005355599
ENSG00000104044	OCA2	4.971245	4.831365	5.248857	1.972967	2.365529	2.030809	4.577524	4.690625	4.934783	6.36E-19	-1.101873377	0.970183927	0.005452558
ENSG00000135999	EPC2	13.369156	14.117198	16.071441	13.610676	13.2255	13.151946	14.072825	12.378708	15.180149	0.980096373	0.003339151	0.961932643	0.005554414
ENSG00000100625	SIX4	2.061053	2.426467	2.480004	1.765327	2.169955	1.967935	2.354391	2.02407	2.271703	0.709198922	-0.075382494	0.975850485	0.005582092
ENSG00000102996	MMP15	9.237205	8.639924	7.065588	6.565406	6.748908	7.381464	7.394397	7.211857	8.864984	0.362759186	-0.119462865	0.969669876	0.005670363
ENSG00000091583	APOH	587.797487	552.258553	500.269276	677.958205	704.320552	656.207877	519.673714	532.573804	489.383723	9.18E-14	0.46113427	0.955495263	0.005692593
ENSG00000149742	SLC22A9	19.288682	18.717376	15.863869	17.52245	17.012024	16.524728	17.122836	16.736828	16.839786	0.550878579	0.070279901	0.963221275	0.005762256
ENSG00000164828	SUN1	50.000075	46.484242	47.582452	43.72897	39.967112	39.299673	45.666918	41.720743	49.050048	0.05414145	-0.128477322	0.943602371	0.005789759
ENSG00000143493	INTS7	15.573042	19.484245	15.61856	14.198112	15.852561	13.678373	15.248727	14.163896	15.067454	0.706004316	-0.035775417	0.950597841	0.005854948
ENSG00000183580	FBXL7	0.211789	0.260162	0.24168	0.374229	0.221973	0.331744	0.320671	0.156843	0.1978	0.296406504	0.444533382	0.991381214	0.006013659
ENSG00000159496	RGL4	1.875184	1.761311	1.680052	1.134634	1.273799	1.004922	2.176241	2.046234	1.52853	8.8E-10	-1.137612789	0.985491423	0.006027501
ENSG00000177084	POLE	18.10348	18.086173	18.073994	17.691435	14.850304	15.268528	16.373729	15.50563	19.312908	0.701877516	-0.035622304	0.94841576	0.006069602
ENSG00000213906	LTB4R2	1.530972	1.693743	2.388957	2.022746	1.906688	1.758263	1.696111	1.675381	1.740073	0.519902479	0.155093905	0.98063789	0.006198596
ENSG00000087338	GMCL1	8.502039	10.121955	10.795449	9.384132	9.438163	8.674744	8.959875	8.581531	10.20708	0.704751069	0.05009537	0.962000402	0.006317671
ENSG00000176896	TCEANC	0.634412	0.727777	0.656875	0.729155	0.778498	0.375711	0.640902	0.478234	0.727232	0.800916816	-0.113893543	0.986971729	0.006373803
ENSG00000172292	CERS6	11.782605	11.431033	11.335878	9.093422	9.101929	8.933141	10.458079	10.619986	11.586033	0.001039587	-0.20696952	0.941592133	0.006414331
ENSG00000137133	HINT2	30.49301	31.859097	30.0965	30.933895	33.205124	31.806801	28.448917	30.577751	27.847845	0.061037342	0.201455244	0.963407501	0.006520209
ENSG00000163631	ALB	8546.066178	7842.683497	7087.911742	7516.318352	7815.650944	7378.693381	7438.89854	7380.566811	7304.561898	0.195969435	0.100277821	0.944041721	0.006563428
ENSG00000163995	ABLIM2	3.806385	3.098365	3.414527	1.166596	1.00764	1.065127	3.205352	2.708094	3.574168	9.17E-20	-1.476150281	0.972805713	0.006565259
ENSG00000123415	SMUG1	17.409502	20.055215	13.740163	13.961997	14.176608	15.6434	13.49513	16.101252	17.874371	0.774947821	-0.045693165	0.968040231	0.006601166
ENSG00000147400	CETN2	32.287849	33.278263	32.268093	35.077602	36.873131	36.290493	31.468658	31.000214	29.603535	0.0000314	0.293710421	0.947317656	0.00691516
ENSG00000197275	RAD54B	5.075569	4.644991	3.7547	3.465962	3.722327	3.254446	4.674921	4.613081	4.544798	0.403900976	-0.160867881	0.972805713	0.006956277
ENSG00000162415	ZSWIM5	3.662001	2.966489	2.661985	2.043446	2.296361	2.336488	2.942487	3.010813	2.798137	0.023342754	-0.32881403	0.968174121	0.007012726
ENSG00000178966	RMI1	11.127351	13.946715	14.809568	12.637755	13.327411	13.1014	12.33327	11.079032	14.190892	0.339140941	0.117926635	0.96260096	0.007035343
ENSG00000176834	VSIG10	2.613996	2.280726	2.479909	2.015972	1.999376	1.866081	2.197005	2.059781	2.732316	0.225166124	-0.180336283	0.968292771	0.007111238
ENSG00000140632	GLYR1	33.835697	33.081602	31.927667	32.707426	30.605762	29.629693	30.829515	30.374064	31.596926	0.376906778	0.062238467	0.928852722	0.007122594
ENSG00000152457	DCLRE1C	2.370422	3.070375	2.870945	3.352295	3.284064	3.057748	2.84022	2.122023	2.832591	0.027708184	0.368010058	0.975009997	0.00722986
ENSG00000117676	RPS6KA1	4.215759	4.107326	3.882384	5.049453	4.614269	4.627033	3.497604	3.921054	4.086664	0.001258811	0.368926743	0.964073816	0.007319508
ENSG00000140157	NIPA2	26.304154	33.847203	29.923641	27.592741	24.741056	27.410442	26.235548	24.78024	28.335995	0.546672065	0.057628454	0.939768355	0.007379793
ENSG00000169359	SLC33A1	8.411033	8.596725	8.203447	11.681279	11.44805	11.243616	9.232287	8.711711	9.411746	4.43E-13	0.445541945	0.941131424	0.007466907
ENSG00000117598	PLPPR5	0.537776	0.833708	0.681014	0.95234	1.087829	0.95875	0.703095	0.56857	0.838665	0.007326116	0.653277373	0.983606271	0.00751721

Gene ID	Gene name	Ctrl-1 FPKM	Ctrl-2 FPKM	Ctrl-3 FPKM	IFNγ-1 FPKM	IFNγ-2 FPKM	IFNγ-3 FPKM	Sora-1 FPKM	Sora-2 FPKM	Sora-3 FPKM	Ctrl vs IFNγ DESeq2 FDR	Ctrl vs IFNγ DESeq2 log2FC	Ctrl vs Sora DESeq2 FDR	Ctrl vs Sora DESeq2 log2FC
ENSG00000100364	KIAA0930	12.686062	11.269373	10.620584	9.823558	9.936596	10.043962	9.889288	10.20403	12.462413	0.40433167	-0.078182397	0.948753146	0.007568264
ENSG00000168297	PXK	1.915475	2.750022	2.026422	12.749791	10.112288	12.136418	2.136849	2.474991	2.267984	9.89E-107	2.523662531	0.975009997	0.007667583
ENSG00000146648	EGFR	20.828239	19.346631	20.439857	17.757578	18.933206	18.014117	18.57625	18.493495	20.216001	0.97208061	-0.00369329	0.923379466	0.007673802
ENSG00000265817	FSBP	1.172216	1.708552	2.223956	1.596092	0.763504	1.122861	1.671237	1.628661	1.273892	0.270905606	-0.463314228	0.985806133	0.00770926
ENSG00000151806	GUF1	20.224094	21.993668	22.37446	22.241449	19.740482	21.09903	20.042061	19.058516	21.999598	0.135947004	0.112913981	0.933298776	0.007742802
ENSG00000087274	ADD1	49.808469	47.302926	46.875511	49.20148	47.949077	46.258451	51.717828	41.69731	48.751816	0.043362264	0.120976021	0.922991283	0.007754485
ENSG00000137547	MRPL15	32.622992	37.389504	31.704567	27.075376	28.194575	26.467383	31.794431	32.516309	31.389826	0.093674347	-0.167080366	0.948446631	0.00780366
ENSG00000157734	SNX22	5.23806	5.378234	4.837797	9.049755	6.979224	7.628166	3.102151	4.769298	6.757505	1.06E-13	0.759672313	0.98616258	0.007820116
ENSG00000241553	ARPC4	62.312226	61.212602	64.75092	61.129972	55.738209	63.769233	71.083592	65.535681	58.204326	0.002090756	-0.184081976	0.940742183	0.007843239
ENSG00000140307	GTF2A2	30.654167	32.121863	34.527641	31.230799	31.795904	32.000289	30.288672	32.220207	30.751337	0.491261591	0.06575313	0.941252586	0.007910887
ENSG00000161091	MFSD12	19.499575	17.703977	18.508295	24.187451	22.824213	24.693578	15.13992	16.452313	18.611667	9.47E-13	0.568398328	0.949436506	0.00793382
NewGene 3565	--	0.735321	0.795518	0.482918	0.786106	0.372922	0.883882	0.525906	0.665296	0.689931	0.384608115	0.3943212	0.986971729	0.007952208
ENSG00000104388	RAB2A	18.436288	18.785491	19.482114	18.776747	17.743576	18.880653	18.457676	16.791252	18.572044	0.060909307	0.130737177	0.928402635	0.00799175
ENSG00000109790	KLHL5	67.736696	73.792995	83.892868	58.123609	57.385825	56.991251	71.411187	68.288141	73.377251	0.002240803	-0.249711512	0.940860894	0.00807244
ENSG00000111142	METAP2	56.459194	47.398256	47.272816	42.892132	40.014145	40.459143	50.876277	44.130938	53.486369	0.027700216	-0.123012188	0.911745981	0.008142679
ENSG00000118217	ATF6	10.126993	10.175255	10.364762	9.162974	8.996775	8.790058	9.66536	9.428224	9.826629	0.637187501	-0.039298938	0.922417813	0.008181597
ENSG00000122882	ECD	15.834129	14.595921	15.721116	14.666056	13.346485	13.992753	14.816174	14.375566	13.986417	0.979566581	0.003259948	0.938215465	0.00828185
ENSG00000144061	NPHP1	0.479995	0.933727	0.548984	0.329249	0.24331	0.510126	0.528309	0.524816	0.432832	0.124838828	-0.713869391	0.98658697	0.008289779
NewGene 863	--	0.559984	0.550442	0.431844	0.480472	0.471323	0.359108	0.490802	0.207475	0.66911	0.79767713	0.136928769	0.987413662	0.008309048
ENSG00000107949	BCCIP	47.287814	44.968957	52.461046	47.341474	42.445755	46.960546	43.591831	42.293266	43.731617	0.568189331	0.058906765	0.936838732	0.008497694
ENSG00000196116	TDRD7	3.787911	3.392854	3.37029	7.724686	7.491323	6.994494	3.441239	3.183286	3.3266	6.6E-39	1.219377459	0.956641805	0.008594209
ENSG00000173214	MFSD4B	1.59193	1.386676	1.312378	1.149169	1.110984	1.076547	1.461527	1.341604	1.238952	0.218522402	-0.217341643	0.966259003	0.008621826
ENSG00000221988	PPT2	25.554899	24.412133	23.911554	21.503848	24.846457	22.902576	23.490526	23.216045	24.111714	0.857080917	0.022473342	0.927431775	0.008686188
ENSG00000126067	PSMB2	53.335097	58.016065	57.174968	63.774381	62.337574	64.797995	55.552048	52.747241	52.364778	3.67E-08	0.305733635	0.919999743	0.008742707
ENSG00000197712	FAM114A1	6.048625	6.003491	6.115025	7.554359	7.370911	7.692665	5.88265	5.419895	6.496515	6.23E-08	0.441120401	0.943611383	0.00875265
ENSG00000170145	SIK2	3.982439	4.224505	3.823218	5.288689	5.121901	5.022777	4.241515	3.424484	3.689596	9.8E-13	0.506384121	0.943158719	0.008872684
ENSG00000166173	LARP6	3.026181	3.354265	3.415668	1.971551	1.917442	2.272418	3.060536	2.700169	3.028312	0.00000812	-0.803826547	0.966437239	0.008980797
ENSG00000100599	RIN3	2.955752	3.161099	2.735179	3.088389	2.892038	3.312883	3.034505	2.555687	2.849882	0.143807113	0.210257143	0.959455123	0.009076693
ENSG00000197050	ZNF420	1.846538	2.079394	2.237077	1.913724	1.606594	1.318134	2.066648	1.837104	1.820593	0.129064508	-0.319123308	0.966437239	0.009355534
ENSG00000091136	LAMB1	33.168792	32.640081	33.165668	23.188911	22.386715	21.566092	30.171119	30.080848	33.0984	4.48E-16	-0.417454675	0.899352327	0.009470755
ENSG00000141522	ARHGDIA	89.138959	84.276517	89.62112	71.764187	66.488281	75.205869	79.662534	77.805039	88.670089	0.001610921	-0.182242539	0.904651837	0.009485668
ENSG00000130023	ERMARD	5.091649	4.923552	4.476356	6.294203	4.823117	4.710334	4.32615	3.799002	5.33266	0.164600206	0.223153263	0.95962379	0.009487548
ENSG00000172663	TMEM134	9.339089	7.329437	7.454747	7.387076	6.033349	6.411851	7.467409	6.620046	7.285582	0.266483672	-0.190009344	0.958374556	0.009675717
ENSG00000137776	SLTM	17.934327	17.699135	18.717816	19.283605	15.507203	15.891647	16.216251	16.035644	18.443992	0.521329854	0.06369178	0.917023416	0.009690688
ENSG00000076685	NT5C2	32.476272	33.959417	36.190724	29.695244	30.232296	31.071503	34.509806	31.030848	32.08527	0.65046204	-0.043797008	0.921579334	0.009693841
ENSG00000152818	UTRN	19.885913	17.554532	16.799088	32.54705	31.273951	30.307524	18.130677	16.27452	17.552241	6.25E-75	0.942370505	0.902212016	0.009763036
ENSG00000184363	PKP3	0.753412	0.438276	0.665417	0.549273	0.594196	0.677503	0.510899	0.569191	0.797822	0.7573537	0.136392789	0.983073681	0.009785979
ENSG00000062716	VMP1	44.18654	42.696104	42.243141	39.170223	39.117625	40.38377	39.724608	42.149253	39.713623	0.659374201	0.034773051	0.912989785	0.009810979
ENSG00000111596	CNOT2	38.104662	39.992439	37.00111	38.768151	36.508197	37.030219	35.535911	33.509289	36.312439	0.09580231	0.103876455	0.900204778	0.009834142
ENSG00000110660	SLC35F2	3.548867	3.629933	4.409471	2.608367	2.112236	2.019125	3.426867	3.836639	3.514671	0.0000275	-0.624751424	0.955921399	0.009857083
ENSG00000141519	CCDC40	2.593325	2.440903	2.328944	1.433756	0.883061	1.031255	2.164621	2.08685	2.151987	0.005446353	-0.561997315	0.965589123	0.010069946
ENSG00000188522	FAM83G	23.936022	22.85524	22.353536	15.3807	16.581469	16.133509	21.373383	19.594883	24.814319	2.46E-09	-0.373212742	0.914505594	0.010158328
ENSG00000079313	REXO1	18.141207	17.761041	17.422152	17.170381	15.471589	15.879801	16.952708	15.843822	19.087443	0.756703557	0.029221572	0.914949978	0.010167682
ENSG00000136574	GATA4	27.156273	26.468869	26.836538	30.736634	29.757048	30.687291	27.291243	23.368265	24.796477	0.000000391	0.296880822	0.913410838	0.01017293
ENSG00000165655	ZNF503	2.83739	2.558125	2.720402	2.191594	1.972099	2.303456	2.265609	2.7287	2.664513	0.301458657	-0.178539895	0.957361172	0.010323246
ENSG00000119698	PPP4R4	5.298052	6.079736	6.120471	6.069209	6.382443	5.870424	6.044141	5.159173	5.446283	0.144593184	0.18587301	0.944926589	0.010364729
ENSG00000198763	MT-ND2	4802.724121	5213.084473	5011.82373	4864.644043	4920.387207	4724.291992	4489.881348	4791.67041	4909.203613	0.076255195	0.096969641	0.892048046	0.01045717
ENSG00000126522	ASL	40.771684	41.303428	37.402122	53.285201	50.150048	54.225214	34.933026	38.706445	37.629574	1.29E-15	0.531774607	0.918515802	0.010482392
ENSG00000125952	MAX	9.014891	8.666698	11.155262	17.921622	17.615667	18.382276	7.806221	7.680419	8.751733	1.25E-35	1.159447422	0.943134105	0.010712147
ENSG00000204304	PBX2	82.527603	78.479668	77.494583	79.706459	78.818375	77.012398	73.282318	74.516647	77.536278	0.02112821	0.129023372	0.885863023	0.010804921
ENSG00000138678	GPAT3	1.982356	2.296833	2.316032	1.345236	1.015037	1.491189	1.908104	2.055863	2.276927	0.003207176	-0.619807276	0.962000402	0.010824153
ENSG00000214063	TSPAN4	48.998015	45.866802	43.309978	34.900438	33.720373	37.598886	43.36709	43.653727	47.277416	0.002215449	-0.245603897	0.912847752	0.010856116
ENSG00000005339	CREBBP	10.929874	9.462832	8.892008	11.414435	9.662692	9.869169	9.198262	7.573207	9.980683	0.000429743	0.232534753	0.919239233	0.010881078
ENSG00000182118	FAM89A	36.535107	33.374599	35.649284	23.998821	22.279547	23.752359	29.307861	33.508839	37.012054	1.56E-09	-0.444902737	0.924793146	0.010968568
ENSG00000169598	DFFB	1.842131	1.80221	1.771396	1.833631	1.960281	1.730924	1.537805	1.67605	1.861746	0.333756279	0.196823278	0.961690266	0.011055472
ENSG00000130768	SMPDL3B	2.412017	2.009727	2.579153	2.005416	2.138809	1.863822	2.102957	1.91	2.584847	0.865037451	-0.052885568	0.966928986	0.011310447
ENSG00000136048	DRAM1	22.828863	22.522403	23.130736	35.426849	39.13408	36.568945	20.109891	19.967996	24.586862	6.02E-43	0.834504243	0.912989785	0.011324124
ENSG00000159147	DONSON	20.727128	25.661414	26.579962	21.952633	20.277655	21.959449	23.008546	23.061136	23.605124	0.878563085	-0.019739625	0.919239233	0.01132486
ENSG00000110429	FBXO3	11.353015	10.884439	9.560469	7.513964	8.929485	8.99434	9.913729	7.94978	11.484205	0.938101317	0.009127882	0.909274213	0.011368328
ENSG00000257727	CNPY2	69.01107	64.476696	61.925348	63.021301	67.407879	66.312752	59.444819	63.279954	60.660986	0.076311844	0.143384967	0.908265371	0.011391198
ENSG00000101888	NXT2	5.053815	6.216258	5.365993	6.913216	7.291792	7.299534	5.530824	5.996299	5.09977	0.000047	0.469675169	0.943551543	0.011421353
ENSG00000215440	NPEPL1	4.537441	5.494952	5.757973	5.923867	5.374406	4.419832	5.211419	4.669596	5.033582	0.502316752	0.122806162	0.946872838	0.011505926
ENSG00000148362	PAXX	13.036987	14.494051	12.790667	13.312807	12.507474	12.302771	12.572194	11.883366	13.668796	0.702609262	0.067314119	0.944926589	0.011525719
ENSG00000161914	ZNF653	3.881448	3.117393	3.471062	2.243203	2.391368	2.932718	3.054036	2.631499	4.234084	0.083796959	-0.335989396	0.961674004	0.011578911
ENSG00000276231	PIK3R6	0.25456	0.300567	0.3406	0.093786	0.10183	0.066895	0.35819	0.256139	0.250923	--	--	0.982862391	0.011603578
ENSG00000120889	TNFRSF10B	22.809926	21.059611	23.733392	23.337589	22.303774	22.126616	21.357604	19.097443	23.220613	0.046202366	0.145150248	0.904651837	0.011638756
ENSG00000120727	PAIP2	47.99405	45.165914	50.506947	40.922826	46.558194	43.380174	46.407996	49.623335	43.602267	0.930719255	0.009347835	0.902212016	0.011696741
ENSG00000152683	SLC30A6	7.469869	8.074328	8.965299	8.969776	7.554335	7.597356	8.060663	7.118644	8.807172	0.532331392	0.072818546	0.922446006	0.011737166
ENSG00000183426	NPIPA1	11.67073	12.484213	11.24879	10.858763	8.309721	8.071692	13.313549	9.426998	12.415108	0.14367307	-0.232182535	0.946733803	0.012009683
ENSG00000170854	RIOX2	6.41342	7.933843	7.083272	6.31608	5.836353	6.260676	8.686809	7.896196	8.778005	0.690404347	-0.063507817	0.937259399	0.012016231
ENSG00000101928	MOSPD1	11.341102	11.367328	13.934838	7.497783	7.780551	7.208348	10.582722	9.79083	12.800562	0.00000275	-0.532892754	0.937019494	0.012056409
ENSG00000119321	FKBP15	6.453496	6.024357	7.776727	6.766754	6.767664	6.39297	6.468901	7.245633	6.829822	0.002346064	0.225067937	0.899985307	0.012198693
ENSG00000152465	NMT2	2.230158	1.795849	1.732414	1.861879	1.482585	1.801833	1.743794	1.748442	1.825355	0.980052589	0.007955338	0.95962379	0.012245005
ENSG00000055950	MRPL43	31.383389	32.207989	30.455129	26.351403	26.028299	25.966255	30.042649	28.086119	30.870509	0.466714693	-0.077051549	0.909289369	0.012365753

Gene ID	Gene name	Ctrl-1 FPKM	Ctrl-2 FPKM	Ctrl-3 FPKM	IFNγ-1 FPKM	IFNγ-2 FPKM	IFNγ-3 FPKM	Sora-1 FPKM	Sora-2 FPKM	Sora-3 FPKM	Ctrl vs IFNγ DESeq2 FDR	Ctrl vs IFNγ DESeq2 log2FC	Ctrl vs Sora DESeq2 FDR	Ctrl vs Sora DESeq2 log2FC
ENSG00000275052	PPP4R3B	19.699809	21.569916	20.538315	22.333037	22.445438	21.057563	20.168445	18.720224	20.536984	0.0000526	0.243865707	0.881000388	0.01242777
ENSG00000141428	C18orf21	9.536261	9.678063	10.375791	10.893597	10.074886	11.406105	10.231559	9.34452	8.803306	0.092631734	0.216070187	0.937289107	0.012442889
ENSG00000153187	HNRNPU	151.262029	156.473011	163.188818	146.467209	141.164349	138.061475	163.466154	177.293303	156.006842	0.994064494	-0.000640369	0.851697047	0.012486972
ENSG00000124299	PEPD	93.433444	89.870724	83.569965	79.490278	86.798915	86.275149	81.555539	80.842369	85.310913	0.273642248	0.085255832	0.878217781	0.012497678
ENSG00000197375	SLC22A5	1.215732	1.250624	1.528535	2.371689	2.257531	2.224091	1.264101	1.44821	1.070804	5.31E-08	0.914299245	0.96521287	0.012510086
ENSG00000068383	INPP5A	7.374044	5.775579	6.967648	5.973777	5.583346	5.132218	6.755246	5.628819	6.678333	0.385514145	-0.127028546	0.935960414	0.012563077
ENSG00000146433	TMEM181	32.14756	32.741467	34.328423	24.190054	23.197531	22.415861	30.816704	29.565666	33.577061	3.83E-11	-0.360440422	0.871800503	0.012666695
ENSG00000129197	RPAIN	24.055379	24.01872	24.457661	20.590519	22.307986	22.368982	25.555811	27.624563	24.782847	0.719826933	0.034423207	0.900728386	0.012673814
ENSG00000108984	MAP2K6	2.622376	3.106715	1.825041	2.911481	2.362708	2.23997	3.323637	2.543068	2.744078	0.71561468	0.060067814	0.937017918	0.012723244
ENSG00000126562	WNK4	4.320092	3.472433	3.193573	4.010621	3.162447	4.028966	3.117794	3.502506	3.819806	0.30607057	0.172502809	0.942949022	0.012840744
ENSG00000114933	INO80D	1.889119	2.32628	2.322828	2.340805	2.260211	2.236133	2.148672	2.103326	2.120906	0.04198821	0.212472498	0.921143129	0.012892905
ENSG00000090316	MAEA	61.96731	58.126342	54.627292	47.424648	50.052807	52.672544	51.190894	50.564831	62.790529	0.200936361	-0.093156348	0.900185759	0.012916894
ENSG00000160633	SAFB	50.831295	48.292902	48.962646	48.441977	45.586578	47.23446	44.681855	44.561515	51.38209	0.32912401	0.065276065	0.877861219	0.012982799
ENSG00000141933	TPGS1	5.997943	6.42759	3.719778	3.768722	4.28084	6.317533	4.76406	5.595977	4.908995	0.967820559	-0.015982988	0.96260096	0.013043465
NewGene_6010	--	4.11285	4.623399	5.429447	6.204235	5.739486	5.039462	4.890701	4.914369	4.444845	0.005561962	0.350738146	0.936427186	0.013060882
ENSG00000150787	PTS	7.30138	6.71358	7.20144	5.058939	5.51552	5.604141	6.076225	6.355615	7.664853	0.206122596	-0.245616033	0.950641313	0.013074581
ENSG00000011422	PLAUR	3.696911	3.227725	3.202533	2.819375	1.739574	2.269096	3.606611	3.447597	2.126035	0.134322428	-0.388549983	0.962348493	0.01315799
ENSG00000197021	CXorf40B	7.855044	8.544672	8.238067	9.63108	8.638704	9.539914	7.515152	8.187784	8.040409	0.006976398	0.320508496	0.93257876	0.013339011
ENSG00000136940	PDCL	10.718568	11.868976	11.523105	10.262036	9.57906	9.604566	10.130363	10.855678	11.398144	0.586946295	-0.061850311	0.906800367	0.013392228
ENSG00000198951	NAGA	4.24126	4.382007	4.507361	3.122758	2.846665	3.461839	4.43529	3.770305	4.359089	0.00741356	-0.345422906	0.927382697	0.013398187
ENSG00000102048	ASB9	4.768379	4.216651	3.393042	5.031991	5.082728	5.070242	4.244551	4.149846	3.197279	0.006855152	0.461483409	0.956673647	0.013398398
ENSG00000127946	HIP1	12.652261	11.116667	11.088846	6.803833	7.017145	7.146803	10.85644	9.891037	12.213038	4.97E-17	-0.587746649	0.893722107	0.013417576
ENSG00000130396	AFDN	37.674643	41.927007	39.573661	36.325412	35.212196	34.392659	37.759596	34.980826	38.132231	0.994074892	-0.000742371	0.872782975	0.01349083
NewGene_4761	--	1.214515	1.219379	0.822553	0.969586	1.267359	0.509623	0.963647	0.994176	1.442752	0.78013289	-0.142732843	0.969669876	0.013566639
ENSG00000284981	AC093668.3	7.937995	8.794202	7.397235	8.159757	8.734533	8.884669	6.814878	7.851021	8.392338	0.136320085	0.243022074	0.944926589	0.013589956
ENSG00000103365	GGA2	30.352906	30.325337	30.183183	25.831631	24.122129	25.115138	29.04291	26.272371	32.091533	0.010314969	-0.142179051	0.871724395	0.013681242
ENSG00000198064	NPIPB13	3.085542	3.630761	3.730461	3.673616	3.198878	3.664286	3.407483	3.167644	3.692059	0.282648666	0.193818196	0.945744818	0.013685273
ENSG00000153094	BCL2L11	7.213578	7.481621	7.093531	11.152338	10.378988	10.380194	8.327863	6.646877	6.673032	1.1E-23	0.702031175	0.912989785	0.013688252
ENSG00000121579	NAA50	56.971866	69.111421	74.876997	71.197334	68.441545	66.006706	66.664377	57.868715	63.195092	0.089883375	0.146664249	0.897750067	0.013697543
ENSG00000137486	ARRB1	4.988017	5.178793	4.680143	2.63776	2.952259	3.474897	4.17256	5.360788	5.173622	0.00000461	-0.590590941	0.936838732	0.013734712
ENSG00000181513	ACBD4	4.323546	4.593658	4.80689	4.564079	3.887536	4.218048	3.894126	4.58106	4.272945	0.84969637	0.041707062	0.943937035	0.013797221
ENSG00000115145	STAM2	4.382985	4.676818	4.453996	3.996446	3.976416	4.061424	4.291284	4.089808	4.418659	0.886374932	-0.019497704	0.904794479	0.013826613
ENSG00000101493	ZNF516	4.554429	3.956449	4.34141	4.427525	4.351034	4.270237	4.376405	3.762743	4.321143	0.135071834	0.140393417	0.902381086	0.014039688
ENSG00000168993	CPLX1	6.39693	6.275486	5.953436	7.10184	7.302802	7.060428	6.27377	5.818578	5.537216	0.001884427	0.348903648	0.927848034	0.014040923
ENSG00000183624	HMCES	17.965612	17.107149	17.014409	18.568103	17.506134	18.571898	17.383597	16.038791	16.612979	0.015228552	0.205642363	0.894291486	0.014047374
ENSG00000116698	SMG7	22.953327	23.438346	23.642496	30.58787	25.772028	25.073923	22.42454	21.11702	23.228137	1.34E-08	0.312678903	0.857344644	0.014085335
ENSG00000101384	JAG1	26.285697	28.874451	29.792765	22.394136	23.090869	23.110608	26.006916	24.244331	30.375477	0.014039877	-0.165747756	0.885612975	0.014127366
ENSG00000169282	KCNAB1	0.210322	0.266462	0.275939	0.313558	0.465964	0.283604	0.181574	0.219644	0.318053	0.101635648	0.647723076	0.977950698	0.014246418
ENSG00000122642	FKBP9	24.400268	24.647551	24.787171	20.711313	19.224038	19.798428	23.525076	21.695342	24.49069	0.018364238	-0.153584124	0.867330156	0.014318987
ENSG00000011105	TSPAN9	13.146806	11.123961	9.264754	9.696337	10.057545	9.766786	10.791358	10.057676	11.44054	0.795560846	-0.040310255	0.917824682	0.014406554
NewGene_2446	--	3.334048	3.499011	2.997645	3.734318	3.14422	2.887581	2.840152	3.249285	3.3269	0.304336618	0.160126749	0.937259399	0.014425127
ENSG00000163714	U2SURP	72.246917	77.542416	87.782311	76.675544	77.20715	77.022667	81.227239	80.664977	79.962084	0.023149594	0.140299478	0.858092839	0.014565481
ENSG00000023892	DEF6	2.355962	1.583946	1.590132	1.226564	1.390063	1.545878	1.450373	1.754429	2.036828	0.364544547	-0.259346697	0.961263703	0.014796596
ENSG00000178882	RFLNA	8.065763	6.023634	6.81756	2.344298	2.666747	2.495245	6.036999	6.001762	7.75822	3.68E-21	-1.333896278	0.933954999	0.014923436
ENSG00000118197	DDX59	9.355074	7.864563	8.377188	6.346019	6.669111	7.426622	7.925133	7.393341	8.371226	0.348372137	-0.134116969	0.914505594	0.014936123
ENSG00000137960	GIPC2	5.490371	6.290666	5.95526	5.971704	5.627928	6.027018	5.554672	5.244231	6.022158	0.208716287	0.139607026	0.906310374	0.014967725
ENSG00000196642	RABL6	50.022358	49.727313	45.580412	39.888522	40.918401	41.792458	44.833622	42.137357	52.794568	0.133589562	-0.107489239	0.876593655	0.015029166
ENSG00000148229	POLE3	43.883634	44.888111	44.283918	36.879447	35.27733	37.892647	41.928247	40.040176	44.406212	0.052338693	-0.12777104	0.85041269	0.015042637
ENSG00000140463	BBS4	4.900631	6.446415	5.298196	3.510335	3.724647	4.011368	5.713337	4.24103	5.941427	0.020024375	-0.314007194	0.927639534	0.015054663
ENSG00000131238	PPT1	59.904738	63.222806	64.389681	42.033816	43.241134	44.017379	56.532653	68.040731	59.177022	8.85E-08	-0.33104607	0.862906245	0.0150803
ENSG00000075336	TIMM21	6.201324	6.347954	6.45236	4.492702	5.226205	4.621664	5.851886	6.445163	5.883871	0.015658593	-0.279996445	0.911553655	0.015184933
ENSG00000170855	TRIAP1	25.013741	25.283964	26.258831	21.859764	23.607609	22.117235	24.33334	23.955463	24.252886	0.795560846	-0.032433695	0.889704878	0.015288695
ENSG00000196776	CD47	14.081549	16.78494	18.027957	18.925039	16.17506	17.553049	15.306792	13.732009	15.079761	0.00000641	0.325549851	0.878159847	0.015314091
ENSG00000103343	ZNF174	4.002644	4.068044	3.233778	3.771406	4.000061	3.610037	3.468863	3.809066	3.581641	0.478418479	0.135514441	0.939506431	0.015360129
ENSG00000156162	DPY19L4	8.528228	9.707916	9.069478	7.696636	7.995522	7.176067	8.561032	8.152661	8.989886	0.126436136	-0.134424638	0.879355906	0.015439857
ENSG00000178078	STAP2	55.099595	52.314494	54.143397	45.074098	42.684808	46.006947	48.118662	50.281952	54.479559	0.083846487	-0.124848031	0.861670128	0.015599957
ENSG00000106701	FSD1L	2.116798	2.704901	2.405088	3.718015	3.077495	3.597152	3.58657	3.030429	2.721136	0.00000825	0.55696258	0.936838732	0.015630273
ENSG00000140992	PDPK1	7.330931	6.677324	6.701332	7.181071	6.667111	7.182198	6.755646	5.950504	7.106377	0.047031046	0.163414663	0.877927524	0.015934711
ENSG00000042980	ADAM28	0.61659	0.754565	0.67007	0.78296	0.575498	0.772067	0.972838	0.683786	0.608233	0.230928016	0.28525268	0.954209314	0.016161043
ENSG00000133789	SWAP70	10.750277	11.863665	11.804183	8.497173	7.74078	8.522232	10.610781	9.743749	12.453781	0.0000173	-0.333023589	0.885688258	0.01616982
ENSG00000118873	RAB3GAP2	14.83466	15.567231	16.188192	15.708589	14.693626	15.154813	14.667682	14.152908	15.493339	0.083656967	0.114839496	0.843281245	0.016202307
ENSG00000103994	ZNF106	9.000081	9.221519	9.066755	8.370226	7.613591	7.86024	8.169363	8.087088	9.07206	0.869467157	-0.015408083	0.847411218	0.016255831
ENSG00000075914	EXOSC7	18.057281	18.245302	16.57358	15.472	13.892116	15.793331	17.386805	16.45285	16.284706	0.566623015	-0.080080841	0.904651837	0.016259676
ENSG00000135845	PIGC	33.959168	33.461485	31.983045	28.107205	26.834687	25.779848	34.139922	31.345614	31.071532	0.043593251	-0.163494899	0.878442552	0.016450509
ENSG00000040531	CTNS	14.326599	13.364346	12.796112	12.804912	11.934229	11.186405	12.175784	11.794513	14.646706	0.900797649	-0.016410555	0.889993893	0.016481436
ENSG00000035115	SH3YL1	2.668105	2.883095	2.328067	2.055308	2.415329	2.24437	2.388932	2.259983	3.294679	0.547587831	-0.132397266	0.940469805	0.016491858
ENSG00000128973	CLN6	36.114441	37.271083	34.261371	29.530706	27.04348	30.34216	33.949341	33.878336	34.911521	0.01540203	-0.180507324	0.849363797	0.016507479
ENSG00000254858	MPV17L2	20.375832	18.398895	20.711607	23.791559	25.396959	23.756552	18.597858	18.248634	19.606335	0.000000334	0.441264139	0.887737202	0.016527186
ENSG00000146463	ZMYM4	8.328802	8.315083	8.361333	8.450189	7.792442	6.946481	8.34202	7.349167	7.642007	0.641277857	0.047757198	0.86531291	0.016556165
ENSG00000146350	TBC1D32	0.715835	0.950006	0.89975	1.21934	0.997672	1.059288	0.862624	0.686432	0.815732	0.011861671	0.532454349	0.950124417	0.016626174
ENSG00000196689	TRPV1	2.341743	3.319083	3.228986	3.139619	2.838315	2.663369	2.586588	2.724299	3.210154	0.800616507	0.05320402	0.927848034	0.016639058
ENSG00000112062	MAPK14	32.645751	34.875766	35.80575	30.879696	27.839826	28.879713	34.648689	30.524708	34.238826	0.478725877	-0.054237631	0.844414201	0.016782493
ENSG00000058272	PPP1R12A	10.773002	11.032032	11.488122	11.968664	11.330073	11.533946	10.365159	9.479776	11.240518	0.002417415	0.205260659	0.858938407	0.016797165
ENSG00000182400	TRAPPC6B	7.877574	8.631733	9.060919	8.334126	7.878214	7.998413	7.954696	7.637258	8.750017	0.568445676	0.067704047	0.890549694	0.016846609

Gene ID	Gene name	Ctrl-1 FPKM	Ctrl-2 FPKM	Ctrl-3 FPKM	IFNγ-1 FPKM	IFNγ-2 FPKM	IFNγ-3 FPKM	Sora-1 FPKM	Sora-2 FPKM	Sora-3 FPKM	Ctrl vs IFNγ DESeq2 FDR	Ctrl vs IFNγ DESeq2 log2FC	Ctrl vs Sora DESeq2 FDR	Ctrl vs Sora DESeq2 log2FC
ENSG00000109381	ELF2	13.145115	12.020893	11.750225	13.052324	14.059885	12.160177	12.139127	10.627414	11.491654	0.000210662	0.303392446	0.874494328	0.016917299
ENSG00000152904	GGPS1	22.377293	23.168268	23.499527	19.07328	19.979174	18.582617	20.630526	21.259133	24.269006	0.513800854	-0.059551426	0.860898481	0.016978489
ENSG00000135829	DHX9	108.123978	107.683815	104.277519	99.408371	99.704659	94.608154	99.580742	100.613945	103.469872	0.740886492	0.023263376	0.805834869	0.017001364
ENSG00000102897	LYRM1	35.054951	36.807419	32.00449	29.210375	31.591654	32.202609	31.026655	32.587697	33.884088	0.998890436	0.000292929	0.864909004	0.017123261
ENSG00000115828	QPCT	3.544373	3.512994	3.088858	1.620523	2.351078	2.101506	2.783775	3.577504	3.212502	0.005374197	-0.584592722	0.941131424	0.017127545
ENSG00000255423	EBLN2	1.866004	2.226036	2.145713	2.08272	2.327038	2.382879	1.719935	2.074735	2.108663	0.265462866	0.27398247	0.951699519	0.017158116
ENSG00000124444	ZNF576	6.445309	6.988679	5.869471	5.384417	5.907422	6.571037	5.809292	6.81323	6.46514	0.967621501	0.007818862	0.911553655	0.017338387
ENSG00000125779	PANK2	18.052023	20.218616	19.5975	19.439938	20.20004	20.190143	19.872057	18.360315	20.22023	0.103230661	0.135058306	0.862289124	0.017383457
ENSG00000069869	NEDD4	6.533017	7.45929	7.81237	4.222961	4.006099	3.907787	6.73722	6.922788	7.027112	5.68E-16	-0.699083148	0.877927524	0.017517772
ENSG00000135119	RNFT2	2.02129	2.545747	2.028861	1.851231	0.933715	1.431037	2.100086	1.986624	2.082752	0.152034841	-0.352248494	0.935366587	0.017524708
NewGene_5990	--	0.804089	1.035143	0.960609	0.633678	0.327987	0.322947	0.801166	1.417252	0.922744	0.03997825	-0.905551066	0.96988122	0.017585276
NewGene_5845	--	2.42619	3.276447	2.902466	2.502466	2.274254	2.387699	4.280673	4.084551	3.070705	0.791479349	-0.068592456	0.944041721	0.017612096
ENSG00000169750	RAC3	42.577045	39.709298	38.096082	33.217673	35.891074	35.848891	36.489982	35.720053	40.590603	0.579485372	-0.063019398	0.869068091	0.017676255
ENSG00000126016	AMOT	2.930608	3.093118	2.975837	2.30604	1.901339	1.968477	3.040536	2.535137	2.926154	0.0000516	-0.448794573	0.893811029	0.017735638
ENSG00000119335	SET	209.970399	224.196117	242.664389	240.636036	233.463764	235.572364	221.298145	207.272079	213.829441	0.000436304	0.205635971	0.831722684	0.017962967
ENSG00000158604	TMED4	59.787143	56.308696	58.332954	47.341358	47.795206	48.229892	56.301389	53.717589	56.995871	0.031652204	-0.135364716	0.81723769	0.018053913
ENSG00000177380	PPFIA3	1.034196	0.704262	0.473839	0.766283	0.901582	0.957769	0.456789	0.447065	0.873317	0.576972113	0.180184166	0.96128358	0.018135748
ENSG00000111678	C12orf57	53.719111	47.005503	44.129413	38.093814	42.387155	42.306183	43.483817	53.309623	42.132456	0.704785519	-0.055021088	0.906232355	0.018138643
ENSG00000139180	NDUFA9	16.108854	14.949124	14.803651	16.34786	16.29003	16.5229	14.904722	15.450536	14.196744	0.0000311	0.254242667	0.850368099	0.018167936
ENSG00000156471	PTDSS1	14.8434	15.016891	14.917714	13.25769	12.397454	12.528248	13.978486	13.967733	14.581145	0.255718681	-0.082834892	0.824864103	0.018314011
ENSG00000227345	PARG	8.918948	10.520412	10.173925	9.601501	8.460932	8.458484	8.921393	8.138592	9.35375	0.724678119	0.039000508	0.862461619	0.01834202
ENSG00000132155	RAF1	60.80144	61.093955	62.040817	70.398347	71.681816	65.868389	58.98907	52.709287	58.708822	4.9E-12	0.335304579	0.80574384	0.018375447
ENSG00000181744	DIPK2A	7.024722	6.918333	7.084302	5.865777	5.879653	5.8526	6.776504	6.59558	6.507617	0.506541049	-0.071312884	0.866930313	0.01849332
ENSG00000111727	HCFC2	1.573879	1.714102	1.83827	1.640884	1.67546	1.489539	1.650336	1.427778	1.736346	0.747516824	0.063412167	0.917773371	0.018497627
ENSG00000136279	DBNL	47.044816	46.488719	36.967777	41.203314	40.718445	39.050977	41.343733	45.945006	50.796984	0.018777324	0.165820668	0.836459502	0.018531772
ENSG00000108395	TRIM37	14.449178	14.232653	14.76377	11.21992	10.968691	10.450703	13.517348	12.596282	14.241157	0.000796372	-0.23815539	0.836757532	0.018620929
ENSG00000123352	SPATS2	16.082932	16.374717	18.568132	12.551716	12.475524	12.972933	16.708823	15.625347	17.021346	0.000270006	-0.297690867	0.857654023	0.018655845
ENSG00000171103	TRMT61B	5.850727	6.848506	7.159046	6.160316	6.250854	5.779641	5.983442	5.625282	7.146965	0.967287456	0.008870164	0.913064601	0.018682703
ENSG00000198301	SDAD1	14.010995	14.081842	13.671045	16.840819	15.419194	15.145324	12.405274	12.805338	14.750489	0.00000623	0.327987921	0.855312131	0.018721679
ENSG00000125170	DOK4	12.581519	12.405759	11.417416	10.317158	10.159493	11.521526	11.598941	12.030258	10.96824	0.563478673	-0.067988816	0.876283568	0.01879302
ENSG00000161533	ACOX1	10.290324	13.299658	11.527744	10.326033	10.761667	9.992218	11.99337	10.707032	11.142263	0.939959435	-0.008365077	0.845094928	0.018846049
ENSG00000140598	EFL1	11.771477	11.091799	10.752462	9.387845	9.469321	9.059335	10.454492	9.93664	11.230599	0.220070946	-0.116270709	0.856243669	0.018970088
ENSG00000030110	BAK1	20.577399	20.264591	18.578578	23.513693	23.833724	26.647696	17.863024	19.204781	19.445091	9.24E-09	0.460665312	0.860402342	0.019005789
ENSG00000125449	ARMC7	7.279316	7.47638	7.502968	6.555901	5.970666	6.605706	5.970302	7.423446	7.262905	0.717220728	-0.058524681	0.908042167	0.01905553
ENSG00000172795	DCP2	6.042784	7.238086	7.820217	4.812398	4.520654	4.935177	6.761612	6.042248	7.284171	0.00000689	-0.415555229	0.877202316	0.019088351
ENSG00000181090	EHMT1	17.489951	16.224234	16.349048	15.745245	15.256957	15.470386	15.420591	16.013829	17.320594	0.890253509	-0.013656786	0.836757532	0.01924333
ENSG00000173153	ESRRA	38.501966	36.682209	31.140117	32.073442	34.179913	34.347686	32.265931	32.091325	35.280142	0.484316923	0.073061661	0.854955032	0.019324944
ENSG00000119203	CPSF3	25.69776	28.617724	28.93242	23.66515	23.787201	23.899569	25.953527	24.565658	28.478916	0.444903642	-0.068831157	0.843229928	0.019345086
ENSG00000148841	ITPRIP	1.117619	1.459656	1.361084	2.618221	2.187923	2.67582	1.377997	1.182894	1.634665	8.44E-14	0.990015436	0.924116	0.019349006
ENSG00000147586	MRPS28	38.656024	37.83603	39.956634	33.378261	34.728363	34.991743	36.890179	37.532429	37.855031	0.656944194	-0.050508254	0.856582279	0.019380914
ENSG00000103275	UBE2I	53.580704	49.416654	52.273375	44.077191	47.879548	45.83079	51.902393	48.757249	48.250691	0.631621264	-0.037969297	0.814652612	0.01939168
ENSG00000214013	GANC	3.410492	3.316735	3.844697	3.241686	4.085519	3.048461	3.468468	3.343328	3.334094	0.054890956	0.274973261	0.902330457	0.019417956
ENSG00000170425	ADORA2B	1.200277	1.66876	1.833571	0.929943	0.678606	0.724587	1.458461	1.605164	1.379817	0.005564569	-0.856574433	0.954097361	0.019425686
ENSG00000115234	SNX17	32.401321	31.496955	31.031766	29.468771	29.998731	31.844182	28.307155	29.579748	33.28412	0.326557793	0.08152712	0.836701916	0.019540311
ENSG00000168701	TMEM208	26.885721	26.190695	25.91198	23.928187	22.605715	24.82888	24.643655	25.235823	24.856881	0.942855622	0.011073844	0.875857998	0.01958551
ENSG00000173581	CCDC106	10.692959	9.541946	9.675101	10.191019	10.087545	8.961437	8.851724	9.27884	10.049863	0.174162401	0.166973626	0.888164732	0.019696572
ENSG00000070061	ELP1	38.049783	39.021133	39.662488	58.261959	62.241513	57.238786	38.049974	35.039041	39.943383	1.48E-46	0.753127469	0.782147827	0.019734321
ENSG00000160087	UBE2J2	41.041207	43.360079	40.769862	39.254184	37.684915	42.058688	41.122454	41.424141	42.12232	0.859271267	0.017102984	0.821088076	0.019755195
ENSG00000174227	PIGG	27.213212	24.639676	25.231386	23.305195	22.365911	20.065566	24.552344	22.702694	26.740386	0.27194744	-0.097758987	0.835719911	0.019798786
ENSG00000091009	RBM27	7.62514	7.721579	7.891868	8.157551	7.910689	8.248052	7.709607	7.030625	7.355285	0.002427576	0.212844801	0.835719911	0.019970748
ENSG00000000003	TSPAN6	51.060493	49.112198	49.995176	34.802426	36.611523	40.277011	45.964166	46.736741	48.850527	0.000000255	-0.317138646	0.796487825	0.019999834
ENSG00000127804	METTL16	11.299494	10.467912	9.802362	9.689641	9.124336	9.712155	10.717157	9.665447	9.553433	0.947063222	0.007810788	0.853901454	0.020079426
ENSG00000242114	MTFP1	12.770121	13.595628	12.403638	9.81249	8.148474	9.490675	10.663718	11.553432	11.945373	0.063201822	-0.246607473	0.890549694	0.020102051
ENSG00000109920	FNBP4	20.115929	20.355883	22.857859	25.807601	22.415566	23.714676	21.643983	18.396281	21.006252	0.00000758	0.325278738	0.843182121	0.020254401
ENSG00000046647	GEMIN8	5.449123	6.414743	5.266859	3.803149	3.280331	3.600859	5.039537	4.419884	6.438769	0.002125533	-0.534573398	0.920339401	0.020352403
ENSG00000165119	HNRNPK	418.181133	424.985283	425.964419	395.966446	393.807165	388.921072	404.14721	406.049713	416.644768	0.560474542	0.032707075	0.749497908	0.020353779
ENSG00000079387	SENP1	8.195771	8.378572	8.270668	8.171551	8.100754	7.683458	8.028068	7.629044	7.970716	0.424073063	0.076422051	0.837614182	0.020546876
ENSG00000038358	EDC4	12.992064	11.590735	11.976716	10.059525	9.727516	10.288439	11.407445	10.564201	12.893566	0.103740036	-0.135270231	0.843316277	0.020591782
ENSG00000197114	ZGPAT	17.41863	15.712064	14.825956	11.666724	10.845553	12.397988	13.159359	16.42806	16.046055	0.001263891	-0.318991283	0.880683172	0.020653577
NewGene_2011	--	2.618961	3.29605	2.880027	3.622362	3.121823	2.417873	2.68864	3.026282	4.168545	0.679308924	-0.088941729	0.909311801	0.020763174
ENSG00000116774	OLFML3	32.375433	29.329586	30.575444	21.454365	22.067671	21.469547	28.544193	28.673886	30.000015	0.0000023	-0.356494255	0.829805918	0.020772836
ENSG00000162409	PRKAA2	3.134961	3.657975	4.190475	2.361705	2.28822	2.077523	3.392043	3.24715	3.823744	0.000000264	-0.560335861	0.879547072	0.020796389
ENSG00000171236	LRG1	34.725739	34.499313	34.138672	24.735184	26.505999	26.860039	32.067333	31.532852	34.815495	0.0000728	-0.256199712	0.790232287	0.020811045
ENSG00000185215	TNFAIP2	7.890755	9.297497	8.658023	13.058436	11.390717	12.629244	9.129478	7.718886	7.903944	1.07E-19	0.644736876	0.842882672	0.020813235
ENSG00000099617	EFNA2	10.623841	8.499523	7.32761	8.556684	7.674317	10.52461	8.377493	8.73526	8.051613	0.464997855	0.162163467	0.921143129	0.020820536
ENSG00000165669	FAM204A	3.120598	3.55754	2.885882	3.873891	3.748038	3.186998	2.683158	3.280306	3.536876	0.633448054	0.062479641	0.862002485	0.020901034
ENSG00000161558	TMEM143	3.045433	2.210374	2.840365	2.744862	2.389511	2.812141	2.327979	2.80478	2.594872	0.756023487	0.074673028	0.925032032	0.021060802
ENSG00000167925	GHDC	12.468817	11.842159	9.87535	10.070053	9.537799	10.872581	11.256375	10.53054	11.093941	0.802968046	-0.038901685	0.876523965	0.021085871
ENSG00000136710	CCDC115	12.227676	12.788375	11.686649	9.413205	11.814072	12.266238	10.580756	11.372181	11.817013	0.894794052	0.023563024	0.869253685	0.021175902
ENSG00000213585	VDAC1	104.298211	109.870996	113.719354	96.037023	93.171272	94.988871	102.670666	98.020247	112.366923	0.381489545	-0.059073842	0.789110571	0.021179894
ENSG00000139428	MMAB	25.49637	23.20004	20.621267	23.781074	20.375724	24.907119	22.764049	19.07528	26.257516	0.008003908	0.236202368	0.849081405	0.021279047
ENSG00000218891	ZNF579	7.753556	7.39704	7.079066	6.550909	6.656402	6.802193	6.733925	6.090289	7.678382	0.858199415	0.027839972	0.875009581	0.021360368
ENSG00000166887	VPS39	14.851729	13.819967	13.728806	12.693621	12.722728	12.16557	13.067488	12.801445	14.497114	0.744019668	-0.031647025	0.81774506	0.021381339
ENSG00000168890	TMEM150A	20.800971	20.879711	19.242078	18.093687	19.366338	18.661129	17.996832	19.271312	20.523379	0.984928337	-0.002701514	0.850650954	0.021389849

Gene ID	Gene name	Ctrl-1 FPKM	Ctrl-2 FPKM	Ctrl-3 FPKM	IFNγ-1 FPKM	IFNγ-2 FPKM	IFNγ-3 FPKM	Sora-1 FPKM	Sora-2 FPKM	Sora-3 FPKM	Ctrl vs IFNγ DESeq2 FDR	Ctrl vs IFNγ DESeq2 log2FC	Ctrl vs Sora DESeq2 FDR	Ctrl vs Sora DESeq2 log2FC
ENSG00000198730	CTR9	21.403482	20.601318	20.675791	19.127529	19.510199	18.985901	19.465438	19.579436	21.395038	0.78013289	0.024435513	0.794391863	0.021471751
ENSG00000131462	TUBG1	51.285486	50.51056	54.007406	45.142507	44.58108	44.264928	48.912078	49.894713	49.569191	0.350892293	-0.071041816	0.806625361	0.02157588
ENSG00000167778	SPRYD3	6.126918	5.808863	5.407529	5.30615	4.979929	5.476354	5.1697	5.104061	6.251496	0.947015568	0.011594699	0.881968957	0.021610463
ENSG00000101608	MYL12A	167.082863	166.566562	180.761466	152.447987	163.265826	161.93303	159.122569	170.015084	159.196951	0.597104271	0.043649889	0.811542717	0.021662614
NewGene_5367	--	1.070622	1.605483	1.874486	1.631497	1.57424	1.202212	1.858227	2.037733	1.252802	0.778099055	0.107883055	0.951677266	0.021706901
ENSG00000164818	DNAAF5	29.671064	28.975366	31.031014	25.00086	22.574271	24.820818	27.675465	26.444115	31.551592	0.018143526	-0.162706892	0.808848362	0.021720694
ENSG00000136875	PRPF4	14.747272	14.521526	16.112206	14.38621	13.098087	14.399357	13.946174	13.402175	16.368445	0.765195447	0.033500796	0.837991965	0.021731043
ENSG00000141027	NCOR1	23.618384	20.491786	19.616233	22.09659	23.779628	22.130984	18.26972	18.366449	19.899501	0.00093962	0.20696475	0.794851986	0.02185079
ENSG00000242715	CCDC169	0.505968	0.705644	0.779798	0.323685	0.259442	0.140981	0.345759	0.337219	0.332319	0.043252455	-0.870044396	0.96128358	0.021924309
ENSG00000169621	APLF	1.02019	1.248562	1.295143	0.706746	0.574467	0.623005	1.212629	1.041144	1.142506	0.000621685	-0.765142941	0.928852722	0.022014687
ENSG00000064313	TAF2	11.780846	12.402997	12.361757	11.545183	11.001826	10.484542	12.017777	10.832822	12.583346	0.925918065	-0.01076736	0.817077265	0.022018318
ENSG00000109171	SLAIN2	6.343143	6.193757	6.518262	6.619337	5.983626	6.016494	6.329295	5.847458	6.38503	0.135829913	0.133308647	0.828753819	0.022053687
ENSG00000261832	AC138894.1	1.066907	0.010443	0.61258	0.359017	0.653721	0.629775	0.597598	0.787488	0.11775	0.986348802	0.035527508	0.991193478	0.022154293
ENSG00000126453	BCL2L12	35.197501	35.515572	30.599771	33.493116	31.954732	33.429346	39.764753	36.446473	46.068832	0.044500189	-0.212782981	0.831722684	0.022220072
ENSG00000104946	TBC1D17	16.06695	17.620841	13.89999	19.183923	17.567999	16.025782	13.980603	15.08746	16.154489	0.002532775	0.284079389	0.859948152	0.022225809
ENSG00000141258	SGSM2	11.157056	10.559848	10.257419	11.203962	9.21961	9.230624	9.867098	9.388746	12.334229	0.73517571	0.040729423	0.848214103	0.022247236
ENSG00000122378	PRXL2A	16.83543	18.560499	15.507454	17.691157	15.996583	17.154107	18.91686	16.161386	16.524508	0.004533769	0.176982727	0.802053213	0.022409008
ENSG00000256269	HMBS	28.006947	26.861995	26.339599	22.417374	20.480828	21.634118	24.396454	25.569814	26.631892	0.022798547	-0.193578032	0.830904793	0.022486666
ENSG00000166479	TMX3	6.183558	7.891812	8.667552	7.924334	7.765937	7.539826	6.827878	7.658655	8.133777	0.329238776	0.127405716	0.878048861	0.022801729
ENSG00000136104	RNASEH2B	4.973711	4.924797	5.098022	5.438079	4.634554	5.374791	4.911908	4.607126	4.551038	0.461640555	0.126607357	0.893671761	0.023062076
ENSG00000114480	GBE1	9.154361	9.107054	8.367914	8.071057	8.28863	7.31215	8.145057	7.92231	9.358397	0.860867148	-0.023888292	0.841097048	0.023159119
ENSG00000106330	MOSPD3	10.64381	11.307883	8.632011	9.566521	9.352583	9.101004	9.482316	9.19698	10.873952	0.782045164	0.05492252	0.897575287	0.023243479
ENSG00000254901	BORCS8	8.156582	10.572241	9.157372	9.512718	8.492885	8.616328	8.781839	8.931509	10.512115	0.633448054	0.076569617	0.879032127	0.023271157
ENSG00000030066	NUP160	49.691624	49.343675	52.118764	46.993657	46.233799	45.979961	46.748779	46.339016	48.530706	0.730017908	0.025039193	0.744527009	0.023301862
ENSG00000165802	NSMF	14.155777	13.53072	11.719741	11.092541	11.55717	10.472715	12.210802	12.125182	13.47861	0.418654007	-0.093579771	0.843940863	0.023340331
ENSG00000104320	NBN	12.606757	14.380046	14.629893	20.789226	19.633446	19.794059	13.029967	12.845134	14.146026	8.39E-24	0.681918698	0.816298198	0.023439162
ENSG00000205937	RNPS1	66.022366	64.79211	70.465854	61.950503	57.149543	61.732177	69.136052	61.001318	68.070949	0.174162401	-0.089494649	0.761397122	0.023627905
ENSG00000110090	CPT1A	6.32444	6.818913	5.490956	12.880275	11.021916	12.554713	6.59895	5.631142	5.569288	6.64E-36	1.114035361	0.866147723	0.023632283
ENSG00000197451	HNRNPAB	116.196867	114.538336	123.012414	105.312487	105.672264	101.92803	110.099331	112.430886	114.892127	0.777573947	-0.021986961	0.755563878	0.023636622
ENSG00000160917	CPSF4	29.320215	29.316554	30.132649	26.93736	25.607172	26.872285	28.048227	28.288962	28.959511	0.736997399	-0.032987643	0.797646009	0.023661346
ENSG00000107937	GTPBP4	21.812767	22.578533	22.68187	19.307809	19.106342	18.869005	20.871658	21.072541	22.08209	0.218378925	-0.081331358	0.761335899	0.023681392
ENSG00000070540	WIPI1	20.110322	17.58903	18.509469	15.21511	19.086948	16.677884	16.155561	18.210217	18.675756	0.938101317	0.013026389	0.845923319	0.023720401
ENSG00000112592	TBP	11.010969	11.026619	11.5878	12.217621	10.336014	11.260836	10.502228	10.427571	11.104053	0.163708642	0.156803325	0.846065263	0.023793333
ENSG00000150893	FREM2	4.119708	4.037366	4.375351	2.419804	2.194031	2.263876	3.889752	3.927346	4.131399	5.74E-26	-0.718729174	0.790456343	0.023804577
ENSG00000124574	ABCC10	6.171469	5.961748	5.317886	5.486081	5.254735	5.877472	5.210375	5.078823	6.422331	0.461920769	0.086862459	0.856243669	0.023840308
ENSG00000156232	WHAMM	2.744393	2.939517	3.289773	3.630579	3.379133	3.387156	2.921151	2.591368	3.054489	0.001499751	0.359140219	0.874214	0.023894145
ENSG00000134243	SORT1	39.157446	37.904823	37.095376	29.722337	31.421699	29.798259	33.782305	35.719408	39.282523	0.001668726	-0.182842398	0.766646116	0.023896276
ENSG00000143450	OAZ3	1.231293	1.109675	0.953024	0.782512	0.907194	1.136002	1.805682	0.878938	1.220737	0.764247373	-0.133883205	0.954382516	0.023939758
ENSG00000144224	UBXN4	50.994667	52.729188	51.984198	61.05168	58.612864	57.763278	48.335133	46.792344	51.102568	3.51E-12	0.328430269	0.729223213	0.02402874
ENSG00000105085	MED26	4.176276	3.810155	3.955672	4.66763	3.721343	4.473833	3.402538	3.523521	4.396148	0.029635235	0.290704253	0.885642971	0.02408702
ENSG00000148516	ZEB1	1.240375	1.521532	1.438463	2.593014	2.227002	2.124677	1.640127	1.307089	1.151631	5.72E-10	0.85841843	0.909274213	0.02409578
ENSG00000170677	SOCS6	5.311681	5.666148	5.639602	4.93281	4.593499	4.592622	5.262039	4.932981	5.680261	0.377497899	-0.09005157	0.822688248	0.02415435
ENSG00000105221	AKT2	31.953078	30.183105	29.020785	27.350976	28.47236	29.247011	27.004161	27.286469	30.660078	0.586085894	0.046955936	0.778155528	0.024179099
NewGene_886	--	2.015256	2.181986	1.848369	1.85132	1.514182	1.935757	1.369829	2.283517	2.087188	0.909231553	-0.042009537	0.941654809	0.024182907
ENSG00000151092	NGLY1	14.678446	14.999367	14.568574	13.987089	12.069902	12.149749	13.176345	13.075501	14.043976	0.927169578	-0.012513519	0.819036416	0.024215846
ENSG00000234906	APOC2	69.800757	59.520072	56.080126	58.367001	56.415062	62.995926	61.240481	66.534842	48.7967	0.507315693	0.079364999	0.878217781	0.024216637
ENSG00000132361	CLUH	29.325226	29.188166	25.91705	30.070617	29.155745	30.825181	25.760779	24.490118	29.517769	0.000100891	0.240376386	0.786234446	0.024295338
ENSG00000008277	ADAM22	2.786782	3.028921	2.807763	2.623034	2.361002	2.543893	2.750682	3.101548	2.980763	0.516051241	-0.081154928	0.84230252	0.024319927
ENSG00000119927	GPAM	6.986992	8.132895	6.484054	9.921485	9.313045	9.329224	7.095634	6.610352	6.931399	3.14E-11	0.548185695	0.837012702	0.024331825
ENSG00000142687	KIAA0319L	16.869498	14.201793	13.678141	12.62589	12.963932	12.465717	15.073824	13.81581	14.591345	0.517072688	-0.071236022	0.822913844	0.024383224
ENSG00000028839	TBPL1	3.669217	4.073622	4.152269	3.151292	2.959013	3.527152	4.026597	3.834561	4.596217	0.166872844	-0.181057071	0.86223767	0.024388459
ENSG00000182326	C1S	4.260213	3.889578	3.983199	80.007924	78.641705	77.88032	3.587114	3.774278	4.193707	0	4.365749951	0.879355906	0.024410264
ENSG00000183784	C9orf66	0.461589	0.426291	0.313453	0.254607	0.358819	0.460253	0.400414	0.348774	0.409088	0.98573325	-0.011601402	0.960099017	0.024418701
ENSG00000141349	G6PC3	26.419345	23.405297	23.638358	21.943555	19.524388	21.104748	22.04157	22.460976	24.115893	0.593938935	-0.063986539	0.834300782	0.024473398
ENSG00000066697	MSANTD3	17.263377	16.420459	16.965309	11.387585	10.566679	12.426505	16.439016	15.425218	15.975736	0.000244997	-0.364331622	0.833875744	0.024543063
ENSG00000126226	PCID2	38.647316	47.408854	42.655342	40.577368	34.209247	37.463996	43.512056	39.669126	44.388356	0.096147794	-0.115820182	0.768060956	0.024801647
ENSG00000197576	HOXA4	1.97046	2.159135	1.616628	1.364023	1.416977	1.374343	1.684272	1.933911	1.820417	0.503710866	-0.211402414	0.934855418	0.024825329
ENSG00000112685	EXOC2	15.089205	14.755931	14.797621	13.939264	13.374745	13.078258	14.12078	13.487793	15.108458	0.998487969	0.000307154	0.770152781	0.024932457
ENSG00000172766	NAA16	5.837126	6.130748	6.898199	6.429478	5.784683	5.804551	6.514262	5.212317	6.551401	0.564256798	0.073074425	0.855942756	0.025176062
ENSG00000033178	UBA6	12.539126	14.19348	14.600836	17.043195	16.3372	16.607594	13.085204	12.322388	14.170979	8.34E-12	0.425309638	0.783997161	0.025188227
ENSG00000163933	RFT1	6.12454	6.397653	6.160704	5.905574	5.756501	5.878337	5.557333	6.236838	6.704069	0.887849044	0.017569586	0.823230393	0.025438285
ENSG00000156469	MTERF3	9.197661	8.428253	9.315113	8.2077	7.893749	7.934601	7.825865	7.70237	9.704812	0.973662659	0.006857582	0.877861219	0.025484362
ENSG00000180357	ZNF609	9.133849	8.52735	8.868099	13.651663	11.703432	11.994535	8.898279	7.901985	8.571673	1.37E-22	0.641051438	0.776487881	0.02550115
ENSG00000165724	ZMYND19	25.348419	23.54929	23.915806	22.744047	22.827421	22.846241	22.990362	22.566553	23.961454	0.593500699	0.05750277	0.808848362	0.025540295
ENSG00000185158	LRRC37B	3.03559	2.825885	2.48924	2.472903	2.393209	2.6292	2.955788	3.296045	2.212736	0.804203592	-0.055793976	0.905636281	0.025566561
ENSG00000211584	SLC48A1	1.800859	1.763534	1.649034	1.033404	1.007875	1.114654	1.954048	2.108296	1.451367	0.018883495	-0.489935008	0.911701031	0.025711983
ENSG00000160199	PKNOX1	2.823286	2.681369	2.714862	2.901004	2.534565	2.866222	2.63618	2.519721	2.777983	0.318676377	0.139785396	0.857654023	0.025778971
ENSG00000121766	ZCCHC17	16.832407	15.7386	16.9193	13.147864	12.99176	14.063129	14.767293	16.721022	15.6263	0.197472764	-0.135674485	0.832486443	0.025821398
ENSG00000126216	TUBGCP3	16.186597	15.340067	17.300809	15.428726	13.689755	14.773575	17.189779	15.840136	17.954656	0.666313346	-0.045429446	0.787594038	0.025832863
ENSG00000158467	AHCYL2	1.678053	1.717519	1.912445	1.210656	1.463901	1.17196	1.779776	1.638401	1.65081	0.065058458	-0.318843224	0.885049652	0.025934915
ENSG00000118985	ELL2	26.599412	28.605782	28.963357	31.101683	30.889178	30.536589	28.763421	26.127574	28.118969	0.00000329	0.256953259	0.739817011	0.026010634
ENSG00000154719	MRPL39	27.024548	28.547932	26.718683	25.038688	22.387007	24.569509	27.181118	26.856	25.71051	0.481923739	-0.077106511	0.817781205	0.026067178
NewGene_4168	--	1.674863	1.453298	1.905203	1.114121	1.418103	1.810079	1.43334	2.024883	1.241419	0.920593913	0.034995032	0.934855418	0.026069372
ENSG00000119408	NEK6	24.276	24.400835	22.140937	21.431584	22.049857	21.714586	23.35175	21.22334	25.639386	0.976455272	-0.003225125	0.771667593	0.02607392

Gene ID	Gene name	Ctrl-1 FPKM	Ctrl-2 FPKM	Ctrl-3 FPKM	IFNγ-1 FPKM	IFNγ-2 FPKM	IFNγ-3 FPKM	Sora-1 FPKM	Sora-2 FPKM	Sora-3 FPKM	Ctrl vs IFNγ DESeq2 FDR	Ctrl vs IFNγ DESeq2 log2FC	Ctrl vs Sora DESeq2 FDR	Ctrl vs Sora DESeq2 log2FC
ENSG00000101901	ALG13	7.520559	8.5878	7.703606	6.675264	5.582266	6.572269	7.532157	7.111273	6.492605	0.524826017	-0.08774714	0.84934804	0.026082865
ENSG00000132819	RBM38	18.892237	19.558173	19.343697	13.728696	13.462036	14.576952	18.217188	16.507017	21.408387	0.0000253	-0.320092467	0.813986977	0.026135881
ENSG00000179195	ZNF664	57.373645	60.083528	60.933771	53.363171	52.695538	51.404864	58.749445	55.76411	59.497062	0.764369226	-0.022225524	0.710892805	0.026138908
ENSG00000183576	SETD3	15.077297	15.44285	15.997623	15.458667	15.051239	14.56651	15.06177	13.610681	15.99887	0.230928016	0.101562309	0.793434567	0.026143835
ENSG00000170876	TMEM43	19.684892	20.086026	21.295762	16.985089	16.715947	17.425988	17.897436	18.353964	22.160603	0.143955046	-0.11230917	0.801269982	0.026172904
ENSG00000137692	DCUN1D5	23.607214	23.212353	23.003454	23.743994	23.679717	22.540528	23.277816	22.960806	22.980698	0.309646596	0.103304464	0.806404352	0.026203913
ENSG00000075790	BCAP29	7.151345	7.844579	7.601031	6.537722	6.606511	6.512766	6.971507	8.51453	9.453968	0.477146229	-0.084313676	0.821956525	0.0262236
ENSG00000175416	CLTB	24.406351	23.97458	26.689855	14.866015	15.87533	17.757298	24.171629	23.90487	25.375915	7.36E-08	-0.47718512	0.80574384	0.026324695
ENSG00000167524	SGK494	1.515199	1.488886	1.831235	1.41669	1.236852	1.962619	1.849759	1.357919	1.3705	0.779727836	0.125769892	0.947483358	0.026350252
ENSG00000072274	TFRC	125.874434	128.566651	126.068323	103.814949	100.71722	103.46555	117.765103	118.380688	127.792282	0.000382685	-0.160296504	0.682558864	0.026361575
ENSG00000167535	CACNB3	0.588268	0.394476	0.519761	0.3891	0.252826	0.273875	0.769204	0.328892	0.383952	0.452750368	-0.382729157	0.958374556	0.026393951
ENSG00000137693	YAP1	35.940346	37.26586	52.87098	25.972608	27.797621	31.069644	35.110821	46.867591	38.693333	0.00000679	-0.342095463	0.798132264	0.026490385
NewGene_4776	--	4.480828	4.666054	5.023289	5.920271	6.17833	5.184555	5.422603	4.238209	4.156304	0.00000096	0.499211993	0.857727917	0.0264979
ENSG00000126261	UBA2	80.072781	80.693543	83.575183	67.226053	65.587475	66.722715	76.865056	77.37554	80.865976	0.001995323	-0.157269669	0.697750171	0.026605577
ENSG00000168385	2-Sep	143.846805	154.656102	155.570063	144.47472	145.983993	144.524042	142.81894	134.996242	158.284614	0.129333769	0.082791937	0.689541448	0.026673745
ENSG00000123395	ATG101	24.105753	22.500405	22.683664	17.14487	16.63035	18.61097	19.459499	22.946841	21.929647	0.014630862	-0.227259549	0.826359998	0.026703125
ENSG00000160075	SSU72	116.790143	115.076185	115.915728	113.256487	112.172978	116.68364	111.889621	116.167516	127.395614	0.042271099	0.11473827	0.724621332	0.026782189
ENSG00000171365	CLCN5	5.806338	5.451438	3.588953	5.620174	5.659734	3.764304	4.942326	4.638006	4.396213	0.004879872	0.235986578	0.798795546	0.026962169
ENSG00000134375	TIMM17A	43.476185	42.671043	47.543697	43.02002	44.301212	41.869812	42.29578	41.440762	44.023029	0.230928016	0.098022585	0.765240502	0.027013493
NewGene_6553	--	2.688102	3.282768	3.084117	3.069912	2.821633	2.807417	3.024609	2.688236	4.327997	0.313559047	0.097714398	0.784026802	0.027084628
ENSG00000136997	MYC	73.403763	70.799282	74.39821	88.019339	85.732097	85.441096	60.525979	66.20509	82.811989	1.39E-15	0.397833734	0.806625361	0.027221508
ENSG00000138398	PPIG	23.573806	26.826207	27.59473	19.630508	23.730078	22.292422	24.295373	23.403288	22.825416	0.693189212	0.039291378	0.769920305	0.027292618
ENSG00000161328	LRRC56	2.618431	2.06678	2.205771	2.027022	2.253578	1.88065	2.212989	1.904112	2.503731	0.95047057	-0.017479743	0.900639897	0.027449544
ENSG00000275718	CCL15	15.089393	15.511927	11.341682	17.599526	17.455117	18.475466	12.647416	14.043892	12.581241	0.0000516	0.520123757	0.877927524	0.027696858
ENSG00000134900	TPP2	23.258991	24.448607	23.26812	23.969694	23.28413	21.300861	23.640919	22.318388	24.828709	0.073682664	0.124333031	0.721146329	0.027826187
ENSG00000185090	MANEAL	11.262812	12.765926	12.550857	10.192642	9.458078	10.255599	11.820818	10.277891	12.874741	0.16740702	-0.140866793	0.810939169	0.027884934
ENSG00000170542	SERPINB9	3.65681	3.393347	3.692469	2.773994	2.245547	2.766656	3.509425	3.33354	3.431483	0.017610986	-0.31651257	0.843316277	0.028059729
ENSG00000129484	PARP2	22.988362	26.64412	24.410856	21.527071	18.984563	19.919617	24.615058	23.702489	22.260178	0.11555196	-0.147139076	0.800004787	0.028157832
ENSG00000153310	FAM49B	35.5227	43.251068	40.434504	32.900885	30.094346	28.497384	35.853347	39.286047	38.722139	0.000147987	-0.24438455	0.741167578	0.028254849
ENSG00000125354	6-Sep	6.458716	6.280471	6.950158	11.167917	9.550708	11.621042	6.298283	6.055082	6.252005	1.63E-30	0.893698565	0.807044216	0.028266779
ENSG00000055208	TAB2	16.53153	16.761609	16.803441	17.763882	17.466736	16.343169	17.086525	14.628753	16.238539	0.00498634	0.188258483	0.758443078	0.028295622
ENSG00000084754	HADHA	36.059725	34.908709	32.721475	29.444531	31.449706	31.573284	32.844346	32.325457	33.521458	0.986113038	0.001840225	0.725884388	0.02829635
ENSG00000163956	LRPAP1	16.036175	14.950073	15.824744	15.985108	15.942792	12.423074	14.644294	16.164002	16.611896	0.124447183	-0.109009794	0.744984343	0.028301677
ENSG00000164754	RAD21	94.58785	95.217907	100.853763	86.752482	86.504456	87.042198	94.002978	90.972649	95.23112	0.866827514	-0.012704682	0.679660215	0.028351923
ENSG00000182518	FAM104B	4.67616	3.946838	3.580893	2.789295	3.411808	2.956378	4.471082	3.887502	3.363134	0.29958674	-0.252474757	0.912207637	0.028379496
ENSG00000167182	SP2	7.578011	7.238182	7.552683	6.601187	6.976903	6.967543	7.029213	6.492189	7.142002	0.649178428	0.060206333	0.818362661	0.028477588
ENSG00000115339	GALNT3	5.308494	6.446974	6.459636	3.083663	3.282696	3.299628	5.542474	5.324984	6.545841	1.71E-12	-0.770648794	0.838782535	0.028516489
ENSG00000131473	ACLY	116.719294	112.479759	113.204781	136.625699	143.968086	133.997272	108.069597	104.552546	115.13896	1.92E-17	0.415830609	0.661315777	0.028529368
ENSG00000153707	PTPRD	0.295478	0.183323	0.30189	0.213774	0.257337	0.178933	0.212164	0.401125	0.220955	0.815713715	-0.095116789	0.937259399	0.028568592
ENSG00000175662	TOM1L2	3.818776	3.436514	3.606724	3.452726	3.617877	3.857948	3.75761	2.80801	4.151542	0.262738212	0.143190398	0.856285443	0.028571845
ENSG00000066557	LRRC40	9.524723	11.431005	11.342038	10.227395	9.360866	9.137766	10.50729	10.029153	10.348496	0.88148001	-0.022057439	0.814411772	0.02877614
ENSG00000230797	YY2	0.55017	0.461045	0.606898	0.737752	0.325895	0.365811	0.458622	0.616359	0.452174	0.959770197	-0.031787637	0.946465498	0.028908432
NewGene_747	--	9.612561	9.176578	12.050642	4.857876	5.011167	5.739149	11.736857	13.961689	10.52524	4.84E-09	-0.791450306	0.87387321	0.028953023
ENSG00000117751	PPP1R8	21.749945	21.579543	21.405986	19.284987	18.706295	19.059274	21.630991	20.971436	19.220392	0.69745326	-0.037536387	0.790393539	0.029013424
ENSG00000265491	RNF115	6.406013	6.616481	7.260481	8.486994	8.151429	8.080667	6.708386	5.949192	6.770277	1.18E-10	0.432480639	0.767715478	0.029174238
ENSG00000171861	MRM3	13.681988	12.800459	14.879332	13.080452	12.790653	13.157527	12.367483	11.68451	15.256902	0.666094926	0.056133213	0.833510634	0.029276004
ENSG00000132837	DMGDH	1.073729	1.221808	1.279563	1.366212	1.24163	0.763376	1.285407	1.002635	1.162038	0.861645551	0.06508153	0.915101678	0.029286561
ENSG00000135913	USP37	3.44077	3.331734	4.277083	4.627903	4.178161	4.035829	3.191452	3.883541	3.085471	0.00250038	0.288745312	0.814123475	0.029293196
ENSG00000167522	ANKRD11	22.369311	21.300732	19.624322	21.441968	18.71525	20.66463	20.019736	19.564537	22.711769	0.049088918	0.144350833	0.768327487	0.029443162
ENSG00000107443	CCNJ	10.538578	11.61266	12.065594	10.620209	10.214549	10.14766	11.329197	9.704753	10.770069	0.631628789	0.051974607	0.788943124	0.029458374
ENSG00000130305	NSUN5	21.320278	21.219463	20.43369	17.427446	17.680831	18.179966	20.163134	19.475759	20.222537	0.421772935	-0.078825263	0.766376918	0.029515211
NewGene_3750	--	1.38827	1.127688	1.482178	1.089447	1.27197	1.397928	1.286282	1.27041	1.825178	0.955226678	0.019085619	0.908115545	0.029568802
ENSG00000102302	FGD1	1.122723	1.245849	0.944018	0.918174	0.808339	0.892734	0.904868	1.082323	1.183961	0.450326648	-0.189310256	0.906232355	0.029658629
ENSG00000059122	FLYWCH1	2.199237	2.171199	1.934949	1.855689	1.715709	2.366385	1.911782	1.749487	2.266351	0.962852033	0.010564466	0.874214	0.02970502
ENSG00000151148	UBE3B	9.631637	9.471178	8.79247	8.840866	7.986841	8.256939	8.72605	7.810045	9.566863	0.890046226	0.015549765	0.785526317	0.029744639
ENSG00000213341	CHUK	16.226992	17.608885	18.359175	16.262289	16.886372	15.85564	16.043674	16.634624	17.275969	0.555296026	0.056710904	0.756486364	0.029745257
ENSG00000111696	NT5DC3	2.462819	2.501081	2.807115	2.491043	2.358857	2.322561	2.594644	2.20597	2.707376	0.894794052	0.021621828	0.830244127	0.02978567
ENSG00000163539	CLASP2	5.568587	5.862252	5.947871	4.992411	4.882524	4.503436	5.58164	5.544738	5.664524	0.180426286	-0.123296297	0.761877501	0.029811682
ENSG00000095383	TBC1D2	3.90617	3.90496	3.364646	4.188261	5.614064	4.460433	3.425947	3.35584	4.070825	0.000161553	0.518166452	0.850606257	0.029885001
ENSG00000166024	R3HCC1L	4.566774	4.453872	5.032291	4.510438	4.564074	3.972159	4.507464	4.190334	4.985284	0.803266367	0.041074557	0.831247731	0.029928686
ENSG00000105325	FZR1	9.662203	8.746352	9.476024	8.590404	7.601154	8.537108	8.520516	7.814229	9.624441	0.889945453	0.016445778	0.772834771	0.029935583
ENSG00000173171	MTX1	42.310469	34.127703	33.550214	27.725608	31.926105	29.013802	31.357632	32.620023	40.638369	0.659905817	-0.052822104	0.792598574	0.030084812
ENSG00000117859	OSBPL9	86.784549	90.462023	94.848656	76.491958	81.428369	78.34514	84.169298	85.879512	90.323161	0.418979827	-0.05987607	0.687390183	0.030185314
ENSG00000116014	KISS1R	12.883095	12.352617	10.082181	8.805187	9.049822	10.422399	11.605254	10.40607	12.32285	0.198654389	-0.190031704	0.84104676	0.030264574
ENSG00000211455	STK38L	9.118733	10.590227	9.979498	7.60439	8.061601	7.222463	8.995815	8.853129	10.400852	0.015748808	-0.220637979	0.780033179	0.030271512
ENSG00000006007	GDE1	15.802667	17.144212	16.414253	14.84402	15.827966	15.497085	15.55241	15.08566	16.84623	0.61623452	0.050359069	0.749867046	0.030406247
ENSG00000137713	PPP2R1B	10.730808	11.367975	10.869159	14.831265	14.830769	15.966396	11.161078	10.531869	11.904949	2.45E-11	0.448873731	0.74696657	0.03042797
ENSG00000169612	RAMAC	22.645893	21.470358	22.71788	22.023039	18.61776	19.773808	20.172178	20.915607	22.987617	0.999202724	0.000252737	0.773864157	0.030540068
ENSG00000169902	TPST1	13.432822	13.604182	14.302043	11.086371	10.585848	11.016323	12.175143	12.609429	14.834584	0.052054962	-0.184146535	0.796266079	0.030585431
ENSG00000125885	MCM8	6.2351	6.70835	7.075214	5.931336	5.404235	5.386207	6.488014	6.821289	7.243048	0.250982286	-0.115202878	0.777054786	0.030674459
ENSG00000198722	UNC13B	8.26329	7.398962	6.661132	5.124244	5.279133	4.78869	7.298336	6.54782	7.50872	0.0000121	-0.405667947	0.790456343	0.030682113
ENSG00000138160	KIF11	14.137207	17.078571	16.687727	13.679298	11.988585	12.410184	16.033056	13.844097	16.054438	0.040031296	-0.184133391	0.775692	0.030696444
ENSG00000130254	SAFB2	28.24538	26.413156	26.757863	26.56877	24.546179	26.517155	25.086851	25.065572	28.322664	0.293506594	0.079555087	0.725035629	0.030716931
ENSG00000196498	NCOR2	7.818607	9.495404	8.454676	6.800277	7.27447	6.982824	7.80112	6.909942	8.29602	0.769170906	-0.033465262	0.798657309	0.030764187

Gene ID	Gene name	Ctrl-1 FPKM	Ctrl-2 FPKM	Ctrl-3 FPKM	IFNγ-1 FPKM	IFNγ-2 FPKM	IFNγ-3 FPKM	Sora-1 FPKM	Sora-2 FPKM	Sora-3 FPKM	Ctrl vs IFNγ DESeq2 FDR	Ctrl vs IFNγ DESeq2 log2FC	Ctrl vs Sora DESeq2 FDR	Ctrl vs Sora DESeq2 log2FC
ENSG00000145715	RASA1	6.903227	7.689313	7.990268	7.466014	7.277733	6.456517	8.111017	7.045237	6.765002	0.304666478	0.107200737	0.76657123	0.030829937
ENSG00000118137	APOA1	297.998917	255.537377	190.990895	210.402232	204.851112	218.054813	257.01475	240.100527	214.621378	0.738990656	-0.087508851	0.911839206	0.030988603
ENSG00000135617	PRADC1	20.534079	17.600748	15.928535	15.473461	16.457066	16.965887	16.966534	18.17684	16.59469	0.99155889	0.002202796	0.839845341	0.030993708
ENSG00000072518	MARK2	19.782723	22.647765	18.480176	19.076599	20.519063	19.297182	21.31064	17.507577	25.242686	0.158570683	0.117300062	0.760696683	0.031142312
ENSG00000171155	C1GALT1C1	19.243746	16.795311	19.142597	17.455931	18.704587	17.024406	16.513596	18.102816	18.256999	0.420487175	0.093827864	0.79612223	0.031466195
ENSG00000181409	AATK	0.658376	0.556659	0.43654	0.826759	1.070577	1.00023	0.594267	0.360571	0.638846	0.0000495	0.887612285	0.925955419	0.031799427
ENSG00000047634	SCML1	9.693839	11.205797	11.657997	19.975823	19.312955	17.736766	9.889638	9.378619	10.230087	1.38E-25	0.956818013	0.807044216	0.031851731
ENSG00000188130	MAPK12	13.914814	14.395461	13.163773	12.770934	12.867896	12.970441	13.022057	12.918284	13.599532	0.804203592	0.031724124	0.781719258	0.031855166
ENSG00000112679	DUSP22	8.752056	7.616412	7.602129	8.014632	7.255508	7.380887	7.398554	8.109195	7.123058	0.097730513	0.194529193	0.828187992	0.031908427
ENSG00000128059	PPAT	22.860941	27.176828	28.648817	26.379912	24.442616	25.288682	24.566194	23.452898	27.223094	0.347127612	0.092135861	0.767715478	0.031971705
ENSG00000116455	WDR77	19.701816	22.740589	21.555269	17.350805	16.025439	16.833686	20.018739	20.401012	22.384691	0.019184228	-0.195063302	0.755099276	0.03205533
ENSG00000163785	RYK	38.422012	43.104951	45.967767	27.974722	26.818757	25.842541	37.868136	38.792742	44.354983	1.34E-13	-0.523258057	0.75038549	0.032106069
ENSG00000171224	FAM241B	6.373741	5.190111	5.278983	3.604761	3.441464	3.548514	5.801095	3.622087	6.560658	0.009842158	-0.508291835	0.902941106	0.032260939
ENSG00000119599	DCAF4	2.007092	1.255565	1.513064	1.825866	1.191058	1.264609	2.007439	1.204716	1.228563	0.771766812	0.10345744	0.921143129	0.03230278
ENSG00000127483	HP1BP3	42.421823	46.001967	47.138629	42.507031	46.483239	41.157521	45.610385	41.761897	44.682051	0.981497638	-0.002447039	0.69391206	0.032516915
ENSG00000126217	MCF2L	9.561976	8.526023	7.607582	9.995106	9.250454	9.357991	7.918748	7.000302	9.431802	0.668989193	0.047305491	0.797859533	0.032517051
ENSG00000123297	TSFM	17.561052	21.28969	17.276194	16.666702	15.37647	16.019652	16.592235	18.234539	19.595224	0.982925209	0.002869091	0.757882483	0.032538698
ENSG00000135931	ARMC9	2.224834	1.742279	1.796439	1.359637	1.134043	1.263774	1.788295	1.643922	1.92451	0.068097874	-0.436793168	0.893310053	0.032719028
ENSG00000091490	SEL1L3	55.018651	56.208486	63.356501	30.135694	29.703964	27.928366	54.101715	53.879805	59.767734	3.08E-46	-0.848204984	0.691592735	0.032873345
ENSG00000184432	COPB2	65.249512	66.897015	62.590349	73.498973	75.27218	75.446561	59.528256	62.483987	63.303238	7.77E-12	0.346402943	0.658753477	0.032886428
ENSG00000139675	HNRNPA1L2	4.444798	4.425663	4.658069	4.245712	4.546359	4.480104	4.402078	4.538579	4.015002	0.563695043	0.120785433	0.875711165	0.03289997
ENSG00000198055	GRK6	12.645315	11.262664	11.665471	13.021052	11.832921	12.381539	11.669231	10.545546	12.521681	0.076793417	0.165545726	0.77529396	0.032920052
ENSG00000178691	SUZ12	23.97762	26.552455	28.708877	25.437383	23.744655	24.482589	25.351543	23.873561	26.264998	0.61623452	0.048848781	0.729899633	0.032953432
ENSG00000112578	BYSL	26.187655	24.687116	23.418555	19.174742	18.655002	18.827309	23.736619	24.310541	27.879372	0.000417308	-0.263743189	0.727225191	0.033038829
ENSG00000136950	ARPC5L	41.141356	38.338978	39.714974	37.149064	35.095346	39.299528	38.641814	37.702136	38.344601	0.398041846	0.072537476	0.704349564	0.033071601
ENSG00000166974	MAPRE2	5.12094	5.272912	5.077199	4.961658	5.04522	5.546588	4.626579	5.176625	5.220397	0.175037187	0.148513136	0.79391381	0.033123339
NewGene_5964	--	10.655153	7.458009	9.767171	7.927126	7.252424	8.072091	9.403628	9.094244	10.722334	0.930136318	-0.012338148	0.773358799	0.033132857
ENSG00000100296	THOC5	40.876059	41.883656	43.166159	36.616616	35.954856	36.176943	41.671276	42.981946	41.638824	0.173021468	-0.090789842	0.681765222	0.033213693
ENSG00000117385	P3H1	15.426316	13.20228	14.181001	12.57093	14.167111	13.08741	12.984911	13.278031	14.574518	0.680498731	0.050512837	0.757938179	0.033226415
ENSG00000198176	TFDP1	95.742246	100.191253	108.103112	95.688787	93.526582	94.945054	95.438929	91.176543	105.445409	0.51269845	0.048838827	0.682480807	0.033384072
ENSG00000144724	PTPRG	5.211962	5.694993	5.679286	5.024291	4.581447	4.484693	5.01953	5.487921	5.508395	0.556247988	-0.060428225	0.737020796	0.03345751
ENSG00000154511	DIPK1A	6.008553	5.799933	6.262566	5.224848	6.253967	5.791243	6.935776	7.473033	6.423469	0.478763256	0.108829711	0.839845341	0.033537111
ENSG00000272047	GTF2H5	2.611966	2.558775	2.330903	1.83463	8.324673	7.345995	2.306541	2.416254	2.178327	0.078678843	-0.235201303	0.844318037	0.03362296
ENSG00000133812	SBF2	7.330136	7.270721	7.127287	6.714429	5.839358	6.028996	7.009603	6.938827	7.259601	0.302347122	-0.091918159	0.699978828	0.033649727
ENSG00000135404	CD63	344.696631	314.823025	319.569008	285.162472	289.021943	289.53974	303.178862	346.419076	316.766444	0.724338191	-0.027150826	0.660591165	0.033733996
ENSG00000177479	ARIH2	23.593486	22.598793	24.769175	22.84948	21.622915	19.201299	25.20814	24.560801	24.908519	0.230889478	-0.084597315	0.682558864	0.033742657
ENSG00000167088	SNRPD1	15.686784	16.503349	16.751275	14.849061	14.45094	15.114916	15.946621	15.943598	15.112915	0.983723075	0.002133706	0.708362463	0.033790274
ENSG00000105928	GSDME	11.48003	11.067965	10.108635	9.407049	9.016954	9.397703	11.213842	10.253018	10.543343	0.289898282	-0.125381301	0.786193849	0.033816798
ENSG00000061273	HDAC7	9.072631	8.472475	8.59867	10.045333	8.404505	10.438417	7.565539	7.808484	9.758251	0.000741822	0.306689694	0.788014843	0.033853525
ENSG00000100714	MTHFD1	33.22933	32.858074	34.804211	33.96433	30.120805	31.191059	32.466884	32.769734	31.236973	0.405245978	0.064782657	0.694398773	0.03390655
ENSG00000099308	MAST3	3.285458	3.265883	3.155654	4.053233	3.453815	3.204448	3.108879	2.891614	3.324612	0.011257068	0.287162906	0.788014843	0.033907994
ENSG00000085998	POMGNT1	9.528832	9.609449	8.584374	6.403322	5.283149	6.229454	7.881103	8.79181	10.004395	0.00000225	-0.475632865	0.795607551	0.033984088
ENSG00000166896	ATP23	3.777611	3.482104	3.536855	3.449277	3.297216	2.943135	3.491929	3.835747	2.989119	0.937840234	-0.023719018	0.892048046	0.034041428
ENSG00000179456	ZBTB18	4.645675	4.413186	4.022828	5.047803	5.190652	4.641244	4.407287	4.124561	3.931988	0.003200727	0.331266148	0.813450975	0.034067995
NewGene_656	--	16.919285	16.685592	19.638044	16.513326	16.859347	13.456461	17.735443	19.965561	19.86779	0.475430489	-0.08209766	0.763410877	0.034311925
ENSG00000105976	MET	29.619639	30.975377	28.743425	33.34962	31.608187	31.743251	28.024106	26.766303	31.735764	0.000000522	0.257623089	0.661815612	0.034634995
ENSG00000165338	HECTD2	3.26211	3.346056	3.23673	3.448945	3.399861	3.004272	3.113761	3.090249	3.105276	0.25272138	0.151589489	0.805229404	0.034672574
ENSG00000174529	TMEM81	1.30765	1.232363	1.350048	0.844152	0.69396	0.859699	1.184624	1.343423	1.214909	0.133779678	-0.555835971	0.925827355	0.034720594
ENSG00000112818	MEP1A	20.037837	19.631506	15.074373	17.678623	17.549326	17.863002	18.496061	18.276932	15.389592	0.37233207	0.10795585	0.813562594	0.034732166
ENSG00000139112	GABARAPL1	6.128128	5.829788	5.775556	7.690947	7.200291	7.128098	5.966117	5.625557	4.771295	0.000015	0.48400349	0.843131841	0.034739646
ENSG00000166387	PPFIBP2	6.16452	6.186542	5.1331	5.873562	4.918828	5.840609	5.889	5.539865	6.241993	0.801532646	0.040946572	0.802271485	0.034789955
ENSG00000130193	THEM6	18.42136	18.843491	15.842385	17.456116	17.170162	18.110216	16.318661	17.135721	17.56307	0.166091016	0.137447885	0.758652942	0.034912338
ENSG00000172113	NME6	6.717844	8.213525	7.691925	7.169462	6.720711	7.336349	7.169501	7.668853	8.427823	0.535208221	0.080091177	0.778108243	0.034933125
ENSG00000157216	SSBP3	9.55492	8.690348	8.455658	6.873996	7.956005	7.786069	9.777819	8.556774	8.688727	0.308723717	-0.123552789	0.761335899	0.034938814
ENSG00000204172	AGAP9	6.461365	6.272593	6.474702	8.282447	7.202414	7.391516	6.920875	5.292474	6.261319	0.000166053	0.398175196	0.818306581	0.034996509
ENSG00000162980	ARL5A	20.683121	23.860152	24.186366	18.247112	18.322531	18.703089	22.637971	21.005748	22.814174	0.05720597	-0.159436637	0.725683559	0.035010063
ENSG00000066827	ZFAT	4.425132	4.204376	4.112064	3.083494	3.5949	3.409151	3.893703	3.987862	4.45424	0.171103869	-0.173513713	0.781055838	0.035202665
ENSG00000166073	GPR176	1.320683	1.174784	1.163502	0.766001	0.883268	0.738853	1.164893	0.994735	1.389093	0.039357839	-0.464080304	0.885483662	0.035232264
ENSG00000134569	LRP4	0.733509	0.806449	0.699716	0.641052	0.642253	0.64351	0.796501	0.660475	0.693636	0.772230526	-0.069443373	0.869068091	0.035322611
ENSG00000129933	MAU2	14.022091	13.325238	13.167674	12.459526	12.57266	12.764232	12.785306	12.020232	14.231415	0.587894819	0.047500273	0.695034892	0.035328566
ENSG00000132600	PRMT7	5.501969	5.320478	6.477603	6.401741	6.208378	6.325785	6.653321	4.957679	6.690196	0.953310197	-0.010164009	0.796211572	0.035511386
ENSG00000115380	EFEMP1	1.382845	1.228911	0.990621	0.682088	0.328231	0.52126	1.248036	1.076169	1.088909	0.0000564	-1.122617149	0.900444707	0.035540958
ENSG00000123374	CDK2	39.491687	36.026986	38.225532	30.418631	29.392883	29.50772	35.657236	35.86764	35.80364	0.007346536	-0.178787397	0.673156336	0.035607821
ENSG00000066926	FECH	8.357501	8.858951	7.967557	7.212425	6.422022	7.140683	7.614655	7.14904	8.277838	0.165520459	-0.141454101	0.768970706	0.035681657
ENSG00000144712	CAND2	1.058013	1.245715	0.965503	3.791071	4.06361	4.298881	0.917968	1.010816	1.212796	2.77E-57	2.035316265	0.882846617	0.035686805
ENSG00000145901	TNIP1	13.028668	11.314908	11.124673	10.456687	9.81621	11.099411	10.270379	11.564579	11.588855	0.856258877	-0.025825233	0.777022818	0.035739627
ENSG00000163864	NMNAT3	6.078069	6.781207	5.498535	5.041977	4.437544	5.630681	6.326999	5.286093	6.499826	0.018531777	-0.307744997	0.814059029	0.035806249
ENSG00000057757	PITHD1	22.516883	23.469929	23.374998	20.664002	19.27075	18.648925	22.3884	21.772888	22.206705	0.281830379	-0.101905851	0.717686557	0.035946275
ENSG00000185332	TMEM105	0.667445	0.550911	0.502387	0.426951	0.298731	0.386848	0.566049	0.526356	0.575639	0.19838059	-0.475515089	0.919718748	0.036066485
ENSG00000102554	KLF5	2.312647	2.392992	2.7988	2.247891	2.495627	2.409832	2.159484	2.225956	2.843637	0.685663623	0.080907216	0.851939602	0.036194616
NewGene_1857	--	1.594001	1.21019	1.458239	1.960911	1.538204	1.795897	1.175725	1.633777	1.499854	0.148824383	0.295681418	0.877861219	0.036239447
ENSG00000146242	TPBG	2.2537	2.058897	1.9433	1.475934	1.54338	1.794518	2.186928	2.828394	1.983838	0.000867516	-0.458544769	0.807044216	0.036350455
ENSG00000113889	KNG1	41.742196	40.61203	37.291112	25.812323	27.370316	25.842095	41.783817	39.644846	36.538226	1.46E-14	-0.442428974	0.709844881	0.036418041
ENSG00000124145	SDC4	66.688934	64.46257	62.252922	43.978001	44.527916	47.390617	62.020199	60.653717	63.355133	7.01E-10	-0.361307065	0.613948872	0.036437108

Gene ID	Gene name	Ctrl-1 FPKM	Ctrl-2 FPKM	Ctrl-3 FPKM	IFNγ-1 FPKM	IFNγ-2 FPKM	IFNγ-3 FPKM	Sora-1 FPKM	Sora-2 FPKM	Sora-3 FPKM	Ctrl vs IFNγ DESeq2 FDR	Ctrl vs IFNγ DESeq2 log2FC	Ctrl vs Sora DESeq2 FDR	Ctrl vs Sora DESeq2 log2FC
ENSG00000165732	DDX21	52.411159	59.129122	63.430543	56.203599	53.982363	55.750986	55.292803	53.569055	59.482022	0.412939124	0.069138548	0.68538727	0.036485302
ENSG00000143252	SDHC	26.238572	24.322027	18.970575	20.638675	23.624257	21.44355	24.958017	23.714827	28.53425	0.899142427	-0.012023328	0.643425318	0.036488463
ENSG00000121644	DESI2	21.103867	20.311337	22.576023	16.477603	16.395842	16.252449	19.466881	19.679361	22.593704	0.000910217	-0.220841394	0.685449504	0.036495746
ENSG00000101464	PIGU	22.846696	20.653482	20.119005	16.624379	17.15675	16.057693	19.958092	20.077078	20.744905	0.040827567	-0.194107523	0.733099897	0.036508789
NewGene_4751	--	1.008145	1.375582	1.625524	1.356954	1.038061	1.071329	1.152341	1.247264	1.405419	0.834585282	-0.069272308	0.892261682	0.036805625
NewGene_309	--	33.052612	34.116784	35.017543	26.815407	23.782076	26.727405	32.915719	32.850463	33.72055	0.00000507	-0.299030065	0.646485993	0.036823909
ENSG00000107581	EIF3A	50.446691	50.923891	49.961127	48.130056	44.391435	45.631922	47.703494	46.647853	51.314871	0.828374963	0.015564284	0.563571388	0.036932178
ENSG00000130703	OSBPL2	12.143366	10.448024	12.608577	13.270698	12.11795	13.679909	11.171367	10.588666	12.477158	0.008179741	0.231628635	0.735866738	0.036941567
ENSG00000176371	ZSCAN2	13.963716	11.936706	13.61888	11.509057	12.854108	13.384902	14.318026	10.565725	11.042407	0.717385568	0.044162814	0.767757317	0.036982519
ENSG00000079819	EPB41L2	78.026886	76.003015	79.974388	72.489269	63.489752	66.721271	75.06815	71.644503	78.258734	0.574667169	-0.042977492	0.587194845	0.037009203
ENSG00000105204	DYRK1B	21.434831	22.107871	19.772349	20.84404	20.294759	20.403113	20.738075	19.664651	20.478476	0.304610409	0.088543994	0.688877457	0.037138034
ENSG00000168509	HJV	3.87441	3.627258	2.838754	2.540221	2.69633	3.06071	3.197459	3.378673	2.775397	0.529990809	-0.135204045	0.856119665	0.037220601
ENSG00000135334	AKIRIN2	23.185955	22.53676	24.37822	21.578672	22.201857	21.093805	23.899393	21.266794	22.298265	0.752056319	0.035235051	0.721198952	0.037289341
ENSG00000165389	SPTSSA	18.206991	18.829832	21.504047	18.268091	17.363029	16.522774	19.550085	18.462856	18.269848	0.876472148	-0.019502621	0.729899633	0.037324594
ENSG00000144320	LNPK	3.588442	4.297556	4.12412	3.834782	3.714433	3.657819	3.881112	3.763379	4.106108	0.837221361	0.028255247	0.751995229	0.037365553
ENSG00000105726	ATP13A1	23.277941	21.539636	21.402398	18.998376	18.276598	19.478088	20.608751	19.259438	24.129432	0.304284041	-0.081134994	0.698055554	0.037425542
NewGene_6666	--	3.901802	3.646016	3.437251	3.183858	3.04821	3.116762	3.770146	4.093137	4.137481	0.013226408	-0.262394015	0.763100023	0.037499327
ENSG00000122335	SERAC1	3.27217	3.36464	3.707941	2.343849	2.553952	2.139712	3.488191	3.489791	2.775723	0.010001888	-0.354899249	0.810006143	0.037554975
ENSG00000102934	PLLP	1.899602	1.952625	1.326333	1.077852	1.168716	1.212668	1.538677	1.363822	1.58242	0.461031705	0.255792363	0.90692555	0.037604318
ENSG00000152894	PTPRK	26.047223	27.808984	27.528713	27.026542	25.246236	24.817285	26.744113	25.845549	28.663853	0.699387101	0.030032981	0.609056732	0.037617348
ENSG00000143553	SNAPIN	37.386211	34.186947	32.489983	29.940796	29.062756	30.348148	33.221165	34.200169	32.691311	0.509053932	-0.073056116	0.739790724	0.037636636
ENSG00000116741	RGS2	37.551193	33.988953	34.61787	23.162685	24.252569	23.880228	33.026749	34.588299	34.534534	4.42E-08	-0.427200674	0.705192609	0.037735196
ENSG00000166037	CEP57	14.702117	14.684261	16.451841	14.484316	12.573705	13.927084	14.062771	14.010007	16.304823	0.801768741	-0.029436346	0.720169021	0.037794085
ENSG00000115942	ORC2	16.414595	16.650208	17.998716	14.749318	13.753506	14.465745	17.466603	15.991524	16.024807	0.196362998	-0.102001319	0.687048488	0.037824327
ENSG00000112697	TMEM30A	45.133446	52.562217	56.094168	47.387041	48.586806	49.413024	48.019231	45.688957	54.550196	0.525988493	0.061029329	0.708356554	0.037927816
ENSG00000005436	GCFC2	11.464328	9.747984	11.531884	11.160459	8.661847	8.56821	8.859007	11.250462	10.859291	0.363400008	-0.10039916	0.740294019	0.037963813
ENSG00000150403	TMCO3	32.534308	31.017737	31.205996	28.212539	27.097283	27.199549	31.104662	29.649315	30.479282	0.461920769	-0.055639201	0.629505231	0.037988805
ENSG00000163466	ARPC2	147.534708	149.414953	152.742755	126.7107	125.183371	126.331189	140.103074	139.408184	154.124287	0.100746975	-0.094104983	0.583272361	0.03802136
ENSG00000162623	TYW3	6.61599	7.876422	8.347463	6.173396	6.218103	5.553526	7.682033	7.23719	7.13041	0.077616521	-0.212375361	0.777022818	0.038050996
ENSG00000147853	AK3	74.90618	86.006176	85.12673	91.125939	88.153725	90.544135	81.319519	76.981891	84.41768	0.00000231	0.283553822	0.63874282	0.038176667
ENSG00000025039	RRAGD	5.707304	6.083982	6.056528	6.03474	5.829939	5.663205	6.242537	5.77951	5.133202	0.215584269	0.120891103	0.767715478	0.038292283
ENSG00000118193	KIF14	5.998708	7.754857	7.767322	5.8778	4.62479	5.207413	7.061513	6.1639	7.720239	0.006151043	-0.309110908	0.768536497	0.038349124
ENSG00000114867	EIF4G1	155.363747	148.878148	150.176173	159.035823	147.305169	157.760305	142.605985	134.714957	164.373234	0.000153015	0.191487938	0.618487461	0.03841131
ENSG00000111181	SLC6A12	6.746124	8.358928	7.310995	6.095248	7.872088	6.362265	7.835795	7.87993	7.330212	0.803266367	0.041446095	0.788414179	0.038591263
ENSG00000089818	NECAP1	8.319389	9.535892	9.07001	10.949401	10.113945	10.65043	8.941875	9.454139	8.464761	0.0000842	0.358026281	0.7627687	0.038881345
ENSG00000136738	STAM	10.72748	11.097354	11.529117	7.753776	7.612976	6.918749	10.740493	10.36391	10.779507	0.00000067	-0.431342455	0.705616373	0.038887046
ENSG00000116266	STXBP3	8.58204	8.273773	8.040829	7.284574	7.132889	7.167402	8.127397	7.106634	8.809137	0.652062089	-0.059426263	0.762251391	0.038893533
ENSG00000214265	AC124312.1	8.201909	8.577623	7.368661	7.425089	7.898686	6.618422	8.063704	7.765998	7.46512	0.969567734	0.008745711	0.816218747	0.038930229
ENSG00000164332	UBLCP1	9.347356	11.109961	12.073544	10.557791	9.435234	9.383702	10.133953	10.904749	10.251276	0.999137923	0.000374865	0.780918997	0.038943657
NewGene_235	--	10.660526	10.215805	10.059472	10.630096	6.136679	8.794072	9.973113	8.119006	16.910837	0.048058138	-0.268263643	0.914949978	0.038986754
ENSG00000158828	PINK1	8.86926	8.753302	7.807846	8.434708	8.805774	8.461773	7.47254	8.373261	8.655354	0.139176384	0.162351228	0.765240502	0.039014555
ENSG00000139597	N4BP2L1	10.299901	10.38995	11.028844	13.217545	13.629835	13.941432	10.075422	9.858696	9.92791	9.61E-08	0.456631616	0.729899633	0.039045068
ENSG00000205302	SNX2	19.247964	19.295925	20.303469	19.271766	19.257944	19.617978	19.072075	18.552635	18.960833	0.116678454	0.130330242	0.682558864	0.039208383
ENSG00000108561	C1QBP	205.354511	212.599721	226.53188	201.848709	201.255858	207.679775	204.729207	203.70479	211.966694	0.303827063	0.069475979	0.594521361	0.039218267
ENSG00000265808	SEC22B	8.562732	9.788973	10.229993	10.247029	10.404449	9.315043	9.885291	8.645933	9.275604	0.014475121	0.211021968	0.706127862	0.039378052
ENSG00000070882	OSBPL3	6.149483	6.117113	7.714926	6.945018	6.551374	6.112279	6.658486	6.11402	7.081031	0.258577514	0.126464634	0.744481779	0.039654304
ENSG00000049656	CLPTM1L	65.696189	61.786381	63.572861	52.494156	51.963321	54.884178	59.534065	57.601892	67.728595	0.071792797	-0.11428593	0.613014461	0.03968842
ENSG00000142252	GEMIN7	15.260358	15.95113	17.707196	14.811222	13.180016	13.998533	11.363066	15.089121	17.605518	0.780766088	0.038740114	0.788414179	0.039731509
ENSG00000185787	MORF4L1	180.291713	185.68742	176.323266	168.786022	168.378798	173.936471	181.421395	189.784312	173.637501	0.401596304	0.05762691	0.593946562	0.039773112
ENSG00000274523	RCC1L	34.173174	29.805429	27.462575	22.969141	23.017449	26.315288	28.615299	26.544361	33.356939	0.059986262	-0.189058623	0.731286515	0.039810296
ENSG00000174165	ZDHHC24	12.571896	12.899538	12.292556	12.527238	12.243433	14.158346	12.538933	11.432972	13.289121	0.012863106	0.203209827	0.659091867	0.039887194
ENSG00000157764	BRAF	4.169302	4.486908	5.130595	4.422909	4.304739	3.789575	6.256676	5.4481	5.745612	0.918130181	-0.01533183	0.727568544	0.040046951
ENSG00000050393	MCUR1	9.646602	10.638498	10.103632	8.857548	8.621121	8.729129	10.443093	8.869177	9.993037	0.52590068	-0.058419127	0.685622507	0.040159147
ENSG00000156531	PHF6	8.180806	9.259609	8.869161	6.97914	7.609374	7.275929	8.229733	8.638748	8.489173	0.273950963	-0.109739365	0.704872465	0.040175541
ENSG00000153714	LURAP1L	4.207322	3.477222	4.149637	3.101742	4.03265	3.53185	4.154491	4.10551	3.131618	0.991229371	-0.003343288	0.857727917	0.040266585
ENSG00000204152	TIMM23B	5.872707	7.474544	6.223133	7.734674	5.824565	6.245525	6.55041	6.587656	6.634683	0.133544742	0.204039936	0.788414179	0.040356266
ENSG00000115290	GRB14	25.788887	25.578959	26.295724	19.521925	21.25143	19.235224	25.189742	23.823788	25.919276	0.003135064	-0.224875008	0.625321048	0.040435477
ENSG00000110721	CHKA	183.253588	189.116863	194.274493	157.927919	141.876059	145.36581	188.158458	174.676053	194.265344	0.000122928	-0.213430935	0.547287953	0.040516479
ENSG00000108100	CCNY	19.452469	17.766677	19.432507	20.006051	20.337358	18.993192	18.404807	17.212433	20.313467	0.003558169	0.214455302	0.668609574	0.040550952
ENSG00000182054	IDH2	97.82832	82.967624	79.952602	85.509769	84.822772	87.350094	84.608949	78.837824	90.04024	0.191614218	0.112282182	0.667677441	0.040575478
ENSG00000008018	PSMB1	201.116669	196.319229	199.467773	211.316513	201.777328	196.507065	187.858948	198.502686	188.737259	0.000959634	0.177093804	0.607865374	0.040590485
ENSG00000178922	HYI	3.056101	1.68357	1.66008	1.19005	1.715167	1.132508	1.70882	3.491609	0.875468	0.210800298	-0.515961366	0.965353182	0.040624589
ENSG00000100568	VTI1B	14.226363	14.47393	13.690591	13.285293	12.119436	12.983239	15.711987	14.128965	14.624187	0.515893978	0.059368319	0.670211079	0.040643573
ENSG00000100014	SPECC1L	12.449509	10.359805	10.837545	9.660599	9.878667	9.710589	10.279994	9.895137	12.552055	0.60543319	-0.052593161	0.723922133	0.040645613
ENSG00000149761	NUDT22	24.131726	20.921622	19.904451	21.274787	21.730722	24.578222	18.641345	20.397093	24.100111	0.074355002	0.209322432	0.77199805	0.040656133
ENSG00000052841	TTC17	33.183515	32.582991	32.071973	27.467353	27.768722	26.779275	32.777095	30.259436	33.095974	0.059986262	-0.114452898	0.568137629	0.041042264
ENSG00000159753	CARMIL2	1.470649	1.422718	1.187685	0.966862	1.130351	0.950527	1.126102	1.310864	1.3951	0.415206783	-0.183011297	0.852231176	0.041048469
ENSG00000067596	DHX8	21.145448	20.314916	19.598062	18.643514	18.02996	19.030955	18.333678	18.173842	21.089493	0.431068844	0.057256047	0.605693726	0.04112801
ENSG00000106100	NOD1	0.243466	0.258227	0.358643	0.792423	0.986675	1.106121	0.298591	0.220986	0.342214	1.46E-13	1.869779284	0.925827355	0.041147204
ENSG00000035403	VCL	10.203407	9.847535	10.307206	6.800234	6.490853	7.348018	9.50803	9.384022	10.44757	9.58E-09	-0.409216477	0.625321048	0.041150321
ENSG00000149418	ST14	15.331023	15.614206	15.308651	9.535856	9.491181	9.556956	14.810198	14.356325	15.490793	4.88E-16	-0.546921602	0.626594138	0.041203086
ENSG00000125870	SNRPB2	27.071653	34.613242	34.896807	30.750356	26.841114	28.946124	31.82985	28.412729	30.562228	0.749549727	0.034935755	0.685186131	0.041417017
ENSG00000008323	PLEKHG6	4.607864	3.73078	3.240748	5.565271	6.127156	5.836967	4.881359	3.069756	3.066618	2.01E-13	0.993124724	0.849692347	0.041433254
ENSG00000076604	TRAF4	50.883749	47.22683	47.216609	32.716645	33.476907	35.690986	41.901093	45.981164	55.341267	3.21E-12	-0.436935031	0.666686039	0.041478928

Gene ID	Gene name	Ctrl-1 FPKM	Ctrl-2 FPKM	Ctrl-3 FPKM	IFNγ-1 FPKM	IFNγ-2 FPKM	IFNγ-3 FPKM	Sora-1 FPKM	Sora-2 FPKM	Sora-3 FPKM	Ctrl vs IFNγ DESeq2 FDR	Ctrl vs IFNγ DESeq2 log2FC	Ctrl vs Sora DESeq2 FDR	Ctrl vs Sora DESeq2 log2FC
ENSG00000079557	AFM	1.091019	1.196919	1.026819	1.077664	1.682965	1.612779	1.0014	1.130568	1.06196	0.057035971	0.547211435	0.899646028	0.041497807
ENSG00000151500	THYN1	11.590281	12.14762	11.300345	8.164589	8.339927	8.164926	10.454053	11.995499	10.843136	0.03407949	-0.291312698	0.800945568	0.041572202
ENSG00000133275	CSNK1G2	54.847269	54.119872	50.757112	48.490134	49.004001	49.857309	50.577016	47.43095	57.248625	0.773430767	0.024301919	0.605102012	0.041653131
ENSG00000168610	STAT3	16.464027	16.342075	17.6517	60.057137	55.976142	57.368321	16.321855	16.089988	17.425748	0	1.926861464	0.60854906	0.041666733
ENSG00000091483	FH	76.904045	76.366211	75.419937	77.810799	73.687279	75.011642	73.875229	73.154022	73.600609	0.018519458	0.133235632	0.5674801	0.041672324
NewGene_4929	--	137.438507	145.83902	95.527466	104.763344	111.24633	60.450069	107.444809	165.337204	89.987068	0.502757131	-0.307567366	0.93003092	0.041698207
ENSG00000143514	TP53BP2	41.450204	38.453521	40.595745	38.141445	38.600652	35.979618	39.938583	36.290684	39.92055	0.427796015	0.057349487	0.572220814	0.041758964
ENSG00000179918	SEPHS2	135.183258	134.818237	133.805222	171.601639	182.61441	175.7173	127.4049	127.1996	135.275818	2.23E-28	0.53996493	0.506673599	0.041779316
ENSG00000076716	GPC4	1.801366	1.49901	1.598048	1.177977	1.048147	1.193884	1.40448	1.586613	1.743532	0.030357271	-0.370407432	0.828187992	0.041859319
ENSG00000101052	IFT52	16.476423	16.767336	16.418898	12.393291	12.674997	11.524885	17.294267	15.530441	15.724189	0.001574313	-0.296203618	0.704208344	0.041914908
ENSG00000163346	PBXIP1	18.89305	16.948256	13.617398	12.588725	13.742771	13.809944	14.803456	16.066592	17.126443	0.202613448	-0.15783321	0.755452004	0.041930809
ENSG00000163069	SGCB	0.406998	0.378786	0.542118	0.325082	0.338001	0.324437	0.406343	0.37403	0.418529	0.78452059	-0.118096729	0.90895672	0.041957932
ENSG00000114744	COMMD2	19.023176	20.991217	20.883291	17.016083	15.892405	15.492066	20.015593	18.065479	21.385174	0.009453872	-0.186786022	0.642571213	0.041993676
ENSG00000103194	USP10	28.805619	28.796196	31.2765	31.001916	28.66829	29.027968	29.179664	27.330153	29.218971	0.036903432	0.144323172	0.603027023	0.042058238
ENSG00000113494	PRLR	3.633879	3.926233	3.122396	4.167194	4.276135	3.872897	3.806743	3.369898	3.386224	0.0000917	0.360195135	0.7304585	0.042067589
ENSG00000185515	BRCC3	10.150656	11.672018	11.377451	11.820134	10.495488	10.179521	11.23956	10.65922	10.260305	0.340701689	0.104171229	0.743689892	0.042079458
NewGene_1298	--	0.930795	0.862078	1.934461	0.887795	0.790193	0.777347	0.963582	1.083495	1.488156	0.318350235	-0.425653844	0.920727663	0.042124906
ENSG00000068394	GPKOW	13.253695	12.284139	11.397172	11.258246	11.599117	11.242058	11.307395	11.006935	13.437659	0.833744224	0.031188126	0.751179587	0.042214055
ENSG00000144306	SCRN3	4.438242	4.230352	3.301376	4.213331	3.601402	4.704401	4.947704	3.187142	3.765016	0.357000658	0.15332599	0.814652612	0.042339159
ENSG00000105053	VRK3	15.173345	14.413598	13.848034	13.644174	12.072679	13.555893	13.129213	13.629935	14.695926	0.975527107	-0.004963166	0.710932447	0.042436751
ENSG00000174780	SRP72	74.581358	78.815975	75.131595	76.138576	72.79409	73.849661	73.902882	72.5611	77.007653	0.046260631	0.10837589	0.521737139	0.042449096
ENSG00000108469	RECQL5	9.234097	8.543141	8.12132	8.101215	6.915232	7.143626	8.497151	7.175881	8.724344	0.782767137	-0.037338104	0.742557146	0.042513858
ENSG00000123545	NDUFAF4	11.839071	11.772749	12.72027	8.236085	8.397253	9.273475	11.836185	11.520354	11.718444	0.000431515	-0.338572307	0.69552553	0.042518044
ENSG00000132780	NASP	90.789702	93.324567	93.996578	104.037141	92.817014	118.825983	99.090601	89.514561	101.861642	0.016073965	0.146087649	0.520524755	0.042665117
ENSG00000143257	NR1I3	1.823057	1.373791	1.559016	1.375405	1.53356	1.152159	1.021897	1.522245	1.556342	0.492913437	0.249379882	0.909047497	0.042702098
ENSG00000103160	HSDL1	8.483538	8.464273	9.028601	7.857576	7.416927	7.690032	8.315496	8.161886	8.634184	0.794878448	-0.031415273	0.682596978	0.042720329
ENSG00000079277	MKNK1	7.235826	6.257741	6.216975	7.717443	7.409297	7.065713	6.723172	6.118085	7.139201	0.024160024	0.262657898	0.760696683	0.042757689
ENSG00000172115	CYCS	80.190525	89.744464	97.19673	86.663418	87.649669	88.017013	85.190805	87.151884	87.35876	0.100953863	0.123502781	0.63440293	0.042772635
ENSG00000128487	SPECC1	15.105632	15.341332	16.726765	12.875537	14.457449	12.189553	17.107568	14.733388	16.29128	0.002203393	-0.232588245	0.642571213	0.042863954
ENSG00000178573	MAF	4.427603	3.433471	2.987553	6.775414	7.768322	7.128809	3.70956	2.974205	3.231348	3.85E-39	1.246477607	0.751499613	0.042888293
ENSG00000139055	ERP27	2.09426	2.665884	2.364355	1.369721	1.697315	1.338141	2.281183	2.358206	2.217672	0.015256049	-0.541500917	0.854509288	0.042968204
ENSG00000197977	ELOVL2	68.724678	74.210449	82.944092	63.690952	64.691986	64.164696	72.783531	69.143707	76.440918	0.31381098	-0.082582184	0.622975428	0.043070078
ENSG00000196924	FLNA	43.998012	44.192466	39.498028	16.915002	14.926591	17.314682	37.760886	38.412609	46.75515	1.48E-84	-1.238935611	0.647104471	0.043076515
ENSG00000113575	PPP2CA	34.199845	35.849506	34.702773	33.371853	33.019946	32.367085	33.523911	32.900232	34.926572	0.324469449	0.063093943	0.532667746	0.04311169
ENSG00000165443	PHYHIPL	20.059999	22.036452	20.329274	19.626358	20.173088	21.153756	18.545821	19.161831	21.340433	0.102165891	0.123724648	0.611006415	0.043113343
ENSG00000174021	GNG5	118.786586	121.764471	125.645608	104.607466	108.912654	112.005318	111.20686	123.474239	114.610014	0.822475674	-0.019662242	0.625711634	0.043318774
ENSG00000171206	TRIM8	6.130458	6.733734	6.172595	5.693433	4.243169	4.939887	4.787331	5.137639	7.87386	0.001790033	-0.296442573	0.706959813	0.043327885
ENSG00000167536	DHRS13	11.985579	10.775586	9.853575	8.49034	8.010701	9.436307	9.874192	9.912972	11.690615	0.141560103	-0.189395476	0.754005687	0.043342687
ENSG00000175573	C11orf68	15.783243	13.966221	12.213611	14.580267	14.348865	14.935817	13.931572	13.756416	12.851049	0.080069268	0.210911502	0.766052664	0.043373753
ENSG00000051009	FAM160A2	16.103465	15.44431	14.324177	11.333938	10.990054	10.937666	14.838418	14.101937	15.363944	0.0000383	-0.311678497	0.638374388	0.04338672
ENSG00000110921	MVK	26.712354	23.621073	21.537431	36.067718	37.549511	35.616339	20.482272	25.383376	21.935711	1.39E-17	0.676435606	0.706368645	0.043450344
ENSG00000122873	CISD1	8.369943	8.010678	8.588246	6.15681	6.505314	6.555729	7.780202	8.512593	7.795865	0.036692962	-0.229540539	0.738670032	0.043514992
ENSG00000164172	MOCS2	15.192373	15.655919	17.16966	11.909346	11.040995	10.844002	16.73919	14.357538	15.9849	0.000271604	-0.353621898	0.716048274	0.043533031
ENSG00000170381	SEMA3E	0.361271	0.336145	0.478834	0.304133	0.25683	0.284134	0.333712	0.367291	0.413066	0.254731076	-0.356765417	0.890499304	0.043539168
ENSG00000103227	LMF1	3.886435	3.182314	3.659105	3.839444	3.8994	3.034826	2.614307	3.650991	3.405374	0.497557849	0.124505263	0.820885588	0.043545295
ENSG00000100485	SOS2	6.877451	7.185143	6.345247	5.596651	5.541845	5.03815	6.476547	6.271327	7.496903	0.094335321	-0.163121096	0.697344033	0.04359737
ENSG00000113328	CCNG1	59.66484	65.335326	62.788849	51.456326	54.547248	51.717548	59.628508	60.437396	60.899905	0.163430759	-0.100166392	0.575520972	0.043645636
ENSG00000158985	CDC42SE2	14.813485	15.498829	15.759177	27.311539	28.088126	27.659597	15.285677	13.560755	14.95538	3.49E-72	0.998063771	0.604896634	0.043739427
ENSG00000165689	ENTR1	46.192957	51.686467	52.951134	46.966995	44.029902	44.03579	46.977455	46.270386	51.225763	0.958758356	0.005452005	0.60184868	0.043746257
ENSG00000196141	SPATS2L	178.407175	150.362712	163.591758	114.753069	122.961492	117.461316	152.157427	158.278527	179.148736	4.75E-08	-0.346801646	0.626594138	0.043765426
ENSG00000173678	SPDYE2B	1.403968	1.30902	1.203183	1.835806	1.369296	1.308973	1.062736	1.199515	1.582188	0.265897739	0.340376339	0.896970296	0.043870995
ENSG00000153487	ING1	4.815589	4.7346	5.556348	4.329467	3.956024	4.182539	4.889474	4.666362	5.334259	0.183728771	-0.168623169	0.738554112	0.043895163
ENSG00000143476	DTL	18.997748	18.536703	19.873984	18.841299	17.529451	17.554585	19.526807	17.369732	18.619633	0.480600193	0.056479387	0.601725332	0.044055635
ENSG00000087299	L2HGDH	2.997899	3.950878	3.62138	3.245482	3.710743	3.726587	3.590263	3.415768	3.956738	0.688744048	0.066801351	0.771177779	0.044062278
ENSG00000179364	PACS2	9.73603	7.920432	7.903843	8.008062	6.932016	7.223255	8.316088	8.739997	9.750867	0.203586904	-0.13962601	0.706959813	0.044242146
ENSG00000175309	PHYKPL	5.163403	4.581187	4.652719	5.67537	5.618087	5.59758	4.39907	4.376731	4.350763	0.003875429	0.407910677	0.808454747	0.044271842
ENSG00000121851	POLR3GL	12.851405	12.37479	11.802778	10.920583	11.055943	10.487626	12.256824	11.808499	12.037113	0.67896931	-0.065170513	0.75368788	0.044338504
ENSG00000107186	MPDZ	7.032158	9.819367	8.257797	6.384831	6.623748	5.35206	6.494652	6.80235	7.188749	0.442723709	-0.077767324	0.635464966	0.04436245
ENSG00000240184	PCDHGC3	2.613165	2.611104	2.281065	3.165522	2.527817	2.446688	2.709282	2.138168	2.338844	0.062610335	0.277099288	0.797899295	0.044432558
ENSG00000114054	PCCB	29.9434	20.751815	29.443437	24.826256	22.389036	24.393107	32.234105	28.095583	28.186036	0.003682386	-0.209117965	0.648654916	0.044439678
ENSG00000203950	RTL8A	18.566618	16.708326	15.743907	13.508792	14.280758	15.4128	14.779079	17.111252	17.446291	0.514619111	-0.091546895	0.747797618	0.044471968
ENSG00000143155	TIPRL	21.454363	21.624554	22.415853	20.328126	20.317385	20.836544	20.867914	20.151502	22.550433	0.524826017	0.053227648	0.590318853	0.044497055
ENSG00000164934	DCAF13	28.344596	28.998313	33.138509	24.135976	21.217995	22.741095	30.091107	29.548835	27.937554	0.006123724	-0.189197759	0.574823476	0.044555269
ENSG00000102743	SLC25A15	5.51798	4.611744	4.249717	3.752748	4.283708	3.72441	4.868145	4.15213	4.925334	0.503407443	-0.143464812	0.823687942	0.044681808
ENSG00000108774	RAB5C	107.824233	104.930162	99.390396	101.570227	103.358908	111.21362	98.525224	102.867893	104.363021	0.020952528	0.149890779	0.54939816	0.044681859
ENSG00000165792	METTL17	29.846367	30.441766	31.798531	28.030812	25.13655	25.319943	28.397056	28.393874	30.317443	0.459572374	-0.066282075	0.597257253	0.044900115
ENSG00000147383	NSDHL	29.484214	27.399904	24.676288	31.413252	33.947095	31.455448	24.98039	27.71502	23.513162	0.0000118	0.365747001	0.719662319	0.044914767
ENSG00000080503	SMARCA2	2.156412	2.28455	2.178514	3.118826	3.319176	2.790874	2.208051	1.944162	2.299858	4.27E-09	0.640149953	0.769920305	0.044948603
ENSG00000131471	AOC3	0.401404	0.358458	0.416202	0.442733	0.384013	0.316146	0.337698	0.516441	0.335241	0.890289578	-0.070742558	0.914976479	0.044970724
ENSG00000136878	USP20	6.049485	4.703475	4.67707	4.220493	4.29945	4.302125	5.191629	4.746279	4.978891	0.417485499	-0.118502942	0.757510399	0.045200594
ENSG00000179104	TMTC2	2.383499	2.784288	2.483026	1.576622	1.774648	1.868867	2.981726	2.669291	2.929466	0.000116338	-0.46934167	0.744890745	0.045225107
ENSG00000108439	PNPO	9.654993	9.768084	10.09336	10.967736	10.225237	10.506793	10.337607	9.409696	9.631655	0.005320696	0.226573766	0.66619098	0.045236721
ENSG00000113068	PFDN1	53.168752	50.169271	49.102612	36.543978	37.87527	40.735263	49.452618	50.553267	47.304521	0.000819355	-0.255912966	0.62882478	0.045324482
ENSG00000198246	SLC29A3	9.025737	9.502919	10.3257	6.363122	6.724941	6.858675	9.388143	8.93905	9.374174	0.000803952	-0.356086329	0.711817535	0.045348265

Gene ID	Gene name	Ctrl-1 FPKM	Ctrl-2 FPKM	Ctrl-3 FPKM	IFNγ-1 FPKM	IFNγ-2 FPKM	IFNγ-3 FPKM	Sora-1 FPKM	Sora-2 FPKM	Sora-3 FPKM	Ctrl vs IFNγ DESeq2 FDR	Ctrl vs IFNγ DESeq2 log2FC	Ctrl vs Sora DESeq2 FDR	Ctrl vs Sora DESeq2 log2FC
ENSG00000006042	TMEM98	26.15104	26.796967	17.656536	11.942649	11.066303	13.91196	21.542213	24.566846	29.181822	1.52E-13	-0.569082253	0.594203992	0.045372992
ENSG00000188739	RBM34	32.071583	31.358744	37.350765	34.577169	31.305352	30.973597	38.73671	38.491255	36.172185	0.447124364	0.06814845	0.630069298	0.045406274
ENSG00000188177	ZC3H6	0.682883	0.864821	0.958826	0.689476	0.750165	0.687887	0.833375	0.765531	0.821787	0.691741011	-0.085484771	0.811954158	0.045443358
ENSG00000014824	SLC30A9	23.318663	24.372613	23.955264	22.536469	22.625946	23.784483	23.843707	22.662342	22.578543	0.243386216	0.072838764	0.558251541	0.04550154
ENSG00000058799	YIPF1	10.592511	10.328336	9.58008	9.566195	9.986479	10.609307	9.489254	10.811151	9.220383	0.294887798	0.135317231	0.763820026	0.045595057
ENSG00000169188	APEX2	6.645405	5.473155	5.620489	5.27659	5.733312	5.753403	5.676708	5.115709	6.425784	0.6672993	0.066514832	0.751995229	0.045628488
ENSG00000111731	C2CD5	21.320839	22.182443	23.796852	18.423451	18.724991	18.437541	20.721537	21.669308	23.08436	0.044703251	-0.137163695	0.575365741	0.045635595
ENSG00000171824	EXOSC10	31.660031	32.210221	29.770672	25.750701	24.039209	24.251333	29.30373	29.606486	31.445267	0.005034776	-0.186965113	0.571558279	0.045638881
ENSG00000154839	SKA1	2.064636	3.417942	3.041162	2.499226	1.92661	1.929031	2.54585	2.651416	2.928867	0.078286774	-0.354540599	0.831505656	0.04565347
ENSG00000166263	STXBP4	3.170811	3.505503	3.336163	2.432247	2.546704	2.517132	3.180814	3.028079	3.100938	0.0000422	-0.330969266	0.638040119	0.045676575
ENSG00000081059	TCF7	6.27133	6.460246	7.240082	4.70125	4.828933	4.547629	6.247524	6.200113	6.575795	0.000113111	-0.415354594	0.713033429	0.045701721
ENSG00000169895	SYAP1	6.16692	6.465607	6.613619	5.891879	5.916646	6.661797	6.262328	5.919633	6.452087	0.381566641	0.089986892	0.631759089	0.045709709
ENSG00000070047	PHRF1	16.134273	15.012399	13.891738	15.119684	14.86363	16.266551	15.183861	12.524261	16.239783	0.016852461	0.182472208	0.66953576	0.045742113
ENSG00000262919	CCNQ	5.702462	5.231808	5.396319	5.712597	5.387107	5.804096	4.406525	5.391254	5.812568	0.271812858	0.198483198	0.826359998	0.045776697
ENSG00000065183	WDR3	4.963651	4.908568	5.316417	5.782333	5.137848	5.481053	4.798578	4.847487	5.067052	0.000516222	0.257647012	0.616452317	0.04580311
ENSG00000077782	FGFR1	6.982787	6.39329	6.560333	1.782175	2.556923	2.191522	6.033816	5.88937	7.319496	1.59E-35	-1.420798545	0.675167061	0.045867929
ENSG00000180596	HIST1H2BC	2.120191	1.779928	2.154512	1.881905	1.522983	1.77266	1.79172	1.95276	2.049352	0.879880883	-0.07995427	0.912989785	0.04590687
ENSG00000073921	PICALM	37.652369	39.62919	42.986677	35.134117	34.909987	33.126669	36.735625	37.599579	40.891578	0.427388447	-0.066533036	0.598245073	0.045921454
ENSG00000164051	CCDC51	7.295781	7.538999	7.239159	6.196831	5.602347	7.040858	6.845772	6.671882	7.655654	0.703839571	-0.071753748	0.766646116	0.045923342
ENSG00000196453	ZNF777	7.291162	5.926816	6.273547	7.09283	6.842233	6.983908	6.684155	5.650553	6.573624	0.027046289	0.24771231	0.741167578	0.045964086
ENSG00000065135	GNAI3	2.896879	3.206127	2.917462	2.702966	2.749399	2.697855	2.903209	2.785268	3.048827	0.991622061	0.001240689	0.582449623	0.046008171
ENSG00000105722	ERF	48.281146	43.99835	41.881572	43.2983	43.757921	45.857861	44.141808	43.878132	42.185495	0.080242546	0.133381921	0.614962148	0.046074209
ENSG00000114346	ECT2	68.793265	75.36949	80.050976	51.549441	50.805089	53.401724	71.280908	74.317547	80.967105	1.1E-14	-0.419031501	0.546340755	0.046172198
ENSG00000186298	PPP1CC	86.77686	87.266648	91.398931	85.480459	82.609993	81.382726	87.420296	82.638862	90.637536	0.431311469	0.05058918	0.493480799	0.046237069
ENSG00000160908	ZNF394	7.359707	6.821437	8.959249	8.901264	8.068571	8.41712	7.340637	7.064078	7.832024	0.0260579	0.284217073	0.76084849	0.046424984
ENSG00000205238	SPDYE2	1.193741	1.227022	1.12657	1.592905	1.100612	1.097215	1.084645	1.00297	1.370587	0.507207332	0.235705025	0.892048046	0.046672736
ENSG00000166477	LEO1	41.708508	40.249763	42.697806	36.282517	37.133474	36.996708	39.342556	39.786793	41.529766	0.753215108	-0.027582992	0.548304958	0.04674653
ENSG00000116212	LRRC42	22.214603	21.439017	21.656193	19.595156	17.085593	19.007934	23.492061	20.671227	20.413203	0.285630401	-0.099227296	0.64905409	0.046775581
ENSG00000139370	SLC15A4	14.748992	13.925505	14.762252	12.365279	12.336975	12.094957	14.89356	14.030888	14.904093	0.304800746	-0.095173639	0.625285662	0.046809568
ENSG00000106591	MRPL32	36.828293	39.141129	39.349288	39.727946	41.64587	38.835114	37.464556	36.959376	38.599852	0.009166877	0.186093023	0.583594003	0.046848149
ENSG00000162923	WDR26	32.88846	33.949255	39.340384	30.744679	29.88266	27.761438	35.332644	32.313545	37.502558	0.062525783	-0.142337239	0.598931509	0.04691482
ENSG00000011198	ABHD5	1.494736	1.739959	1.399186	1.627092	1.793218	1.462733	1.470916	1.397665	1.356732	0.262093652	0.196437458	0.807044216	0.046950712
ENSG00000135299	ANKRD6	2.874195	2.200469	2.896308	2.451927	2.421712	2.370673	2.670866	2.307504	3.248105	0.890289578	-0.02854615	0.790993784	0.046998217
ENSG00000123131	PRDX4	52.792052	53.217873	54.669731	47.067765	49.53714	49.78264	50.083229	54.236019	51.215971	0.913160856	0.012132557	0.622947585	0.047127986
ENSG00000163818	LZTFL1	1.987138	2.304623	2.178727	1.859077	1.759326	1.800301	2.120846	2.254818	2.046565	0.455398368	-0.134805009	0.792598574	0.047151658
ENSG00000117222	RBBP5	9.055428	10.122754	10.568968	9.108556	8.653051	8.800812	9.817607	8.932517	9.910032	0.915332123	-0.013657686	0.648072983	0.047200677
ENSG00000085721	RRN3	22.298494	24.010858	22.979098	18.805334	19.829363	19.353058	23.405566	22.389724	23.204287	0.250273131	-0.087568545	0.555413484	0.047289096
ENSG00000128050	PAICS	119.006384	121.603015	115.283202	71.275732	76.927969	103.253576	101.477974	128.18867	95.736764	0.030522925	-0.109528283	0.451882609	0.04729689
ENSG00000162999	DUSP19	0.429264	0.782431	0.861817	0.412797	0.563267	0.421974	0.731451	0.530603	0.613525	0.297151364	-0.347986187	0.885688258	0.047301269
ENSG00000149485	FADS1	74.49442	73.146979	70.114468	92.465673	88.713095	89.500024	72.079909	68.960722	68.610827	9.38E-24	0.46788434	0.50963505	0.047351277
ENSG00000081791	DELE1	6.686181	6.337	6.699359	5.31198	5.409867	5.21331	5.702409	5.95246	6.173271	0.245838001	-0.118042899	0.642911881	0.047407884
ENSG00000119328	FAM206A	12.343046	13.877323	14.117013	13.95723	14.396533	13.22332	13.060866	12.591518	13.623316	0.065301827	0.185645589	0.674400906	0.047583162
ENSG00000085832	EPS15	8.062371	8.727448	8.920988	9.075432	8.847795	8.54438	8.365183	8.018357	8.528266	0.017610986	0.189261056	0.614243075	0.047596367
ENSG00000123213	NLN	5.962607	7.067353	6.513744	4.879481	4.143335	4.685744	6.360025	6.157325	6.161555	0.000000255	-0.397920883	0.618337765	0.047609632
ENSG00000133619	KRBA1	2.516507	2.940937	2.78353	2.31864	1.842723	2.45168	2.779477	2.287988	3.694542	0.579884468	-0.109039306	0.821821119	0.047691885
ENSG00000130956	HABP4	4.600455	4.40941	5.282715	6.459357	5.690511	6.000289	4.769861	4.551711	4.554731	0.0000377	0.487741545	0.761739684	0.047718354
ENSG00000116525	TRIM62	0.295576	0.497022	0.324274	0.466442	0.176572	0.326715	0.326174	0.407006	0.33502	0.93402599	-0.054906335	0.916597659	0.047765065
ENSG00000094916	CBX5	25.732707	25.759351	25.529064	21.695221	22.268135	21.66405	25.247661	23.993925	26.283311	0.217895081	-0.074102199	0.461321163	0.047801506
ENSG00000121073	SLC35B1	52.141805	52.651046	52.250642	63.547139	54.910128	62.990552	49.042158	53.406588	51.550777	0.0000158	0.311802898	0.615112352	0.047898991
ENSG00000143621	ILF2	258.162397	267.003431	263.089565	232.123031	218.179007	228.437699	251.636879	263.22591	247.803893	0.22725261	-0.067268307	0.530066127	0.047932801
ENSG00000166965	RCCD1	3.153517	3.027698	2.849327	2.582235	2.65313	2.095745	2.80222	2.778945	3.163883	0.451395912	-0.148508802	0.790257897	0.047940822
ENSG00000116754	SRSF11	61.937181	66.889509	69.315198	69.170968	61.623763	60.791203	66.918053	60.869727	71.126181	0.256522863	0.094995776	0.575365741	0.047952789
NewGene_2027	--	0.942744	0.92106	2.089899	0.930026	0.820697	0.575807	1.346092	1.224855	1.259005	0.162234389	-0.618142478	0.911553655	0.047986689
ENSG00000081377	CDC14B	6.840501	7.068431	6.425624	6.593352	6.635096	6.776131	6.683239	6.286775	7.185565	0.391442212	0.083898049	0.642556857	0.04800741
ENSG00000117394	SLC2A1	9.405781	9.018898	8.327011	7.307666	6.98566	7.713394	8.720241	8.867599	8.425125	0.210131408	-0.130458586	0.664183228	0.048012477
ENSG00000123080	CDKN2C	1.37101	1.28645	1.23961	1.890232	1.381098	1.440553	1.498349	1.30532	1.05287	0.057245492	0.473237055	0.882809924	0.048044939
ENSG00000153879	CEBPG	37.420089	37.349733	38.753687	35.659143	36.199876	35.031561	36.992343	36.310876	36.514946	0.360943321	0.060870525	0.50326565	0.048189326
ENSG00000107262	BAG1	39.119363	39.538212	37.242673	30.091903	33.119677	37.105885	43.110188	41.181654	49.61337	0.347184465	-0.080263857	0.57726286	0.048230055
ENSG00000169432	SCN9A	8.333876	9.456818	10.894523	8.041156	7.849534	7.45676	10.299386	9.238939	10.185331	0.093637435	-0.185368551	0.701121911	0.048261139
ENSG00000165795	NDRG2	26.636971	22.867302	24.729455	21.090262	21.453298	22.076403	20.060127	21.960005	27.826133	0.717463207	0.040614645	0.639174276	0.048282262
ENSG00000110713	NUP98	45.601042	45.417764	42.160557	42.320584	41.11364	39.492337	46.473287	43.914613	43.26191	0.940769673	-0.005872078	0.441900971	0.048363807
ENSG00000148334	PTGES2	28.916761	27.863744	25.535938	22.481983	23.516948	21.608664	24.970043	27.989032	27.324506	0.089803868	-0.143032653	0.641953157	0.04838449
ENSG00000129562	DAD1	110.657545	107.189345	105.442133	94.99338	95.858612	97.130434	103.597313	108.552541	97.674232	0.955679584	-0.005681302	0.606979569	0.048465978
ENSG00000167113	COQ4	13.921077	12.671949	12.974138	15.392915	15.713456	15.438522	12.266556	12.831507	13.232686	0.000076	0.394507269	0.713240802	0.048657149
ENSG00000154917	RAB6B	0.340229	0.299714	0.442209	0.303976	0.33395	0.227795	0.337258	0.379175	0.33027	0.681847625	-0.174662534	0.893604567	0.048843923
ENSG00000178719	GRINA	52.263405	49.186877	53.244782	64.191592	64.630365	64.654943	46.7905	51.439979	53.74284	3.16E-15	0.469713238	0.549834084	0.048897387
ENSG00000136891	TEX10	21.056404	22.022892	21.955145	16.926412	17.017128	15.701751	21.688376	20.180526	21.406729	0.000506568	-0.244301764	0.550622258	0.048924081
ENSG00000185340	GAS2L1	3.985932	4.241049	4.285433	3.04005	2.595513	3.326356	3.781773	3.805664	4.355797	0.043469804	-0.302218078	0.738673283	0.048978901
ENSG00000166579	NDEL1	10.902459	10.133906	12.477751	11.530399	12.516889	10.810961	10.791725	10.805046	11.92083	0.144224409	0.180669043	0.716048274	0.048984021
ENSG00000185627	PSMD13	144.327922	130.048985	125.451119	117.890847	99.524029	122.001304	126.49802	134.214594	118.106508	0.47514247	-0.053571265	0.534278242	0.048993893
ENSG00000135424	ITGA7	2.165064	1.793587	1.806995	1.203623	1.291818	1.000371	1.863115	1.75297	2.230007	0.000702204	-0.649995284	0.818216285	0.049011521
ENSG00000135457	TFCP2	4.181219	4.836156	4.561022	3.815817	3.733714	4.003174	4.292603	4.185651	4.547689	0.564767881	-0.088031094	0.729927321	0.049063269
ENSG00000003249	DBNDD1	10.194943	10.948478	10.996173	10.383734	9.427041	10.671129	9.721272	10.146121	11.158313	0.628239168	0.062825256	0.680108779	0.049159103
ENSG00000117362	APH1A	100.611299	95.445543	90.164386	96.121721	98.906478	101.834596	91.202613	91.804329	93.823152	0.001100871	0.201025729	0.510129647	0.04917007

Gene ID	Gene name	Ctrl-1 FPKM	Ctrl-2 FPKM	Ctrl-3 FPKM	IFNγ-1 FPKM	IFNγ-2 FPKM	IFNγ-3 FPKM	Sora-1 FPKM	Sora-2 FPKM	Sora-3 FPKM	Ctrl vs IFNγ DESeq2 FDR	Ctrl vs IFNγ DESeq2 log2FC	Ctrl vs Sora DESeq2 FDR	Ctrl vs Sora DESeq2 log2FC
ENSG00000113558	SKP1	61.967548	59.865646	56.028713	20.709691	44.611542	25.840875	50.524196	22.082969	57.211257	0.59686794	-0.041988002	0.54633979	0.049176272
ENSG00000143771	CNIH4	11.917833	12.040849	10.665732	12.538175	11.441192	12.154479	11.14485	12.037966	10.658813	0.047757304	0.166911318	0.638178553	0.04920727
ENSG00000158435	CNOT11	42.116029	40.86971	41.483746	37.795684	38.0681	37.756434	41.474839	38.792174	40.476396	0.859596371	0.015170087	0.51001417	0.049234614
ENSG00000104341	LAPTM4B	150.456186	190.225223	177.988206	116.605396	149.315608	146.463741	184.616949	184.715643	148.417359	0.000790605	-0.16809278	0.417164998	0.049456013
ENSG00000166840	GLYATL1	2.840393	3.866041	2.834678	1.640954	2.032272	1.865171	2.965167	2.866608	3.718387	0.000918562	-0.727797481	0.833323685	0.049458695
ENSG00000091140	DLD	22.402455	24.415279	26.70768	21.964333	20.241164	21.332059	26.582974	22.918474	21.390902	0.911527461	-0.011349777	0.581563006	0.04946472
ENSG00000047849	MAP4	28.043946	29.027449	26.115421	23.252756	22.27047	22.057057	28.896309	26.663486	29.39405	0.135667833	-0.091294555	0.478980206	0.049505251
ENSG00000004399	PLXND1	10.208724	8.867646	8.017554	5.287772	5.402581	5.110084	8.043831	8.382038	9.991543	9.76E-13	-0.627726851	0.680681727	0.049575072
ENSG00000163795	ZNF513	7.550534	6.438825	7.079475	6.864099	6.747738	6.456363	7.251417	6.224364	6.873089	0.582930144	0.082717087	0.733247068	0.049660403
ENSG00000141873	SLC39A3	33.920769	33.837084	31.451844	29.583435	27.663179	31.190518	30.731929	31.722866	34.520352	0.747114883	-0.035376727	0.608975116	0.049693284
ENSG00000141646	SMAD4	8.156892	8.481832	8.781356	8.75489	8.564518	7.865933	10.376744	9.762377	9.644109	0.271591696	0.090292642	0.565523076	0.049726271
ENSG00000151690	MFSD6	6.678972	6.110208	6.671235	4.796573	4.992728	5.180611	6.178949	5.678169	6.802832	0.018991925	-0.227498855	0.667081897	0.049812188
ENSG00000063761	ADCK1	4.170486	4.175031	4.164528	2.774687	2.835592	2.985502	4.081765	3.707933	4.276979	0.014906169	-0.373359455	0.767715478	0.049827068
ENSG00000136861	CDK5RAP2	26.704409	26.918149	26.609534	20.728919	18.932501	18.894798	24.836953	24.795138	27.080562	0.00000391	-0.257385735	0.464167794	0.049910469
ENSG00000111371	SLC38A1	33.986861	37.14118	39.415461	31.936439	32.338767	31.873963	33.46638	34.765265	39.143775	0.51773497	-0.053503176	0.572904784	0.049982524
ENSG00000129255	MPDU1	97.713256	86.567183	97.059101	95.152155	86.956051	92.16934	79.055944	84.054301	92.413675	0.359750922	0.075305122	0.593109865	0.050025837
ENSG00000115946	PNO1	21.72336	25.180683	26.55305	21.490848	19.320505	21.029849	23.803604	22.778367	24.858292	0.328632861	-0.101178536	0.629622447	0.050080863
ENSG00000121350	PYROXD1	4.016344	5.182407	5.601744	4.542879	4.052233	3.979453	5.717026	5.494769	4.817372	0.826252675	-0.042631701	0.792598574	0.05011492
NewGene_4968	--	0.287374	0.352421	0.352102	0.637157	0.261665	0.361133	0.443559	0.294286	0.210772	0.304758143	0.48489782	0.921143129	0.050180813
ENSG00000125386	FAM193A	16.443066	15.392575	16.922672	15.6392	13.576602	14.022962	16.640019	13.452687	17.099727	0.977395139	0.003356084	0.61541511	0.050209552
ENSG00000244045	TMEM199	15.559018	15.881502	13.695861	14.291992	13.24065	13.362495	16.149736	14.937176	14.029342	0.925460152	0.012149036	0.646423903	0.050239746
ENSG00000100324	TAB1	8.338552	8.185737	7.056485	7.671408	7.639427	8.537156	7.551484	7.434289	7.895527	0.169486396	0.15923244	0.675789958	0.050426737
ENSG00000114745	GORASP1	9.867619	9.218699	9.551064	7.147226	6.519505	6.478464	9.478049	8.672611	8.650496	0.001359069	-0.302770834	0.659354104	0.050445391
ENSG00000005007	UPF1	32.289958	31.26965	30.083445	30.656493	28.915998	31.244256	28.986553	27.817689	34.250019	0.107228837	0.103634486	0.550283378	0.050694609
ENSG00000147457	CHMP7	9.220762	9.461013	10.101339	7.669678	8.01244	8.28631	9.562553	8.563317	10.686649	0.094845332	-0.159300458	0.634695181	0.050767465
ENSG00000156931	VPS8	4.981787	4.502962	4.321569	3.645423	3.744046	3.959423	4.29607	4.586533	4.688891	0.147804446	-0.170716861	0.687510509	0.050795349
ENSG00000100519	PSMC6	57.311831	58.408724	61.773285	48.769684	44.766615	44.194791	59.048047	49.953575	55.298826	0.001732394	-0.199830837	0.505499712	0.050859782
ENSG00000155542	SETD9	5.700028	5.872296	6.30731	3.77866	3.935153	3.54547	5.942586	6.160537	4.9137	0.000775304	-0.547524977	0.797588566	0.050865926
ENSG00000138758	11-Sep	15.822675	17.629223	18.428561	15.762445	15.704666	15.265186	16.210924	16.466241	17.528675	0.981911169	0.002667638	0.564087072	0.050896541
ENSG00000105656	ELL	5.264225	5.336007	4.539239	5.236971	5.315599	5.680309	4.953237	4.667959	5.560098	0.028157638	0.245059801	0.690848398	0.050999482
ENSG00000134802	SLC43A3	73.698736	67.924156	69.890443	54.636726	54.250228	55.566713	65.731935	67.571814	69.733073	0.000128148	-0.208186461	0.466998848	0.051297494
ENSG00000142327	RNPEPL1	7.753916	6.784823	6.914978	6.588208	6.763504	7.245849	6.847943	6.336137	8.043436	0.423035123	0.095648625	0.674400906	0.051412928
ENSG00000116704	SLC35D1	6.662552	7.251767	6.428739	7.775265	7.326702	7.266132	7.00685	6.098448	6.674958	0.000268123	0.283873736	0.616185074	0.051542771
ENSG00000136986	DERL1	18.294712	18.958829	19.931863	21.519577	20.834389	21.507786	18.621352	18.267497	18.472861	0.00000226	0.304547262	0.550283378	0.051623855
ENSG00000138101	DTNB	2.235636	1.884883	1.695554	2.209584	1.61524	1.517136	1.973691	1.92437	1.738897	0.903892401	0.038157835	0.829906023	0.051640225
ENSG00000148832	PAOX	0.972901	0.874404	0.946788	1.439495	1.615445	1.232013	1.08198	0.689036	0.993668	0.005944121	0.763900214	0.892048046	0.051759354
NewGene_1509	--	1.324822	1.227499	1.364391	1.056508	0.93541	0.850041	1.193053	1.163961	1.537845	0.473956343	-0.287379693	0.890058192	0.051763097
ENSG00000078246	TULP3	9.403754	8.838739	10.253693	8.538132	7.378206	7.861238	9.231829	8.892731	9.418324	0.322121821	-0.114399821	0.646527847	0.051785527
ENSG00000178568	ERBB4	0.42731	0.429625	0.439691	0.297181	0.316339	0.254467	0.427118	0.382166	0.433823	0.043369622	-0.421934453	0.812053278	0.051821851
ENSG00000241186	TDGF1	0.728152	0.620839	0.605813	0.407392	0.546298	0.434809	0.594611	0.63688	0.782774	0.388047531	-0.351783712	0.891413828	0.051909533
ENSG00000117519	CNN3	82.202899	77.645263	85.885656	61.862225	65.748364	60.646677	75.373943	81.504699	80.939983	0.000239774	-0.237621838	0.530287719	0.051975178
ENSG00000259024	TVP23C-CDRT4	2.853272	1.655321	2.437981	4.465546	4.942041	3.682834	2.340122	1.947501	2.087003	0.003691893	1.204314883	0.86743895	0.052183731
ENSG00000197121	PGAP1	1.578274	1.609956	1.571661	1.661526	1.41347	1.61734	2.243825	1.422457	2.012324	0.648181426	0.065252557	0.711215443	0.052228934
ENSG00000174437	ATP2A2	67.25088	69.708244	73.765623	75.837929	73.133767	76.00168	68.705736	64.157076	73.01202	2.54E-08	0.281264485	0.511632525	0.05226073
ENSG00000135250	SRPK2	16.78071	17.423613	17.621706	15.764585	16.386814	14.29617	17.304093	17.041445	16.363632	0.550878579	-0.054709176	0.540152312	0.052438465
ENSG00000044115	CTNNA1	69.574929	69.758356	67.57244	57.236742	52.322993	55.561893	65.394779	65.593959	69.193505	0.000792394	-0.182156216	0.426918237	0.052481961
ENSG00000143033	MTF2	15.956412	17.013243	16.115027	17.267776	14.991637	16.398653	14.659268	14.791231	16.228315	0.009644165	0.212535628	0.577574719	0.05252039
ENSG00000165916	PSMC3	152.143317	143.704868	138.749007	124.349441	120.257577	127.943992	137.23183	153.070795	134.007893	0.472549404	-0.04832522	0.479674332	0.052551803
ENSG00000141446	ESCO1	5.106282	6.74172	6.324155	6.112833	5.63805	5.587803	5.778988	5.758124	5.783299	0.579520596	0.076933522	0.688295156	0.052636104
ENSG00000102910	LONP2	7.388212	5.889004	5.472263	6.070472	5.593562	5.950461	5.798021	5.690032	6.976414	0.436534092	0.076972222	0.623627945	0.052661995
ENSG00000161010	MRNIP	7.449195	7.775606	8.361812	8.283068	6.534571	7.583295	9.017828	6.324443	7.803748	0.635008834	0.065439531	0.721198952	0.052704995
ENSG00000099901	RANBP1	247.737761	262.253977	251.196396	223.852013	222.750393	224.05135	252.552433	249.698193	239.387044	0.557019239	-0.03908747	0.481118646	0.052787908
ENSG00000075239	ACAT1	33.096588	37.083422	36.357196	34.989494	35.5776	34.247677	34.914611	34.518153	34.293307	0.117295148	0.124495608	0.561794121	0.052854854
ENSG00000284024	HSPA14	5.680047	6.099884	5.976601	6.039542	5.345875	5.583896	5.889461	5.351789	5.185614	0.539116263	0.071823403	0.639174276	0.052930778
ENSG00000170234	PWWP2A	5.061975	5.07833	4.960545	4.798631	4.39941	4.152949	4.864593	4.278818	5.157204	0.737814529	-0.051568272	0.687510509	0.052949077
ENSG00000168264	IRF2BP2	60.428335	57.347416	55.64517	55.102119	52.616189	56.735285	55.961605	51.879132	61.171579	0.290485578	0.070783338	0.476747135	0.053002952
ENSG00000112312	GMNN	107.460303	102.960355	107.877077	84.90996	76.799702	86.156764	109.92411	100.508231	99.912755	0.000829001	-0.21042148	0.497856593	0.053076743
ENSG00000185414	MRPL30	36.911818	35.538684	35.517136	31.554287	31.530203	30.149832	36.106354	34.189184	34.517346	0.416864429	-0.061197898	0.500867354	0.053080841
ENSG00000149300	C11orf52	4.895444	3.506931	3.767006	3.169844	2.659661	3.865845	4.048717	4.072276	3.613504	0.570303942	-0.164291048	0.834340634	0.053091602
ENSG00000101442	ACTR5	3.468585	2.99959	2.963739	2.68854	2.617878	2.915309	2.670407	2.847064	3.692264	0.81924369	-0.05048907	0.790456343	0.053125803
ENSG00000107201	DDX58	3.184793	3.279501	3.332605	10.211568	10.168863	10.63174	3.449975	3.048843	3.038193	3.92E-115	1.799656876	0.712937838	0.05314071
ENSG00000012660	ELOVL5	83.209809	88.767928	87.772363	105.82902	97.258083	97.6731	90.725353	82.232187	82.479724	4.56E-13	0.350998211	0.489197376	0.053160846
ENSG00000169155	ZBTB43	3.483159	3.271765	3.589777	3.131732	2.962363	3.334824	3.219108	3.543858	3.394621	0.974605169	0.005548236	0.677795452	0.053175147
ENSG00000189067	LITAF	11.375613	11.581788	12.590568	11.460204	12.048448	12.664966	11.451577	11.259992	11.73553	0.061504598	0.192842872	0.642897869	0.053197102
ENSG00000198056	PRIM1	23.95635	23.607763	24.460324	25.746933	22.267477	24.137592	22.775969	22.127829	25.346402	0.106634103	0.148176484	0.592619657	0.053299031
ENSG00000046604	DSG2	17.47909	18.039888	18.618532	13.248127	12.702666	12.917807	17.112283	16.789535	18.862437	1.24E-08	-0.329821878	0.482668964	0.053610586
ENSG00000132840	BHMT2	5.581038	5.847491	6.08577	3.518709	3.710553	3.3995	6.337748	5.477516	5.203036	0.0000149	-0.572202145	0.741714873	0.053629247
ENSG00000129636	ITFG1	10.48865	10.895361	9.541679	9.109233	9.235069	9.180622	9.976439	9.752924	10.390845	0.816390916	-0.029051259	0.607749216	0.053723513
ENSG00000188725	SMIM15	17.232012	18.892841	20.9245	20.263239	17.50749	18.379816	19.051046	17.573942	18.910063	0.213657312	0.123997564	0.600239518	0.053798194
ENSG00000102390	PBDC1	4.796451	6.961951	6.523089	6.124299	5.083147	5.646223	6.309668	5.639818	5.708149	0.852983707	0.049703526	0.806752902	0.053868835
ENSG00000182628	SKA2	33.030312	37.412364	34.4555	33.680311	33.835398	33.2951	34.898898	35.093972	36.619158	0.331634583	0.090951523	0.582359336	0.0538895
ENSG00000167173	C15orf39	15.290712	14.780766	14.543652	12.24611	11.815168	13.108696	14.262756	13.388125	16.605265	0.094925254	-0.13641496	0.577594835	0.053900955
ENSG00000100744	GSKIP	7.852286	8.492259	8.849281	7.495691	6.638488	6.966276	8.05122	7.878669	8.523966	0.492344045	-0.091571531	0.666308731	0.053902951
ENSG00000118680	MYL12B	146.662126	147.539753	149.902229	125.08321	123.401249	128.952508	144.030304	147.664851	139.124623	0.113178407	-0.094016087	0.488575801	0.05391901

Gene ID	Gene name	Ctrl-1 FPKM	Ctrl-2 FPKM	Ctrl-3 FPKM	IFNγ-1 FPKM	IFNγ-2 FPKM	IFNγ-3 FPKM	Sora-1 FPKM	Sora-2 FPKM	Sora-3 FPKM	Ctrl vs IFNγ DESeq2 FDR	Ctrl vs IFNγ DESeq2 log2FC	Ctrl vs Sora DESeq2 FDR	Ctrl vs Sora DESeq2 log2FC
ENSG00000108306	FBXL20	1.258112	1.372015	1.357531	1.322724	1.586118	1.51648	1.519845	1.227101	1.394716	0.177123583	0.182954313	0.73442785	0.054084369
ENSG00000121892	PDS5A	40.816665	44.650271	45.371607	46.677701	44.529313	43.418691	43.925361	40.135642	44.735472	0.002100674	0.179160455	0.459036179	0.054109214
ENSG00000127870	RNF6	8.297139	8.282531	9.302041	9.172927	9.706725	9.055098	8.273474	8.706604	8.410306	0.009077299	0.232768614	0.610712473	0.054264035
ENSG00000176390	CRLF3	4.337788	4.998389	5.050341	3.810372	3.809405	4.216632	4.733198	4.289306	5.032913	0.3435149	-0.141499865	0.710959214	0.054359728
ENSG00000091428	RAPGEF4	4.234857	4.51857	4.029846	2.18469	2.227931	2.426795	4.129964	3.643224	4.656383	1.59E-11	-0.750978014	0.685650719	0.054360503
ENSG00000138759	FRAS1	15.337874	14.492974	15.271284	8.416211	8.172787	7.884041	14.192022	14.362529	16.059624	8.04E-48	-0.734283725	0.453046652	0.054427003
ENSG00000175220	ARHGAP1	40.885488	40.174296	37.71249	41.373724	38.801838	40.502224	38.02107	36.265383	40.63483	0.005708425	0.166069418	0.46244402	0.054473475
ENSG00000157593	SLC35B2	77.652377	71.342439	70.51664	50.559704	56.192487	53.722723	67.904618	70.902677	74.49518	0.00000332	-0.311789497	0.494518252	0.054477417
ENSG00000165650	PDZD8	15.355786	17.2959	17.731348	13.00004	11.802913	13.708484	16.448381	14.759972	17.941298	0.001340848	-0.239980983	0.542725889	0.05448383
ENSG00000100056	ESS2	3.610604	3.453393	3.346115	3.579997	2.947529	3.217241	3.850644	3.149773	3.547364	0.899142427	0.020148411	0.666205779	0.054491854
ENSG00000129534	MIS18BP1	8.510751	8.946689	8.30008	7.523498	6.611824	6.879719	8.351247	7.9454	8.76019	0.117115715	-0.140090973	0.55493181	0.054674891
ENSG00000161960	EIF4A1	947.941459	910.526626	884.725669	822.464118	713.479386	799.605746	822.866146	891.468969	921.503073	0.813360511	0.015945374	0.446819895	0.054728451
ENSG00000130706	ADRM1	93.214339	85.742523	86.709107	78.384204	80.910501	85.108461	82.251567	87.625389	88.071405	0.760821159	0.02815447	0.492641657	0.054748521
ENSG00000205220	PSMB10	2.796118	3.611771	2.56357	58.230292	56.921015	65.396253	2.557609	2.879919	3.143752	0	4.506546966	0.843190963	0.05486281
ENSG00000142694	EVA1B	3.684804	2.919626	2.315213	1.745245	2.677233	1.851091	2.745665	3.019228	2.951152	0.276420042	-0.358771883	0.856449397	0.054924401
ENSG00000196975	ANXA4	49.850782	53.065043	50.852916	33.150894	31.38377	33.401667	49.958617	48.418229	51.065727	3.35E-20	-0.508137657	0.43145599	0.054932489
ENSG00000183172	SMDT1	11.333757	11.386144	11.538371	10.024299	10.569737	12.956027	10.126303	10.65088	11.390687	0.420700474	0.116027852	0.671158522	0.055079636
ENSG00000173409	ARV1	13.138112	13.465963	15.065896	12.88188	11.873293	12.706554	13.964896	13.030444	14.136217	0.753966164	-0.048361722	0.676849848	0.055165178
ENSG00000257103	LSM14A	42.649004	47.612506	49.942884	45.205857	41.252643	41.338402	46.624373	44.145953	45.770629	0.922429647	0.010725572	0.521284331	0.055165975
ENSG00000090686	USP48	17.664159	16.835861	17.247136	18.185619	17.169127	14.042583	16.730114	15.89459	19.253189	0.607793847	-0.051714471	0.497030818	0.055317098
ENSG00000114166	KAT2B	3.286417	3.174871	3.279426	3.570246	3.66439	3.367837	2.862181	3.010569	3.635074	0.015472967	0.268599119	0.697508591	0.055363583
ENSG00000125850	OVOL2	1.374529	1.271412	1.469977	0.692081	0.797015	0.633988	0.954933	1.710096	1.344797	0.01440431	-0.800034691	0.881348086	0.055399828
ENSG00000100225	FBXO7	63.274965	64.25097	64.770156	92.40793	89.560911	89.710004	57.474798	60.663521	60.584793	7.57E-47	0.694257532	0.459552213	0.055469536
NewGene_5303	--	0.868903	0.627934	1.510222	0.718492	0.902373	0.540381	0.772459	1.109848	0.731811	0.678591814	-0.14082695	0.850790651	0.055485445
ENSG00000148358	GPR107	19.862068	19.294539	18.794603	17.388527	16.436167	16.914951	18.810366	18.100452	20.274373	0.49926061	-0.048737294	0.437498934	0.05552348
ENSG00000150551	LYPD1	3.373243	2.217456	2.459734	0.801206	1.270147	1.270748	2.357841	2.907855	2.481858	0.0000167	-1.115881466	0.835174988	0.0556174
ENSG00000161800	RACGAP1	19.719737	22.00118	20.282182	17.420201	14.548505	16.369825	21.07619	20.5914	20.753747	0.007329253	-0.213511549	0.512574388	0.055643192
ENSG00000162664	ZNF326	8.887715	10.636761	9.450408	11.233388	11.470176	10.956283	12.430567	11.893516	13.805029	0.0000878	0.336366138	0.616969492	0.055649364
ENSG00000121060	TRIM25	7.017518	7.451435	6.627953	22.593663	19.507242	20.953868	7.151473	6.245868	7.183232	1E-148	1.725584041	0.583272361	0.055650904
ENSG00000134453	RBM17	22.178554	22.684457	22.976692	22.377815	19.196484	21.234002	22.641402	25.751362	21.309689	0.433728485	0.065775243	0.511039796	0.055789063
ENSG00000106244	PDAP1	44.184598	42.681181	43.94299	38.723249	38.823943	38.801075	41.963232	43.496715	41.973046	0.744787437	-0.026310637	0.490088283	0.055874114
ENSG00000270885	RASL10B	17.694925	17.900209	18.027315	11.908876	11.886271	12.882462	16.827953	17.430288	18.015057	1.65E-08	-0.399695433	0.507627841	0.055938269
ENSG00000089053	ANAPC5	79.226422	76.526179	76.43591	80.458041	74.275264	75.339249	72.272449	72.786438	81.999331	0.028881785	0.123210594	0.438805334	0.05613779
ENSG00000186792	HYAL3	2.405605	1.975884	2.102636	1.190108	1.258309	1.649255	1.72668	2.290937	1.955043	0.169486396	-0.383428435	0.842684352	0.056258059
ENSG00000154767	XPC	12.255903	12.232305	11.423834	12.317272	11.660576	12.298056	12.181625	10.91162	11.994744	0.068837277	0.147039811	0.546340755	0.056274329
ENSG00000137802	MAPKBP1	1.479512	1.494757	1.77769	1.470822	1.226909	1.202343	1.493068	1.196626	2.012177	0.980096373	0.005633518	0.74696657	0.056327971
ENSG00000141543	EIF4A3	52.843973	47.505618	49.512699	50.400841	47.613778	51.05174	47.852785	44.425938	50.861183	0.058276785	0.141229016	0.494271334	0.056335841
ENSG00000136824	SMC2	16.805588	18.300584	19.269464	14.722534	13.318313	13.636362	16.522959	17.327459	19.207089	0.0000602	-0.261471944	0.50743062	0.056348931
ENSG00000159899	NPR2	6.673549	6.21729	6.255308	4.429964	4.467741	4.192845	5.854953	5.910021	6.925913	0.0000406	-0.403188908	0.637397744	0.056350969
ENSG00000134709	HOOK1	12.890288	14.775487	15.000237	13.234246	12.474447	12.046077	13.774269	13.227589	14.621834	0.784214645	-0.029312603	0.532296306	0.05641988
ENSG00000132842	AP3B1	13.405441	10.896771	10.396013	10.571947	12.788832	10.358794	9.757043	10.228315	10.85874	0.047485091	0.145144195	0.509000781	0.056421503
ENSG00000121310	ECHDC2	12.436467	12.013637	10.208158	11.171908	11.548258	11.614248	11.046852	11.217344	11.276652	0.57138994	0.089453523	0.708019973	0.056475954
ENSG00000106638	TBL2	8.54643	11.641947	9.329983	11.901918	9.723385	10.497077	10.856775	8.685774	10.213396	0.008331678	0.236838818	0.600401707	0.056642576
ENSG00000011143	MKS1	4.619129	4.577498	3.31007	4.505937	3.879807	4.045639	4.017627	4.321602	3.644676	0.609939127	0.112084438	0.784797867	0.056732008
ENSG00000164855	TMEM184A	19.446667	18.769996	19.006737	15.139556	14.673107	15.310087	18.521957	16.398912	20.356354	0.001039699	-0.202768436	0.505407513	0.056749516
ENSG00000141424	SLC39A6	21.751554	23.047883	23.435355	18.66492	18.83784	17.723881	21.984942	21.93431	22.205485	0.027267957	-0.155382802	0.481693498	0.056779903
ENSG00000176973	FAM89B	36.293381	32.702657	27.323075	23.845556	24.513006	26.996063	28.681785	30.211798	35.11397	0.069184559	-0.211558459	0.661481546	0.056799338
ENSG00000100220	RTCB	48.161251	45.231888	44.402618	41.329994	39.437012	41.401234	43.323387	44.717346	46.371975	0.769556916	-0.026464234	0.478704754	0.056811588
ENSG00000119616	FCF1	14.325877	12.57134	11.893985	13.792967	12.485516	15.37241	18.459185	14.884191	12.790116	0.838520968	-0.024167772	0.55493181	0.056928502
ENSG00000104714	ERICH1	5.187442	4.825445	5.965215	5.626876	4.508229	5.331082	5.439196	5.031041	5.185923	0.50662851	0.119123804	0.73442785	0.056952085
ENSG00000146574	CCZ1B	51.970654	50.057045	54.712184	50.789738	50.071519	53.7787	47.893272	53.543615	53.359831	0.073283836	0.119736605	0.481813173	0.057075927
NewGene_6266	--	1.30206	1.232171	1.256143	1.621009	1.451292	1.500971	1.076057	0.847598	1.108142	0.00369444	0.401942994	0.75153004	0.057115062
ENSG00000127831	VIL1	79.222001	79.595024	79.716382	75.198717	73.973615	72.340241	80.003122	77.206028	81.493424	0.69534029	0.025754577	0.348603201	0.057116576
ENSG00000140534	TICRR	8.14922	7.778153	7.952974	9.757474	8.214027	8.972621	7.655289	6.3865	7.879162	0.001003104	0.275950584	0.5858007	0.057290923
ENSG00000122741	DCAF10	5.53483	5.896279	6.151862	5.67328	5.475786	5.936225	6.407246	5.627444	6.146275	0.14450255	0.138615325	0.583337958	0.057332368
ENSG00000232838	PET117	6.282105	4.955961	6.082164	4.17164	4.723633	5.792996	6.385825	5.483275	5.045052	0.733285439	-0.088952323	0.792598574	0.05739096
NewGene_610	--	2.259672	2.462621	2.410749	2.789276	2.810651	2.757	3.021797	2.406343	2.032111	0.05435426	0.319487849	0.785764397	0.057397232
ENSG00000023287	RB1CC1	14.692346	15.661021	14.530483	14.258537	14.39555	13.924755	14.487408	14.218222	15.419478	0.331878959	0.070745429	0.44705936	0.057409142
ENSG00000134363	FST	4.268664	3.83176	3.981008	6.740212	5.124624	5.805512	4.274037	3.444161	3.640161	2.17E-08	0.709449943	0.74035621	0.057552891
ENSG00000178188	SH2B1	5.961515	6.420838	6.915417	6.674938	6.028896	6.681708	6.002368	6.461423	6.901977	0.220220527	0.133588121	0.621761146	0.057696991
ENSG00000162430	SELENON	9.182138	8.526042	8.244511	9.658469	7.739202	9.426929	8.477758	7.532883	9.632208	0.069299492	0.190686113	0.598123257	0.057774688
ENSG00000062822	POLD1	19.002066	17.062751	16.591907	15.27237	13.679474	14.265905	15.804494	15.69993	19.507273	0.120143792	-0.130860137	0.581567191	0.057812532
ENSG00000165516	KLHDC2	21.920211	22.007125	22.247122	22.185675	23.190915	21.600843	23.957681	21.124783	21.319737	0.046260631	0.152452039	0.525965413	0.057892037
ENSG00000152779	SLC16A12	0.501577	0.466031	0.581559	0.320561	0.383051	0.245086	0.479642	0.463192	0.569596	0.086221313	-0.565530842	0.856243669	0.057919839
ENSG00000103042	SLC38A7	12.318455	10.884356	10.148031	9.414489	9.558974	9.749856	10.386198	10.741486	11.908887	0.529910005	-0.07003699	0.588040443	0.057928463
ENSG00000173715	C11orf80	16.414888	15.324888	15.449547	10.327325	10.522859	10.125674	16.037716	15.000013	14.723221	0.00000173	-0.434834659	0.59066678	0.057930019
ENSG00000119878	CRIPT	6.167399	6.720661	7.258802	5.395852	5.484807	5.483098	6.244259	6.808478	6.596119	0.302347122	-0.15222933	0.702749631	0.057977292
ENSG00000075945	KIFAP3	4.34782	5.273279	5.208781	4.630585	4.082951	3.559282	5.055796	4.801357	4.688669	0.404611581	-0.135085856	0.694304931	0.058023213
ENSG00000143815	LBR	86.214327	95.473019	97.6033	84.017621	78.505874	78.741069	91.836478	86.189565	95.137842	0.4197293	-0.059304204	0.4347845	0.058053712
ENSG00000161551	ZNF577	2.061622	2.678683	2.44638	1.946755	2.086878	1.620644	3.113425	2.375109	2.09493	0.299940336	-0.213413587	0.793947646	0.058058129
ENSG00000113360	DROSHA	18.656895	17.095384	18.316676	15.539977	13.699702	14.189636	17.142065	17.023539	18.119918	0.213657312	-0.106442897	0.455257324	0.05814528
ENSG00000114503	NCBP2	81.619301	80.211026	84.839059	76.32418	71.994988	75.141565	81.533155	77.655968	81.880763	0.998214326	0.000326146	0.417281328	0.058154407
ENSG00000126070	AGO3	1.28614	1.215551	1.391675	1.339638	1.320462	1.233606	1.650894	1.290108	1.488562	0.466138433	0.096261859	0.645860064	0.058231059
ENSG00000134248	LAMTOR5	33.827379	32.685432	30.142411	24.532839	26.098718	28.750443	33.536742	35.601414	30.53903	0.264119195	-0.132298242	0.672794514	0.058317839

Gene ID	Gene name	Ctrl-1_FPKM	Ctrl-2_FPKM	Ctrl-3_FPKM	IFNγ-1_FPKM	IFNγ-2_FPKM	IFNγ-3_FPKM	Sora-1_FPKM	Sora-2_FPKM	Sora-3_FPKM	Ctrl_vs_IFNγ_DESeq2_FDR	Ctrl_vs_IFNγ_DESeq2_log2FC	Ctrl_vs_Sora_DESeq2_FDR	Ctrl_vs_Sora_DESeq2_log2FC
ENSG00000100023	PPIL2	26.346113	28.731361	24.162994	26.051832	25.728059	23.858994	27.586241	25.204246	25.378218	0.466254712	-0.057823762	0.458111129	0.058378248
ENSG00000157224	CLDN12	24.26762	26.270194	29.640932	21.648799	21.543271	20.361714	25.867807	26.007274	27.571971	0.036756608	-0.180606711	0.544405094	0.05846452
ENSG00000154380	ENAH	32.332394	32.276794	37.075148	27.279229	26.729551	27.997267	36.137328	33.728019	36.587697	0.009506423	-0.175918442	0.4753673	0.058492638
ENSG00000064419	TNPO3	24.343912	24.39255	24.480179	22.736042	21.263378	20.629676	24.809588	23.314179	25.057296	0.630990042	-0.040458485	0.420697107	0.058526789
ENSG00000114854	TNNC1	6.656121	6.008867	5.385654	3.767317	4.632898	5.002373	6.4771	6.001294	5.076879	0.277020398	-0.277428745	0.818216285	0.05854139
ENSG00000181027	FKRP	10.895931	9.270639	11.395698	9.421696	10.168083	10.856586	10.386659	7.942002	10.547641	0.700135039	0.053753115	0.637560455	0.058553047
ENSG00000058262	SEC61A1	148.997546	147.563173	138.958117	160.523036	156.061076	161.879538	138.028989	136.988274	150.36631	5.19E-09	0.28333738	0.371000588	0.058569289
ENSG00000173465	SSSCA1	45.804059	37.92998	40.499029	34.73469	36.288153	38.518107	41.085824	39.342297	42.691654	0.738366218	-0.046557456	0.604112874	0.058601799
ENSG00000244165	P2RY11	2.324733	1.873465	2.265479	1.818755	1.786544	1.809303	1.216349	1.259411	3.941657	0.705871162	-0.106235668	0.938659207	0.0586266
ENSG00000171307	ZDHHC16	13.370681	11.996427	12.895374	12.349063	12.943563	11.006933	11.166363	12.98198	11.81608	0.928764647	0.015607814	0.664199575	0.058677624
ENSG00000165912	PACSIN3	44.052957	42.447574	40.67542	37.702437	41.096904	41.759206	40.500283	39.264636	42.415388	0.527258452	0.060799658	0.472988161	0.058799268
ENSG00000164418	GRIK2	1.568205	1.483959	1.383357	1.048393	0.931196	1.026802	1.529587	1.443239	1.732711	0.012063521	-0.485328464	0.772824558	0.058812195
ENSG00000115211	EIF2B4	14.819714	13.613184	14.114232	11.844607	11.283506	11.886911	15.525481	12.796534	13.89295	0.221949164	-0.132105431	0.63484658	0.05887077
ENSG00000101076	HNF4A	32.256832	24.137394	22.985044	47.120951	39.569334	40.746379	27.826423	30.451168	38.523913	6.29E-29	0.677083779	0.475955121	0.058962446
ENSG00000013583	HEBP1	9.422827	10.263341	11.826544	10.106461	8.455345	9.148295	9.410075	9.741396	9.787203	0.977470158	0.00652749	0.725623866	0.05904879
ENSG00000154001	PPP2R5E	10.252423	10.571586	11.488723	7.833421	8.312277	8.264004	10.067241	9.783255	11.441865	0.000843462	-0.249118519	0.509944768	0.059055037
ENSG00000173598	NUDT4	29.803921	36.740628	60.361286	25.796422	48.598173	22.338869	52.255301	37.935045	39.811441	0.026179616	-0.165184649	0.501725494	0.059146437
ENSG00000001561	ENPP4	4.324244	4.508475	4.254369	4.203049	3.88652	3.787735	4.018801	4.107882	4.674262	0.96728994	0.006702987	0.623368461	0.059324255
ENSG00000021574	SPAST	4.191367	4.187205	4.257825	3.686759	3.375479	3.773599	4.109097	3.599256	4.424431	0.845768677	-0.028472625	0.618938708	0.059329511
ENSG00000128463	EMC4	74.301749	78.038103	79.024041	66.321066	71.155977	68.770842	71.879227	81.163448	73.8217	0.633404195	-0.042949724	0.5304333	0.059543989
ENSG00000131368	MRPS25	15.723537	15.324886	15.286885	14.734769	12.416613	13.414731	18.113955	16.719394	16.514494	0.332663535	-0.080713361	0.453314503	0.059548078
ENSG00000111906	HDDC2	33.558745	32.340319	33.944495	28.028892	25.029205	27.222449	31.353498	34.126519	31.593381	0.03807677	-0.191187754	0.574583325	0.059619979
ENSG00000129925	TMEM8A	31.012653	27.801068	25.821029	21.291477	22.182576	22.059787	23.9962	25.461296	31.888577	0.022601907	-0.184548227	0.603264703	0.059684917
ENSG00000072134	EPN2	4.447805	4.847055	4.553498	4.556504	4.220266	4.031892	4.702838	4.352735	4.818049	0.870039748	0.024201066	0.609523611	0.059806308
ENSG00000143891	GALM	6.82868	6.450943	6.378253	8.772029	9.467446	8.503884	6.387282	6.140869	6.659662	1.94E-09	0.587610022	0.639487406	0.059818086
ENSG00000079931	MOXD1	3.729584	3.778069	3.642101	3.017874	2.683624	2.624748	3.224011	3.953093	3.753048	0.0502879	-0.277514657	0.712290294	0.059829448
ENSG00000168811	IL12A	0.679429	0.572848	0.999151	3.0086	2.617512	2.204057	0.629724	0.792654	0.836201	4.19E-12	1.884714196	0.900532158	0.05984555
ENSG00000164088	PPM1M	1.14495	1.226714	0.986851	1.544164	1.5353	1.319447	1.065002	1.13155	1.095848	0.059262403	0.487365214	0.849195249	0.059870207
ENSG00000056050	HPF1	10.402598	11.038886	10.057038	10.498671	9.167173	9.126768	11.601804	9.130665	10.090014	0.93402599	0.015944409	0.701689632	0.059877863
ENSG00000008394	MGST1	179.329141	196.812809	195.828092	203.967124	193.248537	200.213826	187.087619	185.088388	187.53118	0.000386039	0.207376492	0.426918237	0.059922664
ENSG00000107957	SH3PXD2A	6.721044	6.876947	6.760773	5.514539	4.930758	5.299176	6.699493	6.284147	7.041643	0.000805687	-0.22339773	0.439241228	0.059923721
ENSG00000068724	TTC7A	2.794485	2.568493	2.682814	2.430464	2.321244	2.354292	2.563267	2.622781	2.792199	0.937074513	-0.014585686	0.6769154	0.059937671
ENSG00000239900	ADSL	44.358135	47.672215	39.747963	37.02342	33.72169	36.405275	36.72794	45.317191	38.862283	0.002754991	-0.185295022	0.450704486	0.059980372
ENSG00000162813	BPNT1	29.402976	26.571842	24.101674	22.366037	21.799816	22.914253	22.944919	26.782357	24.325183	0.785223167	-0.027075153	0.50460078	0.059994587
ENSG00000213903	LTB4R	3.183175	3.567547	3.909149	4.134147	3.046378	3.356925	3.326878	3.629638	3.881305	0.699527705	0.075096245	0.688943608	0.060058773
ENSG00000215915	ATAD3C	0.94466	0.648915	0.717849	0.461507	0.474353	0.646571	0.911453	0.556314	0.805847	0.217131563	-0.390039421	0.853820786	0.060079372
ENSG00000205726	ITSN1	8.827719	9.562306	10.39707	6.555972	5.92539	6.033396	9.667105	8.634113	10.378101	2.75E-10	-0.494395286	0.535227351	0.060104303
ENSG00000101247	NDUFAF5	8.64186	8.16444	7.505897	5.618019	5.186489	5.012608	8.067539	10.119172	10.6776	0.000158592	-0.359030124	0.581596829	0.060310215
ENSG00000060642	PIGV	3.472972	2.635329	2.580233	2.534073	2.788872	2.351928	2.663376	2.376454	3.249939	0.728244677	-0.087106126	0.790593362	0.060328216
ENSG00000163950	SLBP	74.733077	74.792677	77.684828	78.929896	71.335908	74.019074	74.885364	70.047106	73.443859	0.202411725	0.082361678	0.39655139	0.06039964
NewGene_4134	--	1.618972	1.87141	0.981366	1.359403	1.027175	1.328394	1.392684	1.816917	1.502053	0.863061552	-0.080238857	0.876283568	0.060454183
ENSG00000100147	CCDC134	4.511745	3.870064	3.692644	6.392364	6.890983	5.708514	4.208462	3.531204	3.980661	0.0000033	0.7931209	0.795693786	0.060531127
ENSG00000122042	UBL3	7.522542	7.426915	7.162848	4.828313	4.87893	5.237254	7.017453	6.696878	7.946622	0.00000153	-0.416378746	0.556500923	0.060572072
ENSG00000150630	VEGFC	3.981973	4.940093	5.689397	1.849624	1.2159	1.215145	4.585005	4.441395	5.273866	2.93E-17	-1.6259039	0.749074007	0.060579454
ENSG00000082293	COL19A1	0.819078	0.61889	0.594488	0.353917	0.210337	0.202104	0.484608	0.732949	0.671253	7.96E-08	-1.388753726	0.821174576	0.06069512
ENSG00000173653	RCE1	40.241112	37.966831	38.849287	30.986543	29.873637	33.454264	37.245524	37.285294	40.464168	0.034750751	-0.170520025	0.477384649	0.060763346
ENSG00000101146	RAE1	20.513146	21.148464	20.992243	17.047223	16.579184	16.915816	20.774139	20.925456	20.80539	0.018531777	-0.177078135	0.483397014	0.060815629
ENSG00000184752	NDUFA12	47.400837	45.846629	51.762287	42.30045	41.676567	41.729789	49.750792	51.19295	41.289596	0.531434521	-0.070408682	0.637352764	0.06110735
ENSG00000164062	APEH	33.690239	33.744757	31.237699	27.967858	27.047954	28.466355	29.792106	34.184819	33.987804	0.124335258	-0.108730026	0.486567031	0.061233329
ENSG00000153071	DAB2	23.689558	23.739297	22.88405	10.035866	9.325372	9.769076	23.054815	21.65527	22.766854	1.65E-80	-1.126623272	0.42826018	0.061282058
ENSG00000122643	NT5C3A	25.801939	27.460741	31.132852	33.999497	32.50345	32.156331	30.119928	27.56283	26.243926	0.00000628	0.370594699	0.585120655	0.061549389
ENSG00000102024	PLS3	60.366063	64.614696	62.758024	58.726269	59.984767	59.827858	60.082555	62.448941	61.521056	0.233484893	0.075456997	0.396103899	0.061559642
ENSG00000178425	NT5DC1	6.382499	7.423263	7.100798	5.709882	5.872325	5.905074	7.176406	6.89667	6.667048	0.248943758	-0.108500052	0.547762723	0.061560542
ENSG00000171560	FGA	19.78715	20.973645	18.344563	27.682117	22.012075	26.065962	21.188513	18.97473	16.561056	5.16E-09	0.490607882	0.61541511	0.061632363
ENSG00000095787	WAC	34.410901	31.617406	34.702973	31.156806	30.910322	31.423331	34.38296	34.263788	36.853092	0.730344062	0.029225368	0.39726365	0.061781822
ENSG00000100029	PES1	50.413854	51.404672	49.170927	43.352555	41.036325	45.513076	47.596104	51.65898	50.717922	0.367236331	-0.068405884	0.417881344	0.061829752
ENSG00000168256	NKIRAS2	22.029225	19.970383	18.700281	18.515176	15.258711	18.905151	18.896471	21.001918	19.251328	0.445247567	-0.081398292	0.525460537	0.061831729
ENSG00000170412	GPRC5C	33.215412	28.178795	24.114939	19.765922	23.684039	22.127015	26.825883	26.811806	28.954462	0.030415851	-0.233170564	0.594482528	0.061916696
ENSG00000164576	SAP30L	2.934478	2.8232	2.75725	3.032424	3.328133	2.899511	2.871058	2.86367	2.633374	0.019594263	0.262903144	0.635179546	0.061970391
ENSG00000108953	YWHAE	251.791755	283.292359	260.849844	228.662256	220.587348	221.307933	260.965923	254.80042	250.35897	0.282362392	-0.05896005	0.393746555	0.062110361
ENSG00000131051	RBM39	145.053876	133.300119	143.808163	137.996198	132.240321	129.369891	139.461786	141.560701	140.019003	0.058218824	0.111488403	0.333961287	0.062130354
ENSG00000146083	RNF44	14.442108	15.047436	16.96507	13.748854	12.972955	13.450582	15.407181	14.768205	16.659026	0.523557096	-0.065114635	0.520978199	0.062208022
ENSG00000058063	ATP11B	21.690267	24.831641	24.296762	19.24847	21.61292	19.195463	23.967099	21.691112	24.206325	0.032584275	-0.154939702	0.461225016	0.062217439
ENSG00000138399	FASTKD1	9.236499	8.905412	8.708843	5.793959	4.971358	5.051249	8.271889	8.574628	10.022753	8.71E-12	-0.650370197	0.61039552	0.062272331
ENSG00000012061	ERCC1	18.450189	14.851353	15.345683	13.110756	16.04682	12.942843	17.333396	16.568387	16.09392	0.546550765	-0.098336614	0.665032827	0.062274823
ENSG00000105835	NAMPT	12.634282	13.238085	14.7585	28.05144	27.161503	26.327262	13.158523	13.290445	13.479288	3.3E-72	1.153901501	0.505780679	0.062334408
NewGene_1425	--	1.915152	1.387577	2.476596	2.549069	2.671474	2.389696	2.035636	2.005768	1.522788	0.050294072	0.521630375	0.852465224	0.062744487
ENSG00000013573	DDX11	16.038601	14.606885	15.65005	14.135428	11.973944	13.120436	15.092268	14.350023	16.467336	0.1784381	-0.123773612	0.475671248	0.062774671
ENSG00000079156	OSBPL6	1.683337	1.973818	1.859793	1.847022	1.909119	1.703723	2.840777	2.122204	2.497952	0.337658631	-0.155809644	0.701847005	0.062855476
ENSG00000138074	SLC5A6	25.957598	27.098604	25.990501	19.051811	17.683997	17.608901	24.610084	25.167984	27.405359	6.66E-11	-0.398889649	0.426959873	0.062894613
ENSG00000125818	PSMF1	32.782333	31.547985	31.610414	38.736209	37.40863	38.294635	31.280365	31.64772	27.614135	1.16E-13	0.391262931	0.383588279	0.063089551
ENSG00000196715	VKORC1L1	10.581476	11.054783	11.645776	9.846134	9.107526	9.753512	10.908364	10.243519	11.523844	0.447941216	-0.066536284	0.45723117	0.063091267
ENSG00000167977	KCTD5	17.331799	18.956596	17.138971	17.867729	17.049906	16.691442	17.269199	16.331654	18.80628	0.285359785	0.097036038	0.50714825	0.063094188
ENSG00000116030	SUMO1	93.226767	101.240758	102.729505	101.879345	96.299225	93.049071	96.598404	99.534198	97.826186	0.592824976	0.043496665	0.418765609	0.063230729

Gene ID	Gene name	Ctrl-1 FPKM	Ctrl-2 FPKM	Ctrl-3 FPKM	IFNγ-1 FPKM	IFNγ-2 FPKM	IFNγ-3 FPKM	Sora-1 FPKM	Sora-2 FPKM	Sora-3 FPKM	Ctrl vs IFNγ DESeq2 FDR	Ctrl vs IFNγ DESeq2 log2FC	Ctrl vs Sora DESeq2 FDR	Ctrl vs Sora DESeq2 log2FC
ENSG00000244687	UBE2V1	54.652519	58.251087	63.00831	57.667722	52.51458	54.617673	52.174442	57.955151	62.688559	0.531117252	0.050898315	0.469386128	0.063250187
ENSG00000122565	CBX3	220.214965	259.640729	279.495127	266.183572	242.549489	254.546906	243.357138	247.424975	249.855872	0.058407526	0.152469242	0.495488606	0.063396535
NewGene 2547	--	1.508377	1.745041	1.316374	1.712656	1.521453	1.695273	1.42182	1.521122	1.438418	0.697407244	0.260578561	0.903429307	0.063517197
ENSG00000123562	MORF4L2	119.61874	110.376611	108.691435	107.616123	113.588427	105.868265	112.799648	109.139348	104.745575	0.086068961	0.10899477	0.428615721	0.063519001
NewGene 1384	--	2.879844	4.204755	3.575824	3.384519	2.931394	4.104873	3.462475	2.778755	4.094982	0.774762547	0.071515376	0.752470807	0.063535715
ENSG00000139725	RHOF	6.621751	6.804791	4.751513	5.353678	6.821569	6.51379	4.175796	5.512247	6.621491	0.178245734	0.250164311	0.769672615	0.06353758
ENSG00000162227	TAF6L	21.909978	21.016842	18.162502	17.764339	17.455764	19.742615	19.961374	19.039023	20.886733	0.959048469	-0.00785933	0.543288228	0.063550019
ENSG00000104689	TNFRSF10A	0.883415	0.78813	0.93791	0.890164	1.199245	1.033691	0.807922	0.875696	0.89809	0.165275385	0.397495513	0.841732594	0.063559403
ENSG00000080845	DLGAP4	26.653352	23.001182	24.731036	13.359096	13.506709	13.341436	26.763045	22.450068	25.227441	4.34E-24	-0.688872095	0.456139938	0.063579481
ENSG00000136167	LCP1	0.851384	0.690874	0.736437	0.547325	0.671188	0.699116	0.64683	0.618823	0.978868	0.777736871	-0.098477522	0.837991965	0.063614566
ENSG00000165775	FUNDC2	9.382211	7.771664	7.84307	6.19263	6.633756	5.971235	7.387715	8.780589	8.39615	0.006627053	-0.26234408	0.596932869	0.06365474
ENSG00000132394	EEFSEC	10.135291	10.240897	8.317227	7.462263	7.563633	8.017643	9.697506	8.579486	9.791349	0.193548162	-0.168368997	0.632801956	0.063659849
ENSG00000205339	IPO7	72.528004	77.818621	81.739795	66.736455	64.199814	66.169372	76.082438	72.436843	79.159011	0.168169476	-0.088161436	0.364923675	0.063781765
ENSG00000166833	NAV2	8.8711	8.389313	8.876427	5.081607	5.841154	4.642222	7.977826	6.127222	9.042295	3.11E-13	-0.48557061	0.434332629	0.063797517
ENSG00000264364	DYNLL2	9.49795	10.350238	9.811749	13.983612	13.033841	14.093327	10.10618	9.829994	9.086609	3.32E-24	0.618534472	0.506098656	0.063813419
ENSG00000224578	HNRNPA1P48	7.109321	6.521705	6.614383	6.611759	5.734629	5.653022	6.810338	7.022368	5.978934	0.902295008	-0.024920095	0.704101911	0.063919617
ENSG00000179988	PSTK	3.183572	2.839872	2.540822	2.419617	1.932296	2.078331	2.772407	2.953485	2.069676	0.317692029	-0.283800742	0.82080509	0.063984137
ENSG00000197483	ZNF628	4.384589	4.721102	4.027461	4.717826	4.285317	3.907348	4.171775	5.096071	4.586096	0.93662433	-0.015594729	0.668809919	0.06410769
ENSG00000125246	CLYBL	7.775628	6.995656	7.845642	9.315176	8.466966	7.205734	6.753043	7.831981	6.045394	0.741293728	0.053902737	0.639439626	0.064112991
ENSG00000112494	UNC93A	2.184787	1.839013	1.487205	0.775441	1.04904	0.649322	1.860053	1.54456	2.031219	0.000338324	-0.983389348	0.816298198	0.064176661
ENSG00000198791	CNOT7	23.883985	27.608705	27.02238	25.575678	25.379545	25.899591	26.683109	27.331846	28.015256	0.628570206	0.041793527	0.417839458	0.064226634
ENSG00000136717	BIN1	5.957853	6.536211	5.14555	5.333454	5.697552	4.918248	5.504428	5.483832	6.081569	0.938578252	0.016367734	0.677094738	0.064353985
ENSG00000183011	NAA38	32.84286	34.020188	28.621668	26.11774	31.241267	30.402929	34.274077	33.477642	26.396162	0.949758019	-0.011019688	0.669137546	0.064535087
ENSG00000011454	RABGAP1	9.918257	8.905318	9.901978	8.931639	8.267033	7.956706	9.498628	8.864987	10.068398	0.647751878	-0.049857466	0.499770639	0.064556195
ENSG00000125459	MSTO1	14.035085	13.06179	13.861305	16.981204	12.818562	14.783026	14.004821	13.552472	14.091073	0.004645348	0.296670879	0.535227351	0.06465361
ENSG00000265241	RBM8A	28.234308	28.387456	26.875327	27.070745	25.671867	24.719515	28.668007	27.61542	26.171354	0.945771509	0.006438876	0.377020072	0.064780824
ENSG00000167325	RRM1	82.478279	85.796906	83.742	68.52623	63.612967	67.128104	81.462119	82.635052	83.50307	0.000292815	-0.186238	0.324135115	0.064864474
ENSG00000133983	COX16	10.889358	12.40588	11.522897	10.314386	9.735949	10.506112	12.067474	11.961301	10.082527	0.784554081	-0.040115914	0.647253097	0.064880982
ENSG00000132912	DCTN4	18.020645	19.523019	19.441261	18.845975	19.142511	19.040457	18.576309	19.135683	18.268999	0.027999314	0.150590503	0.438112633	0.065166435
ENSG00000137404	NRM	14.466192	14.592264	13.820615	10.063827	9.695937	10.670812	14.502873	14.158992	13.45744	0.000650248	-0.342136263	0.569226048	0.065250374
ENSG00000134152	KATNBL1	7.332292	7.49573	8.505203	6.594125	7.072989	7.806684	8.6827	7.968611	7.848728	0.676662538	0.06273158	0.621921649	0.06525559
ENSG00000114544	SLC41A3	7.172706	5.774723	5.857341	6.22789	4.890383	5.874708	5.856047	5.880476	6.13122	0.978043714	0.006685662	0.677795452	0.065346085
ENSG00000167986	DDB1	110.364465	107.127229	102.214981	97.615009	98.604818	98.069701	102.31183	101.497882	110.618014	0.742322054	0.023728884	0.312406041	0.065377122
ENSG00000198959	TGM2	6.693991	6.370201	6.306982	47.780819	45.699585	47.85331	6.208811	6.078956	6.736993	0	3.013415542	0.50646827	0.065447043
ENSG00000155304	HSPA13	23.664333	26.626656	26.883581	26.789179	27.16396	25.79842	24.462908	24.784281	26.484985	0.005550754	0.195208131	0.434445695	0.065499121
ENSG00000111640	GAPDH	1880.071383	1688.354395	1523.590084	1475.738046	1541.79114	1550.581555	1626.612159	1701.105733	1700.017936	0.920016464	-0.011252998	0.4607905	0.065517276
ENSG00000159228	CBR1	11.404029	11.860834	11.110285	10.257178	10.360571	10.812069	10.736779	12.322516	10.844018	0.920016464	0.017679913	0.650785731	0.065518066
ENSG00000228696	ARL17B	4.323763	6.365291	5.687006	5.779696	6.314341	4.901611	6.048211	6.200772	4.703203	0.32607045	0.236189716	0.80096875	0.065534154
ENSG00000107371	EXOSC3	16.701591	16.965179	17.353962	17.459303	15.423343	15.299523	16.666358	16.34438	16.624961	0.684563334	0.048445231	0.530565263	0.065549225
ENSG00000149657	LSM14B	28.505223	27.880363	26.379337	26.972266	24.941413	26.316898	28.053085	25.601878	26.992497	0.400254987	0.069726301	0.446515116	0.065607568
ENSG00000204568	MRPS18B	80.824577	78.002579	76.03492	64.454643	60.345036	63.101196	74.256813	75.15464	81.203896	0.004172896	-0.174835991	0.366928686	0.065737749
ENSG00000120333	MRPS14	19.33038	20.932698	21.076812	20.450333	20.114886	20.086549	20.391909	21.591624	20.531747	0.08973857	0.139670382	0.518068261	0.065743157
ENSG00000163516	ANKZF1	7.550869	6.601568	7.428119	6.847864	5.84346	5.485821	7.451753	5.697021	8.029751	0.495754137	-0.104374836	0.660591165	0.065764029
ENSG00000175166	PSMD2	114.115351	116.065366	115.685877	101.612417	100.528953	102.353006	107.987203	110.892687	121.751531	0.554524723	-0.035736607	0.317685365	0.065780779
ENSG00000100614	PPM1A	6.340132	6.693735	7.814961	7.219343	6.628875	8.191625	8.633755	6.306718	7.458004	0.257020495	0.105976561	0.532747512	0.065834628
ENSG00000181852	RNF41	8.628153	9.264878	10.289932	8.324309	7.991916	9.812255	9.066946	8.369298	9.899967	0.452918304	0.068013237	0.460369732	0.0659422
ENSG00000101266	CSNK2A1	33.993521	46.312315	30.798331	41.158936	44.794935	39.359378	46.362267	43.187365	40.944227	0.206127414	0.074785577	0.331989868	0.066082362
ENSG00000116641	DOCK7	13.032225	12.654963	12.511498	10.132842	10.350655	10.641882	13.641259	12.395434	13.558717	0.003653813	-0.191483216	0.371935492	0.066105588
ENSG00000117569	PTBP2	4.919538	4.733165	5.086855	3.28271	4.186575	4.836835	5.543457	5.165784	3.969623	0.923734367	0.016160937	0.603788317	0.066144228
ENSG00000133195	SLC39A11	14.265979	13.793835	11.956803	10.159222	9.797899	11.102681	12.703485	13.335393	14.366846	0.01761029	-0.238007232	0.548982341	0.066205976
ENSG00000114859	CLCN2	1.960497	2.312523	2.069173	3.148118	2.889265	2.964623	2.353119	1.932711	1.992803	0.0000119	0.624526817	0.738829191	0.06626796
ENSG00000156374	PCGF6	7.380385	8.743807	9.900553	8.918699	8.34227	9.637341	9.231769	8.054433	8.240098	0.157668421	0.193451532	0.658753477	0.066323605
ENSG00000143183	TMCO1	85.238014	89.603663	87.028675	90.012313	96.774834	83.728603	94.497342	86.726848	98.570838	0.090499483	0.108813395	0.342902688	0.066388307
ENSG00000138363	ATIC	116.86619	113.729726	118.920713	93.464158	86.366755	89.383637	112.601462	111.621403	118.890285	0.00000735	-0.229464139	0.294108627	0.066488837
NewGene 4851	--	12.246221	11.263648	11.236987	11.698918	10.585083	11.553427	11.354938	11.045201	11.729905	0.471952017	0.109251636	0.642897869	0.066602792
ENSG00000035928	RFC1	57.585611	60.270192	59.668287	67.727972	70.482803	68.102037	58.259266	58.764582	60.598901	4.48E-13	0.359263943	0.296552234	0.066645938
ENSG00000110628	SLC22A18	8.346659	6.460221	4.831812	6.028187	7.846937	7.606964	6.003467	6.059866	7.01886	0.144343114	0.297226597	0.757373564	0.066672349
ENSG00000120860	WASHC3	4.958473	5.185126	5.204068	3.736645	4.000827	3.803296	5.382322	4.148617	5.602411	0.241268123	-0.264205168	0.775565218	0.06670136
NewGene 4867	--	0.807406	0.75135	0.862266	0.763407	0.605779	0.626657	0.74192	0.855423	0.967238	0.584603465	-0.198130823	0.834744683	0.066837581
ENSG00000087095	NLK	7.629027	7.559455	8.013017	7.627387	7.440106	7.144183	7.30324	6.982812	8.079833	0.550853136	0.067029607	0.530737063	0.066863588
ENSG00000123609	NMI	1.423348	0.661839	0.954203	39.084653	35.117375	38.467731	0.876202	1.107283	1.000679	1.63E-79	5.344145669	0.877861219	0.066896646
ENSG00000166278	C2	55.872095	49.876573	48.417014	258.279073	247.310588	238.295259	52.594105	50.706828	45.831353	0	2.363758609	0.427584228	0.066917641
ENSG00000108883	EFTUD2	51.047399	44.656058	48.420348	45.116358	37.531189	39.535297	42.229632	44.828635	52.572509	0.096744038	-0.093663519	0.325460647	0.066982633
NewGene 4771	--	0.787974	1.340812	1.498493	1.030695	0.929994	1.222959	1.389622	1.068012	0.865044	0.16035839	0.472802754	0.854541877	0.067043983
ENSG00000144034	TPRKB	29.480247	31.621318	32.269637	27.725041	25.205749	26.537922	31.339728	31.373366	28.610984	0.520250114	-0.077583617	0.574827118	0.067130817
ENSG00000133226	SRRM1	31.105753	30.170133	31.247173	32.658713	27.929341	30.487114	32.223499	28.609879	33.785476	0.149905227	0.112416095	0.367332913	0.067137147
ENSG00000196182	STK40	17.768397	18.345232	17.628384	15.082997	15.726771	16.029058	15.990413	17.101165	20.662161	0.258577514	-0.093200917	0.502013402	0.067267738
ENSG00000125648	SLC25A23	42.257142	40.667025	40.354107	41.668891	40.430829	42.388243	38.589048	38.422036	45.413309	0.004652982	0.164523198	0.403966472	0.067270929
ENSG00000213639	PPP1CB	16.38347	17.67376	16.438471	15.582923	16.746131	16.103088	18.315975	16.621275	16.300887	0.253475611	0.089872285	0.476686075	0.067360435
ENSG00000187049	TMEM216	17.740502	15.720067	16.889255	13.844927	14.697643	11.897925	11.514858	16.069775	18.973587	0.328074775	-0.125208691	0.631346902	0.067390028
ENSG00000163655	GMPS	18.941896	18.491062	20.503309	18.005365	17.964453	18.634602	19.498564	17.939049	18.396585	0.238411614	0.073645024	0.355999021	0.067411852
ENSG00000048471	SNX29	1.557196	1.71442	1.496246	1.203802	1.060188	1.180469	1.727965	1.445375	1.609521	0.028390663	-0.298671576	0.642897869	0.0674648
ENSG00000153914	SREK1	22.365962	28.58947	29.95239	32.545713	29.680881	25.477218	23.935043	25.021696	29.206925	0.091033414	0.152796365	0.486567031	0.06747304
ENSG00000103591	AAGAB	26.768779	27.089721	24.53355	23.396677	23.93769	24.03476	25.91857	24.769284	27.032969	0.886314667	0.014915283	0.407399208	0.067672041

Gene ID	Gene name	Ctrl-1 FPKM	Ctrl-2 FPKM	Ctrl-3 FPKM	IFNγ-1 FPKM	IFNγ-2 FPKM	IFNγ-3 FPKM	Sora-1 FPKM	Sora-2 FPKM	Sora-3 FPKM	Ctrl vs IFNγ DESeq2 FDR	Ctrl vs IFNγ DESeq2 log2FC	Ctrl vs Sora DESeq2 FDR	Ctrl vs Sora DESeq2 log2FC
ENSG00000138286	FAM149B1	6.708504	6.39682	6.352242	5.160422	5.84515	5.234452	6.222065	6.594034	6.528671	0.304677506	-0.112539558	0.50616384	0.067815031
ENSG00000198668	CALM1	24.615822	24.648824	24.156122	22.96547	23.749796	22.733728	24.704887	24.837831	24.742204	0.307989647	0.075815532	0.336701839	0.067848915
ENSG00000198399	ITSN2	6.603214	6.958674	7.192443	6.537687	6.667957	6.499886	7.056498	6.206766	7.109372	0.65222638	0.051032424	0.509919108	0.067863016
ENSG00000213995	NAXD	20.635147	22.039259	22.067033	19.834807	17.632247	20.161861	20.995848	20.322296	22.227852	0.781614732	-0.029950475	0.423029212	0.06786947
ENSG00000144645	OSBPL10	1.106611	1.148209	1.276978	0.676168	0.621296	0.650734	1.189707	1.054574	1.253049	0.00000244	-0.76606622	0.709140972	0.067877413
ENSG00000129226	CD68	21.481128	21.357269	18.886447	30.710583	25.773659	28.789791	20.711113	20.81688	19.025272	2.88E-14	0.609637066	0.539704187	0.067931448
ENSG00000146731	CCT6A	266.700689	267.415892	274.738784	243.499822	234.036195	237.44181	265.401268	254.133581	276.318683	0.609666981	-0.030459275	0.238619363	0.067933131
ENSG00000103966	EHD4	7.169987	6.706069	7.004217	5.491727	5.527658	5.711516	6.419991	6.620274	7.504184	0.037278427	-0.171769514	0.476686075	0.067966261
ENSG00000158169	FANCC	10.963403	8.857521	9.039522	6.615657	6.113151	6.839577	9.206437	8.499687	9.57612	0.000092	-0.324611135	0.478655917	0.068065877
ENSG00000132964	CDK8	9.103447	9.466182	9.473204	8.594707	8.66067	8.192794	9.453707	8.656176	9.466208	0.955615997	0.007817322	0.503934379	0.068150924
ENSG00000205581	HMGN1	323.328406	322.975099	329.793541	269.956009	260.4768	271.544744	330.593989	316.03845	314.87311	0.020605566	-0.121902709	0.358107804	0.068194861
ENSG00000104679	R3HCC1	10.851841	10.345977	9.814196	9.768067	8.806404	9.317276	9.441177	10.146051	10.656338	0.955142441	-0.010048494	0.598822714	0.068208564
ENSG00000164649	CDCA7L	43.328005	53.793201	49.46906	45.58376	43.580052	43.524514	45.085868	43.029735	45.066106	0.78779918	-0.021527981	0.3158466	0.06821109
ENSG00000163590	PPM1L	2.379656	2.417489	2.375842	1.629154	1.949116	1.867494	2.515314	2.685223	2.437505	0.054042151	-0.211460619	0.560530183	0.068224221
NewGene_6663	--	10.380993	8.90223	10.093296	8.98647	8.652329	9.28715	9.947145	9.385141	10.477674	0.707481895	-0.049378878	0.545995308	0.068279583
ENSG00000089159	PXN	19.993236	17.504578	18.652803	17.496794	16.367357	15.79977	18.488205	18.078012	18.770944	0.892248176	-0.01455426	0.412225039	0.068320523
ENSG00000130726	TRIM28	189.247851	191.587465	176.348884	161.232492	147.423789	161.591471	169.209954	175.000439	199.362588	0.069163643	-0.106403585	0.366803997	0.068486698
ENSG00000198589	LRBA	10.627171	8.602309	10.158344	9.873612	7.933424	7.418148	7.439818	7.844252	7.815886	0.082737885	0.14225657	0.40540536	0.068510209
ENSG00000146856	AGBL3	1.060138	0.832549	1.094365	1.047963	1.030289	0.88962	1.130984	1.193108	1.696719	0.879880883	0.052188035	0.817955381	0.06851621
ENSG00000065882	TBC1D1	20.452528	19.620463	19.937227	15.326074	15.963417	15.326439	21.931018	19.724312	20.00447	0.037617205	-0.155995485	0.407582252	0.068568634
ENSG00000121653	MAPK8IP1	1.842499	1.952642	1.85783	1.085938	1.326122	1.397564	2.027987	1.699722	1.910607	0.047374823	-0.399800011	0.738673283	0.06857049
ENSG00000129347	KRI1	21.080052	19.065383	21.656426	18.693444	15.827048	16.180644	19.55963	19.28131	22.885873	0.125202495	-0.142674673	0.473120774	0.068710029
ENSG00000111665	CDCA3	19.079633	21.487518	19.739704	15.624647	13.622271	14.466714	21.854681	20.839947	20.406487	0.000947725	-0.300509849	0.506801654	0.068741254
ENSG00000147533	GOLGA7	23.301942	24.86029	25.821094	24.368429	22.49077	24.200321	23.085091	23.508644	26.633186	0.318958103	0.090996139	0.470554452	0.068912956
ENSG00000133704	IPO8	25.871753	26.642631	26.890357	30.361109	28.737426	28.959054	25.103511	23.301578	28.065316	0.000000125	0.273793276	0.363431132	0.068966807
ENSG00000198898	CAPZA2	39.604814	37.681198	37.310862	38.502886	38.419758	38.454063	36.459785	39.893151	40.942213	0.0000473	0.222136622	0.32971271	0.069000054
ENSG00000174371	EXO1	10.690345	9.365647	9.428834	8.888081	7.026828	8.068884	10.409553	9.469353	9.407492	0.147598335	-0.172958274	0.541080343	0.069005963
ENSG00000178301	AQP11	0.806475	0.566699	0.699166	1.726172	1.683013	2.313879	0.759131	0.616208	0.666381	1.62E-10	1.601658743	0.864540586	0.069072169
ENSG00000013523	ANGEL1	4.137237	3.948402	3.462534	3.234226	3.150632	3.057566	3.867831	3.644225	3.834507	0.319739022	-0.137482148	0.608706573	0.069079812
ENSG00000197181	PIWIL2	0.334562	0.246037	0.292202	0.212665	0.297845	0.219327	0.344145	0.286915	0.250839	0.793478382	-0.13200338	0.866812643	0.06918625
ENSG00000182481	KPNA2	171.873429	168.254614	171.646726	129.546047	124.6001	130.088371	169.273009	165.27076	168.267791	1.14E-08	-0.263992722	0.267012423	0.069238357
ENSG00000111802	TDP2	35.204936	35.696553	37.021171	32.723215	31.757248	33.521756	34.899535	35.171866	36.060042	0.931831022	0.008714126	0.373877431	0.069244242
ENSG00000143633	C1orf131	26.37415	25.649016	25.359433	26.773798	24.93843	23.007396	23.768349	26.241588	22.956116	0.283197885	0.102547242	0.488359496	0.069311498
ENSG00000104892	KLC3	2.920499	2.922879	3.258943	0.96805	1.328565	1.33238	3.022749	2.989369	2.925243	4.74E-08	-1.157478849	0.743476421	0.069314407
ENSG00000110042	DTX4	1.9499	1.894095	1.954794	1.704848	1.752591	1.545543	2.040162	1.68061	1.990328	0.708556741	-0.067022817	0.655496071	0.069342029
ENSG00000170296	GABARAP	177.472789	168.898691	168.160216	158.839991	165.995538	161.694601	172.341821	187.669898	157.462272	0.80002832	0.021295992	0.432355142	0.069384267
ENSG00000103671	TRIP4	20.812104	20.20057	20.041264	18.922856	18.211047	18.423436	20.39297	19.042428	20.613095	0.953953312	0.006973626	0.43581165	0.069481853
ENSG00000166797	CIAO2A	54.475725	46.823287	53.131974	81.970376	83.500624	85.405702	52.722254	49.914336	46.63302	7.77E-36	0.820808611	0.481428963	0.069545206
ENSG00000144048	DUSP11	13.791453	11.07745	12.242514	10.705853	11.063136	10.758554	12.292373	12.2547	11.664469	0.777320442	-0.044703973	0.605693726	0.069626299
ENSG00000023839	ABCC2	10.893785	11.267665	10.902277	15.765503	14.823768	14.457293	11.478787	10.138199	11.316876	7.36E-22	0.590529065	0.403055002	0.069656099
ENSG00000253797	UTP14C	1.782581	1.824758	2.195894	2.056532	2.109859	1.51764	1.855928	1.710265	2.157447	0.574791489	0.11520819	0.682385499	0.069666941
ENSG00000168116	KIAA1586	6.210115	7.406421	7.52678	7.290626	7.002063	6.858605	6.974986	6.712115	7.126258	0.236165845	0.145938419	0.586393963	0.069720361
NewGene_624	--	0.508926	0.738875	0.369073	0.54911	0.601435	0.46734	0.463712	0.403677	0.643627	0.83482545	0.095634808	0.859192873	0.069748221
NewGene_3468	--	0.258464	0.244151	0.165339	0.277589	0.259467	0.240754	0.285251	0.215382	0.149928	0.446001023	0.368805536	0.89684612	0.069758901
ENSG00000152475	ZNF837	3.970708	3.82986	2.999525	2.298425	2.570664	2.815073	3.231879	3.718136	3.639983	0.078515438	-0.346861387	0.732426777	0.069914421
ENSG00000160113	NR2F6	44.589409	42.524375	36.044007	45.156022	43.925814	50.644787	40.477816	38.49204	42.141621	0.000108573	0.331198895	0.465084879	0.070191859
ENSG00000067225	PKM	270.276385	251.178702	237.750507	217.041919	204.588582	216.396109	236.143982	252.205606	258.904307	0.118362057	-0.102355687	0.362064387	0.070207716
ENSG00000196588	MRTFA	5.082541	4.656596	5.412908	4.348113	4.480727	4.040441	5.21278	4.447626	6.133505	0.442293061	-0.096974892	0.597525945	0.070288095
ENSG00000113368	LMNB1	57.918667	61.234115	61.06848	53.066638	46.253872	53.219519	60.872812	55.154183	58.6107	0.162055133	-0.100277763	0.308263193	0.070391444
ENSG00000117899	MESD	19.103477	19.007884	19.440865	20.024233	18.878788	20.862398	19.238388	18.417317	19.320088	0.00498634	0.186396136	0.346497378	0.070398485
ENSG00000139990	DCAF5	8.875472	8.63653	8.495984	9.187998	9.444392	10.1798	8.508243	7.2752	8.839903	0.00000584	0.313424186	0.450792963	0.070406416
ENSG00000138814	PPP3CA	7.218201	7.875413	8.60392	6.039261	5.727813	5.641062	8.478422	7.142633	7.794021	0.004182478	-0.281930114	0.539102498	0.070409378
ENSG00000136444	RSAD1	6.835237	6.586096	6.897511	5.732186	5.023366	4.848228	6.656122	6.048282	7.193724	0.045511511	-0.24623503	0.574827118	0.070452525
ENSG00000060656	PTPRU	0.859302	0.792656	0.783299	0.496481	0.406315	0.423382	0.652676	0.76995	0.982337	0.000811326	-0.728838406	0.777509228	0.070468636
ENSG00000084674	APOB	419.329759	434.938691	414.343446	418.714446	393.870672	398.460946	416.549448	402.600844	431.704992	0.113083285	0.080066836	0.210473852	0.070620502
ENSG00000146386	ABRACL	14.844326	16.85877	20.024086	16.052788	15.131287	16.403511	16.933435	17.73513	16.129498	0.879493369	0.030047122	0.664948011	0.070637373
ENSG00000242802	AP5Z1	17.026825	16.273918	18.027727	20.106204	19.793177	20.613084	16.567045	15.856071	19.344334	0.0000139	0.272304541	0.417192644	0.070800292
ENSG00000185420	SMYD3	13.874926	12.980027	13.154433	10.564761	9.86916	9.934193	13.77356	13.17023	12.8591	0.021412366	-0.248204281	0.561120134	0.070819675
ENSG00000075461	CACNG4	0.722874	0.526089	0.31441	0.543194	0.345935	0.419943	0.502044	0.357806	0.700589	0.832865971	-0.111522534	0.875720126	0.070843399
ENSG00000164638	SLC29A4	43.834603	41.711523	37.878319	46.671689	46.807498	46.820051	39.724558	39.915661	44.535584	0.0000763	0.287120039	0.41636168	0.070908979
ENSG00000182890	GLUD2	13.274416	11.461576	10.310347	9.268406	10.361759	9.452113	10.137912	11.413074	13.031046	0.358260968	-0.121900931	0.604235029	0.071044921
ENSG00000167085	PHB	127.835723	124.950273	133.226822	85.885912	93.551847	94.735595	123.508549	127.574098	123.661886	4.43E-09	-0.2963926	0.320545538	0.071074404
ENSG00000075856	SART3	28.379624	29.647971	27.619943	27.257618	26.097395	25.255018	27.227341	25.156886	30.000633	0.378515303	0.06153304	0.357884044	0.07110602
ENSG00000147257	GPC3	408.577434	402.915885	370.309685	468.92363	469.680107	462.391097	386.344341	372.882075	389.000599	9.51E-17	0.415471621	0.283843663	0.071157209
ENSG00000110717	NDUFS8	180.915498	181.089318	165.178973	159.327368	168.766046	176.201398	173.085545	174.607413	169.313074	0.377862146	0.073494783	0.386836883	0.071161871
ENSG00000006282	SPATA20	19.887145	19.165428	18.011651	16.327477	16.257023	15.819741	18.027048	17.897163	20.096185	0.285042012	-0.092799287	0.440408119	0.071303659
ENSG00000107020	PLGRKT	7.678837	8.01338	6.726091	8.491645	9.757402	7.925629	7.222393	7.913733	6.917146	0.029167119	0.368811607	0.719303613	0.071330336
ENSG00000284770	TBCE	26.304805	33.293466	26.620669	23.492348	17.807599	17.181766	34.694285	25.941713	23.385072	0.04233047	-0.180613445	0.473088273	0.071486515
NewGene_3529	--	60.309511	64.005646	69.269657	48.262262	49.047495	47.900036	64.474201	58.481463	67.337179	0.0000362	-0.25553067	0.357024614	0.071620575
ENSG00000128245	YWHAH	74.150206	77.694774	78.133922	59.73459	58.054197	58.629381	76.097637	67.87939	82.557862	0.0000189	-0.235208847	0.364877552	0.071638256
ENSG00000103249	CLCN7	23.171747	21.983259	21.212179	18.86972	18.623731	20.017743	20.77403	20.190023	24.528108	0.490473499	-0.056247727	0.433889063	0.071652537
ENSG00000108604	SMARCD2	36.738668	34.00538	31.863668	36.368097	34.037749	34.912578	33.317965	32.788001	36.159778	0.013292332	0.179951634	0.394201082	0.071684849
ENSG00000177885	GRB2	40.802727	40.946332	39.198416	43.260829	43.904188	43.648392	38.509803	37.152017	44.574428	0.0000244	0.231515271	0.33151127	0.071735564
ENSG00000111361	EIF2B1	30.193984	31.1373	32.447602	27.793394	26.88092	27.586428	30.259226	30.121839	30.984833	0.649953914	-0.038946511	0.354929514	0.071779664

Gene ID	Gene name	Ctrl-1 FPKM	Ctrl-2 FPKM	Ctrl-3 FPKM	IFNγ-1 FPKM	IFNγ-2 FPKM	IFNγ-3 FPKM	Sora-1 FPKM	Sora-2 FPKM	Sora-3 FPKM	Ctrl vs IFNγ DESeq2 FDR	Ctrl vs IFNγ DESeq2 log2FC	Ctrl vs Sora DESeq2 FDR	Ctrl vs Sora DESeq2 log2FC
ENSG00000152061	RABGAP1L	6.859945	7.474708	7.926867	6.950087	7.279543	7.152787	8.907868	8.781311	8.71336	0.083481164	0.170836079	0.526211204	0.071804508
ENSG00000168872	DDX19A	21.925849	22.463674	22.775449	20.055969	19.591262	19.663045	22.445544	20.517764	23.732364	0.520389631	-0.053288008	0.369883424	0.071813228
ENSG00000163558	PRKCI	13.931923	16.601284	17.631969	16.445951	14.564728	14.81622	15.563062	14.937613	17.012987	0.497085925	0.075231149	0.488575801	0.071829512
ENSG00000092871	RFFL	10.338696	10.057221	10.373719	8.832356	10.184219	9.453491	10.503651	10.092927	13.49555	0.798186325	0.026506964	0.376936033	0.071882744
ENSG00000133466	C1QTNF6	7.22931	5.524924	5.30146	7.464854	6.991565	7.961675	5.22583	4.943603	6.771968	0.000202106	0.444706718	0.666646157	0.071951187
ENSG00000171421	MRPL36	34.431273	37.682896	34.660503	41.729426	35.384483	33.973082	34.699672	35.163079	35.436855	0.094192482	0.174939384	0.517473256	0.072211105
ENSG00000009954	BAZ1B	36.170691	33.538261	34.107739	32.880396	31.226982	32.210843	34.885053	31.96116	35.389855	0.643124251	0.034835559	0.304963672	0.072233545
ENSG00000169446	MMGT1	5.745785	6.430653	6.929585	5.909865	6.115684	6.32273	6.781803	5.649566	6.424094	0.478763256	0.090662489	0.569002808	0.072239867
ENSG00000183207	RUVBL2	50.38587	50.346454	46.961643	39.252177	37.602855	39.386111	45.213484	48.778126	50.410469	0.008267579	-0.17467845	0.348035086	0.072396318
ENSG00000104299	INTS9	6.337521	6.151019	6.379451	5.05973	5.601505	5.249467	6.004946	6.018539	6.591298	0.276228736	-0.144266289	0.568764285	0.072452819
ENSG00000054392	HHAT	1.901467	1.994113	1.600319	1.247799	1.389226	1.460343	1.726146	1.767088	1.901517	0.167716986	-0.278790318	0.715922156	0.072573602
ENSG00000136504	KAT7	14.488324	11.756847	12.580087	12.545726	6.911837	11.398057	12.297966	10.473627	16.086139	0.435189541	0.062386594	0.379442393	0.072576351
ENSG00000187325	TAF9B	4.786527	5.022051	5.434548	4.642456	4.702881	4.963327	4.867577	5.565381	4.549492	0.724027352	0.057500681	0.647104471	0.072660835
ENSG00000126803	HSPA2	1.18885	0.770748	1.021597	0.577808	0.706647	0.678024	0.766264	1.185072	1.303397	0.062296408	-0.538753529	0.81723769	0.072850467
ENSG00000112855	HARS2	14.103457	13.333256	12.815284	10.933315	10.725067	11.052926	12.568037	12.842645	14.991268	0.036778223	-0.19190319	0.486593089	0.072864729
ENSG00000177508	IRX3	1.083289	1.325228	1.147727	1.23333	1.099587	0.983743	0.982377	1.18133	1.333801	0.89670426	0.045830955	0.800211877	0.072948303
ENSG00000128311	TST	39.836907	35.693596	29.576394	32.621831	33.958582	33.043917	31.637569	35.709617	33.151447	0.720061088	0.047904543	0.550501653	0.07309625
ENSG00000068079	IFI35	5.894523	5.887685	5.518331	71.231432	71.617164	75.433835	5.358552	5.528275	6.196213	0	3.800001533	0.689690408	0.073102132
ENSG00000130511	SSBP4	17.402576	19.162437	17.772943	30.737776	28.768611	32.477304	17.671036	17.761001	18.754199	1.5E-34	0.913821768	0.466998848	0.073128312
ENSG00000157259	GATAD1	10.36502	11.054135	12.894248	12.305025	11.967715	10.907052	11.275065	10.431026	12.776594	0.062755863	0.187789419	0.51633197	0.073244375
ENSG00000143641	GALNT2	37.245441	35.67881	33.216534	39.741459	40.190353	40.370739	34.476086	32.713665	37.701157	2.34E-08	0.328014644	0.338177782	0.073813577
ENSG00000198498	TMA16	7.780612	7.781466	9.248149	8.258263	8.108515	7.52147	8.081659	7.955721	8.245845	0.718163378	0.061896434	0.609600119	0.073881966
ENSG00000213593	TMX2	111.003055	110.71733	111.353261	102.734388	98.59193	99.481436	106.843702	108.771876	111.04881	0.981681966	-0.001953608	0.257582015	0.073954096
ENSG00000126088	UROD	44.050073	43.315201	38.545099	37.545303	33.926389	39.605383	41.236515	42.886572	43.121901	0.631525671	-0.052967004	0.441900971	0.074197034
ENSG00000086205	FOLH1	1.873732	1.883691	1.977234	1.133467	1.694278	1.129917	1.789325	1.636423	2.265192	0.105323921	-0.393827894	0.743612243	0.074259293
ENSG00000148341	SH3GLB2	11.496706	8.943955	7.946208	8.44665	8.048638	8.609306	8.639446	9.002894	9.58674	0.884679815	-0.026191772	0.598931509	0.074281443
ENSG00000185664	PMEL	0.648793	0.926795	0.772967	0.853659	0.644413	0.85555	1.177652	0.484397	0.613911	0.673007751	0.178197369	0.859192873	0.074307908
ENSG00000039319	ZFYVE16	7.501452	7.793287	7.787576	6.820089	6.749096	6.540855	7.130337	7.363051	7.969184	0.596780401	-0.050137272	0.399443002	0.074391042
ENSG00000172172	MRPL13	52.435365	54.168772	54.386722	45.529448	43.754599	44.443223	52.102009	52.838941	53.770499	0.104175289	-0.122445505	0.361761214	0.07443317
ENSG00000120327	PCDHB14	0.349563	0.298352	0.259707	0.294015	0.212615	0.239081	0.207674	0.371826	0.318495	0.77359479	-0.140588774	0.862906245	0.074563805
ENSG00000196502	SULT1A1	32.408025	33.195524	31.467849	20.675661	22.977157	20.943281	29.649573	27.011269	29.159999	0.00000952	-0.344984981	0.460987109	0.074568742
ENSG00000266173	STRADA	10.22257	10.510658	12.009839	10.383099	8.875055	9.527349	11.305837	9.249677	11.701925	0.548529858	-0.076047965	0.535227351	0.074572342
ENSG00000113716	HMGXB3	13.079795	12.754418	12.592006	12.534418	12.301448	11.929515	11.61468	12.269133	13.95771	0.302515155	0.078349779	0.408280915	0.074725512
ENSG00000075151	EIF4G3	18.224827	18.556322	18.8846	16.468035	16.402218	15.669406	18.263318	17.671799	19.835192	0.699493372	-0.03211084	0.337933758	0.074745202
ENSG00000158966	CACHD1	1.792004	1.605932	1.392447	1.637844	1.299586	1.432651	1.701335	1.2197	1.756033	0.930136318	0.022454872	0.721090773	0.074797494
ENSG00000168394	TAP1	0.436087	0.298781	0.28081	334.175568	315.855469	341.108795	0.456868	0.211639	0.32998	0	10.05399518	0.887084908	0.074875273
ENSG00000087269	NOP14	41.607077	41.10384	42.882594	35.257166	34.351769	35.834471	40.343331	40.729515	43.282497	0.07730396	-0.107018168	0.268852119	0.074998532
ENSG00000196591	HDAC2	34.421391	36.318576	39.296937	46.999327	44.974268	20.788306	27.525408	35.158922	93.789879	8.87E-08	-0.251883293	0.224361001	0.075051964
ENSG00000132768	DPH2	14.008267	14.015965	13.878337	9.892726	9.390152	10.924577	13.044476	13.000605	15.68783	0.000392356	-0.320705888	0.464886897	0.075110778
ENSG00000168303	MPLKIP	33.444923	33.730863	44.291315	41.333332	28.90769	34.731289	38.582073	33.821939	44.52477	0.671927534	-0.038379221	0.346999986	0.075199316
ENSG00000160813	PPP1R35	13.332157	13.025	11.496342	9.703682	10.66291	10.869238	12.474154	11.994489	12.957284	0.414692745	-0.128609924	0.597939868	0.075243397
NewGene_1795	--	1.021204	0.619608	0.685857	0.743588	0.805347	0.744256	0.734121	0.629413	0.921143	0.95551367	0.036738603	0.879547072	0.075259673
ENSG00000164830	OXR1	8.495233	11.177373	9.442444	8.902743	9.327128	8.537139	9.322195	8.600806	9.73678	0.628370944	0.0597615	0.481428963	0.075336331
NewGene_1692	--	3.803232	3.010046	3.612245	2.459473	2.493182	2.726354	3.32353	3.04103	3.68456	0.033380451	-0.294061844	0.608663394	0.07553125
ENSG00000166398	KIAA0355	4.305196	3.910265	3.990175	3.371531	3.072477	3.052407	3.943408	3.606038	4.539252	0.031559467	-0.215763129	0.511273058	0.075568342
ENSG00000141441	GAREM1	1.334759	1.438363	1.844489	0.732214	0.800264	0.630338	1.881395	1.436936	2.058733	8.53E-08	-0.961504223	0.722069303	0.075586399
ENSG00000092470	WDR76	4.08316	4.161699	4.569176	4.062947	4.201691	4.145962	4.033002	4.035551	4.676235	0.722177184	0.055014312	0.562949242	0.075646082
ENSG00000139865	TTC6	3.035815	3.58632	3.373736	3.53001	3.758853	3.292292	3.93772	3.48843	3.974399	0.240002286	0.171015366	0.624555419	0.075660819
ENSG00000183255	PTTG1IP	123.669151	110.926998	108.796012	84.901899	88.434113	87.298051	112.497708	108.521931	118.918642	0.0000672	-0.249041664	0.302586291	0.075668235
ENSG00000090020	SLC9A1	3.004661	2.803024	2.745275	3.129173	2.938922	3.507102	2.572713	2.586084	3.37341	0.010265707	0.322523668	0.62539873	0.075715634
ENSG00000108091	CCDC6	5.770662	6.09658	6.257572	8.250299	7.403976	6.818424	6.125495	5.565622	6.237069	5.86E-08	0.455776298	0.433812485	0.075752577
ENSG00000164053	ATRIP	3.013675	2.01909	1.659175	1.758472	1.809175	2.552741	2.67474	1.810201	2.071763	0.78452059	0.061276831	0.685847365	0.075821374
ENSG00000071564	TCF3	65.94656	64.562141	61.452323	49.983436	46.854723	52.394554	62.746462	60.591889	71.924796	0.0000796	-0.21600144	0.302504679	0.075889678
ENSG00000082014	SMARCD3	4.199119	3.692319	3.872306	6.131231	4.481592	5.383528	3.75695	3.953151	3.516358	0.0000891	0.581019373	0.675789958	0.07589788
ENSG00000130748	TMEM160	10.896944	12.739482	8.998993	8.149578	11.168545	8.218194	11.848639	11.796578	8.47062	0.711483799	-0.097735293	0.747797971	0.075973426
ENSG00000154370	TRIM11	13.964967	12.975016	12.656947	10.367473	12.402629	13.211511	13.304876	11.925081	14.077315	0.065694738	0.160290554	0.389916539	0.076031566
ENSG00000071127	WDR1	111.9542	105.399128	110.147633	78.271655	76.041168	80.017073	103.969731	103.170855	117.317715	2.24E-11	-0.335571737	0.266288911	0.076065919
ENSG00000276180	HIST1H4I	4.220519	4.201537	4.386348	4.049669	3.462803	3.645133	3.561692	4.313075	4.811729	0.843413728	-0.050773488	0.726896666	0.076139492
ENSG00000244405	ETV5	8.157871	8.073617	8.995818	7.561297	7.559075	7.655782	7.513749	8.741958	8.719901	0.933602721	-0.011370988	0.515231789	0.076193099
ENSG00000204922	UQCC3	8.651531	8.138936	7.793485	6.562748	6.906008	7.487995	7.407226	8.728852	8.270773	0.515861124	-0.094673	0.583594003	0.076210713
ENSG00000164398	ACSL6	0.522919	0.291575	0.327025	0.136008	0.110218	0.206451	0.485542	0.29827	0.580472	0.03564748	-0.790778435	0.842545788	0.076317252
ENSG00000184863	RBM33	10.63516	10.412564	10.518195	10.341773	10.630729	10.118314	10.918127	9.740299	11.390117	0.004731287	0.173839843	0.322410441	0.076350504
ENSG00000133863	TEX15	0.933997	1.307099	1.360711	0.569345	0.504071	0.547457	1.173911	1.116084	1.213179	8.52E-11	-1.009661429	0.661931338	0.076450678
ENSG00000084070	SMAP2	9.967069	9.589561	8.774217	10.872666	9.624788	9.832681	9.770676	8.620364	9.602728	0.010001888	0.245824872	0.48846798	0.076485514
ENSG00000088832	FKBP1A	84.806783	84.023445	81.788279	75.337718	77.568889	80.225201	82.744934	83.634396	79.487333	0.546116178	0.047054959	0.329130263	0.07648676
ENSG00000057935	MTA3	18.337341	14.708446	14.793559	14.795289	15.174396	12.782503	17.329557	17.828886	13.478737	0.340494593	-0.099343263	0.44532084	0.076522953
ENSG00000155111	CDK19	7.540929	7.393341	7.841672	5.869529	5.834454	5.700429	7.059153	6.710642	8.352465	0.003898575	-0.218966027	0.432831884	0.076569067
ENSG00000262814	MRPL12	102.190758	96.662605	92.821289	80.969223	85.365807	88.384628	91.29805	97.395126	99.696831	0.605176307	-0.047401538	0.346751771	0.076607097
ENSG00000145736	GTF2H2	4.458091	5.398501	5.710262	4.443547	3.868912	4.563264	5.428226	6.234826	5.494994	0.488648433	-0.111415925	0.611743813	0.076706416
ENSG00000124641	MED20	14.933604	15.625498	14.992551	12.077617	12.069152	12.464965	15.26664	14.41418	14.876624	0.107926039	-0.144690473	0.416424181	0.076879671
ENSG00000186716	BCR	16.342765	14.357786	15.721915	14.494337	13.409054	14.310561	14.370542	14.001418	16.950706	0.978324991	0.003008073	0.389766603	0.076881617
ENSG00000284976	BX255925.3	12.645836	14.166119	12.273728	11.413791	11.723825	12.654284	12.517975	11.506512	14.738066	0.88429621	0.022078499	0.535005405	0.076899834
ENSG00000185088	RPS27L	9.250956	8.739242	11.67594	8.617676	7.381947	7.908787	12.181725	14.143123	12.891925	0.150488836	-0.180714379	0.587343877	0.076909319
ENSG00000097007	ABL1	14.95266	14.467027	13.591276	13.189873	12.329331	13.635415	14.072805	12.286009	16.132207	0.981452818	0.002740995	0.431207696	0.077141473

Gene ID	Gene name	Ctrl-1 FPKM	Ctrl-2 FPKM	Ctrl-3 FPKM	IFNγ-1 FPKM	IFNγ-2 FPKM	IFNγ-3 FPKM	Sora-1 FPKM	Sora-2 FPKM	Sora-3 FPKM	Ctrl vs IFNγ DESeq2 FDR	Ctrl vs IFNγ DESeq2 log2FC	Ctrl vs Sora DESeq2 FDR	Ctrl vs Sora DESeq2 log2FC
ENSG00000020426	MNAT1	6.639518	6.505569	7.390858	5.287285	6.444457	5.79144	6.566787	6.875072	7.003206	0.691741011	-0.063037819	0.539086053	0.077302251
ENSG00000154229	PRKCA	8.149278	7.534543	7.868829	9.200397	8.951374	8.576223	7.89385	7.456568	8.139202	0.0000012	0.318394994	0.341592333	0.077306575
ENSG00000153237	CCDC148	0.384682	0.318515	0.713014	0.471468	0.271334	0.282056	0.281028	0.402709	0.850595	0.658895243	-0.248515462	0.885049652	0.077366823
ENSG00000157036	EXOG	1.796974	2.298938	2.344288	2.120308	1.51529	2.001292	2.199208	1.821452	2.330497	0.995332052	-0.00192894	0.721881015	0.077368466
ENSG00000065600	TMEM206	6.279102	6.898063	6.957169	5.135426	4.411874	4.336417	6.25779	6.308799	7.380573	0.001249817	-0.389441966	0.555568704	0.077429265
ENSG00000125835	SNRPB	229.684511	226.983768	229.566291	190.822511	204.283138	210.331492	217.669051	226.254861	235.369445	0.740379082	-0.028378268	0.225555501	0.077497431
ENSG00000120725	SIL1	9.695459	10.558549	9.420074	8.711821	9.678977	9.611896	9.396556	10.486905	9.452891	0.653844629	0.064577085	0.555719635	0.077579611
ENSG00000170485	NPAS2	16.961001	16.166047	20.023461	12.956265	13.871297	11.951412	16.115412	15.968769	21.174496	0.000185284	-0.33176303	0.514736894	0.077660851
ENSG00000115310	RTN4	94.369362	92.245989	103.721062	75.395839	79.762425	79.745694	93.697394	95.101736	98.420472	0.028945801	-0.147343314	0.287847242	0.077904811
ENSG00000143942	CHAC2	5.1104	5.204487	5.68187	3.23157	3.484657	3.642134	5.19984	5.385556	5.214511	0.006696664	-0.475026451	0.673088192	0.078067432
ENSG00000157426	AASDH	5.021782	5.43229	5.06031	5.330477	4.751832	4.782958	5.345781	4.607022	5.231239	0.501970262	0.088892612	0.528619201	0.078073386
ENSG00000086062	B4GALT1	23.243263	25.370839	24.6954	22.56506	22.707031	22.158895	24.44586	22.784515	25.278114	0.733188311	0.029236613	0.293640874	0.078142344
ENSG00000160446	ZDHHC12	23.299292	21.995682	18.212909	17.117072	18.769226	19.710973	19.998902	21.087716	22.118505	0.835667407	-0.035105241	0.551432645	0.078166185
ENSG00000184205	TSPYL2	18.222487	16.794959	18.2554	22.132898	20.255103	19.669074	17.277119	16.216826	19.504404	0.0000021	0.346662601	0.407239741	0.078177823
ENSG00000100504	PYGL	16.899473	16.368376	17.68049	11.757993	11.282127	10.842432	17.36976	15.43949	16.027479	0.000000232	-0.382292205	0.37536567	0.078201247
ENSG00000104687	GSR	32.051693	33.850003	35.194828	34.856633	32.418694	35.166031	35.556216	31.829353	34.308997	0.011739053	0.166718375	0.310492057	0.078240161
ENSG00000152219	ARL14EP	12.458971	12.83822	12.700628	9.882292	10.545021	9.679243	13.236507	12.534711	12.384797	0.067084025	-0.174627159	0.438232018	0.078278655
ENSG00000198522	GPN1	24.222934	22.85185	20.67022	19.080057	18.038104	17.671418	23.707797	22.019173	24.805875	0.053715309	-0.158298807	0.383265648	0.078282414
ENSG00000125378	BMP4	5.826916	4.552836	4.783936	3.983332	3.782883	4.374803	4.522707	4.843437	5.03055	0.520389631	-0.124811832	0.653319	0.078390111
ENSG00000135932	CAB39	24.546587	24.838823	26.199253	24.699479	25.134012	25.051781	24.761454	23.882975	26.697865	0.04308532	0.132654129	0.291759365	0.078392479
ENSG00000170584	NUDCD2	1.821437	1.9141	2.148571	1.845089	1.730957	1.77407	2.079999	1.809452	1.937115	0.954339943	0.010624452	0.571127586	0.078476521
ENSG00000156873	PHKG2	17.246428	16.765506	17.420986	17.3426	16.618781	13.803698	16.207532	16.16522	17.624693	0.408897006	0.079063653	0.388490781	0.078531984
ENSG00000162627	SNX7	8.150271	8.592438	8.479684	12.502803	12.661924	11.899162	7.894769	7.742526	9.288099	2.45E-13	0.707034407	0.562023499	0.078576961
ENSG00000126432	PRDX5	222.968803	200.986986	186.078333	176.749798	188.263035	194.756755	193.838484	212.350519	198.342577	0.847337056	0.021458721	0.39991728	0.078599265
NewGene_4129	--	0.73043	0.921273	0.76164	0.752732	0.632494	0.462119	0.684513	1.012633	0.712975	0.573838454	-0.225240149	0.828488468	0.078603369
ENSG00000176018	LYSMD3	3.162561	3.723965	3.199934	4.205252	4.199341	4.677161	3.4914	3.00861	3.550288	0.0000109	0.487649863	0.577574719	0.07864519
ENSG00000175265	GOLGA8A	2.799917	3.542181	4.291747	5.794104	3.68586	3.723925	3.596327	3.495589	3.350469	0.198284792	0.438803508	0.657607038	0.078663895
ENSG00000066382	MPPED2	1.156291	1.220963	1.379197	1.251764	0.992215	1.093841	1.451423	1.139236	1.003961	0.902295008	-0.04591142	0.790393539	0.078770977
ENSG00000204308	RNF5	133.14595	129.262115	119.046669	116.482094	125.328552	124.44754	125.258995	128.485519	123.697319	0.253945095	0.089260467	0.329909587	0.078773496
ENSG00000150753	CCT5	133.11654	131.85293	128.865093	110.51556	108.896407	115.743491	128.69809	130.967575	137.73633	0.04233047	-0.102283584	0.228050326	0.078784978
ENSG00000272398	CD24	412.740043	438.839522	454.802801	399.36286	374.995066	403.383886	439.085277	409.070623	428.68696	0.920641847	0.008109198	0.232133899	0.078837988
ENSG00000160799	CCDC12	18.094493	17.067748	15.693031	17.140144	18.96696	17.468582	16.689981	17.347754	16.468804	0.082874749	0.205305289	0.542105497	0.078865538
ENSG00000005189	REXO5	4.040039	4.379255	4.223485	4.236989	3.341617	3.915186	4.259043	3.88731	4.158131	0.820654196	0.0459803	0.600636176	0.078949754
ENSG00000076248	UNG	58.519427	57.453749	57.386979	49.291002	48.493168	51.398841	57.673108	55.148921	59.214537	0.30832416	-0.070630934	0.238627481	0.078986324
ENSG00000157131	C8A	2.449759	1.839666	1.605713	1.406	1.569662	1.520547	1.863843	1.915042	2.079456	0.356485798	-0.244371374	0.754850707	0.079033531
ENSG00000168615	ADAM9	50.852979	53.668774	57.869685	37.505054	37.412742	37.21716	51.38793	53.344038	56.367198	5.34E-11	-0.386030716	0.305208943	0.07908777
ENSG00000136153	LMO7	9.286756	11.163105	10.188333	11.657793	11.272347	11.702322	10.441966	10.589641	10.208133	0.0000846	0.326608327	0.437483299	0.079175035
ENSG00000227500	SCAMP4	32.994551	33.552559	32.759764	25.727993	26.693405	27.98124	31.431746	30.727149	33.948452	0.063871864	-0.134990704	0.311771747	0.079193938
ENSG00000168159	RNF187	34.457195	33.009508	33.220501	36.167265	35.016291	36.895117	33.286676	32.791302	33.585926	0.0000139	0.251741002	0.27111238	0.079205074
NewGene_6177	--	2.793196	2.50667	2.067828	3.134699	2.073676	2.364549	3.295739	2.862385	2.553357	0.511526169	0.122132897	0.651574738	0.079261973
ENSG00000164074	ABHD18	2.423123	3.52051	2.187998	2.940403	2.588337	2.641459	2.530342	2.448589	2.49326	0.488433567	0.128753738	0.657184969	0.079282392
ENSG00000129245	FXR2	6.21235	6.449126	6.970925	5.490241	4.771654	5.559468	6.725751	6.522964	6.16818	0.195490969	-0.162827423	0.529897039	0.079307872
ENSG00000241978	AKAP2	8.101025	6.852912	6.855635	4.48626	4.222991	3.970848	7.17584	7.110224	7.369913	5.73E-09	-0.637518583	0.51635065	0.079336618
ENSG00000254996	NKHD1-EIF4EBI	4.415363	8.600349	10.927453	10.324429	11.352227	7.081361	7.892946	8.655619	6.986895	0.510124624	0.397757626	0.884487544	0.079448626
ENSG00000174996	KLC2	23.254604	22.454151	23.561381	19.61371	16.978124	19.223363	22.593024	20.993681	24.697911	0.022875655	-0.169725408	0.364877552	0.079465647
ENSG00000114473	IQCG	1.938521	1.605126	1.683158	1.04735	1.489373	1.226689	1.481795	2.112706	1.406106	0.221006422	-0.350008956	0.791577753	0.079535187
ENSG00000118900	UBN1	8.851534	8.842154	8.058211	8.42702	8.183149	8.14779	8.755204	7.596999	9.376041	0.457415645	0.069004569	0.413303176	0.079610759
ENSG00000143393	PI4KB	26.588024	23.37978	25.113297	24.597972	23.948211	23.26423	24.746378	24.36185	27.869024	0.338848339	0.079398603	0.353196607	0.079622241
ENSG00000205250	E2F4	30.050606	28.026424	28.2751	28.980122	27.904783	29.829852	26.360127	29.377474	29.731105	0.040634263	0.153623255	0.385928426	0.07970151
ENSG00000165417	GTF2A1	6.899075	8.633238	8.639786	7.938458	7.294304	7.306553	8.33604	6.587664	8.893927	0.724338191	0.04696526	0.542922764	0.079728697
NewGene_5388	--	1.948216	1.198868	0.504485	0.792129	1.746932	1.509729	1.840309	0.701806	1.33846	0.83183933	0.116138253	0.855312131	0.079782341
ENSG00000157916	RER1	53.985623	54.381038	53.829086	55.217375	52.798174	50.069487	54.569751	52.245937	55.010138	0.357996428	0.06043023	0.238008134	0.079787585
ENSG00000138777	PPA2	38.998477	40.329013	32.665995	36.050702	31.237364	31.205987	38.970599	36.200503	33.077425	0.706330466	0.037888453	0.329694475	0.079846316
ENSG00000175279	CENPS	5.558785	5.157994	5.353362	3.382077	3.581266	3.277432	5.368562	5.612334	4.914758	0.018964799	-0.494210775	0.722043552	0.07989165
ENSG00000198088	NUP62CL	1.204001	0.875055	0.673036	0.74551	0.675556	0.743648	0.997042	0.714921	1.14091	0.839583196	-0.10906016	0.856552173	0.07997037
ENSG00000171100	MTM1	4.464042	4.722836	4.467551	5.043133	4.635125	4.125814	4.469817	4.258947	4.814587	0.215528456	0.160654285	0.519911791	0.0800678
NewGene_6414	--	2.060569	1.707896	1.692984	1.013549	1.176264	0.888653	1.814512	1.908271	1.730897	0.006849749	-0.67906317	0.754850707	0.080107187
ENSG00000102595	UGGT2	9.775107	11.082425	11.148068	9.340646	8.858295	9.164209	11.223273	9.923315	11.672255	0.417766531	-0.078918352	0.409244964	0.080143723
ENSG00000141644	MBD1	7.451135	6.258455	6.782093	7.276803	6.114793	7.03173	7.501109	5.305262	7.971986	0.874684317	0.023136035	0.543994409	0.080298432
ENSG00000114686	MRPL3	83.600742	88.976975	89.871973	79.025208	71.330128	77.847251	88.756535	85.444437	84.96101	0.485505864	-0.05383157	0.281884109	0.080369825
ENSG00000205542	TMSB4X	510.776895	488.239163	447.240748	528.447172	584.916306	615.616479	471.932762	540.610719	439.386555	9.52E-09	0.404521597	0.450860706	0.080379573
ENSG00000172661	WASHC2C	13.642486	13.31806	13.151631	16.167745	16.245043	15.225762	13.149132	12.778761	13.927156	3.28E-10	0.396775931	0.304514679	0.080408739
ENSG00000096060	FKBP5	18.374934	19.762088	20.178709	16.063824	14.632484	15.240236	19.23832	18.462764	20.21084	0.007289788	-0.191958436	0.306326533	0.080523218
ENSG00000153339	TRAPPC8	8.841655	8.644673	9.026244	8.318144	7.578055	7.677119	8.378132	8.252554	9.472367	0.800631789	-0.024954917	0.349575135	0.080548853
ENSG00000148498	PARD3	22.787255	22.234773	21.738422	19.782778	20.464412	19.113305	21.253771	21.133404	24.221765	0.872063021	-0.015387045	0.292715364	0.080643279
ENSG00000139517	LNX2	8.885149	8.884171	9.39721	10.400248	9.675047	9.280768	9.51412	7.98653	9.479087	0.000846098	0.258830881	0.410867421	0.080645884
ENSG00000005471	ABCB4	3.212153	2.875129	2.57911	3.981704	3.91993	4.080283	2.896963	2.633198	2.931751	0.000000187	0.596666226	0.598144793	0.080802843
ENSG00000088451	TGDS	9.241673	8.625687	9.450545	9.432453	8.94674	9.038978	9.819272	8.798109	8.447086	0.198985092	0.152083845	0.545995308	0.080803599
ENSG00000169379	ARL13B	3.742954	4.410169	4.900344	4.302646	3.308101	4.072639	3.944755	3.599362	4.169552	0.361542021	0.16283177	0.621619456	0.080831771
ENSG00000068078	FGFR3	78.879295	76.505151	73.370912	62.684016	60.970074	60.377115	66.264871	58.351296	82.049166	0.001006052	-0.170368186	0.265679455	0.080857178
ENSG00000205403	CFI	41.711201	41.336844	42.63974	75.716033	74.079263	73.395093	42.012863	42.059166	39.31146	3.65E-76	0.980822195	0.346681125	0.080942794
ENSG00000103248	MTHFSD	5.362582	4.378065	4.461494	4.804255	3.956124	4.116449	4.867041	3.947474	4.866544	0.439070372	0.122659415	0.589606809	0.081026878
ENSG00000167703	SLC43A2	2.353559	2.252699	1.722413	1.215466	0.79774	1.062857	2.264953	1.737251	2.284443	2.52E-11	-0.993208691	0.634842509	0.0811306
ENSG00000004700	RECQL	14.247331	16.020004	16.321839	14.330361	15.916885	14.422982	15.201794	13.850151	16.793277	0.288654696	0.101083471	0.401276834	0.08118791

Gene ID	Gene name	Ctrl-1 FPKM	Ctrl-2 FPKM	Ctrl-3 FPKM	IFNγ-1 FPKM	IFNγ-2 FPKM	IFNγ-3 FPKM	Sora-1 FPKM	Sora-2 FPKM	Sora-3 FPKM	Ctrl vs IFNγ DESeq2 FDR	Ctrl vs IFNγ DESeq2 log2FC	Ctrl vs Sora DESeq2 FDR	Ctrl vs Sora DESeq2 log2FC
ENSG00000143294	PRCC	55.680511	50.613789	55.83817	56.130843	52.545996	53.762341	51.755356	49.14557	59.535997	0.021792236	0.153255376	0.315110839	0.081197209
ENSG00000075568	TMEM131	8.088523	8.027369	8.645949	8.083155	7.558739	7.544902	8.256492	7.241252	9.097295	0.580891043	0.051584681	0.394768611	0.081220621
ENSG00000109270	LAMTOR3	10.332882	10.979941	10.731462	9.683725	10.058847	9.967581	10.158661	10.320359	11.320349	0.704173012	0.038880937	0.358347864	0.081238299
ENSG00000171148	TADA3	61.249759	53.119281	49.491476	41.922054	44.680965	43.336041	52.362345	51.22854	57.353021	0.038669308	-0.168668879	0.352472866	0.08135925
ENSG00000173726	TOMM20	137.460617	141.716965	145.889114	129.097855	122.679123	124.601112	142.250488	135.717285	143.816833	0.667830333	-0.028316503	0.179011726	0.081365155
ENSG00000178996	SNX18	6.834458	7.67577	7.802599	5.25339	5.320761	5.38206	7.549006	6.83188	7.806755	0.0000755	-0.336947374	0.415386107	0.081416469
ENSG00000131931	THAP1	6.468369	6.243514	6.191829	5.493967	5.040572	5.429317	6.155024	6.30551	6.283942	0.5141941	-0.097706127	0.546293549	0.08153745
ENSG00000259332	ST20-MTHFS	5.098818	3.758799	3.715639	3.796897	5.455028	4.198665	4.126567	5.53976	2.870755	0.457730054	0.213457979	0.79856959	0.081655948
ENSG00000065328	MCM10	6.522773	6.831128	6.947217	6.04867	4.841124	5.62784	7.046683	6.899674	6.707734	0.179060508	-0.156437942	0.472988161	0.08170885
ENSG00000183684	ALYREF	99.662842	85.886055	84.405205	84.418526	74.265945	87.099159	80.786026	89.632523	97.711807	0.931966245	0.011212037	0.416260283	0.081717657
ENSG00000129219	PLD2	6.792554	6.588347	6.886971	5.572668	5.262073	5.826417	6.957136	6.045824	7.063406	0.232172781	-0.133473258	0.467799532	0.081722101
ENSG00000170540	ARL6IP1	167.38675	172.436056	157.200629	138.984604	133.020126	136.088289	168.075053	161.134068	164.607073	0.015062936	-0.136053838	0.226857597	0.081827607
ENSG00000175203	DCTN2	72.356484	66.506196	61.870941	53.601249	58.335025	57.992264	67.014273	66.041561	72.340597	0.059780243	-0.12761626	0.224154276	0.081946755
ENSG00000122696	SLC25A51	3.62742	3.632442	3.270978	3.43132	3.807205	3.254807	3.204084	3.185702	3.638531	0.447812926	0.174426186	0.713638314	0.081971633
ENSG00000111445	RFC5	24.003526	23.795351	23.991613	21.015079	20.563476	21.03347	24.815705	21.367338	23.884871	0.818462962	-0.023253127	0.366241204	0.082033547
ENSG00000127540	UQCR11	62.72368	62.650087	63.702503	56.115489	56.614623	60.572246	63.277499	73.779397	48.142347	0.900752056	0.012966471	0.767631962	0.082276074
ENSG00000092094	OSGEP	10.519884	11.999415	13.059038	10.670869	9.959238	9.630638	14.413986	11.35394	9.913094	0.543981802	-0.080974557	0.592619657	0.082343189
ENSG00000106078	COBL	5.386909	5.501556	5.052402	5.622383	5.701785	5.475252	5.389672	4.831535	5.583789	0.056156023	0.177993472	0.436078924	0.082560303
ENSG00000170836	PPM1D	5.111491	5.151714	5.998581	4.866396	5.360537	4.766584	5.386593	4.62076	5.659559	0.90417337	-0.019941986	0.495562677	0.082598913
ENSG00000096433	ITPR3	33.346397	30.946297	31.271877	21.218015	19.255552	20.381199	29.978848	29.530455	36.076767	4.27E-22	-0.507063831	0.301155943	0.083069641
ENSG00000085644	ZNF213	2.145572	1.801759	1.998874	1.892082	2.12191	2.210034	2.192513	1.65805	1.823879	0.662495039	0.099556426	0.680632614	0.083162657
ENSG00000165609	NUDT5	25.408862	25.017978	28.617751	26.533324	26.07618	24.942407	26.975744	25.696146	26.109093	0.213657312	0.106387101	0.348603201	0.083383509
ENSG00000161847	RAVER1	32.258986	31.460796	30.592865	29.786924	28.31468	31.810649	30.851351	28.616655	35.675621	0.37634884	0.070397851	0.291534463	0.083383547
ENSG00000096080	MRPS18A	47.772122	43.653949	40.174934	42.445709	40.162364	38.894783	44.179182	44.191698	41.872737	0.753062749	0.037647819	0.425112214	0.083478708
NewGene_759	--	2.425077	2.29021	2.15604	2.264518	2.060777	1.937288	2.366383	2.461668	2.410972	0.44371264	-0.094147411	0.455203964	0.083679453
ENSG00000185019	UBOX5	3.22441	3.057359	3.194666	3.208758	3.062149	3.043126	3.289809	2.735776	3.434364	0.383505194	0.116204564	0.545027178	0.083809
ENSG00000164070	HSPA4L	1.360003	1.585812	1.598464	0.827688	0.684441	0.718579	1.692623	1.414151	1.437783	1.47E-12	-0.880200945	0.563596822	0.083824292
ENSG00000085831	TTC39A	5.059574	5.00235	4.145385	3.862494	3.298671	3.794983	4.839413	4.34872	5.91002	0.009544847	-0.353392859	0.596932869	0.083981645
ENSG00000068308	OTUD5	15.342591	13.665395	14.046113	14.276242	15.072642	14.391564	14.163958	13.600245	14.970634	0.15975518	0.133480021	0.393330188	0.08432129
ENSG00000143850	PLEKHA6	11.492278	10.324603	9.551206	10.836942	10.115538	10.112051	10.791708	10.836519	10.981055	0.484316923	0.074060953	0.459475283	0.084377573
ENSG00000111229	ARPC3	227.753289	222.468301	233.511141	239.005083	245.956331	239.728023	230.160113	231.693647	217.395907	0.0000105	0.229082339	0.265314454	0.084389496
ENSG00000198300	PEG3	5.832277	4.967163	4.802843	6.194654	6.984504	6.219052	5.050311	4.334716	5.133389	0.021079369	0.557280429	0.590636108	0.084447242
ENSG00000075711	DLG1	21.367988	21.949571	21.003408	17.653069	16.136402	16.011571	21.693483	22.561038	22.326699	0.001241048	-0.20981107	0.253312417	0.084484019
ENSG00000116771	AGMAT	39.49081	44.236599	38.945606	48.133152	48.940048	50.713863	40.687141	39.311436	41.991005	1.09E-10	0.416726737	0.290772263	0.084556754
ENSG00000142856	ITGB3BP	6.017512	6.981257	6.502937	4.943144	4.390255	5.281251	6.213546	7.033938	7.065517	0.123481438	-0.30005351	0.666277741	0.084597225
ENSG00000177542	SLC25A22	9.250227	8.846772	9.428176	19.654517	16.652867	17.922303	8.891738	9.206228	9.184266	1.07E-47	1.117732823	0.428961707	0.084631052
ENSG00000137842	TMEM62	7.503091	8.107078	7.106903	8.434589	8.149644	7.947543	7.507951	7.985477	7.464415	0.012863106	0.255242057	0.487625021	0.084702851
ENSG00000119392	GLE1	10.356377	10.941642	10.691699	10.076941	10.576329	10.663935	10.866772	10.355265	10.49343	0.217676077	0.115176871	0.371285987	0.084712926
ENSG00000163755	HPS3	20.972697	24.413021	23.920762	31.338347	29.183894	30.425174	21.956931	21.990407	24.174358	2.04E-18	0.542938283	0.304179099	0.084734175
ENSG00000185238	PRMT3	21.908683	26.2459	27.931991	23.136319	21.116645	22.286708	25.905362	24.133961	25.537057	0.701865947	-0.046401236	0.415551332	0.084740064
ENSG00000173418	NAA20	32.699918	35.276762	33.877494	31.30886	30.011501	32.077692	35.181482	31.304259	33.470377	0.54328814	0.058210236	0.35844155	0.084744405
ENSG00000186205	1-Mar	14.785267	15.144613	17.045548	22.876073	24.90327	24.237467	25.029057	21.158686	31.082749	0.00000543	0.303081661	0.278008993	0.084754018
ENSG00000166665	C17orf58	7.880634	8.43638	8.50616	6.707444	7.14156	6.800233	8.731006	7.625566	8.815461	0.341824435	-0.144211568	0.563910927	0.084761832
ENSG00000172638	EFEMP2	23.498792	20.997496	19.863963	12.480737	13.786092	13.959859	21.215326	21.821702	21.894777	6.61E-09	-0.532154901	0.397224763	0.085180355
ENSG00000143337	TOR1AIP1	18.733623	14.158824	19.515424	14.961809	10.831225	14.4889	14.661404	14.454047	16.140783	0.403230942	-0.068093494	0.277584245	0.08526951
ENSG00000130940	CASZ1	1.587567	1.407106	1.394408	1.590689	1.455128	1.614608	1.546651	1.3328	1.449079	0.061417753	0.258276816	0.593906256	0.085280978
ENSG00000105197	TIMM50	45.479385	50.493274	47.985335	41.493205	37.209938	47.720827	49.806994	47.784778	51.708438	0.363224226	-0.079023322	0.285568309	0.085299693
ENSG00000149930	TAOK2	8.8851	8.709052	8.475684	9.050117	8.924892	9.688207	8.4656	8.126791	9.023008	0.001638986	0.239087472	0.33351103	0.085328172
ENSG00000016864	GLT8D1	12.785735	12.666818	12.570289	10.160902	10.761395	9.904679	12.84595	11.693445	13.270232	0.175952887	-0.153545819	0.451882609	0.085339262
ENSG00000188191	PRKAR1B	19.388201	19.777814	18.893332	20.298731	19.71124	19.998356	19.156986	18.418254	20.025967	0.003687213	0.207257329	0.304548808	0.085482751
ENSG00000124207	CSE1L	84.146978	89.784496	87.59646	71.680439	70.424098	70.769268	85.836361	83.392832	90.979077	0.003884231	-0.149782121	0.167564792	0.085516211
ENSG00000150093	ITGB1	202.617843	207.331731	216.509136	155.377318	158.546629	152.054875	202.188357	204.674295	217.389823	2.04E-08	-0.277564209	0.169253393	0.085557432
ENSG00000151135	TMEM263	23.889213	24.213274	24.665273	30.417369	31.762307	31.497107	25.479021	22.520699	25.544139	3.14E-16	0.474562153	0.28255967	0.085711582
ENSG00000130813	C19orf66	7.317883	7.541797	7.783461	31.476341	28.031577	28.241702	7.287875	6.913311	7.830722	9.21E-163	2.080823575	0.502685869	0.085732631
ENSG00000169957	ZNF768	30.146677	28.314287	26.94912	25.658336	27.036498	27.648834	27.762378	27.648001	29.25356	0.475604954	0.067267656	0.30444144	0.085750178
ENSG00000125450	NUP85	35.447763	31.363829	33.862275	26.616302	25.505869	25.720381	30.905943	32.534402	36.413394	0.004130565	-0.204447398	0.348265894	0.085798426
ENSG00000002834	LASP1	74.074465	72.791634	69.429023	76.354279	75.455282	80.098774	70.641831	71.867164	73.672384	0.00000415	0.239057322	0.191300089	0.085815963
ENSG00000214046	SMIM7	41.044722	40.152578	40.193477	35.295039	33.24598	35.259469	40.8386	44.087647	45.036333	0.771319307	0.027453262	0.307128938	0.085852583
ENSG00000116288	PARK7	128.192485	129.063735	124.22137	87.780815	106.281633	106.631108	126.813203	122.123901	126.460363	0.058040605	-0.140274067	0.224361001	0.085852888
ENSG00000070610	GBA2	24.7168	21.849611	23.501275	20.762287	18.524784	18.799123	21.728217	21.741549	24.698928	0.037799335	-0.162899335	0.327359177	0.08586683
ENSG00000090060	PAPOLA	56.355705	58.428323	63.818504	56.461467	55.457017	54.805438	58.566857	56.871985	66.225117	0.483531536	0.050887306	0.257225905	0.085889876
ENSG00000149499	EML3	9.423954	8.31291	9.295717	8.415183	8.066948	8.258268	9.031651	8.191581	9.519911	0.902295008	0.017050617	0.4275423	0.085904246
ENSG00000128833	MYO5C	10.282246	11.558917	11.199883	9.427435	9.130162	8.942238	11.264563	10.194973	12.390707	0.081839343	-0.132084271	0.346999986	0.085992292
ENSG00000092140	G2E3	6.158517	6.547043	6.637013	5.093653	5.342457	5.015255	8.224571	5.63494	6.626853	0.225874858	-0.159259991	0.512393035	0.086042098
ENSG00000198786	MT-ND5	899.102783	810.36261	763.725037	817.630737	771.173706	766.936707	798.336792	791.244019	874.107605	0.30889227	0.076608463	0.261957119	0.086083419
ENSG00000166888	STAT6	2.105438	1.927917	1.755532	2.516602	2.55901	2.157769	2.040485	2.0771	1.645499	0.002296729	0.450709507	0.677094738	0.086149097
ENSG00000148925	BTBD10	10.95918	11.482672	10.313734	10.441491	9.339294	9.973973	10.886063	11.236019	11.526756	0.797151742	-0.033697864	0.432224524	0.08625182
ENSG00000143437	ARNT	14.59752	13.532314	14.217974	13.442876	12.862752	12.405851	13.819941	12.908917	14.561269	0.677702704	0.03710914	0.286492047	0.086289671
ENSG00000215717	TMEM167B	4.446321	5.123586	5.139637	4.121273	4.703727	4.708371	4.82968	4.891495	4.9147	0.866443244	0.02875959	0.495097213	0.086305026
ENSG00000155115	GTF3C6	76.39035	78.489235	82.364876	77.067154	75.618713	81.042496	78.721962	81.05661	76.105225	0.083846487	0.126594534	0.317632242	0.086404367
ENSG00000166377	ATP9B	5.675029	5.460912	5.359851	5.200541	4.328498	4.776331	5.628965	5.049671	6.405803	0.50061725	-0.085973566	0.456592804	0.086419122
ENSG00000214026	MRPL23	84.273961	88.066702	79.049827	85.96294	84.631275	86.942412	89.084823	86.250693	74.962532	0.0258999	0.165519247	0.404859206	0.086476524
ENSG00000197948	FCHSD1	1.534701	1.247601	1.354154	1.164537	1.362452	1.171571	1.555308	1.472753	1.582833	0.904268201	-0.033704275	0.685670634	0.086564228
ENSG00000159692	CTBP1	87.212438	85.913525	85.140936	77.623645	79.014036	81.530963	79.575277	78.99947	90.549564	0.903225199	0.010775387	0.231997698	0.086564293

Gene ID	Gene name	Ctrl-1 FPKM	Ctrl-2 FPKM	Ctrl-3 FPKM	IFNγ-1 FPKM	IFNγ-2 FPKM	IFNγ-3 FPKM	Sora-1 FPKM	Sora-2 FPKM	Sora-3 FPKM	Ctrl vs IFNγ DESeq2 FDR	Ctrl vs IFNγ DESeq2 log2FC	Ctrl vs Sora DESeq2 FDR	Ctrl vs Sora DESeq2 log2FC
ENSG00000164323	CFAP97	15.238277	16.870718	17.105166	15.362094	15.713826	15.024323	16.572775	15.380536	16.813752	0.511053967	0.06000677	0.298919574	0.08671524
ENSG00000123358	NR4A1	3.529893	3.047477	4.100578	0.437408	0.659237	1.092105	2.808392	3.424849	4.175795	1.68E-18	-1.912854333	0.672382189	0.086760024
ENSG00000058091	CDK14	8.600487	10.135728	10.331424	7.961198	8.071004	9.085681	9.96042	9.048143	9.988134	0.696598513	-0.048674208	0.380526165	0.086782441
ENSG00000104613	INTS10	21.266079	21.176496	22.64209	20.890357	22.313586	20.957156	21.629315	20.67587	23.012449	0.122571806	0.124340484	0.287204486	0.086836914
ENSG00000165304	MELK	25.878532	28.487696	27.339398	21.826324	20.310941	20.375153	27.627114	26.698805	27.327329	0.001702577	-0.228111405	0.287871241	0.086885659
ENSG00000139132	FGD4	3.952825	3.960712	6.230095	2.5154	2.64842	2.719183	4.793089	3.520598	3.651939	0.857026623	-0.121869479	0.877861219	0.087006408
ENSG00000123191	ATP7B	5.348832	5.326935	4.635596	4.868239	5.079814	4.988181	4.837902	5.487327	5.238261	0.231420981	0.122889365	0.409059123	0.087019733
ENSG00000053254	FOXN3	12.745868	12.15207	11.673289	13.071392	13.57061	13.478206	12.481313	11.868016	13.51998	0.00000994	0.272394097	0.246268411	0.087095668
ENSG00000169692	AGPAT2	36.393027	33.325622	29.825585	43.109268	39.965302	46.246649	32.95792	33.817601	32.266959	7.41E-10	0.519833918	0.408802083	0.087196565
ENSG00000139726	DENR	45.130745	46.333115	50.669689	42.277626	42.941845	41.038883	47.929886	45.778458	47.862026	0.800575578	-0.023333688	0.253109567	0.087215514
ENSG00000196367	TRRAP	12.770818	12.464986	11.478016	11.952027	11.133976	11.450606	12.711532	10.624114	13.245604	0.403095928	0.067329106	0.342397045	0.087244786
ENSG00000163156	SCNM1	14.546523	12.223747	14.016574	12.513121	11.600564	10.814815	11.086169	13.174494	12.704504	0.912690603	0.017127528	0.462189332	0.087444242
ENSG00000088826	SMOX	9.510938	8.558597	8.697387	5.13548	4.71695	5.385637	9.684784	8.150706	8.774004	8.05E-10	-0.648698707	0.477522147	0.087505196
ENSG00000099204	ABLIM1	8.188522	9.017325	9.279265	6.998084	6.105673	7.457742	9.918213	8.211342	9.386251	0.000117896	-0.301147812	0.315283312	0.087527424
ENSG00000165861	ZFYVE1	2.530763	2.86859	2.267658	2.647059	2.537834	2.623612	2.553769	2.411345	2.705811	0.251467372	0.174504037	0.574014926	0.087573389
ENSG00000131188	PRR7	2.440008	2.769969	2.366563	2.074979	2.165988	1.969703	2.308677	2.448558	2.661645	0.730547585	-0.106167869	0.736913979	0.087616878
ENSG00000136908	DPM2	19.852959	16.754959	17.66018	14.061458	13.916899	14.564674	17.254479	18.359773	18.309845	0.053065403	-0.206901884	0.444099432	0.087692857
ENSG00000113312	TTC1	35.100048	35.758457	36.80639	33.025843	31.167297	33.035756	35.843215	36.274736	34.963573	0.994315455	0.000891582	0.314494163	0.087789672
ENSG00000116035	VAX2	2.131906	1.905042	2.011359	2.084649	2.439179	2.257351	1.503851	2.149217	2.485147	0.200370099	0.341955604	0.7832038	0.087852931
ENSG00000077150	NFKB2	7.79156	7.589553	7.386492	3.026363	3.26286	2.701784	7.225795	6.39588	8.870255	7.02E-29	-1.168854719	0.469778051	0.087893191
ENSG00000181315	ZNF322	9.376745	9.971654	9.600341	9.922721	9.539963	9.516069	9.977479	8.986066	10.105832	0.057087677	0.14727499	0.308064136	0.087895163
ENSG00000148835	TAF5	4.556012	4.526688	4.365631	3.732556	3.690005	3.356492	4.446232	4.251982	4.717108	0.196280012	-0.172650768	0.492766381	0.087904511
ENSG00000136758	YME1L1	37.858313	40.6488	43.063344	40.45664	39.519453	39.570209	39.212614	40.165145	41.364892	0.100860673	0.114212041	0.255775549	0.08793843
ENSG00000175606	TMEM70	14.291177	14.294975	15.104219	14.683264	13.512953	13.744925	17.563257	13.820081	15.078366	0.115634506	0.156018534	0.439605094	0.087960956
ENSG00000146425	DYNLT1	36.60302	32.592171	30.410139	37.839157	39.857483	39.698303	32.303291	35.176014	31.677855	0.0000864	0.384043009	0.491344232	0.087978934
ENSG00000075413	MARK3	18.835234	19.289663	18.748107	16.184799	17.168655	16.086859	21.426451	19.608826	20.036735	0.67091873	-0.040919938	0.275407356	0.088011845
ENSG00000131149	GSE1	18.019201	17.772202	18.243881	17.84869	17.04884	19.545302	20.600648	17.527629	20.630826	0.51291119	0.046892461	0.21223328	0.088057218
ENSG00000166199	ALKBH3	23.399845	23.496072	22.558651	23.294833	24.849201	24.846412	23.338593	21.075184	24.487952	0.012078387	0.221541919	0.354560354	0.088086059
ENSG00000169718	DUS1L	35.675071	37.768251	35.146369	33.907733	30.116953	33.349584	35.887214	34.003725	42.629515	0.914820238	0.012266514	0.328064766	0.088105639
ENSG00000105514	RAB3D	6.509201	7.230212	6.160001	8.359946	7.923052	8.357996	7.044456	6.154165	6.647145	9.46E-08	0.455972991	0.433657889	0.088204407
ENSG00000108433	GOSR2	29.111611	29.814853	29.798812	30.705747	30.491059	29.949205	30.372645	29.244042	29.45885	0.011763012	0.156664786	0.216547523	0.088214844
ENSG00000139405	RITA1	10.724459	10.328115	10.431481	9.836142	8.93438	9.40712	9.63571	10.194502	11.45329	0.953110507	-0.009239213	0.453857272	0.08823787
ENSG00000198700	IPO9	23.808588	22.738655	24.267326	21.386431	21.13546	20.714235	22.956669	23.349331	24.290531	0.833960653	-0.016136419	0.176375337	0.088253286
ENSG00000050165	DKK3	2.703094	2.424219	2.194161	3.336462	3.41568	2.986268	2.52114	2.441859	2.328036	0.000470858	0.549941769	0.655715594	0.088275581
ENSG00000103356	EARS2	15.394937	14.37278	13.828717	11.729785	11.496897	11.032477	15.170968	13.960515	14.586432	0.009013586	-0.201409641	0.305606347	0.088293838
ENSG00000143977	SNRPG	65.319825	67.782326	72.925686	67.352714	78.573793	75.41822	74.441827	78.876709	51.229191	0.004923896	0.253094974	0.761351475	0.088297287
ENSG00000069248	NUP133	18.357479	19.29624	18.969366	15.853396	15.381528	15.386246	18.164961	18.274744	20.037571	0.036132306	-0.133068459	0.222838254	0.088317259
NewGene 6700	--	0.600577	0.913626	0.811745	0.586847	0.572304	1.073148	0.814228	0.772503	0.767317	0.983443072	0.013345545	0.841588554	0.088335296
ENSG00000177981	ASB8	8.297294	9.582299	8.040632	9.316513	9.37272	9.673061	8.346312	8.255425	8.646907	0.01575502	0.265524191	0.479534793	0.088362977
ENSG00000116962	NID1	110.308632	103.124087	107.740944	89.850983	83.599007	88.254028	104.543304	99.860115	115.976791	0.005858095	-0.147322509	0.18208497	0.088391806
ENSG00000125912	NCLN	45.627328	39.225529	40.345822	37.867818	33.524277	38.65718	39.4765	39.889048	47.003054	0.615563554	-0.04967803	0.33652925	0.088400586
ENSG00000141337	ARSG	1.579194	1.781878	1.650586	1.596187	1.582311	1.779994	1.865985	1.629572	1.806845	0.522975328	-0.187050257	0.628074028	0.088428365
ENSG00000100347	SAMM50	37.544876	35.831356	35.792404	39.443699	38.26635	41.394596	38.47646	34.18219	36.233116	0.0000487	0.273116289	0.293984625	0.08848952
ENSG00000079974	RABL2B	5.738585	5.813311	6.056347	5.234445	3.878163	4.295543	6.485275	5.376092	6.342097	0.101087535	-0.256034364	0.527640232	0.088508281
ENSG00000218739	CEBPZOS	5.171729	5.895338	5.739888	5.591795	5.047869	5.235419	6.136495	5.367939	5.458135	0.923581625	0.017713626	0.528642512	0.0885451
NewGene 53	--	0.610591	0.624295	0.62453	0.641557	0.830084	0.56071	0.695665	0.573782	0.581633	0.49802853	0.274185738	0.820817558	0.088550695
ENSG00000198843	SELENOT	24.987638	25.40684	25.8363	22.939511	24.218647	23.58794	24.310271	26.087693	26.838725	0.838525194	0.019874378	0.253947915	0.088593719
ENSG00000120616	EPC1	10.73191	10.5952	9.481334	7.556429	7.877309	8.021816	10.245578	9.346026	10.215943	0.146384776	-0.136505737	0.350145314	0.088601605
ENSG00000133627	ACTR3B	5.853852	5.883751	5.861619	4.058443	4.017757	4.401812	5.904152	5.820826	6.484964	0.006884568	-0.351605174	0.517542981	0.08860474
ENSG00000132676	DAP3	102.494608	100.551914	106.326917	86.398003	85.030227	83.250819	102.688509	100.433951	99.680026	0.033440161	-0.120570647	0.201749854	0.088665787
ENSG00000088970	KIZ	3.773762	3.843355	3.14152	3.965393	3.400762	3.834393	3.607718	3.921498	3.805606	0.32122472	0.183242482	0.624244921	0.088710747
NewGene 3443	--	10.607127	9.783018	10.164248	8.344233	8.831995	7.798514	11.060811	12.173487	10.212785	0.837909154	-0.034768441	0.517606883	0.088740434
ENSG00000198001	IRAK4	3.805865	3.834315	4.049756	3.910732	4.487465	4.128163	5.722009	5.235569	4.515905	0.045054653	0.232258909	0.504512142	0.088742199
ENSG00000167005	NUDT21	25.821941	29.227566	30.853031	26.241549	25.81217	25.031445	29.642443	26.656031	29.489105	0.938840046	-0.008710805	0.308282126	0.088802293
ENSG00000179889	PDXDC1	44.755452	41.565053	45.779295	49.503397	45.756693	42.857451	48.254501	41.610092	47.040381	0.000244019	0.189530771	0.174222692	0.088914301
ENSG00000120647	CCDC77	9.265555	9.577899	10.308892	8.023272	5.899383	7.152138	9.640692	9.563523	10.044646	0.007094154	-0.325145513	0.429626665	0.088986407
ENSG00000145979	TBC1D7	28.189013	34.683356	31.729096	20.096843	18.17172	22.866302	32.012513	32.814646	32.826817	0.000000388	-0.458002558	0.366803997	0.08911605
ENSG00000102763	VWA8	5.285817	5.765971	5.995445	4.830233	4.419742	4.263154	5.679421	5.367511	6.040424	0.080681904	-0.162475773	0.354560354	0.089116176
ENSG00000108830	RND2	0.412804	0.504319	0.277399	0.468041	0.377957	0.364345	0.50024	0.345856	0.351905	0.716320389	0.16396093	0.831291057	0.089129778
ENSG00000104969	SGTA	73.920238	70.50869	67.077405	64.250212	62.252904	63.790233	67.870689	65.989684	77.534631	0.948751648	-0.006007154	0.242382323	0.089243329
ENSG00000157827	FMNL2	27.032713	27.564045	33.176095	21.932298	23.41186	21.757513	27.805636	27.663881	32.166447	0.004102191	-0.23898315	0.356290338	0.089246422
ENSG00000105619	TFPT	11.470963	9.591068	7.333138	10.504247	9.040834	8.649997	9.064833	9.685913	10.317037	0.495996801	0.142388835	0.644854998	0.089302238
ENSG00000104611	SH2D4A	20.367632	19.87281	20.286798	16.987839	17.091472	20.653271	20.418347	18.899575	20.742991	0.495722162	-0.06093722	0.273570619	0.089475495
ENSG00000126091	ST3GAL3	6.449088	5.869427	5.293518	4.355783	4.544362	4.073551	5.832149	6.492053	5.205303	0.052413772	-0.271794082	0.575550036	0.089481481
ENSG00000005194	CIAPIN1	23.764667	27.630962	26.175772	23.285696	19.579175	24.008657	27.261835	27.424663	28.060082	0.358260968	-0.085546295	0.330037356	0.08957116
ENSG00000159720	ATP6V0D1	35.781103	36.861471	35.387465	35.224735	36.56026	35.745035	34.28921	38.085743	38.742772	0.171670948	0.108342297	0.326403917	0.089667457
ENSG00000174231	PRPF8	51.362332	48.706217	47.559248	51.197961	45.079559	47.704736	49.177505	45.779199	53.068794	0.074479633	0.112883948	0.173147736	0.089892575
ENSG00000254685	FPGT	2.051042	2.626708	2.348154	2.381602	2.166952	2.359757	2.41461	2.274332	2.386289	0.605009351	0.106947181	0.626594138	0.089910664
ENSG00000165195	PIGA	3.744006	4.192082	3.998998	4.72031	4.855492	3.785945	4.494687	3.897708	4.12457	0.134927826	0.202187495	0.517799675	0.089972462
ENSG00000198833	UBE2J1	15.64692	16.709126	16.755686	19.198963	19.371902	19.534492	16.37899	15.851877	16.789137	1.49E-10	0.390403726	0.243710671	0.090010653
ENSG00000121775	TMEM39B	6.173352	5.602769	6.125844	5.018784	5.212039	5.366926	5.929264	5.513424	6.31401	0.832431415	-0.040753066	0.549864385	0.090013167
ENSG00000172985	SH3RF3	4.440176	4.94448	5.143664	2.006612	1.915211	1.997508	4.507387	4.403736	5.620587	4.64E-32	-1.14586296	0.459552213	0.090050824
ENSG00000204228	HSD17B8	14.171731	13.847974	10.243091	10.774645	9.302222	9.892075	13.65251	12.927999	11.559138	0.23019049	-0.206581117	0.610989471	0.09008229
ENSG00000006757	PNPLA4	3.60103	1.954043	2.230849	2.391676	2.751283	2.170653	1.898976	3.393666	4.116634	0.215790025	0.249133231	0.647615958	0.090134766

Gene ID	Gene name	Ctrl-1 FPKM	Ctrl-2 FPKM	Ctrl-3 FPKM	IFNγ-1 FPKM	IFNγ-2 FPKM	IFNγ-3 FPKM	Sora-1 FPKM	Sora-2 FPKM	Sora-3 FPKM	Ctrl vs IFNγ DESeq2 FDR	Ctrl vs IFNγ DESeq2 log2FC	Ctrl vs Sora DESeq2 FDR	Ctrl vs Sora DESeq2 log2FC
ENSG00000215375	MYL5	2.851574	2.421113	3.458814	3.759219	3.084244	3.716841	2.769777	2.68807	2.964705	0.03245285	0.386286324	0.676029236	0.090219398
ENSG00000255152	MSH5-SAPCD1	1.843427	2.897931	2.707767	3.196565	2.224235	2.200349	3.054465	1.975122	3.023445	0.285359785	0.295349332	0.737244881	0.090294691
ENSG00000161981	SNRNP25	15.613346	14.3068	16.487219	12.454096	15.377734	13.986007	17.96419	18.956481	15.674935	0.2580715	-0.139375415	0.443289663	0.090302498
ENSG00000110057	UNC93B1	20.021794	17.278794	13.927212	21.582022	20.694487	22.703514	16.054174	17.373749	18.093309	0.0000135	0.478305493	0.506728115	0.09381428
ENSG00000032444	PNPLA6	12.90203	11.04008	11.354749	11.200501	12.462106	11.572103	9.808044	10.486464	14.585495	0.306595688	0.10651764	0.499458112	0.090402138
ENSG00000197223	C1D	19.060459	16.574758	18.52334	14.945873	13.614952	13.596175	16.859489	15.035101	15.637693	0.905937123	-0.017478748	0.426492581	0.090535005
ENSG00000124333	VAMP7	11.16384	11.900118	12.479464	11.508405	10.359896	10.872042	12.170329	11.487033	11.883541	0.822655	0.028449329	0.370468052	0.09062327
ENSG00000213390	ARHGAP19	5.219569	5.531695	5.68487	4.468885	3.588356	4.192968	5.800117	4.999774	5.67114	0.007516835	-0.278582623	0.381573199	0.090812966
ENSG00000076053	RBM7	7.444807	6.494774	8.776992	9.350928	9.65541	8.299651	7.989975	6.014719	8.522883	0.00000478	0.413267985	0.454921641	0.090920039
ENSG00000178809	TRIM73	0.934881	0.837017	1.136804	0.657282	1.048842	1.022126	0.89034	0.685813	1.389279	0.902295008	0.066434545	0.837641334	0.091099989
ENSG00000187091	PLCD1	0.936793	0.747167	0.623861	0.852087	0.895255	0.671576	0.787178	0.802963	0.726009	0.546187168	0.21475306	0.784872396	0.091230576
ENSG00000213694	S1PR3	1.333336	1.149267	1.216645	1.002876	1.186871	1.035557	1.196058	1.135974	1.317221	0.965830514	0.013094425	0.652560719	0.091461214
NewGene_314	--	1.265871	1.017458	0.992592	0.517332	0.594342	0.695443	0.932088	1.281169	1.019927	0.01780734	-0.675106808	0.763100023	0.091535741
ENSG00000188488	SERPINA5	195.678884	166.721072	159.042798	126.52028	147.8179	131.172804	167.89944	178.110148	178.239045	0.013095199	-0.22038037	0.309522988	0.091587484
ENSG00000137642	SORL1	4.429315	4.380274	4.056005	2.450834	2.224372	2.480937	4.364987	4.136924	4.412938	4.38E-19	-0.698380967	0.294725359	0.091656797
ENSG00000125304	TM9SF2	100.826929	101.79887	103.090417	108.839794	110.597364	106.45398	101.231525	99.723767	104.43535	0.000000426	0.240288252	0.115784893	0.09165722
ENSG00000131508	UBE2D2	18.99007	19.006611	20.515491	18.893142	18.165554	19.742585	19.697237	18.842877	19.320084	0.178107663	0.113019418	0.297792237	0.091719813
ENSG00000257949	TEN1	21.567902	19.953407	19.451931	16.138934	15.563716	15.979874	19.954898	20.373001	20.632278	0.057171382	-0.213042588	0.432125571	0.091743594
ENSG00000186815	TPCN1	4.6103	4.106	4.229257	3.479255	3.057671	3.167577	4.535842	4.099652	4.62188	0.004453789	-0.299289199	0.415551332	0.091887548
ENSG00000103061	SLC7A6OS	5.477093	6.489493	3.385177	4.527446	3.818438	5.461112	4.64061	4.542191	5.418023	0.999202724	0.000844737	0.800945568	0.092006141
ENSG00000170873	MTSS1	5.834647	5.103598	5.31425	4.848344	4.827147	5.029136	5.57918	5.09901	5.446998	0.996976826	0.000754	0.410218683	0.092085107
ENSG00000151461	UPF2	6.535727	7.583842	7.273422	6.548676	7.012107	6.24358	6.716872	7.106903	7.568141	0.742322054	0.041116319	0.366163855	0.09216998
ENSG00000138071	ACTR2	121.866586	128.044357	125.606188	129.423587	120.811852	124.029452	125.720205	120.032448	130.081194	0.00435474	0.141409576	0.111390169	0.092201148
ENSG00000115827	DCAF17	4.641764	5.029279	5.261463	4.872661	4.945713	5.260998	4.980227	4.554673	5.52082	0.143938455	0.150839181	0.403569734	0.09225206
ENSG00000171621	SPSB1	0.971297	0.782091	0.716336	1.957172	1.409803	1.770789	0.808753	0.912356	1.146752	2.28E-09	1.24539757	0.767631962	0.092271685
ENSG00000152242	C18orf25	3.068453	2.971666	3.137444	3.153936	3.022218	3.18712	3.490001	2.648083	3.34928	0.116940525	0.186150578	0.492723913	0.092280017
ENSG00000170271	FAXDC2	4.088896	3.352182	3.456574	1.805749	1.918569	1.760584	3.441066	3.47744	3.917239	8.98E-10	-0.933275808	0.571684377	0.092379172
ENSG00000116977	LGALS8	15.899158	16.837844	15.690018	23.407117	22.982132	21.118081	16.643327	15.621473	14.60651	1.11E-32	0.67673999	0.324530699	0.09254459
ENSG00000278259	MYO19	30.751298	29.428221	31.793068	24.79486	24.05521	24.466775	27.951214	29.059081	34.467369	0.00731691	-0.16712034	0.246787734	0.092649799
ENSG00000122786	CALD1	47.527149	54.059484	54.654376	51.77646	47.722464	48.452022	55.012002	53.533452	58.888024	0.310173129	0.078050637	0.251144884	0.092690745
ENSG00000102096	PIM2	2.734443	3.860016	3.304259	4.737451	4.231371	3.820527	2.872399	3.568562	3.341018	0.002907187	0.515574733	0.673589648	0.092705672
ENSG00000101558	VAPA	25.710529	22.908316	25.295596	23.860058	23.475358	21.787272	21.198041	20.549825	23.999163	0.856107824	0.016469709	0.18490615	0.092754818
ENSG00000089685	BIRC5	34.163141	35.651681	33.890113	23.696956	21.969924	23.176736	34.896322	35.129062	35.921308	9.47E-14	-0.444213607	0.206188275	0.092776945
ENSG00000089737	DDX24	31.104876	32.174056	26.373113	27.520635	26.126218	23.549657	27.454962	26.334346	32.349279	0.409313246	-0.0568994	0.180530113	0.09285862
ENSG00000121931	LRIF1	12.901711	15.298076	15.930373	15.278063	15.7423	15.520688	13.768057	13.734147	15.244219	0.039421738	0.199212927	0.383600526	0.092906091
ENSG00000197594	ENPP1	8.531448	9.776553	11.630151	10.745627	10.614648	10.821835	9.53846	8.46164	10.105507	0.0000196	0.306615924	0.303518307	0.092978265
ENSG00000067900	ROCK1	6.064419	7.054356	7.019711	6.519788	6.211566	6.435886	6.752186	6.169018	7.226016	0.426394468	0.075221623	0.317733119	0.093003438
ENSG00000239382	ALKBH6	14.394766	11.786248	14.593148	13.122917	12.4074	13.269558	12.925041	14.098519	14.213388	0.76878188	0.053612998	0.541789238	0.093035124
ENSG00000101624	CEP76	3.694672	3.776456	3.349358	2.9407	2.833942	2.985689	3.8666	3.31274	3.805516	0.288282137	-0.167607149	0.544605239	0.093071311
ENSG00000185950	IRS2	13.167392	11.791919	12.733547	8.341573	9.512539	9.283848	12.034472	10.776661	15.033041	0.0000199	-0.325901028	0.384793906	0.093143892
ENSG00000122218	COPA	57.150529	56.047193	52.98819	65.425193	62.619166	62.337475	59.72295	50.267756	60.603177	3.53E-09	0.31264723	0.174196508	0.093244342
ENSG00000106246	PTCD1	7.280697	6.676113	6.930658	6.914936	6.819836	6.587441	6.644848	6.208031	8.057735	0.266989888	0.103139102	0.38487856	0.093334709
ENSG00000120063	GNA13	15.165464	14.809199	15.6606	18.368151	17.022121	18.016054	16.182608	14.003467	15.517502	4.44E-10	0.37376543	0.255524656	0.09334543
ENSG00000197444	OGDHL	6.649892	6.212557	6.416623	4.27187	4.154168	3.8413	5.987321	6.006178	7.1705	0.000000354	-0.494438993	0.409059123	0.093404557
ENSG00000060491	OGFR	16.209865	15.887798	13.989419	42.686676	49.139853	52.999634	15.086417	13.870041	15.442535	1.71E-178	1.895425992	0.350325888	0.093527541
NewGene_412	--	4.27221	4.121885	4.913805	3.606893	3.681647	3.964153	4.256458	4.106114	4.526388	0.797950476	-0.038175954	0.434113054	0.093616056
NewGene_6474	--	2.578281	3.501692	2.947941	3.413307	2.203387	3.318431	2.995145	2.556089	4.10866	0.946787882	-0.020790861	0.667708623	0.093753542
ENSG00000001461	NIPAL3	2.268224	2.390848	2.368923	2.008838	2.27465	2.201045	2.174554	2.325066	2.493523	0.650022446	0.080003168	0.532340667	0.09376271
ENSG00000002549	LAP3	52.24657	48.705147	49.623027	331.298097	314.839537	331.960001	48.594167	51.233403	51.429339	0	2.813899695	0.242087514	0.093807461
ENSG00000163344	PMVK	12.282247	11.136764	9.837494	9.296861	9.103826	9.132969	9.865283	11.993684	11.421646	0.41140592	-0.126595934	0.546704068	0.093960274
ENSG00000130713	EXOSC2	17.766694	20.144343	20.061867	18.030518	15.931332	17.180982	20.127831	19.23011	18.714164	0.756703557	-0.037392729	0.354524163	0.094070253
ENSG00000242259	C22orf39	9.366803	12.145222	10.761867	7.920344	8.101616	8.172197	11.174768	8.600081	10.018394	0.602488886	-0.068766357	0.528959042	0.094143746
ENSG00000103742	IGDCC4	3.551321	3.015627	3.407106	2.108081	2.182109	1.99263	3.331623	3.195353	3.462352	0.00000177	-0.519593032	0.425747015	0.094148613
ENSG00000138190	EXOC6	13.977494	14.624641	14.360759	11.69854	10.828323	12.001605	13.911329	14.434326	14.784379	0.02482114	-0.176826382	0.263006595	0.094222453
ENSG00000077713	SLC25A43	2.125417	2.299997	2.217478	2.184251	2.176188	2.621445	2.194381	2.266015	2.172416	0.246917084	0.223149391	0.623203992	0.094240266
ENSG00000151247	EIF4E	7.601262	7.666146	8.651833	8.096629	7.859122	9.419246	14.093257	13.63662	13.016922	0.628570206	0.046052735	0.252043983	0.094243897
ENSG00000156711	MAPK13	36.080027	37.410853	33.799906	31.514423	31.48327	31.670705	37.581495	36.541523	41.243279	0.826252675	-0.019492325	0.189379725	0.094507311
NewGene_6713	--	0.956701	1.547979	1.067626	0.929902	1.412367	1.777967	1.227315	1.254597	1.093129	0.47260631	0.365232905	0.846490674	0.094591945
ENSG00000085382	HACE1	3.868318	4.511401	4.07467	3.628383	3.636926	3.648416	4.19243	3.914857	4.490425	0.889945453	-0.022849926	0.460188231	0.094685198
ENSG00000109572	CLCN3	6.896382	8.235759	7.401059	6.684286	6.414923	5.9704	7.038794	7.75725	7.125705	0.763530267	-0.033707491	0.277584245	0.094823227
ENSG00000156697	UTP14A	10.629852	11.967698	11.493928	10.310333	10.764198	9.94882	11.185342	10.922766	12.098455	0.930787487	0.012682392	0.357308744	0.094849613
ENSG00000176658	MYO1D	12.131951	12.492191	12.636764	10.675402	10.290442	10.271787	13.084937	12.107097	13.153687	0.043464972	-0.145422325	0.22955147	0.094876806
ENSG00000065970	FOXJ2	1.045351	1.279852	1.162868	1.507586	1.357016	1.392795	1.30925	0.956702	1.35293	0.005719954	0.446138448	0.647866297	0.094893372
ENSG00000197548	ATG7	8.814104	7.845959	8.062232	7.582595	7.540118	7.317222	8.497755	7.709574	8.731561	0.964700899	-0.006186382	0.339559758	0.09495728
ENSG00000137491	SLCO2B1	1.595558	1.599087	1.767178	3.64117	3.777744	3.555559	1.733293	1.708043	2.411167	8.24E-33	1.556843248	0.660591165	0.094996248
ENSG00000151012	SLC7A11	3.398135	4.16316	5.032603	4.239568	4.827326	4.192208	4.101728	4.011399	4.516474	0.094464403	0.223318711	0.491808181	0.095048655
ENSG00000077235	GTF3C1	18.101136	16.734302	16.003403	15.363861	14.524333	14.278594	16.792668	15.467308	19.051658	0.499860943	-0.05701348	0.271752337	0.095082859
ENSG00000186635	ARAP1	17.048563	15.290512	14.151571	14.744045	14.796458	14.275885	14.909191	15.27131	17.934252	0.621576007	0.053745574	0.354406454	0.095184982
ENSG00000108950	FAM20A	10.147634	7.93355	7.744086	5.855494	5.582362	6.078596	7.978548	8.381609	9.443222	0.000887237	-0.398724575	0.481456356	0.095240468
ENSG00000187608	ISG15	8.829949	7.240525	8.548099	102.302216	104.058562	113.960333	7.753293	8.438162	8.010917	0	3.836535582	0.654161387	0.09532445
ENSG00000197965	MPZL1	27.743407	28.043066	29.499905	26.873711	26.152253	26.226016	28.620629	27.681021	28.340057	0.74911043	0.024910262	0.155381863	0.095439291
ENSG00000123353	ORMDL2	16.773599	14.032269	15.805384	12.901192	16.50526	14.030028	14.559008	17.2782	15.019666	0.819329724	0.036529954	0.44378893	0.095444498
NewGene_2792	--	3.209319	2.422653	2.831726	2.07043	2.521769	2.633437	3.632651	2.364599	3.945406	0.73645097	0.070286418	0.577594835	0.09575936
ENSG00000101363	MANBAL	28.0371	23.852328	24.400171	21.837326	23.428155	24.923129	23.250508	26.736944	22.849042	0.887560905	0.0179977	0.345003454	0.095798231

Gene ID	Gene name	Ctrl-1 FPKM	Ctrl-2 FPKM	Ctrl-3 FPKM	IFNγ-1 FPKM	IFNγ-2 FPKM	IFNγ-3 FPKM	Sora-1 FPKM	Sora-2 FPKM	Sora-3 FPKM	Ctrl vs IFNγ DESeq2 FDR	Ctrl vs IFNγ DESeq2 log2FC	Ctrl vs Sora DESeq2 FDR	Ctrl vs Sora DESeq2 log2FC
ENSG00000285721	AL031281.2	7.854837	3.365417	4.188126	2.089725	4.536928	5.91931	5.91807	6.311984	3.092251	0.877878947	-0.14489047	0.887506551	0.095830872
ENSG00000116678	LEPR	1.953295	1.84141	1.617038	1.792949	1.732667	1.915437	1.684352	1.867366	1.853787	0.440073622	0.134171546	0.568426656	0.095836933
ENSG00000143578	CREB3L4	10.390119	8.666672	7.41495	7.511334	8.105038	7.678415	9.089694	8.204981	9.239608	0.844349546	-0.039499223	0.541080343	0.096006453
ENSG00000169762	TAPT1	9.836892	9.876565	10.788141	10.220214	11.793527	10.226608	10.375206	9.155764	10.550845	0.001695186	0.266644841	0.317248301	0.096027073
ENSG00000089682	RBM41	3.168917	3.360431	3.297142	3.781318	4.272361	3.831223	3.261183	3.033468	3.5852	0.000724962	0.46168305	0.555719635	0.096033474
ENSG00000254598	CSNK2A3	9.667372	10.451047	12.597775	10.747849	12.162395	10.794978	11.068491	11.339321	10.309931	0.192550091	0.191383254	0.529136735	0.096083723
ENSG00000226742	HSBP1L1	12.497874	12.379504	13.554217	15.729015	18.917455	17.665068	11.845944	13.632157	13.358946	0.00000333	0.591259641	0.545029825	0.096154724
ENSG00000104691	UBXN8	15.772848	14.786672	17.254197	11.660861	10.994574	11.424843	17.572803	15.304851	15.621057	0.000322252	-0.365229979	0.409128438	0.096264249
ENSG00000132507	EIF5A	533.041307	548.596309	558.380074	542.784445	536.871015	563.686247	530.740571	545.270323	561.459048	0.00322908	0.139917177	0.108449599	0.096350808
ENSG00000122566	HNRNPA2B1	428.19224	479.749266	515.743343	485.184512	464.264658	456.134693	520.363375	516.966352	505.830167	0.174266768	0.103493306	0.238075698	0.096457135
ENSG00000119326	CTNNAL1	59.765355	63.380248	61.264436	66.846649	67.300836	67.082514	61.319097	59.030582	64.608284	0.000000165	0.273690439	0.137006677	0.096466281
ENSG00000184220	CMSS1	6.174141	7.360097	7.472829	5.671961	5.155121	5.402196	6.958281	6.490683	7.179079	0.096323527	-0.17228685	0.352073235	0.096535353
NewGene_1675	--	1.349263	1.797062	1.570835	1.411466	1.20639	1.50663	1.762086	1.670929	1.450537	0.855435908	0.045072491	0.61427635	0.096615835
ENSG00000163694	RBM47	14.047343	14.846518	12.792163	20.660577	20.536227	21.357174	14.927284	12.751033	13.815811	1.77E-30	0.726855152	0.292761195	0.096619621
ENSG00000161956	SENP3	34.505599	36.219894	39.276205	33.107425	33.708065	33.524929	32.509924	38.752516	38.130104	0.941373914	0.00780437	0.313934007	0.096627713
ENSG00000114735	HEMK1	0.495146	0.46303	0.295389	0.283306	0.298999	0.462005	0.493375	0.609107	0.775487	0.127149268	0.314472063	0.66074749	0.096691571
ENSG00000150054	MPP7	3.639734	3.796464	3.870211	8.100904	7.912756	7.796412	4.183195	3.417127	3.91679	4.38E-56	1.210259749	0.430485328	0.096760355
ENSG00000111726	CMAS	21.469999	22.869492	21.597463	18.232116	18.079996	19.087673	23.522127	20.648739	21.900215	0.250574618	-0.106737199	0.311771747	0.096976753
ENSG00000081870	HSPB11	8.998668	8.572419	9.954841	9.915193	10.642307	10.333955	8.682506	9.977811	8.347091	0.033516766	0.321771656	0.59877202	0.096992482
ENSG00000167733	HSD11B1L	2.637299	2.056745	1.451306	1.522798	1.841639	1.40001	2.032692	1.694766	2.093811	0.375900383	-0.318127946	0.773633019	0.096997386
ENSG00000101343	CRNKL1	10.005968	10.246789	10.12297	8.110542	8.509539	9.124805	10.035126	9.973167	10.470417	0.363395648	-0.090296015	0.259081238	0.09712535
NewGene_5505	--	4.260907	4.175262	4.109695	5.799614	5.477202	5.138776	4.305591	3.937732	4.441105	4.86E-12	0.47415611	0.278657072	0.097226208
ENSG00000143324	XPR1	9.096303	8.892674	8.364512	7.910252	8.8745	7.971285	9.026158	8.312002	8.974555	0.699167942	0.03448409	0.188909648	0.097287892
ENSG00000068912	ERLEC1	29.431469	30.169682	32.819991	31.188781	30.986787	29.966417	32.019615	30.544566	30.47568	0.049372193	0.143560899	0.247223862	0.097368853
ENSG00000135047	CTSL	9.953795	10.074277	8.881319	9.507054	9.698294	10.672027	10.863022	9.633385	8.767196	0.023149594	0.257645445	0.441222673	0.097427511
ENSG00000099795	NDUFB7	76.547142	68.223419	65.469955	63.222198	64.512239	66.989471	67.972908	74.981834	67.61557	0.766766343	0.037140896	0.386012752	0.097461441
NewGene_1159	--	1.084529	0.562384	0.935674	0.44084	0.22881	0.511287	0.864271	0.817986	0.929238	--	--	0.828970786	0.097468572
ENSG00000112303	VNN2	4.398339	3.553659	3.567403	7.759828	7.79425	9.085498	3.282903	3.764859	4.176963	3.37E-21	1.192828664	0.60854906	0.097490132
ENSG00000180900	SCRIB	17.642111	17.305092	15.700157	14.562549	14.458314	15.937264	15.564724	15.492486	19.748274	0.909669446	-0.012832032	0.332920436	0.097515208
ENSG00000213533	STIMATE	6.94572	6.023715	5.400689	8.195482	8.661716	7.97683	6.268794	5.593131	5.86088	2.22E-08	0.575574333	0.458342201	0.097531757
ENSG00000170892	TSEN34	32.496935	33.835728	26.899632	33.460551	30.149913	35.256722	31.515096	35.951381	32.898608	0.008009522	0.242033687	0.327080729	0.097749317
ENSG00000205038	PKHD1L1	0.128036	0.077855	0.059231	0.108132	0.072095	0.094809	0.097238	0.088198	0.079888	0.717997531	0.198649254	0.842061699	0.097804645
ENSG00000130348	QRSL1	9.103104	10.488087	10.816381	10.710131	9.80744	10.314609	10.961512	9.870751	10.552731	0.135679179	0.147073622	0.346751771	0.09782403
ENSG00000100462	PRMT5	46.71138	45.648474	43.923497	38.20966	37.927435	35.612045	45.600895	44.840366	46.603069	0.048182127	-0.131636973	0.159618594	0.097899799
ENSG00000115718	PROC	11.569682	11.148516	10.391913	7.818006	8.151241	7.952442	10.815166	10.582238	11.096641	0.007542744	-0.310888034	0.415259216	0.097904148
NewGene_4844	--	2.219919	2.262214	2.462517	2.135192	2.006867	1.953255	2.252866	2.343689	2.568156	0.61378633	-0.064046043	0.370384543	0.097947614
ENSG00000110395	CBL	3.638629	3.43093	3.44186	2.822326	3.130173	2.976849	3.383896	3.376309	3.805485	0.150502155	-0.13002081	0.28475432	0.097948544
ENSG00000188313	PLSCR1	16.713551	18.470329	15.707655	60.874578	57.20471	61.935946	17.798736	15.62592	14.698536	2.03E-179	1.908957703	0.365296182	0.097954749
ENSG00000152558	TMEM123	134.408825	132.84127	142.087188	106.805344	108.19108	102.963154	135.65108	132.773577	145.533882	0.0000018	-0.226378399	0.115167542	0.098002403
ENSG00000174721	FGFBP3	0.476927	0.798717	0.75777	0.471279	0.398727	0.527867	0.739483	0.655591	0.632431	0.366550441	-0.378659542	0.801640178	0.098170829
ENSG00000077721	UBE2A	13.697968	15.001484	14.291756	15.137913	13.273546	12.996823	15.994511	13.318091	14.772122	0.590833768	0.066211874	0.345552599	0.098187837
ENSG00000183098	GPC6	21.261239	21.426246	21.681332	19.191691	18.899956	18.937318	21.775959	21.246285	21.569115	0.711065074	-0.027301721	0.139889068	0.09819723
ENSG00000134897	BIVM	10.368994	8.808446	13.02126	11.125821	9.576278	10.407556	8.907381	10.044192	11.532101	0.583959106	0.178992173	0.763410877	0.098255554
ENSG00000042286	AIFM2	14.844848	14.719013	15.963936	13.454855	14.021078	13.446514	14.587602	15.448298	15.607027	0.962563224	-0.0058109	0.268654592	0.098314024
ENSG00000116752	BCAS2	18.874361	21.318745	23.431341	19.149132	18.96101	19.502935	21.545443	20.810467	21.486586	0.977525542	0.004743116	0.395625613	0.098369747
ENSG00000080371	RAB21	3.225159	3.955484	4.300087	3.873146	3.876457	3.684423	3.994475	3.540265	3.9994	0.195772757	0.141934589	0.387578492	0.098593256
ENSG00000131591	C1orf159	12.080314	12.018419	12.005416	10.335935	8.611283	11.688157	12.072642	10.844692	13.375394	0.906086591	0.017011312	0.358107804	0.098649109
ENSG00000120451	SNX19	6.386658	6.537846	6.176767	6.754553	5.895938	6.042189	6.781606	5.932671	6.629471	0.16898676	0.118375288	0.270667129	0.098694156
ENSG00000163399	ATP1A1	175.402665	155.546016	175.469833	112.094897	122.463608	113.96161	153.329207	168.73845	187.286822	6.06E-10	-0.393785765	0.245139221	0.099087261
ENSG00000144043	TEX261	24.443477	24.148327	23.531277	25.987189	24.604511	24.067263	23.259924	23.491007	25.976545	0.001588267	0.197316143	0.181284511	0.099153622
ENSG00000100242	SUN2	43.235477	40.404858	37.039548	28.100194	27.180096	29.615637	38.979467	37.895949	44.938791	0.000000048	-0.371632528	0.238136014	0.099170064
ENSG00000115226	FNDC4	6.325604	5.748027	5.279198	3.213344	3.448109	3.628439	5.463338	6.10916	5.848715	0.0000883	-0.604195949	0.549315539	0.099185865
ENSG00000167333	TRIM68	1.557964	1.197603	1.438224	1.591869	1.087015	1.27015	1.46595	1.275827	1.718758	0.926590225	0.030959092	0.664578364	0.099229444
ENSG00000136699	SMPD4	45.860942	44.52295	45.933586	38.70298	34.667908	37.900687	42.097878	41.669764	47.616581	0.133172956	-0.091806825	0.142124765	0.099250755
ENSG00000134291	TMEM106C	72.732189	72.883992	76.643921	52.175773	49.547951	52.69642	69.026397	77.288873	76.966538	8.42E-12	-0.393900113	0.227575484	0.099261371
ENSG00000124571	XPO5	27.262838	27.520574	28.666145	24.389428	21.938954	21.733609	27.088013	27.55593	29.217867	0.027339599	-0.145270431	0.141906904	0.099292847
ENSG00000170099	SERPINA6	57.357731	54.317128	45.458042	49.898141	52.802201	49.896368	54.335658	55.528686	48.368765	0.452996431	0.083288413	0.381141333	0.099406828
ENSG00000196199	MPHOSPH8	6.983223	6.936768	6.165943	6.149886	5.393433	5.323404	6.113108	6.629404	7.459833	0.431484346	-0.093716751	0.39807024	0.099415821
ENSG00000178999	AURKB	55.846269	61.317974	60.595408	41.919638	39.464106	40.015012	62.547375	58.26426	56.936007	1.49E-08	-0.385847491	0.262247071	0.099445057
ENSG00000125351	UPF3B	5.988031	5.842828	6.478229	5.767267	4.895572	5.690256	6.009438	5.856884	6.116321	0.874465156	-0.028298665	0.446691343	0.099454103
ENSG00000179604	CDC42EP4	42.128829	42.109028	43.728224	25.634452	28.773783	27.624939	42.886642	39.381302	47.234096	2.05E-14	-0.50236642	0.166302622	0.099467871
ENSG00000221823	PPP3R1	24.543509	27.500692	31.63479	27.795469	27.048229	27.345127	28.50852	26.487145	29.162922	0.218123254	0.121369747	0.326133249	0.09951756
ENSG00000127837	AAMP	51.692086	48.954289	48.686893	53.985598	55.852269	56.986093	46.409851	49.619734	54.155027	0.00000139	0.297966352	0.219212466	0.099519307
ENSG00000108651	UTP6	16.041256	17.084923	18.178694	16.18575	15.097166	15.41854	17.126764	17.364386	17.146423	0.979353403	0.003037719	0.237506595	0.099569778
ENSG00000164168	TMEM184C	8.04133	8.051349	8.148663	8.19361	8.114173	7.731274	8.023119	7.733015	8.739927	0.134322428	0.133399185	0.280559396	0.09971717
ENSG00000147684	NDUFB9	177.437503	164.849666	152.538087	131.510406	139.933491	139.202117	165.867754	176.757759	156.372669	0.130564074	-0.124514276	0.300929755	0.099742643
ENSG00000168497	CAVIN2	4.983191	4.86482	5.095058	2.574696	2.80738	2.603109	5.186053	4.632517	5.217787	5.19E-11	-0.756212041	0.417881344	0.099837321
ENSG00000100138	SNU13	109.002106	106.306679	113.561418	90.866059	94.14473	93.209864	109.924979	118.60553	106.126645	0.020985321	-0.144099424	0.23989771	0.099865022
ENSG00000221978	CCNL2	81.301652	81.317328	87.359909	91.765984	80.748422	79.962649	87.003234	76.94713	93.574665	0.053066695	0.139226064	0.172578422	0.100028001
ENSG00000137106	GRHPR	44.464706	44.951712	38.145471	40.561405	40.683123	42.445896	40.899089	41.969484	45.906547	0.277745926	0.103926594	0.298696605	0.100056979
NewGene_6046	--	0.806578	1.09773	0.649228	0.65399	0.551327	0.86432	0.903348	0.756461	0.873364	--	--	0.836918955	0.100298784
ENSG00000143742	SRP9	280.971308	312.438559	321.885463	259.621459	248.424095	253.355895	304.409299	296.132233	302.505786	0.075673603	-0.117698922	0.169605333	0.100350875
NewGene_2571	--	2.436345	2.578884	3.580644	4.014382	3.054858	3.078636	3.057733	3.033576	4.043922	0.020999055	0.277410356	0.460188231	0.100528521
ENSG00000088247	KHSRP	253.549337	254.736895	246.612965	237.963995	235.578516	243.428785	258.692409	246.191982	279.796303	0.081196162	0.088154372	0.07975357	0.100554272

Gene ID	Gene name	Ctrl-1 FPKM	Ctrl-2 FPKM	Ctrl-3 FPKM	IFNγ-1 FPKM	IFNγ-2 FPKM	IFNγ-3 FPKM	Sora-1 FPKM	Sora-2 FPKM	Sora-3 FPKM	Ctrl vs IFNγ DESeq2 FDR	Ctrl vs IFNγ DESeq2 log2FC	Ctrl vs Sora DESeq2 FDR	Ctrl vs Sora DESeq2 log2FC
ENSG00000185453	ZSWIM9	2.576056	2.405812	2.593344	2.291717	2.086505	1.919559	2.509912	2.434577	2.729734	0.546808384	-0.11244355	0.524785336	0.100587161
ENSG00000198478	SH3BGRL2	10.281121	11.536197	12.18181	11.04005	10.719663	11.058949	11.617519	10.832098	11.743896	0.304697715	0.096937381	0.282629642	0.100736433
ENSG00000151729	SLC25A4	6.82591	10.191482	7.642364	5.818156	5.97087	7.653872	6.692173	8.950668	13.325293	0.137931737	-0.148602698	0.356030285	0.100763788
ENSG00000101474	APMAP	34.7573	31.571523	29.585563	24.449759	25.072866	23.887659	32.957912	31.589708	31.806022	0.002206155	-0.241714312	0.258979202	0.100955928
ENSG00000099974	DDTL	12.047217	12.601844	12.546942	12.350293	12.992836	12.85074	13.080181	12.944702	11.28206	0.084689863	0.186335048	0.435278965	0.100978109
ENSG00000165905	LARGE2	41.355048	39.975221	39.930161	25.451679	24.37181	26.4721	38.449436	39.837177	44.300517	2.56E-18	-0.528189677	0.181559547	0.101013679
ENSG00000179163	FUCA1	8.071198	7.604958	7.82794	6.400964	6.206933	6.460488	7.872486	7.90632	7.845647	0.222837773	-0.154730522	0.4149939	0.10102076
ENSG00000102898	NUTF2	33.178582	34.116303	34.50043	25.984031	26.275624	26.781997	29.510054	34.937258	32.267621	0.06213259	-0.137036054	0.2602417	0.101111268
ENSG00000162341	TPCN2	13.834772	13.897013	12.90207	11.556559	10.579002	11.670201	13.702203	11.606697	15.669489	0.027153286	-0.188173021	0.295796932	0.10119381
ENSG00000152503	TRIM36	0.246354	0.260097	0.354454	0.148731	0.147436	0.131185	0.415296	0.169066	0.225718	--	--	0.850368099	0.101219021
ENSG00000100926	TM9SF1	32.877167	26.084667	29.67462	24.491097	26.205054	27.230431	29.508878	30.113991	29.262588	0.986044084	-0.002645015	0.315283312	0.101388021
ENSG00000165629	ATP5F1C	135.17685	135.004164	126.939821	123.672099	140.355218	131.26717	134.651012	132.468132	132.747246	0.058932794	0.13956005	0.144404055	0.101497388
ENSG00000127423	AUNIP	0.810138	0.801091	0.633341	0.367527	0.568876	0.394571	1.29772	0.487032	0.613252	0.121752655	-0.625640834	0.821956525	0.101546245
ENSG00000137166	FOXP4	30.777014	22.024767	25.470518	25.140702	21.58553	22.537612	21.779534	20.635971	31.39141	0.786387298	0.026930432	0.289185919	0.101635095
ENSG00000172594	SMPDL3A	9.230848	7.884206	8.191098	6.841202	6.589369	6.080165	9.556204	8.173197	7.732863	0.067295974	-0.240711083	0.490443925	0.101683812
ENSG00000081803	CADPS2	11.601678	9.903545	9.745632	9.832504	10.922978	11.775176	9.980422	10.049551	10.036544	0.031941711	0.222298888	0.328217278	0.101709364
ENSG00000122674	CCZ1	33.45673	26.690717	28.062853	32.687985	32.848827	23.385705	35.937029	24.019261	29.681223	0.565374056	0.148722947	0.472594401	0.101720045
ENSG00000184983	NDUFA6	40.38487	37.495565	35.433764	32.85781	35.418442	31.99024	38.400116	42.687174	31.83687	0.624454778	-0.055420368	0.44009366	0.101774922
ENSG00000162545	CAMK2N1	9.144791	7.924777	8.358175	7.129798	7.561268	7.641805	8.17319	8.169999	9.275702	0.791479349	-0.040218015	0.398862483	0.101851464
ENSG00000081760	AACS	11.143194	10.243674	8.981074	9.426184	8.189154	9.155869	9.989601	9.774795	10.856378	0.78694215	-0.038399363	0.355713016	0.101867419
ENSG00000198721	ECI2	32.776824	33.875956	32.373935	36.831056	36.31843	35.253965	32.977233	32.080454	32.526595	0.0000546	0.280163579	0.233609381	0.10207502
ENSG00000153531	ADPRHL1	2.495385	2.075386	2.022629	2.125978	1.899201	2.215569	2.493572	1.841215	2.704382	0.910266138	-0.03553051	0.682746924	0.102228904
ENSG00000180228	PRKRA	34.114133	36.419827	39.278022	28.630771	27.134236	30.375903	35.777763	36.984486	37.531266	0.020977258	-0.187824415	0.227873649	0.102344368
ENSG00000088035	ALG6	7.054029	5.847242	6.380292	4.964671	4.037724	4.865096	6.797086	5.984173	8.120777	0.001040073	-0.42748816	0.494326801	0.102431721
ENSG00000127314	RAP1B	47.578091	56.541908	38.698011	53.951136	54.578691	51.57393	53.424904	57.157327	35.822668	0.802771587	0.021863776	0.136076771	0.102460053
ENSG00000092969	TGFB2	3.321518	4.020989	4.728331	3.878674	3.580361	3.994667	3.991247	3.770427	4.373106	0.655617105	0.076589802	0.488575801	0.102536904
ENSG00000115241	PPM1G	58.053772	54.343082	56.562279	51.064541	46.93969	49.580463	55.584171	55.352386	59.239651	0.528668375	-0.048247358	0.132963794	0.102540467
ENSG00000197415	VEPH1	5.388954	4.937553	6.118571	3.083102	3.118196	3.245696	5.597133	5.6324	6.114748	0.000000288	-0.718919334	0.490088283	0.102563819
ENSG00000214274	ANG	3.947244	3.018801	2.450004	8.03946	7.568509	7.666715	3.231591	3.475655	2.663394	8.01E-18	1.457299535	0.718598789	0.102604061
ENSG00000106605	BLVRA	30.374546	29.705306	31.215833	35.187587	34.097734	39.879529	31.031173	29.643651	30.967744	0.00000105	0.411278828	0.271361941	0.102704381
ENSG00000100522	GNPNAT1	18.072239	18.953513	20.281313	17.329615	16.041542	17.499981	18.996941	18.931092	20.029945	0.768889118	-0.030510005	0.214300324	0.103023417
ENSG00000108946	PRKAR1A	121.61839	121.374241	117.620808	80.550708	85.284119	84.963729	121.682185	113.833371	126.699019	3.14E-13	-0.352385766	0.083007488	0.103148882
NewGene_6659	--	3.354825	5.186488	4.995108	3.625034	3.716941	3.746725	4.82037	4.203382	4.968763	0.073664294	-0.236542043	0.428961707	0.10330235
ENSG00000135249	RINT1	9.531167	9.829401	9.940403	8.897152	8.254267	8.172615	9.939682	9.797099	9.846681	0.521167381	-0.071267939	0.289513749	0.103374365
ENSG00000057252	SOAT1	19.138065	20.259409	19.095816	22.356934	22.5817	21.313369	20.380275	18.973311	19.555853	3.3E-09	0.327976757	0.14174559	0.103378675
ENSG00000119899	SLC17A5	21.704102	23.666111	24.483107	22.028528	22.172829	21.268234	23.125408	22.429829	24.860168	0.541778217	0.054218973	0.195709587	0.103429667
ENSG00000168209	DDIT4	14.353735	15.938484	17.447792	16.47929	18.692894	18.273664	15.249668	15.354781	17.516615	0.00249485	0.312037748	0.366682203	0.103487239
ENSG00000152402	GUCY1A2	0.139086	0.203178	0.199706	0.209774	0.181911	0.151056	0.172113	0.155063	0.220251	0.665500444	0.148124465	0.735156767	0.103504673
ENSG00000151224	MAT1A	1.590479	1.557934	1.450342	3.365188	3.612143	3.526441	1.690875	1.274229	1.66944	7.64E-24	1.335582484	0.63099367	0.103511344
ENSG00000111786	SRSF9	89.715205	86.603505	77.720972	75.744537	81.601719	83.020184	81.054125	89.131947	84.988655	0.489901321	0.066033878	0.251866455	0.103576897
ENSG00000154473	BUB3	34.017552	37.977651	34.767656	28.977644	27.879904	28.459058	35.255144	38.152916	33.88178	0.024852172	-0.140203906	0.159713231	0.103665818
ENSG00000031823	RANBP3	50.363152	43.671147	43.750697	36.67847	42.960873	40.686319	40.304158	40.797202	51.855636	0.809577148	-0.02187634	0.144404055	0.103668372
ENSG00000143933	CALM2	378.442867	406.925973	395.918736	311.764712	321.980268	327.855557	400.216251	405.364457	408.924814	0.00665744	-0.147532009	0.100291285	0.103712959
ENSG00000186907	RTN4RL2	6.623039	7.488261	6.029197	13.745616	13.6359	14.094249	6.968369	6.396648	6.754901	7.81E-36	1.195406831	0.462997614	0.103744243
ENSG00000062524	LTK	2.669883	2.532943	1.990747	2.200253	1.425327	2.524442	2.468952	2.827577	2.249233	0.622765929	-0.12732641	0.629975713	0.103753963
ENSG00000169372	CRADD	6.452734	7.060446	7.31113	5.528418	5.179624	5.416377	7.457152	6.76896	6.616632	0.175972756	-0.232851296	0.5388578	0.103804188
ENSG00000171700	RGS19	6.655018	5.392474	7.396248	5.539628	6.422583	5.186215	5.537553	7.560149	6.401742	0.933602721	-0.021397017	0.602797684	0.103810042
ENSG00000164010	ERMAP	3.331259	2.929162	2.794686	2.943181	2.534701	2.321904	2.945625	3.442563	2.931854	0.659905817	-0.087116499	0.525460537	0.103819246
ENSG00000168005	SPINDOC	31.063279	29.167728	28.172404	26.108004	24.625659	25.885455	28.38633	28.831247	31.728654	0.562442956	-0.055781049	0.244317184	0.103832682
ENSG00000171475	WIPF2	6.758119	6.557159	6.801854	7.063514	7.071195	6.784258	6.942453	6.223501	7.351909	0.020923918	0.167454689	0.220092764	0.103868205
ENSG00000196535	MYO18A	14.44809	10.476305	10.394406	13.628731	15.129159	12.408949	11.849635	11.866583	13.673189	0.00000461	0.379371077	0.331989868	0.103909781
ENSG00000166452	AKIP1	18.255803	20.034502	21.062321	20.52979	16.943768	17.517241	19.536113	22.950564	19.322256	0.823573963	-0.03312718	0.345314771	0.103936101
ENSG00000170265	ZNF282	11.871683	12.362279	11.958356	13.082662	12.535069	12.766228	11.754967	12.247596	12.219704	0.001194388	0.233324127	0.225702112	0.104080839
ENSG00000163166	IWS1	39.964973	37.090574	39.086533	37.887468	37.967259	34.926571	38.83482	37.988939	39.657082	0.282362392	0.082810828	0.141251983	0.104113506
ENSG00000105486	LIG1	18.268252	17.001695	16.700617	14.994152	12.433872	14.672436	17.632731	17.259046	17.004523	0.065312159	-0.150941738	0.203242614	0.10432378
ENSG00000136098	NEK3	2.624889	2.388768	2.759563	2.415469	2.462388	2.77625	2.382528	2.400365	3.148248	0.650589537	0.103635129	0.608937167	0.104327325
ENSG00000146776	ATXN7L1	1.00385	0.685078	0.663907	0.770781	0.738684	0.638483	0.905552	0.699095	1.084476	0.985908245	-0.00743176	0.723922133	0.104405395
ENSG00000243927	MRPS6	33.060188	30.424978	31.69195	26.292467	31.785624	27.179308	29.784182	35.29377	30.706461	0.943839078	-0.011295159	0.373816907	0.104462618
ENSG00000159079	CFAP298	20.954721	18.758024	23.380633	15.427948	16.480215	17.142117	22.983188	23.317192	21.362036	0.021014907	-0.218407409	0.317248301	0.104469402
ENSG00000154174	TOMM70	35.490944	37.532154	36.896854	44.679802	43.434872	43.238747	38.33239	35.393204	37.082024	2.42E-16	0.404317184	0.122055125	0.104473252
ENSG00000170581	STAT2	7.095336	6.125364	5.825425	50.609697	44.494442	45.029316	6.614522	6.210132	6.237914	0	3.037141067	0.416212287	0.104487875
ENSG00000164024	METAP1	22.57874	23.984976	24.016265	23.881564	25.168043	23.291881	25.261198	22.059619	24.337028	0.014732087	0.180262629	0.20691191	0.104497113
ENSG00000138386	NAB1	18.594349	16.192105	15.466702	12.746718	10.853699	11.056652	18.615345	19.1637	15.423516	0.000101621	-0.307770871	0.254877957	0.104588907
ENSG00000167771	RCOR2	4.164451	3.577488	3.695513	2.713241	2.593825	2.822297	3.740103	3.62043	4.188346	0.025373158	-0.342343233	0.50616399	0.104595204
ENSG00000135900	MRPL44	28.673912	27.74791	28.670622	30.910879	29.744198	30.460653	29.638792	27.086769	29.124504	0.000334412	0.246022855	0.204997615	0.104759827
ENSG00000077312	SNRPA	46.637949	43.826521	43.674788	41.63112	40.976021	43.872178	44.80238	44.273767	48.881633	0.653113211	0.039897065	0.163480787	0.104797367
ENSG00000136159	NUDT15	10.097072	11.763144	10.385358	8.201346	8.37652	7.708247	10.337928	11.083575	11.082047	0.017976293	-0.262128538	0.371289581	0.10493363
ENSG00000165140	FBP1	3.027156	3.038384	2.425165	3.12308	3.108005	2.963731	3.266194	2.541231	2.7428	0.233692402	0.269429199	0.669644175	0.105033178
ENSG00000172478	MAB21L4	6.107472	5.51513	4.114394	5.263489	6.160841	5.304285	5.897785	4.717764	5.502988	0.202411725	0.234680464	0.577594835	0.105076024
ENSG00000085465	OVGP1	2.687848	2.298186	2.004424	1.861988	1.915763	2.004503	2.302737	2.469264	2.283399	0.622764125	-0.126190594	0.634695181	0.105088572
ENSG00000198911	SREBF2	59.582832	57.070211	52.414155	56.915025	57.456235	55.582585	55.57993	55.646467	58.784598	0.007960514	0.166772933	0.15105551	0.105122762
ENSG00000153574	RPIA	9.177794	9.741975	10.92452	8.582683	7.838361	8.091491	9.887165	10.514913	9.635322	0.303098776	-0.135985479	0.424973579	0.105203562
ENSG00000092203	TOX4	20.563587	19.004238	22.164524	19.86869	19.22222	19.741595	18.199013	19.47081	20.20243	0.001121184	0.191759055	0.157062411	0.105222762
ENSG00000009307	CSDE1	134.866136	146.488589	140.88866	131.648222	131.85757	130.616777	142.861389	138.39133	145.864495	0.496142481	0.042995918	0.077576177	0.105405582

Gene ID	Gene name	Ctrl-1 FPKM	Ctrl-2 FPKM	Ctrl-3 FPKM	IFNγ-1 FPKM	IFNγ-2 FPKM	IFNγ-3 FPKM	Sora-1 FPKM	Sora-2 FPKM	Sora-3 FPKM	Ctrl vs IFNγ DESeq2 FDR	Ctrl vs IFNγ DESeq2 log2FC	Ctrl vs Sora DESeq2 FDR	Ctrl vs Sora DESeq2 log2FC
ENSG00000065150	IPO5	109.62776	122.077246	117.473965	103.382747	107.229297	102.61028	115.395394	126.983372	134.265245	0.019660991	-0.113954929	0.059219297	0.105407174
ENSG00000122787	AKR1D1	0.626509	0.422885	0.381651	0.612956	0.477759	0.574178	0.652212	0.483474	0.502629	0.31677003	0.43480178	0.829042123	0.105457795
ENSG00000138138	ATAD1	11.671521	13.194653	13.694796	13.094942	12.547697	12.594092	13.220342	12.706468	13.492667	0.248353066	0.10196214	0.232402896	0.10549268
ENSG00000213782	DDX47	40.415439	39.400673	38.371126	30.461682	31.62355	32.79675	39.638918	41.537426	38.166534	0.024027153	-0.166361815	0.220724916	0.105653009
ENSG00000170899	GSTA4	8.503312	9.223445	7.122917	10.382971	9.228779	8.718035	9.496513	7.783977	7.643694	0.020252295	0.335152368	0.541084326	0.105696331
ENSG00000140740	UQCRC2	67.161468	65.372618	63.735403	57.345755	57.134748	54.55262	61.918456	65.499178	68.568513	0.207772635	-0.081164689	0.132268788	0.105747702
NewGene_556	--	1.479976	1.435176	1.62753	1.205793	1.312806	0.988095	1.331475	1.41876	1.929799	0.529910005	-0.213700371	0.732152912	0.105789423
ENSG00000136240	KDELR2	106.269167	107.584245	106.642955	132.792949	130.875058	136.325817	109.798087	103.298111	110.967825	1.13E-25	0.46894674	0.067847972	0.105842261
ENSG00000003756	RBM5	22.494353	19.774431	20.348333	21.597227	19.926574	18.979486	21.719731	19.775515	22.07167	0.323104676	0.090960119	0.217913065	0.10597387
ENSG00000114904	NEK4	3.147315	3.399789	3.424802	2.871448	2.727415	3.145127	3.012936	3.621023	3.266697	0.388637432	0.115935558	0.428163015	0.106043367
ENSG00000129187	DCTD	37.266053	37.140507	37.999125	36.375366	34.321434	36.572013	37.682821	37.740736	38.386573	0.526190391	0.052428486	0.145804774	0.106294889
NewGene_1915	--	1.754321	1.561586	1.21706	1.384029	1.211894	1.720834	1.423775	1.460078	1.710367	0.891751387	0.056320724	0.731328147	0.106365481
ENSG00000235961	PNMA6A	4.19409	4.550252	4.274984	4.160336	4.452668	5.121663	4.47376	4.118533	4.568664	0.150081465	0.227105586	0.48661727	0.106368842
ENSG00000197822	OCLN	14.155116	10.614293	11.107321	19.13566	21.856392	19.115617	12.114353	14.101808	11.383768	5.51E-35	0.762455453	0.217825422	0.10637426
ENSG00000178971	CTC1	4.225984	3.570521	4.046896	3.258796	2.973199	2.834569	4.14603	4.122569	4.092894	0.025624975	-0.241753263	0.326346427	0.106514887
ENSG00000147996	CBWD5	13.166069	20.176423	19.484351	14.496919	16.894024	12.864322	17.459265	16.566004	16.837368	0.967931529	0.007795517	0.441982215	0.106557489
ENSG00000100028	SNRPD3	63.923358	62.402996	58.076542	58.172456	56.287178	54.799525	62.937153	64.488234	58.517908	0.80002832	0.022858231	0.223545077	0.106559388
ENSG00000197249	SERPINA1	1143.94688	893.542432	798.850859	839.863006	880.845508	797.070724	1015.310771	965.557827	1006.791061	0.657042066	-0.092836316	0.323942018	0.106832253
ENSG00000135387	CAPRIN1	183.50374	182.181852	181.046911	193.205724	190.077844	186.64098	190.599147	179.547301	184.671091	0.00000253	0.205566728	0.067345038	0.106844789
ENSG00000149260	CAPN5	1.043443	1.177332	0.879305	1.360911	1.143199	1.348431	0.854163	0.970363	1.329222	0.027341192	0.440869939	0.675789958	0.106916044
ENSG00000198087	CD2AP	11.152685	13.579576	14.935063	22.684786	20.105843	21.476328	13.541018	12.373814	14.172092	5.59E-23	0.843379491	0.333610353	0.106934176
ENSG00000053702	NRIP2	0.885965	0.985649	0.756851	0.766302	0.553569	0.63987	1.163426	0.65635	0.837485	0.430967231	-0.271919104	0.755099276	0.107103877
ENSG00000174799	CEP135	2.014421	2.454988	2.476012	2.026042	1.845378	1.95843	2.397643	2.147847	2.516836	0.532573651	-0.105358804	0.475252828	0.107119465
ENSG00000108559	NUP88	34.766186	35.600043	34.997064	35.43348	33.639912	32.998659	35.574443	36.48739	34.62577	0.557104553	0.047304373	0.14934017	0.107139241
ENSG00000157349	DDX19B	11.512368	10.496438	10.781128	10.014642	9.069683	10.579194	11.798459	11.298776	11.957002	0.930787487	0.011310016	0.242362037	0.107208329
ENSG00000159111	MRPL10	28.92473	29.587529	25.43217	23.908862	23.142702	23.607582	26.176946	27.53871	30.665729	0.416519809	-0.081092368	0.278049623	0.107215576
ENSG00000168952	STXBP6	3.662659	3.800757	3.712605	4.061105	3.357217	3.851027	3.788447	3.572838	3.78281	0.156603269	0.178598588	0.397094709	0.107334975
ENSG00000204256	BRD2	75.19117	70.60401	70.911736	76.200735	73.216099	73.51666	70.755552	65.813466	79.187691	0.014748907	0.139813782	0.113189481	0.107344125
ENSG00000151348	EXT2	19.395344	17.414671	18.363388	16.484737	17.330161	17.286294	18.579441	17.687944	19.404239	0.628370944	0.049408322	0.196399579	0.107362485
ENSG00000106399	RPA3	17.174564	17.31315	18.704513	16.143034	16.784233	17.338154	19.554222	17.816477	14.918445	0.741077043	0.044739151	0.373824361	0.107423588
ENSG00000126457	PRMT1	276.933683	256.168553	266.212252	206.201389	210.381093	203.269112	252.80335	266.169028	291.302473	0.0000214	-0.225079032	0.117971716	0.107538923
ENSG00000167716	WDR81	14.626004	13.286944	11.108714	14.354289	14.489573	14.618729	12.584023	11.727254	15.640857	0.001426847	0.28683712	0.366370036	0.107558089
ENSG00000237190	CDKN2AIPNL	16.899124	21.19054	18.709262	16.907097	17.111689	17.01423	18.997263	19.457458	18.480194	0.999339859	-0.000150549	0.388335192	0.107608446
ENSG00000176946	THAP4	23.628343	23.853927	22.362357	22.329969	21.063076	21.660031	22.141872	22.755324	25.80277	0.628370944	0.04479541	0.215015293	0.107612397
ENSG00000085365	SCAMP1	6.413057	7.814778	7.383804	6.121429	6.399212	5.46772	7.043917	6.861964	7.888827	0.231658309	-0.138114132	0.320610206	0.107617616
ENSG00000126709	IFI6	1.888583	1.738032	2.703029	43.275433	44.610567	46.988988	2.072602	2.181533	2.168136	5.02E-245	4.534626519	0.77199805	0.107652406
ENSG00000147130	ZMYM3	17.721649	17.805511	16.079764	17.031803	16.913557	15.540465	17.264721	16.674905	18.112653	0.31900895	0.073626925	0.134022341	0.107723847
ENSG00000164976	MYORG	13.34376	13.334741	11.460748	12.872249	14.287097	13.612091	12.546495	12.229345	13.801406	0.002886313	0.244190033	0.220536458	0.107740855
ENSG00000105219	CNTD2	2.968398	2.313056	2.295541	2.375901	2.363578	2.232563	2.982127	2.29871	2.489882	0.916763513	-0.033033652	0.659091867	0.107987045
ENSG00000167992	VWCE	22.747284	20.927773	21.862495	23.075276	21.270671	21.630747	22.11667	20.738623	23.366334	0.022599505	0.159462212	0.164969473	0.108017934
ENSG00000162694	EXTL2	7.248499	9.796339	9.104208	6.52986	6.809186	6.65581	7.201866	9.462207	9.218943	0.515917769	-0.082861995	0.352089485	0.108068714
ENSG00000198171	DDRGK1	34.785541	31.618754	33.635882	30.793448	29.960308	33.147236	32.121842	33.493062	35.737056	0.629155412	0.052120282	0.237413261	0.108100208
ENSG00000099330	OCEL1	8.72935	8.163787	7.365488	9.63012	9.8349	9.40718	9.991579	8.069349	7.028862	0.009878302	0.359455162	0.581090174	0.108250186
ENSG00000204947	ZNF425	1.323184	1.013077	1.048621	1.216308	1.052084	1.217444	0.970228	1.14872	1.309149	0.486231265	0.189972503	0.687510509	0.108480931
ENSG00000169299	PGM2	19.0895	17.957155	17.688594	14.387035	14.787505	14.457122	18.7712	18.253427	19.11622	0.01540203	-0.182085529	0.168639329	0.108552319
ENSG00000139641	ESYT1	31.467915	32.286305	29.827593	23.040662	22.977573	22.641666	30.486571	30.213998	33.505721	5.57E-08	-0.307396054	0.114988846	0.108718362
ENSG00000170634	ACYP2	4.045036	3.215281	4.398169	3.428147	3.61123	3.555642	4.23169	3.800036	3.910074	0.896009013	0.042061548	0.664081137	0.108756145
ENSG00000029639	TFB1M	18.677158	18.112707	18.941311	15.614271	17.433645	15.060508	19.934122	18.263	18.126684	0.611814315	-0.06549421	0.304548808	0.108787231
ENSG00000108523	RNF167	53.306987	51.676964	53.524663	49.782637	47.31893	49.510021	52.215212	53.32671	53.374002	0.96371881	0.004410353	0.160621719	0.108798198
ENSG00000060237	WNK1	46.175205	44.507762	46.955713	43.056898	41.351283	40.777102	45.800021	44.855935	51.299978	0.90262908	0.009032457	0.075988566	0.10883834
ENSG00000128513	POT1	8.257692	8.639696	7.937846	7.533758	7.764327	7.846741	8.051909	7.980712	9.561253	0.576972113	0.060114904	0.267253885	0.108951214
ENSG00000070371	CLTCL1	2.879357	2.320971	2.437589	1.83504	1.872388	1.748966	2.561326	2.400932	2.5733	0.048802418	-0.291888062	0.465008612	0.109289192
ENSG00000105518	TMEM205	32.255246	27.974509	25.144865	26.818616	27.957887	28.302212	29.000621	29.703982	27.695691	0.431417904	0.105738747	0.403051363	0.109293495
ENSG00000166166	TRMT61A	5.609505	5.75609	5.67506	4.403121	3.977983	4.674956	5.197797	5.783734	6.284284	0.045023743	-0.236251161	0.373824361	0.109376044
ENSG00000100416	TRMU	12.19103	14.054026	13.04415	12.506129	12.513062	12.133195	13.522562	11.61473	15.073864	0.47829384	0.085173507	0.341592333	0.109466941
ENSG00000106009	BRAT1	20.937888	19.940643	18.362914	14.471635	14.992498	16.374726	20.033064	18.769085	21.846524	0.011088068	-0.221289484	0.211859434	0.109469805
ENSG00000071537	SEL1L	24.93953	26.084923	25.556452	29.490547	27.734227	27.485115	25.954809	24.377835	27.27118	6.27E-09	0.290829263	0.076762463	0.109572181
ENSG00000130522	JUND	51.617712	42.269507	39.223533	36.719087	40.09731	41.56493	46.237897	43.464507	45.062541	0.88395465	-0.021739439	0.307855782	0.109726479
ENSG00000119681	LTBP2	1.667844	1.52014	1.360509	1.142545	1.20698	1.025201	1.813436	1.391432	1.626998	0.033469075	-0.302734944	0.446623003	0.109727959
ENSG00000111328	CDK2AP1	65.528761	64.256661	66.325203	51.605501	58.642613	53.85762	63.885588	62.785007	73.863518	0.070894376	-0.147195942	0.134827134	0.109796038
ENSG00000214087	ARL16	31.891262	31.013511	34.21257	29.049725	25.236399	26.136766	32.859255	32.583556	31.00549	0.410918746	-0.100184392	0.317984923	0.109915631
ENSG00000110841	PPFIBP1	11.437321	13.586634	14.016177	8.993613	9.636641	8.863067	14.30757	12.843776	14.254858	0.00019166	-0.296440449	0.191713188	0.109996233
ENSG00000171812	COL8A2	0.657555	0.724666	0.775775	0.499286	0.75341	0.496877	0.668554	0.752998	0.737571	0.710663485	-0.128357404	0.673587034	0.109999062
ENSG00000172469	MANEA	8.093867	9.570465	9.214602	8.330413	8.336641	9.077343	8.712886	8.458139	9.828409	0.363629366	0.099136961	0.297739652	0.110094879
ENSG00000108509	CAMTA2	3.454079	3.636665	3.731977	3.573873	3.278083	3.144367	3.617327	3.470192	3.816624	0.829380144	0.034639736	0.361237334	0.110169199
ENSG00000171792	RHNO1	26.300792	28.498353	29.170145	25.047119	25.585476	24.768266	29.672757	26.016565	29.651854	0.958729464	-0.006214311	0.202778601	0.110336343
ENSG00000170142	UBE2E1	79.617314	88.155907	92.65144	83.73721	81.754479	83.884974	88.613434	90.639033	82.839138	0.402645494	0.06655799	0.194923116	0.11034324
ENSG00000222009	BTBD19	2.075211	1.883598	1.844833	1.0083	0.803584	0.845886	1.818053	1.792519	2.587412	0.000146339	-1.026267532	0.685860376	0.110392043
ENSG00000041880	PARP3	4.471546	3.771785	3.779196	9.01512	9.864624	9.661245	3.798638	4.120092	4.524033	2.36E-32	1.33056183	0.531267905	0.110503795
ENSG00000113583	C5orf15	21.296856	24.112991	24.473322	38.57505	35.250477	37.586094	23.280319	21.442146	26.133173	9.63E-34	0.820434176	0.247374802	0.110507517
ENSG00000110104	CCDC86	49.989775	51.252701	53.986815	40.79508	40.059391	42.98934	51.842133	49.781528	55.294318	0.00819543	-0.178160785	0.123125288	0.110538441
ENSG00000159618	ADGRG5	2.241587	2.099241	2.115604	1.781058	1.894733	1.672943	2.109457	2.171979	2.231521	0.353088745	-0.171723436	0.518012703	0.110550448
ENSG00000180628	PCGF5	4.825866	5.213653	5.261849	7.710267	8.080798	8.037009	5.565287	4.905413	5.0108	7.75E-29	0.786986902	0.271840866	0.110741576
ENSG00000122971	ACADS	12.680899	11.388827	9.405442	12.651476	13.750987	12.851494	10.831287	10.864702	12.239888	0.001285334	0.376322834	0.412225039	0.110820541

Gene ID	Gene name	Ctrl-1 FPKM	Ctrl-2 FPKM	Ctrl-3 FPKM	IFNγ-1 FPKM	IFNγ-2 FPKM	IFNγ-3 FPKM	Sora-1 FPKM	Sora-2 FPKM	Sora-3 FPKM	Ctrl vs IFNγ DESeq2 FDR	Ctrl vs IFNγ DESeq2 log2FC	Ctrl vs Sora DESeq2 FDR	Ctrl vs Sora DESeq2 log2FC
NewGene 691	--	1.611019	1.998671	2.019309	1.981503	1.780365	1.340905	1.953034	1.858847	1.904321	0.986348802	-0.004175772	0.452843057	0.110833515
ENSG00000005379	TSPOAP1	0.343971	0.325098	0.191916	0.202913	0.260987	0.226757	0.401883	0.287189	0.298001	0.93402599	-0.04238279	0.771177779	0.110939008
ENSG00000123739	PLA2G12A	5.744574	6.399795	7.192731	6.307321	5.86902	6.329594	6.776633	6.365011	7.231124	0.492243386	0.081177949	0.302990505	0.110970654
ENSG00000109689	STIM2	15.764534	16.181996	15.124639	15.362624	14.961593	13.883502	16.736649	14.617724	17.616378	0.88148001	0.015645634	0.197750661	0.111025394
ENSG00000157823	AP3S2	13.390879	11.33769	11.419435	11.06105	9.262975	8.714046	11.952553	11.161159	12.585203	0.103633811	-0.147426276	0.203820486	0.111091903
ENSG00000131174	COX7B	63.975819	52.78292	52.987488	83.711824	59.731874	65.758307	74.044372	102.921993	40.44144	0.026696938	0.203732518	0.457461487	0.111281814
ENSG00000185442	FAM174B	0.237537	0.420526	0.592034	0.333286	0.396035	0.516103	0.364098	0.677955	0.544195	0.648598178	0.361033464	0.879547072	0.111315134
ENSG00000130175	PRKCSH	173.780004	165.344402	158.784168	154.52932	147.687847	157.332172	172.740675	168.00142	174.750011	0.72998085	0.026181843	0.075648714	0.111325165
ENSG00000148688	RPP30	16.978801	19.031734	19.343534	16.937924	16.376379	17.572057	22.152976	16.069324	15.332953	0.575970302	-0.06447644	0.291759365	0.111373668
ENSG00000108344	PSMD3	82.018802	77.067403	76.209121	76.442131	73.360768	75.136417	77.755436	78.742847	81.893243	0.207604934	0.081704325	0.098673877	0.111387515
ENSG00000128534	LSM8	17.673853	20.126054	20.48555	19.4238	20.762456	21.099542	18.414715	19.487195	16.536104	0.031583846	0.204558902	0.340499241	0.111543392
ENSG00000058453	CROCC	0.707441	0.638788	0.543257	0.4285	0.464722	0.475872	0.726725	0.59596	0.590116	0.202595118	-0.316316566	0.648419998	0.111561897
NewGene 6531	--	1.437857	1.775607	1.902032	2.198322	1.962074	2.016176	1.553285	2.088864	1.871927	0.021079369	0.429154258	0.60572329	0.111567642
ENSG00000119953	SMNDC1	9.634846	10.0061	10.793712	10.259736	9.424393	8.887706	10.350699	9.76927	10.775308	0.524826017	0.069156227	0.224466466	0.111588682
ENSG00000103018	CYB5B	27.962493	27.553244	27.947501	43.036048	40.044775	38.839997	27.755344	30.641003	28.618683	2.37E-32	0.654043637	0.088248589	0.111590983
ENSG00000109113	RAB34	120.817043	119.534979	111.722099	79.205712	84.392371	83.070195	115.151811	123.273535	126.254397	2.53E-11	-0.390627065	0.120794532	0.111727427
ENSG00000198157	HMGN5	1.855245	1.901991	2.021483	2.41442	1.874142	1.660087	1.753003	2.813482	1.974675	0.7263634	-0.153345224	0.732078646	0.111733967
ENSG00000285943	AC112128.1	0.665833	0.850216	0.78426	0.616293	0.538494	0.432233	0.687369	0.918557	0.712655	0.094384956	-0.384382561	0.635395636	0.111776202
ENSG00000185825	BCAP31	72.274109	69.552037	64.22253	56.115507	54.911952	57.004052	67.646972	68.659366	71.625104	0.032552828	-0.146249487	0.126240646	0.111862077
ENSG00000241399	CD302	2.231909	2.471828	2.759715	1.637477	1.859598	1.755885	2.52533	2.101446	2.945514	0.032179029	-0.352895382	0.527947032	0.112131498
ENSG00000105865	DUS4L	4.134643	4.648326	3.51668	3.962154	3.6773	4.601963	4.400387	3.792835	4.225086	0.234675521	0.200052502	0.506929711	0.112200212
ENSG00000123096	SSPN	1.31775	1.472187	1.638703	1.278219	1.160457	1.545921	1.713747	1.196571	1.717097	0.931027733	0.02486671	0.59680645	0.112216286
ENSG00000162129	CLPB	6.163531	5.493302	3.680743	4.244223	4.477398	4.57179	6.16502	5.665952	4.041055	0.007883022	-0.256518988	0.308446149	0.112237766
ENSG00000167987	VPS37C	9.303809	8.711646	8.923008	8.884998	8.546424	8.768397	9.371901	9.039069	9.000466	0.300997147	0.108191309	0.277254288	0.112319828
ENSG00000085433	WDR47	1.344632	1.359703	1.355445	1.201129	1.220776	1.30117	1.263326	1.375083	1.479909	0.994315455	-0.002169095	0.56579553	0.112398371
ENSG00000148343	MIGA2	4.628447	4.451487	4.164642	3.790857	3.619553	4.085466	4.395189	3.976347	5.009856	0.959416255	-0.008875697	0.363506769	0.112434972
ENSG00000181754	AMIGO1	1.077905	1.283133	1.261332	0.975939	0.833519	1.132192	1.134131	1.094157	1.248018	0.827948169	-0.059400808	0.573566931	0.112482054
ENSG00000167693	NXN	28.929763	27.262523	29.538554	14.03967	15.237596	14.676549	28.303554	26.38319	31.083678	5.77E-34	-0.814357543	0.138413927	0.11253677
ENSG00000089006	SNX5	69.653959	74.778273	77.757211	73.613409	69.080035	68.197124	79.633821	72.906533	73.661157	0.400364464	0.065145184	0.153475487	0.112721672
ENSG00000158220	ESYT3	0.860775	0.746784	0.379289	0.786284	0.525299	0.57509	0.780831	0.771602	0.771664	0.993566561	0.004484372	0.751179587	0.112724883
ENSG00000168066	SF1	99.23491	96.703032	101.697844	99.043538	95.975462	100.258306	100.82588	94.558697	105.87331	0.011871359	0.133233697	0.059253074	0.11297916
ENSG00000182621	PLCB1	22.092496	22.412826	22.247738	17.757741	17.860122	17.998294	26.271102	25.605461	27.646784	0.001781663	-0.182078934	0.097882347	0.11302982
ENSG00000167862	MRPL58	23.757677	25.861555	25.177283	23.43501	23.304845	23.459366	24.155311	24.467016	26.619204	0.570948405	0.070548086	0.302119347	0.113196653
ENSG00000186350	RXRA	28.279367	25.543863	20.298368	33.648682	35.208992	35.742603	26.743727	21.94249	26.67153	7.23E-11	0.6438096	0.3829815	0.113222009
ENSG00000119471	HSDL2	21.108726	22.201888	22.118416	23.438151	23.343442	23.737118	23.743922	21.157265	21.714923	0.0000427	0.255288505	0.17068549	0.113310743
ENSG00000173226	IQCB1	12.966157	14.229663	13.748534	14.46324	13.091374	12.624737	13.605246	12.945141	14.527917	0.178922886	0.129936235	0.221512525	0.113417278
ENSG00000010803	SCMH1	12.406988	12.325691	13.350646	11.728124	11.645497	10.530985	12.441565	12.186578	13.927348	0.628127627	-0.053589207	0.204997615	0.113446136
ENSG00000184922	FMNL1	0.942813	0.730945	0.806419	0.389723	0.352741	0.359634	0.750371	0.79078	0.678034	0.00053505	-0.992562801	0.692220076	0.113484003
ENSG00000111879	FAM184A	2.024468	2.301866	2.87715	2.69092	2.693899	2.502057	2.670522	2.172357	2.902873	0.073768688	0.274462371	0.51967373	0.113510658
ENSG00000131732	ZCCHC9	29.701736	33.02925	36.255569	27.43001	26.262528	29.587242	35.941838	35.303559	28.900307	0.109733641	-0.145553911	0.259464768	0.1135151
ENSG00000135148	TRAFD1	14.361403	13.088109	11.533603	27.738071	26.792911	24.892265	12.642577	13.604657	12.557822	2.11E-65	1.204263775	0.265593964	0.113522668
ENSG00000171953	ATPAF2	7.836655	6.771243	7.427913	6.266289	6.852971	6.780085	7.564424	7.192214	7.947012	0.998691563	0.000542738	0.427124241	0.113550337
ENSG00000137575	SDCBP	20.256766	20.315016	20.833337	16.183705	18.671585	15.682136	20.018963	20.20515	21.983916	0.194043638	-0.130714681	0.177958503	0.113614358
ENSG00000197971	MBP	10.779505	10.370766	10.934761	8.205793	8.841471	8.638869	10.689306	10.117832	11.787847	0.028045894	-0.179428576	0.179056159	0.11364976
ENSG00000157557	ETS2	25.523699	23.146463	25.69444	21.419123	19.858983	20.525638	24.857673	26.248851	27.042932	0.068133345	-0.13610081	0.164643569	0.113708459
ENSG00000162642	C1orf52	8.841947	10.847573	11.161017	9.812593	9.553751	8.957766	10.651547	9.456896	11.152102	0.903370328	0.024456985	0.45394586	0.11371582
ENSG00000169925	BRD3	12.252667	11.456858	12.219745	12.65534	11.93012	12.704348	11.956153	11.436278	13.308629	0.005250763	0.193617948	0.165329328	0.113849711
ENSG00000257218	GATC	16.482734	18.738907	19.41173	15.803145	14.351783	15.50857	18.716826	17.540566	19.351309	0.187505981	-0.11538421	0.183385125	0.113869202
ENSG00000160584	SIK3	5.486109	4.793795	5.180519	5.960495	5.387397	5.407058	6.041372	5.346048	5.447113	0.002557631	0.274298473	0.284447346	0.113948949
ENSG00000109063	MYH3	0.852155	1.038831	0.956221	1.30617	1.258218	1.153526	0.911992	0.910054	1.073267	0.001165755	0.530673087	0.571362018	0.114063185
ENSG00000100142	POLR2F	18.748223	24.779619	21.646528	18.738613	16.052445	16.636431	22.349507	24.566833	20.76218	0.664685895	0.060628671	0.442217564	0.11409481
ENSG00000135269	TES	66.567174	63.690474	65.176604	48.953177	49.303862	48.548539	69.529203	64.215281	69.90784	0.000000188	-0.275534115	0.070092895	0.114225258
ENSG00000166529	ZSCAN21	12.494989	13.893198	13.43813	13.987979	11.61473	11.966301	13.551655	11.990624	12.747399	0.686936471	0.058977455	0.335911497	0.114228828
ENSG00000108733	PEX12	4.311798	4.610693	4.72846	4.882913	5.116266	4.796945	4.405708	4.504312	4.254135	0.038532576	0.27629922	0.438112633	0.114243475
ENSG00000179632	MAF1	61.197878	60.416261	51.830103	54.131911	60.162548	59.241913	56.544945	57.031146	64.028249	0.091436182	0.13955529	0.180797518	0.114334437
ENSG00000090534	THPO	7.702807	7.891382	6.819665	12.70033	10.143532	10.581658	7.252347	7.703579	7.823578	6.81E-10	0.71357521	0.422826398	0.114373758
ENSG00000171243	SOSTDC1	2.732528	2.96354	2.698592	0.812226	0.689188	0.945183	2.85359	2.398485	2.732957	2.33E-13	-1.611657135	0.573467	0.114409757
NewGene 1632	--	2.442849	2.329222	2.78393	3.023921	2.596407	2.064977	2.470454	2.442762	2.759181	0.419270294	0.167538353	0.50616399	0.114432934
ENSG00000082996	RNF13	15.566378	17.415974	18.771479	14.714724	14.939932	14.306898	17.918581	16.372823	17.00076	0.678090285	-0.044009421	0.193736171	0.114442204
ENSG00000105576	TNPO2	21.796223	24.861924	24.607381	22.248374	18.724321	18.423635	27.087391	22.738304	25.88015	0.077564084	-0.131319362	0.12979991	0.114450691
ENSG00000110514	MADD	13.83778	12.571431	11.596356	11.532414	12.072884	11.386087	12.641797	11.866207	14.07504	0.89481531	0.01620039	0.220857915	0.114591811
ENSG00000108602	ALDH3A1	0.463637	0.552117	0.513813	0.562351	0.388331	0.530141	0.668116	0.2624	0.721949	0.955226678	-0.036773371	0.828763591	0.114602933
ENSG00000128789	PSMG2	17.245669	18.691786	20.691685	19.946408	20.735996	21.702744	19.622791	19.125075	19.372819	0.001322759	0.300464613	0.292195325	0.114695023
ENSG00000196821	C6orf106	42.485777	42.033324	40.722779	48.510371	50.470614	48.749087	41.885139	41.113868	43.51826	2.71E-14	0.397691664	0.066705012	0.114695663
ENSG00000183258	DDX41	28.932115	29.741552	28.261249	21.161686	22.822581	24.763224	26.730465	29.813574	31.08536	0.016067065	-0.177145378	0.175722227	0.114759294
ENSG00000181192	DHTKD1	5.52131	5.584673	5.10278	5.660048	5.712335	5.14075	5.474086	4.955961	6.069107	0.079941909	0.174070527	0.27672059	0.114773981
ENSG00000036549	AC118549.1	10.97167	11.296907	10.524422	10.373705	9.878752	10.800574	11.209669	9.827418	11.974184	0.32122472	0.088570419	0.20498633	0.114827118
ENSG00000151552	QDPR	26.95463	26.766687	26.270606	21.966093	19.52231	22.357639	26.257844	26.386149	28.56475	0.016230345	-0.208431684	0.178878922	0.114935702
ENSG00000105676	ARMC6	38.689765	39.076337	37.381292	27.969268	29.804162	27.913407	35.001261	38.990231	44.077038	0.00000824	-0.289581589	0.177000118	0.114985335
ENSG00000185043	CIB1	28.202961	27.155744	25.262359	24.515225	25.20687	24.100879	27.257458	25.656841	28.241006	0.827329095	0.027540657	0.231109612	0.115096553
ENSG00000198363	ASPH	79.815973	85.678627	93.322158	70.414368	68.47333	66.376994	82.39202	80.423524	89.900092	0.02747889	-0.141726366	0.108556057	0.115186874
ENSG00000170315	UBB	493.94903	500.407328	464.986946	509.771066	522.156035	508.814859	487.485727	501.259511	504.417654	0.0000187	0.213835353	0.068954751	0.115221699
ENSG00000119888	EPCAM	222.035985	216.416917	232.506849	177.906779	176.056512	175.349454	229.118637	218.693098	234.122086	0.000136847	-0.195298831	0.058203775	0.115223507
ENSG00000105887	MTPN	71.186304	78.239631	82.87153	63.125575	62.591874	65.586149	77.7749	76.595625	81.708145	0.052600997	-0.135025308	0.117781106	0.115332344

Gene ID	Gene name	Ctrl-1 FPKM	Ctrl-2 FPKM	Ctrl-3 FPKM	IFNγ-1 FPKM	IFNγ-2 FPKM	IFNγ-3 FPKM	Sora-1 FPKM	Sora-2 FPKM	Sora-3 FPKM	Ctrl vs IFNγ DESeq2 FDR	Ctrl vs IFNγ DESeq2 log2FC	Ctrl vs Sora DESeq2 FDR	Ctrl vs Sora DESeq2 log2FC
ENSG00000089693	MLF2	105.981249	105.780118	102.657399	91.664044	85.483097	95.355243	97.509848	98.979109	108.143662	0.958082541	0.004800439	0.077922951	0.115543632
ENSG00000175756	AURKAIP1	82.896668	78.803792	72.332965	67.649076	66.813874	76.186785	78.051635	80.216729	82.373716	0.902234421	-0.015137008	0.172884253	0.115560373
ENSG00000115758	ODC1	64.256405	64.486508	64.898567	33.451321	29.419002	30.827433	67.693015	62.30203	66.311166	1.22E-59	-0.896829531	0.0685873	0.11560803
ENSG00000100605	ITPK1	3.769838	5.226615	6.323058	4.416866	4.607026	3.252249	5.98434	3.310598	8.139965	0.104694023	-0.182387067	0.335042443	0.115642808
ENSG00000089351	GRAMD1A	20.893312	19.530943	19.339531	18.382184	15.045596	16.167267	18.803269	20.218746	23.555291	0.085623984	-0.160175272	0.239529081	0.115834209
ENSG00000132773	TOE1	8.209259	8.015182	8.071887	7.191701	6.539569	6.411009	8.089657	7.658706	8.9796	0.305710434	-0.124633503	0.305317697	0.115852869
ENSG00000108963	DPH1	22.800994	21.902115	21.6156	19.255613	18.303603	19.991666	21.30192	23.22585	22.004639	0.810146693	-0.026351446	0.176079565	0.116030149
ENSG00000158710	TAGLN2	126.085759	132.259614	135.965544	101.690554	100.532038	102.394988	132.439343	134.571446	132.726722	0.00003	-0.224236404	0.089474927	0.116033356
ENSG00000141198	TOM1L1	26.458484	27.605499	26.808043	17.663553	19.48846	19.521455	25.004623	24.644917	27.247276	0.003055881	-0.224261335	0.180999766	0.116040974
ENSG00000198015	MRPL42	16.013946	11.522683	15.183321	13.047796	12.635581	10.279351	15.665587	26.853508	16.880272	0.185836222	-0.110897271	0.177943529	0.116067944
ENSG00000083807	SLC27A5	1.524631	2.164105	1.661474	2.059157	1.949138	2.695095	1.884052	2.240139	1.409063	0.04233047	0.553527439	0.733704469	0.116086799
ENSG00000181751	C5orf30	2.853592	3.603018	4.34023	3.453913	2.800219	2.762945	3.567092	3.61228	3.974469	0.650110032	-0.103640165	0.523879488	0.116137039
ENSG00000146457	WTAP	38.374324	38.780082	42.62395	41.809069	40.971802	39.712918	46.997349	39.361107	44.566958	0.000489769	0.226477965	0.16250096	0.116248449
ENSG00000140694	PARN	14.808228	14.957658	14.666281	12.34921	12.57644	12.934499	15.174999	14.116018	16.111105	0.56872144	-0.055212645	0.166104858	0.116408391
ENSG00000138795	LEF1	1.108832	1.074366	0.896554	0.589727	0.765558	0.767126	1.080697	0.918861	1.05411	0.115412855	-0.456761459	0.677094738	0.11641582
ENSG00000137821	LRRC49	1.451594	1.652393	0.830444	0.674243	0.64532	0.794707	1.506088	1.460653	1.665805	0.000095	-0.931144269	0.623132799	0.116427986
ENSG00000181704	YIPF6	7.205889	7.331069	7.546713	6.776664	6.708039	6.519573	7.707786	7.030338	7.735971	0.982075579	0.002661771	0.155514713	0.116429389
ENSG00000173950	XXYLT1	21.380706	20.380112	21.046982	18.883229	18.206544	18.304351	21.461273	20.094087	21.929449	0.758720186	-0.029560334	0.135399487	0.116462086
ENSG00000205609	EIF3CL	70.783812	68.523768	70.30025	61.922152	51.323412	63.500517	75.604198	66.72687	70.864509	0.245838001	-0.099435926	0.091269433	0.116477244
ENSG00000124615	MOCS1	14.360726	13.949636	14.034003	9.16384	9.289482	10.029897	14.162223	14.322891	14.83678	1.45E-08	-0.44615229	0.168080679	0.116527446
ENSG00000131828	PDHA1	18.444624	19.10053	19.981837	18.519777	18.224235	16.866394	19.524518	19.778511	19.12604	0.539184982	0.056166824	0.159713231	0.116558178
ENSG00000099385	BCL7C	8.354806	8.759856	7.385109	5.72625	6.384215	6.211367	8.52272	8.581771	7.962772	0.042587072	-0.277518315	0.403872359	0.116571041
NewGene_4860	--	5.218999	5.083057	5.984804	5.60916	5.383236	5.632695	5.637068	5.487254	5.606892	0.09488738	0.183682882	0.315283312	0.116588783
ENSG00000167136	ENDOG	10.140418	10.320667	7.682724	7.452836	8.386198	8.353733	8.104811	9.7904	10.758518	0.741122866	-0.069756063	0.518012703	0.116604086
ENSG00000146828	SLC12A9	9.26598	9.610245	9.114591	10.178942	9.169077	9.324045	9.834689	9.500907	9.546482	0.045530936	0.1822672	0.218305227	0.116617498
ENSG00000143198	MGST3	32.959103	30.238102	31.326928	22.535485	23.0265	26.783927	30.722084	34.757011	29.913912	0.027148817	-0.232527248	0.302990505	0.116619584
ENSG00000099290	WASHC2A	15.083987	14.163756	14.61106	18.635182	17.318701	17.245225	15.057205	14.288383	15.343674	8.87E-13	0.445241958	0.141322659	0.116788929
ENSG00000197879	MYO1C	41.335469	40.345461	40.528397	30.492492	29.617174	30.9223	39.455019	39.407887	45.369912	2.89E-08	-0.279748164	0.08155144	0.11709832
ENSG00000100196	KDELR3	14.84446	15.273352	17.122024	13.138671	12.993985	12.688526	15.86954	15.666347	16.331336	0.21670228	-0.135813376	0.267282995	0.117185907
ENSG00000151491	EPS8	50.126171	50.31655	53.695887	43.771994	46.711376	44.926278	54.441587	47.987751	54.969765	0.403076126	-0.061170064	0.078943969	0.117201474
ENSG00000148843	PDCD11	15.09958	14.91964	14.594674	13.773906	12.990652	12.870999	14.396803	15.437677	15.788435	0.699143614	-0.03179925	0.110652494	0.117282089
ENSG00000196704	AMZ2	13.699764	15.459953	16.195102	13.659943	11.908299	11.270345	16.095039	11.177703	11.597919	0.294993062	0.121393979	0.298601708	0.117319382
ENSG00000087302	RTRAF	11.478089	11.742513	12.925205	11.245158	10.11824	16.522916	12.963042	12.64236	12.108884	0.648099328	-0.043769854	0.157221062	0.117733107
ENSG00000135637	CCDC142	2.612835	2.914227	2.762516	3.10461	2.595145	3.752768	2.717288	2.103496	3.532181	0.098618042	0.254619435	0.545565966	0.117744342
ENSG00000107872	FBXL15	3.379171	2.750785	2.099008	2.689299	2.320056	2.936271	2.494876	3.074313	3.233169	0.853136186	0.052152997	0.61002878	0.117808417
ENSG00000103254	FAM173A	7.995947	8.145677	7.287036	6.177634	6.987083	7.338205	8.151843	7.594081	8.33572	0.705115068	-0.080534555	0.479274959	0.117884381
ENSG00000180098	TRNAU1AP	5.421048	6.068696	6.155781	5.237839	5.621791	5.516948	5.804222	6.617543	5.471806	0.828857531	0.041430731	0.473396183	0.117903005
ENSG00000184508	HDDC3	9.803452	9.185393	6.681691	7.4704	7.58339	7.895131	7.86478	8.772986	7.9128	0.826643611	0.045146441	0.474844129	0.117917342
ENSG00000133142	TCEAL4	17.862964	16.752978	17.46812	16.13183	15.262151	18.365758	19.097328	18.377952	15.762806	0.678617031	0.05852361	0.364565373	0.118096976
ENSG00000127528	KLF2	1.240146	0.912413	0.625228	0.403352	0.590965	0.619355	0.836905	1.053637	0.926781	0.134591006	-0.616934361	0.763660445	0.118125582
ENSG00000091592	NLRP1	4.023712	3.94383	3.785146	3.365604	2.995902	2.574302	3.579156	3.861481	4.767129	0.0151081	-0.290975257	0.330971885	0.118134742
ENSG00000164221	CCDC112	2.119832	2.065203	2.180861	1.687088	1.233383	1.556397	2.5285	2.332314	1.475932	0.162291948	-0.313690574	0.646348447	0.118172061
NewGene_2171	--	7.160608	7.686161	7.652121	6.074204	5.624136	6.13984	7.417266	7.729645	7.748849	0.07596633	-0.187002744	0.265302327	0.118200414
ENSG00000132005	RFX1	2.483564	2.493703	2.640959	2.678193	2.171685	2.424696	2.524909	2.496981	2.831215	0.757476825	0.058211608	0.406523852	0.118359644
ENSG00000091640	SPAG7	35.398883	32.947733	32.42084	26.578881	26.344663	23.035078	32.557752	34.554957	35.671038	0.004473814	-0.264424104	0.222731781	0.118371649
ENSG00000117450	PRDX1	337.67049	347.176319	351.196437	293.590885	322.161035	310.0787	341.320273	345.801089	366.767645	0.892642181	-0.011626447	0.048170934	0.118613957
ENSG00000100526	CDKN3	26.122193	23.853018	25.668519	21.734223	17.666622	18.5641	25.388362	25.61595	26.790612	0.040671672	-0.240541619	0.280503834	0.118615399
ENSG00000184887	BTBD6	8.505933	8.114632	8.001664	6.796285	6.042505	7.299593	7.666098	8.280137	9.192752	0.273776032	-0.144890306	0.329608054	0.118627256
NewGene_1666	--	0.644461	0.494853	0.513223	0.435929	0.510206	0.419457	0.535867	0.603568	0.538834	0.778685946	-0.121022271	0.734079594	0.118663232
ENSG00000180817	PPA1	74.658098	75.448297	84.546005	121.059446	118.122628	118.534061	79.711877	78.014052	81.017578	5.75E-37	0.755788485	0.131273439	0.118675087
ENSG00000142186	SCYL1	30.320121	30.466772	30.455329	33.225974	33.192387	33.376568	29.770821	28.783866	34.682326	0.00000165	0.27705587	0.128944414	0.118677239
ENSG00000100911	PSME2	45.852274	44.802696	42.756209	261.683701	258.509166	256.513861	44.741459	45.594841	46.831886	0	2.67713628	0.129474764	0.118781383
ENSG00000012174	MBTPS2	5.900294	6.563187	6.767004	6.598109	6.671116	6.511539	6.622433	6.073942	6.903726	0.051635181	0.188182106	0.253552342	0.118859337
ENSG00000089639	GMIP	3.041811	2.974984	2.621452	2.258462	2.422532	2.5429	2.91963	2.555173	3.32756	0.52266686	-0.116017778	0.459763701	0.118876437
ENSG00000154640	BTG3	11.598379	11.741348	11.944916	10.095738	10.377002	10.41241	11.96597	11.304712	12.602041	0.768046031	-0.043778218	0.30489404	0.118923336
ENSG00000141736	ERBB2	12.210116	10.38271	9.738014	7.513352	6.651998	7.264333	10.124755	10.29759	12.920761	0.000000293	-0.464421763	0.289969013	0.119162335
ENSG00000178741	COX5A	97.583174	102.989122	104.224559	90.292579	92.931582	88.414599	109.601937	111.408882	95.390719	0.644191266	-0.037498393	0.191930136	0.119225617
ENSG00000138385	SSB	80.285981	94.219367	97.47691	78.399155	77.197612	82.17965	91.383856	93.479569	92.896898	0.608406962	-0.050154373	0.168080679	0.119335557
ENSG00000088833	NSFL1C	31.571998	22.532946	24.686005	26.368834	23.8337	24.105717	29.326656	30.409885	28.544881	0.950533099	-0.006547425	0.139293384	0.119364351
ENSG00000283189	AC104452.1	1.281642	1.639565	1.407574	1.837651	0.770963	0.726565	1.974091	1.729712	0.664619	0.728234958	-0.235748662	0.854955032	0.119402019
ENSG00000104131	EIF3J	52.171212	55.969155	60.338072	54.903486	53.641036	53.298318	57.470935	55.164554	58.891433	0.340611885	0.075031927	0.11710068	0.119417418
ENSG00000164144	ARFIP1	11.939659	13.255989	13.701203	12.282327	13.148834	11.754763	13.390442	12.904909	14.189981	0.5018875	0.072529563	0.213990276	0.1194208
ENSG00000214941	ZSWIM7	5.147988	5.366773	5.6045	4.262818	3.976838	3.606833	5.541903	5.234761	4.612362	0.270360615	-0.204448872	0.512198389	0.11960563
ENSG00000183889	AC138969.1	6.533549	9.808349	8.267015	13.228976	5.953455	7.820078	8.059487	6.723292	8.433255	0.274842228	0.219232083	0.474230143	0.119632825
ENSG00000173064	HECTD4	3.369631	2.771039	3.063677	3.410635	2.882529	2.964283	3.097426	2.854523	3.664525	0.203410865	0.141752983	0.304612328	0.119663499
ENSG00000137168	PPIL1	70.706406	72.238121	66.338722	65.098953	61.560329	61.666264	68.245903	68.872864	76.276955	0.956046964	-0.005398682	0.096986943	0.119684512
ENSG00000035499	DEPDC1B	21.583512	24.455802	23.808982	18.365241	16.657845	17.398601	22.535586	23.07694	25.143611	0.000306927	-0.283787325	0.165304573	0.11979843
ENSG00000130294	KIF1A	0.190683	0.214515	0.210681	0.147882	0.189146	0.160836	0.187363	0.192441	0.249413	0.705044721	-0.15554535	0.728112428	0.119838239
ENSG00000179528	LBX2	2.75224	3.498599	1.775354	3.603136	4.01849	3.334704	3.084994	2.578433	2.721551	0.01377222	0.612754796	0.688237967	0.119853974
ENSG00000172009	THOP1	27.330819	27.804369	24.59275	24.687995	24.230038	26.404437	26.382683	27.506951	31.568867	0.542009052	0.058514616	0.191943074	0.119914673
ENSG00000158290	CUL4B	18.252139	18.42638	18.876999	17.610751	19.052542	18.354419	19.141076	17.607083	19.780314	0.055672284	0.135311567	0.087505619	0.119936654
ENSG00000203666	EFCAB2	11.858866	12.153213	12.898816	11.673525	9.710045	11.310598	9.099267	11.904308	10.559936	0.645930406	0.067151556	0.394243709	0.11999436
ENSG00000126787	DLGAP5	22.786131	24.775891	24.087539	15.746957	13.844522	14.146301	23.279	24.07816	25.522786	5.39E-16	-0.565467648	0.124950534	0.120071871
ENSG00000148985	PGAP2	24.085269	25.976849	26.411282	19.14839	18.370934	18.939167	25.026858	26.441835	26.485519	0.001676616	-0.252172806	0.153112179	0.120114374

Gene ID	Gene name	Ctrl-1 FPKM	Ctrl-2 FPKM	Ctrl-3 FPKM	IFNγ-1 FPKM	IFNγ-2 FPKM	IFNγ-3 FPKM	Sora-1 FPKM	Sora-2 FPKM	Sora-3 FPKM	Ctrl vs IFNγ DESeq2 FDR	Ctrl vs IFNγ DESeq2 log2FC	Ctrl vs Sora DESeq2 FDR	Ctrl vs Sora DESeq2 log2FC
ENSG00000157483	MYO1E	6.58748	6.525361	5.999481	4.052092	4.307863	3.961359	6.397812	6.456363	6.644248	1.96E-10	-0.483026506	0.146839589	0.120144746
ENSG00000150527	MIA2	28.85931	31.155708	30.456012	34.20351	34.848536	30.641229	30.314364	28.718997	31.433433	4.58E-08	0.302888097	0.074756164	0.120201148
ENSG00000125821	DTD1	18.522822	20.585198	18.836185	16.11986	15.894637	14.874038	20.051369	17.834804	20.801014	0.231931965	-0.121846148	0.21523124	0.120253136
ENSG00000157388	CACNA1D	1.06531	0.954455	1.077249	0.388911	0.538365	0.331148	1.259556	0.997145	1.105614	3.72E-08	-1.091852139	0.454921641	0.120599003
ENSG00000102890	ELMO3	0.601654	0.690276	0.792889	0.750624	0.715083	0.729737	0.497746	0.757013	0.867192	0.589588716	0.206542058	0.753655099	0.120629765
ENSG00000076108	BAZ2A	20.19447	18.103603	19.858366	23.307877	23.041568	22.94992	15.5511	15.532751	18.987648	1.52E-34	0.693149387	0.171215306	0.120720304
ENSG00000106853	PTGR1	0.768327	0.709131	0.833488	0.481201	0.470164	0.794024	1.058167	0.758589	0.683202	0.786075536	-0.154344572	0.801925485	0.120788892
ENSG00000147669	POLR2K	45.593712	47.913358	57.553177	42.492094	40.170899	42.00725	52.586102	52.979919	47.202253	0.214729796	-0.132812739	0.301561847	0.120890253
ENSG00000221829	FANCG	15.259845	15.24663	15.751559	11.847391	10.070834	11.983502	15.375675	15.428281	16.696348	0.000522598	-0.310402879	0.160496225	0.120895901
ENSG00000100395	L3MBTL2	12.466594	11.896166	11.733039	11.131398	11.193075	11.137399	11.885802	11.093128	13.977545	0.688566223	0.042098636	0.223911943	0.12096531
ENSG00000132589	FLOT2	44.68359	41.368697	38.555967	42.49123	41.300302	43.339253	41.710188	40.61334	44.789076	0.014630862	0.176470881	0.125849536	0.120965437
ENSG00000120457	KCNJ5	3.275209	3.091132	3.284514	2.152652	2.345113	2.134359	2.806486	3.108233	3.647865	0.001839459	-0.361675309	0.366787282	0.120973207
ENSG00000147202	DIAPH2	3.022485	3.413779	2.770503	2.854923	2.247499	2.272684	2.869985	3.916815	3.535325	0.113924609	-0.195233324	0.327529213	0.12099843
ENSG00000100216	TOMM22	31.23741	31.820421	32.249771	27.809748	29.006105	30.871658	33.25724	34.288628	29.412287	0.780303356	0.028445644	0.2172327	0.121126988
ENSG00000164244	PRRC1	11.112507	11.781577	12.956527	12.809929	13.103839	13.22528	12.259676	11.297945	12.613263	0.000295063	0.274967807	0.170256389	0.121127357
ENSG00000102886	GDPD3	2.157424	2.141084	2.166296	2.04752	2.60936	2.441634	2.350844	1.7382	2.501943	0.279029025	0.287469799	0.661529513	0.121139917
ENSG00000103066	PLA2G15	4.67364	4.77563	4.735829	3.383583	2.734123	3.287967	5.255162	4.956378	5.109819	0.000876924	-0.452319652	0.375681874	0.12123971
ENSG00000142207	URB1	4.877317	5.036586	4.843697	4.182498	3.696006	3.897894	5.033661	4.355937	5.705772	0.02174997	-0.17872266	0.204997615	0.12138578
ENSG00000196072	BLOC1S2	16.566414	19.691469	19.271615	17.139887	17.02553	15.538254	17.536932	19.28461	19.354672	0.990509047	-0.002063708	0.29844769	0.121390158
ENSG00000198805	PNP	50.077201	47.046393	49.348155	39.989757	39.358311	40.243476	45.760263	52.2881	51.819487	0.036689854	-0.146694164	0.166649015	0.121413641
ENSG00000163032	VSNL1	9.597371	10.179715	9.886923	9.443419	8.289836	6.969739	11.929021	9.494035	9.987664	0.372538841	-0.124997494	0.347718263	0.121421389
ENSG00000157181	ODR4	14.610897	16.3596	17.98011	18.581836	15.709693	16.752923	17.730111	16.23839	16.593552	0.016654232	0.202724021	0.157812229	0.121540821
ENSG00000204084	INPP5B	4.331388	4.462457	4.370789	4.505588	4.004495	4.14082	4.385693	4.038254	5.184978	0.493706792	0.090304658	0.319132961	0.121556526
ENSG00000174886	NDUFA11	141.397849	135.881031	120.59751	109.020832	116.245312	120.297683	145.225784	153.528874	151.035581	0.838520968	-0.022431915	0.121723258	0.121586654
ENSG00000113141	IK	41.59705	43.399944	43.895935	42.801312	39.191748	38.924604	48.006383	43.010272	48.32643	0.92020154	0.00971742	0.085216691	0.121648046
ENSG00000196152	ZNF79	1.760096	1.532718	1.96545	1.937314	1.273238	1.833509	1.899425	1.514796	2.077807	0.87441714	0.053389892	0.627167192	0.121662237
ENSG00000096070	BRPF3	29.01739	24.883143	26.550537	28.103837	25.528135	25.126549	30.009807	25.05042	29.169163	0.579457681	0.046717902	0.133280514	0.121687441
ENSG00000099341	PSMD8	95.229492	85.248134	87.409563	77.205393	70.771325	82.935558	89.691217	93.351337	93.22018	0.421613268	-0.065889833	0.094748949	0.121892324
ENSG00000206053	JPT2	44.636755	45.071188	45.561057	39.656691	39.515156	37.999902	44.664047	45.555812	48.009162	0.262383187	-0.07108337	0.054722951	0.121923152
ENSG00000110955	ATP5F1B	414.050772	405.136802	394.487642	354.086584	360.930578	370.216894	403.037701	420.160822	413.81187	0.836659563	-0.015111154	0.053595399	0.121943254
ENSG00000213923	CSNK1E	54.281918	47.283154	47.600719	36.987669	38.080429	36.420446	52.840701	49.201676	51.679039	0.00000915	-0.271456793	0.080286931	0.121978286
ENSG00000141030	COPS3	72.317812	77.601617	83.63285	76.225915	75.956729	66.755895	80.204899	81.788836	80.3011	0.96728994	0.004802486	0.097364816	0.121989123
ENSG00000071462	BUD23	38.015413	41.358051	42.067271	38.587689	33.820478	38.41397	39.949586	41.589053	41.855671	0.907862454	0.014331544	0.166302622	0.122011842
ENSG00000125454	SLC25A19	14.505675	10.101997	10.876525	8.401041	8.601482	8.759918	11.027129	11.511676	13.518262	0.019077948	-0.305720645	0.382316	0.122015706
ENSG00000056097	ZFR	36.230838	35.33477	35.626055	34.451213	33.964771	33.954503	38.099797	33.416161	37.851909	0.163171623	0.082450474	0.069532832	0.122181638
ENSG00000116497	S100PBP	6.056723	5.857572	6.619689	6.091563	5.565031	5.922649	6.580075	5.801147	6.612199	0.37634884	0.10424995	0.275901745	0.122258705
ENSG00000158747	NBL1	3.427567	4.215491	3.909973	2.239089	2.389631	2.928248	3.761729	4.038879	3.974023	0.010093253	-0.462046588	0.476193667	0.122336331
ENSG00000011485	PPP5C	40.55903	36.561326	40.259428	31.335821	29.502544	30.520911	37.936111	38.667691	43.070969	0.00292884	-0.20867318	0.126588941	0.122342839
ENSG00000136982	DSCC1	7.283204	7.120189	7.069827	4.316606	3.993816	4.581661	6.702873	7.34216	7.882902	0.0000024	-0.587553844	0.320610206	0.122388364
ENSG00000137500	CCDC90B	10.245037	7.781256	9.672043	6.928917	7.097171	7.324662	9.05926	9.18191	9.064923	0.763499535	-0.042920983	0.278743679	0.122447551
ENSG00000132824	SERINC3	27.761492	26.805756	26.930379	27.653036	28.057709	28.971063	26.900129	26.496016	29.514907	0.000461578	0.202513278	0.064249138	0.122567464
ENSG00000004142	POLDIP2	78.877571	78.364151	75.053856	72.356842	69.09642	74.613121	80.575923	76.507743	80.426964	0.561531714	0.04296833	0.05409989	0.122648744
ENSG00000100934	SEC23A	35.810084	38.886498	38.471598	41.187971	43.446689	41.645558	39.165224	36.115765	40.315761	0.00000016	0.305951752	0.071936061	0.122803928
ENSG00000109332	UBE2D3	80.449823	77.038243	86.330631	89.305687	80.453712	81.477872	87.185201	82.633585	86.292038	0.006726834	0.141284605	0.040545786	0.1229023
ENSG00000164414	SLC35A1	10.179042	11.273833	9.821281	8.715729	9.048829	7.609443	10.448536	10.743047	10.613679	0.252336282	-0.151962358	0.297193686	0.122939124
ENSG00000124523	SIRT5	11.071444	11.5908	11.218826	10.19707	10.534633	10.072377	10.982902	11.17727	13.179399	0.602420238	0.055345319	0.169087081	0.122941072
ENSG00000257923	CUX1	17.506277	15.495294	15.780299	15.717808	15.266988	15.843086	19.815208	20.19773	23.692047	0.857097384	0.017864771	0.140602547	0.122956837
ENSG00000204642	HLA-F	2.928556	4.264466	3.799333	13.34213	10.437534	14.626072	4.565288	3.138153	4.548378	1.01E-45	1.887997041	0.568137629	0.123050395
ENSG00000156603	MED19	12.250434	13.494546	12.441327	11.677006	12.440426	13.240097	13.153959	12.969854	12.745093	0.344151725	0.129049381	0.325460647	0.123092122
ENSG00000171202	TMEM126A	17.855863	19.369267	18.909573	21.364579	17.140439	19.036882	18.925912	19.015662	19.399086	0.177428927	0.18504174	0.337933758	0.123139091
ENSG00000122952	ZWINT	41.196229	41.283906	41.341201	32.914099	29.344006	31.273592	39.294082	43.643612	40.220025	0.000483632	-0.233959094	0.153112179	0.123169362
ENSG00000113810	SMC4	38.865639	42.238684	46.564998	38.414998	35.143425	35.163127	46.316824	40.422579	44.329022	0.279764192	-0.092052821	0.169253393	0.123323906
ENSG00000221821	C6orf226	2.266273	2.808254	2.223759	1.851508	2.841466	2.53414	2.022926	2.876811	2.542619	0.770817157	0.143433983	0.767994848	0.123366405
ENSG00000138162	TACC2	3.158085	2.646144	3.02769	2.062572	2.234299	1.730941	2.106556	2.983651	4.109679	0.011280911	-0.33297902	0.39064188	0.123387139
ENSG00000182551	ADI1	34.300276	35.30929	35.958207	29.332083	28.146448	28.917712	36.086017	36.377971	36.05007	0.054814262	-0.130851026	0.100942417	0.123418237
ENSG00000140553	UNC45A	19.48585	18.32667	16.649045	14.502696	14.952503	15.01584	17.710059	17.809983	20.282572	0.093830078	-0.150661764	0.184734813	0.123421739
ENSG00000104881	PPP1R13L	3.341555	3.072457	3.294178	1.513853	1.651909	1.679942	3.106844	2.919331	3.746662	6.94E-08	-0.815036834	0.43618677	0.123715192
ENSG00000163634	THOC7	27.264513	31.978272	34.569866	30.754045	30.492914	30.850563	32.138998	33.296215	29.652523	0.336879028	0.111173365	0.302297744	0.123799666
ENSG00000172086	KRCC1	12.200929	11.997108	10.728383	9.941823	11.831129	10.537416	11.73651	11.938208	12.029863	0.827011529	0.035542545	0.281315517	0.123868559
ENSG00000182093	WRB	21.541435	21.613284	20.976256	14.952443	16.899041	15.533574	21.281803	20.392858	23.197944	0.002672824	-0.279668891	0.170831626	0.123896466
ENSG00000150477	KIAA1328	1.071303	0.865363	1.137004	0.90022	1.015744	1.099816	1.133114	1.05956	0.328986	0.635274741	0.167630633	0.737724838	0.123923964
ENSG00000156026	MCU	12.002712	13.187696	11.560897	8.9231	8.337926	7.635489	13.306429	12.045636	12.275119	0.0000061	-0.413511749	0.213471751	0.123943985
ENSG00000146576	C7orf26	14.611997	13.678406	12.516137	10.494833	9.337242	11.018751	12.938512	13.077769	15.16566	0.031771673	-0.235603962	0.25837972	0.1240478
ENSG00000128626	MRPS12	30.460971	32.354145	29.345036	24.548683	25.940424	25.414201	27.608338	33.647084	34.905043	0.155610801	-0.139832231	0.257542171	0.124340135
ENSG00000153976	HS3ST3A1	1.50253	1.221243	1.400838	1.730487	1.625063	1.670231	1.100568	1.42148	1.705858	0.048060095	0.432315392	0.647615958	0.124429315
ENSG00000105443	CYTH2	18.06113	16.872279	16.775627	13.242261	12.455481	12.127524	17.635195	19.299464	19.507372	0.00000242	-0.33422522	0.11292823	0.124454678
ENSG00000175183	CSRP2	27.334204	29.45889	28.335347	15.376787	18.958661	18.149663	30.842266	29.794991	27.096189	1.73E-08	-0.567276941	0.260276367	0.124517166
ENSG00000206562	METTL6	13.153871	13.836533	13.713469	13.195468	10.847544	12.755444	13.533134	13.947672	13.281897	0.798889633	0.039078829	0.295822382	0.124518258
ENSG00000122359	ANXA11	29.189845	21.127735	29.939168	18.126644	21.274213	20.741895	20.19326	27.726011	21.526572	0.0000437	-0.268456929	0.080488987	0.124543685
ENSG00000104450	SPAG1	4.642914	4.925098	5.770626	3.186523	3.654693	3.119287	5.098203	4.578577	5.988842	0.0000316	-0.513252759	0.357163962	0.124556574
ENSG00000189043	NDUFA4	98.57476	98.174316	91.848808	85.493477	87.488846	88.0476	97.90139	105.967232	90.455429	0.977419026	0.002718941	0.168604412	0.124579173
ENSG00000146007	ZMAT2	29.070733	28.144654	27.436099	25.700893	29.094538	25.398909	29.94002	29.635197	30.419807	0.797847739	0.027747061	0.207336975	0.124641518
ENSG00000151414	NEK7	8.747621	10.405656	9.0698	9.469819	9.204841	9.231082	10.105111	9.135045	9.59665	0.205413928	0.126806278	0.222794906	0.124653475
ENSG00000168152	THAP9	1.747623	2.404875	1.840313	1.931247	1.89021	1.894994	2.280774	2.308375	1.85697	0.552909521	0.121369148	0.52221031	0.12473191

Gene ID	Gene name	Ctrl-1 FPKM	Ctrl-2 FPKM	Ctrl-3 FPKM	IFNγ-1 FPKM	IFNγ-2 FPKM	IFNγ-3 FPKM	Sora-1 FPKM	Sora-2 FPKM	Sora-3 FPKM	Ctrl vs IFNγ DESeq2 FDR	Ctrl vs IFNγ DESeq2 log2FC	Ctrl vs Sora DESeq2 FDR	Ctrl vs Sora DESeq2 log2FC
ENSG00000205978	NYNRIN	1.284048	0.884725	0.810773	1.246053	1.24813	1.227608	1.192808	1.009831	0.836733	0.010327047	0.467648682	0.595397643	0.124923536
ENSG00000113013	HSPA9	165.28208	160.708576	156.028066	147.99348	154.680635	138.033027	170.160389	160.44036	179.996704	0.682336068	-0.027245266	0.027849796	0.125007279
ENSG00000184990	SIVA1	27.983156	30.239589	25.443287	24.664017	26.355089	30.702364	28.706921	29.039099	28.355747	0.195969435	0.161440518	0.284428447	0.125017537
ENSG00000214113	LYRM4	10.630485	11.824918	11.190063	7.560177	9.410989	7.65808	13.673773	11.473871	11.326545	0.00000903	-0.329770753	0.130458823	0.125241399
ENSG00000036054	TBC1D23	4.880303	5.650056	6.397559	6.748532	7.223016	6.470132	5.882978	5.397281	5.88299	0.000147568	0.428665785	0.337933758	0.125281194
ENSG00000242372	EIF6	119.201748	121.414772	111.294364	111.842285	112.799682	119.468014	118.090732	119.252109	121.065895	0.10625703	0.112335207	0.074756164	0.125365648
ENSG00000158062	UBXN11	4.625338	4.136574	3.832198	2.810961	2.486929	3.056184	3.804312	4.134301	4.146923	0.005541856	-0.529478	0.512383829	0.125367628
ENSG00000048544	MRPS10	29.444017	32.664818	34.230774	31.005911	28.405329	30.779444	34.146935	30.787373	33.652454	0.598670031	0.052532418	0.147597338	0.125496367
ENSG00000132341	RAN	231.556826	250.747254	224.720138	190.296305	204.506608	189.719844	244.849745	241.310099	232.392588	0.043250558	-0.110037603	0.039387001	0.125597079
ENSG00000123179	EBPL	18.169375	17.690514	18.368869	19.389323	21.23076	20.627492	19.419409	17.840474	18.005393	0.049797445	0.229918872	0.301186799	0.125616118
ENSG00000145365	TIFA	3.483644	3.763907	3.55649	6.300783	6.176749	6.661027	3.707427	3.68494	3.519428	3.13E-28	0.988476601	0.321578953	0.125706605
ENSG00000165934	CPSF2	6.328938	6.972443	6.526955	6.581786	6.363084	6.355872	6.952646	6.391462	7.057845	0.093352696	0.116032408	0.086187104	0.125718078
ENSG00000138430	OLA1	28.324167	30.423166	33.941741	31.427013	31.193126	29.827993	41.083405	31.23152	36.205875	0.984331945	-0.002182	0.117093982	0.125748246
ENSG00000163808	KIF15	5.64899	6.244848	5.976724	4.733392	3.768025	4.210936	6.101944	5.486327	6.563406	0.001230531	-0.342167635	0.221700315	0.125785961
ENSG00000119760	SUPT7L	12.440862	12.342227	11.672543	10.21451	10.671563	10.205173	12.773962	11.737308	12.636804	0.828432532	-0.02352418	0.131603368	0.125795552
ENSG00000089220	PEBP1	254.94783	238.042831	240.980606	223.496902	246.248581	231.211823	243.700668	246.736984	260.752411	0.252161379	0.081526049	0.041592939	0.125874665
ENSG00000151502	VPS26B	11.728903	10.524653	10.08588	9.381975	9.902526	10.623455	10.55545	10.663995	11.6706	0.637413551	0.056479521	0.172884253	0.125898346
ENSG00000196208	GREB1	11.856565	11.017475	10.51457	9.233395	8.661418	8.865766	11.091229	10.275691	13.018968	0.045550656	-0.145927547	0.160402202	0.125993698
ENSG00000144559	TAMM41	5.593676	4.478068	5.373125	4.317045	4.106291	4.014117	4.596461	5.192121	5.502475	0.669440841	-0.093742856	0.498398589	0.126009259
NewGene_1680	--	1.434606	1.592753	1.38751	1.477048	1.301635	1.434233	0.787163	1.394367	2.370432	0.856847974	0.073175576	0.766942439	0.126149297
ENSG00000158402	CDC25C	11.926626	12.202423	10.908179	8.114853	9.858944	10.88594	13.12388	12.06304	11.686782	0.318916362	-0.133013463	0.267448572	0.126169384
ENSG00000172893	DHCR7	132.018977	115.841445	109.524388	138.031108	145.989217	146.282237	116.150166	119.234342	131.601568	9.17E-09	0.389102265	0.119371153	0.126196777
ENSG00000182504	CEP97	1.238716	1.374072	1.383987	1.160927	1.039707	1.001903	1.359301	1.259925	1.487145	0.199650365	-0.195530256	0.381151934	0.126241946
ENSG00000162972	MAIP1	22.998242	21.074443	22.802494	23.84946	23.048214	22.887325	25.213603	22.423932	20.829368	0.022243913	0.194882992	0.245462447	0.126292237
ENSG00000183814	LIN9	8.013655	9.420826	8.810742	7.719671	7.000074	6.835467	9.561058	8.350911	9.483723	0.160093248	-0.152896767	0.240792802	0.126328091
ENSG00000124920	MYRF	40.551912	37.451063	38.765701	42.044401	40.31116	41.627791	39.381613	36.511429	43.955225	0.0000121	0.232246046	0.079325018	0.126352711
ENSG00000006744	ELAC2	48.732483	46.676764	48.842614	47.200431	42.418867	48.210129	46.528681	47.476487	52.894453	0.579301684	0.042609047	0.057542254	0.126379382
ENSG00000125485	DDX31	9.019258	7.890104	9.248307	7.441059	6.033237	5.926721	8.83705	7.914792	9.616349	0.00214178	-0.306605971	0.183783399	0.126588429
ENSG00000151503	NCAPD3	13.816937	13.881137	13.659482	11.395988	10.734245	11.235134	13.274216	13.895495	15.867456	0.005463768	-0.190465795	0.118568079	0.126589186
ENSG00000173085	COQ2	6.059037	5.578839	5.691543	4.583353	4.963562	4.676679	5.800734	6.107419	5.949364	0.406671255	-0.14212394	0.406674696	0.126601116
ENSG00000168710	AHCYL1	28.91388	29.638087	30.060497	29.158203	29.151197	29.125033	30.185886	29.387283	30.535213	0.018248076	0.138483227	0.052303486	0.12662753
ENSG00000158856	DMTN	12.855622	10.301794	9.844356	6.425433	7.232967	5.963446	10.475821	11.538572	11.868458	0.00000186	-0.581521351	0.316762248	0.126657431
ENSG00000238083	LRRC37A2	4.474297	4.606067	4.623058	4.289417	3.980431	2.953762	3.888623	4.627095	5.275244	0.379118644	-0.120782689	0.262138364	0.126823427
ENSG00000116791	CRYZ	16.14599	17.677477	16.291515	13.61055	14.517959	14.004776	17.722442	15.871259	19.556566	0.226554404	-0.116552629	0.173503099	0.127084037
ENSG00000127399	LRRC61	46.148836	40.915839	41.595774	52.791727	61.993636	61.083627	39.546137	42.673735	49.767685	2.11E-14	0.598349111	0.181373961	0.127233126
ENSG00000047230	CTPS2	14.99182	13.916495	14.056333	11.245959	10.730947	11.003442	15.218519	14.95131	14.746353	0.000255944	-0.26224043	0.126570771	0.127245402
NewGene_6308	--	20.504853	18.546568	16.960429	27.007976	25.981486	25.63192	19.834523	19.029028	18.467464	8.63E-21	0.627650287	0.16463616	0.127262531
ENSG00000134108	ARL8B	58.430056	61.234486	65.277896	59.350514	57.788351	58.390696	64.182292	58.504372	66.969551	0.276228736	0.075176506	0.075073119	0.12742
ENSG00000027847	B4GALT7	7.771162	7.191084	6.532187	7.362044	6.061746	7.360379	6.615981	7.20904	8.254126	0.802417185	0.047002695	0.391104116	0.127429836
ENSG00000119004	CYP20A1	6.298088	6.638539	6.508453	6.18595	5.599683	6.333813	6.730344	6.094011	8.155052	0.393888096	0.120803515	0.383898345	0.127531228
ENSG00000137700	SLC37A4	23.548805	24.933762	20.30698	31.547271	26.932846	31.922776	24.817553	23.90477	24.479311	2.53E-16	0.562733573	0.142919318	0.127539
ENSG00000165028	NIPSNAP3B	1.192735	1.329707	1.486069	1.166003	1.073235	1.358378	1.291695	1.03519	1.4268	0.889945453	0.061596589	0.710717891	0.127542122
ENSG00000133315	MACROD1	15.07118	13.700072	13.041201	12.014469	11.442606	11.792468	12.640656	13.043171	17.322643	0.475430489	-0.100248457	0.38168624	0.127565167
ENSG00000105662	CRTC1	3.484173	4.473258	3.639806	3.750131	3.177431	2.716584	5.859963	5.214033	5.285969	0.50662851	-0.099588185	0.336781599	0.12784391
ENSG00000075218	GTSE1	15.701443	16.334045	15.566247	12.522622	11.95217	13.053946	16.84778	15.185525	16.76782	0.012777856	-0.195021781	0.117104875	0.12784753
ENSG00000142192	APP	400.800326	343.246444	360.612994	313.895257	341.76723	316.076329	341.09185	360.647844	421.547378	0.643627594	-0.037407817	0.093126811	0.127849532
ENSG00000137834	SMAD6	1.230235	1.253224	1.372987	0.814648	1.172372	0.931144	1.476028	1.329014	1.194195	0.221879434	-0.283645346	0.563707549	0.127856605
ENSG00000274276	CBSL	39.149016	35.963076	38.360531	43.691575	39.524765	37.295229	46.062518	26.613997	41.608552	0.000235982	0.245191518	0.677545135	0.127992246
ENSG00000148187	MRRF	13.229315	11.288888	11.669742	12.22268	9.438752	10.232067	13.531321	13.252918	13.681957	0.706706834	-0.039845612	0.12713525	0.128091062
ENSG00000168374	ARF4	116.907277	119.857699	126.692012	120.966907	127.786721	121.488684	127.14041	123.197253	128.838807	0.003257308	0.178657193	0.047369533	0.12811382
ENSG00000042445	RETSAT	18.056914	17.521851	16.188355	14.658567	13.857569	13.705491	17.836413	17.270425	18.042022	0.045610176	-0.169847233	0.134537262	0.12816696
ENSG00000085982	USP40	11.067315	10.132375	9.419096	9.375941	9.188339	9.030544	10.16123	9.92243	10.713073	0.958230986	-0.006842157	0.1500184	0.128304332
ENSG00000130787	HIP1R	9.083988	8.590327	8.198339	10.119535	10.250693	10.301443	8.381571	8.230183	9.958665	0.00000013	0.392646941	0.190213797	0.128359812
ENSG00000166439	RNF169	1.834964	1.983523	1.856259	2.085055	1.89548	1.912382	2.013661	1.862757	1.943012	0.096897882	0.200613342	0.304128694	0.128563478
ENSG00000171928	TVP23B	19.439273	20.857722	22.112085	22.962564	23.65505	23.051098	20.440263	21.420757	20.738301	0.0000264	0.333702961	0.162605169	0.12856475
ENSG00000074964	ARHGEF10L	25.060009	23.285897	20.389396	20.56361	20.23187	19.890657	21.618608	21.983624	26.979687	0.642427547	-0.051613272	0.223288596	0.128624518
ENSG00000197563	PIGN	3.923792	3.455907	4.14791	4.080973	3.79558	3.644061	4.117888	5.129542	4.238595	0.092132163	0.17624187	0.262305259	0.128759027
ENSG00000165898	ISCA2	5.090426	5.101522	4.693856	5.599821	6.17104	4.963435	5.139539	5.851302	4.151866	0.026608729	0.286346789	0.459951218	0.128786221
ENSG00000104884	ERCC2	14.660332	14.771503	13.860019	13.854816	12.524659	14.432014	14.899852	14.2144	17.624659	0.962932659	0.005447165	0.117199223	0.12880106
ENSG00000186166	CCDC84	7.515212	7.515821	8.689745	6.284792	4.625111	5.202011	8.43465	7.668971	8.038277	0.016311196	-0.400212606	0.407178906	0.128811303
ENSG00000138641	HERC3	1.036971	0.951278	0.781485	0.780488	1.009434	0.955722	0.986182	0.998409	0.994453	0.732050855	0.099554482	0.569309623	0.12881951
ENSG00000162959	MEMO1	10.386193	6.8667	8.762116	11.222432	5.848161	5.516375	15.51605	5.710593	6.547432	0.943839078	0.011768969	0.211223324	0.128828297
ENSG00000107798	LIPA	11.111598	10.610671	9.53293	14.817046	15.203697	14.638285	10.366203	11.23855	10.504068	4.64E-16	0.658053352	0.244197004	0.1289165
ENSG00000169592	INO80E	15.564197	14.506414	14.866263	13.364727	13.243364	12.719426	15.991979	16.136717	14.33629	0.874684317	-0.022193522	0.261229704	0.128923677
ENSG00000012211	PRICKLE3	1.514249	1.309747	1.568393	1.624021	1.336205	1.554084	1.478985	1.532521	1.563988	0.64925479	0.135756572	0.618101636	0.12893756
ENSG00000116016	EPAS1	37.849545	41.031651	42.466747	29.093456	29.8979	29.132313	41.350956	39.202644	43.974178	0.000000105	-0.313689818	0.062695893	0.128974225
ENSG00000185803	SLC52A2	19.763198	20.118997	18.326885	15.086055	15.649072	16.986855	19.064625	18.045176	21.156878	0.392278627	-0.093658374	0.190037239	0.12904647
ENSG00000213658	LAT	0.674813	0.858616	0.955864	1.047066	0.849421	0.847601	0.768448	0.897625	0.864712	0.546379589	0.262375904	0.757332598	0.129071698
ENSG00000090263	OGFOD1	8.783253	9.57139	9.690048	8.881914	7.887014	8.163293	9.609547	9.547113	9.655015	0.853136186	-0.022386241	0.143230267	0.129129047
ENSG00000182247	UBE2E2	10.942334	8.680562	8.575687	6.175556	8.168238	7.970629	9.927654	8.137745	10.118042	0.886141116	-0.024471516	0.274509199	0.129151535
ENSG00000115998	C2orf42	3.800393	3.617715	2.985117	3.404163	3.347618	3.144789	2.967888	3.027407	3.089492	0.526534955	0.131197175	0.465802993	0.129173321
ENSG00000175643	RMI2	16.009666	17.980841	16.469171	22.604956	21.111559	22.658283	17.154516	16.322645	18.035265	1.13E-11	0.571048774	0.220092764	0.129180038
ENSG00000156831	NSMCE2	18.935255	19.804019	19.287635	16.673583	16.243754	15.286079	20.304198	20.137018	19.130039	0.290906482	-0.122170495	0.280515708	0.129186915
ENSG00000112186	CAP2	17.034315	17.239918	19.598535	16.638034	16.516098	14.977405	17.587935	18.60773	19.506295	0.54328814	-0.064368898	0.157075468	0.129233847

Gene ID	Gene name	Ctrl-1 FPKM	Ctrl-2 FPKM	Ctrl-3 FPKM	IFNγ-1 FPKM	IFNγ-2 FPKM	IFNγ-3 FPKM	Sora-1 FPKM	Sora-2 FPKM	Sora-3 FPKM	Ctrl vs IFNγ DESeq2 FDR	Ctrl vs IFNγ DESeq2 log2FC	Ctrl vs Sora DESeq2 FDR	Ctrl vs Sora DESeq2 log2FC
ENSG00000196843	ARID5A	6.566899	5.648168	5.335543	5.949513	5.80291	5.860006	5.734895	6.104475	5.627568	0.236906965	0.171270391	0.372887609	0.129279692
ENSG00000175470	PPP2R2D	4.82097	5.002538	5.959951	4.769259	5.019843	3.858812	5.612524	5.754482	5.783934	0.014862092	-0.243236286	0.211136753	0.129505336
ENSG00000007341	ST7L	2.180154	2.119889	2.073305	2.074119	2.171209	2.124232	2.579477	2.209173	2.688717	0.860877732	0.038370827	0.432473992	0.129510661
ENSG00000197355	UAP1L1	26.340657	27.412683	26.081941	25.828184	26.124711	26.235655	28.662163	25.530682	28.100606	0.061563855	0.122406912	0.074476433	0.129527527
ENSG00000092208	GEMIN2	10.333093	11.727755	11.823475	7.773945	8.551305	8.34296	11.506841	12.39618	10.215726	0.004485923	-0.360745785	0.368728723	0.129756719
ENSG00000177169	ULK1	9.342987	8.940798	8.477754	7.96619	8.212604	7.899392	8.739486	7.96015	10.845851	0.964700899	-0.005492348	0.230495601	0.12977273
ENSG00000197982	C1orf122	15.600306	13.816894	13.418001	12.064193	10.821549	13.934001	13.909741	15.43438	15.337154	0.56091668	-0.091716369	0.299889898	0.129935001
ENSG00000118855	MFSD1	18.024658	18.509639	18.493458	17.182988	18.631961	16.589666	18.039768	16.644848	20.119612	0.170538048	0.131712377	0.159950021	0.12993885
ENSG00000124702	KLHDC3	65.615077	68.098926	65.494592	53.124743	53.278296	53.739189	64.65556	68.811635	70.935478	0.004009484	-0.167727452	0.063968525	0.129961238
ENSG00000109919	MTCH2	97.30309	109.801543	111.330152	92.846499	96.399069	97.911469	110.401376	120.650515	111.130457	0.305083664	-0.063046069	0.05997317	0.129976468
ENSG00000149809	TM7SF2	66.176154	57.080702	49.742679	99.436804	100.533145	102.60907	58.751828	57.270105	54.054684	5.97E-28	1.00049371	0.276445058	0.12999083
ENSG00000154889	MPPE1	3.020776	3.853043	3.665217	8.564189	7.238544	7.867837	3.799267	3.337606	3.396093	3.38E-33	1.312922738	0.430559456	0.13003518
ENSG00000213719	CLIC1	615.517938	573.6392	532.417267	572.279979	579.468614	544.530371	567.656618	588.257095	612.274149	0.058700218	0.127501149	0.069649715	0.130080055
ENSG00000080603	SRCAP	13.044161	10.551666	9.267718	13.984619	10.825918	7.497164	9.534949	10.911467	13.428304	0.961297182	0.032789039	0.67236772	0.130132858
ENSG00000167508	MVD	47.110889	42.606742	38.191191	53.965772	60.290606	61.644242	40.786098	43.564861	49.43626	1.16E-12	0.582360075	0.196033456	0.130167183
ENSG00000127452	FBXL12	20.025663	18.851064	18.482838	19.081304	20.245421	19.620739	18.182187	19.47694	19.855943	0.035847957	0.190981182	0.176658251	0.13033514
ENSG00000133943	DGLUCY	2.964312	2.579665	2.529783	7.467818	6.293869	7.286383	2.998283	2.611024	2.518087	3.57E-43	1.609117393	0.478669065	0.13039954
ENSG00000253626	EIF5AL1	4.229702	3.875623	4.197696	4.424997	4.470145	4.17637	4.031283	4.599228	3.966276	0.042658675	0.234174613	0.339604488	0.13053129
ENSG00000284753	EEF1AKMT4	7.30963	7.140547	7.034638	5.808327	5.698534	6.16388	7.992247	6.848783	7.218429	0.356620114	-0.133499127	0.333610353	0.130588111
ENSG00000166333	ILK	70.858057	67.007084	69.408377	54.001331	59.057125	52.693973	69.623215	69.875619	72.205691	0.008129254	-0.180498591	0.051215292	0.130593554
ENSG00000028310	BRD9	17.003073	16.565425	16.553931	14.748032	13.233054	12.36504	18.649264	16.158134	17.534857	0.04200487	-0.178845143	0.10702183	0.130627052
ENSG00000163923	RPL39L	29.001691	29.578119	28.389801	24.00524	23.009716	22.440558	32.633788	30.278351	26.198643	0.098846246	-0.176685712	0.305148099	0.130692479
ENSG00000092330	TINF2	12.43575	11.469033	11.370123	13.851121	13.774329	13.567327	12.728988	12.038263	11.321213	0.002435435	0.295563711	0.255346337	0.130816122
ENSG00000130402	ACTN4	88.865716	85.862042	84.602544	83.766069	85.047915	85.369345	85.922164	84.080025	95.929785	0.031072684	0.119762102	0.036522933	0.130891083
ENSG00000149089	APIP	24.643727	27.077402	27.693572	23.392556	24.192317	23.253958	27.797205	28.685528	29.361994	0.996785659	0.000678698	0.164981195	0.130921975
ENSG00000123983	ACSL3	131.29194	134.291896	140.840913	172.323396	165.358996	166.272172	136.633749	131.927432	150.234978	5.56E-17	0.42883301	0.047722216	0.131000671
ENSG00000161202	DVL3	26.841443	24.885821	26.399696	24.512492	22.853096	21.937282	26.579255	25.083573	28.585844	0.701404901	-0.033484567	0.05546657	0.131000767
ENSG00000111832	RWDD1	18.756114	20.099494	19.256529	20.888424	21.745558	19.1883	18.608256	19.218162	18.609163	0.031318678	0.182392169	0.144245746	0.131044233
ENSG00000073060	SCARB1	78.17112	77.633985	77.106217	67.478698	61.228658	67.412519	77.008861	77.743489	83.372021	0.069651575	-0.10896497	0.031518019	0.131075925
ENSG00000166136	NDUFB8	53.046759	55.473001	51.934484	46.494308	47.17229	51.5707	56.087325	57.842661	50.40522	0.968322666	0.005519568	0.215574891	0.131124034
ENSG00000115568	ZNF142	6.623459	6.330097	6.270958	5.735734	5.3069	5.923869	6.562767	6.187006	7.575924	0.442525059	-0.080460068	0.19283346	0.131198753
ENSG00000171793	CTPS1	22.434052	24.466915	25.024594	21.739559	22.651872	21.243132	25.912506	24.623079	22.105738	0.944748774	-0.007387445	0.10685865	0.131221788
ENSG00000170017	ALCAM	77.30426	84.373356	77.939551	78.510742	76.749317	72.326872	83.020815	76.570336	84.679182	0.060214541	0.119916694	0.068254047	0.131303323
ENSG00000117481	NSUN4	8.169479	8.48372	8.826235	7.934275	8.194497	7.657815	9.438667	8.863399	10.311893	0.860456165	-0.023737932	0.22197152	0.131345011
NewGene_4521	--	2.605119	2.115608	2.542881	2.062678	1.894285	2.184919	2.459914	2.755527	2.336372	0.620357377	-0.096293408	0.401646081	0.13158655
ENSG00000026025	VIM	246.025026	237.256799	265.348898	66.121402	64.138769	66.915479	236.000397	240.052905	270.581733	8.7E-261	-1.73292867	0.051036692	0.131602481
ENSG00000104671	DCTN6	25.280115	23.953572	26.631266	22.552266	21.465417	22.889253	25.699053	25.686362	26.792248	0.743053709	-0.041151761	0.183626917	0.131608654
ENSG00000154723	ATP5PF	111.825192	108.033017	107.767013	107.329862	121.357708	117.703741	117.918403	124.933735	97.390268	0.002819891	0.216152612	0.229657389	0.131615473
ENSG00000100473	COCH	32.222038	36.354161	35.706219	24.979006	25.599727	25.301058	33.606932	34.124487	36.807084	0.00000542	-0.30780145	0.091623891	0.13169547
ENSG00000090054	SPTLC1	54.162652	50.62328	55.087308	51.375833	53.429452	49.899278	45.995831	54.335446	58.607425	0.555296026	0.050163914	0.063577696	0.131897595
ENSG00000088305	DNMT3B	8.164108	9.48556	9.419839	12.57465	10.909227	11.463154	9.568875	8.594448	9.879611	6.48E-10	0.507727137	0.184595911	0.131943457
ENSG00000138668	HNRNPD	132.015767	132.574578	151.130092	146.11191	142.022374	132.878129	144.689323	152.932578	133.747466	0.008857527	0.155496421	0.104971831	0.131997821
ENSG00000138381	ASNSD1	31.010403	35.795393	34.257379	28.752037	28.404491	27.972245	35.662955	31.803946	34.802094	0.346970102	-0.075964586	0.098032359	0.132080354
ENSG00000073803	MAP3K13	9.355743	9.155983	10.03869	9.601712	8.186121	8.860761	10.046667	10.120516	9.363829	0.149342692	0.166534551	0.263198568	0.13227754
ENSG00000127616	SMARCA4	70.660409	63.251272	62.483331	56.622275	51.017403	53.693136	64.604677	59.364336	78.289848	0.100803465	-0.100712807	0.084261719	0.132346923
ENSG00000151779	NBAS	2.809065	2.776014	2.729418	2.651193	2.587128	2.577781	2.896145	2.848158	2.823406	0.684351547	0.053284415	0.234756689	0.132450111
ENSG00000186153	WWOX	5.03344	4.600753	4.054737	6.28988	6.174448	5.385993	4.23247	4.145357	4.822301	0.003016988	0.405151709	0.408802083	0.132581161
NewGene_4439	--	0.937777	1.017661	0.816568	0.87848	0.717791	0.962662	0.909645	1.267895	0.699729	0.978492511	0.017947718	0.788943124	0.132667492
ENSG00000198189	HSD17B11	69.154716	70.968231	64.825509	74.958132	76.337993	75.954332	71.483591	70.640852	68.674442	0.000000262	0.298115316	0.074756164	0.132722865
ENSG00000205352	PRR13	76.383727	75.593821	80.128167	71.4047	68.927343	74.860784	79.609961	85.885695	83.684456	0.236321572	0.085070085	0.065574713	0.132727539
ENSG00000138433	CIR1	9.729479	10.579631	10.240422	14.763289	14.234333	15.043831	10.577785	10.309176	10.590612	9.75E-15	0.659744105	0.232105764	0.132809323
ENSG00000033050	ABCF2	26.51913	26.522978	26.511575	24.052956	22.894685	23.928153	26.376877	25.633712	29.87042	0.745077666	-0.02696338	0.065357647	0.132829787
ENSG00000204314	PRRT1	0.870873	0.762537	0.96288	0.83513	0.443161	1.070398	0.786926	0.895672	1.013679	0.993484931	0.005528241	0.721198952	0.133050816
ENSG00000135372	NAT10	25.552406	30.288366	29.628763	25.385283	21.616616	22.960776	28.359672	26.260597	32.502139	0.372543588	-0.084130104	0.114645511	0.133071354
ENSG00000159399	HK2	54.041087	55.635164	55.589467	49.100067	43.168958	46.44877	55.192322	51.851861	63.178551	0.084218833	-0.105402437	0.05499371	0.13307891
ENSG00000172986	GXYLT2	0.342373	0.443321	0.5464	0.26723	0.267999	0.287995	0.395321	0.482359	0.486927	0.261821291	-0.494884128	0.749995374	0.133087419
ENSG00000253305	PCDHGB6	0.327437	0.343408	0.295679	0.225127	0.248222	0.245809	0.528132	0.217309	0.245335	0.542974992	-0.266220893	0.774931078	0.13310727
ENSG00000137497	NUMA1	19.879717	19.854571	17.68365	25.740267	20.255484	21.382726	20.418067	18.566768	20.191488	0.476307241	0.069952822	0.115938962	0.133113942
ENSG00000134905	CARS2	16.366135	16.325504	15.087941	14.365395	12.754907	12.845145	16.543292	14.62659	16.308566	0.25260974	-0.128598667	0.187487868	0.133136327
ENSG00000115415	STAT1	28.701762	29.424411	32.426839	685.91307	667.957646	650.808848	31.32156	29.71639	32.938968	0	4.398107793	0.067254955	0.133149315
ENSG00000160305	DIP2A	8.947272	8.457293	8.629721	8.191205	7.263334	7.664937	8.681768	7.763489	10.253829	0.460504429	-0.066347468	0.169247357	0.133149478
ENSG00000139684	ESD	38.8064	42.66055	38.736527	36.516771	37.826682	37.698329	40.124439	41.485766	39.764975	0.581865603	0.054432927	0.131787441	0.133316503
ENSG00000169903	TM4SF4	68.195602	53.645939	50.294689	27.060144	29.57312	28.06806	56.331059	60.472724	60.344498	1.15E-20	-0.875693714	0.232036397	0.133366317
ENSG00000006534	ALDH3B1	2.898211	2.990161	2.464876	2.932551	2.999585	2.96164	2.656814	2.442597	2.866571	0.06591835	0.309635205	0.47112252	0.133399194
ENSG00000105971	CAV2	4.09152	4.206803	5.143248	3.74312	3.716154	3.484042	4.872651	4.397921	5.26448	0.278484889	-0.16478394	0.354560354	0.133419129
ENSG00000188234	AGAP4	3.153089	3.491192	3.640624	3.547456	3.763249	3.411996	3.902208	3.40324	3.993105	0.43355889	0.131748121	0.413579652	0.133510599
ENSG00000138085	ATRAID	47.900758	47.953971	42.696494	36.521984	37.863929	41.579254	46.78443	45.848257	48.879225	0.699143614	-0.046541165	0.157000554	0.133537593
ENSG00000150995	ITPR1	1.861843	1.990277	1.98962	1.523229	1.567908	1.423473	2.088263	2.095277	2.359808	0.017662421	-0.281032715	0.263198568	0.133687792
ENSG00000138413	IDH1	120.943982	125.207922	109.062414	172.39904	182.775612	168.513963	119.713635	119.310247	125.747426	4.16E-32	0.709888732	0.059604429	0.13373852
ENSG00000163702	IL17RC	9.859178	9.472642	8.254331	16.210447	16.117981	16.42701	10.133833	9.902852	9.121487	9.26E-27	0.950794414	0.259771767	0.133765493
ENSG00000136631	VPS45	13.268681	15.001901	15.232784	12.301882	12.344953	13.145728	14.029578	13.899614	16.402185	0.526388098	-0.065834195	0.152149638	0.133887313
ENSG00000149131	SERPING1	16.632093	16.63986	17.064542	610.867077	652.145351	655.271296	17.472852	17.353858	16.83756	0	5.215106894	0.151207938	0.133898334
ENSG00000139194	RBP5	4.231585	3.712003	3.269244	4.561883	4.766171	4.85103	3.678337	3.8	3.768959	0.124838828	0.366706065	0.603319236	0.133915656
ENSG00000122140	MRPS2	35.94188	33.749382	35.109422	29.39898	28.233279	30.174733	36.513969	34.10575	36.479618	0.224001974	-0.101575352	0.086107705	0.13395727

Gene ID	Gene name	Ctrl-1 FPKM	Ctrl-2 FPKM	Ctrl-3 FPKM	IFNγ-1 FPKM	IFNγ-2 FPKM	IFNγ-3 FPKM	Sora-1 FPKM	Sora-2 FPKM	Sora-3 FPKM	Ctrl vs IFNγ DESeq2 FDR	Ctrl vs IFNγ DESeq2 log2FC	Ctrl vs Sora DESeq2 FDR	Ctrl vs Sora DESeq2 log2FC
ENSG00000183134	PTGDR2	1.086672	0.88303	0.881718	2.107981	1.609048	1.732277	1.195755	0.92793	0.817016	0.000000161	1.070429756	0.657626916	0.133993281
ENSG00000156096	UGT2B4	42.683678	36.420779	30.930606	39.791136	45.732534	39.236778	34.864806	37.356786	40.959276	0.001163509	0.341516665	0.249626994	0.1340032
ENSG00000136156	ITM2B	61.190408	39.547899	55.141011	81.841367	67.798922	82.295769	64.259302	56.322759	60.65569	3.04E-20	0.484621694	0.04031225	0.134025255
ENSG00000122068	FYTTD1	6.013246	6.672584	5.959465	6.951407	6.962826	7.235895	7.106737	6.500927	6.289662	0.0000168	0.314962732	0.132783568	0.134047261
NewGene_1179	--	30.044291	41.031314	21.988931	39.30466	22.961389	39.742033	64.147806	68.884268	41.160951	0.011640454	0.206719625	0.131519838	0.13407449
ENSG00000083635	NUFIP1	2.330343	2.333604	2.374288	2.285438	1.985019	2.001478	2.900632	2.040687	2.314396	0.931690576	-0.01983624	0.465661649	0.134128097
ENSG00000109180	OCIAD1	121.157201	115.357917	126.594613	111.829938	109.502002	115.58047	126.099272	119.795641	125.281988	0.47412868	0.052270167	0.044948417	0.134188701
ENSG00000062485	CS	123.16567	120.728892	120.778638	124.375186	118.78025	121.18216	118.966454	125.266178	123.043583	0.000489346	0.177426072	0.020975795	0.13423089
ENSG00000131269	ABCB7	7.368782	7.10234	8.177462	7.10307	6.358967	7.156021	7.36332	6.941241	9.708693	0.925199945	0.013703465	0.206543163	0.134365894
ENSG00000110801	PSMD9	7.819675	8.57752	9.444745	8.792999	6.648513	7.937249	8.422171	13.387774	9.186242	0.385272953	-0.118197908	0.27834068	0.134552759
ENSG00000131148	EMC8	16.899563	17.376858	17.619409	15.716383	16.039261	17.932974	18.17893	17.073028	18.063506	0.493369507	0.077019168	0.131487066	0.134573717
ENSG00000168243	GNG4	0.905784	0.504323	0.840505	0.596518	0.491979	0.322933	0.637239	0.623117	0.801366	0.023052984	-0.681566139	0.637219433	0.134661413
ENSG00000138663	COPS4	14.265435	14.964322	14.345923	15.854668	13.658881	12.746864	15.551115	14.526409	14.592049	0.300889377	0.115866763	0.171535851	0.134740857
ENSG00000103449	SALL1	2.486419	1.999406	2.122257	1.699093	1.473961	1.623669	2.305669	2.261107	2.26139	0.046752528	-0.306695364	0.372777188	0.134749973
NewGene_3509	--	1.093809	1.488027	1.124289	1.588142	0.690912	1.048253	1.483534	0.965118	1.928201	0.623900885	-0.181585095	0.681156977	0.134787192
ENSG00000196497	IPO4	36.452103	23.656022	31.739653	13.710166	19.48474	22.869674	36.266557	32.785171	31.624827	0.042119769	-0.508620719	0.285823291	0.134799568
ENSG00000162869	PPP1R21	9.683055	8.324318	8.424516	7.80338	7.978822	6.596308	8.862456	8.384672	10.155174	0.517902581	-0.088176814	0.223223065	0.134848489
ENSG00000188986	NELFB	40.86755	41.481598	38.07902	34.336639	30.554274	34.572207	35.68358	35.193091	45.459793	0.711463412	-0.032105036	0.065677556	0.134938314
ENSG00000078902	TOLLIP	29.82415	27.91209	26.421047	26.60481	26.262853	27.918962	28.222891	27.405905	30.338201	0.214628725	0.094189326	0.067184242	0.135097306
ENSG00000115091	ACTR3	36.244118	38.989902	43.104198	33.338412	33.319034	32.800602	39.730762	38.962526	43.268196	0.157523259	-0.105524234	0.077621498	0.135203221
ENSG00000145741	BTF3	277.266033	267.862198	299.190906	257.073982	254.584604	256.763393	292.889084	295.177051	285.596299	0.95151895	0.005690741	0.071652662	0.135260464
ENSG00000105401	CDC37	99.321485	94.785094	102.645145	82.616676	83.452152	80.905056	95.636139	100.858547	106.066161	0.05868027	-0.119829946	0.054811013	0.135325942
ENSG00000144028	SNRNP200	72.198497	68.690271	64.456276	62.384169	58.716919	59.590565	68.108482	67.807134	75.784004	0.610454246	-0.038162208	0.044671188	0.135399237
ENSG00000197429	IPP	2.342769	2.119324	1.962394	1.665371	1.543413	1.736367	2.18044	1.947182	2.457889	0.26239509	-0.224142673	0.471851146	0.135400765
ENSG00000126107	HECTD3	10.112559	10.085635	8.724613	9.662838	9.252788	9.623461	9.050138	9.540488	11.285076	0.215462563	0.124933516	0.219104997	0.135411737
ENSG00000133818	RRAS2	28.120339	30.554751	32.211616	25.428346	21.798944	24.001194	32.481388	29.213372	32.500659	0.037889071	-0.185156941	0.136571344	0.135443927
ENSG00000107551	RASSF4	6.25994	5.359844	5.648628	5.05698	5.418521	4.856962	5.697488	6.086331	6.086029	0.882274355	-0.024064443	0.235904512	0.135526744
ENSG00000139329	LUM	3.662814	4.174266	4.630727	2.006855	2.293323	1.832133	4.166578	3.962553	4.72733	2.51E-09	-0.876371685	0.358012259	0.135586718
ENSG00000115652	UXS1	89.235869	89.499292	97.885787	56.011069	60.713461	60.004265	92.252382	93.976253	99.598003	8.12E-17	-0.506591906	0.048000636	0.135652242
ENSG00000147548	NSD3	11.563842	13.270174	14.73542	12.70679	12.83592	11.617228	18.287555	16.941691	17.326772	0.742681436	0.035086302	0.111000447	0.135685616
ENSG00000100528	CNIH1	24.722651	28.941659	28.496604	21.930648	22.317549	21.402768	27.796511	27.601833	31.214925	0.008202547	-0.18236814	0.071197072	0.135753588
ENSG00000106367	AP1S1	20.218947	20.545539	20.554853	17.921795	19.252102	18.680177	20.291755	22.102639	20.3894	0.89670426	0.017646507	0.198896844	0.1357704
ENSG00000136518	ACTL6A	119.770133	120.437754	116.293856	92.840314	93.002444	92.305974	121.57969	122.086974	125.280316	0.0000104	-0.218817871	0.031205467	0.135892512
ENSG00000137070	IL11RA	4.465721	4.555585	4.608245	3.730335	3.224216	3.285203	4.869824	3.772923	4.281804	0.687131787	-0.085768991	0.422587993	0.135956517
ENSG00000163382	NAXE	60.565182	63.677382	56.625503	48.043731	49.527804	49.883441	62.652293	62.480937	62.42811	0.067148083	-0.143772542	0.095616604	0.135985325
ENSG00000137200	CMTR1	26.541332	26.061983	24.657884	33.190381	31.408558	30.675684	27.502961	25.405753	26.811955	1.08E-14	0.440622099	0.061826548	0.135999181
ENSG00000198563	DDX39B	163.168947	166.654471	158.556853	150.477088	143.241041	137.221552	177.085906	176.17184	170.69427	0.138851068	-0.089126431	0.019196381	0.136171655
ENSG00000143162	CREG1	19.430769	19.170344	18.033366	13.1418	12.230801	13.694549	20.305855	18.497094	19.589994	0.00000423	-0.38758365	0.130668302	0.136231662
ENSG00000186364	NUDT17	2.580426	2.523573	1.958963	1.973703	1.981853	1.497485	2.606507	2.278358	2.402996	0.44602101	-0.22833951	0.594203992	0.136241911
ENSG00000174547	MRPL11	153.821573	147.420551	137.749873	121.17659	128.133266	125.414246	149.753442	163.321244	149.395334	0.138347249	-0.105960668	0.094805872	0.136296943
ENSG00000107742	SPOCK2	7.097662	6.356964	5.768463	4.699506	4.760077	5.726696	6.129181	6.583731	7.024853	0.194117452	-0.628222815	0.224823781	0.136323572
ENSG00000152359	POC5	3.772563	3.960303	4.553739	3.630151	2.974384	3.027626	4.401	4.06479	4.003242	0.234449578	-0.212348972	0.40530297	0.136380019
ENSG00000002822	MAD1L1	17.057079	16.783969	14.847761	15.376954	13.579479	16.18798	17.357162	15.550091	16.907914	0.668451346	0.053476621	0.151881299	0.136406149
ENSG00000079435	LIPE	0.664178	0.595815	0.33693	0.638158	0.919343	0.550126	0.524978	0.582441	0.783769	0.216445726	0.43986601	0.719662319	0.13646808
ENSG00000135315	CEP162	2.956132	3.392446	3.308029	4.377314	3.92323	3.399919	3.536582	3.340674	3.272979	0.000452819	0.417115923	0.286386246	0.136473237
ENSG00000151320	AKAP6	0.213014	0.202903	0.213444	0.107608	0.082388	0.110384	0.314475	0.273022	0.171548	0.037370755	-0.65484313	0.67309458	0.136474771
ENSG00000105699	LSR	69.868834	61.011873	57.217049	59.630232	57.809936	61.570905	62.518791	63.604613	68.850968	0.553071427	0.058370114	0.122685449	0.136628037
ENSG00000168958	MFF	39.868564	37.315452	42.206755	38.791925	39.437794	37.34917	38.595548	41.434577	47.386899	0.457856491	0.061985683	0.066846392	0.136641744
ENSG00000173207	CKS1B	206.280799	201.9467	199.755631	159.669657	159.294417	159.446165	210.844352	213.782969	201.346025	0.000229628	-0.20011121	0.061658339	0.136822901
ENSG00000108639	SYNGR2	50.968595	51.660348	51.240994	39.327774	40.246582	43.676161	50.895217	49.361335	59.253154	0.021538249	-0.158256418	0.058099354	0.136884076
ENSG00000168078	PBK	8.001536	7.583396	7.403456	6.951957	6.303673	6.166473	8.018185	7.816986	7.848183	0.452996431	-0.103123867	0.251144884	0.136891839
ENSG00000114395	CYB561D2	7.123196	5.199668	5.109075	5.032415	5.892009	5.198917	5.893337	5.736424	7.090191	0.824402837	0.056248946	0.489356886	0.136938942
ENSG00000168743	NPNT	6.936958	7.692612	7.435338	5.224041	5.064626	4.903554	7.104889	6.777542	8.400937	0.0000416	-0.354016489	0.173234413	0.136976456
ENSG00000197119	SLC25A29	7.73143	8.039874	7.526885	6.28427	6.468031	6.924956	8.061317	8.114556	8.795883	0.200229718	-0.127639972	0.153653075	0.13699546
ENSG00000131143	COX4I1	247.753624	233.429592	224.354781	174.86591	198.470725	193.66549	245.308659	255.683712	244.482684	0.032242246	-0.154906509	0.057758323	0.137077059
ENSG00000173818	ENDOV	6.867055	5.958783	5.436186	5.522579	4.502321	4.622953	5.410127	5.099262	6.357687	0.083425197	-0.301805704	0.410867421	0.137135061
ENSG00000135926	TMBIM1	77.224176	72.448053	71.136337	68.600609	73.261739	72.915242	75.413933	80.020271	81.775887	0.430466296	0.061683952	0.045817863	0.137217453
ENSG00000164039	BDH2	15.80017	17.550509	16.171944	12.982402	10.836409	11.211485	17.62719	17.787029	16.688552	0.00180202	-0.362410741	0.254337172	0.137414942
ENSG00000133794	ARNTL	4.1043	3.983837	3.823273	4.902065	4.920868	4.456358	3.965194	3.505164	4.35281	0.000165649	0.473700954	0.368229562	0.137541886
ENSG00000174579	MSL2	8.472711	9.299875	8.644156	8.583141	9.180384	8.526552	9.099154	8.264126	8.8676	0.355424407	0.08920581	0.116460325	0.137548064
ENSG00000142507	PSMB6	126.375798	123.119131	119.373453	112.318199	119.034919	119.011317	122.957425	132.362932	130.651245	0.493314965	0.053894203	0.047912766	0.137607874
ENSG00000136783	NIPSNAP3A	16.501417	15.756654	15.350883	16.705923	17.167976	16.609011	15.569763	16.448988	17.104719	0.01166684	0.231153026	0.174538612	0.137645131
ENSG00000119048	UBE2B	14.304567	15.91672	18.001321	15.697652	16.283451	15.489104	17.406527	15.606887	17.754621	0.364287593	0.102261559	0.201826492	0.137829944
NewGene_110	--	2.361578	2.708154	2.700831	2.618442	2.5362	2.688072	2.729344	2.60706	2.679255	0.244559415	0.16099461	0.306801458	0.137838753
ENSG00000100601	ALKBH1	4.60318	4.213638	4.527685	4.269198	3.599963	4.034614	4.261025	4.703321	5.23164	0.734443219	-0.061511218	0.339055254	0.137863937
ENSG00000134809	TIMM10	52.435019	56.686904	51.178934	48.790649	44.988857	47.098531	57.837315	61.547816	45.864448	0.620817749	-0.055427436	0.319494346	0.137941651
NewGene_3311	--	0.580489	0.82886	0.372916	0.423612	0.409481	0.463877	0.774544	0.677826	0.473968	--	--	0.79391381	0.137983731
ENSG00000185608	MRPL40	19.766293	18.396284	17.295589	19.003906	20.286938	19.543814	19.379057	19.712528	18.058636	0.018924211	0.232188984	0.220329946	0.13804684
ENSG00000110455	ACCS	1.164445	1.62822	1.675159	1.579532	1.287197	1.291366	1.618335	1.468028	1.627568	0.887902321	0.050485323	0.598931509	0.138185281
ENSG00000147955	SIGMAR1	61.013604	61.927302	58.90708	56.91727	55.07415	58.648606	61.083624	61.749951	66.340702	0.414134795	0.061009814	0.044062426	0.138250304
ENSG00000152234	ATP5F1A	205.665829	89.417432	198.966691	176.092653	189.468462	176.595186	213.637047	85.715919	83.396146	0.623900885	0.032190889	0.016402204	0.138324752
ENSG00000169019	COMMD8	24.907839	31.414665	34.219681	25.084044	23.422415	25.410515	30.393353	29.127728	34.022114	0.244936812	-0.144321509	0.251929501	0.138361097
ENSG00000171222	SCAND1	37.43451	34.706805	26.416052	28.769582	32.20702	31.834153	33.744537	34.201678	37.460834	0.602293131	0.075016673	0.232313564	0.138393057
ENSG00000126749	EMG1	20.848159	21.152535	21.453708	18.333043	18.849992	19.159282	21.092062	22.043617	21.914622	0.885564102	-0.013124358	0.040036493	0.138433501

Gene ID	Gene name	Ctrl-1 FPKM	Ctrl-2 FPKM	Ctrl-3 FPKM	IFNγ-1 FPKM	IFNγ-2 FPKM	IFNγ-3 FPKM	Sora-1 FPKM	Sora-2 FPKM	Sora-3 FPKM	Ctrl vs IFNγ DESeq2 FDR	Ctrl vs IFNγ DESeq2 log2FC	Ctrl vs Sora DESeq2 FDR	Ctrl vs Sora DESeq2 log2FC
ENSG00000122033	MTIF3	26.744779	24.910433	25.612172	28.401695	28.362182	25.69677	29.169456	25.331089	25.786636	0.013769993	0.233655975	0.196089764	0.138534728
ENSG00000135587	SMPD2	6.274623	5.982229	5.642444	4.698043	4.540418	4.646359	6.122156	5.625119	6.685785	0.23371725	-0.196341462	0.364877552	0.138592871
ENSG00000115128	SF3B6	68.210197	61.88287	65.790062	57.566067	61.995461	58.984226	66.650871	68.7948	66.611282	0.907788808	0.013815238	0.11341463	0.138611877
ENSG00000144445	KANSL1L	4.664644	4.628938	5.047079	3.822311	3.838519	3.780468	4.906447	4.093692	4.201142	0.605412914	-0.077220927	0.285743516	0.138624305
ENSG00000074201	CLNS1A	28.400159	29.379775	32.396025	43.006859	41.164386	37.001379	30.57181	31.268223	31.553518	2.88E-13	0.447196876	0.070552333	0.13866578
ENSG00000117118	SDHB	35.263451	33.257011	34.189892	31.612157	32.262749	33.011951	34.270905	34.508789	37.317303	0.525887297	0.06329836	0.106217064	0.138716006
ENSG00000136930	PSMB7	164.969065	159.69634	153.860562	136.790514	143.179876	139.717261	162.286287	172.372709	158.574659	0.601679585	-0.040970939	0.076097643	0.138730037
ENSG00000130309	COLGALT1	28.763791	29.014094	29.248432	20.162044	18.817718	20.24139	29.02143	29.995011	30.948062	7.37E-13	-0.408354857	0.039391108	0.138748476
ENSG00000099940	SNAP29	10.545409	10.05168	10.724294	10.996637	11.961991	11.582221	10.785758	10.498863	11.06164	0.000189263	0.285954104	0.091126535	0.138843376
ENSG00000140854	KATNB1	9.855466	9.069773	9.109915	7.218568	8.061722	7.729653	9.402414	8.668379	10.899568	0.203104513	-0.145317225	0.208570078	0.138853492
ENSG00000124596	OARD1	25.504197	27.059562	26.675516	21.753669	24.23653	21.209287	29.134625	29.427555	25.83492	0.4757562	-0.065105593	0.141813561	0.138854774
ENSG00000159063	ALG8	43.860105	42.362704	44.800374	37.418693	38.689538	37.958528	43.798925	44.222865	44.221886	0.611426258	-0.0437504	0.073322976	0.13886589
ENSG00000116459	ATP5PB	52.769325	52.410233	52.724819	44.479191	44.02441	43.955425	52.792679	54.351738	55.965202	0.0608846	-0.106155847	0.027702564	0.138943985
ENSG00000131236	CAP1	78.463234	87.938081	80.942255	59.805692	65.425173	66.932305	81.859083	89.823321	89.160417	0.00011696	-0.225495794	0.068995092	0.139003704
ENSG00000168061	SAC3D1	12.080376	11.414436	8.791583	11.228405	10.758976	11.967358	11.831258	10.533298	10.490927	0.124951035	0.218705302	0.345947853	0.139062402
ENSG00000140471	LINS1	1.786312	2.049405	2.317552	2.641529	2.443835	2.229507	2.26065	2.062257	1.8405	0.00399271	0.447749932	0.460647131	0.139102825
ENSG00000109458	GAB1	2.332482	2.54011	3.328632	3.375854	2.712608	2.417051	2.915949	2.609799	2.649731	0.154523529	0.171829368	0.29199845	0.139135974
ENSG00000110497	AMBRA1	9.351487	9.654683	8.898472	10.606161	9.540108	10.455503	9.883048	8.890654	9.801237	0.000114696	0.285927938	0.102872259	0.139194464
ENSG00000177105	RHOG	26.503008	25.725672	25.71462	24.151257	24.255707	24.091847	26.555488	25.985636	27.998699	0.699346345	0.042104575	0.111276812	0.139290865
ENSG00000077254	USP33	9.58149	9.228688	10.395219	13.93995	12.236012	11.956813	9.527335	8.799056	9.874726	9.65E-18	0.584785074	0.10548289	0.139395637
ENSG00000077157	PPP1R12B	1.379406	1.264329	1.499971	1.388093	1.045123	1.440362	1.903079	1.972779	1.728777	0.930787487	-0.014653997	0.211939796	0.139404965
ENSG00000111752	PHC1	5.834342	5.621848	6.638701	8.080292	5.202604	5.007436	5.630063	6.340986	6.62915	0.531247802	0.303917145	0.253270165	0.139405781
ENSG00000005302	MSL3	3.558124	3.97806	4.289144	4.539312	4.008384	3.863533	4.081572	4.203177	3.8197	0.201018879	0.192324523	0.36214006	0.13951214
ENSG00000173451	THAP2	1.899296	2.56867	2.773492	1.982728	1.835455	1.624295	1.62594	1.407659	1.414885	0.335252127	-0.200961246	0.462173809	0.139549439
ENSG00000172482	AGXT	6.817312	6.142878	4.020703	6.391807	7.137627	6.925292	5.839513	6.568306	5.07029	0.021928632	0.41519609	0.51967373	0.139550135
ENSG00000167815	PRDX2	174.106928	162.181028	163.407257	129.032393	125.636907	132.694174	170.46822	181.862301	174.241848	0.000234138	-0.234916975	0.065357647	0.139551614
ENSG00000127481	UBR4	17.316658	15.802741	18.873703	18.998045	17.454004	15.78802	19.309109	17.337491	20.116242	0.011555885	0.164219193	0.063406024	0.139561795
ENSG00000217555	CKLF	11.137287	10.894692	9.808628	8.229041	8.715116	10.513725	11.913634	11.323943	9.693316	0.791895315	-0.065672768	0.465008612	0.139573826
ENSG00000215114	UBXN2B	4.797596	4.244916	4.57595	4.553441	4.287683	4.019825	4.93045	4.434	4.698075	0.529910005	0.080711528	0.195197495	0.139577704
ENSG00000165678	GHITM	68.33252	68.779388	66.191948	64.315521	64.038368	63.749649	68.890572	69.210144	71.933075	0.307480123	0.06564757	0.027110385	0.13981265
ENSG00000183431	SF3A3	29.015036	29.603785	28.63418	27.52981	25.905607	26.222452	30.67477	28.779428	30.711159	0.856006078	0.015593099	0.033258028	0.139829673
ENSG00000100814	CCNB1IP1	70.632967	69.321694	69.711672	48.67453	46.791979	44.925178	74.110083	73.70764	71.618818	3.96E-14	-0.418933491	0.044495168	0.139838989
ENSG00000176624	MEX3C	10.360532	9.89553	10.381342	8.60082	8.106932	8.870571	10.769808	8.930892	12.053325	0.22592017	-0.112620173	0.186993156	0.139899151
ENSG00000121316	PLBD1	2.504416	2.979161	2.738368	3.196683	3.493381	3.162859	2.973918	2.425853	3.121507	0.0163521	0.389269712	0.455813051	0.139998492
ENSG00000185842	DNAH14	3.36217	3.173397	3.371101	3.096033	2.694681	3.200668	5.062106	4.082671	4.020422	0.918672101	0.016712679	0.259037651	0.140098908
ENSG00000133731	IMPA1	20.483818	21.846437	21.971581	19.357291	20.557054	19.276661	21.725751	20.720539	22.729421	0.681847625	0.043789838	0.105156849	0.14012281
NewGene 3546	--	43.478637	42.546351	48.174861	35.90052	34.948353	36.407823	45.862305	43.029555	50.331017	0.003956278	-0.172853736	0.044224203	0.140158369
ENSG00000159403	C1R	6.198999	6.081814	5.519158	127.943635	124.764904	125.942683	6.673628	5.708268	5.967653	0	4.538282727	0.286799967	0.140205268
ENSG00000105983	LMBR1	10.451275	10.382644	11.394792	11.089695	9.127511	9.662306	12.583674	11.592924	10.9763	0.83480464	-0.019242945	0.053116164	0.140259253
ENSG00000136021	SCYL2	16.745984	17.554743	17.233645	16.923634	18.396685	16.338914	18.544672	16.956624	18.010746	0.052846301	0.144550305	0.03930778	0.140288476
ENSG00000159128	IFNGR2	41.638894	39.355856	45.636817	34.518121	31.728167	32.229407	44.003086	44.935376	46.670704	0.000891982	-0.240038652	0.084818827	0.140362457
ENSG00000151470	C4orf33	2.441098	2.97014	2.218286	2.87932	3.095565	2.855605	2.84755	2.779916	3.808299	0.06537535	0.349038115	0.480667273	0.140370148
NewGene 3207	--	1.935844	2.054572	1.69925	1.463391	2.087105	1.854047	2.129381	2.032537	1.714172	0.89670426	0.06644746	0.721619385	0.140408245
ENSG00000162994	CLHC1	2.24739	3.052254	3.401175	2.239086	2.21594	1.844261	2.835511	2.597652	2.505054	0.414134795	-0.221120977	0.565735363	0.140439605
ENSG00000145220	LYAR	37.275887	36.374963	45.131283	40.186135	36.198048	35.61607	40.045303	40.621966	42.076553	0.607204927	0.061552166	0.158328866	0.140566686
ENSG00000123561	SERPINA7	3.254433	3.664588	1.843719	30.38863	28.829688	29.049658	3.527114	3.377419	2.885599	2.12E-235	3.312683564	0.517806453	0.140660259
ENSG00000185246	PRPF39	19.005684	19.759826	23.29153	18.556048	18.712892	17.537628	20.938145	21.245719	22.435551	0.851525349	-0.023280866	0.127187896	0.140734183
ENSG00000152684	PELO	3.132789	2.916714	2.801842	2.63475	2.791468	2.512288	3.181997	2.970915	2.996431	0.95290796	-0.011812714	0.295734806	0.140735413
ENSG00000150990	DHX37	11.800513	11.538619	11.321451	9.223183	8.097821	8.844138	11.502712	11.319541	13.372836	0.001100915	-0.25602486	0.097804209	0.140797599
ENSG00000113946	CLDN16	0.34023	0.398207	0.755264	0.397148	0.521466	0.399895	0.420513	0.521405	0.595905	0.960983569	-0.029589647	0.746994746	0.140846161
NewGene 6718	--	1.220301	1.288326	1.011932	0.372491	0.589202	0.640427	1.068094	0.91489	1.260842	0.00016933	-1.094202602	0.597991501	0.141141951
ENSG00000113282	CLINT1	23.093552	23.738742	26.97741	23.252562	21.061927	21.658789	24.468785	25.224614	26.785922	0.622765929	0.045840457	0.079189441	0.141151109
ENSG00000142945	KIF2C	26.776635	27.25342	25.930146	19.204353	17.636273	17.764102	26.997409	26.343781	27.288722	2.29E-08	-0.372278753	0.050983847	0.14121265
ENSG00000121680	PEX16	17.591109	14.603364	15.050724	15.000161	14.477944	15.186365	15.450329	15.230476	16.985232	0.609223996	0.074209174	0.257243473	0.14122016
ENSG00000178096	BOLA1	10.477881	9.791987	8.832668	8.818345	9.005797	9.210766	9.367717	10.545365	10.560786	0.891757531	0.030402629	0.401418605	0.141232731
ENSG00000092841	MYL6	354.090637	352.188507	337.532687	322.705443	355.040856	351.641383	350.773719	426.493515	332.391436	0.070216639	0.117621909	0.171591604	0.141311439
ENSG00000095794	CREM	6.874503	6.752418	6.705863	6.708587	7.438299	7.444372	7.529071	5.178901	7.451665	0.073690592	0.264550263	0.389920836	0.14132763
ENSG00000168724	DNAJC21	10.698813	10.689709	11.089871	10.448156	10.589882	8.430768	9.667424	9.417929	9.53583	0.530915218	0.062675833	0.108486295	0.141365359
ENSG00000136425	CIB2	5.285565	3.414097	3.64518	3.765777	3.627994	2.985706	4.399332	4.270618	3.817129	0.826335502	-0.066768308	0.552730269	0.141389115
ENSG00000144535	DIS3L2	5.825763	5.991466	5.367236	4.883521	3.682831	4.329793	5.898482	5.437649	5.643859	0.068364114	-0.228689242	0.226054354	0.141427789
ENSG00000128294	TPST2	4.482303	4.154477	3.854004	4.184364	4.405767	4.152373	4.446238	4.483708	4.838525	0.159758215	0.163924877	0.229876441	0.141485015
ENSG00000112759	SLC29A1	53.38808	52.332024	49.609778	37.133619	33.936832	35.379349	54.448155	54.878165	56.126323	6.88E-11	-0.388301556	0.037051049	0.141505454
ENSG00000165416	SUGT1	3.213958	2.828505	3.123892	3.504084	2.802093	2.969838	4.817649	5.270222	4.09365	0.359198398	0.126595695	0.273049009	0.141801608
ENSG00000131116	ZNF428	17.551543	17.843368	14.473827	14.285817	11.474503	15.493949	15.356157	18.219174	18.488333	0.306875526	-0.140740734	0.255386618	0.141913487
ENSG00000275895	U2AF1L5	53.749273	55.328651	63.859063	60.587913	56.969068	64.323399	63.501936	59.218742	57.60572	0.011555735	0.22142365	0.139809843	0.14192228
ENSG00000126215	XRCC3	7.08157	6.616565	6.417457	8.476513	7.227935	6.523536	6.832354	7.043704	8.346327	0.070476659	0.207335401	0.221290886	0.142043065
ENSG00000181991	MRPS11	12.477765	10.390541	10.149724	8.796392	9.173385	10.246597	10.018187	12.497081	11.279229	0.86761117	0.022355666	0.155381863	0.142100897
ENSG00000153140	CETN3	3.862733	4.531135	3.802296	4.156806	4.435234	4.296184	4.517948	4.412385	5.595083	0.362816929	0.156786176	0.37719391	0.142117418
ENSG00000251369	ZNF550	2.504343	2.918111	2.884885	2.775395	2.666635	2.25414	3.354155	2.503315	2.777009	0.977007109	-0.006536785	0.354560354	0.142357586
ENSG00000170759	KIF5B	24.874352	27.161465	27.782198	29.569536	27.849329	28.315369	27.345276	25.040762	30.369221	0.0000234	0.250477816	0.064760632	0.142411356
ENSG00000152284	TCF7L1	13.117702	10.585241	10.905763	9.435711	8.873635	9.148939	11.545386	12.035446	12.344134	0.076311844	-0.194286603	0.19832922	0.142450875
ENSG00000103266	STUB1	27.591916	23.590809	26.089705	26.86846	24.910184	25.766756	24.840414	25.460995	27.360595	0.11948575	0.147392	0.134369807	0.142651578
ENSG00000197122	SRC	4.144836	3.43649	3.289187	3.114106	2.921565	3.116702	3.895342	3.768429	3.780797	0.441775345	-0.115824386	0.275407356	0.142663845
ENSG00000101255	TRIB3	41.665674	44.472185	44.536251	56.141304	60.991991	57.455866	40.400948	44.210004	52.189599	5.13E-21	0.556571549	0.100359159	0.142716811

Gene ID	Gene name	Ctrl-1_FPKM	Ctrl-2_FPKM	Ctrl-3_FPKM	IFNγ-1_FPKM	IFNγ-2_FPKM	IFNγ-3_FPKM	Sora-1_FPKM	Sora-2_FPKM	Sora-3_FPKM	Ctrl_vs_IFNγ_DESeq2_FDR	Ctrl_vs_IFNγ_DESeq2_log2FC	Ctrl_vs_Sora_DESeq2_FDR	Ctrl_vs_Sora_DESeq2_log2FC
ENSG00000273749	CYFIP1	39.351441	35.701666	36.741342	35.121557	32.556756	33.05276	38.043616	34.659829	40.510653	0.66024309	0.030570974	0.028777084	0.142834195
ENSG00000241697	TMEFF1	13.256776	14.25973	14.25395	7.743402	7.831218	7.981475	15.987043	13.04223	14.239669	8.67E-17	-0.677539826	0.162671082	0.142858184
ENSG00000175216	CKAP5	50.247709	51.294929	48.408207	41.52247	37.560863	39.258096	52.02635	50.344864	52.288465	0.000344459	-0.189593004	0.017117104	0.142968419
ENSG00000127980	PEX1	6.747307	7.341835	7.017745	6.509781	5.868029	6.013048	7.361514	6.630627	7.595942	0.616131418	-0.057051843	0.127217689	0.14305582
ENSG00000149573	MPZL2	35.597802	38.347619	42.121005	39.790703	40.031033	36.646065	39.887702	37.701679	43.861743	0.064052103	0.151403341	0.088771727	0.143093759
ENSG00000149050	ZNF214	0.438083	0.425607	0.328384	0.308448	0.388014	0.218354	0.391263	0.500556	0.336058	0.672790554	-0.238883786	0.758781725	0.143159082
ENSG00000068366	ACSL4	128.989234	143.240752	148.349447	161.394448	163.584358	155.235147	143.426292	132.778847	145.297666	1.69E-09	0.360635644	0.051746319	0.143194048
ENSG00000197774	EME2	4.896932	4.789077	4.671582	5.201784	4.793521	4.496276	4.904283	4.725032	5.462902	0.334407097	0.120147492	0.230005139	0.143234102
ENSG00000174456	C12orf76	4.568902	3.661078	4.008915	3.821444	3.081945	3.657381	4.409979	4.164994	4.482505	0.925862045	-0.028882351	0.502329316	0.143267165
ENSG00000110243	APOA5	0.794014	0.941347	0.345284	0.350263	0.592545	0.508514	0.935292	0.630722	0.609474	0.526750986	-0.356468381	0.77199805	0.143318374
ENSG00000165828	PRAP1	24.257488	21.687803	16.480723	18.099578	20.86453	18.804063	20.708591	18.017223	20.396225	0.532678788	0.111251141	0.35381978	0.14351851
ENSG00000280071	GATD3B	22.859058	21.022503	21.987509	16.85425	18.67695	19.51181	19.859854	24.559127	23.74097	0.260593374	-0.117810987	0.213950833	0.143585458
ENSG00000163161	ERCC3	26.089043	25.642269	25.641382	20.759849	21.876045	21.441304	25.643288	25.514977	31.179003	0.035065825	-0.146707386	0.057342304	0.143775908
ENSG00000132661	NXT1	25.772226	24.02054	24.584135	20.565115	18.821821	22.500988	24.421467	26.659729	25.931564	0.311554078	-0.116889379	0.159554835	0.143849767
ENSG00000182472	CAPN12	0.631898	0.639223	0.630349	0.531059	0.523467	0.602903	0.576836	0.61787	0.649313	0.930787487	-0.045144187	0.704101911	0.143876551
ENSG00000166831	RBPMS2	7.667344	8.41095	7.944148	5.926365	5.362099	5.726158	7.660888	7.965657	9.279684	0.002858525	-0.349539614	0.252039552	0.143954306
ENSG00000159377	PSMB4	294.030243	284.29483	272.087769	286.338074	298.836884	287.701569	282.966339	304.489746	292.891357	0.00117532	0.185137143	0.044062426	0.143992736
ENSG00000102225	CDK16	29.823214	27.068374	25.391726	24.196026	21.961428	23.016785	26.84279	27.122812	31.740457	0.339513305	-0.087106336	0.115784893	0.144206395
ENSG00000136485	DCAF7	17.790513	18.032037	17.782759	17.706975	17.169802	17.57364	18.437697	16.846854	19.22577	0.108333003	0.100060298	0.028654119	0.14430675
NewGene_3570	--	2.604908	2.627128	2.453844	2.154123	3.079329	1.786542	2.761507	2.73184	2.420273	0.863061552	-0.043165656	0.412712546	0.144329486
ENSG00000171863	RPS7	552.641226	493.462138	456.15414	361.64908	386.723882	381.926647	517.311876	538.44909	525.239683	0.0000565	-0.259501025	0.062625492	0.144349343
ENSG00000125166	GOT2	50.271088	50.990277	49.999124	46.11134	44.510706	44.028088	53.86466	50.712784	52.075543	0.805101174	-0.019425975	0.02891346	0.14436443
ENSG00000135535	CD164	98.243061	101.648795	104.538198	85.882895	86.184385	85.997391	93.898029	101.853675	104.276111	0.115634506	-0.097624173	0.026226016	0.14443178
ENSG00000102316	MAGED2	27.088636	25.403985	23.53206	23.477222	26.144366	25.042072	24.109967	25.08851	27.959682	0.204486281	0.122199334	0.124747176	0.144453878
ENSG00000109775	UFSP2	6.334258	6.580664	6.61336	6.836057	7.555014	6.041755	6.427038	7.392474	6.671868	0.189343735	0.18854202	0.325357722	0.144458227
ENSG00000139620	KANSL2	24.06833	25.081944	23.834243	24.666165	23.32628	19.366054	25.97986	22.802018	23.305305	0.656469631	-0.047344185	0.093679924	0.144467991
ENSG00000179010	MRFAP1	196.888364	205.66503	192.458333	184.381831	174.422125	179.589513	208.991115	201.625501	210.308278	0.7240127	-0.024871558	0.009361864	0.144475792
ENSG00000143409	MINDY1	6.965308	6.530648	6.02314	5.233267	6.820455	5.592581	6.358507	5.884481	6.522195	0.777316478	0.056321059	0.317685365	0.14448559
ENSG00000125772	GPCPD1	2.460337	2.786404	2.485431	2.227395	1.829896	2.094337	2.975052	2.393183	2.657036	0.203317112	-0.181949649	0.291759365	0.144487814
ENSG00000150967	ABCB9	2.072594	1.969184	2.03736	1.802942	1.753552	2.167963	2.254434	2.029154	2.535101	0.933602721	-0.019019822	0.328865993	0.144556643
ENSG00000116221	MRPL37	67.544199	60.41769	58.862635	54.634708	49.086192	52.185121	64.125455	62.164097	67.676664	0.152267289	-0.118800522	0.066705012	0.144588379
ENSG00000105298	CACTIN	15.033332	14.266661	12.640024	13.146905	12.226385	13.858391	14.540703	13.56915	16.18744	0.760502089	0.036743998	0.136926941	0.144636839
ENSG00000101057	MYBL2	74.549974	82.9548	77.06991	59.532148	53.259374	58.732432	80.324686	77.61339	85.175223	0.000000676	-0.305418703	0.028879623	0.144839667
ENSG00000181924	COA4	39.810913	37.086446	37.006004	35.563905	36.052961	37.029934	39.208407	39.270505	38.084875	0.435161385	0.08067764	0.123878547	0.144948277
ENSG00000087053	MTMR2	8.472067	9.182014	8.757639	6.386319	7.709554	7.026917	10.325146	8.2106	7.760215	0.215528456	-0.115767966	0.156068283	0.145018572
ENSG00000116675	DNAJC6	0.163358	0.215496	0.30048	0.213441	0.249189	0.185832	0.251917	0.350655	0.240368	0.881705247	0.082327192	0.734890045	0.145089451
ENSG00000143774	GUK1	61.260242	56.619565	43.059319	93.906695	99.729602	105.647461	53.450403	56.098496	54.995798	2.53E-28	1.042980962	0.21451921	0.145119723
ENSG00000100979	PLTP	6.924065	5.767347	6.494321	5.602698	4.386078	4.561977	6.120167	6.085368	7.757825	0.114751838	-0.251908265	0.358847783	0.1451482
ENSG00000239264	TXNDC5	178.086614	178.248878	182.330906	178.380491	168.056542	171.597941	181.061022	178.420284	199.789581	0.074782834	0.090205056	0.011606888	0.145217539
ENSG00000152154	TMEM178A	5.540653	4.827183	4.886964	2.730616	2.80644	2.86602	4.262072	5.168741	6.12853	0.0000843	-0.660700703	0.446093919	0.145226809
ENSG00000100297	MCM5	58.004566	57.529407	54.994402	50.651974	49.850509	51.866467	56.980646	57.940542	62.189954	0.72685146	-0.026828677	0.020528098	0.145282156
ENSG00000174243	DDX23	46.959129	40.704755	41.023585	55.081509	53.495178	52.729467	42.918365	42.352373	48.691169	4.23E-13	0.467859617	0.074756164	0.145353898
ENSG00000166233	ARIH1	4.590754	4.725582	4.847612	4.729633	4.629492	4.52299	5.046943	4.744026	5.138699	0.053289319	0.126507694	0.034873484	0.145402953
ENSG00000111339	ART4	1.316753	1.430613	1.374513	0.928674	0.934439	1.001239	1.810932	1.289971	1.55812	0.051620153	-0.364467143	0.441451872	0.145560231
ENSG00000109971	HSPA8	544.573473	535.612308	557.515654	456.485061	417.961745	417.197135	589.817215	647.973897	601.773045	0.000783019	-0.17989868	0.012345734	0.145566022
ENSG00000086232	EIF2AK1	95.80153	88.830442	92.135954	88.320937	89.886842	86.181974	98.791807	92.495281	99.511744	0.189189985	0.080500558	0.01437918	0.145714916
ENSG00000087111	PIGS	42.822726	39.502594	40.67139	36.735624	33.707426	37.597657	40.497143	40.889437	46.001253	0.792359148	-0.025983757	0.04182468	0.14573265
ENSG00000080618	CPB2	14.781588	13.154332	11.639913	8.490859	8.977702	8.617277	13.136973	14.739612	13.083028	0.0000939	-0.44952592	0.268244572	0.145814581
ENSG00000129968	ABHD17A	34.955888	33.684045	30.414181	26.703149	29.854408	32.197146	32.549518	31.656833	36.001734	0.910266138	0.01636157	0.123826341	0.145871091
ENSG00000137710	RDX	22.362217	25.657555	25.218459	28.1664	26.530077	26.29747	25.329462	23.55748	26.909283	0.0000123	0.294711885	0.066023273	0.145889285
ENSG00000107669	ATE1	7.180155	7.523276	6.684301	6.477277	6.073584	6.716026	7.538289	7.320533	7.928766	0.800155312	-0.029754212	0.109183611	0.145982034
ENSG00000157540	DYRK1A	11.662505	15.444977	15.873483	13.433869	15.541162	16.045622	17.479239	12.293918	11.814134	0.000452863	0.217941272	0.057274304	0.146011185
ENSG00000171004	HS6ST2	19.216427	18.871925	18.076871	13.462048	12.275974	13.513518	18.854367	19.591336	21.123121	1.39E-09	-0.404910119	0.048949722	0.146079631
ENSG00000104442	ARMC1	10.826083	11.850494	11.181646	10.312947	9.733084	10.155584	11.591179	11.434303	12.090973	0.86382996	-0.021165289	0.105857084	0.146103786
ENSG00000269190	FBXO17	15.484821	13.781563	13.045045	9.334692	8.104316	9.58329	15.730634	12.924038	14.770307	0.000000289	-0.503051357	0.176898707	0.146115882
ENSG00000100802	C14orf93	3.432011	4.008581	4.087149	4.033881	3.614097	3.153755	3.634474	4.738028	3.788152	0.225744862	-0.217857745	0.363155216	0.146205252
ENSG00000068438	FTSJ1	11.083068	11.451868	11.939094	11.464879	10.072383	10.600976	12.218229	12.689913	10.879629	0.762588527	0.041252101	0.234827893	0.14620538
ENSG00000164109	MAD2L1	16.436838	18.813844	18.959471	12.307183	10.679046	12.149502	18.872995	16.842541	20.557789	4.89E-08	-0.415327361	0.076762463	0.146219024
ENSG00000079335	CDC14A	0.662555	1.148791	0.876789	0.834281	0.94075	0.732759	1.01514	0.780261	0.90351	0.838301353	0.073320054	0.599244814	0.146223542
ENSG00000162368	CMPK1	40.719103	45.165825	49.292049	46.030469	46.544253	47.203941	47.355782	43.97805	49.644131	0.006338631	0.199332981	0.069931489	0.146367162
ENSG00000136868	SLC31A1	24.074087	25.314728	25.173237	31.595863	30.922638	29.716543	25.564138	24.080359	27.838093	1.32E-17	0.454061681	0.031748836	0.146451157
ENSG00000011451	WIZ	16.930733	17.593667	18.93419	18.411451	16.920854	17.446183	19.736033	16.564862	20.961613	0.133693422	0.113051973	0.06960169	0.146606401
ENSG00000177646	ACAD9	22.534583	22.667113	23.094584	20.284024	19.315892	20.341624	23.151503	23.365689	24.273138	0.626581457	-0.044519418	0.053181023	0.14687525
ENSG00000204356	NELFE	99.405866	96.281206	92.693839	79.438946	79.825888	82.915047	104.012113	103.036919	90.9762	0.13261482	-0.114156469	0.086374593	0.146876638
ENSG00000198899	MT-ATP6	7026.701172	7058.657227	7123.946289	6510.413574	6579.563965	6191.033691	6820.899902	7307.189453	7905.11084	0.889945453	0.009933698	0.020288683	0.147025519
ENSG00000133935	ERG28	27.634539	27.203352	26.754375	33.646626	35.357819	32.894226	27.545818	28.179316	28.989456	2.13E-14	0.467743955	0.042061825	0.147111522
ENSG00000161647	MPP3	1.095059	1.0062	0.948158	0.682521	0.472753	0.761736	0.939377	0.620927	1.584818	0.085248977	-0.515467326	0.647239709	0.147122875
ENSG00000115540	MOB4	15.795086	17.793059	18.706309	20.125019	18.511795	20.654599	18.460107	17.482802	21.037573	0.019271684	0.183701997	0.082730548	0.147301265
ENSG00000126767	ELK1	9.003373	9.837129	8.723936	7.624087	7.62203	7.35684	9.492447	9.301809	10.19485	0.13959317	-0.15905553	0.152579239	0.14762446
ENSG00000134452	FBH1	13.144421	11.121156	11.075142	10.532714	10.585504	9.804332	12.025309	11.854751	12.715269	0.699527705	-0.048116577	0.13036333	0.147657537
ENSG00000124535	WRNIP1	20.530116	17.583653	18.200116	16.196965	19.139351	14.959845	18.09675	17.390384	19.917484	0.698507083	-0.039979429	0.073798358	0.147665237
ENSG00000151576	QTRT2	10.079582	10.703595	11.026306	9.40897	8.552935	9.226162	11.445612	10.588411	11.049204	0.526534955	-0.063413642	0.083780618	0.14768321
ENSG00000073969	NSF	12.499856	13.571722	12.893344	13.074074	13.890115	13.138578	13.097586	13.406383	13.895814	0.011634213	0.192585617	0.063088998	0.147753681
NewGene_1444	--	3.12152	3.083253	3.848278	3.061434	3.133945	3.152248	3.151864	3.321388	4.352961	0.932098044	0.015091073	0.28812767	0.147800866

Gene ID	Gene name	Ctrl-1 FPKM	Ctrl-2 FPKM	Ctrl-3 FPKM	IFNγ-1 FPKM	IFNγ-2 FPKM	IFNγ-3 FPKM	Sora-1 FPKM	Sora-2 FPKM	Sora-3 FPKM	Ctrl vs IFNγ DESeq2 FDR	Ctrl vs IFNγ DESeq2 log2FC	Ctrl vs Sora DESeq2 FDR	Ctrl vs Sora DESeq2 log2FC
ENSG00000077232	DNAJC10	20.425625	21.110455	21.500082	22.067648	21.814569	18.408532	17.77688	20.55236	23.009697	0.362083345	0.064367947	0.023735951	0.147878329
ENSG00000175806	MSRA	2.428708	3.026002	3.204036	2.520519	2.409127	2.574654	2.990393	3.201467	2.749305	0.866443244	-0.051011131	0.526983498	0.148120563
ENSG00000145945	FAM50B	4.361151	4.531302	4.862775	5.27597	5.759293	6.012105	4.789303	4.359151	5.211863	0.001611233	0.44885608	0.359374861	0.148165326
ENSG00000214253	FIS1	52.812678	41.720237	42.528774	40.939357	44.26867	42.435869	45.65079	50.663297	45.397978	0.707365486	0.05361781	0.227173918	0.148199844
ENSG00000133639	BTG1	18.554144	19.022928	17.307684	26.412338	27.28322	27.036295	19.821309	18.161105	18.80617	1.43E-33	0.707572009	0.053879998	0.148224455
ENSG00000253313	C1orf210	15.16898	15.296795	13.277736	7.988344	8.805717	8.755387	13.57599	16.161068	15.877196	1.01E-08	-0.621254532	0.230270344	0.148247937
ENSG00000132196	HSD17B7	21.153673	22.591958	22.503541	35.66589	33.140862	35.74886	22.411616	22.130604	24.098295	9.9E-27	0.807859869	0.129145217	0.148255547
ENSG00000196470	SIAH1	14.189184	13.131312	13.863769	13.847559	14.306447	12.899742	15.100834	11.841361	15.455962	0.175228627	0.128616709	0.15718094	0.148264346
ENSG00000135090	TAOK3	2.508769	2.320311	2.923586	2.462959	2.388766	2.224378	2.30474	2.748114	2.955945	0.537808445	0.111214993	0.374001851	0.14829507
ENSG00000146072	TNFRSF21	11.260402	10.761619	12.973653	23.321178	21.365997	25.040894	11.632407	10.884871	13.954867	6.35E-46	1.141696945	0.177097986	0.148295307
ENSG00000173918	C1QTNF1	1.383208	1.200087	0.937072	0.577265	0.417259	0.640388	1.06758	1.17037	1.614388	0.004482031	-0.870475486	0.636695873	0.148348579
ENSG00000115661	STK16	9.540868	10.67703	10.366013	8.69257	8.994404	9.9828	9.192375	12.358522	12.308796	0.959048469	-0.008326218	0.242725732	0.148484026
ENSG00000168785	TSPAN5	0.755113	0.932206	0.587782	0.778861	0.701131	0.55911	0.752388	0.787662	0.830226	0.930787487	-0.038084881	0.62646351	0.148500994
ENSG00000111641	NOP2	42.850714	40.503743	44.54073	43.941095	39.123038	40.99745	45.011135	50.156229	47.434043	0.638454871	0.040433931	0.0361108	0.148514159
ENSG00000129235	TXNDC17	15.444928	15.905724	16.368354	15.054451	15.127842	16.527604	16.816208	18.794047	12.976067	0.491881066	0.077143335	0.270478993	0.149071256
ENSG00000108448	TRIM16L	3.19372	3.421541	3.597441	2.694133	2.773356	2.728312	3.182448	3.218434	4.243595	0.397929519	-0.16833448	0.430557798	0.149086244
ENSG00000168268	NT5DC2	78.95526	75.418754	65.40112	46.164989	42.872926	45.115208	72.086813	72.995908	83.067541	2.69E-15	-0.568632299	0.090046306	0.14908981
ENSG00000170266	GLB1	36.557677	34.715775	33.839314	27.840059	29.575761	29.025764	34.846215	37.14499	38.405687	0.069892605	-0.131229403	0.045743992	0.149191224
ENSG00000186187	ZNRF1	6.811534	7.294043	7.138296	6.507544	6.394207	6.428147	7.413222	6.899467	8.714141	0.935248325	-0.011245528	0.108762718	0.149300076
ENSG00000177868	SVBP	7.146903	7.946524	6.049991	6.342108	6.906311	6.520864	7.181821	7.588432	7.148481	0.83723163	0.058611922	0.501093169	0.149301173
ENSG00000169564	PCBP1	209.217392	199.202164	196.037689	169.094513	166.952667	174.261398	208.074539	201.619186	219.320267	0.098227217	-0.096861488	0.011364238	0.149378131
ENSG00000105398	SULT2A1	36.494064	35.810898	33.350033	66.2761	72.320808	65.915993	39.135807	36.651657	33.95977	1.89E-66	1.100146403	0.102732243	0.149395915
ENSG00000167904	TMEM68	3.795133	5.019944	4.13738	4.001688	3.548114	3.255425	4.77894	4.18597	4.706977	0.67090278	-0.086341356	0.347391793	0.149490664
ENSG00000172046	USP19	16.421698	15.831991	15.071365	14.291697	13.869062	14.843525	15.562649	14.905395	19.140592	0.998233071	0.000370691	0.112342628	0.149533615
ENSG00000148459	PDSS1	9.507211	9.504625	11.032933	8.328165	8.139258	8.231087	10.198191	10.206719	10.576827	0.271132154	-0.152840397	0.241781926	0.149574631
ENSG00000137880	GCHFR	32.372464	33.560278	27.801033	29.282861	33.617531	34.624016	33.573406	35.298463	28.284008	0.091319701	0.204385455	0.272070733	0.149635384
ENSG00000154813	DPH3	20.327367	22.262905	23.378666	18.016952	18.267963	19.143526	22.906211	22.673321	23.14148	0.239666122	-0.102940219	0.068580799	0.14966176
ENSG00000130640	TUBGCP2	13.904279	12.053363	13.906896	11.91644	11.815173	12.941089	12.745615	13.056032	14.452255	0.59246621	0.05620833	0.091342718	0.149679324
ENSG00000243335	KCTD7	3.876398	4.4563	4.503343	3.852553	3.694575	3.256608	4.139792	4.235446	4.749311	0.413299173	-0.099333318	0.169247357	0.149828179
ENSG00000160221	GATD3A	27.273744	28.929059	23.955891	25.112828	21.931113	22.362498	28.580557	27.397929	26.750626	0.632564467	-0.059247779	0.141461375	0.149870474
NewGene_284	--	2.424584	2.843282	2.912404	2.146127	2.249373	2.03897	3.124657	3.208388	3.14992	0.042887893	-0.335157555	0.33926134	0.150091905
ENSG00000185009	AP3M1	16.03013	16.023338	16.694113	14.186153	13.843341	14.659437	17.139204	16.833405	16.36752	0.637919123	-0.041194272	0.050542229	0.150268051
ENSG00000134480	CCNH	11.87725	12.433653	14.262466	12.656998	11.519121	11.345269	13.506738	14.63859	13.986659	0.999571371	0.000101574	0.28639533	0.150335364
ENSG00000025796	SEC63	30.613928	32.695481	34.266385	35.757018	34.323312	34.546486	34.291079	32.647547	35.321358	0.0000246	0.242968781	0.026062402	0.150367566
ENSG00000094841	UPRT	1.224946	1.227399	1.014281	1.300574	1.568376	1.174824	1.139394	1.08302	1.573089	0.211994398	0.356487678	0.611930319	0.150421988
ENSG00000106665	CLIP2	15.436139	13.021737	14.449767	8.871579	10.544236	9.161981	13.247039	13.586808	17.969992	0.000000545	-0.443509708	0.181315971	0.150479103
ENSG00000021826	CPS1	5.20602	5.202924	3.655367	3.20551	2.854181	2.799607	5.369772	4.842305	4.434835	0.0000887	-0.520039973	0.326220339	0.150595265
ENSG00000175556	LONRF3	0.865789	0.975355	1.154645	0.350684	0.419754	0.612645	0.887595	1.136725	0.903309	0.005749087	-0.871228367	0.612887008	0.150637631
ENSG00000137216	TMEM63B	14.576461	12.4355	12.411779	12.013244	11.803774	10.889437	13.978096	12.77298	14.733947	0.896584156	-0.018033669	0.135226109	0.150657491
ENSG00000114956	DGUOK	45.953237	44.116874	43.756164	41.510105	45.322011	45.396347	48.36853	48.198063	45.060828	0.32823744	0.093537504	0.086107705	0.150697959
ENSG00000189056	RELN	55.663789	49.718795	49.484433	53.299286	53.459268	50.330225	51.271334	51.263486	58.95273	0.009203167	0.166082184	0.038879507	0.150811748
NewGene_3421	--	1.703173	1.5365	1.39272	1.707288	1.583135	1.367284	1.715053	1.387784	1.924004	0.727472409	0.082000094	0.439575808	0.15083254
ENSG00000138709	LARP1B	7.728956	8.456099	9.05333	9.068028	8.946199	7.768213	8.976885	7.751265	7.84748	0.011871605	0.252064367	0.165598475	0.151141895
ENSG00000101166	PRELID3B	27.602135	29.413952	30.952652	31.661931	29.708919	30.067784	31.759712	28.879283	30.395282	0.003536084	0.21301531	0.073101853	0.151244267
ENSG00000187109	NAP1L1	117.023377	100.346228	112.833774	81.965502	76.074737	70.555119	126.308762	113.895907	134.926166	4.27E-12	-0.392164751	0.023895226	0.151395737
ENSG00000176020	AMIGO3	0.286902	0.449406	0.630304	0.560831	0.274618	0.40693	0.37893	0.561752	0.477527	0.981452818	0.015932233	0.7443383	0.151432314
ENSG00000108829	LRRC59	65.482097	64.027154	66.406199	73.573842	65.330501	73.41447	69.540948	66.141809	68.354639	0.00000841	0.260545185	0.014447211	0.151518556
ENSG00000163798	SLC4A1AP	11.056669	10.254048	10.696083	10.771296	11.040514	9.888039	11.044183	10.045212	10.505028	0.387296147	0.094496172	0.114042333	0.151530313
ENSG00000135549	PKIB	49.589617	53.360088	54.809493	35.611559	34.508466	37.974047	55.251525	53.158119	54.849221	4.08E-08	-0.373023739	0.0401807	0.151634511
NewGene_1686	--	3.028837	3.824645	4.055054	4.111026	4.507569	3.755289	4.820406	3.556823	2.988788	0.155571602	0.315817222	0.567732995	0.151671336
ENSG00000120265	PCMT1	64.746921	67.63166	67.318094	58.835608	62.034656	60.053533	68.5091	71.996783	69.496414	0.759804519	0.026057972	0.039298278	0.151766001
ENSG00000141458	NPC1	10.529922	10.071761	10.333842	10.399582	10.084897	9.913936	10.413986	9.911556	11.266714	0.064741632	0.144866713	0.058951929	0.151797063
ENSG00000196262	PPIA	1339.237821	1350.727007	1271.966876	1118.181139	1199.87249	1162.267019	1394.948472	1445.469937	1348.226485	0.526534955	-0.042553934	0.046137712	0.15200905
ENSG00000120802	TMPO	80.320299	86.576327	83.322914	76.091454	71.897932	73.167144	90.139266	81.748504	92.075429	0.631051442	-0.033404178	0.015129872	0.15206837
ENSG00000169180	XPO6	28.052504	27.923833	27.908492	28.039948	25.660417	27.225371	28.493475	27.93388	32.857782	0.161649913	0.091825125	0.038525075	0.152070615
ENSG00000180233	ZNRF2	3.992255	4.039179	3.31153	4.204024	4.506568	4.378881	4.026057	3.590427	4.225248	0.005034776	0.353614193	0.280914085	0.152168772
ENSG00000169764	UGP2	52.620936	50.661525	51.226259	52.690457	50.525613	50.216161	54.99372	52.965765	55.078935	0.032308951	0.131479971	0.022165145	0.152248184
ENSG00000106723	SPIN1	30.917965	32.326916	34.56094	28.302862	26.147505	27.891525	34.250683	32.063492	35.679367	0.15624769	-0.100866327	0.028914235	0.152304327
ENSG00000173039	RELA	31.065343	29.628932	29.240771	29.38838	29.521909	29.405844	30.739833	30.115871	32.616445	0.039330309	0.141819875	0.027849796	0.152317577
ENSG00000165092	ALDH1A1	333.313988	365.066793	389.084791	370.353182	387.250176	369.978257	375.81889	370.389065	387.251156	0.001848385	0.200057017	0.02936532	0.152333455
ENSG00000049167	ERCC8	5.712519	7.329583	6.275881	5.622358	5.564654	5.593645	6.831703	6.41125	7.207479	0.891402334	-0.026814048	0.283657115	0.15233815
ENSG00000145012	LPP	8.530127	9.501907	9.163057	9.766706	9.922713	9.713612	9.919499	9.809011	10.821076	0.000955823	0.193491806	0.028313577	0.152353492
ENSG00000067182	TNFRSF1A	28.846409	28.659732	25.491402	38.036476	37.744032	38.54929	26.971055	27.306189	32.65111	2.89E-20	0.59893552	0.08835911	0.152391959
ENSG00000099377	HSD3B7	14.506376	14.320699	14.22585	21.41865	26.33835	23.75856	14.220858	14.606474	16.05726	2.2E-24	0.881187492	0.101684412	0.152539089
ENSG00000102900	NUP93	24.016028	25.299076	26.266362	19.469319	17.768871	20.293325	26.358227	26.242955	27.299902	0.000336897	-0.270399885	0.056342783	0.152563784
ENSG00000172943	PHF8	2.931139	2.822577	2.856178	3.140886	3.226731	3.322802	2.880242	2.879175	2.950691	0.003615005	0.306053177	0.190484278	0.152571639
ENSG00000158526	TSR2	14.465793	13.607798	13.15175	12.80725	11.722094	11.380275	14.498781	14.799704	13.640513	0.695091178	-0.054329327	0.181503176	0.152589139
ENSG00000100811	YY1	22.256197	22.222627	22.611083	21.977507	21.018872	22.645195	23.667955	21.742112	24.884023	0.109437093	0.097312792	0.018223399	0.152697282
ENSG00000095203	EPB41L4B	21.413222	22.856741	21.908061	33.479854	34.131403	33.528223	23.838022	21.097142	24.090155	1.05E-44	0.760193742	0.037103106	0.152714175
ENSG00000023909	GCLM	2.653069	2.803761	3.499157	4.055913	3.356179	3.529433	3.553439	2.612975	2.973739	0.000268629	0.48001532	0.365115051	0.15278576
ENSG00000021300	PLEKHB1	56.329601	51.782231	53.435241	18.447442	21.402156	19.594987	50.28964	55.869678	61.00819	5.07E-68	-1.300399008	0.077924365	0.152827825
ENSG00000099331	MYO9B	11.219346	10.926642	10.233118	9.575332	9.329154	9.162666	11.336006	10.678387	13.366882	0.241768744	-0.09642049	0.118306718	0.15286414
ENSG00000092607	TBX15	2.949261	2.982176	2.748045	3.490726	3.835128	3.603737	3.239353	2.955715	2.967005	0.0000673	0.465328424	0.284071514	0.152956984
ENSG00000105677	TMEM147	88.758575	77.369414	80.264015	65.616953	72.107799	72.726429	78.434481	90.973087	87.85814	0.354397892	-0.088434548	0.097882347	0.153038941

Gene ID	Gene name	Ctrl-1 FPKM	Ctrl-2 FPKM	Ctrl-3 FPKM	IFNγ-1 FPKM	IFNγ-2 FPKM	IFNγ-3 FPKM	Sora-1 FPKM	Sora-2 FPKM	Sora-3 FPKM	Ctrl vs IFNγ DESeq2 FDR	Ctrl vs IFNγ DESeq2 log2FC	Ctrl vs Sora DESeq2 FDR	Ctrl vs Sora DESeq2 log2FC
ENSG00000133250	ZNF414	7.650424	8.168507	7.150764	6.353476	7.923434	8.232131	9.331494	8.124895	8.951795	0.733285439	-0.055944588	0.201662149	0.15305556
ENSG00000065615	CYB5R4	2.654416	2.527721	2.629231	2.245073	2.310716	2.331567	2.638218	2.822732	2.676244	0.79752497	-0.033971514	0.146277669	0.153321071
ENSG00000185808	PIGP	2.636598	3.527301	2.548043	2.171497	3.116152	3.141539	3.686175	2.924121	3.084094	0.386455389	0.170938672	0.386790427	0.153353158
ENSG00000061936	SFSWAP	20.026635	18.326896	17.528306	18.425199	17.098977	18.460251	20.100717	17.645291	21.176555	0.29069717	0.093074288	0.080531471	0.153359716
ENSG00000185404	SP140L	0.723182	0.751807	0.696466	10.513846	8.906752	10.240862	0.74356	0.787954	0.667474	2.54E-174	3.917799559	0.646812543	0.153372966
NewGene 2905	--	1.730038	2.008021	2.065408	1.629505	2.025214	1.841544	2.021597	1.970942	1.701802	0.724027352	0.078890958	0.402468661	0.153419988
ENSG00000108592	FTSJ3	25.090626	26.189363	25.500553	25.28251	24.034331	23.461114	25.93515	26.898129	27.323961	0.335128747	0.070754709	0.029270604	0.153525968
ENSG00000134153	EMC7	42.927468	41.215629	42.61423	41.10661	40.644567	43.705416	43.896413	43.308289	44.861911	0.102000869	0.132793777	0.051036692	0.153613574
ENSG00000141867	BRD4	18.313772	17.791037	19.328584	18.28114	16.250227	17.929745	19.819381	18.965043	21.507166	0.631132258	0.042747122	0.033126567	0.153746075
ENSG00000136819	C9orf78	21.857281	22.000916	22.315149	19.408754	18.524664	19.656963	23.628778	22.238373	23.136557	0.603321742	-0.052478038	0.06567984	0.153831063
ENSG00000168671	UGT3A2	4.629087	4.591068	4.533979	2.847643	3.312364	2.681336	5.050482	4.448985	4.393607	0.000961246	-0.48191241	0.291020375	0.15385544
ENSG00000090863	GLG1	27.274459	24.32743	24.421056	24.190892	22.863056	24.369569	28.67693	26.811892	29.541348	0.428643506	0.063679063	0.032411391	0.153924128
ENSG00000100387	RBX1	42.452774	47.769039	44.144123	43.091755	43.371883	43.549614	49.117424	50.48518	40.097565	0.241382656	0.100528453	0.186362435	0.153942095
ENSG00000114993	RTKN	40.986593	38.86975	37.969904	37.929601	36.361592	35.690752	39.402642	42.293068	42.530336	0.753708896	0.029625088	0.054055736	0.15397852
ENSG00000034152	MAP2K3	22.171237	20.512173	20.782046	17.711637	17.432428	18.848022	20.801717	21.583963	23.988561	0.331918824	-0.09137101	0.065922314	0.153999797
ENSG00000182512	GLRX5	38.660896	35.654787	36.439409	38.971692	41.364989	41.322826	38.370051	36.970474	38.956699	0.0000862	0.282142886	0.046813081	0.154010661
ENSG00000128708	HAT1	49.537894	52.949632	59.07128	49.392434	44.303474	46.422336	55.637387	55.07936	58.271042	0.552011226	-0.059291453	0.065236135	0.154162346
ENSG00000033030	ZCCHC8	16.312334	15.955428	17.417541	19.353413	17.886015	16.885053	18.678466	16.726994	16.559157	0.000168847	0.298543781	0.092713517	0.154261474
ENSG00000233954	UQCRHL	1.469715	1.464066	1.351344	1.022449	1.260303	1.185272	1.556263	2.025174	0.856985	0.615427513	-0.157699354	0.658526607	0.154395725
ENSG00000117593	DARS2	24.061892	24.076064	24.369466	17.096727	14.17457	14.464189	24.540342	24.109243	25.094002	1.72E-13	-0.500800833	0.024029026	0.154411483
ENSG00000198933	TBKBP1	6.786054	6.184661	6.050038	6.971522	6.403427	5.998032	6.820169	6.137944	7.263717	0.234650764	0.13105445	0.135997223	0.154430996
ENSG00000121057	AKAP1	27.857978	27.84769	27.251111	30.49714	29.211797	30.649582	29.456404	26.754199	30.427817	0.00000192	0.262650669	0.020030513	0.154517055
ENSG00000165175	MID1IP1	13.863718	11.673772	9.800114	13.734641	15.547746	14.385204	12.121631	12.895778	13.637106	0.000000298	0.473078071	0.173147736	0.154543401
ENSG00000162734	PEA15	31.855754	33.122559	33.080831	25.052454	24.299934	24.064547	33.191753	36.233837	32.488054	0.0000167	-0.2688418	0.072075699	0.15454809
ENSG00000181191	PJA1	3.36552	3.783072	3.567442	3.806584	3.814763	3.27816	3.81727	3.687024	3.735794	0.295443448	0.169623996	0.298696605	0.154560774
ENSG00000118640	VAMP8	57.209426	60.504607	50.720556	60.509243	61.776002	62.349821	54.853848	61.633691	59.517661	0.001211599	0.274994841	0.126570771	0.154561894
ENSG00000145912	NHP2	48.280741	48.910594	44.871359	42.153897	42.35272	44.85474	49.050827	53.872186	44.01249	0.876472148	0.018835478	0.166765892	0.154616414
ENSG00000184014	DENND5A	19.209013	17.880173	18.481534	22.280313	21.590635	21.923426	19.4102	18.75763	20.890125	3.74E-12	0.385626974	0.025436824	0.154732348
ENSG00000132429	POPDC3	1.721341	1.786634	1.530845	1.453487	1.347974	1.388859	1.731808	1.619289	1.898634	0.819799385	-0.090565119	0.614716876	0.154749772
ENSG00000131153	GINS2	8.054217	9.779603	9.577711	9.056098	9.074221	8.538792	10.193233	9.589524	9.044287	0.399906704	0.106304586	0.202778601	0.154998207
ENSG00000256235	SMIM3	0.800204	0.613883	0.48269	0.53908	0.389942	0.434058	0.989932	0.494271	0.499003	0.485568092	-0.321007574	0.733029818	0.155020528
ENSG00000187630	DHRS4L2	2.594294	2.189884	2.172184	1.644682	2.183747	2.03644	2.848684	2.078842	2.604884	0.761503002	-0.106681245	0.574014926	0.155037115
ENSG00000130638	ATXN10	66.495255	64.97575	66.811497	55.40143	55.249147	54.475568	67.23475	65.136577	74.820753	0.035358881	-0.115302383	0.013597463	0.155078149
ENSG00000163468	CCT3	331.490122	335.259518	323.668057	296.912678	293.463181	304.039193	331.151426	351.390543	368.591078	0.804860179	-0.0160137	0.010221786	0.155131616
ENSG00000125863	MKKS	6.900626	6.546424	6.933295	5.220595	5.831625	5.486069	7.330306	7.180178	6.728914	0.092039987	-0.15260682	0.085796747	0.155248088
ENSG00000144597	EAF1	16.765338	16.694815	18.648692	18.582314	17.377785	18.73021	18.226361	17.126107	19.334804	0.002069815	0.21802816	0.046307387	0.155261209
ENSG00000168517	HEXIM2	6.892284	7.311175	6.699333	6.315105	6.160127	5.628443	7.376326	7.108819	9.454785	0.577762496	-0.102472466	0.312130007	0.155306752
ENSG00000137804	NUSAP1	60.948198	69.215815	65.10483	52.683418	44.740977	43.399425	67.440933	64.282128	72.978741	0.000332924	-0.294608181	0.040174578	0.155335449
ENSG00000162775	RBM15	6.0858	6.008928	5.766848	5.782191	6.00959	6.087562	5.779074	6.271095	6.455554	0.15815236	0.155520414	0.159821495	0.155351159
ENSG00000145782	ATG12	10.823631	12.611283	13.632615	13.699468	12.658473	11.981884	13.52265	12.359388	15.067431	0.226377475	0.135059706	0.154334209	0.155530037
ENSG00000155099	PIP4P2	9.880046	8.321175	9.050459	5.025492	4.848457	4.717183	8.894089	9.048383	10.769946	1.49E-09	-0.628203487	0.179466501	0.155622653
ENSG00000097033	SH3GLB1	7.669059	8.326601	8.250798	7.209047	6.852477	7.234138	9.575233	8.095812	9.015438	0.781743957	-0.028469551	0.051829238	0.155648219
ENSG00000177628	GBA	56.160689	55.271406	50.209907	45.976971	44.247111	44.674085	56.482685	56.915361	55.951993	0.080703786	-0.120183326	0.036741239	0.155702421
ENSG00000103335	PIEZO1	13.923114	13.373607	14.554812	11.889863	9.874883	9.915542	13.935034	14.134158	17.979175	0.0000614	-0.299167166	0.094232617	0.155720606
ENSG00000066455	GOLGA5	14.099457	12.305178	12.331143	15.106067	14.581936	13.834357	13.226994	12.6846	14.798475	0.000248584	0.316439804	0.110903124	0.155874822
ENSG00000131507	NDFIP1	13.521297	15.332504	15.13199	24.889414	24.556805	24.827227	15.598795	14.590806	15.759484	4.15E-46	0.903861285	0.061041693	0.155909254
ENSG00000100644	HIF1A	48.377121	48.549722	56.475807	45.718348	46.572079	43.670493	50.20069	50.923279	59.444295	0.797959614	-0.025773321	0.066164681	0.155926049
ENSG00000173442	EHBP1L1	8.031146	7.273145	7.084292	6.75515	6.831228	5.971985	7.496247	7.381167	8.548203	0.684459843	-0.050431246	0.103490377	0.155933402
ENSG00000205629	LCMT1	14.845911	14.97969	13.536313	12.56109	13.127019	12.787217	15.619521	14.034854	15.476495	0.891354098	-0.021397854	0.163520077	0.155955347
ENSG00000102760	RGCC	2.169927	1.837267	3.293416	1.0063	0.80469	0.680067	2.099622	2.706	2.786097	0.000102994	-1.38945767	0.650630051	0.155956222
ENSG00000170498	KISS1	4.506625	3.545612	3.787321	3.795023	3.514531	3.44817	4.366818	3.652367	4.489379	0.862457111	-0.066158654	0.581387275	0.155996994
ENSG00000129083	COPB1	74.680859	75.44337	74.360683	83.727527	80.457307	82.438795	76.854395	75.824495	81.872128	7.66E-10	0.280543413	0.005877907	0.156003852
ENSG00000141012	GALNS	3.855403	3.731495	4.163232	3.301949	3.282782	2.832334	3.678134	3.68175	4.624617	0.343671765	-0.16919926	0.327873708	0.156041582
ENSG00000177469	CAVIN1	6.750627	6.612272	6.753586	3.322096	3.742883	3.994334	6.993363	6.507421	7.550399	1.78E-12	-0.712635426	0.109603854	0.15609465
ENSG00000125868	DSTN	24.46121	28.728542	31.165267	23.025278	25.035095	21.45986	31.936528	24.555385	31.363781	0.077596862	-0.171507528	0.109663636	0.156117057
ENSG00000170558	CDH2	18.393921	18.403534	18.192711	13.722635	12.417372	12.955876	19.675814	17.779172	20.111692	3.41E-08	-0.346596037	0.026418337	0.156134466
ENSG00000006468	ETV1	6.39521	6.535005	8.181466	9.403957	9.676458	10.186045	7.384084	6.442911	7.571428	1.11E-17	0.630448104	0.091479282	0.156159435
ENSG00000090924	PLEKHG2	14.194335	12.947851	13.71542	10.775145	9.271202	9.24169	14.090398	13.563132	16.772282	0.0000205	-0.313531146	0.089263666	0.156265631
ENSG00000136319	TTC5	2.455217	2.975806	2.710193	2.690842	2.399831	2.61041	2.807694	2.91772	2.992082	0.714186459	0.06252061	0.250446731	0.156273284
ENSG00000181163	NPM1	582.287658	609.927792	634.971993	525.118127	492.232444	503.80086	647.771361	642.694459	638.812467	0.032594046	-0.117032988	0.011922091	0.156539614
NewGene 3595	--	9.361264	11.034111	10.223121	10.813617	11.001338	11.276752	12.629586	13.764857	14.848881	0.598666832	0.069074129	0.157597704	0.15660423
ENSG00000122126	OCRL	13.492417	12.416298	13.89522	12.872373	11.814723	12.527702	13.733066	13.391659	14.467437	0.654890292	0.043213173	0.045286605	0.156785478
ENSG00000119950	MXI1	3.267775	3.651352	3.857579	3.079991	3.438877	3.43404	3.998034	3.770959	4.289893	0.884511742	-0.030133003	0.267028539	0.156792656
ENSG00000234127	TRIM26	54.459343	43.641005	49.29085	71.717166	64.401281	59.585346	45.28598	51.135774	47.661305	3.31E-31	0.601643367	0.016844753	0.156825881
ENSG00000106686	SPATA6L	0.824812	1.089337	1.126932	0.918281	0.507925	0.542543	1.409249	1.033232	0.814055	0.152801298	-0.530611176	0.66074749	0.156842285
ENSG00000100403	ZC3H7B	17.134064	17.155876	15.069128	21.973372	20.469919	20.945377	16.464008	16.938076	18.221945	1.67E-15	0.507651008	0.048804471	0.156862336
ENSG00000172380	GNG12	6.603748	7.007931	7.389715	4.363336	4.437835	4.746885	7.313663	6.37168	8.324724	0.000000107	-0.483383272	0.146379481	0.156915907
ENSG00000075142	SRI	22.290938	23.210033	21.568773	21.616494	24.11922	22.350581	26.382089	21.845818	21.29881	0.687029379	0.04264251	0.080478883	0.156998803
ENSG00000181929	PRKAG1	21.898342	22.929061	19.788745	16.940378	18.158662	17.352742	21.535218	22.967886	22.306342	0.151332036	-0.143893886	0.11727341	0.157009627
ENSG00000101871	MID1	3.301935	3.511234	3.698422	3.43439	3.172885	3.626333	3.776436	3.301602	3.970379	0.346732108	0.112582385	0.15559719	0.15702808
ENSG00000133027	PEMT	27.80569	26.535354	24.17256	24.650712	27.734432	27.839791	28.295894	26.431465	27.425741	0.098381425	0.180918649	0.12725854	0.157102507
ENSG00000163602	RYBP	3.598172	4.045126	4.261271	3.553675	3.49927	3.193499	4.371778	4.058067	4.005164	0.592824976	-0.068604845	0.146222252	0.157137588
ENSG00000175581	MRPL48	23.611711	20.532201	22.518439	21.44111	18.837328	20.540343	25.173757	26.025861	27.921351	0.173522671	-0.147948586	0.136929373	0.157159708
ENSG00000006715	VPS41	18.137398	18.267095	18.751221	14.632421	15.108543	14.699661	18.602891	20.222059	22.464518	0.025399621	-0.141806557	0.019035964	0.157199429

Gene ID	Gene name	Ctrl-1_FPKM	Ctrl-2_FPKM	Ctrl-3_FPKM	IFNγ-1_FPKM	IFNγ-2_FPKM	IFNγ-3_FPKM	Sora-1_FPKM	Sora-2_FPKM	Sora-3_FPKM	Ctrl_vs_IFNγ_DESeq2_FDR	Ctrl_vs_IFNγ_DESeq2_log2FC	Ctrl_vs_Sora_DESeq2_FDR	Ctrl_vs_Sora_DESeq2_log2FC
ENSG00000175602	CCDC85B	5.444078	5.179876	4.970633	5.554733	5.406054	7.043277	4.537758	5.871495	5.90341	0.030357271	0.35749002	0.377036347	0.157558626
ENSG00000267368	UPK3BL1	3.405536	3.090569	11.574582	10.831339	4.947392	11.51259	4.726383	5.639611	8.645082	0.447638222	0.737482722	0.859438118	0.157688273
ENSG00000096717	SIRT1	5.873563	6.371035	6.814949	6.056698	6.031442	5.413928	6.770187	6.16803	7.201397	0.946098761	0.010752182	0.142613353	0.157716106
ENSG00000134759	ELP2	14.143174	12.419766	13.626689	10.185052	17.486904	13.485274	11.64138	17.399673	15.394167	0.538626878	-0.051010716	0.031553661	0.157753762
ENSG00000100150	DEPDC5	2.6575	2.350351	2.678718	2.219785	2.448531	2.361126	2.863447	2.520325	2.94267	0.862638124	0.030397045	0.248635525	0.157938873
ENSG00000154518	ATP5MC3	28.70611	27.766725	30.156646	24.694499	28.967478	25.360695	27.814166	28.132399	28.072421	0.691467427	-0.036374554	0.028710751	0.157975255
ENSG00000172336	POP7	43.084783	39.001752	36.759999	37.959129	36.517522	37.578642	40.423082	42.712578	41.125095	0.588430787	0.061397551	0.114090312	0.158028747
NewGene_2150	--	24.512259	24.996317	26.935575	15.832972	15.403961	15.542352	27.056215	27.6828	25.112146	8.49E-20	-0.560869117	0.064280146	0.158129791
ENSG00000111667	USP5	39.285129	38.754	34.475432	29.606541	29.104091	32.256266	37.5011	37.60878	42.727736	0.045737002	-0.157674393	0.046759693	0.158178526
ENSG00000135045	C9orf40	5.838417	5.660177	5.866007	4.890628	5.172565	5.337466	6.249072	5.870219	6.048796	0.891995645	-0.024221754	0.207516429	0.15819757
ENSG00000105220	GPI	89.84482	75.181043	74.408142	64.999332	61.929722	67.134162	82.528355	89.261141	90.05073	0.000417427	-0.205047217	0.031075761	0.158197847
ENSG00000118257	NRP2	1.040398	1.294112	1.357468	0.540937	0.782545	0.708924	1.281436	1.253169	1.394838	0.001060749	-0.656449634	0.406698911	0.158199929
ENSG00000148803	FUOM	16.801457	17.374795	16.351295	12.864707	16.133178	15.881588	18.434925	18.776342	16.099911	0.936280995	-0.017037221	0.267855649	0.158267128
ENSG00000132182	NUP210	46.420113	45.446148	41.246765	44.464752	39.142269	40.929771	44.168121	43.57848	51.785599	0.538626878	0.050339385	0.036136199	0.158273748
ENSG00000018189	RUFY3	12.116475	13.291209	13.482333	11.093456	10.81702	12.16879	16.206954	14.202045	14.26265	0.295429776	-0.100779886	0.070095161	0.158311438
ENSG00000170631	ZNF16	4.187298	4.519081	4.261647	4.068795	3.877158	3.568128	4.559254	5.143347	3.836078	0.889945453	-0.027437909	0.343680288	0.15831927
ENSG00000172977	KAT5	37.768251	38.027678	36.075326	39.234269	37.719027	37.541905	36.233568	37.390006	38.961492	0.030074737	0.146112429	0.031121037	0.158367797
ENSG00000109103	UNC119	15.389233	11.781768	11.232837	9.848888	11.301318	10.350203	12.331414	12.436411	14.073724	0.487961229	-0.089569983	0.1395679	0.158506119
ENSG00000173757	STAT5B	7.040906	7.1074	7.340553	6.584994	6.003344	6.491139	7.34883	6.942985	8.218962	0.834621204	-0.02434586	0.075709859	0.158519014
ENSG00000102103	PQBP1	34.032524	28.927038	30.083053	32.630541	29.945945	34.522326	29.893426	33.64501	32.938472	0.039088671	0.210634855	0.13717366	0.158550639
ENSG00000103202	NME4	90.969441	87.633113	80.126448	82.821632	87.047479	89.066745	89.04663	92.104987	89.629873	0.035220864	0.160084145	0.047732041	0.158680397
ENSG00000174943	KCTD13	4.286339	3.676857	3.620298	3.77618	3.290907	3.465421	3.801873	3.781455	4.04149	0.930512049	0.019815553	0.305606347	0.158835405
ENSG00000076555	ACACB	0.749647	0.65518	0.725519	1.211369	1.082039	0.939653	1.213049	0.845493	0.706162	0.00000418	0.713520772	0.426207098	0.158869797
ENSG00000136144	RCBTB1	4.076181	4.733121	5.09481	3.756538	3.788518	3.513662	4.559447	4.471341	5.525373	0.154884321	-0.188640979	0.228530484	0.158880814
ENSG00000179335	CLK3	13.604368	10.211523	10.137413	10.639992	10.550187	8.709375	12.881529	13.655103	13.59762	0.864192776	0.024018542	0.108491191	0.158932346
ENSG00000103199	ZNF500	1.672828	1.603953	1.51779	1.402968	1.188994	1.457103	1.524723	1.604747	1.84335	0.457113775	-0.143015589	0.304477323	0.158949641
ENSG00000168522	FNTA	42.591506	45.398809	45.738779	33.785408	34.351062	32.617878	50.53774	52.179036	51.99192	0.005934189	-0.178179911	0.025902349	0.158955716
ENSG00000169976	SF3B5	101.137558	91.59079	81.695503	80.190323	78.059593	91.382568	89.280235	100.104813	97.822777	0.940013734	0.011037671	0.114119557	0.159016981
ENSG00000171723	GPHN	3.244755	3.728143	3.576337	3.745069	3.265572	3.546688	3.841489	3.458658	3.730255	0.258716682	0.150698322	0.201827721	0.159079994
ENSG00000102580	DNAJC3	10.489862	10.886302	11.043773	15.433559	14.798034	14.895704	11.775717	10.701683	11.475009	5.29E-27	0.6243658	0.037145414	0.159174626
ENSG00000179841	AKAP5	0.548522	0.612278	0.828045	0.362378	0.438991	0.525125	0.652638	0.569908	0.555186	0.36526347	-0.260352055	0.517300494	0.159284591
ENSG00000135002	RFK	10.567755	12.090121	12.958958	11.316019	10.059625	11.322748	12.332521	12.069274	12.88438	0.861392209	0.025440291	0.125487669	0.159320075
ENSG00000130876	SLC7A10	3.934763	2.965731	4.213323	2.771415	4.039704	3.559945	4.018872	3.169724	4.495604	0.798752291	0.071777812	0.443746134	0.159324203
ENSG00000103485	QPRT	137.778311	129.080707	121.093568	116.128238	126.077247	128.886857	134.626205	134.98065	136.730625	0.2953168	0.084411594	0.021261568	0.159421868
ENSG00000162736	NCSTN	64.88256	59.109642	59.992963	56.122846	54.950828	53.4913	62.298752	64.876378	66.26933	0.752382676	-0.026054073	0.019536272	0.159489712
ENSG00000101417	PXMP4	2.893109	3.015055	2.868344	2.586101	2.714278	2.956664	3.343208	3.13899	2.860741	0.744017431	0.052208017	0.202336102	0.159499696
ENSG00000132522	GPS2	37.816328	38.356727	38.231857	39.796247	33.340652	31.598845	34.195739	42.498734	44.117954	0.941507567	0.009685222	0.136347081	0.159606816
ENSG00000164187	LMBRD2	2.482979	3.163716	3.098262	3.106599	3.545343	3.324455	2.92897	2.767472	3.478243	0.003053039	0.339071344	0.214024197	0.159629065
ENSG00000198612	COPS8	10.842726	11.356765	11.34496	8.738993	8.311009	9.099902	10.851793	11.203805	10.755606	0.018758768	-0.200970289	0.060936226	0.159718482
ENSG00000087191	PSMC5	88.488606	93.374657	99.233648	84.855647	80.681494	81.756032	93.656079	96.481882	100.780721	0.74303067	-0.025963954	0.018632946	0.159858677
NewGene_6646	--	1.097445	0.646272	0.926402	0.714301	0.496433	0.532216	0.809282	1.036163	0.861565	--	--	0.737642005	0.159892407
ENSG00000124795	DEK	145.863071	164.394667	177.137233	161.46989	149.660771	158.400426	173.11058	161.392915	183.39606	0.214643132	0.098987296	0.041501507	0.159903953
ENSG00000139211	AMIGO2	3.098266	2.69363	2.818077	1.961786	1.886896	2.117077	3.045708	2.890325	2.620808	0.011871605	-0.386591228	0.299720628	0.159916453
ENSG00000083457	ITGAE	2.786046	3.130656	2.831099	2.005424	2.122067	1.85856	3.13834	3.184025	2.830113	0.004764772	-0.401406777	0.277854766	0.159973325
ENSG00000181045	SLC26A11	5.174621	5.43361	4.949099	5.273942	4.974581	4.764179	5.791157	5.004784	5.70722	0.528064488	0.09240486	0.209841463	0.15998301
ENSG00000132581	SDF2	28.184614	28.114849	26.818674	23.335547	26.067792	25.249431	26.350256	28.627912	30.935361	0.73517571	-0.038241744	0.079480558	0.160156693
ENSG00000177311	ZBTB38	26.107443	25.850355	28.262902	13.969594	16.587963	12.956594	19.672125	27.375071	22.584356	0.000128844	-0.302087546	0.044667855	0.160331937
ENSG00000114354	TFG	120.755558	123.220581	136.48152	153.18807	148.832889	151.245597	132.831336	127.69603	135.033589	1.48E-19	0.422181515	0.007786197	0.160409341
ENSG00000126953	TIMM8A	4.159107	4.925279	4.25206	3.59424	3.730707	3.844292	5.002914	4.809923	4.575305	0.65734144	-0.084566179	0.286874482	0.160604581
ENSG00000174080	CTSF	4.349464	4.257884	3.417598	3.496935	3.101521	4.064288	3.980895	3.703857	4.713531	0.95987988	-0.013673135	0.356077006	0.16062163
ENSG00000082212	ME2	1.685508	1.817935	3.973121	3.426591	5.985458	2.147017	3.237209	1.843447	1.997787	0.00047043	0.37190623	0.204705398	0.160739746
ENSG00000026950	BTN3A1	2.93127	3.474202	3.450319	23.074012	19.666151	19.512792	3.70886	3.504942	3.490736	6.74E-197	2.760382149	0.314263121	0.160853791
ENSG00000167770	OTUB1	71.49518	64.369725	64.574316	58.124864	57.020779	61.939343	68.685522	67.428358	68.854774	0.700848952	-0.036189065	0.023922072	0.160890474
ENSG00000169122	FAM110B	2.200677	2.352118	2.210611	2.186597	2.248391	2.149878	2.448401	2.085724	2.568676	0.565009014	0.108539677	0.329420919	0.160899714
ENSG00000112983	BRD8	16.995816	16.594862	18.925056	14.255955	15.252049	13.940718	18.87319	17.601406	19.107079	0.192531727	-0.112480656	0.046168214	0.160976853
ENSG00000158623	COPG2	17.82316	16.976055	15.408898	14.963396	13.559038	14.135363	17.380445	17.550378	17.705347	0.355858472	-0.089405929	0.064711478	0.16104221
ENSG00000119689	DLST	29.950833	27.328089	27.292411	25.395086	25.145729	23.666453	28.936178	28.082752	32.053304	0.636109696	-0.044547191	0.038758776	0.161054673
ENSG00000105499	PLA2G4C	3.481976	3.221373	2.792434	1.027487	1.008211	1.14846	2.636708	3.110139	3.573757	1.85E-14	-1.524880417	0.404579167	0.161078984
ENSG00000166275	BORCS7	9.331182	11.763272	11.247471	10.831732	10.777238	12.350652	11.463364	10.028829	11.884606	0.052743632	0.22307347	0.16547335	0.161243706
ENSG00000154556	SORBS2	4.266464	2.952396	3.52583	2.940171	2.996708	3.291163	4.114501	3.108637	3.596918	0.758556721	0.072336376	0.439470089	0.161253886
ENSG00000141551	CSNK1D	49.474217	44.178645	43.788702	42.164977	41.560607	43.858711	51.130764	49.049734	56.12718	0.891402334	0.01291935	0.029383718	0.16138187
ENSG00000117475	BLZF1	7.187397	7.600565	7.973781	9.31093	8.187129	8.242591	7.709334	7.914252	7.948219	0.000650248	0.342753096	0.141567314	0.161439516
ENSG00000109536	FRG1	17.254061	16.63629	18.082273	18.585292	21.580381	18.935854	18.9463	19.739205	15.763993	0.002061671	0.333786347	0.23882279	0.161459951
ENSG00000106538	RARRES2	116.563066	106.887321	98.851894	107.88544	121.16765	118.783313	101.873226	114.212173	106.86696	0.0000143	0.335705535	0.074036621	0.161528105
ENSG00000118418	HMGN3	68.522942	70.681935	76.482504	84.2983	85.81797	88.146031	77.748695	74.137016	74.550572	8.63E-10	0.41171158	0.0490502	0.161566965
ENSG00000117797	RALBP1	11.294692	11.049226	10.612899	10.884375	10.55405	9.800856	12.059069	11.160906	11.886988	0.889442145	0.016603913	0.043245649	0.161773752
ENSG00000272333	KMT2B	6.317867	5.983841	5.901121	7.334705	5.978631	6.424939	6.374459	5.343566	7.451335	0.001716362	0.262292112	0.122710462	0.161859443
ENSG00000111554	MDM1	5.178191	5.087215	4.809157	3.873284	3.99109	4.333186	5.797726	4.92822	5.292966	0.214544923	-0.168817517	0.200524995	0.16204634
ENSG00000176148	TCP11L1	2.637557	2.27552	2.056696	2.513704	1.759528	1.962508	2.34521	2.255778	2.307851	0.860111892	0.047089214	0.368229562	0.162336899
ENSG00000135624	CCT7	181.62683	174.356155	169.047603	142.390789	145.186156	143.709753	173.470805	183.680445	193.714442	0.020486859	-0.1306479	0.012186813	0.162387729
ENSG00000137203	TFAP2A	1.961995	1.72838	1.974708	3.493169	3.272577	3.668813	1.757401	1.709868	1.941318	1.4E-10	0.871826692	0.368352805	0.162483195
ENSG00000140545	MFGE8	9.782968	7.727685	7.541064	5.717261	6.188762	5.764267	8.353377	8.068941	9.601736	0.004304518	-0.40208063	0.262325526	0.16259046
ENSG00000197006	METTL9	64.231313	63.574156	62.126391	54.367939	57.765864	53.946833	66.48515	70.469023	68.38965	0.075562191	-0.117116862	0.010724085	0.162816061
ENSG00000244274	DBNDD2	33.061185	31.990668	35.577413	25.799017	28.507181	28.560236	32.404817	37.515196	36.705591	0.221591528	-0.12460313	0.104371588	0.16282692

Gene ID	Gene name	Ctrl-1 FPKM	Ctrl-2 FPKM	Ctrl-3 FPKM	IFNγ-1 FPKM	IFNγ-2 FPKM	IFNγ-3 FPKM	Sora-1 FPKM	Sora-2 FPKM	Sora-3 FPKM	Ctrl vs IFNγ DESeq2 FDR	Ctrl vs IFNγ DESeq2 log2FC	Ctrl vs Sora DESeq2 FDR	Ctrl vs Sora DESeq2 log2FC
ENSG00000074356	NCBP3	5.627196	5.152447	5.338756	5.52903	4.714859	4.901668	6.241792	5.193893	6.191516	0.398946277	0.069275796	0.048255823	0.162831895
ENSG00000145703	IQGAP2	24.817498	26.541261	25.648526	24.812918	24.517227	24.223896	27.460346	25.374344	28.025345	0.19784544	0.08167237	0.011064676	0.162880393
ENSG00000100804	PSMB5	157.516022	150.096711	148.420816	130.203295	137.165767	131.255979	151.369672	171.654283	144.501468	0.745077666	-0.027178924	0.063930528	0.16305184
ENSG00000169139	UBE2V2	25.516446	27.584652	27.940003	28.703071	26.579881	25.019967	35.085173	27.308805	29.521746	0.379509794	0.080414082	0.052410709	0.163080704
ENSG00000162390	ACOT11	0.503261	0.817407	0.609329	0.289795	0.219709	0.32973	0.622259	0.823133	0.501984	0.005614708	-0.994881547	0.645034427	0.163167132
ENSG00000117862	TXNDC12	39.486717	36.927911	35.870391	26.645777	27.249086	26.702724	38.309046	42.003023	37.350204	0.000000403	-0.329822401	0.064249138	0.163189727
ENSG00000124570	SERPINB6	41.092929	38.000211	37.648286	36.949616	38.524475	39.223329	42.615405	38.830241	44.535111	0.052609981	0.155667077	0.039800552	0.163328693
ENSG00000140990	NDUFB10	37.536324	42.372552	35.585461	39.225888	41.718075	42.03763	41.317943	41.479492	35.605827	0.018124526	0.238308847	0.186871927	0.163422302
ENSG00000170175	CHRNB1	6.123227	5.77037	5.134069	5.832008	4.754994	5.668084	5.597844	5.884173	6.017925	0.887488995	0.029826487	0.267382828	0.163427529
ENSG00000106384	MOGAT3	2.683642	2.763259	1.851784	1.507252	1.518685	1.306846	3.329823	2.273479	2.344286	0.004440085	-0.653522344	0.507837785	0.163436252
ENSG00000125651	GTF2F1	49.087347	46.199996	46.369164	42.153503	41.950069	43.40823	49.424748	47.836322	51.643417	0.975935104	-0.002997416	0.011254622	0.163508593
ENSG00000132382	MYBBP1A	20.472802	20.81558	22.001634	18.348551	14.877894	17.913375	22.145824	20.964745	24.407662	0.027662516	-0.17311331	0.028526527	0.163538937
ENSG00000119725	ZNF410	15.322366	15.097982	14.146671	15.857481	14.329848	14.05192	15.100176	16.741726	16.027373	0.495722162	0.082816723	0.155921865	0.163615861
ENSG00000245848	CEBPA	175.781357	186.705154	166.25267	183.185791	192.833847	198.237488	189.734772	175.480484	190.357437	0.00000873	0.267165111	0.01130575	0.163630995
ENSG00000169857	AVEN	13.933683	14.865932	15.096566	11.131488	12.082254	11.831107	15.140073	14.407723	16.613943	0.106915848	-0.176376262	0.108311883	0.164186406
ENSG00000128165	ADM2	2.757274	2.731508	2.719258	3.800151	3.982457	3.644522	2.969294	2.25811	3.446736	7.76E-09	0.624774849	0.307350062	0.164188516
ENSG00000141448	GATA6	3.5733	3.626196	3.299335	2.649217	2.565427	2.339508	3.992086	3.228258	3.886095	0.011828284	-0.331481448	0.221536895	0.164232307
ENSG00000125812	GZF1	5.690029	5.824142	6.409314	6.100223	5.989471	5.835183	6.327997	5.719782	7.012554	0.08042452	0.166576297	0.097882347	0.164264096
ENSG00000115761	NOL10	16.479105	16.326816	17.648488	16.937224	15.456151	16.513465	17.331522	16.897466	19.033565	0.256370056	0.101382495	0.049382249	0.164351294
ENSG00000177239	MAN1B1	21.744829	22.158335	20.070062	18.106876	14.605443	16.646351	21.273319	21.609227	24.816291	0.002837414	-0.252347056	0.070351499	0.164392815
ENSG00000141499	WRAP53	14.439048	13.382744	12.791931	10.374107	10.822724	10.808727	11.651175	12.027206	14.909606	0.075554224	-0.174264886	0.089798469	0.164530349
ENSG00000145337	PYURF	25.436193	27.473867	25.161154	27.177444	26.25049	28.074568	26.950449	27.790459	27.255873	0.012816667	0.209779271	0.072081905	0.164675558
ENSG00000092531	SNAP23	34.892686	36.057648	37.083452	30.548633	30.562617	30.529363	39.340641	34.717677	39.95428	0.034157983	-0.139392259	0.020746464	0.164731997
ENSG00000180198	RCC1	36.727347	41.071153	41.35919	32.857515	28.615203	29.676931	41.82472	39.429089	42.124117	0.000790153	-0.2370196	0.028561746	0.164738949
ENSG00000138623	SEMA7A	2.583106	2.911412	2.619291	1.146337	1.482484	1.07316	2.377233	3.032891	3.354202	3.64E-11	-1.081208525	0.330994571	0.164747582
ENSG00000156787	TBC1D31	3.893242	4.008137	4.024647	3.825821	3.418331	3.03541	4.307883	4.083562	4.116854	0.654497365	-0.077470742	0.200077913	0.164818627
ENSG00000188706	ZDHHC9	14.443046	13.363561	13.347937	13.887606	13.06563	13.830451	14.357766	14.573079	14.896051	0.077523332	0.133743019	0.037145414	0.164915137
ENSG00000126012	KDM5C	14.98023	14.894338	13.740868	15.209649	14.330065	14.054686	14.740127	14.104474	16.764255	0.164647845	0.104448846	0.035950863	0.164943781
ENSG00000172301	COPRS	33.062518	34.029765	31.960874	24.788935	24.682199	24.760316	36.937357	35.032349	32.503804	0.001867702	-0.269440137	0.099488249	0.164973864
ENSG00000056972	TRAF3IP2	0.64561	0.692368	0.548665	0.650304	0.543886	0.770914	0.681198	1.194843	0.606818	0.68791148	0.126664986	0.530849562	0.165021932
ENSG00000158864	NDUFS2	103.642063	106.848344	108.777583	88.710948	86.03663	91.418541	105.899366	111.68984	118.062727	0.048182127	-0.114655193	0.010879762	0.165069997
ENSG00000066422	ZBTB11	7.949616	8.969668	10.209334	9.317499	9.679435	9.503911	9.544359	8.984855	9.941855	0.021792236	0.217014461	0.097124106	0.165076115
ENSG00000130066	SAT1	69.119546	68.53859	73.275599	89.751975	96.368934	89.728874	72.939223	76.242816	73.090952	8.49E-17	0.5284443	0.038890931	0.165209958
ENSG00000142657	PGD	100.571685	96.871302	94.735244	86.881729	83.538838	88.83378	101.811452	102.961343	104.489582	0.697322753	-0.029757024	0.00914038	0.16533667
ENSG00000134056	MRPS36	11.391651	12.406787	13.916367	10.838394	11.499095	11.107332	14.019311	13.962563	11.524304	0.887249822	-0.025412599	0.256880494	0.165344358
ENSG00000160691	SHC1	44.765933	45.757068	47.899017	38.465154	35.733379	36.320687	46.305266	46.400841	51.753425	0.001039699	-0.196308873	0.014262498	0.165407975
ENSG00000158863	FAM160B2	4.539908	4.915833	4.392482	4.408051	3.695293	4.251489	4.888947	4.681522	4.956444	0.982392413	-0.003895408	0.123181098	0.165430765
ENSG00000188243	COMMD6	5.246363	3.294184	3.691558	2.214169	3.017609	3.473659	2.46134	4.709095	2.795211	0.774270945	0.09847258	0.577011534	0.165664783
ENSG00000089009	RPL6	1262.773732	1197.667652	1200.985517	802.151425	797.944306	801.667737	1240.963863	1279.908475	1264.808683	3.12E-18	-0.415300236	0.009438239	0.16567349
ENSG00000169599	NFU1	15.731853	16.176057	16.373543	15.383151	14.576887	14.167884	18.799939	16.659767	16.85544	0.77383775	-0.038377578	0.098944984	0.165682291
ENSG00000149100	EIF3M	45.366271	45.865053	52.112975	44.207312	43.865676	43.965708	60.393676	54.935181	53.425369	0.509363993	0.048259027	0.012118011	0.165748729
ENSG00000151923	TIAL1	20.982824	23.843544	24.850583	23.441579	21.285387	19.721762	25.561193	23.848878	26.641303	0.83645645	0.025811214	0.079106844	0.165808442
ENSG00000168781	PPIP5K1	2.033161	2.394607	2.169653	1.297906	1.57214	1.597503	2.3902	2.448484	2.468373	0.001165755	-0.455475076	0.224862053	0.16590174
ENSG00000177302	TOP3A	15.336696	14.090767	19.380668	13.499231	13.755883	12.434397	17.470429	14.000801	15.557792	0.832611806	0.023430045	0.04875932	0.165977878
ENSG00000160712	IL6R	2.301683	1.984095	2.0805	4.394897	4.585864	4.025601	2.273859	2.130734	2.145731	1.43E-31	1.194250516	0.228992012	0.166002819
ENSG00000105063	PPP6R1	24.99168	23.217177	21.260406	21.889698	21.675287	22.606366	23.73757	21.983666	27.580036	0.407965364	0.073978039	0.066261458	0.16604878
ENSG00000171603	CLSTN1	17.036385	17.164852	17.726328	15.847665	15.776518	15.474311	18.622524	17.847847	19.2923	0.728238034	-0.029854678	0.019998706	0.166093298
ENSG00000204149	AGAP6	7.380311	8.839242	9.118565	8.997882	7.020367	7.817568	7.731085	8.074509	10.616442	0.612869226	0.079174746	0.233413494	0.166115518
ENSG00000233024	NPIPA9	10.828925	10.03739	11.039075	10.516258	7.512539	7.926948	10.626011	9.924282	12.152501	0.615247691	-0.087218945	0.191061122	0.16617042
ENSG00000166689	PLEKHA7	20.362196	18.759242	18.534947	16.186785	14.182114	15.646515	20.164374	19.086423	21.272702	0.05143833	-0.153120066	0.024197077	0.166204644
ENSG00000087365	SF3B2	240.920223	235.330595	244.009305	237.268701	231.907908	229.968036	254.822276	247.147666	248.828676	0.012552179	0.122600924	0.004594081	0.166284508
ENSG00000280670	CCDC163	2.505907	2.8292	2.141953	3.501866	2.869617	2.891154	2.905022	2.562893	2.623988	0.021956512	0.441866274	0.438386835	0.166317988
ENSG00000110925	CSRNP2	7.698003	7.29439	7.663405	5.384868	5.772014	5.180909	8.096875	8.11846	8.508997	0.0000902	-0.355989666	0.058489225	0.16637386
ENSG00000140829	DHX38	15.030888	14.886307	14.864877	14.190586	12.853593	13.952161	14.930705	14.28171	18.534319	0.820690829	0.021823186	0.070570315	0.166402454
ENSG00000204435	CSNK2B	269.463704	257.283156	277.591246	248.408109	239.786695	244.448365	282.469193	283.890051	283.612054	0.814836743	0.018034298	0.009906301	0.166430916
ENSG00000128607	KLHDC10	5.22034	5.80106	6.040982	5.985944	5.70323	5.406721	6.014234	5.49653	6.478421	0.128484377	0.147206605	0.083070998	0.166451462
ENSG00000112304	ACOT13	14.852907	15.068698	14.379366	14.556868	14.556041	13.643885	15.953948	16.180003	14.464004	0.221225362	0.096129798	0.065585067	0.166507388
ENSG00000179094	PER1	3.607132	3.186847	3.126462	3.021787	3.606988	3.205209	3.209	3.444441	4.016015	0.442221571	0.116764885	0.204263426	0.166529679
ENSG00000138796	HADH	21.873831	21.981427	22.657306	23.152475	24.243826	21.964403	23.362288	22.359987	25.561859	0.005502991	0.207747867	0.044457025	0.166557136
ENSG00000072786	STK10	0.98198	0.974644	0.941778	0.650376	0.745215	0.784308	0.817632	0.829848	1.349091	0.262802336	-0.238228639	0.464559542	0.166580218
ENSG00000024422	EHD2	2.695031	2.736574	2.79015	1.687918	1.27636	1.453509	2.780596	2.75367	3.158469	0.000000861	-0.745859268	0.249214723	0.1666411
ENSG00000164180	TMEM161B	5.933905	6.926591	6.380432	6.367052	5.688218	6.271136	6.215666	5.958854	6.710298	0.146492356	0.154401858	0.109981421	0.166771346
ENSG00000049759	NEDD4L	25.236444	26.406433	22.99523	19.612967	19.742413	17.168469	29.201338	34.440476	28.827531	0.946787882	-0.006543958	0.010547133	0.166777391
ENSG00000130202	NECTIN2	70.776029	64.895875	64.595097	62.109232	66.793524	66.830688	66.96484	67.636236	76.300914	0.158229386	0.107369071	0.027612151	0.166827996
ENSG00000179152	TCAIM	8.790425	11.454202	10.651949	9.148083	9.324823	9.324268	10.431029	9.431525	11.282072	0.78013289	0.04237126	0.176270719	0.166973749
ENSG00000106004	HOXA5	2.338931	3.019659	3.170976	2.720538	3.243618	2.730152	3.271368	3.220084	2.444233	0.480482584	0.176378431	0.488963393	0.167050615
ENSG00000114302	PRKAR2A	5.832567	6.094234	5.803316	5.3995	5.555694	5.63479	6.551004	6.047466	6.1312	0.618596415	0.051772752	0.056890392	0.167098291
ENSG00000101182	PSMA7	150.0706	144.458006	142.430127	127.209045	133.109113	137.662502	152.364229	154.799981	152.441539	0.88891228	0.013177933	0.013050265	0.16711465
ENSG00000146038	DCDC2	32.685176	37.951	40.183965	21.907344	20.717895	22.06183	38.90355	35.653096	42.248567	7.26E-19	-0.633185688	0.054367627	0.167133628
ENSG00000141431	ASXL3	0.105575	0.214164	0.155953	0.063701	0.070069	0.077774	0.152605	0.088563	0.152275	0.11161461	-0.65416914	0.679803373	0.167138023
ENSG00000006704	GTF2IRD1	9.592317	10.140376	9.725325	8.231839	9.532243	8.898523	10.555231	9.736715	11.290263	0.920644904	0.012264922	0.048760301	0.167258729
ENSG00000205309	NT5M	0.769121	0.88515	0.694566	0.623611	0.553535	0.472502	0.996194	0.719848	0.765241	0.430466296	-0.369459817	0.688168942	0.167324942
ENSG00000154310	TNIK	9.395408	9.003868	9.365728	9.344004	8.039674	8.162675	10.014051	9.038791	10.171963	0.717885906	0.038003308	0.027400473	0.167347005
ENSG00000051825	MPHOSPH9	8.523	9.915954	9.127708	9.030455	7.929736	7.887607	11.150048	9.134965	10.043229	0.804860179	-0.02798447	0.044265847	0.167403013

Gene ID	Gene name	Ctrl-1 FPKM	Ctrl-2 FPKM	Ctrl-3 FPKM	IFNγ-1 FPKM	IFNγ-2 FPKM	IFNγ-3 FPKM	Sora-1 FPKM	Sora-2 FPKM	Sora-3 FPKM	Ctrl vs IFNγ DESeq2 FDR	Ctrl vs IFNγ DESeq2 log2FC	Ctrl vs Sora DESeq2 FDR	Ctrl vs Sora DESeq2 log2FC
ENSG00000166881	NEMP1	9.713321	11.99631	12.043929	9.150482	8.290259	8.675851	11.874207	11.373754	12.372297	0.012955861	-0.228549762	0.076529748	0.167415128
ENSG00000142089	IFITM3	195.763883	181.388894	159.754357	1195.317303	1208.940078	1251.281149	186.425248	195.298588	182.806382	0	2.913798351	0.063689565	0.167483586
ENSG00000187051	RPS19BP1	43.795922	41.590367	40.310914	31.583163	34.283909	35.806732	40.953922	46.024115	45.20434	0.128776075	-0.149430377	0.078413802	0.167577135
ENSG00000183696	UPP1	6.77312	7.481769	6.442933	3.989589	5.564955	5.009915	5.984612	7.258155	9.592493	0.038630306	-0.380696335	0.310563676	0.167609676
ENSG00000150907	FOXO1	9.118323	8.976913	10.924207	7.972208	7.955561	7.984623	9.78607	9.214354	11.636193	0.189553485	-0.131348271	0.111901244	0.167637812
ENSG00000133597	ADCK2	5.658217	5.276109	4.563753	5.788309	4.511568	5.149943	5.330472	5.236305	5.584082	0.358260968	0.15221019	0.24006421	0.1677381
ENSG00000197256	KANK2	22.714953	24.37988	21.812182	16.788559	16.940867	17.004401	24.282837	22.581844	26.601083	0.00000669	-0.26841407	0.034620712	0.167808129
ENSG00000163467	TSACC	2.423672	2.14881	1.701431	1.338564	1.489837	1.415315	2.734889	2.544089	1.500939	0.39727772	-0.370770805	0.68785349	0.167822846
ENSG00000168283	BMI1	18.927383	19.575188	20.160027	17.48849	16.664636	18.036828	20.510224	19.511433	22.293795	0.805331117	-0.024192503	0.027700076	0.167832456
ENSG00000171735	CAMTA1	19.692529	20.717521	19.627785	20.343089	21.287727	20.59942	23.473881	25.927099	17.147038	0.28021548	0.133387089	0.214717377	0.167875982
ENSG00000135778	NTPCR	7.945805	8.517227	8.363772	6.251172	5.864934	5.914559	8.971043	8.679333	10.228252	0.00000858	-0.327554324	0.032064775	0.168169079
ENSG00000172830	SSH3	33.821566	33.713001	30.071513	27.225746	26.786093	29.543343	33.48824	32.542763	36.322988	0.372323592	-0.079210159	0.028259886	0.168176637
ENSG00000160191	PDE9A	30.17246	28.965128	28.072003	22.058233	22.987131	22.486964	29.638594	28.643146	33.419318	0.003320198	-0.220653321	0.039968473	0.168232182
ENSG00000122008	POLK	1.983051	2.664929	2.080941	2.181027	1.449367	1.342616	3.401597	1.731749	1.827548	0.761829818	-0.073348319	0.310492057	0.168241399
ENSG00000154447	SH3RF1	4.088472	3.682143	4.559582	2.663167	2.524022	2.394457	5.221012	4.057363	5.109599	0.000000771	-0.552010856	0.168321404	0.168281996
ENSG00000139624	CERS5	8.015479	8.588849	8.670496	6.040463	5.472425	7.29184	8.837598	8.158831	9.445388	0.175119538	-0.173094453	0.147184742	0.168360661
ENSG00000106628	POLD2	94.813124	96.425213	91.70847	82.508314	76.606262	77.063138	94.559021	99.540858	104.484298	0.061309465	-0.112669025	0.010862021	0.168411254
ENSG00000204560	DHX16	21.657395	21.806154	20.614668	21.415554	19.615881	20.005451	21.727094	22.406075	23.432359	0.404054563	0.067624086	0.024023778	0.168436852
ENSG00000072954	TMEM38A	3.798892	3.897743	3.647384	3.195116	4.107348	3.92399	3.554687	3.765768	4.653837	0.451811012	0.13522498	0.27658305	0.168463651
ENSG00000163297	ANTXR2	5.283287	5.058045	5.155691	3.377686	3.476582	3.732512	5.31692	5.01836	6.090035	0.000000478	-0.402247156	0.059370731	0.168485114
ENSG00000140961	OSGIN1	4.612898	3.857441	3.243849	3.265907	3.108919	3.524055	3.969001	4.521284	3.849723	0.575970302	-0.127047827	0.389920836	0.168486343
ENSG00000110047	EHD1	8.583268	7.226568	6.50646	13.509797	15.070797	14.348135	7.448586	7.426524	8.706687	8.41E-32	1.08775529	0.154465037	0.168526508
ENSG00000182004	SNRPE	77.177589	89.022083	87.823183	70.732666	76.564093	73.387292	91.495222	100.877341	72.860032	0.609666981	-0.052159965	0.188473107	0.168614916
ENSG00000108852	MPP2	3.463836	3.05165	2.733478	1.672218	1.528606	1.880819	3.184994	3.24082	3.424076	0.000000236	-0.729485396	0.221567968	0.168792233
ENSG00000198818	SFT2D1	33.233456	33.091503	35.825092	32.713753	35.412342	34.778725	34.990356	35.601013	37.101627	0.081513139	0.158930912	0.054916784	0.16881766
ENSG00000143401	ANP32E	42.654961	53.277878	55.36336	48.985686	43.525611	48.509087	54.430627	49.538377	55.963597	0.728238034	0.041940254	0.075794654	0.168924596
ENSG00000075089	ACTR6	15.277066	16.252928	14.808349	17.731703	18.412678	17.63816	17.048198	17.232025	17.237345	0.0000362	0.327887851	0.060936226	0.168950877
NewGene_4668	--	2.941471	2.487963	3.056485	2.276495	2.792115	2.832415	2.606403	2.66673	2.63358	0.223613741	0.213773094	0.304514679	0.169025731
ENSG00000114841	DNAH1	0.59821	0.484454	0.357441	0.546397	0.452587	0.462824	0.543184	0.423956	0.555582	0.51448991	0.164568975	0.459390989	0.169046528
ENSG00000134996	OSTF1	10.99804	9.675754	9.584213	10.633772	9.156622	9.14676	10.565516	10.572554	10.788817	0.628257663	0.08027939	0.196166892	0.169058086
ENSG00000085563	ABCB1	7.73418	7.423573	6.718586	17.743523	17.671828	17.765114	6.665988	7.68801	8.683328	2.84E-98	1.429665287	0.133375134	0.169103324
ENSG00000069966	GNB5	7.004237	7.091241	6.304604	4.594194	5.428892	5.436168	7.097153	6.689966	8.189818	0.121282955	-0.193220401	0.11600206	0.169268538
ENSG00000178053	MLF1	3.52547	2.944487	2.165401	1.656563	1.680367	2.098545	3.022472	3.121535	2.939745	0.022182789	-0.519294528	0.436373418	0.169294687
ENSG00000110108	TMEM109	50.691355	51.35283	51.589932	43.25185	42.995156	45.033056	52.008873	54.281641	55.751179	0.231781604	-0.079473409	0.009988621	0.169299567
ENSG00000102858	MGRN1	12.984852	11.863537	12.488173	13.515786	13.528552	11.99684	13.633261	15.05063	14.906377	0.00020747	0.254787628	0.047100029	0.169363675
ENSG00000111271	ACAD10	5.501098	5.452181	4.423221	3.816763	3.634507	4.121439	5.325776	5.595909	5.646421	0.021418739	-0.289603038	0.160454446	0.169371791
ENSG00000106299	WASL	19.048326	20.261047	22.58827	25.012903	23.297293	24.254021	22.767258	19.809532	22.763819	5.86E-08	0.376960633	0.04871872	0.169478097
ENSG00000155393	HEATR3	9.441605	11.206304	10.239012	9.66636	9.615582	9.85663	11.741567	9.939804	10.894416	0.568435863	0.064563442	0.097065936	0.169582187
ENSG00000165238	WNK2	8.381468	7.834277	7.990547	6.002991	5.474857	5.617141	8.274635	7.711264	10.467297	0.00000672	-0.349197829	0.109327277	0.16958494
ENSG00000154582	ELOC	31.379424	33.603703	36.529929	33.155578	36.52146	34.459199	35.54958	39.715372	34.677019	0.023376851	0.192758731	0.081918611	0.169655652
ENSG00000130762	ARHGEF16	16.897783	17.025663	16.358061	9.899712	10.10064	11.305199	17.321781	15.555014	20.898539	8.49E-14	-0.548549309	0.06871827	0.169750734
ENSG00000105647	PIK3R2	65.418406	14.639	58.131109	47.451349	9.833509	57.172766	46.206244	42.751393	51.369515	0.955226678	-0.090987267	0.83914466	0.169820376
NewGene_3286	--	0.413724	0.236551	0.363568	0.035271	0.064936	0.069374	0.313501	0.338309	0.457153	--	--	0.74111276	0.169845169
ENSG00000116649	SRM	45.227746	43.436287	46.740482	45.906177	40.200122	46.158132	44.606526	47.945194	50.604399	0.245625607	0.104791479	0.062451208	0.169919679
ENSG00000100883	SRP54	37.322378	41.624988	45.282072	41.683814	46.528281	44.03556	44.459929	45.588004	49.128308	0.002628572	0.208316852	0.02833922	0.169964315
ENSG00000170458	CD14	1.674616	1.300423	1.153391	2.642665	1.895762	1.845283	1.4016	1.358218	1.762013	0.002310716	0.762216561	0.561102516	0.169994828
ENSG00000153391	INO80C	17.340122	20.424771	17.65392	17.159194	17.565847	16.363659	20.761198	20.682611	19.711665	0.975527107	-0.005610288	0.148846283	0.17020322
ENSG00000167323	STIM1	11.471059	12.222054	10.12695	11.374362	11.774304	11.547364	11.874157	10.643243	12.156898	0.005585045	0.226128948	0.054593252	0.170228384
ENSG00000158457	TSPAN33	8.387193	7.445488	6.32551	5.844176	5.779866	5.538975	7.561742	8.118774	7.695623	0.144346389	-0.222641142	0.246689161	0.17042906
ENSG00000100577	GSTZ1	12.095567	11.315792	10.15328	9.426944	10.815801	9.931517	11.796735	12.640166	12.477639	0.998196506	0.000739187	0.207336975	0.170583806
ENSG00000197448	GSTK1	37.288548	32.016785	31.527578	60.002666	61.921187	60.321488	33.770047	34.126105	37.884251	5.95E-45	1.010548339	0.070752525	0.170613184
ENSG00000077044	DGKD	3.479763	3.4683	3.539365	3.143589	2.916048	3.256343	3.966159	2.973551	4.096613	0.869727425	-0.024208319	0.173910452	0.170624253
ENSG00000105997	HOXA3	6.625269	6.295837	6.986869	6.780004	4.767034	7.006711	8.013279	7.328749	6.262635	0.769272494	-0.056126782	0.192120062	0.1706339
ENSG00000139168	ZCRB1	19.829463	21.301244	20.191432	15.069934	14.75091	17.476737	17.392714	19.507975	19.049161	0.405270253	-0.083891606	0.078938997	0.170634908
ENSG00000090013	BLVRB	21.971684	21.96348	18.414426	17.743175	18.379126	19.06217	21.337815	23.702414	21.102517	0.883258844	-0.025390002	0.190068679	0.170772201
ENSG00000164199	ADGRV1	1.206655	0.957964	1.205399	1.555724	1.314163	1.144483	1.02816	0.965937	1.170222	0.895214398	0.027417294	0.250938561	0.170818357
ENSG00000180902	D2HGDH	3.683078	3.252927	2.906378	3.592765	3.453822	3.808958	3.579139	2.790533	3.402319	0.077664351	0.265036483	0.290772263	0.170865388
ENSG00000118162	KPTN	12.278707	12.167672	11.093102	9.903672	10.611785	10.876032	11.715482	13.054698	12.72025	0.853136186	-0.029687376	0.146053259	0.170951988
ENSG00000160789	LMNA	39.680445	35.812333	35.664144	25.311684	27.707682	28.948691	37.182285	38.924938	41.312437	0.000140227	-0.288510839	0.023447286	0.171005095
ENSG00000130227	XPO7	21.428914	20.338285	20.284142	18.577075	17.803655	16.938296	21.491283	21.804089	21.924779	0.355617291	-0.069634157	0.01363486	0.17109815
ENSG00000165525	NEMF	4.907881	5.07501	5.340249	4.586748	4.23152	4.510881	5.358346	5.029097	5.749754	0.626755378	-0.055458525	0.061771738	0.171126183
ENSG00000170291	ELP5	42.153652	36.775536	38.764158	41.229061	36.815187	46.423736	42.796595	46.666649	40.990032	0.117789238	0.134633586	0.075092438	0.171207172
ENSG00000214413	BBIP1	2.736708	1.920786	2.512382	2.906688	3.327086	1.994104	2.600943	2.517446	1.960629	0.120044528	0.390438688	0.504674802	0.171225827
ENSG00000130881	LRP3	28.450321	27.333344	23.48489	28.859259	32.340393	33.45047	27.092764	25.766291	30.992981	0.00000122	0.404153294	0.057896189	0.17128492
ENSG00000106789	CORO2A	5.122417	4.890269	5.128789	5.629488	5.321483	4.889512	5.563786	4.932781	5.506301	0.027153286	0.210656811	0.073260516	0.171317752
ENSG00000075188	NUP37	14.060089	22.833109	15.395635	12.033687	12.529582	13.12007	16.977696	16.419398	20.487781	0.0252232	-0.207120625	0.101667472	0.171339257
ENSG00000160293	VAV2	22.055502	20.592983	18.26085	20.766749	21.554421	21.321458	21.194561	19.812029	23.449682	0.005132939	0.212659271	0.041227666	0.171350067
ENSG00000075785	RAB7A	123.487381	127.696926	134.245904	121.29843	119.138951	115.560258	133.061159	131.346486	142.630533	0.64301327	0.033708038	0.005350703	0.171440576
ENSG00000085377	PREP	33.566178	37.043807	39.022905	28.919152	27.665579	26.999697	39.698138	36.589195	41.933446	0.001102419	-0.225607603	0.022868096	0.171449709
ENSG00000205670	SMIM11A	10.956505	9.29141	9.277989	7.364287	6.93421	7.615357	9.213877	13.116521	9.101987	0.094845332	-0.282973619	0.39655139	0.171545718
NewGene_5399	--	3.799022	3.318117	4.416544	2.462202	2.226553	2.904813	3.436719	4.75425	3.872451	0.166091016	-0.445711186	0.57688443	0.171549827
ENSG00000169862	CTNND2	35.321247	31.676602	32.293342	27.823611	27.547823	28.350475	35.156527	31.496823	37.498209	0.078837023	-0.115890794	0.017059509	0.171556517
ENSG00000137509	PRCP	8.496223	8.771143	8.096275	6.211673	6.908678	6.481462	8.778061	9.363753	8.578918	0.005335957	-0.268036387	0.102410899	0.17161805
ENSG00000162909	CAPN2	17.812658	17.36982	19.32069	10.01542	9.51769	9.190309	18.067007	18.336082	21.239677	8.68E-28	-0.77751929	0.042503054	0.171664842

Gene ID	Gene name	Ctrl-1 FPKM	Ctrl-2 FPKM	Ctrl-3 FPKM	IFNγ-1 FPKM	IFNγ-2 FPKM	IFNγ-3 FPKM	Sora-1 FPKM	Sora-2 FPKM	Sora-3 FPKM	Ctrl vs IFNγ DESeq2 FDR	Ctrl vs IFNγ DESeq2 log2FC	Ctrl vs Sora DESeq2 FDR	Ctrl vs Sora DESeq2 log2FC
ENSG00000106066	CPVL	24.14983	23.773407	22.236789	23.457089	24.242192	23.715	23.425533	26.162671	26.303817	0.033122612	0.162378428	0.042609834	0.1716965
ENSG00000131747	TOP2A	100.948219	109.306854	101.303551	82.378389	73.854897	79.586998	109.21407	107.442162	112.546088	0.00000309	-0.254832758	0.003622262	0.171995746
ENSG00000175324	LSM1	23.987859	25.847326	25.295145	27.662812	27.584638	24.757106	26.87374	25.358751	27.331479	0.008472546	0.235936386	0.06567984	0.172135231
ENSG00000093134	VNN3	2.44735	2.033093	2.617166	3.61844	3.805695	3.382588	2.056217	2.670643	2.969933	0.000784501	0.633733353	0.481162547	0.172190418
ENSG00000118507	AKAP7	1.747668	1.828494	1.528816	3.28179	3.022877	3.796585	1.699952	1.487661	2.152999	2.18E-12	1.121989391	0.459552213	0.172238064
ENSG00000213160	KLHL23	20.320474	21.517185	22.843056	16.755349	14.693195	15.664655	22.702512	22.43025	22.445945	0.000000203	-0.357170679	0.02336478	0.172245957
ENSG00000127561	SYNGR3	2.155448	1.720318	1.506333	0.345114	1.128252	1.070058	1.725884	1.842719	1.995829	0.020923918	-0.894775476	0.486908645	0.172254025
ENSG00000049089	COL9A2	9.904655	10.73603	9.797217	3.491445	3.874894	3.414911	10.047099	10.703017	11.897637	1.07E-41	-1.366417993	0.096141757	0.172310968
ENSG00000133678	TMEM254	7.654547	8.521703	7.693525	6.917178	6.991177	6.744377	8.654194	8.377607	8.421458	0.680494698	-0.061935361	0.142784789	0.172357293
ENSG00000145191	EIF2B5	19.604913	19.703288	20.252107	18.974618	17.991305	18.164047	19.624045	20.153026	21.847317	0.457256765	0.064978099	0.030786353	0.172422484
ENSG00000161513	FDXR	4.273106	3.536453	3.493988	3.653346	2.903214	3.245719	3.611579	4.54731	3.683245	0.811881627	-0.057612436	0.37934842	0.172450233
ENSG00000135541	AHI1	2.264925	2.277216	2.580709	2.04024	1.811316	2.111794	2.346843	2.095048	2.551144	0.623900885	-0.090358205	0.241809855	0.172575076
ENSG00000094914	AAAS	24.839	24.724108	21.150522	22.628683	21.679614	23.110149	23.538253	25.98339	25.702085	0.578957591	0.060989084	0.076221255	0.172589889
ENSG00000113712	CSNK1A1	28.406439	29.444262	27.822914	29.07859	29.685952	29.292277	34.690451	31.308823	29.014195	0.0000136	0.245288294	0.007631413	0.172635827
ENSG00000172059	KLF11	0.921093	0.642118	0.710395	0.895415	1.067029	0.733071	0.68002	0.846293	0.819583	0.169424095	0.386090514	0.550977535	0.172648208
ENSG00000198825	INPP5F	2.221626	2.299903	2.499152	1.519506	1.314284	1.299031	2.713041	2.10213	2.738908	0.0000798	-0.546002988	0.239240927	0.172686417
ENSG00000125505	MBOAT7	27.926575	28.136407	31.171643	28.862103	27.837328	27.978555	27.903071	30.54355	31.31898	0.281869896	0.087079263	0.019786664	0.172723595
ENSG00000165471	MBL2	1.045167	1.196007	0.902714	0.975079	0.869527	0.701941	1.157528	0.980532	1.194671	0.615806569	-0.153580884	0.472927038	0.172740122
ENSG00000171130	ATP6V0E2	11.225465	12.80415	14.375746	10.666876	14.203143	14.358003	12.622026	15.949641	16.874827	0.998321415	-0.000490179	0.089637374	0.172840191
ENSG00000125970	RALY	102.572452	100.263042	93.499106	75.249352	77.236753	80.142578	113.684161	114.729262	117.07222	0.009019167	-0.172387979	0.013573176	0.172857895
ENSG00000111321	LTBR	42.163084	41.162441	41.579945	43.20948	39.286633	40.38985	40.731481	44.768925	48.444045	0.013615315	0.1620995	0.03002928	0.172905789
ENSG00000100038	TOP3B	13.157391	11.740585	13.264874	10.316947	9.038015	9.928064	13.426695	13.836639	14.184524	0.010370201	-0.224571181	0.052802948	0.173066812
ENSG00000161999	JMJD8	31.906682	31.286088	31.059528	25.580301	24.569494	25.627507	31.346558	31.502418	37.439387	0.017124092	-0.169245094	0.039161736	0.173105655
ENSG00000153048	CARHSP1	84.29836	74.503251	70.081054	73.308538	84.100598	77.82832	86.188016	86.435514	90.952669	0.108317644	0.131524147	0.015299625	0.173177866
ENSG00000110871	COQ5	12.657671	12.65247	9.359537	11.107351	10.985207	9.768989	13.039757	12.112592	11.306218	0.896405511	0.025549445	0.229072789	0.173184627
ENSG00000179532	DNHD1	1.283311	1.374212	1.389714	1.412915	1.641987	1.438244	1.337965	1.214115	1.46449	0.000741822	0.376612294	0.176898707	0.173220713
ENSG00000169813	HNRNPF	148.697197	143.236154	139.92378	135.099387	127.226034	134.152752	151.822173	143.351773	164.963242	0.335602504	0.056100657	0.001689804	0.173289702
ENSG00000120159	CAAP1	12.120672	13.268383	14.363364	11.932879	12.307027	11.737495	14.890847	13.571311	14.479125	0.876744628	0.018779366	0.048142761	0.173311506
ENSG00000105618	PRPF31	43.457052	44.658367	41.032749	38.432492	37.55256	38.610827	43.135651	47.167514	47.261228	0.507888312	-0.053755878	0.010446153	0.173321697
ENSG00000143537	ADAM15	10.598658	9.478269	9.558539	10.518064	9.540434	10.143213	9.785168	9.744419	12.257971	0.094394003	0.163814739	0.109642267	0.173375008
ENSG00000144504	ANKMY1	5.905708	6.145184	5.825738	5.549057	5.109211	4.674851	6.447254	5.726111	6.915614	0.453615273	-0.103018665	0.161908714	0.173390856
ENSG00000134058	CDK7	16.969119	18.275018	18.81672	14.651346	14.647841	15.484854	17.534362	19.009744	19.238609	0.363224226	-0.104263303	0.101238204	0.17354502
ENSG00000141503	MINK1	22.170912	18.57583	21.552673	19.670537	16.971684	17.128525	22.116575	21.755554	25.948403	0.673575193	-0.046466562	0.053427254	0.173637891
ENSG00000143457	GOLPH3L	13.990719	14.292334	13.928123	14.280834	14.029918	15.729247	14.451836	14.990702	16.781532	0.006448335	0.216474154	0.042339552	0.173832092
ENSG00000102879	CORO1A	1.735373	2.026609	1.717833	1.2405	1.466134	1.628013	1.689546	1.775219	2.363523	0.528811999	-0.192056781	0.502406793	0.174033756
ENSG00000140365	COMMD4	86.690581	92.36595	81.706063	73.119233	66.05765	79.314449	90.231288	84.611208	89.330373	0.966314133	0.004751468	0.023962264	0.174160794
ENSG00000147065	MSN	10.734447	11.33667	11.422685	11.105816	9.134534	10.479996	12.110557	11.554523	11.762601	0.857560584	0.022576275	0.03477003	0.174241817
ENSG00000183978	COA3	47.68252	45.707643	48.036574	40.614013	40.641893	45.635547	50.759719	50.742687	47.64784	0.960001811	-0.007056965	0.059959075	0.174384969
ENSG00000080823	MOK	0.490324	0.580618	0.465913	0.557028	0.656106	0.400103	0.628526	0.375065	0.506943	0.981400982	0.016211976	0.713614278	0.174425867
ENSG00000099282	TSPAN15	8.581573	8.077221	7.010562	6.039992	5.117728	6.086911	7.701843	8.057101	9.336781	0.032436619	-0.308026566	0.212045312	0.17449241
ENSG00000230989	HSBP1	8.610563	8.862083	8.443064	7.844691	8.66988	8.157981	9.302003	9.554991	8.579231	0.292445153	0.088603094	0.039968473	0.174592873
ENSG00000161692	DBF4B	3.076754	2.718321	3.09265	2.662569	2.171613	2.185048	3.465602	2.99123	2.961233	0.298050039	-0.191217336	0.292606103	0.174651397
ENSG00000078369	GNB1	151.990848	151.563023	156.73466	134.718503	128.525682	131.331552	159.096802	158.238899	170.876253	0.153403616	-0.075834399	0.00161134	0.174791846
ENSG00000145425	RPS3A	1023.049881	1075.455664	1032.947248	758.712204	800.515914	808.349843	1095.761733	1067.47799	1010.29201	0.00000116	-0.268080599	0.011200687	0.174849288
ENSG00000162604	TM2D1	8.247697	10.516097	10.987559	9.035984	9.664607	7.71112	11.308885	11.50123	9.288375	0.809218755	0.051131864	0.284668659	0.174865286
ENSG00000131966	ACTR10	11.036742	10.854372	11.399602	9.261878	9.545687	8.990096	12.862154	12.302581	11.820841	0.279835908	-0.117292628	0.098132835	0.174977218
ENSG00000162298	SYVN1	19.659293	19.329259	20.981411	27.267339	24.924623	25.902563	20.948896	19.379856	23.260172	1.25E-16	0.526689036	0.034242731	0.1751229
ENSG00000204469	PRRC2A	72.963844	68.559341	63.921427	69.498878	64.540014	68.108284	71.928219	65.620304	80.391639	0.058981693	0.123004434	0.019748293	0.175194051
ENSG00000102078	SLC25A14	2.42871	2.273766	1.733426	3.144662	2.16113	2.081684	2.671355	2.222486	1.909531	0.357190729	0.266642896	0.502329316	0.17543847
ENSG00000138744	NAAA	2.340765	2.930927	3.430492	5.192594	6.117762	6.129857	3.272934	3.124732	3.127364	1.25E-10	1.074134026	0.422711753	0.175441935
ENSG00000119778	ATAD2B	2.343993	2.453375	2.046178	2.30567	2.496465	2.052809	2.793297	2.131006	2.586656	0.377486409	0.118630936	0.150751357	0.175529985
ENSG00000077463	SIRT6	8.590284	8.762789	7.891458	6.705694	6.044336	6.774233	9.497133	9.039101	8.471367	0.106736293	-0.219955541	0.177958503	0.175618993
ENSG00000142330	CAPN10	7.122688	6.396718	5.770702	6.219456	5.295652	5.919404	6.800288	6.197362	7.546872	0.857080917	-0.034478576	0.230284539	0.175634326
ENSG00000105538	RASIP1	0.316827	0.323894	0.31615	0.397269	0.608946	0.349229	0.427534	0.337187	0.246418	0.113787573	0.643803038	0.703392584	0.175661339
ENSG00000166483	WEE1	56.075086	57.452319	52.056429	51.442615	48.403625	47.27927	67.170423	56.288189	59.670175	0.566623015	-0.042750753	0.028879623	0.175684994
ENSG00000174989	FBXW8	6.973528	6.136811	5.753209	7.559647	6.651771	6.804155	6.18736	6.540133	7.201558	0.000295958	0.347177412	0.105543893	0.17575358
ENSG00000151655	ITIH2	264.881348	254.713431	238.542439	238.699899	237.235713	233.697748	263.408238	267.202999	270.429489	0.401141043	0.055735919	0.005829937	0.175941869
ENSG00000172244	C5orf34	3.295916	3.147094	3.801057	2.466529	2.026588	2.070525	4.073583	3.072602	3.866495	0.003979426	-0.483149511	0.323183116	0.176149251
ENSG00000164176	EDIL3	1.817115	1.926662	1.813209	3.18866	3.103832	2.858601	1.95977	2.023952	1.85799	1.66E-13	0.861817706	0.263252372	0.17620826
ENSG00000153037	SRP19	17.344415	17.13293	16.766645	17.609172	19.392306	17.558044	18.105077	19.562939	17.590665	0.08342029	0.153654488	0.045915152	0.176316723
ENSG00000054611	TBC1D22A	11.560844	10.918337	10.949334	8.582565	8.362892	7.803361	11.665726	10.854997	12.141125	0.012541974	-0.220772421	0.044616566	0.176357566
ENSG00000186432	KPNA4	16.68766	17.572504	18.660337	14.479529	14.086723	14.176161	19.453939	17.463985	19.189939	0.010631308	-0.16074979	0.012433582	0.176408223
ENSG00000142230	SAE1	105.27378	104.052081	99.064669	81.695714	74.824112	80.669694	107.934078	105.771413	113.656787	0.0000642	-0.227018191	0.002984348	0.176445289
ENSG00000171843	MLLT3	1.763511	1.078237	1.398784	1.328406	1.498943	1.093272	1.269265	1.279644	1.489057	0.580003398	0.11966226	0.309619657	0.176456944
NewGene_6171	--	4.587838	4.111645	3.192109	2.86583	2.902136	3.085016	3.774359	3.631087	3.921047	0.128704758	-0.227272285	0.207058315	0.17647927
ENSG00000013288	MAN2B2	7.718434	7.258298	6.813612	7.216846	7.384398	7.115735	7.352907	6.998713	8.396171	0.062730653	0.173182456	0.061912913	0.176488585
ENSG00000103811	CTSH	8.443379	7.716485	7.837073	5.46773	5.687805	5.546059	9.262938	7.531602	9.099433	0.000725412	-0.382442987	0.153017107	0.176491676
ENSG00000129460	NGDN	32.744346	33.019569	29.269889	27.724782	26.20183	25.429232	23.833411	31.119281	25.355586	0.653163359	-0.049627757	0.048760301	0.176665309
ENSG00000164985	PSIP1	45.832871	51.160907	55.049665	49.06723	47.125417	46.008232	54.339782	51.801821	57.798257	0.486933115	0.059245932	0.017676527	0.17675226
ENSG00000119041	GTF3C3	18.033169	18.897097	18.317046	17.399106	15.647497	14.514686	20.761504	19.332594	19.644075	0.278695812	-0.09324973	0.018076099	0.176918623
ENSG00000198960	ARMCX6	8.69743	7.26374	7.866821	6.334333	5.066716	6.769137	8.538197	7.55044	9.224753	0.113496278	-0.242589578	0.180273693	0.176918842
ENSG00000140548	ZNF710	11.22338	9.846126	10.366984	8.858162	9.415706	8.629511	11.691296	11.022886	11.999932	0.405571402	-0.081878909	0.038226388	0.176979139
ENSG00000213920	MDP1	4.921363	4.47827	3.854064	3.758481	3.902725	4.250639	4.67369	5.471483	3.827789	0.999202724	0.000487392	0.521008198	0.177034779
ENSG00000011426	ANLN	52.099402	57.831988	55.027786	35.294221	33.636534	36.824943	58.00601	55.915439	59.672719	9.51E-19	-0.496266952	0.005469661	0.177111804

Gene ID	Gene name	Ctrl-1 FPKM	Ctrl-2 FPKM	Ctrl-3 FPKM	IFNγ-1 FPKM	IFNγ-2 FPKM	IFNγ-3 FPKM	Sora-1 FPKM	Sora-2 FPKM	Sora-3 FPKM	Ctrl vs IFNγ DESeq2 FDR	Ctrl vs IFNγ DESeq2 log2FC	Ctrl vs Sora DESeq2 FDR	Ctrl vs Sora DESeq2 log2FC
ENSG00000084774	CAD	13.782745	13.427395	13.691415	11.813955	9.778339	11.061627	14.308538	13.244149	17.392097	0.002632187	-0.211193065	0.03491389	0.177148768
ENSG00000147654	EBAG9	10.672978	11.320443	12.992352	12.67459	11.425355	12.208327	14.680646	11.988135	13.541464	0.10040026	0.178690263	0.10796466	0.177149244
ENSG00000155100	OTUD6B	5.272683	6.677209	7.094753	5.645132	5.739293	6.05754	7.359609	6.657364	6.163988	0.915091073	0.022891592	0.264385165	0.177233455
ENSG00000101773	RBBP8	13.557949	14.260701	14.518299	14.630234	12.728986	12.441289	14.666635	15.305759	15.78607	0.634870881	0.05168051	0.032064775	0.177314702
ENSG00000176182	MYPOP	2.492683	1.990518	2.610039	3.045594	2.464254	2.670664	2.106715	2.948658	2.445635	0.102121688	0.350417142	0.464559542	0.177339396
ENSG00000115520	COQ10B	12.840819	13.132999	14.130412	17.970526	17.984021	17.639469	14.17413	13.212212	14.65647	1.35E-13	0.576325848	0.06395842	0.177449138
ENSG00000102978	POLR2C	32.069767	33.448202	31.129562	26.575847	26.011305	27.718365	34.731258	34.508008	32.834742	0.112515833	-0.122208573	0.02726592	0.177506868
ENSG00000112651	MRPL2	20.958685	19.257874	18.62492	16.457227	16.26327	18.286452	20.094092	21.9199	20.937388	0.65734144	-0.056761969	0.085627684	0.17753002
ENSG00000124440	HIF3A	0.354256	0.211038	0.200663	0.124526	0.145491	0.180788	0.19453	0.361558	0.341835	0.083126074	-0.745381567	0.681156965	0.177549102
ENSG00000125458	NT5C	30.52055	30.449207	27.694368	21.879789	24.724136	22.67129	30.694428	30.441018	31.172093	0.110079057	-0.173738989	0.064427587	0.177630255
ENSG00000167705	RILP	3.678854	3.091642	2.115464	2.37744	2.899242	3.447884	2.839926	3.577726	3.446914	0.702844211	0.122700068	0.489126102	0.177664817
ENSG00000130349	C6orf203	5.556086	6.196392	5.953462	5.29641	5.13675	4.78135	6.031606	5.395386	6.097736	0.924946646	0.024825077	0.328110779	0.177736937
ENSG00000264343	NOTCH2NLA	2.79126	1.843312	1.888694	1.643473	1.907032	2.06011	2.309459	2.937372	2.492344	0.980706027	0.007887358	0.352957841	0.177885647
ENSG00000114902	SPCS1	18.847339	20.057346	22.488927	14.903147	13.783058	18.361902	31.222323	25.361151	36.509076	0.750219939	-0.033315492	0.022085684	0.177918512
ENSG00000162517	PEF1	27.202415	23.859154	23.11631	18.200993	20.925482	21.567522	23.698828	26.343325	28.704258	0.222035401	-0.140568879	0.085371142	0.177941839
ENSG00000120690	ELF1	25.249552	27.289299	30.901465	31.7363	29.736338	29.57562	30.265762	28.29599	31.636647	0.004094346	0.242318606	0.054567339	0.177954164
ENSG00000105643	ARRDC2	2.113547	2.105229	2.126325	1.436669	1.763932	1.438812	2.154578	2.590703	2.034736	0.091799772	-0.366198096	0.398286819	0.177967555
NewGene_6156	--	3.61401	4.149321	3.076692	2.791321	2.412356	2.106327	3.377521	4.117037	3.694488	0.004094346	-0.37494755	0.159618594	0.177979564
ENSG00000115364	MRPL19	17.045663	19.925835	20.306813	19.248581	17.12364	18.111071	18.912408	20.56432	19.491609	0.286557758	0.098940852	0.044674512	0.178013649
ENSG00000023191	RNH1	49.684687	49.946278	47.944718	46.159496	48.387111	49.566387	50.008685	54.843892	56.445403	0.288648708	0.081728627	0.024203687	0.178051414
ENSG00000237651	C2orf74	7.981184	7.025006	7.924327	6.445385	7.570726	7.13363	6.905564	9.236447	9.970111	0.917209425	0.027658151	0.353444201	0.178055443
ENSG00000072736	NFATC3	7.599924	7.648858	7.664021	8.060967	8.069771	7.7237	8.557923	7.438502	8.607661	0.011657492	0.184335644	0.033115042	0.178147671
ENSG00000001630	CYP51A1	57.571416	56.344421	57.506204	78.955455	78.944663	75.502058	61.648082	58.494167	61.585829	5.11E-35	0.597873283	0.002936517	0.178220987
ENSG00000113811	SELENOK	16.84898	17.669809	18.557266	19.013537	19.051506	14.038739	14.536952	15.012388	21.678926	0.026932666	0.240489037	0.150967337	0.178271002
ENSG00000142409	ZNF787	48.240887	50.569432	46.368925	42.8344	49.441521	49.058108	50.865726	51.366722	51.510928	0.221879434	0.109346471	0.01236302	0.17838608
ENSG00000141524	TMC6	32.061055	28.222514	27.154847	17.112955	17.366992	17.842125	32.447074	30.384205	37.387029	2.26E-21	-0.634516641	0.055547608	0.178455729
ENSG00000186998	EMID1	4.012023	2.719111	2.926319	2.244124	2.47175	2.666698	3.540225	3.069292	3.7154	0.289784289	-0.240379403	0.377217682	0.178528315
ENSG00000156709	AIFM1	44.670825	41.810994	46.343502	36.161046	38.15185	36.017003	43.475932	47.247418	50.560072	0.115613264	-0.119548719	0.024011464	0.178576168
ENSG00000024526	DEPDC1	5.742782	6.838765	6.884345	5.063105	4.191219	4.539626	7.260119	6.618352	6.930079	0.000342444	-0.365892393	0.087989439	0.178583179
ENSG00000074071	MRPS34	71.376902	73.0621	67.378615	64.073381	68.974949	71.313888	73.51132	73.216218	78.236898	0.262194212	0.096092961	0.013590562	0.178602128
ENSG00000164258	NDUFS4	30.743513	32.126316	28.939067	27.299516	25.298271	25.595848	36.322327	32.071362	29.315477	0.463202577	-0.089711971	0.137272986	0.178712709
ENSG00000187239	FNBP1	6.1489	5.97459	6.097134	6.062701	6.651921	6.796517	7.205972	6.422653	7.872008	0.218363427	0.133511385	0.063589812	0.178887481
ENSG00000038274	MAT2B	12.53673	12.56088	12.081224	14.971611	15.446898	15.383727	11.468194	12.78641	13.664932	1.68E-09	0.476807072	0.076475166	0.178937044
ENSG00000255529	POLR2M	20.070658	20.144146	21.457092	18.887725	18.316425	18.555294	21.607371	21.028088	24.046032	0.833744224	-0.019519492	0.015946164	0.179013055
NewGene_5587	--	1.338141	2.575062	2.967389	2.065066	1.514788	2.019581	2.154115	2.513629	2.621063	0.685663623	-0.166668427	0.588636303	0.179102629
ENSG00000250479	CHCHD10	1.657861	2.741869	3.178472	2.423542	2.988356	3.214445	2.273369	4.141665	2.074932	0.215110374	0.373538905	0.598815692	0.179191933
ENSG00000105700	KXD1	47.865049	48.222478	42.527404	39.312582	40.783917	39.786634	47.186142	49.141209	54.210093	0.275737745	-0.097870922	0.035103258	0.179206703
ENSG00000196419	XRCC6	301.620129	313.022972	319.359762	271.25376	288.549758	285.274663	335.434786	310.165811	340.994149	0.726764287	0.023041428	0.001165585	0.179225638
ENSG00000165915	SLC39A13	11.512652	11.283798	9.747077	8.151525	8.458376	8.905759	10.615323	10.786699	12.86878	0.123797187	-0.174846234	0.115113068	0.179237446
ENSG00000196378	ZNF34	1.358217	2.003854	1.329315	1.322748	1.214689	1.254745	2.02618	1.290291	1.740645	0.666548026	-0.148004111	0.536300693	0.179274088
ENSG00000068885	IFT80	6.72719	6.853048	7.972669	6.541944	5.941406	6.131327	7.549317	6.680193	8.639366	0.714712773	-0.048871137	0.120437071	0.179304671
ENSG00000090889	KIF4A	14.182424	14.654535	13.921774	11.321798	10.44319	10.421634	16.227739	13.76134	15.463088	0.000154696	-0.263230977	0.026677805	0.179520426
ENSG00000125846	ZNF133	5.170768	5.966363	5.859034	5.860581	5.492954	6.491962	6.075257	5.274481	5.049823	0.290291926	0.145232913	0.181681574	0.17962001
ENSG00000122958	VPS26A	26.510553	25.551526	26.404635	25.679685	27.033046	25.750199	31.273949	28.079727	29.201899	0.886584442	0.015840809	0.022831783	0.179665689
ENSG00000156973	PDE6D	12.323927	11.04819	12.373427	11.991099	13.746575	12.809043	11.806028	12.489266	13.126799	0.036978594	0.264289978	0.157475575	0.179717731
ENSG00000127884	ECHS1	69.083359	70.38179	64.586586	63.082443	61.715061	63.541073	69.859169	68.888351	78.189529	0.703543164	0.03194529	0.01118041	0.179741946
ENSG00000175756	FAM173B	7.106778	8.312937	7.452635	6.678575	5.740691	6.233211	7.64112	7.98937	8.284123	0.387773968	-0.133005786	0.171535851	0.179861914
ENSG00000119938	PPP1R3C	7.530947	6.920856	5.977114	8.494044	8.446564	8.294967	8.784608	7.197527	5.650564	0.0000351	0.451114095	0.302990505	0.179955299
ENSG00000128944	KNSTRN	37.723813	41.377382	36.898842	29.972239	27.739075	28.495273	39.491085	41.011877	42.553908	0.00000982	-0.298394658	0.015315999	0.179965472
ENSG00000134107	BHLHE40	20.914114	20.889944	23.135632	30.069965	26.107998	28.129354	22.933609	22.976486	23.044231	8.05E-15	0.52324484	0.020699505	0.180000307
ENSG00000197694	SPTAN1	75.251315	72.689186	71.061582	57.678808	56.851276	57.578032	78.059213	73.249741	83.936669	0.000179351	-0.179037351	0.001688372	0.180022243
ENSG00000138801	PAPSS1	23.108557	21.815134	22.24873	16.560221	18.552914	17.177282	21.668425	24.307549	25.399702	0.007676839	-0.213419964	0.034525451	0.180025447
ENSG00000123119	NECAB1	0.408075	0.821656	0.628695	0.300666	0.43182	0.473695	0.591727	0.592883	0.747373	0.172209939	-0.463583114	0.550283378	0.180122537
ENSG00000109320	NFKB1	15.142325	14.791918	15.938108	12.124157	12.181085	12.0653	16.275684	14.678253	18.638615	0.013268037	-0.182072561	0.051538814	0.180174593
ENSG00000196923	PDLIM7	16.686497	16.511264	16.539215	9.680108	10.400662	11.401054	16.518667	17.324755	18.987455	0.00000023	-0.516562165	0.064877969	0.180196313
ENSG00000154654	NCAM2	0.364102	0.299976	0.351969	0.4579	0.560941	0.360354	0.340478	0.315948	0.25324	0.003860438	0.762972368	0.585803195	0.180271427
ENSG00000102974	CTCF	24.969054	24.797183	26.869953	22.504829	22.085137	22.256793	27.482451	25.095433	28.657232	0.601656761	-0.042699024	0.01127307	0.180299019
ENSG00000153066	TXNDC11	25.460354	24.08618	24.253046	30.057739	32.690935	30.939614	25.709646	25.263917	26.691372	2.21E-15	0.511888179	0.012195459	0.180307909
ENSG00000132004	FBXW9	4.964523	6.505236	5.962809	4.757416	4.845679	4.939791	5.842284	5.715125	6.921974	0.570948405	-0.10779226	0.260234512	0.180387815
ENSG00000102543	CDADC1	0.735493	0.807843	0.734247	0.552753	1.23141	0.882149	0.707816	1.044761	0.952635	0.653798287	0.182726664	0.591738285	0.180435491
ENSG00000169972	PUSL1	22.019571	21.035797	18.916239	17.550145	15.583052	19.863253	20.981338	21.029813	22.523023	0.775522931	-0.044316487	0.075709859	0.180463622
ENSG00000172757	CFL1	661.473299	636.037261	643.665477	545.378896	543.568702	558.153173	685.581867	683.779733	698.007501	0.055842143	-0.096907582	0.001063499	0.180870383
ENSG00000109062	SLC9A3R1	112.064335	104.758582	90.636285	129.837055	149.246828	155.611134	112.051016	115.779808	131.746533	3.29E-13	0.518861837	0.033839731	0.180871275
ENSG00000058600	POLR3E	16.419344	15.916934	14.806041	12.744396	12.46614	11.331811	17.134919	14.844274	15.735121	0.542021431	-0.059318262	0.029204981	0.180936749
ENSG00000086619	ERO1B	0.814759	1.05685	1.052198	1.675854	1.829222	1.69362	1.205858	0.812804	1.17375	7.99E-10	1.008781783	0.441552269	0.180939185
ENSG00000204366	ZBTB12	10.735185	11.221779	9.490904	12.293063	12.857841	13.11509	10.565715	10.865833	12.040572	0.0000169	0.43123802	0.116877488	0.180966611
ENSG00000144827	ABHD10	15.231312	15.489932	15.887962	12.41662	13.147476	12.996957	16.146745	15.804382	17.325559	0.088380234	-0.147565844	0.026783174	0.180972114
ENSG00000135972	MRPS9	25.211222	25.801761	25.828561	21.455708	21.986388	20.978812	27.489904	25.135291	29.05618	0.255190252	-0.104027897	0.031766204	0.181004382
ENSG00000249853	HS3ST5	0.269536	0.234693	0.380385	0.167613	0.085367	0.125538	0.229063	0.302909	0.323888	--	--	0.708502568	0.181099267
ENSG00000113504	SLC12A7	19.668491	18.20932	16.244591	31.779478	30.290101	31.370235	19.609464	17.018667	21.014239	4.11E-46	0.934801386	0.04550373	0.18122248
ENSG00000109685	NSD2	45.688071	45.752222	43.48973	42.607016	39.601767	41.43732	44.805967	42.876245	52.357452	0.817601831	-0.016926362	0.004932774	0.181240235
ENSG00000249915	PDCD6	53.538949	56.226877	59.081503	49.880117	50.295649	51.08153	60.169002	61.564217	58.280963	0.870039748	-0.017130059	0.026216578	0.181286365
ENSG00000134575	ACP2	38.461496	37.203285	39.175215	48.35867	48.239366	49.18766	39.09737	40.397515	44.155782	8.74E-21	0.521129276	0.026828919	0.181332849
ENSG00000012504	NR1H4	9.153592	10.376736	10.179729	13.030392	15.209019	14.065248	10.765493	9.845587	9.558088	5.38E-10	0.62429439	0.153947833	0.181373455

Gene ID	Gene name	Ctrl-1 FPKM	Ctrl-2 FPKM	Ctrl-3 FPKM	IFNγ-1 FPKM	IFNγ-2 FPKM	IFNγ-3 FPKM	Sora-1 FPKM	Sora-2 FPKM	Sora-3 FPKM	Ctrl vs IFNγ DESeq2 FDR	Ctrl vs IFNγ DESeq2 log2FC	Ctrl vs Sora DESeq2 FDR	Ctrl vs Sora DESeq2 log2FC
ENSG00000117697	NSL1	22.839315	22.309936	21.028533	19.517971	20.257917	20.496921	23.870705	23.250612	23.853005	0.901330634	0.014016272	0.020639734	0.18157868
ENSG00000160062	ZBTB8A	1.061895	1.056711	1.170858	0.998391	1.164836	0.871015	1.409704	1.266411	1.224143	0.943687676	0.017979539	0.318360662	0.181605727
ENSG00000188643	S100A16	71.292121	69.539701	66.438648	56.251169	60.798656	60.848797	70.274054	71.684062	77.298848	0.487961229	-0.062469221	0.011489354	0.181667407
ENSG00000103423	DNAJA3	29.262774	29.202485	29.117895	28.956389	27.09478	26.646926	29.951491	30.848375	31.934756	0.508771073	0.052914017	0.008880491	0.181672859
ENSG00000112624	BICRAL	2.443597	2.512313	2.631705	2.462629	2.399126	2.450927	2.797789	2.419391	2.865987	0.468166947	0.095244481	0.122248691	0.181860351
ENSG00000122484	RPAP2	0.496739	0.517575	0.521742	0.577873	0.479461	0.556341	0.56007	0.499839	0.576321	0.167495506	0.219163495	0.227481023	0.181866755
ENSG00000112367	FIG4	6.575057	6.74883	7.0043	4.86865	4.961703	4.673596	7.250564	6.973529	7.293414	0.001271821	-0.335063159	0.079134502	0.181896488
ENSG00000147121	KRBOX4	3.660189	3.740594	4.572037	3.690418	2.661979	3.249034	4.635964	3.787429	3.55921	0.378515303	-0.159320061	0.251866455	0.18195688
ENSG00000141560	FN3KRP	10.410166	11.254444	9.14015	8.826095	10.180854	10.034448	11.844201	10.363238	10.239905	0.636761614	0.07677527	0.166739367	0.18206206
ENSG00000234545	FAM133B	12.065589	13.136128	15.018062	12.801628	11.200777	13.40344	14.060652	12.539971	14.994172	0.796933828	0.037983584	0.087497871	0.182340799
ENSG00000070476	ZXDC	8.886746	8.349697	8.985715	9.689177	8.646669	9.46883	9.913091	8.524482	10.029881	0.005749087	0.247056081	0.055974709	0.18237422
ENSG00000138698	RAP1GDS1	6.511367	5.800552	6.222711	5.661937	5.393571	5.26473	7.425389	6.70418	7.018503	0.196157222	-0.158517527	0.12587039	0.182418133
ENSG00000204248	COL11A2	0.77272	0.464811	0.461665	0.193816	0.141497	0.213048	0.629126	0.562932	0.587036	0.00000466	-1.432612623	0.536817243	0.182484367
ENSG00000180574	EIF2S3B	2.436269	2.648503	2.34625	2.29115	2.396728	2.709028	2.7448	2.522048	2.651736	0.598604664	0.139620315	0.408066277	0.18260647
ENSG00000065923	SLC9A7	0.773914	0.818236	0.798392	0.755729	0.777523	0.636349	0.901291	0.690742	0.963044	0.982392413	0.005640064	0.287970706	0.182743626
ENSG00000108826	MRPL27	40.100039	48.508131	40.559418	39.342351	38.386168	39.217314	47.956193	51.846154	48.878689	0.358260968	-0.088735375	0.043351669	0.18288921
ENSG00000177879	AP3S1	21.252096	21.938295	21.868816	21.898113	21.368451	21.569182	24.06431	23.647264	21.34058	0.083643975	0.142974407	0.059810048	0.182934672
ENSG00000153904	DDAH1	16.731184	19.228491	19.598282	17.341506	16.317632	16.486792	20.058015	18.855257	20.262924	0.99775749	-0.000505681	0.027527597	0.182935551
ENSG00000073578	SDHA	63.72811	60.41503	59.131924	59.846166	60.630625	59.218195	65.071903	64.036647	68.171159	0.077594628	0.116089896	0.005738876	0.182970669
ENSG00000204498	NFKBIL1	21.095058	20.40529	19.635685	17.687628	19.509218	19.964792	20.959409	22.588542	21.690073	0.669612923	0.053689844	0.058921763	0.183039736
ENSG00000163902	RPN1	162.747639	157.609352	144.850585	163.134718	165.302693	160.129299	161.076591	163.531552	171.246254	0.000191173	0.217700492	0.005446377	0.183107732
ENSG00000080608	PUM3	26.612316	29.977274	32.539973	26.345873	23.245079	25.004877	29.908447	32.044424	32.622135	0.286501381	-0.10629368	0.046602957	0.183126094
ENSG00000167969	ECI1	62.085337	58.294323	52.720918	48.493628	50.27137	52.179308	58.160437	62.742595	64.079665	0.773354751	-0.0330644	0.027509802	0.183132734
ENSG00000123094	RASSF8	5.193128	5.637921	5.138326	5.604814	5.296041	5.277546	6.286562	5.181692	5.633218	0.076851823	0.16489334	0.075581511	0.183154405
ENSG00000143375	CGN	16.577711	14.746984	13.65874	11.463522	11.311842	10.936201	15.357734	15.838398	16.857553	0.000898879	-0.271980318	0.037469857	0.183154993
ENSG00000167306	MYO5B	2.548917	2.147027	2.496742	3.892938	3.963713	3.495128	2.629365	2.302645	2.482311	1.85E-26	0.899896862	0.089756897	0.183272988
ENSG00000105974	CAV1	5.908982	6.153816	6.233811	3.061037	3.570344	3.038166	6.803769	6.309143	6.949248	5.75E-12	-0.831288261	0.101044815	0.183288677
ENSG00000123838	C4BPA	95.794522	96.788252	88.953061	332.130942	345.104895	344.970508	98.946642	102.075089	98.698588	0	2.006747836	0.007810978	0.183296695
ENSG00000072401	UBE2D1	8.921693	11.26882	10.650785	10.499253	11.31305	10.773772	10.286462	11.230125	11.316487	0.039272411	0.227680822	0.111947663	0.18330997
ENSG00000112039	FANCE	7.311584	6.149129	6.41049	5.385585	4.872038	5.503822	7.644374	6.802497	6.728993	0.165783663	-0.186109177	0.15311119	0.183336981
ENSG00000174672	BRSK2	10.756202	9.270049	10.145223	8.903055	8.523497	8.243841	9.518637	10.959562	11.877377	0.375292738	-0.098573837	0.075050136	0.183508268
ENSG00000131759	RARA	14.581035	13.746891	11.730444	12.965889	12.639273	13.295805	14.101318	13.704213	15.956017	0.151915243	0.142650616	0.054732681	0.18353965
ENSG00000110107	PRPF19	110.932394	112.445271	105.17525	101.554135	96.802386	102.253734	112.734968	116.534536	120.698018	0.810512876	0.018565341	0.003397231	0.183654823
ENSG00000197345	MRPL21	98.026181	98.777253	86.82531	81.395052	81.297376	86.206451	98.004504	104.500558	96.057258	0.744131125	-0.032876921	0.035046884	0.183811203
ENSG00000086598	TMED2	86.634573	92.169273	89.026576	106.397928	102.383234	105.704642	97.619628	92.419521	95.472452	2.29E-15	0.37969988	0.002707885	0.183821861
ENSG00000164120	HPGD	13.244776	16.67004	18.782171	7.537972	6.289078	6.935866	17.177672	16.692018	18.621038	8.91E-23	-1.148130146	0.166302622	0.183828846
ENSG00000109265	KIAA1211	4.166434	3.681506	3.094348	2.282816	2.390603	2.190935	4.281109	3.578867	4.416027	0.00000723	-0.563245698	0.160410421	0.18404875
ENSG00000087460	GNAS	580.378763	540.293282	548.490731	510.999047	512.429424	503.530357	570.983702	574.499643	633.70286	0.747488932	0.0223858	0.001737407	0.184166225
ENSG00000124733	MEA1	36.609135	33.206258	33.509585	33.139909	31.929289	29.553945	34.720325	37.930708	35.400979	0.710662327	0.037208857	0.026018391	0.184176466
ENSG00000146963	LUC7L2	54.151409	58.897659	61.133796	55.396906	55.692291	55.381734	61.42564	55.232657	66.753754	0.422372218	0.059450953	0.011112273	0.184460221
ENSG00000101940	WDR13	5.727964	4.406407	3.898259	4.346362	3.896984	3.188379	5.972857	5.510489	5.743562	0.004682852	-0.371488018	0.160410421	0.184521878
ENSG00000090097	PCBP4	37.695747	38.292879	35.698428	26.011529	23.860932	24.456427	39.064469	39.462995	41.714058	1.77E-11	-0.436491335	0.014961068	0.184738548
ENSG00000198060	5-Mar	12.052969	13.211844	14.332124	11.79595	12.158109	12.101208	14.105953	13.696382	14.398861	0.919409805	0.013096785	0.035746483	0.184849387
ENSG00000147883	CDKN2B	12.743374	11.988609	16.509736	2.565899	3.037088	2.964348	11.966876	14.382377	17.314332	7.62E-70	-2.107207786	0.464902231	0.184914234
ENSG00000140319	SRP14	364.864046	355.816078	336.868611	300.878243	307.531286	303.006644	369.28819	387.047237	374.703599	0.28877032	-0.067736853	0.005566954	0.184943674
ENSG00000166825	ANPEP	37.301583	28.470883	25.533445	29.351279	30.0674	27.836773	32.326107	32.042299	33.853685	0.784402651	0.074783847	0.121546214	0.18514373
ENSG00000156110	ADK	17.906442	15.420439	13.237784	15.532303	17.18256	12.55485	13.778296	17.850232	13.536069	0.537220785	0.072599587	0.051569551	0.18526325
ENSG00000239672	NME1	139.629497	160.64503	142.322453	129.272522	88.977951	107.953301	151.456837	162.229348	165.113516	0.001853217	-0.308610476	0.015294356	0.185264625
ENSG00000107862	GBF1	13.603719	11.820093	12.258139	13.548943	12.330539	12.391732	13.471054	11.984906	14.817652	0.032376163	0.168407069	0.033108137	0.185342666
ENSG00000101246	ARFRP1	7.868342	9.530626	8.95564	7.800958	8.600375	7.059982	9.722734	9.055069	9.620408	0.965075207	-0.007458107	0.09301856	0.18536897
ENSG00000101574	METTL4	5.263633	6.435067	6.081819	5.20358	5.191519	4.886313	6.325239	5.772835	5.99507	0.705115068	-0.060743405	0.135951134	0.185373314
ENSG00000004779	NDUFAB1	84.579612	88.118279	86.613078	89.938667	85.512652	95.668089	90.226415	94.692886	89.701199	0.003602911	0.209171819	0.020985651	0.185394275
ENSG00000108960	MMD	54.035117	61.838133	75.47697	59.745956	67.296454	61.969893	62.258344	64.520963	78.515104	0.53817511	0.128921894	0.360174187	0.185440327
ENSG00000138131	LOXL4	15.27905	15.339282	15.921403	5.744874	5.502081	6.014038	15.664887	15.836669	18.185957	1.76E-69	-1.281760501	0.020005025	0.185469979
ENSG00000115109	EPB41L5	15.314873	16.026744	14.581008	14.755732	14.024458	13.743107	19.722989	19.061134	19.116203	0.88113175	0.015282257	0.015657925	0.185838088
ENSG00000273590	SMIM11B	5.069333	7.354386	8.276152	6.532882	6.430552	6.659588	8.357715	5.269972	8.376495	0.953204292	0.016315135	0.343171644	0.185855506
ENSG00000123143	PKN1	58.279042	54.011437	49.550132	44.397682	45.464675	46.511241	55.606338	54.193792	63.108695	0.217490258	-0.098926679	0.01799171	0.186041153
ENSG00000054983	GALC	9.482815	8.949775	9.747828	9.307413	10.794182	9.034549	9.677767	10.045636	10.592361	0.0261848	0.196435986	0.02844861	0.18609908
ENSG00000150779	TIMM8B	24.264105	22.454498	22.626285	21.613516	22.603658	25.064809	24.269625	31.529388	19.249167	0.418979827	0.105660513	0.238265634	0.186237288
ENSG00000137364	TPMT	14.010786	16.611326	17.350407	15.608238	13.732339	15.586332	16.98827	17.833038	16.265219	0.669741566	0.053173486	0.07404295	0.186270987
ENSG00000175182	FAM131A	3.662878	3.022411	3.598188	2.583907	2.910871	3.25987	3.614968	3.363571	4.105969	0.79071149	0.058175985	0.267202887	0.186282896
ENSG00000118689	FOXO3	7.626142	7.074557	6.985455	6.270822	6.356328	6.202372	8.40104	6.720675	8.060036	0.534910798	-0.059015619	0.04620785	0.186348974
ENSG00000285304	Z83844.3	2.328686	0.597105	3.202029	1.191854	4.173717	2.021351	1.335933	0.490106	1.003676	0.736301226	0.424032393	0.877927524	0.186382779
ENSG00000163527	STT3B	46.504169	49.569622	48.381744	53.588181	53.541016	52.524227	51.929741	49.526718	52.712139	6.67E-09	0.291832215	0.00180242	0.186409347
ENSG00000187778	MCRS1	52.753863	47.435753	46.454622	44.452341	40.98951	44.427479	49.038375	54.32273	52.475362	0.720925121	-0.033475063	0.015913544	0.186459083
ENSG00000060762	MPC1	59.016477	68.161611	71.257489	58.297219	67.592399	61.492882	71.10138	68.658157	77.109237	0.58630852	0.061277981	0.032942475	0.186532288
ENSG00000158769	F11R	79.940097	76.816256	76.103554	61.441668	64.022052	59.963272	79.994978	82.365334	89.798433	0.001163946	-0.180495703	0.005795797	0.186828804
ENSG00000178921	PFAS	11.929791	11.906009	12.027544	9.252003	8.427761	9.500932	12.818172	11.339885	14.69866	0.00064625	-0.259379046	0.028602337	0.18685676
ENSG00000196636	SDHAF3	6.487496	8.425689	6.98521	6.609253	7.379453	7.065842	7.384689	7.498203	7.309045	0.78013289	0.051406516	0.173809599	0.186858388
ENSG00000121578	B4GALT4	9.806457	8.467201	8.824843	9.429163	9.626211	8.749005	11.197134	9.753285	9.447296	0.003957127	0.256773384	0.065115452	0.186999468
ENSG00000036672	USP2	0.632317	0.73455	0.442575	0.259714	0.503788	0.289114	0.554666	0.674658	0.985988	0.272821468	-0.451021495	0.565462152	0.1871027
ENSG00000005001	PRSS22	1.200735	0.620911	0.877882	0.884686	0.54373	0.76408	0.947127	1.300588	0.593684	0.828494394	-0.126005049	0.70580001	0.187106604
ENSG00000149476	TKFC	11.410722	11.438355	11.133656	10.214467	8.965536	10.099254	10.671882	10.830889	12.405582	0.294954729	-0.107759517	0.043880264	0.187288071
ENSG00000176490	DIRAS1	2.381803	1.948332	2.433426	1.86541	1.703975	1.708933	2.189685	2.665122	2.285729	0.275017692	-0.210262451	0.312892238	0.187349057

Gene ID	Gene name	Ctrl-1 FPKM	Ctrl-2 FPKM	Ctrl-3 FPKM	IFNγ-1 FPKM	IFNγ-2 FPKM	IFNγ-3 FPKM	Sora-1 FPKM	Sora-2 FPKM	Sora-3 FPKM	Ctrl vs IFNγ DESeq2 FDR	Ctrl vs IFNγ DESeq2 log2FC	Ctrl vs Sora DESeq2 FDR	Ctrl vs Sora DESeq2 log2FC
ENSG00000117298	ECE1	16.237132	13.363013	12.191348	13.616659	12.324788	13.510058	14.79971	13.693275	16.10123	0.561945236	0.072634476	0.075795652	0.187350502
ENSG00000198026	ZNF335	8.268308	8.277741	7.974363	7.422629	7.072237	7.183931	8.427855	7.68643	10.153513	0.780303356	-0.03106901	0.064037227	0.187575304
ENSG00000187801	ZFP69B	1.860286	2.210306	1.871548	1.421705	1.614092	1.435108	1.699866	2.284493	2.407106	0.250251635	-0.2833279	0.435667684	0.187636653
ENSG00000143622	RIT1	7.036221	7.444136	7.59441	5.694517	6.132256	6.147518	7.804364	7.395586	8.962731	0.216445726	-0.137052096	0.055497781	0.187656617
NewGene_510	--	3.967123	4.443075	3.473277	2.970321	3.644645	3.694933	4.092531	4.783993	3.734669	0.663413193	-0.120703894	0.407209122	0.187701126
NewGene_4568	--	0.49404	0.582118	1.064614	1.091377	1.070819	0.49023	0.649871	1.184675	0.448454	0.303398559	0.451717696	0.689541448	0.18794106
ENSG00000155085	AK9	1.409355	1.123851	1.513397	1.140525	0.601561	1.220248	1.633767	1.366002	1.75983	0.313770974	-0.260880523	0.388179223	0.188027952
ENSG00000136261	BZW2	123.920236	120.35613	137.999612	96.789442	92.539987	92.77685	127.82631	131.21412	144.525545	0.00000246	-0.265425667	0.004661503	0.188270898
ENSG00000156273	BACH1	15.833072	17.176519	18.175712	25.408566	25.265737	24.808418	17.607702	17.517573	19.51839	1.06E-31	0.704854342	0.014333938	0.188279135
ENSG00000162885	B3GALNT2	7.255961	7.853861	6.88845	6.515769	6.234722	6.592645	7.956405	7.230788	8.187149	0.811423463	-0.039408591	0.124747176	0.188382245
ENSG00000173540	GMPPB	5.20138	5.251429	4.647127	5.161041	5.212826	5.779327	5.53824	6.006523	5.920623	0.145664952	0.163608185	0.06987834	0.188390579
ENSG00000152767	FARP1	22.703774	21.29451	24.308866	13.95105	13.056891	12.963199	25.774809	22.561989	26.190928	8.23E-26	-0.650700403	0.014135226	0.188399628
ENSG00000067560	RHOA	161.171475	166.062461	173.337587	149.487376	149.292732	152.082953	175.588728	173.979915	182.05121	0.995201613	0.000548553	0.001123782	0.188628328
ENSG00000175048	ZDHHC14	3.499893	3.451969	2.791587	4.193959	3.677892	4.467137	3.113902	3.571737	4.291293	0.0000107	0.483713819	0.11835193	0.188800876
ENSG00000126524	SBDS	41.939207	43.340044	46.918832	33.970327	36.012155	32.819154	45.300711	45.606984	50.192027	0.017484344	-0.195265967	0.018935641	0.188852788
ENSG00000138829	FBN2	2.255117	2.779542	2.930751	1.313495	1.506857	1.386114	2.86243	2.643046	3.033097	7.12E-12	-0.754831171	0.110780158	0.188858246
ENSG00000144589	STK11IP	5.890059	6.434426	6.341076	5.029498	4.933137	4.748079	7.123371	6.532794	8.156998	0.035211942	-0.231286895	0.134369807	0.188900724
ENSG00000185504	FAAP100	11.97016	11.453899	11.582388	9.701745	10.172882	11.43169	11.886227	11.368848	14.614102	0.64925479	-0.053316464	0.045423035	0.188901545
ENSG00000198517	MAFK	11.567199	11.894286	13.289161	9.642558	9.340966	10.783295	12.230369	12.617295	13.760134	0.163034718	-0.143757184	0.038114882	0.189168375
ENSG00000214595	EML6	0.577474	0.576265	0.61924	0.474508	0.484724	0.416046	0.649703	0.492827	0.762934	0.336788989	-0.220493895	0.386725831	0.189284226
ENSG00000134371	CDC73	16.034286	17.679186	17.819742	14.113415	13.965467	13.723875	19.472147	16.818196	20.118176	0.031885034	-0.151947254	0.015453381	0.189303472
ENSG00000135454	B4GALNT1	2.820787	3.082118	1.892424	1.165101	1.598931	1.739416	2.418614	2.416987	2.888312	0.007374992	-0.647766756	0.39655139	0.189351584
ENSG00000091164	TXNL1	8.671532	9.479867	9.941949	10.388993	8.431688	10.612883	12.75406	11.672569	12.171691	0.8731785	-0.016148534	0.008939006	0.189502102
ENSG00000174738	NR1D2	13.602007	15.64874	16.148532	14.618877	14.964057	14.945561	17.091419	15.07751	16.70698	0.181343408	0.116590095	0.027100567	0.189585567
ENSG00000114942	EEF1B2	518.356673	483.483538	476.55287	388.136115	364.091199	392.579951	514.778193	545.505964	507.25801	0.000430858	-0.199323693	0.003541672	0.189738756
NewGene_6662	--	4.721929	4.760564	7.831545	5.788913	5.067349	5.817342	6.43152	6.421057	5.600025	0.742176808	0.095056053	0.421141076	0.189887349
ENSG00000138823	MTTP	29.45003	29.395735	22.858427	39.345154	43.225401	38.37656	29.984331	30.541243	27.370171	8.87E-20	0.741987813	0.063406024	0.190004538
ENSG00000112763	BTN2A1	10.009214	9.479911	10.267792	10.86092	9.539326	9.42099	10.93487	10.039749	11.15328	0.154339596	0.146694724	0.038722695	0.190101759
ENSG00000137936	BCAR3	3.316472	3.356552	2.944207	2.352179	2.423559	2.289636	3.389974	2.622902	3.488061	0.096139651	-0.278552384	0.254646065	0.190106567
ENSG00000100726	TELO2	7.831587	8.607599	7.560014	6.280087	5.101014	6.320353	7.883046	7.67209	10.183288	0.011727793	-0.283231108	0.111468923	0.190190635
ENSG00000059758	CDK17	8.024314	9.031605	8.886732	7.734897	7.522568	6.553514	9.199874	8.969451	10.297178	0.431823652	-0.093238058	0.05182201	0.190215745
ENSG00000167962	ZNF598	10.544573	9.824997	11.105867	9.867932	9.12518	10.472498	11.656303	9.655474	12.515511	0.85863081	0.023472834	0.060215057	0.190272097
ENSG00000131669	NINJ1	42.691013	40.198711	40.163422	33.654541	36.162247	32.990753	39.222473	44.089417	48.460171	0.207604934	-0.112227854	0.037806312	0.190299557
ENSG00000114796	KLHL24	9.687125	9.479478	10.443405	8.629011	9.240904	9.307196	11.062861	10.061513	10.938356	0.954329006	-0.007173636	0.014967099	0.190440795
ENSG00000163681	SLMAP	11.988754	14.180044	14.107857	13.709952	14.49995	13.020529	14.46889	14.203625	15.955367	0.025955896	0.182696012	0.027630674	0.19049251
ENSG00000147676	MAL2	119.507099	145.335754	145.121403	211.691635	222.902133	212.972906	143.896253	137.080465	158.144476	1.95E-32	0.809025558	0.021989455	0.190502324
ENSG00000132635	PCED1A	20.610106	21.201221	20.30838	18.930941	19.083807	16.527988	21.374268	21.45734	22.162191	0.77527327	-0.034320262	0.018935641	0.190505964
ENSG00000133612	AGAP3	25.708724	22.672268	23.460689	21.664345	20.976376	22.155134	24.911203	25.016781	28.577808	0.928861081	-0.010079557	0.01528844	0.190587985
ENSG00000213722	DDAH2	110.981288	103.652818	91.751579	93.017067	92.791602	98.781442	103.981011	108.607656	112.407861	0.40591535	0.074256113	0.017669187	0.190630024
ENSG00000125844	RRBP1	72.188588	67.124491	64.227924	72.30762	72.001124	70.295573	75.075423	68.914047	76.915156	0.006856668	0.165398723	0.004104111	0.190658321
ENSG00000130988	RGN	8.618737	7.249494	6.566705	8.741841	8.609274	7.770082	8.938732	7.185628	7.520538	0.041309707	0.275995225	0.200306673	0.190680974
ENSG00000054179	ENTPD2	1.776571	1.360456	0.912628	0.760653	1.189311	1.018665	1.599433	1.188315	1.583428	0.427074339	-0.299535477	0.54728341	0.190767109
ENSG00000257017	HP	0.816372	1.092278	0.749253	1.853543	1.529226	1.830558	1.098264	1.040733	0.990918	0.000545928	1.02783441	0.638550337	0.190791104
ENSG00000068697	LAPTM4A	65.024467	66.752602	63.992413	68.176353	70.004593	71.763803	69.085297	69.562584	70.842789	0.0000174	0.248816784	0.003123339	0.190851726
ENSG00000175634	RPS6KB2	85.46844	80.037732	74.07663	76.674232	81.446536	79.709854	81.600248	84.760988	89.41937	0.066950414	0.137184917	0.010724085	0.191007298
ENSG00000049449	RCN1	96.296518	95.109919	103.276865	169.453789	156.46249	163.532519	100.119854	106.747119	108.179166	1.19E-68	0.878562579	0.00491341	0.191098249
ENSG00000105323	HNRNPUL1	110.754213	110.803996	106.850177	101.725233	101.96897	101.741381	113.482318	113.715419	123.358841	0.453528678	0.045679315	0.000877498	0.191121379
ENSG00000120438	TCP1	109.409034	113.193264	121.276115	102.763095	100.409015	98.217492	127.676333	124.806784	128.82106	0.326557793	-0.06427063	0.00230951	0.191227119
NewGene_283	--	10.264376	10.265913	10.424342	8.197312	8.438303	6.857183	11.280329	10.814942	11.0737	0.026619615	-0.268293793	0.061235844	0.191290457
ENSG00000181019	NQO1	4.193795	4.275581	4.59945	2.060903	3.147213	2.107804	4.416444	4.396609	5.066383	0.000102018	-0.673999667	0.192278243	0.191318922
ENSG00000221926	TRIM16	4.462211	4.752145	4.874707	3.972719	3.592282	4.614847	5.229696	4.553268	5.002288	0.588430787	-0.085383965	0.115737219	0.191336418
ENSG00000100596	SPTLC2	1.976846	1.902644	1.814351	1.535574	1.796866	1.616819	2.150745	2.128201	2.012879	0.526388098	-0.095191476	0.113841193	0.191342563
ENSG00000025770	NCAPH2	31.520691	31.758419	30.657494	29.886191	29.463211	29.52914	32.438587	35.592742	36.497841	0.363307202	0.071748611	0.011098011	0.19145685
ENSG00000169814	BTD	13.861863	11.417147	11.754078	10.908059	14.726941	15.965921	15.364087	14.797332	12.499914	0.16740702	0.148256667	0.079291726	0.191494926
NewGene_4780	--	6.199265	5.830058	5.74965	5.80361	5.979119	6.492054	6.778155	6.261475	5.993688	0.119705756	0.187594058	0.111947483	0.191498222
ENSG00000163541	SUCLG1	48.310455	46.818905	43.541988	35.001308	38.848469	38.783516	47.257881	50.925144	50.270432	0.077129858	-0.151963237	0.018178567	0.191704824
ENSG00000129566	TEP1	2.730403	2.861628	2.82539	3.73446	3.523044	3.106117	2.802891	2.887945	2.84378	0.008857527	0.276728812	0.097970876	0.191791207
ENSG00000165661	QSOX2	13.571353	14.152326	14.789732	11.934229	11.21403	12.067362	14.593362	14.235368	16.81662	0.112239868	-0.124503172	0.017501046	0.191842214
ENSG00000127554	GFER	12.044093	11.788908	10.700044	10.759197	11.062301	12.061205	12.379781	12.337311	12.976862	0.406018705	0.09693404	0.053059292	0.191930674
ENSG00000112394	SLC16A10	5.003096	5.034029	4.7547	2.818748	3.88704	3.711695	6.918397	6.3898	6.744628	0.003697118	-0.328526202	0.050994204	0.192176485
ENSG00000146833	TRIM4	12.6432	11.17208	12.010386	10.35941	10.449283	11.503514	13.164454	11.839806	15.792735	0.899142427	-0.016378127	0.059055395	0.192197912
ENSG00000100836	PABPN1	64.095064	67.798761	68.183115	63.744659	65.769284	64.287679	67.918289	71.615204	68.591771	0.112780621	0.102028645	0.006272955	0.192213295
ENSG00000143178	TBX19	0.631516	0.500213	0.636169	0.487688	0.505578	0.432456	0.638855	0.667954	0.731895	0.928013124	-0.047844726	0.597525945	0.192219325
ENSG00000165271	NOL6	16.134789	15.961259	16.114292	14.371032	12.842765	13.07479	16.177648	16.624486	18.936507	0.113787573	-0.117771024	0.011126177	0.192488776
ENSG00000180479	ZNF571	1.229481	1.621181	0.957984	0.959331	1.003957	1.188303	0.958448	0.941692	1.214473	0.444873104	-0.223049606	0.438386835	0.192555244
ENSG00000079459	FDFT1	139.33018	116.135739	102.746177	171.476765	153.986215	163.842173	129.833033	138.092412	126.740292	9.31E-16	0.576285145	0.027911145	0.192566779
NewGene_1345	--	0.485004	0.534996	0.402064	0.693451	0.459747	0.441741	0.546304	0.61151	0.348154	0.434776317	0.31964965	0.63555484	0.1926848
ENSG00000181029	TRAPPC5	8.852148	8.358752	5.075461	5.307858	7.903325	5.945204	7.885955	10.391967	7.301844	0.620761547	-0.086162836	0.148509961	0.192746796
ENSG00000188612	SUMO2	100.707883	98.490491	94.375167	88.994967	88.926349	87.80236	100.665456	99.269925	110.758006	0.024285353	0.124348649	0.003005574	0.192747347
ENSG00000073050	XRCC1	11.222398	10.166048	9.804553	6.955368	6.595136	7.03606	11.304635	9.981412	11.858008	0.0000165	-0.403209311	0.046604969	0.19277016
ENSG00000166295	ANAPC16	13.553047	15.175157	13.930124	10.769165	11.23573	10.487161	14.849175	14.461123	16.12046	0.002933943	-0.24685603	0.018636272	0.192942633
ENSG00000137513	NARS2	12.670247	12.389494	12.011493	9.987069	10.021589	9.391875	13.319036	13.340202	13.727053	0.040746066	-0.191436517	0.035662534	0.19297182
ENSG00000175318	GRAMD2A	0.952432	1.22649	1.173371	10.391607	9.139319	9.127054	1.246433	0.876367	1.424231	2.75E-171	3.256945511	0.457461487	0.192999596
ENSG00000145242	EPHA5	0.506807	0.512839	0.663593	1.292649	1.195404	1.210738	0.71617	0.499646	0.669634	7.87E-18	1.296599857	0.396740104	0.193022567

Gene ID	Gene name	Ctrl-1 FPKM	Ctrl-2 FPKM	Ctrl-3 FPKM	IFNγ-1 FPKM	IFNγ-2 FPKM	IFNγ-3 FPKM	Sora-1 FPKM	Sora-2 FPKM	Sora-3 FPKM	Ctrl vs IFNγ DESeq2 FDR	Ctrl vs IFNγ DESeq2 log2FC	Ctrl vs Sora DESeq2 FDR	Ctrl vs Sora DESeq2 log2FC
ENSG00000166734	CASC4	11.067534	12.30816	12.727457	14.003925	12.786671	12.657479	13.12064	11.714409	13.963099	0.000695393	0.272637897	0.030808751	0.193201961
ENSG00000133872	SARAF	40.434087	40.892639	38.817533	55.201606	54.032242	51.372314	43.541082	40.387413	44.805618	6.49E-22	0.548913737	0.003977942	0.193268193
ENSG00000163660	CCNL1	64.766236	68.97759	66.58758	87.89664	77.599767	73.562009	80.436152	71.030821	76.404506	6.78E-10	0.352493435	0.002877521	0.193422687
ENSG00000001497	LAS1L	13.581921	13.086969	15.018162	10.833644	10.428764	12.44706	15.168891	14.375754	15.778682	0.036658332	-0.199666364	0.024553235	0.193447159
ENSG00000187699	C2orf88	4.17707	4.016828	4.676383	5.550703	6.239183	5.52938	4.592568	4.843676	4.701026	0.00000007	0.596480614	0.135128714	0.193709508
ENSG00000117280	RAB29	9.106269	8.084499	8.902194	7.547402	7.856156	7.331531	8.211933	8.103424	10.754434	0.647091532	-0.057806276	0.114308073	0.193886947
ENSG00000253846	PCDHGA10	1.844945	1.722886	1.472002	1.253756	1.230772	1.392989	1.739023	1.65168	2.019136	0.208051697	-0.238254963	0.256536876	0.193906975
ENSG00000130560	UBAC1	16.329933	15.36225	13.843096	13.292584	12.612745	13.283965	17.238604	14.70033	16.987492	0.573447371	-0.0697653	0.064877969	0.193951788
ENSG00000066651	TRMT11	15.726601	19.311836	21.394376	16.521074	15.915729	15.585106	19.752134	21.421771	21.200263	0.823527431	-0.031610813	0.060876898	0.19398892
ENSG00000120341	SEC16B	2.584337	2.70997	2.261311	12.034365	10.775528	11.280961	2.77045	2.549604	2.781411	5.96E-147	2.301584065	0.183928205	0.194022633
ENSG00000155959	VBP1	23.088648	24.497332	23.705744	21.182178	21.467846	21.426435	25.850201	25.492762	25.073912	0.961700555	-0.005997777	0.022103523	0.194030774
ENSG00000162344	FGF19	4.909478	4.548624	5.120712	2.949403	2.779476	3.304641	4.246518	5.324729	6.094411	0.0000913	-0.541109067	0.220349856	0.194031485
ENSG00000101224	CDC25B	13.533994	14.610246	13.6809	31.423566	28.10898	31.283007	14.547082	15.210357	17.102254	4.18E-85	1.229139786	0.014865207	0.194059782
ENSG00000081177	EXD2	6.978261	4.970603	4.843458	5.761222	4.736933	4.007275	6.087707	6.921911	7.239393	0.296389022	-0.126049137	0.082605057	0.194128683
ENSG00000182919	C11orf54	11.170254	12.024433	13.750995	11.776082	11.20405	10.204521	12.748027	10.961018	12.139295	0.713953442	0.044628951	0.041744311	0.194193964
ENSG00000004059	ARF5	79.632408	74.354805	73.14524	59.638783	61.795747	67.413244	78.35511	79.154604	86.414166	0.155615361	-0.119363814	0.007459505	0.194304526
ENSG00000082641	NFE2L1	59.892346	54.92916	51.701985	48.147008	47.52861	48.326962	57.596697	57.01799	64.147679	0.414626109	-0.063362886	0.007287923	0.194366268
ENSG00000164116	GUCY1A1	0.776437	0.981246	0.936992	1.216196	1.109059	1.293095	0.88809	0.845043	0.734581	0.014848544	0.632193222	0.522312488	0.194551073
ENSG00000127445	PIN1	41.133258	38.048359	35.262039	32.801475	34.545221	33.66008	41.246793	40.306931	40.746091	0.812230324	-0.029996581	0.035972922	0.194584255
ENSG00000174442	ZWILCH	11.230079	13.237948	13.127735	12.271913	10.276168	12.117739	13.488583	13.313468	14.238354	0.999571371	0.0000681	0.021349424	0.194622029
ENSG00000137822	TUBGCP4	7.964899	8.012931	8.897067	8.129896	7.806069	7.619392	8.669386	9.199399	9.965191	0.775078831	0.028457713	0.020884332	0.194696705
ENSG00000185252	ZNF74	4.72703	5.089162	4.910079	3.471986	3.395435	3.86092	5.489442	4.798616	6.087082	0.019040229	-0.288157941	0.10748862	0.194850147
ENSG00000076984	MAP2K7	16.956644	17.714323	16.617287	14.443687	15.609959	15.129219	17.663239	18.332985	19.065071	0.706647526	-0.037627188	0.010228454	0.194938449
ENSG00000149179	C11orf49	12.770746	11.635087	10.961734	7.850616	8.628256	7.676356	11.544802	12.691249	14.327658	0.000475362	-0.407505325	0.103036122	0.195003496
ENSG00000174669	SLC29A2	63.256744	62.994699	64.116263	56.953455	56.241679	55.465059	63.289765	64.285581	77.519686	0.570948405	-0.039132142	0.010351783	0.19500855
ENSG00000112531	QKI	18.244275	18.311205	18.761029	15.364885	16.297325	17.831164	21.792599	19.943418	23.182894	0.784214645	0.029028267	0.020107096	0.195104121
ENSG00000174903	RAB1B	268.063726	258.86847	244.008272	217.010245	231.440517	231.375334	264.70765	278.192289	285.022327	0.646566852	-0.036290075	0.002343382	0.195116315
ENSG00000135525	MAP7	12.777411	12.328216	12.585019	10.702112	9.791682	10.222374	13.84953	12.522773	14.401974	0.044819252	-0.152707025	0.010935001	0.195191232
ENSG00000126267	COX6B1	157.939795	162.474508	163.791893	137.886833	136.67376	150.425835	180.353902	205.755625	146.556151	0.119713814	-0.107333028	0.098032359	0.195603261
ENSG00000132376	INPP5K	7.423732	7.169543	6.850438	5.899607	6.66392	6.546129	7.338654	7.106104	8.804584	0.923985945	0.015628626	0.082730548	0.195622724
ENSG00000185359	HGS	33.019798	33.984209	30.692907	30.509154	27.525296	28.250503	31.521423	31.620391	37.824727	0.734609442	-0.032923074	0.020012553	0.195632285
ENSG00000007384	RHBDF1	3.99939	3.658965	4.358926	2.461116	1.885726	2.200959	4.630276	4.271568	4.318349	0.000000369	-0.734243349	0.159732207	0.195684665
ENSG00000129195	PIMREG	18.013952	17.102802	17.688983	12.707268	10.21367	10.944969	18.736758	18.623081	20.071511	0.000000253	-0.485054865	0.026863983	0.195741422
ENSG00000145604	SKP2	49.984943	57.322956	49.091809	49.941133	44.255609	37.723392	67.097542	41.964584	47.926873	0.701726103	-0.036791912	0.012102286	0.195960026
ENSG00000164896	FASTK	20.454471	20.7708	19.226873	20.002291	18.928193	19.46131	20.57963	21.281239	24.908081	0.265907504	0.107051467	0.044000829	0.195979482
ENSG00000183077	AFMID	31.258916	31.01221	31.376659	39.499537	38.324145	38.432322	36.278927	36.183068	29.468768	5.66E-14	0.494890911	0.041685971	0.195981478
ENSG00000163875	MEAF6	18.370621	17.380349	18.796901	12.314054	12.150794	13.008829	19.360267	17.714516	19.543209	0.000000791	-0.42937993	0.026047828	0.1959829
ENSG00000101199	ARFGAP1	15.645225	15.963862	15.796651	16.512973	15.488282	16.959041	16.840364	15.843263	18.687069	0.011115145	0.189422102	0.014222253	0.195985484
ENSG00000101882	NKAP	1.642444	1.271603	1.630895	1.703048	1.538394	1.553752	1.444223	1.608143	1.836773	0.184834127	0.224480781	0.253518479	0.195996084
ENSG00000060566	CREB3L3	2.055837	1.972794	1.010237	4.33326	4.600914	4.983001	1.717734	1.35673	1.656851	0.0000187	1.810960712	0.559374212	0.196097145
ENSG00000174917	C19orf70	23.096677	23.961753	22.557998	20.193762	21.231528	23.316787	26.147927	29.299034	19.537977	0.772162563	0.042251587	0.214264045	0.196222822
ENSG00000132792	CTNNBL1	21.179292	23.262722	25.542243	22.296831	22.372084	22.666685	23.97824	24.856647	25.728126	0.195850818	0.118783935	0.025626026	0.19635049
ENSG00000162300	ZFPL1	31.35197	29.761002	28.770534	29.072927	29.847006	29.251883	31.066001	32.687617	34.57448	0.225434177	0.108124131	0.020815141	0.196366208
ENSG00000104365	IKBKB	7.489085	7.153141	8.1041	6.56597	6.887109	6.681234	7.79757	7.736949	9.318608	0.788285263	-0.034666082	0.051286302	0.196402615
ENSG00000244115	DNAJC25-GNG1	2.852146	2.715289	2.918468	3.638083	2.300441	5.258914	3.423502	2.969716	2.707173	0.045462007	0.548100952	0.383265648	0.196446692
ENSG00000101161	PRPF6	35.675766	32.789555	31.274439	30.187361	30.13666	29.767548	35.519081	32.881622	38.964672	0.999202724	0.000194518	0.011940841	0.196528417
ENSG00000135919	SERPINE2	14.419821	13.3718	13.534295	5.170321	4.84526	4.913396	13.813336	14.202218	16.370038	7.49E-46	-1.321238212	0.044497792	0.196585116
ENSG00000171557	FGG	80.427312	82.950396	70.706775	116.870621	95.935878	105.231113	96.969592	82.718049	75.393369	9.64E-16	0.578875932	0.064261858	0.196741414
ENSG00000147649	MTDH	20.225669	22.348414	22.401516	27.497182	26.173384	27.21831	23.917299	20.389918	26.49448	1.53E-16	0.46763646	0.013393162	0.196898105
ENSG00000134590	RTL8C	17.292027	15.396922	15.711833	12.885539	12.621086	13.105114	17.650557	17.12352	17.271988	0.132353902	-0.179593486	0.075026007	0.196945738
ENSG00000173221	GLRX	4.778462	3.301564	3.381377	7.868125	9.27444	8.510615	4.064777	4.176582	4.181892	4.49E-15	1.319867791	0.392056136	0.196972505
ENSG00000105202	FBL	171.886922	172.032728	168.153296	166.336444	164.072852	166.462294	178.665356	193.76375	181.483088	0.133209106	0.085505573	0.004277627	0.19709512
ENSG00000198677	TTC37	15.548392	15.709549	16.471144	15.436236	14.778273	13.71676	16.869146	16.211496	18.095439	0.285434635	0.075973967	0.003373431	0.197101022
ENSG00000023041	ZDHHC6	18.447123	20.215748	20.539009	18.417218	18.3221	18.899784	22.166184	20.283713	21.129368	0.639181794	0.048805381	0.021275669	0.197404461
ENSG00000198961	PJA2	10.949441	11.768284	9.614726	13.30436	13.582544	12.685817	11.383936	11.430071	11.920817	6.38E-08	0.436768052	0.031463403	0.197492857
ENSG00000005889	ZFX	5.94427	7.095852	6.136127	7.652451	6.819066	5.325911	6.451748	6.441267	6.238913	0.004233229	0.243720609	0.026688378	0.197507835
ENSG00000170310	STX8	6.383456	6.332486	4.930067	4.807866	5.053568	3.77432	6.457255	6.157342	6.513828	0.280191956	-0.235065634	0.271978261	0.197563426
ENSG00000254999	BRK1	84.100014	79.915009	79.418175	61.890133	69.028259	72.399162	88.520988	94.065781	78.531479	0.202159687	-0.111360421	0.035762142	0.197586096
ENSG00000145335	SNCA	1.412671	0.885007	1.116229	0.303103	0.492587	0.525833	0.908057	1.021785	1.677696	0.018815323	-0.97939189	0.624371975	0.197817282
ENSG00000011478	QPCTL	9.36292	9.062967	9.393482	8.539907	8.521169	8.101673	9.551046	10.011766	10.513291	0.960644742	0.007704488	0.053086109	0.197829797
ENSG00000143314	MRPL24	21.101769	19.661716	21.254198	19.75847	20.215972	20.986856	22.450025	25.046079	18.503601	0.278835108	0.132597318	0.178200649	0.197918002
ENSG00000174744	BRMS1	112.715984	101.109363	99.488878	101.043691	99.447477	104.920995	107.733318	118.809328	116.947304	0.165783663	0.112861938	0.016152092	0.197951884
ENSG00000148950	IMMP1L	8.088244	8.287433	8.789607	8.1036	6.65486	7.112802	9.983623	9.919808	8.632739	0.751062949	-0.06636477	0.221512525	0.19802272
ENSG00000205571	SMN2	7.756282	8.388134	9.392866	7.309965	6.716043	6.583472	9.910462	12.037917	7.426735	0.053061186	-0.299579859	0.237491503	0.198027997
ENSG00000151292	CSNK1G3	8.648383	8.797334	9.114285	8.660008	7.141243	7.536923	9.661625	8.293967	9.911886	0.919517538	0.013755657	0.03461079	0.198074118
ENSG00000132016	C19orf57	0.721597	0.730125	0.643479	1.01938	0.967658	1.014059	0.952709	0.469862	1.033055	0.077678402	0.583625812	0.635179546	0.198301203
ENSG00000136273	HUS1	8.231391	9.480155	8.830902	8.046124	7.45547	7.873381	10.790138	9.683973	9.449003	0.649986257	-0.059094397	0.05192003	0.198417307
ENSG00000138303	ASCC1	15.873847	15.990383	15.774564	13.067852	12.909598	13.129912	16.179674	17.044371	17.585129	0.044184329	-0.180943005	0.023085859	0.198481308
ENSG00000110811	P3H3	1.879796	1.43129	1.490789	2.019618	1.881445	2.030699	1.421472	1.855234	1.898535	0.028321746	0.449500293	0.405509451	0.198523315
ENSG00000130816	DNMT1	65.161639	64.204595	63.070895	64.952938	64.259305	65.485617	65.59073	65.956469	77.735862	0.00102707	0.158875797	0.002591282	0.1985606
ENSG00000175221	MED16	37.863214	34.366042	30.60896	31.417916	35.624847	34.754643	36.360343	34.031418	42.274946	0.504983321	0.070820627	0.038110655	0.198653981
ENSG00000131748	STARD3	20.482516	16.057046	16.606684	15.33628	14.729727	15.06307	18.292287	18.652381	20.622365	0.564981043	-0.075416129	0.073130988	0.198672477
ENSG00000171566	PLRG1	25.285906	24.705008	23.804559	24.959232	23.426464	21.711396	29.647057	25.26401	25.046547	0.263355903	0.085290383	0.012812469	0.198686132
ENSG00000148308	GTF3C5	43.389892	43.057233	42.419255	37.604606	37.16477	37.78895	45.867436	43.924242	48.832136	0.719645105	-0.031970261	0.005036915	0.198815092

Gene ID	Gene name	Ctrl-1 FPKM	Ctrl-2 FPKM	Ctrl-3 FPKM	IFNγ-1 FPKM	IFNγ-2 FPKM	IFNγ-3 FPKM	Sora-1 FPKM	Sora-2 FPKM	Sora-3 FPKM	Ctrl vs IFNγ DESeq2 FDR	Ctrl vs IFNγ DESeq2 log2FC	Ctrl vs Sora DESeq2 FDR	Ctrl vs Sora DESeq2 log2FC
ENSG00000178531	CTXN1	8.049429	9.923386	7.502503	4.05027	4.713954	5.639313	8.803781	9.989305	8.527859	0.0000883	-0.668823501	0.226146316	0.199003795
ENSG00000131375	CAPN7	12.191149	12.735143	11.637535	14.769235	15.651556	14.679977	14.028736	12.29787	13.098097	1.22E-10	0.448923177	0.019037984	0.199027931
ENSG00000113273	ARSB	10.104785	10.022278	10.784573	9.221832	8.632262	9.014414	11.071582	10.491084	12.695157	0.355321348	-0.08138887	0.017253228	0.199065957
ENSG00000118260	CREB1	4.964951	5.298382	5.259935	6.249146	5.110654	5.100878	6.395139	5.532209	6.295792	0.059826162	0.172448997	0.023624505	0.199112402
ENSG00000166902	MRPL16	42.624967	39.26019	40.300568	43.322637	41.25298	41.525466	43.642357	45.742012	41.765891	0.012552179	0.19001119	0.028430753	0.199134171
ENSG00000137942	FNBP1L	17.145726	18.537899	17.58355	14.87623	14.159931	13.427503	19.447013	18.548752	18.611355	0.006849749	-0.185975654	0.00569567	0.199238384
ENSG00000241837	ATP5PO	174.293932	170.68822	155.978143	131.1653	139.802584	134.310844	176.748659	187.442905	171.345804	0.0252707	-0.154924354	0.013650915	0.199301336
ENSG00000198925	ATG9A	27.535884	26.232874	24.877642	22.182403	24.102696	23.400867	25.491165	27.262475	30.874671	0.645093327	-0.046555431	0.009024345	0.199363938
ENSG00000109501	WFS1	12.446528	11.384785	11.253936	11.475365	10.926171	12.205273	12.332809	11.201121	13.921423	0.091319701	0.14782999	0.028689869	0.199415148
ENSG00000182718	ANXA2	186.391023	189.193708	192.632951	105.490679	114.428687	107.934861	211.451249	208.753514	208.010472	3.08E-46	-0.69490848	0.001124206	0.199436873
ENSG00000128609	NDUFA5	27.199085	31.041531	30.093025	26.063032	28.592193	23.860627	27.91003	28.298797	33.297304	0.70040515	0.048806232	0.046678861	0.199482553
ENSG00000198380	GFPT1	7.586527	7.958247	8.526306	9.904585	9.772816	9.626022	8.340178	8.097792	9.51252	1.09E-11	0.434676582	0.013631208	0.199569211
ENSG00000235173	HGH1	15.02916	14.746945	12.799813	12.194386	12.050079	13.247661	14.193484	14.481264	17.262091	0.786785017	-0.035572392	0.053153819	0.199587465
ENSG00000204673	AKT1S1	30.036606	30.08057	29.690254	28.190355	32.744438	28.946317	34.68774	35.678173	36.333727	0.044664924	0.136796701	0.005714477	0.199595589
ENSG00000100554	ATP6V1D	25.069492	22.52457	24.812888	18.320091	17.098585	17.657165	25.516842	25.31176	23.463366	0.003899517	-0.253821429	0.036840322	0.199676352
ENSG00000106537	TSPAN13	64.343811	74.177391	77.997498	46.423607	47.140545	47.248608	78.140869	72.831062	82.405869	2.17E-11	-0.472523583	0.013653686	0.199728442
ENSG00000161638	ITGA5	6.92457	6.771776	6.733711	4.634937	5.227051	5.276792	6.853029	7.384544	7.766973	0.003521748	-0.283105775	0.031329923	0.199737329
ENSG00000159685	CHCHD6	9.442376	10.627815	9.953666	5.686764	5.831706	6.639703	11.045204	11.555204	8.681696	0.000155748	-0.559843716	0.218477841	0.199902403
ENSG00000163041	H3F3A	320.358862	383.450684	346.331754	298.200113	325.238568	345.299058	383.229954	387.944393	379.746617	0.95418762	0.005147085	0.001885769	0.200039104
ENSG00000173141	MRPL57	10.766471	9.247026	8.690391	8.741848	8.301242	9.299326	10.065534	10.929651	9.91188	0.894203329	0.022449735	0.10548289	0.200174067
ENSG00000137824	RMDN3	10.902452	11.513037	10.455413	15.499483	15.329505	16.574759	11.508109	10.654657	12.667879	6.68E-18	0.691518872	0.048791433	0.200228491
ENSG00000171490	RSL1D1	42.241518	43.818091	45.737945	44.883159	40.439	41.174322	50.435472	46.364879	47.458407	0.365436707	0.062397749	0.002094913	0.200240028
ENSG00000136003	ISCU	37.307261	34.757075	35.778668	35.149377	31.723713	33.46506	36.460017	39.533047	39.260446	0.818902102	0.028105011	0.035830899	0.200271246
ENSG00000113648	H2AFY	99.747765	102.736546	103.823273	81.81859	77.437586	87.34407	107.228297	102.29592	110.911852	0.087057191	-0.102190127	0.000549847	0.200298947
ENSG00000137941	TTLL7	0.636627	0.724732	0.857645	0.609951	0.670254	0.699607	0.705693	0.908393	0.918485	0.695799929	-0.098428725	0.316894991	0.200603472
NewGene_5135	--	1.646109	1.795779	1.337632	1.848948	1.758565	3.188367	1.933114	1.441779	1.733883	0.058867235	0.669891333	0.582055611	0.200644154
ENSG00000172878	METAP1D	3.944166	3.986838	3.641932	2.866616	2.611603	2.553468	4.107664	3.677055	4.726398	0.00323629	-0.380432941	0.12979991	0.200716709
ENSG00000135744	AGT	468.628296	401.458771	336.132355	628.597534	652.714478	642.123291	417.122955	446.42572	435.615143	6.06E-08	0.82065532	0.050868815	0.200928911
ENSG00000079950	STX7	4.594072	4.554221	5.191774	4.101264	3.983268	3.615494	5.110999	5.071098	5.458513	0.184538693	-0.122530059	0.017695507	0.200967333
ENSG00000146540	C7orf50	37.159537	33.905497	35.826757	29.053083	29.30895	31.599696	36.327678	40.426518	35.461172	0.077564084	-0.154806444	0.02449232	0.200994574
ENSG00000146122	DAAM2	0.25341	0.257705	0.303539	0.153581	0.176455	0.192332	0.273768	0.207378	0.407413	0.203095587	-0.488311528	0.591786097	0.201076036
ENSG00000114784	EIF1B	35.411396	34.234093	35.723751	30.826954	29.918987	30.052956	37.778305	36.57317	39.277546	0.503366953	-0.067539588	0.015754818	0.201130425
ENSG00000008130	NADK	34.080395	34.226208	34.327286	33.519071	34.371376	36.144581	37.618168	35.703123	42.257315	0.01665657	0.152441994	0.004203275	0.201167216
ENSG00000159840	ZYX	56.104694	56.183084	61.225441	42.551491	43.153938	43.649266	55.769053	59.313861	66.053221	0.0000221	-0.268475026	0.009639105	0.201423262
ENSG00000104324	CPQ	14.454068	14.233304	12.650126	16.024668	17.593914	17.274921	15.044578	14.557162	14.954781	0.00000734	0.447717647	0.042514538	0.201490215
ENSG00000135916	ITM2C	45.692187	43.509508	39.268505	33.711856	37.753145	36.238461	44.609657	48.093835	46.164506	0.19838059	-0.114635385	0.017130544	0.20164025
ENSG00000168071	CCDC88B	1.893454	1.264216	1.259889	1.258038	1.01125	0.934022	1.91418	1.439477	1.628388	0.067421027	-0.45063341	0.386790427	0.201709413
ENSG00000204843	DCTN1	48.511278	46.405351	45.014098	41.003433	40.333862	41.914898	48.03489	48.925758	53.198842	0.729607621	-0.0273765	0.002081225	0.20208703
ENSG00000160213	CSTB	34.477085	28.546148	26.604078	30.163088	30.13042	29.671531	31.345203	32.145861	33.077649	0.139773611	0.151934225	0.044369647	0.202194407
ENSG00000007047	MARK4	28.803972	29.293918	29.645031	19.480854	21.835855	21.360742	29.806861	30.497201	32.33828	0.000000685	-0.299060038	0.00235004	0.202239268
ENSG00000127720	METTL25	4.629179	3.903858	3.739751	3.169635	2.915532	3.476668	4.521472	4.21904	4.324886	0.297029868	-0.193603168	0.203232585	0.20227128
ENSG00000116819	TFAP2E	0.529404	0.415633	0.462688	0.318699	0.321282	0.36733	0.419813	0.454564	0.656797	--	--	0.653442987	0.202430376
ENSG00000116005	PCYOX1	18.311115	18.098663	17.975707	18.603639	20.235395	19.225433	19.724356	18.978843	19.992906	0.000160139	0.241405524	0.001442564	0.20257441
ENSG00000119383	PTPA	54.719606	54.558462	53.452276	48.322275	57.310801	61.106967	57.790175	61.588669	55.603781	0.019455034	0.149966706	0.003709961	0.202578862
ENSG00000103495	MAZ	181.006539	157.392342	158.512351	148.768858	159.076007	160.600224	170.895281	169.593359	172.060335	0.159124174	0.101728482	0.003262288	0.202621458
ENSG00000172531	PPP1CA	226.644784	221.733126	206.648127	183.951735	184.848415	189.288929	219.366472	234.80243	252.687692	0.223037764	-0.08155544	0.003920008	0.202710448
ENSG00000117360	PRPF3	23.330528	22.988232	24.472401	23.124561	20.789602	19.980545	25.245979	25.328882	25.807909	0.988555396	-0.001751797	0.007073778	0.202846163
ENSG00000105393	BABAM1	48.303389	47.98127	44.065683	37.391218	35.910191	37.147953	48.824809	50.079862	50.833449	0.000314171	-0.25231348	0.007067447	0.20292605
ENSG00000166912	MTMR10	4.138241	4.35825	4.467055	4.929136	4.910954	4.007233	5.05104	4.206564	5.015539	0.096076724	0.199311185	0.050074783	0.202947521
ENSG00000135686	KLHL36	3.147439	3.199895	3.187187	2.909579	2.804427	2.884519	3.417697	3.202801	3.576163	0.960237998	0.012676074	0.2199547	0.203000805
ENSG00000154269	ENPP3	2.668142	2.424304	2.159162	1.148865	1.318849	1.031889	2.700633	2.824721	2.215708	0.00000018	-0.923161828	0.268365518	0.203089623
ENSG00000144401	METTL21A	8.993484	11.065527	9.603176	8.187903	6.822739	7.904695	9.901424	11.989018	10.951649	0.412938387	-0.107059405	0.071424291	0.20337635
ENSG00000173210	ABLIM3	3.089295	2.671767	2.981643	1.380426	1.455929	1.514955	2.921501	3.124252	3.376857	4.47E-10	-0.841113503	0.138636427	0.203380458
ENSG00000102172	SMS	58.820199	64.760809	68.830973	52.917845	50.021735	53.28094	70.25476	65.223861	73.111691	0.036029176	-0.15772316	0.008014468	0.203399215
ENSG00000188493	C19orf54	10.801226	10.579442	11.166182	9.698107	9.615883	10.271171	11.720685	10.416831	13.364153	0.93662433	0.011544515	0.058604618	0.203413938
ENSG00000105290	APLP1	0.661786	0.416277	0.552182	0.222469	0.222383	0.286331	0.298937	0.600491	0.77261	0.053532802	-0.901765292	0.661959846	0.203473347
ENSG00000198342	ZNF442	0.190705	0.220533	0.156482	0.38426	0.188298	0.120407	0.377246	0.421002	0.114076	0.93666192	-0.048550781	0.670514411	0.203656448
ENSG00000134970	TMED7	28.116266	29.771366	29.625134	40.068668	39.169086	39.812756	31.647472	30.232048	32.652428	1.59E-30	0.591666943	0.001479989	0.203663202
ENSG00000099991	CABIN1	7.77282	7.221454	6.26703	6.661001	6.081451	6.137981	7.656996	6.814195	8.494453	0.959048469	-0.007695266	0.045901005	0.203702974
ENSG00000148584	A1CF	14.959533	14.181942	14.175607	18.589719	15.263109	14.541035	15.809953	14.770744	19.618649	0.0000016	0.269706462	0.001671388	0.203744087
ENSG00000225921	NOL7	96.851398	99.288622	109.25956	102.879177	101.52482	107.505981	109.899002	113.255201	105.338143	0.009610978	0.178202501	0.011586934	0.203787364
ENSG00000110697	PITPNM1	21.564333	20.64584	18.09946	15.695764	16.571958	16.311323	20.021156	19.896928	25.782418	0.041316076	-0.173718781	0.044349226	0.203813383
ENSG00000205155	PSENEN	17.695287	18.495295	17.444815	20.311137	19.296217	21.313462	18.807292	19.950579	20.806868	0.000483261	0.318841285	0.051226356	0.203899811
ENSG00000164902	PHAX	8.975504	8.52845	8.318878	8.029391	7.8353	8.302294	9.399793	8.449775	10.095681	0.628562029	0.05176475	0.021777775	0.203905353
ENSG00000184163	C1QTNF12	1.198573	0.990715	0.624246	0.625412	0.741994	1.024794	1.015147	0.946409	1.080806	0.923985945	-0.066114522	0.668763689	0.203913049
ENSG00000065717	TLE2	6.615507	5.518236	4.343032	4.942619	4.6686	3.611818	5.825175	5.218318	6.743368	0.434882476	-0.152608987	0.211444846	0.204009476
ENSG00000185885	IFITM1	5.185682	5.111011	5.891014	40.433086	37.561986	41.597179	5.511329	6.596865	5.292704	2.37E-177	3.026466311	0.352458587	0.204115939
ENSG00000136877	FPGS	18.160133	17.795903	16.898367	14.965052	15.408824	16.88518	19.854288	18.709058	18.784906	0.862905334	-0.021818674	0.015195426	0.204160015
ENSG00000253368	TRNP1	68.280607	58.487039	58.628243	36.711934	36.691119	38.876071	62.499576	65.998524	72.33621	3.67E-16	-0.57573344	0.014206691	0.204181124
ENSG00000214114	MYCBP	6.857813	7.895638	8.730358	6.647654	5.866998	6.239849	8.652057	8.587729	7.85451	0.194669345	-0.166295628	0.090279361	0.204199988
ENSG00000005175	RPAP3	10.273823	9.840564	10.349819	10.020147	9.535677	9.549748	13.869276	13.000952	11.864224	0.986348802	-0.002324887	0.035678534	0.204219435
ENSG00000170004	CHD3	9.758106	9.35172	8.324491	10.540462	9.839708	9.606919	9.668492	9.279413	10.983758	0.00043548	0.273349559	0.020127503	0.204277257
ENSG00000173473	SMARCC1	32.089698	33.47036	32.848625	28.056536	26.593372	28.57262	35.830872	33.375835	37.20063	0.114997195	-0.09439193	0.000466312	0.204381796
ENSG00000155438	NIFK	27.205827	28.298603	30.065022	27.104024	23.926212	26.20597	30.994712	28.22891	32.856178	0.96066024	0.00644127	0.014115316	0.204479315

Gene ID	Gene name	Ctrl-1 FPKM	Ctrl-2 FPKM	Ctrl-3 FPKM	IFNγ-1 FPKM	IFNγ-2 FPKM	IFNγ-3 FPKM	Sora-1 FPKM	Sora-2 FPKM	Sora-3 FPKM	Ctrl vs IFNγ DESeq2 FDR	Ctrl vs IFNγ DESeq2 log2FC	Ctrl vs Sora DESeq2 FDR	Ctrl vs Sora DESeq2 log2FC
ENSG00000119411	BSPRY	4.378852	4.292395	3.96485	2.870989	2.917816	2.533874	4.756364	4.525899	4.373918	0.002227451	-0.45729736	0.157974837	0.204486324
ENSG00000174748	RPL15	471.77831	463.871425	436.256838	331.86949	366.368232	373.391212	554.025249	461.600004	438.333493	0.006557102	-0.156370706	0.003173743	0.204543704
ENSG00000169062	UPF3A	37.998401	36.620397	41.065982	36.699778	36.088228	33.682819	41.56911	39.640219	44.748343	0.798398664	0.024776237	0.004817715	0.204596276
ENSG00000105355	PLIN3	67.048147	69.352425	51.732962	55.203247	54.325294	62.626014	68.748002	61.163389	78.229879	0.310173129	0.083916921	0.004231834	0.20462934
ENSG00000159346	ADIPOR1	52.911358	50.954887	52.723131	44.284283	45.091845	44.966315	55.421686	57.033756	56.046421	0.202917913	-0.08918625	0.002343418	0.204676108
ENSG00000169288	MRPL1	11.497966	11.630685	11.03761	9.553068	9.685625	9.152928	12.788416	12.358083	11.749273	0.349173674	-0.120631624	0.0752412	0.204686568
ENSG00000074219	TEAD2	33.379865	32.735321	31.185387	25.097921	27.32226	25.166675	35.122496	36.023936	35.148924	0.005067685	-0.213954951	0.013977541	0.20475865
ENSG00000115275	MOGS	25.224949	25.350043	22.3021	21.848531	22.991952	22.061912	24.512137	25.131033	30.720767	0.89670426	-0.013948198	0.013009473	0.204791902
ENSG00000049541	RFC2	43.091745	37.407596	37.782757	33.941037	37.040518	32.47182	43.226736	40.801198	41.754882	0.736587455	-0.034054446	0.007160798	0.20479798
ENSG00000174010	KLHL15	1.139899	1.468309	1.531899	1.440926	1.48504	1.306018	1.28019	1.440765	1.757448	0.336143224	0.178944769	0.261123423	0.204838447
ENSG00000111325	OGFOD2	9.700778	9.586855	9.36723	9.683361	9.093171	8.288239	10.442922	9.234107	11.255953	0.590833768	0.079687323	0.101727715	0.204963196
ENSG00000163001	CFAP36	4.460631	4.941191	4.609642	3.106774	2.772783	2.812644	5.08415	4.645157	4.673718	0.002440781	-0.481432432	0.180329276	0.204990104
ENSG00000111647	UHRF1BP1L	12.155769	12.295495	12.643162	12.709039	13.615786	13.079977	14.107558	13.437009	14.085085	0.01012175	0.191170154	0.005151468	0.205188817
ENSG00000171234	UGT2B7	11.324101	10.808064	9.322604	9.567718	10.981559	10.676097	11.545966	11.026757	11.46425	0.373675704	0.135280681	0.126422431	0.205216474
ENSG00000106077	ABHD11	8.186047	7.945989	9.824326	6.713086	5.836476	6.010244	10.385911	8.524692	9.654377	0.03807677	-0.307336013	0.153759845	0.205381387
ENSG00000215712	TMEM242	4.042441	3.929158	4.852964	3.613993	3.471889	3.358105	4.718693	5.050295	4.297463	0.287717568	-0.153005852	0.135970721	0.205461983
ENSG00000067992	PDK3	0.559201	0.378818	0.290588	0.762278	0.454752	0.559816	0.537779	0.550191	0.756224	0.79692611	0.134221337	0.635548099	0.20560477
ENSG00000104412	EMC2	18.35109	18.602655	21.455717	21.936966	20.367079	20.575904	21.074961	21.00487	20.889695	0.011063813	0.258538406	0.056250969	0.205613899
ENSG00000176681	LRRC37A	2.125119	2.404129	2.346339	2.281984	1.742186	2.3265	3.599585	2.175274	2.330171	0.758133675	-0.058961404	0.176388068	0.205653944
ENSG00000179091	CYC1	93.755875	89.692764	85.624313	75.748482	80.71991	84.641167	89.901352	97.07415	104.129349	0.927622951	-0.010083244	0.006416608	0.205657645
ENSG00000080822	CLDND1	10.367663	11.459273	9.682053	8.274145	8.729212	8.1027	11.957278	12.293951	10.774686	0.052743632	-0.20185552	0.053116164	0.205762934
ENSG00000219200	RNASEK	90.274964	83.829185	77.169796	74.257841	74.164394	81.632184	97.811706	103.8862	89.483425	0.901719606	-0.014435488	0.029372904	0.205928516
ENSG00000169567	HINT1	151.60848	146.567376	137.426418	142.699349	155.53723	154.199078	154.467131	158.796446	154.36325	0.000951359	0.225072073	0.010601891	0.205939244
ENSG00000168393	DTYMK	46.773818	45.440337	43.139184	33.745242	31.603992	36.237482	47.596082	48.613134	48.54519	0.000949071	-0.264907908	0.008521297	0.206025
ENSG00000111252	SH2B3	4.840126	4.965991	5.299438	6.753054	6.856569	6.305495	5.169993	5.103743	5.891014	1.61E-11	0.54755996	0.034011379	0.206076175
ENSG00000160310	PRMT2	31.090664	29.1655	29.634338	22.971513	23.778338	25.078357	28.098111	30.808501	35.924602	0.031663799	-0.170955031	0.014169197	0.206253279
ENSG00000064666	CNN2	130.742351	129.647321	130.96649	87.631521	82.544085	89.218996	140.355332	133.333577	149.769606	4.05E-21	-0.45150607	0.000291366	0.206314447
ENSG00000109519	GRPEL1	17.247574	19.80588	19.247652	18.766993	20.560234	20.150974	18.813831	21.68951	20.312056	0.006694415	0.228055593	0.032064775	0.206387421
ENSG00000159352	PSMD4	143.764366	146.588512	127.528387	116.14829	112.354032	113.8583	149.320443	153.624599	148.01788	0.040372713	-0.144232592	0.008197561	0.206489828
ENSG00000023734	STRAP	96.073973	106.432655	113.456455	95.52335	88.734146	89.481949	114.832769	104.904392	122.855064	0.389094949	-0.070387419	0.007067447	0.2066729
ENSG00000009830	POMT2	4.655138	4.341899	4.392846	3.290481	3.284063	3.183903	4.994782	4.73545	5.646855	0.001296128	-0.348437623	0.064410212	0.206748321
ENSG00000119771	KLHL29	2.057898	2.201442	2.442836	1.434036	1.585393	1.561791	2.017378	2.160886	3.095608	0.007559665	-0.399418732	0.260913586	0.206797881
ENSG00000169679	BUB1	24.971345	28.524619	26.670358	17.380579	15.454598	16.405681	29.595537	28.058634	29.022727	5.04E-16	-0.55461059	0.005654229	0.207065122
ENSG00000147872	PLIN2	344.652776	318.353272	304.229238	314.547878	334.748353	318.890392	339.477084	354.045909	354.153056	0.021313448	0.150905969	0.002343067	0.20719568
ENSG00000115414	FN1	214.029386	180.225592	184.499117	174.792634	178.801338	174.063489	197.130663	203.482384	225.218639	0.955226678	0.005962606	0.00657392	0.207237077
ENSG00000092108	SCFD1	24.950176	24.751457	25.880504	25.826767	24.65235	25.394467	27.799626	28.189754	29.177191	0.009916517	0.194837301	0.007599883	0.207420846
ENSG00000215021	PHB2	217.484042	189.576363	196.018265	182.87031	190.68249	195.396036	218.133563	218.032915	240.070874	0.423642727	0.056629823	0.001459609	0.207438181
ENSG00000117122	MFAP2	53.421055	46.197766	46.118061	34.309149	32.540942	33.248674	51.662844	53.544632	52.946795	0.000000457	-0.400102733	0.01708732	0.207510437
ENSG00000145216	FIP1L1	36.558998	34.729674	37.070615	35.383872	31.248766	33.017709	41.851583	35.971804	42.089812	0.968109572	0.004357648	0.00493062	0.207651717
ENSG00000177707	NECTIN3	15.850563	16.357383	17.098214	12.980172	15.204171	13.772129	16.215424	16.249486	19.209281	0.439366612	-0.07991394	0.013198906	0.207658702
ENSG00000165972	CCDC38	0.53464	0.671134	0.663951	0.621068	0.365978	0.316199	0.61308	0.638774	0.754406	0.209029843	-0.514966645	0.556713849	0.207785372
ENSG00000204262	COL5A2	37.378246	39.761539	37.65081	33.220879	31.010731	31.037239	41.645908	39.520935	43.269222	0.032185219	-0.121937224	0.000329141	0.207796921
ENSG00000164815	ORC5	10.451902	11.525258	11.153307	11.636548	9.563438	10.992205	12.02811	11.944034	12.044666	0.42054311	0.101907042	0.043223727	0.207879837
ENSG00000153113	CAST	159.331837	153.797624	168.82659	251.554553	252.829007	254.223794	164.947037	155.168407	150.046562	1.61E-49	0.832746242	0.00234657	0.20795017
ENSG00000134716	CYP2J2	5.165492	5.155346	4.889052	4.865715	4.742796	5.238949	5.625085	5.746318	5.013707	0.576972113	0.096297489	0.167081661	0.207977037
ENSG00000142864	SERBP1	102.118327	97.80031	100.885546	100.970013	92.21474	93.355277	114.773794	113.867914	118.166745	0.399641434	0.052908429	0.000350637	0.20805015
ENSG00000125352	RNF113A	4.550623	4.328944	4.461334	5.654424	5.144708	5.321375	4.961719	5.135456	4.327549	0.009101443	0.418392684	0.265159732	0.208094758
ENSG00000140474	ULK3	28.196041	27.034347	27.795667	21.843277	18.974957	22.608843	28.674002	29.779542	30.920292	0.001759926	-0.238807866	0.003306365	0.208158989
ENSG00000091262	ABCC6	24.331691	24.133653	21.408351	29.940454	29.738238	29.831074	21.129143	19.751312	19.200835	3.93E-13	0.473370893	0.008615194	0.2082467
ENSG00000135486	HNRNPA1	495.010903	451.182183	453.944145	391.132532	354.364113	383.542181	488.274568	501.24061	504.678709	0.27844951	-0.05866203	0.000661443	0.2084203
ENSG00000156804	FBXO32	0.330153	0.204589	0.219548	0.09398	0.173247	0.164717	0.201748	0.217319	0.281573	0.357476058	-0.414511009	0.572662522	0.20842369
ENSG00000161204	ABCF3	17.254614	16.63265	16.341342	16.785718	15.475791	17.000077	17.727867	18.082489	18.73818	0.170084088	0.118811532	0.009117753	0.208623281
ENSG00000077942	FBLN1	110.704841	100.35063	108.010606	77.063973	75.307192	79.036775	112.495569	111.514393	128.692509	1.42E-08	-0.305809999	0.002487646	0.20875251
ENSG00000124214	STAU1	79.122542	81.463468	84.369876	99.895821	98.944993	100.627876	88.858569	84.909239	94.024693	5.27E-21	0.440390038	0.000350481	0.208927942
NewGene 4661	--	1.137803	1.14523	0.749095	0.970126	1.125595	1.245819	1.116265	1.038219	1.134251	0.271132154	0.291344997	0.39726365	0.208936582
ENSG00000110274	CEP164	3.002348	3.115978	3.054701	3.17184	2.297111	2.493014	3.342362	3.080879	3.573922	0.737831657	-0.055616005	0.068696059	0.20904434
ENSG00000111707	SUDS3	12.31607	12.779765	12.513275	9.739922	10.578376	9.550334	12.988784	13.200771	14.598589	0.01798043	-0.184809744	0.004646465	0.209144568
ENSG00000102981	PARD6A	0.675944	1.271273	0.811252	0.838278	0.805797	0.72074	0.9689	0.908063	1.02494	0.968309314	-0.026235004	0.640094108	0.209217789
ENSG00000064490	RFXANK	33.126672	31.588729	29.719954	30.282533	30.388863	31.110004	34.870056	34.035357	32.869552	0.289784289	0.101717938	0.017011376	0.209243487
NewGene 1691	--	0.824422	0.493759	0.57137	0.966268	0.535289	0.550486	0.799337	0.844815	0.757009	--	--	0.67926421	0.209249386
ENSG00000156502	SUPV3L1	14.719457	15.304267	16.003998	14.914929	13.411759	13.38789	15.91452	16.438387	17.555494	0.972379201	0.004847741	0.014449846	0.209282416
ENSG00000100461	RBM23	0.34547	0.238713	0.454543	0.294306	0.270958	0.221797	1.103841	0.200246	0.320524	0.568285727	-0.238211319	0.536789531	0.209372277
ENSG00000132481	TRIM47	8.987025	7.901359	8.215684	7.744946	7.234598	8.104123	10.377108	7.961572	9.449946	0.552246723	0.085971186	0.114303831	0.209408653
ENSG00000197903	HIST1H2BK	64.915321	56.642956	59.497337	53.283798	56.551239	54.113029	56.085041	70.107704	70.068993	0.98194363	0.003775733	0.089699929	0.209410421
ENSG00000109083	IFT20	10.264144	11.547142	10.283277	9.718325	8.58312	8.796122	10.050942	11.30786	12.177798	0.837334154	-0.041312751	0.161159568	0.209414275
ENSG00000167978	SRRM2	53.639614	50.226592	49.386374	54.324679	46.881038	49.453942	53.14541	51.252768	61.98378	0.043773754	0.132067123	0.002563097	0.209424572
ENSG00000160447	PKN3	9.337742	9.188197	9.958291	7.049982	8.274646	8.716124	9.94829	10.203563	10.743336	0.454092152	-0.095072317	0.019208377	0.209547551
ENSG00000099968	BCL2L13	12.599286	13.848403	13.452911	12.675723	10.770054	11.715718	12.62367	13.898534	14.64533	0.54542798	-0.056638746	0.010547133	0.209659872
ENSG00000055163	CYFIP2	3.709733	2.995483	4.477881	2.594142	1.909876	2.830494	5.12392	4.859872	4.747775	0.000000462	-0.562289117	0.073891923	0.209660016
ENSG00000061794	MRPS35	50.366357	56.675084	57.020833	48.79888	47.183386	46.798427	61.007508	57.114744	60.87622	0.547587831	-0.052485658	0.004535884	0.209685806
ENSG00000167747	C19orf48	58.231653	57.836499	55.487876	50.522246	47.928336	52.394155	60.335694	60.008669	65.795018	0.810146693	-0.022016485	0.001899665	0.209694618
ENSG00000152939	MARVELD2	9.874097	9.50325	8.89761	8.397252	11.279961	10.03482	8.975971	9.964941	10.402795	0.000017	0.373862473	0.021181242	0.209746254
ENSG00000107077	KDM4C	8.210512	7.810257	7.66257	7.77902	7.41619	7.412026	9.009141	8.124857	9.347117	0.680199287	0.045957192	0.019325398	0.20977736
ENSG00000110169	HPX	24.666019	23.193695	20.486656	25.930601	27.389519	24.813709	25.854982	24.342546	23.931089	0.00016841	0.339362887	0.035417273	0.209780792

Gene ID	Gene name	Ctrl-1 FPKM	Ctrl-2 FPKM	Ctrl-3 FPKM	IFNγ-1 FPKM	IFNγ-2 FPKM	IFNγ-3 FPKM	Sora-1 FPKM	Sora-2 FPKM	Sora-3 FPKM	Ctrl vs IFNγ DESeq2 FDR	Ctrl vs IFNγ DESeq2 log2FC	Ctrl vs Sora DESeq2 FDR	Ctrl vs Sora DESeq2 log2FC
ENSG00000111199	TRPV4	5.327718	5.839425	5.198052	2.712026	2.823228	2.932026	5.818936	5.797949	5.966663	6.83E-13	-0.800732629	0.057709531	0.209916909
ENSG00000170638	TRABD	15.038299	13.9957	13.932121	13.764417	12.014448	14.111618	15.273099	14.303734	17.208802	0.797804134	0.033283246	0.024083438	0.209953319
ENSG00000162542	TMCO4	3.176766	2.884216	2.963387	3.383185	2.980753	3.388593	3.037842	3.102396	3.680174	0.092428022	0.253398241	0.164995229	0.209972234
ENSG00000006453	BAIAP2L1	49.126247	49.212021	52.474232	39.551636	37.963802	40.289742	54.713326	51.53595	57.468781	0.000259313	-0.208910892	0.000718618	0.210072965
ENSG00000076826	CAMSAP3	25.582781	24.914094	24.455937	24.76592	23.834459	25.557194	26.693296	24.42647	30.186748	0.048476442	0.129357387	0.005218987	0.210094849
ENSG00000145349	CAMK2D	15.669674	15.667945	16.116794	14.585271	14.707286	14.863253	18.429837	17.452361	19.428249	0.994863922	-0.000755057	0.002759057	0.210564264
ENSG00000177045	SIX5	9.40421	7.834025	8.114781	8.161494	8.453425	9.741603	8.433695	8.678678	10.397026	0.029066604	0.246500214	0.060884037	0.210656778
ENSG00000163918	RFC4	67.992225	67.62629	68.83049	60.144651	54.656655	57.126368	73.557306	68.975105	74.252199	0.194043638	-0.103645107	0.003196377	0.210658882
ENSG00000183763	TRAIP	6.175007	5.346366	4.566138	4.1459	3.495469	4.007857	6.142241	5.617871	5.559958	0.040722672	-0.350938017	0.191713188	0.210807469
ENSG00000122390	NAA60	29.18439	27.642796	27.292069	29.734778	30.739528	31.632528	29.445208	29.457259	31.924301	0.00000352	0.294697046	0.002624325	0.210974645
ENSG00000140395	WDR61	24.751435	23.19501	22.706376	18.977888	18.629092	21.570965	26.441724	26.048357	28.731218	0.268049032	-0.123268912	0.024246224	0.210980498
ENSG00000134758	RNF138	10.562123	11.88648	12.733205	12.527047	11.343954	11.825851	13.217502	11.353015	13.622688	0.07181558	0.173688543	0.035881619	0.211010558
ENSG00000243244	STON1	2.073959	1.753508	1.960673	0.810094	0.88964	0.978508	1.839855	2.166279	2.247709	1.49E-10	-0.956565202	0.155747298	0.211188787
ENSG00000167863	ATP5PD	112.610434	116.203352	114.831216	101.437176	102.540573	98.551599	125.861218	134.759518	111.18589	0.674017482	-0.037181571	0.030550057	0.21125486
ENSG00000065427	KARS	69.534651	67.167259	74.401453	90.611588	90.034729	87.477229	70.827287	80.117633	76.01067	4.39E-22	0.47355195	0.003816384	0.211261942
ENSG00000146143	PRIM2	15.558677	17.695881	17.633832	13.466127	12.317934	11.449725	18.195132	18.414248	18.739074	0.000967927	-0.306105356	0.017924658	0.211268178
ENSG00000101365	IDH3B	67.955134	62.253702	64.136879	52.475981	53.206759	52.82307	70.693581	71.775748	69.808845	0.027360386	-0.151125964	0.003983911	0.211308786
ENSG00000130724	CHMP2A	73.534091	76.054218	71.846056	70.829059	70.733405	70.42781	77.391755	84.545144	78.935423	0.336369961	0.074683116	0.011328848	0.211312242
ENSG00000182359	KBTBD3	0.567525	0.460922	0.732022	0.3715	0.628935	0.462508	0.56615	0.838972	0.512647	0.786387298	0.131919059	0.587057532	0.211329021
ENSG00000070214	SLC44A1	11.983642	12.497434	12.061487	15.364758	15.379184	15.215589	14.327199	12.032129	12.722025	1.15E-20	0.468800559	0.001380305	0.211385952
ENSG00000151929	BAG3	8.309319	7.37665	7.546951	6.577057	6.454069	7.017969	8.249784	8.000038	9.228946	0.615513379	-0.069903551	0.051746319	0.21140967
ENSG00000166145	SPINT1	54.886437	51.608334	52.863115	20.742064	20.902399	22.20433	56.463644	55.146281	69.381877	6.17E-89	-1.194523738	0.00718582	0.211460216
ENSG00000112249	ASCC3	10.813418	12.309492	12.741558	13.988108	13.803305	13.490022	13.035768	12.654354	13.596013	6.61E-08	0.331889065	0.002538763	0.211644768
ENSG00000156171	DRAM2	12.08679	11.575365	11.222888	9.136915	8.061406	8.281873	12.630871	12.676847	12.333499	0.00220548	-0.298933699	0.029404475	0.211658302
ENSG00000004864	SLC25A13	57.938604	59.211143	56.974645	52.105812	54.148939	53.053699	63.725914	61.949959	63.154751	0.804135004	0.019468494	0.000420617	0.211738487
ENSG00000147535	PLPP5	17.452108	16.564868	18.464477	18.22355	16.306841	19.6091	19.42611	17.659247	18.393269	0.079941909	0.177109423	0.021262223	0.21184505
ENSG00000078618	NRDC	42.504119	43.986122	43.318033	47.434122	48.998047	45.509294	47.235539	45.305922	48.316423	0.000000411	0.267344276	0.000252222	0.211851969
ENSG00000075643	MOCOS	2.608881	2.592033	2.675291	3.308124	3.571054	3.687675	2.711484	2.85838	2.984314	6.12E-09	0.572176936	0.060479654	0.211931339
NewGene_3408	--	2.50466	2.507487	2.968685	2.593489	2.020537	1.995866	2.981019	3.030863	3.160809	0.045702726	-0.297733206	0.120255866	0.211944733
ENSG00000138587	MNS1	0.963147	1.323792	1.444935	1.147663	0.41694	0.77758	1.45887	1.209926	1.38343	0.188794798	-0.524299875	0.472107337	0.211956412
ENSG00000127220	ABHD8	8.208001	8.693963	7.344433	7.355054	7.82805	7.849732	8.766757	8.242633	9.342833	0.633220559	0.072012406	0.070839926	0.212053128
ENSG00000100600	LGMN	17.32488	16.434091	16.825121	65.936068	73.47517	72.930112	17.643024	18.29365	19.084391	4.44E-251	2.173682301	0.014239475	0.212088076
ENSG00000187446	CHP1	26.956928	27.868154	27.603639	28.600194	27.039189	27.813501	31.758762	28.5429	29.06709	0.005628742	0.167311439	0.00387543	0.212118211
ENSG00000187954	CYHR1	15.403903	15.088501	13.642231	14.893458	13.515801	14.902706	15.252195	15.125098	17.453603	0.280099524	0.114874371	0.028094947	0.212182861
ENSG00000141456	PELP1	25.826095	26.201274	26.396767	24.753923	23.115441	24.861808	28.379112	26.690091	30.147306	0.612441225	0.040320519	0.001633038	0.212194326
ENSG00000008838	MED24	24.793118	25.654922	22.480851	22.409413	20.842326	20.417312	24.829576	25.256385	30.340183	0.242628287	-0.090287505	0.004893207	0.212199163
ENSG00000169826	CSGALNACT2	2.354135	2.391754	2.298355	2.33478	1.899924	2.25265	2.500106	2.503206	2.649348	0.902378968	0.026832263	0.144675837	0.212259566
ENSG00000186063	AIDA	13.531309	15.551497	15.441686	21.953268	19.636209	20.105192	16.809889	14.559892	17.058559	1.01E-16	0.617775457	0.020698076	0.212269075
ENSG00000116213	WRAP73	5.666494	6.490406	5.403317	4.545084	4.473952	4.528501	5.647016	6.381912	7.242806	0.164647845	-0.21991231	0.172275224	0.212315285
ENSG00000119977	TCTN3	12.424945	12.341981	11.556254	10.03181	9.759155	10.277437	13.753814	13.617253	12.838535	0.23493834	-0.121316395	0.018560997	0.212418107
ENSG00000119640	ACYP1	2.00348	2.617633	2.344804	1.717906	1.451348	1.483679	2.107612	2.836457	2.423614	0.367127877	-0.377753208	0.566023342	0.212543438
ENSG00000169221	TBC1D10B	17.16831	18.384734	16.887419	17.359042	17.490948	17.601088	17.855507	17.39938	21.634209	0.053531152	0.145878391	0.01664873	0.212568033
ENSG00000122778	KIAA1549	3.23451	3.995726	3.902689	2.706645	2.644483	2.448749	3.581169	3.519617	4.146565	2.63E-08	-0.457646367	0.022302391	0.212598631
ENSG00000065154	OAT	12.249089	11.737838	11.063969	9.915534	10.615213	9.973826	14.122375	12.125148	11.804562	0.680278173	-0.054525667	0.060479654	0.212616997
ENSG00000180881	CAPS2	0.432098	0.62113	0.671589	0.564801	0.658846	0.573095	0.868005	0.442216	0.69282	0.529401778	0.232407466	0.541080343	0.212683485
ENSG00000129535	NRL	0.545172	0.458689	0.672922	0.626169	0.614567	0.75729	0.470144	0.839473	0.649983	0.426973896	0.348906434	0.639481672	0.212747151
ENSG00000128524	ATP6V1F	71.082031	66.361716	62.145356	59.376269	64.488404	61.325097	75.464366	71.391951	69.908795	0.732426208	0.039503174	0.017531698	0.212769576
ENSG00000254986	DPP3	91.868526	89.343818	83.324429	75.886472	76.761016	82.052892	88.59239	94.551368	103.98882	0.934266959	0.007964891	0.002649211	0.21278231
ENSG00000130741	EIF2S3	53.206329	55.370248	55.170591	53.278006	49.357243	50.812919	60.007332	56.398395	61.734761	0.420971908	0.054146076	0.000359379	0.212851758
ENSG00000178338	ZNF354B	1.687201	1.548401	1.873443	2.034983	1.784562	1.808875	1.828232	1.800066	2.042477	0.225725226	0.254982374	0.292133955	0.213113766
ENSG00000102882	MAPK3	29.949028	27.906431	25.586082	41.203474	44.299551	40.865443	28.050178	29.944722	32.893391	3.9E-23	0.698189855	0.012083953	0.213305702
ENSG00000276547	PCDHGB5	9.037138	8.342454	7.098532	6.318241	5.679595	6.618952	8.450872	8.429198	9.136673	0.033570834	-0.242834473	0.04620785	0.213358623
ENSG00000166557	TMED3	44.764057	44.040186	44.562346	39.028691	37.678814	38.940462	46.83114	51.310854	54.108416	0.471397231	-0.056776473	0.004608652	0.213421859
ENSG00000119318	RAD23B	97.135486	98.235202	100.672835	112.256971	111.538804	111.208925	108.731692	107.704443	104.303293	6.65E-14	0.329219717	0.000631967	0.213527919
ENSG00000104695	PPP2CB	34.024573	33.891923	37.523773	26.147844	25.186602	25.605611	38.09166	36.807502	39.621015	0.0000101	-0.309377561	0.004471192	0.213545032
ENSG00000114107	CEP70	8.759266	9.609468	8.714282	8.329421	6.906114	7.299673	11.231698	10.048316	10.212989	0.331728434	-0.120925955	0.048795998	0.213590934
ENSG00000104147	OIP5	19.56891	21.705952	20.364795	17.932028	15.20381	14.936155	23.251031	19.712211	24.121936	0.044626991	-0.221219902	0.03927455	0.213680219
ENSG00000100353	EIF3D	105.816218	105.239489	98.21803	86.03603	83.757672	85.439731	113.033197	111.394693	116.915747	0.02302172	-0.130898441	0.000617227	0.213952045
ENSG00000155465	SLC7A7	1.395361	1.799262	1.570682	1.297879	0.81395	0.969204	1.761455	1.49266	1.660555	0.044626991	-0.537834528	0.37291899	0.213954753
ENSG00000141959	PFKL	32.232417	29.568514	24.549174	27.549931	25.683725	27.521785	31.509828	29.229313	33.268408	0.681847625	0.050771602	0.029148649	0.213972702
ENSG00000111711	GOLT1B	24.322431	27.503891	26.832778	25.812576	25.837253	26.878355	29.150368	25.547631	29.765691	0.047131496	0.151330637	0.007711969	0.213975581
ENSG00000186073	C15orf41	8.336443	9.279606	10.063312	7.184064	6.374399	6.436254	10.467171	9.942594	10.182875	0.00378436	-0.310732654	0.045063071	0.214085536
ENSG00000124380	SNRNP27	19.130457	20.591552	19.84803	20.659047	19.426367	19.689669	22.159983	20.509347	21.133372	0.250941747	0.108564673	0.01687955	0.214125855
ENSG00000121621	KIF18A	8.595268	11.391177	10.722622	10.773722	10.249639	9.994556	10.844162	11.246919	11.271653	0.162335998	0.162260535	0.056664308	0.21414714
ENSG00000076928	ARHGEF1	28.848751	28.008297	30.238256	19.652012	19.018113	19.438892	29.298292	29.834657	37.814914	4.78E-12	-0.409054131	0.013827197	0.214216811
NewGene_3743	--	1.161225	1.347586	1.086719	1.014307	1.116179	1.242353	1.36839	1.194153	1.34332	0.871600571	0.060572397	0.428495874	0.214284591
ENSG00000110888	CAPRIN2	6.072025	6.449244	7.791923	7.349425	8.25782	6.873503	6.441779	6.711719	7.637444	0.006294172	0.30497079	0.06267264	0.214304662
ENSG00000165458	INPPL1	6.195289	5.658509	5.49992	6.802745	6.753217	6.365635	6.059274	5.890918	7.088519	0.0000137	0.37569928	0.032250295	0.214311617
ENSG00000205981	DNAJC19	20.521126	22.571656	19.371364	19.67653	20.667864	22.808931	23.722113	23.668058	21.264055	0.169360394	0.148609227	0.048628441	0.214405269
ENSG00000185875	THNSL1	2.067315	2.363398	1.908753	2.100487	1.845227	1.741384	2.258179	2.24766	2.553761	0.88938211	-0.033403626	0.188222706	0.214523132
NewGene_3525	--	0.606438	0.323057	0.424793	0.513	0.455349	0.316914	0.311044	0.608153	0.557407	--	--	0.687510509	0.214543548
ENSG00000166165	CKB	77.778276	71.539006	64.959467	47.775271	48.771701	52.83333	78.062206	75.451111	78.662809	0.00000147	-0.34709093	0.003209532	0.214762036
ENSG00000113119	TMCO6	7.244904	7.601518	7.725482	7.09374	6.765066	7.546301	7.507986	8.032738	8.471286	0.812383489	0.040343566	0.085401949	0.21482816
ENSG00000160753	RUSC1	14.841933	12.972557	15.056625	15.026939	14.305052	13.630088	15.373329	15.310338	16.807749	0.11885191	0.164149082	0.036731769	0.214914662

Gene ID	Gene name	Ctrl-1 FPKM	Ctrl-2 FPKM	Ctrl-3 FPKM	IFNγ-1 FPKM	IFNγ-2 FPKM	IFNγ-3 FPKM	Sora-1 FPKM	Sora-2 FPKM	Sora-3 FPKM	Ctrl vs IFNγ DESeq2 FDR	Ctrl vs IFNγ DESeq2 log2FC	Ctrl vs Sora DESeq2 FDR	Ctrl vs Sora DESeq2 log2FC
ENSG00000065057	NTHL1	11.923306	12.663961	10.276433	10.551115	12.054241	10.359077	11.689273	11.911022	14.497217	0.758497248	0.05810853	0.130866334	0.215068315
ENSG00000164011	ZNF691	2.638752	2.645687	2.963599	3.16592	2.794808	3.18878	3.433958	2.823057	2.485614	0.786075536	0.073020801	0.322852323	0.215096295
ENSG00000059691	GATB	9.963395	10.83189	10.616974	9.598878	8.961458	9.709998	12.027446	10.838734	11.050204	0.790056551	-0.035184391	0.031034742	0.21515314
ENSG00000181544	FANCB	1.297985	1.398506	1.300577	1.401981	0.951706	0.947345	1.246668	1.550734	1.443699	0.837717023	-0.066918621	0.335899288	0.215179243
ENSG00000203995	ZYG11A	1.434664	1.460104	1.489365	1.637171	1.686363	1.783468	1.581435	1.586023	1.588363	0.015657469	0.384781129	0.203324315	0.215270522
ENSG00000149269	PAK1	6.256767	5.486247	6.223166	5.780778	5.131723	5.533105	6.715571	6.954461	6.263527	0.833264092	-0.03033736	0.047689257	0.215358427
ENSG00000163444	TMEM183A	40.077282	41.03894	39.496719	36.134708	37.600872	38.233253	43.4772	42.195683	45.701336	0.607539184	0.040644706	0.000412997	0.215504904
ENSG00000105519	CAPS	4.273139	4.348529	4.427983	2.283298	2.323475	2.383266	4.090725	4.575523	5.527471	7E-11	-0.747485121	0.104772999	0.215579958
ENSG00000164118	CEP44	2.06347	2.83919	2.692687	2.799134	2.455291	2.994288	2.975502	2.222847	3.239446	0.107744213	0.26242618	0.203965178	0.215656414
ENSG00000136238	RAC1	260.735306	277.890228	282.369053	243.008873	233.881696	240.893703	294.633503	287.696819	304.0262	0.783661849	-0.020691666	0.00028142	0.215665222
ENSG00000181222	POLR2A	18.193808	16.903002	15.489854	16.336061	15.71033	16.111521	17.745871	17.621199	19.805834	0.496435822	0.075222354	0.022522413	0.215668346
ENSG00000013374	NUB1	14.901393	15.292912	14.866821	51.1028	50.760116	52.820245	15.848079	15.399998	16.354373	1.1E-253	1.975094035	0.009572841	0.215718338
ENSG00000162929	KIAA1841	3.799394	5.240001	4.874914	4.771989	4.214699	3.896732	5.11648	4.791004	5.202163	0.961910939	-0.009480773	0.090142383	0.215728175
ENSG00000132383	RPA1	56.921108	54.914453	55.878922	43.779219	42.742287	44.970441	59.295212	59.384378	66.51129	0.000034	-0.21063966	0.00018474	0.215792229
ENSG00000182196	ARL6IP4	71.177036	57.342951	63.331661	64.558107	66.517043	68.814376	75.141543	73.52937	79.228901	0.631132258	0.051721446	0.003886037	0.215891396
ENSG00000165948	IFI27L1	5.356843	4.161992	4.152205	4.462091	3.956456	5.407539	4.350735	5.308779	5.403151	0.66999417	0.138770008	0.403488618	0.215990033
ENSG00000091436	MAP3K20	5.27251	5.56085	5.388399	5.237038	5.002023	4.911068	5.387654	5.530532	5.935968	0.566202306	0.067285633	0.026940977	0.215994242
ENSG00000151748	SAV1	2.482299	2.928631	2.584882	2.00991	2.213982	2.303457	2.472623	2.583747	3.680358	0.500822861	-0.133149027	0.240752482	0.216084255
ENSG00000176407	KCMF1	9.496295	9.833442	10.184334	8.43563	8.18465	8.333355	11.203395	10.240502	10.810512	0.303344509	-0.078240694	0.003023346	0.21610201
ENSG00000136938	ANP32B	121.838348	132.99707	136.326736	122.924004	116.270142	119.457825	150.756271	130.541138	145.054123	0.793123459	0.022253702	0.002825046	0.216173347
ENSG00000204576	PRR3	12.30148	12.334773	13.533059	15.13331	13.708633	14.406743	14.630493	12.890068	13.838719	0.000164183	0.36006736	0.032397626	0.21620425
ENSG00000132541	RIDA	17.654785	21.081825	22.345772	16.009894	15.449088	17.102016	21.361764	21.609879	23.161796	0.17413457	-0.175499015	0.058198924	0.216325187
ENSG00000006451	RALA	33.035536	32.972557	34.680143	30.568036	30.088009	29.256919	36.514505	35.386116	37.249028	0.902295008	-0.01130986	0.000849811	0.216357174
ENSG00000184897	H1FX	51.29525	48.440361	42.231857	45.784904	50.454834	52.876297	54.385078	50.867039	49.316662	0.018651985	0.218796259	0.020000115	0.216358888
ENSG00000197070	ARRDC1	10.574319	12.11157	10.131605	12.023948	12.010693	10.787977	12.796662	11.473671	12.255214	0.062525783	0.23182236	0.075696533	0.216408197
ENSG00000196781	TLE1	60.426195	58.397908	60.751923	51.518961	48.131962	50.79497	66.337977	58.605465	69.970575	0.322541716	-0.066253758	0.002085507	0.216539383
ENSG00000074276	CDHR2	0.602907	0.712397	0.582332	0.409221	0.556887	0.56927	0.950559	0.589599	0.742217	0.536805737	-0.21144126	0.424082188	0.216643183
NewGene_653	--	4.3253	3.994211	4.007994	4.032325	3.769195	3.439197	4.248545	4.677466	3.770698	0.977698944	0.006051196	0.149417312	0.216643253
ENSG00000185669	SNAI3	1.224547	1.278816	1.068014	1.100524	1.143232	0.93955	1.156625	1.26291	1.487152	0.947982103	-0.02929712	0.481506875	0.216652683
ENSG00000070761	CFAP20	18.094118	17.900877	17.224512	15.600462	15.70995	15.277896	19.203186	19.821859	18.905617	0.724056896	-0.044914199	0.030158942	0.216656631
ENSG00000143811	PYCR2	37.885411	36.190269	36.401119	30.419835	30.673722	32.040273	39.848633	39.064377	40.259761	0.215582261	-0.100548826	0.003061605	0.21674621
ENSG00000090061	CCNK	5.449522	6.244364	6.053991	6.379049	6.611996	5.684969	7.189028	5.476883	6.295333	0.010512086	0.295116989	0.100120327	0.216821993
ENSG00000267673	FDX2	16.414613	14.295925	14.475235	10.912584	9.100067	10.141982	14.765451	16.53738	17.89269	0.000577422	-0.442484954	0.088898134	0.216851983
ENSG00000114770	ABCC5	8.687407	7.569011	8.175469	5.527409	5.674846	5.129884	8.250145	8.921663	9.769796	0.000000512	-0.430109111	0.0174835	0.216947511
ENSG00000013375	PGM3	16.003213	17.509442	15.861791	21.106975	18.681362	20.605055	17.026088	18.081756	18.520912	2.7E-17	0.484086729	0.001297198	0.216963484
ENSG00000123178	SPRYD7	0.851679	0.844137	1.035973	0.505612	0.770023	0.916813	0.884003	0.98019	1.103524	0.694750703	-0.150579198	0.417862694	0.217020615
ENSG00000102174	PHEX	0.246922	0.201833	0.194229	0.131767	0.127422	0.137594	0.225165	0.188464	0.295176	0.217638033	-0.535194261	0.582359336	0.217061759
ENSG00000145919	BOD1	39.951613	40.581059	40.300883	30.472263	29.981144	31.812335	45.783361	44.911882	44.493054	0.000117998	-0.258358073	0.003102601	0.21718458
ENSG00000134461	ANKRD16	1.533179	1.812371	1.513441	1.946202	1.867315	1.443724	1.323656	1.643513	1.957361	0.66999417	0.132140013	0.385444968	0.217246757
ENSG00000181031	RPH3AL	6.59579	5.911444	5.959226	5.608796	5.121878	5.582388	6.58869	6.440623	7.325718	0.855383339	-0.032539462	0.075039533	0.217373499
ENSG00000225663	MCRIP1	42.640653	41.622768	34.884933	32.507674	27.253588	33.238014	41.482745	42.0892	43.512304	0.172441132	-0.133162784	0.012775162	0.217387911
ENSG00000134690	CDCA8	18.768763	18.238314	18.825675	15.495633	13.583017	15.773758	20.726551	20.919515	20.53158	0.035725566	-0.184171373	0.006903724	0.217402603
NewGene_6594	--	4.036663	3.827744	4.89493	4.057688	3.854428	3.40456	4.886197	4.327501	4.584432	0.95551367	-0.014257965	0.185854214	0.217420352
ENSG00000075624	ACTB	2970.325905	3014.033913	3254.341995	1754.891496	1654.047772	2046.949868	3225.57771	3393.311897	3488.524492	2.13E-27	-0.446091199	0.0000319	0.21757513
NewGene_2693	--	0.690927	0.820215	0.606729	0.451309	0.730933	0.702905	0.76055	0.780732	0.760845	0.972449296	-0.01967968	0.528241767	0.217634847
ENSG00000137054	POLR1E	11.17158	12.245624	11.042774	10.089601	9.472183	10.197164	12.499417	12.793233	12.229554	0.710595378	-0.050996149	0.04205952	0.217648313
ENSG00000166710	B2M	301.064843	299.598938	277.399959	1844.255814	1872.908592	1947.088659	319.127578	333.900171	291.494327	0	2.809609731	0.000344057	0.217653038
ENSG00000145029	NICN1	0.720586	0.823041	0.956014	0.417632	0.585827	0.799309	0.901831	0.377319	1.448675	0.577514052	-0.232918979	0.626594138	0.217737386
ENSG00000124783	SSR1	34.090589	35.087312	35.601774	49.644996	51.145933	49.141844	37.64272	35.411084	41.890162	4.46E-46	0.663183033	0.000455247	0.217769952
ENSG00000178605	GTPBP6	16.133835	13.857687	13.980979	12.438511	11.117846	12.562685	14.450033	15.366402	18.24177	0.239006324	-0.137163565	0.051962312	0.217793092
ENSG00000198900	TOP1	49.045776	48.338787	53.124424	52.615913	49.046364	48.599167	52.930401	53.270275	58.025673	0.02235461	0.144514337	0.000964554	0.217832616
ENSG00000139182	CLSTN3	26.399537	23.812165	20.635889	36.090118	34.582296	35.033655	25.746479	24.711154	26.677395	1.38E-21	0.717229404	0.015358207	0.217926686
ENSG00000014216	CAPN1	51.31004	47.698215	45.8543	53.555199	55.190283	56.985178	50.131234	50.417985	57.310824	8.92E-08	0.336009054	0.003168244	0.218028993
ENSG00000204619	PPP1R11	54.579836	52.152437	53.008971	48.111646	49.102262	47.846225	57.895871	58.76349	57.294501	0.928337486	0.008769062	0.002035797	0.218029834
ENSG00000112977	DAP	72.333252	69.963631	70.802551	70.637085	72.191666	73.995232	75.795181	74.991264	81.786911	0.002310716	0.172661633	0.000239399	0.218052668
NewGene_2137	--	5.668487	7.312032	6.821844	5.177669	6.129412	5.913365	7.176792	7.643974	6.274204	0.933602721	-0.024580637	0.286492047	0.218163398
ENSG00000057663	ATG5	14.75827	14.005329	14.409645	12.90956	12.912775	11.476325	15.822187	14.95546	16.629307	0.404122671	-0.082555955	0.004600076	0.218205463
ENSG00000071626	DAZAP1	85.252401	88.088811	95.860792	81.401459	84.270262	81.552361	98.074162	95.496697	99.073271	0.688630564	0.03408919	0.000995966	0.218293616
ENSG00000143436	MRPL9	79.403315	80.794898	78.319189	68.010845	64.108531	66.626605	90.616605	87.648201	85.725611	0.045324782	-0.132091813	0.001418686	0.218353128
ENSG00000167283	ATP5MG	51.929035	56.868112	54.643077	47.146001	48.588064	48.535894	60.749994	65.539436	52.065772	0.740379082	-0.030302334	0.037335886	0.21842131
ENSG00000059728	MXD1	4.234194	4.750187	5.365704	6.664141	7.151583	6.738666	5.607033	4.971959	5.216372	1.12E-12	0.664078712	0.050599044	0.218531683
ENSG00000007255	TRAPPC6A	13.225544	10.901237	9.276844	7.522772	9.010199	8.496848	12.309781	12.754211	11.240082	0.192895144	-0.250500118	0.207659738	0.218583987
ENSG00000197746	PSAP	264.983456	252.721427	246.631909	240.667234	240.075222	249.517473	272.356269	273.078872	288.754807	0.165495265	0.081563303	0.000119885	0.218584013
ENSG00000105483	CARD8	5.891144	5.726923	7.128536	5.780442	4.903662	4.680654	6.030564	5.795132	6.133201	0.648181426	-0.066490579	0.048294021	0.218618911
ENSG00000103260	METRN	4.479883	4.727933	2.751572	3.331439	3.824829	2.947211	3.930048	4.694798	5.951124	0.331634583	-0.204720981	0.242125203	0.218655957
ENSG00000178982	EIF3K	137.80426	126.106361	127.278246	112.385177	114.862801	108.245775	140.845396	149.092599	133.354133	0.431417904	-0.059100016	0.005036915	0.218726527
ENSG00000163832	ELP6	12.999622	13.070072	12.661293	10.808756	9.725291	11.29647	13.790633	14.034121	14.739912	0.174666558	-0.177141935	0.050517164	0.218771879
ENSG00000168090	COPS6	47.157339	43.504186	40.901765	41.215913	40.773914	42.21098	45.465703	48.194616	49.879542	0.508855206	0.063248101	0.00862013	0.218781628
ENSG00000143842	SOX13	16.853444	17.766935	18.134441	16.375871	15.347923	15.608476	19.00126	18.396668	19.852466	0.865635807	-0.017362556	0.002487646	0.218808912
ENSG00000148248	SURF4	127.453246	117.499702	115.212268	133.541621	134.472541	134.770615	129.823506	127.407306	135.782717	1.75E-08	0.30335932	0.000398488	0.218918287
ENSG00000072062	PRKACA	34.927271	31.888264	32.806478	31.901262	34.963117	34.355913	35.554843	35.484827	38.453723	0.031885034	0.158010966	0.001737407	0.218951001
ENSG00000164941	INTS8	13.854365	13.848838	14.591574	13.261391	13.413838	13.457884	15.320182	15.214649	16.195578	0.640558181	0.04244694	0.00181529	0.219161029
ENSG00000147164	SNX12	16.041285	16.065158	17.124859	12.64536	13.273308	14.752854	17.989203	18.398158	17.748704	0.171788299	-0.137516869	0.013449054	0.219179564
ENSG00000149503	INCENP	19.977582	20.455958	20.215464	16.176065	14.113884	16.168411	22.679194	19.488866	24.419676	0.000845266	-0.239120574	0.005244518	0.219204717

Gene ID	Gene name	Ctrl-1 FPKM	Ctrl-2 FPKM	Ctrl-3 FPKM	IFNγ-1 FPKM	IFNγ-2 FPKM	IFNγ-3 FPKM	Sora-1 FPKM	Sora-2 FPKM	Sora-3 FPKM	Ctrl vs IFNγ DESeq2 FDR	Ctrl vs IFNγ DESeq2 log2FC	Ctrl vs Sora DESeq2 FDR	Ctrl vs Sora DESeq2 log2FC
ENSG00000072506	HSD17B10	32.396243	32.787483	30.925798	29.179458	26.919093	28.68334	34.545894	34.348267	35.091616	0.886374932	-0.018687699	0.014468901	0.219217926
ENSG00000117592	PRDX6	220.078568	230.210678	228.094406	215.956284	222.810791	213.414291	251.089264	245.753342	243.225311	0.10908377	0.090874449	0.000268721	0.219280822
ENSG00000131171	SH3BGRL	3.574531	4.33327	4.102923	3.265644	4.054499	3.697576	4.930969	4.152012	4.019142	0.92020154	0.02554449	0.205186174	0.219284285
ENSG00000163110	PDLIM5	11.319736	13.217088	14.296652	15.046754	13.20947	13.812602	16.475873	15.321841	17.161868	0.002023512	0.264137293	0.016400345	0.219357874
ENSG00000196507	TCEAL3	1.46994	1.627719	1.294066	1.576442	1.378281	1.970981	1.338207	1.592929	1.867622	0.361787243	0.334546421	0.532565245	0.219373327
ENSG00000177548	RABEP2	5.280164	5.491576	5.487303	5.061104	4.834812	5.307075	7.457592	6.235005	5.908706	0.41855344	0.1136353	0.107135346	0.21952037
ENSG00000131100	ATP6V1E1	117.805485	104.417984	114.08524	85.501505	94.146283	100.715547	114.401561	118.651357	111.147227	0.607147528	-0.043788593	0.000773134	0.219630043
ENSG00000146263	MMS22L	3.329473	3.799332	4.11755	3.493329	2.960371	3.134809	4.176492	4.025626	4.435855	0.682100128	-0.055058784	0.037103106	0.219664189
ENSG00000134882	UBAC2	59.53058	57.929098	55.82514	54.312392	56.108305	54.98591	62.38241	60.779323	66.78878	0.253249073	0.077609342	0.000334711	0.219684532
ENSG00000112305	SMAP1	17.001917	16.896893	15.327521	18.252958	17.616062	18.751342	18.613919	16.958055	17.285476	0.000126759	0.269490866	0.005676882	0.219725549
ENSG00000117143	UAP1	30.503339	33.177419	32.098388	38.030692	39.198976	37.788518	33.895105	35.054055	35.056423	3.54E-10	0.398625312	0.002803887	0.219747564
ENSG00000071051	NCK2	27.378255	28.31583	28.897294	16.781862	16.639956	17.443557	29.294605	25.798977	35.048321	1.05E-17	-0.551552884	0.016149432	0.219763504
ENSG00000103429	BFAR	41.398603	40.026659	40.63072	35.852361	36.668253	36.378235	44.813312	43.10319	49.998257	0.453632995	-0.05841526	0.000588113	0.219823558
ENSG00000114388	NPRL2	10.023482	7.322467	7.937328	8.370947	8.393758	8.918474	8.60792	9.296123	8.302116	0.188384933	0.192490765	0.127217689	0.219869137
ENSG00000163507	CIP2A	16.172423	18.32976	16.252659	12.991541	12.028846	13.574414	13.665834	19.008972	19.513446	0.005054684	-0.244789506	0.007922839	0.219968527
ENSG00000106927	AMBP	855.353573	760.705151	701.511292	737.0541	755.706634	746.135258	807.135262	836.679386	849.795726	0.156182781	0.120586515	0.008569738	0.220021315
ENSG00000160445	ZER1	7.15813	6.572724	6.016654	8.235633	7.499943	8.245729	6.72871	6.46745	8.553874	0.00000376	0.41802736	0.057624057	0.220112346
ENSG00000103381	CPPED1	16.936572	15.921286	15.174242	19.497078	21.557737	20.320927	17.637801	17.80117	17.345566	2.07E-11	0.424188292	0.003496827	0.220118697
ENSG00000086300	SNX10	6.740444	8.196992	7.855713	5.210637	5.045832	5.742168	8.243282	7.68174	9.163662	0.000304633	-0.412443303	0.06132881	0.220289883
NewGene_4969	--	0.774458	0.847885	0.733889	0.920452	0.705465	0.695743	1.022529	0.943057	0.594684	0.80470149	0.122865686	0.598931509	0.220340588
ENSG00000122861	PLAU	0.365333	0.311764	0.229651	0.36347	0.424671	0.335531	0.474368	0.356926	0.177888	--	--	0.707601438	0.220448359
ENSG00000107438	PDLIM1	66.415512	65.790718	64.378532	50.579182	47.564022	50.670589	73.144043	70.094414	71.533745	0.0000262	-0.254255641	0.000850198	0.220574019
ENSG00000006625	GGCT	71.275676	79.534406	84.475335	81.331662	76.336954	77.505229	85.810903	87.220345	82.87254	0.070837755	0.142853348	0.010189383	0.220614053
ENSG00000187024	PTRH1	2.224937	2.561928	3.181104	2.821484	2.773881	3.111076	2.989691	3.517259	2.272244	0.540294185	0.205691576	0.452845309	0.220675571
ENSG00000111077	TNS2	6.901555	6.5711	6.330492	6.263079	5.112704	5.363586	7.589658	6.686421	7.470675	0.375729054	-0.101975988	0.014733467	0.220807688
ENSG00000173163	COMMD1	6.092105	6.775169	5.702064	6.741241	5.791386	6.969862	6.710089	7.552659	6.073487	0.308478711	0.208660751	0.273366067	0.220848036
ENSG00000163528	CHCHD4	14.901013	15.324327	15.854024	15.198246	14.717217	14.96232	17.386396	15.715383	17.234156	0.3461826	0.104798894	0.025626026	0.220877655
ENSG00000182973	CNOT10	11.035797	11.652455	10.879094	12.017411	10.484848	11.662731	12.297618	12.239642	12.36922	0.054613646	0.171921454	0.01098145	0.220912208
ENSG00000107404	DVL1	27.04289	24.726074	23.788845	21.444472	22.174057	22.66614	27.045481	25.890913	29.469569	0.641526564	-0.047092294	0.005762031	0.220915295
ENSG00000115685	PPP1R7	22.227453	28.228787	27.549969	26.05605	22.280412	26.424455	27.109055	23.118451	40.012761	0.058407526	0.153567783	0.00593312	0.221165632
NewGene_582	--	1.396692	1.679532	1.540465	1.761273	1.592996	1.142314	1.691117	1.827662	1.516061	0.738990656	0.098511009	0.306995574	0.221222039
ENSG00000101158	NELFCD	59.767876	61.042477	62.281986	56.920398	55.845931	57.440163	65.347999	66.703582	69.699602	0.615563554	0.036587397	0.000386001	0.221222138
ENSG00000141384	TAF4B	0.599037	0.741351	0.622661	0.336117	0.39778	0.316018	0.70473	0.606509	0.945465	0.002503825	-0.777245308	0.367577794	0.221539904
ENSG00000051620	HEBP2	4.078297	4.299233	3.303567	3.529326	3.399944	4.063553	4.38253	4.392278	3.873232	0.696596193	-0.052708966	0.046031295	0.221606074
ENSG00000011376	LARS2	8.00875	7.843715	7.453138	7.906753	7.493435	7.812477	8.500339	7.930879	9.15013	0.048720859	0.176178346	0.011586934	0.221718422
ENSG00000048828	FAM120A	22.55396	24.324673	25.035419	27.93529	26.486224	27.423744	26.401336	23.763733	29.512263	1.46E-08	0.341306148	0.006662538	0.221752806
ENSG00000125733	TRIP10	33.51052	32.105542	35.685569	40.992524	39.256287	39.33912	37.610879	36.751386	36.27231	5.09E-10	0.393785835	0.004309733	0.221938722
ENSG00000104872	PIH1D1	85.624571	83.976307	79.249574	74.508614	65.887628	77.217622	87.801098	93.856234	93.733362	0.548174624	-0.055192521	0.002129298	0.221971666
ENSG00000198858	R3HDM4	57.08577	56.50941	52.533555	50.70107	49.751088	49.812483	61.381821	58.731618	63.673982	0.945158579	0.006876282	0.001019732	0.222106961
ENSG00000122705	CLTA	187.063116	178.960566	169.418884	145.137619	145.443501	152.843896	196.512673	189.394453	199.843194	0.055672284	-0.125452163	0.000430418	0.222189697
ENSG00000179222	MAGED1	44.027041	41.134871	39.657398	47.354953	47.808653	46.231899	45.478426	42.803938	47.871912	9.5E-09	0.343579491	0.001157688	0.222433571
ENSG00000102226	USP11	18.297988	18.072122	18.507032	17.189898	16.894733	17.421571	18.563583	19.274512	22.200245	0.525433607	0.053325728	0.004517588	0.222498628
ENSG00000146701	MDH2	135.37492	130.016822	129.679651	106.383822	110.509439	111.004271	133.012527	141.259731	159.153436	0.037102749	-0.122043225	0.001179276	0.222520697
ENSG00000173020	GRK2	43.136364	42.738483	40.445408	36.155693	36.739674	36.695	44.395733	42.608841	51.464916	0.419723634	-0.057428008	0.001932897	0.222554138
ENSG00000184271	POU6F1	1.179436	1.428581	1.091061	0.898064	1.017926	0.97843	1.103158	1.224585	1.59491	0.775049346	-0.072189446	0.287847242	0.222568943
ENSG00000120992	LYPLA1	30.253569	36.06266	36.924285	33.222995	32.146833	32.949413	36.955561	36.657338	38.172595	0.368884299	0.08633169	0.010466895	0.22260272
ENSG00000106460	TMEM106B	19.839282	21.327495	21.747327	19.453947	21.397241	18.85847	24.9113	25.422402	25.142837	0.26085478	0.098665718	0.00701316	0.222620559
ENSG00000185104	FAF1	6.17107	6.113462	6.217457	5.032136	4.518548	4.623828	6.879233	6.394667	7.08529	0.003072484	-0.23827081	0.004703141	0.222648638
ENSG00000165832	TRUB1	8.650822	9.015491	9.509663	8.805495	7.691037	8.19997	10.226908	9.149905	10.372694	0.945771509	0.009626662	0.014860059	0.222656149
ENSG00000183955	KMT5A	13.340667	13.24625	15.348708	16.137213	14.627603	13.977361	15.377529	14.46949	19.136015	0.336600804	0.098539429	0.016453293	0.222711426
ENSG00000039068	CDH1	19.027574	18.995068	17.883725	42.715479	40.695837	42.928084	22.032473	19.332638	19.811716	7.21E-143	1.325597196	0.004423362	0.222798869
ENSG00000085449	WDFY1	23.800055	25.41811	25.973255	46.090107	43.260414	44.993529	28.08238	26.122057	28.096123	1.83E-79	0.984596473	0.00083201	0.222818918
ENSG00000149823	VPS51	62.104696	56.93016	54.628702	48.973766	50.272093	56.567314	59.618842	61.258384	68.919992	0.586616169	-0.047618914	0.001320086	0.223010596
NewGene_4864	--	6.353284	6.019234	5.894295	6.047487	6.230188	6.449796	6.415784	6.839519	7.783388	0.032838403	0.209463376	0.029537982	0.223018363
ENSG00000151892	GFRA1	1.719164	1.57538	1.719356	1.047981	1.610633	1.135399	1.775392	2.437182	1.913672	0.008075949	-0.360762499	0.067064115	0.223040294
ENSG00000142303	ADAMTS10	1.033396	0.771393	0.536228	0.398894	0.278584	0.321328	0.894408	0.810239	0.702972	0.007079717	-0.844497877	0.443657092	0.223141412
NewGene_5025	--	1.878317	2.051375	2.624904	0.969666	1.163931	1.768258	2.232571	2.248212	2.669652	0.084218833	-0.581380825	0.419504228	0.223161804
ENSG00000095321	CRAT	14.39919	13.275097	10.845824	14.715411	14.954264	14.099523	15.123218	13.162613	13.67683	0.000932956	0.336215784	0.054024032	0.223187995
ENSG00000213648	SULT1A4	6.644776	7.769412	7.830159	7.02965	6.292287	6.080576	6.022282	10.007189	8.624752	0.837140511	-0.042026084	0.252264346	0.223219608
ENSG00000073111	MCM2	79.658899	81.345743	83.943495	86.08069	81.519196	87.550218	84.165398	88.506593	101.852153	0.00000171	0.242649565	0.000240006	0.223262585
ENSG00000278845	MRPL45	59.32109	57.897888	58.965778	47.685403	51.084003	48.07948	62.229827	64.835499	65.718444	0.102965083	-0.115737357	0.000819972	0.223323158
ENSG00000178607	ERN1	3.769238	3.394964	3.770034	5.236077	4.333989	4.571395	4.338266	3.882207	4.344772	0.000000048	0.496090179	0.024044442	0.223368465
ENSG00000170144	HNRNPA3	109.793137	119.404957	119.840972	119.701316	114.916165	121.395159	141.507212	141.24656	135.213109	0.024529642	0.14244404	0.001452475	0.223409883
ENSG00000117410	ATP6V0B	50.957677	50.357161	47.586112	37.044855	39.467923	38.47885	53.033708	52.750798	57.993975	0.001629775	-0.221865569	0.002167314	0.223438848
ENSG00000152348	ATG10	3.436223	4.158514	3.790341	2.801097	3.208173	3.018721	4.465032	3.461851	4.125707	0.396599345	-0.15077826	0.152586235	0.223531502
ENSG00000177697	CD151	127.819623	124.013808	117.2358	79.113484	82.617784	87.439243	128.353776	143.715537	134.480651	1.47E-13	-0.449545687	0.001505519	0.223532515
ENSG00000242616	GNG10	18.584879	21.481842	19.925089	20.288479	18.19639	18.64962	21.649794	21.130035	22.932364	0.507315693	0.076911395	0.01743312	0.223692275
ENSG00000141698	NT5C3B	43.650301	47.112337	48.941477	42.085077	38.73568	40.811755	51.131373	49.803235	51.61285	0.602260956	-0.055086995	0.007772233	0.223877241
ENSG00000176909	MAMSTR	2.860396	2.956608	2.831698	3.658809	3.218964	3.971328	3.31205	3.725321	2.573399	0.007969491	0.481319423	0.290491629	0.223934363
ENSG00000100151	PICK1	9.712078	8.496498	9.672339	7.788155	8.155903	7.085425	9.891455	10.359607	10.869681	0.312542851	-0.135640076	0.050426873	0.22406538
ENSG00000172062	SMN1	17.773486	18.828799	18.414719	18.564476	17.638536	18.028495	20.444512	19.536134	20.201044	0.205413928	0.123685997	0.013546788	0.224112698
ENSG00000111581	NUP107	12.718139	13.805524	15.190241	11.915657	11.350704	10.059866	15.079774	15.328667	15.135533	0.002223278	-0.228278843	0.003541695	0.224127153
ENSG00000179361	ARID3B	9.124814	8.624198	8.459846	6.604728	6.125613	6.423951	9.538092	8.196026	11.09283	0.00021275	-0.306643272	0.028964613	0.224296823
ENSG00000065485	PDIA5	12.56265	14.757741	15.6652	21.026062	20.080247	20.335015	16.441905	15.965174	14.876577	1.53E-13	0.662873177	0.054653349	0.224302154

Gene ID	Gene name	Ctrl-1_FPKM	Ctrl-2_FPKM	Ctrl-3_FPKM	IFNγ-1_FPKM	IFNγ-2_FPKM	IFNγ-3_FPKM	Sora-1_FPKM	Sora-2_FPKM	Sora-3_FPKM	Ctrl_vs_IFNγ_DESeq2_FDR	Ctrl_vs_IFNγ_DESeq2_log2FC	Ctrl_vs_Sora_DESeq2_FDR	Ctrl_vs_Sora_DESeq2_log2FC
ENSG00000099834	CDHR5	0.670022	0.693288	0.353711	1.417193	1.017945	1.487274	0.954362	0.537093	0.669974	0.0000911	1.060116566	0.544210042	0.224306111
ENSG00000140577	CRTC3	3.497938	3.12861	3.30669	3.883212	3.422683	3.384165	3.938696	3.550357	3.52641	0.038473247	0.243052573	0.060476275	0.224345147
ENSG00000150540	HNMT	12.524494	12.09797	13.266869	11.796021	11.636311	12.051815	14.975243	15.585574	14.854037	0.252817505	0.111898822	0.014113152	0.224381138
ENSG00000120925	RNF170	4.888303	4.525921	4.937912	4.725251	4.700227	4.550319	4.747141	5.069118	5.343226	0.463025823	0.097021581	0.047473014	0.224412429
ENSG00000136270	TBRG4	80.878786	77.773616	71.451851	64.880564	64.875231	63.194329	77.934658	84.398933	88.250483	0.1166117	-0.109929133	0.002536452	0.224480163
ENSG00000243279	PRAF2	2.627985	3.131005	2.125405	2.226441	2.035954	2.649023	2.179816	2.876402	3.977532	0.92133605	-0.036993714	0.424103068	0.224491579
ENSG00000154814	OXNAD1	6.811158	5.565609	5.540039	5.611882	5.527771	5.188795	8.123554	6.491456	6.370348	0.594618052	-0.073186918	0.022738069	0.224493617
ENSG00000137074	APTX	15.829527	16.242346	16.169755	15.850199	14.670488	16.852428	17.107088	16.390424	17.791071	0.351886461	0.111342071	0.012722372	0.224542944
ENSG00000167881	SRP68	42.539515	40.104935	42.159974	42.837253	43.034849	45.696135	45.602255	43.605073	48.853184	0.000798396	0.214152262	0.000890283	0.224556143
ENSG00000221914	PPP2R2A	15.462324	16.797095	19.284472	15.805084	15.67631	14.562829	19.357303	18.245751	19.339506	0.852272749	-0.024123341	0.01364571	0.224657961
ENSG00000168899	VAMP5	29.312206	26.819328	23.86828	56.840103	58.347557	64.156281	26.822762	31.872854	28.822338	1.31E-45	1.311402546	0.084144316	0.224725344
ENSG00000131788	PIAS3	6.239175	5.922662	5.406247	5.343063	4.985914	5.07376	6.43126	6.007358	6.89464	0.763724685	-0.046563527	0.050918078	0.224888398
ENSG00000101150	TPD52L2	66.25632	59.995728	64.006185	48.474994	49.331528	49.250949	66.258155	68.033685	74.248345	0.000325689	-0.223965522	0.001191529	0.224945959
NewGene_2775	--	13.101521	18.949678	16.790474	9.751598	12.593899	14.778131	19.250353	17.666096	16.507685	0.178364828	-0.24387665	0.117160407	0.225023726
ENSG00000196998	WDR45	13.933943	13.340976	10.936255	12.932751	14.51233	14.188955	13.19841	13.911304	15.405441	0.049507211	0.244036442	0.079613832	0.225070958
NewGene_1443	--	1.546942	1.432716	1.78002	1.000265	0.898845	0.835601	1.629098	1.558552	2.001841	0.006803997	-0.670754363	0.339104594	0.225328136
ENSG00000261740	BOLA2-SMG1P6	6.977027	2.119443	4.608135	5.71948	6.951395	7.209036	2.279928	8.094008	7.578792	0.014389242	0.470156368	0.729899633	0.225425819
ENSG00000164442	CITED2	10.631981	8.618358	9.17301	10.333116	11.021002	10.523162	10.402331	9.677764	11.028323	0.002999729	0.317143001	0.043960578	0.225447021
ENSG00000113296	THBS4	57.167126	50.009853	48.68285	33.095692	31.210958	31.260111	52.096752	57.896336	60.938992	6.02E-17	-0.559079238	0.007537367	0.225476848
ENSG00000133318	RTN3	94.36477	78.588272	79.445548	87.515811	95.276389	67.728653	104.923925	88.438279	109.352522	0.756023487	0.027025533	0.000511045	0.225487799
ENSG00000172354	GNB2	37.811669	39.68086	37.201813	38.815488	38.090257	39.035488	41.10232	40.499492	44.561466	0.007697423	0.177186274	0.001460924	0.225540077
NewGene_6571	--	0.703816	1.246741	1.13507	0.60589	1.155652	1.015074	1.09658	1.278163	0.962139	0.815222874	0.076509373	0.338963345	0.225670674
ENSG00000127328	RAB3IP	8.368669	8.858643	8.646307	5.87243	6.017732	5.973216	12.593331	10.464359	11.737226	0.00263569	-0.248440727	0.008779388	0.225716998
ENSG00000101181	MTG2	8.926899	8.465507	7.933474	8.680401	7.476187	8.14719	8.658717	8.548259	10.562233	0.458595055	0.09011634	0.037473368	0.225728683
ENSG00000136718	IMP4	30.111704	29.89747	29.138167	26.021784	23.597306	26.712047	31.529036	31.319267	37.103867	0.363307202	-0.081584453	0.003282117	0.225736759
ENSG00000101868	POLA1	6.774755	6.476739	6.096319	5.749258	5.769827	5.98027	7.087332	6.936191	7.210196	0.987778124	0.002024584	0.009126679	0.225764462
ENSG00000108819	PPP1R9B	5.802444	5.66205	5.048504	4.653371	4.165989	4.773095	5.847947	5.718377	6.570736	0.275921871	-0.133419424	0.031065897	0.225831366
ENSG00000167965	MLST8	31.965279	31.396604	28.938827	27.562059	24.737844	27.17383	32.809779	33.884028	35.062849	0.763724685	-0.032720978	0.00576165	0.225841052
ENSG00000110074	FOXRED1	16.993261	17.560498	15.620646	16.590027	14.72551	16.141849	16.96839	18.230382	19.727346	0.691410328	0.047167459	0.017126169	0.225897057
ENSG00000113838	TBCCD1	10.500991	11.064213	8.606499	10.910239	10.550128	10.908606	10.760433	9.351987	12.631138	0.005463768	0.278425765	0.031563695	0.225981537
ENSG00000111790	FGFR1OP2	10.819676	11.833921	12.396457	11.157531	11.153835	10.757203	12.408431	12.201645	12.972507	0.245838001	0.124275047	0.022513372	0.226011445
ENSG00000177731	FLII	38.629142	36.011187	35.323137	35.968763	35.675256	34.985783	37.071583	39.140124	43.950789	0.100128301	0.108414906	0.002825046	0.226263834
ENSG00000158019	BABAM2	8.577953	10.192632	9.001092	8.399033	8.201324	7.172306	9.922605	9.484849	10.858865	0.723140002	-0.059835382	0.062161032	0.226286352
ENSG00000171530	TBCA	56.804325	52.755609	55.568703	52.581886	53.402544	54.211463	55.185171	54.978675	48.539998	0.164600206	0.120774567	0.015284506	0.226350015
ENSG00000108828	VAT1	95.488151	91.425936	89.807434	54.767415	57.795085	57.936107	96.523023	100.806993	106.636777	9.83E-25	-0.552439815	0.000282883	0.226395378
ENSG00000241468	ATP5MF	75.454328	74.503689	80.778879	76.177554	89.890573	86.252163	91.15764	104.472484	58.962256	0.044239773	0.190288363	0.484697982	0.226449131
ENSG00000137100	DCTN3	27.939061	28.650557	25.461371	25.465198	24.742976	27.376685	29.879329	30.24708	29.98579	0.606015642	0.068359884	0.02874782	0.226513836
ENSG00000172586	CHCHD1	22.610183	22.29298	22.201536	20.400841	20.728171	22.024061	27.316149	27.836673	21.015509	0.89497379	0.019976614	0.076058269	0.226606211
ENSG00000078808	SDF4	88.995225	83.688058	79.764243	81.096133	77.881196	80.60249	87.552573	86.190182	103.013791	0.494620374	0.052739253	0.002640824	0.226766324
ENSG00000013561	RNF14	4.169987	4.824741	4.121742	4.980063	4.146188	4.608896	5.194632	4.267979	4.890293	0.031427069	0.245472108	0.058332246	0.226783848
ENSG00000134864	GGACT	1.600769	1.046096	0.618897	1.071372	0.914748	1.315117	0.811448	1.079736	1.477692	0.321765742	0.321649415	0.464314726	0.226790664
ENSG00000117054	ACADM	19.718718	17.048366	16.024466	16.986876	17.605366	18.974873	19.191483	17.2328	21.502664	0.106429864	0.140220051	0.006619385	0.22693517
ENSG00000139343	SNRPF	33.740929	37.562405	37.149635	39.889127	40.551174	40.710062	42.334415	46.644333	29.315113	0.0000254	0.305749008	0.405877657	0.226975419
ENSG00000129696	TTI2	10.292837	8.841318	9.191011	8.790618	8.439052	8.505485	11.017593	10.228198	11.523549	0.985025296	0.003235488	0.041158919	0.226995462
ENSG00000122545	7-Sep	58.274648	60.313512	57.762981	44.46671	44.566926	45.000915	60.941755	62.214046	63.731675	0.000864455	-0.196996125	0.000462913	0.227099832
ENSG00000166851	PLK1	61.314631	63.708285	64.758511	48.001589	42.474548	47.751927	71.704579	67.024638	70.630651	0.000000553	-0.309220465	0.000277824	0.227224374
ENSG00000185737	NRG3	0.619692	0.375013	0.478315	0.376052	0.39862	0.254824	0.495913	0.540329	0.696453	0.323870664	-0.485428962	0.5858007	0.227353848
ENSG00000065621	GSTO2	1.058552	1.5861	1.49162	0.821685	1.062726	1.133671	1.40243	1.915586	1.93547	0.08913509	-0.410567902	0.289486101	0.227397376
ENSG00000147099	HDAC8	7.408072	6.71011	7.873226	6.022349	6.270719	6.09593	7.817023	9.869831	9.013629	0.268049032	-0.154633068	0.056069669	0.227478735
ENSG00000235162	C12orf75	19.022381	20.959238	22.659331	12.498991	11.495669	11.089649	22.588938	21.879765	24.366168	1.68E-12	-0.69236254	0.021781095	0.227537161
ENSG00000138834	MAPK8IP3	9.083557	7.288642	6.932322	7.099788	6.823859	6.367259	9.430223	7.153758	9.083095	0.703877635	-0.047174615	0.016634675	0.227537894
ENSG00000112787	FBRSL1	16.591986	16.581913	16.362456	14.260517	13.244344	14.718005	17.998482	17.3078	20.933926	0.22159189	-0.094720402	0.00254014	0.227771959
ENSG00000068489	PRR11	23.440859	24.83935	22.180159	16.436199	14.404631	16.570702	26.465506	25.042563	25.848984	9.59E-08	-0.42161948	0.003909608	0.227792154
ENSG00000104964	AES	331.154251	307.171054	291.780472	224.709247	238.429228	242.043334	329.879237	344.525659	342.993441	0.00030477	-0.235845634	0.000910184	0.227807689
NewGene_972	--	11.933907	11.236561	12.324054	10.038878	10.947735	8.837117	11.853472	14.239597	12.407121	0.551003434	-0.103272896	0.104019868	0.227829709
ENSG00000167393	PPP2R3B	4.913814	3.321956	3.209653	2.465256	2.285671	3.063627	4.780595	3.807323	4.031212	0.06837829	-0.398887986	0.253109567	0.227847684
ENSG00000130702	LAMA5	15.671064	14.758615	12.500307	12.292456	11.278776	12.319364	14.823457	14.292264	18.041592	0.238411614	-0.109343831	0.016405386	0.227854683
ENSG00000100124	ANKRD54	6.717143	5.974031	5.987997	4.702162	5.517079	5.077687	7.069909	6.608251	6.951451	0.286006037	-0.16382205	0.065955423	0.227904059
ENSG00000131871	SELENOS	29.04568	29.213615	25.700763	33.16071	29.934077	32.062849	30.070106	30.831948	30.762277	0.000130888	0.322756452	0.013631208	0.227912662
ENSG00000148672	GLUD1	134.086685	132.327728	116.225647	197.31221	199.431046	190.320435	145.50856	136.199036	138.397263	2.3E-39	0.764867951	0.001659235	0.228039561
ENSG00000117133	RPF1	24.611831	24.003048	24.030833	20.970539	20.410744	20.846472	28.708019	25.310969	25.708124	0.452901723	-0.075405887	0.013480272	0.228282991
ENSG00000100211	CBY1	14.27765	13.652142	13.012124	11.898129	11.458972	11.386756	15.94881	14.916665	14.866384	0.440881865	-0.110363527	0.060018432	0.228307851
ENSG00000168310	IRF2	4.675853	5.009765	4.347404	10.223432	11.481801	10.682723	5.024641	4.961985	5.46448	1.97E-37	1.318650714	0.092819546	0.228372258
ENSG00000273899	NOL12	10.15614	12.746674	10.325481	10.764587	13.718477	9.013742	16.244312	15.691595	16.381909	0.964683269	-0.008057118	0.057991343	0.228383618
ENSG00000162676	GFI1	3.248347	3.21622	3.666689	3.470248	3.464471	3.364513	3.759763	3.601218	3.736383	0.239963086	0.184015421	0.113682236	0.228419462
ENSG00000090238	YPEL3	16.165139	15.342707	15.264888	11.602383	12.359169	12.159402	17.589563	17.578506	17.828388	0.178652259	-0.172357853	0.044000829	0.228424031
ENSG00000104518	GSDMD	22.127434	19.104018	18.24766	36.44681	35.293472	37.749655	21.617384	20.212658	22.517576	4.02E-42	1.086671761	0.023871396	0.228440666
ENSG00000146376	ARHGAP18	11.866081	11.591477	12.972444	11.685199	15.540137	12.67327	11.68721	13.608765	14.743443	0.009616083	0.280213965	0.018600512	0.228508951
ENSG00000143889	HNRNPLL	14.525221	15.1507	15.514888	12.84958	13.663028	13.434609	16.624244	16.522253	18.872478	0.688080663	-0.042614606	0.00598989	0.228609374
ENSG00000162385	MAGOH	22.678466	26.333316	28.040048	28.458642	30.162126	28.890812	29.43169	33.478097	21.301516	0.004297072	0.319903423	0.179817679	0.22861611
ENSG00000167635	ZNF146	40.157619	42.981789	42.51006	38.163665	36.331518	35.350508	43.710965	45.829961	49.725305	0.623800526	-0.038259147	0.001118273	0.228718565
ENSG00000095539	SEMA4G	23.789161	20.729152	19.903092	24.711166	23.775765	21.610058	24.002561	23.232923	23.947531	0.0000115	0.290771158	0.001439208	0.228736795
ENSG00000178772	CPN2	5.128279	5.355805	5.391882	5.943395	5.252107	4.949068	6.270052	5.94283	5.427596	0.22497337	0.155315236	0.056655404	0.228780875
ENSG00000105875	WDR91	2.471258	2.62716	2.420927	2.322029	2.678869	2.341896	2.750998	2.571769	2.957027	0.48873123	0.112874049	0.076210608	0.228844208

Gene ID	Gene name	Ctrl-1 FPKM	Ctrl-2 FPKM	Ctrl-3 FPKM	IFNγ-1 FPKM	IFNγ-2 FPKM	IFNγ-3 FPKM	Sora-1 FPKM	Sora-2 FPKM	Sora-3 FPKM	Ctrl vs IFNγ DESeq2 FDR	Ctrl vs IFNγ DESeq2 log2FC	Ctrl vs Sora DESeq2 FDR	Ctrl vs Sora DESeq2 log2FC
ENSG00000132423	COQ3	11.930517	13.599858	13.324451	11.713154	11.60851	10.797731	12.543454	14.86324	15.208045	0.754752285	-0.048605074	0.064211005	0.228967209
ENSG00000130413	STK33	4.631455	4.829861	4.09996	3.508021	3.199989	3.119684	5.356157	4.939156	5.615116	0.00143591	-0.427850831	0.073791755	0.228967698
ENSG00000107789	MINPP1	17.329834	18.398068	18.600367	20.505864	19.197182	20.418772	19.434494	19.188721	20.74395	0.0000322	0.301531713	0.003868981	0.22906472
ENSG00000104953	TLE6	2.487215	1.864677	1.737694	1.657912	1.7388	1.52157	2.521802	1.934707	2.303937	0.358260968	-0.261814526	0.345945372	0.229089972
ENSG00000111196	MAGOHB	9.280453	12.346621	11.843257	13.883705	10.026168	12.499789	16.812758	14.883661	14.32409	0.009666062	0.316908538	0.085216691	0.229098182
ENSG00000186010	NDUFA13	56.469204	53.23838	50.515527	49.957813	60.781485	60.912162	60.71646	72.049336	50.300896	0.027036654	0.226338221	0.04777517	0.229152142
ENSG00000164211	STARD4	15.41472	16.373072	17.293221	30.811254	32.419281	29.046228	19.88892	18.458883	21.643972	5.59E-53	1.056656954	0.007349458	0.229177166
ENSG00000247077	PGAM5	33.588663	34.410047	32.10383	30.383901	26.422743	29.935186	37.22282	34.922057	39.166644	0.557202369	-0.053258774	0.000547062	0.229227263
ENSG00000163453	IGFBP7	4.099519	4.605017	4.788913	3.678575	3.69337	3.827451	4.464845	4.913415	5.359086	0.579301684	-0.121031195	0.193423567	0.229242212
ENSG00000164919	COX6C	52.749133	55.712616	59.013127	55.61734	63.315321	52.926892	66.235489	67.153421	51.12756	0.111399492	0.142555692	0.054823315	0.229299655
ENSG00000090512	FETUB	3.449147	2.549318	3.136017	3.718231	3.612382	3.663037	2.920484	2.90535	3.534878	0.049803766	0.388541328	0.27672059	0.229399621
ENSG00000248643	RBM14-RBM4	24.946722	20.136761	22.148033	22.882221	23.20858	24.742079	22.614387	26.866845	23.792497	0.029312332	0.213090233	0.037176863	0.22944003
ENSG00000133393	FOPNL	17.139453	20.579172	20.925008	18.446115	16.822	17.287503	22.345759	19.948701	22.029843	0.973611733	-0.005102496	0.016810476	0.229517193
ENSG00000124614	RPS10	1595.327253	1553.368594	1377.556484	1186.629894	1184.605552	1289.299178	1622.75243	1683.6743	1605.588376	0.036381249	-0.135682599	0.001303979	0.229621808
ENSG00000172500	FIBP	54.725372	53.165321	52.143316	43.432085	43.124547	44.922459	55.760252	58.673477	64.170582	0.037778703	-0.156379819	0.002772006	0.22971515
ENSG00000050426	LETMD1	17.502085	20.654107	21.040668	17.196141	16.36765	15.555993	23.802565	23.309495	21.771761	0.225836853	-0.131096521	0.018144951	0.2297321
ENSG00000134490	TMEM241	1.473782	1.770969	1.972945	1.169277	1.608674	1.258015	1.777268	1.853758	2.192745	0.407904039	-0.216614226	0.277801372	0.22977139
ENSG00000005893	LAMP2	52.563691	49.228489	51.192881	35.942893	39.940388	37.191176	50.682658	53.047772	59.640447	0.0000368	-0.265253539	0.000497131	0.229778217
ENSG00000164405	UQCRQ	28.341264	32.005979	31.766097	30.077679	38.659241	39.771907	36.444701	40.226169	25.079203	0.001719754	0.357043034	0.15541013	0.229893672
ENSG00000156639	ZFAND3	59.873803	55.871133	57.71746	52.170318	52.940036	53.348481	64.6911	61.474759	65.968677	0.889239124	0.012179281	0.000179829	0.229916673
ENSG00000005156	LIG3	6.631113	6.312075	6.43099	5.609637	5.419177	5.455483	7.435614	6.949029	7.221909	0.327390185	-0.082544907	0.001781551	0.229941068
ENSG00000112695	COX7A2	107.704152	105.678918	104.612399	90.878201	98.85998	97.410551	129.346793	129.179961	99.647473	0.859828272	0.019215749	0.045028976	0.22995585
ENSG00000067369	TP53BP1	4.701876	5.142733	5.033232	5.122839	4.66511	4.25673	5.366695	5.996309	6.379897	0.750219939	0.033230745	0.00281589	0.230014424
ENSG00000084234	APLP2	130.176297	124.000251	124.151515	102.903797	100.727818	101.520409	138.759451	133.519114	146.01102	0.000897861	-0.166131751	0.0000368	0.230022776
ENSG00000179950	PUF60	94.771589	95.280836	87.397216	76.49454	82.116406	82.958435	102.890192	103.318615	113.025928	0.381651811	-0.06722354	0.00065304	0.230056816
ENSG00000010256	UQCRC1	79.716668	73.547075	68.995793	52.766725	55.518874	55.749309	75.675715	85.53157	83.156442	0.0000309	-0.289328199	0.005211426	0.230165141
ENSG00000114125	RNF7	15.75484	17.242696	15.483581	13.320076	13.847841	13.419125	19.008235	17.321031	19.730325	0.994974947	-0.001007314	0.012074414	0.230170226
ENSG00000168818	STX18	16.161302	14.586742	16.532256	13.708923	13.247773	12.501961	17.422014	16.704415	17.636948	0.302347122	-0.110572929	0.012210405	0.230177855
ENSG00000112992	NNT	13.925142	15.365759	14.519943	13.013692	12.570338	11.589683	17.225223	16.598852	17.68145	0.257989858	-0.084667064	0.000882164	0.230251303
ENSG00000155970	MICU3	1.37774	1.396818	1.429223	1.041796	1.090659	1.180369	1.544249	1.448818	1.694999	0.376274004	-0.191180118	0.203654556	0.230277743
ENSG00000171680	PLEKHG5	0.768554	0.701529	0.676232	0.815866	0.700291	0.680754	0.663116	0.740996	0.960889	0.645603948	0.126250278	0.332530086	0.230315838
ENSG00000106588	PSMA2	116.256196	123.896284	119.475667	149.386146	151.889321	151.953716	125.204315	130.256383	133.122019	5.16E-28	0.534865813	0.000198412	0.230411363
ENSG00000081087	OSTM1	6.976877	7.95648	9.101562	6.060347	6.589893	6.62883	8.421047	8.050699	10.012827	0.156648822	-0.168966158	0.043124681	0.230431031
ENSG00000183751	TBL3	9.393065	10.963063	10.758169	4.951281	4.422193	5.142586	7.230804	7.860607	14.719183	0.517902581	-0.076140315	0.014101224	0.230471963
ENSG00000160803	UBQLN4	29.906502	31.657278	30.778498	25.440056	23.928238	23.794247	32.951591	33.156738	35.511929	0.001927288	-0.188888543	0.000313017	0.230552507
ENSG00000172296	SPTLC3	2.425373	2.031361	1.675948	4.260589	4.458494	4.114013	2.665902	2.052043	2.46298	5.92E-21	1.122742087	0.143484866	0.230590098
ENSG00000104897	SF3A2	39.052642	38.803504	39.006081	35.714325	35.929479	36.278563	43.292069	39.103182	46.597159	0.706330466	0.03200994	0.001242887	0.230792784
ENSG00000174628	IQCK	1.933847	1.277911	1.423827	1.468046	1.704861	1.044188	1.940737	1.703822	1.429407	0.10186545	-0.410977123	0.311635394	0.230857992
ENSG00000168913	ENHO	0.662174	1.156987	1.090502	0.609301	0.713795	0.790516	1.086511	1.188761	0.908301	--	--	0.612500692	0.230977073
ENSG00000173915	ATP5MD	67.362204	72.147174	80.76813	65.319321	80.18811	81.833934	86.354664	95.921626	53.153795	0.170509594	0.16356043	0.441900971	0.231190415
ENSG00000138459	SLC35A5	7.842767	7.209232	7.338425	6.106597	6.494991	6.177654	7.929503	8.284861	8.743457	0.354060512	-0.098800234	0.011326433	0.23122365
ENSG00000179403	VWA1	12.67324	12.171381	10.717532	10.514245	11.553467	11.441169	12.109113	12.927442	13.158275	0.781128801	0.034258308	0.009849057	0.23128259
ENSG00000157456	CCNB2	62.387316	58.272238	62.423703	46.407708	38.680965	41.256886	64.421793	65.514345	64.317116	6.59E-08	-0.372180341	0.000799178	0.231329103
ENSG00000135722	FBXL8	1.58572	2.527768	1.640145	1.560103	1.165254	1.381929	2.589344	2.212001	1.661366	0.482162862	-0.26492047	0.48692875	0.231546042
ENSG00000141741	MIEN1	27.577975	28.334223	27.957644	23.624075	24.594519	25.514046	29.787882	32.736517	28.487189	0.763501467	-0.039034582	0.034873713	0.231559777
ENSG00000108479	GALK1	20.190829	15.306694	12.112134	17.753774	20.503996	20.07279	16.114839	16.355702	22.584111	0.001138639	0.412827409	0.094430835	0.231569134
ENSG00000204344	STK19	10.362906	11.164734	11.035803	13.517986	14.696912	13.722623	11.768296	11.476873	12.899714	0.000000868	0.495552521	0.041837231	0.231590601
ENSG00000088808	PPP1R13B	6.27144	5.497876	5.854285	5.414036	5.479414	5.530929	6.568581	6.157684	6.676083	0.606304113	0.060045954	0.011614878	0.231594907
NewGene_1053	--	0.925157	0.70852	0.532959	0.600886	0.582448	0.642269	1.116421	0.578819	0.898986	0.70907804	0.171891832	0.558587099	0.231827172
ENSG00000087074	PPP1R15A	20.90979	19.722339	21.008289	13.900541	12.256909	14.16564	22.391936	21.20613	24.455771	2.48E-09	-0.466428204	0.003209532	0.231871069
ENSG00000176101	SSNA1	60.204607	51.801532	48.898779	45.678169	47.333694	50.20087	56.504735	58.250646	62.473396	0.889945453	-0.019529529	0.015344849	0.2320699
ENSG00000161526	SAP30BP	36.87498	30.606437	33.770818	26.637364	29.75768	26.009896	38.769519	30.57174	34.555448	0.525514746	-0.057498051	0.00142099	0.232077078
ENSG00000186625	KATNA1	5.465734	6.011126	6.313116	4.390912	4.464681	4.287196	6.420817	5.645953	7.482479	0.043139715	-0.287783082	0.105459904	0.232086096
ENSG00000143248	RGS5	0.300276	0.22806	0.287941	0.1598	0.274756	0.224408	0.305056	0.334122	0.261777	0.750219939	-0.154850187	0.514807255	0.232090338
ENSG00000021852	C8B	2.102397	2.035502	1.340118	1.018452	0.883347	1.069757	1.91218	1.980167	2.112471	0.00625001	-0.730760699	0.350956712	0.232105495
ENSG00000204310	AGPAT1	59.565372	59.232168	59.442933	53.85477	51.387129	56.491781	62.307879	61.992625	72.620282	0.950533099	0.005645513	0.00070777	0.232165953
ENSG00000138028	CGREF1	1.705076	1.550358	2.128929	1.498626	1.214948	1.388036	1.870566	1.757433	1.951271	0.671087384	-0.145580511	0.372240046	0.232219069
ENSG00000149923	PPP4C	109.866048	102.171134	106.864031	88.699506	104.909568	105.162924	107.881338	129.922277	135.828833	0.440073622	-0.059930613	0.001050396	0.232244656
ENSG00000137825	ITPKA	7.207965	7.425151	5.733951	6.638857	6.399863	8.183172	7.567738	7.133499	7.67169	0.213697222	0.209405394	0.103978821	0.232274037
ENSG00000065518	NDUFB4	112.384707	123.264605	123.564199	104.310398	118.736117	119.475413	133.504945	138.525966	119.642359	0.366508886	0.086910013	0.014436912	0.232411508
ENSG00000162337	LRP5	127.611616	114.143039	104.724249	103.865179	105.804143	107.309655	115.21741	117.902638	147.114558	0.894794052	0.014329008	0.012303477	0.232528618
ENSG00000144837	PLA1A	15.601237	13.523088	12.841717	55.976342	59.342156	56.607632	14.108359	15.995656	15.372883	1.24E-194	2.191988484	0.037203578	0.232560637
ENSG00000132591	ERAL1	37.315128	37.837438	35.623576	31.083459	32.140546	29.356714	39.770138	37.860641	44.031106	0.104737221	-0.121337926	0.00132663	0.23256581
ENSG00000065268	WDR18	93.822182	92.500285	86.638089	74.670192	75.77565	84.963169	93.981574	100.230746	107.329737	0.666313346	-0.039984074	0.000798511	0.232809291
ENSG00000158373	HIST1H2BD	10.392005	10.206094	9.817767	9.766204	9.993649	11.667278	11.356816	11.036538	10.967717	0.211455968	0.218315839	0.132616914	0.233058794
NewGene_2164	--	1.984306	2.233121	2.54067	2.803299	2.516468	2.496408	2.524087	2.126929	2.815166	0.035058986	0.338246405	0.175680036	0.233315891
ENSG00000178028	DMAP1	19.152354	19.65505	18.231574	20.193251	17.828442	17.481491	21.510764	19.83789	21.11782	0.248641077	0.119951284	0.012133461	0.233563256
ENSG00000099817	POLR2E	85.149423	82.472544	79.288987	74.780527	81.072498	76.137055	98.364197	104.231074	102.966273	0.800916816	0.020180103	0.000175433	0.23359342
ENSG00000188352	FOCAD	7.647498	6.895688	6.500111	5.342797	5.420727	5.684375	7.741246	7.784861	8.137234	0.006687004	-0.239118122	0.007476215	0.233642895
ENSG00000136152	COG3	4.451439	4.331248	4.728756	5.348455	4.780727	4.664463	5.002905	4.619103	5.249381	0.011098373	0.268984312	0.027812453	0.233698607
ENSG00000211450	SELENOH	35.775888	36.434201	34.206104	24.485069	29.028453	30.008625	41.031684	41.158003	40.608938	0.0472742	-0.220325839	0.010111591	0.233716381
ENSG00000160014	CALM3	97.601309	101.597499	90.908254	194.003897	185.840795	195.492198	105.797149	109.777891	106.682112	5.55E-108	1.133702549	0.000638113	0.233770918
ENSG00000137103	TMEM8B	1.295816	0.952413	1.093902	1.344371	1.023528	1.201238	0.93774	1.099051	1.275852	0.347037625	0.230206972	0.308340852	0.233790933
ENSG00000104522	TSTA3	49.987466	46.421131	44.302443	42.084005	43.468946	41.980563	49.257566	50.35745	55.80104	0.967621501	0.005054341	0.004374458	0.233854083

Gene ID	Gene name	Ctrl-1 FPKM	Ctrl-2 FPKM	Ctrl-3 FPKM	IFNγ-1 FPKM	IFNγ-2 FPKM	IFNγ-3 FPKM	Sora-1 FPKM	Sora-2 FPKM	Sora-3 FPKM	Ctrl vs IFNγ DESeq2 FDR	Ctrl vs IFNγ DESeq2 log2FC	Ctrl vs Sora DESeq2 FDR	Ctrl vs Sora DESeq2 log2FC
ENSG00000066583	ISOC1	18.446507	18.733479	19.740795	17.165823	18.410982	18.426095	21.944101	21.119995	19.408798	0.488380833	0.071532323	0.013392008	0.23386726
ENSG00000120029	ARMH3	4.507724	4.159412	4.28268	4.357003	4.084102	4.060588	4.554271	4.913785	4.80611	0.367255323	0.112054993	0.032892129	0.233902981
ENSG00000151304	SRFBP1	4.101721	4.938282	4.92899	4.534494	4.12526	4.270885	5.151226	4.861898	5.398219	0.848789089	0.035319574	0.071628001	0.233911872
ENSG00000156298	TSPAN7	3.552365	3.419988	3.526054	2.555918	2.047504	1.879711	3.857077	3.408816	4.303689	0.003520381	-0.545537534	0.165999534	0.233915657
ENSG00000166140	ZFYVE19	6.753851	6.082731	6.370901	5.890175	5.977391	5.585043	7.393507	6.674883	7.007067	0.776285708	-0.04734193	0.049498818	0.233936802
ENSG00000090487	SPG21	69.090541	71.280477	68.359615	61.928773	63.202558	63.917409	74.470689	75.2742	78.816692	0.937438776	-0.007046906	0.000173132	0.233958733
ENSG00000156261	CCT8	183.16312	194.681999	195.186671	169.511628	168.63673	169.202548	211.124252	214.392105	204.135379	0.571088014	-0.036384023	0.000283426	0.234094285
ENSG00000090273	NUDC	84.772857	80.37881	82.817745	67.608047	65.931205	71.825894	87.45217	90.753244	95.003886	0.060595602	-0.122846522	0.000212408	0.23412537
ENSG00000169738	DCXR	47.386232	45.573811	38.89052	43.415617	43.015396	45.563086	48.361371	51.089652	46.481876	0.182657227	0.140560247	0.026761034	0.234376451
ENSG00000197912	SPG7	15.474732	15.091747	16.315951	15.587956	12.085119	13.369324	16.940319	15.933341	17.737169	0.811098049	-0.031295493	0.002595904	0.234430205
ENSG00000188010	MORN2	2.7722	3.554155	2.198176	2.685488	2.68264	3.001065	3.817119	3.676382	1.851427	0.793623764	0.107992005	0.539221979	0.234471668
ENSG00000107521	HPS1	9.154944	8.14751	9.189186	8.187779	8.859821	8.056669	9.600605	9.847883	11.527502	0.36010074	-0.101727149	0.011555958	0.234745595
ENSG00000143321	HDGF	269.48677	270.386513	273.817085	238.035567	249.55146	247.022284	299.184313	296.794323	303.179624	0.95580749	0.00457656	0.0000226	0.234812478
ENSG00000139131	YARS2	11.195765	10.888494	10.901981	8.65154	9.462495	9.005821	10.673914	12.114193	13.233094	0.302047108	-0.118514737	0.028367251	0.234996987
ENSG00000133739	LRRCC1	2.661815	3.457863	3.421011	3.669018	3.503394	3.46176	3.746637	3.225092	3.649193	0.048572556	0.274113001	0.096887874	0.235045297
ENSG00000198242	RPL23A	409.838861	362.174844	390.458056	339.130333	379.138977	350.252912	438.004543	468.628434	371.956583	0.816830827	0.022543301	0.01552021	0.235080079
ENSG00000170312	CDK1	40.006933	48.726879	50.131507	31.801377	28.945139	32.752869	53.305887	49.041772	50.467869	0.00000142	-0.422457152	0.010805263	0.235085699
ENSG00000176809	LRRC37A3	0.929878	0.680882	1.001292	0.808051	0.510161	0.837848	1.062843	1.276618	1.063941	0.534643599	-0.184806787	0.320536646	0.235104128
ENSG00000163872	YEATS2	13.941805	13.358918	14.1223	11.91979	10.994142	11.17237	14.659778	13.714088	16.521904	0.067355857	-0.126511443	0.001783357	0.235177547
ENSG00000206527	HACD2	44.812559	48.19048	49.942954	42.379737	41.672771	41.766516	53.329348	48.51579	57.205341	0.661802607	-0.034669517	0.00078719	0.235262974
ENSG00000121022	COPS5	24.551704	24.291802	24.461351	23.037233	22.117373	22.952223	24.62421	27.898671	27.936735	0.719826933	0.03867523	0.010942036	0.235383831
ENSG00000014641	MDH1	115.498989	123.366686	117.424175	96.840928	103.159165	96.469863	130.296138	132.706739	130.362408	0.036763413	-0.129277794	0.000272556	0.2353989
ENSG00000144659	SLC25A38	13.518687	13.348921	14.407185	11.355301	13.461044	12.511891	14.585351	14.56433	16.594995	0.986031869	0.002834111	0.011930006	0.235517681
ENSG00000213347	MXD3	4.54032	4.785889	4.370728	6.057392	4.196181	4.992678	5.055141	5.393515	4.199455	0.057982649	0.35455936	0.187487868	0.235543844
ENSG00000128595	CALU	101.402964	108.460191	102.255388	94.719192	92.788682	101.697649	108.553515	116.921756	120.513801	0.629307326	0.031015037	0.000019	0.235553122
ENSG00000116871	MAP7D1	8.875547	8.369564	8.585988	9.677575	9.701492	10.270584	9.279604	9.18505	10.156336	0.0000491	0.338922324	0.008728895	0.23557403
ENSG00000176393	RNPEP	23.466448	24.463649	23.435032	21.808064	19.972992	21.621658	26.418502	25.145148	27.941268	0.682509168	-0.038409019	0.001051374	0.235855662
ENSG00000114316	USP4	9.285676	8.986016	8.322702	8.489553	7.67212	8.121885	9.477939	10.094389	10.332955	0.86810552	0.022444273	0.011583595	0.235964727
ENSG00000156463	SH3RF2	0.889018	0.935241	0.707533	0.92763	1.218718	1.097091	1.120775	1.039252	0.924743	0.028157638	0.506122078	0.338945633	0.236117091
ENSG00000214575	CPEB1	0.559521	0.585419	0.630999	0.736437	0.363444	0.529372	0.763206	0.655631	0.812843	0.555966774	-0.21072542	0.400032555	0.236156297
ENSG00000156411	ATP5MPL	29.412415	28.461138	29.278422	26.204658	29.854348	29.624117	38.592604	44.794671	26.828561	0.548336376	0.078684009	0.155970546	0.236231799
ENSG00000123144	TRIR	101.250748	83.08863	83.000237	80.28692	80.478888	83.571109	96.611426	97.965047	100.265867	0.875055826	0.019718421	0.008014468	0.236277204
ENSG00000188566	NDOR1	13.944136	13.699518	11.944374	12.349593	10.387471	12.280998	15.067622	13.186759	15.087564	0.872313771	0.022948835	0.017857836	0.236286207
ENSG00000092010	PSME1	54.763193	53.711006	48.296324	344.21208	366.240357	362.107828	56.076996	59.494106	53.462083	0	2.921031269	0.007068317	0.236303744
ENSG00000188021	UBQLN2	3.822542	4.215618	4.04353	4.039346	3.816	4.221564	4.641078	4.57339	4.095903	0.222722289	0.149107146	0.049995731	0.236333314
NewGene_4529	--	6.361537	5.506304	7.557243	6.872965	7.207947	7.017155	7.664152	7.33123	6.437546	0.22842296	0.265671235	0.280835324	0.236342766
ENSG00000049860	HEXB	49.216876	48.380178	52.108323	40.728277	39.200015	39.362022	54.416916	54.841747	57.230397	0.005725626	-0.179506372	0.000510268	0.236484319
ENSG00000196872	KIAA1211L	0.809326	0.777662	0.733937	0.571905	0.49329	0.508682	1.081026	0.771108	0.770526	0.152316246	-0.399213439	0.357163962	0.236495053
ENSG00000173083	HPSE	0.606824	0.752566	0.747688	0.47557	0.569086	0.467269	0.761748	0.648875	0.921401	0.252588167	-0.326520935	0.349368991	0.236558886
ENSG00000023608	SNAPC1	1.614132	1.336545	1.452401	0.865803	0.700836	0.913111	1.836915	1.597409	1.415369	0.005467661	-0.674216257	0.308262976	0.236573838
ENSG00000160392	C19orf47	7.768615	6.967602	8.810822	5.641949	6.358987	6.717841	8.915618	9.305549	9.909425	0.905633686	-0.020411619	0.033539672	0.236690502
ENSG00000072682	P4HA2	3.863141	2.845706	2.335231	2.396513	2.730446	2.543769	3.611438	2.723735	3.532822	0.748728932	-0.085740369	0.260700871	0.236700218
ENSG00000174684	B4GAT1	87.626022	81.286903	79.235466	72.021797	79.997269	79.06105	88.959877	91.077827	94.161919	0.623487342	0.045307539	0.000371802	0.236775091
ENSG00000176485	PLA2G16	17.942324	17.247609	17.003149	21.893533	21.784144	25.962012	17.822536	20.29572	17.996167	7.03E-13	0.600246031	0.015624491	0.236783577
ENSG00000136731	UGGT1	16.614588	16.871081	15.600604	19.289717	18.574725	18.94308	18.41571	17.374663	19.48301	4.4E-12	0.353381637	0.0000775	0.236857414
ENSG00000120254	MTHFD1L	16.099195	16.604517	17.433405	11.365273	12.282644	11.54723	17.884033	18.248392	19.363319	0.00000816	-0.337655879	0.001583716	0.236870778
ENSG00000156587	UBE2L6	1.758841	1.925821	1.606207	225.846401	223.35924	226.406819	1.988708	1.643813	1.971313	0	7.158550632	0.388342461	0.236876384
ENSG00000165637	VDAC2	74.715191	68.838914	73.928579	58.312644	58.532975	56.315461	77.331417	79.233518	75.69525	0.11505373	-0.105590762	0.000351524	0.236895462
ENSG00000182934	SRPRA	34.233849	34.918677	36.689361	36.465443	34.719219	36.434534	37.774227	37.839329	41.241944	0.004452457	0.173960627	0.000327684	0.236980693
ENSG00000167964	RAB26	3.366146	3.204672	2.86126	2.630763	2.592006	3.017316	3.945209	3.333639	3.298353	0.860442578	-0.048422019	0.228050326	0.23698257
ENSG00000162174	ASRGL1	14.564172	14.876664	14.586066	13.16353	13.078947	12.112578	16.255561	15.869081	18.019507	0.650110032	-0.052479444	0.007231556	0.237107531
ENSG00000180329	CCDC43	17.724146	20.36001	21.858072	18.721132	17.732195	18.810009	23.063082	20.665801	21.805586	0.831830017	0.028215537	0.016508546	0.237277405
ENSG00000147155	EBP	54.326763	49.18015	48.315671	60.780474	64.982917	66.754583	52.857625	58.243143	58.352871	7.3E-10	0.433841021	0.005113499	0.237459752
ENSG00000048991	R3HDM1	13.443379	13.42927	14.739827	14.019803	13.085357	12.177746	15.636015	14.32976	15.852099	0.609666981	0.051763343	0.002333083	0.237568639
ENSG00000100410	PHF5A	53.368114	51.212597	49.065998	43.585857	45.39502	43.465855	53.069542	56.638741	60.246696	0.466659179	-0.066940628	0.002994501	0.237603956
ENSG00000081154	PCNP	72.297511	79.679734	83.755424	81.585255	76.481107	77.28835	86.81831	80.488688	92.848613	0.045737002	0.141551838	0.001295347	0.237609589
ENSG00000159199	ATP5MC1	53.197762	50.201833	49.377768	46.140497	46.933828	54.271163	54.878568	57.550214	56.04685	0.434254913	0.094402968	0.010487613	0.237683227
ENSG00000108582	CPD	63.455329	64.766335	62.610459	69.370702	72.596509	68.566872	68.978775	67.084921	75.137703	3.04E-09	0.289365307	0.0000115	0.237738712
ENSG00000197696	NMB	15.278784	12.614685	13.182715	9.613069	9.20348	11.364145	13.543598	15.193247	16.729867	0.047722976	-0.293532154	0.080388804	0.237820766
ENSG00000120699	EXOSC8	41.266342	44.937343	46.246548	41.680478	42.879821	42.903185	48.140225	47.915451	51.636291	0.472052918	0.068034266	0.003126984	0.237862736
ENSG00000171174	RBKS	1.000212	1.106451	0.930101	1.03615	0.983307	0.593575	0.964664	1.154349	1.247078	0.857097384	-0.083932426	0.445991819	0.237924723
ENSG00000198945	L3MBTL3	3.229831	3.437353	3.345494	4.127241	2.96034	2.948456	4.052927	3.886305	3.907144	0.684126189	0.06657196	0.055466257	0.237933142
NewGene_6188	--	4.586645	4.195901	3.752353	3.213628	3.386573	3.288828	4.457367	4.646425	5.234241	0.139022942	-0.204715258	0.057860659	0.238046508
ENSG00000135537	AFG1L	1.310211	1.253974	1.346988	1.081755	0.842815	1.155134	1.553402	1.516154	1.288908	0.465430933	-0.167214975	0.190437092	0.238059715
ENSG00000249115	HAUS5	6.713462	6.210302	6.747546	5.999806	4.982005	5.558831	8.321248	6.492751	7.658457	0.411741878	-0.098524371	0.021188187	0.23819329
ENSG00000111885	MAN1A1	95.060089	100.071884	100.044212	127.562408	125.408607	125.362221	107.244804	99.658623	120.160286	6.67E-31	0.505489089	0.000187466	0.238237491
ENSG00000102738	MRPS31	4.429067	3.444167	4.111683	3.684603	3.798713	2.945717	4.003447	4.671025	4.595156	0.82800526	-0.059542145	0.213387821	0.238309038
ENSG00000129657	SEC14L1	17.85767	17.260987	16.976036	22.866919	22.607062	21.039411	16.672837	18.538898	21.98114	2.52E-14	0.49396787	0.005003902	0.238449356
ENSG00000112246	SIM1	0.173566	0.264929	0.23684	0.204035	0.225313	0.235376	0.190696	0.294605	0.257746	0.768913663	0.123044261	0.493480799	0.238607436
ENSG00000166925	TSC22D4	9.185856	8.800678	8.218426	8.553073	10.992109	11.674992	9.023396	9.962413	10.293485	0.000124472	0.452371088	0.030846804	0.238640615
ENSG00000160999	SH2B2	0.56121	0.663226	0.662377	0.465721	0.475375	0.477466	0.703366	0.592726	0.772282	0.634559449	-0.238262101	0.547816623	0.238663228
ENSG00000065978	YBX1	450.764661	455.460677	469.532368	405.512079	407.888941	403.524959	497.672718	516.106528	507.727187	0.635482085	-0.029693921	0.000057	0.238706372
ENSG00000122026	RPL21	504.859113	490.561892	501.458715	425.34768	447.070289	437.118957	578.459335	583.09671	489.741696	0.515325439	-0.043181428	0.005915662	0.238774822
ENSG00000197978	GOLGA6L9	1.451679	0.841961	0.989477	0.914157	0.693078	0.580335	0.912285	1.183085	1.54438	0.282723393	-0.440403866	0.51528127	0.238776818

Gene ID	Gene name	Ctrl-1 FPKM	Ctrl-2 FPKM	Ctrl-3 FPKM	IFNγ-1 FPKM	IFNγ-2 FPKM	IFNγ-3 FPKM	Sora-1 FPKM	Sora-2 FPKM	Sora-3 FPKM	Ctrl vs IFNγ DESeq2 FDR	Ctrl vs IFNγ DESeq2 log2FC	Ctrl vs Sora DESeq2 FDR	Ctrl vs Sora DESeq2 log2FC
ENSG00000064547	LPAR2	4.440793	3.593866	4.065589	3.275002	3.41971	2.724335	3.90017	3.545666	5.766399	0.170845851	-0.269938355	0.225924139	0.239113268
ENSG00000137955	RABGGTB	33.114552	36.525993	38.519591	33.218601	28.72137	29.553996	39.703882	39.888131	38.522994	0.237784249	-0.111127736	0.004469931	0.239123858
NewGene_5123	--	0.243991	0.380827	0.395438	0.747785	0.47299	0.444955	0.439151	0.336521	0.34819	0.003626712	0.848229452	0.464314726	0.239156665
ENSG00000171853	TRAPPC12	10.678546	10.397492	9.042523	9.292601	7.882936	9.140798	11.209742	9.879015	12.38789	0.803529716	-0.037550333	0.030234861	0.239208116
ENSG00000143374	TARS2	16.899956	16.414717	15.878927	15.781758	13.273589	14.957643	16.650625	17.492378	18.944513	0.794603923	-0.031530569	0.003899049	0.239400924
ENSG00000101457	DNTTIP1	21.841247	21.593763	20.287423	19.689736	18.561323	19.479333	22.699486	23.946043	23.827089	0.979402462	0.003837063	0.011027607	0.239519135
ENSG00000127191	TRAF2	16.172333	17.050417	15.425663	17.566664	17.412798	17.29386	18.411089	16.181166	19.349598	0.0099917	0.209741491	0.00620271	0.239575429
ENSG00000183291	SELENOF	89.487093	93.463354	98.291229	90.015158	89.126411	86.630004	109.694367	101.612432	108.201799	0.437296885	0.059454418	0.000431056	0.239672869
NewGene_2913	--	0.649407	0.918182	0.912718	0.659482	0.623933	0.600572	1.421224	0.765056	1.138057	0.43925095	-0.248126636	0.405877657	0.239729458
ENSG00000095380	NANS	24.396331	20.399173	23.635842	27.618443	27.629596	28.858966	25.587734	25.611277	25.268104	0.00000142	0.446562061	0.023544497	0.239792815
ENSG00000164346	NSA2	16.581412	16.380652	18.144527	14.543946	15.762589	14.533167	19.594387	20.877559	17.276402	0.786392269	-0.028974305	0.007561186	0.239805116
ENSG00000243725	TTC4	7.894251	8.226356	7.220181	5.711361	5.369484	5.165544	9.12851	8.195389	8.524083	0.000784557	-0.376725377	0.030925709	0.239819683
ENSG00000189050	RNFT1	8.970936	9.438335	8.888869	7.066115	6.611912	6.795864	9.942184	10.167997	9.617196	0.025184409	-0.252703484	0.029227044	0.239859473
ENSG00000112699	GMDS	21.544359	18.036699	21.986275	16.010374	17.929262	16.330868	21.101297	22.104538	25.060564	0.212940742	-0.144886896	0.021519344	0.239905999
ENSG00000100263	RHBDD3	9.097669	9.398955	8.167956	7.60577	7.662448	7.062559	10.595315	10.112972	8.933294	0.53375449	-0.095997135	0.065848754	0.239923531
ENSG00000082515	MRPL22	7.692267	10.176894	9.070706	10.797304	10.573059	8.224983	8.742832	9.949328	8.709812	0.278242765	0.125300561	0.015086101	0.2402112
ENSG00000158286	RNF207	1.778982	1.852266	2.387998	1.534417	1.159174	1.084293	2.168506	2.098181	2.406569	0.007673446	-0.525781827	0.16347675	0.240265732
ENSG00000013275	PSMC4	86.590473	85.491983	83.717354	81.077239	84.23793	82.803262	90.925405	89.780602	99.275199	0.038265483	0.12309248	0.000121011	0.240366211
ENSG00000142444	TIMM29	9.594397	10.172787	9.641324	9.153961	9.663627	9.729001	10.229594	10.390404	11.272045	0.441994879	0.111078338	0.047836234	0.240440659
ENSG00000235272	RAMACL	12.081548	12.088853	11.459844	7.587112	10.40755	10.736878	12.748311	13.882508	12.6993	0.311195941	-0.156547253	0.021217993	0.240468698
ENSG00000196576	PLXNB2	43.326536	39.249359	36.293114	34.421784	33.122192	34.745979	41.048307	41.286451	50.719619	0.320263478	-0.072996144	0.001633038	0.240760345
ENSG00000101337	TM9SF4	52.373527	49.098844	47.563128	44.878039	42.407631	42.189118	53.083233	52.09539	60.648855	0.396248151	-0.060676872	0.000469508	0.240900895
ENSG00000161970	RPL26	940.658055	877.410941	881.957577	635.765103	722.84434	706.345029	1056.205291	1071.988959	876.427662	0.001394292	-0.165429695	0.013930803	0.241018553
ENSG00000181458	TMEM45A	7.171627	5.980296	5.848533	5.360163	6.02633	5.772668	6.221995	7.405073	7.118069	0.980409599	0.006023597	0.122371739	0.241053138
ENSG00000182197	EXT1	8.538276	8.589221	9.06343	7.842289	7.635361	7.547483	9.740302	9.103418	10.209444	0.668665996	-0.037761765	0.000556547	0.241131506
NewGene_5212	--	5.547201	5.067111	5.022044	6.675986	6.669427	7.354558	6.042754	5.979552	5.280311	0.03968352	0.550006297	0.418302097	0.241146764
ENSG00000089902	RCOR1	5.374638	5.807562	6.03983	8.195902	8.211211	8.7791	6.065105	5.551254	7.538821	3.64E-20	0.696335516	0.026872732	0.241152065
ENSG00000187922	LCN10	2.168018	2.750199	2.040071	1.337871	1.404804	0.930604	3.005482	2.950491	1.762555	0.005327595	-0.733152262	0.385295913	0.241191081
ENSG00000118816	CCNI	46.055773	47.871916	50.700813	43.381542	42.944672	42.889432	51.996663	49.692808	59.043872	0.872045495	-0.014453978	0.000799322	0.241376312
ENSG00000285897	AC004687.2	0.276647	0.269687	0.114841	0.076916	0.196897	0.180205	0.207931	0.210486	0.320232	0.515571125	-0.385612924	0.597911862	0.241393177
ENSG00000184481	FOXO4	2.30584	2.023056	1.974059	1.403245	2.040099	1.944287	2.441431	1.867022	2.711261	0.776915702	-0.074757027	0.188579088	0.241451565
ENSG00000184281	TSSC4	28.789133	27.320547	24.158603	26.129826	25.574369	28.850001	27.532524	29.650893	29.797575	0.30661191	0.11323801	0.013902464	0.241491684
ENSG00000080819	CPOX	11.079914	11.687861	11.692322	10.110223	9.459185	9.785312	12.574403	11.771327	14.274892	0.434493546	-0.081181444	0.008521297	0.241600971
ENSG00000164597	COG5	9.488696	9.285433	8.81878	9.267373	10.119717	9.712966	10.175677	9.47797	10.319076	0.006171661	0.240941753	0.004380988	0.241622646
ENSG00000064961	HMG20B	37.434697	35.780926	34.416598	32.419225	32.864736	33.327493	39.819332	41.259475	40.203741	0.999202724	-0.000146903	0.002035797	0.24173056
ENSG00000006125	AP2B1	24.00892	24.869673	27.42069	26.778402	20.589324	21.439452	27.746389	32.205524	29.522907	0.542335562	-0.046371047	0.0000845	0.241788267
ENSG00000100823	APEX1	193.75366	174.787687	177.14328	148.873199	156.827088	153.499576	197.899022	193.974719	201.129664	0.04767688	-0.119215936	0.0000884	0.241793642
ENSG00000126777	KTN1	46.09783	47.303248	48.133344	44.40025	45.013083	42.295473	51.543076	51.704794	54.76309	0.709900042	0.027810215	0.0000282	0.241830835
ENSG00000123064	DDX54	23.079385	22.755815	22.550559	20.356955	20.39516	20.351605	24.687093	23.700078	27.577409	0.851553571	-0.016249538	0.000305044	0.241949704
ENSG00000130734	ATG4D	7.632915	7.460119	7.269077	6.407715	6.196441	6.885275	8.10939	8.033477	9.073666	0.887978081	-0.026132971	0.046585219	0.242102929
ENSG00000164978	NUDT2	10.080562	6.31548	5.74042	5.096712	6.236591	6.268753	8.684686	8.660007	6.437191	0.580003398	-0.157867747	0.316762248	0.242152758
ENSG00000077380	DYNC1I2	31.744049	31.407218	30.512388	23.071629	22.860561	23.986893	34.794068	34.685486	33.470287	0.0000232	-0.272733874	0.000939501	0.242390579
ENSG00000164105	SAP30	4.622195	5.469389	4.737223	4.78033	3.74331	4.577203	5.987016	5.385198	5.042791	0.913492494	-0.030977147	0.206858915	0.242429321
NewGene_2834	--	6.956265	7.640196	8.348697	6.385839	6.019594	9.905542	12.438709	15.859563	7.722353	0.077457487	-0.224607347	0.461023442	0.24247898
ENSG00000119314	PTBP3	20.528704	23.524676	22.979391	19.703081	19.950169	20.714091	26.005013	23.278311	25.555975	0.947628636	0.006904099	0.000944573	0.242561472
ENSG00000048028	USP28	3.275889	3.47316	3.336301	3.214776	3.387448	2.995284	3.659648	3.601871	3.642053	0.796794532	0.04284658	0.0435993	0.242621123
ENSG00000182858	ALG12	6.089257	5.730287	5.42056	6.09119	5.551051	5.627219	6.119602	6.216517	6.854243	0.172562244	0.140711359	0.009446013	0.242652695
ENSG00000164885	CDK5	10.637339	8.439914	8.218687	9.601493	7.301234	8.505902	8.824857	10.377684	10.700366	0.78013289	0.052723764	0.083980557	0.242673729
ENSG00000134186	PRPF38B	12.892932	14.54769	16.641701	15.74401	13.732839	13.537286	15.935177	15.283284	16.160033	0.581858476	0.070289068	0.015130557	0.242736841
ENSG00000079462	PAFAH1B3	47.386552	38.687099	35.894968	30.532705	34.564326	34.451595	41.872853	46.093063	46.963976	0.236313502	-0.146268628	0.027400473	0.242768861
ENSG00000174808	BTC	1.596325	1.92837	2.182994	2.413718	2.657735	2.5493	2.046277	2.185511	2.143304	0.001623656	0.557274266	0.224528544	0.242807979
ENSG00000104413	ESRP1	25.547493	29.473641	28.560345	22.887221	20.399161	22.480531	31.926274	28.989734	29.117771	0.008261414	-0.196241417	0.001952681	0.242932498
ENSG00000117305	HMGCL	14.579807	13.284165	12.542976	11.080971	11.296102	10.935239	15.792217	14.482111	14.421422	0.445124498	-0.097795547	0.024404555	0.242940933
ENSG00000166478	ZNF143	12.909776	12.886077	13.588099	11.13382	10.851156	10.775973	13.653183	14.161168	14.471605	0.244784409	-0.110187049	0.003887169	0.242990113
ENSG00000145020	AMT	10.789378	9.470148	8.599144	7.572306	8.985073	8.539151	10.381271	10.277253	11.635794	0.920614293	-0.018984379	0.044473762	0.242993637
ENSG00000103549	RNF40	19.626605	18.595126	17.373146	17.450273	17.470114	19.209224	19.898759	19.519776	22.820164	0.357618152	0.071643782	0.001289141	0.243023487
ENSG00000166340	TPP1	75.649355	77.195163	68.975745	72.732	60.349718	62.253645	76.113346	81.849179	88.838332	0.276606198	-0.067434446	0.0000434	0.243183016
ENSG00000132128	LRRC41	14.161231	12.867847	11.42313	14.736994	15.099677	15.640934	13.569986	13.60873	15.263167	0.0000321	0.341554388	0.006792778	0.243211973
ENSG00000160695	VPS11	12.672077	12.197528	11.868149	12.171047	12.108977	11.870497	13.105408	13.481818	14.335314	0.204640866	0.110768703	0.002572675	0.2432158
ENSG00000067177	PHKA1	0.482618	0.69056	0.798176	0.721739	0.857601	0.887063	0.735159	0.688998	0.766289	0.043102258	0.479104706	0.320597779	0.243227981
ENSG00000100024	UPB1	1.138469	1.033755	0.841499	0.64615	0.733297	0.778084	1.183208	0.976417	1.185023	0.191614218	-0.417669877	0.360141921	0.24340611
NewGene_3522	--	19.931973	19.902508	22.359192	16.49932	14.929028	16.181918	18.951474	19.94926	23.537798	0.066526981	-0.173245492	0.011776955	0.243614743
ENSG00000165672	PRDX3	151.758987	160.14209	161.941483	128.967606	134.610779	133.268921	172.391953	171.334595	182.467712	0.077664351	-0.10780366	0.0000286	0.243715258
ENSG00000168894	RNF181	75.596908	82.540097	71.630655	75.767999	80.083094	86.627259	82.731601	95.695324	74.863345	0.009766675	0.233200021	0.034820879	0.243747303
ENSG00000141452	RMC1	9.633531	7.988259	8.971002	16.072272	16.636795	14.83468	9.224678	9.261901	11.454604	2.41E-27	0.99675932	0.037589133	0.243779759
ENSG00000132386	SERPINF1	232.770829	205.8306	187.870295	215.367871	216.305495	214.703679	234.047729	232.799241	232.46878	0.012698165	0.192281851	0.002733162	0.24391205
ENSG00000123136	DDX39A	117.088926	114.128944	111.200913	96.380133	94.993425	98.082899	125.303455	131.733108	133.592841	0.089780459	-0.102817553	0.000150122	0.243926873
ENSG00000102054	RBBP7	42.928667	42.48651	43.251476	39.464962	40.244249	38.657902	46.526863	43.512275	52.964348	0.478311063	0.053603505	0.000486006	0.244026336
ENSG00000122034	GTF3A	85.459102	84.650831	86.218807	86.340719	85.673385	85.623339	92.645655	95.00241	95.244887	0.024114529	0.133839307	0.0000796	0.244268487
ENSG00000181817	LSM10	12.375648	8.880472	9.655218	9.157667	8.256714	9.457388	9.842677	11.864845	12.668689	0.808837686	-0.056853758	0.173014004	0.244408173
ENSG00000166788	SAAL1	21.989818	20.412014	21.725795	18.568759	16.853247	16.819734	25.411022	23.763202	23.45684	0.105304803	-0.157175878	0.006594305	0.244410851
ENSG00000166681	BEX3	73.460067	82.19867	78.230383	72.412488	73.15101	77.413054	86.070649	91.139813	81.489394	0.352922502	0.079751397	0.005189628	0.244460839
ENSG00000162944	RFTN2	1.869056	2.068419	2.34587	1.27115	1.332163	1.151034	2.230301	2.47795	2.440325	0.0000217	-0.600341733	0.073322976	0.244480333
ENSG00000183783	KCTD8	1.261863	1.512902	1.36649	0.830653	0.7183	0.759106	1.738435	1.433073	1.269275	0.006728729	-0.699670001	0.327681238	0.244533984

Gene ID	Gene name	Ctrl-1 FPKM	Ctrl-2 FPKM	Ctrl-3 FPKM	IFNγ-1 FPKM	IFNγ-2 FPKM	IFNγ-3 FPKM	Sora-1 FPKM	Sora-2 FPKM	Sora-3 FPKM	Ctrl vs IFNγ DESeq2 FDR	Ctrl vs IFNγ DESeq2 log2FC	Ctrl vs Sora DESeq2 FDR	Ctrl vs Sora DESeq2 log2FC
ENSG00000105723	GSK3A	30.100028	25.478201	26.843246	25.458415	23.870725	25.383889	29.788176	29.058511	35.650276	0.745861198	-0.034933126	0.005811901	0.244669133
ENSG00000168140	VASN	11.511121	10.20167	9.630231	7.690934	8.437762	7.742422	10.910255	11.852371	12.079404	0.022124674	-0.246555658	0.017591406	0.244692677
ENSG00000010361	FUZ	2.848247	3.338106	2.485853	1.997835	1.755816	1.455171	3.061466	2.870904	3.616167	0.03724554	-0.514938969	0.276657525	0.244770274
ENSG00000075234	TTC38	41.507275	39.331321	35.52893	39.527435	39.971502	41.243182	43.377143	42.743667	43.677624	0.008342266	0.192185759	0.001135073	0.244808993
NewGene_591	--	1.942781	2.171012	1.352771	2.810393	2.019135	2.887546	2.385322	2.070525	1.419894	0.089168282	0.644866733	0.57575844	0.244944263
NewGene_6390	--	1.539523	1.054962	0.877692	1.334928	1.310532	1.308903	1.331276	1.347357	1.135584	0.282362392	0.326179345	0.409059123	0.244948033
ENSG00000164008	C1orf50	1.333336	1.197925	1.054304	1.24753	1.191289	1.151022	1.397736	1.274553	1.315467	0.511437252	0.145797489	0.189942889	0.244980437
ENSG00000109854	HTATIP2	36.127655	38.980727	40.159207	35.270905	36.131693	34.866583	41.066844	41.882948	44.442305	0.475778007	0.066104274	0.002528059	0.245042985
ENSG00000100813	ACIN1	70.414258	66.620268	67.074718	67.353305	66.294653	63.989289	73.370096	75.72103	79.502086	0.194716545	0.080696768	0.0000698	0.245167887
ENSG00000165282	PIGO	10.698916	11.726374	10.236927	9.037549	8.815957	8.699126	11.228715	11.221365	12.66627	0.253199201	-0.110576347	0.004235116	0.245180129
ENSG00000108961	RANGRF	11.004102	11.654093	10.192995	7.442185	7.423062	8.417665	11.791751	11.107719	14.145266	0.011657492	-0.355737751	0.0685873	0.245185325
ENSG00000139354	GAS2L3	8.867354	9.694479	10.262078	8.776642	8.411084	8.550351	11.379267	10.046964	11.63824	0.446866766	-0.089958753	0.012127564	0.245187592
ENSG00000101132	PFDN4	12.79325	14.95278	15.1209	13.689502	14.159374	14.030978	16.584023	17.464531	13.419649	0.35980689	0.114865615	0.076431403	0.245191691
ENSG00000123975	CKS2	109.866928	119.680122	125.868011	106.939178	99.201775	101.299118	123.703087	140.132736	130.600174	0.498487741	-0.062124336	0.0056942	0.245228777
ENSG00000156500	FAM122C	0.605458	0.406638	0.603798	0.871745	1.326935	1.041352	0.850306	0.463269	0.593388	0.000386305	1.087693157	0.557354716	0.245318359
ENSG00000196352	CD55	4.01058	5.662537	4.928623	6.831522	7.420456	7.092106	5.500336	4.960057	6.361496	4.02E-09	0.714855365	0.080185683	0.245418529
ENSG00000136271	DDX56	65.583942	65.244969	70.407989	65.832813	63.192445	64.939515	74.111002	73.982666	67.887297	0.303344509	0.066130507	0.0000407	0.245474906
ENSG00000168434	COG7	8.034966	7.567754	6.665785	6.275077	6.514663	6.968794	8.364836	7.735886	8.521595	0.864647521	-0.0291628	0.03362042	0.245489417
ENSG00000242612	DECR2	27.160624	27.140048	24.293084	25.819843	26.102187	28.215361	29.230506	25.546395	32.898042	0.058560539	0.17268494	0.011229107	0.245498053
ENSG00000164967	RPP25L	7.783008	6.869134	5.980348	5.714699	6.665546	6.421896	7.618776	7.977057	7.29908	0.941722654	0.019809566	0.163010153	0.245566601
ENSG00000143228	NUF2	18.003935	19.332426	19.16755	14.303492	13.285386	13.518541	20.803523	19.737872	22.234996	0.000657362	-0.291104279	0.004264451	0.245616362
ENSG00000065029	ZNF76	24.79344	20.912044	22.49171	19.049784	20.112676	18.779366	22.914419	23.601304	26.327395	0.38763228	-0.085875543	0.003898917	0.245629876
ENSG00000105289	TJP3	11.461289	11.0496	9.756337	8.789434	9.944371	9.030081	11.565362	11.424693	12.765552	0.511614641	-0.086530281	0.011151777	0.24566011
ENSG00000015532	XYLT2	3.14191	2.678	2.509437	2.481219	2.774204	2.43653	2.768464	2.897416	3.331229	0.93341284	0.02034859	0.129266829	0.245702541
ENSG00000150459	SAP18	25.860116	26.267621	24.611155	25.652031	26.980026	26.954291	30.567007	29.033459	26.669449	0.006020148	0.200016411	0.005194683	0.245703461
ENSG00000188807	TMEM201	6.841321	7.15224	6.41148	6.050659	4.772825	6.511089	7.457494	7.082579	8.231546	0.546808384	-0.090593886	0.011180448	0.245713697
ENSG00000143028	SYPL2	3.786328	3.334382	3.672107	2.175989	2.891878	2.674666	3.633567	4.067902	4.305635	0.031318678	-0.330689419	0.059205281	0.245725844
ENSG00000164741	DLC1	10.929733	11.481743	12.465403	8.095667	8.63389	8.617346	13.91168	13.61435	15.642705	0.0000391	-0.334901494	0.003796006	0.245903157
ENSG00000163882	POLR2H	48.59137	53.902301	48.42709	49.290236	55.329427	55.741342	47.106646	58.951234	55.837721	0.005034395	0.198016722	0.001170488	0.245929954
ENSG00000067191	CACNB1	0.537611	0.672893	0.489771	0.428699	0.40726	0.578019	0.696189	0.470753	0.793082	0.77178977	-0.123160924	0.44378893	0.246029132
ENSG00000165283	STOML2	108.390217	105.743994	103.562168	87.033611	84.137364	89.733293	116.587248	118.583837	118.470312	0.025025655	-0.137940357	0.000108981	0.24612337
ENSG00000076201	PTPN23	8.072004	8.492922	8.429722	8.267207	7.636208	8.248086	9.022474	8.676122	10.147881	0.267734255	0.096930348	0.002299374	0.24619239
ENSG00000163050	COQ8A	31.855285	28.533069	32.105551	28.661339	34.073944	29.243092	32.312604	32.730404	40.957953	0.241157525	0.104457236	0.004599262	0.2463728
ENSG00000113638	TTC33	7.890467	7.675996	7.921888	6.888752	6.408492	6.874916	8.011627	8.798816	9.001349	0.65752142	-0.058931142	0.018456225	0.246385959
ENSG00000182552	RWDD4	8.364916	6.274907	6.76397	7.462415	6.570788	7.055463	9.484444	7.311907	8.185747	0.058447676	0.211559923	0.031228621	0.246428384
ENSG00000206535	LNP1	1.629622	2.031836	1.590284	1.403056	2.056579	1.949437	2.140861	1.415738	2.298791	0.498153777	0.194491904	0.326914332	0.246508718
ENSG00000173846	PLK3	2.216333	2.263472	2.573752	1.047078	1.114234	1.011376	2.096929	2.70748	3.052346	0.000000185	-1.006560988	0.217922144	0.246525429
ENSG00000147687	TATDN1	22.042462	26.482413	29.381702	22.784817	22.846721	23.175959	29.678885	29.985213	27.721263	0.799101885	-0.039110304	0.035501888	0.246578202
ENSG00000063245	EPN1	17.806869	19.11031	16.099456	16.003081	17.363271	19.131383	21.618755	20.260894	27.513058	0.001252001	0.201296739	0.00071975	0.246615599
ENSG00000272636	DOC2B	1.787373	1.324127	1.436444	2.994052	2.898665	2.977438	1.623752	1.607764	1.834465	2E-19	1.108547348	0.128246521	0.246708731
ENSG00000089820	ARHGAP4	11.75277	10.348643	9.889711	10.253803	8.896582	10.491063	11.25006	11.759928	12.727275	0.932344399	0.013029214	0.011808255	0.24677397
ENSG00000139514	SLC7A1	15.173308	15.422418	16.812761	13.625201	14.262666	14.120908	17.443073	16.440971	18.941124	0.777913942	-0.026349307	0.000453475	0.24680465
ENSG00000120885	CLU	372.23103	305.719574	314.356544	336.422491	363.564099	375.079172	370.843363	362.804367	363.015264	0.083796959	0.140165539	0.002370085	0.246817223
ENSG00000196391	ZNF774	0.765831	0.828116	0.808473	0.662736	0.709363	0.615913	0.958482	0.631746	0.937074	0.879186347	-0.055052617	0.374491709	0.246886493
ENSG00000162076	FLYWCH2	3.666693	3.90661	3.903271	3.020386	2.841933	3.810212	4.915321	3.738317	3.926126	0.79193399	-0.082207635	0.278208519	0.246909516
ENSG00000105640	RPL18A	793.569017	767.886493	668.759229	776.94669	801.592526	825.388365	828.848938	830.727907	805.754244	0.00000856	0.270437198	0.00065237	0.246922415
ENSG00000141858	SAMD1	18.329031	19.225653	18.801718	15.665679	13.721986	15.276477	19.420525	19.47434	23.940985	0.030528069	-0.18757495	0.007208959	0.246989173
ENSG00000105552	BCAT2	13.100328	13.863544	13.198339	14.621135	13.896569	13.974033	12.437606	14.917213	15.651457	0.071854661	0.18866863	0.01742558	0.24703476
ENSG00000100243	CYB5R3	105.196317	97.267281	97.472663	89.759238	92.74257	97.816455	107.448849	106.264983	121.60813	0.413140707	0.057833352	0.000137697	0.247157202
ENSG00000138674	SEC31A	48.865659	47.031686	45.231196	51.060485	49.51577	49.014532	52.069639	49.346781	50.471887	0.0000114	0.232410115	0.0000407	0.247205861
ENSG00000073792	IGF2BP2	33.201427	32.546259	34.551668	31.5419	29.71018	28.729335	37.073775	35.928116	38.18759	0.991868551	-0.001105961	0.000102319	0.247319544
ENSG00000138029	HADHB	37.438799	37.420216	34.903166	34.568564	36.148075	34.807499	39.937266	41.88382	40.498692	0.336369961	0.074849659	0.000786646	0.247633835
ENSG00000196476	C20orf96	6.097115	4.9144	6.052955	4.907774	3.846031	4.08615	6.181191	6.047226	6.743583	0.165286653	-0.262132939	0.115163837	0.247645082
ENSG00000119655	NPC2	60.161229	61.600375	58.797336	57.724675	62.238617	58.809146	67.411986	68.777728	59.780454	0.034361767	0.162246106	0.005507426	0.247753438
ENSG00000170430	MGMT	7.256736	7.393663	6.521718	6.357276	5.728903	6.92996	8.786467	7.710001	7.023471	0.980052589	-0.005434603	0.08148618	0.247950344
ENSG00000130545	CRB3	37.029764	36.796345	31.848104	25.924479	27.003648	31.043191	38.470051	38.652391	42.941459	0.02398644	-0.256483065	0.012673555	0.248025564
ENSG00000187867	PALM3	0.20635	0.418797	0.626571	0.3024	0.499038	0.284568	0.420491	0.422281	0.538602	--	--	0.637560455	0.2482549
ENSG00000139637	C12orf10	26.243976	25.36362	25.727317	21.475562	23.954787	22.760214	28.62038	29.151186	28.271931	0.794864124	-0.033185008	0.005741424	0.248336442
ENSG00000025293	PHF20	5.508367	5.697667	5.735719	5.079187	4.667315	5.080572	6.643425	5.8481	6.654888	0.67896931	-0.046673733	0.004151927	0.24876726
ENSG00000104936	DMPK	9.113196	8.696259	9.920303	7.490354	7.24974	6.467293	10.139792	8.763916	12.128455	0.017848763	-0.246586283	0.031908089	0.248787587
ENSG00000179832	MROH1	4.186066	4.250743	3.887361	3.83347	3.73467	3.816411	4.350293	4.390463	5.119072	0.946993724	0.010744201	0.021300072	0.248969329
ENSG00000172845	SP3	27.80064	28.394261	24.434177	25.040513	24.975289	25.30296	29.688418	28.283311	31.345365	0.703839571	0.029361289	0.0000728	0.249111749
ENSG00000119820	YIPF4	3.425577	4.218344	3.886806	3.612202	3.33715	3.24446	4.290221	4.157863	4.354365	0.834585282	-0.027195541	0.006155469	0.24923493
ENSG00000143761	ARF1	207.864406	203.470367	199.317343	209.564168	205.721234	210.754741	221.268433	213.589516	242.893101	0.00014015	0.182561813	0.0000123	0.249271449
ENSG00000214967	NPIPA7	4.022532	3.592283	6.232285	4.652414	3.575508	3.457377	5.742655	4.043914	7.108423	0.960142463	-0.017644992	0.275774567	0.249315872
NewGene_2273	--	18.955611	16.326944	16.283901	16.025388	15.354683	17.852774	21.895599	19.516478	19.556835	0.581657825	0.09160201	0.05811976	0.249341382
ENSG00000115884	SDC1	27.981997	28.048397	24.44867	23.531128	24.375754	24.54007	29.215199	28.524956	32.313552	0.926205078	-0.010866222	0.001351105	0.249365499
ENSG00000064933	PMS1	11.062096	11.765784	11.796975	9.8809	9.621559	9.949238	13.135179	11.831	14.018119	0.596780401	-0.060509618	0.005400853	0.24949213
ENSG00000174891	RSRC1	15.270431	19.180095	17.640019	16.751392	16.174398	17.268305	20.674182	18.767107	22.463276	0.360943321	0.101523935	0.009838883	0.249652397
ENSG00000174775	HRAS	14.055809	15.62855	14.517745	12.930165	12.633934	13.613848	15.555933	15.567464	17.561757	0.834756165	-0.034853779	0.034128628	0.249741083
ENSG00000175471	MCTP1	6.671383	6.494657	7.235013	7.154117	5.938966	6.078257	7.66757	7.439545	6.862015	0.376868833	0.115650277	0.022238506	0.249811763
ENSG00000064601	CTSA	160.369032	158.580629	164.449682	178.175094	181.582981	166.773644	174.372802	170.062569	195.48566	0.000000014	0.288792794	0.0000603	0.249881928
ENSG00000092758	COL9A3	37.12381	31.623814	31.564673	13.670989	15.249079	15.10674	34.273322	36.503156	41.226343	2.54E-37	-1.040284188	0.005566277	0.249945688
ENSG00000135749	PCNX2	2.903266	3.313642	3.23168	1.962749	1.623353	1.750437	3.124521	3.003429	4.438476	7.14E-12	-0.771637693	0.029123171	0.249989994

Gene ID	Gene name	Ctrl-1 FPKM	Ctrl-2 FPKM	Ctrl-3 FPKM	IFNγ-1 FPKM	IFNγ-2 FPKM	IFNγ-3 FPKM	Sora-1 FPKM	Sora-2 FPKM	Sora-3 FPKM	Ctrl vs IFNγ DESeq2 FDR	Ctrl vs IFNγ DESeq2 log2FC	Ctrl vs Sora DESeq2 FDR	Ctrl vs Sora DESeq2 log2FC
ENSG00000114026	OGG1	12.690435	12.010984	11.390799	11.021278	10.632221	10.789467	12.763681	13.211041	14.675453	0.891475251	-0.020184902	0.018414409	0.249998743
ENSG00000165264	NDUFB6	26.946618	29.413069	23.349106	19.965916	23.353704	23.990089	28.825351	30.267437	28.877595	0.351963537	-0.113102932	0.011335409	0.250186149
ENSG00000156860	FBRS	10.093558	8.633836	10.16532	10.964677	10.898321	10.829824	11.20334	9.5539	10.270303	0.003356073	0.233148764	0.005507426	0.250215964
ENSG00000148700	ADD3	35.809905	36.718003	35.155816	28.593793	27.809926	31.09753	42.658514	36.402762	42.300562	0.033440161	-0.13410248	0.000121368	0.250259086
ENSG00000203760	CENPW	26.964059	29.550837	27.058639	19.827405	18.245806	17.803152	29.333729	29.914793	23.288845	0.004455881	-0.342102642	0.080490924	0.250282128
NewGene_3492	--	4.456414	4.337951	4.815905	4.745471	4.313725	4.214005	5.349791	4.182684	4.550436	0.029996375	0.231028847	0.02101587	0.250340276
ENSG00000113391	FAM172A	2.190262	2.433287	1.955345	1.658757	1.385898	1.179242	2.445667	2.551343	2.808296	0.01111914	-0.441603755	0.088269764	0.250416865
ENSG00000164548	TRA2A	35.526837	37.043532	41.564033	36.397276	31.798006	32.825669	41.899694	42.715013	44.133872	0.788165344	-0.029607661	0.001859766	0.250613499
ENSG00000163754	GYG1	26.506922	27.817531	26.205169	28.683561	28.004738	27.681157	29.098106	27.757284	32.358608	0.009478471	0.189082483	0.002116256	0.250626926
ENSG00000120662	MTRF1	4.318728	5.375935	5.734112	4.309483	4.306688	3.99968	5.881035	5.307391	5.288213	0.407175392	-0.153892431	0.095409985	0.250774219
ENSG00000135821	GLUL	175.88696	160.744267	146.704205	244.30178	258.746445	243.501982	189.668186	181.754069	180.440433	3.51E-30	0.74557017	0.001295547	0.250886417
ENSG00000169562	GJB1	19.585864	16.966286	16.474447	16.141898	15.691546	15.823194	17.755221	19.08697	21.739224	0.941722654	-0.010767434	0.018420929	0.250962046
ENSG00000115419	GLS	46.262888	53.307222	61.283178	38.069918	40.201772	34.658454	56.355247	57.20025	67.066736	0.0000787	-0.346249565	0.008167997	0.251061004
ENSG00000204520	MICA	18.06057	17.052959	17.71918	17.611362	17.082456	17.155384	19.022486	18.81245	21.965949	0.009590396	0.230436424	0.006610719	0.251101515
ENSG00000149554	CHEK1	14.172263	14.688943	19.488186	12.545932	12.367339	12.869652	17.50916	18.46942	18.701168	0.136397733	-0.141414846	0.003067472	0.251154647
ENSG00000086061	DNAJA1	165.648193	170.65889	181.071121	225.8228	217.87973	223.832016	186.810654	193.451782	197.01268	1.08E-26	0.514919619	0.0000514	0.251218149
ENSG00000145916	RMND5B	10.407156	9.22815	6.137701	8.34327	8.536784	9.580494	8.062108	9.10712	8.175689	0.987497051	0.002865332	0.024606097	0.251233605
ENSG00000137760	ALKBH8	2.868606	2.866217	3.133452	2.646485	2.964577	2.761361	3.651303	2.976052	3.247134	0.910437552	0.023769002	0.065759266	0.251420499
ENSG00000116786	PLEKHM2	9.533553	8.745019	8.753077	7.483458	7.090172	7.845995	10.029726	8.882378	11.255356	0.19604991	-0.12790561	0.007797359	0.251471813
ENSG00000128951	DUT	20.706709	21.389793	20.337818	20.931732	18.580006	18.759956	24.067233	23.313587	25.149216	0.941722654	0.009569442	0.002841291	0.251472918
ENSG00000198034	RPS4X	280.343597	261.179718	248.982941	210.504547	206.879578	205.268295	289.534851	296.308838	296.278961	0.00070535	-0.197238861	0.000137697	0.251620265
ENSG00000162928	PEX13	13.859814	15.002532	15.359373	16.18689	16.919701	16.734813	17.525638	17.076644	18.219283	0.000000507	0.332622711	0.000646837	0.251661761
ENSG00000090581	GNPTG	18.447294	19.14979	18.143733	17.018143	16.88356	16.249712	20.922408	20.6493	20.458596	0.950555755	-0.007900712	0.003061605	0.251785067
ENSG00000151726	ACSL1	5.808953	5.621949	5.444288	4.393344	4.143555	3.937564	5.967851	5.907526	6.846939	0.010055579	-0.278341113	0.014793371	0.251786192
ENSG00000130958	SLC35D2	61.361189	61.192574	63.596042	57.06468	55.944766	60.717099	72.326642	65.94485	75.184236	0.923972107	0.009689282	0.000352533	0.251920239
ENSG00000153044	CENPH	11.325306	12.718224	13.557933	10.732556	10.491942	11.537912	14.664261	13.252637	14.079137	0.745077666	-0.051957734	0.030480492	0.251987157
ENSG00000140941	MAP1LC3B	27.078951	28.594943	31.426076	23.164126	22.459954	23.466365	31.971789	30.514052	35.027574	0.017042457	-0.186154575	0.001667082	0.252012614
ENSG00000174928	C3orf33	3.218376	3.289528	3.639298	2.998792	3.270027	3.273653	3.608854	3.161807	4.392598	0.778685946	0.066331029	0.161887986	0.252055611
ENSG00000173511	VEGFB	41.408487	35.890001	35.566946	40.131647	38.496515	39.448333	42.616078	40.651491	42.774753	0.048957127	0.178900679	0.005036915	0.252083643
ENSG00000203965	EFCAB7	0.960418	0.839966	0.773725	0.801388	0.918773	0.795379	0.852437	0.898785	1.134226	0.777135163	0.113163518	0.417947481	0.252142281
ENSG00000222011	FAM185A	1.667829	1.872877	1.992555	2.047702	1.658334	1.490659	1.890395	1.795822	2.452858	0.839850264	0.07003644	0.283654503	0.252177759
ENSG00000232388	SMIM26	25.984434	25.914804	30.031567	26.586144	26.174532	29.328827	33.066984	32.432127	25.537023	0.241213983	0.159004324	0.089234864	0.252277832
ENSG00000144036	EXOC6B	4.470282	4.451908	4.534748	3.880979	3.535691	3.664275	5.286784	4.983992	4.748779	0.19404995	-0.133200679	0.009838883	0.252347194
ENSG00000065665	SEC61A2	3.342857	3.318962	3.358385	4.097301	3.443567	3.239092	4.206135	3.541969	3.444216	0.150124885	0.255772533	0.144870201	0.252440728
ENSG00000179348	GATA2	8.693227	8.941104	8.910691	7.620541	6.864436	6.972541	10.313458	9.426736	10.452532	0.119888337	-0.159000972	0.006092155	0.252473206
ENSG00000166002	SMCO4	19.157141	18.07902	20.628858	24.276775	24.001334	24.71906	21.540835	23.482799	20.190025	0.000000496	0.459541595	0.039800552	0.252565497
ENSG00000067829	IDH3G	13.713196	10.659096	10.840772	10.597561	8.502393	9.57035	14.2013	11.880452	13.861025	0.340753738	-0.141762257	0.04739668	0.252740499
ENSG00000080293	SCTR	0.548636	0.444321	0.763971	0.351155	0.435513	0.305724	0.559173	0.590448	0.814125	--	--	0.568137629	0.252752417
ENSG00000166311	SMPD1	20.389976	17.77515	16.741233	25.440029	25.59314	25.694804	19.546105	19.173739	20.786436	1.06E-16	0.645130554	0.006085631	0.25283064
NewGene_1196	--	6.74702	4.666808	3.939311	4.314853	3.919365	4.069864	4.226078	6.382221	6.331304	0.681847625	-0.168751077	0.455759893	0.252926211
ENSG00000105707	HPN	89.774672	75.413717	63.617197	70.946537	70.0346	72.861943	84.829285	84.017759	91.197487	0.511307153	0.079218515	0.012881749	0.252956766
ENSG00000285920	AC087721.2	16.236796	10.973836	19.841709	10.508595	13.299026	17.477757	20.575506	18.155451	13.688191	0.946121012	-0.039202677	0.518808895	0.253000581
ENSG00000132604	TERF2	9.949411	9.333053	9.960613	10.379715	10.162698	9.791526	11.45027	9.88096	10.738542	0.042869899	0.192628599	0.007780684	0.253065218
ENSG00000167130	DOLPP1	22.64698	20.820464	21.801915	18.083039	17.937001	18.156196	23.419558	24.461988	25.488503	0.139177055	-0.125165101	0.001610005	0.253347765
ENSG00000159210	SNF8	38.84651	39.796294	53.723368	52.847438	46.762573	50.13864	53.612565	43.069796	65.930121	0.76716022	0.027262524	0.000399214	0.253358732
ENSG00000112473	SLC39A7	142.17733	137.354278	133.058355	149.836498	146.397068	149.361294	149.777981	154.366095	154.837155	1.69E-08	0.270334915	0.0000203	0.253451571
ENSG00000100991	TRPC4AP	37.336795	35.912746	34.501169	38.249402	37.928272	40.426585	38.01183	38.030445	44.509787	0.0000212	0.256473916	0.000337721	0.253486667
ENSG00000145214	DGKQ	7.246956	6.289996	5.850696	7.769683	7.026401	7.546228	5.963684	5.866802	7.003513	0.227348016	0.149055346	0.029270604	0.253747044
ENSG00000138760	SCARB2	44.295847	44.320574	46.404517	38.458976	39.168184	38.884467	48.8249	49.058021	54.558598	0.317269945	-0.064372997	0.0000357	0.253998105
ENSG00000143258	USP21	18.10767	16.64434	19.618793	18.323589	13.668012	14.424903	21.754145	21.291538	23.97961	0.109029647	-0.152685256	0.003019706	0.254027082
ENSG00000063244	U2AF2	87.328916	87.273709	87.403872	78.573445	76.801176	77.525036	96.561606	96.495909	104.332609	0.744943386	-0.023767995	0.0000195	0.254036893
ENSG00000165233	CARD19	2.840088	3.12377	2.202774	2.429749	1.880434	2.851354	2.266909	3.798597	3.892859	0.958729464	-0.021774944	0.416359112	0.254074088
NewGene_4374	--	292.948456	308.822254	337.457794	270.688268	311.353882	305.02713	370.16629	325.583466	361.231261	0.449303072	0.066106172	0.00026731	0.254271788
ENSG00000103091	WDR59	9.691831	10.228644	9.33005	11.728875	11.356345	10.567324	10.656736	10.707481	11.902281	0.000000383	0.339511823	0.000753405	0.254367686
ENSG00000089154	GCN1	34.978477	32.815887	30.19199	32.407917	30.452213	30.954264	33.523819	34.417747	41.89716	0.25260974	0.083995484	0.002204227	0.254515248
ENSG00000137154	RPS6	1060.352511	1003.410869	989.232037	710.928427	737.596842	746.801718	1166.309913	1168.409409	1086.059732	7.18E-11	-0.324638119	0.000204744	0.254702366
ENSG00000100325	ASCC2	18.661937	17.307574	16.568337	18.48597	16.700052	16.600075	19.463445	18.903411	20.225451	0.170993488	0.122539926	0.001486582	0.254806731
ENSG00000171914	TLN2	2.605298	2.465454	2.390285	1.860612	2.122907	2.11248	2.828645	2.74454	3.155551	0.230928016	-0.134186857	0.006524323	0.254845689
ENSG00000003509	NDUFAF7	5.841245	6.542863	6.016203	5.716021	5.522739	5.213273	6.454513	6.029946	7.702251	0.822462446	-0.040306749	0.046031295	0.254896172
ENSG00000134602	STK26	13.197547	14.626589	16.361597	12.034297	13.795633	12.186245	15.243779	16.629669	17.883451	0.574621814	-0.070476403	0.009389972	0.255004355
ENSG00000148300	REXO4	22.011166	22.102894	21.774246	19.135095	20.387912	19.982427	24.807648	22.330815	26.661338	0.958230986	-0.006212099	0.000938665	0.255158173
ENSG00000140464	PML	3.996744	3.859344	3.262516	14.547825	12.085079	15.144611	4.142245	4.04295	4.838861	1.91E-111	2.038165866	0.04875932	0.255214208
ENSG00000136960	ENPP2	40.944644	44.269336	47.656382	30.21543	29.366556	28.699754	47.43047	46.250383	52.017321	5.41E-10	-0.411593514	0.000599203	0.25530058
ENSG00000139192	TAPBPL	1.523789	1.571352	1.256927	13.185153	13.139248	12.831639	1.813	1.477549	1.586501	1.58E-145	3.353520399	0.356622596	0.255311928
ENSG00000014919	COX15	12.469338	12.434436	14.160116	11.373169	10.621908	11.816518	14.216853	13.687413	16.133542	0.593412701	-0.063713376	0.00675786	0.255312685
ENSG00000102030	NAA10	21.84251	19.577025	20.758225	14.588109	16.78178	14.99094	20.318801	21.632817	27.714001	0.419207561	-0.08455349	0.005567285	0.255349267
ENSG00000135476	ESPL1	12.346562	13.566375	12.54616	11.052618	9.728644	11.084596	14.437579	13.257528	15.452331	0.12853989	-0.121567405	0.000360691	0.255777545
ENSG00000183648	NDUFB1	9.125248	11.266998	9.78176	9.613113	10.596198	12.532435	12.556943	12.793718	6.380068	0.123224734	0.350984101	0.374845365	0.255816151
ENSG00000127184	COX7C	142.032445	141.790996	150.801874	126.712061	154.351297	148.524268	179.839797	190.988187	114.866731	0.113262554	0.143931079	0.378792565	0.255882553
NewGene_5983	--	5.621162	7.537436	5.470658	6.838084	5.474141	4.132089	9.011079	5.593512	6.262124	0.242210729	0.17075052	0.052769406	0.256050579
ENSG00000101391	CDK5RAP1	15.425941	13.40008	14.995291	14.215802	12.257959	12.35202	16.146733	15.377563	17.173596	0.937316548	-0.0119363	0.007322461	0.25607148
ENSG00000116711	PLA2G4A	1.205068	0.988577	1.261573	0.290276	0.448831	0.473885	1.0684	1.058393	1.774926	0.00000225	-1.3590604	0.355942759	0.256140529
ENSG00000163637	PRICKLE2	0.955183	0.959154	1.207814	0.700212	0.533831	0.649389	1.07552	1.046557	1.115023	0.002202367	-0.472624543	0.059481795	0.256168052
ENSG00000135677	GNS	29.269253	27.178018	29.709813	23.103224	23.357671	23.046618	31.150226	30.651605	34.769342	0.010686128	-0.161724029	0.000153594	0.256193151

Gene ID	Gene name	Ctrl-1 FPKM	Ctrl-2 FPKM	Ctrl-3 FPKM	IFNγ-1 FPKM	IFNγ-2 FPKM	IFNγ-3 FPKM	Sora-1 FPKM	Sora-2 FPKM	Sora-3 FPKM	Ctrl vs IFNγ DESeq2 FDR	Ctrl vs IFNγ DESeq2 log2FC	Ctrl vs Sora DESeq2 FDR	Ctrl vs Sora DESeq2 log2FC
ENSG00000151746	BICD1	3.567077	3.68209	4.041396	2.740854	2.588594	2.170618	4.534038	4.538581	5.583269	0.000103637	-0.508411014	0.028405653	0.256198333
ENSG00000152078	TMEM56	7.787933	9.646013	10.233999	10.667632	10.537313	10.472275	10.193145	9.518372	11.417424	0.000156157	0.346767177	0.012210405	0.25622807
ENSG00000135318	NT5E	30.672004	27.329657	27.041409	23.391513	26.180085	23.898771	31.111438	32.677318	32.122448	0.472564888	-0.069378649	0.000803075	0.256244124
ENSG00000169919	GUSB	40.865668	40.104149	40.330721	38.130781	35.707102	36.5259	43.241597	42.781066	49.50552	0.843662595	0.017117424	0.000195952	0.256276459
ENSG00000084733	RAB10	26.711309	27.273487	27.046776	28.399103	26.388126	28.390732	31.4821	28.006477	31.345661	0.002357354	0.185008303	0.000110493	0.256295579
ENSG00000106052	TAX1BP1	77.014749	77.329891	85.286097	81.40863	77.350127	74.914091	84.184533	84.327316	95.610609	0.04016686	0.120528638	0.0000159	0.256314731
ENSG00000137767	SQOR	15.946115	15.438232	14.880772	46.400175	40.306836	44.631861	18.296489	16.757818	16.71554	7.38E-128	1.653057541	0.006161647	0.256341815
ENSG00000172037	LAMB2	14.774632	14.309919	12.700688	13.296971	12.811166	13.530045	14.492911	14.630862	17.779421	0.43631722	0.070971058	0.004013544	0.256485611
ENSG00000266967	AARSD1	26.943604	28.064877	26.078615	19.642905	21.274883	18.59411	31.362507	27.59632	33.467104	0.002063625	-0.305272679	0.003867784	0.256536487
ENSG00000100439	ABHD4	3.871751	4.084991	3.748517	4.556812	3.614008	3.594389	4.324367	4.720737	4.034765	0.414398291	0.124602253	0.052708119	0.25658063
ENSG00000106804	C5	37.752491	39.107246	35.927582	47.819344	47.100735	46.17207	41.645103	41.692242	43.004322	1.64E-20	0.47021153	0.0000284	0.256608633
ENSG00000095139	ARCN1	42.456175	45.229449	46.259544	49.187308	45.818686	48.437066	50.341755	45.841616	53.959802	0.00000577	0.255312505	0.0000851	0.25663802
ENSG00000097021	ACOT7	14.291614	14.276041	15.548124	21.325398	22.803207	22.303102	16.19637	17.026055	18.046464	4.45E-15	0.620158903	0.006661069	0.256778084
ENSG00000164904	ALDH7A1	16.290327	21.594791	15.710017	15.072611	14.895239	14.293976	28.05303	18.72178	19.463052	0.980706027	0.002556706	0.000242405	0.256942562
ENSG00000138036	DYNC2LI1	5.289993	5.291042	6.801242	4.560888	5.459606	4.777183	6.805179	6.661167	5.746565	0.741446036	-0.078814628	0.153653075	0.257001246
ENSG00000111679	PTPN6	10.159384	9.252435	9.433225	8.599033	9.552746	9.006743	10.291083	10.952084	10.953242	0.656879312	0.062460414	0.013681745	0.257019132
ENSG00000142655	PEX14	8.408368	8.203641	8.024136	7.934738	7.917335	7.732152	9.451402	9.123425	8.939386	0.47167166	0.095820251	0.021349424	0.257061094
ENSG00000163607	GTPBP8	11.294622	12.29639	11.228607	10.194109	9.144876	9.0774	13.691096	12.322609	13.020994	0.43355889	-0.108145238	0.021692647	0.257281268
ENSG00000149932	TMEM219	20.071094	21.207813	19.947363	20.45318	21.378705	23.359511	22.884691	21.030512	24.654252	0.00093164	0.318830387	0.009958616	0.257388572
ENSG00000116337	AMPD2	10.057825	9.287509	9.981037	9.32673	8.34064	10.023804	10.059801	10.394806	12.635295	0.695091178	0.048893781	0.007590284	0.257393586
NewGene_1454	--	4.482121	5.505724	4.478575	5.016059	5.348412	4.838447	6.162185	5.950359	7.121243	0.029967238	0.307449126	0.083479939	0.257501699
ENSG00000100982	PCIF1	14.605744	13.850828	14.422026	11.951872	11.854329	12.227894	16.051296	14.685212	17.454169	0.241268123	-0.106997481	0.002240434	0.257533518
ENSG00000168528	SERINC2	55.268901	47.716486	45.426476	47.900573	48.282316	47.888477	53.920276	55.177122	56.872002	0.242258958	0.106341873	0.002141564	0.25755693
NewGene_1230	--	0.412038	0.468187	0.358945	0.591868	0.453766	0.385889	0.485085	0.573689	0.298042	0.368746001	0.369253022	0.541592582	0.257692142
ENSG00000066044	ELAVL1	23.82023	24.678471	26.431322	21.347975	20.780675	21.548265	29.093869	27.023071	29.934652	0.162792002	-0.097901676	0.00012144	0.257829258
ENSG00000146085	MUT	15.081923	15.83707	14.350451	14.641737	15.399067	14.666028	16.8617	16.905136	16.978079	0.110937157	0.129488746	0.000765497	0.257974842
ENSG00000134548	SPX	2.019918	1.592421	1.654738	0.750706	0.918626	0.867136	1.893929	1.583896	2.166959	0.000358548	-0.881330452	0.251929501	0.257999771
ENSG00000065054	SLC9A3R2	31.205742	27.574619	30.358809	27.070994	26.205144	26.51337	31.048341	32.252366	36.530778	0.856829401	0.019089748	0.000976575	0.258039823
ENSG00000039523	RIPOR1	10.943986	11.682584	11.545935	7.119841	6.520689	6.688738	11.9552	12.140472	13.923657	9.8E-16	-0.588955757	0.000963104	0.258116576
ENSG00000172922	RNASEH2C	17.672783	18.18388	16.753312	13.684367	14.418246	13.804633	19.77382	20.456603	20.790648	0.007795268	-0.210027658	0.000830287	0.258143821
ENSG00000185883	ATP6V0C	71.463309	73.134767	71.847511	62.457723	64.528716	68.778887	77.678596	80.629367	84.130452	0.999202724	0.000169859	0.000110782	0.258214034
ENSG00000144895	EIF2A	63.458746	64.817905	66.108148	59.969519	57.565714	57.641887	72.108854	72.313953	71.967183	0.902378968	0.009969363	0.0000282	0.258224218
ENSG00000248333	CDK11B	35.823768	31.75267	36.623453	32.752645	30.401759	33.631473	36.74405	38.175841	42.115823	0.589442996	0.050986938	0.000780032	0.258295889
ENSG00000285437	POLR2J3	0.470344	1.758824	1.476824	2.069728	1.247049	1.052195	2.047832	1.01458	0.917437	0.049060028	0.582660853	0.396740104	0.258448335
ENSG00000188428	BLOC1S5	23.869822	23.313121	22.281459	20.407754	17.880993	19.144055	26.155537	24.716532	27.093733	0.166328965	-0.115795426	0.000359379	0.258603846
ENSG00000205436	EXOC3L4	2.260689	2.802644	2.638951	4.938092	4.496541	4.235715	3.601035	2.561047	2.76511	8.93E-10	0.952132241	0.238348683	0.25867968
ENSG00000168653	NDUFS5	154.589972	164.149635	155.046078	121.499827	138.219166	127.421507	175.635275	203.506314	150.111605	0.077479631	-0.142819882	0.022878691	0.258760237
ENSG00000153815	CMIP	6.622899	5.903014	6.32181	6.299527	6.361698	6.156133	6.556875	6.514185	8.302116	0.153838545	0.14436718	0.017195632	0.258910007
ENSG00000250021	C15orf38-AP3S2	1.865	1.734585	1.376754	1.411431	2.312124	2.119303	1.71824	2.236514	1.610942	0.145499929	0.379064694	0.292845734	0.258912291
ENSG00000140688	C16orf58	16.274986	15.673529	13.251103	14.36693	15.006306	15.326638	16.561527	17.471849	16.676876	0.228220163	0.128038794	0.00905173	0.258952828
ENSG00000196547	MAN2A2	13.571286	14.080363	13.445752	12.508543	10.670493	11.72362	13.593325	14.796385	16.953916	0.625091318	-0.043012547	0.000718939	0.258989541
ENSG00000213614	HEXA	45.712825	38.304456	40.041332	38.28728	32.643483	42.074694	43.059076	45.77132	47.268297	0.464621682	0.059765913	0.000109498	0.258990293
ENSG00000163913	IFT122	3.906736	4.12203	3.688295	3.009052	3.035155	2.771535	4.056669	4.460716	4.630373	0.027678984	-0.275341507	0.029612391	0.259109029
ENSG00000112234	FBXL4	3.009714	2.962708	2.882449	3.085616	3.014693	3.244871	3.60369	3.167891	3.700884	0.075974526	0.179531576	0.007285702	0.259142448
ENSG00000068400	GRIPAP1	7.770953	7.271187	7.701144	8.417132	7.837986	7.871746	8.705914	7.7995	9.136231	0.021543948	0.224841466	0.009228149	0.259392823
ENSG00000172340	SUCLG2	27.183324	31.603621	28.812353	30.463755	28.895363	28.786362	32.801565	32.500966	32.906651	0.057384816	0.150945133	0.001079177	0.259428403
ENSG00000165487	MICU2	13.638466	13.970582	14.630933	14.00093	13.816298	13.109275	15.992977	15.633205	15.744876	0.333889598	0.101604027	0.004682262	0.259530526
ENSG00000117419	ERI3	18.716363	17.569558	18.013693	14.118363	13.322787	14.678683	20.211446	19.980938	22.264494	0.015725959	-0.223773789	0.003050933	0.259743464
ENSG00000099800	TIMM13	56.81299	54.481983	53.839805	41.360461	45.975609	49.207631	61.154246	64.182722	60.234857	0.128004269	-0.128186497	0.00030864	0.259760513
ENSG00000099785	2-Mar	4.969808	5.948575	4.866676	5.301115	4.764851	4.103812	6.413328	5.832628	6.355484	0.800677603	-0.057913596	0.098293429	0.259765966
ENSG00000103653	CSK	30.973784	30.803842	29.898833	31.512095	29.468205	31.157289	32.729703	31.582506	36.344878	0.013683399	0.159996319	0.000488244	0.25986577
ENSG00000118960	HS1BP3	9.540731	9.09471	7.332942	6.103962	6.033965	7.294579	8.222	8.931055	8.460232	0.165383069	-0.209040876	0.055633882	0.259891822
ENSG00000119421	NDUFA8	30.213724	29.139774	26.692259	24.786333	25.245823	23.7638	31.834822	35.21933	29.346758	0.547964042	-0.075469304	0.026428773	0.259911826
ENSG00000183655	KLHL25	2.809551	2.530411	2.254589	2.20303	2.049513	2.135702	2.872557	3.000149	2.649614	0.589073899	-0.102951232	0.092817556	0.260209342
ENSG00000243660	ZNF487	0.306901	0.493866	0.500909	0.540575	0.657864	0.391207	0.353636	0.493319	0.570781	0.430136587	0.405343609	0.597911862	0.26024664
ENSG00000196961	AP2A1	23.917747	21.146786	20.905983	22.800919	22.843731	24.372728	24.178814	22.114008	28.064983	0.00242499	0.233088181	0.003274015	0.260291177
ENSG00000161896	IP6K3	2.036289	1.793442	1.760933	1.680313	1.46999	1.315971	2.577268	2.112934	1.816958	0.33353585	-0.231525393	0.241333079	0.260351576
ENSG00000110011	DNAJC4	15.576189	12.429802	11.584535	13.520316	11.365414	11.263405	13.961793	16.470095	14.085057	0.975935104	-0.007176735	0.085504649	0.260453159
ENSG00000204682	CASC10	0.255858	0.262704	0.251901	0.313256	0.390447	0.211938	0.281524	0.388656	0.182239	0.400379137	0.403369743	0.588727021	0.260478688
ENSG00000183569	SERHL2	2.243517	1.27045	1.808582	1.377559	1.124413	1.881285	1.225302	1.359156	1.132157	0.793623764	0.13949317	0.532154349	0.260484243
NewGene_5719	--	3.029425	3.109106	3.055046	2.039477	1.85272	2.075413	3.812551	3.328233	3.190007	0.008804126	-0.474806841	0.134073656	0.260508587
ENSG00000186470	BTN3A2	4.055	4.000518	5.022705	14.734779	17.310343	15.299197	5.36054	5.120703	4.799062	4.03E-142	2.048392265	0.047841078	0.260964536
ENSG00000170043	TRAPPC1	67.851611	68.461837	63.533203	60.878844	60.302342	60.597557	70.19758	78.458719	76.055041	0.863942345	0.018267441	0.002783157	0.261034769
ENSG00000137522	RNF121	7.967672	7.159387	7.327734	7.262697	7.69766	8.487277	7.889727	8.343447	9.001632	0.126748223	0.191974885	0.019825034	0.261042513
ENSG00000133884	DPF2	20.714877	19.419256	19.600388	19.363331	18.301162	19.414474	22.085268	21.319224	22.625291	0.476730089	0.063622842	0.000210161	0.261355503
ENSG00000073350	LLGL2	34.661411	33.99978	28.828171	27.091193	31.176781	31.388525	35.766475	38.565732	44.124764	0.70932612	-0.039336379	0.001723411	0.261798756
ENSG00000140612	SEC11A	87.181706	78.427859	83.301604	77.176975	79.591053	77.256088	95.392909	97.849737	96.224865	0.668273969	-0.033876264	0.00011134	0.261829028
ENSG00000143363	PRUNE1	13.571878	14.030554	12.489746	17.017508	15.724069	16.52944	14.949071	14.470115	14.849703	8.6E-09	0.433562031	0.001892335	0.261939315
ENSG00000121410	A1BG	9.403142	6.084043	4.537181	8.516496	8.90921	8.930737	6.7304	7.439538	8.118482	0.166611614	0.543906234	0.501374845	0.262069237
ENSG00000172915	NBEA	0.295991	0.275165	0.275068	0.20287	0.274664	0.218035	0.3496	0.283392	0.351615	0.747482861	-0.108618214	0.279397303	0.262114694
ENSG00000261052	SULT1A3	8.111156	8.428583	9.200599	8.614914	6.861438	7.246163	10.427351	9.764795	10.084406	0.648680892	-0.087515157	0.059055395	0.262114786
ENSG00000168096	ANKS3	5.33607	5.845433	5.297968	4.136322	4.153015	4.624177	6.65435	5.440586	6.284018	0.131526291	-0.217646322	0.043888532	0.262170758
ENSG00000142484	TM4SF5	27.566431	21.828114	21.791065	28.970438	32.466427	31.773214	27.93166	25.014687	27.210428	0.00000529	0.535840436	0.041104868	0.262193931
ENSG00000100359	SGSM3	7.969413	8.309112	7.667857	5.952099	5.392105	5.983954	7.500912	7.875102	10.271492	0.003967132	-0.310318009	0.034259156	0.262241233

Gene ID	Gene name	Ctrl-1 FPKM	Ctrl-2 FPKM	Ctrl-3 FPKM	IFNγ-1 FPKM	IFNγ-2 FPKM	IFNγ-3 FPKM	Sora-1 FPKM	Sora-2 FPKM	Sora-3 FPKM	Ctrl vs IFNγ DESeq2 FDR	Ctrl vs IFNγ DESeq2 log2FC	Ctrl vs Sora DESeq2 FDR	Ctrl vs Sora DESeq2 log2FC
ENSG00000108061	SHOC2	6.556645	6.388991	6.673223	7.673191	7.641098	7.63954	7.600137	7.199329	7.262344	0.00000809	0.367405187	0.004818477	0.262248721
ENSG00000084072	PPIE	14.150775	12.848863	13.472613	11.395195	9.997355	11.433065	16.145746	14.768722	14.815437	0.207604934	-0.159941468	0.0139557	0.262249523
ENSG00000143612	C1orf43	165.798656	160.420912	163.101884	130.356578	137.39194	138.003168	170.48978	177.393885	173.628787	0.045303227	-0.115712328	0.00000701	0.262260322
ENSG00000125843	AP5S1	2.885116	2.261459	2.260343	2.885467	3.816881	3.385969	2.921166	3.041466	3.140468	0.005112678	0.399301647	0.063409814	0.262281009
ENSG00000134955	SLC37A2	0.543561	0.276225	0.316665	0.233472	0.19192	0.157671	0.487543	0.415203	0.37095	--	--	0.559639634	0.262419383
ENSG00000175106	TVP23C	2.55146	3.783733	3.049488	2.400458	3.72623	3.711219	3.244173	3.567889	2.823198	0.319879873	0.252294024	0.289360055	0.262461322
NewGene_4388	--	0.37621	0.356235	0.262971	0.524502	0.307953	0.300347	0.447099	0.361864	0.300606	--	--	0.582773031	0.262544866
NewGene_2958	--	0.559215	0.386677	0.498848	0.705562	0.532119	0.325567	0.319612	0.559035	0.704884	0.701359354	0.216638294	0.579299476	0.262592602
NewGene_6463	--	0.908039	0.857677	0.856242	1.279508	1.416035	1.406275	0.887387	0.947227	1.209335	0.000326755	0.785076994	0.319141935	0.262616112
ENSG00000158874	APOA2	7857.874738	7279.54939	6959.917095	9083.628729	9513.207979	9322.316525	8218.477983	10046.39282	6501.1273	4.06E-19	0.4853991	0.310428013	0.262651422
ENSG00000169696	ASPSCR1	17.196539	15.456652	14.839859	13.731438	13.025255	13.790313	17.371156	19.008926	19.237623	0.338848526	-0.112039189	0.009509005	0.262941716
ENSG00000188529	SRSF10	77.47957	75.969326	81.089005	64.407761	59.33392	62.376606	89.137367	81.448602	90.178891	0.087023145	-0.107455158	0.0000226	0.263074997
ENSG00000149262	INTS4	14.356868	13.490674	13.939115	13.494801	13.741832	13.778599	15.643312	17.091981	15.309836	0.526388098	0.062396914	0.003161119	0.263161579
ENSG00000197461	PDGFA	10.185691	8.475662	8.29544	6.07537	5.46596	5.397682	10.944397	10.129104	8.469745	0.000000688	-0.575731966	0.057503135	0.263188358
ENSG00000147010	SH3KBP1	5.490021	5.437512	5.720999	4.16092	4.441403	3.71167	5.758181	6.057055	6.806451	0.008494616	-0.277568612	0.00701316	0.263194697
ENSG00000136854	STXBP1	0.55511	0.704239	0.528572	0.795741	0.818901	0.703401	0.687329	0.738219	0.662723	0.072992498	0.493907681	0.353069295	0.263214421
ENSG00000171763	SPATA5L1	8.377554	9.274537	10.085484	9.508485	8.738461	10.042607	11.004161	10.923958	10.674427	0.071024365	0.19847901	0.010715052	0.263248264
ENSG00000143222	UFC1	76.016609	75.500656	73.168663	62.208576	62.518799	62.430817	81.548286	88.06916	82.932556	0.089397931	-0.116100187	0.000486006	0.263263158
ENSG00000115350	POLE4	17.18799	19.282499	15.862933	14.302786	13.396693	14.795744	19.358665	19.669035	19.859291	0.22497337	-0.151850327	0.015099021	0.263356491
ENSG00000164808	SPIDR	5.486845	7.339956	6.408272	6.475122	6.674735	6.117518	6.305175	5.549086	6.824289	0.144068746	0.160069482	0.013393162	0.263498443
ENSG00000106333	PCOLCE	1.172289	0.711812	0.527865	0.946851	0.658279	0.903505	0.961886	0.593959	1.164182	0.685556873	0.208952641	0.555719635	0.263596504
ENSG00000185189	NRBP2	1.028485	0.939055	1.144643	1.257891	1.076515	0.950352	1.183494	1.076684	1.27926	0.508019345	0.178580574	0.234052533	0.263688209
ENSG00000176248	ANAPC2	13.651958	12.404943	11.893677	12.489477	12.008405	12.275437	13.098709	14.204679	15.473191	0.326915083	0.101100828	0.006577175	0.263924327
ENSG00000018510	AGPS	13.730727	15.270589	16.300144	12.702824	12.956304	12.195431	16.505752	16.477436	18.348079	0.140683123	-0.119992129	0.000682409	0.263969865
ENSG00000184182	UBE2F	26.524761	30.000508	26.907499	22.478262	22.381184	21.141859	26.578214	28.257421	25.274053	0.697572728	0.041594611	0.002490067	0.26418547
ENSG00000182117	NOP10	133.630905	130.701218	123.779633	104.517166	106.452835	106.859947	140.573212	164.600693	130.468353	0.055542868	-0.140492009	0.01139203	0.264264512
NewGene_4177	--	0.585236	0.987538	0.730608	1.257691	1.242742	0.323688	0.888999	0.849851	0.958493	0.467011414	0.447157995	0.572219719	0.264733985
ENSG00000185432	METTL7A	9.896676	10.313863	8.529132	10.618952	11.794711	11.133288	11.783194	10.282596	10.576641	0.00057642	0.343435359	0.011438129	0.264969045
ENSG00000162927	PUS10	5.095712	4.884534	4.704605	6.055663	6.687529	6.162498	6.190412	5.619192	5.664767	0.00031134	0.471170026	0.057758323	0.26502423
ENSG00000125691	RPL23	194.144233	180.079346	183.976657	146.750251	149.970123	155.179983	220.256312	244.767864	161.054588	0.000351642	-0.189481322	0.191997635	0.265042602
ENSG00000159788	RGS12	8.040596	6.98904	7.217809	6.254973	6.155371	6.201746	8.327143	8.511212	8.306352	0.181834502	-0.140709207	0.005260789	0.265070247
ENSG00000182173	TSEN54	14.599423	14.280478	15.483557	12.148293	13.500859	12.723006	16.773029	16.360562	17.694193	0.528980803	-0.075690508	0.003692562	0.265092014
ENSG00000196369	SRGAP2B	3.196153	2.808082	3.18	2.741649	3.137984	2.975117	3.342503	3.368974	6.370611	0.606710225	0.070038597	0.010385952	0.265102241
ENSG00000119705	SLIRP	33.333009	33.883202	37.20514	39.641203	43.19754	48.785855	43.845611	52.477176	22.956907	0.000074	0.452469496	0.572158219	0.26512929
ENSG00000223501	VPS52	45.902061	43.457233	44.342152	40.297555	38.204175	37.561296	49.795863	49.107822	52.485468	0.298362157	-0.071451186	0.0000141	0.265386325
ENSG00000149091	DGKZ	16.850361	16.132164	16.225181	14.453287	14.295549	15.308254	17.707536	16.395536	20.864679	0.837221361	0.021860014	0.002724311	0.265702459
ENSG00000102158	MAGT1	29.1341	31.107683	26.934004	36.939171	34.703409	37.681507	33.740319	32.063222	35.291721	4.93E-13	0.444178203	0.000066	0.265788645
ENSG00000161542	PRPSAP1	20.448094	22.764869	23.368506	24.144949	26.335849	24.224297	28.055592	24.245705	24.382178	1.75E-08	0.341694092	0.000180318	0.265864673
ENSG00000133112	TPT1	155.399313	135.111037	163.127099	149.069203	126.262899	136.140876	194.489201	184.601487	149.202051	0.772162563	-0.023847509	0.000221145	0.265876448
ENSG00000160688	FLAD1	41.922667	41.025434	37.517752	34.617852	34.236039	32.028842	44.244386	47.858411	46.458926	0.583345778	-0.048436339	0.000643616	0.265897687
ENSG00000170270	GON7	4.68602	5.720435	4.988657	4.443223	5.260613	4.95827	5.63458	5.262354	6.463146	0.741254352	0.080961969	0.141369559	0.265918697
ENSG00000101782	RIOK3	8.277742	10.223606	10.136644	11.402851	10.488528	10.198158	10.92128	10.172306	11.031591	0.000795309	0.314291182	0.006326718	0.266186366
ENSG00000122678	POLM	8.724963	7.548859	8.535684	9.123158	8.213167	8.794534	8.816321	8.947666	9.520773	0.032661366	0.214285637	0.007727623	0.266236309
ENSG00000141985	SH3GL1	69.824642	65.591234	62.383362	65.086877	64.909321	65.41184	72.001119	73.684006	76.483378	0.050294072	0.12935657	0.0000711	0.266336318
ENSG00000143013	LMO4	5.745305	6.648945	5.153578	8.198833	8.239222	6.26282	5.543313	6.136627	4.308094	1.5E-10	0.584130768	0.019972132	0.266387531
ENSG00000173040	EVC2	1.538464	1.539164	1.584565	1.403638	1.300846	1.245449	1.650413	1.771471	1.835076	0.574667169	-0.113698039	0.086886951	0.266413882
ENSG00000180210	F2	142.065405	127.403317	113.184436	105.181812	111.823108	110.467816	143.345408	139.651318	148.348714	0.424171694	-0.076636907	0.000854352	0.266475374
ENSG00000127463	EMC1	14.588702	13.719959	14.327982	13.088928	11.386083	11.863681	16.411382	15.938362	17.532963	0.150274104	-0.109634211	0.000184638	0.266727969
ENSG00000160410	SHKBP1	22.132007	21.78585	21.757242	19.053246	20.379904	20.267429	24.080416	23.174128	28.47348	0.856107824	-0.019284929	0.001100766	0.266821308
ENSG00000042493	CAPG	28.430456	28.149996	23.246618	17.88679	18.587731	16.786905	28.560002	29.008736	31.844767	0.00000277	-0.47489881	0.008676613	0.266898894
ENSG00000197498	RPF2	20.279925	18.620595	19.104814	18.478191	16.982123	18.317463	18.042311	19.384138	24.481128	0.957795774	0.007254584	0.00257067	0.266915145
ENSG00000032389	EIPR1	18.714988	16.466885	17.033607	15.31777	15.857339	14.758312	18.230687	18.672278	22.130008	0.828161465	-0.028575918	0.008270673	0.266966864
ENSG00000040341	STAU2	16.022938	18.054002	15.747465	14.505552	15.213759	14.669218	19.331789	18.767678	20.507689	0.616131418	-0.050065184	0.000433686	0.266971861
ENSG00000126456	IRF3	30.557863	29.631377	27.107046	33.271647	29.823138	33.564928	31.887224	33.206011	36.263594	0.002864174	0.220495556	0.000487326	0.267002021
NewGene_3446	--	5.824999	6.240869	6.514089	7.313222	6.910094	6.549148	6.710803	6.552373	6.619088	0.000905541	0.298613347	0.007318124	0.267003112
ENSG00000157020	SEC13	95.926429	91.706413	92.629477	97.074003	93.736169	98.770745	103.228762	102.356768	110.698191	0.000183616	0.209366918	0.0000102	0.267091526
ENSG00000204070	SYS1	13.971458	13.695772	12.757714	9.743029	9.337814	11.160902	14.446103	15.911906	15.458859	0.001657006	-0.292764179	0.00434055	0.267273025
ENSG00000228300	C19orf24	61.520096	57.663479	51.683974	50.292499	54.94523	58.755285	60.041939	66.210033	66.275684	0.452750368	0.084884295	0.002900976	0.26728125
ENSG00000177917	ARL6IP6	13.731662	13.929944	15.231552	13.059077	12.994431	13.398711	15.642602	15.232202	16.689203	0.656037012	0.049077596	0.00159917	0.267366555
ENSG00000178950	GAK	27.800175	26.646755	25.986062	28.227075	26.190607	28.582994	28.468365	27.430575	35.444664	0.002895672	0.191614647	0.001505519	0.267564894
ENSG00000104823	ECH1	55.007534	47.973951	45.324474	60.160448	60.288852	59.026172	54.713967	55.308417	56.458724	2.31E-08	0.425705563	0.001971551	0.267650162
ENSG00000089775	ZBTB25	0.443054	0.695151	0.634916	0.425443	0.468032	0.5018	0.650872	0.415233	0.737774	0.784458984	-0.086657277	0.31397817	0.267674189
ENSG00000137409	MTCH1	120.863422	120.265966	114.275913	102.602094	99.054769	101.421502	127.6358	131.425648	140.243347	0.091799772	-0.09803635	0.0000086	0.267807267
ENSG00000106397	PLOD3	34.5511	33.681322	32.99608	28.697485	26.612014	26.692248	38.208807	35.981615	40.301425	0.005397611	-0.178051258	0.0000229	0.268004696
ENSG00000171759	PAH	157.368471	156.673661	150.035862	131.995935	143.309951	132.074252	178.762152	174.187372	178.428424	0.47654295	-0.053036142	0.00000111	0.268125312
ENSG00000138050	THUMPD2	9.528503	11.267249	11.198633	9.90532	9.30282	9.715843	13.158267	11.424264	11.519827	0.889945453	0.02181577	0.014978611	0.268133095
ENSG00000136943	CTSV	6.496568	7.74499	7.119039	4.802704	4.319303	5.331508	8.642658	8.666047	9.332045	0.00034745	-0.445186958	0.026242667	0.268144023
ENSG00000147140	NONO	189.914733	182.259725	179.653364	169.170784	166.592831	157.18321	209.920649	199.696237	216.506221	0.570948405	-0.036306147	0.000000502	0.268145625
ENSG00000187514	PTMA	447.734323	496.791237	519.199325	603.297096	592.583537	585.611213	521.423507	517.161499	543.265843	1.48E-18	0.404350008	0.00000246	0.268202257
ENSG00000111602	TIMELESS	33.820527	32.291626	31.789522	30.691561	28.587156	29.413658	37.586386	35.641842	41.870109	0.992844663	-0.000896561	0.0000301	0.268219152
ENSG00000153132	CLGN	14.824356	14.587918	14.598991	11.89123	12.32676	12.294819	16.848057	16.566411	16.169392	0.17064769	-0.116900646	0.000713184	0.268342272
ENSG00000164877	MICALL2	2.9972	3.261272	2.898242	3.944037	3.30521	3.745951	3.47888	3.270612	3.605548	0.002320159	0.410821851	0.052076446	0.268572282
ENSG00000125730	C3	288.114261	278.860873	272.827398	795.664082	752.427081	758.107345	310.008185	295.326307	345.481954	0	1.602763196	0.0000046	0.268629299
ENSG00000160961	ZNF333	2.402162	2.45795	2.331952	2.256181	2.372563	2.345882	2.549733	2.4359	2.59099	0.166277491	0.216417465	0.063169225	0.268638093

Gene ID	Gene name	Ctrl-1_FPKM	Ctrl-2_FPKM	Ctrl-3_FPKM	IFNγ-1_FPKM	IFNγ-2_FPKM	IFNγ-3_FPKM	Sora-1_FPKM	Sora-2_FPKM	Sora-3_FPKM	Ctrl_vs_IFNγ_DESeq2_FDR	Ctrl_vs_IFNγ_DESeq2_log2FC	Ctrl_vs_Sora_DESeq2_FDR	Ctrl_vs_Sora_DESeq2_log2FC
[truncated: 1,763,297 more chars]
