# Supplementary material for: IFNγ augments TKI efficacy by alleviating protein unfolding stress to promote GSDME-mediated pyroptosis in hepatocellular carcinoma
Source: Cell Death Dis. 2025 Jul 11;16(1):512. doi: 10.1038/s41419-025-07839-y (PMC12254303; doi:10.1038/s41419-025-07839-y)

Figure 2e

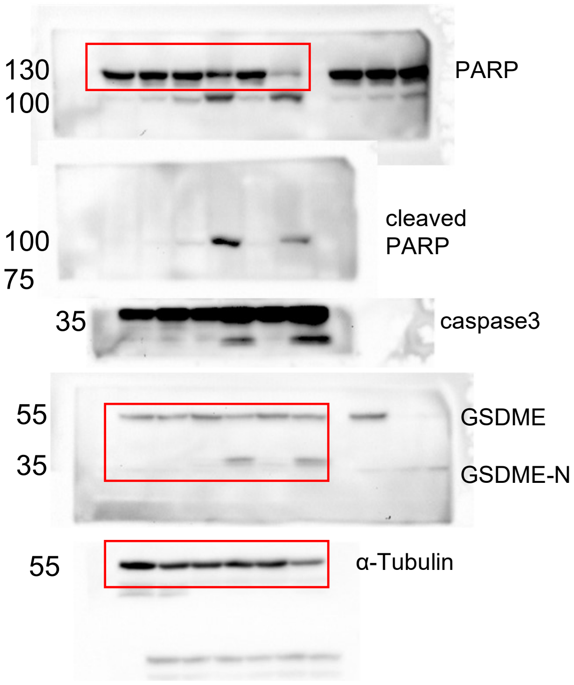

Figure 2f

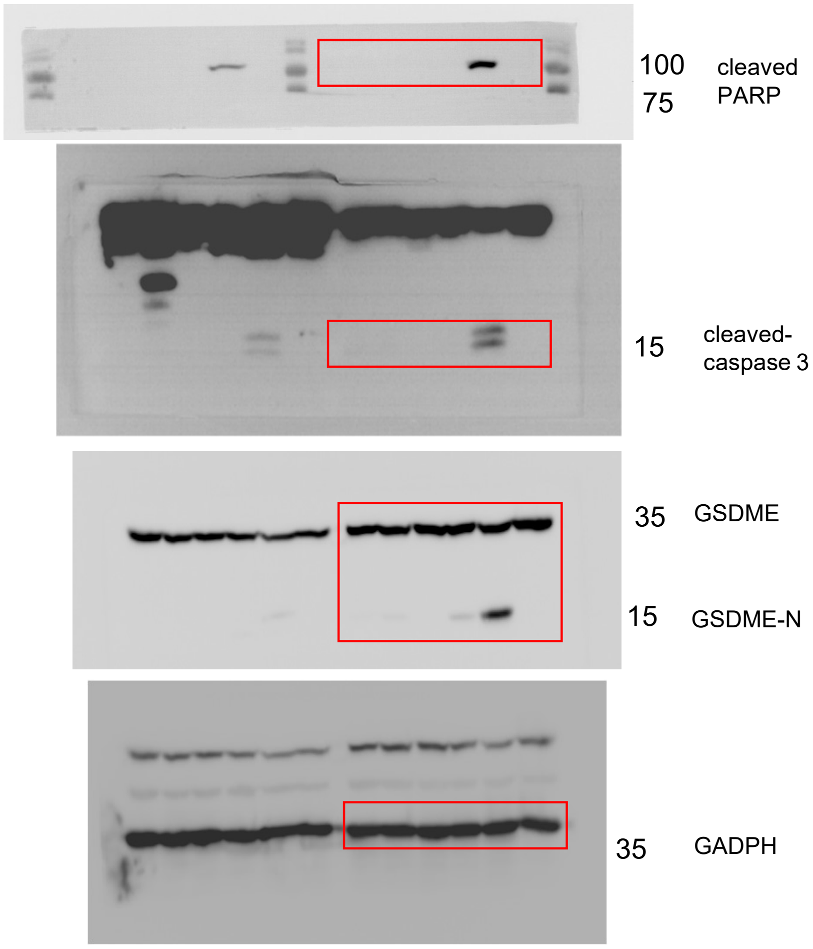

Figure 2i

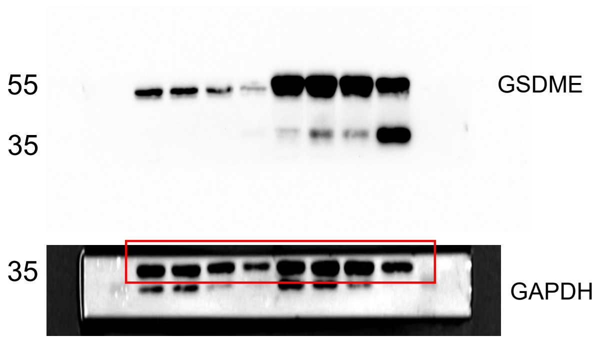

Figure 3c

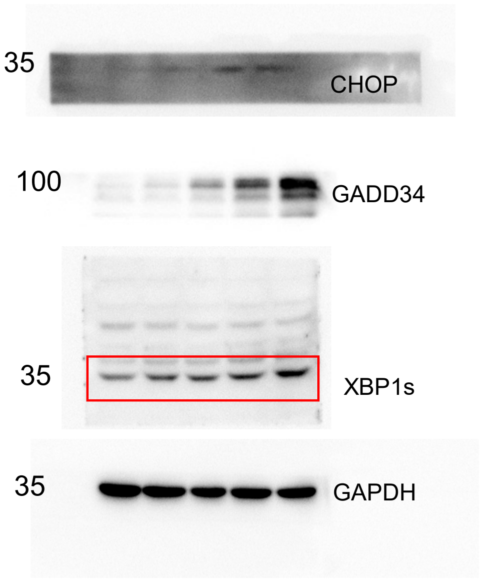

Figure 3f

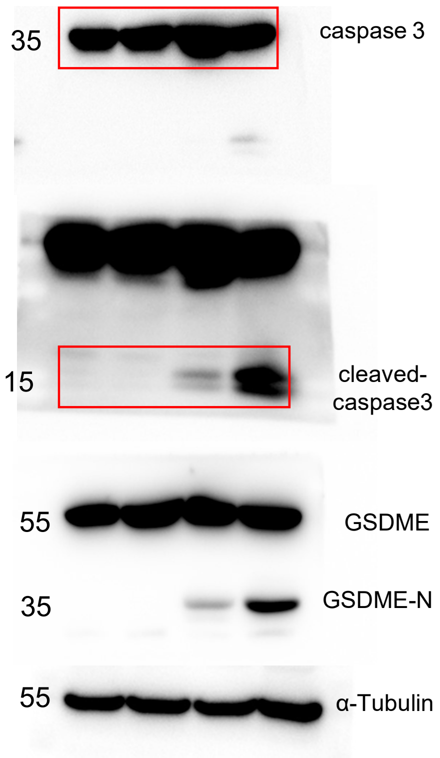

Figure 3h

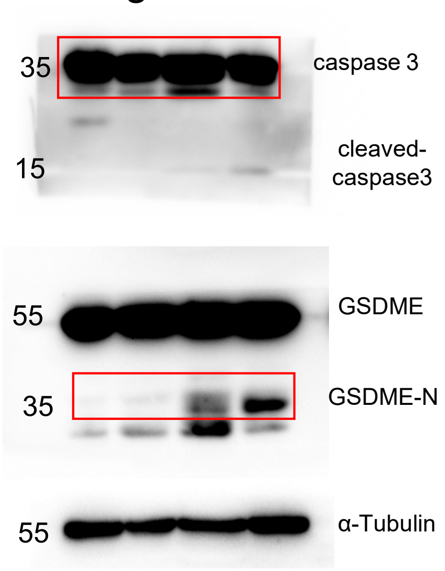

Figure 4c

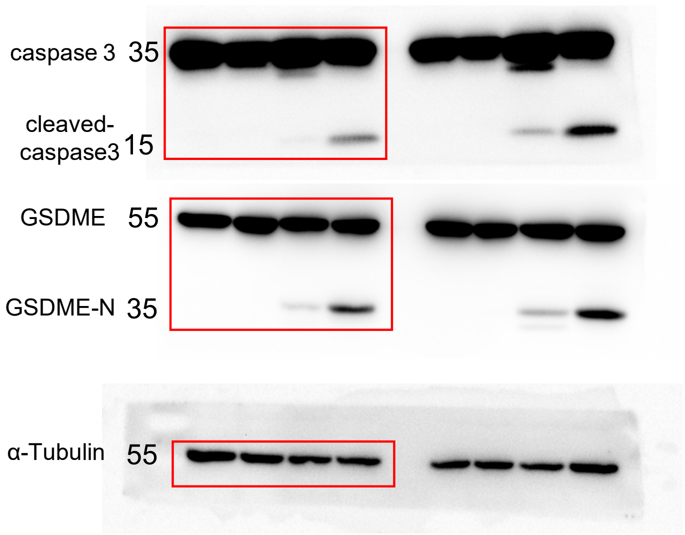

Figure 4e

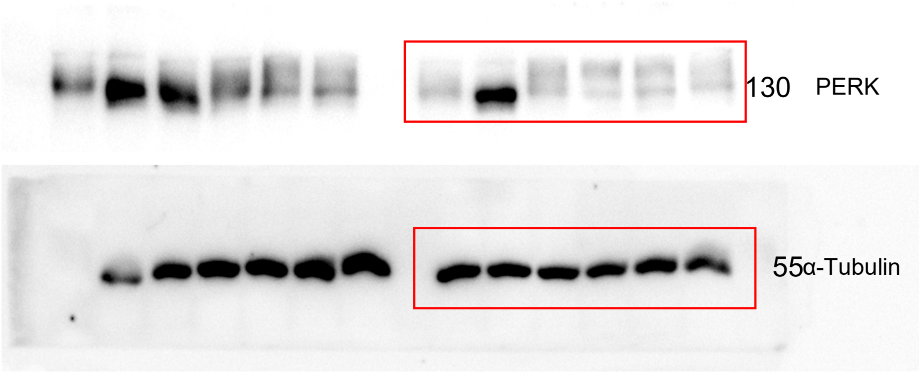

Figure 4f

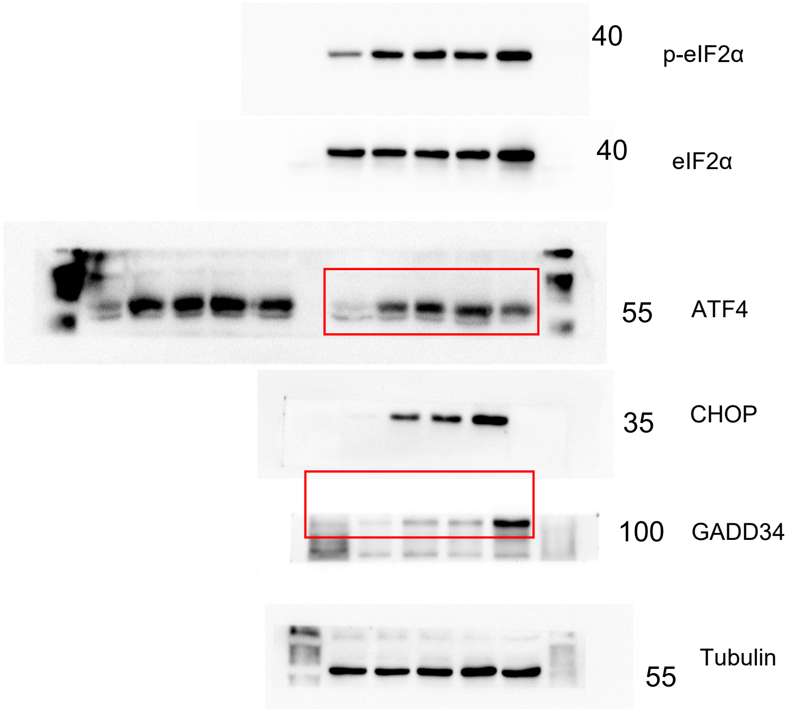

Figure 5b

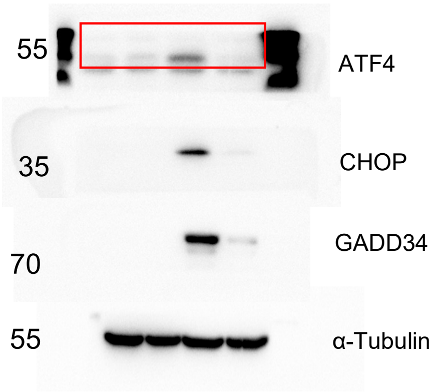

Figure 5d

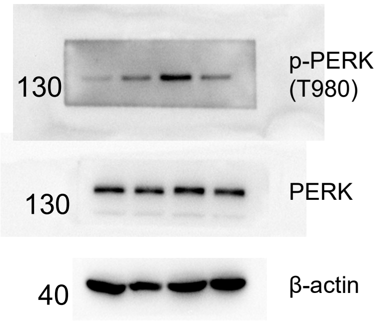

Figure 5l

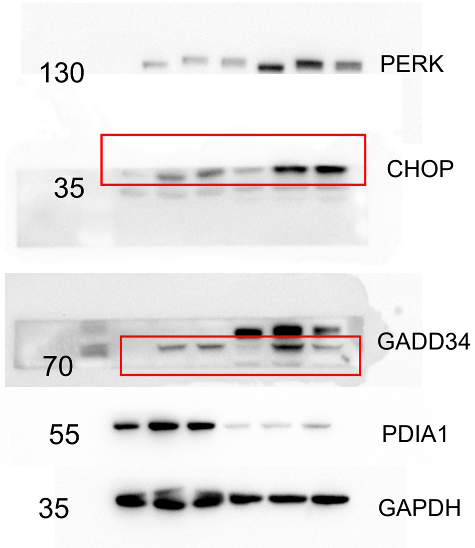

Figure 5n

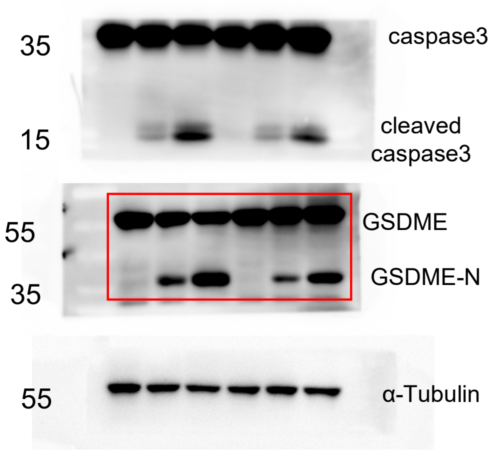

Figure 5o

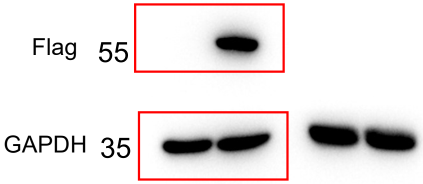

Figure S2e

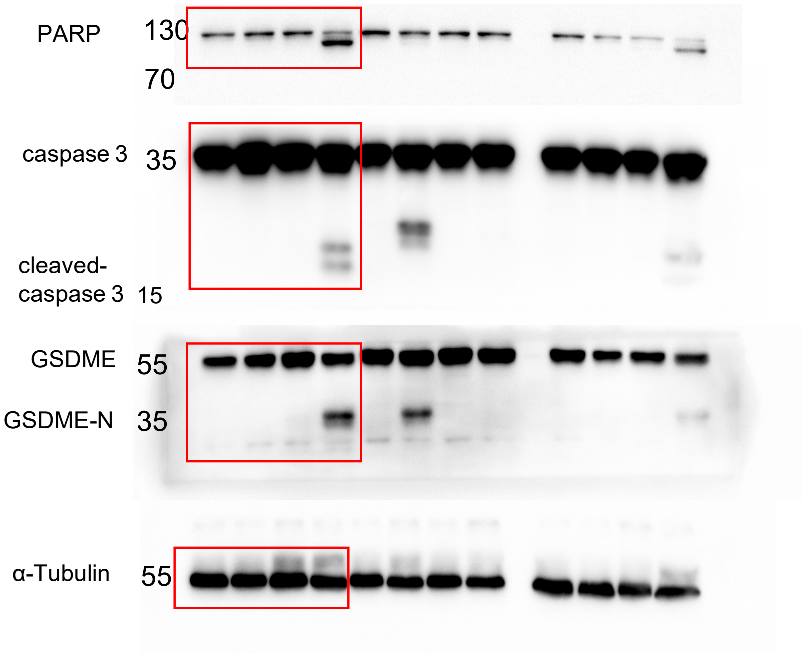

Figure S2f

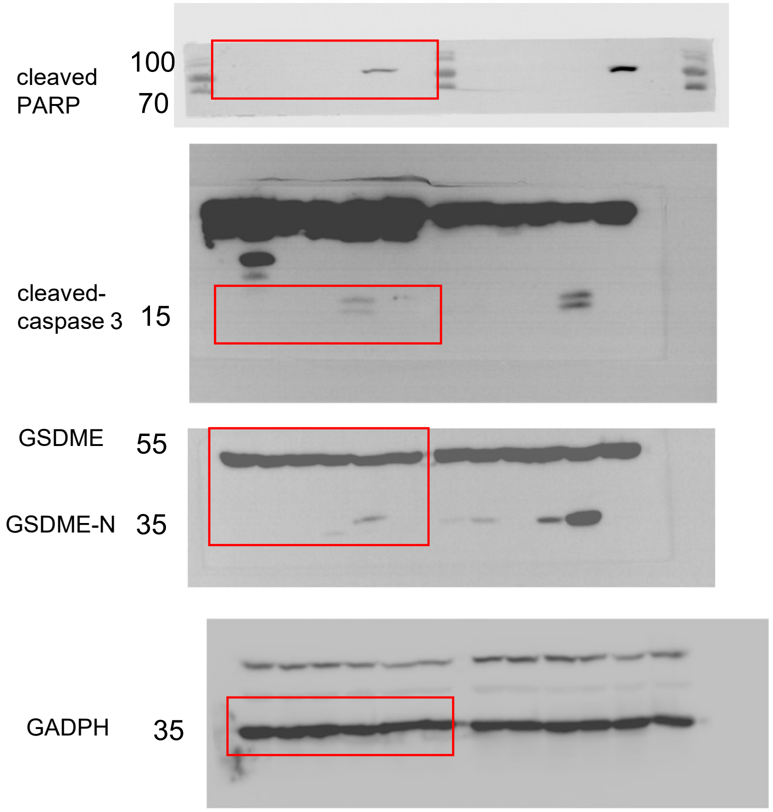

Figure S2g

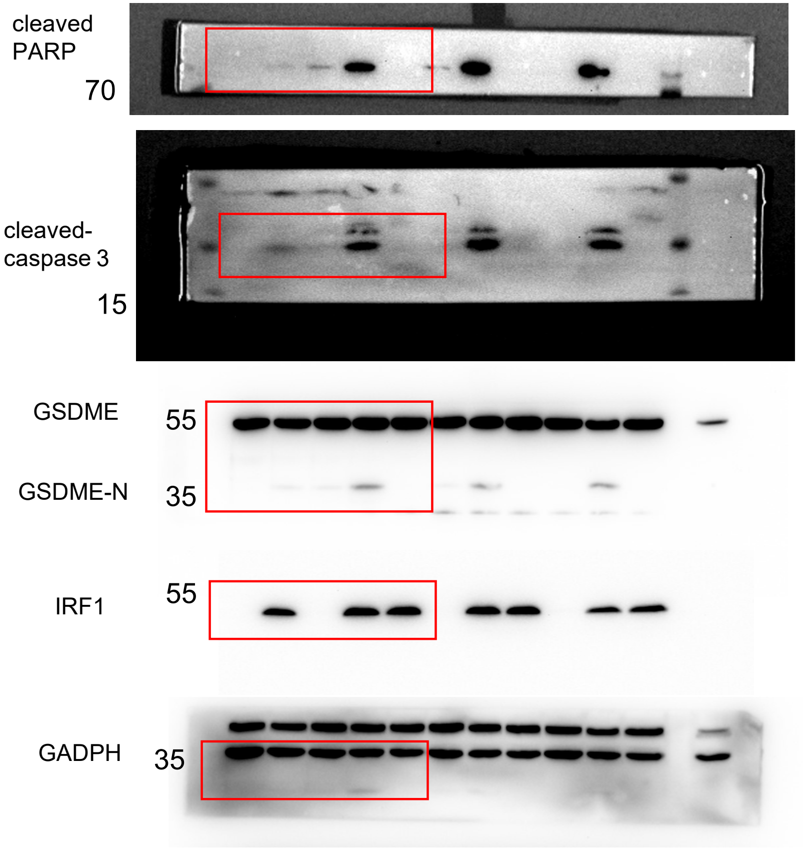

Figure S2h

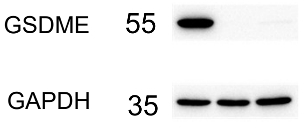

Figure S2k

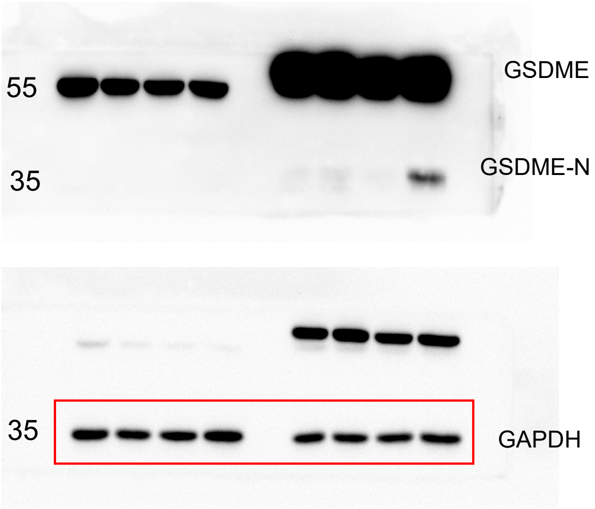

Figure S2i

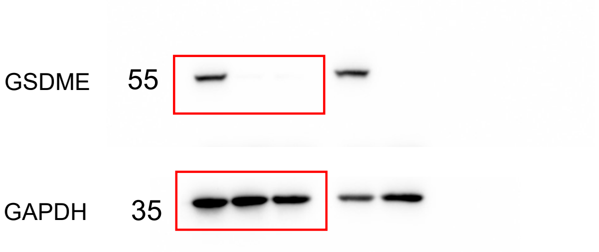

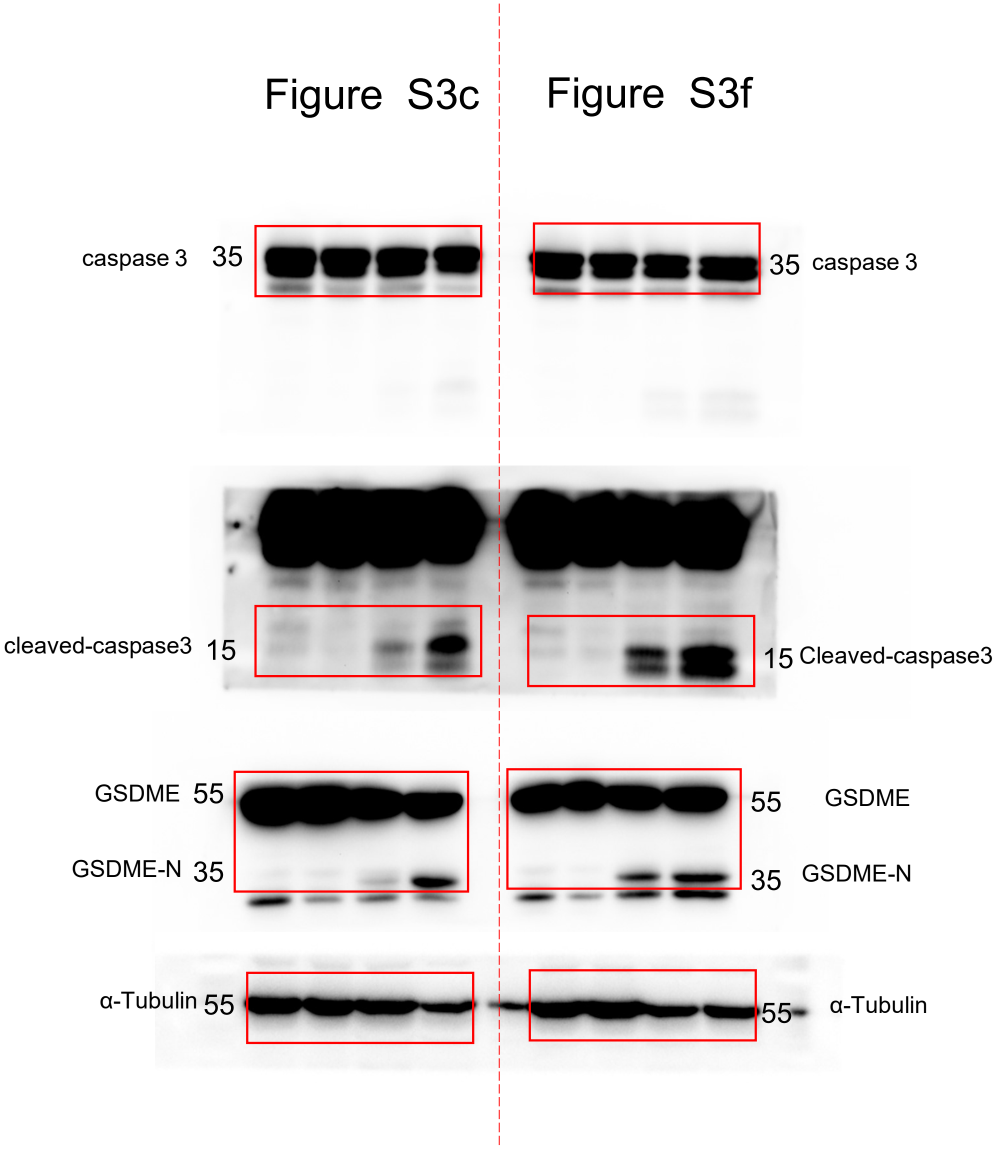

Figure S3c
Figure S3f
caspase 3
35
35
caspase 3
cleaved-caspase3
15
15
Cleaved-caspase3
GSDME
55
55
GSDME
GSDME-N
35
35
GSDME-N
α-Tubulin
55
55
α-Tubulin

Figure S4d

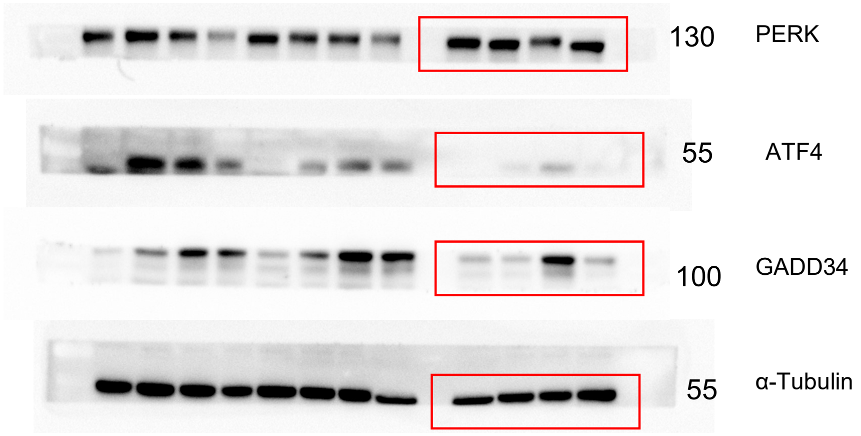

Figure S5b

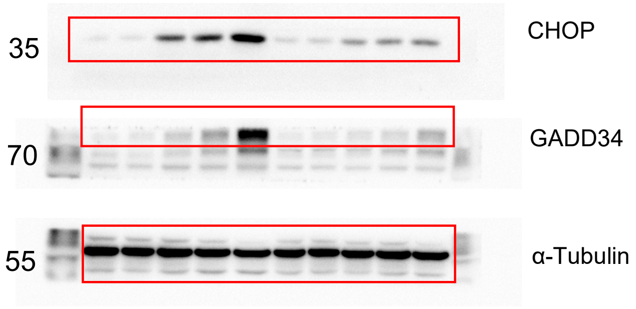

Figure S5c

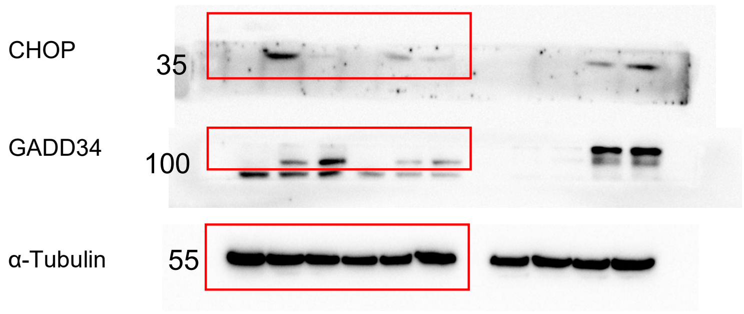

Figure S5d

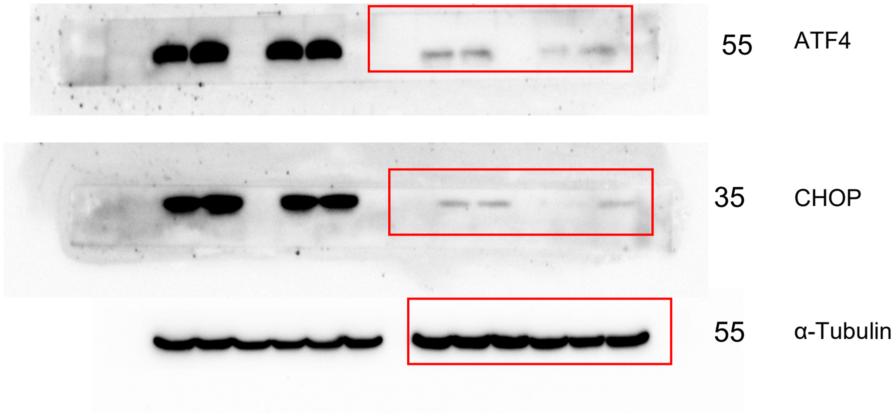

Figure S5f

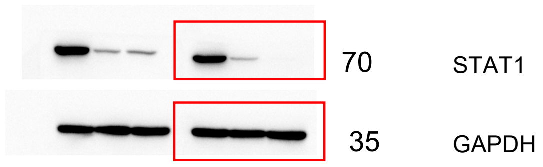

Figure S5g

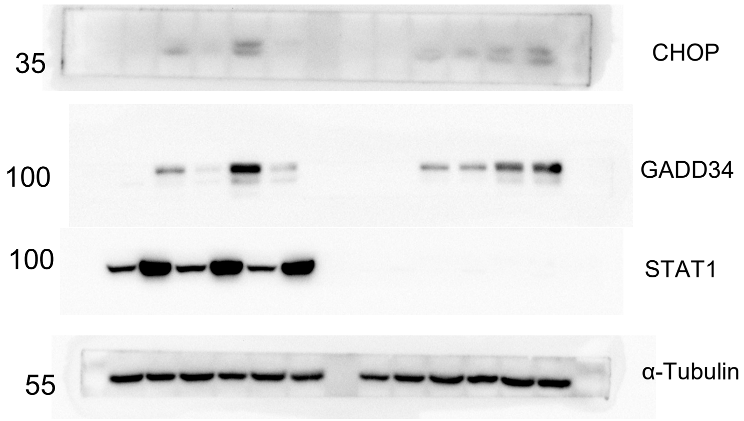

Figure S5k

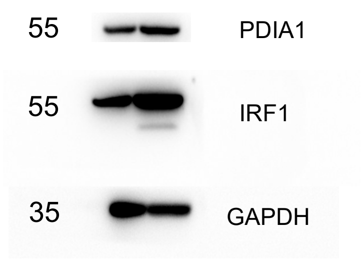

Figure S5o

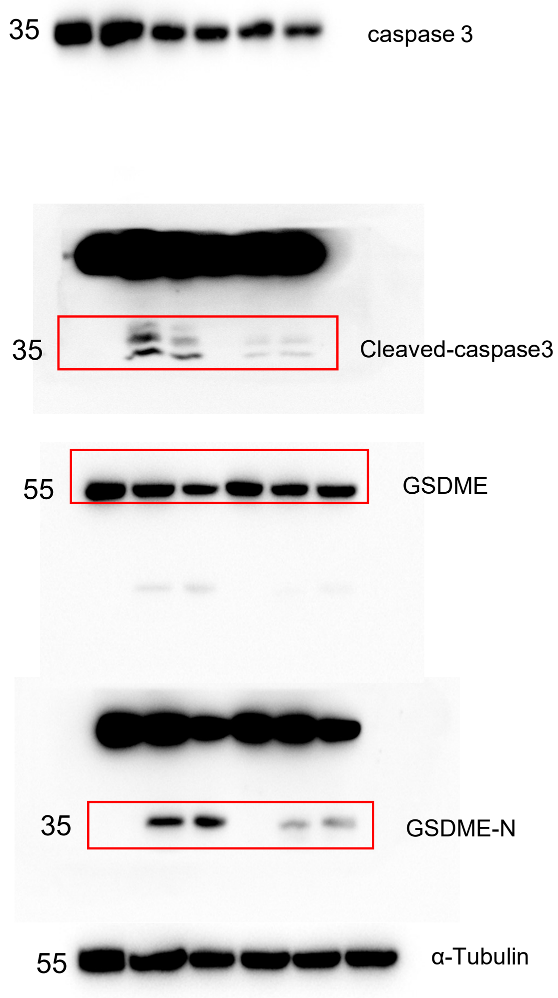

Supplement: Supplementary file 3 — Unprocessed original images of Western blots [file 41419_2025_7839_MOESM3_ESM.pdf]
